# Comparative genomics of chytrid fungi reveal insights into the obligate biotrophic and pathogenic lifestyle of *Synchytrium endobioticum*

**DOI:** 10.1038/s41598-019-45128-9

**Published:** 2019-06-17

**Authors:** Bart T. L. H. van de Vossenberg, Sven Warris, Hai D. T. Nguyen, Marga P. E. van Gent-Pelzer, David L. Joly, Henri C. van de Geest, Peter J. M. Bonants, Donna S. Smith, C. André Lévesque, Theo A. J. van der Lee

**Affiliations:** 1 Wageningen University & Research, Droevendaalsesteeg 1, Plant Science Group, 6708PB Wageningen, The Netherlands; 2 Dutch National Plant Protection Organization, National Reference Centre, Geertjesweg 15, 6706EA Wageningen, The Netherlands; 3 Agriculture and Agri-Food Canada, 960 Carling Avenue, Ottawa, Canada; 4 Université de Moncton, 18 avenue Antonine-Maillet, Moncton, Canada; 5 Canadian Food Inspection Agency, 93 Mount Edward Road, Charlottetown, Canada

**Keywords:** Data mining, Phylogeny, Gene ontology, Protein function predictions, Functional clustering

## Abstract

*Synchytrium endobioticum* is an obligate biotrophic soilborne Chytridiomycota (chytrid) species that causes potato wart disease, and represents the most basal lineage among the fungal plant pathogens. We have chosen a functional genomics approach exploiting knowledge acquired from other fungal taxa and compared this to several saprobic and pathogenic chytrid species. Observations linked to obligate biotrophy, genome plasticity and pathogenicity are reported. Essential purine pathway genes were found uniquely absent in *S. endobioticum*, suggesting that it relies on scavenging guanine from its host for survival. The small gene-dense and intron-rich chytrid genomes were not protected for genome duplications by repeat-induced point mutation. Both pathogenic chytrids *Batrachochytrium dendrobatidis* and *S. endobioticum* contained the largest amounts of repeats, and we identified *S. endobioticum* specific candidate effectors that are associated with repeat-rich regions. These candidate effectors share a highly conserved motif, and show isolate specific duplications. A reduced set of cell wall degrading enzymes, and LysM protein expansions were found in *S. endobioticum*, which may prevent triggering plant defense responses. Our study underlines the high diversity in chytrids compared to the well-studied Ascomycota and Basidiomycota, reflects characteristic biological differences between the phyla, and shows commonalities in genomic features among pathogenic fungi.

## Introduction

Species of the Chytridiomycota, also called chytrids, represent a basal lineage of fungi that arose in the Mesoproterozoic Era about 1,000 to 1,600 million years ago^1^. Most are described as free-living saprophytes, inhabiting aquatic and terrestrial environments, with some species being notorious pathogens. The knowledge of the role of chytrids in ecosystems is sparse and fragmented^2^, but recent studies indicate they may dominate ecological niches with low nutrient availability such as periglacial soils^3^. Despite being ubiquitous in nature, only of a few genomes of the entire chytrid phylum have been sequenced and studied so far. Here we present the first comprehensive functional comparative genomic study of fungi belonging to Chytridiomycota.

There are approximately 1,500 formally described chytrid species which is likely an underestimation as the relatively well-studied genus *Synchytrium* alone contains over 200 described species^4^. Most of the *Synchytrium* species are obligate biotrophic plant pathogens but recently the first saprobic free-living *Synchytrium* species, *Synchytrium microbalum*, was isolated and reported^5^. One of best studied species in the genus is *Synchytrium endobioticum* (Schilb.) Percival, the causal agent of potato wart disease, which is believed to originate from the Andes. It spread to the United Kingdom in the late 19^th^ century when breeders were in search of new potato varieties following the great Irish potato famine. From Europe potato wart spread and today it is reported from all continents with potato cultivation. Isolates are currently grouped as pathotypes (races) based on their virulence on a differential set of potato varieties, reviewed by Obidiegwu *et al*.^6^.

Like other chytrids, *S. endobioticum* does not produce hyphae or a mycelium often considered to be characteristic for fungi, but rather sporangia with motile zoospores. Upon infection, host cells are triggered to grow uncontrollably resulting in warted tissues, rendering tubers unmarketable^7^. Thin-walled, short-lived summer sporangia are formed in infected potato tissue and give rise to zoospores that can infect epidermal cells of potato in growing sprout or stolon tissues. The zoospores have been reported to conjugate possibly resulting in a (para)sexual cycle generating thick-walled resting sporangia that are released into the soil from decomposing warted potato tissue. Zoospores released from these resting sporangia can start a new infection cycle on potato tubers^8^. The lack of effective chemical treatments and the longevity resting spores further increase the devastating impact this organism has on food security^9^. Consequently, potato wart disease is one of the most important quarantine diseases on cultivated potato or on any crop^10^.

So far, studies on *S. endobioticum* focused on its life cycle, epidemiology and management of the pest, and more recently, molecular tools for detection, identification and characterization of isolates have been published, reviewed by Obidiegwu *et al*.^6^. However, still very little is known about the molecular mechanisms underlying the obligate biotrophic or pathogenic lifestyle of this pathogen. To identify genes linked to obligate biotrophy, cell wall degradation, pathogenicity and sexuality of chytrid fungi, we assembled the genomes of two *S. endobioticum* isolates from different geographic origins and of different pathotypes, and compared those to nine culturable chytrid isolates, including the amphibian pathogen *Batrachochytrium dendrobatidis*, and six fungal organisms from other phyla.

## Results

### Genome assembly and annotation

**Figure 1:**
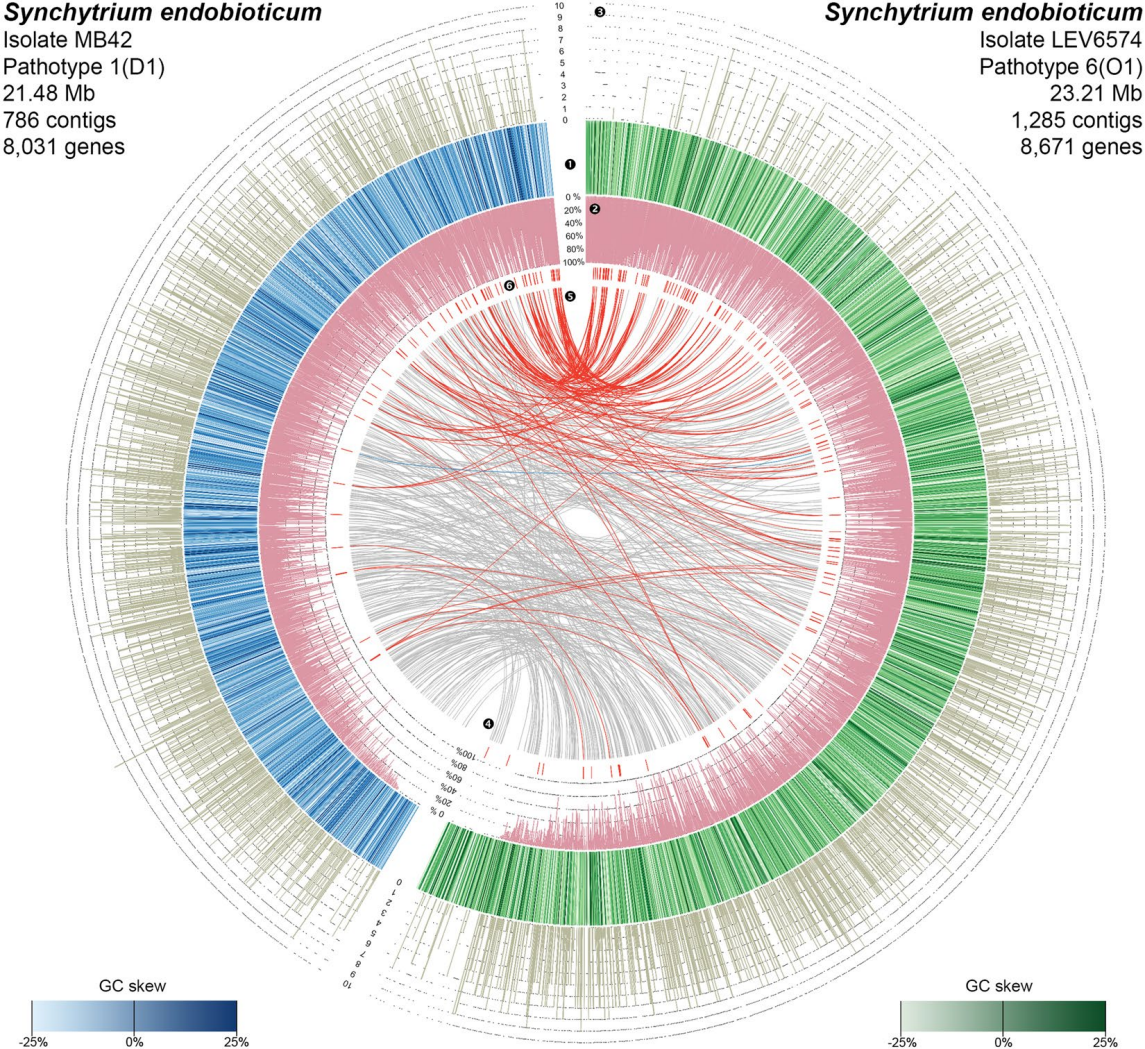
Circos plot representing *S. endobioticum* genomes of isolates MB42 (❶ blue) and LEV6574 (❶ green). Different shades of green and blue used indicate the GC skew ((G-C)/(G + C)) for the respective scaffolds and contigs. Purple annotations (❷) represent the repeat content given in percentage of 1 kb windows. Number of GO-terms predicted for each gene in the *S. endobioticum* genomes are shown in ❸. Internal links in grey (❹) represent bidirectional best hits between both isolates of single copy orthologs from the chytrid core genes. Red internal links (❺) represent COG membership of candidate effector genes indicated with ❻. The contigs and scaffolds of each genome are sorted by total repeat content. Key observations are: both genomes are gene dense and genes across the genomes are annotated (❸). No obvious GC-content clustering (❶) is observed, but both genomes contain many repeats (❷). The LEV6574 genome is larger on account of these repetitive sequences. Candidate effector genes (❻) are strongly associated to genomic regions with high repeat content (❸).

We independently sequenced and assembled the genomes of *S. endobioticum* pathotype 1(D1) isolate MB42 and pathotype 6(O1) isolate LEV6574, originating from the Netherlands and Canada respectively (Fig. 1). Although we purified the sporangia from infected potatoes as much as possible by filtration and centrifugation, the obligate biotrophic nature of *S. endobioticum* implies that our initial assembly contained genomic information from *S. endobioticum*, the potato host, as well as microbial contaminants. Identification of *S. endobioticum* specific sequences from the metagenomic assemblies was achieved using a comparative read-mapping approach which resulted in assembly sizes of 21.48 and 23.21 megabases (Mb), for MB42 and LEV6574 respectively (Figs S1–S3). Both genomes were highly similar with 98.71% average nucleotide identity (Fig. S4) and encoded 8,031 and 8,671 protein coding genes for MB42 and LEV6574 respectively. To conduct additional comparative analyses, we sequenced, assembled and annotated the genomes of four culturable chytrid species: *Chytridium confervae* CBS 675.73, *Powellomyces hirtus* CBS 809.83, *Spizellomyces palustris* ( = *Phlyctochytrium palustre*) CBS 455.65, and *Synchytrium microbalum* JEL517. Species identity was confirmed using rDNA sequences (NCBI: MH660417- MH660420^11^). Additional data from five publicly available chytrid genomes were included, namely, *Batrachochytrium dendrobatidis* JAM81 and JEL423, *Gonapodya prolifera* JEL478, *Spizellomyces punctatus* DAOM BR117 and *Homolaphlyctis polyrhiza* JEL142 (Tables S1 and S2). Based on conserved eukaryotic genes, we obtained 84.1% to 97.2% annotation completeness for the chytrid genomes analyzed based on conserved core fungal single-copy genes^12^ (Table S3).

### Phylogenomics

**Figure 2:**
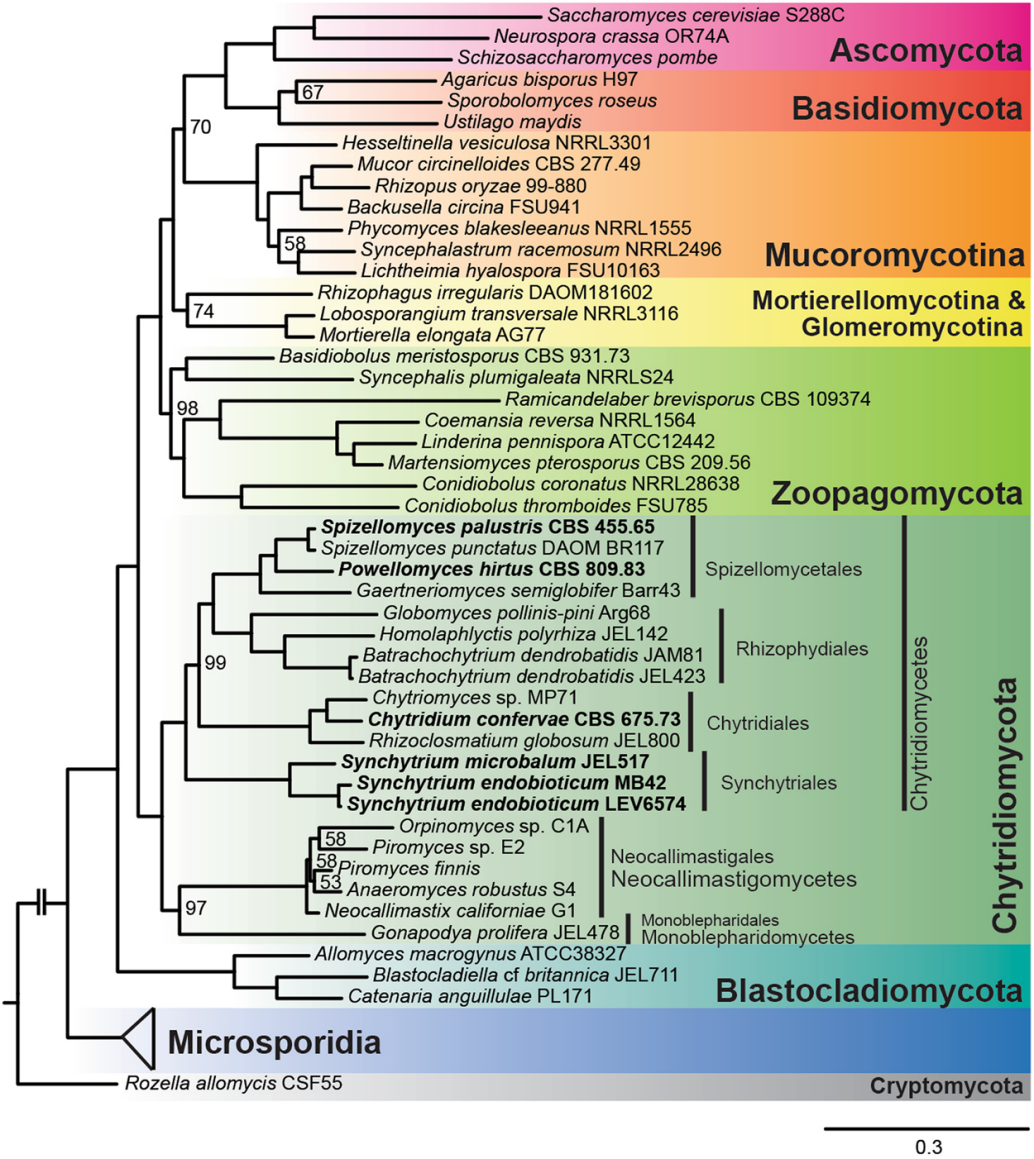
Phylogenomic maximum likelihood phylogenetic analysis of 47 fungal isolates based on a trimmed amino acid alignment containing 54,485 positions extracted from 192 genes. Genomic sequences assembled and annotated in this study are highlighted in bold. Bootstrap values other than 100% are shown on the nodes, and the scale bar indicates the mean number of nucleotide substitutions per site.

To verify and further specify the phylogenetic position of the chytrid species analyzed, we performed a maximum likelihood phylogenetic analysis, with 47 fungal isolates representing the major fungal phyla (Table S4). Based on a trimmed amino acid alignment containing 54,485 positions extracted from 192 conserved eukaryotic genes as defined by Spatafora *et al*.^13^. This concatenated tree (Fig. 2) shows 100% bootstrap support for all major phyla, as treated by Spatafora *et al*.^13^, except for Mucoromycota. There is strong support for the three classes Monoblepharidomycetes, Neocallimastigomycetes and Chytridiomycetes. The ASTRAL greedy consensus tree (Fig. S5), inferred from the combined phylogeny from each individual alignment, showed similar support for the major phyla, again with the exception of the Mucoromycota, and with a few inconsistencies in the backbone of the tree above the class level. The topologies of both trees are identical within the Chytridiomycetes confirming the placement of *S. endobioticum* within this class. The genus *Synchytrium* is currently classified in the order Chytridiales. However, our phylogenomic analysis shows that *Synchytrium* species form a distinct clade separated from members of the Chytridiales, and supports the potential transfer of the genus *Synchytrium* to Synchytriales, an order previously erected by Doweld^14^ and emended by Longcore *et al*.^5^. Our data further underlines the high diversity in the Chytridiomycota compared to the well-studied Ascomycota and Basidiomycota.

### Chytrid biology and obligate biotrophy

To identify genomic features in *S. endobioticum* that could explain its dependency on the host for survival, we performed a search of all predicted proteins against the Kyoto Encyclopedia of Genes and Genomes (KEGG)^15^, and functional annotations of *S. endobioticum* genes were compared with those of nine other chytrid species, and representative Ascomycota and Basidiomycota of which three are axenically culturable (*Saccharomyces cerevisiae*, *Neurospora crassa*, and *Cryptococcus neoformans*), and two are obligate biotrophic (*Melamspora larici-populina*, and *Puccinia graminis* f.sp. *tritici*), and one is obligate biotrophic in its diploid pathogenic phase (*Ustilago maydis*). We further performed a functional classification of genes using a Gene Ontology (GO-)term analysis, and central in this analysis were orthologous genes shared by all chytrid species as well as those unique to, or absent in *S. endobioticum*. Additionally, we performed an analysis of carbohydrate-active enzymes (CAZymes) in *S. endobioticum*, as the ability to use different types of carbohydrates is an important feature of plant pathogens.

#### Clusters of orthologous genes

**Figure 3:**
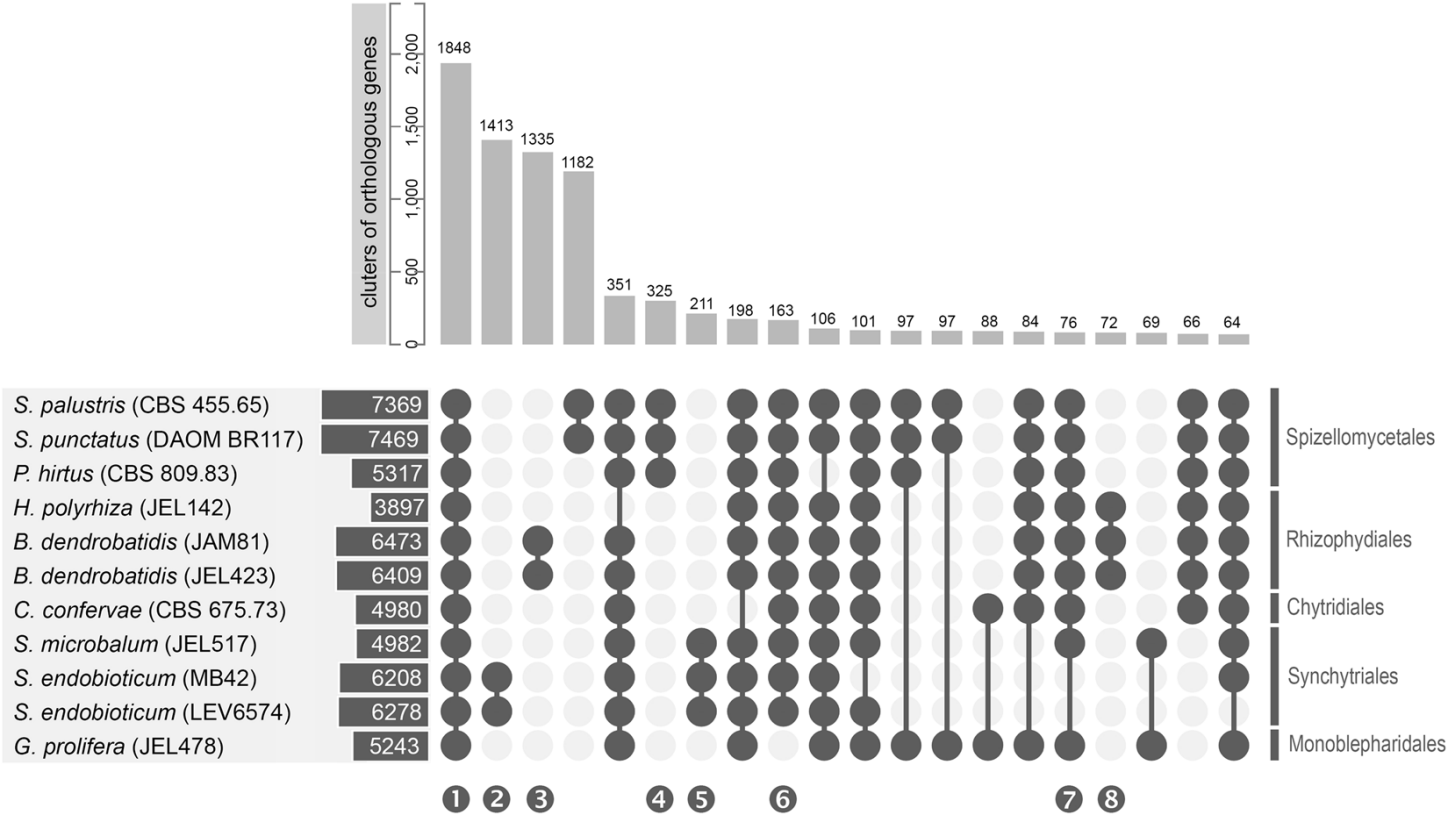
Protein families (COGs) predicted for *S. endobioticum*, *B. dendrobatidis*, *C. confervae*, *G. prolifera*, *H. polyrhiza*, *P. hirtus*, *P. palustre*, *S. microbalum*, and *S. punctatus* (sorted based on taxonomical classification). Horizontal bars indicate the number of COGs to which protein sequences are assigned for the respective isolates. Vertical bars indicate the number of COGs in intersecting selections. The number of COGs for selected intersections, indicated with connected dark grey circles, are shown sorted from those with the most abundant to the least abundant ones for intersections with >60 COGs in the intersection. Selected intersections are highlighted: ❶ shared by all chytrid species (chytrid core), ❷ *S. endobioticum* specific, ❸ *B. dendrobatidis* specific, ❹❺❽ order specific, ❻ Chytridiomycetes (class) specific, and ❼ present in all chytrids but absent in *S. endobioticum*.

On average, 87.9% of all proteins were assigned to one of 11,462 clusters of orthologous genes (COG) with more than one member. Both pathogenic species, *S. endobioticum* and *B. dendrobatidis*, have the highest number of species specific COGs compared to other chytrid species with similar gene content (Fig. 3; Table S5). In total 1,848 COGs were shared by all chytrid species, referred to as “chytrid core”, containing 694 single copy orthologs (SCOs). The second and third largest intersections of COGs were specific to both pathogenic species: 1,413 COGs with 1,179 SCOs for *S. endobioticum*; and 1,335 COGs with 1,066 SCOs for *B. dendrobatidis*. We identified 76 COGs present in all culturable chytrid species, which were absent in *S. endobioticum*. For both *S. endobioticum* isolates, a similar percentage of all genes were assigned to COGs or the chytrid core. However, the LEV6574 genome had a significantly higher percentage of genes assigned to the *S. endobioticum* specific COGs (LEV6574: 22.7%, MB42: 14.6%, two-sample binomial test: *p* < 0.001).

#### Functional annotation

GO-terms identified in chytrid species were compared to those identified in the six fungal organisms from other phyla. Terms linked to the presence of whiplash flagella were found exclusively in the chytrid genomes, i.e. “motile cilium”; “cilium”; “ciliary basal body”; “ciliary transition zone”; “MKS complex”; “outer dynein arm”; and “axonemal dynein complex”. Linked to cell motility, the biological processes term “cilium movement” was found exclusively in the chytrid genomes (Fig. S6, Table S6). Similarly, the term “chitosanase activity” was found exclusively in all studied chytrid species. To further understand the lifestyle of *S. endobioticum*, a level 2 GO-term analysis was performed on the COG intersections “*S. endobioticum* specific genes”, and “genes present in culturable chytrid species but absent in *S. endobioticum*” (Fig. S7). Compared to the genes in the COG intersection “chytrid core”, *S. endobioticum* specific genes show an increase of genes associated with signal transduction. In the underlying terms, no specific terms unique to *S. endobioticum* were found, but for some functions, an increased number of proteins with that particular function was observed, namely, “small GTPase mediated signal transduction” and “G protein coupled receptor signaling pathway”. In the COG intersection “genes present in all chytrid species but absent in *S. endobioticum*”, GO-terms for “methylation”, “regulation of biological quality”, and “cellular component organization” were more abundant compared to the chytrid core. However, most of the lower-level terms that were found in these genes were also found in genes belonging to the chytrid core, indicating that the other chytrid species simply have more genes with the same functionality. One term was an exception: “regulation of mitochondrial membrane permeability involved in programmed necrotic cell death”, which was specifically absent in *S. endobioticum*.

#### KEGG pathways

**Figure 4:**
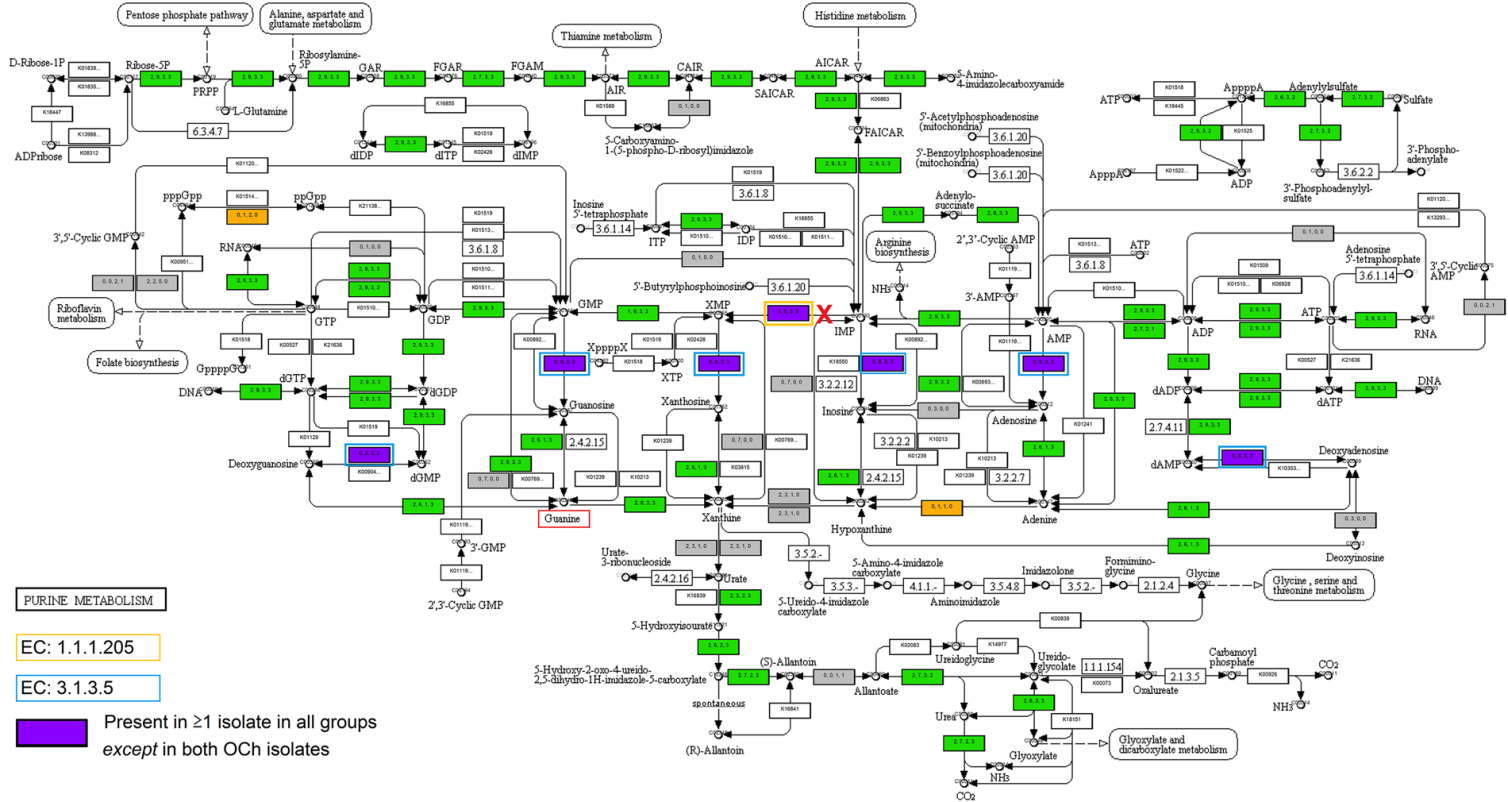
KEGG reference pathway 230: Purine metabolism^15^^©^ Kanehisa Laboratories. Numbers (*a, b, c, d*) in the enzymatic steps indicate the number of isolates in a given group for which the corresponding gene was detected. Colors indicate which groups are represented for a given enzymatic step: present in ≥1 isolate in all groups (green); present in ≥1 isolate in all groups except *S. endobioticum* (purple); present in ≥1 isolate in both culturable groups, but not in the obligate or facultative biotrophs (orange); unassigned (white). The red “X” indicates the pathway for *de novo* Guanidine triphosphate (GTP) synthesis that is blocked in *S. endobioticum*. Boxed in orange is inosine-5′-monophosphate dehydrogenase (EC:1.1.1.205), and boxed in blue is Pyrimidine 5′-nucleotidase (EC:3.1.3.5), which were both found to be present in all genomes analyzed except in *S. endobioticum*. Boxed in red is guanine that could be scavenged by *S. endobioticum* from its host allowing synthesis of GTP.

We benchmarked the analysis using the six fungal organisms from other phyla with known biochemical pathways included in the KEGG database, demonstrating an overall accuracy of 84.5% (Table S7, Supplementary file 4), and subsequently we compared these to the functional annotation of the chytrid genomes. InterProScan function assignment was found to be accurate with a high percentage of specificity (average = 93%). However, we found that the KEGG pathway analysis was particularly prone to false negatives (Figs S8 and S9) resulting in incomplete function prediction (e.g. the fatty acid biosynthesis pathway). Therefore, absence of specific genes was verified with tblastn. On average, 7.3% of the genes in the chytrid genomes were assigned to a KEGG pathway, compared to 5.9% in the fungal organisms with known KEGG pathways. For both *S. endobioticum* isolates, 6.2% of the gene models were assigned to a KEGG pathway (Table S8). Enzymes that were absent in *S. endobioticum* but were present in the majority of proteomes of the other species analyzed fell in 26 of 72 reference biochemical pathways. After tblastn verification, 22 enzymes were found to be absent in 18 biochemical pathways (Table S9; Supplementary file 3). The absence of two enzymes in the purine metabolism pathway, i.e. inosine-5′-monophosphate dehydrogenase (IMPDH, EC:1.1.1.205) and pyrimidine 5′-nucleotidase (EC:3.1.3.5), could provide further insight on the obligate biotrophic lifestyle of *S. endobioticum* (Fig. 4). The latter enzyme also plays a role in the pyrimidine pathway. Unique to the chytrid species is uracil phosphoribosyltransferase (EC:2.4.2.9) which catalyzes the reaction from UMP to uracil in the pyrimidine pathway (Supplementary file 3, Pyrimidine pathway).

#### CAZymes

Altogether 246 different CAZyme modules were detected in the chytrid species and the six fungal organisms from other phyla. Six families were only found in chytrids (GT92, GH46, GT14, PL14, AA1 subfamily 1, GH5 subfamily 27); a single family (GT67) was found to be unique to *S. endobioticum*; and 29 families were absent in *S. endobioticum* but present in all species analyzed (Table S10). The Carbohydrate-Binding Module Family 35 (CBM35) family is present in all obligate and facultative biotrophic species analyzed but missing in the culturable species. In *S. endobioticum*, genes with CBM50, also known as LysM, were found to be enriched compared to the other species included in the analysis (means: 22 to 7, T-test: *p* < 0.001). Similarly, CBM18 was found enriched in *B. dendrobatidis* (means: 83 to 8; T-test: *p* < 0.001). Cluster analysis based on all assigned CAZyme modules grouped the two *S. endobioticum* isolates and also the closely related species *S. microbalum*, *P. hirtus*, *S. punctatus* and *S. palustris* group into a clade (Fig. S10). The two *B. dendrobatidis* isolates, causal agents of amphibian chytridiomycosis, also clustered together but were clearly an outgroup compared to the other fifteen species. The three species with obligate or facultative biotrophic lifestyles (*U. maydis*, *M. larici-populina*, and *P. graminis* f. sp. *tritici*) grouped together but were distant from the two *S. endobioticum* isolates.

### Genome plasticity

Genome plasticity is an important feature of fungal plant pathogens, and is often facilitated by repeats. We therefore studied the repeat content of all chytrid genomes, and analyzed if the repeat induced point mutation (RIP) mechanism^16^, which protects fungal genomes against duplications and transposable elements, was active in chytrid species. As a sexual cycle has great impact on genome dynamics, we further performed an *in-silico* analysis for the potential of sexual reproduction in chytrids.

#### Repeat content and transposable elements

**Figure 5:**
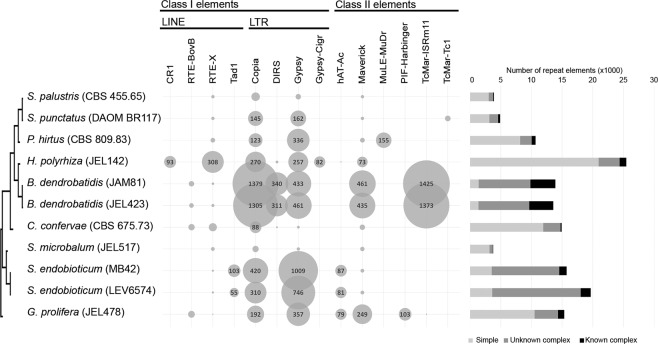
Repeat and transposable element (TE) content in chytrid species analyzed. Chytrid isolates are sorted based on taxonomical classification, and spheres indicate the number of class I and II TEs were identified in the individual isolates. Horizontal bars indicate the number of repeat elements per genome, which is further differentiated as small simple repeats (light grey), unknown complex repeats (dark grey), and complex repeats with TE identity (black).

Together with the amphibian pathogen *B. dendrobatidis*, *S. endobioticum* displayed the highest percentage of repetitive sequences in its genome (Table S12). The repeat content could be split in small simple repeats (SSR) and complex repeats. In total, 399 complex repeat families were identified of which 15 were shared by all chytrid genomes analyzed. For the culturable chytrid species the repeat repertoire mainly consisted of small simple repeats (64.1 to 84.4%). In contrast, the repeat repertoire of both pathogenic species mainly consists of complex repeats (77.6 to 89.9%). Over a third of all repeat families was unique to the two *S. endobioticum* isolates, and *S. endobioticum* has significantly the most diverse repeat repertoire (ANOVA: *p* < 0.001) compared to other chytrid species (Fig. S11). Both Class I (retro) and Class II (DNA) transposable elements were identified in the two *S. endobioticum* genomes. Most repeats could not be classified to any known element. For the elements that could be classified, Gypsy and Copia retrotransposons were most abundant. These are also the dominant transposable elements in other chytrid species. Unique to *S. endobioticum* is the non-LTR retrotransposon Tad1 (Fig. 5). In the pathogenic *B. dendrobatidis*, repeats were clustered in repeat-rich compartments. In contrast, the repeats were found to be dispersed over the entire genome in both *S. endobioticum* isolates (Fig. 1).

#### RIP activity

RIP activity can be observed by a bimodal distribution of GC content as a result of CpA to TpA transitions. Such a distribution was not observed for both *S. endobioticum* genomes, nor for any other chytrid genome indicating that no apparent RIP affected regions were present in these genomes (Fig. S12). To further determine if repeat families in *S. endobioticum* were specifically affected by RIP, the 10 most abundant species specific repeat families matching the prerequisites for RIP (400 bp repeats^17^ with ≥80% sequence similarity^18^ in *N. crassa*) were subjected to the alignment based RipCal analysis. For some repeat families, the copy number of (near) complete gene models represented was similar for both isolates, while for others expansions in one of the isolates were observed (Figs S13 and S14). RIP signatures were not detected in any of the analyzed alignments. In addition, the protein essential for RIP, RID (RIP defective)^19^, could not be detected in the *S. endobioticum* isolates using blastp or tblastn. RID exhibits methyltransferase activity and belongs to the protein family of C-5 cytosine methyltransferases. All C-5 cytosine methyltransferases contain a motif (motif VI) with two conserved amono acids (NV: asparagine-valine), except for RID and the gene associated with Methylation Induced Premeiotically (MIP: another fungal defense mechanism against TEs) *Masc1*^20^. In the latter two proteins NV is replaced with either QT or ET. Thirty-seven C-5 cytosine methyltransferase proteins were identified in the chytrid species analyzed. The motif VI sequences of these proteins did not possess the consecutive QT or ET amino acid sequences associated with RID and *Masc1* (Fig. S15), which confirmed that RID is not present in the analyzed chytrids.

#### Sexual cycle

GO-terms linked to meiosis were identified in all chytrid genomes (Fig. S16). Subsequently, we investigated if their genomes possess the genes required for meiosis referred to as the meiotic toolbox. A set of 86 genes required for successful meiotic recombination in *S. cerevisiae* and *C. neoformans* was used to identify orthologs in Chytridiomycota. Of this set, 31 are regarded core meiotic proteins with ten considered as meiosis specific proteins^21^. Homologs were found in one or more chytrid species for 90% of the meiotic core proteins (Table S11). Verification by tblastn was performed for the meiotic core and meiosis-specific proteins to determine if they were truly absent. Of the ten meiosis specific genes, only six were detected in all chytrid species analyzed, and three were detected in all but one or two isolates. One (*Rec8*) could not be detected using the *S. cerevisiae* protein, while it was detected in six of the chytrid fungi analyzed using the *Allomyces macrogynus* homolog. In contrast, the mitotic homolog of *Rec8* (*Rad21*) was detected in all chytrid species analyzed. For the remaining 21 meiotic core genes, homologs were identified in all chytrid species for 16 genes. Similar to the findings of Halary *et al*.^21^, we were unable to identify a homolog of *Scc3* in any of the chytrid species. For the remaining four genes (*Mlh2*, *Mlh3*, *Mus81*, *Rad51*), a patchy distribution was obtained with the detection of homologs in four or more chytrid species. Clearly some genes from the meiotic toolbox are absent, however, the consequences for meiosis are yet unclear.

Compatibility during sexual reproduction is regulated by mating types, and in filamentous Ascomycetes, typically two mating types are present referred to as *Mat1-1* and *Mat1-2*. The *Mat1-1* gene encodes a protein with a high mobility-group (HMG) DNA-binding motif, whereas the *Mat1-2* encodes a protein containing an alpha box motif^22^. A blastp analysis using representative protein sequences of each of the two mating type genes did not result in significant hits for any of the chytrid species analyzed. In filamentous fungi, the mating type locus is either flanked by or closely associated with genes coding for Sla2 and Apn2^23^. Homologs of these genes were identified for all chytrid species in COGs OG1211 and OG530 respectively. However, no putative MAT genes encoding HMG DNA binding or alpha box motifs were found on the same scaffolds with the *Sla2* and *Apn2* homologs. If sexual compatibility in chytrid species is regulated similarly compared to Ascomycetes, the MAT genes are likely diverged beyond recognition for blast analyses, and the MAT locus is likely not associated to the *Sla2* and *Apn2* genes as is the case for Ascomycetes.

### Pathogenicity

Plant pathogens facilitate host colonization by manipulating the plant or its microbiome by secreting effector proteins which are typically species or even isolate specific and may contain specific motifs, reviewed by Selin *et al*.^24^. We analyzed the secretomes of both *S. endobioticum* isolates to identify candidate effector protein that could act as elicitors during host infection.

#### Candidate effectors

**Figure 6:**
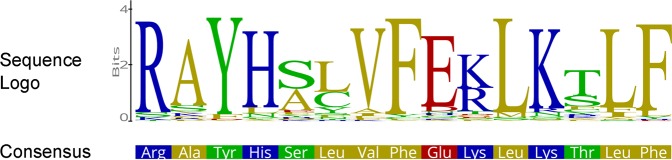
Sequence logo of the *S. endobioticum* specific RAYHxxVFExLKxLF sequence motif, referred to as “RAYH-motif” based on the first four conserved amino acids, found in 148 LEV6574 and 75 MB42 candidate effector proteins.

For both pathogenic species, *S. endobioticum* and *B. dendrobatidis*, the majority of secreted proteins is species specific. Screening of the *S. endobioticum* secretome, one particular 15 amino acid motif was found: i.e. RAYHxxVFExLKxLF, which we refer to as the RAYH-motif (Fig. 6). Proteins with the RAYH-motif were found exclusively in *S. endobioticum* with 148 and 75 proteins for the isolates LEV6574 and MB42 respectively. The majority of these proteins had one occurrence of this motif, but 41 and 28 proteins for the respective isolates had two (Fig. S17; Table S14). Significant expansions were found between the two isolates (Figs 1 and S18). While many orthologous groups were found to be expanded in LEV6574 this was rarely the case for MB42. However, read coverage on these candidate effector genes indicates a higher copy number variation for MB42, suggesting that in some cases these genes were not resolved in the MB42 genome assembly (Fig. S19).

**Figure 7:**
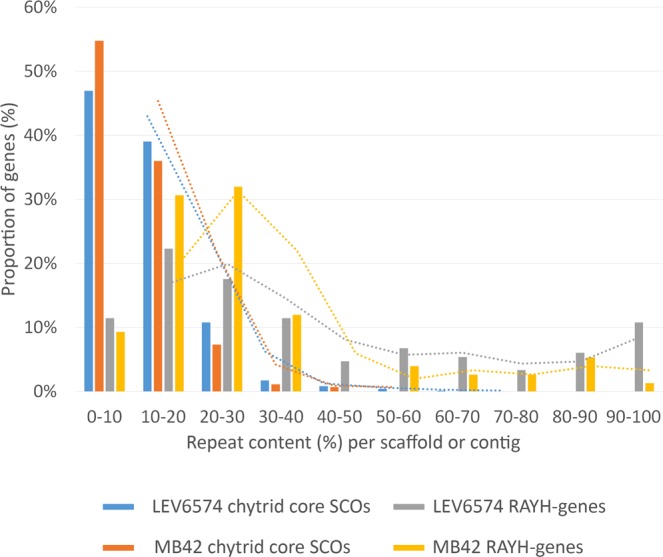
Occurrence of *S. endobioticum* chytrid core SCO genes and RAYH-genes in function of scaffold repeat content percentages. The proportion of candidate effector or SCO genes are compared against the scaffolds/contigs where repeats occur. The trend is shown as dotted lines. The *S. endobioticum* SCO chytrid core genes are mainly found on scaffolds and contigs with 0 to 30% repeat content. Candidate effector genes are found mainly on scaffolds and contigs with 0 to 40% repeat content but also on genomic sequences with up to 100% repeat content.

The RAYH-genes appear to be overrepresented in regions that show depletion of chytrid core genes (Fig. 7). Moreover, they were significantly closer to terminal ends of genomic scaffolds compared to chytrid core SCOs (Fig. S20), which is possibly a consequence of their association with repeats that break up the assembly. We observed several (near) identical members of these candidate effectors, and an alignment of the genes coding for RAYH-proteins revealed a conserved architecture (Fig. S21) with some gene models showing discrepancies. Interestingly, most genes that were not predicted to be secreted were among those showing additional or missing amino acids in the N-terminal region, which could indicate errors in the gene predictions or pseudogenized genes.

Apart from conservation in some structural features (intron-exon boundaries) and the motif, the proteins were highly variable. RAYH-proteins were found to be separated within 70 orthologous groups (41 when excluding singletons). This could indicate that they may have different functions but share a similar localization or translocation mechanism. We therefore further analyzed the possible localization of the proteins and compared proteins with this motif with chytrid core SCOs. Proteins carrying a mitochondrial targeting peptide (mTP) and/or a nuclear localization signal (NLS) were found to be overrepresented in secreted proteins having the RAYH-motif. In LEV6574, mTP was found in 39.1% of secreted proteins carrying the motif, versus 12.6% when considering the whole secretome. Similarly, a NLS was found in 43.4% of secreted proteins harboring the motif, versus 24.1% of the whole secretome. In many cases, more than one targeting peptide or motif was present (e.g. 16.3% of motif-containing proteins had both mTP and NLS, compared to 3.6% for the whole secretome). No significant differences were observed when comparing the occurrence of the chloroplastic transit peptide.

## Discussion

We assembled and annotated the genomes of two *S. endobioticum* isolates representing different pathotypes from different continents. As this is an obligate pathogen, the DNA and RNA had to be extracted from resting spores purified from infected potatoes. Apart from the fungal pathogen and its host, these nucleic acid extracts also represented other microbial soil organisms. The comparative read-mapping approach used to identify sequences of the target species was successful in removing contaminant sequences and resulted in genome sizes with a gene content similar to other chytrid fungi (Fig. S22). A high level of annotation completeness was obtained as indicated by the BUSCO scores obtained from the predicted proteins. From our phylogenomic analysis, we get very strong support for placement of the *Synchytrium* species in the order Synchytriales. Genes shared by all chytrid species (chytrid core), specific to *S. endobioticum* and present in all chytrid species except *S. endobioticum* were identified. We postulate that the chytrid core genes reflect shared biological features and processes of species in the Chytridiomycota. On the other hand, genes specific to *S. endobioticum* are hypothesized to hold the key to pathogenicity while genes absent in *S. endobioticum* but present in other chytrids could potentially explain the obligate biotrophic lifestyle of the pathogen. Functional annotation and analysis of these genes resulted in several important observations.

### Chytrid biology and obligate biotrophy

#### Motility

Our functional analysis reflects the presence of flagella and associated motility in Chytridiomycota, which are absent in Ascomycota and Basidiomycota. Motility and sensing of the environment using G coupled receptors reflects a fundamental difference between Chytridiomycota and Ascomycota and Basidiomycota. The lack of a self-directed motile life stage makes ascomycetes and basidiomycetes dependent on passive spore dispersal or physical growth, resulting in sometimes extreme body sizes such as found for *Armillaria mellea*^25^. The motile life stage of Chytridiomycota may allow them to be more selective and grow more efficiently (energy-wise) in a targeted (pathogenic) lifestyle or under low nutrient conditions. This could perhaps explain why chytrids dominate periglacial soils^3^ or are well adapted for a pathogenic lifestyle in which a self–directed motile, search life stage could be of critical importance. Associated with movement is taxis, the directed movement of a motile cell in response to an external stimulus. Both pathogenic chytrids, *S. endobioticum* and *B. dendrobatidis* as well as *H. polyrhiza* possess genes linked to chemotaxis. A distinct phylogenetic group, the oomycetes, share a self-directed motile life stage and include mostly plant and animal pathogens, such as *Phytophthora* and *Saprolegnia* species.

#### Obligate biotrophy

*S. endobioticum* lacks genes encoding the enzymes IMPDH and pyrimidine 5′-nucleotidase as opposed to chytrids with a saprophytic lifestyle (including *S. microbalum*) where these enzymes are present. These genes encode enzymes that are essential in the purine and pyrimidine pathways. IMPDH irreversibly catalyzes the conversion of inosine monophosphate (IMP) to xanthosine monophosphate (XMP), which serves as an intermediate for the nucleotide guanosine monophosphate (GMP). This indicates that *S. endobioticum* is not able to synthesize Guanosine 5′-triphosphate (GTP) from IMP *de novo*, and therefore would rely on scavenging guanine to synthesize GMP which is converted to GDP and GTP. Guanine nucleosides are crucial prerequisites for many cellular functions including transmembrane and intracellular signaling, DNA replication, and RNA and protein synthesis. This is demonstrated by null mutants of *Saccharomyces cerevisiae* for all four IMPHD orthologs. The simultaneous deletion of these genes was lethal unless growth media were supplemented with guanine^26^. Lack of the pyrimidine 5′-nucleotidase restricts the catalysis of phosphorylytic cleavage of ribonucleotide monophosphates GMP, AMP, IMP and XMP to their respective nucleosides guanosine, adenosine, inosine and xanthosine in the purine pathway and of UMP, CMP and TMP to uridine, cytidine and thymidine in the pyrimidine pathway. To create the before mentioned nucleosides, we hypothesize that *S. endobioticum* relies on scavenging of the purine and pyrimidine bases from its host for survival, as was previously described for pathogenic Mycobacteria^27^ which also cannot be cultured axenically.

#### CAzymes

The major polysaccharides present in plant cell walls are cellulose, hemicellulose and pectin. The CAZymes that are involved in degradation of these molecules are referred to as cell wall degrading enzymes (CWDE). We split the CAZyme families into several functional categories following Kubicek *et al*.^28^. Overall, *S. endobioticum* has the ability to process complex sugars, which could include cellulose and starch. It can degrade only some hemicellulose because it is lacking the alpha-galactosidases, beta-glucuronidases and alpha-arabinosidases. As for pectin degradation, it is not as clear because only a single family (GH28) with one copy was found in both *S. endobioticum* isolates. We noted however that there were discrepancies between the CAzyme analysis results, and results linked to CAzymes obtained with KEGG and GO-terms (Fig. S14). For its energy needs, *S. endobioticum* has the ability to process starch and fructose and it is therefore not directly dependent on the degradation of cellulose (Supplementary file 3, starch and sucrose metabolism pathway).

### Genome plasticity

#### Repeats, TE, and RIP

The *S. endobioticum* assemblies are relatively fragmented compared to other genomes assembled in this study which is a direct result of the repeat content of the *S. endobioticum* genome. Both *S. endobioticum* isolates, together with *B. dendrobatidis*, have the highest repeat content of all chytrid species analyzed. For both species the repeat content mainly comprises complex repeats, opposed to small simple repeats which are the predominant form of repeats in other chytrid species. The repeat repertoire and TE activity of filamentous fungi have been linked to pathogenic lifestyle in many cases^29^. In *U. maydis* for instance, repetitive compartments containing TEs were significantly associated with virulence gene clusters^30^. In the pathogenic *B. dendrobatidis*, repeats are clustered together in repeat rich compartments. However, in both *S. endobioticum* isolates the repeats are dispersed over the genome. The majority of the complex repeats could not be identified as either class I or II elements, suggesting that novel families of transposable elements could exist in Chytridiomycota. In the context of the other chytrids, the non-LTR retrotransposon Tad1, which was found to be active in a RIP-proficient isolate of *N. crassa*^31^, was unique to *S. endobioticum*. The genomes of chytrids do not appear to be protected by RIP, other defense mechanisms against active TEs such as post-transcriptional gene silencing (quelling)^32^ could play a role to restrict genome expansions of the Chytridiomycota. Interestingly, the small genome of *S. endobioticum* contained many repeats and introns, and the condensed size of the genome is achieved by the combination of a relatively low number of genes and short intergenic spaces.

#### Sexual cycle

A sexual cycle in Chytridiomycota was described from species in several genera, for instance *Synchytrium*, *Chytridium*, *Micromyces*, and *Rhizophydium*^33^, but the occurrence of a sexual cycle is disputed for others including the best studied species *B. dendrobatidis* and *S. endobioticum*. The fusion of *S. endobioticum* zoospores, which were postulated to act as isogametes, and the formation of biflagellated zygote were observed as early as 1921 by Curtis^8^. Her observations on the fusion of zoospores were confirmed by several authors^34–38^ but comprehensive electron microscopy studies were not able to provide ultrastructural evidence for karyogamy and the meiotic divisions that hallmark meiosis. Halary *et al*.^21^ used the publically available genomes of *B. dendrobatidis* and *A. macrogynus* to define the presence of meiotic genes in Chytridiomycota. However, the latter species was previously included in Chytridiomycota but is now regarded to be a member of the Blastocladiomycota. This resulted in an inventory of chytrid meiotic genes which also included specific presence or absence of meiotic genes in Blastocladiomycota. The results from our study paint a more complete picture regarding the meiotic toolbox of chytrid species. However, because of the patchy presence and absence of some specific meiotic genes, and absence of mating type genes in the chytrid species analyzed, a conclusive answer regarding a sexual cycle in chytrids remains elusive. Another indicator of a cryptic sexual cycle, i.e. RIP activity^18^, was not found in chytrids. Active Copia and Gypsy retrotransposons however, which were suggested to be a sign for a sexual cycle^39^, were found in the chytrids analyzed.

## Pathogenicity

### Host recognition

Our CAZyme analyses point to a reduced number of cell wall degrading hemicellulases in *S. endobioticum* compared to its saprobic sister species *S. microbalum*. Reduced CWDE content was previously found in the stealth like pathogen *Zymoseptoria tritici* (formely known as *Mycosphaerella graminicola*)^40^, and the biotrophic and symbiotic fungus *Lacaria bicolor*^41^, which may reduce or prevent the triggering of defense responses as a result of damage-associated molecular patterns from cell wall degradation^42^. In *B. dendrobatidis*, genes encoding proteins belonging to the CBM18 family were found to be more abundant. Proteins from this family binds to the N-acetyl-D-glucosamine and sialic acid found in chitin and mucous membranes, respectively. In *S. endobioticum*, genes with CBM50, also known as LysM were found to be enriched. LysM containing proteins could be involved in chitin metabolism, or could have a function in the interference of chitin-triggered immunity^43^, as was suggested for the endophytic fungus *Piriformospora indica*. Also, the arbuscular mycorrhizal fungus *Rhizophagus irregularis* encodes LysM effectors and candidate chitinases (which were also identified in the chytrid species analyzed) on its genome, and it is suggested that these mutualistic fungi remodel their cell wall during intracellular colonization^44^. Similar remodeling of cell walls could be a viable strategy for both pathogenic species *S. endobioticum* and *B. dendrobatidis* to avoid recognition by their host.

#### Candidate Effectors

Effector proteins are expected to play a central role in the pathogenic lifestyle of *S. endobioticum* as they manipulate the host or its microbiome to facilitate infection. *S. endobioticum* has no mycelium and is intracellular. In some pathogenic groups, motifs such as the RXLR motif in oomycetes^45^ have been identified in secreted proteins and are now routinely used to predict candidate effectors. We found a novel 15 amino acid motif, referred here as the RAYH-motif, and RAYH-proteins are regarded candidate effectors as they are species specific, secreted, associated with repeat rich regions and carry localization signals. The conservation in the gene architecture of genes carrying the motif is surprisingly high, and the number of members is numerous in the two *S. endobioticum* isolates.

Effectors from various pathogenic organisms are preferentially located in repeat-rich and gene-poor regions of the genome^46^. Although the RAYH-proteins were associated with repeat rich regions in general, no direct association with specific repeat content, as is the case in *Fusarium* species with two specific miniature inverted-repeat transposable elements (MITEs)^47^, was observed. The specialization of pathogens is reflected by the fact that both pathogenic chytrid species have the highest species specific gene content. Also, the majority of the secretome was found to be species specific.

When intersecting RAYH-proteins with EffectorP, only a few members were predicted as candidate effectors (e.g. in LEV6574, only 3% of proteins with the motif were classified as effectors by EffectorP, while this was the case for 23% of the entire secretome). It is likely that the features of the chytrid RAYH-proteins are different from effector proteins from ascomycetes and basidiomyctes which were used to train the EffectorP tool. Also, the tool is optimized for recognition of putative apoplastic effectors. In the intracellulary obligate biotrophic *S. endobioticum* we expect to find an abundancy of cytoplasmatic effectors. The RAYH-proteins could act as cytoplasmatic effectors, which could explain why they were not detected by EffectorP.

This conserved motif could be important in interacting with other proteins or lipids, to relocate these RAYH-motif proteins into or within the host cell. The motif was not found in other chytrid species including the saprobic sister species *S. microbalum*. *S. endobioticum*-specific secreted proteins with the RAYH-motif are diverse and carry further localization signals, such as mitochondrial targeting peptides or a nuclear localization signal. Mitochondrial targeting peptides could allow targeting to the plant mitochondria to enhance susceptibility, a strategy that is used by several intracellular bacteria (e.g. *Vibrio cholerae*^48^, *Anaplasma phagocytophilum*^49^ and *Legionella pneumophila*^50^). Besides energy production, mitochondria are also involved in cellular processes such as programmed cell death, calcium homeostasis, biosynthesis of amino acids, lipids and nucleotides, and innate immune signaling against viruses and bacteria^51^. Effectors with nuclear localization signals have been observed in bacteria and fungi^52,53^, and were shown to induce transcriptional reprogramming, reviewed by Sinclair *et al*.^54^. We hypothesize that for an obligate intracellular pathogen such as *S. endobioticum*, manipulating the potato host cell by targeting both the mitochondria and nucleus directly is a likely scenario.

Verification of the function of these proteins will require innovative approaches since no transformation protocols are described for *S. endobioticum* and that the current bioassays for pathotyping and virulence testing are cumbersome and time consuming.

## Conclusions

This study is the first comprehensive comparative and functional genome analysis of chytrid fungi in general and *S*. *endobioticum* in particular, providing insights and understanding of fundamental biological questions in an economically important and a highly regulated plant pathogen. Particularly when working with obligate biotrophic organisms, the scientific community relies on the accuracy of computational tools for functional predictions to direct and design future experiments. The use of a redundancy in sequenced strains, combined with overlapping tools that are benchmarked, validated and verified, provide a robust basis for functional comparative studies of non-culturable organisms.

Two *S*. *endobioticum* isolates from different geographical locations representing different pathotypes were independently sequenced and functionally annotated. These were compared to the genomes of nine culturable chytrids, including the amphibian pathogen *Batrachochytrium dendrobatidis*, and six fungal organisms from other phyla of which three are axenically culturable, and three are obligate or facultative biotrophic. Analysis of these genomes resulted in several important observations linked to:

1. chytrid lifestyle and obligate biotrophy: functional analysis reflects the presence of flagella and associated (directed) motility in Chytridiomycota, which are absent in Ascomycota and Basidiomycota. The motile life stage of chytrids may allow them to be more selective and grow more efficiently (energy-wise) in a targeted (pathogenic) lifestyle or under low nutrient conditions. *S. endobioticum* lacks essential genes in the purine and pyrimidine pathways as opposed to the other chytrids analyzed including the saprobic sister species of *S. endobioticum*. We hypothesize that *S. endobioticum* relies on scavenging of the purine and pyrimidine bases from its host for survival, as was shown for pathogenic Mycobacteria which also cannot be cultured axenically;
2. genome plasticity: genome plasticity is often facilitated by repeats and mobile genomic elements, which have been associated with pathogens in many instances. Indeed, the pathogenic chytrid species *B. dendrobatidis* and *S. endobioticum* contain the largest amounts of complex repeats among chytrids. The chytrid genomes do not appear to be protected for genome duplications by RIP, yet they maintain their small genome size. As a sexual cycle has great impact on genome dynamics, we performed an *in-silico* analysis for the potential of sexual reproduction in chytrids. Genes from the meiotic toolbox are partially present, and a conclusive answer regarding a sexual cycle in chytrids remains elusive. We do provide the most comprehensive inventory of meiosis related genes in chytrids, which will foster the research on a sexual cycle in chytrid species; and
3. pathogenicity: essential to pathogens is avoiding detection by the host, thus preventing triggering immune responses. A reduced set of cell wall degrading enzymes was found in *S. endobioticum* relative to its saprobic sister species, which may reduce or prevent the triggering of defense responses as a result of DAMPs from cell wall degradation. In addition, remodeling of cell walls has been reported from other pathogens to avoid recognition of PAMPs by the host. This could be a viable strategy for both pathogenic chytrids. Proteins containing LysM domains were found enriched in *S. endobioticum*, which could be involved in chitin metabolism, or could have a function in the interference of chitin-triggered immunity, as was show for the arbuscular mycorrhizal fungus *Rhizophagus irregularis*. Effector proteins are expected to play a central role in the pathogenic lifestyle of *S. endobioticum* as they manipulate the host or its microbiome to facilitate infection. We identified a new class of candidate effectors specific to *S. endobioticum* that share a highly conserved RAYH-motif and gene architecture but also show isolate specific expansions and gene duplications.

In fungi, the focus for (genomic) research lies with representatives of Ascomycota and Basidiomycota. With this comparative study, we significantly increased the number of chytrid genomes and added the first economically important plant pathogen to the annotated Chytridiomycota genomes. They can now be included in comparative fungal genomic studies on a systematic basis as they provide a unique phylogenetic view and can shed new light on fundamental aspects of fungal biology.

## Materials and Methods

Detailed descriptions on materials and methods used are provided in Supplementary file 1, Material and Methods. In brief, the genomes of two *S. endobioticum* isolates (MB42 and LEV6574) were sequenced from DNA and RNA extracted from resting spores. Additionally, cultures of *C. confervae* (CBS 675.73), *P. hirtus* (CBS 809.83), *S. palustris* ( = *Phlyctochytrium palustre*) (CBS 455.65) and *S. microbalum* (JEL517) maintained on ARCH medium were sequenced. Sequence data was generated with one or more of the following techniques: Roche 454 GS-FLX Titanium, Illumina HiSeq, Illumina MiSeq, PacBio SMRT. A comparative read-mapping approach was used to identify *S. endobioticum* sequences from the metagenomic assemblies. Hybrid assemblies were obtained and RNAseq data was used in the structural annotation of the *S. endobioticum* genomes. Genomes were functionally annotated with InterProScan v5.1.6^55^ and data were deposited in NCBI GenBank under accession numbers QEAN00000000, QEAM00000000, QEAP00000000, QEAQ00000000, QEAR00000000, and QEAO00000000. Phylogenomic analyses were carried out using 192 Profile Hidden Markov Models (HMM) built from phylogenetically informative markers. The analysis and determination of CAZymes encoding genes were performed using a similar methodology described in^56^. For the analysis of meiotic toolbox genes, the methodology described in^21^ was followed. RepeatModeler^57^ was run on the individual genome sequences and consensus repeat models were created with 90% similarity to allow comparison of repeat content for the genomes under investigation. Presence of RIP was investigated with Occultercut^58^ and RIPcal^59^ and by determining the presence of the RID gene which is required for RIP. Protein motifs were identified in the LEV6574 secretome using MEME^60^, which were used to screen the entire protein set of all chytrid isolates using MAST^61^. Analyses results of different tools were intersected and verified to assess genomic features linked to the utilization energy sources, obligate biotrophy, cell wall degradation, pathogenicity and sexuality of chytrid fungi.

## Supplementary information

Supplementary file 1

Supplementary file 2

Supplementary file 3

Supplementary file 4

## Data Availability

Assembled and annotated genomes, and NextGen sequence data generated in this project manuscript are deposited in public databases NCBI and ENA. Accession numbers can be found in Table S1.
