# Supplementary material for: Comparative genomics of chytrid fungi reveal insights into the obligate biotrophic and pathogenic lifestyle of Synchytrium endobioticum
Source: Sci Rep. 2019 Jun 17;9:8672. doi: 10.1038/s41598-019-45128-9 (PMC6572847; doi:10.1038/s41598-019-45128-9)
Supplement: Supplementary file 1 [file 41598_2019_45128_MOESM1_ESM.pdf]

Supplementary file 1 with:

Comparative genomic analyses of chytrid fungi reveal insights into the obligate biotrophic and pathogenic lifestyle of *Synchytrium endobioticum*

Bart T.L.H. van de Vossenberg[1,2*#] & Sven Warris[1#], Hai D.T. Nguyen[3], Marga P.E. van Gent-Pelzer[1], David L. Joly[4], Henri C. van de Geest[1], Peter J.M. Bonants[1], Donna S. Smith[5], C. André Lévesque[3], Theo A.J. van der Lee[1]

* Corresponding author: b.t.l.h.vandevossenberg@nvwa.nl, 0031 317 496 911
# These authors contributed equally

1. Wageningen UR, Plant Science Group, Droevendaalsesteeg 1, Wageningen, the Netherlands
2. Dutch National Plant Protection Organization, National Reference Centre, Geertjesweg 15, Wageningen, The Netherlands
3. Agriculture and Agri-Food Canada, 960 Carling Avenue, Ottawa, Canada,
4. Université de Moncton, 18 avenue Antonine-Maillet, Moncton, Canada
5. Canadian Food Inspection Agency, 93 Mount Edward Road, Charlottetown, Canada

Contents

1. Supplementary Materials and Methods 2
2. Identification of *S. endobioticum* sequences from a metagenome assembly using “ZOO selection” 9
3. Genome synteny between *S. endobioticum* isolates MB42 and LEV6574 12
4. Phylogenomics 13
5. Gene Onthology (GO) term analysis 14
6. Kyoto Encyclopedia of Genes and Genomes (KEGG) pathway analysis 16
7. Carbohydrate active enzymes (CAZymes)....................18
8. Repeats and repeat induced point mutation (RIP) 20
9. Meiotic toolbox....................25
10. Candidate effector genes 26
11. Genome size comparison across major fungal phyla....................31
Literature....................32

1. Supplementary Materials and Methods

Fungal material and Next Generation Sequencing Potato wart material containing *S. endobioticum* pathotype 6(O1) isolate LEV6574 (SeLEV6574) was collected on cv. Russet Burbank in a field in St. Eleanors, Prince Edward Island, Canada in 2012. Potato wart material containing *S. endobioticum* pathotype 1(D1) isolate MB42 (SeMB42) was field collected in Langenboom, the Netherlands in 2002. This isolate is used as reference in the Dutch potato wart resistance testing program. Sampling, DNA extraction and Next Generation Sequencing of these isolates using one or more of three sequencing platforms, i.e. HiSeq 2500 (Illumina), MiSeq (Illumina), PacBio RSII (Pacific Biosciences), was previously described by Van de Vossenberg *et al.* [1]. In addition, four Roche 454 datasets from total genomic DNA of SeMB42 derived from purified resting spores as described previously [2], were produced. Total RNA was extracted from about 80 mg of purified LEV6574 spores using the Nucleospin RNA Plant kit (Machery-Nagel, Düren, Germany) following the manufacturer's instructions. Chilled spores were lysed in buffer RA1 (Machery-Nagel) containing 1 % beta-mercaptoethanol (Sigma-Aldrich) and 1 g of pre-chilled 2.0 mm zirconia beads (Biospec) for 1 min at room temperature. RNA was recovered from the lysate following the kit instructions and quantified using the Qubit RNA quantification system (ThermoFisher) followed by cDNA was preparation from 20 ng total RNA extracted from LEV6574 spores using the SMARTer PCR cDNA Synthesis Kit following the manufacturer's instructions (Clontech, Mountain View CA). The reverse transcription reaction was incubated for 110 min, and the cDNA was amplified for 20 cycles. The cDNA was sent to the London Regional Genomics Centre at the Western University (London, ON), where a Nextera XT (Illumina, San Diego CA) library was prepared and sequenced on a MiSeq (Illumina). For MB42, total RNA was extracted from about 1,5 gram of purified resting spores using RNeasy Plant mini kit (Qiagen) following manufacturer's instruction. Chilled spores were lysed in buffer RLC containing beta-mercaptoethanol and grinded droplet by droplet under liquid Nitrogen in a mortar using a pestle. Culturing, DNA extraction and Next Generation Sequencing of four culturable chytrid species, i.e. *Chytridium confervae* (CBS 675.73), *Powellomyces hirtus* (CBS 809.83), *Spizellomyces palustris* (=*Phlyctochytrium palustre*) (CBS 455.65) and *Synchytrium microbalum* (JEL517), was described in [1]. Sequencing information on materials used in this study are provided in table S1.

Genome assembly For SeLEV6574, both MiSeq and PacBio CCS and CLR reads were quality trimmed with Trimmomatic v0.35 [3]. Bbmap v35 was used to merge overlaps in MiSeq reads. These resulting single end MiSeq reads were mapped to the potato (*Solanum tuberosum*) genome [4] with bbmap v35. Unmapped reads were collected and considered not belonging to the host and potentially belonging to the pathogen, *S. endobioticum*. Using the unmapped MiSeq reads combined with CCS and CLR PacBio reads, a hybrid genome assembly was performed with SPAdes v3.8.1 [5] (k=21, 33, 55, 77, 99, 127) with error correction and mismatch correction enabled. Using BLAST, the assembled contigs were compared to a collection of bacterial genomes and again to the potato genome. Contigs that were > 90% identical and possessed an e-value of < 1e-50 to bacteria or potato were reported. These matches were manually inspected. Contigs, where the match length to a bacteria and

potato sequence divided by the total length of the contig were > 10%, were filtered out. For SeMB42, host derived reads were identified by mapping against the potato genome assembly [4] with BWA v7.5 [6], and were removed from the datasets. A hybrid assembly using 454 and HiSeq data was performed with Celera v7.0. [7] following the authors' recommendations for hybrid assemblies with Illumina and 454 data. HiSeq reads were used for base correction, resolving mis-assemblies and gap-filling with three iterations of the Pilon v1.16 assembly improvement tool [8]. Further inspection of contaminant contigs and scaffolds for both isolates was performed using a comparative read-mapping approach referred to as "ZOO selection" (see below). Contigs smaller than 1kb were omitted from the assemblies. Paired Illumina HiSeq data of chytrid species *C. confervae*, *P. hirtus*, *S. palustris*, and *S. microbalum* were used, after quality trimming (quality limit: 0.05, ambiguous limit: 2, min read length: 75), for *de novo* assembly with CLC genomics workbench v. 8.0.2 (word size: automatic, bubble size: automatic, scaffolding: on). Scaffolds were corrected using a read mapping approach (length fraction: 0.5, similarity fraction: 0.8) and consensus sequences were extracted for regions with ≥ 5x coverage.

ZOO selection As *S. endobioticum* cannot be cultured, generating sequence data for this fungus free from its host and other contaminants is virtually impossible. ZOO selection, which is a comparative read mapping approach containing three separate elements, was performed to identify *S. endobioticum* contigs and scaffolds from the metagenomic assemblies. The ZOO selection was performed on both assemblies of the *S. endobioticum* reference isolates MB42 and LEV6574. In both cases, the metagenomic assemblies of these isolates served as reference, and based on the mapping statistics of different datasets, true *S. endobioticum* scaffolds and contigs could be identified. The following datasets were used in the procedure: **A.** $ZOO_{mapping}$: Fourteen HiSeq or MiSeq datasets generated for nine *S. endobioticum* isolates comprising six different pathotypes (i.e. 1(D1), 2(G1), 6(O1), 8(F1), 18(T1) and 38(Nevsehir)); **B.** ZOO_{RNAseq}: RNAseq data from *S. endobioticum* isolates MB42 and LEV6574; and **C.** $ZOO_{heel\text{-}end}$: HiSeq data from 24 potato heel end samples (healthy or infected with pathogens other than *S. endobioticum*) (table S1). For each reference isolate the procedure was as follows: HiSeq and MiSeq sequence data were stringently mapped to the metagenomic scaffolds and contigs in CLC genomics workbench (length fraction: 0.8, similarity fraction: 0.9) and average coverage scores per scaffold were determined. For the analysis of DNA and RNA derived *S. endobioticum* sequence data ($ZOO_{mapping}$ and ZOO_{RNAseq}), normalisation of the data using median coverage values from regions with high correlation between the different isolates was performed per isolate (Fig. S1). As average read coverage of genomic scaffolds and contigs present in all *S. endobioticum* isolates are believed to have a uniform distribution, the median average coverage of the majority of scaffolds with a similar average coverage were used for normalization. For each scaffold-dataset combination, the absolute difference of the normalized coverage score to the median coverage value was determined. Per scaffold the sum of absolute differences for all datasets was calculated, and divided by the sum of normalized average read mapping coverage for all datasets. The resulting fractions were used to assign a $ZOO_{mapping}$ and ZOO_{RNAseq} score per scaffold. For the heel-end datasets, average coverage was directly used to assign the $ZOO_{heel\text{-}end}$ score. Total ZOO scores were calculated based on awards and penalties assigned to the individual ZOO scores: $ZOO_{mapping}$ (<0.9: 10; ≥0.9 – 1: 8; ≥1: 0), ZOO_{RNAseq} (<1: 10; ≥1 – 10: 4; ≥10: 0), and $ZOO_{heel\text{-}end}$ (0-0.1x: 0; ≥0.1 - 10x: -2; ≥10 - 100x: -5; ≥100 - 1000x: -10; ≥1000x: -20) (Fig. S2). Read mappings, blastn and blastx results were manually inspected for selected

strong candidates, all potential candidates and selected weak candidates. The effect of the ZOO selection was visualized with Blobology [9] using a custom script (github.com/blaxterlab/blobology/pulls) (Fig. S3).

Structural and Functional Genome Annotation Respective *S. endobioticum* RNAseq reads were mapped to their respective genome assembly with tophat v2.1.1 [10]. The resulting BAM files were used as input for the BRAKER1 pipeline [11] for structural annotation of both *S. endobioticum* isolates. Genomic contigs of *C. confervae* CBS 675.73, *P. hirtus* CBS 809.83, *S. palustris* CBS 455.65, and *S. microbalum* JEL517*,* generated in this study, and the publically available *H. polyrhiza* genome were structurally annotated using the MAKER pipeline v2.31.8 [12]. Within the MAKER pipeline, the Genemark-ET v4.10 program [13,14] was enabled and protein homology evidence from several closely related species, downloaded from JGI MycoCosm (*Batrachochytrium dendrobatidis* JAM81 v1.0, *Chytriomyces* sp. MP 71 v1.0, *Gaertneriomyces semiglobifer* Barr 43 v1.0, *Globomyces pollinis-pini* Arg68 v1.0, *Gonapodya prolifera* [15], *Rhizoclosmatium globosum* JEL800 Mondo [16], *Spizellomyces punctatus* DAOM BR117 v1.0 [17], *Anaeromyces robustus* S4 v1.0 [18], *Neocallimastix californiae* G1 v1.0 [18], *Piromyces finnis* v3.0 [18], *Piromyces* sp. E2 v1.0 [18], *Orpinomyces* sp C1A v1.0 [19]), as well as from *Synchytrium endobioticum* LEV6574 determined in this study, were provided to the pipeline for gene model prediction. Predicted gene models were validated using the NCBI tool tbl2asn and erroneous models were filtered out from the final annotations. Annotation completeness was assessed using version 2 of the fungal Benchmarking Universal Single Copy Orthologs (BUSCO) dataset [20]. Incomplete gene models and gene models with internal stop codons were removed from the dataset. For protein family classification, Gene Onthology (GO) analysis and the analysis of biochemical pathways, we used InterProScan v5.1.6 [21] to query multiple protein oriented databases such as Pfam v28.0, PANTHER v10.0, Phobius v1.01, SUPERFAMILY v1.75, PRINTS v42.0.

Genome statistics Basic genome statistics were determined with QUAST v4.5 [22]. Obtained genome sizes, gene lengths and mean exons counts were compared to 172 fungal and Oomycete genome sizes published in Mohanta and Bae [23] with the addition of publically available Chytridiomycota genomes (*Batrachochytrium dendrobatidis* JAM81 and JEL423, *Gonapodya prolifera* JEL478, *Homoloaphlyctis polyrhiza* JEL142, and *Spizellomyces punctatus* DAOM BR117).

Orthologous genes and functional analysis Orthologous genes shared between the two *S. endobioticum* isolates were identified using a Smith-Waterman alignment [24]. Inference of clusters of orthologous genes (COGs) was performed with OrthoFinder v1.1.4 [25] with default settings on protein datasets of both *S. endobioticum* and nine additional culturable chytrid species: *Chytridium confervae* (CBS 675.73), *Powellomyces hirtus* (CBS 809.83), *Spizellomyces palustris* (=*Phlyctochytrium palustre*) (CBS 455.65), *Synchytrium microbalum* (JEL517), *Batrachochytrium dendrobatidis* JAM81 and JEL423, *Gonapodya prolifera* JEL478, *Homoloaphlyctis polyrhiza* JEL142, and *Spizellomyces punctatus* DAOM BR117. Gene Onthologies (GO) and KEGG pathways for COGs specific to *S. endobioticum* and COGs absent in *S. endobioticum* but present in the culturable chytrid species were compared to COGs present in all isolates included (i.e. chytrid core COGs).

Phylogenetic Reconstruction Phylogenomic analyses were carried out following similar methodology described in Spatafora et al. [26]. Briefly, each of the 192 Profile Hidden Markov Models (HMM), built from phylogenetically informative markers [27], was searched against the predicted proteome from 59 species (table S3) across kingdom Fungi with hmmsearch from the hmmer3.1b package [28]. For each marker, a cutoff of 1e-20 was used to find the best scoring protein sequence in each species. Sequence alignment by profile HMM was carried out using hmmalign. These alignments were trimmed in two steps with trimAl v1.4rev15 [29], first with options -resoverlap 0.50 -seqoverlap 60, and then with the -automated1 option. The alignments were concatenated into a single super matrix alignment using the catfasta2phyml.pl script (Johan A. A. Nylander - https://github.com/nylander/catfasta2phyml). A maximum likelihood phylogenetic analysis was performed using RAxML v8.2.9 [30] with the fast bootstrap method (option -f a) and 100 bootstrap replicates. The -m PROTCATAUTO option was called to determine the best model of amino acid substitution. To assess potential conflict among markers, a phylogeny for each individual alignment was inferred with RAxML using the same parameters. The resulting trees were analyzed in Astral v4.10.12 [31] to construct a greedy consensus tree of the 100 bootstrapped replicate trees.

KEGG pathway analysis (see www.biorxiv.org/content/early/2018/07/16/369785). For the KEGG pathway comparison and InterProScan output validation, the results from the seventeen protein sets were processed using the comparePathway.py and controlPathway.py scripts (https://github.com/swarris/sendo). Both scripts use the KEGG API to associate EC to KO annotations and count the number of times an EC was predicted. For the comparison, included isolates were grouped in four categories: obligate biotrophic chytrids (OCh; i.e. *S. endobioticum*); culturable chytrids (CCh); culturable higher fungi (CHF); and obligate or facultative biotrophic higher fungi (OHF). The coloring in the resulting PDFs per pathway is based on the group each organism belongs to (table 1).

Table 1. Coloring scheme for the pathway PDFs based on the InterProScan output of all seventeen protein sets. A '+' means the EC was found in that particular group, whereas '-' indicates an EC was not predicted for one or more isolates in that group. Grey was used to color other undefined combinations.

Group	Species in group	EC Coloring						
		**Green**	**Orange**	**Blue**	**Purple**	**Light blue**	**White**	**Grey**
OCh	*S. endobioticum*	+	-	+	-	+	-	Other
CCh	*S. microbalum, C. confervae, B. dendrobatidis, H. polyrhiza, P. hirtus, S. punctatus, S. palustris, G. prolifera*	+	+	-	+	-	-	
CHF	*S. cerevisiae , N. crassa , C. neoformans*	+	+	-	+	-	-	
OHF	*U. maydis , P. graminis* f.sp. *tritici , M. larici-populina*	+	-	+	+	-	-	

To benchmark the analysis, results from our pipeline were compared to the reference pathways in the KEGG database for the six fungal organisms with known pathways (CHF, OHF). The coloring in the resulting PDFs for the benchmark results were as follows: true positives: blue; false positives: red; false negative: light blue; and true negative: white. The counts for the reference pathways of the 6 control species were used to calculate the sensitivity, specificity and accuracy of the InterProScan predictions (pathwayOverview.rmd) using the following formulas:

sensitivity or true positive rate (TPR): $TPR = \frac{TP}{TP+FN}$

specificity or true negative rate (TNR): $TNR = \frac{TN}{TN+FP}$

accuracy (ACC): $TPR = \frac{TP+TN}{TP+TN+FP+FN}$

Definitions

True positive (TP): element in the reference pathway correctly predicted by InterProScan

True negative (TN): element in the not reference pathway and not predicted by InterProScan

False positive (FP): element in the not reference pathway but predicted by InterProScan. Note: this could also be a valid new prediction

False negative (FN): element in the reference pathway but not predicted by InterProScan.

Some elements in the pathways have no EC number record in the database. These cannot be mapped to the InterProScan output containing only EC numbers and are left out of the calculations.

GO-term analysis The GO-term analysis is available from www.biorxiv.org/content/early/2018/07/16/369785 (last accessed 13 February 2019).

Cazymes The analysis and determination of Carbohydrate-active enzymes (CAZymes)-encoding genes were performed using a similar methodology described in Zerillo et al. [32]. Briefly, the CAZymes for the 11 chytrids and control species were predicted using the online tool dbCAN [33], which searches for protein domains signatures search of CAZymes, via hidden Markov models constructed for each of the CAZy families, based on the CAZy (Carbohydrate-Active Enzyme) database classification [34], using default parameters. The CAZymes were categorized according to the type of reaction catalyzed (table S10): carbohydrate esterases (CE), glycoside hydrolases (GH), glycosyl transferases (GT), polysaccharide lyases (PL) and carbohydrate-binding modules (CBM), as described by [34]. Clustering based on the CAZyme results was done by counting the number of times a particular CAZyme was found in the protein set. These counts were subsequently normalized by dividing the counts by the total number of proteins in the dataset. The resulting matrix was clustered using pvclust in R (http://stat.sys.i.kyoto-u.ac.jp/prog/pvclust/).

Meiotic toolbox and mating type genes Protein sequences of each chytrid species were formatted into a BLAST database to identify genes involved in meiosis, following the methodology described by [35]. Meiotic genes in *Saccharomyces cerevisiae* and *Cryptococcus neoformans* were chosen as input queries (e-value < 1E-5) for the individual protein BLAST databases. When no blast hits were obtained with the *S. cerevisiae* and *C. neoformans* proteins, the analysis was repeated with *Allomyces macrogynes* orthologs. Absence of meiotic genes was verified with tblastn and manual curation (table S11). Presence of mating type genes was determined similarly (e-value < 1E-20), using six representative references for *Mat1-1* (AJD14755, AAR00949, EME86922, AEZ02234, AEZ02255, AAO37757) and six references for *Mat2-1* (AIE44386, MAT2_COCSA, AIE44390, AIE44395, MAT2_COCHE, ABO72590).

Analysis of repeats and RIP activity RepeatModeler v1.0.4 [36] was run on the individual genome sequences with default settings. Consensus repeat models were created with a 90% similarity for the combined chytrid species to allow comparison of repeat content shared between the species under investigation. Repeat sequences were masked with RepeatMasker v4.0.7 [37]. Repeats unique to *S. endobioticum* with repeat models of ≥1kb were selected and aligned using MAFFT [38] incorporated in Geneious R10 [39]. Repeat sequences in the alignment with complete gene models, gene size ≥ 800 nt, and similarity ≥80% were extracted from the alignment, realigned and tested for signatures of RIP activity using RIPcal [40] using the degenerate consensus sequence as reference. Putative RID proteins were identified using protein domains identified by InterProScan, and by blastp of known RID proteins of 18 fungal isolates (e-value < 1E-5) (table S13).

Analysis of candidate effectors Secretomes were defined in a broad sense as proteins having a secretion signal peptide as determined by SignalP v4.1 [41], lacking transmembrane domains as determined by TMHMM2.0 [42]. From the secretome, motifs were identified with MEME [43] which used screen the entire protein set with MAST [44]. Subcellular location of chytrid proteins was determined using TargetP v1.1 [45]. Small secreted candidate effector proteins were identified using EffectorP [46], ApoplastP [47], and LOCALIZER [48]. Candidate effector protein sequences were aligned per COG with MAFFT, and a consensus alignment was created for the complete protein set in Geneious. A Maximum Likelihood phylogeny was performed on the protein alignment in CLC genomics workbench using the WAG substitution model with 500 bootstrap replicates.

2. Identification of *S. endobioticum* sequences from a metagenome assembly using “ZOO selection”

ZOO selection is a comparative read mapping approach used to identify scaffolds and contigs for your species of interest from a metagenomic assembly. To allow comparison of the average read mapping coverage per scaffold or contig for the different *S. endobioticum* isolates included in the analyses, normalization of the data was applied. Particularly for the SeMB42 genome, a selection had to be made for scaffolds suitable for normalization as initial experiments showed that more contaminant scaffolds were present in the pre-ZOO selected assembly.

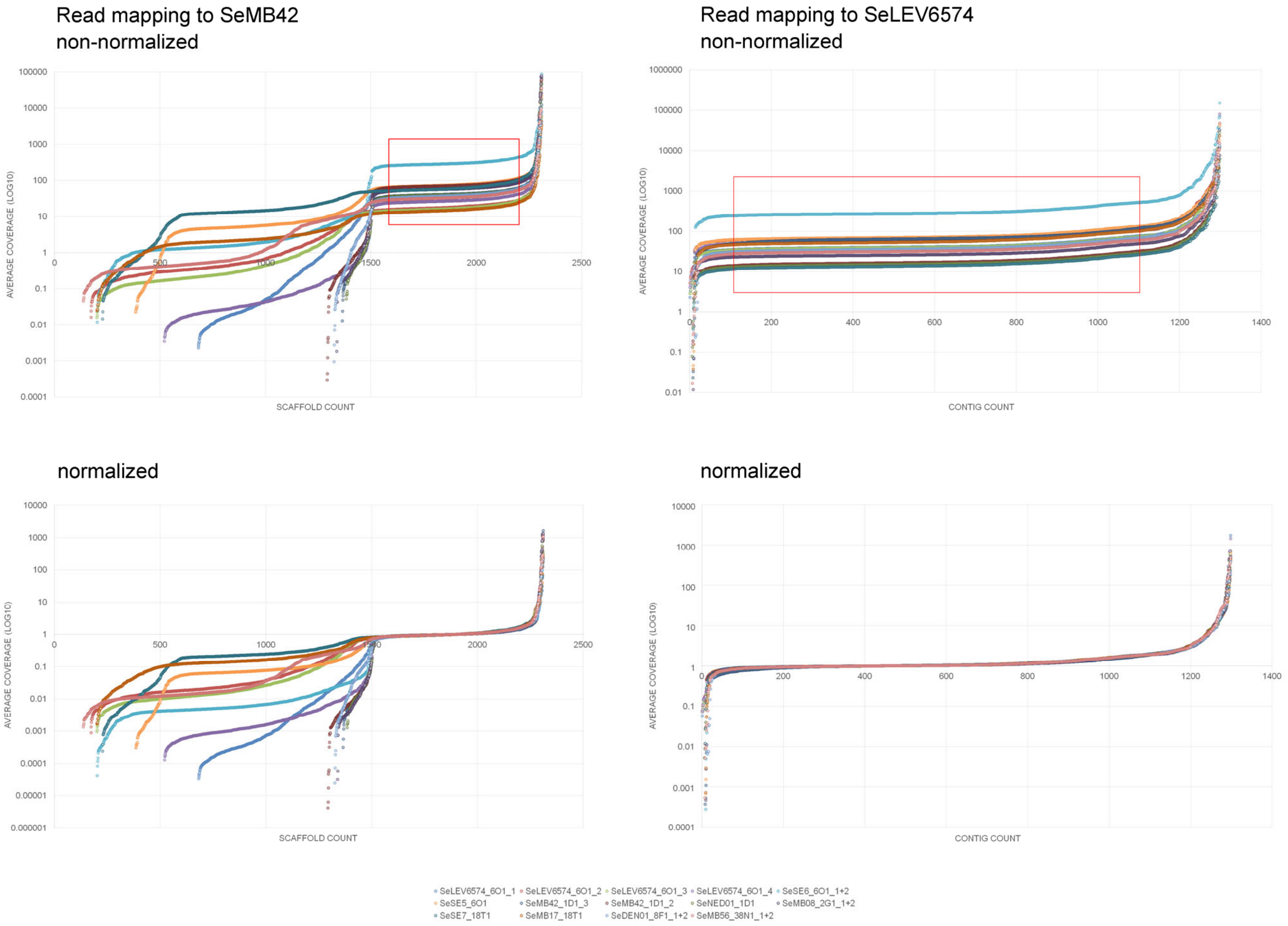

Figure S1. Selection of scaffolds and contigs for normalization (top) and the effect of normalization on distribution of average read coverage (bottom). Per isolate, average read mapping coverage values were sorted low to high for the 2310 SeMB42 pre-ZOO scaffolds and 1298 pre-ZOO SeLEV6574 contigs. Based on the similarities in average read coverage between isolates, areas boxed in red were believed to be true *S. endobioticum* sequences and were used for normalization.

For each of the three read mapping elements in the ZOO selection (i.e. 1. $ZOO_{mapping}$: Fourteen HiSeq or MiSeq datasets generated for nine *S. endobioticum* mapped to metagenomic scaffolds and contigs; 2. ZOO_{RNAseq}: RNAseq data from *S. endobioticum* isolates MB42 and LEV6574 mapped to metagenomic scaffolds and contigs; and 3. $ZOO_{heel\text{-}end}$: HiSeq data from 24 potato heel end samples (healthy or infected with pathogens other than *S. endobioticum*) mapped to metagenomic scaffolds and contigs), scores and penalties were awarded resulting in a total ZOO score (Fig. S2). These scores were used to identify strong, potential and weak candidate *S. endobioticum* derived sequences. Potential and selected weak candidate scaffolds and contigs were manually checked to determine if they were derived from *S. endobioticum*.

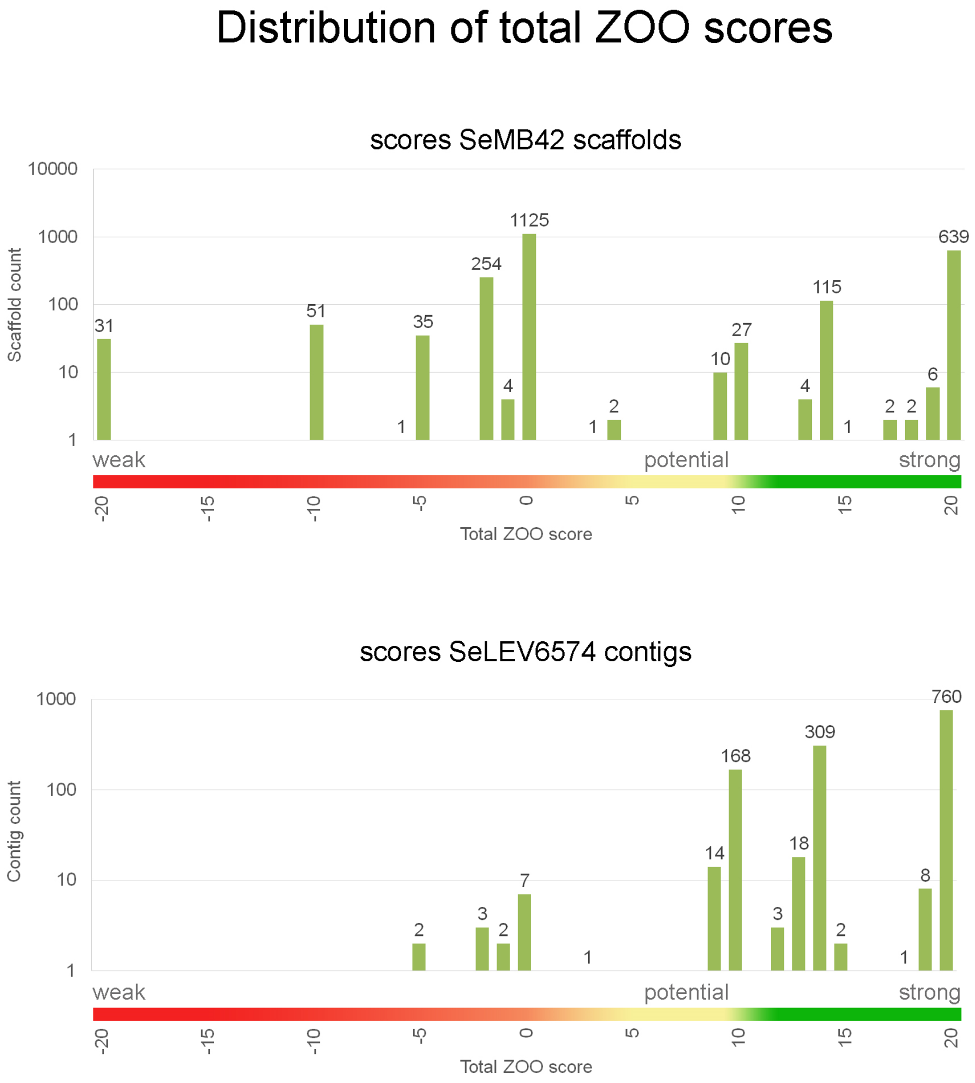

Figure S2. Total ZOO scores for the 2310 SeMB42 pre-ZOO scaffolds (top) and 1298 pre-ZOO SeLEV6574 contigs (bottom). The number of scaffolds or contigs with a particular score is presented above the bars. The higher the total ZOO score, the more likely the scaffold truly belongs to *S. endobioticum*: total ZOO score of ≥12 were considered strong candidates, 1-11 were regarded as potential candidates, and scaffolds with total ZOO scores ≤ 0 were considered as weak candidates.

Based on the ZOO scores and manual verification of potential and selected weak candidate scaffolds and contigs, a post-ZOO selection was made representing *S. endobioticum* derived sequences. The effect of this selection is visualized in figure S3.

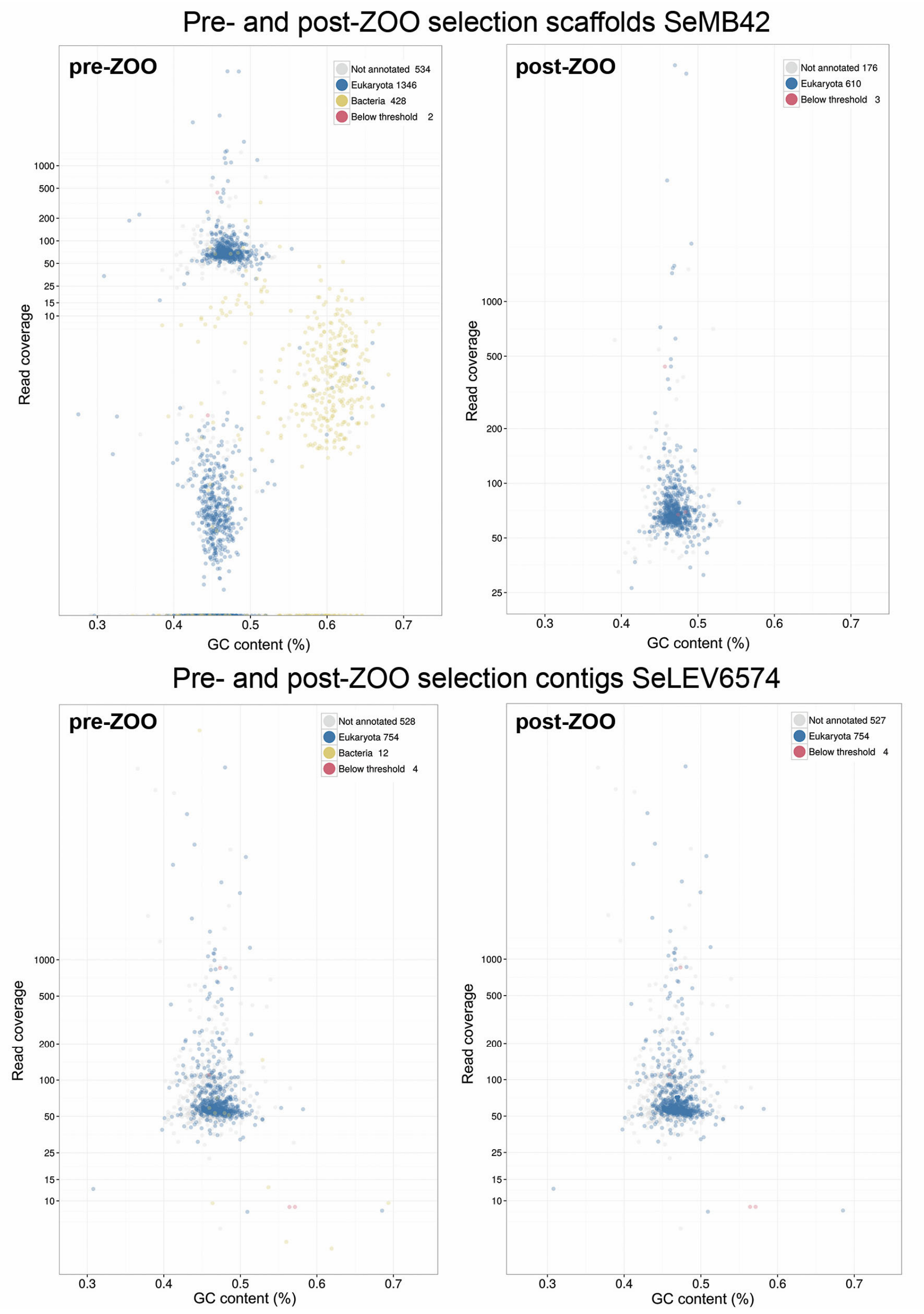

Figure S3. Effect of the ZOO selection illustrated with Blobology plots: 2310 pre-ZOO and 786 post-ZOO SeMB42 scaffolds (top), and 1298 pre-ZOO and 1285 post-ZOO SeLEV6574 contigs (bottom). Dots in the plots represent individual scaffolds or contigs which are plotted by read coverage (Y-axis) in function of GC content (X-axis). Kingdom level blastn based identification is used to color individual scaffolds. Scaffolds and contigs with the annotation “unassigned” did not produce a blastn hit, and scaffolds and contigs with the annotation “below threshold” did produce a blastn hit, but this hit was obtained with sequences smaller than 200 bp.

3. Genome synteny between *S. endobioticum* isolates MB42 and LEV6574

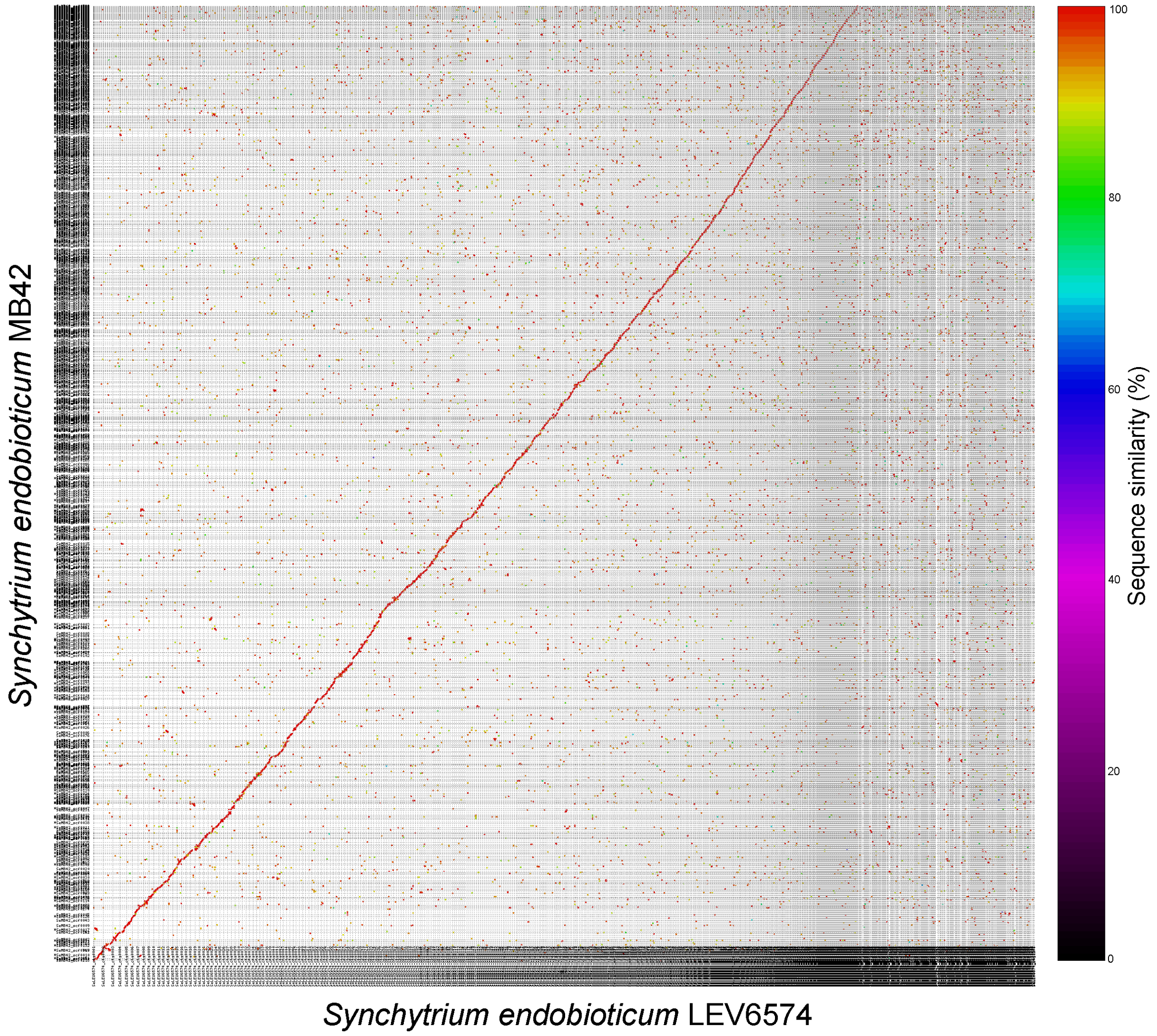

Figure S4. Mummerplot showing synteny between *S. endobioticum* pathotype 1(D1) isolate MB42 and pathotype 6(O1) isolate LEV6574. The 23.21 Mb genome of SeLEV6574 (x-axis, contigs sorted by sequence length in descending order) was used as reference for alignment of scaffolds from the 21.48 Mb SeMB42 genome (y-axis). The color scale indicates the sequence similarity between the two genomes, and the majority of sequences is (close to) 100% identical.

4. Phylogenomics

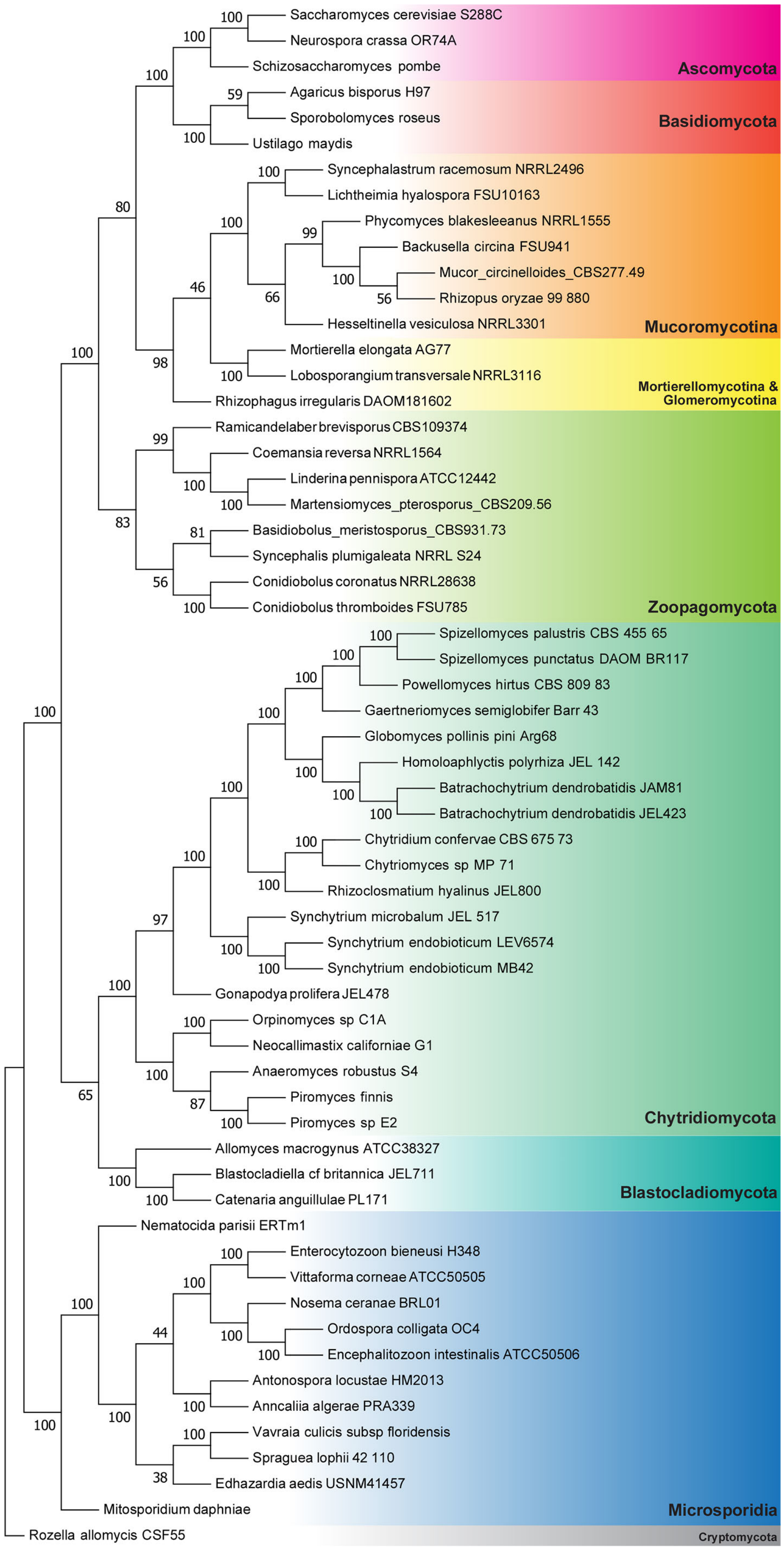

Figure S5. ASTRAL greedy consensus cladogram based on analyses of individual bootstrap trees for each of 192 conserved orthologous proteins. Support values show the percentage of bootstrap replicates that contain that branch.

5. Gene Onthology (GO) term analysis

Chytridiomycota possess flagella, whereas higher fungi lack these cellular structures. The difference in this biological feature was used as a control for the GO-term analysis performed. Indeed, GO-terms linked to flagella and movement were exclusively found in Chytridiomycota and not in the higher fungi analyzed.

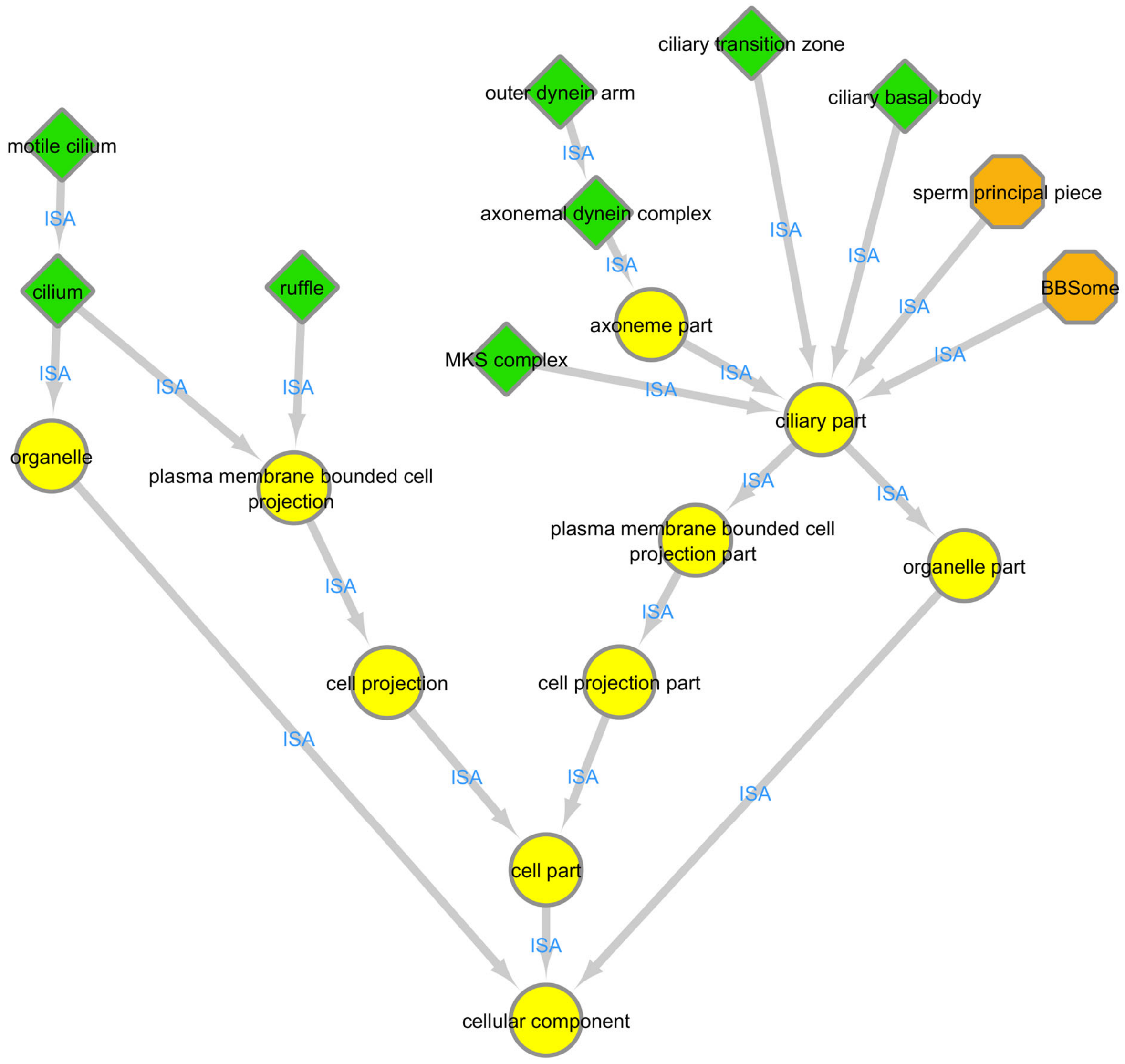

Figure S6. Cellular component GO-terms associated to flagella and movement, and their occurrence in chytrid species and higher fungi (Ascomycota and Basidiomycota) analyzed visualized with CytoScape. Shapes in the network indicate the different GO-terms with “Cellular Compartment” being the highest level term. Grey arrow indicate an “is a” relation between the connected GO-terms. The different shapes indicate if a given GO-term is present in culturable and obligate biotrophic chytrid species but not in higher fungi (green ◆), present in one or more culturable chytrid species (orange ●), and unclassified (yellow ●).

At level 2 biological processes GO-terms, 26 terms were identified in *S. endobioticum* specific genes, and genes present in culturable chytrid species but absent in *S. endobioticum*. These terms were also predicted for the chytrid core genes, and could be grouped under six level 1 terms of which metabolic processes were the most abundant (Fig. S7). At level 2, no GO-terms unique to *S. endobioticum* specific genes, and genes present in culturable chytrid species but absent in *S. endobioticum* were observed.

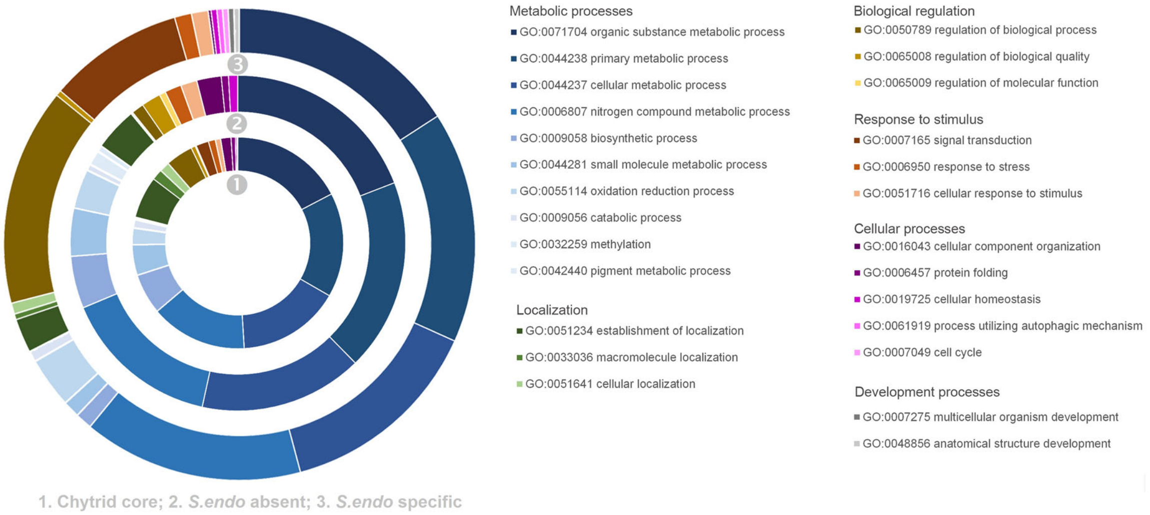

Figure S7. Predicted level 2 biological processes GO-term functions of genes ❶ shared by all chytrid species (1848 COGs), ❷ present in all chytrids except in *S. endobioticum* (76 COGs), and ❸ unique to *S. endobioticum* (1413 COGs). Level 2 terms are grouped are under respective level 1 biological processes terms (Metabolic processes: blue, Localization: green, Biological regulation: brown, Response to stimulus: red, cellular processes: pink, developmental processes: grey) and sorted on high to low incidence.

6. Kyoto Encyclopedia of Genes and Genomes (KEGG) pathway analysis

The KEGG pathway analysis was benchmarked using three culturable fungal organisms (*Saccharomyces cerevisiae*, *Neurospora crassa*, and *Cryptococcus neoformans*), and three obligate or facultative biotrophic organisms (*Ustilago maydis*, *Melamspora larici-populina*, and *Puccinia graminis* f.sp. *tritici*) with known biochemical pathways included in the KEGG database. Overall accuracy (84.5%) was mainly influenced by false negative results. This is illustrated by the aconitate hydratase (EC:4.2.1.3) (Fig S9), an enzyme from the citrate (TCA) cycle interconverting citrate and cis-aconitate, which was detected in all of the genomes analyzed using InterProScan, but was not colored in the KEGG pathway because of a missing link in the KEGG database

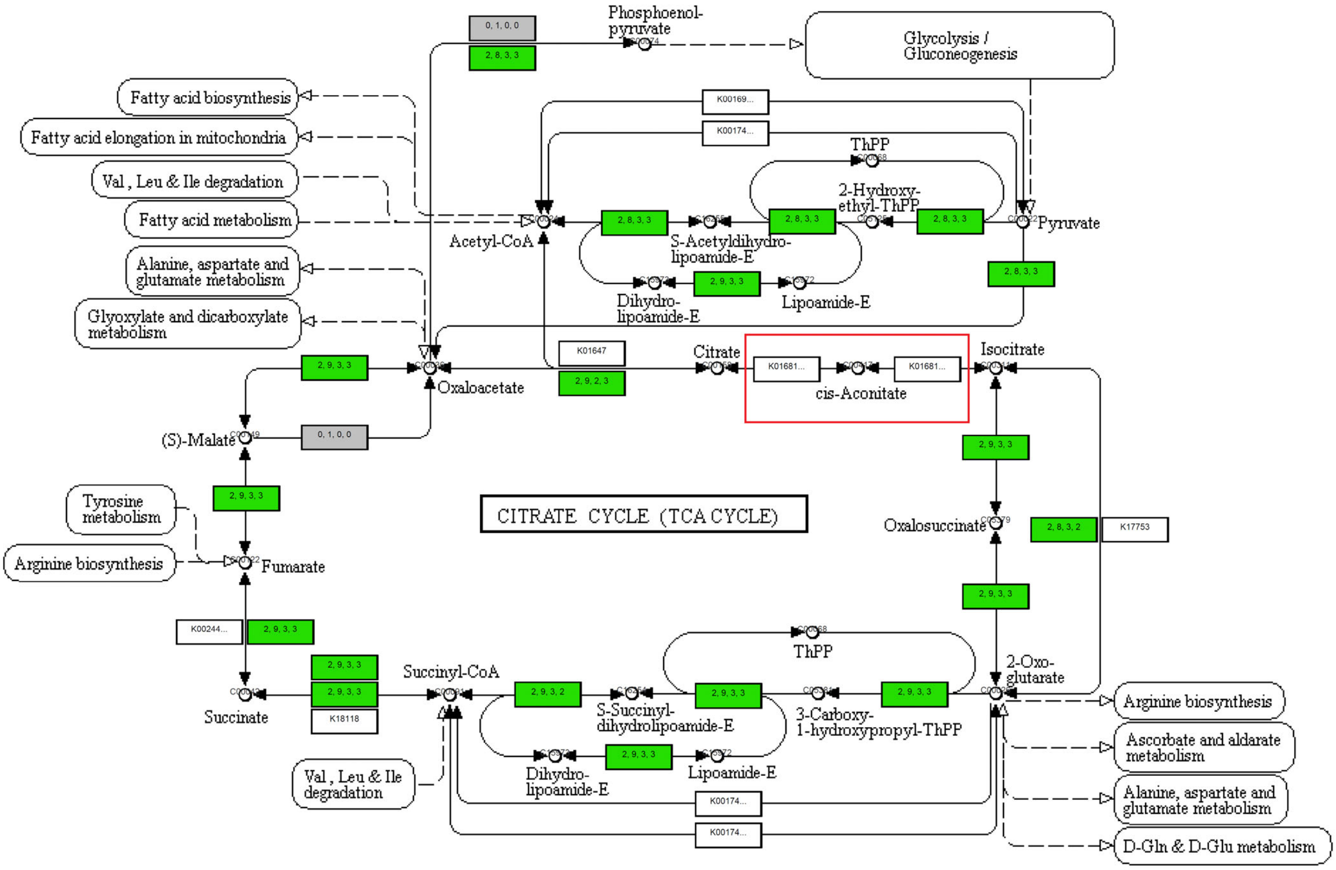

Figure S8. KEGG reference pathway 20: citrate cycle (TCA). Numbers (*a, b, c, d*) in the enzymatic steps indicate the number of isolates in a given group for which the corresponding gene was detected, in which *a*: obligate biotrophic chytrids (OCh, max = 2); *b*: culturable chytrids (CCh, max = 9); *c*: culturable higher fungi (CHF, max = 3), and *d*: obligate or facultative biotrophic higher fungi (OHF, max = 3). The maximum score that can be obtained is “2,9,3,3”. Colors indicate which groups (OCh, CCh, CHF, OHF) are represented for a given enzymatic step: present in ≥1 isolate in OCh, CCh, CHF and OHF (green); present in ≥1 isolate but not in a combination of interest linked to lifestyle or taxonomic placement (see supplementary file 3) (grey); unassigned (white). Boxed in red is the false-negative aconitate hydratase (EC:4.2.1.3).

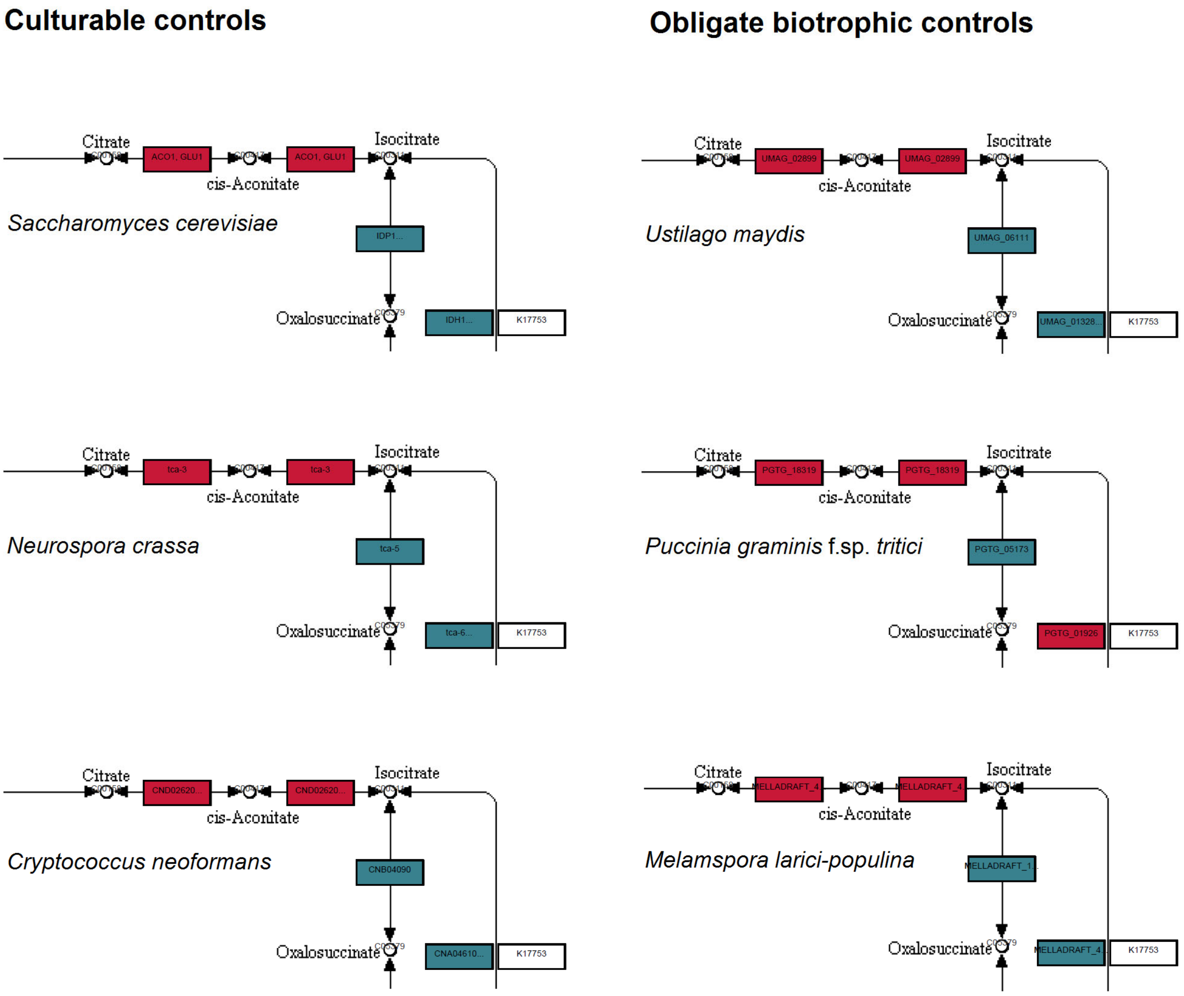

Figure S9. Detail KEGG reference pathway 20: citrate cycle (TCA) for six Ascomycota and Basidiomycota species with known KEGG pathways, focusing on aconitate hydratase (EC:4.2.1.3). Blue colors represent true positives, i.e. the element is described in the reference pathway and was correctly predicted in our analysis. Red colors represent false negatives, i.e. the element is described in the reference pathway but was not predicted in our analysis. White enzymatic steps are unassigned.

7. Carbohydrate active enzymes (CAZymes)

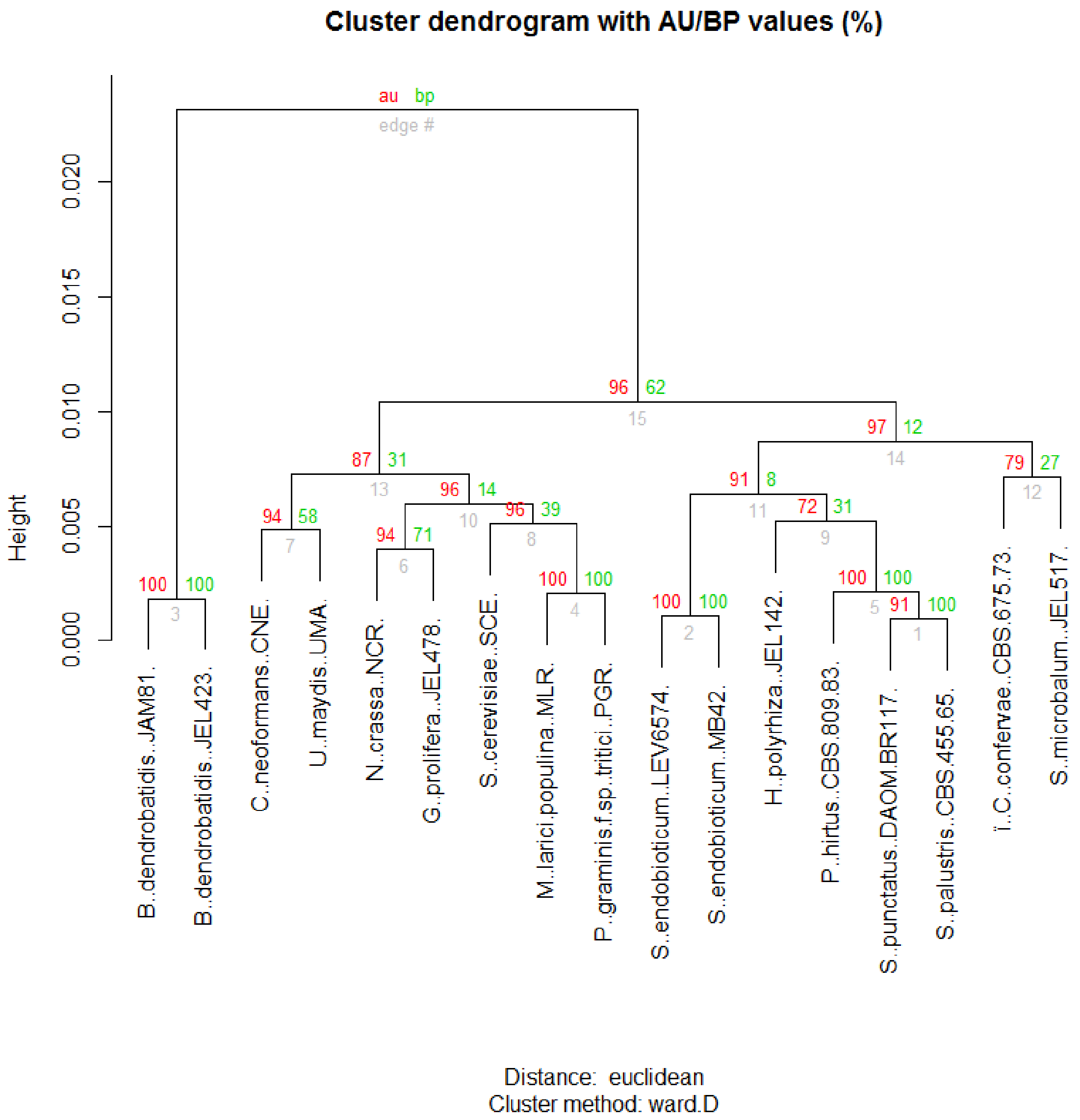

Figure S10. Cluster analysis based on all assigned CAZyme modules. Clustering was performed by pvclust (http://stat.sys.i.kyoto-u.ac.jp/prog/pvclust/) in R. AU = Approximately Unbiased p-value and BP = Bootstrap Probability value.

By combining the KEGG, GO and CAZyme annotations an increased reliability of the predicted functions of genes analyzed was found. However, in some cases discrepancies between the different sources also became apparent, making definitive conclusions in these cases difficult. The complexities that can be encountered of is illustrated below.

The KEGG analysis of *S. endobioticum* shows that the enzymes for degrading cellulose are missing (EC 3.2.1.4 and EC 3.2.1.91) of which endoglucanase (EC 3.2.1.4) was found in eleven of the fifteen other isolates, which suggests endoglucanase could indeed be missing in *S. endobioticum*. However, cellulose 1,4-beta-cellobiosidase (EC 3.2.1.91) was not detected with InterProScan for all isolates analyzed, but it should be found in at least the control species. Hence the missing of enzyme EC 3.2.1.91 is a false negative in the control species, and the presence or absence of this enzyme in *S. endobioticum* cannot be deduced based on our data.

When connecting KEGG to GO, the KEGG database shows that the GO Term ‘cellulase activity’ (GO:0008810) is associated with endoglucanase (EC 3.2.1.4) and the GO Term ‘cellulose 1,4-beta-cellobiosidase activity’ (GO:0016162) is associated with cellulose 1,4-betacellobiosidase (EC 3.2.1.91). The InterProScan output indicates that these GO Terms, similar to the enzymes, are both missing in *S. endobioticum*.

When intersecting CAZyme predictions with KEGG and GO output, this further complicates the story further. The CAZyme family ‘Glycoside Hydrolase Family 7’ (GH7) is linked by InterProScan to both EC-numbers (EC 3.2.1.4 and EC 3.2.1.91), but not to the mentioned GO Terms (GO:0008810 and GO:0016162) which are linked to GH12 in the InterPro database.

- The KEGG website states, however, that GH7 is linked to ‘carbohydrate metabolic process’ (GO:0005975) and ‘hydrolase activity, hydrolyzing O-glycosyl compounds’ (GO:0004553). The latter, GO:0004553, is the parent GO term of both GO:0008810 and GO:0016162 and is found abundantly in all species, including *S. endobioticum*. This indicates that the more specific GO terms which were not detected in *S. endobioticum* by InterProScan are likely to be false negatives.
- According the the CAZyme website, enzymes belonging to the GH7 family are linked to four EC-numbers including EC 3.2.1.4, but not EC 3.2.1.91.

These results show that links of terms and features are not consistent between the different databases. So, relying on a single data source is not recommended to make definitive conclusions and even when using multiple sources and should not be regarded as absolute truths as these databases can be contradictory or give inconclusive results.

8. Repeats and repeat induced point mutation (RIP)

Repeat content of the chytrid genomes analyzed was determined with RepeatModeler [36]. Models were built on repetitive sequences the combined chytrid isolates to allow comparison of repeat content across the different species. Apart from small simple repeats (SSRs), larger complex repeats were identified which were grouped in 399 repeat families. Both *S. endobioticum* and *B. dendrobatidis* have the highest repeat content in their genomes which mainly consists of complex repeats: 77.6 – 89.9% in both pathogenic species opposed to 15.6 – 35.9% in the other chytrid genomes. Occurrence of repeat families in the different chytrid genomes analyzed is show in figure S15.

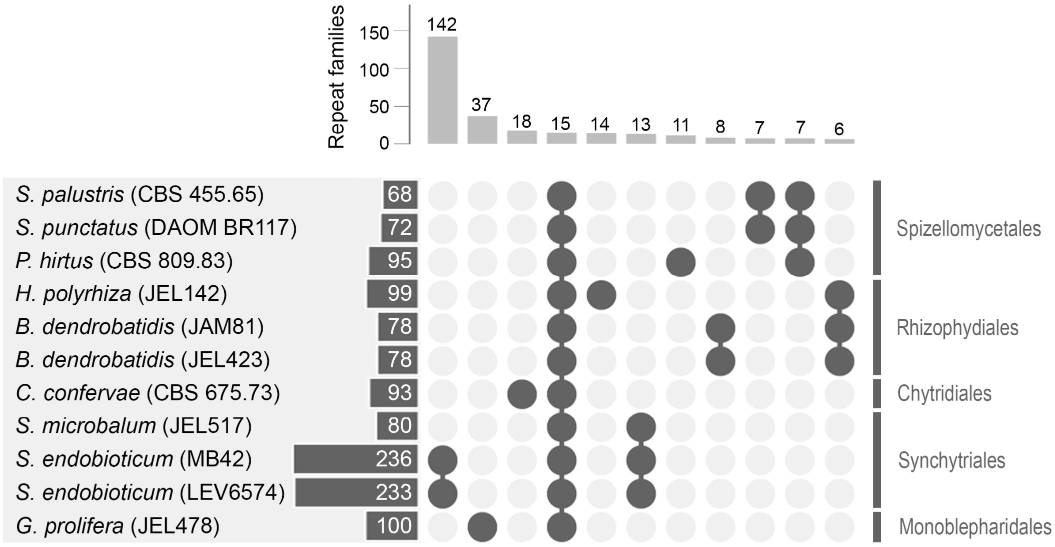

Figure S11. Repeat family content in *S. endobioticum*, *B. dendrobatidis*, *C. confervae*, *G. prolifera*, *H. polyrhiza*, *P. hirtus*, *P. palustre*, *S. microbalum*, and *S. punctatus* (sorted based on their taxonomical classification)**.** Horizontal bars indicate the number of repeat families in the respective isolates. Vertical bars indicate the number of repeat families in intersecting selections. Intersections with more than five repeat families are shown sorted from the most abundant to the least abundant repeat families. With 236 and 233 different repeat families, *S. endobioticum* isolates MB42 and LEV6574 have the highest diversity of complex repeats on their respective genomes. In addition, the largest intersection of repeat families, with 142 different repeat families, is specific to *S. endobioticum*. This in contrast to 15 repeat families being shared by all chytrid genomes, and only eight being shared by both *B. dendrobatidis* genomes.

As repeat-induced point mutation (RIP) targets repetitive DNA sequences (≥ 0.4kb in length with ≥80% sequence similarity in *N. crassa*) in fungi reducing their GC content, AT-rich regions could be signatures of RIP activity. The Occultercut tool [49] was used to detect AT-rich regions and to identify potential RIP affected areas in the chytrid fungal genomes. Potential RIP affected regions would be recognized as AT-rich segments (see Fig. S14, *N. crassa*).

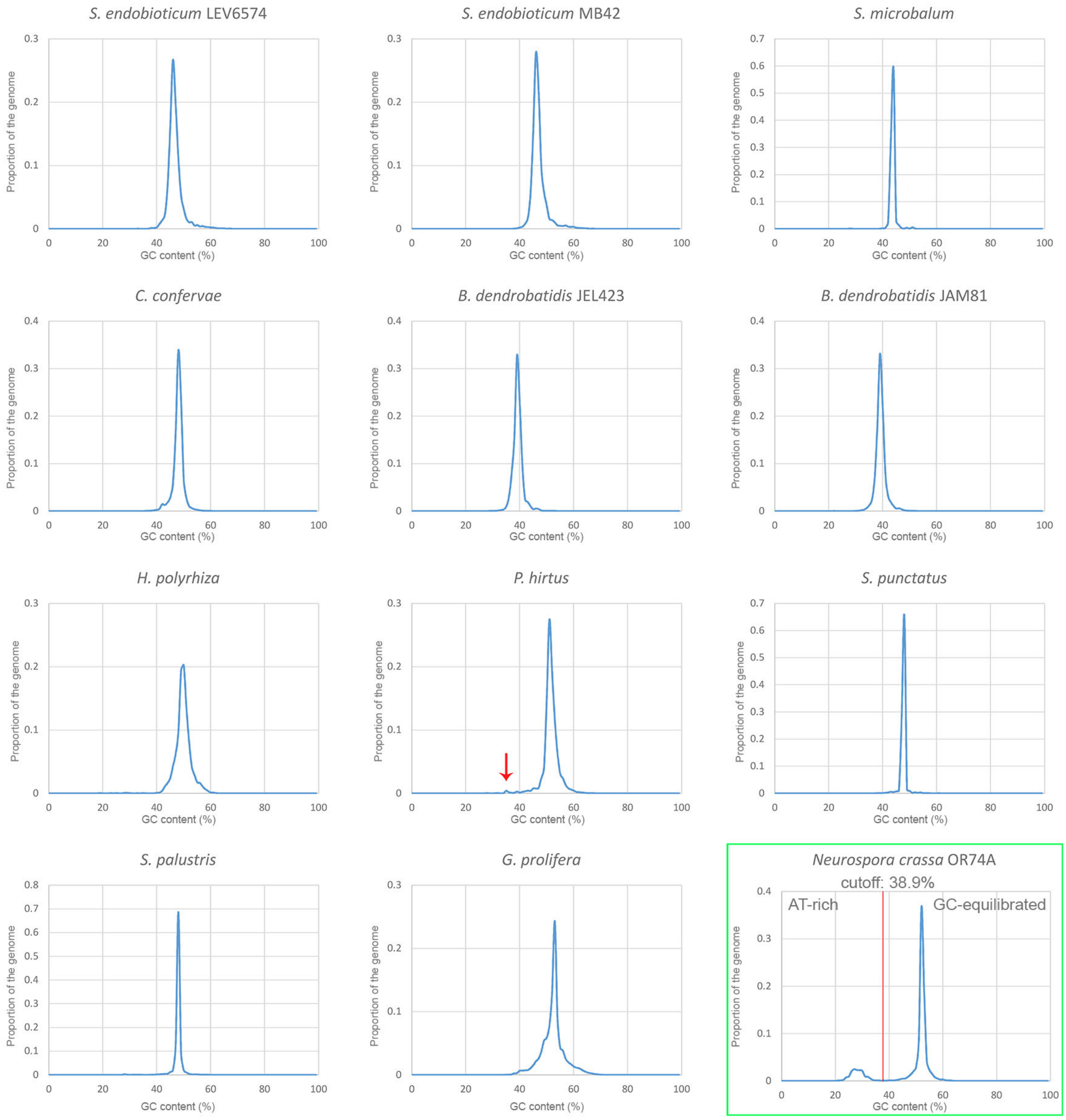

Figure S12. GC-content (x-axis) and their frequencies (y-axis) in eleven chytrid genomes determined with Occultercut. In the chytrid species analyzed, no AT-rich segments could be identified and GC-content plots show unimodal distribution. In the RIP proficient *N. crassa* genome (boxed in green), categorization of AT-rich segments and GC-equilibrated could be performed (cutoff: 38.9% GC-content). In *P. hirtus*, a second peak was identified (red arrow) but no categorization of AT-rich segments and GC-equilibrated could be made. This is likely the result of AT-rich segments overlapping too much with GC-equilibrated segments as described by [49]. The latter would suggest the AT-rich proportion of the *P. hirtus* genome is not the result of RIP activity.

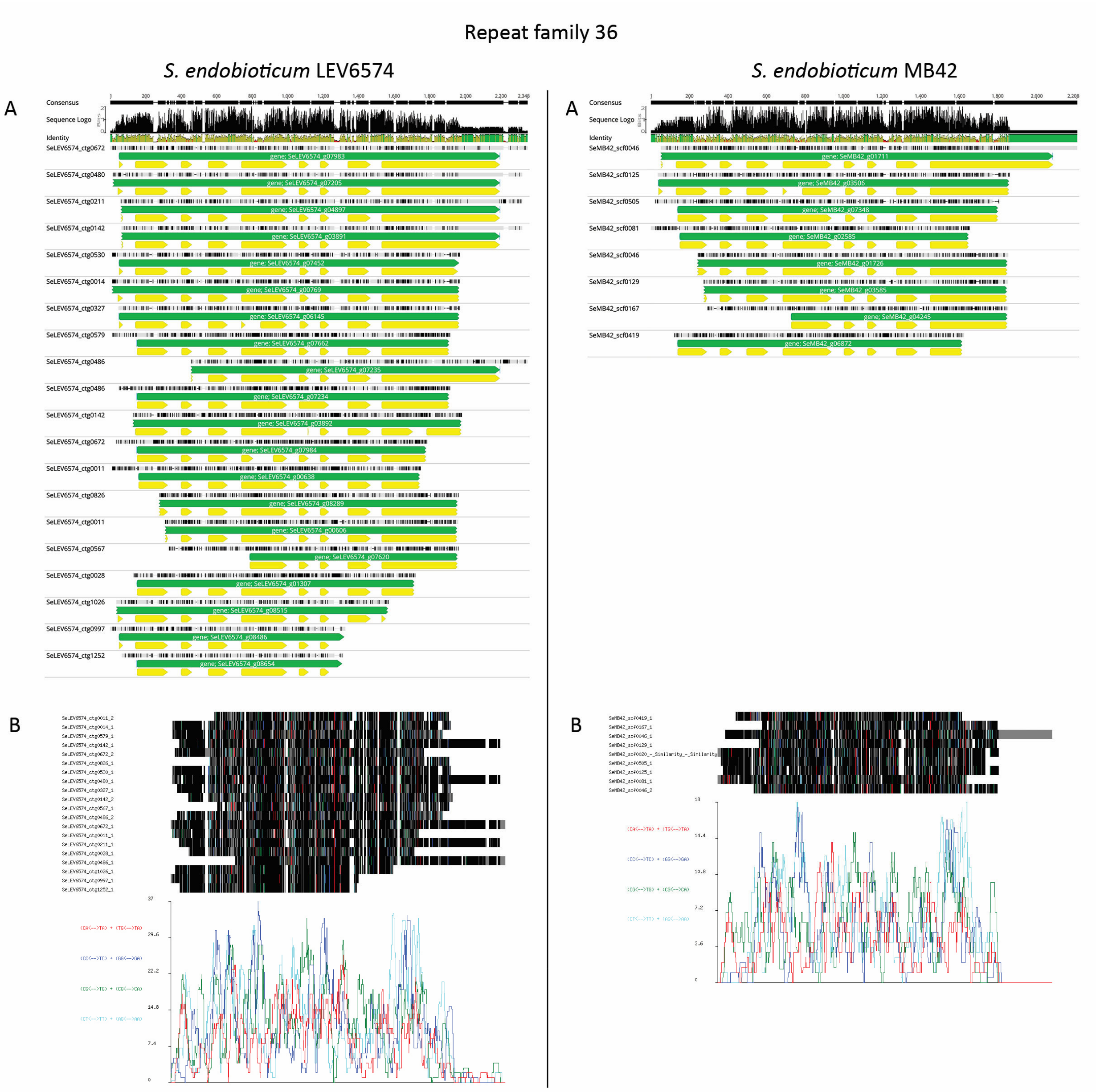

Figure S13. A. MAFFT alignment of repeat sequences identified as "family 36" containing (near) complete gene models of *S. endobioticum* isolates LEV6574 (left) and MB42 (right), representing 20 and 8 sequences with alignment lengths of 2348 bp and 2208 bp respectively. Gene annotations are green whereas coding sequences are annotated in yellow. In the alignment, differences to the overall consensus sequence are highlighted in black. **B.** RIPcal analysis of the alignments presented under **A** using the degenerate consensus sequence as reference. Substitution frequencies between CpA – TpA + TpG – TpA (red), CpC – TpC + GpG – GpA (dark blue), CpG – TpG + CpG – CpA (green), and CpT – TpT + ApG – ApA (light blue) are similar and no dominance of CpA – TpA mutations (red) which are typical for RIP [40] was observed.

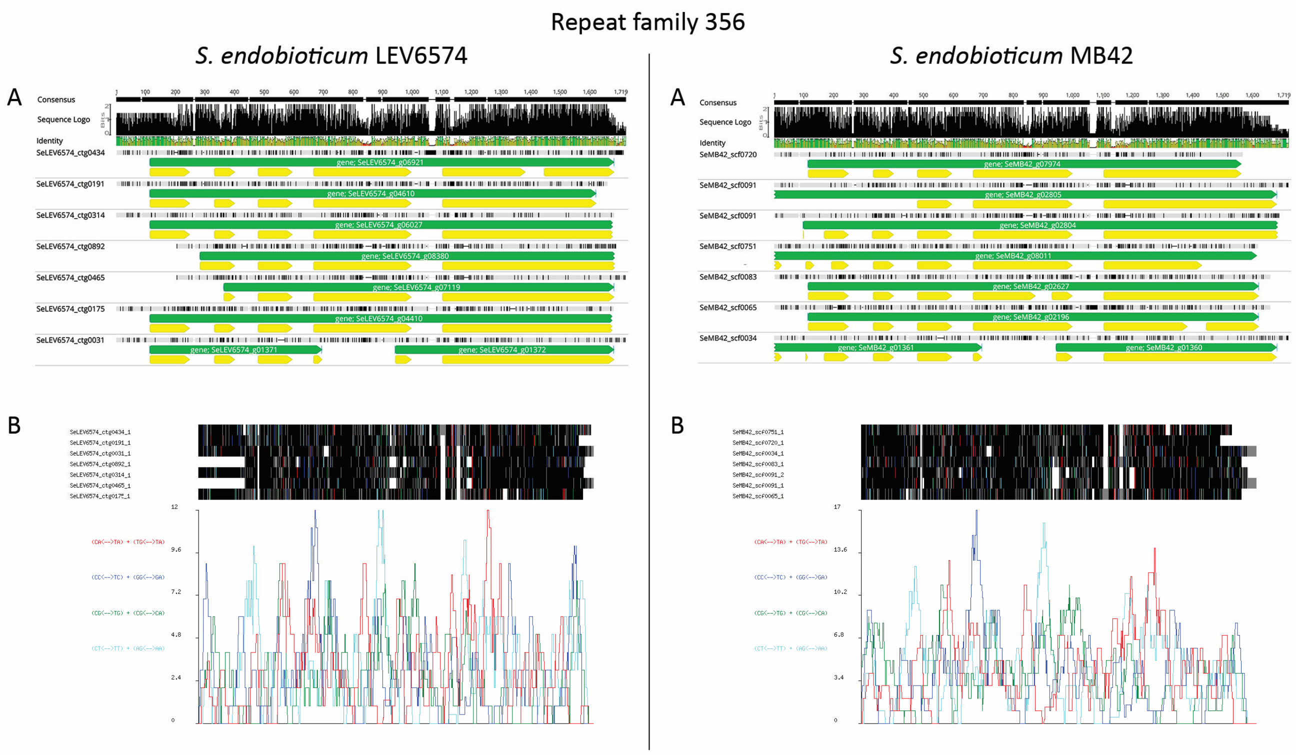

Figure S14. A. MAFFT alignment of repeat sequences identified as “family 356” containing (near) complete gene models of *S. endobioticum* isolates LEV6574 (left) and MB42 (right), representing 7 sequences with alignment lengths of 1719 bp for both isolates. Gene annotations are green whereas coding sequences are annotated in yellow. In the alignment, differences to the overall consensus sequence are highlighted in black. **B.** RIPcal analysis of the alignments presented under **A** using the degenerate consensus sequence as reference. Substitution frequencies between CpA – TpA + TpG – TpA (red), CpC – TpC + GpG – GpA (dark blue), CpG – TpG + CpG – CpA (green), and CpT – TpT + ApG – ApA (light blue) are similar and no dominance of CpA – TpA mutations (red) which are typical for RIP [40] was observed.

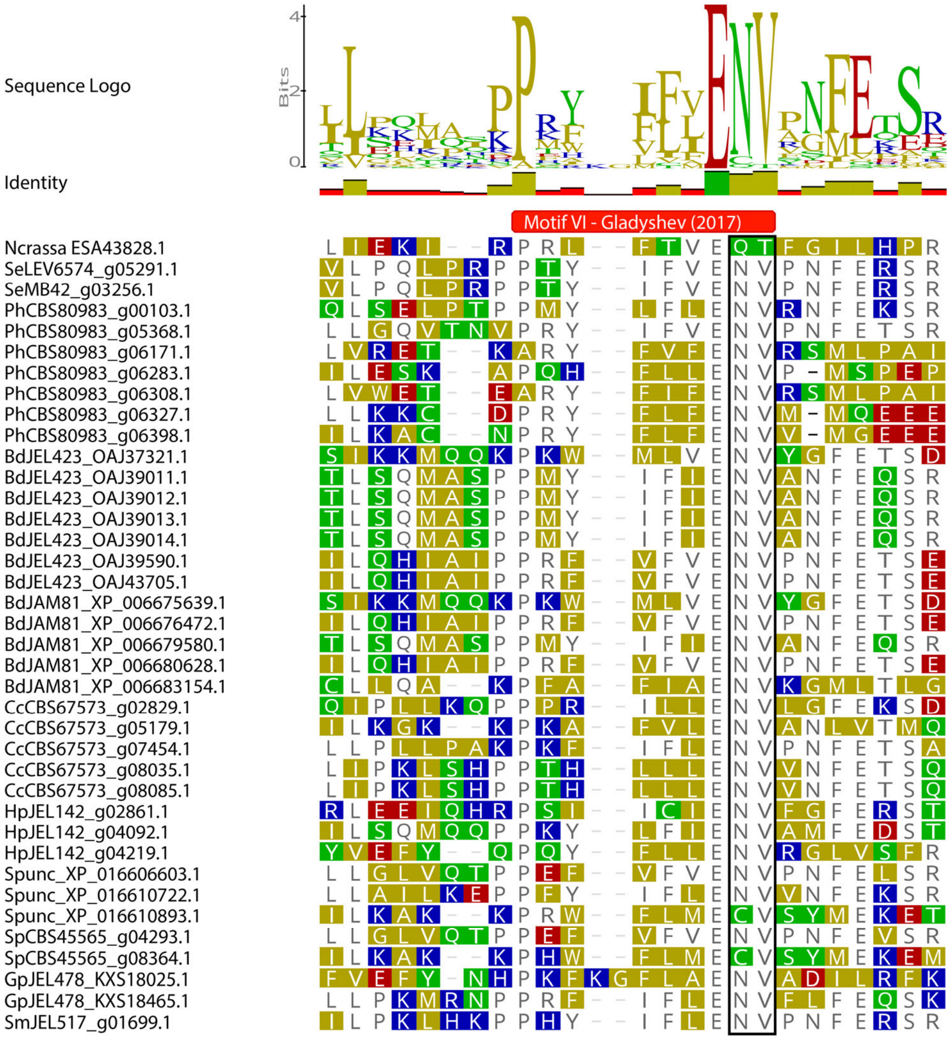

Figure S15. Detail of motif VI as defined by [50] in chytrid proteins with predicted C-5 cytosine methyltransferase activity. Proteins with such activity were detected in all chytrid isolates analysed: *S. endobioticum* (SeLEV6574 and SeMB42), *C. confervae* (CcCBS67573), *P. hirtus* (PhCBS80983), *S. palustris* (SpCBS45565), *S. microbalum* (SmJEL517), *B. dendrobatidis* (BdJAM81 and BdJEL423), *G. prolifera* (GpJEL478), *S. punctatus* (Spunc), and *H. polyrhiza* (HpJEL142). The conserved NV (asparagine-valine) diad (boxed) is present in all chytrid proteins, except for two proteins from *S. punctatus* and *S. palustris* which have a CV diad (cysteine-valine). In none of the chytrid proteins the consecutive QT or ET amino acid sequences specific to the RIP defective protein (RID) and methylation induced premeiotically protein (MIP) were observed. The QT di-amino acid sequence is present in the *N. crassa* RID protein which is included as the first sequence in the alignment as positive control.

9. Meiotic toolbox

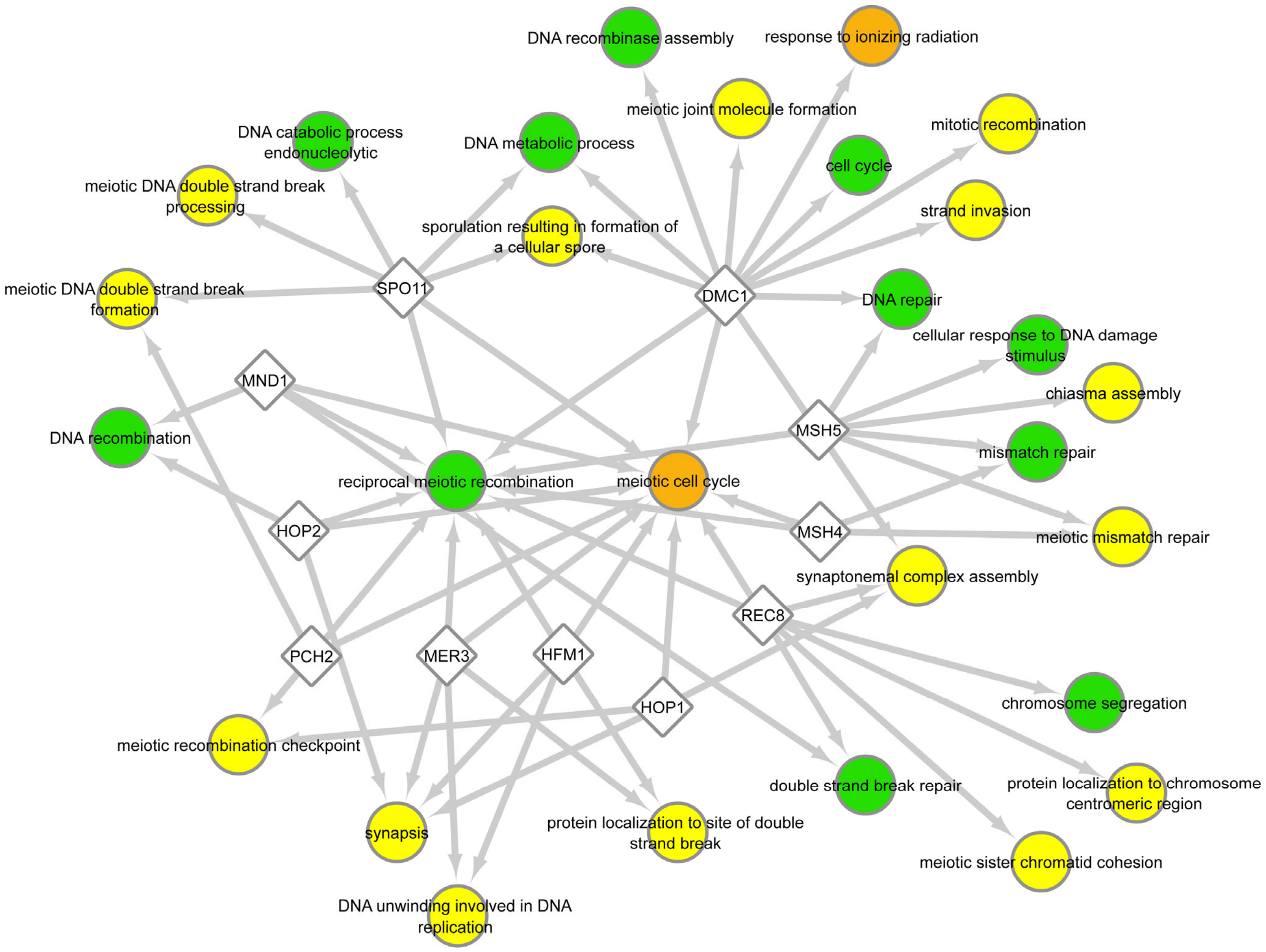

Figure S16. GO-terms associated with eleven meiosis specific proteins and their presence in the proteomes of chytrid species analyzed. Meiosis specific proteins are represented as white diamonds and associated GO-terms (circles) are indicated with grey links. GO-terms detected in proteomes of both obligate biotrophic chytrids and culturable chytrids are colored green, whereas GO-terms detected in only culturable chytrids are colored orange. Unassigned terms are colored yellow.

10. Candidate effector genes

Identified in *S. endobioticum* isolate LEV6574 using MEME-ChIP, a particular *S. endobioticum* specific motif was found to be present in 148 and 75 proteins of isolates LEV6574 and MB42 respectively. Some proteins carry a single motif, while others have two. Proteins with the motif are members to 67 COGs (55 when excluding singletons). Significant differences were found between the pathotype 1(D1) MB42 and pathotype 6(O1) LEV6574 isolate. Expansions of candidate effector proteins carrying the motif were mainly found in LEV6574.

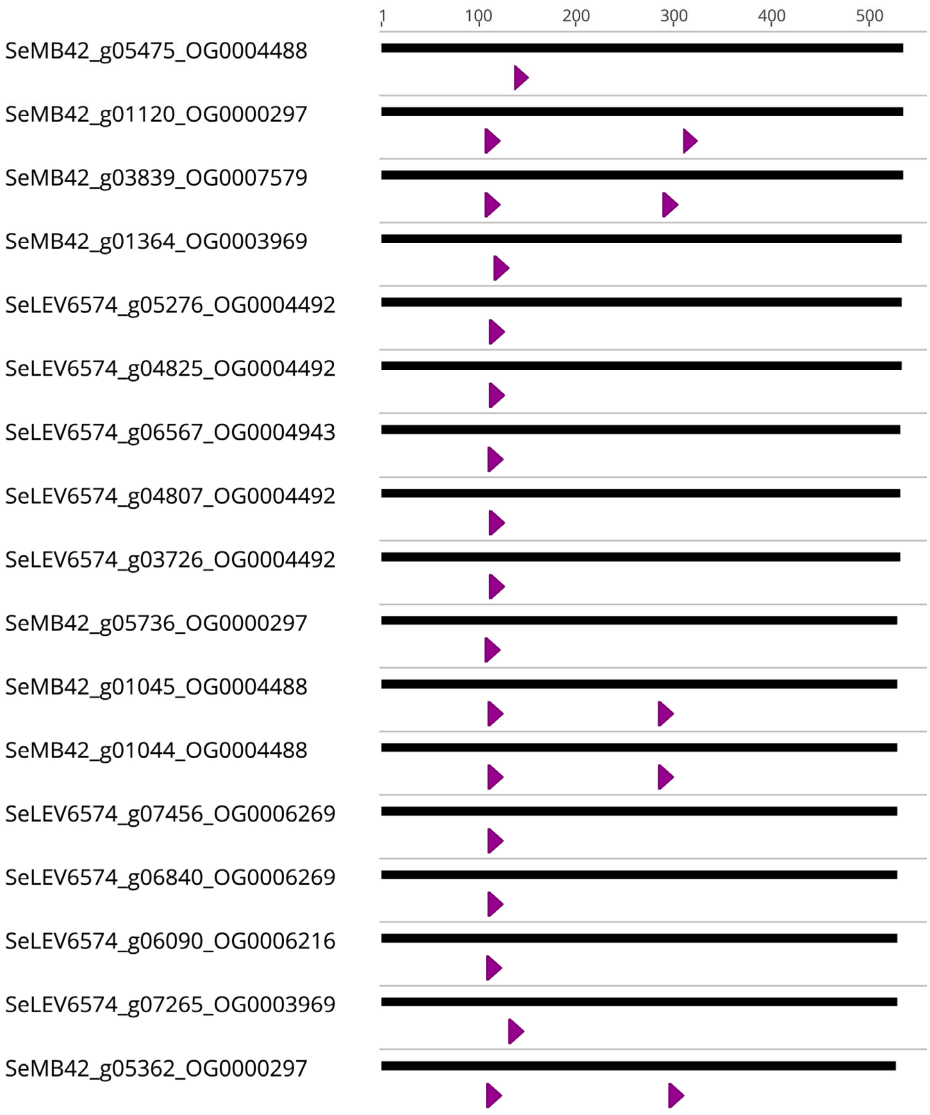

Figure S17. Annotation of the RAYH-motif (purple) on several LEV6574 and MB42 protein sequences (not aligned). Sequence names include COG membership. The scale bar indicates the protein length in number of amino acids. The first occurrence of the motif is typically found between amino acid positions 90 and 120, whereas the second motif (if present) is typically found between amino acid positions 280 and 310.

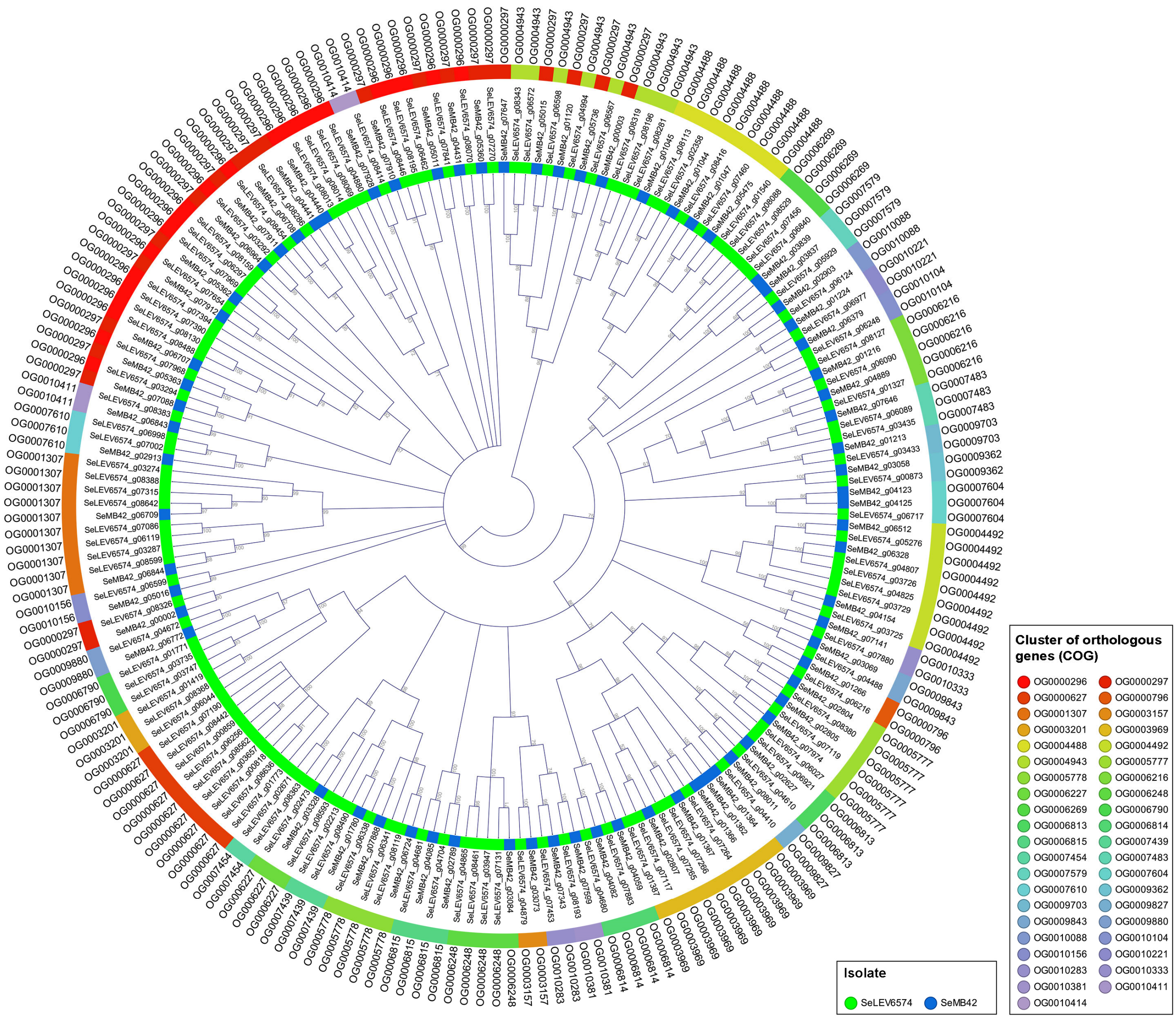

Figure S18. Maximum Likelihood (WAG substitution model, 500 bootstraps) cladogram of 193 *S. endobioticum* proteins carrying the RAYH-motif (122 LEV6574, and 71 MB42 sequences respectively). Bootstrap values ≥50% are shown on tree nodes. The inner colored ring represents the isolate identity where LEV6574 is shown in green and MB42 is shown in blue. The outer colored ring represents the COG membership of the individual protein sequences. The gene names are displayed as labels in the inner node and COG identifiers are shown as labels on the outer node.

To determine if the increased number of candidate effector genes in *S. endobioticum* isolate LEV6574 could be the result of genomic regions that were assembled separately for LEV6574, but were collapsed for MB42, the mean normalized read coverage for candidate effector genes and chytrid core SCOs were determined per isolate.

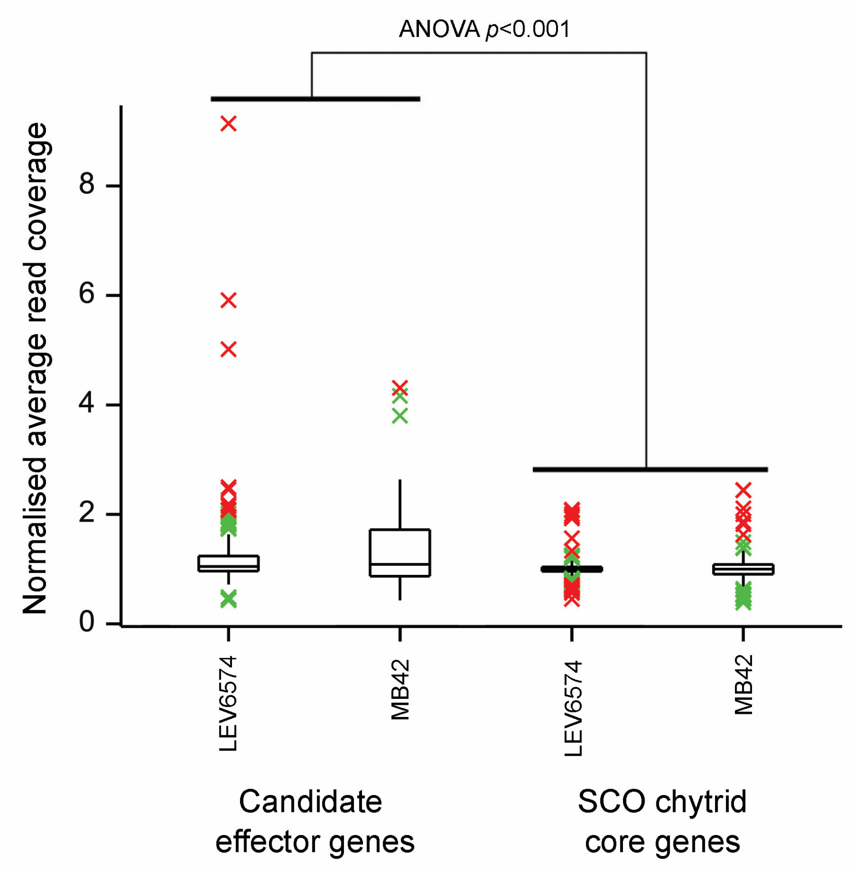

Figure S19. Normalized average read coverage for candidate effectors (LEV6574: 148, MB42: 75 proteins) and chytrid core SCOs (LEV6574 and MB42: 694 proteins). The MB42 candidate effector genes have a much wider range in the second and third quartiles compared to those of LEV6574.

To investigate the characteristics of the candidate effector genes carrying the RAYH-motif, these genes were compared to *S. endobioticum* single copy orthologs (SCO) from the chytrid core COGs for a number of traits. Gene and protein lengths, and the number of introns were compared to demonstrate that the group of candidate effector genes is very conserved. As we observed that the candidate effector genes were often found at terminal ends of scaffolds or contigs, we compared these to the SCOs to see if they are more strongly associated with terminal sequences. The termination of the sequence assembly could be caused by the presence of specific (complex) repeat structures.

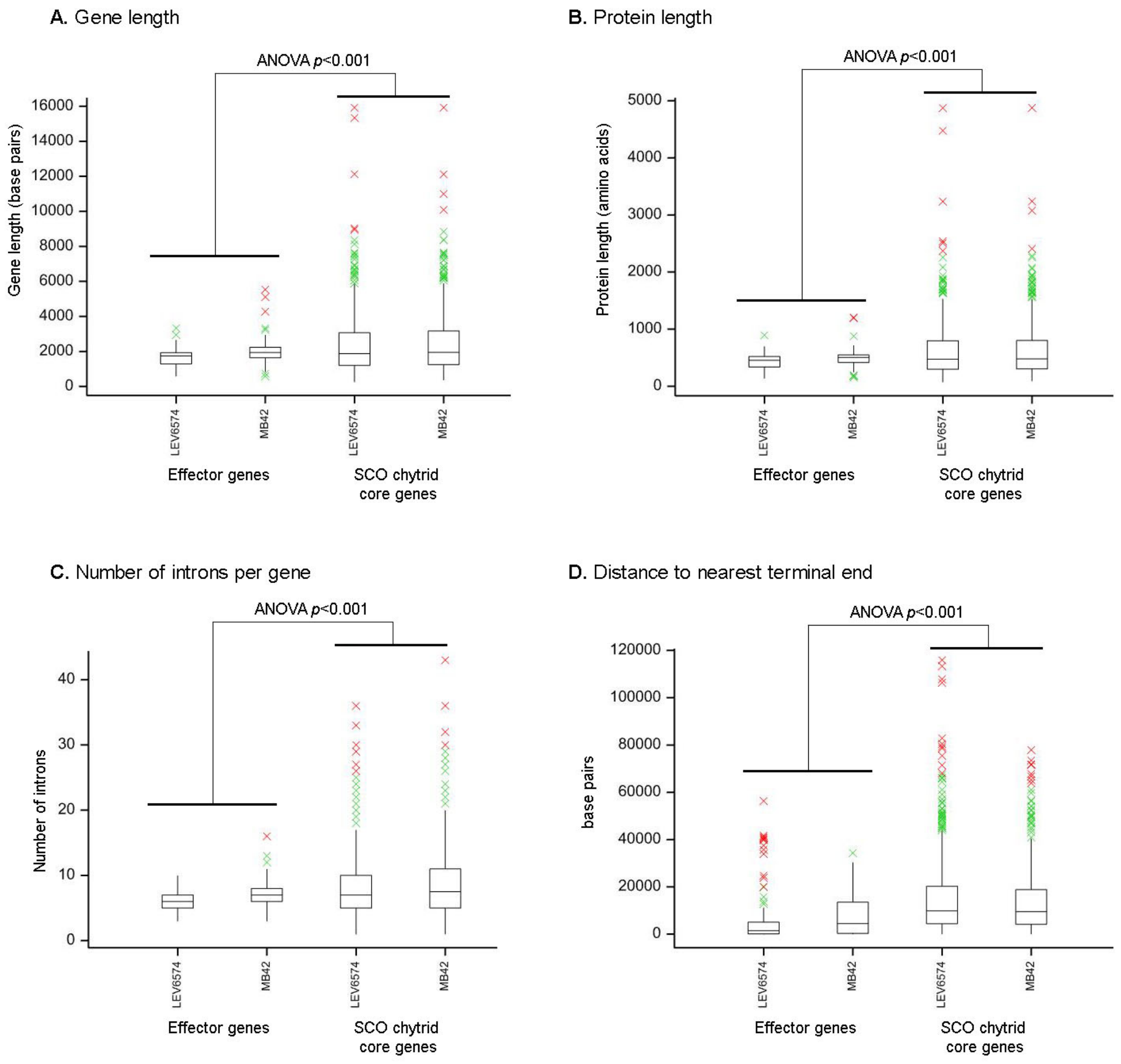

Figure S20. Comparison of gene and protein statistics for candidate effectors (LEV6574: 148, MB42: 75 proteins) and chytrid core SCOs (LEV6574 and MB42: 694 proteins) with **A.** Gene lengths, **B.** protein lengths, **C.** the number of introns, and **D.** the distance to terminal ends of scaffolds and contigs.

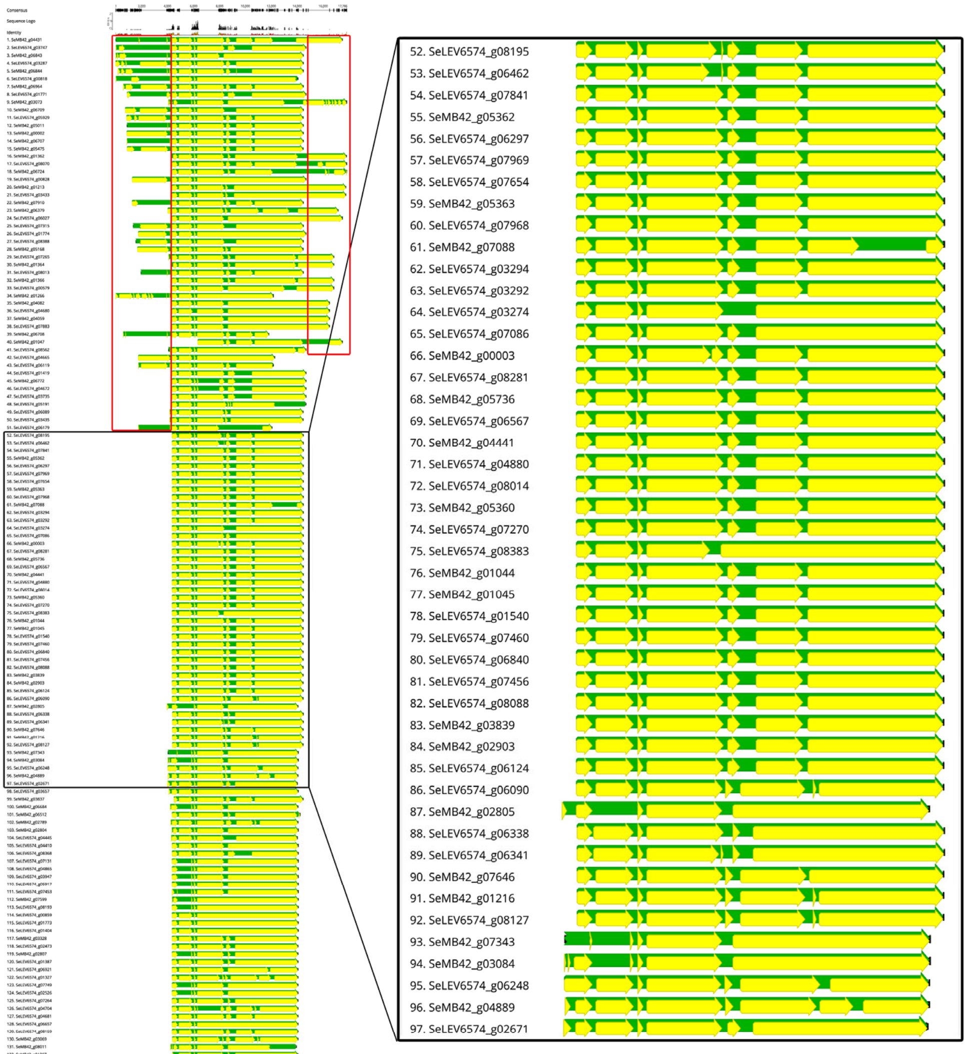

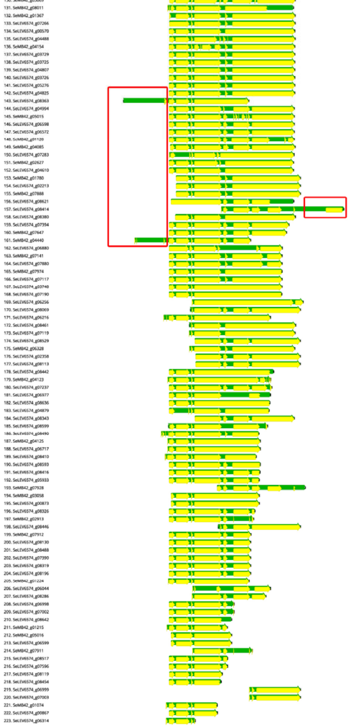

Figure S21. MAFFT nucleotide alignment of the 223 candidate effector genes from *S. endobioticum* isolates MB42 and LEV6574 containing the RAYH-motif. Gene annotations are green whereas coding sequences are annotated in yellow. Boxed in red are 5’ and 3’ end gene sequences that could be predicted incorrectly, resulting (in case of 5’ end sequences) in a failure to predict the gene models as being part of the secretome. Shown in the black box, is a detail of gene alignment clearly showing the high level conservation of intron-exon structure.

11. Genome size comparison across major fungal phyla

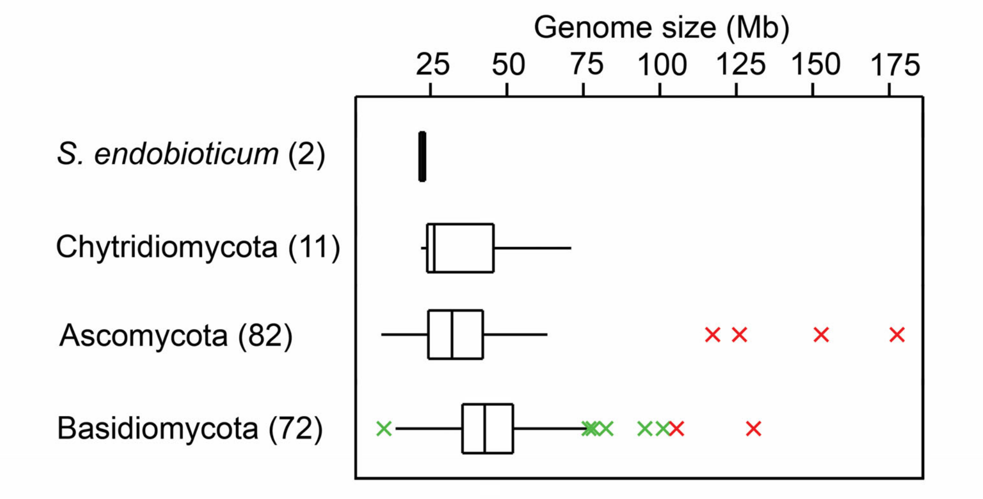

Figure S22. Fungal genome sizes in Mb for two *S. endobioticum*, 11 Chytridiomycota (excluding both *S. endobioticum isolates*), 82 Ascomycota, and 72 Basidiomycota isolates. Genome sizes for species not included in the comparative functional genomics in this study were taken from Mohanta and Bae [23].

Literature

1 van de Vossenberg, B. T. L. H. *et al.* The linear mitochondrial genome of the quarantine chytrid Synchytrium endobioticum; insights into the evolution and recent history of an obligate biotrophic plant pathogen. *BMC Evolutionary Biology* **18**, 136, doi:10.1186/s12862-018-1246-6 (2018).

2 Bonants, P. J. M., van Gent-Pelzer, M. P. E., van Leeuwen, G. C. M. & van der Lee, T. A. J. A real-time TaqMan PCR assay to discriminate between pathotype 1 (D1) and non-pathotype 1 (D1) isolates of Synchytrium endobioticum. *European Journal of Plant Pathology* **143**, 495-506, doi:10.1007/s10658-015-0702-z (2015).

3 Bolger, A. M., Lohse, M. & Usadel, B. Trimmomatic: a flexible trimmer for Illumina sequence data. *Bioinformatics* **30**, 2114-2120, doi:10.1093/bioinformatics/btu170 (2014).

4 The Potato Genome Sequencing, C. *et al.* Genome sequence and analysis of the tuber crop potato. *Nature* **475**, 189 (2011).

5 Bankevich, A. *et al.* SPAdes: A New Genome Assembly Algorithm and Its Applications to Single-Cell Sequencing. *Journal of Computational Biology* **19**, 455-477, doi:10.1089/cmb.2012.0021 (2012).

6 Li, H. & Durbin, R. Fast and accurate short read alignment with Burrows-Wheeler transform. *Bioinformatics* **25**, 1754-1760, doi:10.1093/bioinformatics/btp324 (2009).

7 Myers, E. W. *et al.* A whole-genome assembly of Drosophila. *Science* **287**, 2196-2204 (2000).

8 Walker, B. J. *et al.* Pilon: an integrated tool for comprehensive microbial variant detection and genome assembly improvement. *PLoS One* **9**, e112963, doi:10.1371/journal.pone.0112963 (2014).

9 Kumar, S., Jones, M., Koutsovoulos, G., Clarke, M. & Blaxter, M. Blobology: exploring raw genome data for contaminants, symbionts and parasites using taxon-annotated GC-coverage plots. *Frontiers in genetics* **4**, 237, doi:10.3389/fgene.2013.00237 (2013).

10 Trapnell, C., Pachter, L. & Salzberg, S. L. TopHat: discovering splice junctions with RNA-Seq. *Bioinformatics* **25**, 1105-1111 (2009).

11 Hoff, K. J., Lange, S., Lomsadze, A., Borodovsky, M. & Stanke, M. BRAKER1: Unsupervised RNA-Seq-Based Genome Annotation with GeneMark-ET and AUGUSTUS. *Bioinformatics* **32**, 767-769, doi:10.1093/bioinformatics/btv661 (2016).

12 Holt, C. & Yandell, M. MAKER2: an annotation pipeline and genome-database management tool for second-generation genome projects. *BMC Bioinformatics* **12**, 491-491, doi:10.1186/1471-2105-12-491 (2011).

13 Lomsadze, A., Burns, P. D. & Borodovsky, M. Integration of mapped RNA-Seq reads into automatic training of eukaryotic gene finding algorithm. *Nucleic Acids Res* **42**, e119, doi:10.1093/nar/gku557 (2014).

14 Ter-Hovhannisyan, V., Lomsadze, A., Chernoff, Y. O. & Borodovsky, M. Gene prediction in novel fungal genomes using an ab initio algorithm with unsupervised training. *Genome research* **18**, 1979-1990, doi:10.1101/gr.081612.108 (2008).

15 Chang, Y. *et al.* Phylogenomic Analyses Indicate that Early Fungi Evolved Digesting Cell Walls of Algal Ancestors of Land Plants. *Genome biology and evolution* **7**, 1590-1601, doi:10.1093/gbe/evv090 (2015).

16 Mondo, S. J. *et al.* Widespread adenine N6-methylation of active genes in fungi. *Nature genetics* **49**, 964-968, doi:10.1038/ng.3859 (2017).

17 Russ, C. *et al.* Genome Sequence of Spizellomyces punctatus. *Genome Announc* **4**, doi:10.1128/genomeA.00849-16 (2016).
18 Haitjema, C. H. *et al.* A parts list for fungal cellulosomes revealed by comparative genomics. *Nature microbiology* **2**, 17087, doi:10.1038/nmicrobiol.2017.87 (2017).
19 Youssef, N. H. *et al.* The genome of the anaerobic fungus Orpinomyces sp. strain C1A reveals the unique evolutionary history of a remarkable plant biomass degrader. *Appl Environ Microbiol* **79**, 4620-4634, doi:10.1128/aem.00821-13 (2013).
20 Simao, F. A., Waterhouse, R. M., Ioannidis, P., Kriventseva, E. V. & Zdobnov, E. M. BUSCO: assessing genome assembly and annotation completeness with single-copy orthologs. *Bioinformatics* **31**, 3210-3212, doi:10.1093/bioinformatics/btv351 (2015).
21 Jones, P. *et al.* InterProScan 5: genome-scale protein function classification. *Bioinformatics* **30**, 1236-1240, doi:10.1093/bioinformatics/btu031 (2014).
22 Gurevich, A., Saveliev, V., Vyahhi, N. & Tesler, G. QUAST: quality assessment tool for genome assemblies. *Bioinformatics* **29**, 1072-1075, doi:10.1093/bioinformatics/btt086 (2013).
23 Mohanta, T. K. & Bae, H. The diversity of fungal genome. *Biological procedures online* **17**, 8, doi:10.1186/s12575-015-0020-z (2015).
24 Smith, T. F. & Waterman, M. S. Identification of common molecular subsequences. *Journal of molecular biology* **147**, 195-197 (1981).
25 Emms, D. M. & Kelly, S. OrthoFinder: solving fundamental biases in whole genome comparisons dramatically improves orthogroup inference accuracy. *Genome biology* **16**, 1-14, doi:10.1186/s13059-015-0721-2 (2015).
26 Spatafora, J. W. *et al.* A phylum-level phylogenetic classification of zygomycete fungi based on genome-scale data. *Mycologia* **108**, 1028-1046, doi:10.3852/16-042 (2016).
27 James, Timothy Y. *et al.* Shared Signatures of Parasitism and Phylogenomics Unite Cryptomycota and Microsporidia. *Current Biology* **23**, 1548-1553, doi:https://doi.org/10.1016/j.cub.2013.06.057 (2013).
28 Eddy, S. R. Accelerated Profile HMM Searches. *PLoS computational biology* **7**, e1002195 (2011).
29 Capella-Gutiérrez, S., Silla-Martínez, J. M. & Gabaldón, T. trimAl: a tool for automated alignment trimming in large-scale phylogenetic analyses. *Bioinformatics* **25**, 1972-1973, doi:10.1093/bioinformatics/btp348 (2009).
30 Stamatakis, A. RAxML version 8: a tool for phylogenetic analysis and post-analysis of large phylogenies. *Bioinformatics* **30**, 1312-1313 (2014).
31 Mirarab, S. & Warnow, T. ASTRAL-II: coalescent-based species tree estimation with many hundreds of taxa and thousands of genes. *Bioinformatics* **31**, i44-52, doi:10.1093/bioinformatics/btv234 (2015).
32 Zerillo, M. M. *et al.* Carbohydrate-Active Enzymes in Pythium and Their Role in Plant Cell Wall and Storage Polysaccharide Degradation. *PLOS ONE* **8**, e72572 (2013).
33 Yin, Y. *et al.* dbCAN: a web resource for automated carbohydrate-active enzyme annotation. *Nucleic Acids Research* **40**, W445-W451, doi:10.1093/nar/gks479 (2012).
34 Cantarel, B. L. *et al.* The Carbohydrate-Active EnZymes database (CAZy): an expert resource for Glycogenomics. *Nucleic Acids Res* **37**, D233-238, doi:10.1093/nar/gkn663 (2009).
35 Halary, S. *et al.* Conserved meiotic machinery in Glomus spp., a putatively ancient asexual fungal lineage. *Genome biology and evolution* **3**, 950-958, doi:10.1093/gbe/evr089 (2011).
36 Smit, A. F. A. & Hubley, R. *RepeatModeler Open-1.0*, <http://www.repeatmasker.org> (2008).
37 Smit, A. F. A. & Hubley, R. *RepeatMasker Open-4.0*, <www.repeatmasker.org> (2013-2015).

38 Katoh, K. & Standley, D. M. MAFFT multiple sequence alignment software version 7: improvements in performance and usability. *Molecular biology and evolution* **30**, 772-780, doi:10.1093/molbev/mst010 (2013).
39 Kearse, M. *et al.* Geneious Basic: an integrated and extendable desktop software platform for the organization and analysis of sequence data. *Bioinformatics* **28**, 1647-1649, doi:10.1093/bioinformatics/bts199 (2012).
40 Hane, J. K. & Oliver, R. P. RIPCAL: a tool for alignment-based analysis of repeat-induced point mutations in fungal genomic sequences. *BMC Bioinformatics* **9**, 478, doi:10.1186/1471-2105-9-478 (2008).
41 Petersen, T. N., Brunak, S., von Heijne, G. & Nielsen, H. SignalP 4.0: discriminating signal peptides from transmembrane regions. **8**, 785 (2011).
42 Krogh, A., Larsson, B., von Heijne, G. & Sonnhammer, E. L. Predicting transmembrane protein topology with a hidden Markov model: application to complete genomes. *Journal of molecular biology* **305**, 567-580, doi:10.1006/jmbi.2000.4315 (2001).
43 Bailey, T. L. & Elkan, C. Fitting a mixture model by expectation maximization to discover motifs in biopolymers. *Proceedings. International Conference on Intelligent Systems for Molecular Biology* **2**, 28-36 (1994).
44 Bailey, T. L. & Gribskov, M. Combining evidence using p-values: application to sequence homology searches. *Bioinformatics* **14**, 48-54 (1998).
45 Emanuelsson, O., Nielsen, H., Brunak, S. & von Heijne, G. Predicting subcellular localization of proteins based on their N-terminal amino acid sequence. *Journal of molecular biology* **300**, 1005-1016, doi:10.1006/jmbi.2000.3903 (2000).
46 Sperschneider, J. *et al.* EffectorP: predicting fungal effector proteins from secretomes using machine learning. *The New phytologist* **210**, 743-761, doi:10.1111/nph.13794 (2016).
47 Sperschneider, J., Dodds, P. N., Singh, K. B. & Taylor, J. M. ApoplastP: prediction of effectors and plant proteins in the apoplast using machine learning. *bioRxiv* (2017).
48 Sperschneider, J. *et al.* LOCALIZER: subcellular localization prediction of both plant and effector proteins in the plant cell. **7**, 44598 (2017).
49 Testa, A. C., Oliver, R. P. & Hane, J. K. OcculterCut: A Comprehensive Survey of AT-Rich Regions in Fungal Genomes. *Genome biology and evolution* **8**, 2044-2064, doi:10.1093/gbe/evw121 (2016).
50 Gladyshev, E. Repeat-Induced Point Mutation (RIP) and Other Genome Defense Mechanisms in Fungi. *Microbiology spectrum* **5**, 10.1128/microbiolspec.FUNK-0042-2017, doi:10.1128/microbiolspec.FUNK-0042-2017 (2017).
