# Supplementary material for: Comparative genomics of chytrid fungi reveal insights into the obligate biotrophic and pathogenic lifestyle of Synchytrium endobioticum
Source: Sci Rep. 2019 Jun 17;9:8672. doi: 10.1038/s41598-019-45128-9 (PMC6572847; doi:10.1038/s41598-019-45128-9)
Supplement: Supplementary file 3 [file 41598_2019_45128_MOESM3_ESM.pdf]

Supplementary file with: **Comparative genomics of chytrid fungi reveal insights into the obligate biotrophic and pathogenic lifestyle of *Synchytrium endobioticum***

Bart T.L.H. van de Vossenberg & Sven Warris, Hai D.T. Nguyen, Marga P.E. van Gent-Pelzer, David L. Joly, Henri C. van de Geest, Peter J.M. Bonants, Donna S. Smith, C. André Lévesque, Theo A.J. van der Lee

Supplementary file 3: KEGG pathway analysis

Chytridiomycota versus Ascomycota and Basidiomycota

The following color codes are used:

- (green) Present in ≥1 in all groups: Obligate biotrophic chytrid (Obl_Chyt), Culturable chytrid (Cult_Chyt), Culturable control species (Cult_CTRL), and Obligate or facultative biotrophic Control species (Obl_CTRL)
- (orange) Absent in both Obl_Chyt *and* all Obl_CTRL, *but* present both in ≥1 Cult_Chyt *and* ≥1 Cult_CTRL
- (blue) Present in ≥1 Obl_Chyt *and* ≥1 Obl_CTRL, *but* absent in all Cult_Chyt *and* Cult_CTRL
- (purple) Present in ≥1 in all groups *except* Obl_Chyt
- (light blue) Present in ≥1 Obl_Chyt, *but* absent in all other groups (Cult_Chyt, Cult_CTRL, Obl_CTRL)
- (grey) Present in ≥1 in any specimen included, but not following the grouping as described above

Numerical values in colored boxed {a, b, c, d} represent the number of Obl_Chyt strains (a, max = 2), Cult_Chyt strains (b, max = 9), Cult_CTRL strains (c, max = 3) and Obl_CTRL strains (d, max = 3) for which a element was predicted by Interproscan.

Species and strains included

Obl_Chyt:
***Synchytrium endobioticum* LEV6574**
***Synchytrium endobioticum* MB42**

Cult_Chyt:
***Batrachochytrium dendrobatidis* JAM81**
***Batrachochytrium dendrobatidis* JEL423**
***Chytridium confervae* CBS 675.73**
***Gonapodya prolifera* JEL478**
***Homolaphlyctis polyrhiza* JEL142**
***Powellomyces hirtus* CBS 809.83**
***Spizellomyces palustris*(= *Phlyctochytrium palustre*) CBS 455.65**
***Spizellomyces punctatus* DAOM BR117**
***Synchytrium microbalum* JEL517**

Cult_CTRL:
***Saccharomyces cerevisiae* (SCE)**
***Cryptococcus neoformans* (CNE)**
***Neurospora crassa* (NCR)**

Obl_CTRL:
***Ustilago maydis* (UMA)**
***Melamspora larici-populina* (MLR)**
***Puccinia graminis* f.sp. *tritici* (PGR)**

1. Carbohydrate metabolism

MAP	PATHWAY
**10**	Glycolysis / Gluconeogenesis
**20**	Citrate cycle (TCA cycle)
**30**	Pentose phosphate pathway
**40**	Pentose and glucuronate interconversions
**51**	Fructose and mannose metabolism
**52**	Galactose metabolism
**53**	Ascorbate and aldarate metabolism
**500**	Starch and sucrose metabolism
**520**	Amino sugar and nucleotide sugar metabolism
**562**	Inositol phosphate metabolism
**620**	Pyruvate metabolism
**630**	Glyoxylate and dicarboxylate metabolism
**640**	Propanoate metabolism
**650**	Butanoate metabolism
**660**	C5-Branched dibasic acid metabolism

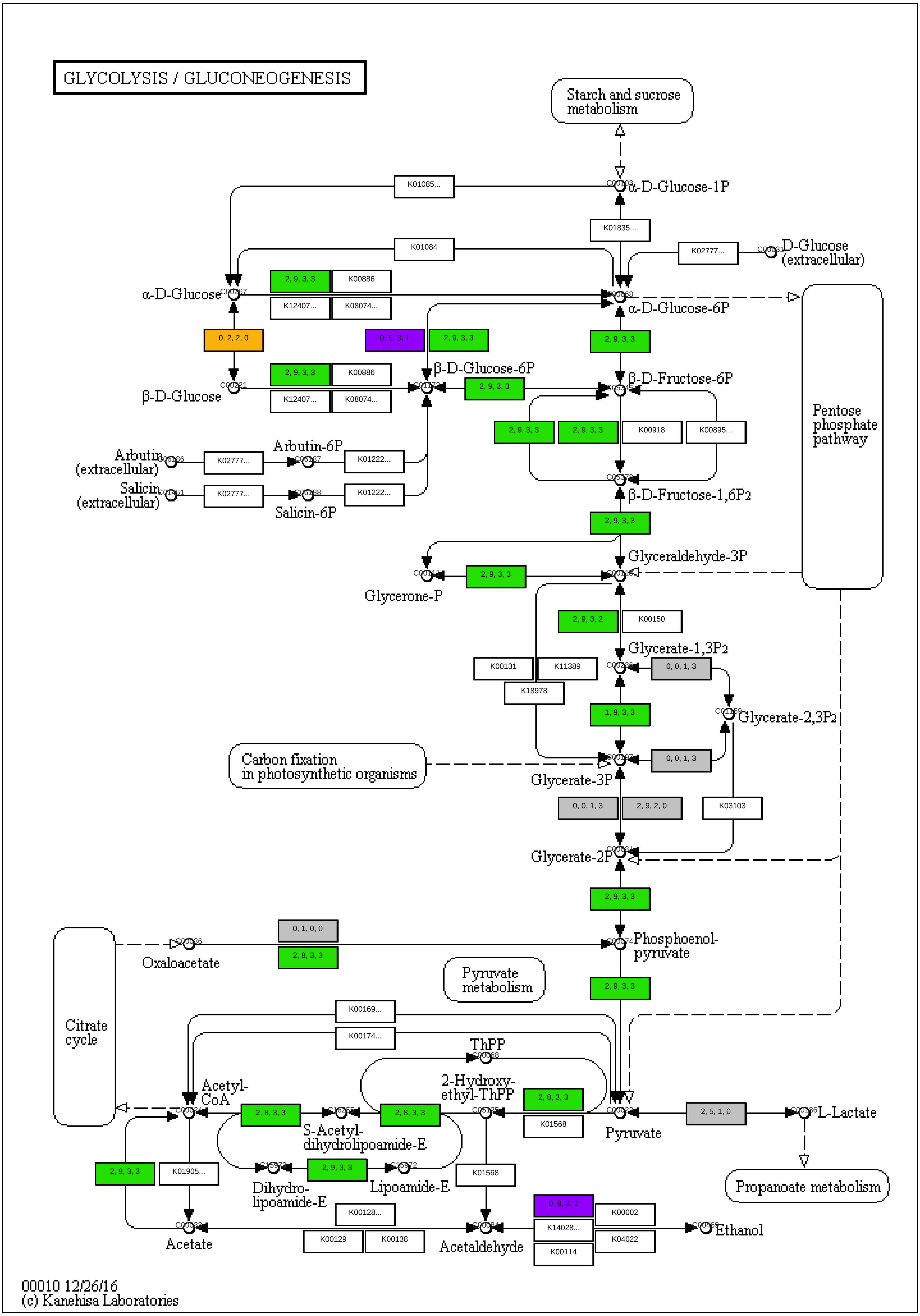
GLYCOLYSIS / GLUCONEOGENESIS
Starch and sucrose metabolism
α-D-Glucose-1P
K01085...
K01835...
K01084
K02777...
D-Glucose (extracellular)
α-D-Glucose
2, 9, 3, 3
K00886
K12407...
K08074...
α-D-Glucose-6P
Pentose phosphate pathway
0, 2, 2, 0
0, 6, 3, 1
β-D-Glucose-6P
β-D-Glucose
β-D-Fructose-6P
K00918
K00895...
Arbutin (extracellular)
Arbutin-6P
K01222...
Salicin (extracellular)
Salicin-6P
β-D-Fructose-1,6P2
Glyceraldehyde-3P
Glycerone-P
2, 9, 3, 2
K00150
Glycerate-1,3P2
K00131
K11389
K18978
0, 0, 1, 3
1, 9, 3, 3
Glycerate-2,3P2
Carbon fixation in photosynthetic organisms
Glycerate-3P
2, 9, 2, 0
K03103
Glycerate-2P
Phosphoenol-pyruvate
0, 1, 0, 0
2, 8, 3, 3
Oxaloacetate
Citrate cycle
Pyruvate metabolism
K00169...
K00174...
ThPP
2-Hydroxy-ethyl-ThPP
Acetyl-CoA
S-Acetyl-dihydrolipoamide-E
K01568
Pyruvate
2, 5, 1, 0
L-Lactate
K01905...
Dihydro-lipoamide-E
Lipoamide-E
Propanoate metabolism
0, 8, 3, 2
K00002
K14028...
K04022
K00114
K00128...
K00129
K00138
Acetate
Acetaldehyde
Ethanol
00010 12/26/16
(c) Kanehisa Laboratories

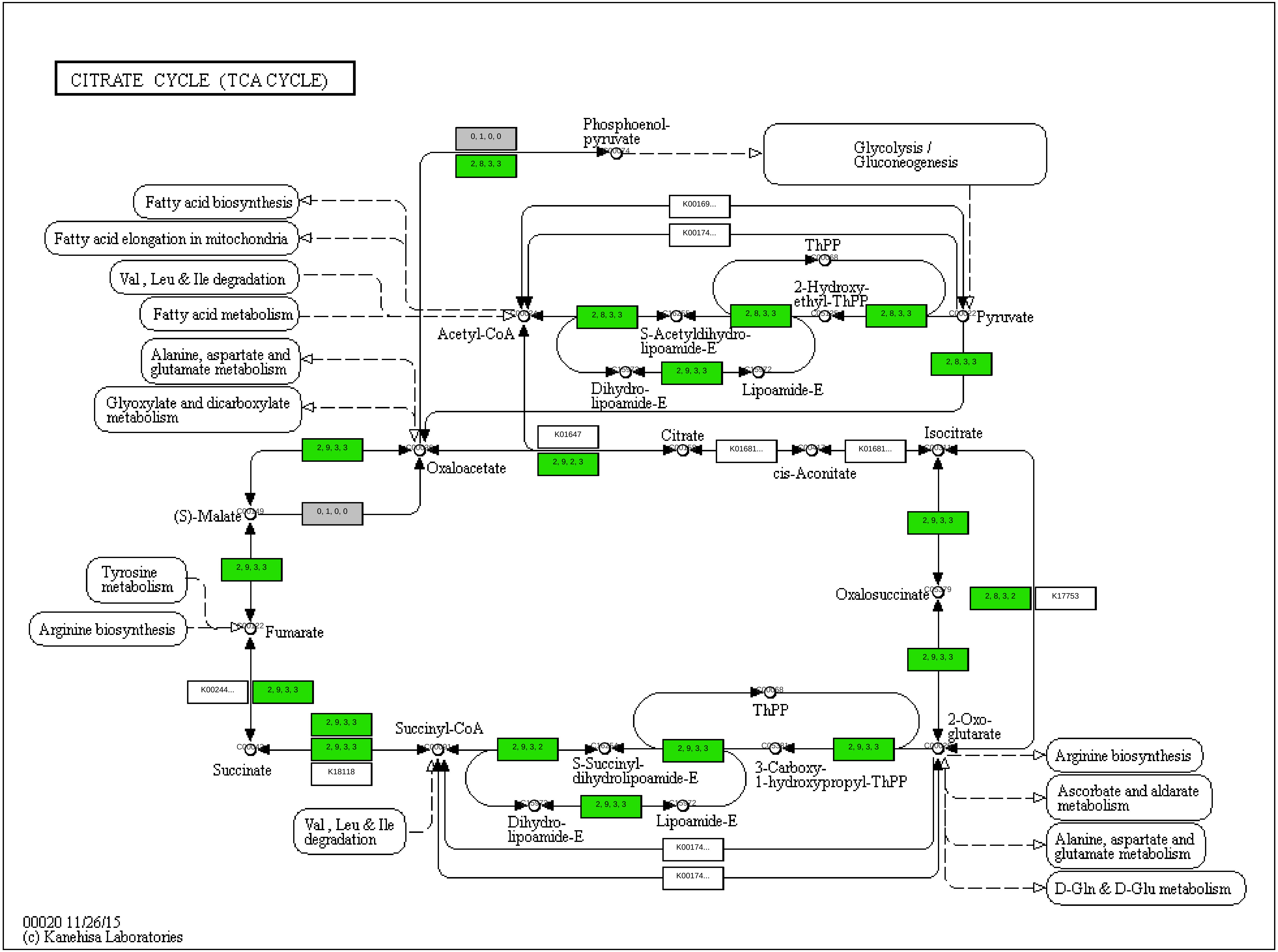
CITRATE CYCLE (TCA CYCLE)
Phosphoenol-pyruvate
Glycolysis / Gluconeogenesis
0, 1, 0, 0
2, 8, 3, 3
Fatty acid biosynthesis
Fatty acid elongation in mitochondria
Val, Leu & Ile degradation
Fatty acid metabolism
Alanine, aspartate and glutamate metabolism
Glyoxylate and dicarboxylate metabolism
K00169...
K00174...
ThPP
2-Hydroxy-ethyl-ThPP
Acetyl-CoA
Pyruvate
S-Acetyldihydro-lipoamide-E
Dihydro-lipoamide-E
Lipoamide-E
2, 9, 3, 3
K01647
2, 9, 2, 3
Citrate
K01681...
cis-Aconitate
Isocitrate
Oxaloacetate
(S)-Malate
Oxalosuccinate
2, 8, 3, 2
K17753
Tyrosine metabolism
Arginine biosynthesis
Fumarate
K00244...
Succinate
K18118
Succinyl-CoA
2, 9, 3, 2
S-Succinyl-dihydrolipoamide-E
3-Carboxy-1-hydroxypropyl-ThPP
2-Oxo-glutarate
Ascorbate and aldarate metabolism
D-Gln & D-Glu metabolism
00020 11/26/15
(c) Kanehisa Laboratories

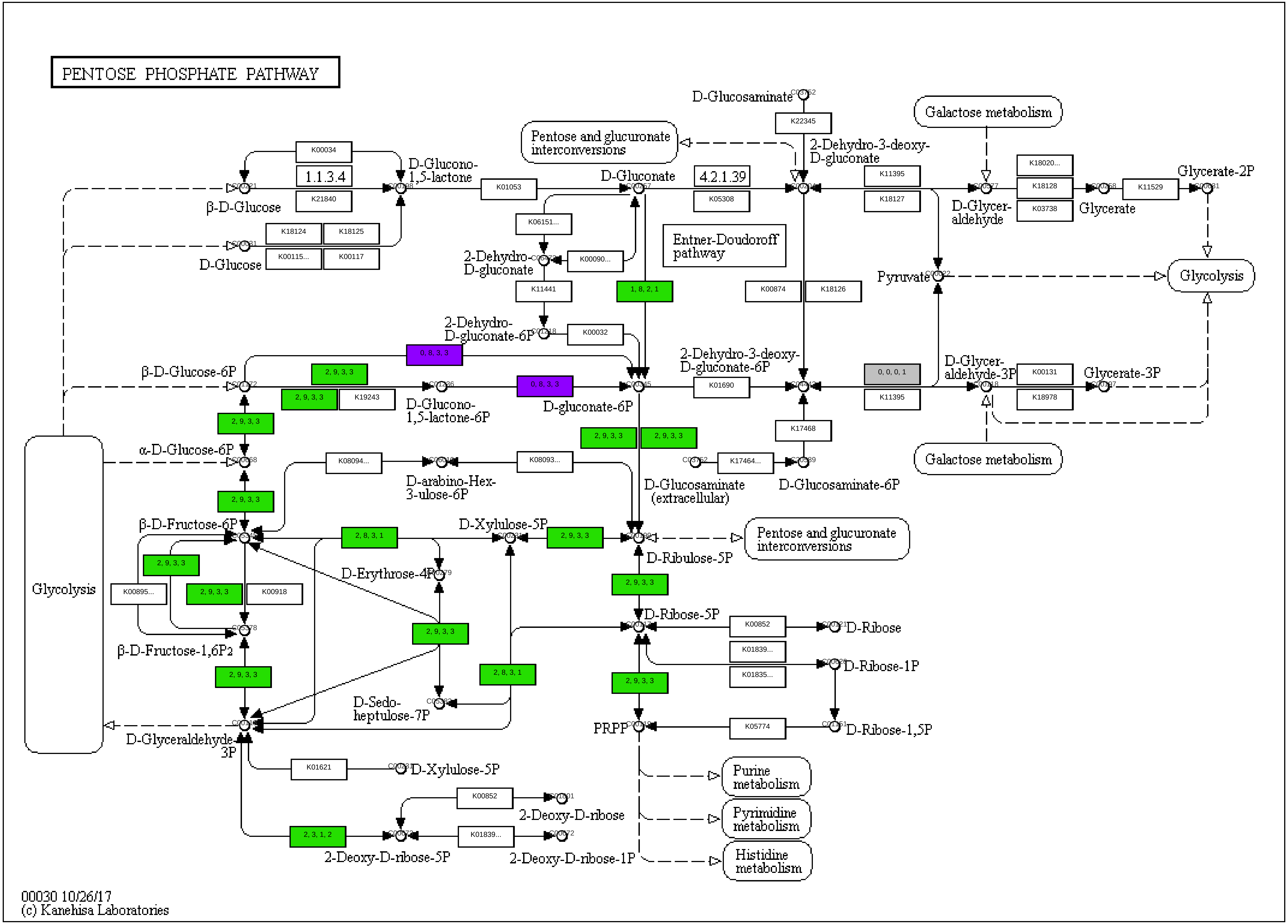
PENTOSE PHOSPHATE PATHWAY
D-Glucosaminate
K22345
Galactose metabolism
Pentose and glucuronate interconversions
2-Dehydro-3-deoxy-D-gluconate
K00034
D-Glucono-1,5-lactone
1.1.3.4
K21840
β-D-Glucose
K01053
D-Gluconate
4.2.1.39
K05308
K11395
K18127
K18020...
K18128
K03738
D-Glyceraldehyde
Glycerate
K11529
Glycerate-2P
K18124
K18125
K06151...
Entner-Doudoroff pathway
K00115...
K00117
D-Glucose
2-Dehydro-D-gluconate
K00090...
K11441
1, 8, 2, 1
K00874
K18126
Pyruvate
Glycolysis
2-Dehydro-D-gluconate-6P
K00032
0, 8, 3, 3
2, 9, 3, 3
2-Dehydro-3-deoxy-D-gluconate-6P
0, 0, 0, 1
D-Glyceraldehyde-3P
K00131
Glycerate-3P
β-D-Glucose-6P
K19243
K01690
K18978
D-Glucono-1,5-lactone-6P
D-gluconate-6P
K17468
α-D-Glucose-6P
K08094...
K08093...
K17464...
D-arabino-Hex-3-ulose-6P
D-Glucosaminate (extracellular)
D-Glucosaminate-6P
β-D-Fructose-6P
2, 8, 3, 1
D-Xylulose-5P
D-Ribulose-5P
D-Erythrose-4P
Glycolysis
K00895...
K00918
D-Ribose-5P
K00852
D-Ribose
K01839...
D-Ribose-1P
K01835...
β-D-Fructose-1,6P2
D-Sedo-heptulose-7P
PRPP
K05774
D-Ribose-1,5P
D-Glyceraldehyde-3P
K01621
D-Xylulose-5P
2-Deoxy-D-ribose
2, 3, 1, 2
2-Deoxy-D-ribose-5P
2-Deoxy-D-ribose-1P
Purine metabolism
Pyrimidine metabolism
Histidine metabolism
00030 10/26/17
(c) Kanehisa Laboratories

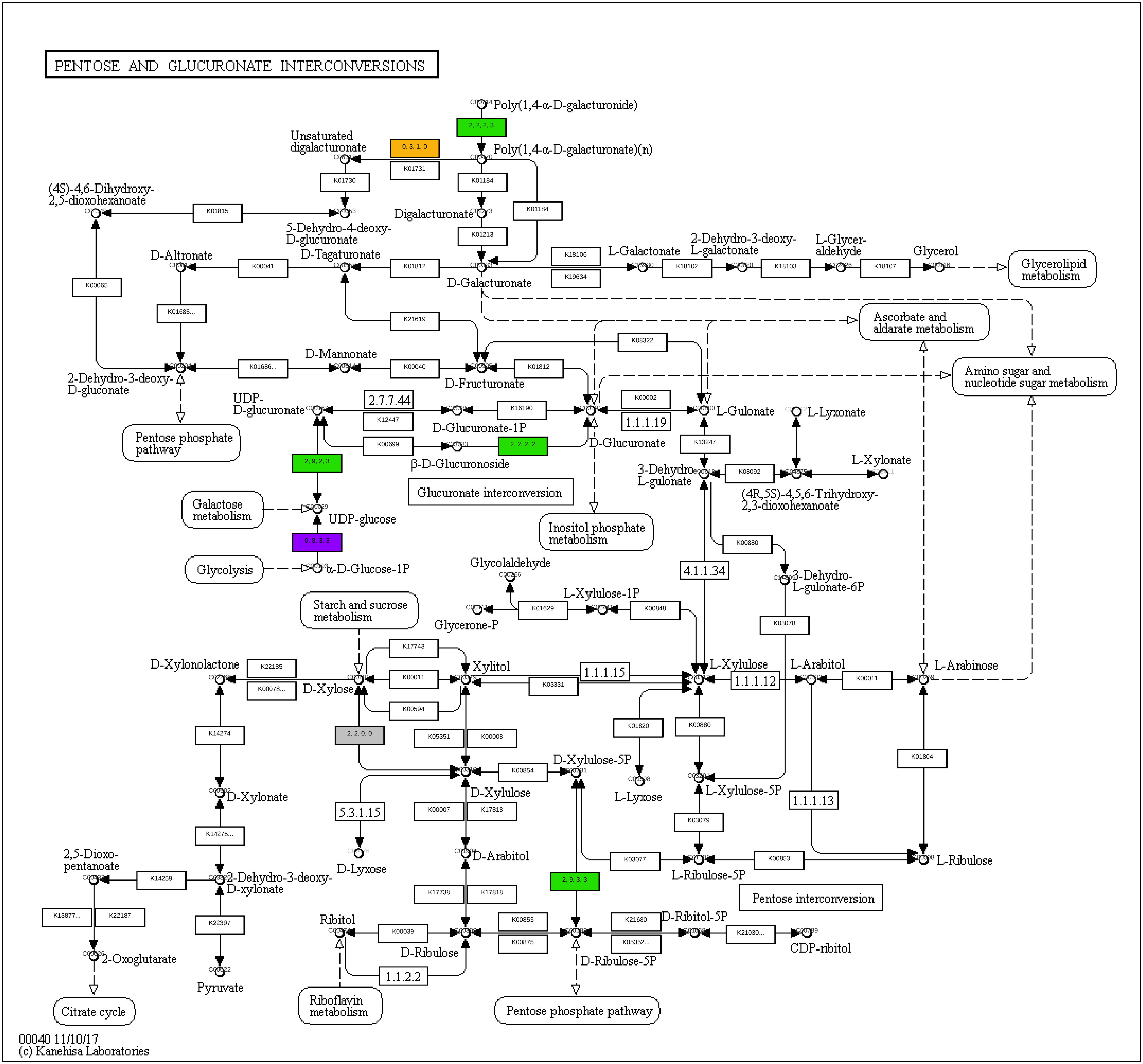
PENTOSE AND GLUCURONATE INTERCONVERSIONS
Poly(1,4-α-D-galacturonide)
2, 2, 2, 3
Poly(1,4-α-D-galacturonate)(n)
Unsaturated digalacturonate
0, 3, 1, 0
K01731
K01730
K01184
(4S)-4,6-Dihydroxy-2,5-dioxohexanoate
K01815
5-Dehydro-4-deoxy-D-glucuronate
Digalacturonate
K01184
K01213
D-Altronate
K00041
D-Tagaturonate
K01812
D-Galacturonate
K18106
K19634
L-Galactonate
K18102
2-Dehydro-3-deoxy-L-galactonate
K18103
L-Glyceraldehyde
K18107
Glycerol
Glycerolipid metabolism
K00065
K01685...
K21619
Ascorbate and aldarate metabolism
K08322
Amino sugar and nucleotide sugar metabolism
2-Dehydro-3-deoxy-D-gluconate
K01686...
D-Mannonate
K00040
K01812
D-Fructuronate
UDP-D-glucuronate
2.7.7.44
K16190
K00002
L-Gulonate
L-Lyxonate
1.1.1.19
K12447
D-Glucuronate-1P
Pentose phosphate pathway
K00699
2, 2, 2, 2
D-Glucuronate
K13247
2, 9, 2, 3
β-D-Glucuronoside
3-Dehydro-L-gulonate
K08092
L-Xylonate
Glucuronate interconversion
(4R,5S)-4,5,6-Trihydroxy-2,3-dioxohexanoate
Galactose metabolism
UDP-glucose
Inositol phosphate metabolism
K00880
0, 8, 3, 3
4.1.1.34
3-Dehydro-L-gulonate-6P
Glycolysis
α-D-Glucose-1P
Glycolaldehyde
L-Xylulose-1P
K01629
K00848
Glycerone-P
K03078
Starch and sucrose metabolism
K17743
Xylitol
1.1.1.15
L-Xylulose
L-Arabitol
L-Arabinose
D-Xylonolactone
K22185
K00011
1.1.1.12
K00011
K00078...
D-Xylose
K03331
K00594
K01820
K00880
K14274
2, 2, 0, 0
K05351
K00008
K01804
D-Xylulose-5P
K00854
L-Lyxose
D-Xylonate
D-Xylulose
L-Xylulose-5P
5.3.1.15
K00007
K17818
1.1.1.13
K14275...
K03079
D-Lyxose
D-Arabitol
K03077
K00853
L-Ribulose
2,5-Dioxopentanoate
K14259
2-Dehydro-3-deoxy-D-xylonate
L-Ribulose-5P
2, 9, 3, 3
K17738
K17818
Pentose interconversion
K13877...
K22187
K22397
Ribitol
K00039
K00853
K21680
D-Ribitol-5P
K00875
K05352...
K21030...
CDP-ribitol
2-Oxoglutarate
D-Ribulose
D-Ribulose-5P
Pyruvate
1.1.2.2
Citrate cycle
Riboflavin metabolism
Pentose phosphate pathway
00040 11/10/17
(c) Kanehisa Laboratories

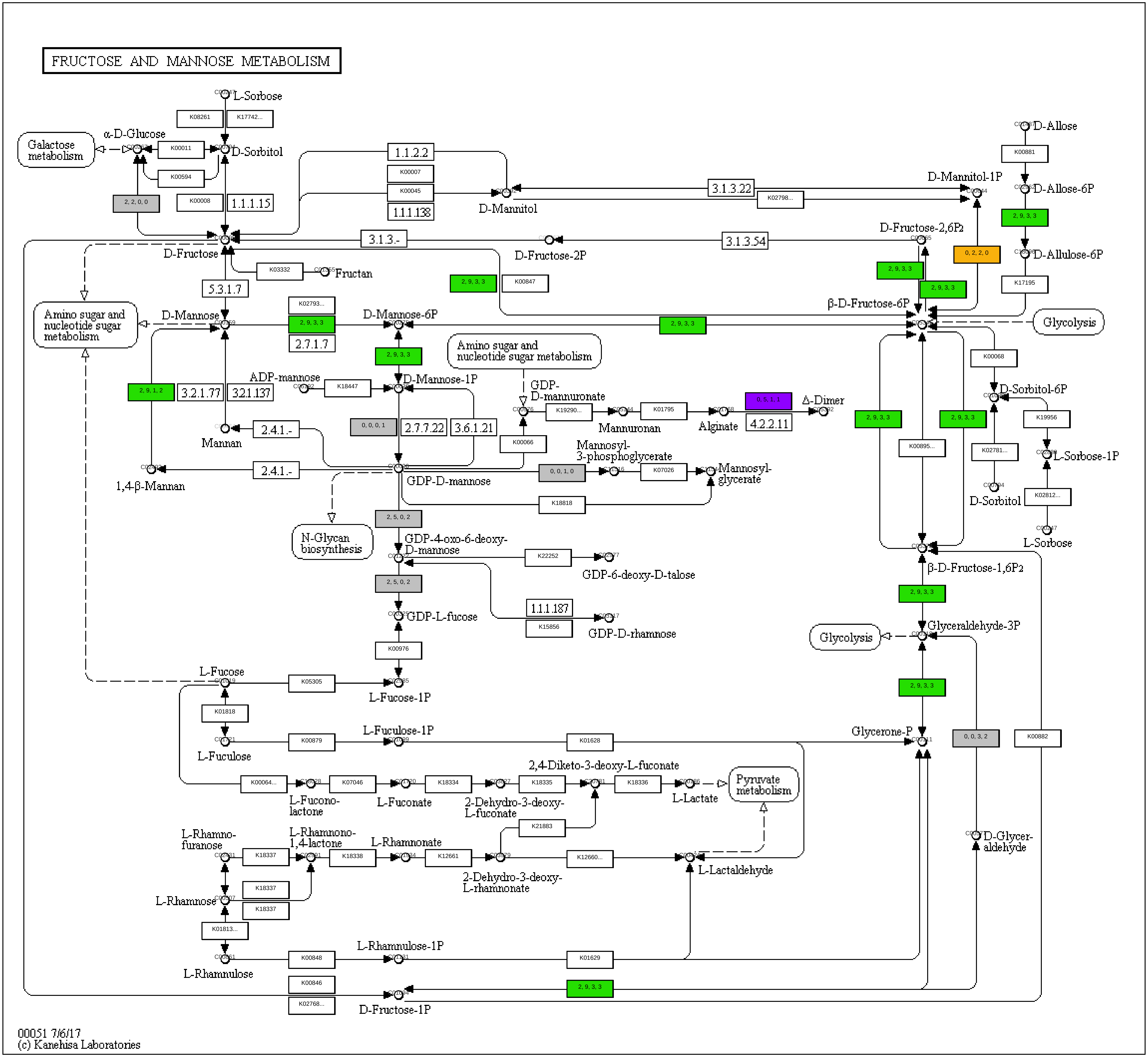
FRUCTOSE AND MANNOSE METABOLISM
L-Sorbose
K08261
K17742...
α-D-Glucose
Galactose metabolism
K00011
D-Sorbitol
K00594
2, 2, 0, 0
K00008
1.1.1.15
1.1.2.2
K00007
K00045
1.1.1.138
D-Mannitol
3.1.3.22
K02798...
D-Mannitol-1P
D-Allose
K00881
D-Allose-6P
2, 9, 3, 3
3.1.3.-
D-Fructose
D-Fructose-2P
3.1.3.54
D-Fructose-2,6P2
D-Allulose-6P
0, 2, 2, 0
K03332
Fructan
5.3.1.7
2, 9, 3, 3
K00847
K17195
Amino sugar and nucleotide sugar metabolism
D-Mannose
K02793...
2, 9, 3, 3
2.7.1.7
D-Mannose-6P
β-D-Fructose-6P
Glycolysis
2, 9, 1, 2
3.2.1.77
3.2.1.137
ADP-mannose
K18447
D-Mannose-1P
Amino sugar and nucleotide sugar metabolism
GDP-D-mannuronate
K19290...
Mannuronan
K01795
Alginate
0, 5, 1, 1
Δ-Dimer
4.2.2.11
K00068
D-Sorbitol-6P
0, 0, 0, 1
2.7.7.22
3.6.1.21
K00066
Mannan
2.4.1.-
Mannosyl-3-phosphoglycerate
K00895...
K02781...
K19956
L-Sorbose-1P
1,4-β-Mannan
2.4.1.-
GDP-D-mannose
0, 0, 1, 0
K07026
Mannosyl-glycerate
K18818
D-Sorbitol
K02812...
L-Sorbose
N-Glycan biosynthesis
2, 5, 0, 2
GDP-4-oxo-6-deoxy-D-mannose
K22252
GDP-6-deoxy-D-talose
β-D-Fructose-1,6P2
2, 5, 0, 2
1.1.1.187
K15856
GDP-D-rhamnose
GDP-L-fucose
Glyceraldehyde-3P
Glycolysis
K00976
L-Fucose
K05305
L-Fucose-1P
K01818
L-Fuculose-1P
K00879
K01628
Glycerone-P
L-Fuculose
0, 0, 3, 2
K00882
2,4-Diketo-3-deoxy-L-fuconate
K00064...
K07046
K18334
K18335
K18336
Pyruvate metabolism
L-Fucono-lactone
L-Fuconate
2-Dehydro-3-deoxy-L-fuconate
L-Lactate
K21883
L-Rhamno-furanose
L-Rhamnono-1,4-lactone
L-Rhamnonate
K18337
K18338
K12661
K12660...
D-Glycer-aldehyde
2-Dehydro-3-deoxy-L-rhamnonate
L-Lactaldehyde
K18337
L-Rhamnose
K18337
K01813...
L-Rhamnulose-1P
K00848
K01629
L-Rhamnulose
K00846
K02768...
D-Fructose-1P
00051 7/6/17
(c) Kanehisa Laboratories

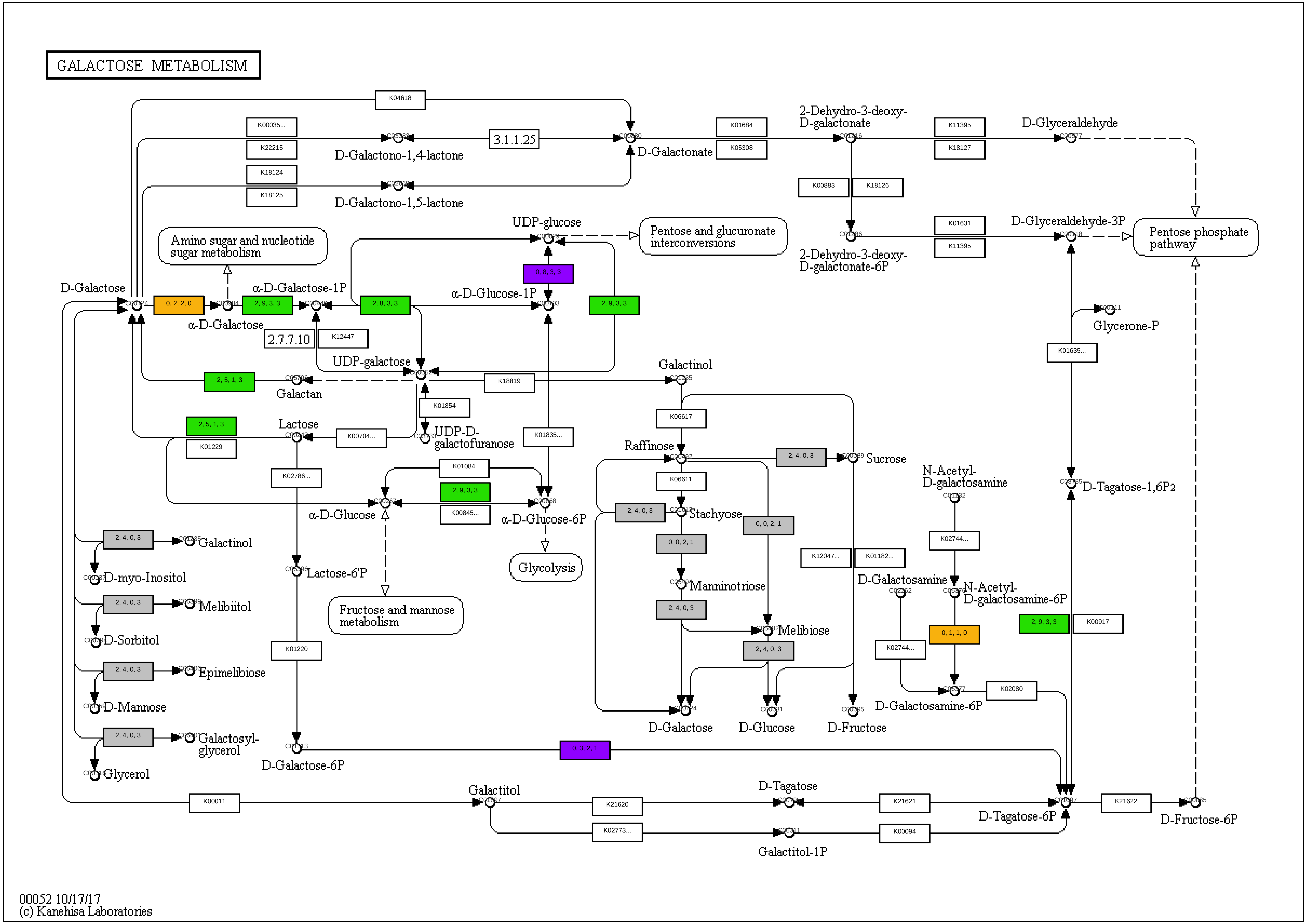
GALACTOSE METABOLISM
K04618
K00035...
K22215
K18124
K18125
3.1.1.25
D-Galactono-1,4-lactone
D-Galactono-1,5-lactone
D-Galactonate
K01684
K05308
2-Dehydro-3-deoxy-D-galactonate
K11395
K18127
D-Glyceraldehyde
K00883
K18126
K01631
K11395
D-Glyceraldehyde-3P
2-Dehydro-3-deoxy-D-galactonate-6P
Pentose phosphate pathway
Pentose and glucuronate interconversions
Amino sugar and nucleotide sugar metabolism
UDP-glucose
0, 8, 3, 3
D-Galactose
0, 2, 2, 0
α-D-Galactose
2, 9, 3, 3
α-D-Galactose-1P
2, 8, 3, 3
α-D-Glucose-1P
2, 9, 3, 3
2.7.7.10
K12447
UDP-galactose
Glycerone-P
K01635...
2, 5, 1, 3
Galactan
K18819
Galactinol
K01854
2, 5, 1, 3
K01229
Lactose
K00704...
UDP-D-galactofuranose
K01835...
K06617
Raffinose
2, 4, 0, 3
Sucrose
K02786...
K01084
2, 9, 3, 3
K00845...
α-D-Glucose
α-D-Glucose-6P
K06611
2, 4, 0, 3
Stachyose
0, 0, 2, 1
0, 0, 2, 1
N-Acetyl-D-galactosamine
D-Tagatose-1,6P2
2, 4, 0, 3
Galactinol
D-myo-Inositol
Glycolysis
Lactose-6'P
K12047...
K01182...
K02744...
D-Galactosamine
N-Acetyl-D-galactosamine-6P
Mannintriose
2, 4, 0, 3
Melibiitol
D-Sorbitol
Fructose and mannose metabolism
2, 4, 0, 3
Melibiose
0, 1, 1, 0
2, 9, 3, 3
K00917
K01220
2, 4, 0, 3
K02744...
2, 4, 0, 3
Epimelibiose
D-Mannose
K02080
D-Galactosamine-6P
D-Galactose
D-Glucose
D-Fructose
2, 4, 0, 3
Galactosyl-glycerol
Glycerol
D-Galactose-6P
0, 3, 2, 1
Galactitol
K00011
K21620
D-Tagatose
K21621
K21622
D-Tagatose-6P
D-Fructose-6P
K02773...
K00094
Galactitol-1P
00052 10/17/17
(c) Kanehisa Laboratories

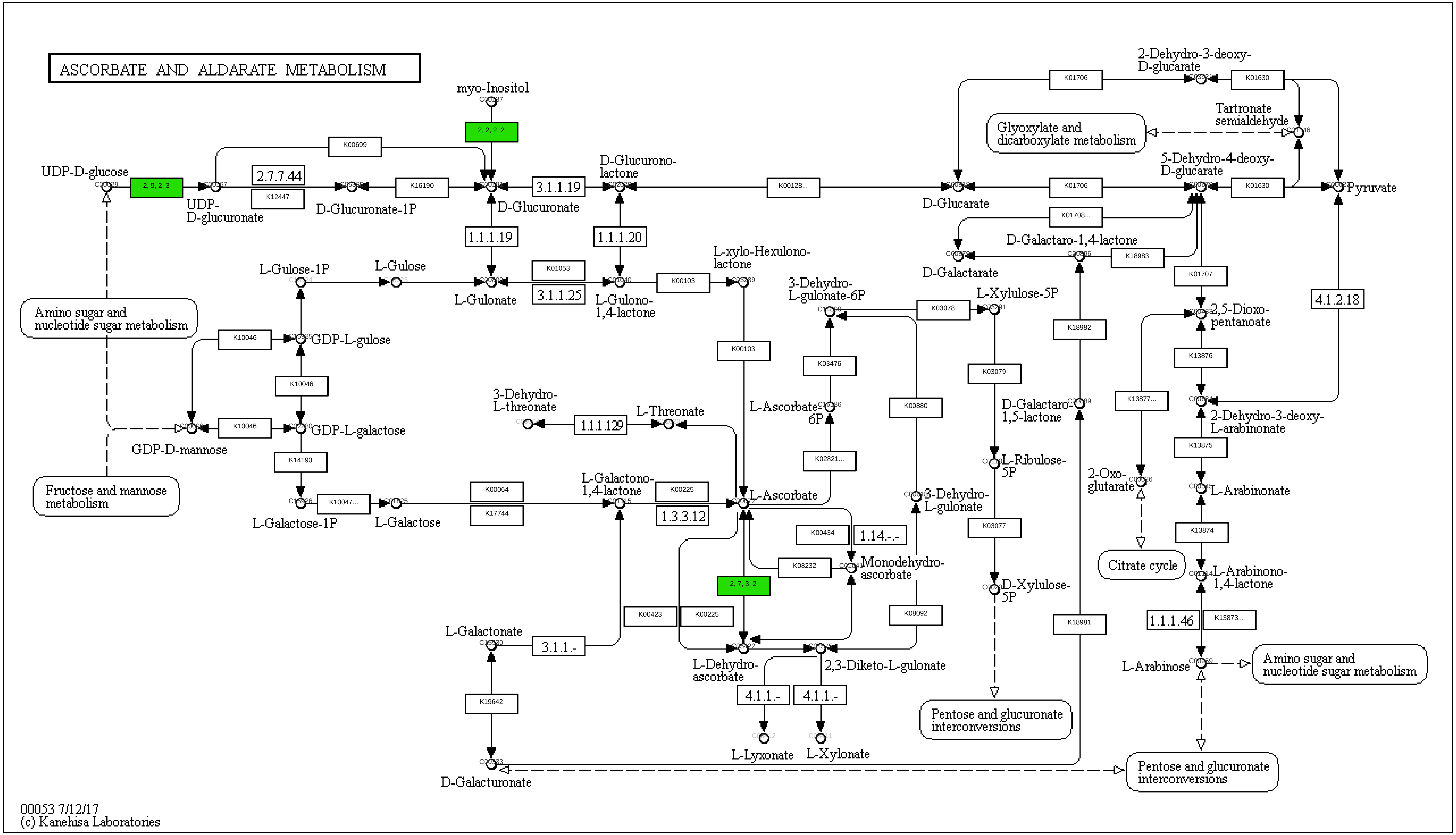
ASCORBATE AND ALDARATE METABOLISM
myo-Inositol
2, 2, 2, 2
K00699
UDP-D-glucose
2, 9, 2, 3
UDP-
D-glucuronate
2.7.7.44
K12447
D-Glucuronate-1P
K16190
D-Glucuronate
3.1.1.19
D-Glucurono-
lactone
K00128...
D-Glucarate
K01706
5-Dehydro-4-deoxy-
D-glucarate
K01630
Pyruvate
2-Dehydro-3-deoxy-
D-glucarate
Tartronate
semialdehyde
Glyoxylate and
dicarboxylate metabolism
K01708...
D-Galactaro-1,4-lactone
K18983
D-Galactarate
K01707
4.1.2.18
1.1.1.19
1.1.1.20
L-Gulose-1P
L-Gulose
K01053
3.1.1.25
L-Gulonate
L-Gulono-
1,4-lactone
K00103
L-xylo-Hexulono-
lactone
3-Dehydro-
L-gulonate-6P
K03078
L-Xylulose-5P
K18982
2,5-Dioxo-
pentanoate
K13876
Amino sugar and
nucleotide sugar metabolism
K10046
GDP-L-gulose
K10046
K00103
K03476
K00880
K03079
K13877...
2-Dehydro-3-deoxy-
L-arabinonate
3-Dehydro-
L-threonate
1.1.1.129
L-Threonate
L-Ascorbate-
6P
D-Galactaro-
1,5-lactone
GDP-D-mannose
K10046
GDP-L-galactose
K14190
K02821...
L-Ribulose-
5P
K13875
2-Oxo-
glutarate
L-Arabinonate
Fructose and mannose
metabolism
K10047...
L-Galactose-1P
L-Galactose
K00064
K17744
L-Galactono-
1,4-lactone
K00225
1.3.3.12
L-Ascorbate
3-Dehydro-
L-gulonate
K03077
K13874
K00434
1.14.-.-
K08232
Monodehydro-
ascorbate
2, 7, 3, 2
Citrate cycle
L-Arabinono-
1,4-lactone
D-Xylulose-
5P
1.1.1.46
K13873...
K00423
K00225
K08092
K18981
L-Galactonate
3.1.1.-
L-Dehydro-
ascorbate
2,3-Diketo-L-gulonate
L-Arabinose
Amino sugar and
nucleotide sugar metabolism
K19642
4.1.1.-
4.1.1.-
Pentose and glucuronate
interconversions
L-Lyxonate
L-Xylonate
D-Galacturonate
Pentose and glucuronate
interconversions
00053 7/12/17
(c) Kanehisa Laboratories

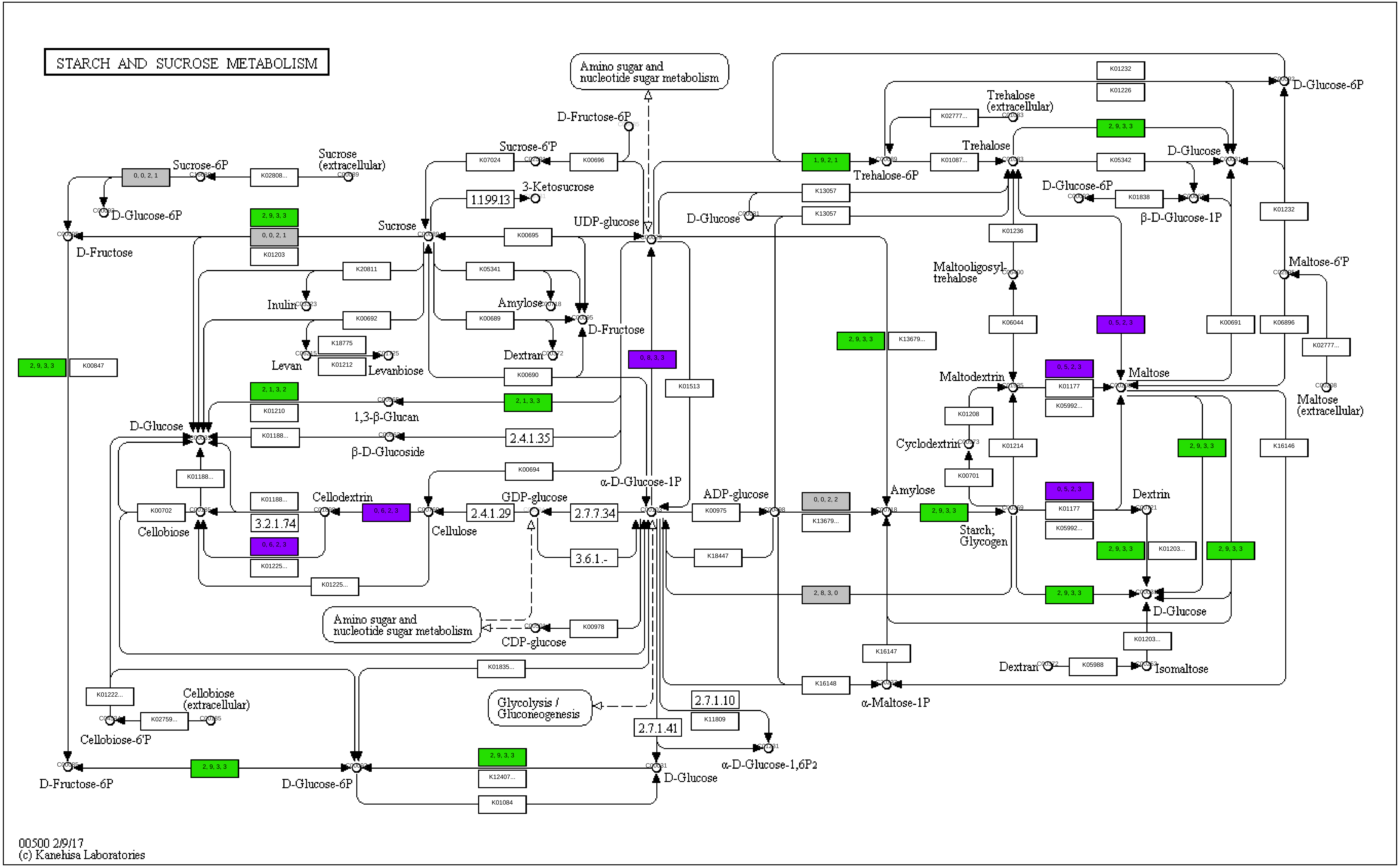
STARCH AND SUCROSE METABOLISM
Amino sugar and nucleotide sugar metabolism
D-Fructose-6P
Sucrose-6'P
Sucrose-6P
Sucrose (extracellular)
3-Ketosucrose
1.1.99.13
D-Glucose-6P
D-Fructose
Sucrose
UDP-glucose
D-Glucose
Trehalose (extracellular)
Trehalose
Trehalose-6P
D-Glucose-6P
β-D-Glucose-1P
Maltose-6'P
Maltooligosyl-trehalose
Inulin
Amylose
D-Fructose
Levan
Levanbiose
Dextran
Maltodextrin
Maltose
Maltose (extracellular)
1,3-β-Glucan
D-Glucose
β-D-Glucoside
2.4.1.35
Cyclodextrin
Cellodextrin
GDP-glucose
α-D-Glucose-1P
ADP-glucose
Amylose
Dextrin
Cellobiose
3.2.1.74
2.4.1.29
Cellulose
2.7.7.34
Starch; Glycogen
3.6.1.-
D-Glucose
Amino sugar and nucleotide sugar metabolism
CDP-glucose
Dextran
Isomaltose
Glycolysis / Gluconeogenesis
2.7.1.10
α-Maltose-1P
Cellobiose (extracellular)
Cellobiose-6'P
2.7.1.41
α-D-Glucose-1,6P2
D-Fructose-6P
D-Glucose-6P
D-Glucose
00500 2/9/17
(c) Kanehisa Laboratories

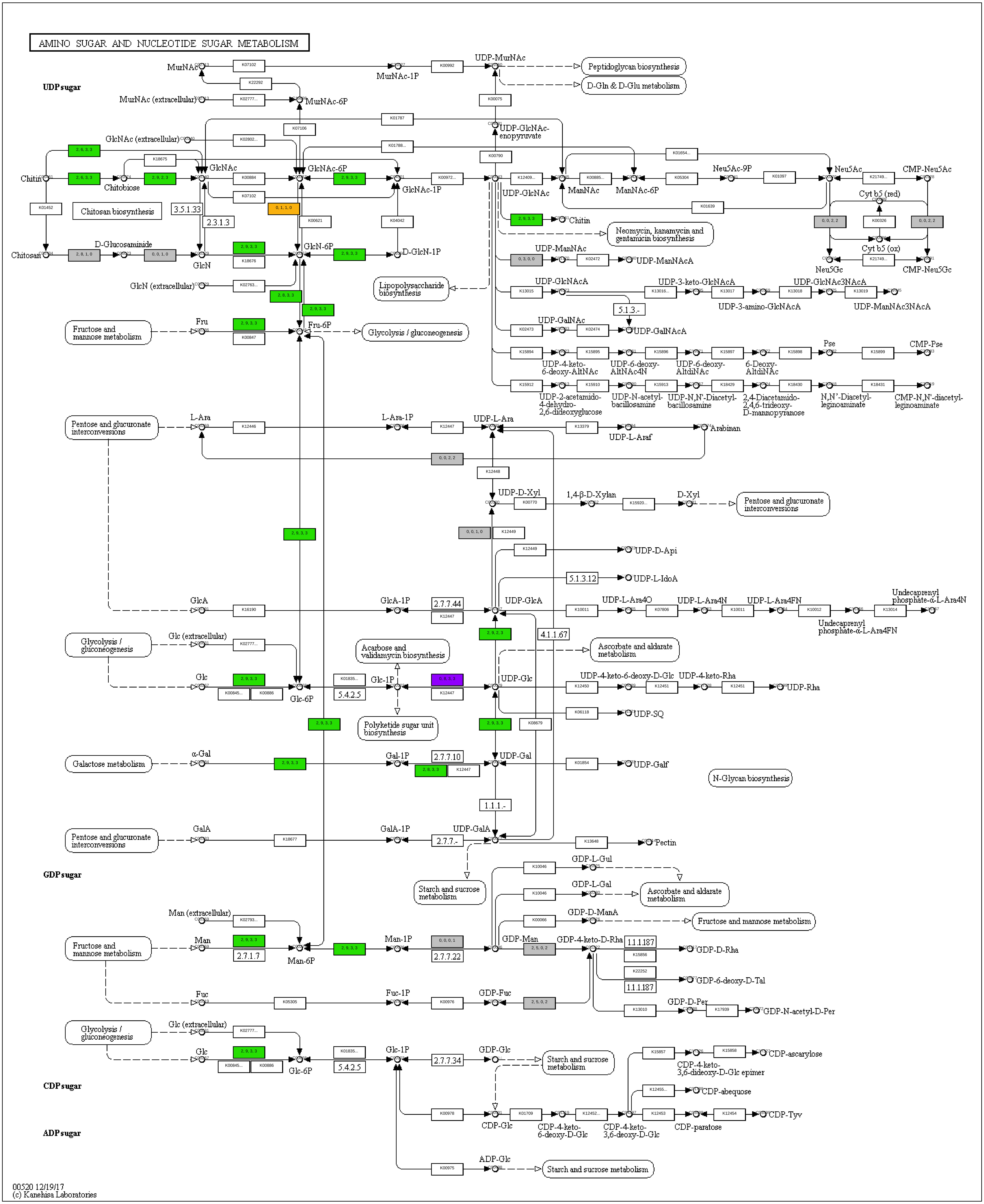

AMINO SUGAR AND NUCLEOTIDE SUGAR METABOLISM
UDP sugar
GDP sugar
CDP sugar
ADP sugar
MurNAc
MurNAc-1P
UDP-MurNAc
Peptidoglycan biosynthesis
D-Gln & D-Glu metabolism
MurNAc (extracellular)
MurNAc-6P
UDP-GlcNAc-enopyruvate
GlcNAc (extracellular)
Chitin
Chitobiose
GlcNAc
GlcNAc-6P
GlcNAc-1P
UDP-GlcNAc
ManNAc
ManNAc-6P
Neu5Ac-9P
Neu5Ac
CMP-Neu5Ac
Cyt b5 (red)
Cyt b5 (ox)
Neu5Gc
CMP-Neu5Gc
Chitosan biosynthesis
3.5.1.33
2.3.1.3
Chitosan
D-Glucosaminide
GlcN
GlcN-6P
D-GlcN-1P
Neomycin, kanamycin and gentamicin biosynthesis
UDP-ManNAc
UDP-ManNAcA
GlcN (extracellular)
Lipopolysaccharide biosynthesis
UDP-GlcNAcA
UDP-3-keto-GlcNAcA
UDP-GlcNAc3NAcA
UDP-3-amino-GlcNAcA
UDP-ManNAc3NAcA
5.1.3.-
Fructose and mannose metabolism
Fru
Fru-6P
Glycolysis / gluconeogenesis
UDP-GalNAc
Pse
CMP-Pse
UDP-4-keto-6-deoxy-AltNAc
UDP-6-deoxy-AltNAc4N
UDP-6-deoxy-AltdiNAc
6-Deoxy-AltdiNAc
UDP-2-acetamido-4-dehydro-2,6-dideoxyglucose
UDP-N-acetyl-bacillosamine
UDP-N,N'-Diacetyl-bacillosamine
2,4-Diacetamido-2,4,6-trideoxy-D-mannopyranose
N,N'-Diacetyl-legionaminate
CMP-N,N'-diacetyl-legionaminate
Pentose and glucuronate interconversions
L-Ara
L-Ara-1P
UDP-L-Ara
UDP-L-Araf
Arabinan
UDP-D-Xyl
1,4-β-D-Xylan
D-Xyl
UDP-D-Api
5.1.3.12
UDP-L-IdoA
GlcA
GlcA-1P
2.7.7.44
UDP-GlcA
UDP-L-Ara4O
UDP-L-Ara4N
UDP-L-Ara4FN
Undecaprenyl phosphate-α-L-Ara4N
Undecaprenyl phosphate-α-L-Ara4FN
4.1.1.67
Glc (extracellular)
Acarbose and validamycin biosynthesis
Ascorbate and aldarate metabolism
Glc
Glc-6P
Glc-1P
5.4.2.5
UDP-Glc
UDP-4-keto-6-deoxy-D-Glc
UDP-4-keto-Rha
UDP-Rha
UDP-SQ
Polyketide sugar unit biosynthesis
Galactose metabolism
α-Gal
Gal-1P
2.7.7.10
UDP-Gal
UDP-Galf
N-Glycan biosynthesis
1.1.1.-
GalA
GalA-1P
2.7.7.-
UDP-GalA
Pectin
Starch and sucrose metabolism
GDP-L-Gul
GDP-L-Gal
GDP-D-ManA
Man (extracellular)
Man
Man-6P
2.7.1.7
Man-1P
2.7.7.22
GDP-Man
GDP-4-keto-D-Rha
1.1.1.187
GDP-D-Rha
GDP-6-deoxy-D-Tal
Fuc
Fuc-1P
GDP-Fuc
GDP-D-Per
GDP-N-acetyl-D-Per
GDP-Glc
2.7.7.34
CDP-ascarylose
CDP-4-keto-3,6-dideoxy-D-Glc epimer
CDP-abequose
CDP-Glc
CDP-4-keto-6-deoxy-D-Glc
CDP-4-keto-3,6-deoxy-D-Glc
CDP-paratose
CDP-Tyv
ADP-Glc
00520 12/19/17
(c) Kanehisa Laboratories

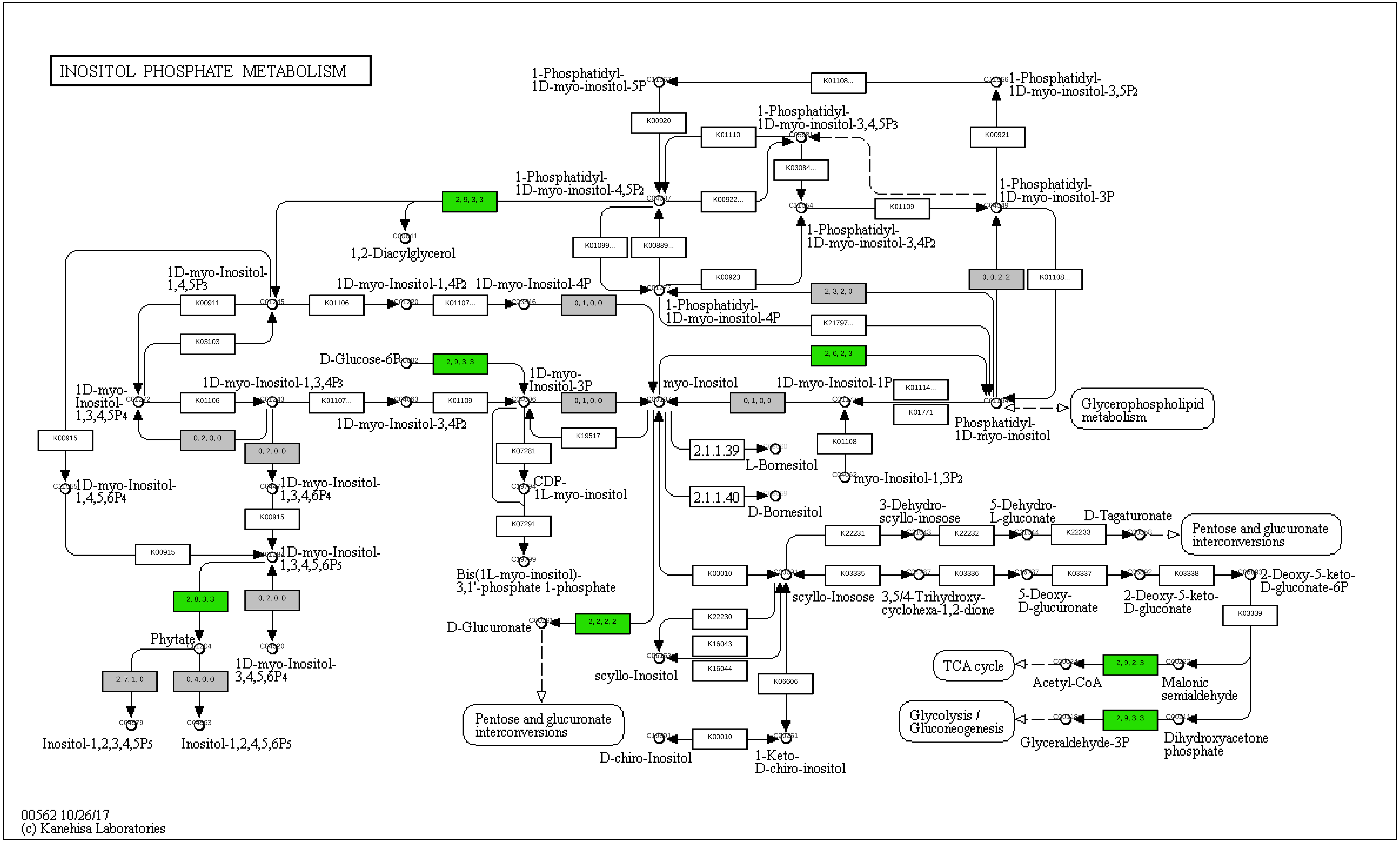
INOSITOL PHOSPHATE METABOLISM
1-Phosphatidyl-1D-myo-inositol-5P
K01108...
1-Phosphatidyl-1D-myo-inositol-3,5P2
K00920
K01110
1-Phosphatidyl-1D-myo-inositol-3,4,5P3
K00921
K03084...
2, 9, 3, 3
1-Phosphatidyl-1D-myo-inositol-4,5P2
K00922...
1-Phosphatidyl-1D-myo-inositol-3P
K01109
1,2-Diacylglycerol
1-Phosphatidyl-1D-myo-inositol-3,4P2
K01099...
K00889...
1D-myo-Inositol-1,4,5P3
K00923
0, 0, 2, 2
K01108...
1D-myo-Inositol-1,4P2
1D-myo-Inositol-4P
2, 3, 2, 0
K00911
K01106
K01107...
0, 1, 0, 0
1-Phosphatidyl-1D-myo-inositol-4P
K21797...
K03103
2, 6, 2, 3
D-Glucose-6P
2, 9, 3, 3
1D-myo-Inositol-3P
myo-Inositol
1D-myo-Inositol-1P
K01114...
1D-myo-Inositol-1,3,4P3
1D-myo-Inositol-1,3,4,5P4
K01106
K01107...
K01109
0, 1, 0, 0
0, 1, 0, 0
K01771
Glycerophospholipid metabolism
Phosphatidyl-1D-myo-inositol
K00915
0, 2, 0, 0
1D-myo-Inositol-3,4P2
K19517
K07281
0, 2, 0, 0
2.1.1.39
L-Bornesitol
K01108
myo-Inositol-1,3P2
1D-myo-Inositol-1,4,5,6P4
1D-myo-Inositol-1,3,4,6P4
CDP-1L-myo-inositol
2.1.1.40
D-Bornesitol
K00915
K07291
3-Dehydro-scyllo-inosose
5-Dehydro-L-gluconate
D-Tagaturonate
K22231
K22232
K22233
Pentose and glucuronate interconversions
K00915
1D-myo-Inositol-1,3,4,5,6P5
Bis(1L-myo-inositol)-3,1'-phosphate 1-phosphate
K00010
K03335
K03336
K03337
K03338
2-Deoxy-5-keto-D-gluconate-6P
2, 8, 3, 3
0, 2, 0, 0
scyllo-Inosose
3,5/4-Trihydroxy-cyclohexa-1,2-dione
5-Deoxy-D-glucuronate
2-Deoxy-5-keto-D-gluconate
K03339
D-Glucuronate
2, 2, 2, 2
K22230
Phytate
K16043
1D-myo-Inositol-3,4,5,6P4
TCA cycle
2, 9, 2, 3
Acetyl-CoA
Malonic semialdehyde
2, 7, 1, 0
0, 4, 0, 0
scyllo-Inositol
K16044
K06606
Glycolysis / Gluconeogenesis
2, 9, 3, 3
Glyceraldehyde-3P
Dihydroxyacetone phosphate
Pentose and glucuronate interconversions
K00010
Inositol-1,2,3,4,5P5
Inositol-1,2,4,5,6P5
D-chiro-Inositol
1-Keto-D-chiro-inositol
00562 10/26/17
(c) Kanehisa Laboratories

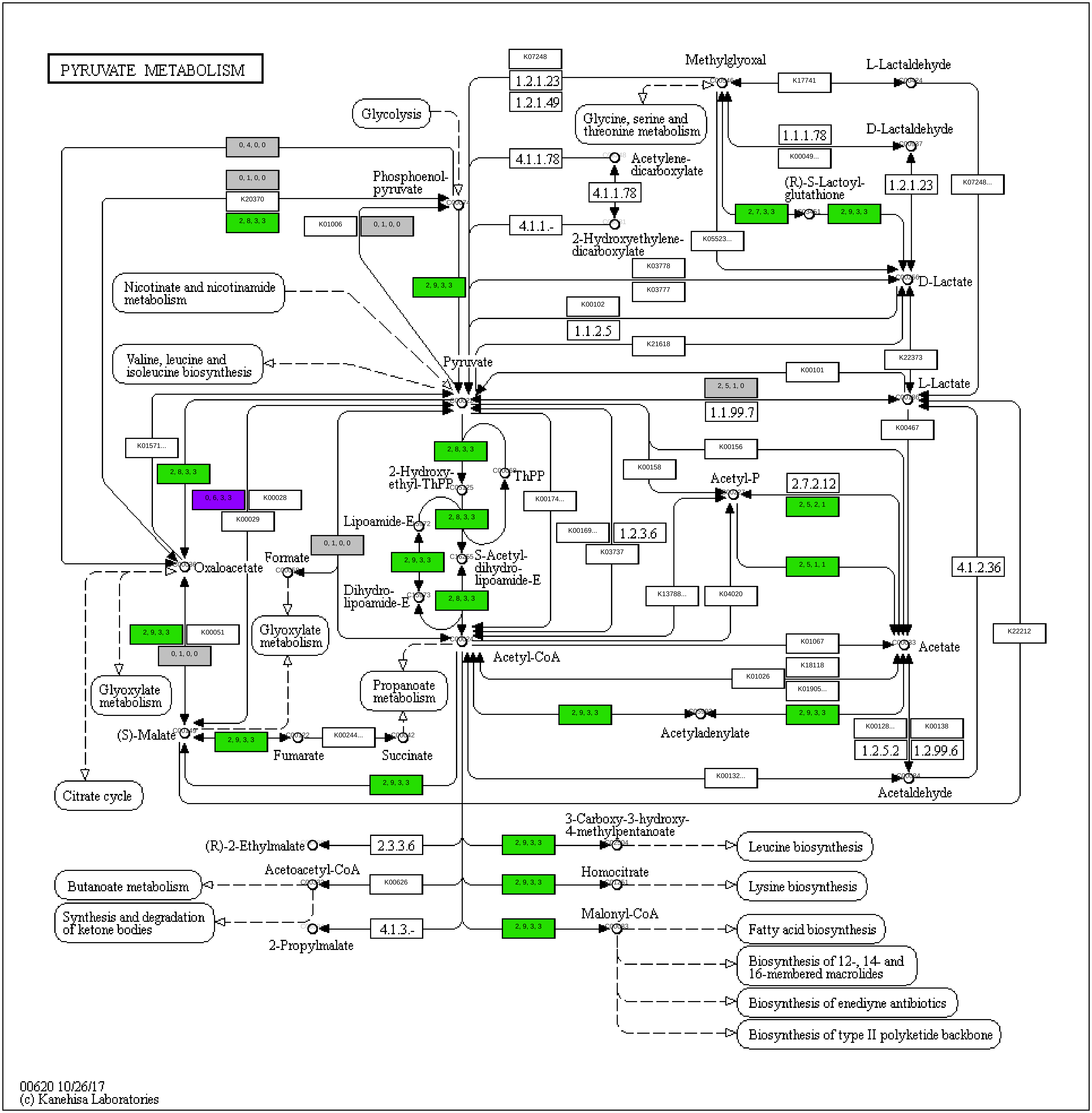
PYRUVATE METABOLISM
Glycolysis
Phosphoenol-pyruvate
Methylglyoxal
L-Lactaldehyde
D-Lactaldehyde
Glycine, serine and threonine metabolism
Acetylene-dicarboxylate
2-Hydroxyethylene-dicarboxylate
(R)-S-Lactoyl-glutathione
D-Lactate
L-Lactate
Nicotinate and nicotinamide metabolism
Valine, leucine and isoleucine biosynthesis
Pyruvate
2-Hydroxy-ethyl-ThPP
ThPP
Lipoamide-E
S-Acetyl-dihydro-lipoamide-E
Dihydro-lipoamide-E
Acetyl-P
Acetyl-CoA
Oxaloacetate
Formate
Glyoxylate metabolism
Propanoate metabolism
(S)-Malate
Fumarate
Succinate
Citrate cycle
Acetyladenylate
Acetate
Acetaldehyde
(R)-2-Ethylmalate
3-Carboxy-3-hydroxy-4-methylpentanoate
Leucine biosynthesis
Acetoacetyl-CoA
Homocitrate
Lysine biosynthesis
Butanoate metabolism
Synthesis and degradation of ketone bodies
2-Propylmalate
Malonyl-CoA
Fatty acid biosynthesis
Biosynthesis of 12-, 14- and 16-membered macrolides
Biosynthesis of enediyne antibiotics
Biosynthesis of type II polyketide backbone
K07248
1.2.1.23
1.2.1.49
K17741
1.1.1.78
K00049...
1.2.1.23
K07248...
4.1.1.78
4.1.1.78
4.1.1.-
2, 7, 3, 3
2, 9, 3, 3
K05523...
K03778
K03777
K00102
1.1.2.5
K21618
K22373
K00101
2, 5, 1, 0
1.1.99.7
K00467
0, 4, 0, 0
0, 1, 0, 0
K20370
2, 8, 3, 3
K01006
0, 1, 0, 0
2, 9, 3, 3
K01571...
2, 8, 3, 3
0, 6, 3, 3
K00028
K00029
0, 1, 0, 0
2, 8, 3, 3
2, 8, 3, 3
2, 9, 3, 3
2, 8, 3, 3
K00174...
K00156
K00158
2.7.2.12
2, 5, 2, 1
K00169...
1.2.3.6
K03737
2, 5, 1, 1
4.1.2.36
K13788...
K04020
K22212
2, 9, 3, 3
K00051
0, 1, 0, 0
K01067
K18118
K01026
K01905...
2, 9, 3, 3
2, 9, 3, 3
K00128...
K00138
1.2.5.2
1.2.99.6
2, 9, 3, 3
K00244...
2, 9, 3, 3
K00132...
2.3.3.6
2, 9, 3, 3
K00626
2, 9, 3, 3
4.1.3.-
2, 9, 3, 3
00620 10/26/17
(c) Kanehisa Laboratories

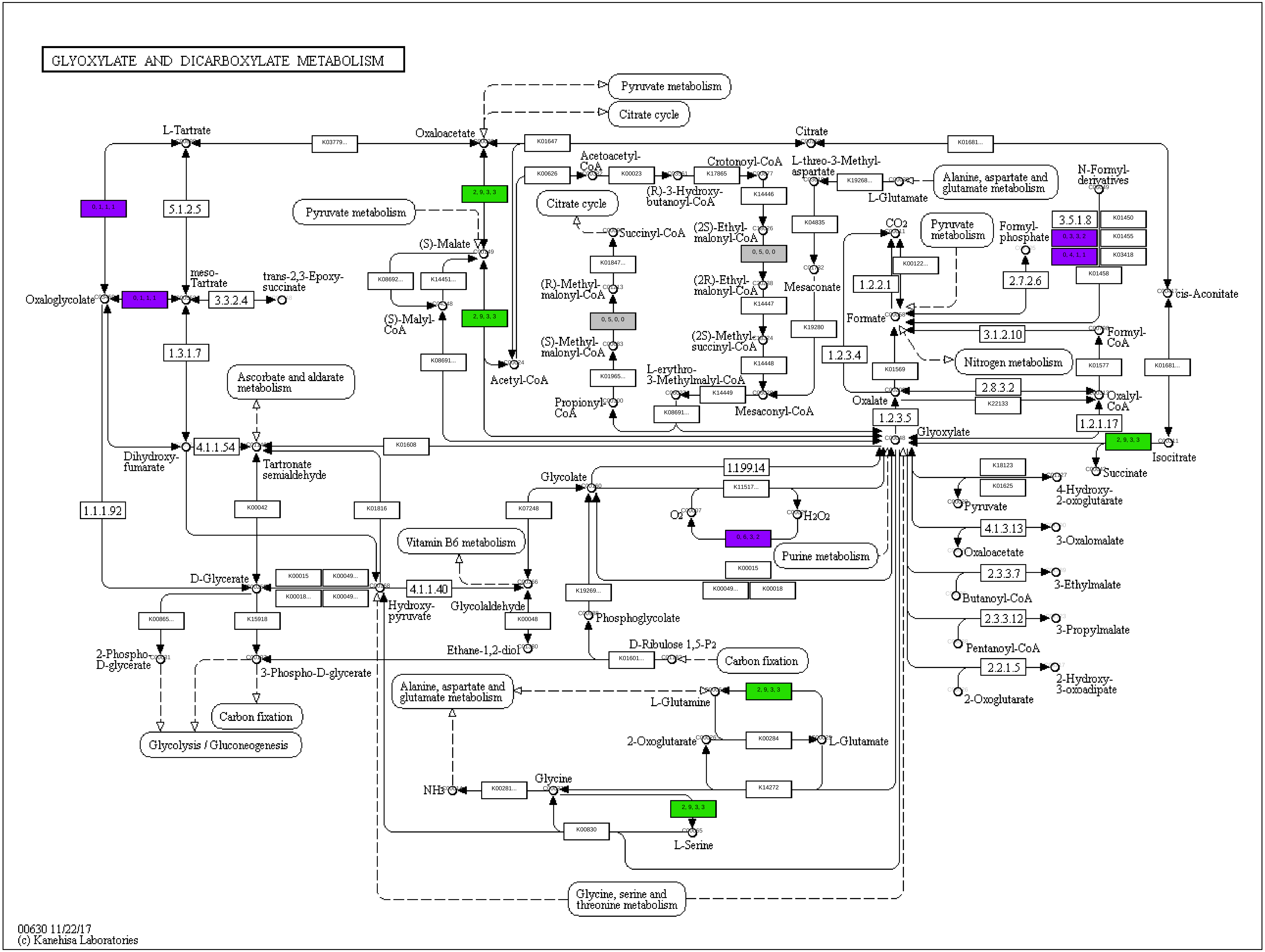
GLYOXYLATE AND DICARBOXYLATE METABOLISM
Pyruvate metabolism
Citrate cycle
L-Tartrate
K03779...
Oxaloacetate
K01647
Citrate
K01681...
0, 1, 1, 1
5.1.2.5
2, 9, 3, 3
K00626
Acetoacetyl-CoA
K00023
Crotonoyl-CoA
K17865
L-threo-3-Methyl-aspartate
K19268...
Alanine, aspartate and glutamate metabolism
L-Glutamate
N-Formyl-derivatives
Pyruvate metabolism
(S)-Malate
Citrate cycle
(R)-3-Hydroxy-butanoyl-CoA
K14446
Succinyl-CoA
(2S)-Ethyl-malonyl-CoA
K04835
CO2
Pyruvate metabolism
Formyl-phosphate
3.5.1.8
K01450
0, 3, 3, 2
K01455
0, 4, 1, 1
K03418
K01458
K08692...
K14451...
K01847...
0, 5, 0, 0
K00122...
Oxaloglycolate
0, 1, 1, 1
meso-Tartrate
3.3.2.4
trans-2,3-Epoxy-succinate
(R)-Methyl-malonyl-CoA
(2R)-Ethyl-malonyl-CoA
Mesaconate
1.2.2.1
2.7.2.6
cis-Aconitate
(S)-Malyl-CoA
2, 9, 3, 3
0, 5, 0, 0
K14447
Formate
K19280
3.1.2.10
Formyl-CoA
1.3.1.7
K08691...
(S)-Methyl-malonyl-CoA
(2S)-Methyl-succinyl-CoA
1.2.3.4
Nitrogen metabolism
Ascorbate and aldarate metabolism
Acetyl-CoA
K01965...
K14448
K01569
K01577
K01681...
L-erythro-3-Methylmalyl-CoA
K14449
2.8.3.2
Oxalyl-CoA
Propionyl-CoA
K08691...
Mesaconyl-CoA
Oxalate
K22133
1.2.3.5
1.2.1.17
Glyoxylate
Dihydroxy-fumarate
4.1.1.54
K01608
2, 9, 3, 3
Isocitrate
Tartronate semialdehyde
1.1.99.14
K18123
Succinate
Glycolate
K11517...
K01625
4-Hydroxy-2-oxoglutarate
Pyruvate
1.1.1.92
K00042
K01816
K07248
O2
H2O2
4.1.3.13
3-Oxalomalate
Vitamin B6 metabolism
0, 6, 3, 2
Oxaloacetate
Purine metabolism
K00015
2.3.3.7
3-Ethylmalate
D-Glycerate
K00015
K00049...
K00018...
K00049...
4.1.1.40
K19269...
K00049...
K00018
Butanoyl-CoA
Hydroxy-pyruvate
Glycolaldehyde
2.3.3.12
K00865...
K15918
K00048
Phosphoglycolate
3-Propylmalate
Pentanoyl-CoA
2-Phospho-D-glycerate
Ethane-1,2-diol
D-Ribulose 1,5-P2
K01601...
Carbon fixation
2.2.1.5
3-Phospho-D-glycerate
2-Hydroxy-3-oxoadipate
Alanine, aspartate and glutamate metabolism
2, 9, 3, 3
L-Glutamine
2-Oxoglutarate
Carbon fixation
Glycolysis / Gluconeogenesis
2-Oxoglutarate
K00284
L-Glutamate
Glycine
NH3
K00281...
K14272
2, 9, 3, 3
K00830
L-Serine
Glycine, serine and threonine metabolism
00630 11/22/17
(c) Kanehisa Laboratories

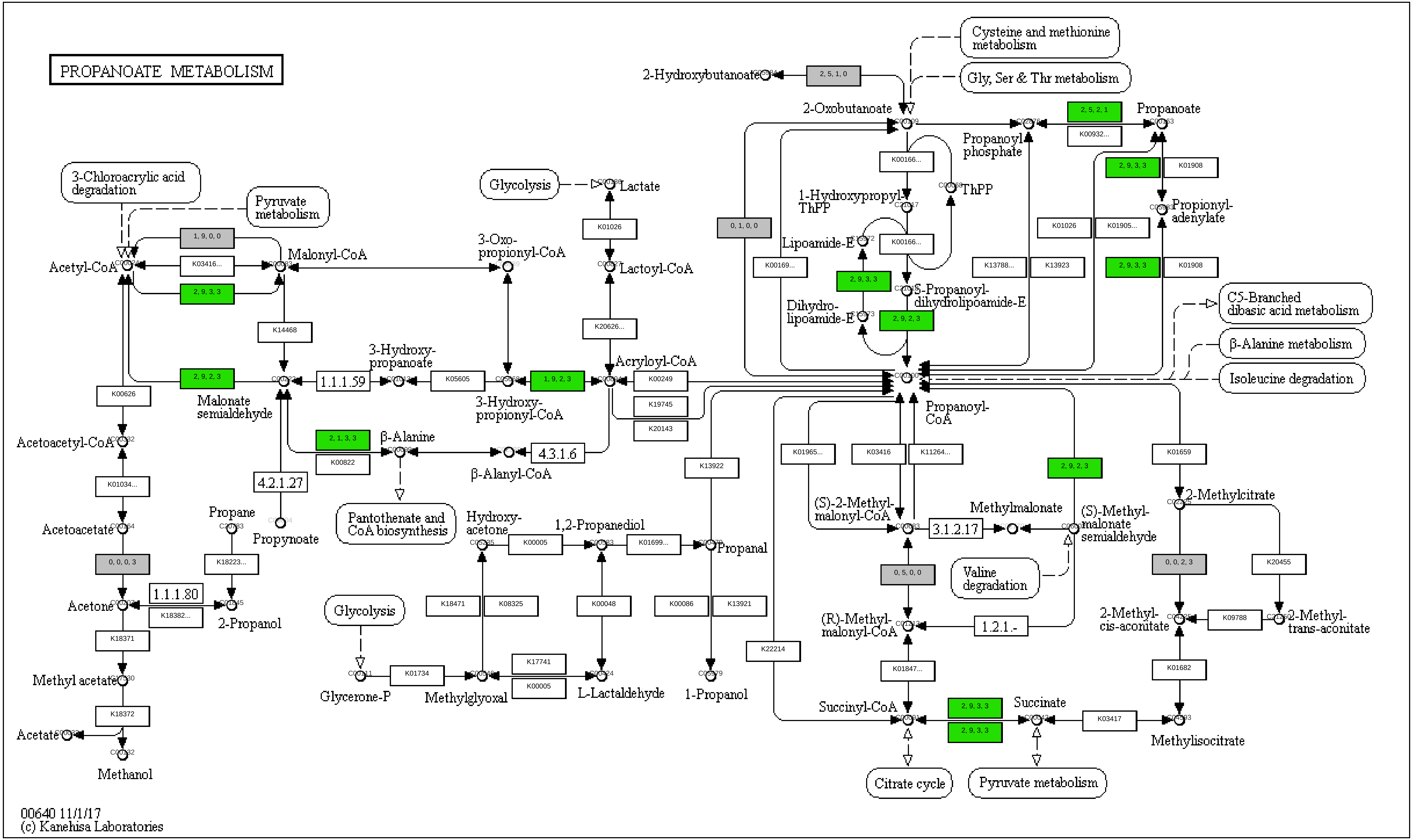
PROPANOATE METABOLISM
2-Hydroxybutanoate
2, 5, 1, 0
Cysteine and methionine metabolism
Gly, Ser & Thr metabolism
2-Oxobutanoate
2, 5, 2, 1
K00932...
Propanoate
Propanoyl phosphate
K00166...
1-Hydroxypropyl-ThPP
ThPP
0, 1, 0, 0
Lipoamide-E
K00166...
K00169...
2, 9, 3, 3
S-Propanoyl-dihydrolipoamide-E
Dihydro-lipoamide-E
2, 9, 2, 3
2, 9, 3, 3
K01908
Propionyl-adenylate
K01026
K01905...
K13788...
K13923
2, 9, 3, 3
K01908
C5-Branched dibasic acid metabolism
β-Alanine metabolism
Isoleucine degradation
3-Chloroacrylic acid degradation
Pyruvate metabolism
Glycolysis
Lactate
K01026
Lactoyl-CoA
K20626...
Acryloyl-CoA
1, 9, 0, 0
K03416...
2, 9, 3, 3
Acetyl-CoA
Malonyl-CoA
3-Oxo-propionyl-CoA
K14468
3-Hydroxy-propanoate
2, 9, 2, 3
1.1.1.59
K05605
1, 9, 2, 3
K00249
K19745
K20143
K00626
Malonate semialdehyde
3-Hydroxy-propionyl-CoA
Propanoyl-CoA
2, 1, 3, 3
K00822
β-Alanine
4.3.1.6
β-Alanyl-CoA
4.2.1.27
Propynoate
Acetoacetyl-CoA
K01034...
Acetoacetate
Propane
Pantothenate and CoA biosynthesis
Hydroxy-acetone
1,2-Propanediol
K00005
K01699...
Propanal
K13922
K01965...
K03416
K11264...
2, 9, 2, 3
K01659
2-Methylcitrate
(S)-2-Methyl-malonyl-CoA
Methylmalonate
3.1.2.17
(S)-Methyl-malonate semialdehyde
0, 0, 0, 3
K18223...
0, 5, 0, 0
Valine degradation
0, 0, 2, 3
K20455
1.1.1.80
Acetone
2-Propanol
K18382...
K18471
K08325
K00048
K00086
K13921
(R)-Methyl-malonyl-CoA
1.2.1.-
2-Methyl-cis-aconitate
K09788
2-Methyl-trans-aconitate
K18371
Glycolysis
K01734
K17741
K00005
K22214
K01847...
K01682
Methyl acetate
Glycerone-P
Methylglyoxal
L-Lactaldehyde
1-Propanol
Succinyl-CoA
2, 9, 3, 3
2, 9, 3, 3
Succinate
K03417
Methylisocitrate
K18372
Acetate
Methanol
Citrate cycle
Pyruvate metabolism
00640 11/1/17
(c) Kanehisa Laboratories

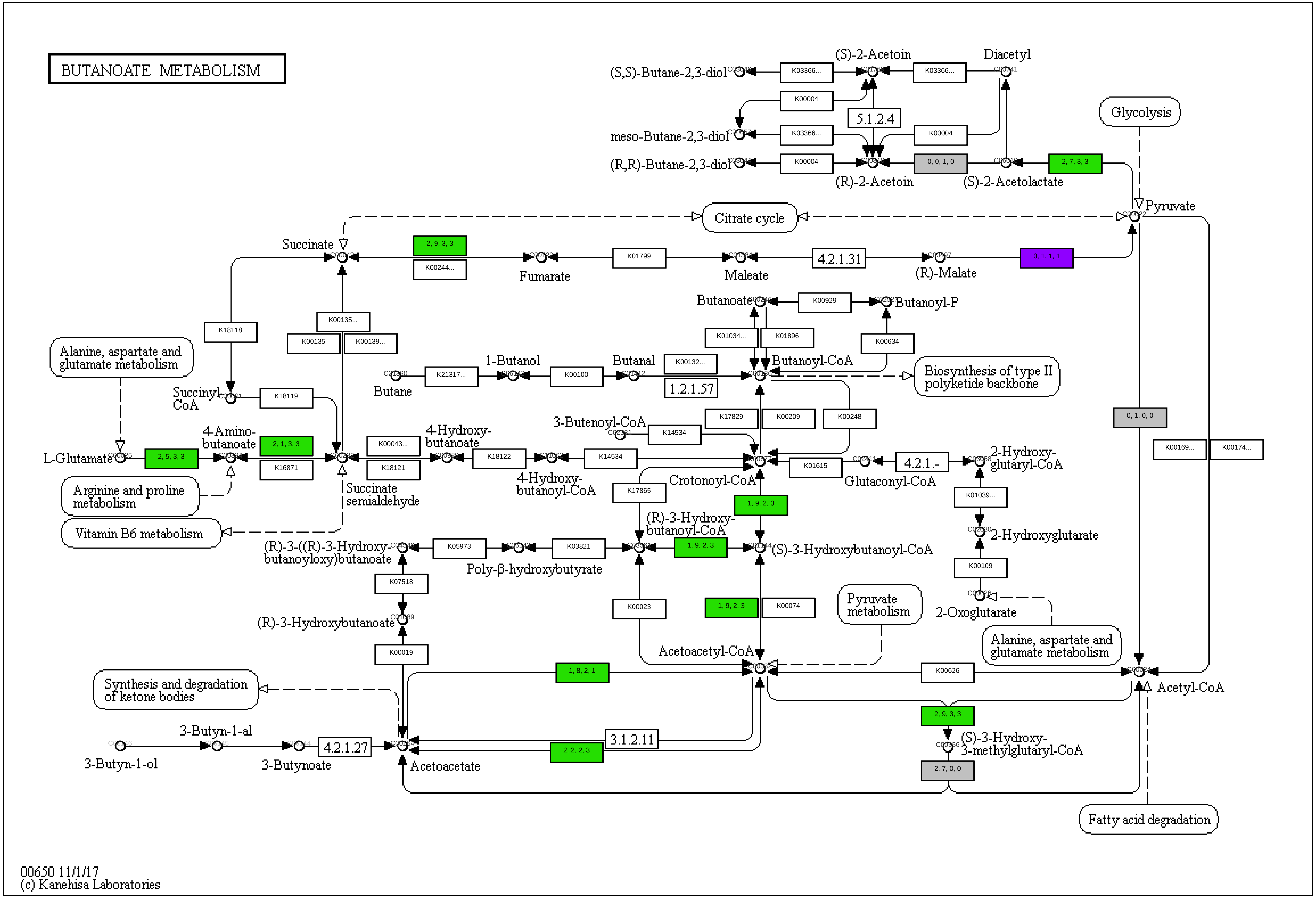
BUTANOATE METABOLISM
(S,S)-Butane-2,3-diol
(S)-2-Acetoin
Diacetyl
K03366...
K00004
5.1.2.4
meso-Butane-2,3-diol
(R,R)-Butane-2,3-diol
(R)-2-Acetoin
0, 0, 1, 0
(S)-2-Acetolactate
2, 7, 3, 3
Glycolysis
Pyruvate
Citrate cycle
Succinate
2, 9, 3, 3
K00244...
Fumarate
K01799
Maleate
4.2.1.31
(R)-Malate
0, 1, 1, 1
Butanoate
K00929
Butanoyl-P
K18118
K00135...
K00135
K00139...
K01034...
K01896
K00634
Alanine, aspartate and glutamate metabolism
1-Butanol
Butanal
K00132...
Butanoyl-CoA
Biosynthesis of type II polyketide backbone
Butane
K21317...
K00100
1.2.1.57
Succinyl-CoA
K18119
3-Butenoyl-CoA
K17829
K00209
K00248
0, 1, 0, 0
4-Amino-butanoate
4-Hydroxy-butanoate
K14534
L-Glutamate
2, 5, 3, 3
2, 1, 3, 3
K16871
K00043...
K18121
K18122
4-Hydroxy-butanoyl-CoA
K01615
Glutaconyl-CoA
4.2.1.-
2-Hydroxy-glutaryl-CoA
K00169...
K00174...
Arginine and proline metabolism
Succinate semialdehyde
Crotonoyl-CoA
K17865
1, 9, 2, 3
K01039...
Vitamin B6 metabolism
(R)-3-Hydroxy-butanoyl-CoA
2-Hydroxyglutarate
(R)-3-((R)-3-Hydroxy-butanoyloxy)butanoate
K05973
K03821
Poly-β-hydroxybutyrate
(S)-3-Hydroxybutanoyl-CoA
K00109
K07518
K00023
K00074
Pyruvate metabolism
2-Oxoglutarate
(R)-3-Hydroxybutanoate
Alanine, aspartate and glutamate metabolism
K00019
Acetoacetyl-CoA
1, 8, 2, 1
K00626
Acetyl-CoA
Synthesis and degradation of ketone bodies
2, 9, 3, 3
3-Butyn-1-al
3.1.2.11
(S)-3-Hydroxy-3-methylglutaryl-CoA
4.2.1.27
2, 2, 2, 3
3-Butyn-1-ol
3-Butynoate
Acetoacetate
2, 7, 0, 0
Fatty acid degradation
00650 11/1/17
(c) Kanehisa Laboratories

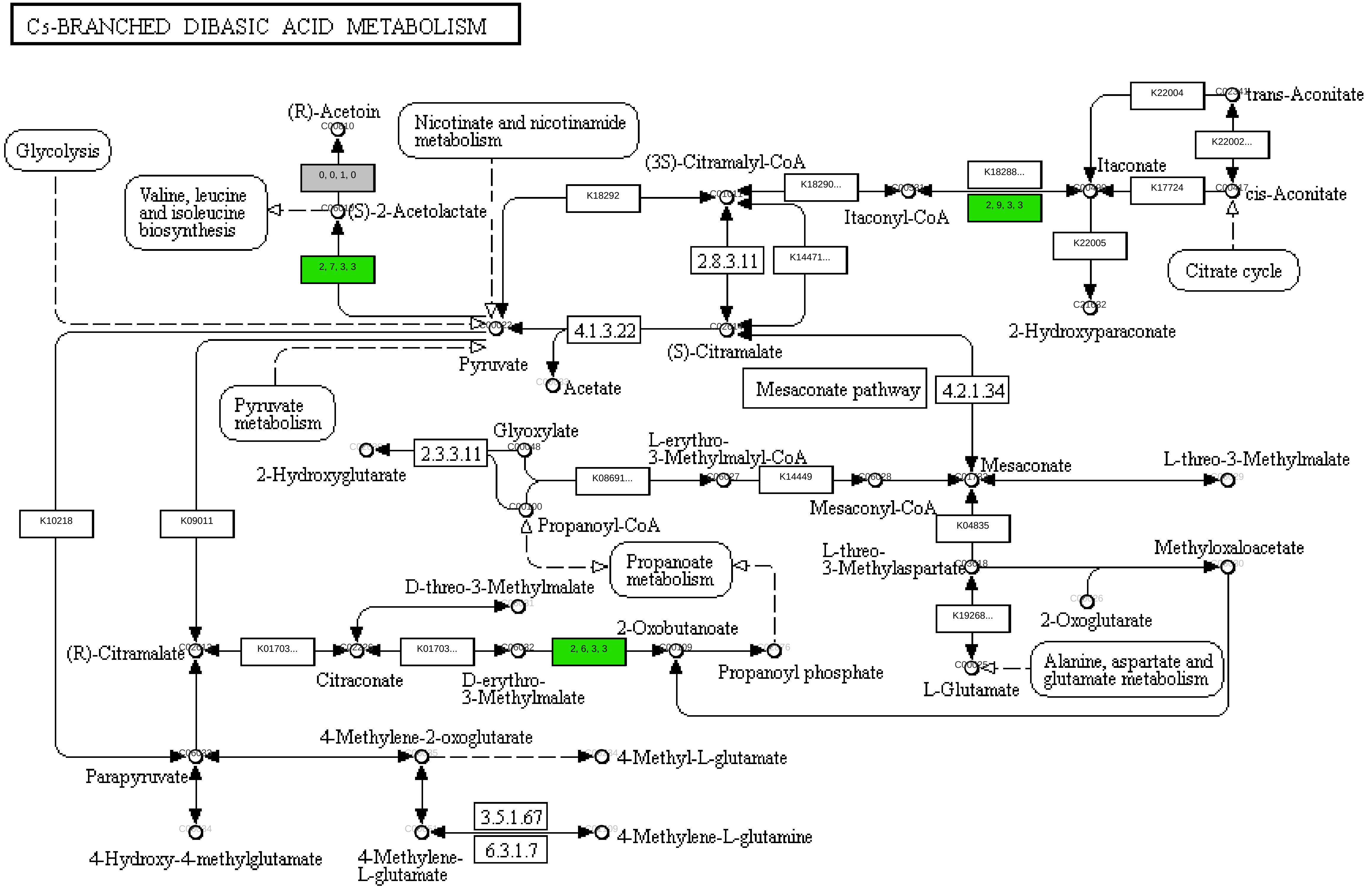

00660 11/1/17
(c) Kanehisa Laboratories

2. Energy metabolism

MAP	PATHWAY
**190**	Oxidative phosphorylation
**680**	Methane metabolism
**910**	Nitrogen metabolism
**920**	Sulfur metabolism

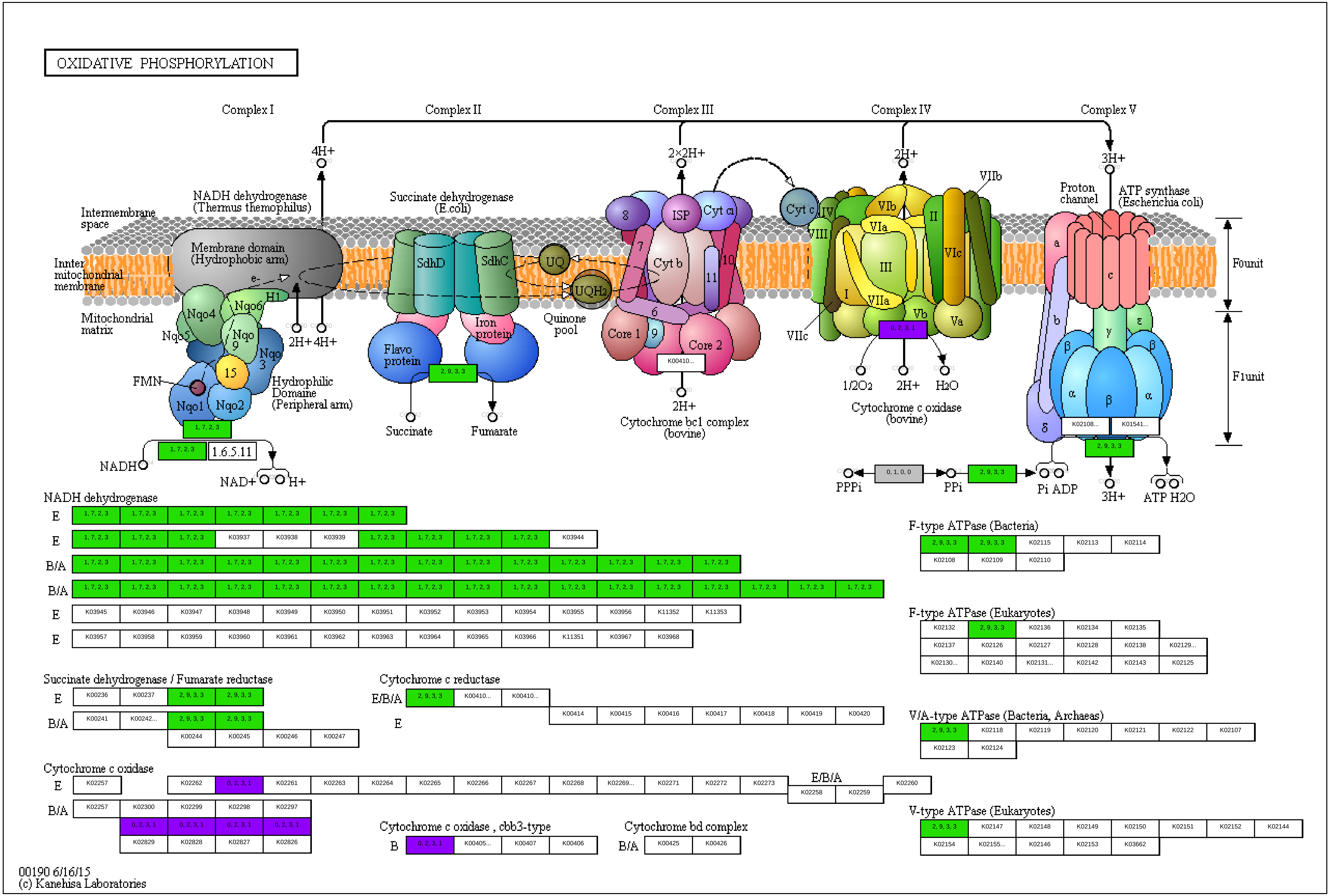
OXIDATIVE PHOSPHORYLATION
Complex I
Complex II
Complex III
Complex IV
Complex V
4H+
2×2H+
2H+
3H+
Intermembrane space
Innter mitochondrial membrane
Mitochondrial matrix
NADH dehydrogenase (Thermus themophilus)
Membrane domain (Hydrophobic arm)
e-
H1
Nqo4
Nqo6
Nqo5
Nqo 9
Nqo 3
15
FMN
Nqo1
Nqo2
Hydrophilic Domaine (Peripheral arm)
2H+
4H+
1, 7, 2, 3
1.6.5.11
NADH
NAD+
H+
Succinate dehydrogenase (E.coli)
SdhD
SdhC
Flavo protein
Iron protein
2, 9, 3, 3
Succinate
Fumarate
UQ
UQH2
Quinone pool
8
ISP
Cyt a
7
Cyt b
10
11
6
Core 1
9
Core 2
K00410...
2H+
Cytochrome bc1 complex (bovine)
Cyt c
IV
VIII
VIb
VIa
III
II
VIc
VIIb
I
VIIa
Vb
Va
VIIc
0, 2, 3, 1
1/2O2
2H+
H2O
Cytochrome c oxidase (bovine)
Proton channel
ATP synthase (Escherichia coli)
a
c
b
γ
ε
β
α
δ
Founit
F1unit
K02108...
K01541...
2, 9, 3, 3
PPPi
0, 1, 0, 0
PPi
2, 9, 3, 3
Pi ADP
3H+
ATP H2O
NADH dehydrogenase
E 1, 7, 2, 3 | 1, 7, 2, 3 | 1, 7, 2, 3 | 1, 7, 2, 3 | 1, 7, 2, 3 | 1, 7, 2, 3 | 1, 7, 2, 3
E 1, 7, 2, 3 | 1, 7, 2, 3 | 1, 7, 2, 3 | K03937 | K03938 | K03939 | 1, 7, 2, 3 | 1, 7, 2, 3 | 1, 7, 2, 3 | 1, 7, 2, 3 | K03944
B/A 1, 7, 2, 3 | 1, 7, 2, 3 | 1, 7, 2, 3 | 1, 7, 2, 3 | 1, 7, 2, 3 | 1, 7, 2, 3 | 1, 7, 2, 3 | 1, 7, 2, 3 | 1, 7, 2, 3 | 1, 7, 2, 3 | 1, 7, 2, 3 | 1, 7, 2, 3 | 1, 7, 2, 3 | 1, 7, 2, 3
B/A 1, 7, 2, 3 | 1, 7, 2, 3 | 1, 7, 2, 3 | 1, 7, 2, 3 | 1, 7, 2, 3 | 1, 7, 2, 3 | 1, 7, 2, 3 | 1, 7, 2, 3 | 1, 7, 2, 3 | 1, 7, 2, 3 | 1, 7, 2, 3 | 1, 7, 2, 3 | 1, 7, 2, 3 | 1, 7, 2, 3 | 1, 7, 2, 3 | 1, 7, 2, 3 | 1, 7, 2, 3
E K03945 | K03946 | K03947 | K03948 | K03949 | K03950 | K03951 | K03952 | K03953 | K03954 | K03955 | K03956 | K11352 | K11353
E K03957 | K03958 | K03959 | K03960 | K03961 | K03962 | K03963 | K03964 | K03965 | K03966 | K11351 | K03967 | K03968
Succinate dehydrogenase / Fumarate reductase
E K00236 | K00237 | 2, 9, 3, 3 | 2, 9, 3, 3
B/A K00241 | K00242... | 2, 9, 3, 3 | 2, 9, 3, 3
K00244 | K00245 | K00246 | K00247
Cytochrome c reductase
E/B/A 2, 9, 3, 3 | K00410... | K00410...
E K00414 | K00415 | K00416 | K00417 | K00418 | K00419 | K00420
Cytochrome c oxidase
E K02257 | K02262 | 0, 2, 3, 1 | K02261 | K02263 | K02264 | K02265 | K02266 | K02267 | K02268 | K02269... | K02271 | K02272 | K02273
E/B/A
K02258 | K02259 | K02260
B/A K02257 | K02300 | K02299 | K02298 | K02297
0, 2, 3, 1 | 0, 2, 3, 1 | 0, 2, 3, 1 | 0, 2, 3, 1
K02829 | K02828 | K02827 | K02826
Cytochrome c oxidase , cbb3-type
B 0, 2, 3, 1 | K00405... | K00407 | K00406
Cytochrome bd complex
B/A K00425 | K00426
F-type ATPase (Bacteria)
2, 9, 3, 3 | 2, 9, 3, 3 | K02115 | K02113 | K02114
K02108 | K02109 | K02110
F-type ATPase (Eukaryotes)
K02132 | 2, 9, 3, 3 | K02136 | K02134 | K02135
K02137 | K02126 | K02127 | K02128 | K02138 | K02129...
K02130... | K02140 | K02131... | K02142 | K02143 | K02125
V/A-type ATPase (Bacteria, Archaeas)
2, 9, 3, 3 | K02118 | K02119 | K02120 | K02121 | K02122 | K02107
K02123 | K02124
V-type ATPase (Eukaryotes)
2, 9, 3, 3 | K02147 | K02148 | K02149 | K02150 | K02151 | K02152 | K02144
K02154 | K02155... | K02146 | K02153 | K03662
00190 6/16/15
(c) Kanehisa Laboratories

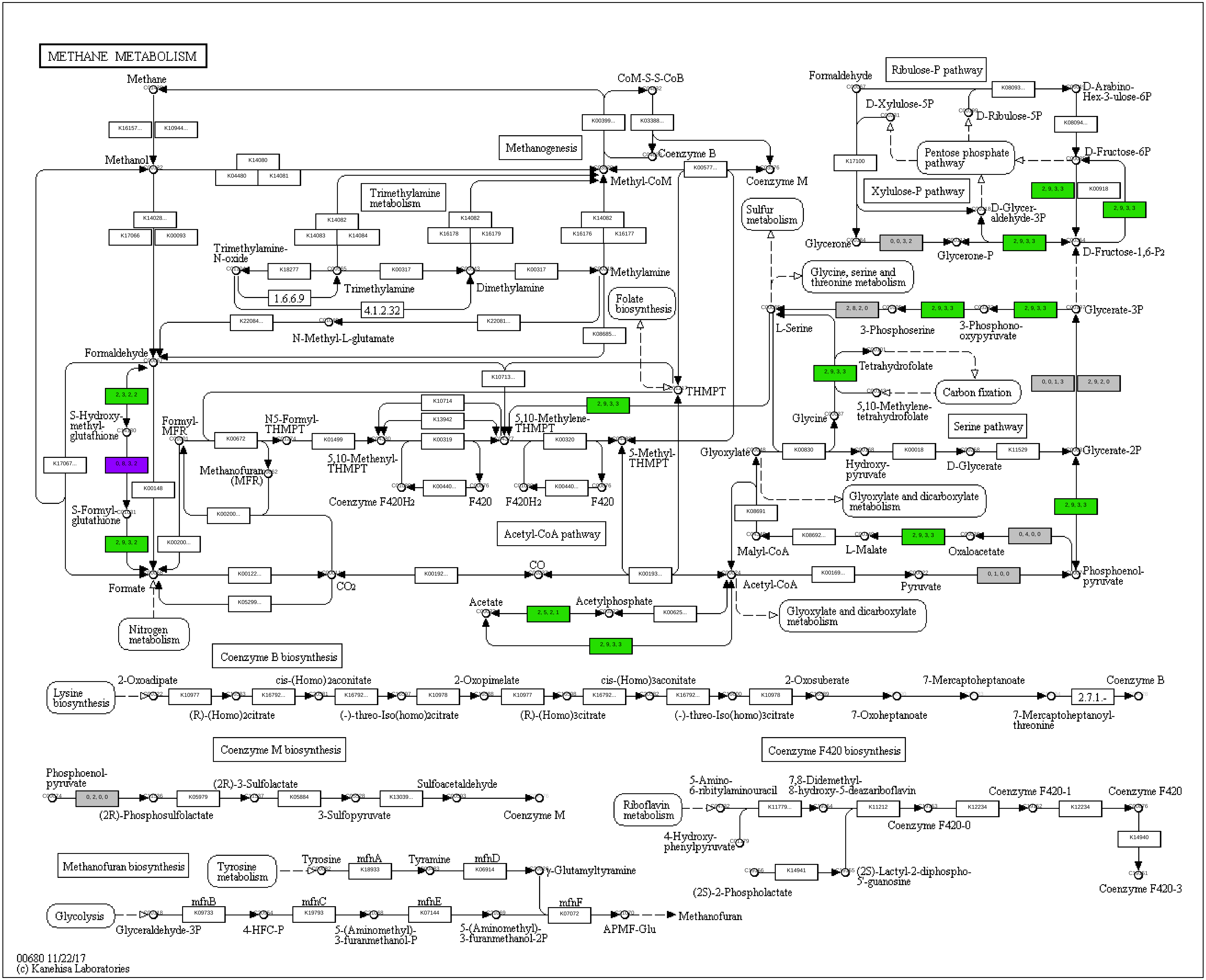

METHANE METABOLISM
Methane
CoM-S-S-CoB
K16157...
K10944...
K00399...
K03388...
Methanogenesis
Coenzyme B
Methanol
K14080
K04480
K14081
Methyl-CoM
K00577...
Coenzyme M
Trimethylamine metabolism
K14028...
K14082
K14082
K14082
K17066
K00093
K14083
K14084
K16178
K16179
K16176
K16177
Sulfur metabolism
Trimethylamine-N-oxide
K18277
K00317
K00317
Methylamine
Trimethylamine
Dimethylamine
1.6.6.9
4.1.2.32
Folate biosynthesis
K22084...
K22081...
N-Methyl-L-glutamate
K08685...
Formaldehyde
K10713...
THMPT
2.3.2.2
S-Hydroxy-methyl-glutathione
Formyl-MFR
N5-Formyl-THMPT
K10714
K13942
5,10-Methylene-THMPT
2.9.3.3
K17067...
0.8.3.2
K00148
K00672
K01499
K00319
K00320
5-Methyl-THMPT
Methanofuran (MFR)
5,10-Methenyl-THMPT
K00440...
K00440...
Coenzyme F420H2
F420
F420H2
F420
S-Formyl-glutathione
K00200...
2.9.3.2
K00200...
Acetyl-CoA pathway
CO
K00122...
K00192...
K00193...
Formate
CO2
K05299...
Acetate
2.5.2.1
Acetylphosphate
K00625...
Nitrogen metabolism
2.9.3.3
Formaldehyde
Ribulose-P pathway
K08093...
D-Arabino-Hex-3-ulose-6P
D-Xylulose-5P
D-Ribulose-5P
K08094...
Pentose phosphate pathway
D-Fructose-6P
K17100
Xylulose-P pathway
2.9.3.3
K00918
2.9.3.3
D-Glycer-aldehyde-3P
Glycerone
0.0.3.2
Glycerone-P
2.9.3.3
D-Fructose-1,6-P2
Glycine, serine and threonine metabolism
2.8.2.0
2.9.3.3
2.9.3.3
Glycerate-3P
L-Serine
3-Phosphoserine
3-Phosphono-oxypyruvate
Tetrahydrofolate
2.9.3.3
Carbon fixation
0.0.1.3
2.9.2.0
Glycine
5,10-Methylene-tetrahydrofolate
Serine pathway
Glyoxylate
K00830
K00018
K11529
Glycerate-2P
Hydroxy-pyruvate
D-Glycerate
Glyoxylate and dicarboxylate metabolism
K08691
2.9.3.3
K08692...
2.9.3.3
0.4.0.0
Malyl-CoA
L-Malate
Oxaloacetate
K00169...
0.1.0.0
Phosphoenol-pyruvate
Acetyl-CoA
Pyruvate
Glyoxylate and dicarboxylate metabolism
Coenzyme B biosynthesis
Lysine biosynthesis
2-Oxoadipate
K10977
cis-(Homo)2aconitate
K16792...
K16792...
K10978
2-Oxopimelate
K10977
cis-(Homo)3aconitate
K16792...
K16792...
K10978
2-Oxosuberate
7-Mercaptoheptanoate
Coenzyme B
2.7.1.-
(R)-(Homo)2citrate
(-)-threo-Iso(homo)2citrate
(R)-(Homo)3citrate
(-)-threo-Iso(homo)3citrate
7-Oxoheptanoate
7-Mercaptoheptanoyl-threonine
Coenzyme M biosynthesis
Coenzyme F420 biosynthesis
Phosphoenol-pyruvate
0.2.0.0
(2R)-3-Sulfolactate
K05979
K05884
Sulfoacetaldehyde
K13039...
(2R)-Phosphosulfolactate
3-Sulfopyruvate
Coenzyme M
5-Amino-6-ribitylaminouracil
7,8-Didemethyl-8-hydroxy-5-deazariboflavin
Coenzyme F420-1
Coenzyme F420
Riboflavin metabolism
K11779...
K11212
K12234
K12234
Coenzyme F420-0
4-Hydroxy-phenylpyruvate
K14940
K14941
(2S)-Lactyl-2-diphospho-5'-guanosine
(2S)-2-Phospholactate
Coenzyme F420-3
Methanofuran biosynthesis
Tyrosine metabolism
Tyrosine
mfnA
K18933
Tyramine
mfnD
K06914
γ-Glutamyltyramine
Glycolysis
mfnB
K09733
mfnC
K19793
mfnE
K07144
mfnF
K07072
Methanofuran
Glyceraldehyde-3P
4-HFC-P
5-(Aminomethyl)-3-furanmethanol-P
5-(Aminomethyl)-3-furanmethanol-2P
APMF-Glu
00680 11/22/17
(c) Kanehisa Laboratories

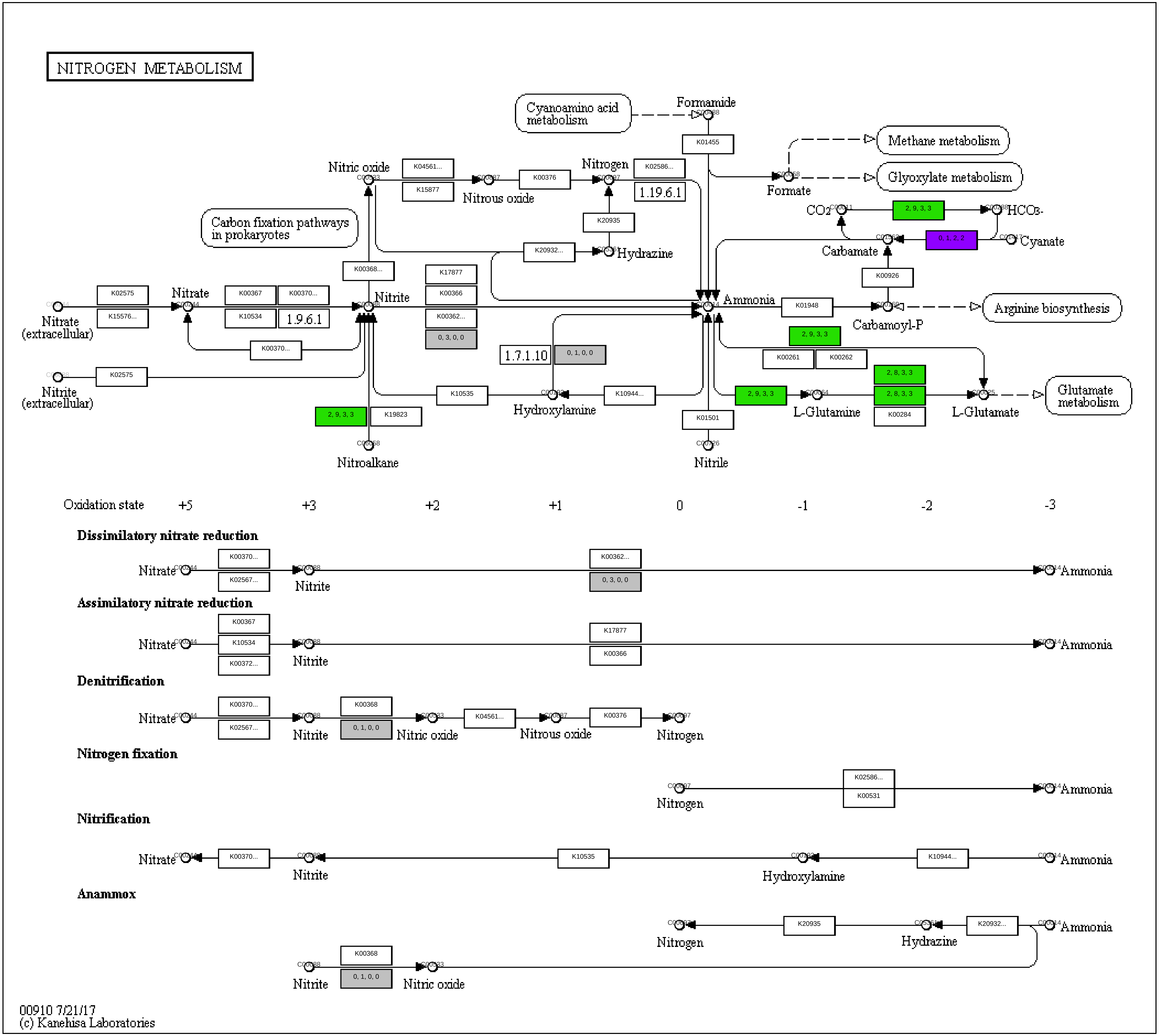
NITROGEN METABOLISM
Cyanoamino acid metabolism
Formamide
K01455
Methane metabolism
Glyoxylate metabolism
Formate
Nitric oxide
K04561...
K15877
Nitrous oxide
K00376
Nitrogen
K02586...
1.19.6.1
K20935
K20932...
Hydrazine
CO2
2, 9, 3, 3
HCO3-
Carbamate
0, 1, 2, 2
Cyanate
Carbon fixation pathways in prokaryotes
K00368...
K00926
K17877
K00366
K00362...
0, 3, 0, 0
K02575
K15576...
Nitrate
K00367
K00370...
K10534
1.9.6.1
Nitrite
Ammonia
K01948
Carbamoyl-P
Arginine biosynthesis
Nitrate (extracellular)
K00370...
1.7.1.10
0, 1, 0, 0
2, 9, 3, 3
K00261
K00262
2, 8, 3, 3
Nitrite (extracellular)
K02575
K10535
Hydroxylamine
K10944...
2, 9, 3, 3
L-Glutamine
2, 8, 3, 3
L-Glutamate
Glutamate metabolism
2, 9, 3, 3
K19823
K01501
K00284
Nitroalkane
Nitrile
Oxidation state +5 +3 +2 +1 0 -1 -2 -3
Dissimilatory nitrate reduction
Nitrate K00370... K02567... Nitrite K00362... 0, 3, 0, 0 Ammonia
Assimilatory nitrate reduction
Nitrate K00367 K10534 K00372... Nitrite K17877 K00366 Ammonia
Denitrification
Nitrate K00370... K02567... Nitrite K00368 0, 1, 0, 0 Nitric oxide K04561... Nitrous oxide K00376 Nitrogen
Nitrogen fixation
Nitrogen K02586... K00531 Ammonia
Nitrification
Nitrate K00370... Nitrite K10535 Hydroxylamine K10944... Ammonia
Anammox
Nitrogen K20935 Hydrazine K20932... Ammonia
Nitrite K00368 0, 1, 0, 0 Nitric oxide
00910 7/21/17
(c) Kanehisa Laboratories

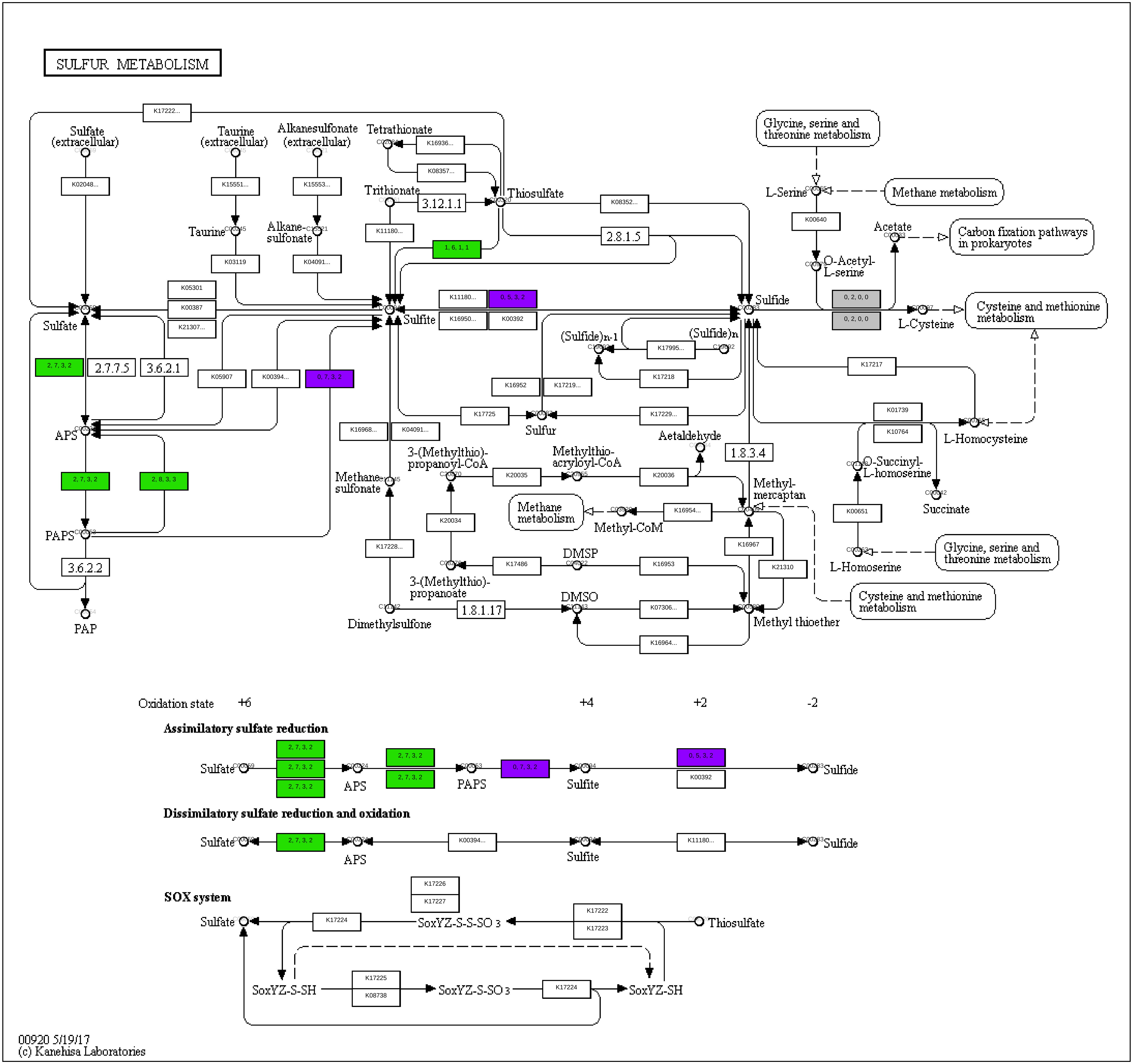

SULFUR METABOLISM
K17222...
Sulfate (extracellular)
Taurine (extracellular)
Alkanesulfonate (extracellular)
Tetrathionate
K16936...
K08357...
K02048...
K15551...
K15553...
Trithionate
3.12.1.1
Thiosulfate
K08352...
Glycine, serine and threonine metabolism
L-Serine
Methane metabolism
K00640
Acetate
Carbon fixation pathways in prokaryotes
O-Acetyl-L-serine
Taurine
K03119
Alkane-sulfonate
K04091...
K11180...
1, 6, 1, 1
2.8.1.5
K05301
K00387
K21307...
K11180...
0, 5, 3, 2
K16950...
K00392
Sulfide
0, 2, 0, 0
0, 2, 0, 0
L-Cysteine
Cysteine and methionine metabolism
Sulfate
Sulfite
(Sulfide)n-1
(Sulfide)n
K17995...
K17218
K17217
2, 7, 3, 2
2.7.7.5
3.6.2.1
K05907
K00394...
0, 7, 3, 2
K16952
K17219...
K17725
K17229...
K01739
K10764
APS
K16968...
K04091...
Sulfur
Aetaldehyde
L-Homocysteine
3-(Methylthio)-propanoyl-CoA
Methylthio-acryloyl-CoA
1.8.3.4
O-Succinyl-L-homoserine
2, 7, 3, 2
2, 8, 3, 3
Methane sulfonate
K20035
K20036
Methyl-mercaptan
Succinate
Methane metabolism
Methyl-CoM
K16954...
K00651
PAPS
K20034
K16967
K17228...
DMSP
K17486
K16953
K21310
Glycine, serine and threonine metabolism
L-Homoserine
3.6.2.2
3-(Methylthio)-propanoate
DMSO
K07306...
Cysteine and methionine metabolism
PAP
1.8.1.17
Dimethylsulfone
Methyl thioether
K16964...
Oxidation state
+6
+4
+2
-2
Assimilatory sulfate reduction
Sulfate
2, 7, 3, 2
2, 7, 3, 2
2, 7, 3, 2
APS
2, 7, 3, 2
2, 7, 3, 2
PAPS
0, 7, 3, 2
Sulfite
0, 5, 3, 2
K00392
Sulfide
Dissimilatory sulfate reduction and oxidation
Sulfate
2, 7, 3, 2
APS
K00394...
Sulfite
K11180...
Sulfide
SOX system
K17226
K17227
Sulfate
K17224
SoxYZ-S-S-SO 3
K17222
K17223
Thiosulfate
SoxYZ-S-SH
K17225
K08738
SoxYZ-S-SO 3
K17224
SoxYZ-SH
00920 5/19/17
(c) Kanehisa Laboratories

3. Lipid metabolism

MAP	PATHWAY
**61**	Fatty acid biosynthesis
**62**	Fatty acid elongation
**71**	Fatty acid degradation
**72**	Synthesis and degradation of ketone bodies
**100**	Steroid biosynthesis
**561**	Glycerolipid metabolism
**564**	Glycerophospholipid metabolism
**565**	Ether lipid metabolism
**590**	Arachidonic acid metabolism
**592**	alpha-Linolenic acid metabolism
**600**	Sphingolipid metabolism

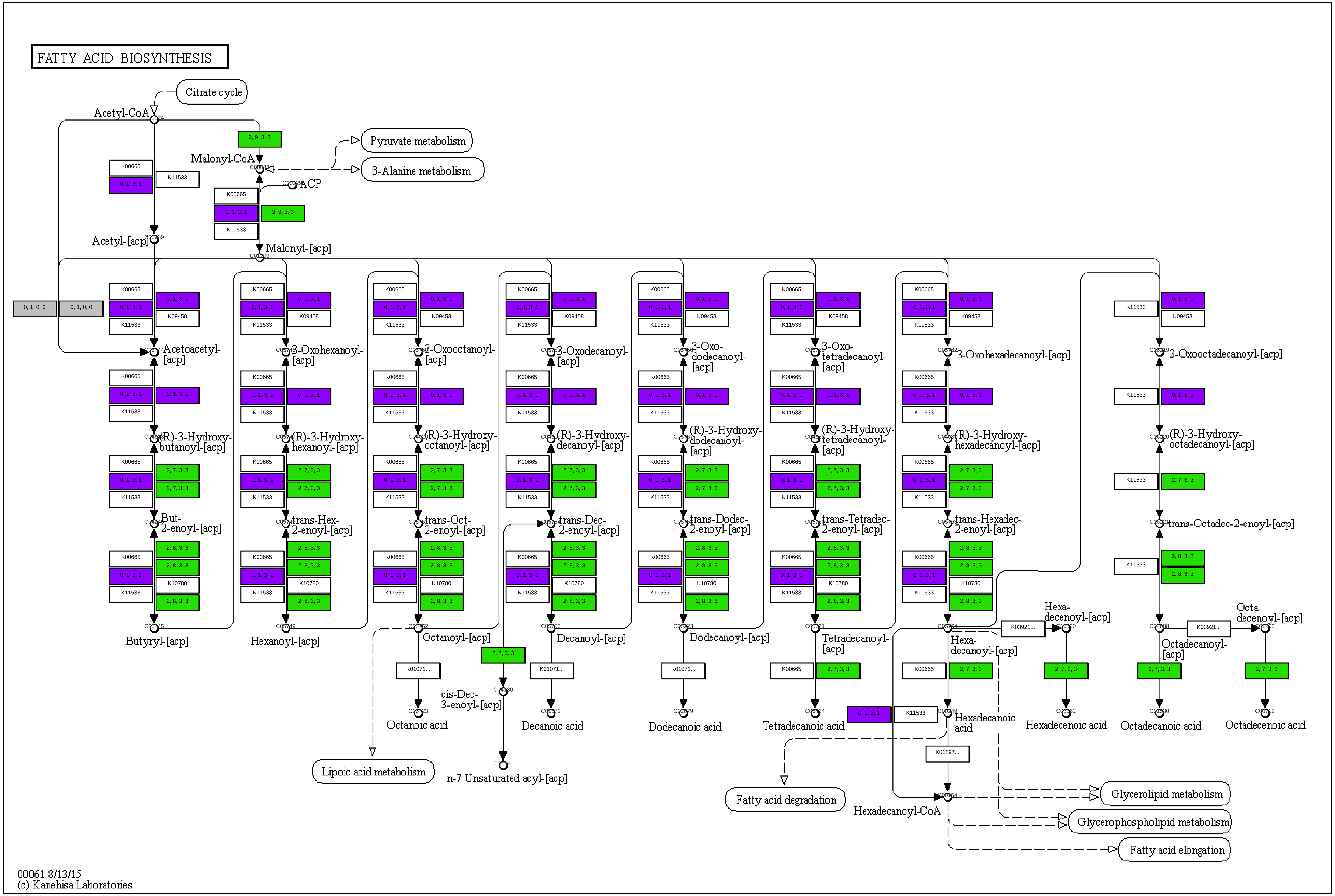
FATTY ACID BIOSYNTHESIS
Citrate cycle
Acetyl-CoA
Malonyl-CoA
Pyruvate metabolism
β-Alanine metabolism
ACP
Malonyl-[acp]
Acetyl-[acp]
2, 9, 3, 3
K00665
0, 1, 3, 1
K11533
K09458
K10780
2, 7, 3, 3
2, 8, 3, 3
0, 1, 0, 0
Acetoacetyl-[acp]
(R)-3-Hydroxy-butanoyl-[acp]
But-2-enoyl-[acp]
Butyryl-[acp]
3-Oxohexanoyl-[acp]
(R)-3-Hydroxy-hexanoyl-[acp]
trans-Hex-2-enoyl-[acp]
Hexanoyl-[acp]
3-Oxooctanoyl-[acp]
(R)-3-Hydroxy-octanoyl-[acp]
trans-Oct-2-enoyl-[acp]
Octanoyl-[acp]
K01071...
Octanoic acid
Lipoic acid metabolism
3-Oxodecanoyl-[acp]
(R)-3-Hydroxy-decanoyl-[acp]
trans-Dec-2-enoyl-[acp]
Decanoyl-[acp]
cis-Dec-3-enoyl-[acp]
n-7 Unsaturated acyl-[acp]
Decanoic acid
3-Oxo-dodecanoyl-[acp]
(R)-3-Hydroxy-dodecanoyl-[acp]
trans-Dodec-2-enoyl-[acp]
Dodecanoyl-[acp]
Dodecanoic acid
3-Oxo-tetradecanoyl-[acp]
(R)-3-Hydroxy-tetradecanoyl-[acp]
trans-Tetradec-2-enoyl-[acp]
Tetradecanoyl-[acp]
Tetradecanoic acid
Fatty acid degradation
3-Oxohexadecanoyl-[acp]
(R)-3-Hydroxy-hexadecanoyl-[acp]
trans-Hexadec-2-enoyl-[acp]
Hexa-decanoyl-[acp]
Hexadecanoic acid
K01897...
Hexadecanoyl-CoA
K03921...
Hexa-decenoyl-[acp]
Hexadecenoic acid
3-Oxooctadecanoyl-[acp]
(R)-3-Hydroxy-octadecanoyl-[acp]
trans-Octadec-2-enoyl-[acp]
Octadecanoyl-[acp]
Octadecanoic acid
Octa-decenoyl-[acp]
Octadecenoic acid
Glycerolipid metabolism
Glycerophospholipid metabolism
Fatty acid elongation
00061 8/13/15
(c) Kanehisa Laboratories

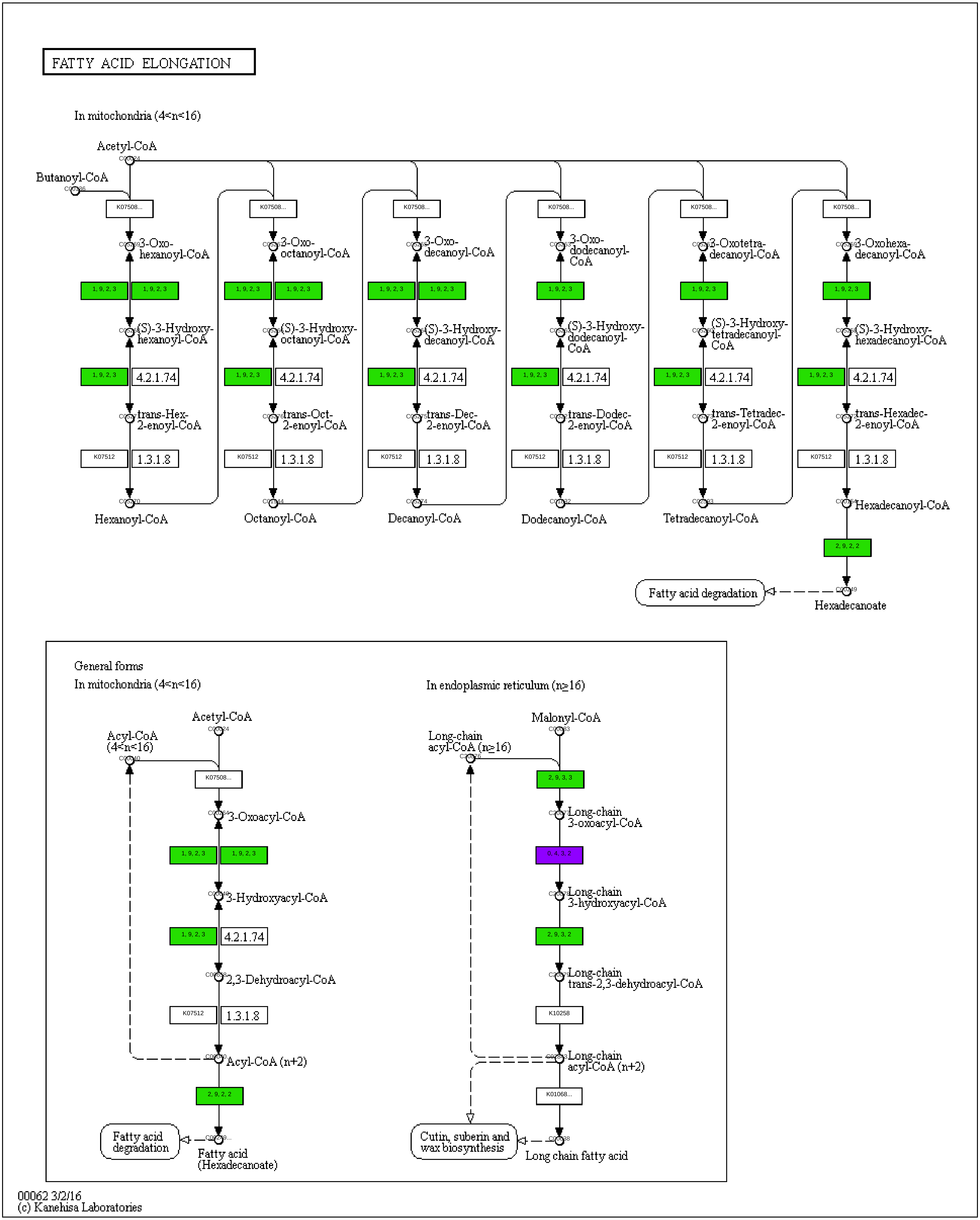
FATTY ACID ELONGATION
In mitochondria (4<n<16)
Acetyl-CoA
Butanoyl-CoA
K07508...
3-Oxo-hexanoyl-CoA
3-Oxo-octanoyl-CoA
3-Oxo-decanoyl-CoA
3-Oxo-dodecanoyl-CoA
3-Oxotetra-decanoyl-CoA
3-Oxohexa-decanoyl-CoA
1, 9, 2, 3
(S)-3-Hydroxy-hexanoyl-CoA
(S)-3-Hydroxy-octanoyl-CoA
(S)-3-Hydroxy-decanoyl-CoA
(S)-3-Hydroxy-dodecanoyl-CoA
(S)-3-Hydroxy-tetradecanoyl-CoA
(S)-3-Hydroxy-hexadecanoyl-CoA
4.2.1.74
trans-Hex-2-enoyl-CoA
trans-Oct-2-enoyl-CoA
trans-Dec-2-enoyl-CoA
trans-Dodec-2-enoyl-CoA
trans-Tetradec-2-enoyl-CoA
trans-Hexadec-2-enoyl-CoA
K07512
1.3.1.8
Hexanoyl-CoA
Octanoyl-CoA
Decanoyl-CoA
Dodecanoyl-CoA
Tetradecanoyl-CoA
Hexadecanoyl-CoA
2, 9, 2, 2
Fatty acid degradation
Hexadecanoate
General forms
In mitochondria (4<n<16)
In endoplasmic reticulum (n≥16)
Acyl-CoA (4<n<16)
3-Oxoacyl-CoA
3-Hydroxyacyl-CoA
2,3-Dehydroacyl-CoA
Acyl-CoA (n+2)
Fatty acid (Hexadecanoate)
Malonyl-CoA
Long-chain acyl-CoA (n≥16)
2, 9, 3, 3
Long-chain 3-oxoacyl-CoA
0, 4, 3, 2
Long-chain 3-hydroxyacyl-CoA
2, 9, 3, 2
Long-chain trans-2,3-dehydroacyl-CoA
K10258
Long-chain acyl-CoA (n+2)
K01068...
Cutin, suberin and wax biosynthesis
Long chain fatty acid
00062 3/2/16
(c) Kanehisa Laboratories

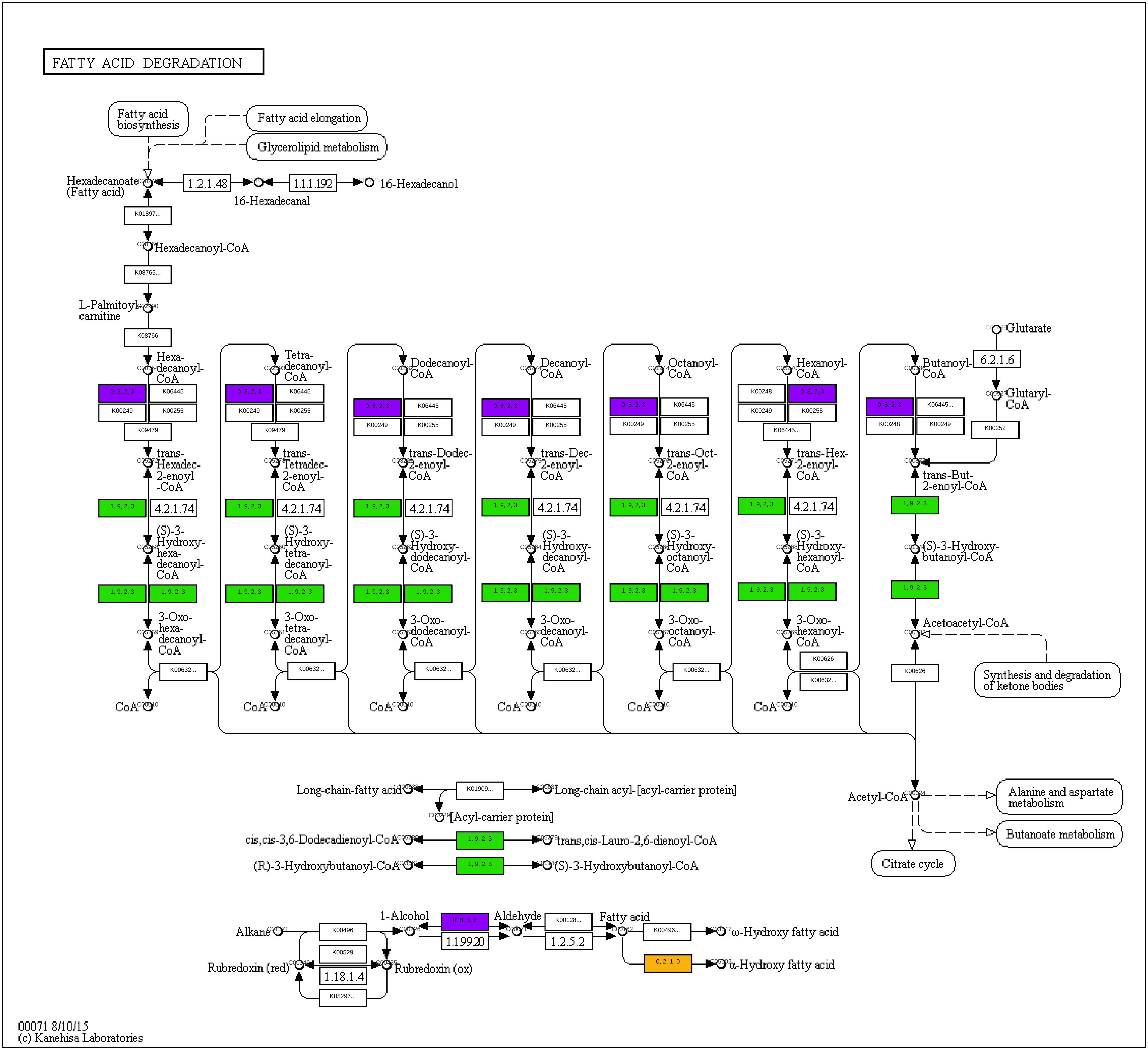
FATTY ACID DEGRADATION
Fatty acid biosynthesis
Fatty acid elongation
Glycerolipid metabolism
Hexadecanoate (Fatty acid)
1.2.1.48
1.1.1.192
16-Hexadecanal
16-Hexadecanol
K01897...
Hexadecanoyl-CoA
K08765...
L-Palmitoyl-carnitine
K08766
Hexa-decanoyl-CoA
Tetra-decanoyl-CoA
Dodecanoyl-CoA
Decanoyl-CoA
Octanoyl-CoA
Hexanoyl-CoA
Butanoyl-CoA
Glutarate
6.2.1.6
Glutaryl-CoA
K00252
0, 8, 2, 3
K06445
K00249
K00255
K09479
K00248
K06445...
trans-Hexadec-2-enoyl-CoA
trans-Tetradec-2-enoyl-CoA
trans-Dodec-2-enoyl-CoA
trans-Dec-2-enoyl-CoA
trans-Oct-2-enoyl-CoA
trans-Hex-2-enoyl-CoA
trans-But-2-enoyl-CoA
1, 9, 2, 3
4.2.1.74
(S)-3-Hydroxy-hexa-decanoyl-CoA
(S)-3-Hydroxy-tetra-decanoyl-CoA
(S)-3-Hydroxy-dodecanoyl-CoA
(S)-3-Hydroxy-decanoyl-CoA
(S)-3-Hydroxy-octanoyl-CoA
(S)-3-Hydroxy-hexanoyl-CoA
(S)-3-Hydroxy-butanoyl-CoA
3-Oxo-hexa-decanoyl-CoA
3-Oxo-tetra-decanoyl-CoA
3-Oxo-dodecanoyl-CoA
3-Oxo-decanoyl-CoA
3-Oxo-octanoyl-CoA
3-Oxo-hexanoyl-CoA
Acetoacetyl-CoA
K00632...
K00626
CoA
Synthesis and degradation of ketone bodies
Acetyl-CoA
Alanine and aspartate metabolism
Butanoate metabolism
Citrate cycle
Long-chain-fatty acid
K01909...
Long-chain acyl-[acyl-carrier protein]
[Acyl-carrier protein]
cis,cis-3,6-Dodecadienoyl-CoA
trans,cis-Lauro-2,6-dienoyl-CoA
(R)-3-Hydroxybutanoyl-CoA
(S)-3-Hydroxybutanoyl-CoA
Alkane
K00496
K00529
1.18.1.4
K05297...
Rubredoxin (red)
Rubredoxin (ox)
1-Alcohol
0, 8, 3, 2
1.1.99.20
Aldehyde
K00128...
1.2.5.2
Fatty acid
K00496...
ω-Hydroxy fatty acid
0, 2, 1, 0
α-Hydroxy fatty acid
00071 8/10/15
(c) Kanehisa Laboratories

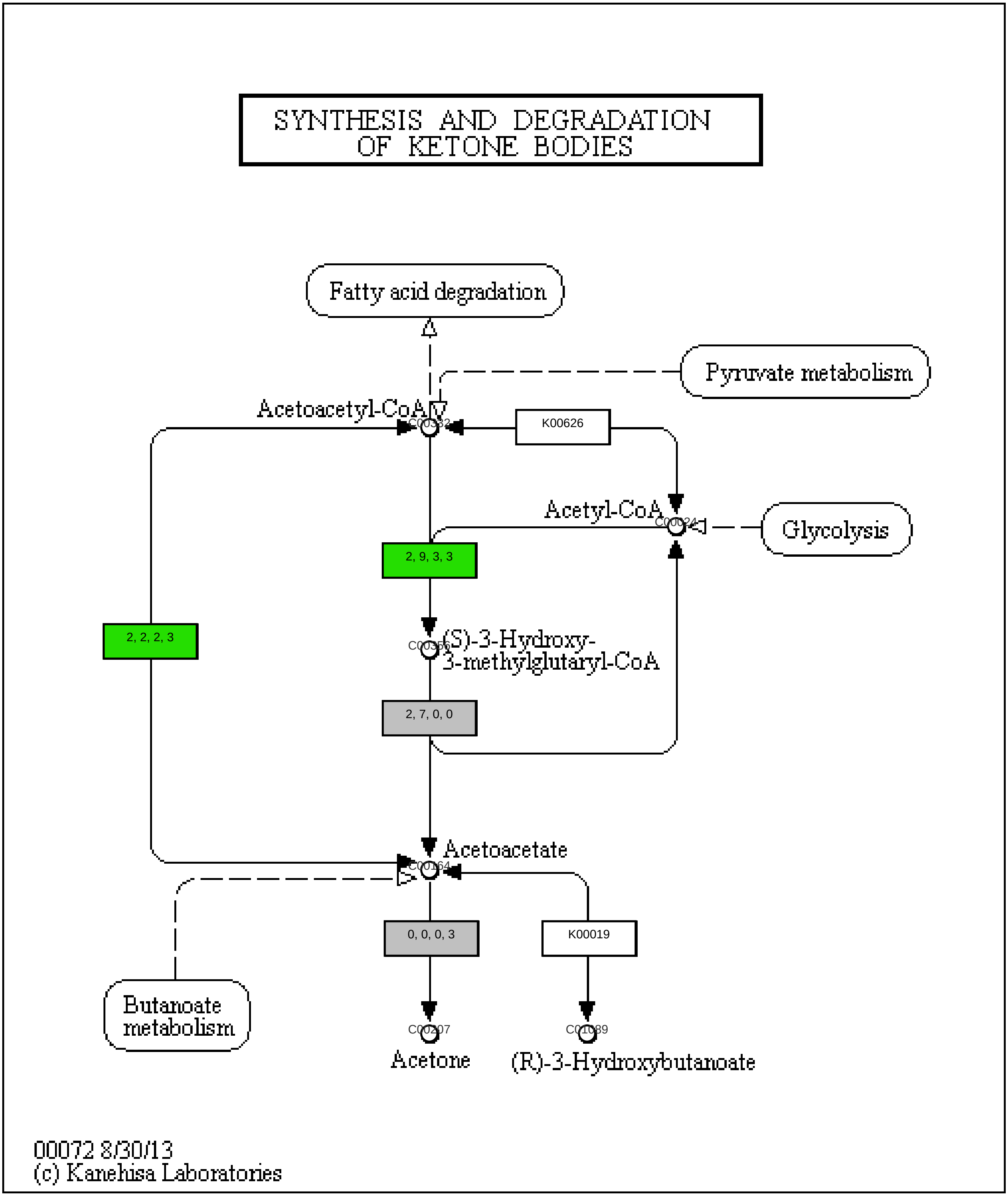
SYNTHESIS AND DEGRADATION
OF KETONE BODIES
Fatty acid degradation
Pyruvate metabolism
Acetoacetyl-CoA
C00332
K00626
Acetyl-CoA
C00024
Glycolysis
2, 9, 3, 3
2, 2, 2, 3
(S)-3-Hydroxy-
3-methylglutaryl-CoA
C00356
2, 7, 0, 0
Acetoacetate
C00164
0, 0, 0, 3
K00019
Butanoate
metabolism
C00207
Acetone
C01089
(R)-3-Hydroxybutanoate
00072 8/30/13
(c) Kanehisa Laboratories

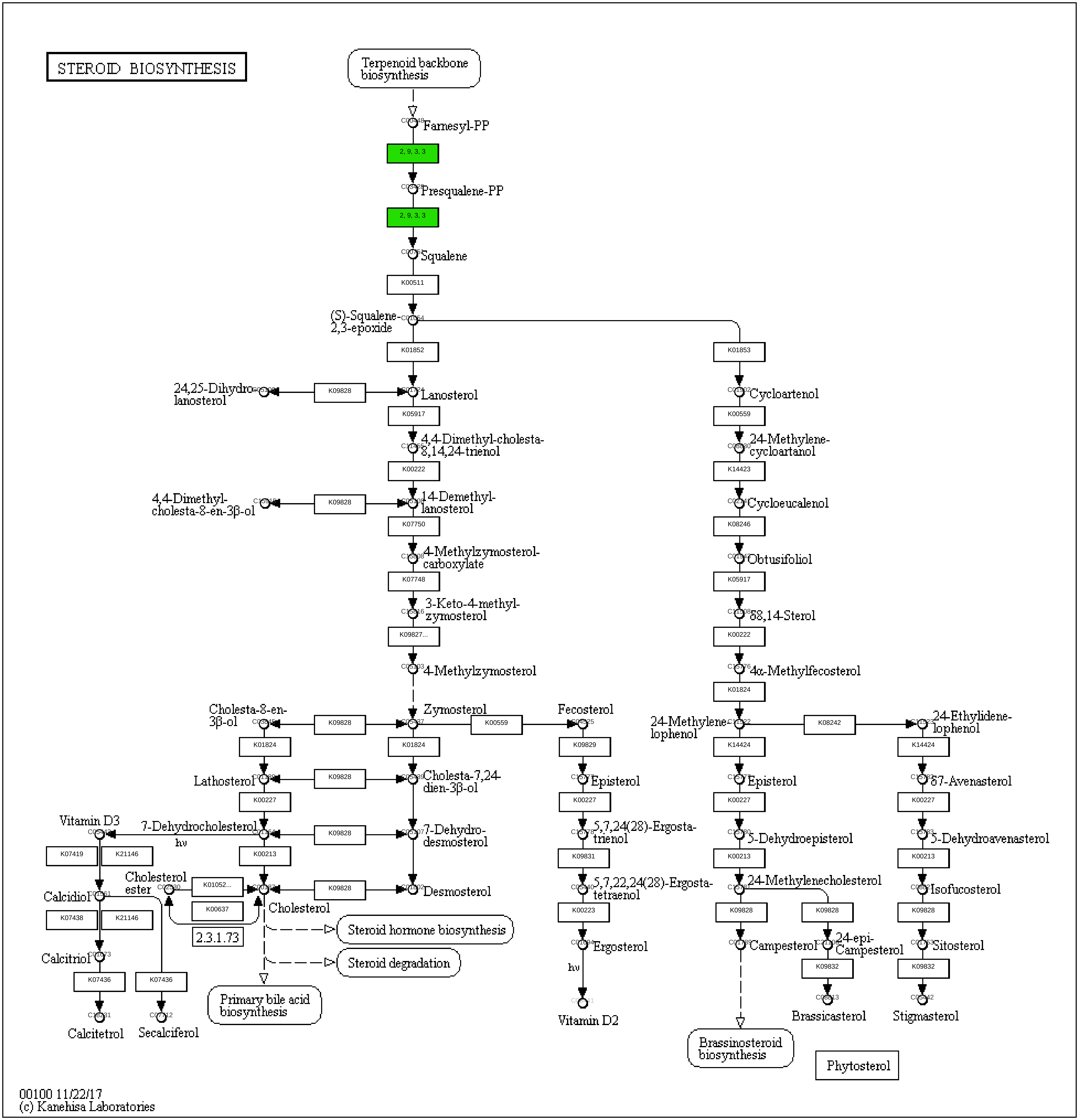

STEROID BIOSYNTHESIS
Terpenoid backbone biosynthesis
Farnesyl-PP
2, 9, 3, 3
Presqualene-PP
2, 9, 3, 3
Squalene
K00511
(S)-Squalene-2,3-epoxide
K01852
K01853
24,25-Dihydro-lanosterol
K09828
Lanosterol
Cycloartenol
K05917
K00559
4,4-Dimethyl-cholesta-8,14,24-trienol
24-Methylene-cycloartanol
K00222
K14423
4,4-Dimethyl-cholesta-8-en-3β-ol
K09828
14-Demethyl-lanosterol
Cycloeucalenol
K07750
K08246
4-Methylzymosterol-carboxylate
Obtusifoliol
K07748
K05917
3-Keto-4-methyl-zymosterol
δ8,14-Sterol
K09827...
K00222
4-Methylzymosterol
4α-Methylfecosterol
K01824
Cholesta-8-en-3β-ol
K09828
Zymosterol
K00559
Fecosterol
24-Methylene-lophenol
K08242
24-Ethylidene-lophenol
K01824
K01824
K09829
K14424
K14424
Lathosterol
K09828
Cholesta-7,24-dien-3β-ol
Episterol
Episterol
δ7-Avenasterol
K00227
K00227
K00227
K00227
Vitamin D3
7-Dehydrocholesterol
K09828
7-Dehydro-desmosterol
5,7,24(28)-Ergosta-trienol
5-Dehydroepisterol
5-Dehydroavenasterol
hν
K07419
K21146
K00213
K09831
K00213
K00213
Cholesterol ester
K01052...
K09828
Desmosterol
5,7,22,24(28)-Ergosta-tetraenol
24-Methylenecholesterol
Isofucosterol
Calcidiol
K00637
Cholesterol
K00223
K09828
K09828
K09828
K07438
K21146
2.3.1.73
Steroid hormone biosynthesis
Calcitriol
Steroid degradation
Ergosterol
Campesterol
24-epi-Campesterol
Sitosterol
hν
K09832
K09832
K07436
K07436
Primary bile acid biosynthesis
Calcitetrol
Secalciferol
Vitamin D2
Brassicasterol
Stigmasterol
Brassinosteroid biosynthesis
Phytosterol
00100 11/22/17
(c) Kanehisa Laboratories

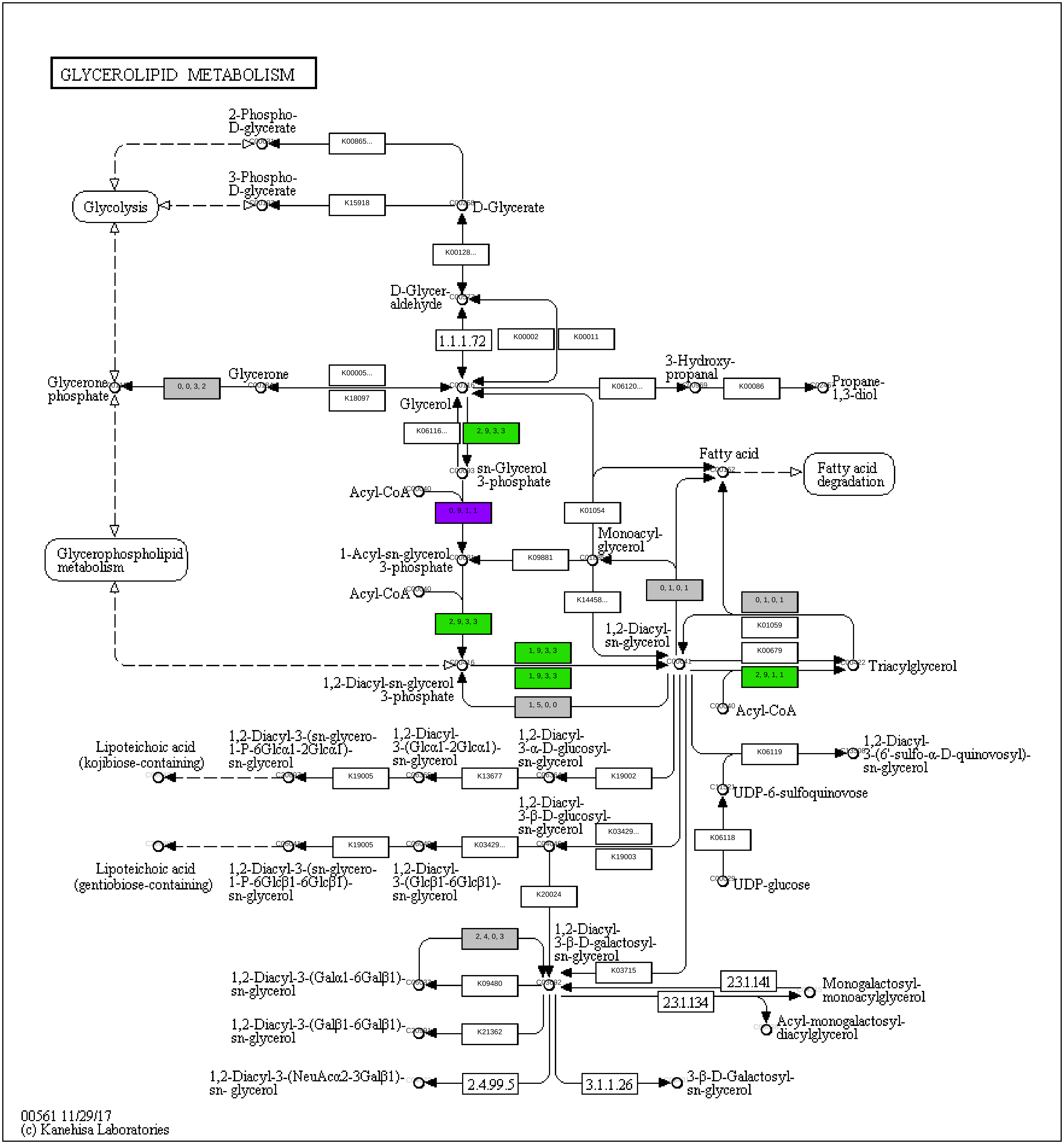
GLYCEROLIPID METABOLISM
2-Phospho-D-glycerate
K00865...
3-Phospho-D-glycerate
K15918
Glycolysis
D-Glycerate
K00128...
D-Glycer-aldehyde
1.1.1.72
K00002
K00011
Glycerone phosphate
0, 0, 3, 2
Glycerone
K00005...
K18097
Glycerol
K06120...
3-Hydroxy-propanal
K00086
Propane-1,3-diol
K06116...
2, 9, 3, 3
sn-Glycerol 3-phosphate
Fatty acid
Fatty acid degradation
Acyl-CoA
0, 9, 1, 1
K01054
Glycerophospholipid metabolism
Monoacyl-glycerol
1-Acyl-sn-glycerol 3-phosphate
K09881
0, 1, 0, 1
K14458...
0, 1, 0, 1
K01059
Acyl-CoA
2, 9, 3, 3
1,2-Diacyl-sn-glycerol
1, 9, 3, 3
K00679
Triacylglycerol
1, 9, 3, 3
2, 9, 1, 1
1,2-Diacyl-sn-glycerol 3-phosphate
1, 5, 0, 0
Acyl-CoA
Lipoteichoic acid (kojibiose-containing)
1,2-Diacyl-3-(sn-glycero-1-P-6Glcα1-2Glcα1)-sn-glycerol
1,2-Diacyl-3-(Glcα1-2Glcα1)-sn-glycerol
1,2-Diacyl-3-α-D-glucosyl-sn-glycerol
K19005
K13677
K19002
K06119
1,2-Diacyl-3-(6'-sulfo-α-D-quinovosyl)-sn-glycerol
UDP-6-sulfoquinovose
1,2-Diacyl-3-β-D-glucosyl-sn-glycerol
K19005
K03429...
K03429...
K19003
K06118
Lipoteichoic acid (gentiobiose-containing)
1,2-Diacyl-3-(sn-glycero-1-P-6Glcβ1-6Glcβ1)-sn-glycerol
1,2-Diacyl-3-(Glcβ1-6Glcβ1)-sn-glycerol
K20024
UDP-glucose
2, 4, 0, 3
1,2-Diacyl-3-β-D-galactosyl-sn-glycerol
K03715
1,2-Diacyl-3-(Galα1-6Galβ1)-sn-glycerol
K09480
2.3.1.141
2.3.1.134
Monogalactosyl-monoacylglycerol
Acyl-monogalactosyl-diacylglycerol
1,2-Diacyl-3-(Galβ1-6Galβ1)-sn-glycerol
K21362
1,2-Diacyl-3-(NeuAcα2-3Galβ1)-sn- glycerol
2.4.99.5
3.1.1.26
3-β-D-Galactosyl-sn-glycerol
00561 11/29/17
(c) Kanehisa Laboratories

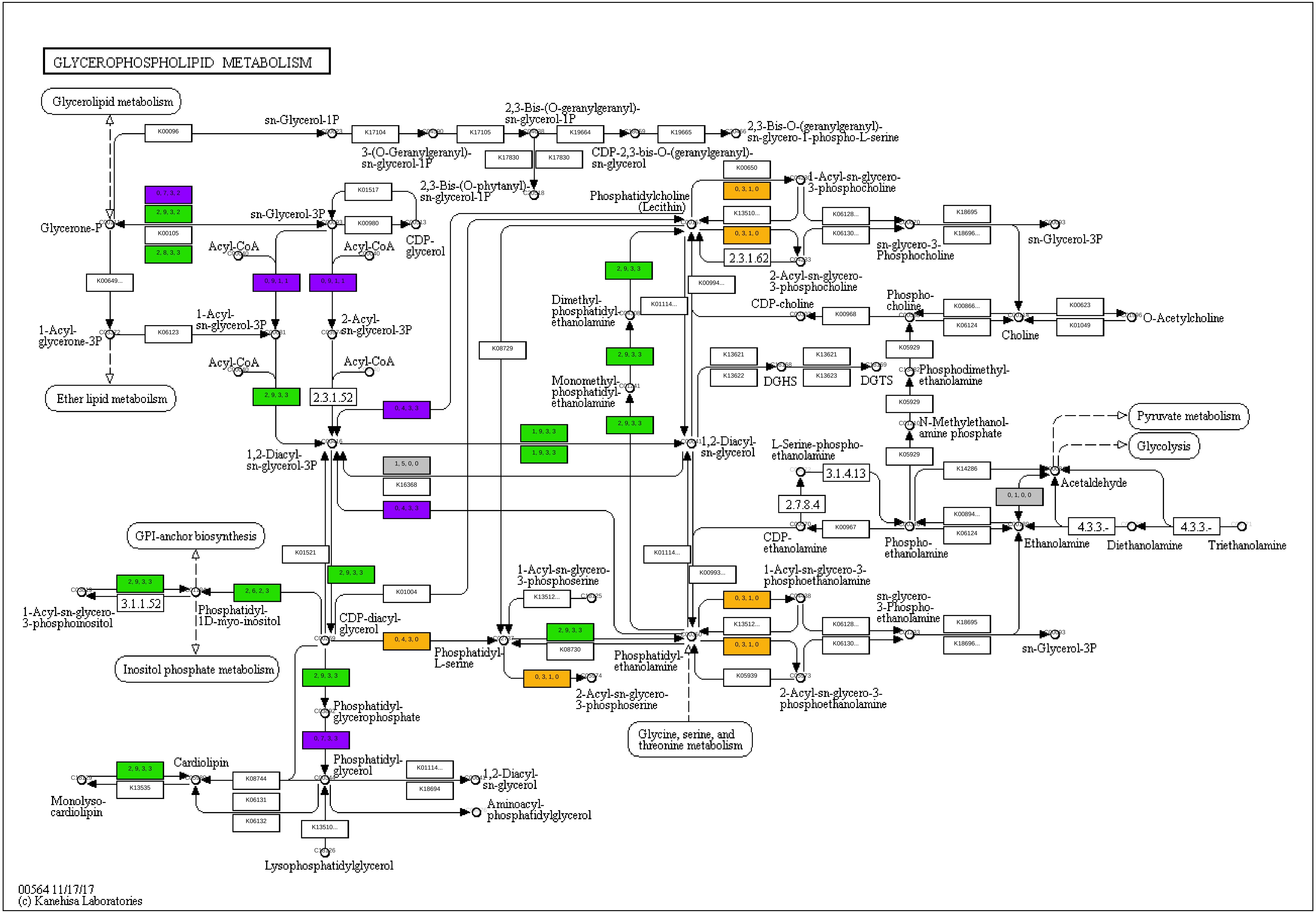
GLYCEROPHOSPHOLIPID METABOLISM
Glycerolipid metabolism
Ether lipid metaboilsm
GPI-anchor biosynthesis
Inositol phosphate metabolism
Glycine, serine, and threonine metabolism
Pyruvate metabolism
Glycolysis
Glycerone-P
1-Acyl-glycerone-3P
sn-Glycerol-1P
3-(O-Geranylgeranyl)-sn-glycerol-1P
2,3-Bis-(O-geranylgeranyl)-sn-glycerol-1P
CDP-2,3-bis-O-(geranylgeranyl)-sn-glycerol
2,3-Bis-O-(geranylgeranyl)-sn-glycero-1-phospho-L-serine
2,3-Bis-(O-phytanyl)-sn-glycerol-1P
sn-Glycerol-3P
CDP-glycerol
Acyl-CoA
1-Acyl-sn-glycerol-3P
2-Acyl-sn-glycerol-3P
1,2-Diacyl-sn-glycerol-3P
CDP-diacyl-glycerol
Phosphatidyl-1D-myo-inositol
1-Acyl-sn-glycero-3-phosphoinositol
Phosphatidyl-glycerophosphate
Phosphatidyl-glycerol
Cardiolipin
Monolyso-cardiolipin
Lysophosphatidylglycerol
1,2-Diacyl-sn-glycerol
Aminoacyl-phosphatidylglycerol
Phosphatidyl-L-serine
1-Acyl-sn-glycero-3-phosphoserine
2-Acyl-sn-glycero-3-phosphoserine
Phosphatidyl-ethanolamine
Dimethyl-phosphatidyl-ethanolamine
Monomethyl-phosphatidyl-ethanolamine
Phosphatidylcholine (Lecithin)
1-Acyl-sn-glycero-3-phosphocholine
2-Acyl-sn-glycero-3-phosphocholine
sn-glycero-3-Phosphocholine
CDP-choline
Phospho-choline
Choline
O-Acetylcholine
DGHS
DGTS
Phosphodimethyl-ethanolamine
N-Methylethanol-amine phosphate
L-Serine-phospho-ethanolamine
Acetaldehyde
CDP-ethanolamine
Phospho-ethanolamine
Ethanolamine
Diethanolamine
Triethanolamine
1-Acyl-sn-glycero-3-phosphoethanolamine
sn-glycero-3-Phospho-ethanolamine
2-Acyl-sn-glycero-3-phosphoethanolamine
K00096
K17104
K17105
K19664
K19665
K17830
K01517
K00980
K00105
K00649...
K06123
K00650
K13510...
K06128...
K06130...
K18695
K18696...
K00994...
K01114...
K00968
K00866...
K06124
K00623
K01049
K08729
K13621
K13622
K13623
K05929
K14286
K00894...
K00967
K16368
K01521
K01004
K13512...
K08730
K00993...
K05939
K08744
K06131
K06132
K13535
K18694
2.3.1.62
2.3.1.52
3.1.4.13
2.7.8.4
4.3.3.-
3.1.1.52
0, 7, 3, 2
2, 9, 3, 2
2, 8, 3, 3
0, 9, 1, 1
2, 9, 3, 3
0, 4, 3, 3
1, 5, 0, 0
1, 9, 3, 3
0, 3, 1, 0
0, 1, 0, 0
2, 6, 2, 3
0, 4, 3, 0
0, 7, 3, 3
00564 11/17/17
(c) Kanehisa Laboratories

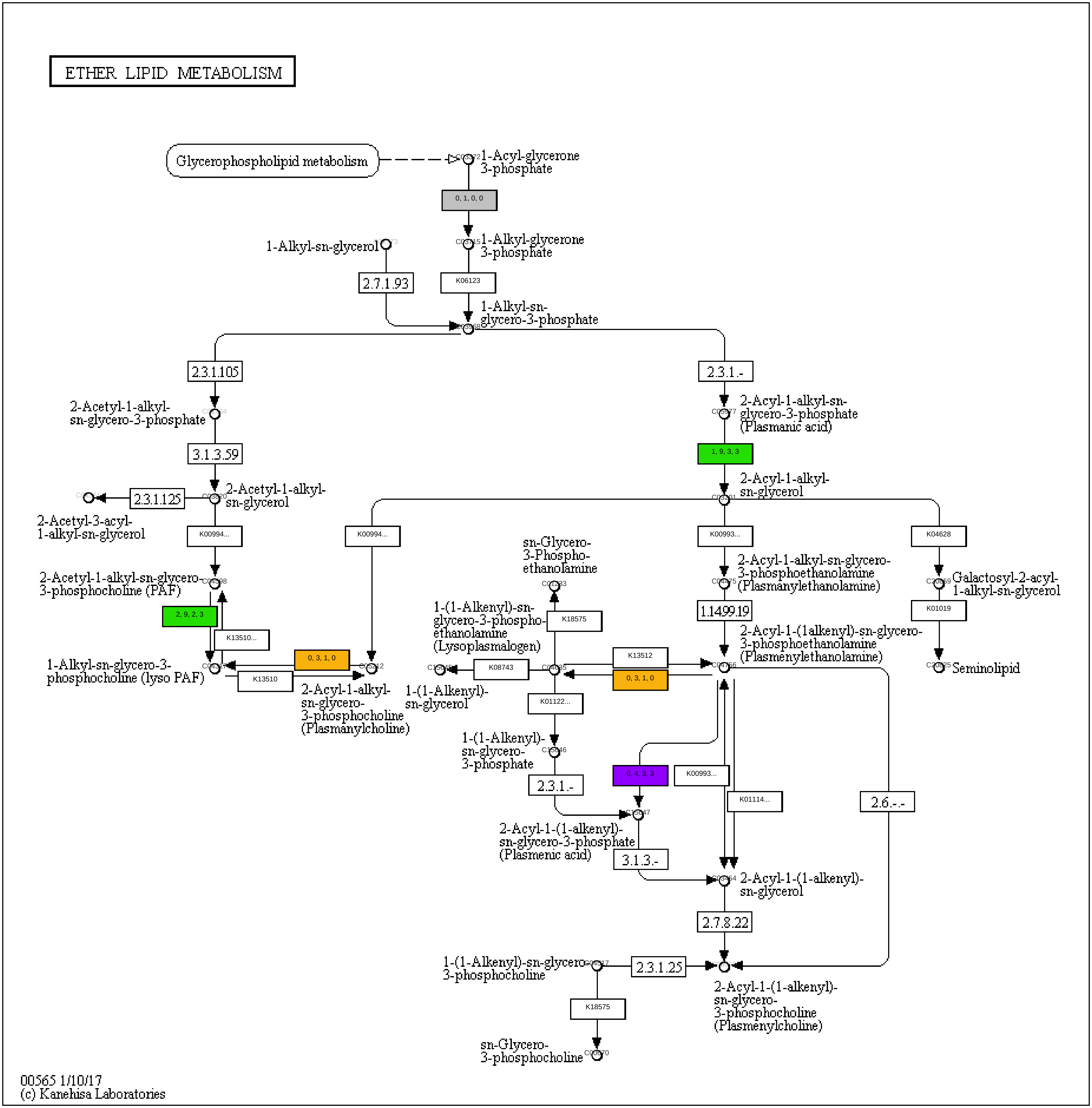
ETHER LIPID METABOLISM
Glycerophospholipid metabolism
1-Acyl-glycerone 3-phosphate
0, 1, 0, 0
1-Alkyl-sn-glycerol
1-Alkyl-glycerone 3-phosphate
2.7.1.93
K06123
1-Alkyl-sn-glycero-3-phosphate
2.3.1.105
2.3.1.-
2-Acetyl-1-alkyl-sn-glycero-3-phosphate
2-Acyl-1-alkyl-sn-glycero-3-phosphate (Plasmanic acid)
3.1.3.59
1, 9, 3, 3
2.3.1.125
2-Acetyl-1-alkyl-sn-glycerol
2-Acyl-1-alkyl-sn-glycerol
2-Acetyl-3-acyl-1-alkyl-sn-glycerol
K00994...
K00994...
K00993...
K04628
sn-Glycero-3-Phospho-ethanolamine
2-Acetyl-1-alkyl-sn-glycero-3-phosphocholine (PAF)
2-Acyl-1-alkyl-sn-glycero-3-phosphoethanolamine (Plasmanylethanolamine)
Galactosyl-2-acyl-1-alkyl-sn-glycerol
1-(1-Alkenyl)-sn-glycero-3-phospho-ethanolamine (Lysoplasmalogen)
K18575
1.14.99.19
K01019
2, 9, 2, 3
K13510...
2-Acyl-1-(1alkenyl)-sn-glycero-3-phosphoethanolamine (Plasmenylethanolamine)
K13512
0, 3, 1, 0
K08743
0, 3, 1, 0
Seminolipid
1-Alkyl-sn-glycero-3-phosphocholine (lyso PAF)
K13510
2-Acyl-1-alkyl-sn-glycero-3-phosphocholine (Plasmanylcholine)
1-(1-Alkenyl)-sn-glycerol
K01122...
1-(1-Alkenyl)-sn-glycero-3-phosphate
2.3.1.-
0, 4, 3, 3
K00993...
K01114...
2.6.-.-
2-Acyl-1-(1-alkenyl)-sn-glycero-3-phosphate (Plasmenic acid)
3.1.3.-
2-Acyl-1-(1-alkenyl)-sn-glycerol
2.7.8.22
1-(1-Alkenyl)-sn-glycero-3-phosphocholine
2.3.1.25
2-Acyl-1-(1-alkenyl)-sn-glycero-3-phosphocholine (Plasmenylcholine)
K18575
sn-Glycero-3-phosphocholine
00565 1/10/17
(c) Kanehisa Laboratories

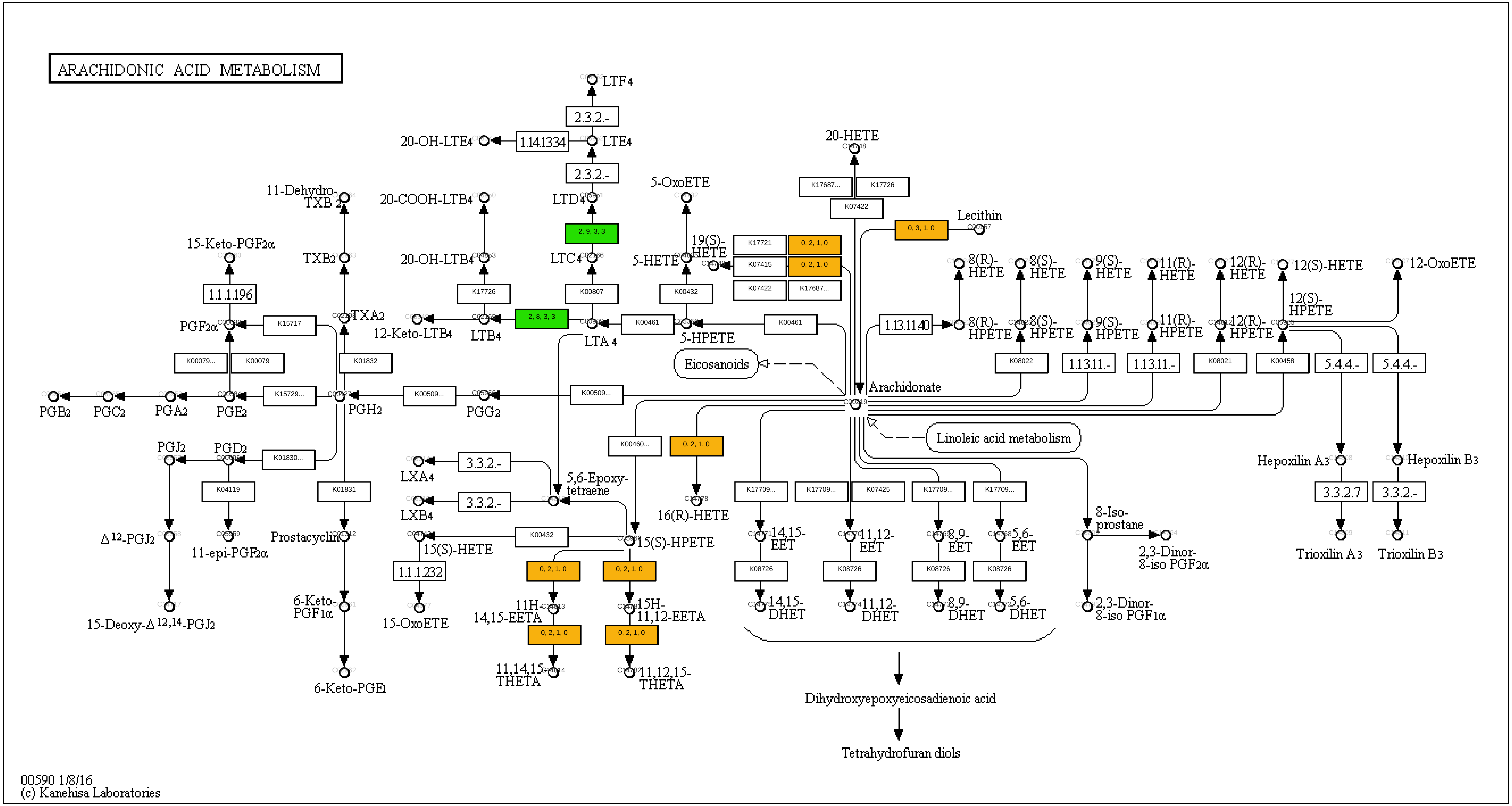
ARACHIDONIC ACID METABOLISM
LTF4
2.3.2.-
20-OH-LTE4
1.14.13.34
LTE4
2.3.2.-
11-Dehydro-TXB 2
20-COOH-LTB4
LTD4
5-OxoETE
20-HETE
K17687...
K17726
K07422
Lecithin
0, 3, 1, 0
15-Keto-PGF2α
2, 9, 3, 3
19(S)-HETE
K17721
0, 2, 1, 0
K07415
0, 2, 1, 0
K07422
K17687...
TXB2
20-OH-LTB4
LTC4
5-HETE
8(R)-HETE
8(S)-HETE
9(S)-HETE
11(R)-HETE
12(R)-HETE
12(S)-HETE
12-OxoETE
1.1.1.196
K17726
K00807
K00432
12(S)-HPETE
TXA2
2, 8, 3, 3
PGF2α
K15717
12-Keto-LTB4
LTB4
LTA 4
K00461
5-HPETE
K00461
1.13.11.40
8(R)-HPETE
8(S)-HPETE
9(S)-HPETE
11(R)-HPETE
12(R)-HPETE
K00079...
K00079
K01832
Eicosanoids
K08022
1.13.11.-
1.13.11.-
K08021
K00458
5.4.4.-
5.4.4.-
Arachidonate
PGB2
PGC2
PGA2
PGE2
K15729...
PGH2
K00509...
PGG2
K00509...
K00460...
0, 2, 1, 0
Linoleic acid metabolism
PGJ2
PGD2
K01830...
LXA4
3.3.2.-
5,6-Epoxy-tetraene
Hepoxilin A3
Hepoxilin B3
K04119
K01831
LXB4
3.3.2.-
16(R)-HETE
K17709...
K17709...
K07425
K17709...
K17709...
8-Iso-prostane
3.3.2.7
3.3.2.-
Δ12-PGJ2
11-epi-PGF2α
Prostacyclin
K00432
15(S)-HPETE
15(S)-HETE
14,15-EET
11,12-EET
8,9-EET
5,6-EET
2,3-Dinor-8-iso PGF2α
Trioxilin A3
Trioxilin B3
1.1.1.232
0, 2, 1, 0
0, 2, 1, 0
K08726
K08726
K08726
K08726
6-Keto-PGF1α
11H-14,15-EETA
15H-11,12-EETA
14,15-DHET
11,12-DHET
8,9-DHET
5,6-DHET
2,3-Dinor-8-iso PGF1α
15-Deoxy-Δ12,14-PGJ2
15-OxoETE
0, 2, 1, 0
0, 2, 1, 0
11,14,15-THETA
11,12,15-THETA
6-Keto-PGE1
Dihydroxyepoxyeicosadienoic acid
Tetrahydrofuran diols
00590 1/8/16
(c) Kanehisa Laboratories

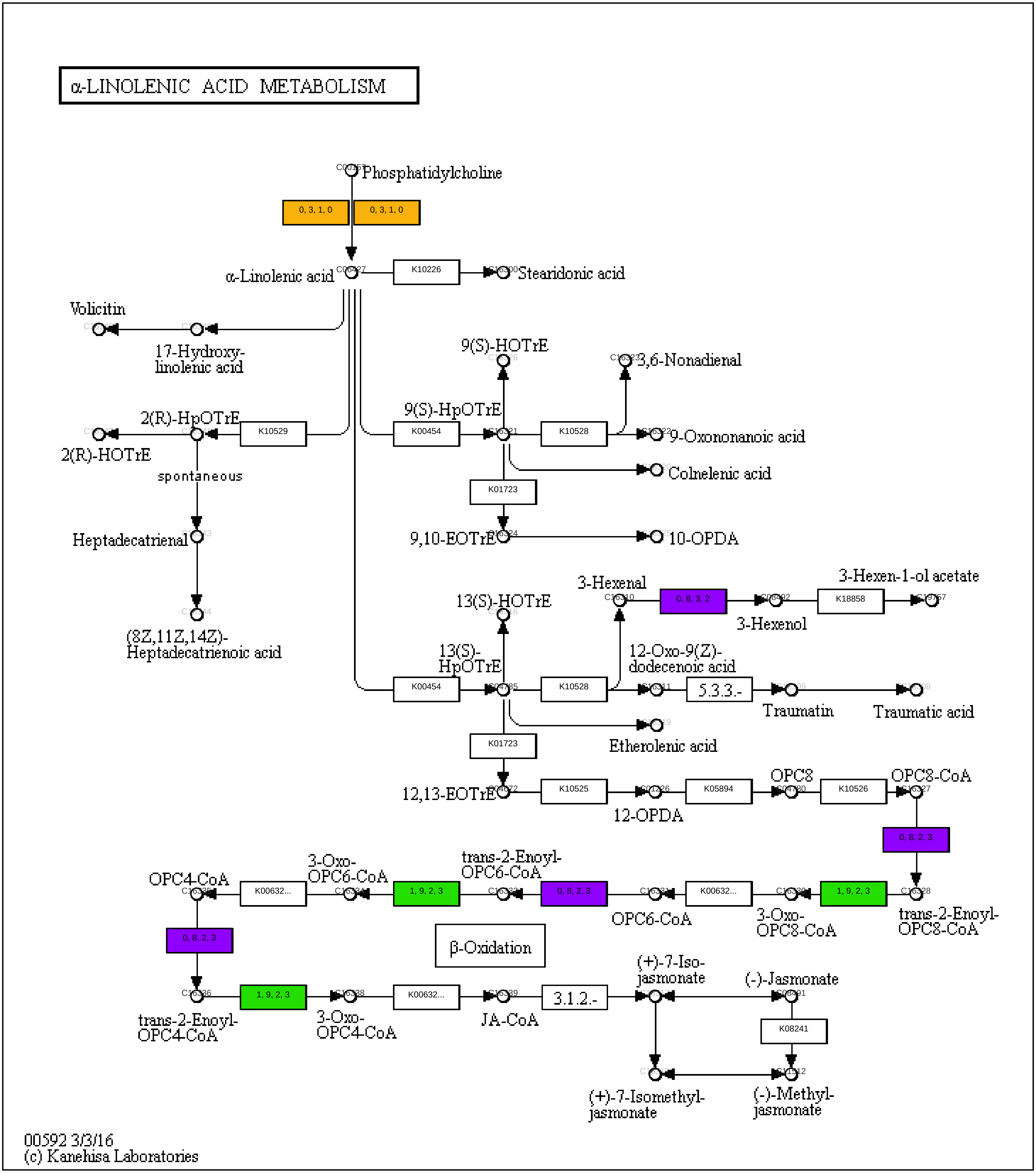
α-LINOLENIC ACID METABOLISM
Phosphatidylcholine
0, 3, 1, 0
0, 3, 1, 0
α-Linolenic acid
K10226
Stearidonic acid
Volicitin
17-Hydroxy-linolenic acid
9(S)-HOTrE
3,6-Nonadienal
2(R)-HpOTrE
K10529
2(R)-HOTrE
9(S)-HpOTrE
K00454
K10528
9-Oxononanoic acid
Colnelenic acid
spontaneous
K01723
Heptadecatrienal
9,10-EOTrE
10-OPDA
(8Z,11Z,14Z)-Heptadecatrienoic acid
3-Hexenal
0, 8, 3, 2
K18858
3-Hexen-1-ol acetate
3-Hexenol
13(S)-HOTrE
13(S)-HpOTrE
12-Oxo-9(Z)-dodecenoic acid
K00454
K10528
5.3.3.-
Traumatin
Traumatic acid
Etherolenic acid
K01723
12,13-EOTrE
K10525
12-OPDA
K05894
OPC8
K10526
OPC8-CoA
0, 8, 2, 3
OPC4-CoA
K00632...
3-Oxo-OPC6-CoA
1, 9, 2, 3
trans-2-Enoyl-OPC6-CoA
0, 8, 2, 3
K00632...
OPC6-CoA
3-Oxo-OPC8-CoA
1, 9, 2, 3
trans-2-Enoyl-OPC8-CoA
β-Oxidation
0, 8, 2, 3
1, 9, 2, 3
K00632...
3.1.2.-
(+)-7-Iso-jasmonate
(-)-Jasmonate
trans-2-Enoyl-OPC4-CoA
3-Oxo-OPC4-CoA
JA-CoA
K08241
(+)-7-Isomethyl-jasmonate
(-)-Methyl-jasmonate
00592 3/3/16
(c) Kanehisa Laboratories

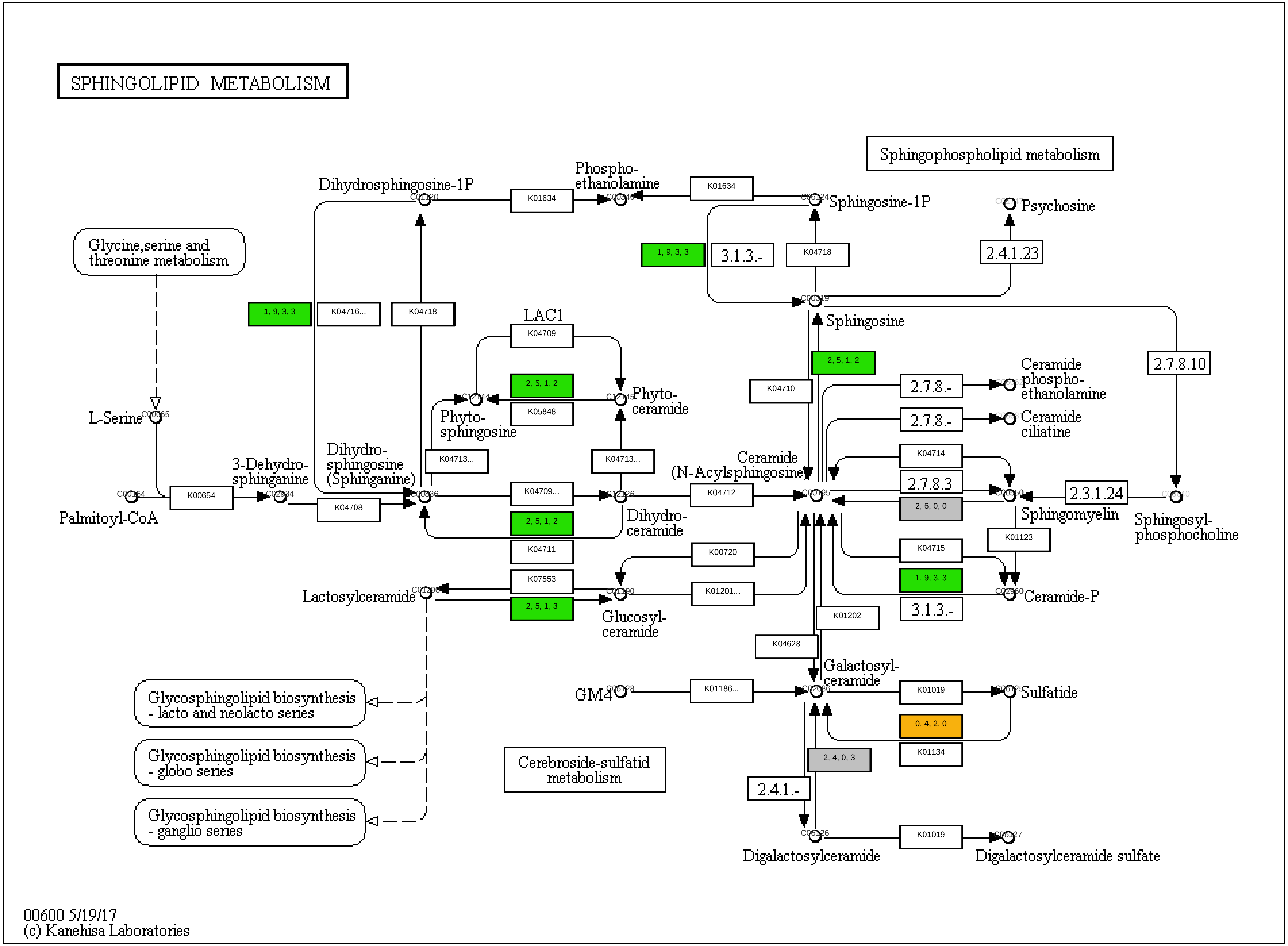
SPHINGOLIPID METABOLISM
Sphingophospholipid metabolism
Glycine,serine and threonine metabolism
L-Serine
C00065
Palmitoyl-CoA
C00154
K00654
3-Dehydro-sphinganine
C02934
K04708
Dihydro-sphingosine (Sphinganine)
C00836
Dihydrosphingosine-1P
C01120
K01634
Phospho-ethanolamine
C00346
K01634
Sphingosine-1P
C06124
1, 9, 3, 3
K04716...
K04718
1, 9, 3, 3
3.1.3.-
K04718
Psychosine
2.4.1.23
Sphingosine
C00319
LAC1
K04709
2, 5, 1, 2
K05848
Phyto-sphingosine
C12144
Phyto-ceramide
C12145
K04713...
K04713...
K04709...
2, 5, 1, 2
K04711
Dihydro-ceramide
C12126
K04712
Ceramide (N-Acylsphingosine)
C00195
2, 5, 1, 2
K04710
2.7.8.-
Ceramide phospho-ethanolamine
2.7.8.-
Ceramide ciliatine
2.7.8.10
K04714
2.7.8.3
2, 6, 0, 0
2.3.1.24
Sphingomyelin
C00550
Sphingosyl-phosphocholine
K01123
K04715
1, 9, 3, 3
Ceramide-P
C02960
3.1.3.-
K00720
K07553
Lactosylceramide
C01290
2, 5, 1, 3
K01201...
Glucosyl-ceramide
C01190
K01202
K04628
Galactosyl-ceramide
C02686
GM4
C05128
K01186...
K01019
Sulfatide
C06125
0, 4, 2, 0
K01134
2, 4, 0, 3
2.4.1.-
Glycosphingolipid biosynthesis - lacto and neolacto series
Glycosphingolipid biosynthesis - globo series
Glycosphingolipid biosynthesis - ganglio series
Cerebroside-sulfatid metabolism
Digalactosylceramide
C06126
K01019
Digalactosylceramide sulfate
C06127
00600 5/19/17
(c) Kanehisa Laboratories

4. Nucleotide metabolism

MAP	PATHWAY
**230**	Purine metabolism
**240**	Pyrimidine metabolism

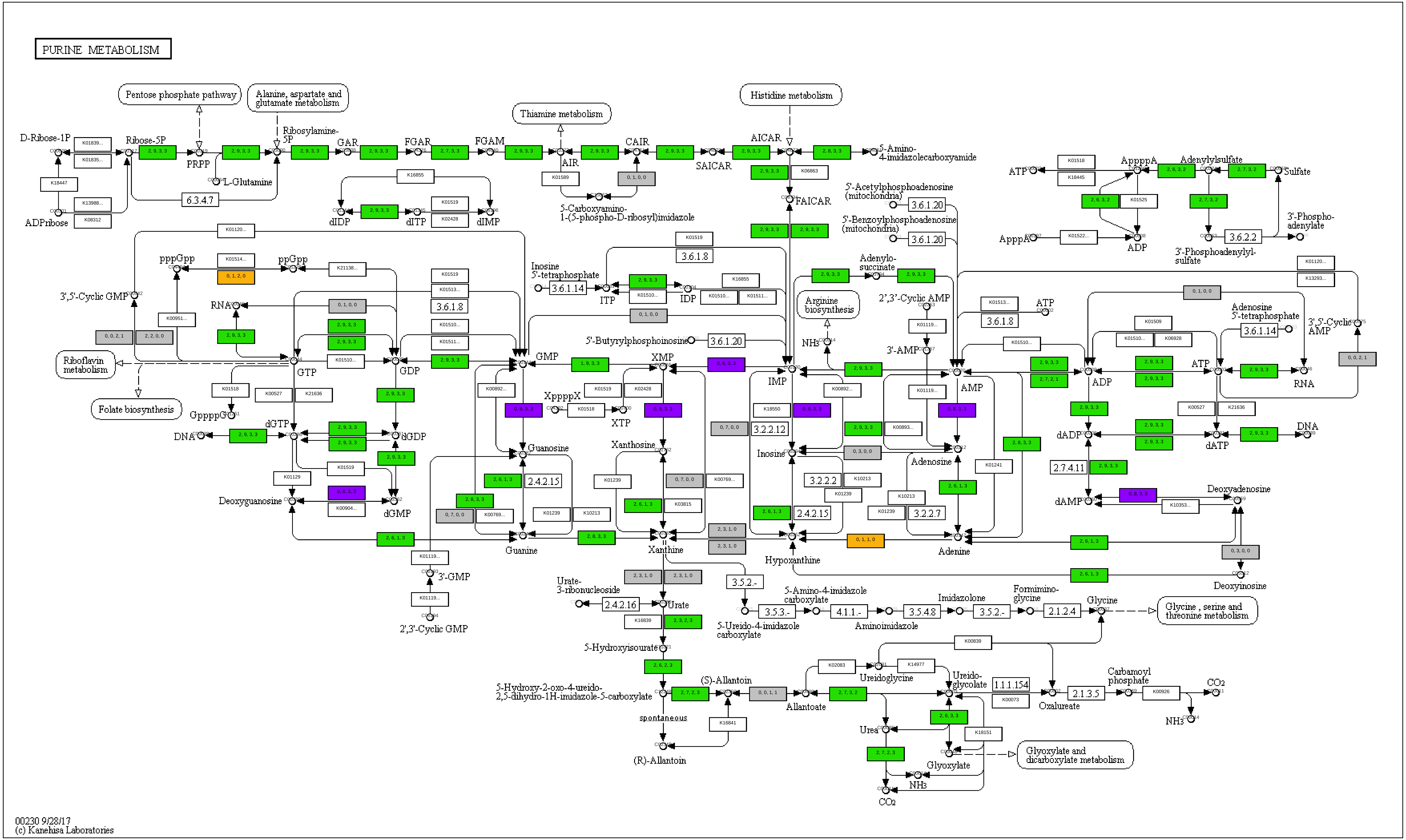
PURINE METABOLISM
Pentose phosphate pathway
Alanine, aspartate and glutamate metabolism
Thiamine metabolism
Histidine metabolism
D-Ribose-1P
Ribose-5P
PRPP
L-Glutamine
Ribosylamine-5P
GAR
FGAR
FGAM
AIR
CAIR
SAICAR
AICAR
5-Amino-4-imidazolecarboxyamide
FAICAR
ADPribose
6.3.4.7
5-Carboxyamino-1-(5-phospho-D-ribosyl)imidazole
dIDP
dITP
dIMP
ATP
ApppA
Adenylylsulfate
Sulfate
ADP
AppppA
3'-Phospho-adenylate
3'-Phosphoadenylyl-sulfate
3.6.2.2
5'-Acetylphosphoadenosine (mitochondria)
3.6.1.20
5'-Benzoylphosphoadenosine (mitochondria)
pppGpp
ppGpp
3',5'-Cyclic GMP
RNA
Riboflavin metabolism
Folate biosynthesis
GTP
GDP
GMP
GppppG
dGTP
dGDP
DNA
dGMP
Deoxyguanosine
3.6.1.8
Inosine 5'-tetraphosphate
3.6.1.14
ITP
IDP
3.6.1.8
Adenylo-succinate
Arginine biosynthesis
2',3'-Cyclic AMP
NH3
5'-Butyrylphosphoinosine
3.6.1.20
XMP
XppppX
XTP
Guanosine
Xanthosine
2.4.2.15
IMP
3.2.2.12
Inosine
3.2.2.2
2.4.2.15
3'-AMP
AMP
3.6.1.8
Adenosine
3.2.2.7
Adenosine 5'-tetraphosphate
3.6.1.14
3',5'-Cyclic AMP
ADP
ATP
RNA
dADP
dATP
DNA
2.7.4.11
dAMP
Deoxyadenosine
Guanine
Xanthine
Hypoxanthine
Adenine
Deoxyinosine
3'-GMP
2',3'-Cyclic GMP
Urate-3-ribonucleoside
2.4.2.16
Urate
3.5.2.-
5-Amino-4-imidazole carboxylate
3.5.3.-
4.1.1.-
Aminoimidazole
3.5.4.8
Imidazolone
3.5.2.-
Formimino-glycine
2.1.2.4
Glycine
Glycine, serine and threonine metabolism
5-Ureido-4-imidazole carboxylate
5-Hydroxyisourate
5-Hydroxy-2-oxo-4-ureido-2,5-dihydro-1H-imidazole-5-carboxylate
(S)-Allantoin
spontaneous
(R)-Allantoin
Allantoate
Ureidoglycine
Ureido-glycolate
1.1.1.154
Oxalureate
2.1.3.5
Carbamoyl phosphate
CO2
NH3
Urea
Glyoxylate
Glyoxylate and dicarboxylate metabolism
CO2
NH3
00230 9/28/17
(c) Kanehisa Laboratories

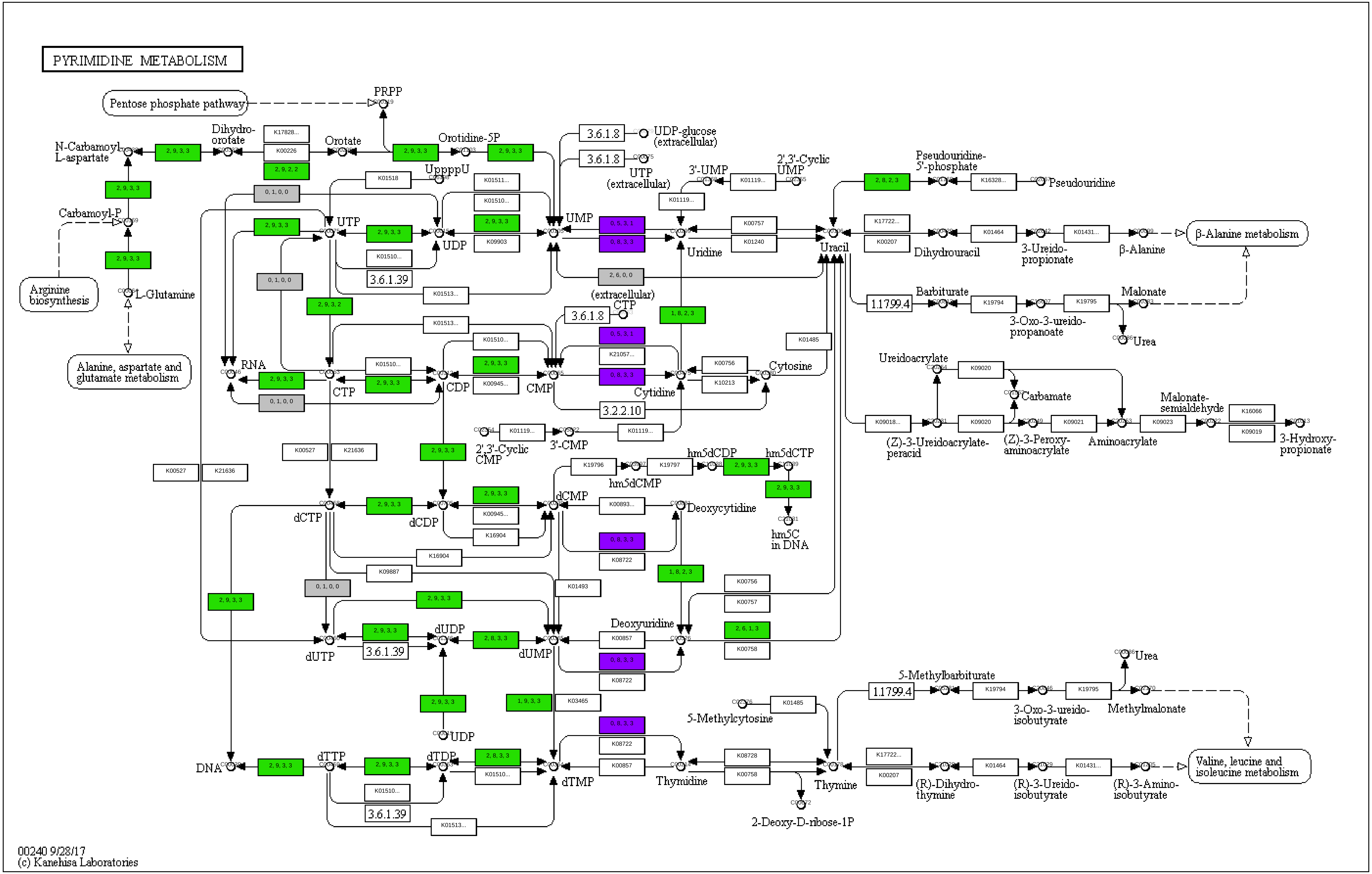
PYRIMIDINE METABOLISM
Pentose phosphate pathway
PRPP
N-Carbamoyl-L-aspartate
Dihydro-orotate
Orotate
Orotidine-5P
UppppU
UDP-glucose (extracellular)
UTP (extracellular)
3'-UMP
2',3'-Cyclic UMP
Pseudouridine-5'-phosphate
Pseudouridine
Carbamoyl-P
L-Glutamine
Arginine biosynthesis
Alanine, aspartate and glutamate metabolism
UTP
UDP
UMP
Uridine
Uracil
Dihydrouracil
3-Ureido-propionate
β-Alanine
β-Alanine metabolism
Barbiturate
3-Oxo-3-ureido-propanoate
Malonate
Urea
(extracellular)
CTP
RNA
CTP
CDP
CMP
Cytidine
Cytosine
Ureidoacrylate
Carbamate
Malonate-semialdehyde
(Z)-3-Ureidoacrylate-peracid
(Z)-3-Peroxy-aminoacrylate
Aminoacrylate
3-Hydroxy-propionate
2',3'-Cyclic CMP
3'-CMP
hm5dCDP
hm5dCTP
hm5dCMP
hm5C in DNA
dCTP
dCDP
dCMP
Deoxycytidine
Deoxyuridine
dUTP
dUDP
dUMP
UDP
5-Methylbarbiturate
3-Oxo-3-ureido-isobutyrate
Methylmalonate
Urea
5-Methylcytosine
DNA
dTTP
dTDP
dTMP
Thymidine
Thymine
(R)-Dihydro-thymine
(R)-3-Ureido-isobutyrate
(R)-3-Amino-isobutyrate
Valine, leucine and isoleucine metabolism
2-Deoxy-D-ribose-1P
3.6.1.8
3.6.1.39
3.2.2.10
1.17.99.4
K17828...
K00226
K01518
K01511...
K01510...
K09903
K01513...
K01119...
K00757
K01240
K16328...
K17722...
K00207
K01464
K01431...
K19794
K19795
K01485
K00756
K10213
K21057...
K00945...
K09020
K09018...
K09021
K09023
K16066
K09019
K00527
K21636
K19796
K19797
K00893...
K08722
K16904
K09887
K01493
K00857
K00758
K03465
K08728
2, 9, 3, 3
2, 9, 2, 2
2, 9, 3, 2
2, 8, 2, 3
2, 8, 3, 3
2, 6, 1, 3
1, 8, 2, 3
1, 9, 3, 3
0, 5, 3, 1
0, 8, 3, 3
0, 1, 0, 0
2, 6, 0, 0
00240 9/28/17
(c) Kanehisa Laboratories

5. Amino Acid metabolism

MAP	PATHWAY
**220**	Arginine biosynthesis
**250**	Alanine, aspartate and glutamate metabolism
**260**	Glycine, serine and threonine metabolism
**270**	Cysteine and methionine metabolism
**280**	Valine, leucine and isoleucine degradation
**290**	Valine, leucine and isoleucine biosynthesis
**300**	Lysine biosynthesis
**310**	Lysine degradation
**330**	Arginine and proline metabolism
**340**	Histidine metabolism
**350**	Tyrosine metabolism
**360**	Phenylalanine metabolism
**380**	Tryptophan metabolism
**400**	Phenylalanine, tyrosine and tryptophan biosynthesis

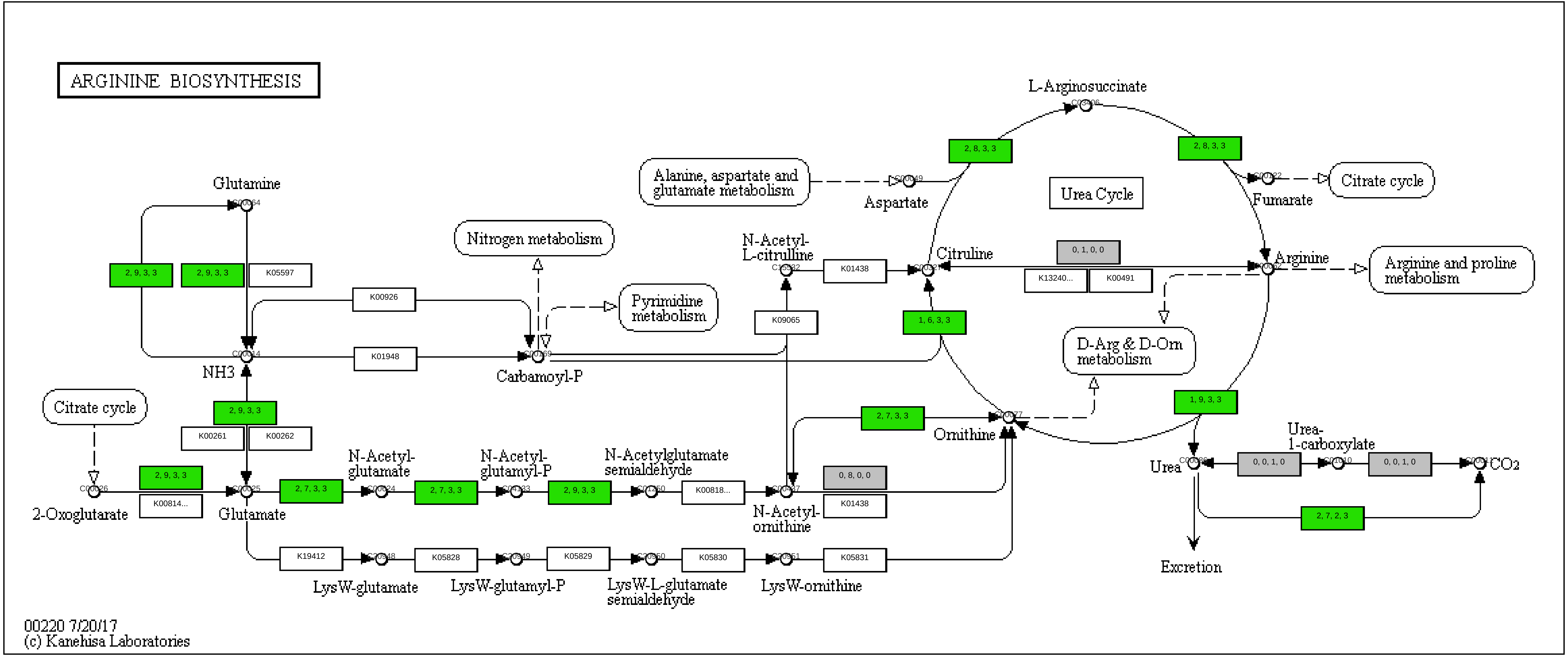

ARGININE BIOSYNTHESIS
Glutamine
2, 9, 3, 3
2, 9, 3, 3
K05597
K00926
Nitrogen metabolism
Pyrimidine metabolism
K01948
NH3
Carbamoyl-P
Citrate cycle
2, 9, 3, 3
K00261
K00262
2, 9, 3, 3
K00814...
2-Oxoglutarate
Glutamate
2, 7, 3, 3
N-Acetyl-glutamate
2, 7, 3, 3
N-Acetyl-glutamyl-P
2, 9, 3, 3
N-Acetylglutamate semialdehyde
K00818...
N-Acetyl-ornithine
0, 8, 0, 0
K01438
K19412
LysW-glutamate
K05828
LysW-glutamyl-P
K05829
LysW-L-glutamate semialdehyde
K05830
LysW-ornithine
K05831
Alanine, aspartate and glutamate metabolism
Aspartate
N-Acetyl-L-citrulline
K01438
K09065
Citruline
1, 6, 3, 3
L-Arginosuccinate
2, 8, 3, 3
2, 8, 3, 3
Urea Cycle
Fumarate
Citrate cycle
0, 1, 0, 0
K13240...
K00491
Arginine
Arginine and proline metabolism
D-Arg & D-Orn metabolism
2, 7, 3, 3
Ornithine
1, 9, 3, 3
Urea
Urea-1-carboxylate
0, 0, 1, 0
0, 0, 1, 0
CO2
2, 7, 2, 3
Excretion
00220 7/20/17
(c) Kanehisa Laboratories

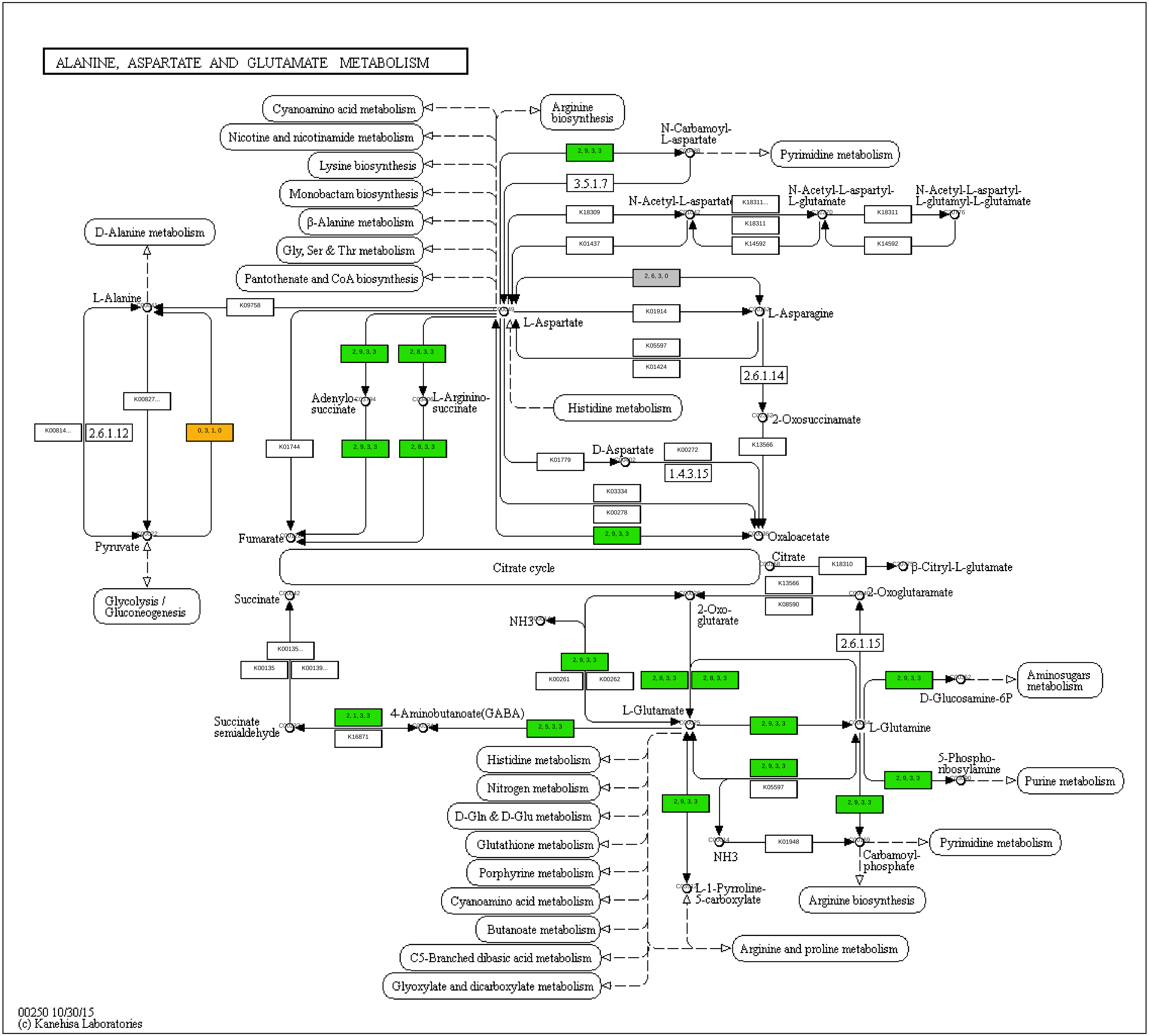
ALANINE, ASPARTATE AND GLUTAMATE METABOLISM
Cyanoamino acid metabolism
Nicotine and nicotinamide metabolism
Lysine biosynthesis
Monobactam biosynthesis
β-Alanine metabolism
Gly, Ser & Thr metabolism
Pantothenate and CoA biosynthesis
Arginine biosynthesis
N-Carbamoyl-L-aspartate
Pyrimidine metabolism
2, 9, 3, 3
3.5.1.7
K18309
N-Acetyl-L-aspartate
K18311...
K18311
K14592
N-Acetyl-L-aspartyl-L-glutamate
N-Acetyl-L-aspartyl-L-glutamyl-L-glutamate
K01437
D-Alanine metabolism
L-Alanine
K09758
2, 6, 3, 0
L-Aspartate
K01914
L-Asparagine
K05597
K01424
2, 8, 3, 3
Adenylo-succinate
L-Arginino-succinate
K00827...
K00814...
2.6.1.12
0, 3, 1, 0
K01744
2.6.1.14
Histidine metabolism
2-Oxosuccinamate
K13566
D-Aspartate
K01779
K00272
1.4.3.15
K03334
K00278
Fumarate
Pyruvate
Oxaloacetate
Citrate cycle
Citrate
K18310
β-Citryl-L-glutamate
Glycolysis / Gluconeogenesis
Succinate
K08590
2-Oxoglutaramate
NH3
2-Oxo-glutarate
2.6.1.15
K00135...
K00135
K00139...
K00261
K00262
Aminosugars metabolism
D-Glucosamine-6P
2, 1, 3, 3
4-Aminobutanoate(GABA)
2, 5, 3, 3
L-Glutamate
L-Glutamine
Succinate semialdehyde
K16871
Histidine metabolism
Nitrogen metabolism
D-Gln & D-Glu metabolism
Glutathione metabolism
Porphyrine metabolism
Cyanoamino acid metabolism
Butanoate metabolism
C5-Branched dibasic acid metabolism
Glyoxylate and dicarboxylate metabolism
5-Phospho-ribosylamine
Purine metabolism
K01948
Carbamoyl-phosphate
Pyrimidine metabolism
Arginine biosynthesis
L-1-Pyrroline-5-carboxylate
Arginine and proline metabolism
00250 10/30/15
(c) Kanehisa Laboratories

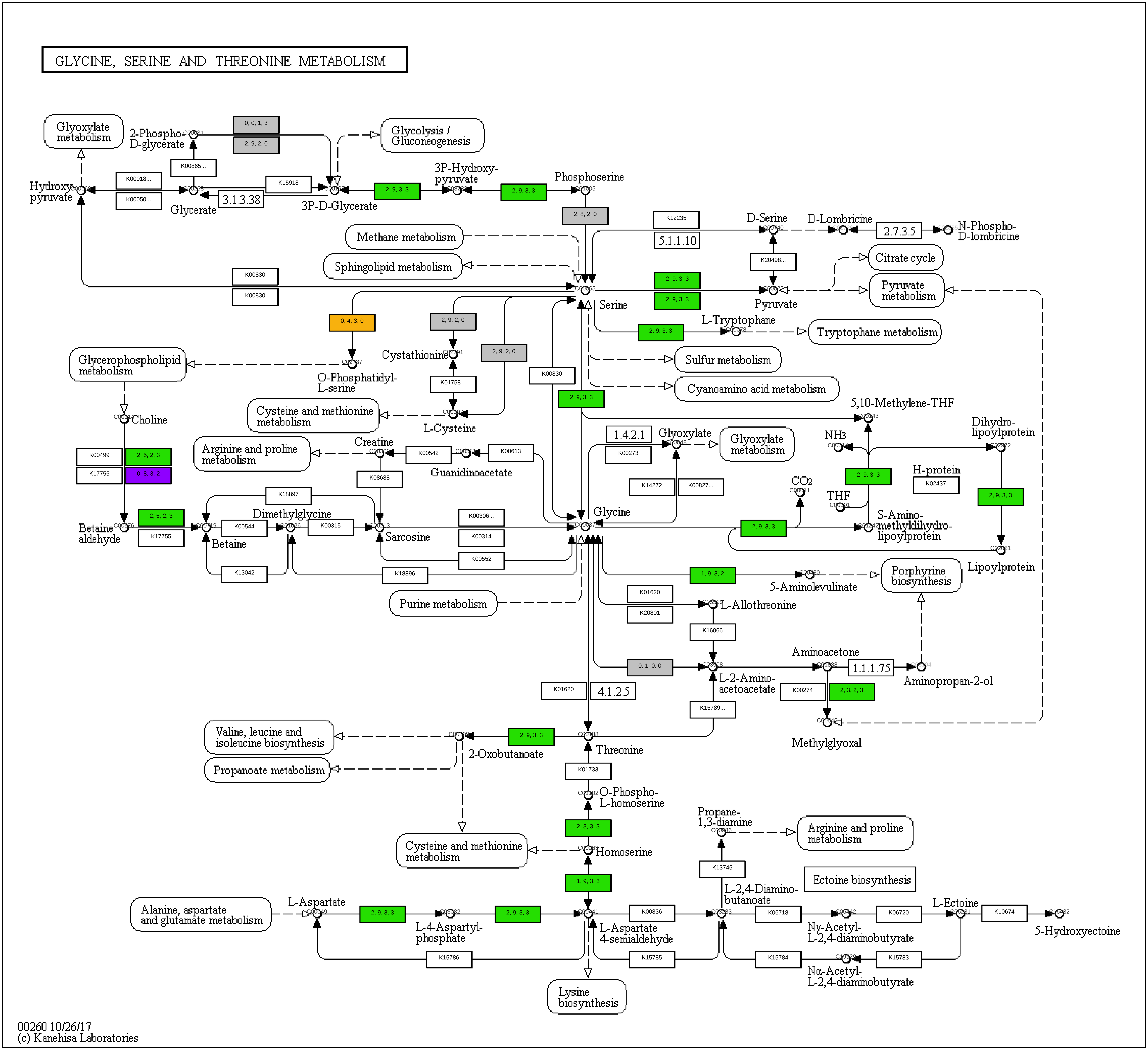
GLYCINE, SERINE AND THREONINE METABOLISM
Glyoxylate metabolism
2-Phospho-D-glycerate
0, 0, 1, 3
2, 9, 2, 0
Glycolysis / Gluconeogenesis
K00865...
K00018...
K00050...
K15918
3.1.3.38
Hydroxy-pyruvate
Glycerate
3P-D-Glycerate
2, 9, 3, 3
3P-Hydroxy-pyruvate
Phosphoserine
2, 8, 2, 0
K12235
5.1.1.10
D-Serine
D-Lombricine
2.7.3.5
N-Phospho-D-lombricine
Methane metabolism
Sphingolipid metabolism
K20498...
Citrate cycle
Pyruvate metabolism
K00830
Serine
Pyruvate
L-Tryptophan
Tryptophan metabolism
0, 4, 3, 0
2, 9, 2, 0
Glycerophospholipid metabolism
Cystathionine
O-Phosphatidyl-L-serine
K01758...
Sulfur metabolism
Cyanoamino acid metabolism
Choline
Cysteine and methionine metabolism
L-Cysteine
5,10-Methylene-THF
Dihydro-lipoylprotein
1.4.2.1
Glyoxylate
Glyoxylate metabolism
NH3
K00273
Arginine and proline metabolism
Creatine
K00542
K00613
K00499
2, 5, 2, 3
K17755
0, 8, 3, 2
Guanidinoacetate
K08688
H-protein
K02437
K14272
K00827...
CO2
THF
K18897
Dimethylglycine
Glycine
S-Amino-methyldihydro-lipoylprotein
Betaine aldehyde
K00544
K00315
K00306...
K00314
K00552
Betaine
Sarcosine
K13042
K18896
1, 9, 3, 2
5-Aminolevulinate
Porphyrine biosynthesis
Lipoylprotein
Purine metabolism
K01620
K20801
L-Allothreonine
K16066
0, 1, 0, 0
Aminoacetone
1.1.1.75
Aminopropan-2-ol
L-2-Amino-acetoacetate
K00274
2, 3, 2, 3
4.1.2.5
K15789...
Methylglyoxal
Valine, leucine and isoleucine biosynthesis
2-Oxobutanoate
Threonine
Propanoate metabolism
K01733
O-Phospho-L-homoserine
2, 8, 3, 3
Propane-1,3-diamine
Arginine and proline metabolism
Cysteine and methionine metabolism
Homoserine
K13745
1, 9, 3, 3
Ectoine biosynthesis
L-2,4-Diamino-butanoate
Alanine, aspartate and glutamate metabolism
L-Aspartate
L-4-Aspartyl-phosphate
K00836
L-Aspartate 4-semialdehyde
K06718
Nγ-Acetyl-L-2,4-diaminobutyrate
K06720
L-Ectoine
K10674
5-Hydroxyectoine
K15786
K15785
K15784
K15783
Nα-Acetyl-L-2,4-diaminobutyrate
Lysine biosynthesis
00260 10/26/17
(c) Kanehisa Laboratories

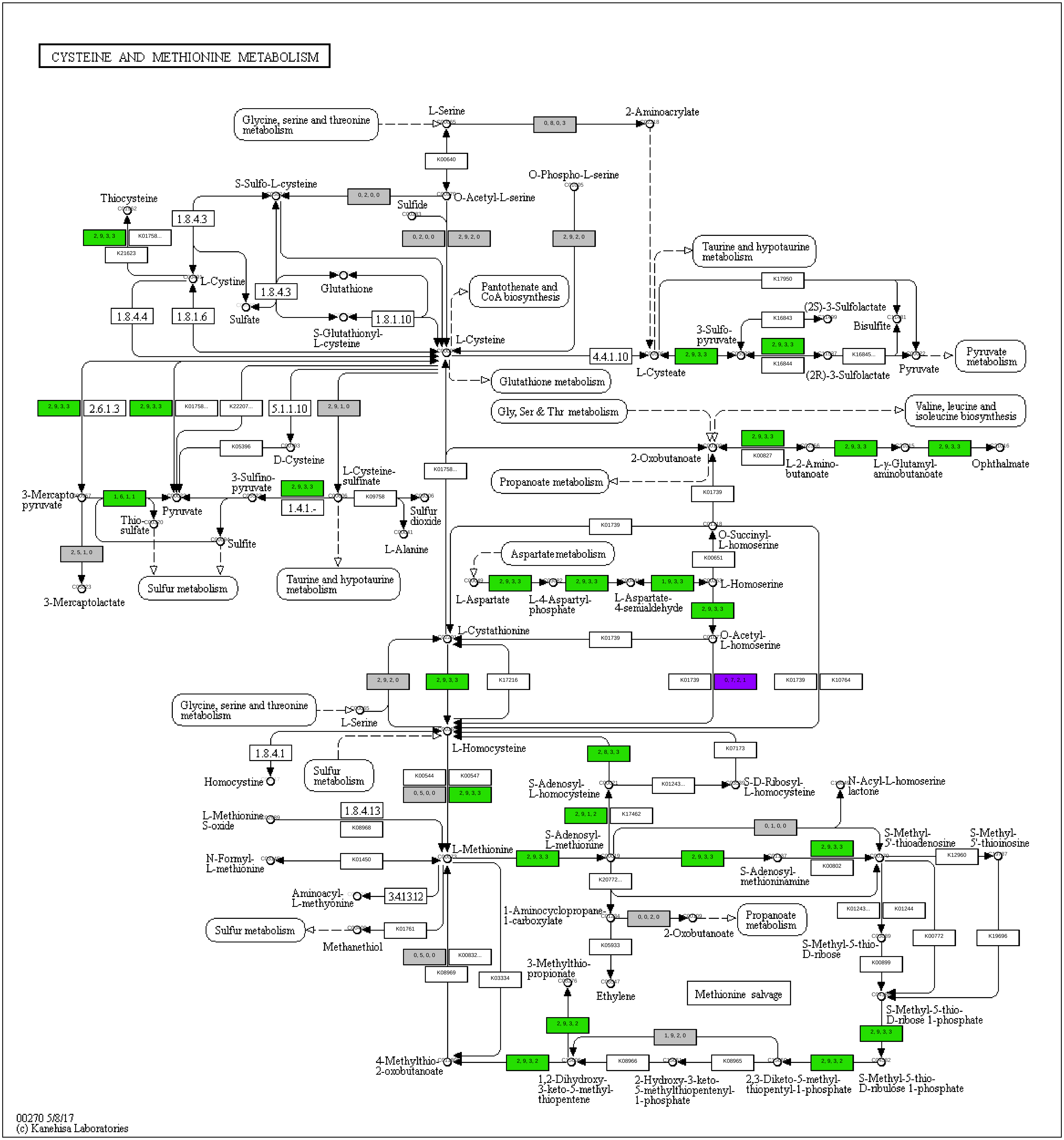
CYSTEINE AND METHIONINE METABOLISM
Glycine, serine and threonine metabolism
L-Serine
0, 8, 0, 3
2-Aminoacrylate
K00640
O-Phospho-L-serine
S-Sulfo-L-cysteine
0, 2, 0, 0
O-Acetyl-L-serine
Sulfide
Thiocysteine
1.8.4.3
2, 9, 3, 3
K01758...
K21623
0, 2, 0, 0
2, 9, 2, 0
2, 9, 2, 0
Taurine and hypotaurine metabolism
L-Cystine
1.8.4.3
Glutathione
Pantothenate and CoA biosynthesis
K17950
1.8.4.4
1.8.1.6
Sulfate
S-Glutathionyl-L-cysteine
1.8.1.10
L-Cysteine
(2S)-3-Sulfolactate
K16843
Bisulfite
3-Sulfo-pyruvate
2, 9, 3, 3
2, 9, 3, 3
4.4.1.10
K16844
K16845...
Pyruvate
Pyruvate metabolism
L-Cysteate
(2R)-3-Sulfolactate
Glutathione metabolism
2, 9, 3, 3
2.6.1.3
2, 9, 3, 3
K01758...
K22207...
5.1.1.10
2, 9, 1, 0
Gly, Ser & Thr metabolism
Valine, leucine and isoleucine biosynthesis
2, 9, 3, 3
2, 9, 3, 3
2, 9, 3, 3
K05396
D-Cysteine
K00827
2-Oxobutanoate
L-2-Amino-butanoate
L-γ-Glutamyl-aminobutanoate
Ophthalmate
K01758...
Propanoate metabolism
3-Sulfino-pyruvate
2, 9, 3, 3
L-Cysteine-sulfinate
3-Mercapto-pyruvate
1, 6, 1, 1
Pyruvate
K09758
K01739
1.4.1.-
Sulfur dioxide
Thio-sulfate
Sulfite
K01739
O-Succinyl-L-homoserine
L-Alanine
2, 5, 1, 0
Aspartate metabolism
K00651
Sulfur metabolism
Taurine and hypotaurine metabolism
2, 9, 3, 3
2, 9, 3, 3
1, 9, 3, 3
L-Homoserine
3-Mercaptolactate
L-Aspartate
L-4-Aspartyl-phosphate
L-Aspartate-4-semialdehyde
2, 9, 3, 3
L-Cystathionine
K01739
O-Acetyl-L-homoserine
2, 9, 2, 0
2, 9, 3, 3
K17216
K01739
0, 7, 2, 1
K01739
K10764
Glycine, serine and threonine metabolism
L-Serine
L-Homocysteine
2, 8, 3, 3
K07173
1.8.4.1
Sulfur metabolism
K00544
K00547
0, 5, 0, 0
2, 9, 3, 3
S-Adenosyl-L-homocysteine
K01243...
S-D-Ribosyl-L-homocysteine
N-Acyl-L-homoserine lactone
Homocystine
2, 9, 1, 2
K17462
L-Methionine S-oxide
1.8.4.13
K08968
0, 1, 0, 0
S-Adenosyl-L-methionine
S-Methyl-5'-thioadenosine
S-Methyl-5'-thioinosine
L-Methionine
2, 9, 3, 3
2, 9, 3, 3
2, 9, 3, 3
N-Formyl-L-methionine
K01450
K12960
K00802
S-Adenosyl-methioninamine
K20772...
Aminoacyl-L-methyonine
3.4.13.12
1-Aminocyclopropane-1-carboxylate
0, 0, 2, 0
Propanoate metabolism
2-Oxobutanoate
K01243...
K01244
Sulfur metabolism
K01761
Methanethiol
K05933
S-Methyl-5-thio-D-ribose
K00772
K19696
0, 5, 0, 0
K00832...
K00899
K08969
K03334
3-Methylthio-propionate
Ethylene
Methionine salvage
S-Methyl-5-thio-D-ribose 1-phosphate
2, 9, 3, 2
2, 9, 3, 3
1, 9, 2, 0
4-Methylthio-2-oxobutanoate
2, 9, 3, 2
K08966
K08965
2, 9, 3, 2
1,2-Dihydroxy-3-keto-5-methyl-thiopentene
2-Hydroxy-3-keto-5-methylthiopentenyl-1-phosphate
2,3-Diketo-5-methyl-thiopentyl-1-phosphate
S-Methyl-5-thio-D-ribulose 1-phosphate
00270 5/8/17
(c) Kanehisa Laboratories

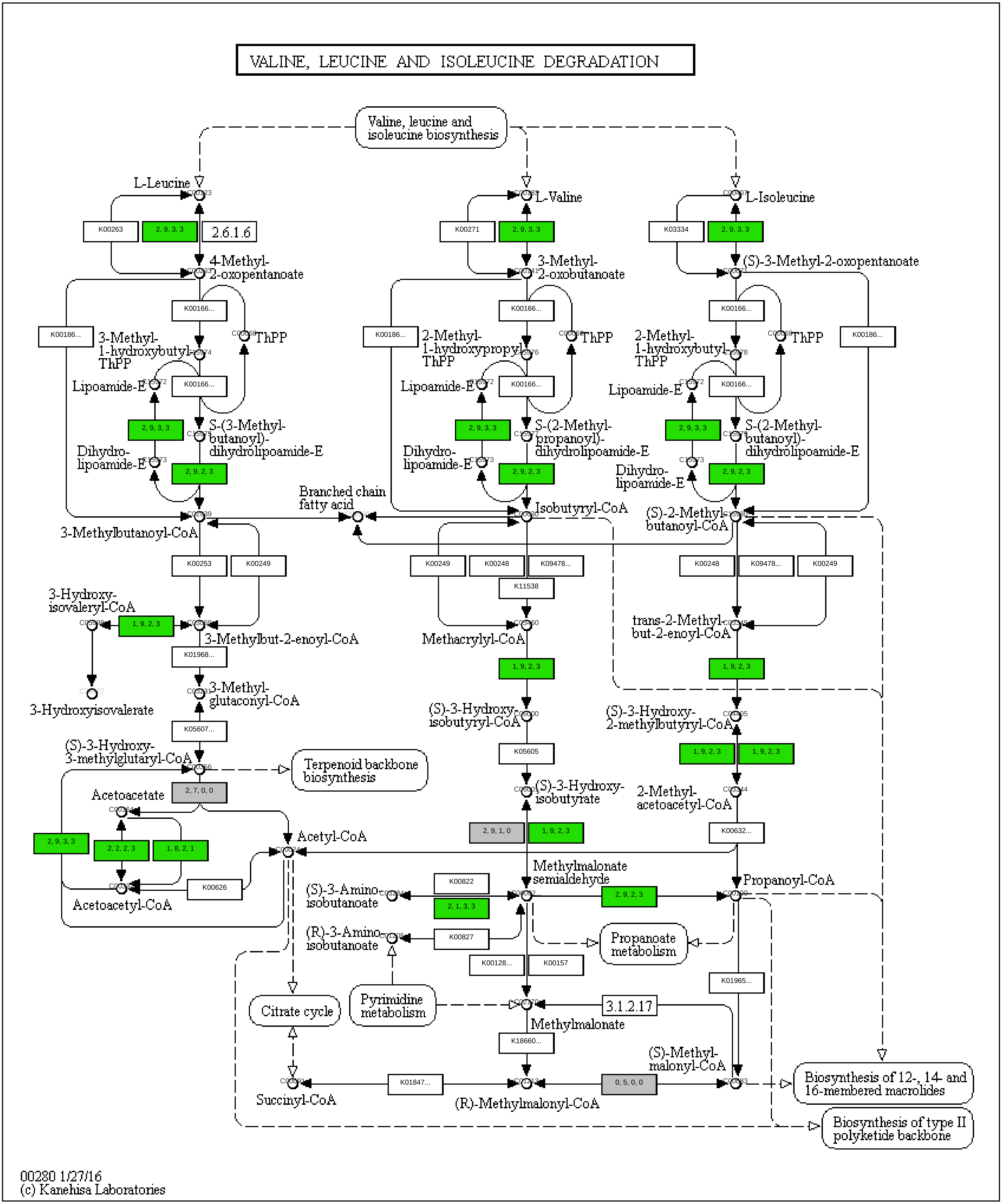
VALINE, LEUCINE AND ISOLEUCINE DEGRADATION
Valine, leucine and isoleucine biosynthesis
L-Leucine
L-Valine
L-Isoleucine
K00263
2, 9, 3, 3
2.6.1.6
K00271
K03334
4-Methyl-2-oxopentanoate
3-Methyl-2-oxobutanoate
(S)-3-Methyl-2-oxopentanoate
K00166...
K00186...
ThPP
3-Methyl-1-hydroxybutyl-ThPP
2-Methyl-1-hydroxypropyl-ThPP
2-Methyl-1-hydroxybutyl-ThPP
Lipoamide-E
S-(3-Methylbutanoyl)-dihydrolipoamide-E
S-(2-Methylpropanoyl)-dihydrolipoamide-E
S-(2-Methylbutanoyl)-dihydrolipoamide-E
Dihydrolipoamide-E
2, 9, 2, 3
Branched chain fatty acid
3-Methylbutanoyl-CoA
Isobutyryl-CoA
(S)-2-Methylbutanoyl-CoA
K00253
K00249
K00248
K09478...
K11538
3-Hydroxyisovaleryl-CoA
1, 9, 2, 3
3-Methylbut-2-enoyl-CoA
Methacrylyl-CoA
trans-2-Methylbut-2-enoyl-CoA
K01968...
3-Hydroxyisovalerate
3-Methylglutaconyl-CoA
K05607...
(S)-3-Hydroxyisobutyryl-CoA
(S)-3-Hydroxy-2-methylbutyryl-CoA
(S)-3-Hydroxy-3-methylglutaryl-CoA
Terpenoid backbone biosynthesis
K05605
2, 7, 0, 0
Acetoacetate
(S)-3-Hydroxyisobutyrate
2-Methylacetoacetyl-CoA
2, 9, 1, 0
K00632...
2, 2, 2, 3
1, 8, 2, 1
Acetyl-CoA
K00626
Acetoacetyl-CoA
Methylmalonate semialdehyde
Propanoyl-CoA
K00822
2, 1, 3, 3
(S)-3-Aminoisobutanoate
(R)-3-Aminoisobutanoate
K00827
Propanoate metabolism
K00128...
K00157
K01965...
Pyrimidine metabolism
Citrate cycle
3.1.2.17
Methylmalonate
K18660...
(S)-Methylmalonyl-CoA
K01847...
0, 5, 0, 0
Succinyl-CoA
(R)-Methylmalonyl-CoA
Biosynthesis of 12-, 14- and 16-membered macrolides
Biosynthesis of type II polyketide backbone
00280 1/27/16
(c) Kanehisa Laboratories

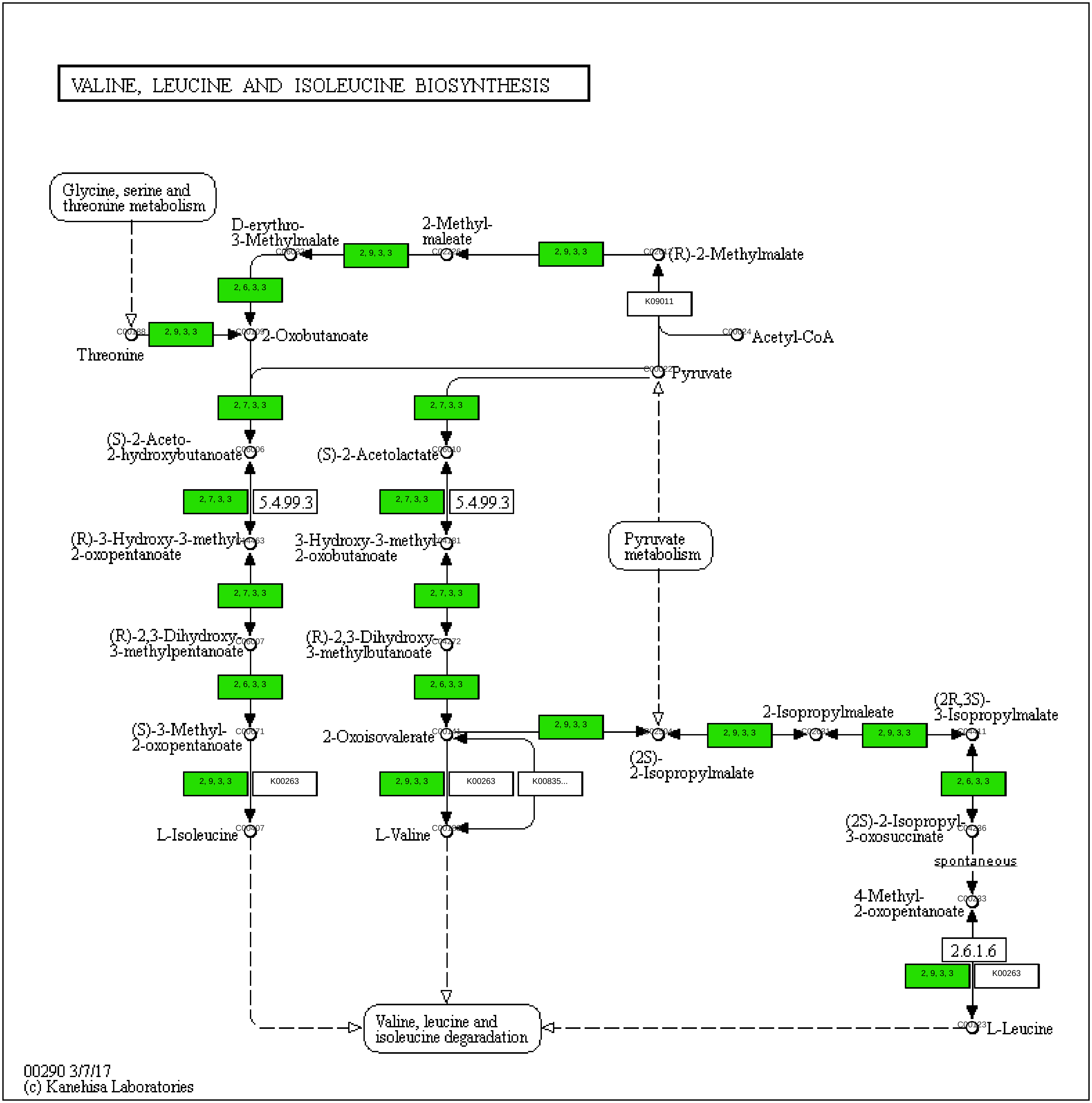

VALINE, LEUCINE AND ISOLEUCINE BIOSYNTHESIS
Glycine, serine and threonine metabolism
D-erythro-3-Methylmalate
2-Methyl-maleate
(R)-2-Methylmalate
2, 9, 3, 3
2, 6, 3, 3
K09011
Threonine
2-Oxobutanoate
Acetyl-CoA
Pyruvate
2, 7, 3, 3
(S)-2-Aceto-2-hydroxybutanoate
(S)-2-Acetolactate
5.4.99.3
(R)-3-Hydroxy-3-methyl-2-oxopentanoate
3-Hydroxy-3-methyl-2-oxobutanoate
Pyruvate metabolism
(R)-2,3-Dihydroxy-3-methylpentanoate
(R)-2,3-Dihydroxy-3-methylbutanoate
(S)-3-Methyl-2-oxopentanoate
2-Oxoisovalerate
2-Isopropylmaleate
(2R,3S)-3-Isopropylmalate
(2S)-2-Isopropylmalate
K00263
K00835...
L-Isoleucine
L-Valine
(2S)-2-Isopropyl-3-oxosuccinate
spontaneous
4-Methyl-2-oxopentanoate
2.6.1.6
Valine, leucine and isoleucine degaradation
L-Leucine
00290 3/7/17
(c) Kanehisa Laboratories

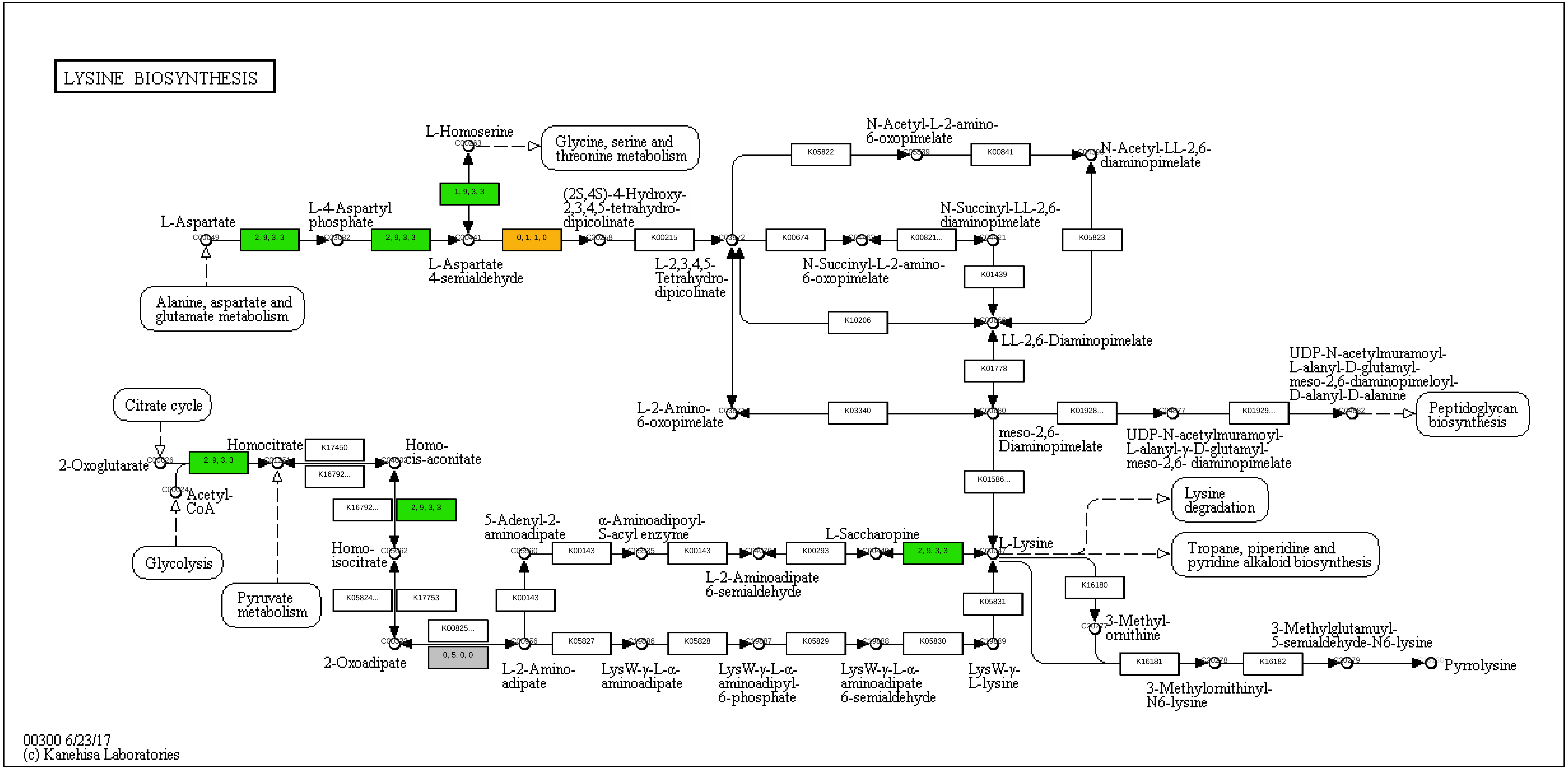
LYSINE BIOSYNTHESIS
L-Homoserine
Glycine, serine and threonine metabolism
1, 9, 3, 3
L-Aspartate
L-4-Aspartyl phosphate
2, 9, 3, 3
2, 9, 3, 3
0, 1, 1, 0
(2S,4S)-4-Hydroxy-2,3,4,5-tetrahydro-dipicolinate
K00215
L-Aspartate 4-semialdehyde
L-2,3,4,5-Tetrahydro-dipicolinate
Alanine, aspartate and glutamate metabolism
N-Acetyl-L-2-amino-6-oxopimelate
K05822
K00841
N-Acetyl-LL-2,6-diaminopimelate
N-Succinyl-LL-2,6-diaminopimelate
K00674
K00821...
K05823
N-Succinyl-L-2-amino-6-oxopimelate
K01439
K10206
LL-2,6-Diaminopimelate
K01778
UDP-N-acetylmuramoyl-L-alanyl-D-glutamyl-meso-2,6-diaminopimeloyl-D-alanyl-D-alanine
Peptidoglycan biosynthesis
L-2-Amino-6-oxopimelate
K03340
K01928...
K01929...
meso-2,6-Diaminopimelate
UDP-N-acetylmuramoyl-L-alanyl-γ-D-glutamyl-meso-2,6- diaminopimelate
Citrate cycle
2-Oxoglutarate
2, 9, 3, 3
Homocitrate
K17450
K16792...
Homo-cis-aconitate
Acetyl-CoA
K16792...
2, 9, 3, 3
K01586...
Lysine degradation
5-Adenyl-2-aminoadipate
α-Aminoadipoyl-S-acyl enzyme
L-Saccharopine
L-Lysine
Tropane, piperidine and pyridine alkaloid biosynthesis
Glycolysis
Homo-isocitrate
K00143
K00143
K00293
2, 9, 3, 3
L-2-Aminoadipate 6-semialdehyde
K16180
Pyruvate metabolism
K05824...
K17753
K00143
K05831
3-Methyl-ornithine
3-Methylglutamuyl-5-semialdehyde-N6-lysine
K00825...
0, 5, 0, 0
K05827
K05828
K05829
K05830
K16181
K16182
Pyrrolysine
2-Oxoadipate
L-2-Amino-adipate
LysW-γ-L-α-aminoadipate
LysW-γ-L-α-aminoadipyl-6-phosphate
LysW-γ-L-α-aminoadipate 6-semialdehyde
LysW-γ-L-lysine
3-Methylornithinyl-N6-lysine
00300 6/23/17
(c) Kanehisa Laboratories

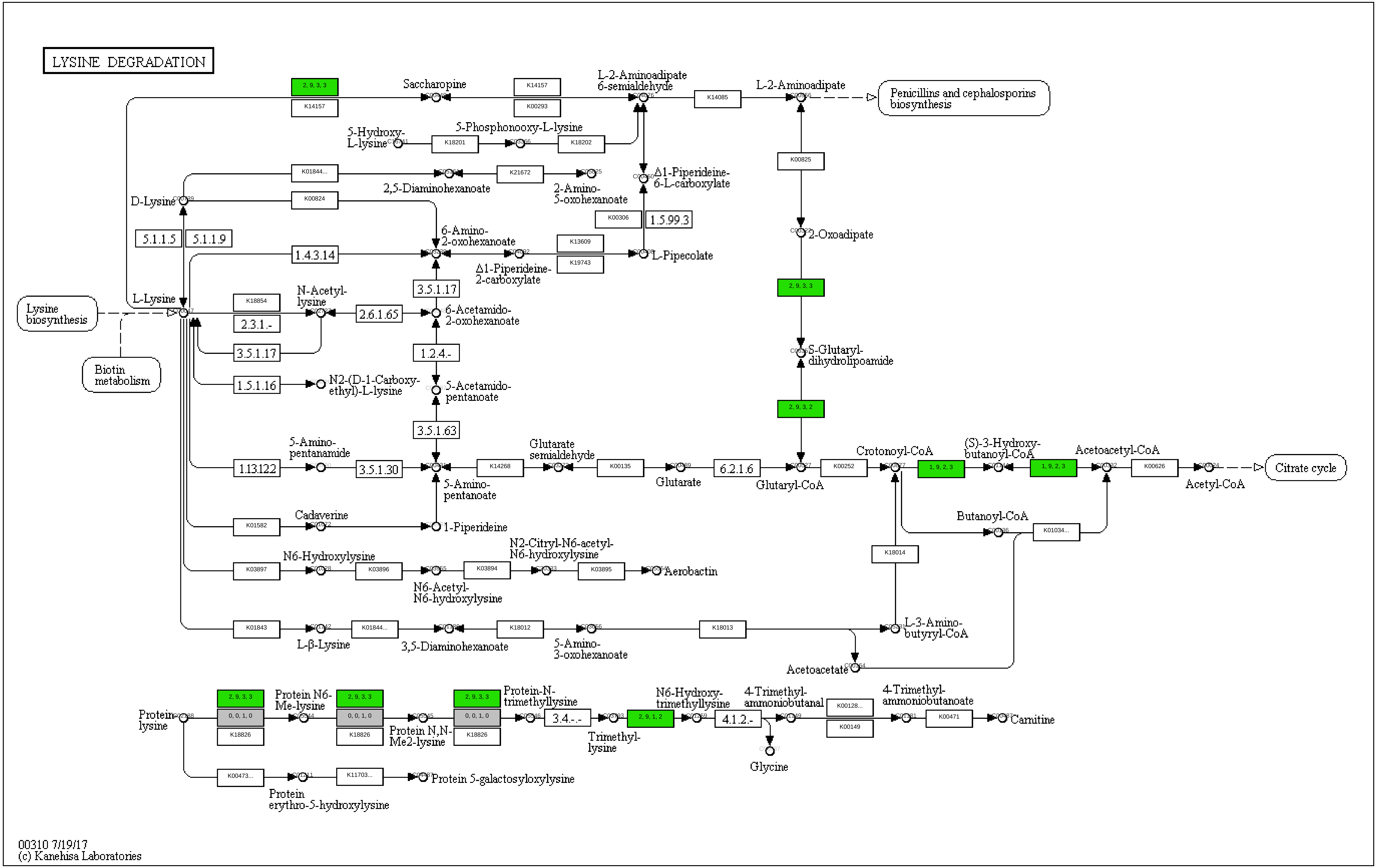

LYSINE DEGRADATION
2, 9, 3, 3
K14157
Saccharopine
K14157
K00293
L-2-Aminoadipate 6-semialdehyde
K14085
L-2-Aminoadipate
Penicillins and cephalosporins biosynthesis
5-Hydroxy-L-lysine
K18201
5-Phosphonooxy-L-lysine
K18202
K01844...
K21672
2,5-Diaminohexanoate
2-Amino-5-oxohexanoate
Δ1-Piperideine-6-L-carboxylate
K00825
D-Lysine
K00824
K00306
1.5.99.3
5.1.1.5
5.1.1.9
6-Amino-2-oxohexanoate
2-Oxoadipate
1.4.3.14
K13609
K19743
L-Pipecolate
Δ1-Piperideine-2-carboxylate
3.5.1.17
2, 9, 3, 3
L-Lysine
Lysine biosynthesis
K18854
N-Acetyl-lysine
2.6.1.65
6-Acetamido-2-oxohexanoate
2.3.1.-
3.5.1.17
1.2.4.-
S-Glutaryl-dihydrolipoamide
Biotin metabolism
1.5.1.16
N2-(D-1-Carboxyethyl)-L-lysine
5-Acetamido-pentanoate
2, 9, 3, 2
3.5.1.63
5-Amino-pentanamide
Glutarate semialdehyde
Crotonoyl-CoA
(S)-3-Hydroxy-butanoyl-CoA
Acetoacetyl-CoA
1.13.12.2
3.5.1.30
K14268
K00135
6.2.1.6
K00252
1, 9, 2, 3
1, 9, 2, 3
K00626
Citrate cycle
5-Amino-pentanoate
Glutarate
Glutaryl-CoA
Acetyl-CoA
Cadaverine
Butanoyl-CoA
K01582
1-Piperideine
K01034...
K18014
N6-Hydroxylysine
N2-Citryl-N6-acetyl-N6-hydroxylysine
K03897
K03896
K03894
K03895
Aerobactin
N6-Acetyl-N6-hydroxylysine
K01843
K01844...
K18012
K18013
L-3-Amino-butyryl-CoA
L-β-Lysine
3,5-Diaminohexanoate
5-Amino-3-oxohexanoate
Acetoacetate
Protein N6-Me-lysine
Protein-N-trimethyllysine
N6-Hydroxy-trimethyllysine
4-Trimethyl-ammoniobutanal
4-Trimethyl-ammoniobutanoate
2, 9, 3, 3
2, 9, 3, 3
2, 9, 3, 3
0, 0, 1, 0
0, 0, 1, 0
0, 0, 1, 0
Protein-lysine
K18826
K18826
K18826
3.4.-.-
2, 9, 1, 2
4.1.2.-
K00128...
K00149
K00471
Carnitine
Protein N,N-Me2-lysine
Trimethyl-lysine
Glycine
K00473...
K11703...
Protein 5-galactosyloxylysine
Protein erythro-5-hydroxylysine
00310 7/19/17
(c) Kanehisa Laboratories

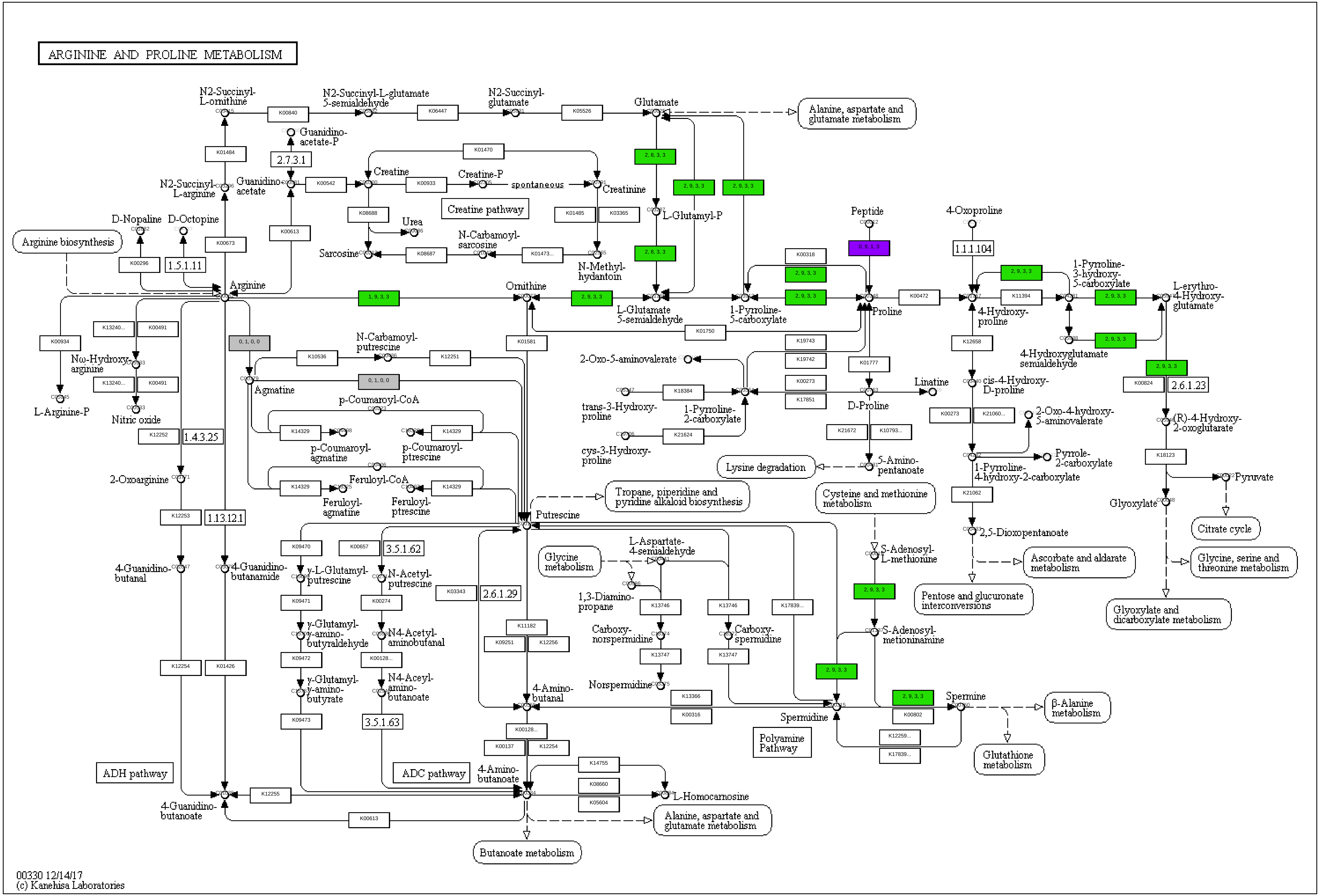
ARGININE AND PROLINE METABOLISM
N2-Succinyl-L-ornithine
K00840
N2-Succinyl-L-glutamate 5-semialdehyde
K06447
N2-Succinyl-glutamate
K05526
Glutamate
Alanine, aspartate and glutamate metabolism
Guanidino-acetate-P
2.7.3.1
K01470
K01484
N2-Succinyl-L-arginine
Guanidino-acetate
K00542
Creatine
K00933
Creatine-P
spontaneous
Creatinine
2.8.3.3
2.9.3.3
2.9.3.3
Creatine pathway
K08688
Urea
K01485
K03365
L-Glutamyl-P
Peptide
4-Oxoproline
Arginine biosynthesis
D-Nopaline
D-Octopine
K00673
K00613
Sarcosine
K08687
N-Carbamoyl-sarcosine
K01473...
0.9.1.3
1.1.1.104
K00296
1.5.1.11
2.8.3.3
N-Methyl-hydantoin
K00318
2.9.3.3
2.9.3.3
1-Pyrroline-3-hydroxy-5-carboxylate
Arginine
1.9.3.3
Ornithine
2.9.3.3
L-Glutamate 5-semialdehyde
1-Pyrroline-5-carboxylate
2.9.3.3
K00472
Proline
K11394
2.9.3.3
L-erythro-4-Hydroxy-glutamate
4-Hydroxy-proline
K01750
K00934
K13240...
K00491
0.1.0.0
K01581
K19743
K01777
K12658
2.9.3.3
4-Hydroxyglutamate semialdehyde
Nω-Hydroxy-arginine
K10536
N-Carbamoyl-putrescine
K12251
2-Oxo-5-aminovalerate
K19742
2.9.3.3
K13240...
K00491
0.1.0.0
K00273
Linatine
cis-4-Hydroxy-D-proline
K00824
2.6.1.23
L-Arginine-P
Agmatine
p-Coumaroyl-CoA
trans-3-Hydroxy-proline
K18384
1-Pyrroline-2-carboxylate
K17851
D-Proline
K00273
K21060...
2-Oxo-4-hydroxy-5-aminovalerate
(R)-4-Hydroxy-2-oxoglutarate
Nitric oxide
K12252
1.4.3.25
K14329
K14329
K21624
cys-3-Hydroxy-proline
K21672
K10793...
Pyrrole-2-carboxylate
p-Coumaroyl-agmatine
p-Coumaroyl-ptrescine
Lysine degradation
5-Amino-pentanoate
1-Pyrroline-4-hydroxy-2-carboxylate
K18123
Pyruvate
2-Oxoarginine
Feruloyl-CoA
K14329
K14329
Feruloyl-agmatine
Feruloyl-ptrescine
Tropane, piperidine and pyridine alkaloid biosynthesis
Cysteine and methionine metabolism
K21062
Glyoxylate
Citrate cycle
K12253
1.13.12.1
Putrescine
2,5-Dioxopentanoate
K09470
K00657
3.5.1.62
Glycine metabolism
L-Aspartate-4-semialdehyde
S-Adenosyl-L-methionine
Ascorbate and aldarate metabolism
Glycine, serine and threonine metabolism
4-Guanidino-butanal
4-Guanidino-butanamide
γ-L-Glutamyl-putrescine
N-Acetyl-putrescine
K03343
2.6.1.29
1,3-Diamino-propane
2.9.3.3
Pentose and glucuronate interconversions
Glyoxylate and dicarboxylate metabolism
K09471
K00274
K13746
K13746
K17839...
K11182
Carboxy-norspermidine
Carboxy-spermidine
S-Adenosyl-metionamine
γ-Glutamyl-γ-amino-butyraldehyde
N4-Acetyl-aminobutanal
K09251
K12256
K09472
K00128...
K13747
K13747
2.9.3.3
K12254
K01426
γ-Glutamyl-γ-amino-butyrate
N4-Aceyl-amino-butanoate
4-Amino-butanal
Norspermidine
K13366
2.9.3.3
Spermine
β-Alanine metabolism
K09473
3.5.1.63
K00316
Spermidine
K00802
K00128...
Polyamine Pathway
K12259...
K00137
K12254
K17839...
Glutathione metabolism
ADH pathway
ADC pathway
4-Amino-butanoate
K14755
K08660
L-Homocarnosine
K12255
K05604
4-Guanidino-butanoate
K00613
Alanine, aspartate and glutamate metabolism
Butanoate metabolism
00330 12/14/17
(c) Kanehisa Laboratories

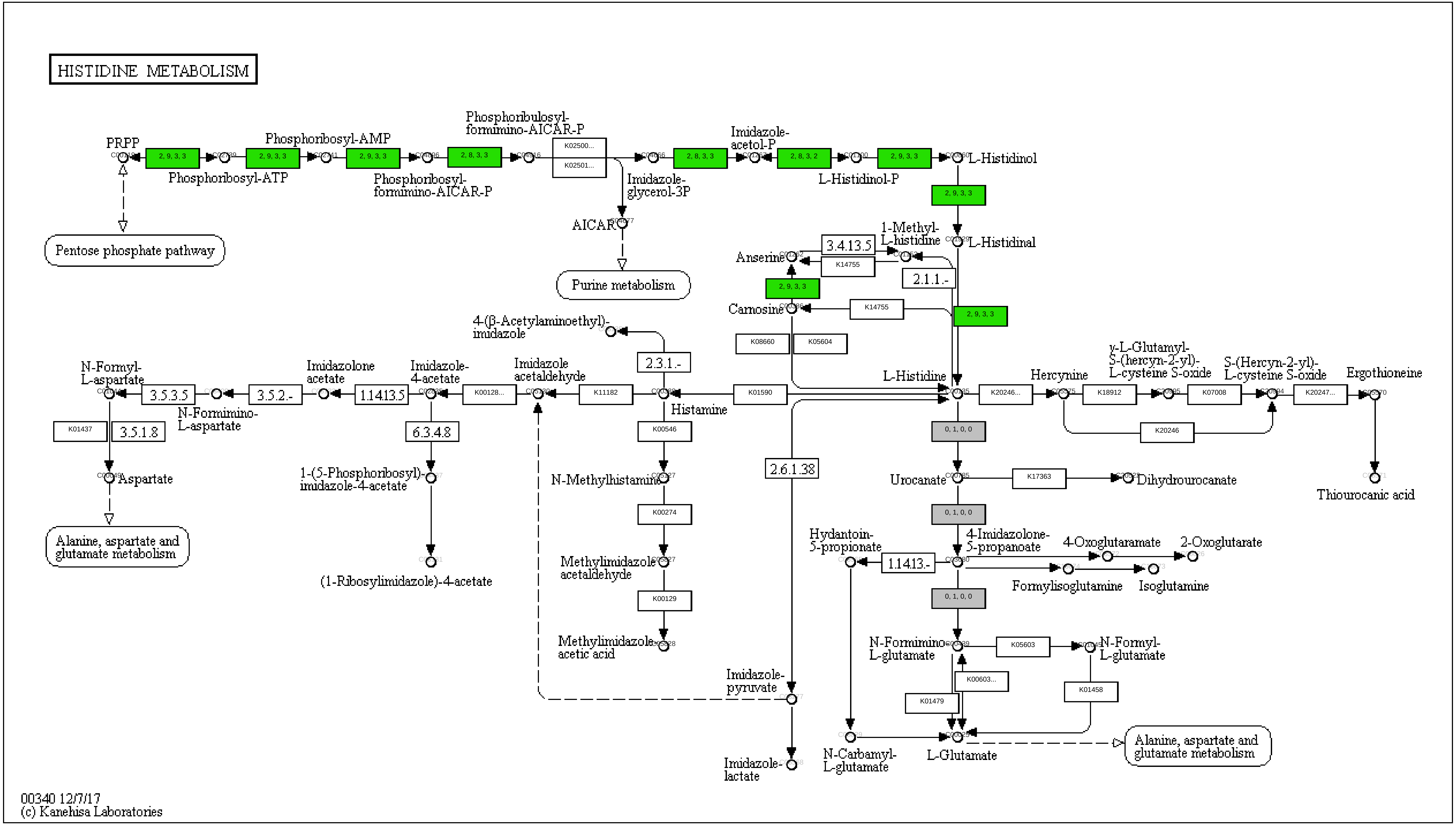
HISTIDINE METABOLISM
PRPP
Phosphoribosyl-ATP
Phosphoribosyl-AMP
Phosphoribosyl-formimino-AICAR-P
Phosphoribulosyl-formimino-AICAR-P
K02500...
K02501...
Imidazole-glycerol-3P
Imidazole-acetol-P
L-Histidinol-P
L-Histidinol
AICAR
Pentose phosphate pathway
Purine metabolism
2, 9, 3, 3
2, 8, 3, 3
2, 8, 3, 2
L-Histidinal
1-Methyl-L-histidine
3.4.13.5
K14755
Anserine
2.1.1.-
Carnosine
K08660
K05604
4-(β-Acetylaminoethyl)-imidazole
2.3.1.-
N-Formyl-L-aspartate
3.5.3.5
N-Formimino-L-aspartate
3.5.2.-
Imidazolone acetate
1.14.13.5
Imidazole-4-acetate
K00128...
Imidazole acetaldehyde
K11182
Histamine
K01590
L-Histidine
Hercynine
K20246...
γ-L-Glutamyl-S-(hercyn-2-yl)-L-cysteine S-oxide
K18912
K07008
S-(Hercyn-2-yl)-L-cysteine S-oxide
K20247...
Ergothioneine
K20246
K01437
3.5.1.8
Aspartate
Alanine, aspartate and glutamate metabolism
6.3.4.8
1-(5-Phosphoribosyl)-imidazole-4-acetate
(1-Ribosylimidazole)-4-acetate
K00546
N-Methylhistamine
K00274
Methylimidazole acetaldehyde
K00129
Methylimidazole-acetic acid
2.6.1.38
0, 1, 0, 0
Urocanate
K17363
Dihydrourocanate
Thiourocanic acid
Hydantoin-5-propionate
1.14.13.-
4-Imidazolone-5-propanoate
4-Oxoglutaramate
2-Oxoglutarate
Formylisoglutamine
Isoglutamine
N-Formimino-L-glutamate
K05603
N-Formyl-L-glutamate
K00603...
K01479
K01458
Imidazole-pyruvate
Imidazole-lactate
N-Carbamyl-L-glutamate
L-Glutamate
Alanine, aspartate and glutamate metabolism
00340 12/7/17
(c) Kanehisa Laboratories

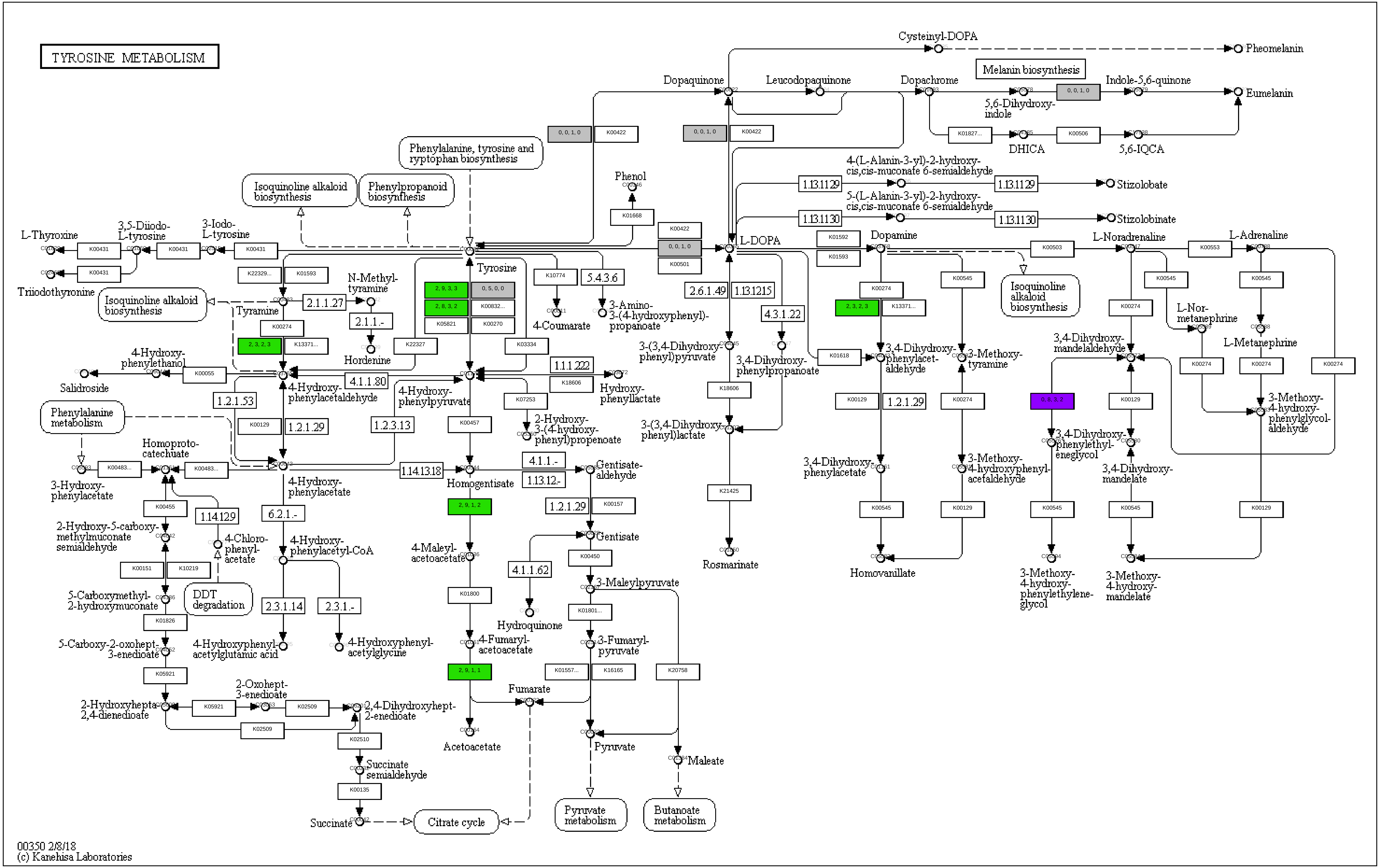
TYROSINE METABOLISM
Cysteinyl-DOPA
Pheomelanin
Melanin biosynthesis
Dopaquinone
Leucodopaquinone
Dopachrome
Indole-5,6-quinone
Eumelanin
5,6-Dihydroxy-indole
DHICA
5,6-IQCA
K01827...
K00506
0, 0, 1, 0
K00422
Phenylalanine, tyrosine and ryptophan biosynthesis
4-(L-Alanin-3-yl)-2-hydroxy-cis,cis-muconate 6-semialdehyde
5-(L-Alanin-3-yl)-2-hydroxy-cis,cis-muconate 6-semialdehyde
1.13.11.29
1.13.11.30
Stizolobate
Stizolobinate
Isoquinoline alkaloid biosynthesis
Phenylpropanoid biosynthesis
Phenol
K01668
L-Thyroxine
3,5-Diiodo-L-tyrosine
3-Iodo-L-tyrosine
K00431
Triiodothyronine
L-DOPA
Dopamine
L-Noradrenaline
L-Adrenaline
K01592
K01593
K00503
K00553
K00501
Tyrosine
K22329...
K01593
N-Methyl-tyramine
2.1.1.27
Tyramine
2.1.1.-
Hordenine
K00274
2, 3, 2, 3
K13371...
2, 9, 3, 3
2, 8, 3, 2
0, 5, 0, 0
K00832...
K05821
K00270
K22327
K03334
K10774
5.4.3.6
4-Coumarate
3-Amino-3-(4-hydroxyphenyl)-propanoate
2.6.1.49
1.13.11.15
4.3.1.22
K00545
Isoquinoline alkaloid biosynthesis
L-Nor-metanephrine
L-Metanephrine
3,4-Dihydroxy-mandelaldehyde
4-Hydroxy-phenylethanol
Salidroside
K00055
4-Hydroxy-phenylacetaldehyde
4.1.1.80
4-Hydroxy-phenylpyruvate
1.1.1.222
K18606
Hydroxy-phenyllactate
K07253
2-Hydroxy-3-(4-hydroxy-phenyl)propenoate
3-(3,4-Dihydroxy-phenyl)pyruvate
3,4-Dihydroxy-phenylpropanoate
3,4-Dihydroxy-phenylacet-aldehyde
3-Methoxy-tyramine
K01618
3-Methoxy-4-hydroxy-phenylglycol-aldehyde
1.2.1.53
K00129
1.2.1.29
1.2.3.13
K00457
0, 8, 3, 2
Phenylalanine metabolism
Homoproto-catechuate
3-Hydroxy-phenylacetate
K00483...
4-Hydroxy-phenylacetate
1.14.13.18
Homogentisate
4.1.1.-
1.13.12.-
Gentisate-aldehyde
3-(3,4-Dihydroxy-phenyl)lactate
3,4-Dihydroxy-phenylethyl-eneglycol
3,4-Dihydroxy-phenylacetate
3-Methoxy-4-hydroxyphenyl-acetaldehyde
3,4-Dihydroxy-mandelate
K00455
1.14.12.9
6.2.1.-
2, 9, 1, 2
1.2.1.29
K00157
K21425
2-Hydroxy-5-carboxy-methylmuconate semialdehyde
4-Chloro-phenyl-acetate
4-Hydroxy-phenylacetyl-CoA
4-Maleyl-acetoacetate
Gentisate
K00450
Rosmarinate
Homovanillate
3-Methoxy-4-hydroxy-phenylethylene-glycol
3-Methoxy-4-hydroxy-mandelate
K00151
K10219
4.1.1.62
3-Maleylpyruvate
5-Carboxymethyl-2-hydroxymuconate
DDT degradation
2.3.1.14
2.3.1.-
K01800
K01801...
K01826
Hydroquinone
5-Carboxy-2-oxohept-3-enedioate
4-Hydroxyphenyl-acetylglutamic acid
4-Hydroxyphenyl-acetylglycine
4-Fumaryl-acetoacetate
3-Fumaryl-pyruvate
K05921
2, 9, 1, 1
K01557...
K16165
K20758
2-Oxohept-3-enedioate
Fumarate
2-Hydroxyhepta-2,4-dienedioate
K02509
2,4-Dihydroxyhept-2-enedioate
K02510
Acetoacetate
Pyruvate
Maleate
Succinate semialdehyde
K00135
Succinate
Citrate cycle
Pyruvate metabolism
Butanoate metabolism
00350 2/8/18
(c) Kanehisa Laboratories

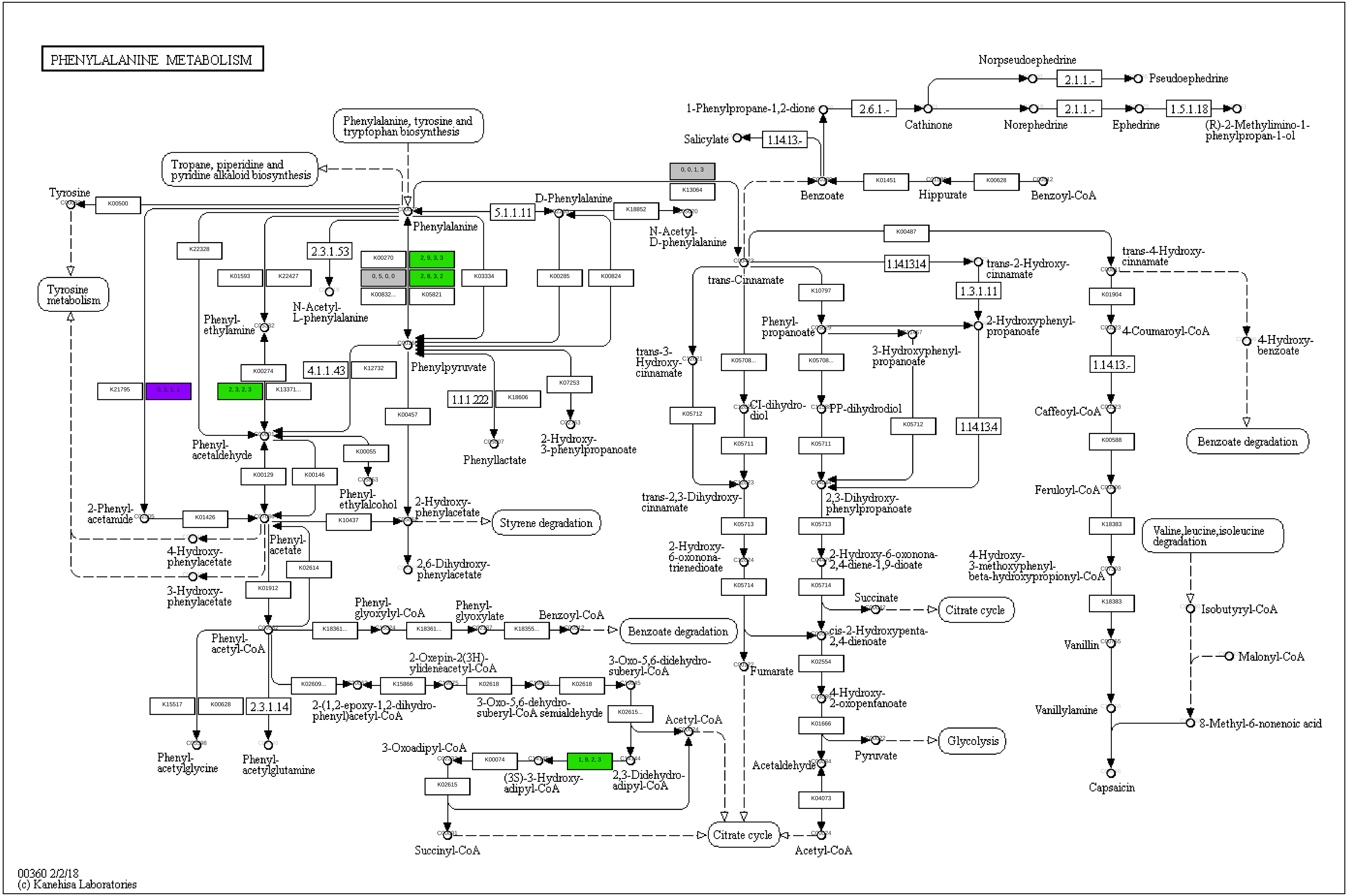
PHENYLALANINE METABOLISM
Phenylalanine, tyrosine and tryptophan biosynthesis
Tropane, piperidine and pyridine alkaloid biosynthesis
Tyrosine
K00500
Tyrosine metabolism
Phenylalanine
5.1.1.11
D-Phenylalanine
K18852
N-Acetyl-D-phenylalanine
0, 0, 1, 3
K13064
K22328
2.3.1.53
N-Acetyl-L-phenylalanine
K00270
2, 9, 3, 3
0, 5, 0, 0
2, 8, 3, 2
K00832...
K05821
K03334
K00285
K00824
K01593
K22427
Phenyl-ethylamine
Phenylpyruvate
4.1.1.43
K12732
K00274
K21795
0, 3, 1, 1
2, 3, 2, 3
K13371...
1.1.1.222
K18606
K07253
Phenyllactate
2-Hydroxy-3-phenylpropanoate
K00457
Phenyl-acetaldehyde
K00055
Phenyl-ethylalcohol
K00129
K00146
2-Phenyl-acetamide
K01426
K10437
2-Hydroxy-phenylacetate
Styrene degradation
4-Hydroxy-phenylacetate
Phenyl-acetate
2,6-Dihydroxy-phenylacetate
3-Hydroxy-phenylacetate
K02614
K01912
Phenyl-acetyl-CoA
K18361...
Phenyl-glyoxylyl-CoA
Phenyl-glyoxylate
K18355...
Benzoyl-CoA
Benzoate degradation
2-Oxepin-2(3H)-ylideneacetyl-CoA
3-Oxo-5,6-didehydro-suberyl-CoA
K02609...
K15866
K02618
2-(1,2-epoxy-1,2-dihydro-phenyl)acetyl-CoA
3-Oxo-5,6-dehydro-suberyl-CoA semialdehyde
K02615...
Acetyl-CoA
K15517
K00628
2.3.1.14
Phenyl-acetylglycine
Phenyl-acetylglutamine
3-Oxoadipyl-CoA
K00074
1, 9, 2, 3
(3S)-3-Hydroxy-adipyl-CoA
2,3-Didehydro-adipyl-CoA
K02615
Succinyl-CoA
Citrate cycle
Norpseudoephedrine
2.1.1.-
Pseudoephedrine
1-Phenylpropane-1,2-dione
2.6.1.-
Cathinone
Norephedrine
Ephedrine
1.5.1.18
(R)-2-Methylimino-1-phenylpropan-1-ol
Salicylate
1.14.13.-
Benzoate
K01451
Hippurate
K00628
Benzoyl-CoA
K00487
trans-Cinnamate
1.14.13.14
trans-2-Hydroxy-cinnamate
trans-4-Hydroxy-cinnamate
1.3.1.11
K10797
Phenyl-propanoate
2-Hydroxyphenyl-propanoate
3-Hydroxyphenyl-propanoate
K01904
4-Coumaroyl-CoA
4-Hydroxy-benzoate
1.14.13.-
trans-3-Hydroxy-cinnamate
K05708...
CI-dihydro-diol
PP-dihydrodiol
K05712
1.14.13.4
Caffeoyl-CoA
Benzoate degradation
K05711
K00588
trans-2,3-Dihydroxy-cinnamate
2,3-Dihydroxy-phenylpropanoate
Feruloyl-CoA
K05713
K18383
Valine, leucine, isoleucine degradation
2-Hydroxy-6-oxonona-trienedioate
2-Hydroxy-6-oxonona-2,4-diene-1,9-dioate
4-Hydroxy-3-methoxyphenyl-beta-hydroxypropionyl-CoA
K05714
Succinate
Citrate cycle
Isobutyryl-CoA
cis-2-Hydroxypenta-2,4-dienoate
Vanillin
Malonyl-CoA
K02554
Fumarate
4-Hydroxy-2-oxopentanoate
Vanillylamine
8-Methyl-6-nonenoic acid
K01666
Pyruvate
Glycolysis
Acetaldehyde
Capsaicin
K04073
Acetyl-CoA
00360 2/2/18
(c) Kanehisa Laboratories

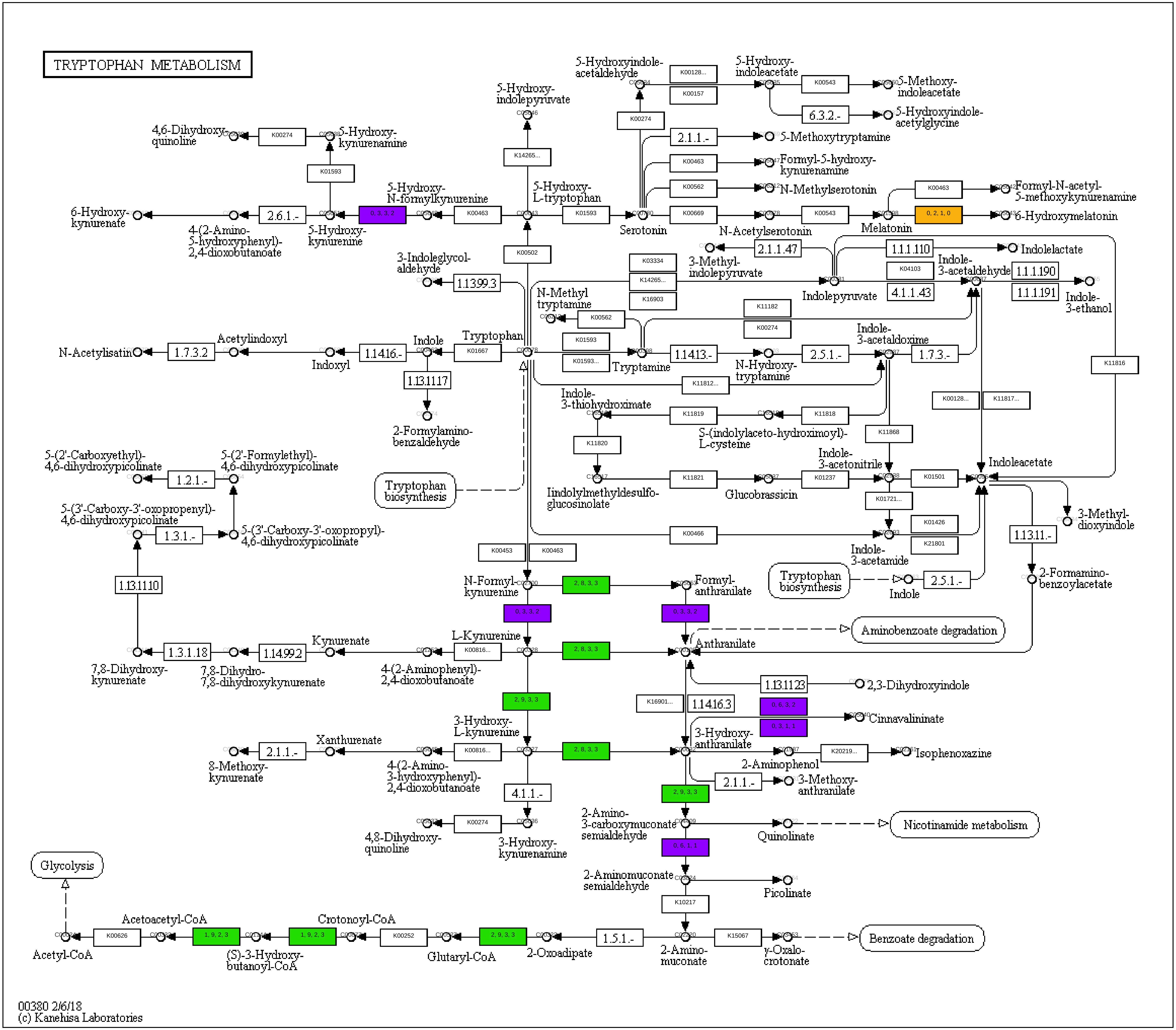
TRYPTOPHAN METABOLISM
5-Hydroxyindole-acetaldehyde
5-Hydroxy-indoleacetate
5-Methoxy-indoleacetate
5-Hydroxyindole-acetylglycine
5-Hydroxy-indolepyruvate
4,6-Dihydroxy-quinoline
5-Hydroxy-kynurenamine
5-Methoxytryptamine
Formyl-5-hydroxy-kynurenamine
N-Methylserotonin
Formyl-N-acetyl-5-methoxykynurenamine
6-Hydroxymelatonin
6-Hydroxy-kynurenate
4-(2-Amino-5-hydroxyphenyl)-2,4-dioxobutanoate
5-Hydroxy-kynurenine
5-Hydroxy-N-formylkynurenine
5-Hydroxy-L-tryptophan
Serotonin
N-Acetylserotonin
Melatonin
3-Indoleglycol-aldehyde
3-Methyl-indolepyruvate
Indolelactate
Indole-3-acetaldehyde
Indolepyruvate
Indole-3-ethanol
N-Methyl tryptamine
N-Acetylisatin
Acetylindoxyl
Indoxyl
Indole
Tryptophan
Tryptamine
N-Hydroxy-tryptamine
Indole-3-acetaldoxime
2-Formylamino-benzaldehyde
Indole-3-thiohydroximate
S-(indolylaceto-hydroximoyl)-L-cysteine
Indole-3-acetonitrile
Indoleacetate
5-(2'-Carboxyethyl)-4,6-dihydroxypicolinate
5-(2'-Formylethyl)-4,6-dihydroxypicolinate
Tryptophan biosynthesis
Iindolylmethyldesulfo-glucosinolate
Glucobrassicin
3-Methyl-dioxyindole
5-(3'-Carboxy-3'-oxopropenyl)-4,6-dihydroxypicolinate
5-(3'-Carboxy-3'-oxopropyl)-4,6-dihydroxypicolinate
Indole-3-acetamide
N-Formyl-kynurenine
Formyl-anthranilate
Indole
2-Formamino-benzoylacetate
Aminobenzoate degradation
L-Kynurenine
Kynurenate
Anthranilate
7,8-Dihydroxy-kynurenate
7,8-Dihydro-7,8-dihydroxykynurenate
4-(2-Aminophenyl)-2,4-dioxobutanoate
2,3-Dihydroxyindole
Cinnavalininate
3-Hydroxy-L-kynurenine
Xanthurenate
3-Hydroxy-anthranilate
Isophenoxazine
2-Aminophenol
8-Methoxy-kynurenate
4-(2-Amino-3-hydroxyphenyl)-2,4-dioxobutanoate
3-Methoxy-anthranilate
2-Amino-3-carboxymuconate semialdehyde
Quinolinate
Nicotinamide metabolism
4,8-Dihydroxy-quinoline
3-Hydroxy-kynurenamine
Glycolysis
2-Aminomuconate semialdehyde
Picolinate
Acetoacetyl-CoA
Crotonoyl-CoA
Acetyl-CoA
(S)-3-Hydroxy-butanoyl-CoA
Glutaryl-CoA
2-Oxoadipate
2-Amino-muconate
γ-Oxalo-crotonate
Benzoate degradation
00380 2/6/18
(c) Kanehisa Laboratories

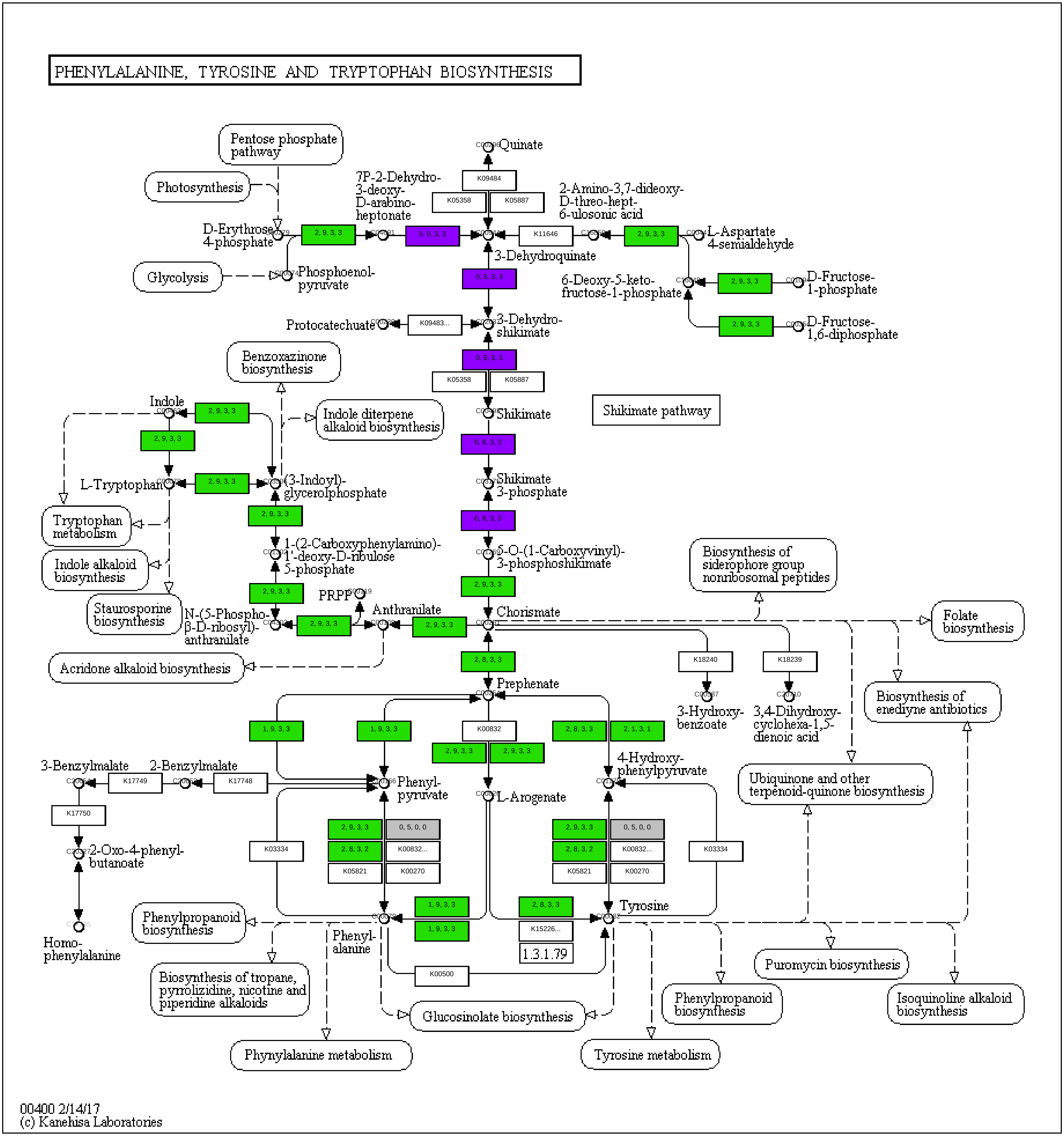

PHENYLALANINE, TYROSINE AND TRYPTOPHAN BIOSYNTHESIS
Pentose phosphate pathway
Photosynthesis
Glycolysis
D-Erythrose 4-phosphate
Phosphoenol-pyruvate
7P-2-Dehydro-3-deoxy-D-arabino-heptonate
Quinate
K09484
K05358
K05887
2-Amino-3,7-dideoxy-D-threo-hept-6-ulosonic acid
K11646
L-Aspartate 4-semialdehyde
3-Dehydroquinate
6-Deoxy-5-keto-fructose-1-phosphate
D-Fructose-1-phosphate
D-Fructose-1,6-diphosphate
Protocatechuate
K09483...
3-Dehydro-shikimate
Benzoxazinone biosynthesis
Indole diterpene alkaloid biosynthesis
Indole
Shikimate
Shikimate pathway
L-Tryptophan
(3-Indoyl)-glycerolphosphate
Shikimate 3-phosphate
Tryptophan metabolism
Indole alkaloid biosynthesis
1-(2-Carboxyphenylamino)-1'-deoxy-D-ribulose 5-phosphate
5-O-(1-Carboxyvinyl)-3-phosphoshikimate
Biosynthesis of siderophore group nonribosomal peptides
Staurosporine biosynthesis
PRPP
N-(5-Phospho-β-D-ribosyl)-anthranilate
Anthranilate
Chorismate
Folate biosynthesis
Acridone alkaloid biosynthesis
K18240
K18239
Prephenate
3-Hydroxy-benzoate
3,4-Dihydroxy-cyclohexa-1,5-dienoic acid
Biosynthesis of enediyne antibiotics
K00832
3-Benzylmalate
2-Benzylmalate
K17749
K17748
4-Hydroxy-phenylpyruvate
Phenyl-pyruvate
L-Arogenate
Ubiquinone and other terpenoid-quinone biosynthesis
K17750
2-Oxo-4-phenyl-butanoate
K03334
K00832...
K05821
K00270
Tyrosine
Phenylpropanoid biosynthesis
K15226...
1.3.1.79
Homo-phenylalanine
Phenyl-alanine
K00500
Biosynthesis of tropane, pyrrolizidine, nicotine and piperidine alkaloids
Glucosinolate biosynthesis
Puromycin biosynthesis
Phenylpropanoid biosynthesis
Isoquinoline alkaloid biosynthesis
Phynylalanine metabolism
Tyrosine metabolism
00400 2/14/17
(c) Kanehisa Laboratories

6. Metabolism of other amino acids

MAP	PATHWAY
**410**	beta-Alanine metabolism
**430**	Taurine and hypotaurine metabolism
**440**	Phosphonate and phosphinate metabolism
**450**	Selenocompound metabolism
**460**	Cyanoamino acid metabolism
**480**	Glutathione metabolism

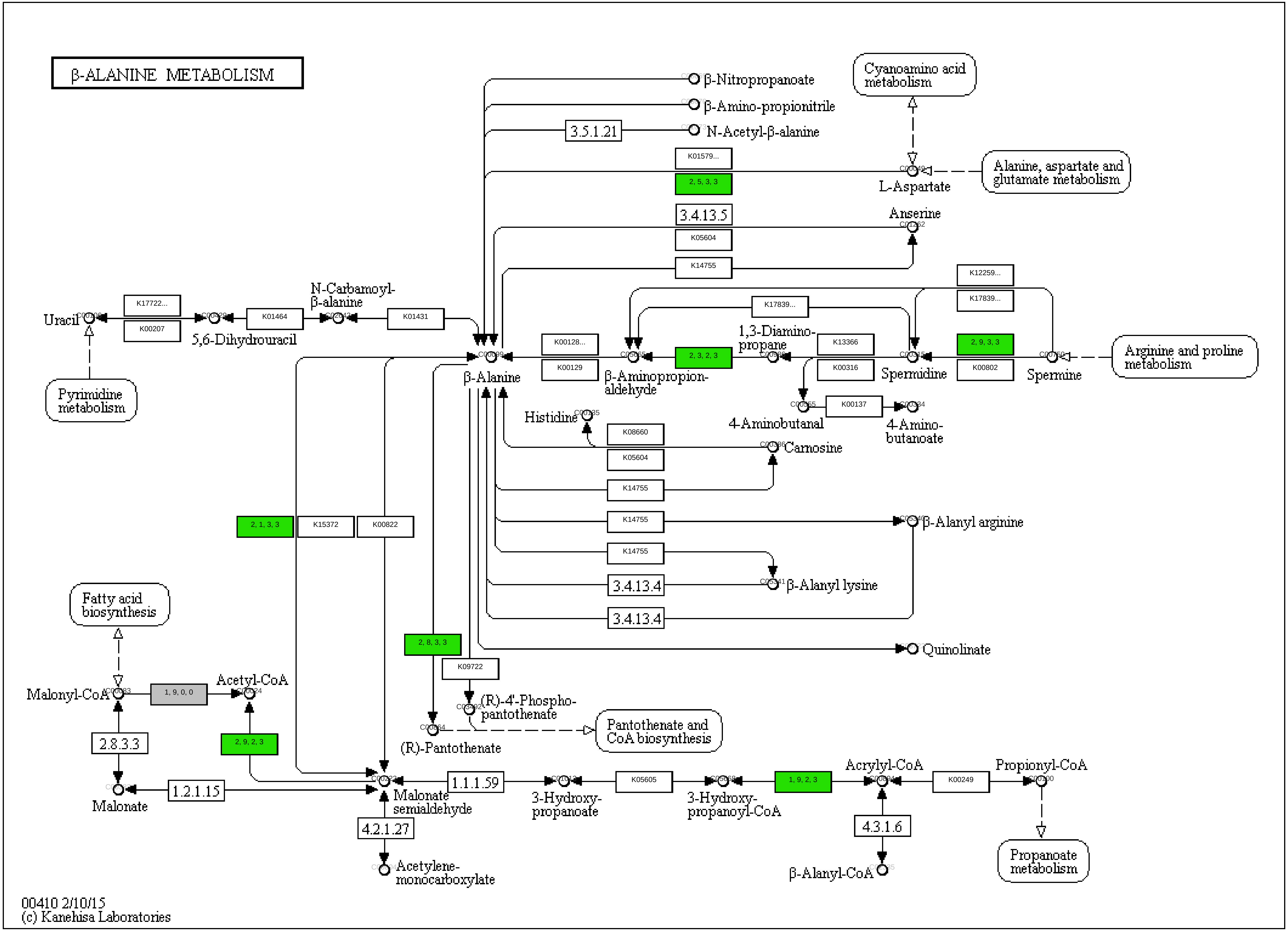
β-ALANINE METABOLISM
β-Nitropropanoate
β-Amino-propionitrile
N-Acetyl-β-alanine
3.5.1.21
Cyanoamino acid metabolism
K01579...
2, 5, 3, 3
L-Aspartate
Alanine, aspartate and glutamate metabolism
3.4.13.5
Anserine
K05604
K14755
K12259...
K17839...
K17839...
Uracil
K17722...
K00207
5,6-Dihydrouracil
K01464
N-Carbamoyl-β-alanine
K01431
K00128...
K00129
2, 3, 2, 3
1,3-Diamino-propane
K13366
K00316
2, 9, 3, 3
K00802
Arginine and proline metabolism
Pyrimidine metabolism
β-Alanine
β-Aminopropion-aldehyde
Spermidine
Spermine
4-Aminobutanal
K00137
4-Amino-butanoate
Histidine
K08660
K05604
Carnosine
K14755
K14755
β-Alanyl arginine
2, 1, 3, 3
K15372
K00822
K14755
3.4.13.4
β-Alanyl lysine
3.4.13.4
Quinolinate
Fatty acid biosynthesis
2, 8, 3, 3
K09722
Malonyl-CoA
1, 9, 0, 0
Acetyl-CoA
(R)-4'-Phospho-pantothenate
Pantothenate and CoA biosynthesis
2.8.3.3
2, 9, 2, 3
(R)-Pantothenate
Acrylyl-CoA
Propionyl-CoA
1.1.1.59
K05605
1, 9, 2, 3
K00249
Malonate
1.2.1.15
Malonate semialdehyde
3-Hydroxy-propanoate
3-Hydroxy-propanoyl-CoA
4.2.1.27
4.3.1.6
Propanoate metabolism
Acetylene-monocarboxylate
β-Alanyl-CoA
00410 2/10/15
(c) Kanehisa Laboratories

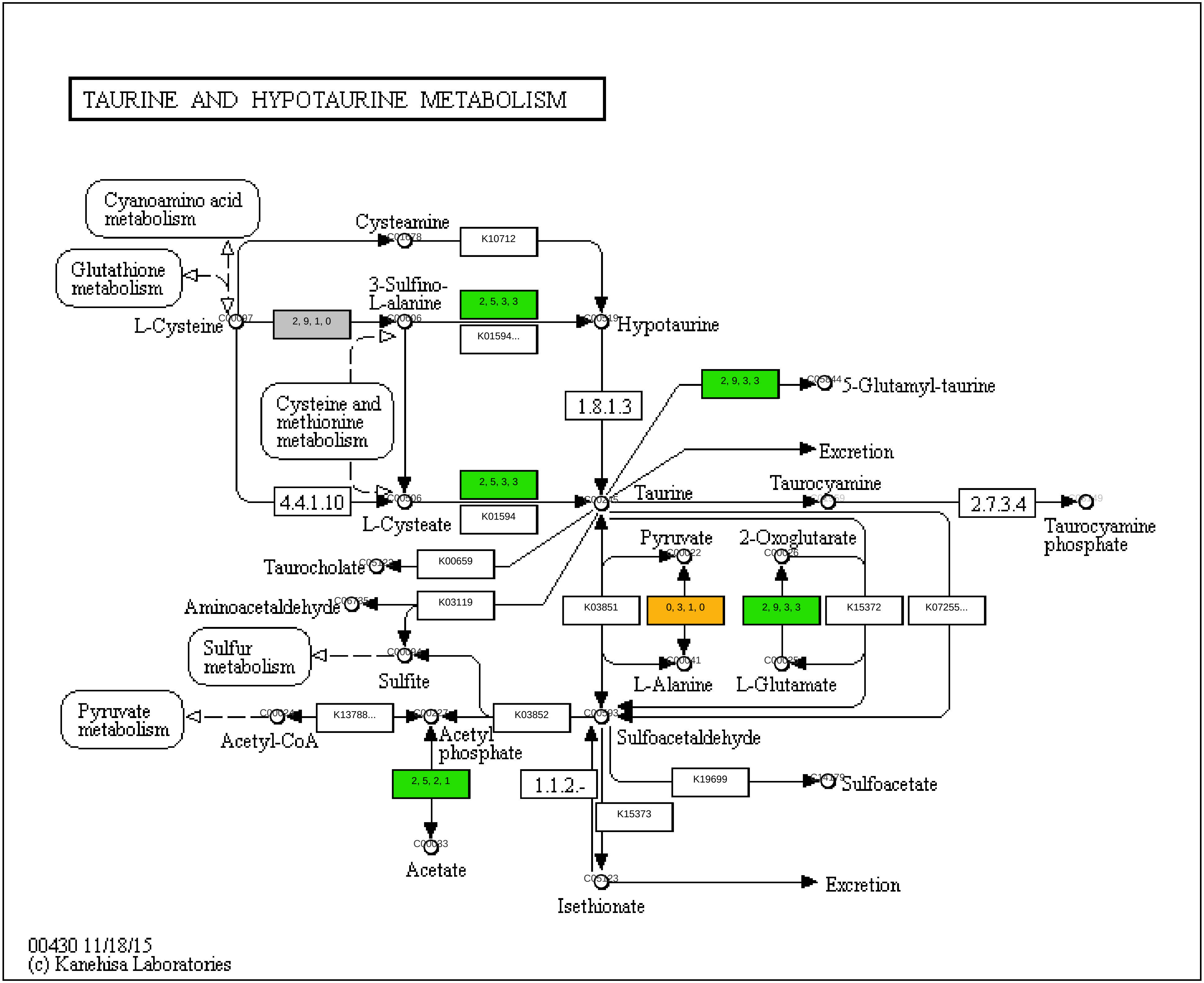
TAURINE AND HYPOTAURINE METABOLISM
Cyanoamino acid metabolism
Glutathione metabolism
Cysteamine
K10712
3-Sulfino-L-alanine
2, 5, 3, 3
K01594...
L-Cysteine
2, 9, 1, 0
Hypotaurine
Cysteine and methionine metabolism
1.8.1.3
2, 9, 3, 3
5-Glutamyl-taurine
Excretion
4.4.1.10
2, 5, 3, 3
K01594
L-Cysteate
Taurine
Taurocyamine
2.7.3.4
Taurocyamine phosphate
Pyruvate
2-Oxoglutarate
Taurocholate
K00659
Aminoacetaldehyde
K03119
K03851
0, 3, 1, 0
2, 9, 3, 3
K15372
K07255...
Sulfur metabolism
Sulfite
L-Alanine
L-Glutamate
Pyruvate metabolism
K13788...
K03852
Sulfoacetaldehyde
Acetyl-CoA
Acetyl phosphate
2, 5, 2, 1
1.1.2.-
K19699
Sulfoacetate
K15373
Acetate
Excretion
Isethionate
00430 11/18/15
(c) Kanehisa Laboratories

PHOSPHONATE AND PHOSPHINATE METABOLISM

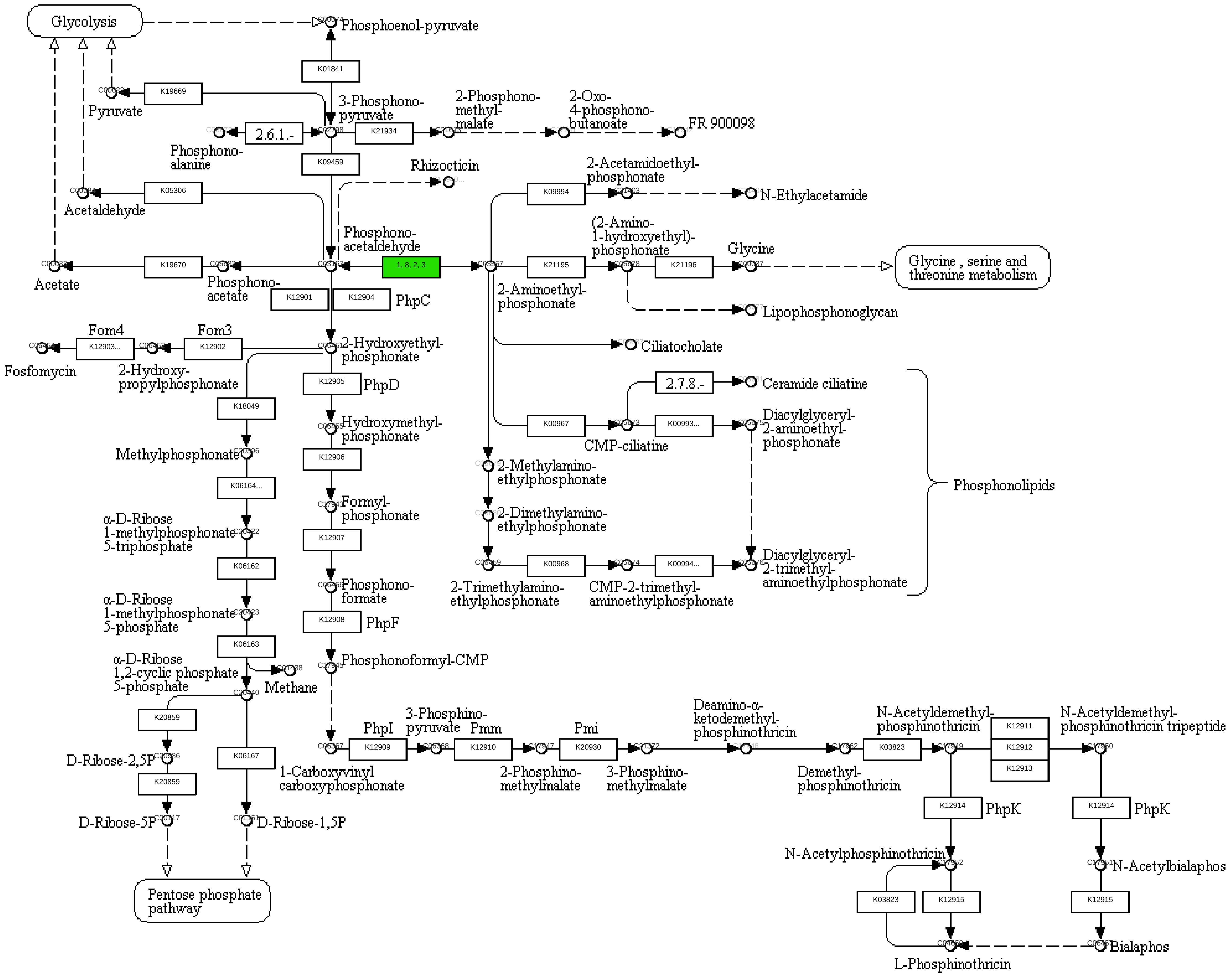

00440 1/19/18
(c) Kanehisa Laboratories

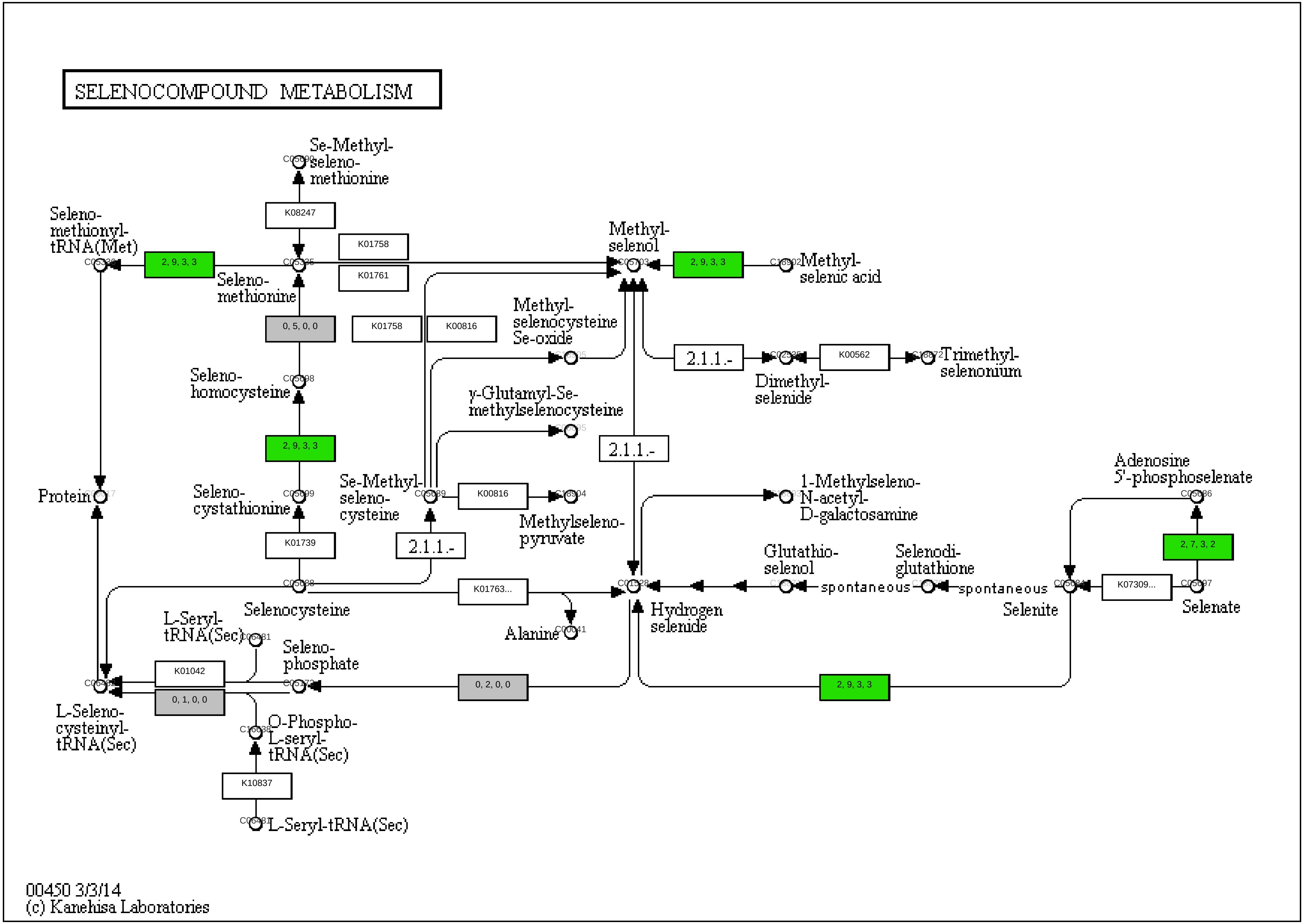
SELENOCOMPOUND METABOLISM
Se-Methyl-seleno-methionine
K08247
Seleno-methionyl-tRNA(Met)
2, 9, 3, 3
K01758
K01761
Seleno-methionine
0, 5, 0, 0
K01758
K00816
Methyl-selenol
2, 9, 3, 3
Methyl-selenic acid
Methyl-selenocysteine Se-oxide
2.1.1.-
K00562
Trimethyl-selenonium
Dimethyl-selenide
Seleno-homocysteine
γ-Glutamyl-Se-methylselenocysteine
2, 9, 3, 3
2.1.1.-
Adenosine 5'-phosphoselenate
Protein
Seleno-cystathionine
Se-Methyl-seleno-cysteine
K00816
Methylseleno-pyruvate
1-Methylseleno-N-acetyl-D-galactosamine
2, 7, 3, 2
K01739
2.1.1.-
Glutathio-selenol
Selenodi-glutathione
K01763...
spontaneous
spontaneous
K07309...
Selenocysteine
Hydrogen selenide
Selenite
Selenate
L-Seryl-tRNA(Sec)
Alanine
Seleno-phosphate
K01042
0, 1, 0, 0
0, 2, 0, 0
2, 9, 3, 3
L-Seleno-cysteinyl-tRNA(Sec)
O-Phospho-L-seryl-tRNA(Sec)
K10837
L-Seryl-tRNA(Sec)
00450 3/3/14
(c) Kanehisa Laboratories

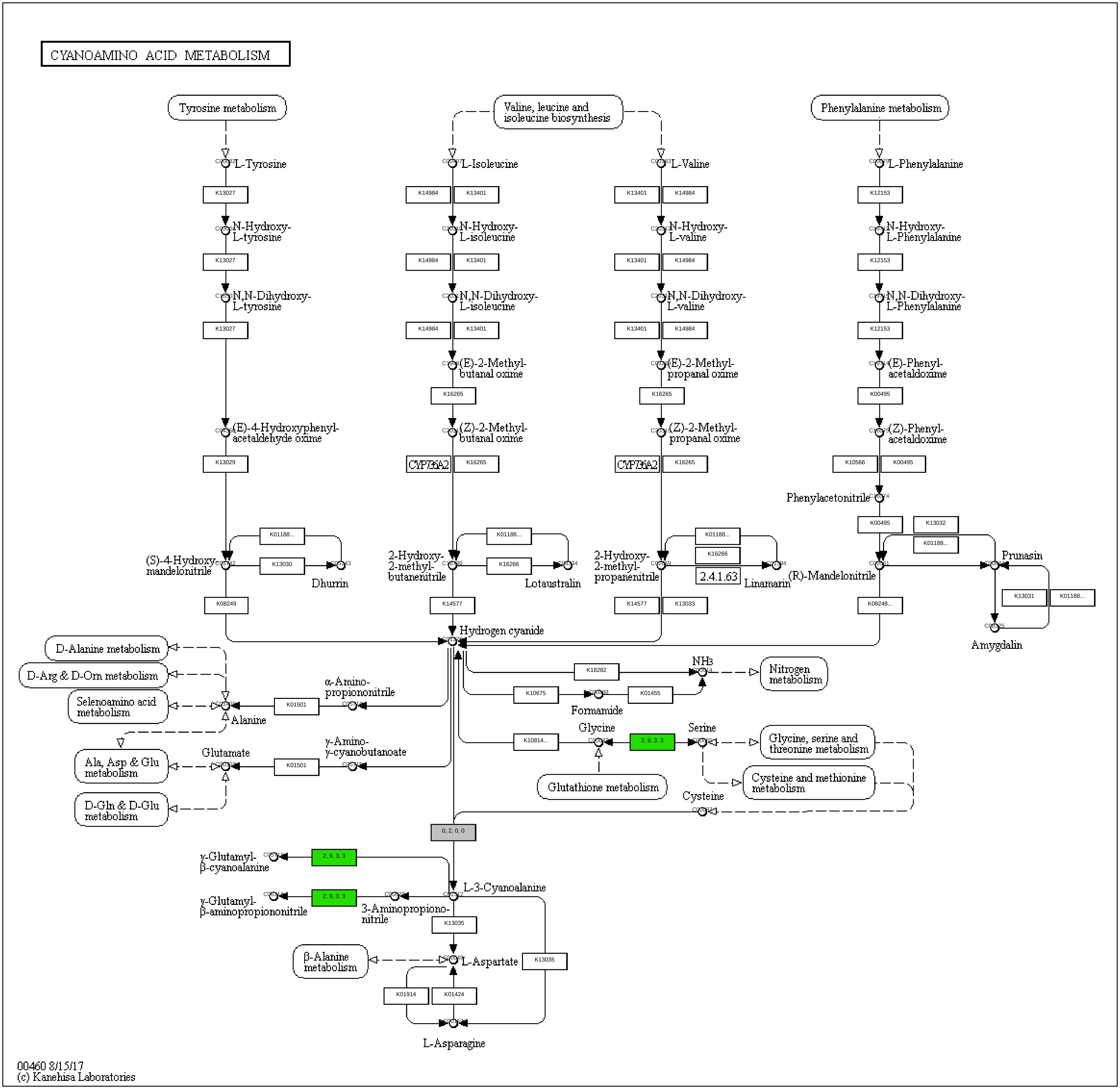
CYANOAMINO ACID METABOLISM
Tyrosine metabolism
Valine, leucine and isoleucine biosynthesis
Phenylalanine metabolism
L-Tyrosine
L-Isoleucine
L-Valine
L-Phenylalanine
K13027
K14984
K13401
K12153
N-Hydroxy-L-tyrosine
N-Hydroxy-L-isoleucine
N-Hydroxy-L-valine
N-Hydroxy-L-Phenylalanine
N,N-Dihydroxy-L-tyrosine
N,N-Dihydroxy-L-isoleucine
N,N-Dihydroxy-L-valine
N,N-Dihydroxy-L-Phenylalanine
(E)-2-Methyl-butanal oxime
(E)-2-Methyl-propanal oxime
(E)-Phenyl-acetaldoxime
K16265
K00495
(E)-4-Hydroxyphenyl-acetaldehyde oxime
(Z)-2-Methyl-butanal oxime
(Z)-2-Methyl-propanal oxime
(Z)-Phenyl-acetaldoxime
K13029
CYP736A2
K10566
Phenylacetonitrile
K13032
K01188...
K13030
K16266
2.4.1.63
(S)-4-Hydroxy-mandelonitrile
Dhurrin
2-Hydroxy-2-methyl-butanenitrile
Lotaustralin
2-Hydroxy-2-methyl-propanenitrile
Linamarin
(R)-Mandelonitrile
Prunasin
K13031
Amygdalin
K08249
K14577
K13033
K08248...
Hydrogen cyanide
D-Alanine metabolism
D-Arg & D-Orn metabolism
Selenoamino acid metabolism
Alanine
α-Amino-propiononitrile
K01501
K18282
NH3
Nitrogen metabolism
K10675
K01455
Formamide
Glycine
Serine
K10814...
2, 9, 3, 3
Glycine, serine and threonine metabolism
Cysteine and methionine metabolism
Glutathione metabolism
Cysteine
Ala, Asp & Glu metabolism
Glutamate
γ-Amino-γ-cyanobutanoate
D-Gln & D-Glu metabolism
0, 2, 0, 0
γ-Glutamyl-β-cyanoalanine
L-3-Cyanoalanine
γ-Glutamyl-β-aminopropiononitrile
3-Aminopropiono-nitrile
K13035
β-Alanine metabolism
L-Aspartate
K01914
K01424
L-Asparagine
00460 8/15/17
(c) Kanehisa Laboratories

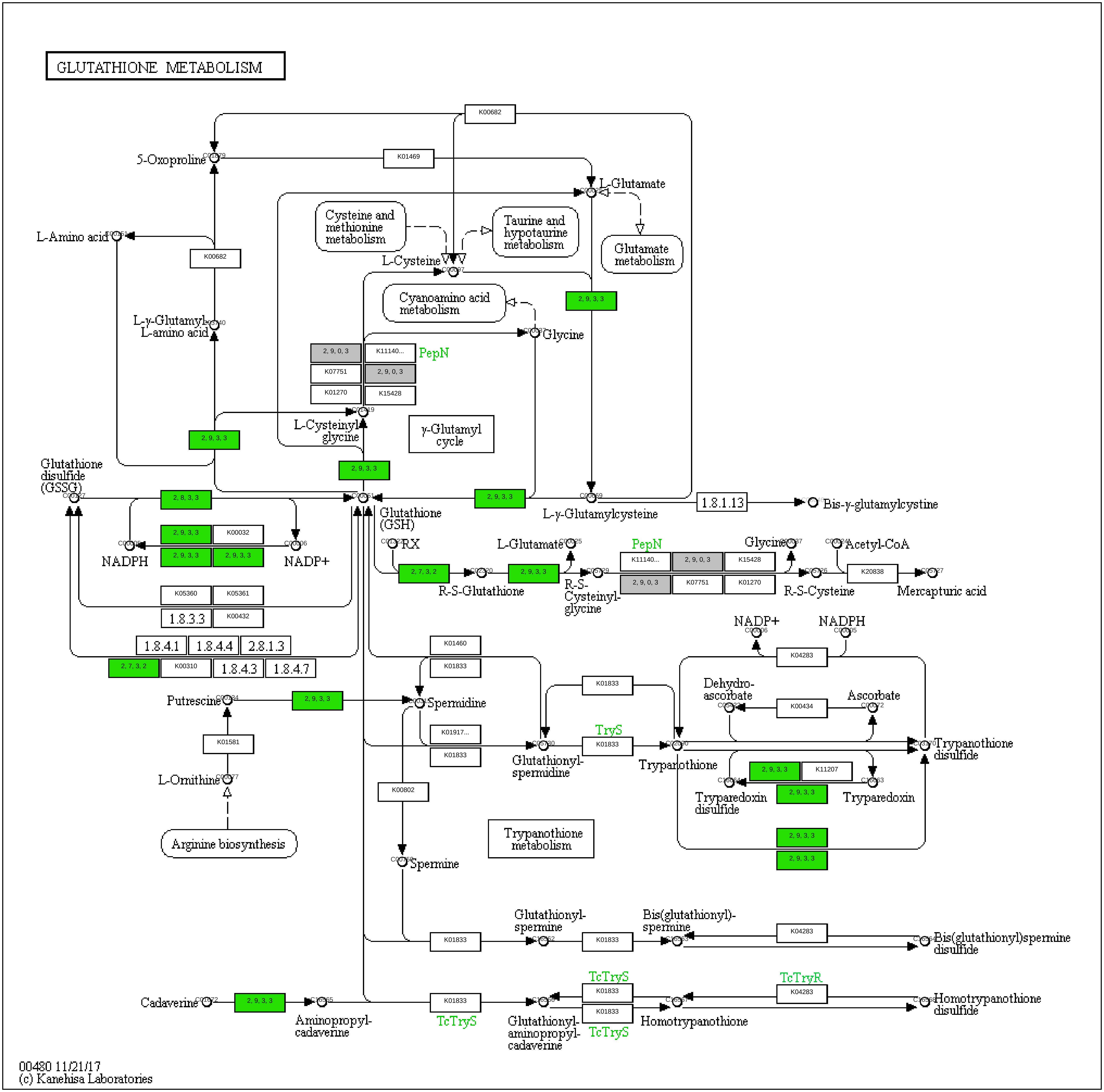
GLUTATHIONE METABOLISM
K00682
5-Oxoproline
K01469
L-Glutamate
Cysteine and methionine metabolism
Taurine and hypotaurine metabolism
Glutamate metabolism
L-Amino acid
K00682
L-Cysteine
Cyanoamino acid metabolism
2, 9, 3, 3
L-γ-Glutamyl-L-amino acid
Glycine
2, 9, 0, 3
K11140...
PepN
K07751
2, 9, 0, 3
K01270
K15428
L-Cysteinyl-glycine
γ-Glutamyl cycle
2, 9, 3, 3
2, 9, 3, 3
Glutathione disulfide (GSSG)
2, 8, 3, 3
2, 9, 3, 3
Glutathione (GSH)
L-γ-Glutamylcysteine
1.8.1.13
Bis-γ-glutamylcystine
2, 9, 3, 3
K00032
RX
L-Glutamate
PepN
Glycine
Acetyl-CoA
NADPH
2, 9, 3, 3
2, 9, 3, 3
NADP+
2, 7, 3, 2
2, 9, 3, 3
K11140...
2, 9, 0, 3
K15428
K20838
R-S-Glutathione
R-S-Cysteinyl-glycine
2, 9, 0, 3
K07751
K01270
R-S-Cysteine
Mercapturic acid
K05360
K05361
1.8.3.3
K00432
1.8.4.1
1.8.4.4
2.8.1.3
2, 7, 3, 2
K00310
1.8.4.3
1.8.4.7
K01460
K01833
NADP+
NADPH
K04283
Putrescine
2, 9, 3, 3
Spermidine
K01833
Dehydro-ascorbate
K00434
Ascorbate
K01581
K01917...
TryS
K01833
K01833
Glutathionyl-spermidine
Trypanothione
Trypanothione disulfide
L-Ornithine
2, 9, 3, 3
K11207
K00802
2, 9, 3, 3
Tryparedoxin disulfide
Tryparedoxin
Arginine biosynthesis
Trypanothione metabolism
2, 9, 3, 3
2, 9, 3, 3
Spermine
Glutathionyl-spermine
Bis(glutathionyl)-spermine
K01833
K01833
K04283
Bis(glutathionyl)spermine disulfide
TcTryS
TcTryR
K01833
K04283
Cadaverine
2, 9, 3, 3
K01833
K01833
Homotrypanothione disulfide
Aminopropyl-cadaverine
TcTryS
Glutathionyl-aminopropyl-cadaverine
TcTryS
Homotrypanothione
00480 11/21/17
(c) Kanehisa Laboratories

7. Glycan biosynthesis and metabolism

MAP	PATHWAY
**510**	N-Glycan biosynthesis
**513**	Various types of N-glycan biosynthesis
**531**	Glycosaminoglycan degradation
**563**	Glycosylphosphatidylinositol (GPI)-anchor biosynthesis

N-GLYCAN BIOSYNTHESIS

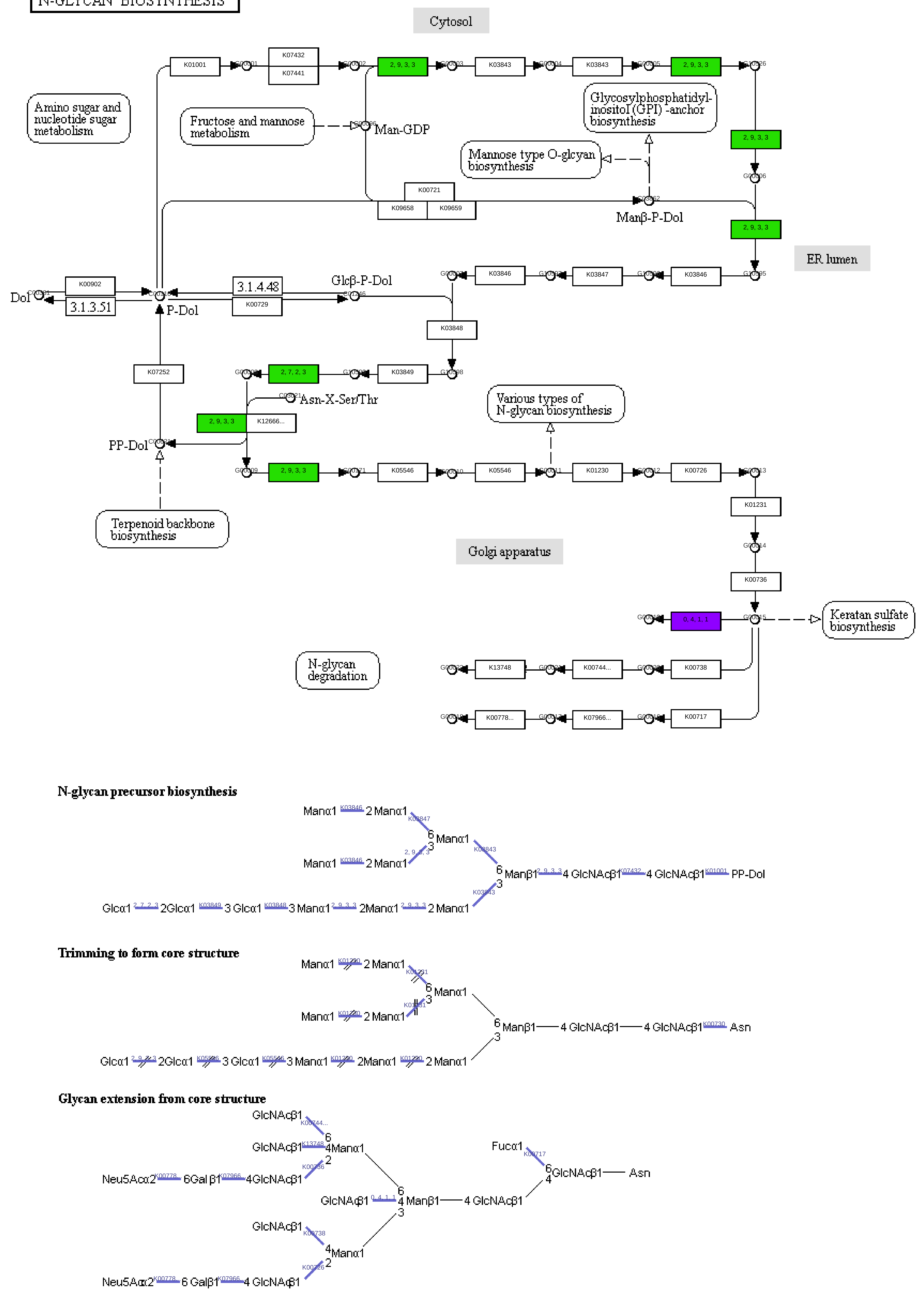

00510 12/8/16
(c) Kanehisa Laboratories

VARIOUS TYPES OF N-GLYCAN BIOSYNTHESIS

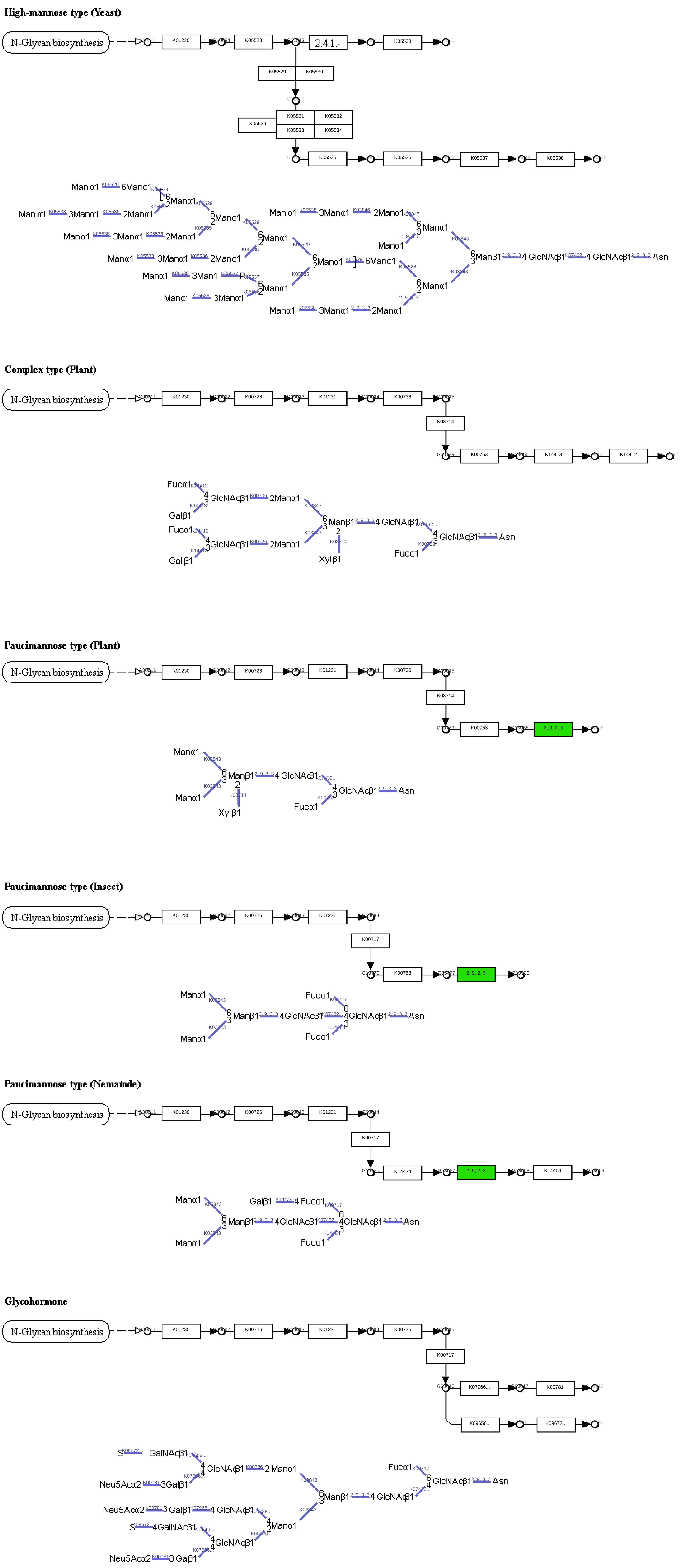

00513 9/7/16
(c) Kanehisa Laboratories

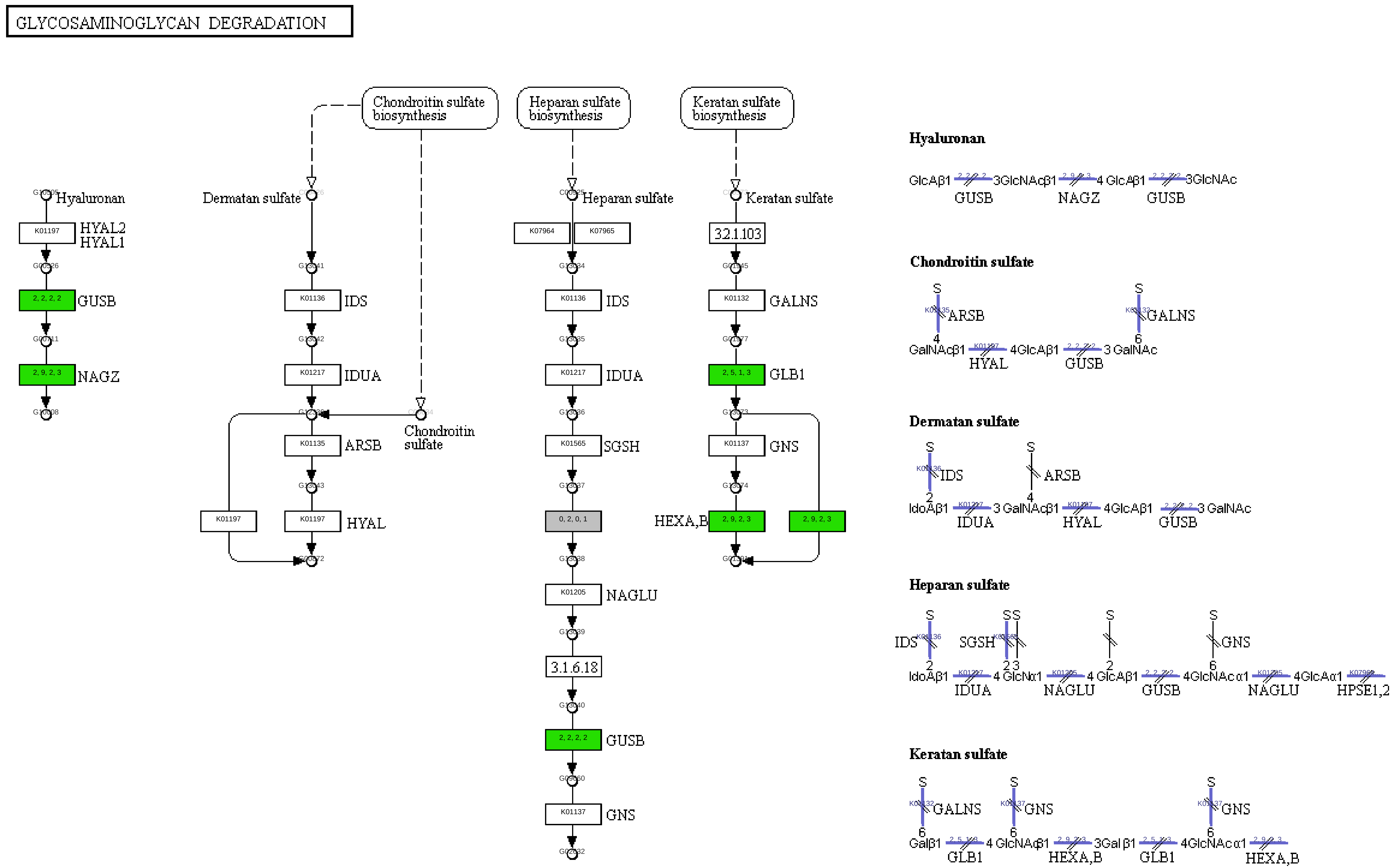

00531 5/22/15
(c) Kanehisa Laboratories

GLYCOSYLPHOSPHATIDYLINOSITOL (GPI) - ANCHOR BIOSYNTHESIS

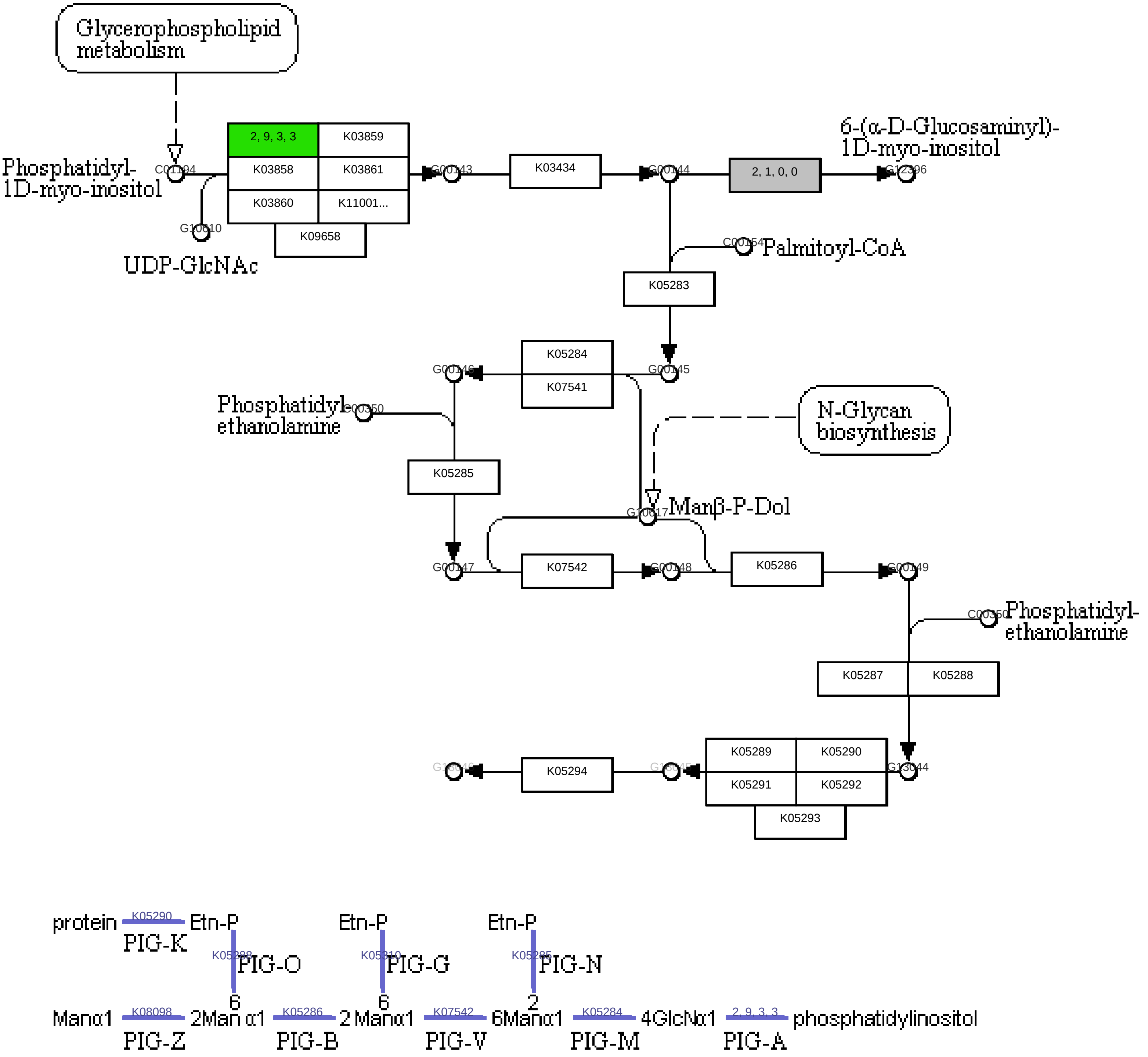

00563 12/27/16

(c) Kanehisa Laboratories

8. Metabolism of cofactors and vitamins

MAP	PATHWAY
**130**	Ubiquinone and other terpenoid-quinone biosynthesis
**670**	One carbon pool by folate
**730**	Thiamine metabolism
**740**	Riboflavin metabolism
**750**	Vitamin B6 metabolism
**760**	Nicotinate and nicotinamide metabolism
**770**	Pantothenate and CoA biosynthesis
**780**	Biotin metabolism
**785**	Lipoic acid metabolism
**790**	Folate biosynthesis
**860**	Porphyrin and chlorophyll metabolism

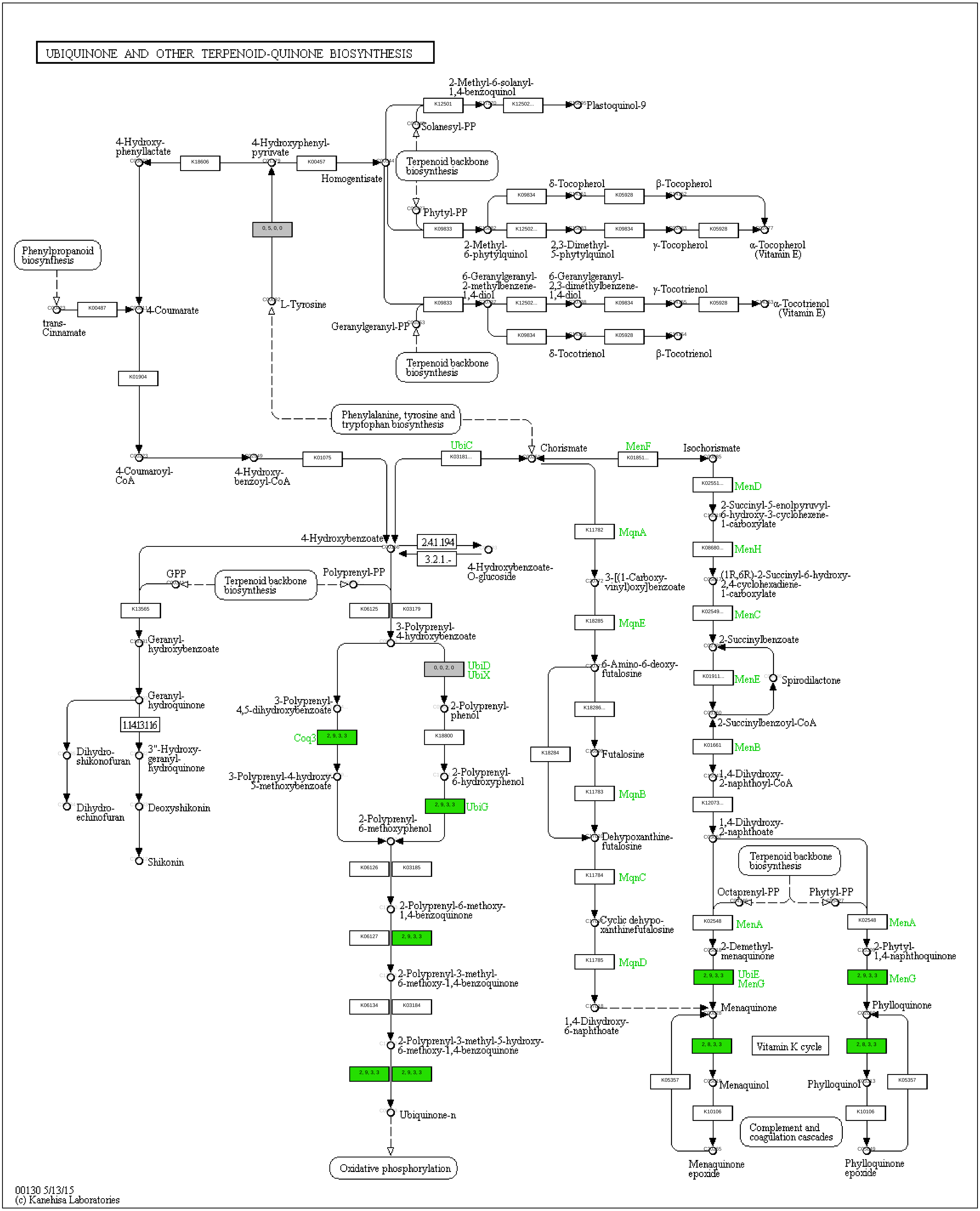
UBIQUINONE AND OTHER TERPENOID-QUINONE BIOSYNTHESIS
2-Methyl-6-solanyl-1,4-benzoquinol
K12501
K12502...
Plastoquinol-9
Solanesyl-PP
4-Hydroxy-phenyllactate
K18606
4-Hydroxyphenyl-pyruvate
K00457
Homogentisate
Terpenoid backbone biosynthesis
δ-Tocopherol
β-Tocopherol
K09834
K05928
Phytyl-PP
K09833
K12502...
K09834
K05928
2-Methyl-6-phytylquinol
2,3-Dimethyl-5-phytylquinol
γ-Tocopherol
α-Tocopherol (Vitamin E)
0, 5, 0, 0
Phenylpropanoid biosynthesis
6-Geranylgeranyl-2-methylbenzene-1,4-diol
6-Geranylgeranyl-2,3-dimethylbenzene-1,4-diol
γ-Tocotrienol
K00487
4-Coumarate
L-Tyrosine
K09833
K12502...
K09834
K05928
α-Tocotrienol (Vitamin E)
trans-Cinnamate
Geranylgeranyl-PP
K09834
K05928
δ-Tocotrienol
β-Tocotrienol
Terpenoid backbone biosynthesis
K01904
Phenylalanine, tyrosine and tryptophan biosynthesis
UbiC
K03181...
Chorismate
MenF
K01851...
Isochorismate
4-Coumaroyl-CoA
4-Hydroxy-benzoyl-CoA
K01075
K02551...
MenD
2-Succinyl-5-enolpyruvyl-6-hydroxy-3-cyclohexene-1-carboxylate
K11782
MqnA
4-Hydroxybenzoate
2.4.1.194
3.2.1.-
4-Hydroxybenzoate-O-glucoside
K08680...
MenH
GPP
Terpenoid backbone biosynthesis
Polyprenyl-PP
3-[(1-Carboxy-vinyl)oxy]benzoate
(1R,6R)-2-Succinyl-6-hydroxy-2,4-cyclohexadiene-1-carboxylate
K13565
K06125
K03179
K18285
MqnE
K02549...
MenC
3-Polyprenyl-4-hydroxybenzoate
2-Succinylbenzoate
Geranyl-hydroxybenzoate
0, 0, 2, 0
UbiD
UbiX
6-Amino-6-deoxy-futalosine
K01911...
MenE
Spirodilactone
Geranyl-hydroquinone
3-Polyprenyl-4,5-dihydroxybenzoate
2-Polyprenyl-phenol
1.14.13.116
K18286...
2-Succinylbenzoyl-CoA
Coq3
2, 9, 3, 3
K18800
K18284
K01661
MenB
Dihydro-shikonofuran
3''-Hydroxy-geranyl-hydroquinone
Futalosine
3-Polyprenyl-4-hydroxy-5-methoxybenzoate
2-Polyprenyl-6-hydroxyphenol
1,4-Dihydroxy-2-naphthoyl-CoA
K11783
MqnB
Dihydro-echinofuran
Deoxyshikonin
2, 9, 3, 3
UbiG
K12073...
2-Polyprenyl-6-methoxyphenol
Dehypoxanthine-futalosine
1,4-Dihydroxy-2-naphthoate
Shikonin
Terpenoid backbone biosynthesis
K06126
K03185
K11784
MqnC
Octaprenyl-PP
Phytyl-PP
2-Polyprenyl-6-methoxy-1,4-benzoquinone
Cyclic dehypo-xanthinefutalosine
K02548
MenA
K02548
MenA
K06127
2, 9, 3, 3
2-Demethyl-menaquinone
2-Phytyl-1,4-naphthoquinone
2-Polyprenyl-3-methyl-6-methoxy-1,4-benzoquinone
K11785
MqnD
2, 9, 3, 3
UbiE
MenG
2, 9, 3, 3
MenG
K06134
K03184
Menaquinone
Phylloquinone
1,4-Dihydroxy-6-naphthoate
2-Polyprenyl-3-methyl-5-hydroxy-6-methoxy-1,4-benzoquinone
2, 8, 3, 3
Vitamin K cycle
2, 8, 3, 3
2, 9, 3, 3
2, 9, 3, 3
K05357
Menaquinol
Phylloquinol
K05357
Ubiquinone-n
K10106
K10106
Complement and coagulation cascades
Oxidative phosphorylation
Menaquinone epoxide
Phylloquinone epoxide
00130 5/13/15
(c) Kanehisa Laboratories

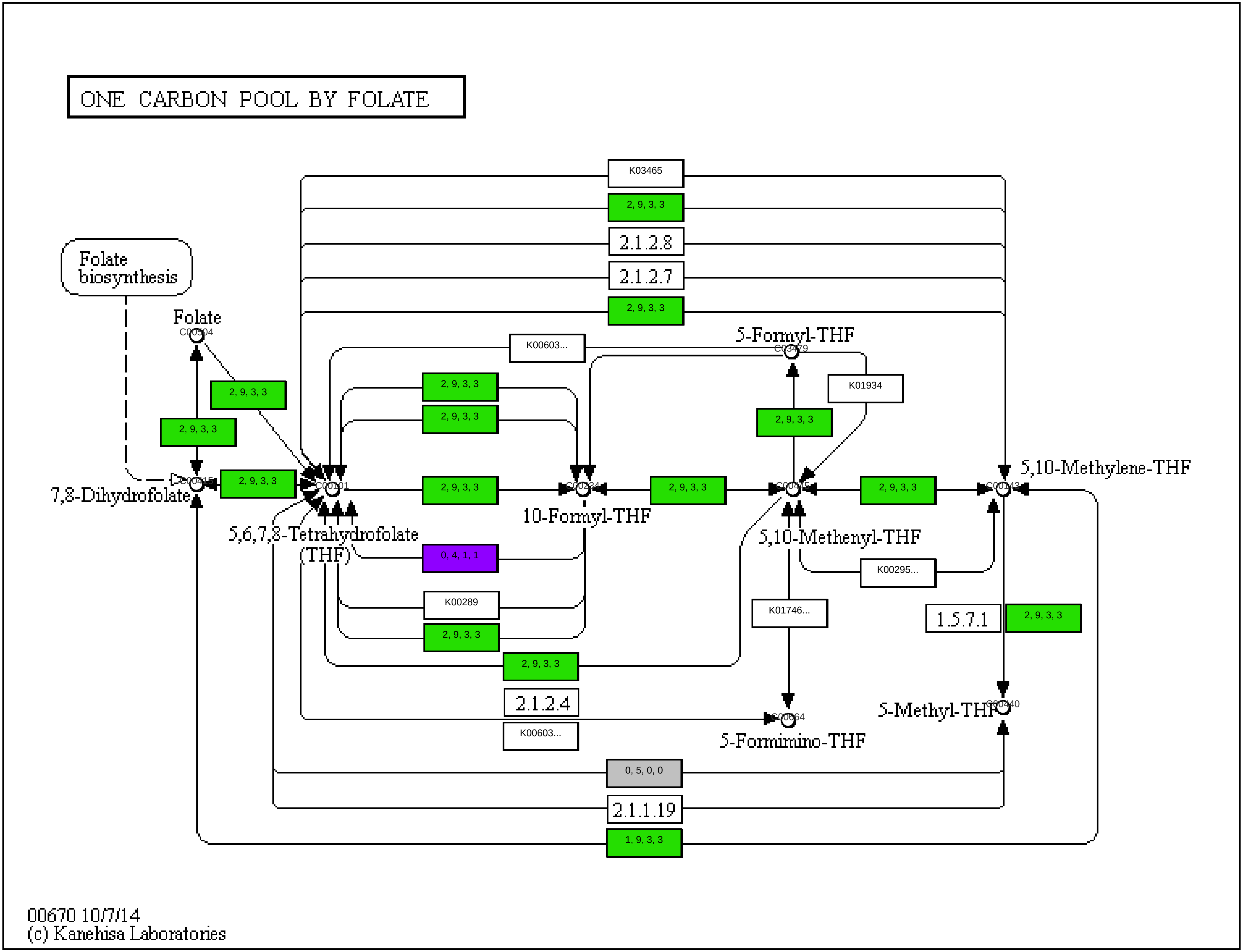
ONE CARBON POOL BY FOLATE
Folate biosynthesis
Folate
C00504
7,8-Dihydrofolate
5,6,7,8-Tetrahydrofolate (THF)
10-Formyl-THF
5-Formyl-THF
5,10-Methenyl-THF
5,10-Methylene-THF
5-Formimino-THF
5-Methyl-THF
C00440
K03465
2, 9, 3, 3
2.1.2.8
2.1.2.7
2, 9, 3, 3
K00603...
K01934
2, 9, 3, 3
2, 9, 3, 3
2, 9, 3, 3
2, 9, 3, 3
2, 9, 3, 3
2, 9, 3, 3
2, 9, 3, 3
2, 9, 3, 3
2, 9, 3, 3
0, 4, 1, 1
K00295...
K00289
K01746...
1.5.7.1
2, 9, 3, 3
2, 9, 3, 3
2, 9, 3, 3
2.1.2.4
K00603...
0, 5, 0, 0
2.1.1.19
1, 9, 3, 3
00670 10/7/14
(c) Kanehisa Laboratories

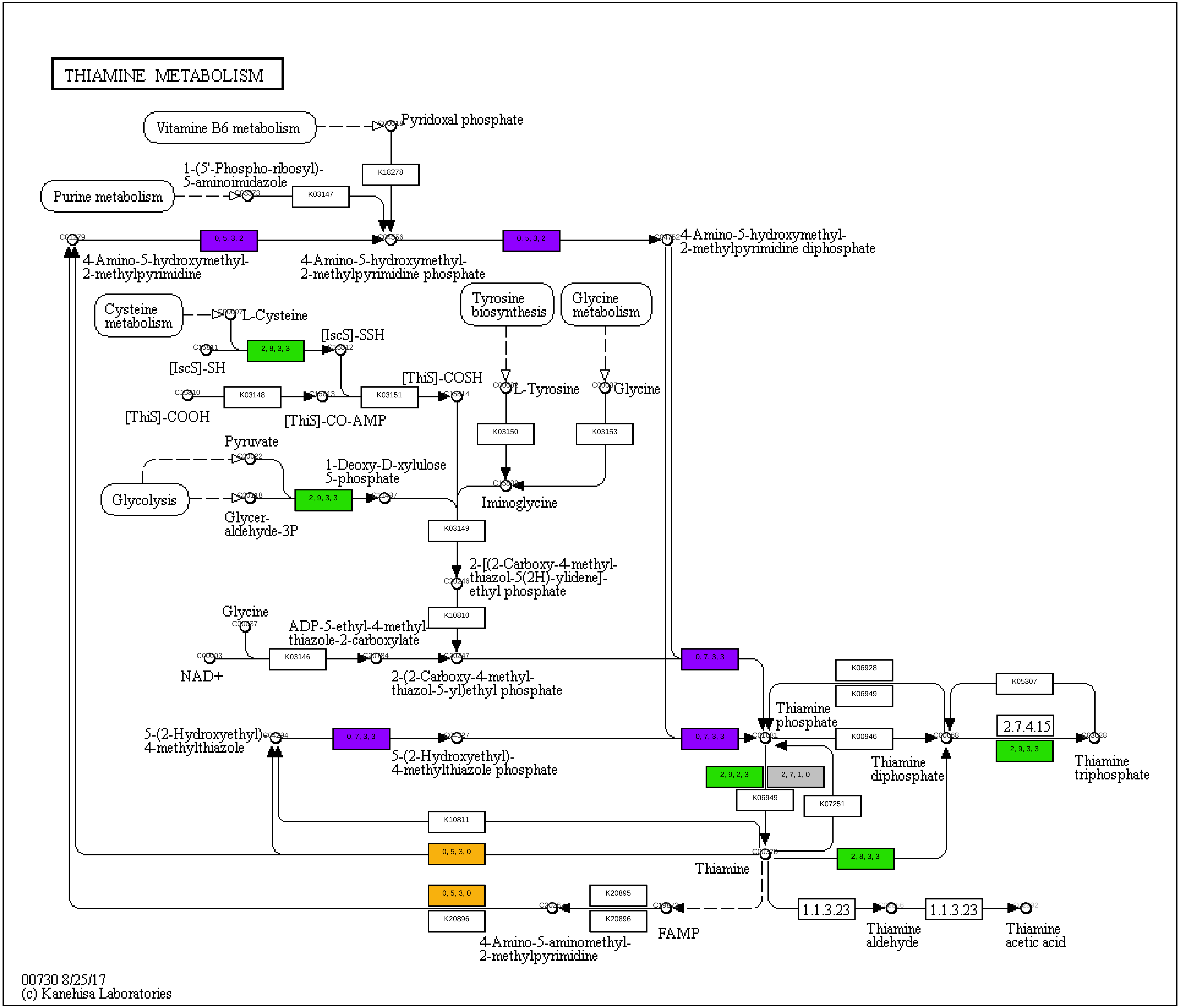

THIAMINE METABOLISM
Vitamine B6 metabolism
Pyridoxal phosphate
K18278
Purine metabolism
1-(5'-Phospho-ribosyl)-
5-aminoimidazole
K03147
4-Amino-5-hydroxymethyl-
2-methylpyrimidine
4-Amino-5-hydroxymethyl-
2-methylpyrimidine phosphate
4-Amino-5-hydroxymethyl-
2-methylpyrimidine diphosphate
0, 5, 3, 2
0, 5, 3, 2
Cysteine
metabolism
L-Cysteine
[IscS]-SSH
[IscS]-SH
2, 8, 3, 3
Tyrosine
biosynthesis
Glycine
metabolism
[ThiS]-COSH
K03148
K03151
[ThiS]-COOH
[ThiS]-CO-AMP
L-Tyrosine
Glycine
K03150
K03153
Pyruvate
1-Deoxy-D-xylulose
5-phosphate
Glycolysis
2, 9, 3, 3
Iminoglycine
Glycer-
aldehyde-3P
K03149
2-[(2-Carboxy-4-methyl-
thiazol-5(2H)-ylidene]-
ethyl phosphate
Glycine
K10810
ADP-5-ethyl-4-methyl-
thiazole-2-carboxylate
K03146
NAD+
2-(2-Carboxy-4-methyl-
thiazol-5-yl)ethyl phosphate
0, 7, 3, 3
K06928
K06949
K05307
Thiamine
phosphate
5-(2-Hydroxyethyl)-
4-methylthiazole
0, 7, 3, 3
5-(2-Hydroxyethyl)-
4-methylthiazole phosphate
0, 7, 3, 3
K00946
2.7.4.15
2, 9, 3, 3
Thiamine
diphosphate
Thiamine
triphosphate
2, 9, 2, 3
2, 7, 1, 0
K06949
K07251
K10811
0, 5, 3, 0
2, 8, 3, 3
Thiamine
0, 5, 3, 0
K20895
K20896
K20896
1.1.3.23
1.1.3.23
4-Amino-5-aminomethyl-
2-methylpyrimidine
FAMP
Thiamine
aldehyde
Thiamine
acetic acid
00730 8/25/17
(c) Kanehisa Laboratories

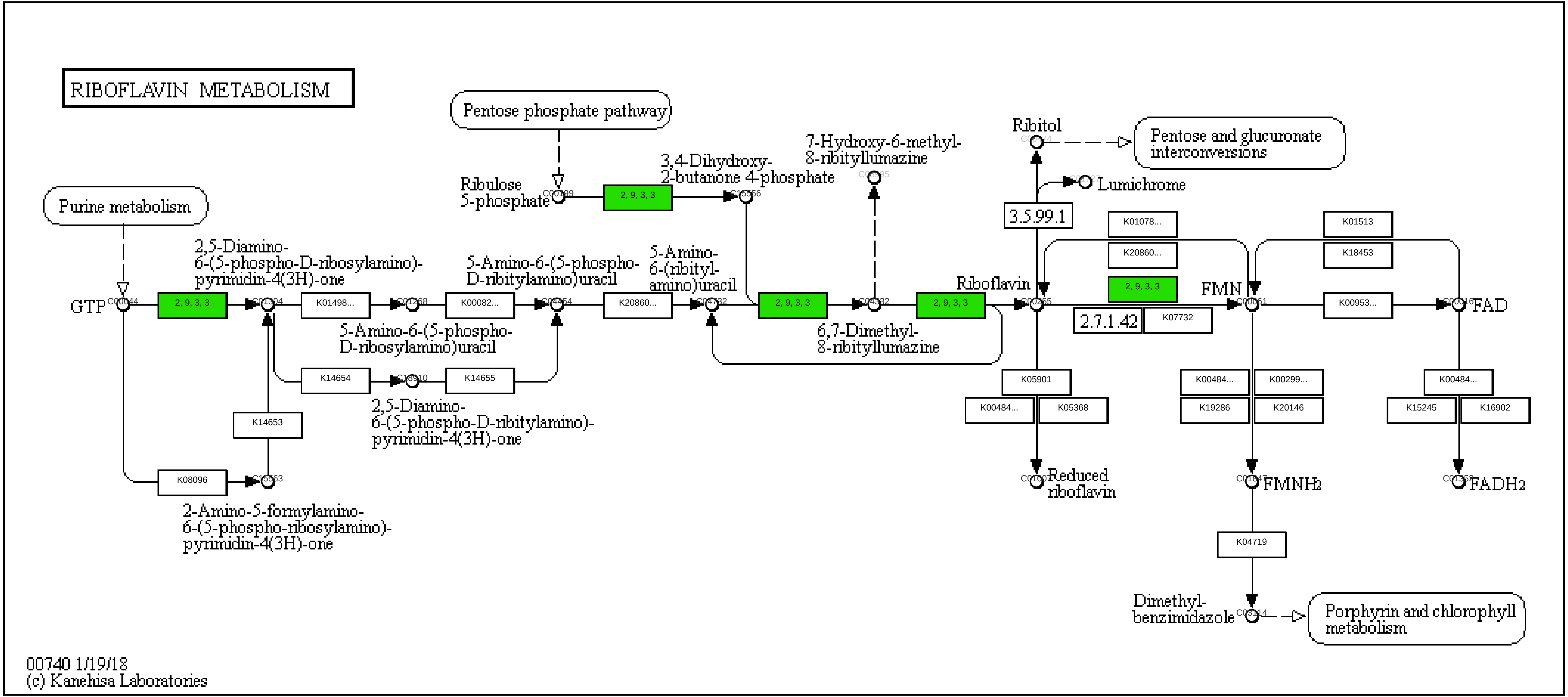
RIBOFLAVIN METABOLISM
Pentose phosphate pathway
Purine metabolism
Ribulose 5-phosphate
3,4-Dihydroxy-2-butanone 4-phosphate
7-Hydroxy-6-methyl-8-ribityllumazine
Ribitol
Pentose and glucuronate interconversions
Lumichrome
3.5.99.1
GTP
2,5-Diamino-6-(5-phospho-D-ribosylamino)-pyrimidin-4(3H)-one
5-Amino-6-(5-phospho-D-ribitylamino)uracil
5-Amino-6-(ribityl-amino)uracil
Riboflavin
FMN
FAD
5-Amino-6-(5-phospho-D-ribosylamino)uracil
6,7-Dimethyl-8-ribityllumazine
2,5-Diamino-6-(5-phospho-D-ribitylamino)-pyrimidin-4(3H)-one
2-Amino-5-formylamino-6-(5-phospho-ribosylamino)-pyrimidin-4(3H)-one
2, 9, 3, 3
K01498...
K00082...
K20860...
K14654
K14655
K14653
K08096
K01078...
K20860...
K01513
K18453
2.7.1.42
K07732
K00953...
K05901
K00484...
K05368
K00484...
K00299...
K19286
K20146
K00484...
K15245
K16902
Reduced riboflavin
FMNH2
FADH2
K04719
Dimethyl-benzimidazole
Porphyrin and chlorophyll metabolism
00740 1/19/18
(c) Kanehisa Laboratories

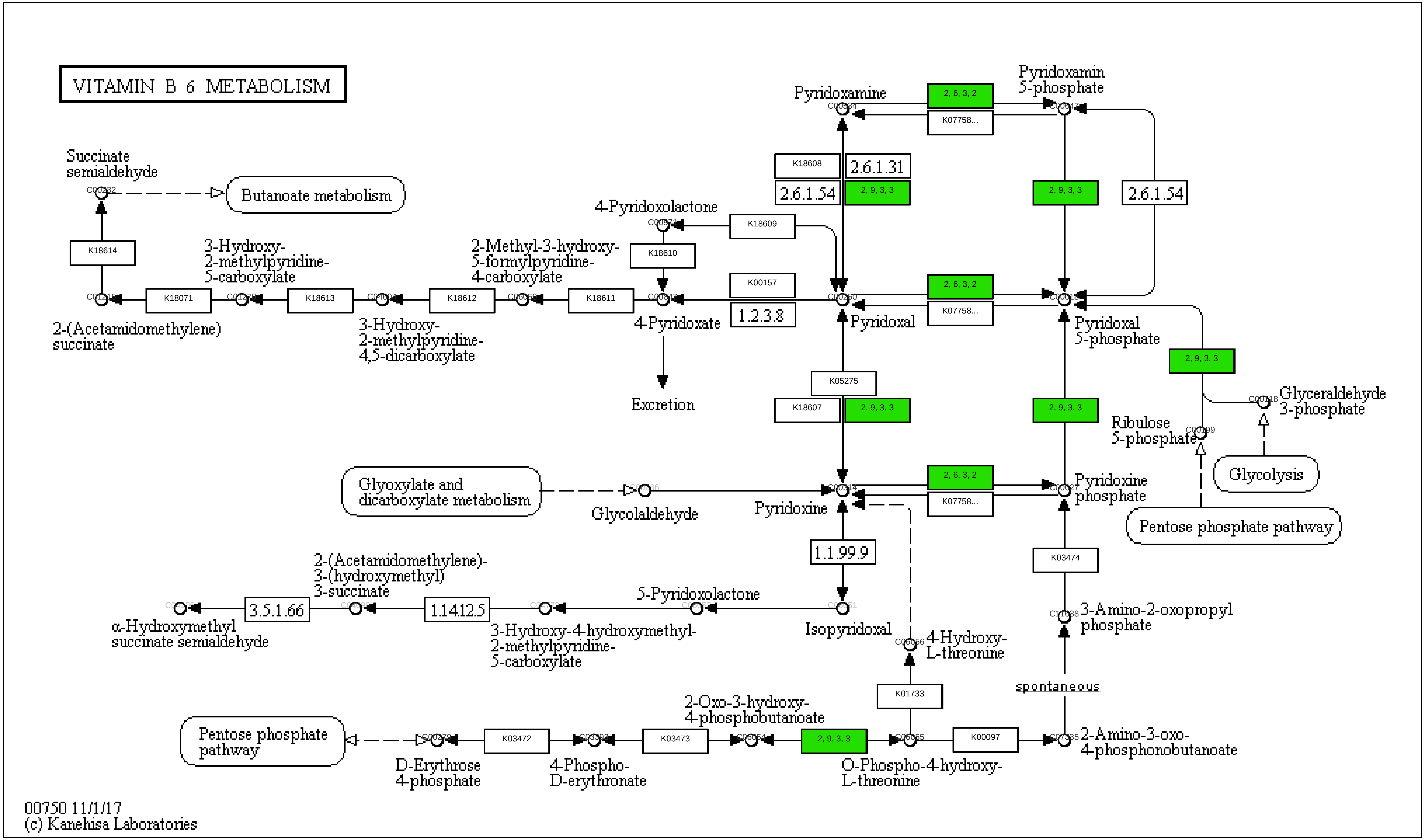
VITAMIN B 6 METABOLISM
Succinate semialdehyde
C00232
Butanoate metabolism
K18614
3-Hydroxy-2-methylpyridine-5-carboxylate
2-Methyl-3-hydroxy-5-formylpyridine-4-carboxylate
4-Pyridoxolactone
K18609
K18610
K18071
K18613
K18612
K18611
K00157
1.2.3.8
2-(Acetamidomethylene) succinate
3-Hydroxy-2-methylpyridine-4,5-dicarboxylate
4-Pyridoxate
Excretion
Pyridoxamine
Pyridoxamin 5-phosphate
2, 6, 3, 2
K07758...
K18608
2.6.1.31
2.6.1.54
2, 9, 3, 3
2.6.1.54
2, 9, 3, 3
2.6.1.54
2, 6, 3, 2
K07758...
Pyridoxal
Pyridoxal 5-phosphate
2, 9, 3, 3
K05275
K18607
2, 9, 3, 3
2, 9, 3, 3
Glyceraldehyde 3-phosphate
Ribulose 5-phosphate
Glycolysis
Glyoxylate and dicarboxylate metabolism
Glycolaldehyde
Pyridoxine
2, 6, 3, 2
K07758...
Pyridoxine phosphate
Pentose phosphate pathway
1.1.99.9
K03474
2-(Acetamidomethylene)-3-(hydroxymethyl)3-succinate
5-Pyridoxolactone
α-Hydroxymethyl succinate semialdehyde
3.5.1.66
1.14.12.5
3-Hydroxy-4-hydroxymethyl-2-methylpyridine-5-carboxylate
Isopyridoxal
3-Amino-2-oxopropyl phosphate
C11638
4-Hydroxy-L-threonine
C06056
K01733
spontaneous
2-Oxo-3-hydroxy-4-phosphobutanoate
Pentose phosphate pathway
K03472
K03473
2, 9, 3, 3
K00097
D-Erythrose 4-phosphate
4-Phospho-D-erythronate
O-Phospho-4-hydroxy-L-threonine
2-Amino-3-oxo-4-phosphonobutanoate
00750 11/1/17
(c) Kanehisa Laboratories

NICOTINATE AND NICOTINAMIDE METABOLISM

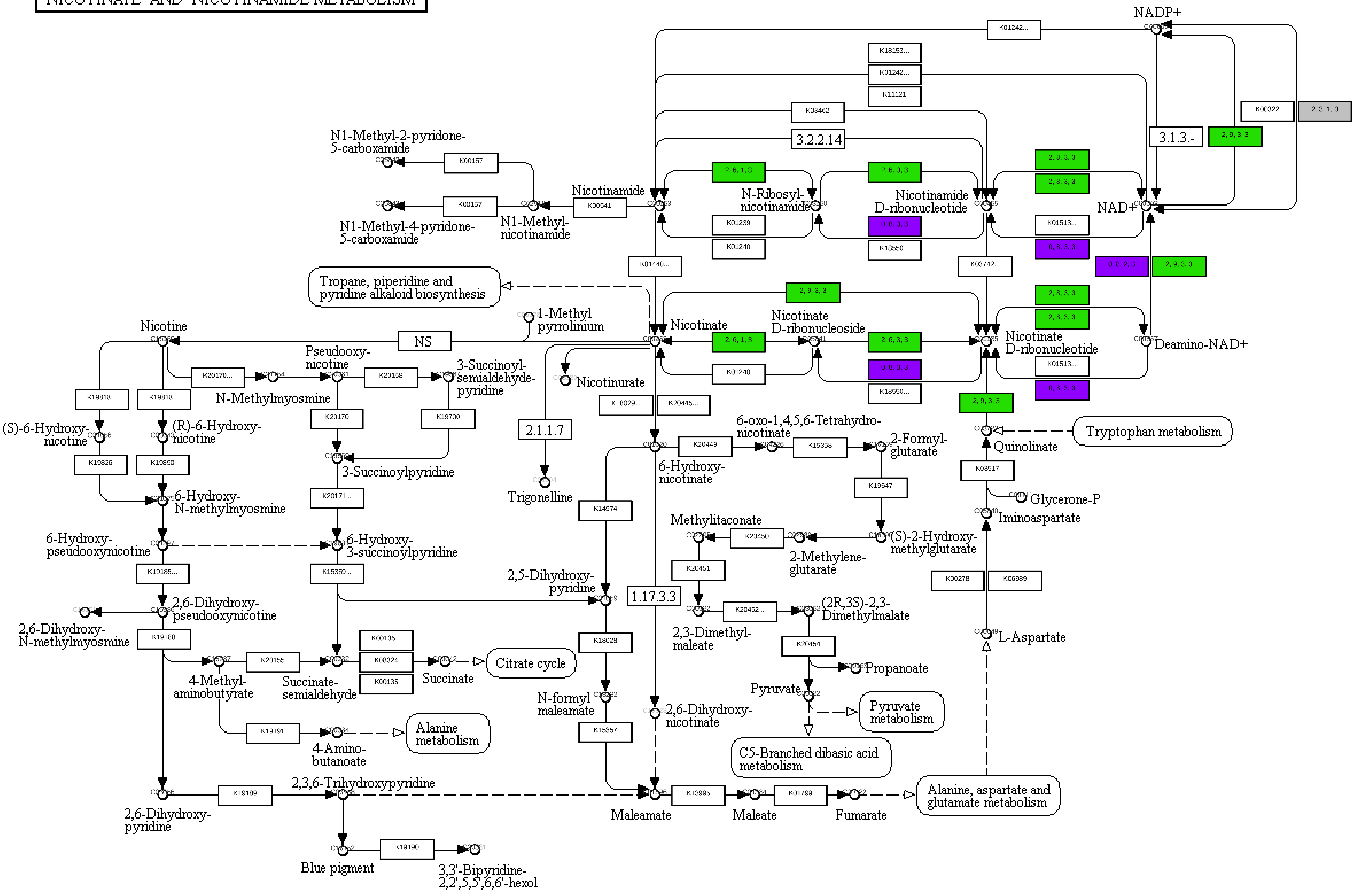

00760 9/21/16

(c) Kanehisa Laboratories

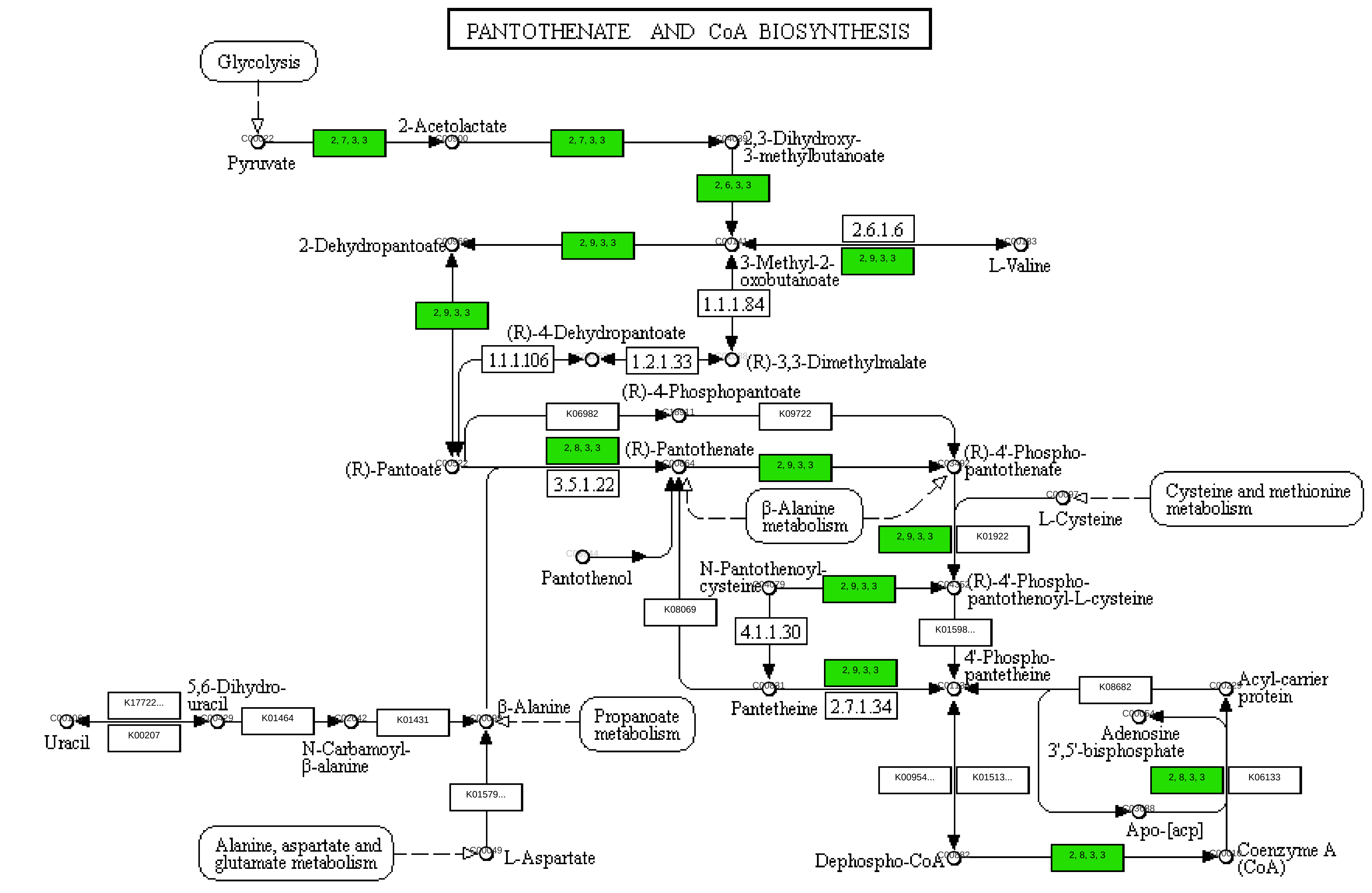

00770 7/27/17
(c) Kanehisa Laboratories

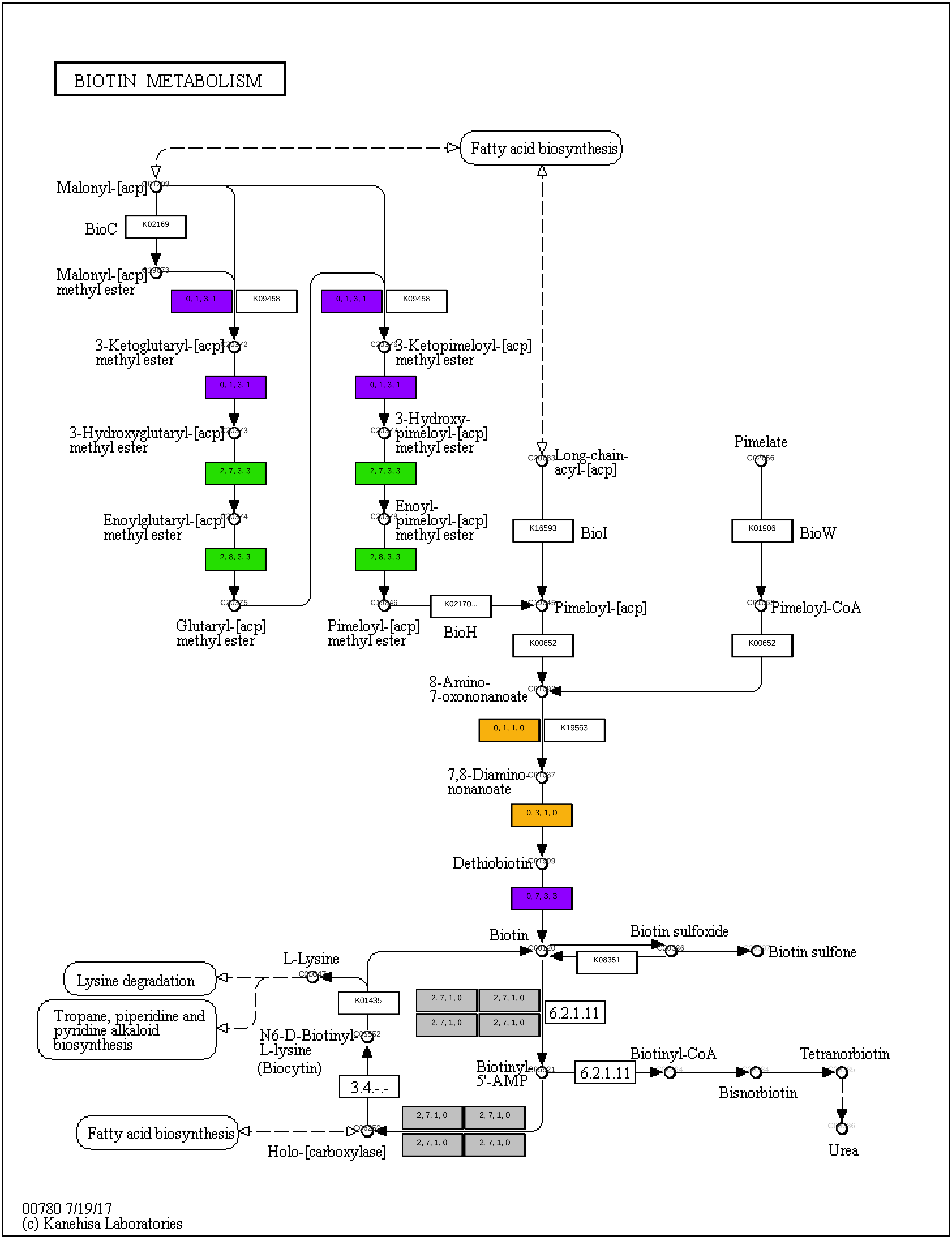
BIOTIN METABOLISM
Fatty acid biosynthesis
Malonyl-[acp]
BioC
K02169
Malonyl-[acp] methyl ester
0, 1, 3, 1
K09458
0, 1, 3, 1
K09458
3-Ketoglutaryl-[acp] methyl ester
C20372
3-Ketopimeloyl-[acp] methyl ester
0, 1, 3, 1
0, 1, 3, 1
3-Hydroxyglutaryl-[acp] methyl ester
3-Hydroxy-pimeloyl-[acp] methyl ester
2, 7, 3, 3
2, 7, 3, 3
Enoylglutaryl-[acp] methyl ester
Enoyl-pimeloyl-[acp] methyl ester
2, 8, 3, 3
2, 8, 3, 3
Glutaryl-[acp] methyl ester
Pimeloyl-[acp] methyl ester
K02170...
BioH
Long-chain-acyl-[acp]
K16593
BioI
Pimelate
K01906
BioW
Pimeloyl-[acp]
Pimeloyl-CoA
K00652
K00652
8-Amino-7-oxononanoate
0, 1, 1, 0
K19563
7,8-Diamino-nonanoate
0, 3, 1, 0
Dethiobiotin
0, 7, 3, 3
Biotin
Biotin sulfoxide
Biotin sulfone
K08351
L-Lysine
Lysine degradation
Tropane, piperidine and pyridine alkaloid biosynthesis
K01435
2, 7, 1, 0
2, 7, 1, 0
2, 7, 1, 0
2, 7, 1, 0
6.2.1.11
N6-D-Biotinyl-L-lysine (Biocytin)
3.4.-.-
Biotinyl-5'-AMP
6.2.1.11
Biotinyl-CoA
Tetranorbiotin
Bisnorbiotin
Urea
2, 7, 1, 0
2, 7, 1, 0
2, 7, 1, 0
2, 7, 1, 0
Fatty acid biosynthesis
Holo-[carboxylase]
00780 7/19/17
(c) Kanehisa Laboratories

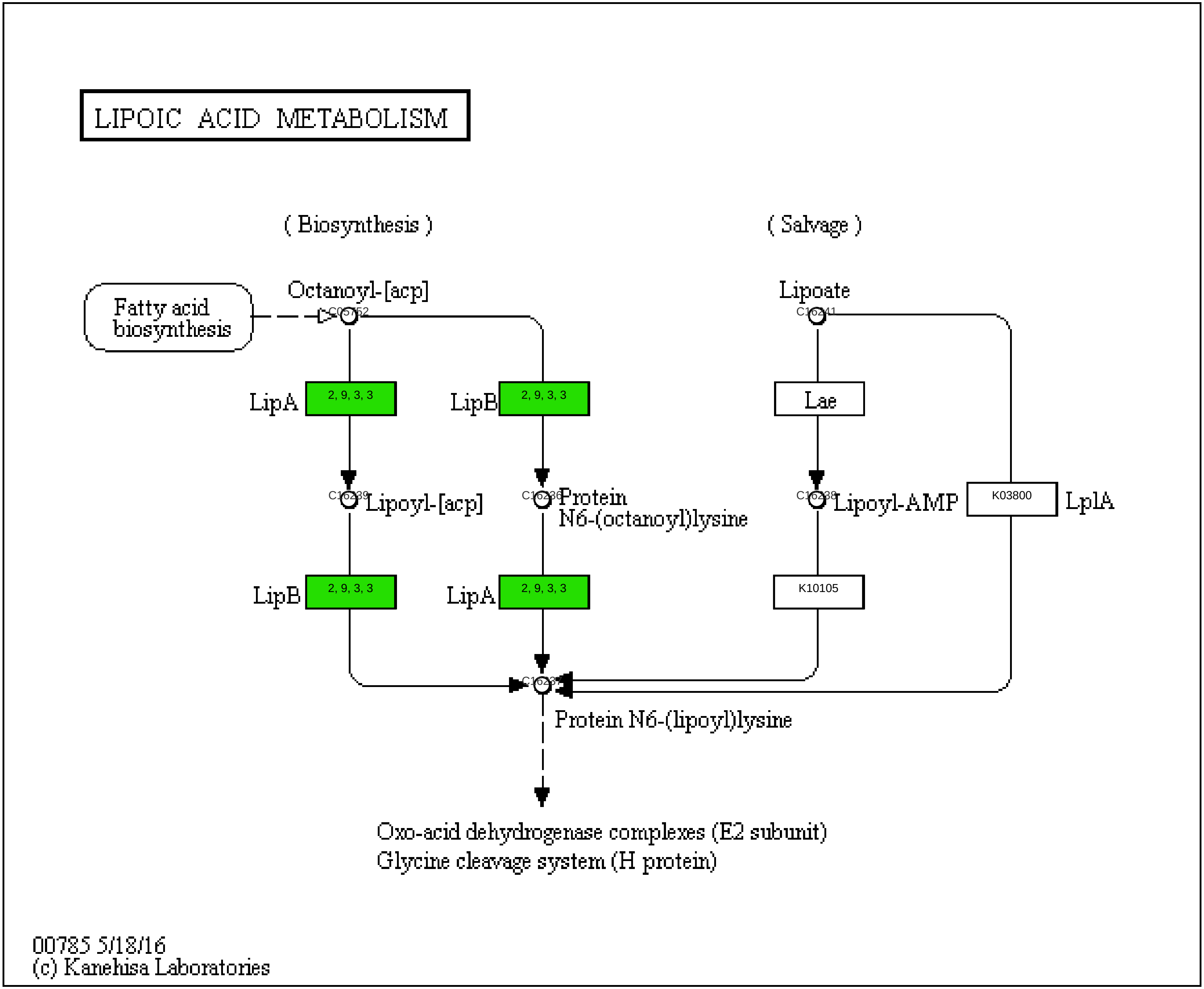
LIPOIC ACID METABOLISM
(Biosynthesis)
(Salvage)
Fatty acid biosynthesis
Octanoyl-[acp]
C05752
Lipoate
C16241
LipA
2, 9, 3, 3
LipB
2, 9, 3, 3
Lae
C16239
Lipoyl-[acp]
C16236
Protein N6-(octanoyl)lysine
C16238
Lipoyl-AMP
K03800
LplA
LipB
2, 9, 3, 3
LipA
2, 9, 3, 3
K10105
Protein N6-(lipoyl)lysine
Oxo-acid dehydrogenase complexes (E2 subunit)
Glycine cleavage system (H protein)
00785 5/18/16
(c) Kanehisa Laboratories

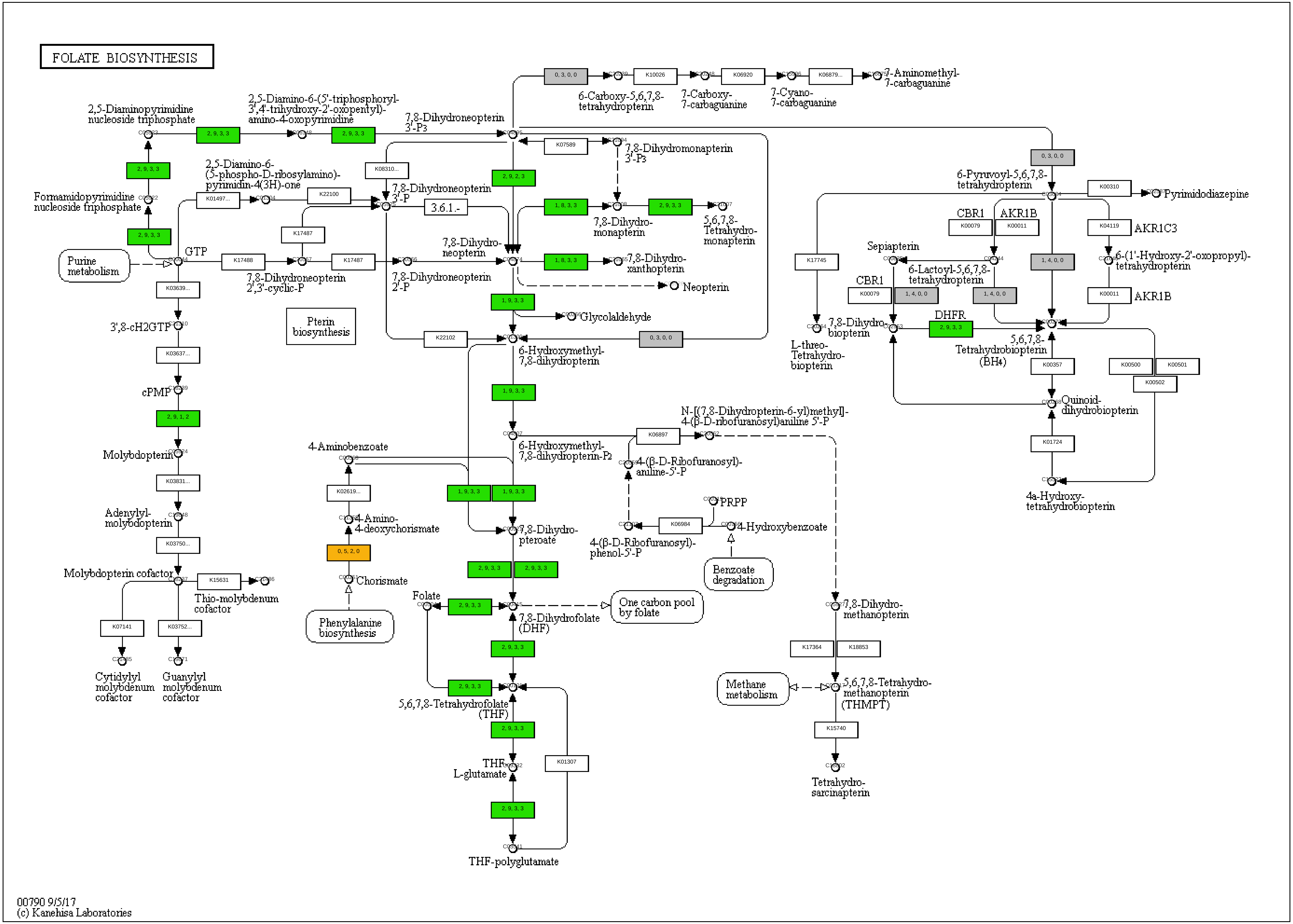
FOLATE BIOSYNTHESIS
2,5-Diaminopyrimidine nucleoside triphosphate
2,5-Diamino-6-(5'-triphosphoryl-3',4'-trihydroxy-2'-oxopentyl)-amino-4-oxopyrimidine
7,8-Dihydroneopterin 3'-P3
6-Carboxy-5,6,7,8-tetrahydropterin
7-Carboxy-7-carbaguanine
7-Cyano-7-carbaguanine
7-Aminomethyl-7-carbaguanine
Formamidopyrimidine nucleoside triphosphate
2,5-Diamino-6-(5-phospho-D-ribosylamino)-pyrimidin-4(3H)-one
7,8-Dihydroneopterin 3'-P
3.6.1.-
7,8-Dihydromonapterin 3'-P3
7,8-Dihydro-monapterin
5,6,7,8-Tetrahydro-monapterin
Purine metabolism
GTP
7,8-Dihydro-neopterin
7,8-Dihydroneopterin 2',3'-cyclic-P
7,8-Dihydroneopterin 2'-P
7,8-Dihydro-xanthopterin
Neopterin
Glycolaldehyde
Pterin biosynthesis
3',8-cH2GTP
cPMP
Molybdopterin
Adenylyl-molybdopterin
Molybdopterin cofactor
Thio-molybdenum cofactor
Cytidylyl molybdenum cofactor
Guanylyl molybdenum cofactor
6-Hydroxymethyl-7,8-dihydropterin
6-Hydroxymethyl-7,8-dihydropterin-P2
N-[(7,8-Dihydropterin-6-yl)methyl]-4-(β-D-ribofuranosyl)aniline 5'-P
4-(β-D-Ribofuranosyl)-aniline-5'-P
PRPP
4-Hydroxybenzoate
4-(β-D-Ribofuranosyl)-phenol-5'-P
Benzoate degradation
4-Aminobenzoate
4-Amino-4-deoxychorismate
Chorismate
Phenylalanine biosynthesis
7,8-Dihydro-pteroate
Folate
7,8-Dihydrofolate (DHF)
One carbon pool by folate
5,6,7,8-Tetrahydrofolate (THF)
THF-L-glutamate
THF-polyglutamate
7,8-Dihydro-methanopterin
Methane metabolism
5,6,7,8-Tetrahydro-methanopterin (THMPT)
Tetrahydro-sarcinapterin
6-Pyruvoyl-5,6,7,8-tetrahydropterin
Pyrimidodiazepine
CBR1
AKR1B
AKR1C3
Sepiapterin
6-Lactoyl-5,6,7,8-tetrahydropterin
6-(1'-Hydroxy-2'-oxopropyl)-tetrahydropterin
DHFR
L-threo-Tetrahydro-biopterin
7,8-Dihydro-biopterin
5,6,7,8-Tetrahydrobiopterin (BH4)
Quinoid-dihydrobiopterin
4a-Hydroxy-tetrahydrobiopterin
00790 9/5/17
(c) Kanehisa Laboratories

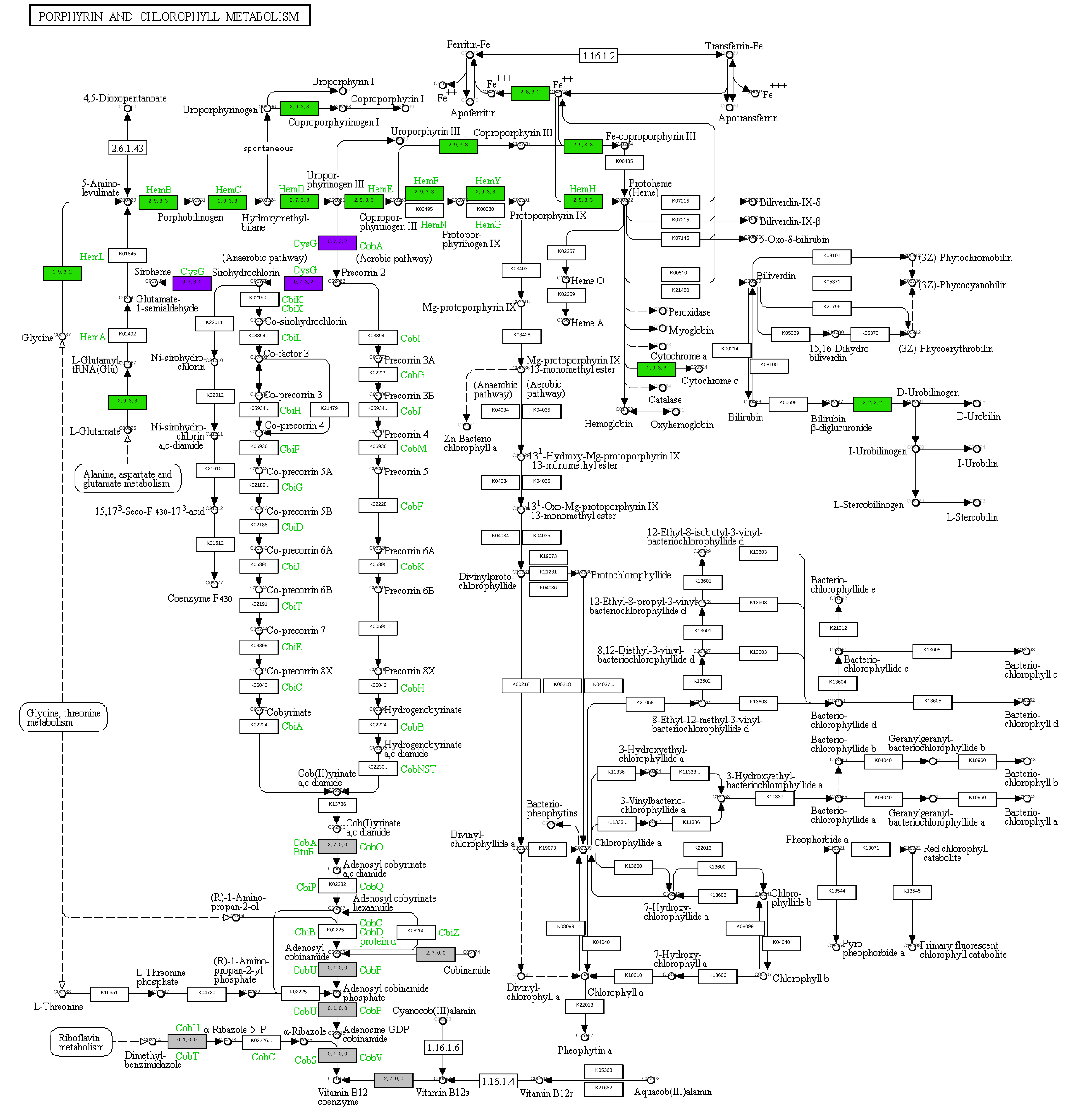

00860 12/22/17

(c) Kanehisa Laboratories

9. Metabolism of terpenoids and polyketides

MAP	PATHWAY
**900**	Terpenoid backbone biosynthesis
**909**	Sesquiterpenoid and triterpenoid biosynthesis

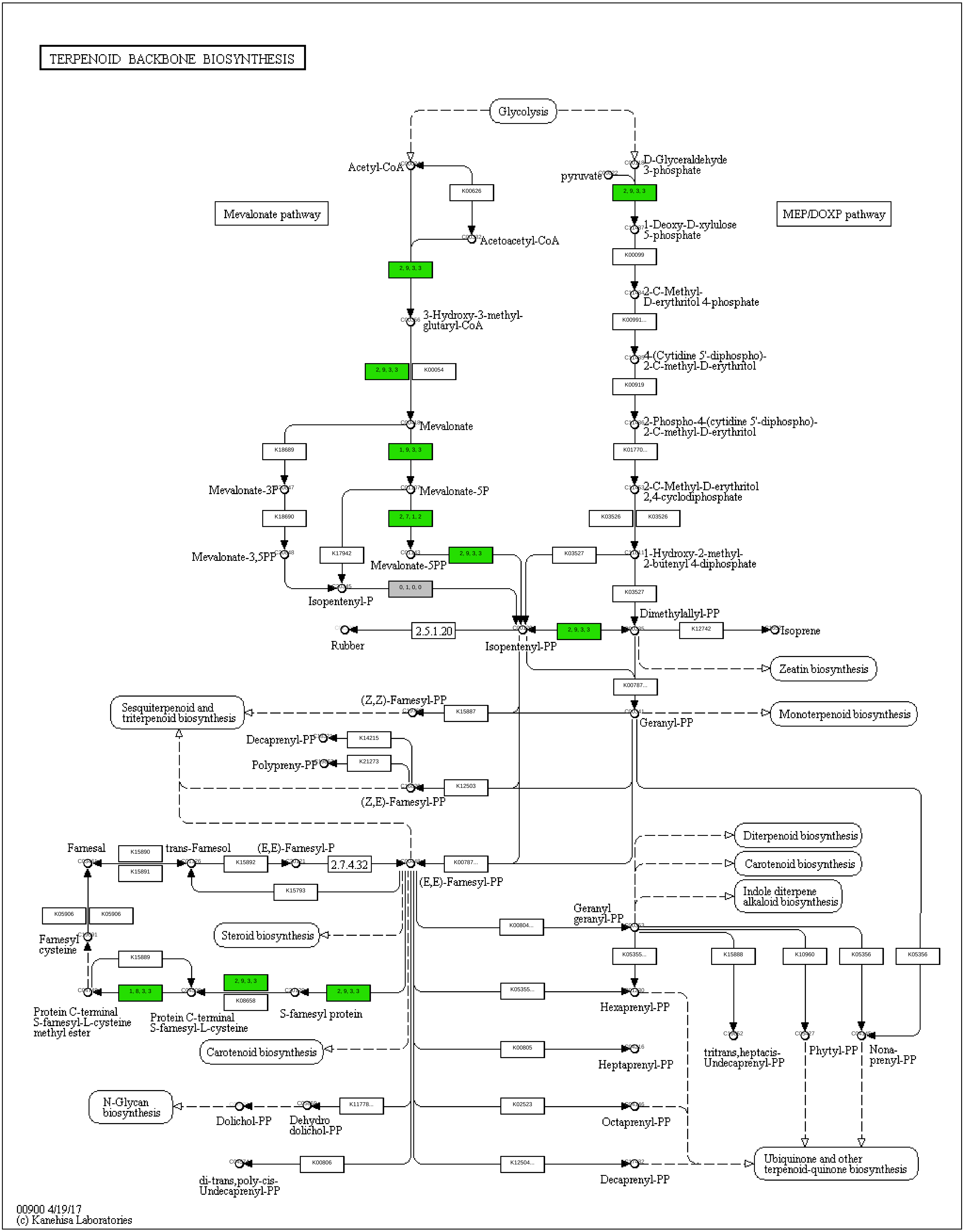
TERPENOID BACKBONE BIOSYNTHESIS
Glycolysis
Acetyl-CoA
K00626
Acetoacetyl-CoA
Mevalonate pathway
2, 9, 3, 3
3-Hydroxy-3-methyl-
glutaryl-CoA
2, 9, 3, 3
K00054
Mevalonate
K18689
1, 9, 3, 3
Mevalonate-3P
Mevalonate-5P
K18690
2, 7, 1, 2
Mevalonate-3,5PP
K17942
2, 9, 3, 3
Mevalonate-5PP
Isopentenyl-P
0, 1, 0, 0
D-Glyceraldehyde
3-phosphate
pyruvate
2, 9, 3, 3
MEP/DOXP pathway
1-Deoxy-D-xylulose
5-phosphate
K00099
2-C-Methyl-
D-erythritol 4-phosphate
K00991...
4-(Cytidine 5'-diphospho)-
2-C-methyl-D-erythritol
K00919
2-Phospho-4-(cytidine 5'-diphospho)-
2-C-methyl-D-erythritol
K01770...
2-C-Methyl-D-erythritol
2,4-cyclodiphosphate
K03526
K03526
K03527
1-Hydroxy-2-methyl-
2-butenyl 4-diphosphate
K03527
Dimethylallyl-PP
Rubber
2.5.1.20
Isopentenyl-PP
2, 9, 3, 3
K12742
Isoprene
Zeatin biosynthesis
K00787...
Sesquiterpenoid and
triterpenoid biosynthesis
(Z,Z)-Farnesyl-PP
K15887
Geranyl-PP
Monoterpenoid biosynthesis
Decaprenyl-PP
K14215
Polypreny-PP
K21273
K12503
(Z,E)-Farnesyl-PP
Diterpenoid biosynthesis
Farnesal
K15890
K15891
trans-Farnesol
K15892
(E,E)-Farnesyl-P
2.7.4.32
K00787...
(E,E)-Farnesyl-PP
Carotenoid biosynthesis
K15793
Indole diterpene
alkaloid biosynthesis
K05906
K05906
Farnesyl
cysteine
Steroid biosynthesis
K00804...
Geranyl
geranyl-PP
K15889
2, 9, 3, 3
1, 8, 3, 3
K08658
2, 9, 3, 3
K05355...
K15888
K10960
K05356
K05356
Protein C-terminal
S-farnesyl-L-cysteine
methyl ester
Protein C-terminal
S-farnesyl-L-cysteine
S-farnesyl protein
K05355...
Hexaprenyl-PP
Carotenoid biosynthesis
K00805
Heptaprenyl-PP
tritrans,heptacis-
Undecaprenyl-PP
Phytyl-PP
Nona-
prenyl-PP
N-Glycan
biosynthesis
Dolichol-PP
Dehydro
dolichol-PP
K11778...
K02523
Octaprenyl-PP
Ubiquinone and other
terpenoid-quinone biosynthesis
K00806
di-trans,poly-cis-
Undecaprenyl-PP
K12504...
Decaprenyl-PP
00900 4/19/17
(c) Kanehisa Laboratories

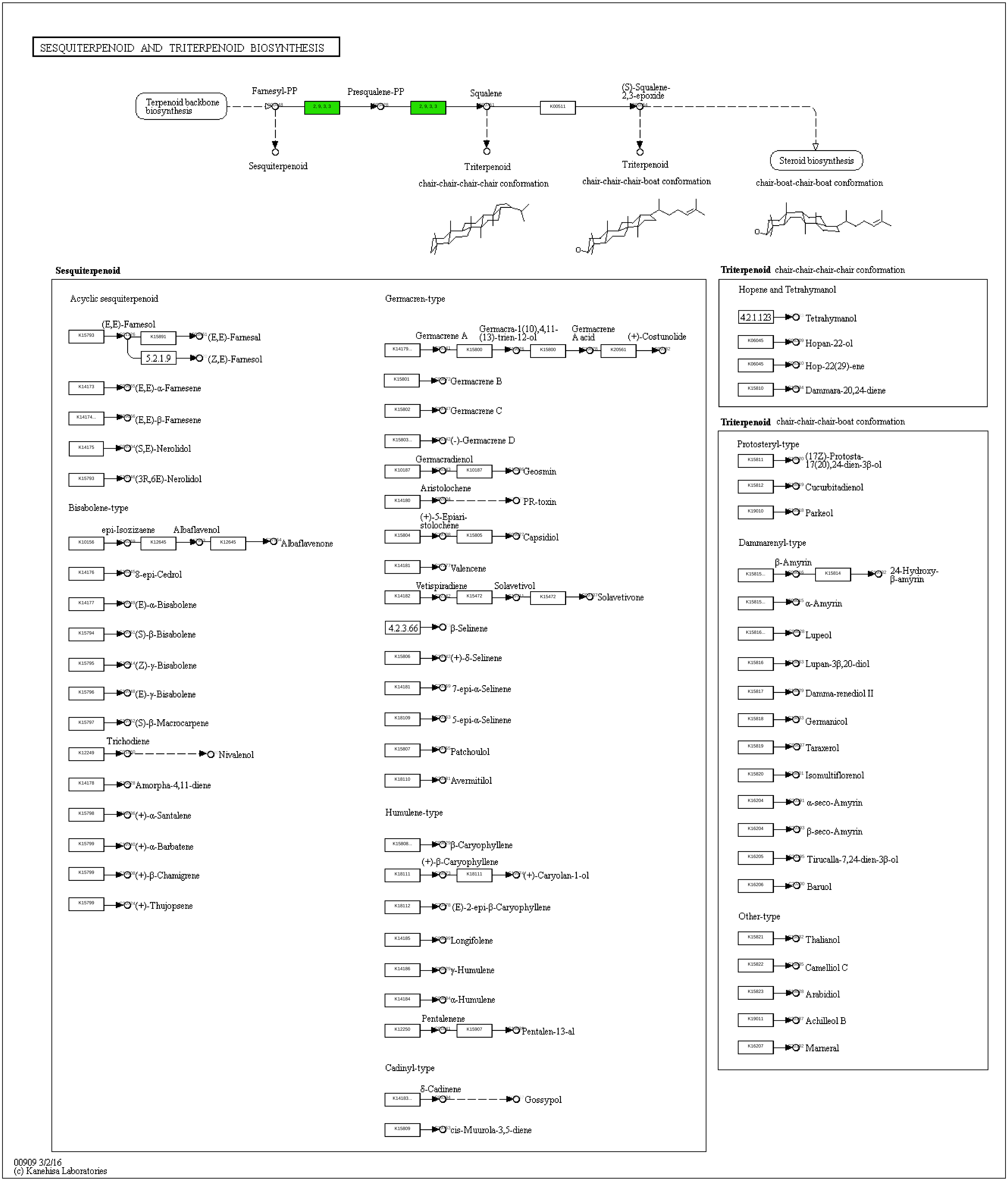
SESQUITERPENOID AND TRITERPENOID BIOSYNTHESIS
Terpenoid backbone biosynthesis
Farnesyl-PP
2.5.1.21
Presqualene-PP
1.14.14.17
Squalene
K00511
(S)-Squalene-2,3-epoxide
Sesquiterpenoid
Triterpenoid
chair-chair-chair-chair conformation
Triterpenoid
chair-chair-chair-boat conformation
Steroid biosynthesis
chair-boat-chair-boat conformation
Sesquiterpenoid
Acyclic sesquiterpenoid
(E,E)-Farnesol
K15793
K15891
(E,E)-Farnesal
5.2.1.9
(Z,E)-Farnesol
K14173
(E,E)-α-Farnesene
K14174...
(E,E)-β-Farnesene
K14175
(S,E)-Nerolidol
K15793
(3R,6E)-Nerolidol
Bisabolene-type
epi-Isozizaene
Albaflavenol
K10156
K12645
K12645
Albaflavenone
K14176
8-epi-Cedrol
K14177
(E)-α-Bisabolene
K15794
(S)-β-Bisabolene
K15795
(Z)-γ-Bisabolene
K15796
(E)-γ-Bisabolene
K15797
(S)-β-Macrocarpene
Trichodiene
K12249
Nivalenol
K14178
Amorpha-4,11-diene
K15798
(+)-α-Santalene
K15799
(+)-α-Barbatene
K15799
(+)-β-Chamigrene
K15799
(+)-Thujopsene
Germacren-type
Germacrene A
Germacra-1(10),4,11-(13)-trien-12-ol
Germacrene A acid
(+)-Costunolide
K14179...
K15800
K15800
K20561
K15801
Germacrene B
K15802
Germacrene C
K15803...
(-)-Germacrene D
Germacradienol
K10187
K10187
Geosmin
Aristolochene
K14180
PR-toxin
(+)-5-Epiaristolochene
K15804
K15805
Capsidiol
K14181
Valencene
Vetispiradiene
Solavetivol
K14182
K15472
K15472
Solavetivone
4.2.3.66
β-Selinene
K15806
(+)-δ-Selinene
K14181
7-epi-α-Selinene
K18109
5-epi-α-Selinene
K15807
Patchoulol
K18110
Avermitilol
Humulene-type
K15808...
β-Caryophyllene
(+)-β-Caryophyllene
K18111
K18111
(+)-Caryolan-1-ol
K18112
(E)-2-epi-β-Caryophyllene
K14185
Longifolene
K14186
γ-Humulene
K14184
α-Humulene
Pentalenene
K12250
K15907
Pentalen-13-al
Cadinyl-type
δ-Cadinene
K14183...
Gossypol
K15809
cis-Muurola-3,5-diene
Triterpenoid chair-chair-chair-chair conformation
Hopene and Tetrahymanol
4.2.1.123
Tetrahymanol
K06045
Hopan-22-ol
K06045
Hop-22(29)-ene
K15810
Dammara-20,24-diene
Triterpenoid chair-chair-chair-boat conformation
Protosteryl-type
K15811
(17Z)-Protosta-17(20),24-dien-3β-ol
K15812
Cucurbitadienol
K19010
Parkeol
Dammarenyl-type
β-Amyrin
K15815...
K15814
24-Hydroxy-β-amyrin
K15815...
α-Amyrin
K15816...
Lupeol
K15816
Lupan-3β,20-diol
K15817
Damma-renediol II
K15818
Germanicol
K15819
Taraxerol
K15820
Isomultiflorenol
K16204
α-seco-Amyrin
K16204
β-seco-Amyrin
K16205
Tirucalla-7,24-dien-3β-ol
K16206
Baruol
Other-type
K15821
Thalianol
K15822
Camelliol C
K15823
Arabidiol
K19011
Achilleol B
K16207
Marneral
00909 3/2/16
(c) Kanehisa Laboratories

10. Biosynthesis of other secondary metabolites

MAP	PATHWAY
**261**	Monobactam biosynthesis
**332**	Carbapenem biosynthesis

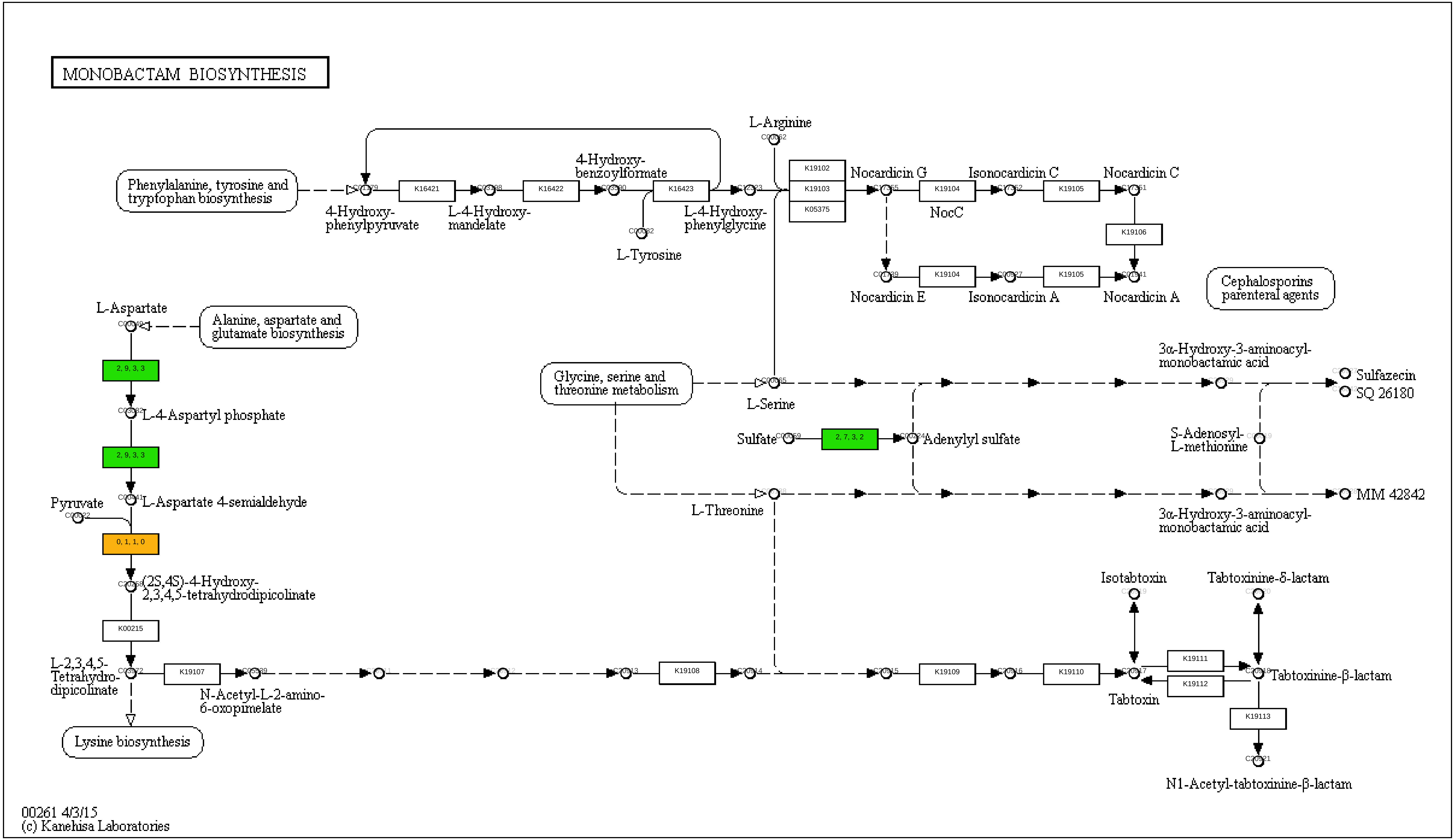

MONOBACTAM BIOSYNTHESIS
Phenylalanine, tyrosine and tryptophan biosynthesis
4-Hydroxy-phenylpyruvate
K16421
L-4-Hydroxy-mandelate
K16422
4-Hydroxy-benzoylformate
K16423
L-Tyrosine
L-4-Hydroxy-phenylglycine
L-Arginine
K19102
K19103
K05375
Nocardicin G
K19104
NocC
Isonocardicin C
K19105
Nocardicin C
K19106
Nocardicin E
K19104
Isonocardicin A
K19105
Nocardicin A
Cephalosporins parenteral agents
L-Aspartate
Alanine, aspartate and glutamate biosynthesis
2, 9, 3, 3
L-4-Aspartyl phosphate
2, 9, 3, 3
L-Aspartate 4-semialdehyde
Pyruvate
0, 1, 1, 0
(2S,4S)-4-Hydroxy-2,3,4,5-tetrahydrodipicolinate
K00215
L-2,3,4,5-Tetrahydro-dipicolinate
K19107
N-Acetyl-L-2-amino-6-oxopimelate
Lysine biosynthesis
Glycine, serine and threonine metabolism
L-Serine
Sulfate
2, 7, 3, 2
Adenylyl sulfate
L-Threonine
3α-Hydroxy-3-aminoacyl-monobactamic acid
Sulfazecin
SQ 26180
S-Adenosyl-L-methionine
MM 42842
3α-Hydroxy-3-aminoacyl-monobactamic acid
K19108
K19109
K19110
Isotabtoxin
Tabtoxinine-δ-lactam
K19111
K19112
Tabtoxinine-β-lactam
Tabtoxin
K19113
N1-Acetyl-tabtoxinine-β-lactam
00261 4/3/15
(c) Kanehisa Laboratories

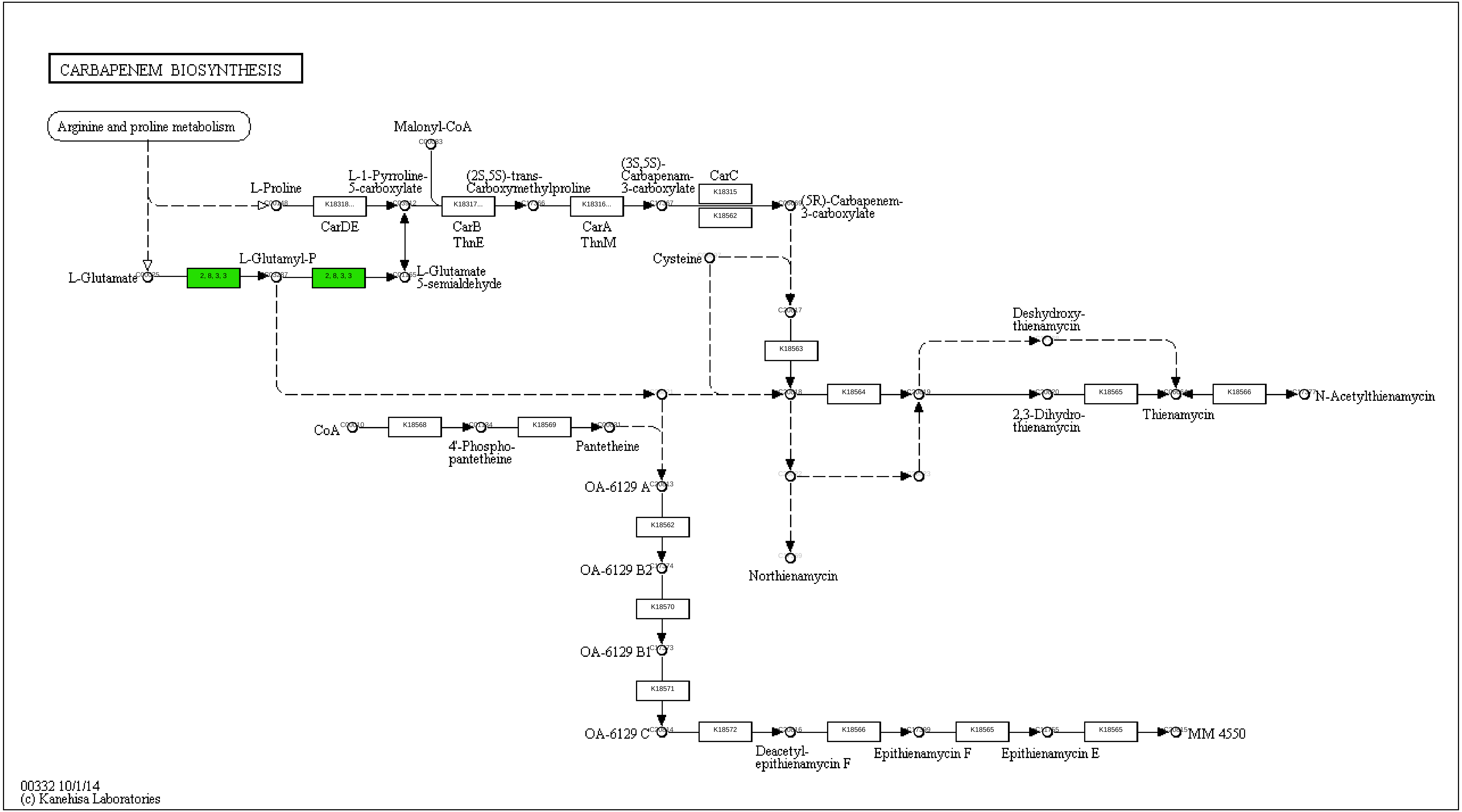
CARBAPENEM BIOSYNTHESIS
Arginine and proline metabolism
Malonyl-CoA
L-Proline
L-1-Pyrroline-5-carboxylate
(2S,5S)-trans-Carboxymethylproline
(3S,5S)-Carbapenam-3-carboxylate
CarC
K18315
K18562
(5R)-Carbapenem-3-carboxylate
K18318...
CarDE
K18317...
CarB
ThnE
K18316...
CarA
ThnM
L-Glutamyl-P
L-Glutamate
2, 8, 3, 3
2, 8, 3, 3
L-Glutamate 5-semialdehyde
Cysteine
K18563
Deshydroxy-thienamycin
K18564
K18565
K18566
2,3-Dihydro-thienamycin
Thienamycin
N-Acetylthienamycin
CoA
K18568
4'-Phospho-pantetheine
K18569
Pantetheine
OA-6129 A
K18562
OA-6129 B2
K18570
OA-6129 B1
K18571
OA-6129 C
K18572
Deacetyl-epithienamycin F
K18566
Epithienamycin F
K18565
Epithienamycin E
K18565
MM 4550
Northienamycin
00332 10/1/14
(c) Kanehisa Laboratories

11. Xenobiotics biodegradation and metabolism

MAP	PATHWAY
**791**	Atrazine degradation

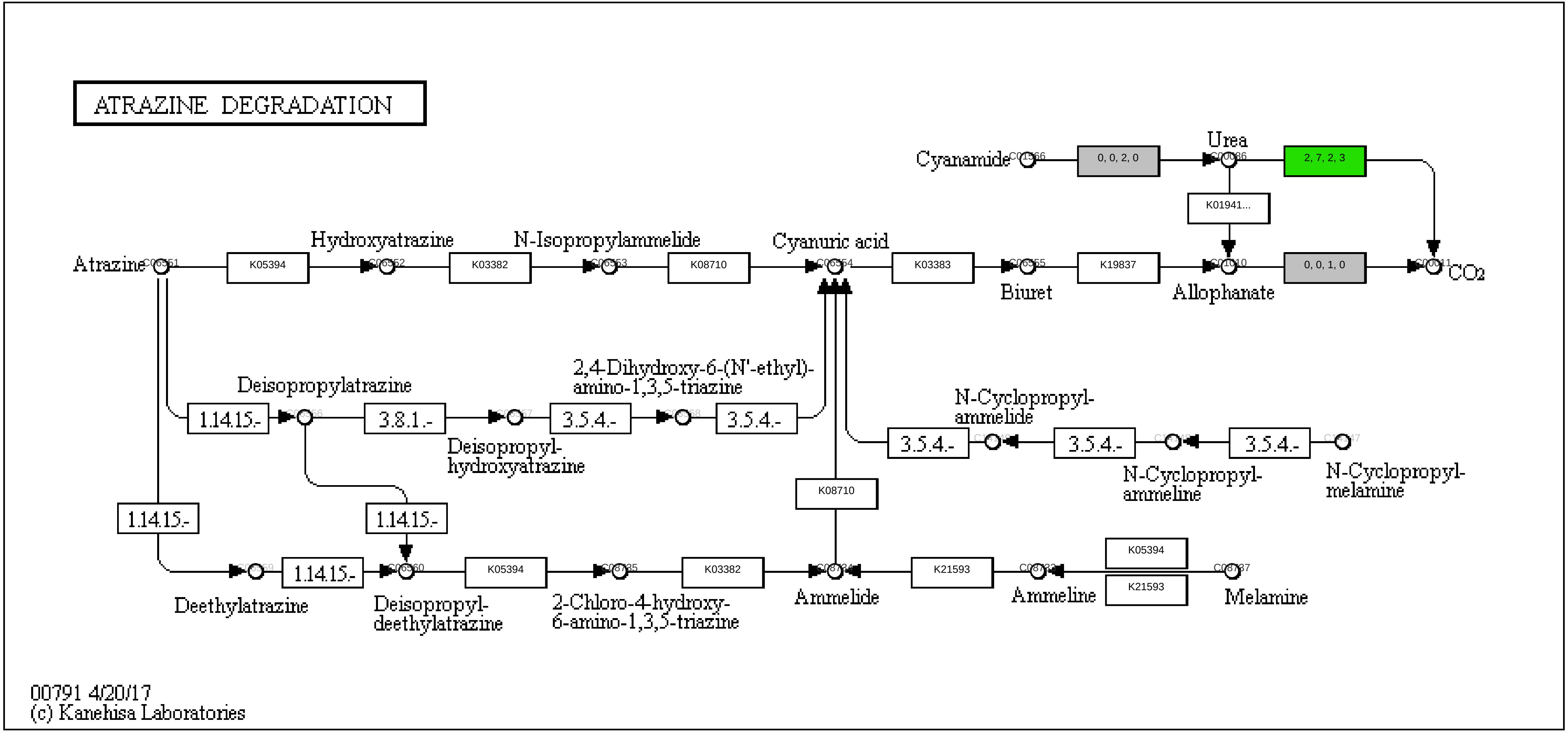
ATRAZINE DEGRADATION
Cyanamide
0, 0, 2, 0
Urea
2, 7, 2, 3
K01941...
Atrazine
K05394
Hydroxyatrazine
K03382
N-Isopropylammelide
K08710
Cyanuric acid
K03383
Biuret
K19837
Allophanate
0, 0, 1, 0
CO2
Deisopropylatrazine
1.14.15.-
3.8.1.-
Deisopropyl-
hydroxyatrazine
2,4-Dihydroxy-6-(N'-ethyl)-
amino-1,3,5-triazine
3.5.4.-
3.5.4.-
N-Cyclopropyl-
ammelide
3.5.4.-
3.5.4.-
3.5.4.-
N-Cyclopropyl-
ammeline
N-Cyclopropyl-
melamine
K08710
1.14.15.-
1.14.15.-
1.14.15.-
Deethylatrazine
Deisopropyl-
deethylatrazine
K05394
2-Chloro-4-hydroxy-
6-amino-1,3,5-triazine
K03382
Ammelide
K21593
Ammeline
K05394
K21593
Melamine
00791 4/20/17
(c) Kanehisa Laboratories
