# Supplementary material for: Comparative genomics of chytrid fungi reveal insights into the obligate biotrophic and pathogenic lifestyle of Synchytrium endobioticum
Source: Sci Rep. 2019 Jun 17;9:8672. doi: 10.1038/s41598-019-45128-9 (PMC6572847; doi:10.1038/s41598-019-45128-9)
Supplement: Supplementary file 4 [file 41598_2019_45128_MOESM4_ESM.pdf]

Supplementary file with: **Comparative genomics of chytrid fungi reveal insights into the obligate biotrophic and pathogenic lifestyle of *Synchytrium endobioticum***

Bart T.L.H. van de Vossenberg & Sven Warris, Hai D.T. Nguyen, Marga P.E. van Gent-Pelzer, David L. Joly, Henri C. van de Geest, Peter J.M. Bonants, Donna S. Smith, C. André Lévesque, Theo A.J. van der Lee

**Supplementary file 4 KEGG pathway analysis**

# *Ascomycota and Basidiomycota (benchmarks)*

**The following color codes are used:**

True positive (TP): element in the reference pathway correctly predicted by Interproscan

True negative (TN): element in the *not* reference pathway and *not* predicted by Interproscan

False negative (FN): element in the reference pathway but *not* predicted by Interproscan.

False positive (FP): element in the *not* reference pathway but predicted by Interproscan. Note: could also be a valid new prediction

**Species:** ***Saccharomyces cerevisiae***

**Acronym:** **SCE**

**Lifestyle:** **Culturable**

# 1. Carbohydrate metabolism

| MAP | PATHWAY |
|---|---|
| **10** | Glycolysis / Gluconeogenesis |
| **20** | Citrate cycle (TCA cycle) |
| **30** | Pentose phosphate pathway |
| **40** | Pentose and glucuronate interconversions |
| **51** | Fructose and mannose metabolism |
| **52** | Galactose metabolism |
| **53** | Ascorbate and aldarate metabolism |
| **500** | Starch and sucrose metabolism |
| **520** | Amino sugar and nucleotide sugar metabolism |
| **562** | Inositol phosphate metabolism |
| **620** | Pyruvate metabolism |
| **630** | Glyoxylate and dicarboxylate metabolism |
| **640** | Propanoate metabolism |
| **650** | Butanoate metabolism |
| **660** | C5-Branched dibasic acid metabolism |

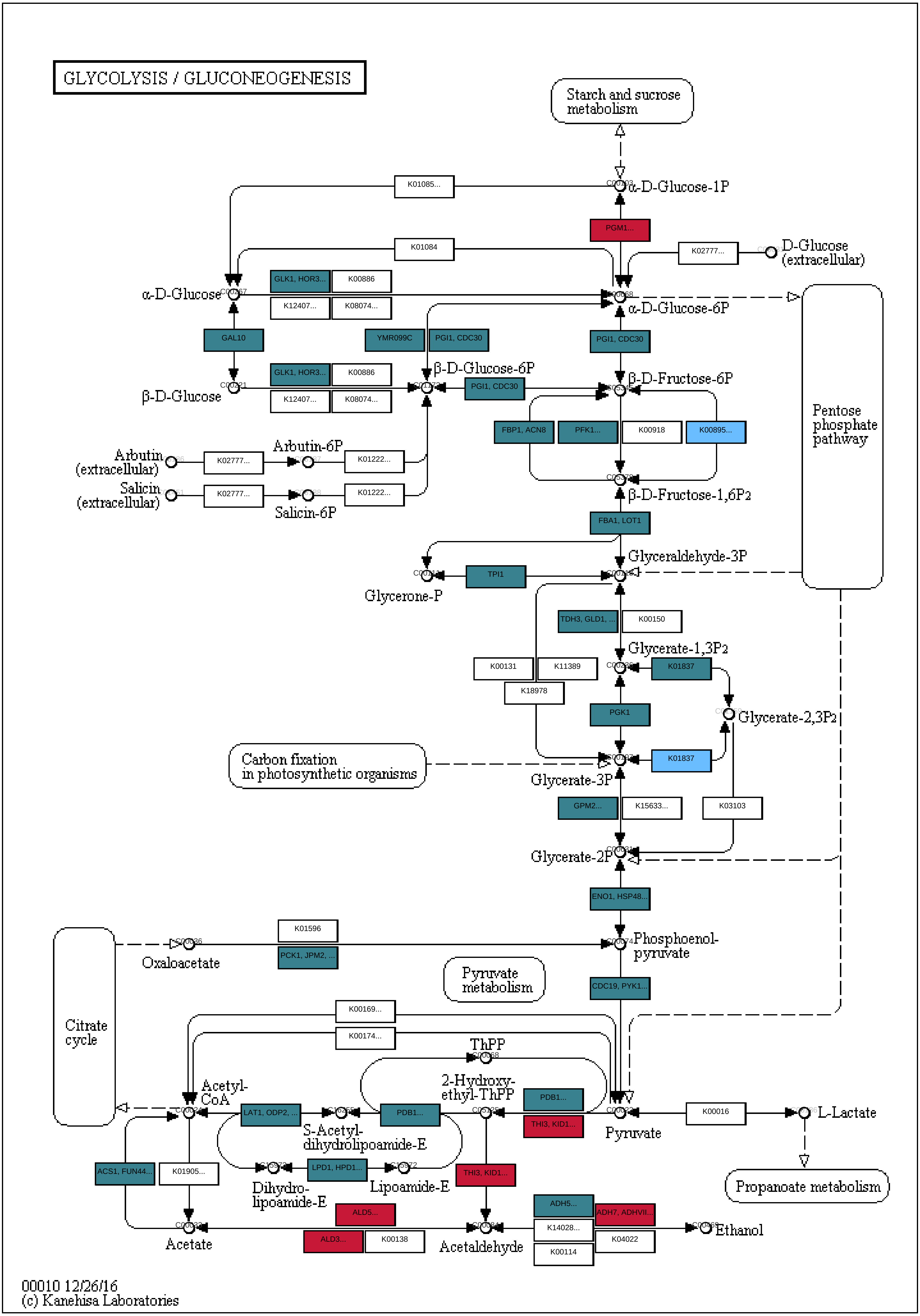
GLYCOLYSIS / GLUCONEOGENESIS
Starch and sucrose metabolism
α-D-Glucose-1P
K01085...
PGM1...
K01084
K02777...
D-Glucose (extracellular)
α-D-Glucose
GLK1, HOR3...
K00886
K12407...
K08074...
α-D-Glucose-6P
Pentose phosphate pathway
GAL10
YMR099C
PGI1, CDC30
β-D-Glucose-6P
β-D-Fructose-6P
β-D-Glucose
FBP1, ACN8
PFK1...
K00918
K00895...
Arbutin (extracellular)
Arbutin-6P
K01222...
Salicin (extracellular)
Salicin-6P
β-D-Fructose-1,6P2
FBA1, LOT1
Glyceraldehyde-3P
TPI1
Glycerone-P
TDH3, GLD1, ...
K00150
Glycerate-1,3P2
K00131
K11389
K18978
K01837
PGK1
Glycerate-2,3P2
Carbon fixation in photosynthetic organisms
Glycerate-3P
GPM2...
K15633...
K03103
Glycerate-2P
ENO1, HSP48...
K01596
PCK1, JPM2, ...
Phosphoenol-pyruvate
Oxaloacetate
Citrate cycle
Pyruvate metabolism
CDC19, PYK1...
K00169...
K00174...
ThPP
2-Hydroxy-ethyl-ThPP
Acetyl-CoA
LAT1, ODP2, ...
S-Acetyl-dihydrolipoamide-E
PDB1...
THI3, KID1...
K00016
L-Lactate
Pyruvate
Propanoate metabolism
ACS1, FUN44...
K01905...
LPD1, HPD1...
Dihydro-lipoamide-E
Lipoamide-E
ALD5...
ALD3...
K00138
ADH5...
ADH7, ADHVII...
K14028...
K04022
K00114
Acetate
Acetaldehyde
Ethanol
00010 12/26/16
(c) Kanehisa Laboratories

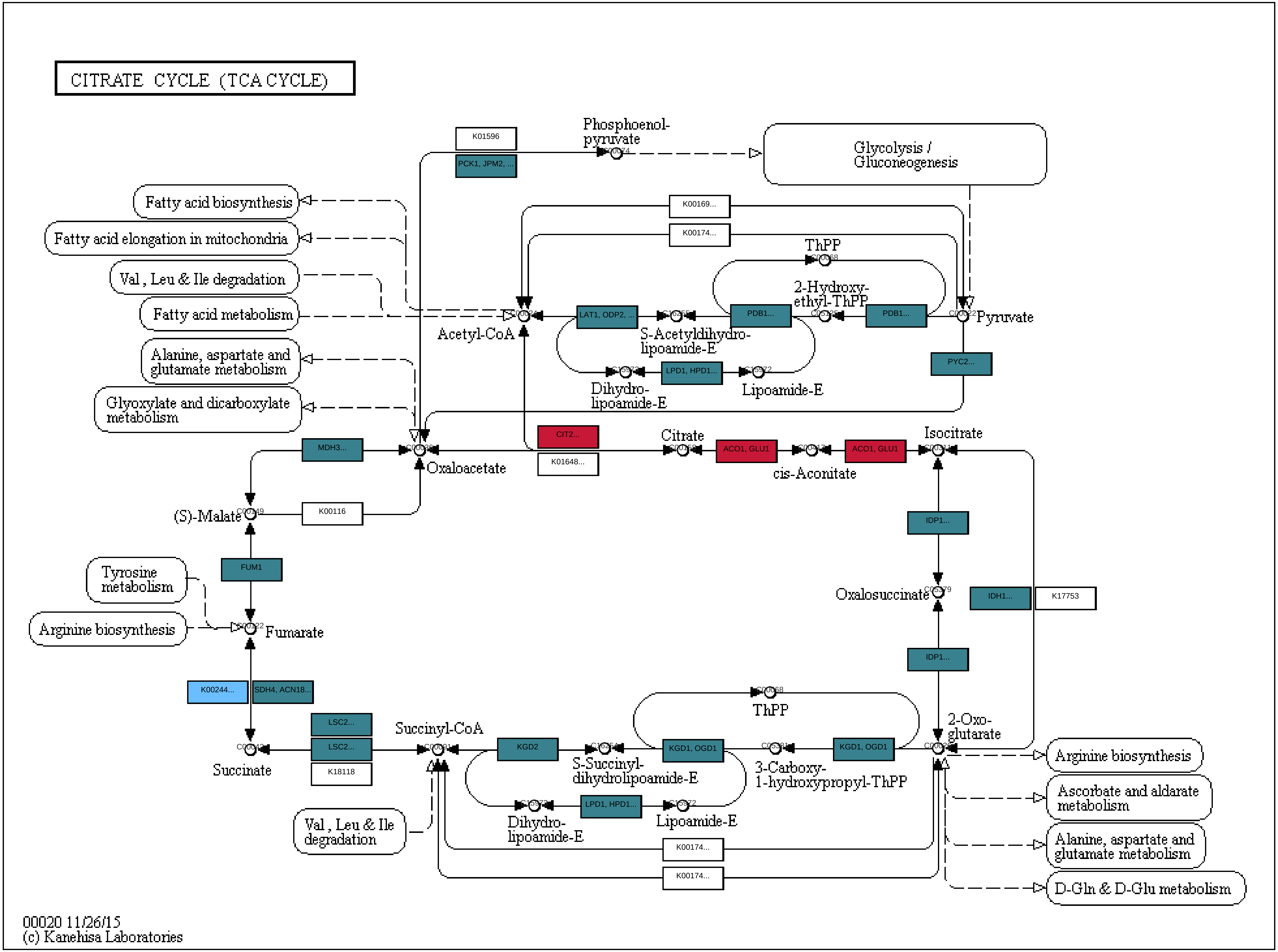

CITRATE CYCLE (TCA CYCLE)
K01596
PCK1, JPM2, ...
Phosphoenol-pyruvate
Glycolysis / Gluconeogenesis
Fatty acid biosynthesis
Fatty acid elongation in mitochondria
Val, Leu & Ile degradation
Fatty acid metabolism
Alanine, aspartate and glutamate metabolism
Glyoxylate and dicarboxylate metabolism
K00169...
K00174...
ThPP
2-Hydroxy-ethyl-ThPP
PDB1...
PDB1...
Pyruvate
LAT1, ODP2, ...
S-Acetyldihydro-lipoamide-E
Acetyl-CoA
LPD1, HPD1...
Dihydro-lipoamide-E
Lipoamide-E
PYC2...
CIT2...
K01648...
Citrate
ACO1, GLU1
ACO1, GLU1
cis-Aconitate
Isocitrate
MDH3...
Oxaloacetate
(S)-Malate
K00116
IDP1...
Oxalosuccinate
IDH1...
K17753
FUM1
Tyrosine metabolism
Arginine biosynthesis
Fumarate
IDP1...
K00244...
SDH4, ACN18...
LSC2...
LSC2...
K18118
Succinate
Succinyl-CoA
KGD2
S-Succinyl-dihydrolipoamide-E
KGD1, OGD1
ThPP
3-Carboxy-1-hydroxypropyl-ThPP
KGD1, OGD1
2-Oxo-glutarate
LPD1, HPD1...
Dihydro-lipoamide-E
Lipoamide-E
Val, Leu & Ile degradation
K00174...
K00174...
Arginine biosynthesis
Ascorbate and aldarate metabolism
Alanine, aspartate and glutamate metabolism
D-Gln & D-Glu metabolism
00020 11/26/15
(c) Kanehisa Laboratories

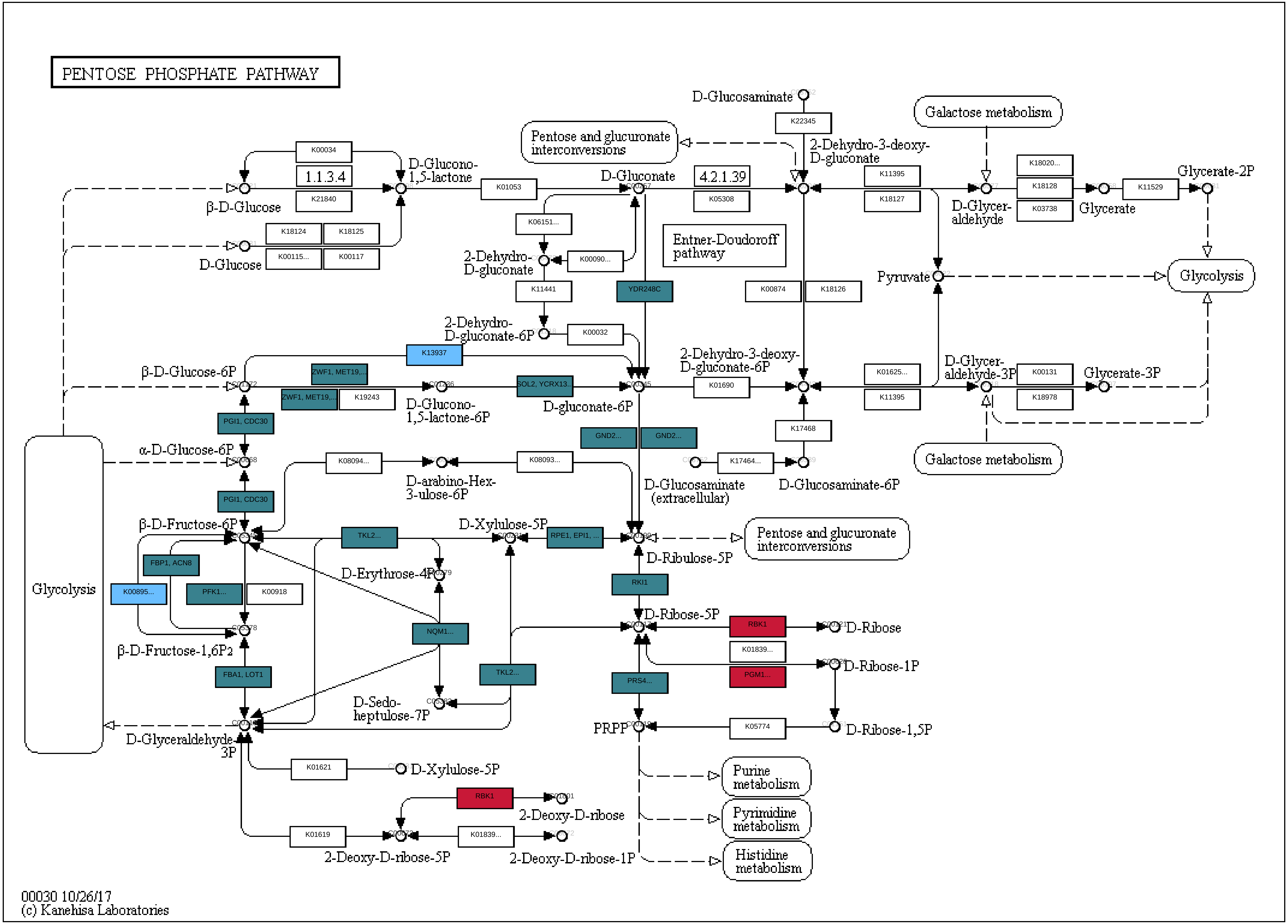

PENTOSE PHOSPHATE PATHWAY
D-Glucosaminate
K22345
Galactose metabolism
Pentose and glucuronate interconversions
2-Dehydro-3-deoxy-D-gluconate
K00034
D-Glucono-1,5-lactone
1.1.3.4
K21840
K01053
D-Gluconate
4.2.1.39
K05308
K11395
K18127
K18020...
K18128
K03738
K11529
Glycerate-2P
D-Glyceraldehyde
Glycerate
β-D-Glucose
K18124
K18125
K06151...
Entner-Doudoroff pathway
K00115...
K00117
D-Glucose
2-Dehydro-D-gluconate
K00090...
Pyruvate
Glycolysis
K11441
YDR248C
K00874
K18126
2-Dehydro-D-gluconate-6P
K00032
K13937
2-Dehydro-3-deoxy-D-gluconate-6P
β-D-Glucose-6P
ZWF1, MET19,...
SOL2, YCRX13...
K01690
K01625...
D-Glyceraldehyde-3P
K00131
Glycerate-3P
ZWF1, MET19,...
K19243
D-Glucono-1,5-lactone-6P
D-gluconate-6P
K11395
K18978
PGI1, CDC30
K17468
GND2...
GND2...
α-D-Glucose-6P
K08094...
K08093...
K17464...
Galactose metabolism
D-arabino-Hex-3-ulose-6P
D-Glucosaminate (extracellular)
D-Glucosamine-6P
PGI1, CDC30
β-D-Fructose-6P
D-Xylulose-5P
TKL2...
RPE1, EPI1, ...
Pentose and glucuronate interconversions
FBP1, ACN8
D-Ribulose-5P
D-Erythrose-4P
Glycolysis
K00895...
PFK1...
K00918
RKI1
NQM1...
D-Ribose-5P
RBK1
D-Ribose
β-D-Fructose-1,6P2
K01839...
FBA1, LOT1
TKL2...
D-Ribose-1P
PGM1...
PRS4...
D-Sedo-heptulose-7P
PRPP
K05774
D-Ribose-1,5P
D-Glyceraldehyde-3P
K01621
D-Xylulose-5P
Purine metabolism
RBK1
2-Deoxy-D-ribose
Pyrimidine metabolism
K01619
K01839...
2-Deoxy-D-ribose-5P
2-Deoxy-D-ribose-1P
Histidine metabolism
00030 10/26/17
(c) Kanehisa Laboratories

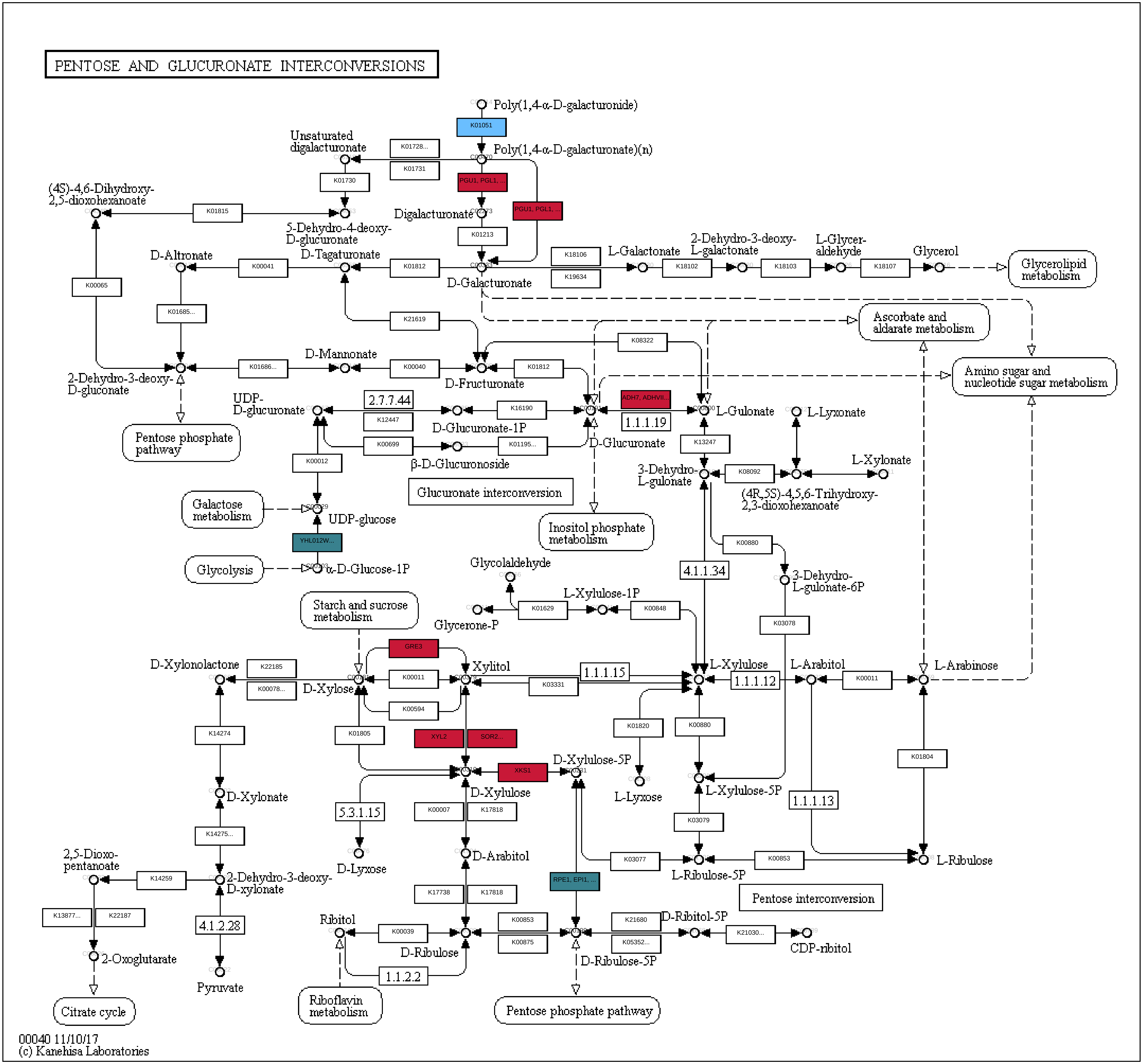

PENTOSE AND GLUCURONATE INTERCONVERSIONS
Poly(1,4-α-D-galacturonide)
K01051
Poly(1,4-α-D-galacturonate)(n)
Unsaturated digalacturonate
K01728...
K01731
K01730
PGU1, PGL1, ...
PGU1, PGL1, ...
(4S)-4,6-Dihydroxy-2,5-dioxohexanoate
K01815
5-Dehydro-4-deoxy-D-glucuronate
Digalacturonate
K01213
D-Altronate
K00041
D-Tagaturonate
K01812
D-Galacturonate
K18106
K19634
L-Galactonate
K18102
2-Dehydro-3-deoxy-L-galactonate
K18103
L-Glyceraldehyde
K18107
Glycerol
Glycerolipid metabolism
K00065
K01685...
K21619
Ascorbate and aldarate metabolism
K08322
D-Mannonate
K01686...
K00040
K01812
D-Fructuronate
Amino sugar and nucleotide sugar metabolism
2-Dehydro-3-deoxy-D-gluconate
Pentose phosphate pathway
UDP-D-glucuronate
2.7.7.44
K12447
K16190
D-Glucuronate-1P
ADH7, ADHVII...
1.1.1.19
L-Gulonate
L-Lyxonate
K00699
K01195...
β-D-Glucuronoside
D-Glucuronate
K13247
K00012
3-Dehydro-L-gulonate
K08092
L-Xylonate
(4R,5S)-4,5,6-Trihydroxy-2,3-dioxohexanoate
Glucuronate interconversion
Galactose metabolism
UDP-glucose
YHL012W...
Inositol phosphate metabolism
K00880
Glycolysis
α-D-Glucose-1P
Glycolaldehyde
4.1.1.34
3-Dehydro-L-gulonate-6P
L-Xylulose-1P
K01629
K00848
Glycerone-P
K03078
Starch and sucrose metabolism
GRE3
D-Xylonolactone
K22185
K00078...
D-Xylose
K00011
Xylitol
1.1.1.15
L-Xylulose
1.1.1.12
L-Arabitol
K00011
L-Arabinose
K03331
K00594
K14274
K01805
XYL2
SOR2...
K01820
K00880
K01804
XKS1
D-Xylulose-5P
D-Xylonate
D-Xylulose
L-Lyxose
L-Xylulose-5P
5.3.1.15
K00007
K17818
1.1.1.13
K14275...
K03079
D-Lyxose
D-Arabitol
2,5-Dioxopentanoate
K14259
2-Dehydro-3-deoxy-D-xylonate
RPE1, EPI1, ...
K03077
K00853
L-Ribulose
L-Ribulose-5P
K17738
K17818
Pentose interconversion
K13877...
K22187
4.1.2.28
Ribitol
K00039
K00853
K00875
K21680
K05352...
D-Ribitol-5P
K21030...
CDP-ribitol
D-Ribulose
D-Ribulose-5P
2-Oxoglutarate
1.1.2.2
Pyruvate
Citrate cycle
Riboflavin metabolism
Pentose phosphate pathway
00040 11/10/17
(c) Kanehisa Laboratories

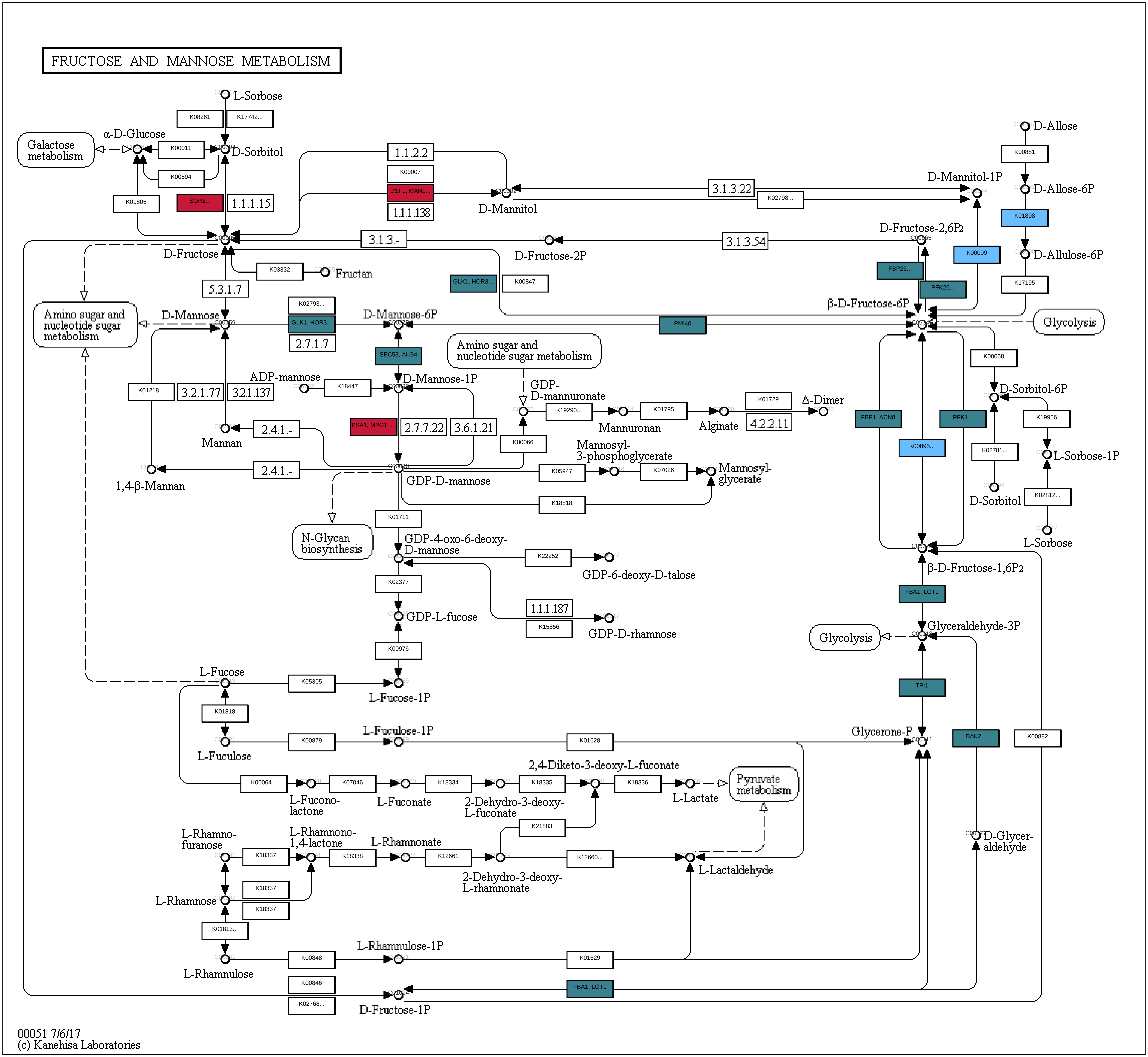
FRUCTOSE AND MANNOSE METABOLISM
L-Sorbose
K08261
K17742...
α-D-Glucose
Galactose metabolism
K00011
D-Sorbitol
K00594
1.1.2.2
K00007
DSF1, MAN1...
1.1.1.138
D-Mannitol
K01805
SOR2...
1.1.1.15
3.1.3.22
K02798...
D-Mannitol-1P
D-Allose
K00881
D-Allose-6P
K01808
D-Fructose
3.1.3.-
D-Fructose-2P
3.1.3.54
D-Fructose-2,6P2
K00009
D-Allulose-6P
K03332
Fructan
5.3.1.7
GLK1, HOR3...
K00847
FBP26...
PFK26...
K17195
Amino sugar and nucleotide sugar metabolism
D-Mannose
K02793...
GLK1, HOR3...
2.7.1.7
D-Mannose-6P
PMI40
β-D-Fructose-6P
Glycolysis
SEC53, ALG4
Amino sugar and nucleotide sugar metabolism
K00068
K01218...
3.2.1.77
3.2.1.137
ADP-mannose
K18447
D-Mannose-1P
GDP-D-mannuronate
K19290...
Mannuronan
K01795
Alginate
K01729
4.2.2.11
Δ-Dimer
D-Sorbitol-6P
FBP1, ACN8
PFK1...
K19956
Mannan
2.4.1.-
PSA1, MPG1, ...
2.7.7.22
3.6.1.21
K00066
Mannosyl-3-phosphoglycerate
K00895...
K02781...
L-Sorbose-1P
1,4-β-Mannan
2.4.1.-
GDP-D-mannose
K05947
K07026
Mannosyl-glycerate
K18818
D-Sorbitol
K02812...
N-Glycan biosynthesis
K01711
GDP-4-oxo-6-deoxy-D-mannose
K22252
GDP-6-deoxy-D-talose
β-D-Fructose-1,6P2
L-Sorbose
K02377
1.1.1.187
K15856
GDP-D-rhamnose
FBA1, LOT1
GDP-L-fucose
Glyceraldehyde-3P
Glycolysis
K00976
L-Fucose
K05305
L-Fucose-1P
TPI1
K01818
L-Fuculose-1P
L-Fuculose
K00879
K01628
Glycerone-P
DAK2...
K00882
2,4-Diketo-3-deoxy-L-fuconate
K00064...
K07046
K18334
K18335
K18336
Pyruvate metabolism
L-Fucono-lactone
L-Fuconate
2-Dehydro-3-deoxy-L-fuconate
L-Lactate
K21883
L-Rhamno-furanose
K18337
L-Rhamnono-1,4-lactone
K18338
L-Rhamnonate
K12661
K12660...
D-Glycer-aldehyde
2-Dehydro-3-deoxy-L-rhamnonate
L-Lactaldehyde
K18337
L-Rhamnose
K18337
K01813...
L-Rhamnulose-1P
K00848
K01629
L-Rhamnulose
K00846
FBA1, LOT1
K02768...
D-Fructose-1P
00051 7/6/17
(c) Kanehisa Laboratories

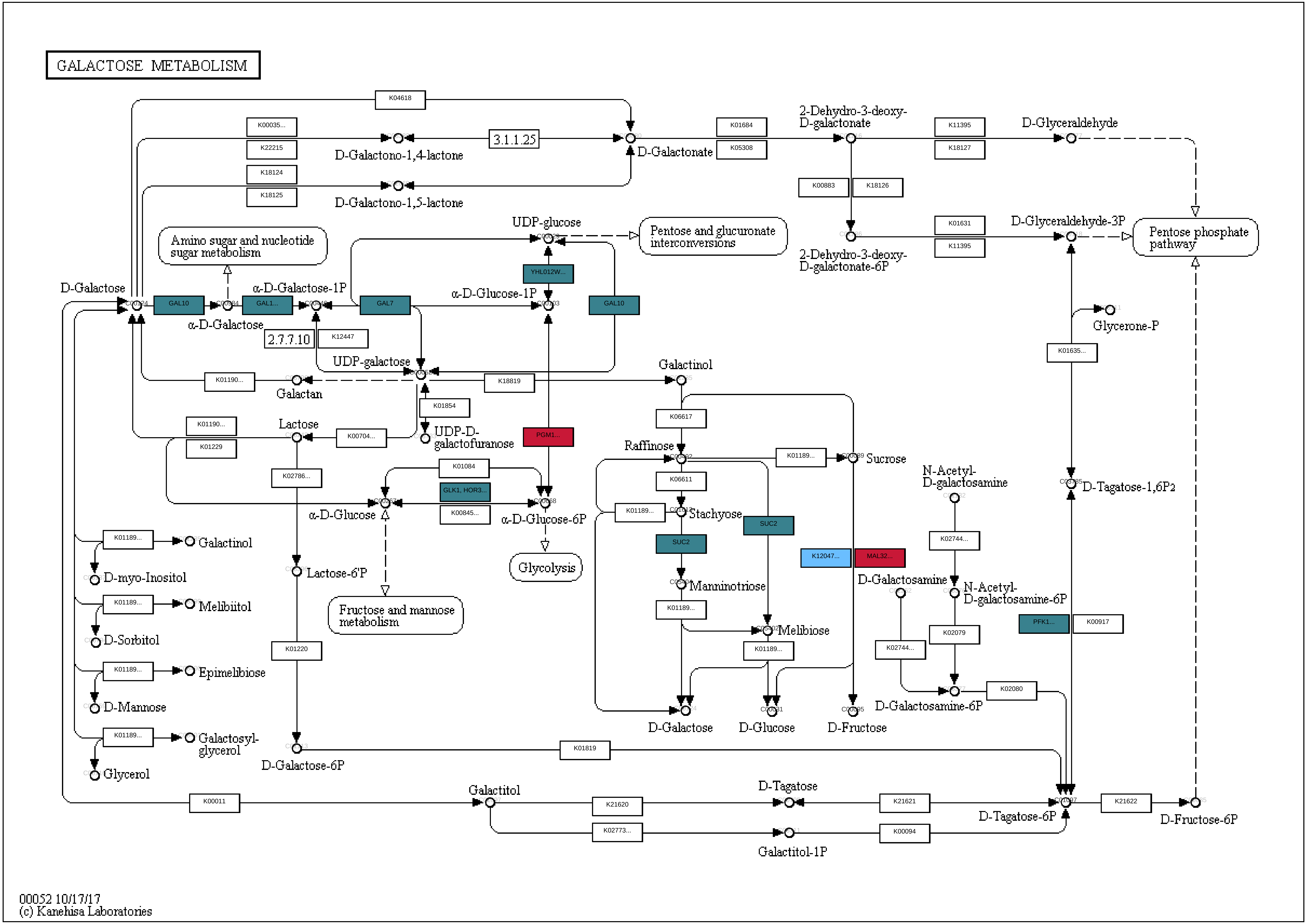
GALACTOSE METABOLISM
K04618
K00035...
K22215
K18124
K18125
3.1.1.25
D-Galactono-1,4-lactone
D-Galactono-1,5-lactone
D-Galactonate
K01684
K05308
2-Dehydro-3-deoxy-D-galactonate
K11395
K18127
D-Glyceraldehyde
K00883
K18126
K01631
K11395
D-Glyceraldehyde-3P
2-Dehydro-3-deoxy-D-galactonate-6P
Pentose phosphate pathway
Pentose and glucuronate interconversions
Amino sugar and nucleotide sugar metabolism
UDP-glucose
YHL012W...
D-Galactose
GAL10
α-D-Galactose
GAL1...
α-D-Galactose-1P
GAL7
α-D-Glucose-1P
GAL10
2.7.7.10
K12447
UDP-galactose
Glycerone-P
K01635...
K01190...
Galactan
K18819
Galactinol
K01854
UDP-D-galactofuranose
K01190...
K01229
Lactose
K00704...
PGM1...
K06617
Raffinose
K01189...
Sucrose
N-Acetyl-D-galactosamine
K02786...
K01084
GLK1, HOR3...
K00845...
α-D-Glucose
α-D-Glucose-6P
K06611
K01189...
Stachyose
SUC2
D-Tagatose-1,6P2
K01189...
Galactinol
D-myo-Inositol
Glycolysis
SUC2
K12047...
MAL32...
K02744...
Lactose-6P
Manninotriose
D-Galactosamine
N-Acetyl-D-galactosamine-6P
K01189...
Melibiitol
D-Sorbitol
Fructose and mannose metabolism
K01189...
Melibiose
PFK1...
K00917
K02079
K01220
K01189...
K02744...
K01189...
Epimelibiose
D-Mannose
K02080
D-Galactosamine-6P
D-Galactose
D-Glucose
D-Fructose
K01189...
Galactosyl-glycerol
Glycerol
K01819
D-Galactose-6P
K00011
Galactitol
K21620
D-Tagatose
K21621
K21622
D-Tagatose-6P
D-Fructose-6P
K02773...
K00094
Galactitol-1P
00052 10/17/17
(c) Kanehisa Laboratories

**NO REFERENCE PATHWAY AVAILABLE:**

**map 53 Ascorbate and aldarate metabolism**

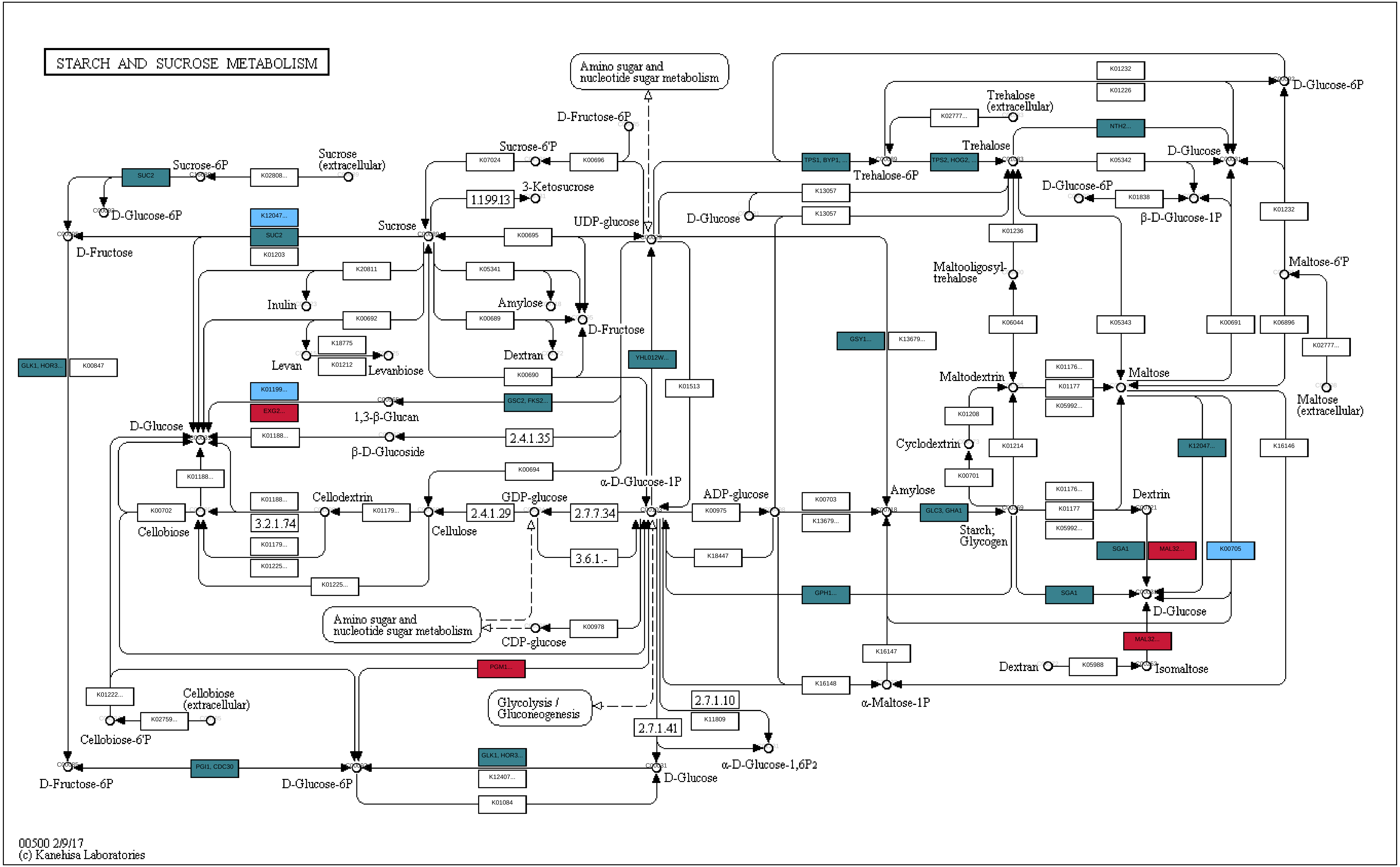

STARCH AND SUCROSE METABOLISM
Amino sugar and nucleotide sugar metabolism
D-Fructose-6P
Sucrose-6'P
K07024
K00696
3-Ketosucrose
1.1.99.13
Sucrose
K00695
UDP-glucose
Sucrose-6P
SUC2
K02808...
Sucrose (extracellular)
D-Glucose-6P
K12047...
D-Fructose
K01203
K20811
Inulin
K05341
Amylose
K00692
K00689
D-Fructose
Levan
K18775
K01212
Levanbiose
Dextran
K00690
GLK1, HOR3...
K00847
K01199...
EXG2...
GSC2, FKS2...
1,3-β-Glucan
D-Glucose
K01188...
2.4.1.35
β-D-Glucoside
K00694
K01188...
Cellodextrin
K01179...
GDP-glucose
2.4.1.29
2.7.7.34
α-D-Glucose-1P
K00702
Cellobiose
3.2.1.74
Cellulose
K01179...
K01225...
K01225...
3.6.1.-
YHL012W...
K01513
Amino sugar and nucleotide sugar metabolism
K00978
CDP-glucose
PGM1...
K01222...
Cellobiose (extracellular)
K02759...
Cellobiose-6'P
Glycolysis / Gluconeogenesis
2.7.1.10
K11809
2.7.1.41
α-D-Glucose-1,6P2
D-Fructose-6P
PGI1, CDC30
D-Glucose-6P
GLK1, HOR3...
K12407...
K01084
D-Glucose
K01232
K01226
D-Glucose-6P
Trehalose (extracellular)
K02777...
NTH2...
Trehalose
D-Glucose
TPS1, BYP1, ...
TPS2, HOG2, ...
K05342
Trehalose-6P
K13057
K13057
D-Glucose
D-Glucose-6P
K01838
β-D-Glucose-1P
K01232
K01236
Maltooligosyl-trehalose
Maltose-6'P
K06044
K05343
K00691
K06896
GSY1...
K13679...
K02777...
Maltodextrin
K01176...
K01177
K05992...
Maltose
Maltose (extracellular)
K01208
Cyclodextrin
K01214
K12047...
K16146
K00701
ADP-glucose
K00703
K13679...
K00975
Amylose
GLC3, GHA1
Starch; Glycogen
K01176...
K01177
K05992...
Dextrin
K18447
SGA1
MAL32...
K00705
GPH1...
SGA1
D-Glucose
MAL32...
K16147
Dextran
K05988
Isomaltose
K16148
α-Maltose-1P
00500 2/9/17
(c) Kanehisa Laboratories

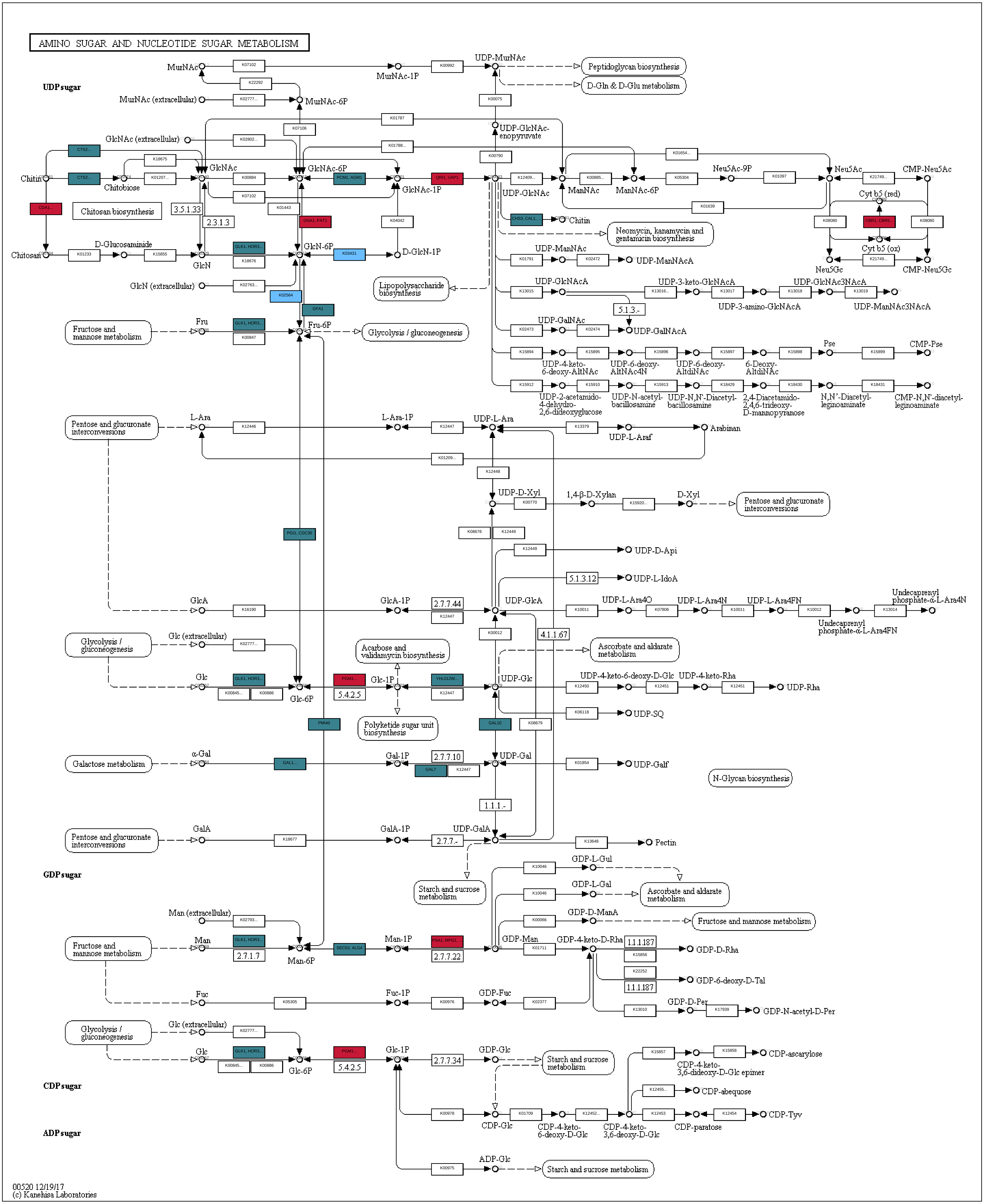

AMINO SUGAR AND NUCLEOTIDE SUGAR METABOLISM
UDP sugar
MurNAc
MurNAc-1P
UDP-MurNAc
Peptidoglycan biosynthesis
D-Gln & D-Glu metabolism
MurNAc (extracellular)
MurNAc-6P
UDP-GlcNAc-enopyruvate
GlcNAc (extracellular)
Chitin
Chitobiose
GlcNAc
GlcNAc-6P
GlcNAc-1P
UDP-GlcNAc
ManNAc
ManNAc-6P
Neu5Ac-9P
Neu5Ac
CMP-Neu5Ac
Cyt b5 (red)
Cyt b5 (ox)
Neu5Gc
CMP-Neu5Gc
Chitosan biosynthesis
3.5.1.33
2.3.1.3
Chitin
Neomycin, kanamycin and gentamicin biosynthesis
Chitosan
D-Glucosaminide
GlcN
GlcN-6P
D-GlcN-1P
UDP-ManNAc
UDP-ManNAcA
GlcN (extracellular)
Lipopolysaccharide biosynthesis
UDP-GlcNAcA
UDP-3-keto-GlcNAcA
UDP-GlcNAc3NAcA
UDP-3-amino-GlcNAcA
UDP-ManNAc3NAcA
5.1.3.-
Fructose and mannose metabolism
Fru
Fru-6P
Glycolysis / gluconeogenesis
UDP-GalNAc
UDP-GalNAcA
Pse
CMP-Pse
UDP-4-keto-6-deoxy-AltNAc
UDP-6-deoxy-AltNAc4N
UDP-6-deoxy-AltdiNAc
6-Deoxy-AltdiNAc
UDP-2-acetamido-4-dehydro-2,6-dideoxyglucose
UDP-N-acetyl-bacillosamine
UDP-N,N'-Diacetyl-bacillosamine
2,4-Diacetamido-2,4,6-trideoxy-D-mannopyranose
N,N'-Diacetyl-legionaminate
CMP-N,N'-diacetyl-legionaminate
Pentose and glucuronate interconversions
L-Ara
L-Ara-1P
UDP-L-Ara
UDP-L-Araf
Arabinan
UDP-D-Xyl
1,4-β-D-Xylan
D-Xyl
Pentose and glucuronate interconversions
UDP-D-Api
5.1.3.12
UDP-L-IdoA
GlcA
GlcA-1P
2.7.7.44
UDP-GlcA
UDP-L-Ara4O
UDP-L-Ara4N
UDP-L-Ara4FN
Undecaprenyl phosphate-α-L-Ara4N
Undecaprenyl phosphate-α-L-Ara4FN
4.1.1.67
Glycolysis / gluconeogenesis
Glc (extracellular)
Acarbose and validamycin biosynthesis
Ascorbate and aldarate metabolism
Glc
Glc-6P
Glc-1P
5.4.2.5
UDP-Glc
UDP-4-keto-6-deoxy-D-Glc
UDP-4-keto-Rha
UDP-Rha
UDP-SQ
Polyketide sugar unit biosynthesis
Galactose metabolism
α-Gal
Gal-1P
2.7.7.10
UDP-Gal
UDP-Galf
N-Glycan biosynthesis
1.1.1.-
Pentose and glucuronate interconversions
GalA
GalA-1P
2.7.7.-
UDP-GalA
Pectin
GDP sugar
GDP-L-Gul
Starch and sucrose metabolism
GDP-L-Gal
Ascorbate and aldarate metabolism
GDP-D-ManA
Fructose and mannose metabolism
Man (extracellular)
Fructose and mannose metabolism
Man
2.7.1.7
Man-6P
Man-1P
2.7.7.22
GDP-Man
GDP-4-keto-D-Rha
1.1.1.187
GDP-D-Rha
GDP-6-deoxy-D-Tal
1.1.1.187
GDP-D-Per
GDP-N-acetyl-D-Per
Fuc
Fuc-1P
GDP-Fuc
Glycolysis / gluconeogenesis
Glc (extracellular)
Glc
Glc-6P
5.4.2.5
Glc-1P
2.7.7.34
GDP-Glc
Starch and sucrose metabolism
CDP-ascarylose
CDP-4-keto-3,6-dideoxy-D-Glc epimer
CDP sugar
CDP-abequose
ADP sugar
CDP-Glc
CDP-4-keto-6-deoxy-D-Glc
CDP-4-keto-3,6-deoxy-D-Glc
CDP-paratose
CDP-Tyv
ADP-Glc
Starch and sucrose metabolism
00520 12/19/17
(c) Kanehisa Laboratories

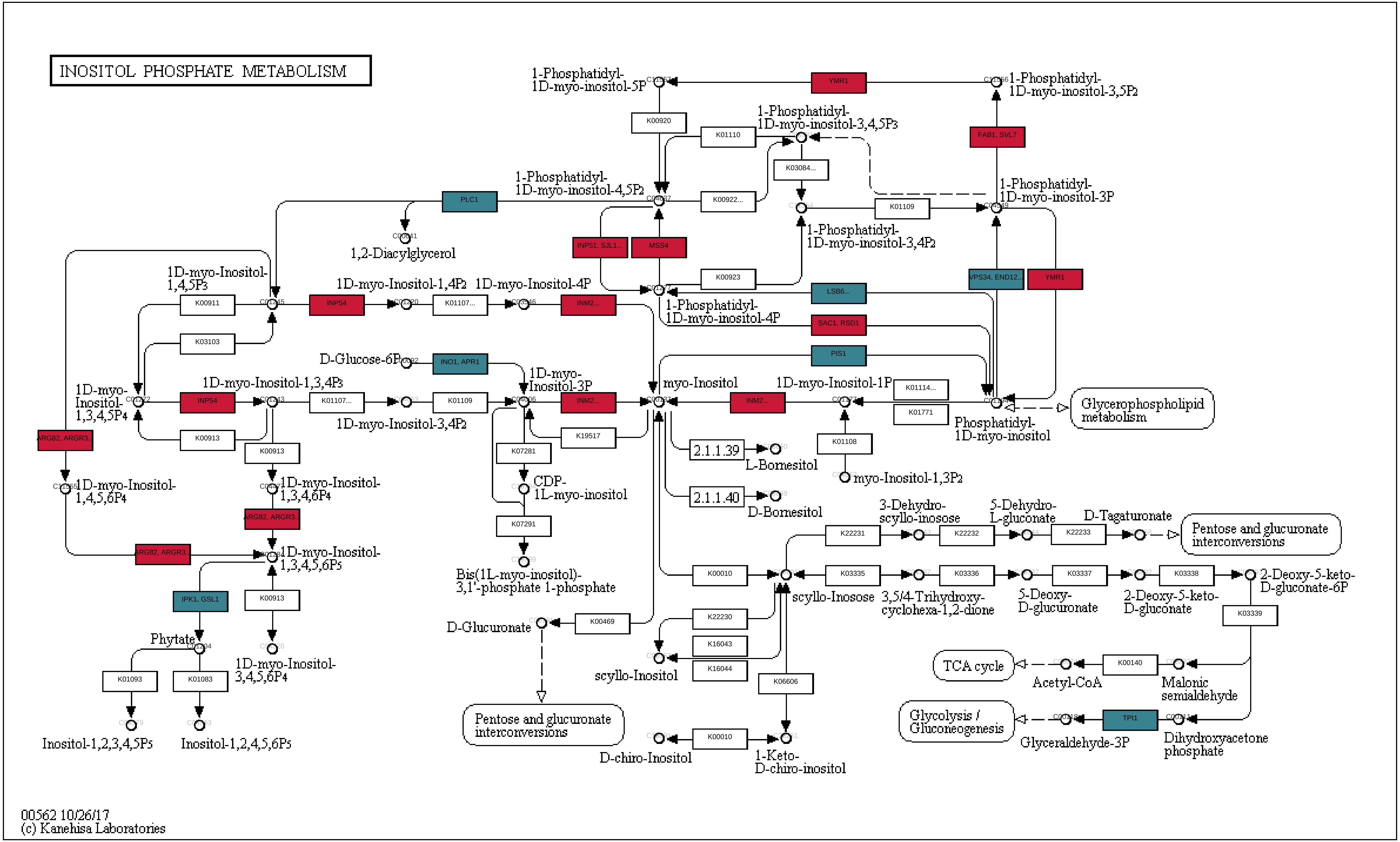
INOSITOL PHOSPHATE METABOLISM
1-Phosphatidyl-1D-myo-inositol-5P
YMR1
1-Phosphatidyl-1D-myo-inositol-3,5P2
K00920
K01110
1-Phosphatidyl-1D-myo-inositol-3,4,5P3
FAB1, SVL7
K03084...
1-Phosphatidyl-1D-myo-inositol-4,5P2
PLC1
K00922...
1-Phosphatidyl-1D-myo-inositol-3P
K01109
1,2-Diacylglycerol
INP51, SJL1...
MSS4
1-Phosphatidyl-1D-myo-inositol-3,4P2
1D-myo-Inositol-1,4,5P3
1D-myo-Inositol-1,4P2
1D-myo-Inositol-4P
K00923
VPS34, END12...
YMR1
LSB6...
K00911
INP54
K01107...
INM2...
1-Phosphatidyl-1D-myo-inositol-4P
SAC1, RSD1
K03103
D-Glucose-6P
INO1, APR1
PIS1
1D-myo-Inositol-3P
1D-myo-Inositol-1,3,4P3
myo-Inositol
1D-myo-Inositol-1P
K01114...
1D-myo-Inositol-1,3,4,5P4
INP54
K01107...
K01109
INM2...
INM2...
K01771
Glycerophospholipid metabolism
Phosphatidyl-1D-myo-inositol
ARG82, ARGR3...
K00913
1D-myo-Inositol-3,4P2
K19517
K00913
K07281
2.1.1.39
L-Bornesitol
K01108
myo-Inositol-1,3P2
1D-myo-Inositol-1,4,5,6P4
1D-myo-Inositol-1,3,4,6P4
CDP-1L-myo-inositol
2.1.1.40
D-Bornesitol
ARG82, ARGR3...
3-Dehydro-scyllo-inosose
5-Dehydro-L-gluconate
D-Tagaturonate
K07291
K22231
K22232
K22233
Pentose and glucuronate interconversions
ARG82, ARGR3...
1D-myo-Inositol-1,3,4,5,6P5
Bis(1L-myo-inositol)-3,1'-phosphate 1-phosphate
K00010
K03335
K03336
K03337
K03338
2-Deoxy-5-keto-D-gluconate-6P
IPK1, GSL1
K00913
scyllo-Inosose
3,5/4-Trihydroxy-cyclohexa-1,2-dione
5-Deoxy-D-glucuronate
2-Deoxy-5-keto-D-gluconate
K03339
D-Glucuronate
K00469
K22230
Phytate
K16043
TCA cycle
K00140
K01093
K01083
1D-myo-Inositol-3,4,5,6P4
scyllo-Inositol
K16044
Acetyl-CoA
Malonic semialdehyde
K06606
Pentose and glucuronate interconversions
Glycolysis / Gluconeogenesis
TPI1
Inositol-1,2,3,4,5P5
Inositol-1,2,4,5,6P5
K00010
Glyceraldehyde-3P
Dihydroxyacetone phosphate
D-chiro-Inositol
1-Keto-D-chiro-inositol
00562 10/26/17
(c) Kanehisa Laboratories

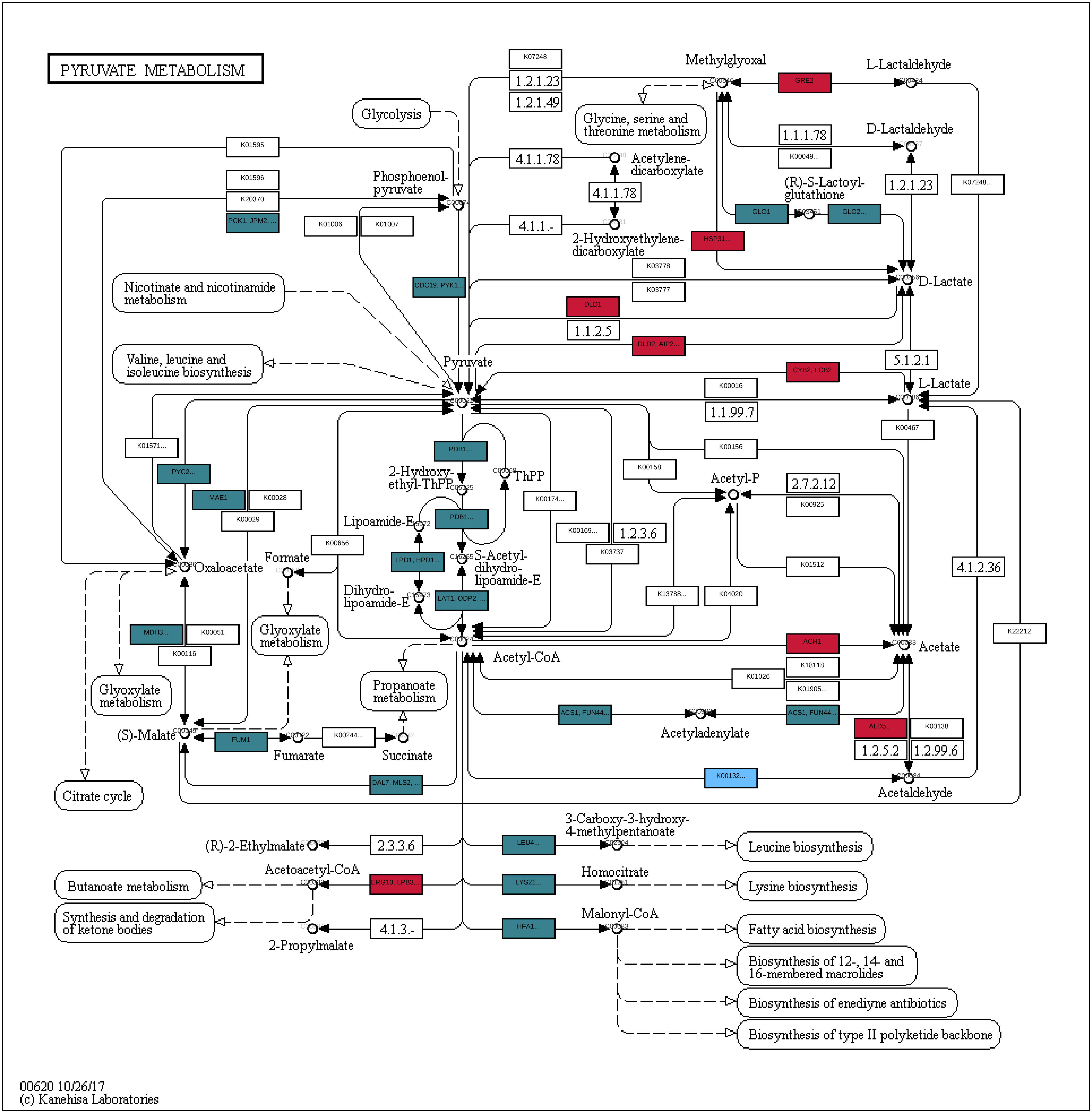
PYRUVATE METABOLISM
Glycolysis
Phosphoenol-pyruvate
Methylglyoxal
L-Lactaldehyde
D-Lactaldehyde
Glycine, serine and threonine metabolism
Acetylene-dicarboxylate
2-Hydroxyethylene-dicarboxylate
(R)-S-Lactoyl-glutathione
D-Lactate
L-Lactate
Pyruvate
Nicotinate and nicotinamide metabolism
Valine, leucine and isoleucine biosynthesis
2-Hydroxy-ethyl-ThPP
ThPP
Lipoamide-E
S-Acetyl-dihydro-lipoamide-E
Dihydro-lipoamide-E
Acetyl-P
Acetyl-CoA
Acetate
Acetyladenylate
Acetaldehyde
Oxaloacetate
Formate
Glyoxylate metabolism
Propanoate metabolism
(S)-Malate
Fumarate
Succinate
Citrate cycle
3-Carboxy-3-hydroxy-4-methylpentanoate
(R)-2-Ethylmalate
Acetoacetyl-CoA
Homocitrate
Malonyl-CoA
2-Propylmalate
Leucine biosynthesis
Lysine biosynthesis
Butanoate metabolism
Synthesis and degradation of ketone bodies
Fatty acid biosynthesis
Biosynthesis of 12-, 14- and 16-membered macrolides
Biosynthesis of enediyne antibiotics
Biosynthesis of type II polyketide backbone
K07248
1.2.1.23
1.2.1.49
GRE2
1.1.1.78
K00049...
1.2.1.23
K07248...
K01595
K01596
K20370
PCK1, JPM2, ...
K01006
K01007
4.1.1.78
4.1.1.78
4.1.1.-
GLO1
GLO2...
HSP31...
K03778
K03777
CDC19, PYK1...
DLD1
1.1.2.5
DLD2, AIP2...
CYB2, FCB2
5.1.2.1
K00016
1.1.99.7
K00467
K01571...
PYC2...
MAE1
K00028
K00029
PDB1...
PDB1...
K00174...
K00156
K00158
2.7.2.12
K00925
K00169...
1.2.3.6
K03737
K00656
LPD1, HPD1...
LAT1, ODP2, ...
K13788...
K04020
K01512
4.1.2.36
K22212
MDH3...
K00051
K00116
ACH1
K18118
K01026
K01905...
ACS1, FUN44...
ACS1, FUN44...
ALD5...
K00138
1.2.5.2
1.2.99.6
FUM1
K00244...
DAL7, MLS2, ...
K00132...
2.3.3.6
LEU4...
ERG10, LPB3,...
LYS21...
4.1.3.-
HFA1...
00620 10/26/17
(c) Kanehisa Laboratories

# GLYOXYLATE AND DICARBOXYLATE METABOLISM

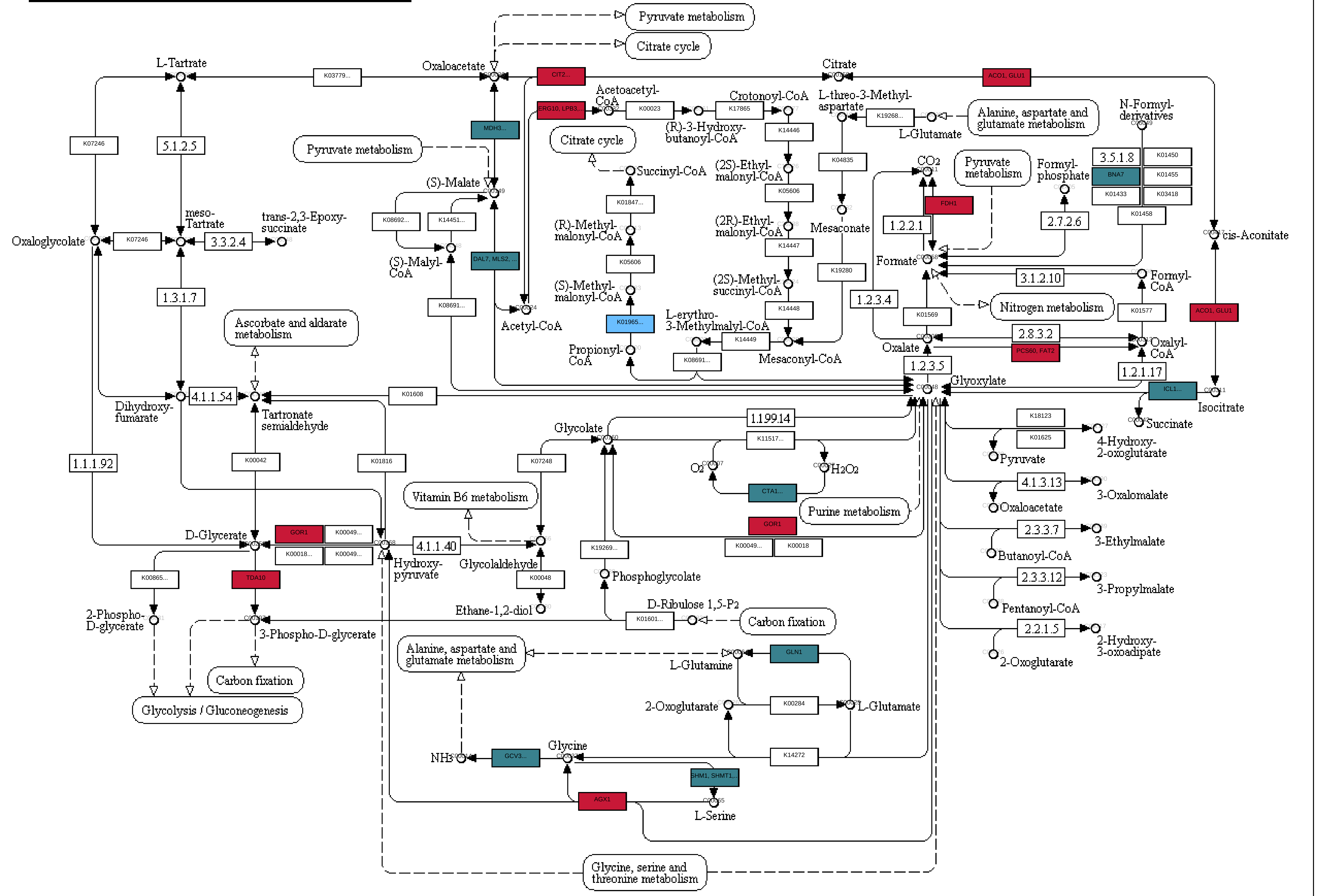


00630 11/22/17

(c) Kanehisa Laboratories

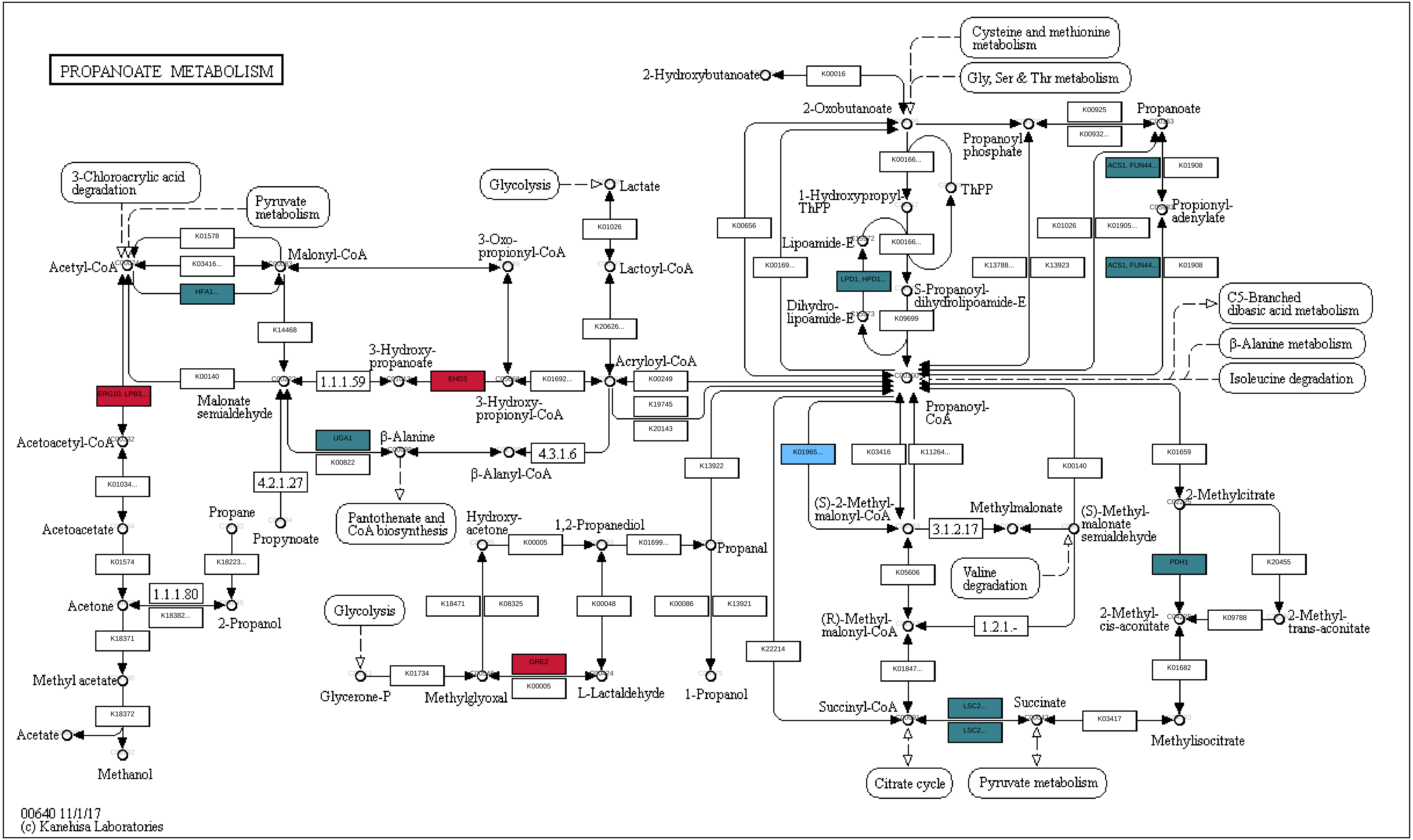
PROPANOATE METABOLISM
Cysteine and methionine metabolism
Gly, Ser & Thr metabolism
2-Hydroxybutanoate
K00016
2-Oxobutanoate
K00925
K00932...
Propanoate
Propanoyl phosphate
K00166...
ThPP
1-Hydroxypropyl-ThPP
ACS1, FUN44...
K01908
Propionyl-adenylate
K00656
Lipoamide-E
K00166...
K01026
K01905...
K00169...
LPD1, HPD1...
K13788...
K13923
ACS1, FUN44...
K01908
S-Propanoyl-dihydrolipoamide-E
Dihydro-lipoamide-E
K09699
C5-Branched dibasic acid metabolism
β-Alanine metabolism
Isoleucine degradation
3-Chloroacrylic acid degradation
Pyruvate metabolism
Glycolysis
Lactate
K01026
Lactoyl-CoA
K01578
K03416...
HFA1...
Acetyl-CoA
Malonyl-CoA
3-Oxo-propionyl-CoA
K14468
K20626...
Acryloyl-CoA
3-Hydroxy-propanoate
K00140
1.1.1.59
EHD3
K01692...
K00249
K19745
K20143
ERG10, LPB3,...
Malonate semialdehyde
3-Hydroxy-propionyl-CoA
Propanoyl-CoA
UGA1
β-Alanine
K00822
4.3.1.6
β-Alanyl-CoA
Acetoacetyl-CoA
K01034...
4.2.1.27
K13922
K01965...
K03416
K11264...
K00140
K01659
2-Methylcitrate
Acetoacetate
Propane
Propynoate
Pantothenate and CoA biosynthesis
Hydroxy-acetone
1,2-Propanediol
K00005
K01699...
Propanal
(S)-2-Methyl-malonyl-CoA
Methylmalonate
3.1.2.17
(S)-Methyl-malonate semialdehyde
K01574
K18223...
K05606
Valine degradation
PDH1
K20455
1.1.1.80
Acetone
K18382...
2-Propanol
Glycolysis
K18471
K08325
K00048
K00086
K13921
(R)-Methyl-malonyl-CoA
1.2.1.-
2-Methyl-cis-aconitate
K09788
2-Methyl-trans-aconitate
K18371
K22214
Methyl acetate
GRE2
K01734
K00005
Glycerone-P
Methylglyoxal
L-Lactaldehyde
1-Propanol
K01847...
K01682
K18372
Succinyl-CoA
LSC2...
LSC2...
Succinate
K03417
Acetate
Methylisocitrate
Methanol
Citrate cycle
Pyruvate metabolism
00640 11/1/17
(c) Kanehisa Laboratories

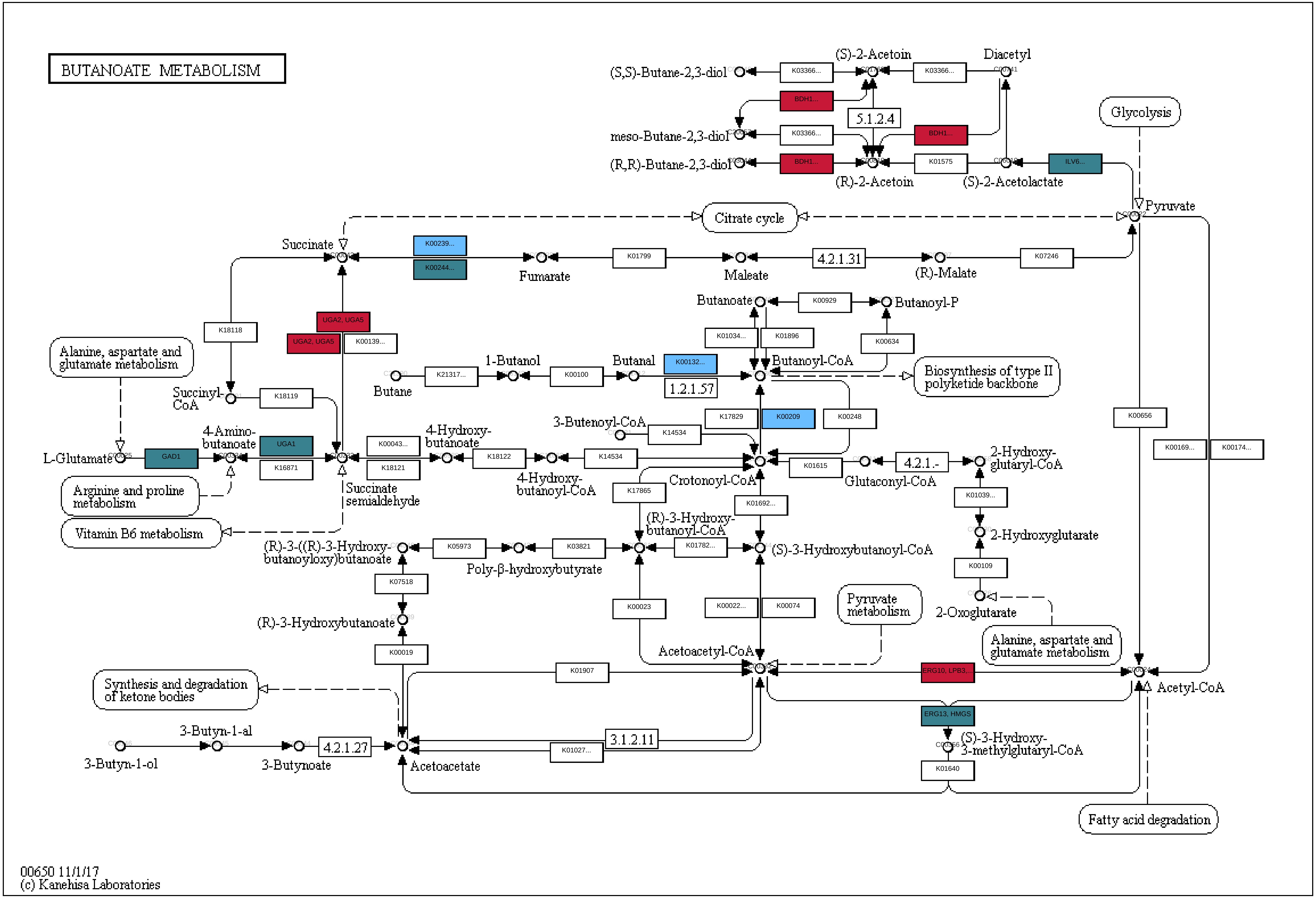
BUTANOATE METABOLISM
(S)-2-Acetoin
Diacetyl
(S,S)-Butane-2,3-diol
K03366...
BDH1...
5.1.2.4
meso-Butane-2,3-diol
(R,R)-Butane-2,3-diol
K01575
ILV6...
(R)-2-Acetoin
(S)-2-Acetolactate
Glycolysis
Pyruvate
Citrate cycle
Succinate
K00239...
K00244...
Fumarate
K01799
Maleate
4.2.1.31
(R)-Malate
K07246
Butanoate
K00929
Butanoyl-P
K18118
UGA2, UGA5
K00139...
K01034...
K01896
K00634
Alanine, aspartate and glutamate metabolism
Butanoyl-CoA
K21317...
1-Butanol
K00100
Butanal
K00132...
1.2.1.57
Biosynthesis of type II polyketide backbone
Butane
Succinyl-CoA
K18119
K17829
K00209
K00248
K00656
3-Butenoyl-CoA
K14534
4-Amino-butanoate
UGA1
K00043...
4-Hydroxy-butanoate
K00169...
K00174...
L-Glutamate
GAD1
K16871
K18121
K18122
K14534
K01615
Glutaconyl-CoA
4.2.1.-
2-Hydroxy-glutaryl-CoA
Arginine and proline metabolism
Succinate semialdehyde
4-Hydroxy-butanoyl-CoA
Crotonoyl-CoA
K17865
K01692...
K01039...
Vitamin B6 metabolism
(R)-3-Hydroxy-butanoyl-CoA
2-Hydroxyglutarate
(R)-3-((R)-3-Hydroxy-butanoyloxy)butanoate
K05973
K03821
K01782...
(S)-3-Hydroxybutanoyl-CoA
Poly-β-hydroxybutyrate
K00109
K07518
K00023
K00022...
K00074
Pyruvate metabolism
2-Oxoglutarate
(R)-3-Hydroxybutanoate
Alanine, aspartate and glutamate metabolism
K00019
Acetoacetyl-CoA
K01907
ERG10, LPB3...
Synthesis and degradation of ketone bodies
Acetyl-CoA
ERG13, HMGS
3-Butyn-1-al
(S)-3-Hydroxy-3-methylglutaryl-CoA
3.1.2.11
3-Butyn-1-ol
4.2.1.27
K01027...
3-Butynoate
Acetoacetate
K01640
Fatty acid degradation
00650 11/1/17
(c) Kanehisa Laboratories

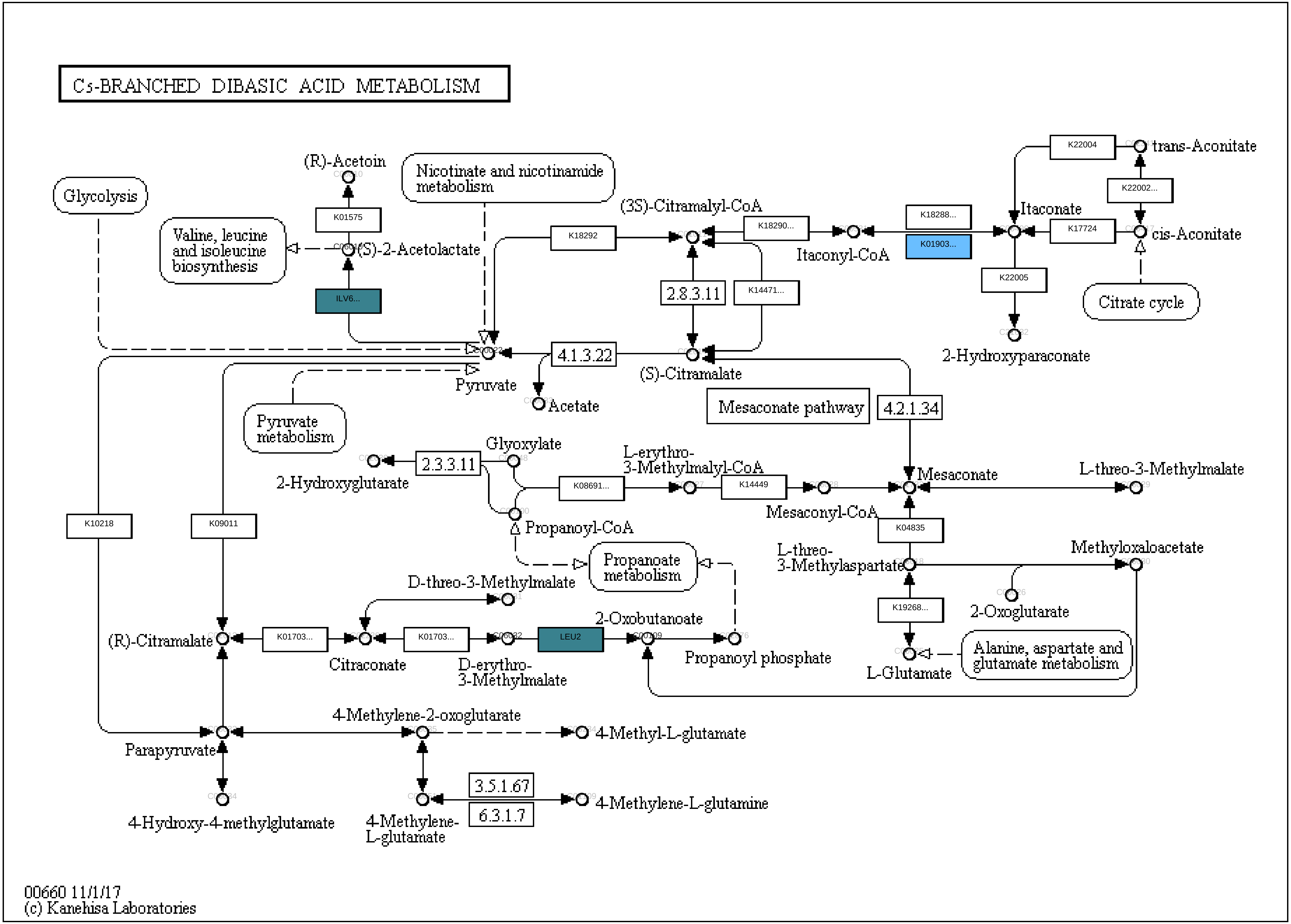
C5-BRANCHED DIBASIC ACID METABOLISM
Glycolysis
(R)-Acetoin
K01575
Valine, leucine and isoleucine biosynthesis
(S)-2-Acetolactate
ILV6...
Nicotinate and nicotinamide metabolism
K18292
(3S)-Citramalyl-CoA
K18290...
K18288...
K01903...
Itaconyl-CoA
2.8.3.11
K14471...
K22004
trans-Aconitate
K22002...
Itaconate
K17724
cis-Aconitate
K22005
Citrate cycle
2-Hydroxyparaconate
C00022
Pyruvate
4.1.3.22
Acetate
(S)-Citramalate
Mesaconate pathway
4.2.1.34
Pyruvate metabolism
2.3.3.11
Glyoxylate
2-Hydroxyglutarate
L-erythro-3-Methylmalyl-CoA
K08691...
K14449
Mesaconate
L-threo-3-Methylmalate
Mesaconyl-CoA
Propanoyl-CoA
K04835
K10218
K09011
L-threo-3-Methylaspartate
Methyloxaloacetate
Propanoate metabolism
2-Oxoglutarate
D-threo-3-Methylmalate
K19268...
2-Oxobutanoate
(R)-Citramalate
K01703...
Citraconate
K01703...
D-erythro-3-Methylmalate
LEU2
C00109
Propanoyl phosphate
L-Glutamate
Alanine, aspartate and glutamate metabolism
4-Methylene-2-oxoglutarate
4-Methyl-L-glutamate
Parapyruvate
3.5.1.67
6.3.1.7
4-Methylene-L-glutamine
4-Hydroxy-4-methylglutamate
4-Methylene-L-glutamate
00660 11/1/17
(c) Kanehisa Laboratories

# 2. Energy metabolism

| MAP | PATHWAY |
|---|---|
| **190** | Oxidative phosphorylation |
| **680** | Methane metabolism |
| **910** | Nitrogen metabolism |
| **920** | Sulfur metabolism |

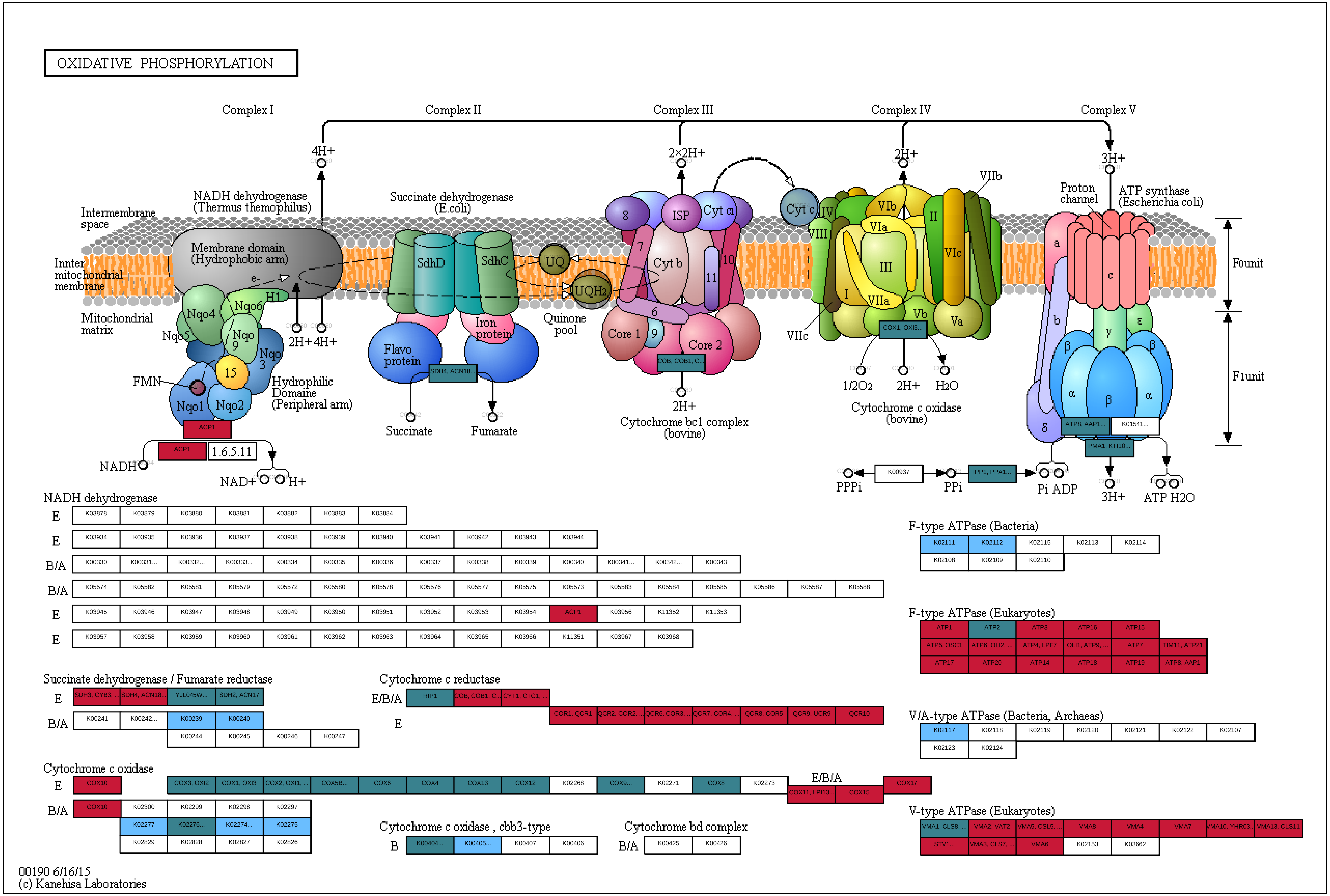

OXIDATIVE PHOSPHORYLATION
Complex I
Complex II
Complex III
Complex IV
Complex V
4H+
2×2H+
2H+
3H+
NADH dehydrogenase
(Thermus themophilus)
Succinate dehydrogenase
(E.coli)
Intermembrane space
Innter mitochondrial membrane
Mitochondrial matrix
Membrane domain
(Hydrophobic arm)
e-
H1
Nqo4
Nqo6
Nqo5
Nqo 9
Nqo 3
15
FMN
Nqo1
Nqo2
Hydrophilic Domaine
(Peripheral arm)
2H+
4H+
ACP1
ACP1
1.6.5.11
NADH
NAD+
H+
SdhD
SdhC
Flavo protein
Iron protein
SDH4, ACN18...
Succinate
Fumarate
UQ
UQH2
Quinone pool
8
ISP
Cyt a
7
Cyt b
10
11
6
Core 1
9
Core 2
COB, COB1, C...
2H+
Cytochrome bc1 complex
(bovine)
Cyt c
IV
VIII
VIb
VIa
II
VIc
III
I
VIIa
Vb
Va
VIIb
VIIc
COX1, OXI3...
1/2O2
2H+
H2O
Cytochrome c oxidase
(bovine)
Proton channel
ATP synthase
(Escherichia coli)
a
c
b
γ
ε
β
β
α
β
α
δ
ATP8, AAP1...
K01541...
PMA1, KTI10...
Founit
F1unit
K00937
PPPi
PPi
IPP1, PPA1...
Pi ADP
3H+
ATP H2O
NADH dehydrogenase
E K03878 K03879 K03880 K03881 K03882 K03883 K03884
E K03934 K03935 K03936 K03937 K03938 K03939 K03940 K03941 K03942 K03943 K03944
B/A K00330 K00331... K00332... K00333... K00334 K00335 K00336 K00337 K00338 K00339 K00340 K00341... K00342... K00343
B/A K05574 K05582 K05581 K05579 K05572 K05580 K05578 K05576 K05577 K05575 K05573 K05583 K05584 K05585 K05586 K05587 K05588
E K03945 K03946 K03947 K03948 K03949 K03950 K03951 K03952 K03953 K03954 ACP1 K03956 K11352 K11353
E K03957 K03958 K03959 K03960 K03961 K03962 K03963 K03964 K03965 K03966 K11351 K03967 K03968
Succinate dehydrogenase / Fumarate reductase
E SDH3, CYB3, ... SDH4, ACN18... YJL045W... SDH2, ACN17
B/A K00241 K00242... K00239 K00240
K00244 K00245 K00246 K00247
Cytochrome c reductase
E/B/A RIP1 COB, COB1, C... CYT1, CTC1, ...
E COR1, QCR1 QCR2, COR2, ... QCR6, COR3, ... QCR7, COR4, ... QCR8, COR5 QCR9, UCR9 QCR10
Cytochrome c oxidase
E COX10 COX3, OXI2 COX1, OXI3 COX2, OXI1, ... COX5B... COX6 COX4 COX13 COX12 K02268 COX9... K02271 COX8 K02273
E/B/A COX11, LPI13... COX15 COX17
B/A COX10 K02300 K02299 K02298 K02297
K02277 K02276... K02274... K02275
K02829 K02828 K02827 K02826
Cytochrome c oxidase , cbb3-type
B K00404... K00405... K00407 K00406
Cytochrome bd complex
B/A K00425 K00426
F-type ATPase (Bacteria)
K02111 K02112 K02115 K02113 K02114
K02108 K02109 K02110
F-type ATPase (Eukaryotes)
ATP1 ATP2 ATP3 ATP16 ATP15
ATP5, OSC1 ATP6, OLI2, ... ATP4, LPF7 OLI1, ATP9, ... ATP7 TIM11, ATP21
ATP17 ATP20 ATP14 ATP18 ATP19 ATP8, AAP1
V/A-type ATPase (Bacteria, Archaeas)
K02117 K02118 K02119 K02120 K02121 K02122 K02107
K02123 K02124
V-type ATPase (Eukaryotes)
VMA1, CLS8, ... VMA2, VAT2 VMA5, CSL5, ... VMA8 VMA4 VMA7 VMA10, YHR03... VMA13, CLS11
STV1... VMA3, CLS7, ... VMA6 K02153 K03662
00190 6/16/15
(c) Kanehisa Laboratories

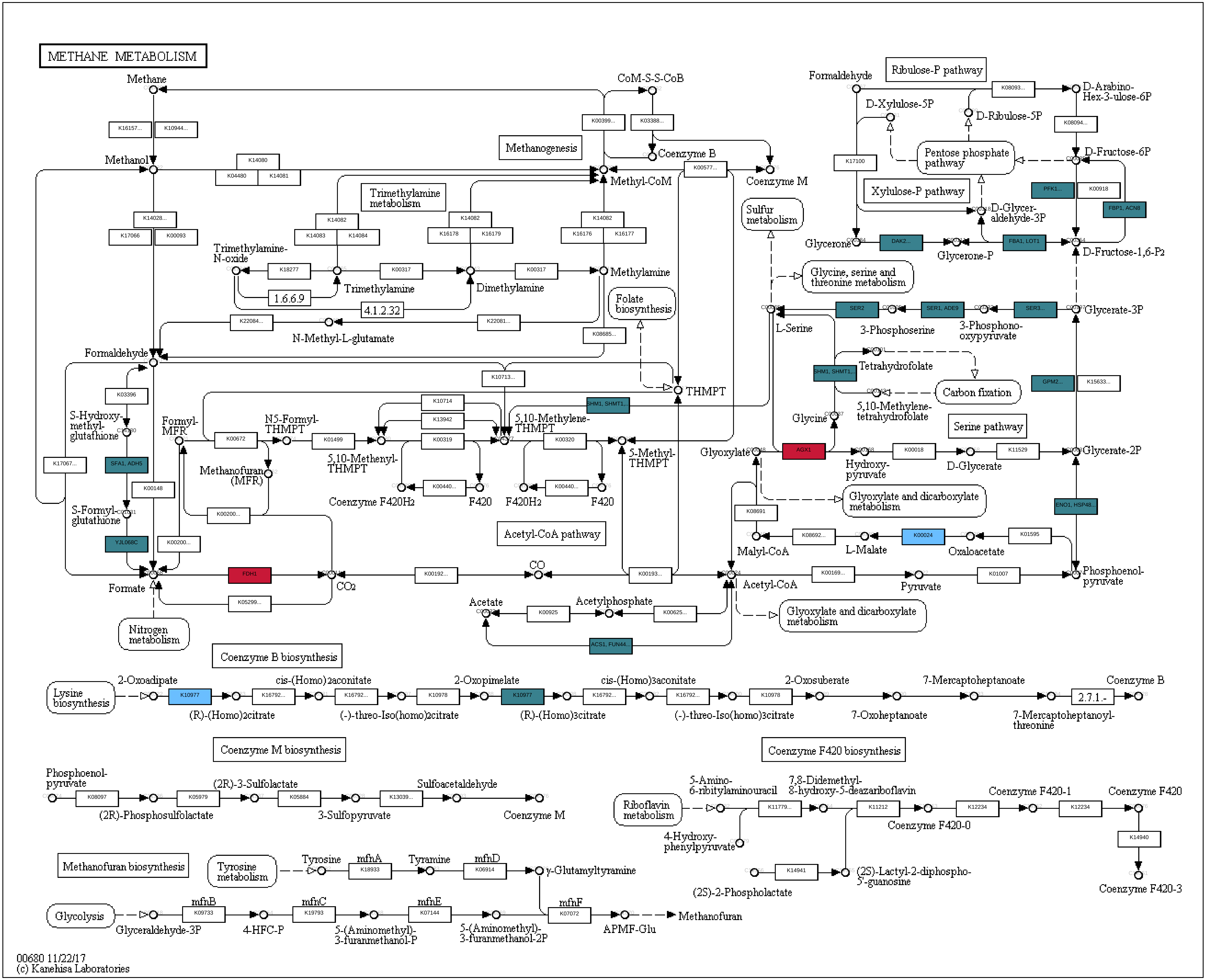
METHANE METABOLISM
Methane
CoM-S-S-CoB
Methanogenesis
Coenzyme B
Coenzyme M
Methyl-CoM
Methanol
Trimethylamine metabolism
Trimethylamine-N-oxide
Trimethylamine
Dimethylamine
Methylamine
1.6.6.9
4.1.2.32
N-Methyl-L-glutamate
Folate biosynthesis
Sulfur metabolism
Formaldehyde
S-Hydroxy-methyl-glutathione
S-Formyl-glutathione
Formyl-MFR
N5-Formyl-THMPT
5,10-Methenyl-THMPT
5,10-Methylene-THMPT
5-Methyl-THMPT
THMPT
Methanofuran (MFR)
Coenzyme F420H2
F420
F420H2
Acetyl-CoA pathway
Formate
CO2
CO
Acetate
Acetylphosphate
Acetyl-CoA
Nitrogen metabolism
Ribulose-P pathway
D-Arabino-Hex-3-ulose-6P
D-Xylulose-5P
D-Ribulose-5P
Pentose phosphate pathway
Xylulose-P pathway
D-Fructose-6P
D-Glyceraldehyde-3P
Glycerone
Glycerone-P
D-Fructose-1,6-P2
Glycine, serine and threonine metabolism
L-Serine
3-Phosphoserine
3-Phosphono-oxypyruvate
Glycerate-3P
Tetrahydrofolate
Carbon fixation
5,10-Methylene-tetrahydrofolate
Glycine
Serine pathway
Glyoxylate
Hydroxy-pyruvate
D-Glycerate
Glycerate-2P
Glyoxylate and dicarboxylate metabolism
Malyl-CoA
L-Malate
Oxaloacetate
Pyruvate
Phosphoenol-pyruvate
SER2
SER1, ADE9
SER3...
SHM1, SHMT1...
AGX1
SFA1, ADH5
YJL068C
FDH1
DAK2...
FBA1, LOT1
PFK1...
FBP1, ACN8
GPM2...
ENO1, HSP48...
ACS1, FUN44...
Coenzyme B biosynthesis
Lysine biosynthesis
2-Oxoadipate
(R)-(Homo)2citrate
cis-(Homo)2aconitate
(-)-threo-Iso(homo)2citrate
2-Oxopimelate
(R)-(Homo)3citrate
cis-(Homo)3aconitate
(-)-threo-Iso(homo)3citrate
2-Oxosuberate
7-Oxoheptanoate
7-Mercaptoheptanoate
7-Mercaptoheptanoyl-threonine
2.7.1.-
Coenzyme M biosynthesis
Phosphoenol-pyruvate
(2R)-Phosphosulfolactate
(2R)-3-Sulfolactate
3-Sulfopyruvate
Sulfoacetaldehyde
Coenzyme F420 biosynthesis
Riboflavin metabolism
5-Amino-6-ribitylaminouracil
7,8-Didemethyl-8-hydroxy-5-deazariboflavin
4-Hydroxy-phenylpyruvate
Coenzyme F420-0
Coenzyme F420-1
Coenzyme F420
Coenzyme F420-3
(2S)-Lactyl-2-diphospho-5'-guanosine
(2S)-2-Phospholactate
Methanofuran biosynthesis
Tyrosine metabolism
Tyrosine
mfnA
Tyramine
mfnD
γ-Glutamyltyramine
Glycolysis
Glyceraldehyde-3P
mfnB
4-HFC-P
mfnC
5-(Aminomethyl)-3-furanmethanol-P
mfnE
5-(Aminomethyl)-3-furanmethanol-2P
mfnF
APMF-Glu
Methanofuran
00680 11/22/17
(c) Kanehisa Laboratories

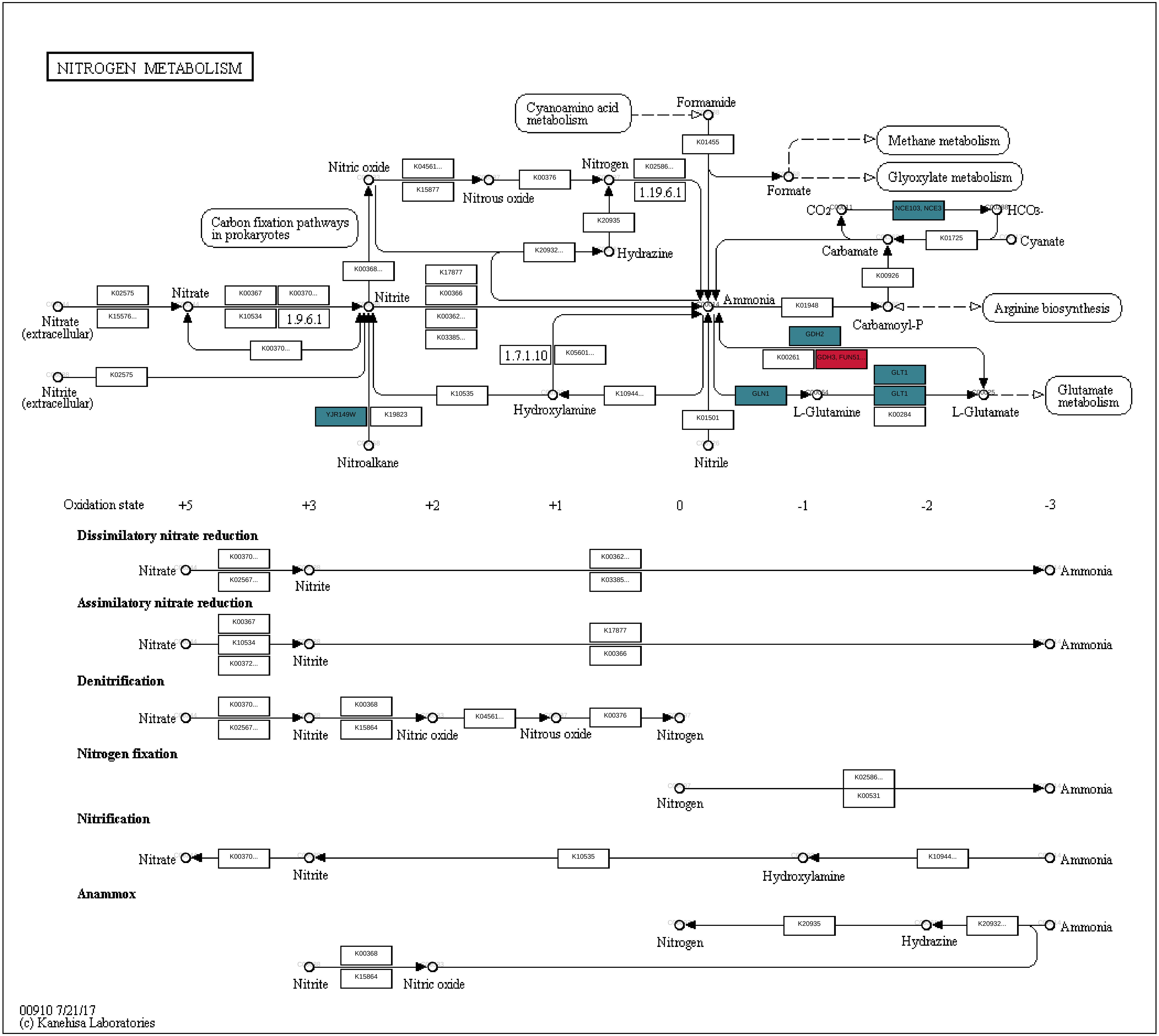

NITROGEN METABOLISM
Cyanoamino acid metabolism
Formamide
K01455
Methane metabolism
Glyoxylate metabolism
Formate
Nitric oxide
K04561...
K15877
Nitrous oxide
K00376
Nitrogen
K02586...
1.19.6.1
K20935
K20932...
Hydrazine
CO2
NCE103, NCE3
HCO3-
K01725
Cyanate
Carbamate
K00926
Carbon fixation pathways in prokaryotes
K00368...
K17877
K00366
K00362...
K03385...
Ammonia
K01948
Carbamoyl-P
Arginine biosynthesis
K02575
K15576...
Nitrate
K00367
K00370...
K10534
1.9.6.1
Nitrite
Nitrate (extracellular)
K00370...
GDH2
K00261
GDH3, FUN51...
1.7.1.10
K05601...
Nitrite (extracellular)
K02575
GLT1
GLT1
K00284
K10535
K10944...
GLN1
Hydroxylamine
L-Glutamine
L-Glutamate
Glutamate metabolism
YJR149W
K19823
K01501
Nitroalkane
Nitrile
Oxidation state +5 +3 +2 +1 0 -1 -2 -3
Dissimilatory nitrate reduction
Nitrate
K00370...
K02567...
Nitrite
K00362...
K03385...
Ammonia
Assimilatory nitrate reduction
Nitrate
K00367
K10534
K00372...
Nitrite
K17877
K00366
Ammonia
Denitrification
Nitrate
K00370...
K02567...
Nitrite
K00368
K15864
Nitric oxide
K04561...
Nitrous oxide
K00376
Nitrogen
Nitrogen fixation
Nitrogen
K02586...
K00531
Ammonia
Nitrification
Nitrate
K00370...
Nitrite
K10535
Hydroxylamine
K10944...
Ammonia
Anammox
Nitrogen
K20935
Hydrazine
K20932...
Ammonia
Nitrite
K00368
K15864
Nitric oxide
00910 7/21/17
(c) Kanehisa Laboratories

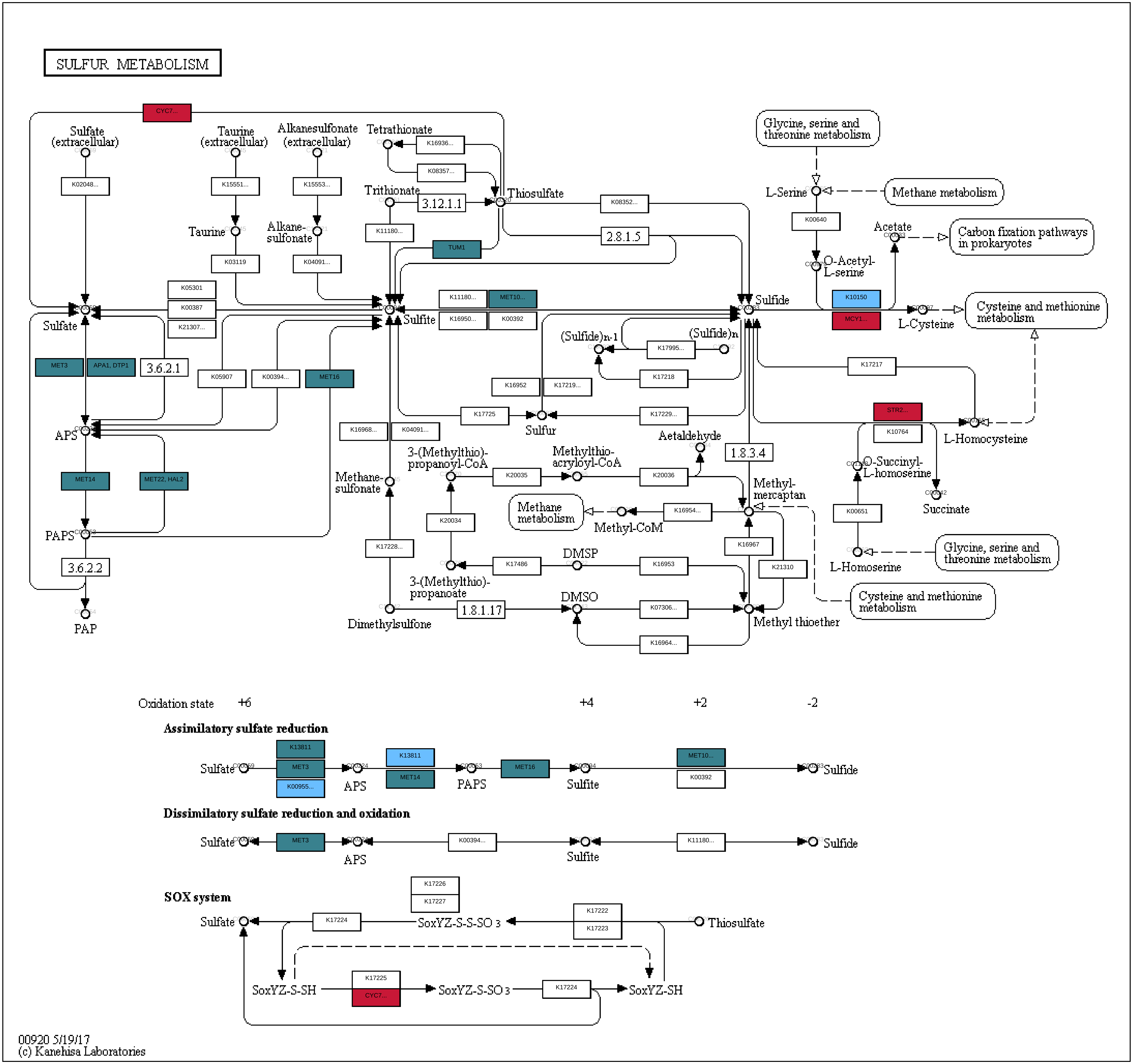
SULFUR METABOLISM
CYC7...
Sulfate (extracellular)
Taurine (extracellular)
Alkanesulfonate (extracellular)
Tetrathionate
K16936...
K08357...
Trithionate
3.12.1.1
Thiosulfate
K08352...
2.8.1.5
K02048...
K15551...
K15553...
Taurine
Alkane-sulfonate
K03119
K04091...
K11180...
TUM1
K05301
K00387
K21307...
Sulfate
Sulfite
K11180...
MET10...
K16950...
K00392
Sulfide
MET3
APA1, DTP1
3.6.2.1
K05907
K00394...
MET16
APS
MET14
MET22, HAL2
PAPS
3.6.2.2
PAP
Glycine, serine and threonine metabolism
L-Serine
Methane metabolism
K00640
O-Acetyl-L-serine
Acetate
Carbon fixation pathways in prokaryotes
K10150
MCY1...
L-Cysteine
Cysteine and methionine metabolism
(Sulfide)n-1
(Sulfide)n
K17995...
K17218
K17217
K16952
K17219...
K17725
Sulfur
K17229...
STR2...
K10764
L-Homocysteine
K16968...
K04091...
Aetaldehyde
1.8.3.4
3-(Methylthio)-propanoyl-CoA
Methylthio-acryloyl-CoA
K20035
K20036
Methane-sulfonate
O-Succinyl-L-homoserine
Succinate
Methyl-mercaptan
Methane metabolism
Methyl-CoM
K16954...
K00651
K20034
K17228...
K16967
K17486
DMSP
K16953
K21310
L-Homoserine
Glycine, serine and threonine metabolism
3-(Methylthio)-propanoate
DMSO
1.8.1.17
K07306...
Cysteine and methionine metabolism
Dimethylsulfone
Methyl thioether
K16964...
Oxidation state +6 +4 +2 -2
Assimilatory sulfate reduction
Sulfate
K13811
MET3
K00955...
APS
K13811
MET14
PAPS
MET16
Sulfite
MET10...
K00392
Sulfide
Dissimilatory sulfate reduction and oxidation
Sulfate
MET3
APS
K00394...
Sulfite
K11180...
Sulfide
SOX system
Sulfate
K17224
K17226
K17227
SoxYZ-S-S-SO3
K17222
K17223
Thiosulfate
SoxYZ-S-SH
K17225
CYC7...
SoxYZ-S-S-SO3
K17224
SoxYZ-SH
00920 5/19/17
(c) Kanehisa Laboratories

# 3. Lipid metabolism

| MAP | PATHWAY |
|---|---|
| **61** | Fatty acid biosynthesis |
| **62** | Fatty acid elongation |
| **71** | Fatty acid degradation |
| **72** | Synthesis and degradation of ketone bodies |
| **100** | Steroid biosynthesis |
| **561** | Glycerolipid metabolism |
| **564** | Glycerophospholipid metabolism |
| **565** | Ether lipid metabolism |
| **590** | Arachidonic acid metabolism |
| **592** | alpha-Linolenic acid metabolism |
| **600** | Sphingolipid metabolism |

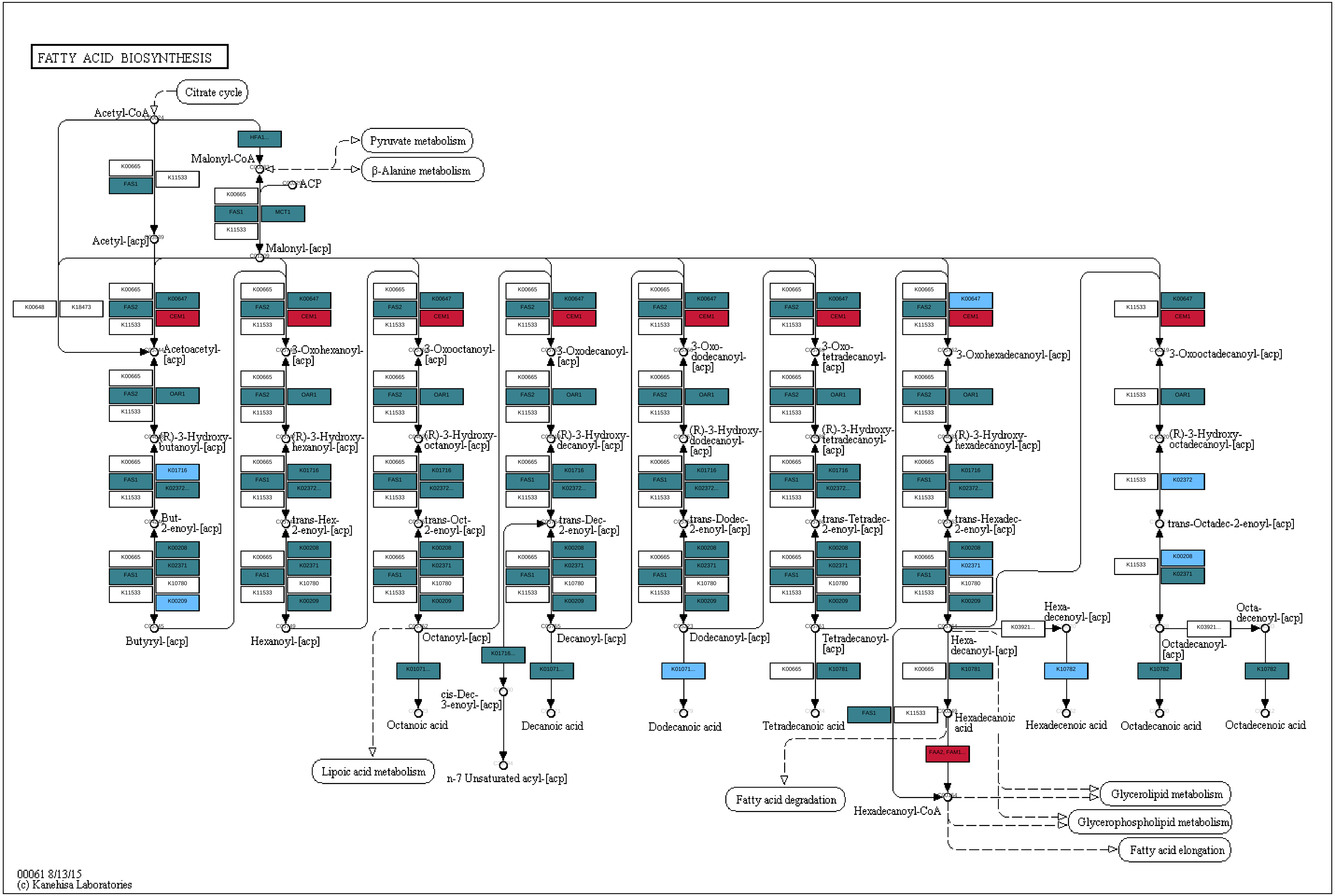
FATTY ACID BIOSYNTHESIS
Citrate cycle
Acetyl-CoA
Malonyl-CoA
Pyruvate metabolism
β-Alanine metabolism
ACP
Acetyl-[acp]
Malonyl-[acp]
HFA1...
K00665
FAS1
FAS2
K11533
MCT1
K00647
CEM1
OAR1
K01716
K02372...
K00208
K02371
K10780
K00209
K00648
K18473
Acetoacetyl-[acp]
3-Oxohexanoyl-[acp]
3-Oxooctanoyl-[acp]
3-Oxodecanoyl-[acp]
3-Oxo-dodecanoyl-[acp]
3-Oxo-tetradecanoyl-[acp]
3-Oxohexadecanoyl-[acp]
3-Oxooctadecanoyl-[acp]
(R)-3-Hydroxy-butanoyl-[acp]
(R)-3-Hydroxy-hexanoyl-[acp]
(R)-3-Hydroxy-octanoyl-[acp]
(R)-3-Hydroxy-decanoyl-[acp]
(R)-3-Hydroxy-dodecanoyl-[acp]
(R)-3-Hydroxy-tetradecanoyl-[acp]
(R)-3-Hydroxy-hexadecanoyl-[acp]
(R)-3-Hydroxy-octadecanoyl-[acp]
But-2-enoyl-[acp]
trans-Hex-2-enoyl-[acp]
trans-Oct-2-enoyl-[acp]
trans-Dec-2-enoyl-[acp]
trans-Dodec-2-enoyl-[acp]
trans-Tetradec-2-enoyl-[acp]
trans-Hexadec-2-enoyl-[acp]
trans-Octadec-2-enoyl-[acp]
Butyryl-[acp]
Hexanoyl-[acp]
Octanoyl-[acp]
Decanoyl-[acp]
Dodecanoyl-[acp]
Tetradecanoyl-[acp]
Hexadecanoyl-[acp]
Octadecanoyl-[acp]
Hexa-decenoyl-[acp]
Octa-decenoyl-[acp]
K01071...
K01716...
K10781
K10782
K03921...
FAA2, FAM1...
cis-Dec-3-enoyl-[acp]
n-7 Unsaturated acyl-[acp]
Octanoic acid
Decanoic acid
Dodecanoic acid
Tetradecanoic acid
Hexadecanoic acid
Hexadecenoic acid
Octadecanoic acid
Octadecenoic acid
Hexadecanoyl-CoA
Lipoic acid metabolism
Fatty acid degradation
Glycerolipid metabolism
Glycerophospholipid metabolism
Fatty acid elongation
00061 8/13/15
(c) Kanehisa Laboratories

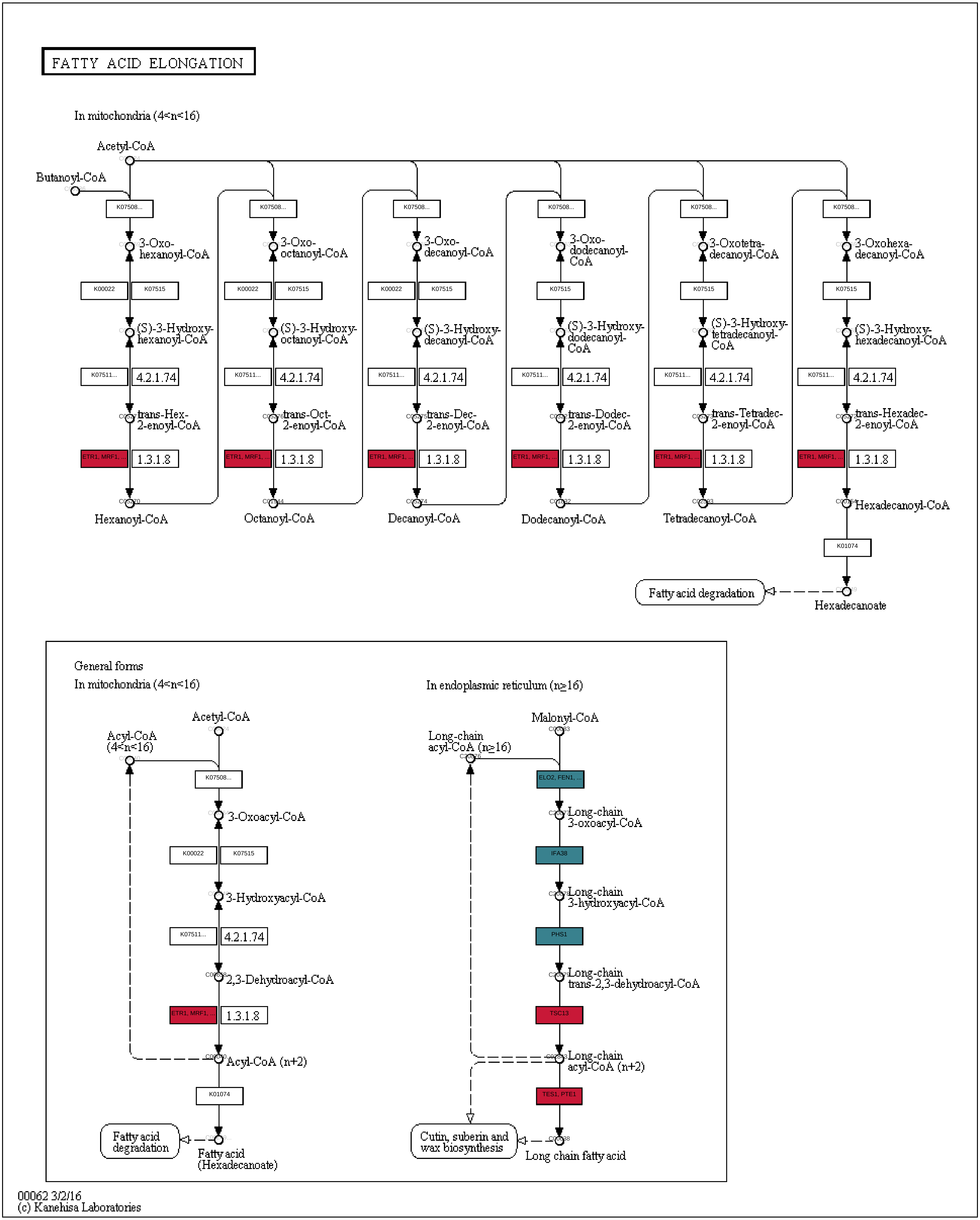
FATTY ACID ELONGATION
In mitochondria (4<n<16)
Acetyl-CoA
Butanoyl-CoA
K07508...
3-Oxo-hexanoyl-CoA
3-Oxo-octanoyl-CoA
3-Oxo-decanoyl-CoA
3-Oxo-dodecanoyl-CoA
3-Oxotetra-decanoyl-CoA
3-Oxohexa-decanoyl-CoA
K00022
K07515
(S)-3-Hydroxy-hexanoyl-CoA
(S)-3-Hydroxy-octanoyl-CoA
(S)-3-Hydroxy-decanoyl-CoA
(S)-3-Hydroxy-dodecanoyl-CoA
(S)-3-Hydroxy-tetradecanoyl-CoA
(S)-3-Hydroxy-hexadecanoyl-CoA
K07511...
4.2.1.74
trans-Hex-2-enoyl-CoA
trans-Oct-2-enoyl-CoA
trans-Dec-2-enoyl-CoA
trans-Dodec-2-enoyl-CoA
trans-Tetradec-2-enoyl-CoA
trans-Hexadec-2-enoyl-CoA
ETR1, MRF1, ...
1.3.1.8
Hexanoyl-CoA
Octanoyl-CoA
Decanoyl-CoA
Dodecanoyl-CoA
Tetradecanoyl-CoA
Hexadecanoyl-CoA
K01074
Fatty acid degradation
Hexadecanoate
General forms
In mitochondria (4<n<16)
Acetyl-CoA
Acyl-CoA (4<n<16)
K07508...
3-Oxoacyl-CoA
K00022
K07515
3-Hydroxyacyl-CoA
K07511...
4.2.1.74
2,3-Dehydroacyl-CoA
ETR1, MRF1, ...
1.3.1.8
Acyl-CoA (n+2)
K01074
Fatty acid degradation
Fatty acid (Hexadecanoate)
In endoplasmic reticulum (n≥16)
Malonyl-CoA
Long-chain acyl-CoA (n≥16)
ELO2, FEN1, ...
Long-chain 3-oxoacyl-CoA
IFA38
Long-chain 3-hydroxyacyl-CoA
PHS1
Long-chain trans-2,3-dehydroacyl-CoA
TSC13
Long-chain acyl-CoA (n+2)
TES1, PTE1
Cutin, suberin and wax biosynthesis
Long chain fatty acid
00062 3/2/16
(c) Kanehisa Laboratories

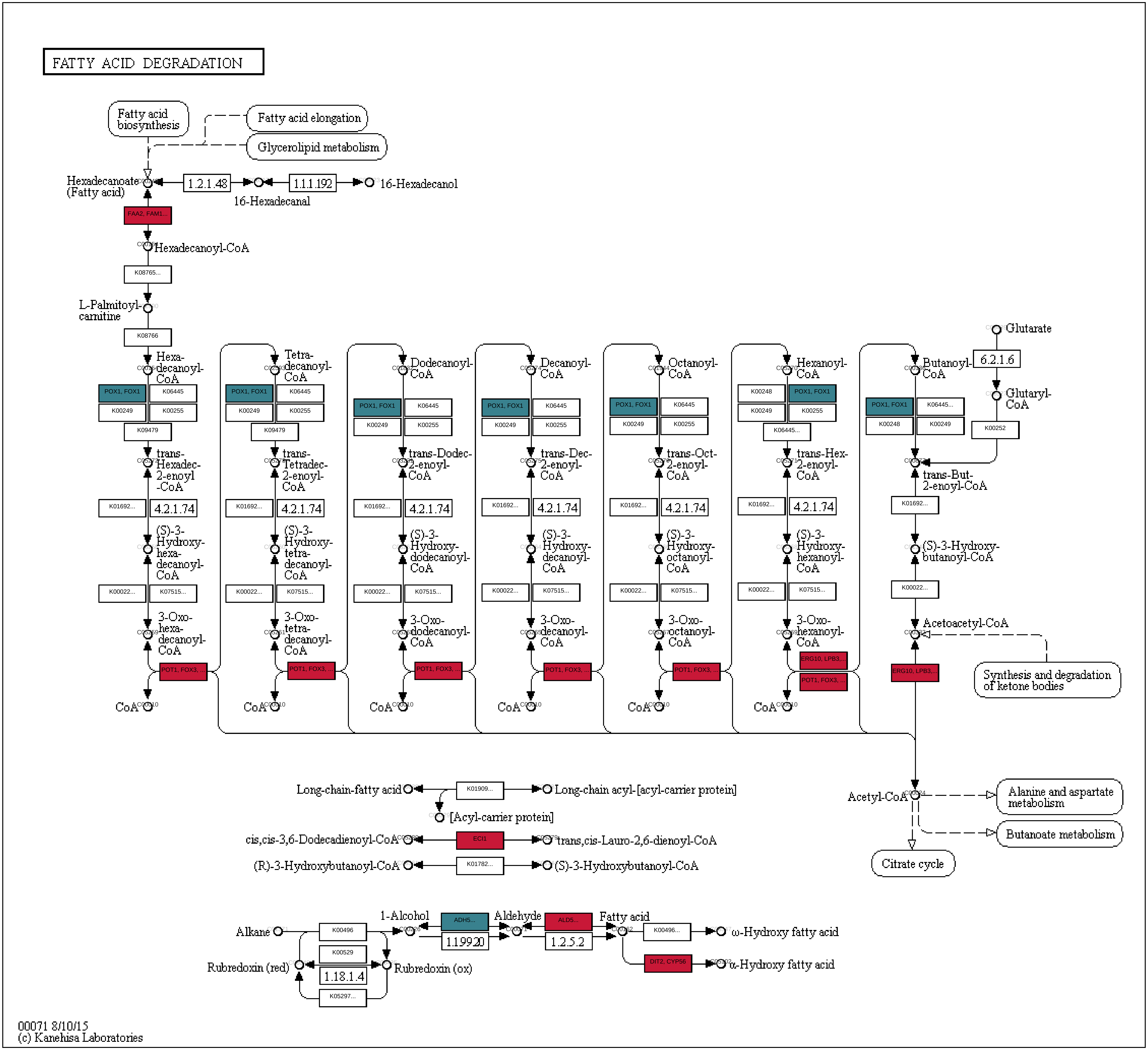
FATTY ACID DEGRADATION
Fatty acid biosynthesis
Fatty acid elongation
Glycerolipid metabolism
Hexadecanoate (Fatty acid)
1.2.1.48
16-Hexadecanal
1.1.1.192
16-Hexadecanol
FAA2, FAM1...
Hexadecanoyl-CoA
K08765...
L-Palmitoyl-carnitine
K08766
Hexa-decanoyl-CoA
Tetra-decanoyl-CoA
Dodecanoyl-CoA
Decanoyl-CoA
Octanoyl-CoA
Hexanoyl-CoA
Butanoyl-CoA
Glutarate
6.2.1.6
Glutaryl-CoA
K00252
POX1, FOX1
K06445
K00249
K00255
K09479
K00248
K06445...
trans-Hexadec-2-enoyl-CoA
trans-Tetradec-2-enoyl-CoA
trans-Dodec-2-enoyl-CoA
trans-Dec-2-enoyl-CoA
trans-Oct-2-enoyl-CoA
trans-Hex-2-enoyl-CoA
trans-But-2-enoyl-CoA
K01692...
4.2.1.74
(S)-3-Hydroxy-hexa-decanoyl-CoA
(S)-3-Hydroxy-tetra-decanoyl-CoA
(S)-3-Hydroxy-dodecanoyl-CoA
(S)-3-Hydroxy-decanoyl-CoA
(S)-3-Hydroxy-octanoyl-CoA
(S)-3-Hydroxy-hexanoyl-CoA
(S)-3-Hydroxy-butanoyl-CoA
K00022...
K07515...
3-Oxo-hexa-decanoyl-CoA
3-Oxo-tetra-decanoyl-CoA
3-Oxo-dodecanoyl-CoA
3-Oxo-decanoyl-CoA
3-Oxo-octanoyl-CoA
3-Oxo-hexanoyl-CoA
Acetoacetyl-CoA
POT1, FOX3, ...
ERG10, LPB3,...
Synthesis and degradation of ketone bodies
CoA
Acetyl-CoA
Alanine and aspartate metabolism
Butanoate metabolism
Citrate cycle
Long-chain-fatty acid
K01909...
Long-chain acyl-[acyl-carrier protein]
[Acyl-carrier protein]
cis,cis-3,6-Dodecadienoyl-CoA
ECI1
trans,cis-Lauro-2,6-dienoyl-CoA
(R)-3-Hydroxybutanoyl-CoA
K01782...
(S)-3-Hydroxybutanoyl-CoA
Alkane
K00496
K00529
1.18.1.4
K05297...
Rubredoxin (red)
Rubredoxin (ox)
1-Alcohol
ADH5...
1.1.99.20
Aldehyde
ALD5...
1.2.5.2
Fatty acid
K00496...
ω-Hydroxy fatty acid
DIT2, CYP56
α-Hydroxy fatty acid
00071 8/10/15
(c) Kanehisa Laboratories

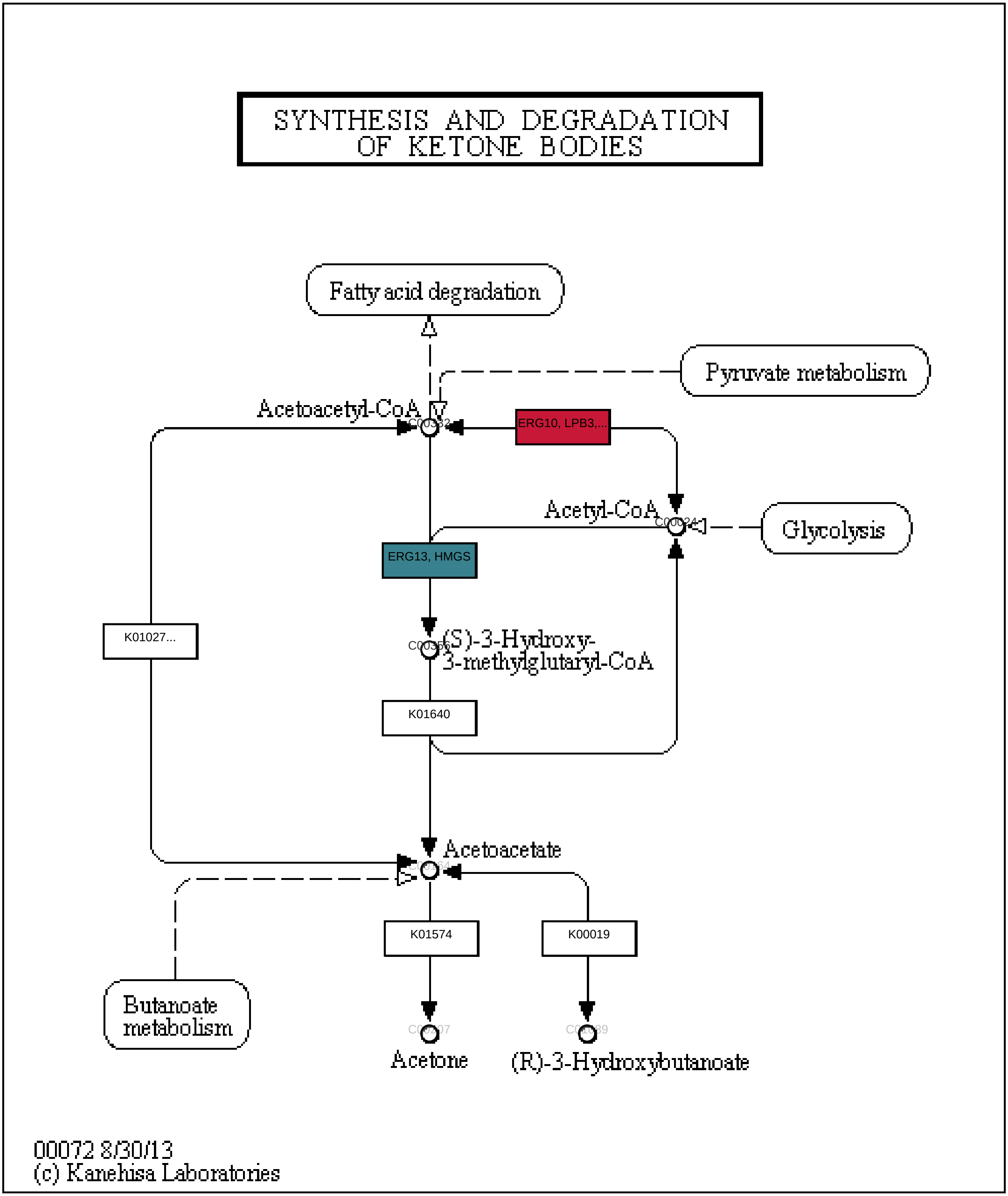
SYNTHESIS AND DEGRADATION OF KETONE BODIES
Fatty acid degradation
Pyruvate metabolism
Acetoacetyl-CoA
C00332
ERG10, LPB3,...
Acetyl-CoA
C00024
Glycolysis
ERG13, HMGS
K01027...
C00356
(S)-3-Hydroxy-3-methylglutaryl-CoA
K01640
Acetoacetate
K01574
K00019
Butanoate metabolism
Acetone
(R)-3-Hydroxybutanoate
00072 8/30/13
(c) Kanehisa Laboratories

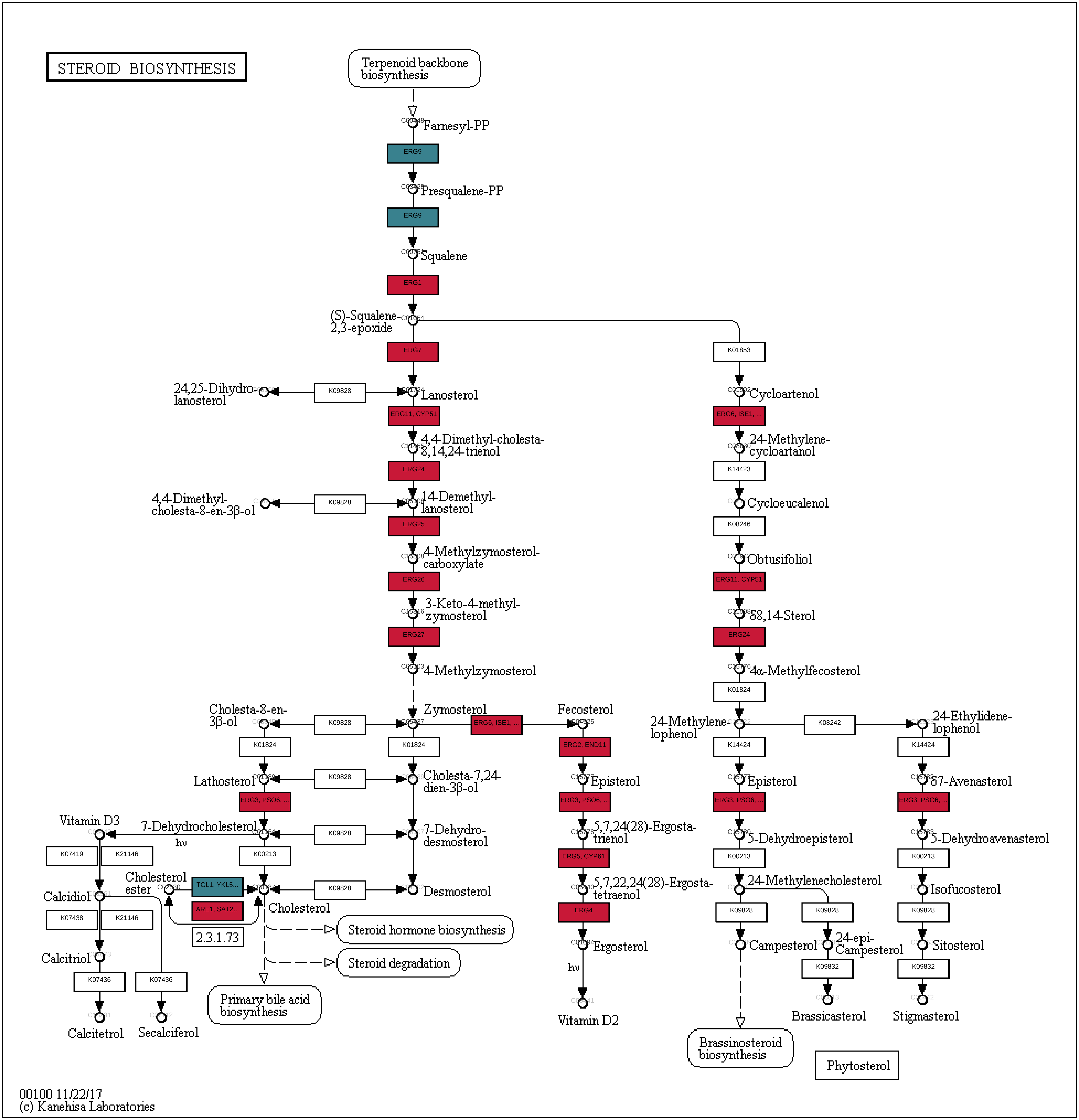
STEROID BIOSYNTHESIS
Terpenoid backbone biosynthesis
Farnesyl-PP
ERG9
Presqualene-PP
ERG9
Squalene
ERG1
(S)-Squalene-2,3-epoxide
ERG7
K01853
24,25-Dihydro-lanosterol
K09828
Lanosterol
Cycloartenol
ERG11, CYP51
ERG6, ISE1, ...
4,4-Dimethyl-cholesta-8,14,24-trienol
24-Methylene-cycloartanol
ERG24
K14423
4,4-Dimethyl-cholesta-8-en-3β-ol
K09828
14-Demethyl-lanosterol
Cycloeucalenol
ERG25
K08246
4-Methylzymosterol-carboxylate
Obtusifoliol
ERG26
ERG11, CYP51
3-Keto-4-methyl-zymosterol
δ8,14-Sterol
ERG27
ERG24
4-Methylzymosterol
4α-Methylfecosterol
K01824
Cholesta-8-en-3β-ol
K09828
Zymosterol
ERG6, ISE1, ...
Fecosterol
24-Methylene-lophenol
K08242
24-Ethylidene-lophenol
K01824
K01824
ERG2, END11
K14424
K14424
Lathosterol
K09828
Cholesta-7,24-dien-3β-ol
Episterol
Episterol
δ7-Avenasterol
ERG3, PSO6, ...
ERG3, PSO6, ...
ERG3, PSO6, ...
ERG3, PSO6, ...
Vitamin D3
7-Dehydrocholesterol
K09828
7-Dehydro-desmosterol
5,7,24(28)-Ergosta-trienol
5-Dehydroepisterol
5-Dehydroavenasterol
K07419
K21146
hν
K00213
ERG5, CYP61
K00213
K00213
Cholesterol ester
TGL1, YKL5...
K09828
Desmosterol
5,7,22,24(28)-Ergosta-tetraenol
24-Methylenecholesterol
Isofucosterol
Calcidiol
ARE1, SAT2...
Cholesterol
ERG4
K09828
K09828
K09828
K07438
K21146
2.3.1.73
Steroid hormone biosynthesis
Campesterol
24-epi-Campesterol
Sitosterol
Calcitriol
Ergosterol
K07436
K07436
Steroid degradation
hν
K09832
K09832
Primary bile acid biosynthesis
Calcitetrol
Secalciferol
Vitamin D2
Brassicasterol
Stigmasterol
Brassinosteroid biosynthesis
Phytosterol
00100 11/22/17
(c) Kanehisa Laboratories

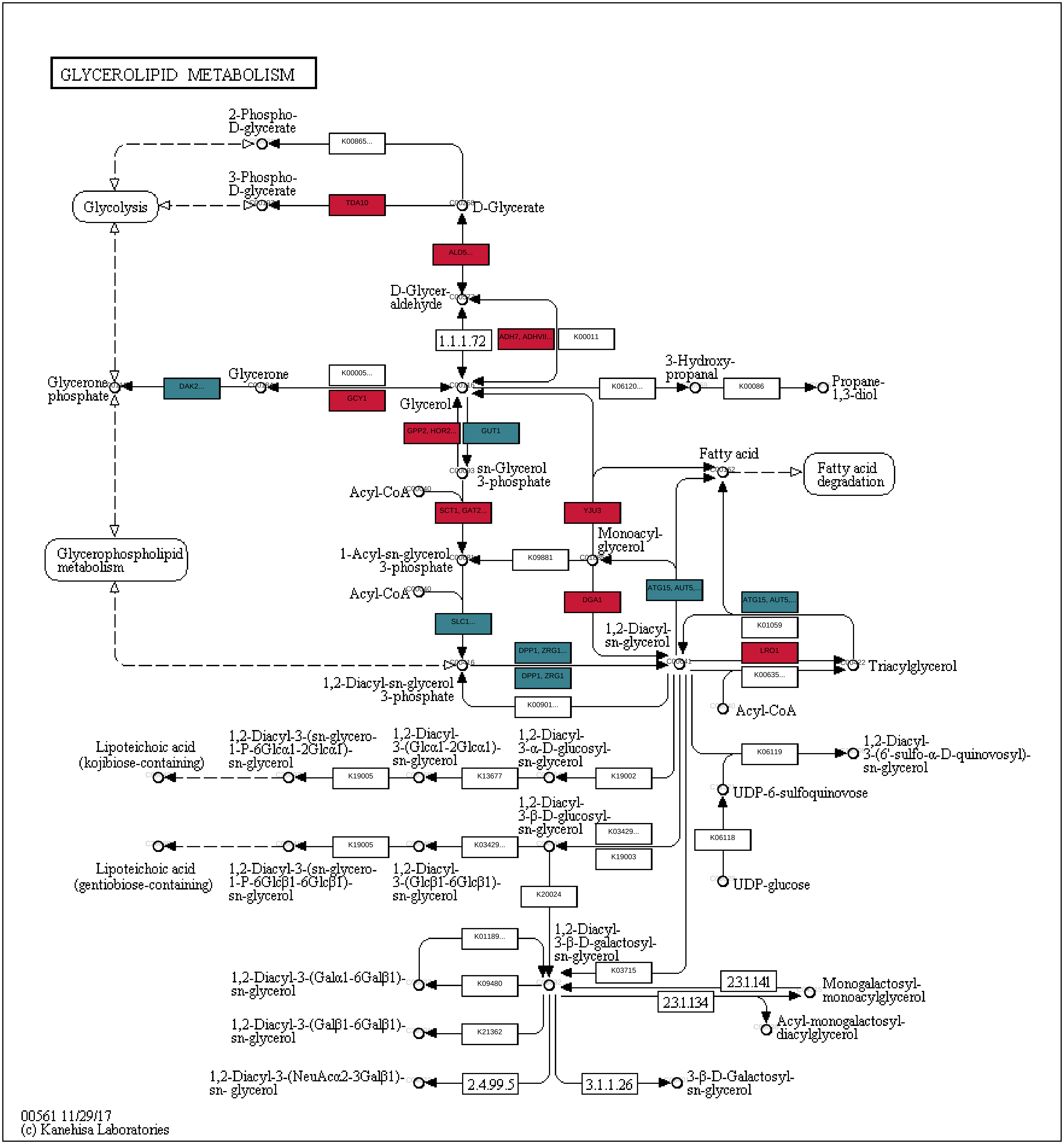
GLYCEROLIPID METABOLISM
2-Phospho-D-glycerate
K00865...
3-Phospho-D-glycerate
TDA10
D-Glycerate
Glycolysis
ALD5...
D-Glyceraldehyde
1.1.1.72
ADH7, ADHVII...
K00011
Glycerone phosphate
DAK2...
Glycerone
K00005...
GCY1
Glycerol
K06120...
3-Hydroxypropanal
K00086
Propane-1,3-diol
GPP2, HOR2...
GUT1
sn-Glycerol 3-phosphate
Acyl-CoA
SCT1, GAT2...
YJU3
Fatty acid
Fatty acid degradation
Glycerophospholipid metabolism
1-Acyl-sn-glycerol 3-phosphate
K09881
Monoacylglycerol
ATG15, AUT5,...
DGA1
SLC1...
1,2-Diacyl-sn-glycerol
K01059
LRO1
K00635...
Triacylglycerol
DPP1, ZRG1...
1,2-Diacyl-sn-glycerol 3-phosphate
K00901...
Lipoteichoic acid (kojibiose-containing)
1,2-Diacyl-3-(sn-glycero-1-P-6Glcα1-2Glcα1)-sn-glycerol
1,2-Diacyl-3-(Glcα1-2Glcα1)-sn-glycerol
1,2-Diacyl-3-α-D-glucosyl-sn-glycerol
K19005
K13677
K19002
K06119
1,2-Diacyl-3-(6'-sulfo-α-D-quinovosyl)-sn-glycerol
UDP-6-sulfoquinovose
1,2-Diacyl-3-β-D-glucosyl-sn-glycerol
K03429...
K19003
K06118
Lipoteichoic acid (gentiobiose-containing)
1,2-Diacyl-3-(sn-glycero-1-P-6Glcβ1-6Glcβ1)-sn-glycerol
1,2-Diacyl-3-(Glcβ1-6Glcβ1)-sn-glycerol
UDP-glucose
K20024
K01189...
1,2-Diacyl-3-β-D-galactosyl-sn-glycerol
1,2-Diacyl-3-(Galα1-6Galβ1)-sn-glycerol
K09480
K03715
2.3.1.141
Monogalactosyl-monoacylglycerol
2.3.1.134
Acyl-monogalactosyl-diacylglycerol
1,2-Diacyl-3-(Galβ1-6Galβ1)-sn-glycerol
K21362
1,2-Diacyl-3-(NeuAcα2-3Galβ1)-sn- glycerol
2.4.99.5
3.1.1.26
3-β-D-Galactosyl-sn-glycerol
00561 11/29/17
(c) Kanehisa Laboratories

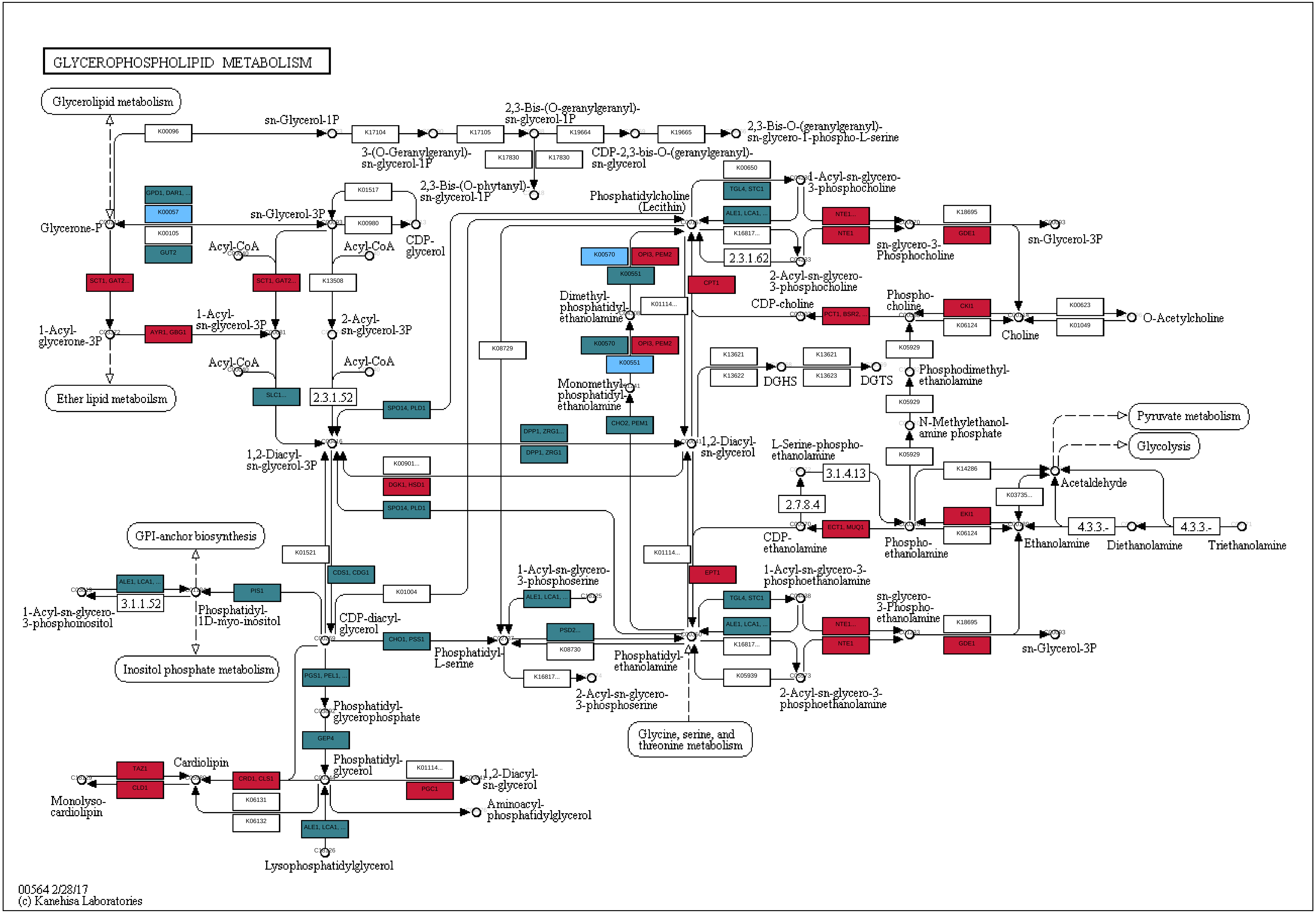

GLYCEROPHOSPHOLIPID METABOLISM
Glycerolipid metabolism
Ether lipid metaboilsm
GPI-anchor biosynthesis
Inositol phosphate metabolism
Glycine, serine, and threonine metabolism
Pyruvate metabolism
Glycolysis
K00096
sn-Glycerol-1P
K17104
K17105
K19664
K19665
2,3-Bis-(O-geranylgeranyl)-sn-glycerol-1P
2,3-Bis-O-(geranylgeranyl)-sn-glycero-1-phospho-L-serine
3-(O-Geranylgeranyl)-sn-glycerol-1P
K17830
K17830
CDP-2,3-bis-O-(geranylgeranyl)-sn-glycerol
2,3-Bis-(O-phytanyl)-sn-glycerol-1P
GPD1, DAR1, ...
K00057
K00105
GUT2
Glycerone-P
sn-Glycerol-3P
K01517
K00980
CDP-glycerol
Acyl-CoA
Acyl-CoA
SCT1, GAT2...
SCT1, GAT2...
K13508
1-Acyl-glycerone-3P
AYR1, GBG1
1-Acyl-sn-glycerol-3P
2-Acyl-sn-glycerol-3P
Acyl-CoA
Acyl-CoA
SLC1...
2.3.1.52
SPO14, PLD1
1,2-Diacyl-sn-glycerol-3P
K00901...
DGK1, HSD1
SPO14, PLD1
K01521
CDS1, CDG1
K01004
CDP-diacyl-glycerol
ALE1, LCA1, ...
PIS1
3.1.1.52
1-Acyl-sn-glycero-3-phosphoinositol
Phosphatidyl-1D-myo-inositol
CHO1, PSS1
PGS1, PEL1, ...
Phosphatidyl-glycerophosphate
GEP4
Phosphatidyl-glycerol
TAZ1
CLD1
CRD1, CLS1
Cardiolipin
K06131
K06132
Monolyso-cardiolipin
ALE1, LCA1, ...
Lysophosphatidylglycerol
K01114...
PGC1
1,2-Diacyl-sn-glycerol
Aminoacyl-phosphatidylglycerol
K00650
TGL4, STC1
ALE1, LCA1, ...
K16817...
Phosphatidylcholine (Lecithin)
1-Acyl-sn-glycero-3-phosphocholine
NTE1...
NTE1
K18695
GDE1
sn-Glycerol-3P
sn-glycero-3-Phosphocholine
2.3.1.62
2-Acyl-sn-glycero-3-phosphocholine
K00570
OPI3, PEM2
K00551
CPT1
Dimethyl-phosphatidyl-ethanolamine
K01114...
CDP-choline
PCT1, BSR2, ...
Phospho-choline
CKI1
K06124
K00623
K01049
Choline
O-Acetylcholine
K08729
K00570
OPI3, PEM2
K00551
K13621
K13622
K13621
K13623
DGHS
DGTS
K05929
Phosphodimethyl-ethanolamine
K05929
Monomethyl-phosphatidyl-ethanolamine
CHO2, PEM1
N-Methylethanol-amine phosphate
K05929
DPP1, ZRG1...
DPP1, ZRG1
1,2-Diacyl-sn-glycerol
L-Serine-phospho-ethanolamine
3.1.4.13
K14286
Acetaldehyde
2.7.8.4
K03735...
EKI1
K06124
ECT1, MUQ1
CDP-ethanolamine
Phospho-ethanolamine
Ethanolamine
4.3.3.-
Diethanolamine
4.3.3.-
Triethanolamine
K01114...
EPT1
1-Acyl-sn-glycero-3-phosphoethanolamine
1-Acyl-sn-glycero-3-phosphoserine
ALE1, LCA1, ...
TGL4, STC1
ALE1, LCA1, ...
K16817...
sn-glycero-3-Phospho-ethanolamine
NTE1...
NTE1
K18695
GDE1
sn-Glycerol-3P
PSD2...
K08730
Phosphatidyl-L-serine
Phosphatidyl-ethanolamine
K16817...
2-Acyl-sn-glycero-3-phosphoserine
K05939
2-Acyl-sn-glycero-3-phosphoethanolamine
00564 2/28/17
(c) Kanehisa Laboratories

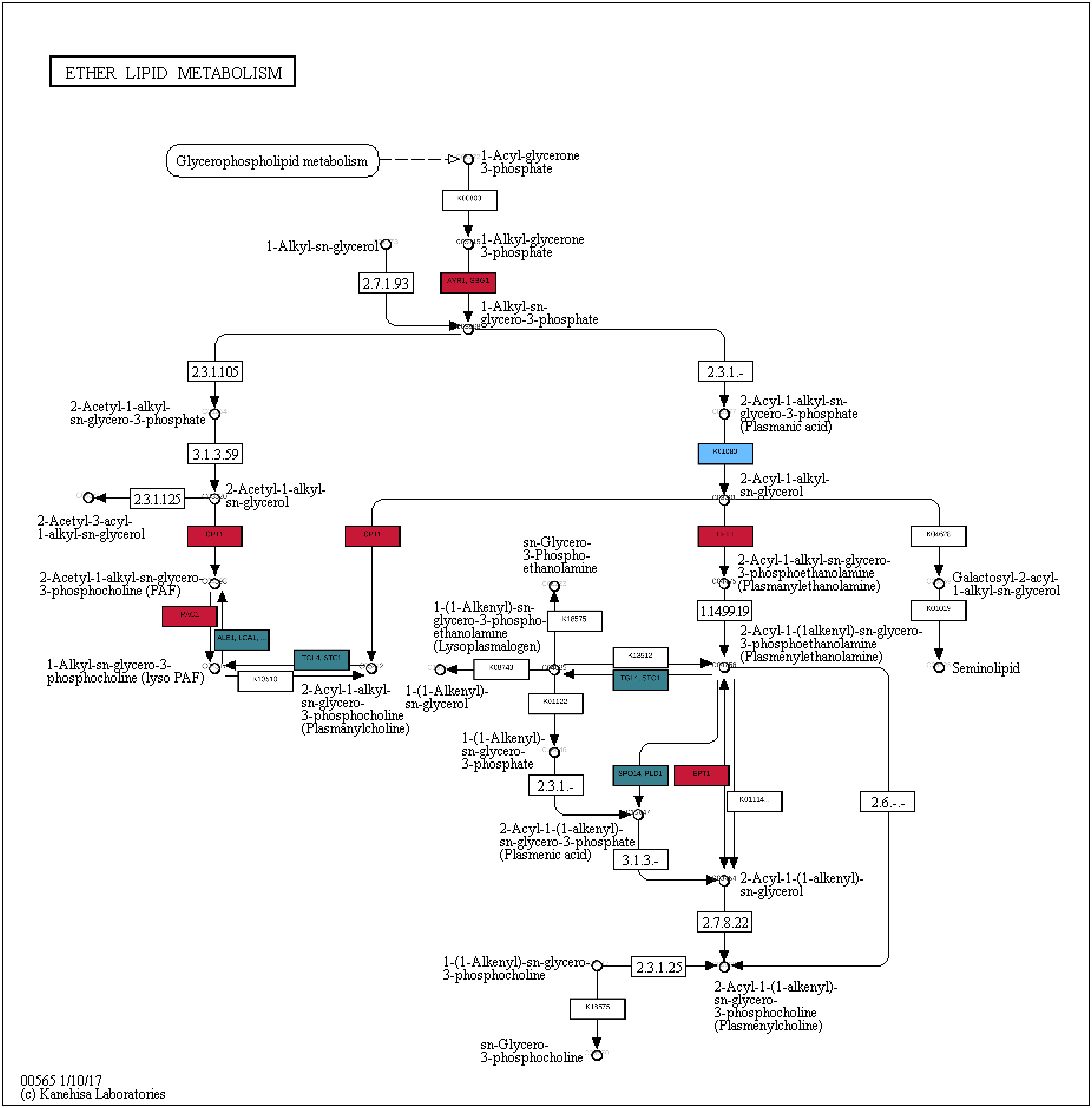
ETHER LIPID METABOLISM
Glycerophospholipid metabolism
1-Acyl-glycerone 3-phosphate
K00803
1-Alkyl-glycerone 3-phosphate
1-Alkyl-sn-glycerol
2.7.1.93
AYR1, GBG1
1-Alkyl-sn-glycero-3-phosphate
2.3.1.105
2.3.1.-
2-Acetyl-1-alkyl-sn-glycero-3-phosphate
2-Acyl-1-alkyl-sn-glycero-3-phosphate (Plasmanic acid)
3.1.3.59
K01080
2.3.1.125
2-Acetyl-1-alkyl-sn-glycerol
2-Acyl-1-alkyl-sn-glycerol
2-Acetyl-3-acyl-1-alkyl-sn-glycerol
CPT1
CPT1
EPT1
K04628
sn-Glycero-3-Phospho-ethanolamine
2-Acetyl-1-alkyl-sn-glycero-3-phosphocholine (PAF)
2-Acyl-1-alkyl-sn-glycero-3-phosphoethanolamine (Plasmanylethanolamine)
Galactosyl-2-acyl-1-alkyl-sn-glycerol
1-(1-Alkenyl)-sn-glycero-3-phospho-ethanolamine (Lysoplasmalogen)
K18575
1.14.99.19
K01019
PAC1
ALE1, LCA1, ...
2-Acyl-1-(1alkenyl)-sn-glycero-3-phosphoethanolamine (Plasmenylethanolamine)
TGL4, STC1
K13512
1-Alkyl-sn-glycero-3-phosphocholine (lyso PAF)
K08743
TGL4, STC1
Seminolipid
K13510
2-Acyl-1-alkyl-sn-glycero-3-phosphocholine (Plasmanylcholine)
1-(1-Alkenyl)-sn-glycerol
K01122
1-(1-Alkenyl)-sn-glycero-3-phosphate
SPO14, PLD1
EPT1
K01114...
2.3.1.-
2.6.-.-
2-Acyl-1-(1-alkenyl)-sn-glycero-3-phosphate (Plasmenic acid)
3.1.3.-
2-Acyl-1-(1-alkenyl)-sn-glycerol
2.7.8.22
1-(1-Alkenyl)-sn-glycero-3-phosphocholine
2.3.1.25
2-Acyl-1-(1-alkenyl)-sn-glycero-3-phosphocholine (Plasmenylcholine)
K18575
sn-Glycero-3-phosphocholine
00565 1/10/17
(c) Kanehisa Laboratories

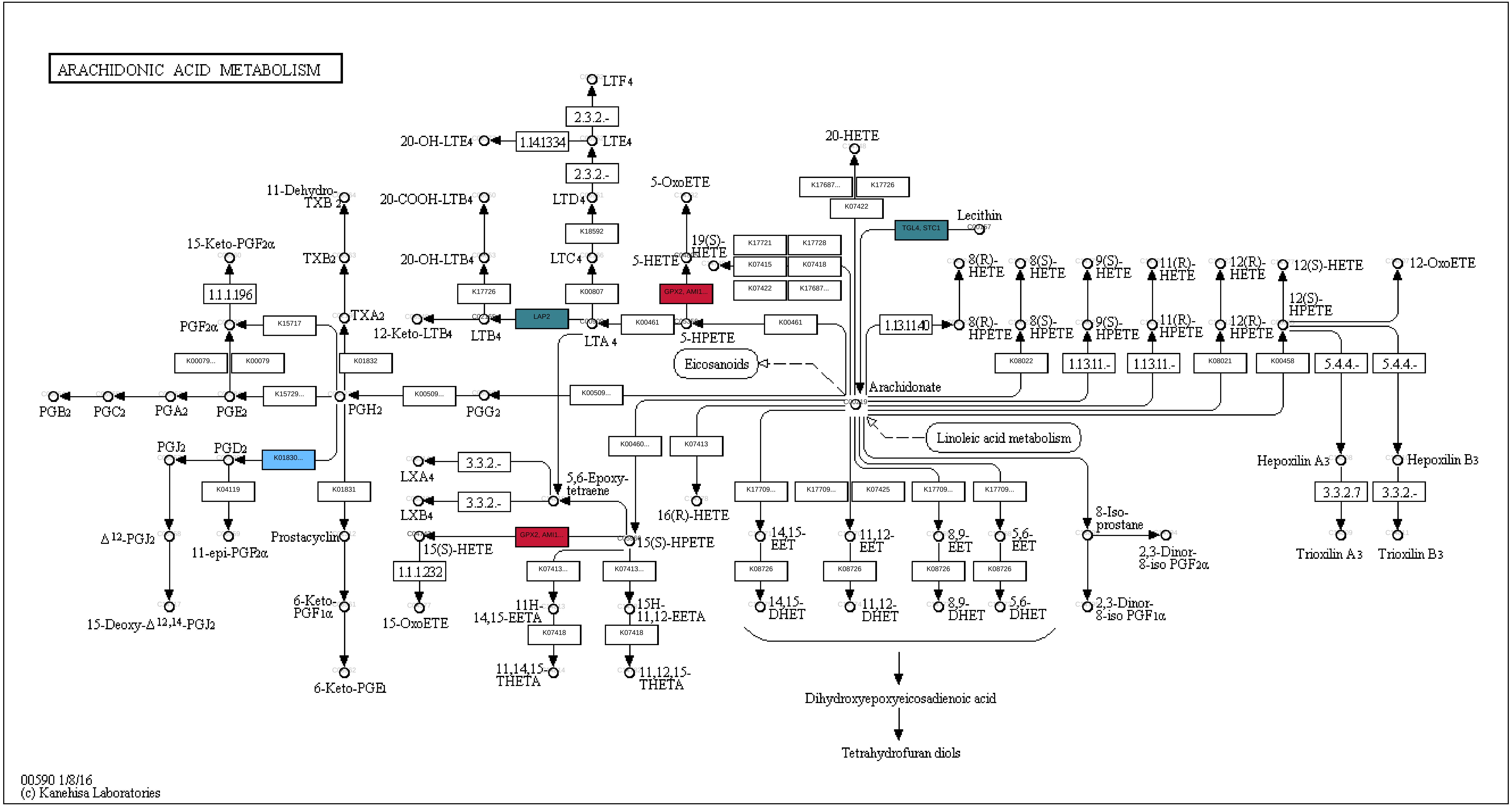

ARACHIDONIC ACID METABOLISM
LTF4
2.3.2.-
20-OH-LTE4
1.14.13.34
LTE4
2.3.2.-
LTD4
K18592
LTC4
K00807
20-COOH-LTB4
20-OH-LTB4
K17726
12-Keto-LTB4
LTB4
LAP2
LTA4
K00461
5-OxoETE
19(S)-HETE
5-HETE
GPX2, AMI1...
5-HPETE
K00461
Eicosanoids
11-Dehydro-TXB2
TXB2
TXA2
K01832
15-Keto-PGF2α
1.1.1.196
PGF2α
K15717
K00079...
K00079
PGB2
PGC2
PGA2
PGE2
K15729...
PGH2
K00509...
PGG2
K00509...
PGJ2
PGD2
K01830...
K04119
K01831
Δ12-PGJ2
11-epi-PGF2α
Prostacyclin
15-Deoxy-Δ12,14-PGJ2
6-Keto-PGF1α
6-Keto-PGE1
LXA4
3.3.2.-
LXB4
3.3.2.-
5,6-Epoxy-tetraene
GPX2, AMI1...
15(S)-HETE
15(S)-HPETE
1.1.1.232
15-OxoETE
K07413...
K07413...
11H-14,15-EETA
15H-11,12-EETA
K07418
K07418
11,14,15-THETA
11,12,15-THETA
K00460...
K07413
16(R)-HETE
20-HETE
K17687...
K17726
K07422
K17721
K17728
K07415
K07418
K07422
K17687...
TGL4, STC1
Lecithin
Arachidonate
Linoleic acid metabolism
1.13.11.40
8(R)-HETE
8(R)-HPETE
8(S)-HETE
8(S)-HPETE
9(S)-HETE
9(S)-HPETE
11(R)-HETE
11(R)-HPETE
12(R)-HETE
12(R)-HPETE
12(S)-HETE
12(S)-HPETE
12-OxoETE
K08022
1.13.11.-
1.13.11.-
K08021
K00458
5.4.4.-
5.4.4.-
Hepoxilin A3
Hepoxilin B3
3.3.2.7
3.3.2.-
Trioxilin A3
Trioxilin B3
K17709...
K17709...
K07425
K17709...
K17709...
14,15-EET
11,12-EET
8,9-EET
5,6-EET
K08726
K08726
K08726
K08726
14,15-DHET
11,12-DHET
8,9-DHET
5,6-DHET
8-Iso-prostane
2,3-Dinor-8-iso PGF2α
2,3-Dinor-8-iso PGF1α
Dihydroxyepoxyeicosadienoic acid
Tetrahydrofuran diols
00590 1/8/16
(c) Kanehisa Laboratories

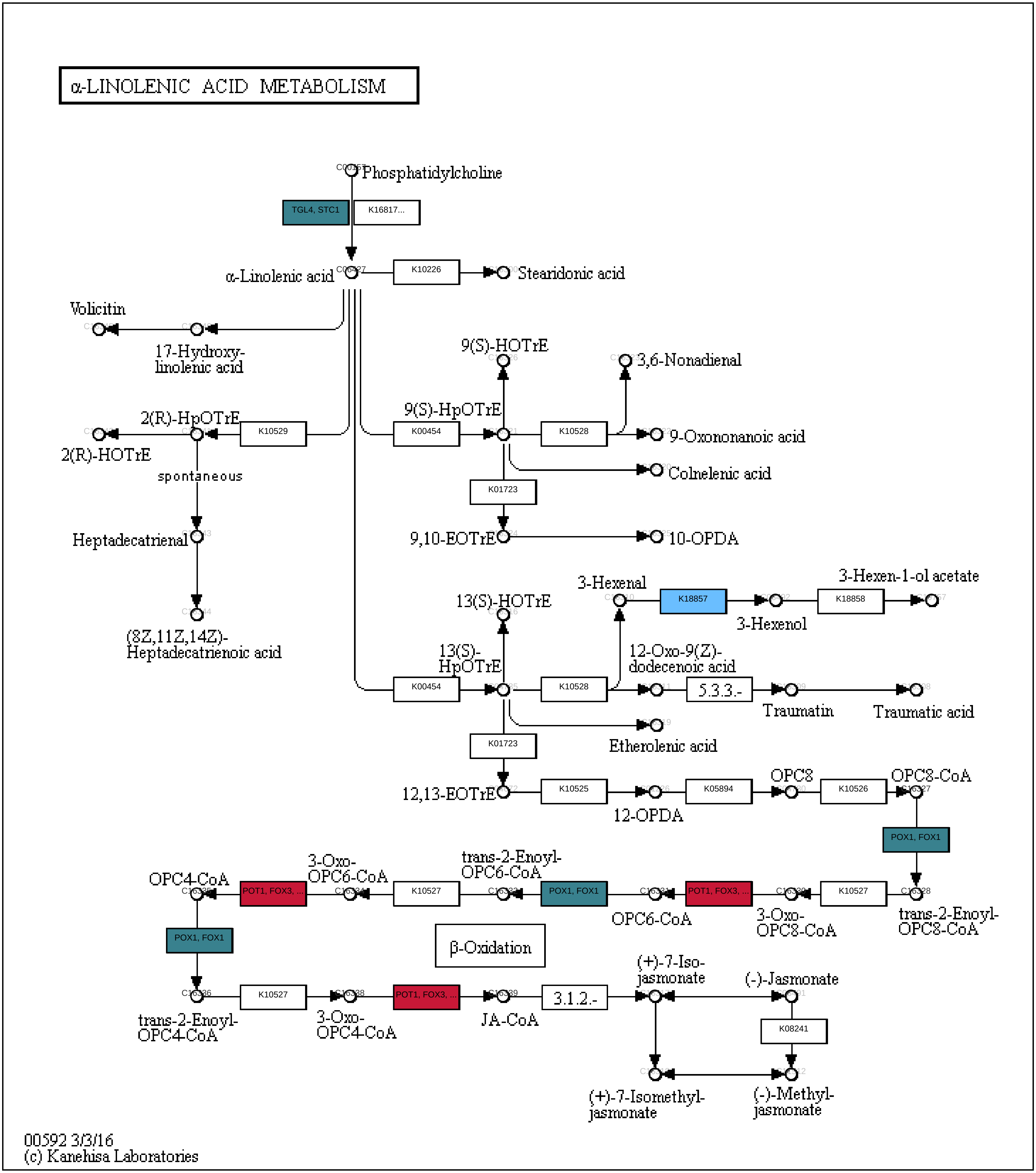
α-LINOLENIC ACID METABOLISM
Phosphatidylcholine
TGL4, STC1
K16817...
α-Linolenic acid
K10226
Stearidonic acid
Volicitin
17-Hydroxy-
linolenic acid
9(S)-HOTrE
3,6-Nonadienal
9(S)-HpOTrE
2(R)-HpOTrE
K10529
K00454
K10528
9-Oxononanoic acid
2(R)-HOTrE
spontaneous
Colnelenic acid
K01723
Heptadecatrienal
9,10-EOTrE
10-OPDA
(8Z,11Z,14Z)-
Heptadecatrienoic acid
3-Hexenal
K18857
K18858
3-Hexen-1-ol acetate
3-Hexenol
13(S)-HOTrE
13(S)-
HpOTrE
12-Oxo-9(Z)-
dodecenoic acid
K00454
K10528
5.3.3.-
Traumatin
Traumatic acid
Etherolenic acid
K01723
12,13-EOTrE
K10525
12-OPDA
K05894
OPC8
K10526
OPC8-CoA
POX1, FOX1
OPC4-CoA
POT1, FOX3, ...
3-Oxo-
OPC6-CoA
K10527
trans-2-Enoyl-
OPC6-CoA
POX1, FOX1
POT1, FOX3, ...
K10527
OPC6-CoA
3-Oxo-
OPC8-CoA
trans-2-Enoyl-
OPC8-CoA
POX1, FOX1
β-Oxidation
K10527
POT1, FOX3, ...
3.1.2.-
(+)-7-Iso-
jasmonate
(-)-Jasmonate
trans-2-Enoyl-
OPC4-CoA
3-Oxo-
OPC4-CoA
JA-CoA
K08241
(+)-7-Isomethyl-
jasmonate
(-)-Methyl-
jasmonate
00592 3/3/16
(c) Kanehisa Laboratories

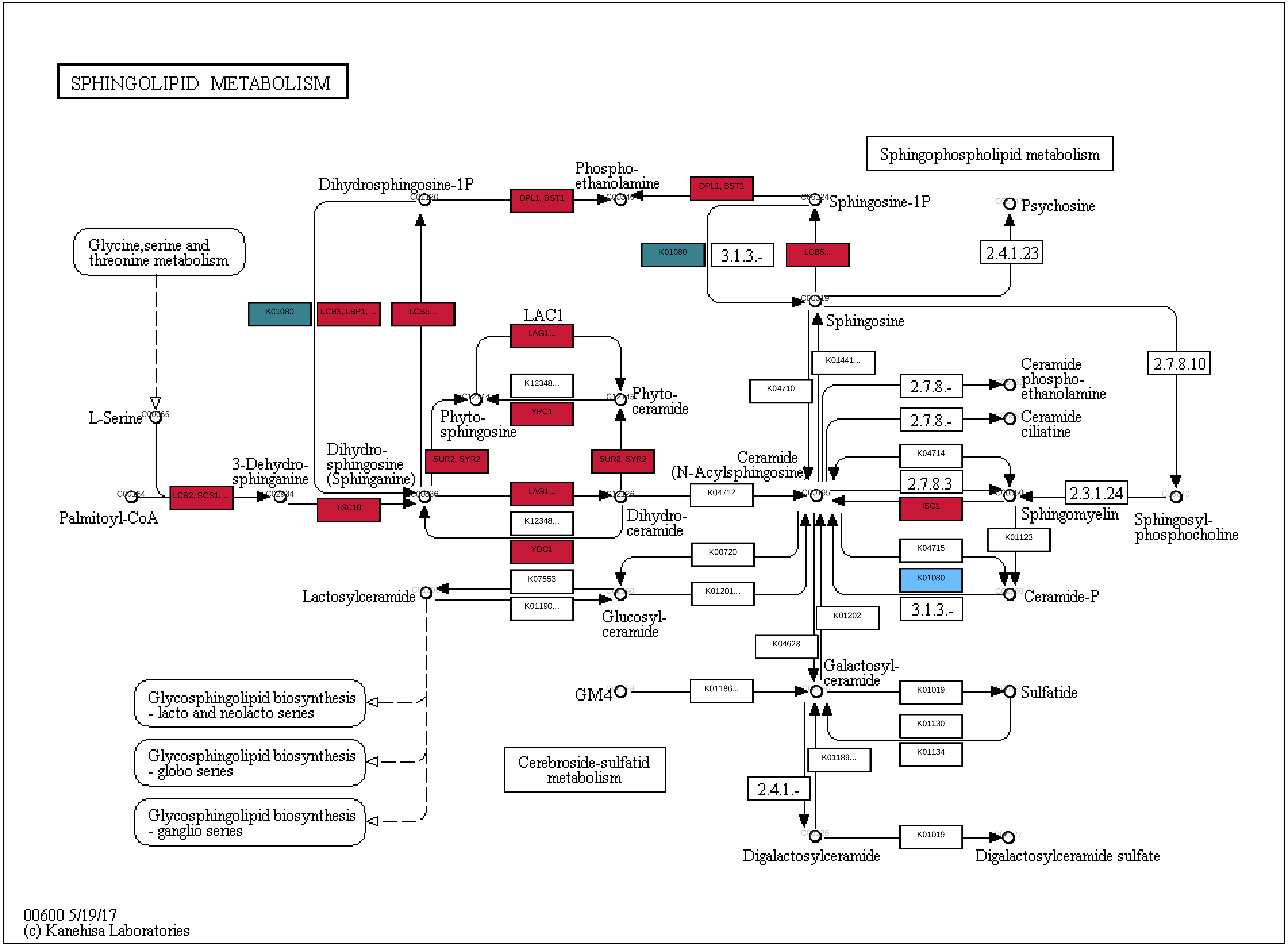

SPHINGOLIPID METABOLISM
Sphingophospholipid metabolism
Glycine,serine and threonine metabolism
Dihydrosphingosine-1P
Phospho-ethanolamine
Sphingosine-1P
Psychosine
DPL1, BST1
DPL1, BST1
K01080
3.1.3.-
LCB5...
2.4.1.23
K01080
LCB3, LBP1, ...
LCB5...
Sphingosine
LAC1
LAG1...
K12348...
YPC1
Phyto-ceramide
Phyto-sphingosine
K01441...
K04710
2.7.8.-
Ceramide phospho-ethanolamine
2.7.8.-
Ceramide ciliatine
2.7.8.10
L-Serine
3-Dehydro-sphinganine
Dihydro-sphingosine (Sphinganine)
SUR2, SYR2
SUR2, SYR2
Ceramide (N-Acylsphingosine)
K04714
2.7.8.3
Palmitoyl-CoA
LCB2, SCS1, ...
TSC10
LAG1...
K12348...
K04712
ISC1
2.3.1.24
Sphingomyelin
Sphingosyl-phosphocholine
Dihydro-ceramide
YDC1
K00720
K04715
K01123
K01080
K07553
Lactosylceramide
K01190...
K01201...
Glucosyl-ceramide
Ceramide-P
3.1.3.-
K01202
K04628
Galactosyl-ceramide
GM4
K01186...
K01019
Sulfatide
K01130
K01134
Glycosphingolipid biosynthesis - lacto and neolacto series
Glycosphingolipid biosynthesis - globo series
Glycosphingolipid biosynthesis - ganglio series
Cerebroside-sulfatid metabolism
K01189...
2.4.1.-
K01019
Digalactosylceramide
Digalactosylceramide sulfate
00600 5/19/17
(c) Kanehisa Laboratories

# 4. Nucleotide metabolism

| MAP | PATHWAY |
|---|---|
| **230** | Purine metabolism |
| **240** | Pyrimidine metabolism |

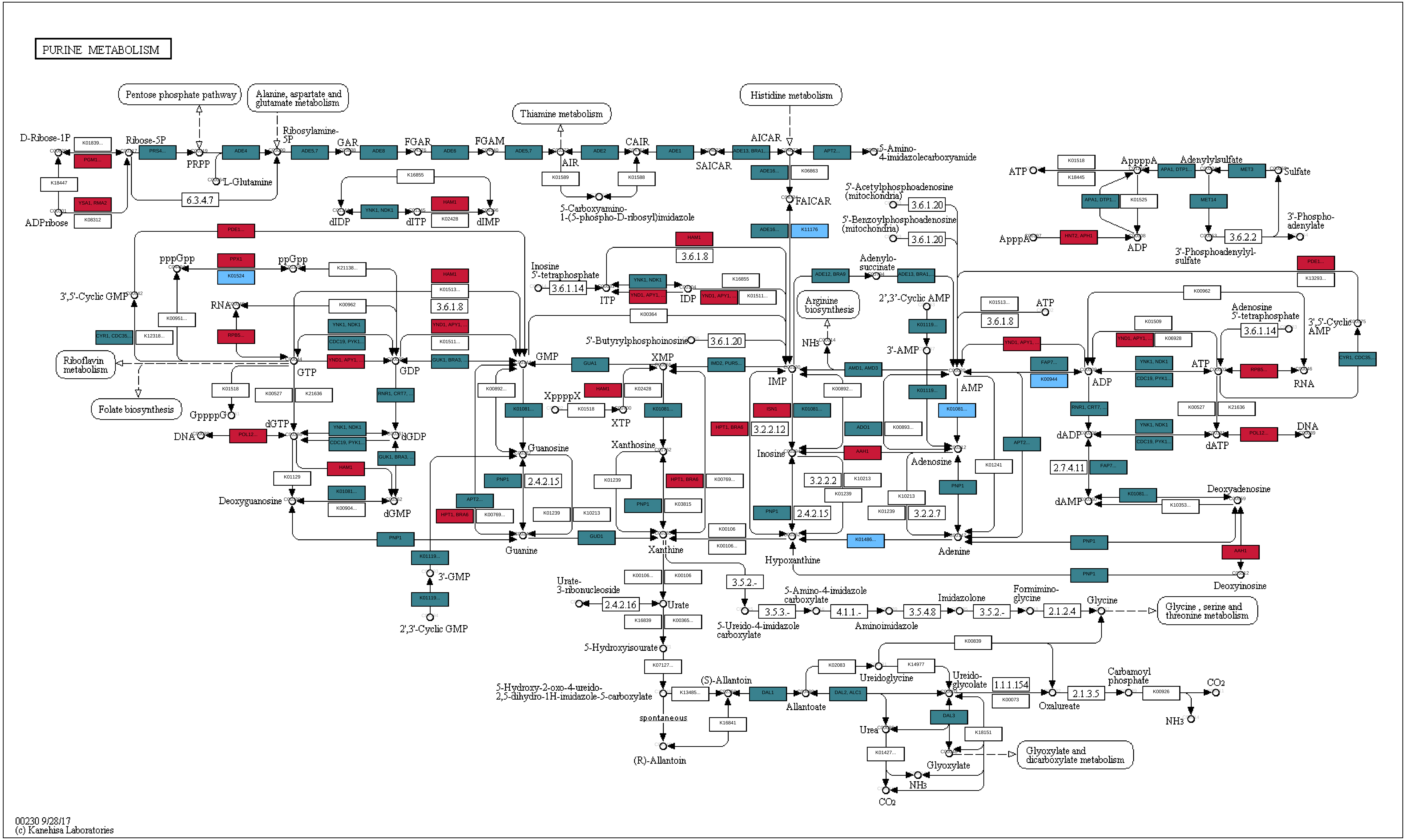
PURINE METABOLISM
Pentose phosphate pathway
Alanine, aspartate and glutamate metabolism
Thiamine metabolism
Histidine metabolism
D-Ribose-1P
Ribose-5P
PRPP
L-Glutamine
Ribosylamine-5P
GAR
FGAR
FGAM
AIR
CAIR
SAICAR
AICAR
5-Amino-4-imidazolecarboxyamide
FAICAR
5-Carboxyamino-1-(5-phospho-D-ribosyl)imidazole
ADPribose
6.3.4.7
dIDP
dITP
dIMP
Inosine 5'-tetraphosphate
3.6.1.14
ITP
IDP
3.6.1.8
ppGpp
pppGpp
3',5'-Cyclic GMP
RNA
Riboflavin metabolism
Folate biosynthesis
GppppG
GTP
GDP
GMP
dGTP
dGDP
dGMP
DNA
Deoxyguanosine
Guanosine
Guanine
2.4.2.15
3'-GMP
2',3'-Cyclic GMP
XppppX
XTP
XMP
Xanthosine
Xanthine
5'-Butyrylphosphoinosine
3.6.1.20
IMP
Inosine
Hypoxanthine
3.2.2.12
3.2.2.2
2.4.2.15
Arginine biosynthesis
NH3
Adenylosuccinate
2',3'-Cyclic AMP
3'-AMP
AMP
Adenosine
Adenine
3.2.2.7
5'-Acetylphosphoadenosine (mitochondria)
5'-Benzoylphosphoadenosine (mitochondria)
ATP
ApppA
AppppA
ADP
Adenylylsulfate
Sulfate
3'-Phosphoadenylyl-sulfate
3'-Phospho-adenylate
3.6.2.2
Adenosine 5'-tetraphosphate
3',5'-Cyclic AMP
RNA
dADP
dATP
dAMP
DNA
2.7.4.11
Deoxyadenosine
Deoxyinosine
Urate-3-ribonucleoside
2.4.2.16
Urate
5-Hydroxyisourate
5-Hydroxy-2-oxo-4-ureido-2,5-dihydro-1H-imidazole-5-carboxylate
spontaneous
(R)-Allantoin
(S)-Allantoin
Allantoate
Ureidoglycine
Ureido-glycolate
1.1.1.154
Urea
CO2
NH3
Glyoxylate
Glyoxylate and dicarboxylate metabolism
Oxalureate
2.1.3.5
Carbamoyl phosphate
5-Amino-4-imidazolecarboxylate
5-Ureido-4-imidazole carboxylate
3.5.2.-
3.5.3.-
4.1.1.-
3.5.4.8
Aminoimidazole
Imidazolone
Formimino-glycine
2.1.2.4
Glycine
Glycine, serine and threonine metabolism
00230 9/28/17
(c) Kanehisa Laboratories

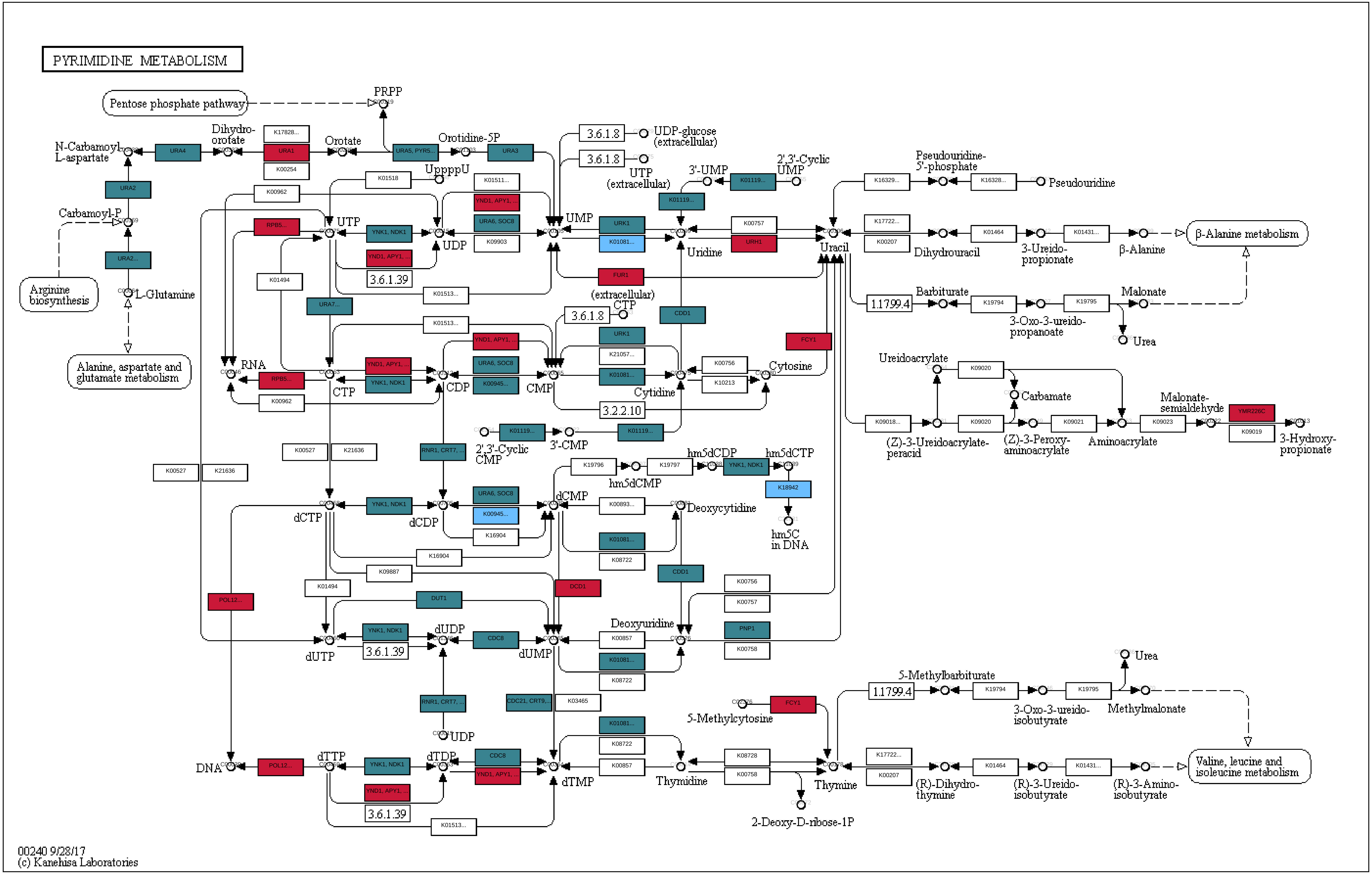
PYRIMIDINE METABOLISM
Pentose phosphate pathway
PRPP
N-Carbamoyl-
L-aspartate
Dihydro-
orotate
Orotate
Orotidine-5P
URA4
URA1
K17828...
K00254
URA5, PYR5...
URA3
URA2
Carbamoyl-P
URA2...
Arginine
biosynthesis
L-Glutamine
Alanine, aspartate and
glutamate metabolism
3.6.1.8
UDP-glucose
(extracellular)
3.6.1.8
UTP
(extracellular)
UppppU
K01518
K01511...
K00962
YND1, APY1, ...
URA6, SOC8
K09903
RPB5...
UTP
YNK1, NDK1
UDP
UMP
YND1, APY1, ...
3.6.1.39
K01494
K01513...
URK1
K01081...
FUR1
(extracellular)
3'-UMP
K01119...
2',3'-Cyclic
UMP
K01119...
K00757
URH1
Uridine
Uracil
URA7...
K01513...
YND1, APY1, ...
URA6, SOC8
K00945...
RNA
RPB5...
YND1, APY1, ...
YNK1, NDK1
CTP
CDP
CMP
K00962
3.6.1.8
CTP
URK1
K21057...
K01081...
Cytidine
CDD1
K00756
K10213
Cytosine
FCY1
3.2.2.10
K01119...
K01119...
2',3'-Cyclic
CMP
3'-CMP
K00527
K21636
K00527
K21636
RNR1, CRT7, ...
hm5dCDP
hm5dCTP
K19796
hm5dCMP
K19797
YNK1, NDK1
K18942
hm5C
in DNA
dCTP
YNK1, NDK1
dCDP
URA6, SOC8
K00945...
K16904
dCMP
K00893...
Deoxycytidine
K01081...
K08722
K16904
K09887
K01494
DCD1
CDD1
K00756
K00757
POL12...
DUT1
YNK1, NDK1
dUTP
3.6.1.39
dUDP
CDC8
dUMP
Deoxyuridine
K00857
K01081...
K08722
PNP1
K00758
RNR1, CRT7, ...
CDC21, CRT9,...
K03465
UDP
DNA
POL12...
dTTP
YNK1, NDK1
YND1, APY1, ...
3.6.1.39
K01513...
dTDP
CDC8
YND1, APY1, ...
dTMP
K01081...
K08722
K00857
Thymidine
K08728
K00758
2-Deoxy-D-ribose-1P
5-Methylcytosine
FCY1
Thymine
Pseudouridine-
5'-phosphate
K16329...
K16328...
Pseudouridine
K17722...
K00207
Dihydrouracil
K01464
3-Ureido-
propionate
K01431...
β-Alanine
β-Alanine metabolism
1.17.99.4
Barbiturate
K19794
3-Oxo-3-ureido-
propanoate
K19795
Malonate
Urea
Ureidoacrylate
K09020
Carbamate
K09018...
(Z)-3-Ureidoacrylate-
peracid
K09020
(Z)-3-Peroxy-
aminoacrylate
K09021
Aminoacrylate
K09023
Malonate-
semialdehyde
YMR226C
K09019
3-Hydroxy-
propionate
Urea
5-Methylbarbiturate
1.17.99.4
K19794
K19795
3-Oxo-3-ureido-
isobutyrate
Methylmalonate
Valine, leucine and
isoleucine metabolism
K17722...
K00207
(R)-Dihydro-
thymine
K01464
(R)-3-Ureido-
isobutyrate
K01431...
(R)-3-Amino-
isobutyrate
00240 9/28/17
(c) Kanehisa Laboratories

# 5. Amino Acid metabolism

| MAP | PATHWAY |
|---|---|
| **220** | Arginine biosynthesis |
| **250** | Alanine, aspartate and glutamate metabolism |
| **260** | Glycine, serine and threonine metabolism |
| **270** | Cysteine and methionine metabolism |
| **280** | Valine, leucine and isoleucine degradation |
| **290** | Valine, leucine and isoleucine biosynthesis |
| **300** | Lysine biosynthesis |
| **310** | Lysine degradation |
| **330** | Arginine and proline metabolism |
| **340** | Histidine metabolism |
| **350** | Tyrosine metabolism |
| **360** | Phenylalanine metabolism |
| **380** | Tryptophan metabolism |
| **400** | Phenylalanine, tyrosine and tryptophan biosynthesis |

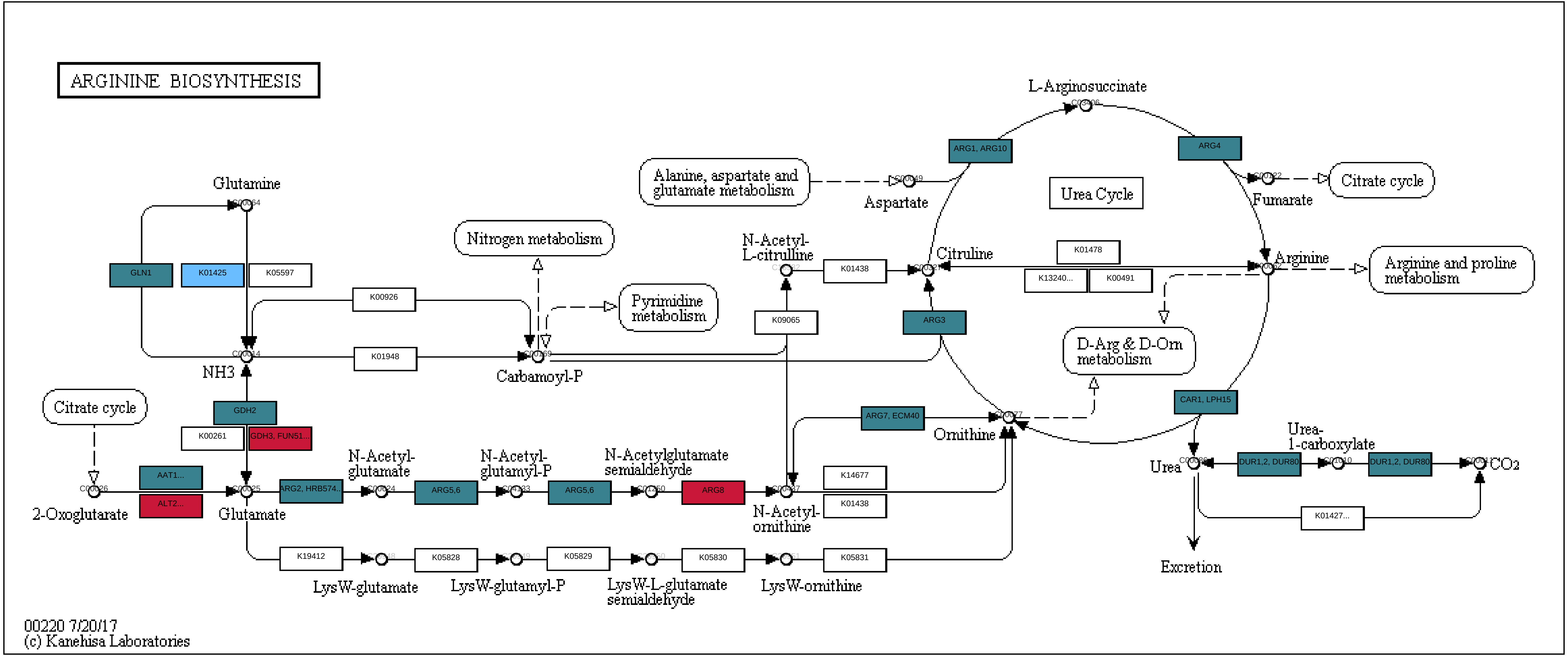

ARGININE BIOSYNTHESIS
L-Arginosuccinate
ARG1, ARG10
ARG4
Alanine, aspartate and glutamate metabolism
Aspartate
Urea Cycle
Citrate cycle
Fumarate
Glutamine
GLN1
K01425
K05597
Nitrogen metabolism
N-Acetyl-L-citrulline
K01438
Citruline
K01478
K13240...
K00491
Arginine
Arginine and proline metabolism
K00926
Pyrimidine metabolism
K09065
ARG3
D-Arg & D-Orn metabolism
NH3
K01948
Carbamoyl-P
Citrate cycle
GDH2
K00261
GDH3, FUN51...
CAR1, LPH15
ARG7, ECM40
Ornithine
Urea-1-carboxylate
N-Acetyl-glutamate
N-Acetyl-glutamyl-P
N-Acetylglutamate semialdehyde
AAT1...
ALT2...
ARG2, HRB574...
ARG5,6
ARG5,6
ARG8
K14677
K01438
Urea
DUR1,2, DUR80
DUR1,2, DUR80
CO2
2-Oxoglutarate
Glutamate
N-Acetyl-ornithine
K01427...
Excretion
K19412
K05828
K05829
K05830
K05831
LysW-glutamate
LysW-glutamyl-P
LysW-L-glutamate semialdehyde
LysW-ornithine
00220 7/20/17
(c) Kanehisa Laboratories

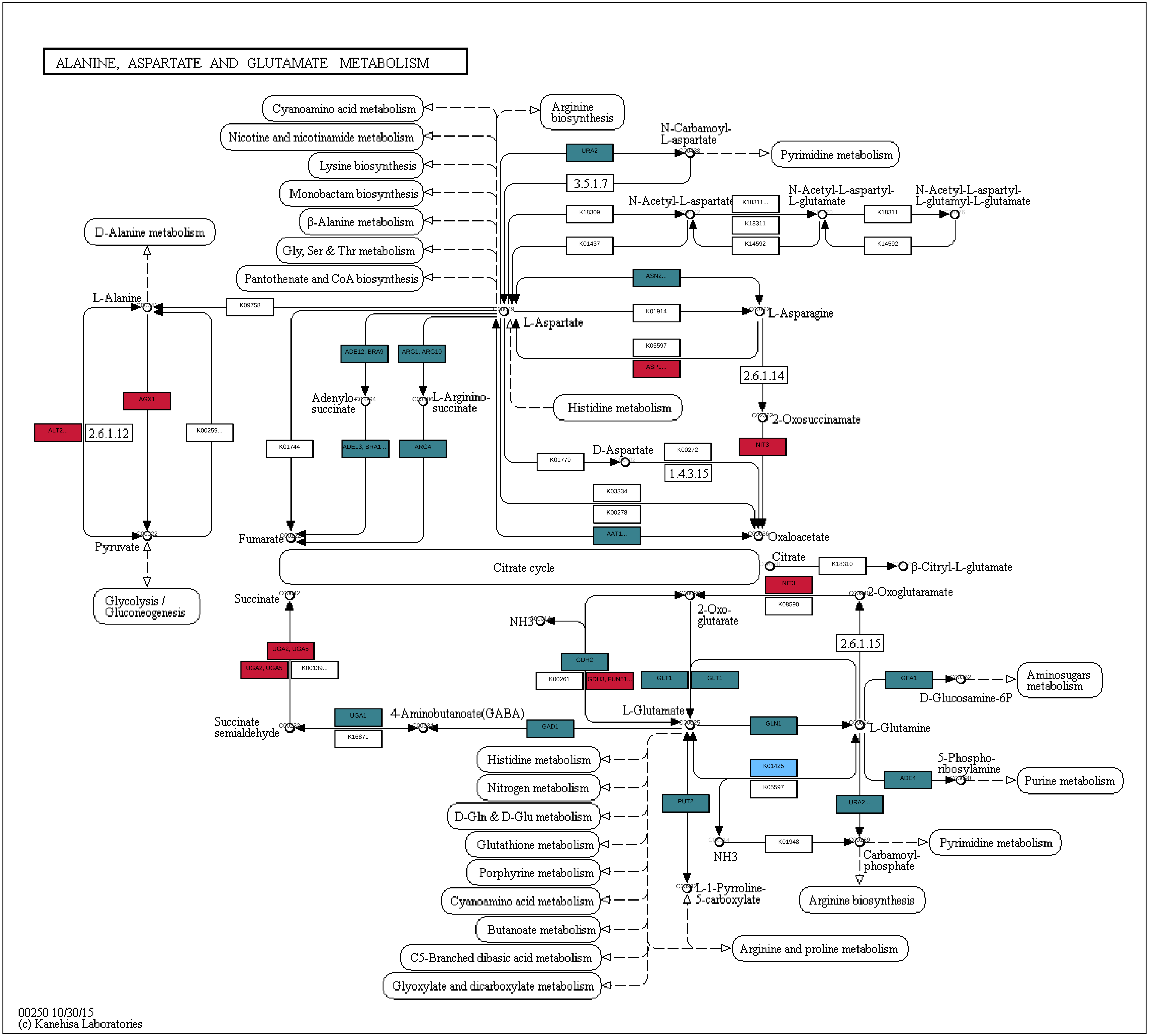
ALANINE, ASPARTATE AND GLUTAMATE METABOLISM
Cyanoamino acid metabolism
Nicotine and nicotinamide metabolism
Lysine biosynthesis
Monobactam biosynthesis
β-Alanine metabolism
Gly, Ser & Thr metabolism
Pantothenate and CoA biosynthesis
Arginine biosynthesis
N-Carbamoyl-L-aspartate
Pyrimidine metabolism
URA2
3.5.1.7
N-Acetyl-L-aspartate
N-Acetyl-L-aspartyl-L-glutamate
N-Acetyl-L-aspartyl-L-glutamyl-L-glutamate
K18309
K18311...
K18311
K14592
K01437
K18311
K14592
D-Alanine metabolism
L-Alanine
K09758
ASN2...
K01914
L-Asparagine
L-Aspartate
K05597
ASP1...
2.6.1.14
ADE12, BRA9
ARG1, ARG10
AGX1
Adenylo-succinate
L-Arginino-succinate
Histidine metabolism
2-Oxosuccinamate
ALT2...
2.6.1.12
K00259...
K01744
ADE13, BRA1,...
ARG4
NIT3
K01779
D-Aspartate
K00272
1.4.3.15
K03334
K00278
AAT1...
Pyruvate
Fumarate
Oxaloacetate
Citrate cycle
Citrate
K18310
β-Citryl-L-glutamate
NIT3
K08590
Glycolysis / Gluconeogenesis
Succinate
NH3
2-Oxo-glutarate
2-Oxoglutaramate
2.6.1.15
UGA2, UGA5
UGA2, UGA5
K00139...
GDH2
K00261
GDH3, FUN51...
GLT1
GLT1
GFA1
Aminosugars metabolism
D-Glucosamine-6P
L-Glutamate
Succinate semialdehyde
UGA1
K16871
4-Aminobutanoate(GABA)
GAD1
GLN1
L-Glutamine
5-Phospho-ribosylamine
ADE4
Purine metabolism
Histidine metabolism
Nitrogen metabolism
D-Gln & D-Glu metabolism
Glutathione metabolism
Porphyrine metabolism
Cyanoamino acid metabolism
Butanoate metabolism
C5-Branched dibasic acid metabolism
Glyoxylate and dicarboxylate metabolism
K01425
K05597
PUT2
URA2...
NH3
K01948
Carbamoyl-phosphate
Pyrimidine metabolism
Arginine biosynthesis
L-1-Pyrroline-5-carboxylate
Arginine and proline metabolism
00250 10/30/15
(c) Kanehisa Laboratories

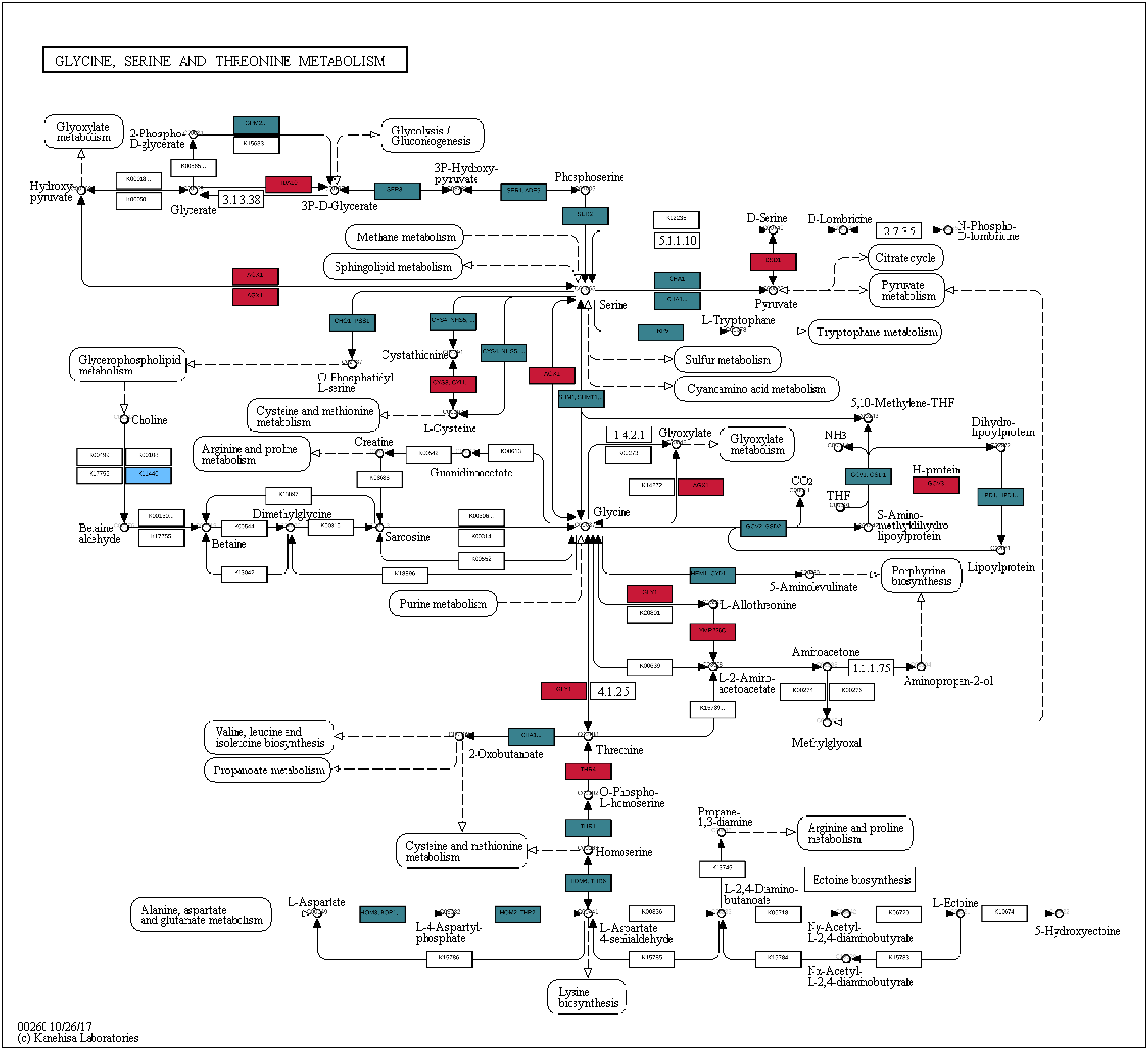
GLYCINE, SERINE AND THREONINE METABOLISM
Glyoxylate metabolism
2-Phospho-D-glycerate
GPM2...
K15633...
Glycolysis / Gluconeogenesis
K00865...
K00018...
K00050...
TDA10
Hydroxy-pyruvate
Glycerate
3.1.3.38
3P-D-Glycerate
SER3...
3P-Hydroxy-pyruvate
SER1, ADE9
Phosphoserine
SER2
K12235
5.1.1.10
D-Serine
D-Lombricine
2.7.3.5
N-Phospho-D-lombricine
DSD1
Citrate cycle
Pyruvate metabolism
Methane metabolism
Sphingolipid metabolism
AGX1
AGX1
CHA1
CHA1...
Serine
Pyruvate
TRP5
L-Tryptophane
Tryptophane metabolism
CHO1, PSS1
CYS4, NHS5, ...
CYS4, NHS5, ...
Glycerophospholipid metabolism
Cystathionine
O-Phosphatidyl-L-serine
CYS3, CYI1, ...
AGX1
Sulfur metabolism
Cyanoamino acid metabolism
SHM1, SHMT1...
Cysteine and methionine metabolism
L-Cysteine
5,10-Methylene-THF
Choline
Arginine and proline metabolism
Creatine
K00542
K00613
Guanidinoacetate
1.4.2.1
Glyoxylate
K00273
Glyoxylate metabolism
NH3
Dihydro-lipoylprotein
K00499
K00108
K17755
K11440
K08688
K14272
AGX1
CO2
GCV1, GSD1
H-protein
GCV3
THF
LPD1, HPD1...
K18897
Dimethylglycine
K00130...
K00544
K00315
K00306...
Glycine
S-Amino-methyldihydro-lipoylprotein
Betaine aldehyde
K17755
Betaine
Sarcosine
K00314
GCV2, GSD2
K00552
K13042
K18896
HEM1, CYD1, ...
5-Aminolevulinate
Porphyrine biosynthesis
Lipoylprotein
Purine metabolism
GLY1
L-Allothreonine
K20801
YMR226C
Aminoacetone
K00639
1.1.1.75
Aminopropan-2-ol
L-2-Amino-acetoacetate
K00274
K00276
GLY1
4.1.2.5
K15789...
Methylglyoxal
Valine, leucine and isoleucine biosynthesis
CHA1...
Threonine
2-Oxobutanoate
Propanoate metabolism
THR4
O-Phospho-L-homoserine
Propane-1,3-diamine
Arginine and proline metabolism
THR1
Cysteine and methionine metabolism
Homoserine
K13745
Ectoine biosynthesis
HOM6, THR6
L-2,4-Diamino-butanoate
L-Aspartate
L-Ectoine
Alanine, aspartate and glutamate metabolism
HOM3, BOR1, ...
HOM2, THR2
K00836
K06718
K06720
K10674
L-4-Aspartyl-phosphate
L-Aspartate 4-semialdehyde
Nγ-Acetyl-L-2,4-diaminobutyrate
5-Hydroxyectoine
K15786
K15785
K15784
K15783
Nα-Acetyl-L-2,4-diaminobutyrate
Lysine biosynthesis
00260 10/26/17
(c) Kanehisa Laboratories

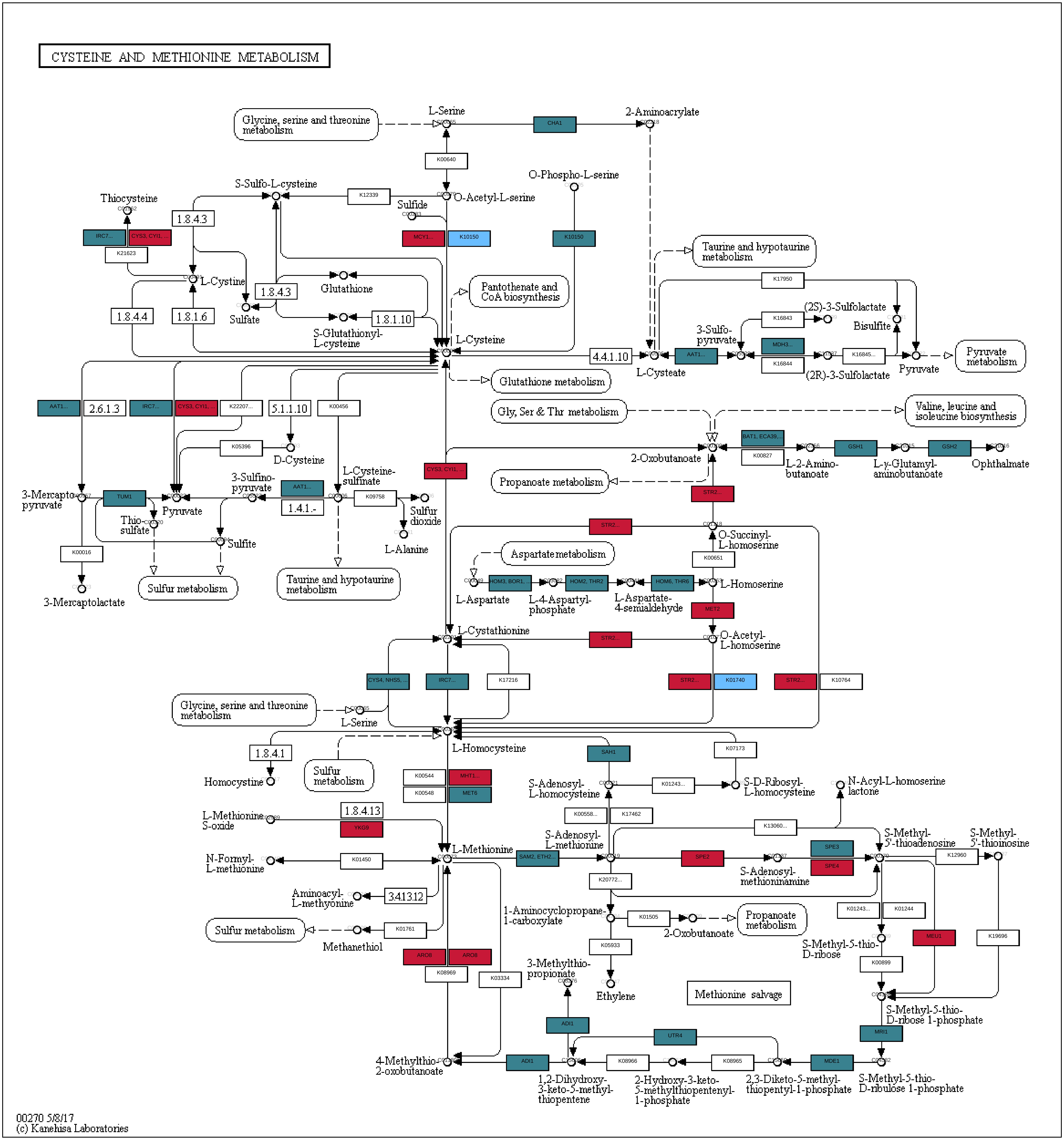

CYSTEINE AND METHIONINE METABOLISM
Glycine, serine and threonine metabolism
L-Serine
CHA1
2-Aminoacrylate
K00640
O-Phospho-L-serine
S-Sulfo-L-cysteine
K12339
Sulfide
O-Acetyl-L-serine
Thiocysteine
1.8.4.3
IRC7...
CYS3, CYI1, ...
MCY1...
K10150
K10150
K21623
L-Cystine
1.8.4.3
Glutathione
Pantothenate and CoA biosynthesis
Taurine and hypotaurine metabolism
K17950
1.8.4.4
1.8.1.6
Sulfate
S-Glutathionyl-L-cysteine
1.8.1.10
L-Cysteine
4.4.1.10
L-Cysteate
AAT1...
3-Sulfo-pyruvate
K16843
(2S)-3-Sulfolactate
Bisulfite
MDH3...
K16844
K16845...
(2R)-3-Sulfolactate
Pyruvate
Pyruvate metabolism
Glutathione metabolism
AAT1...
2.6.1.3
IRC7...
CYS3, CYI1, ...
K22207...
5.1.1.10
K00456
Gly, Ser & Thr metabolism
Valine, leucine and isoleucine biosynthesis
K05396
D-Cysteine
BAT1, ECA39...
GSH1
GSH2
K00827
2-Oxobutanoate
L-2-Amino-butanoate
L-γ-Glutamyl-aminobutanoate
Ophthalmate
CYS3, CYI1, ...
Propanoate metabolism
3-Sulfino-pyruvate
AAT1...
L-Cysteine-sulfinate
3-Mercapto-pyruvate
TUM1
Pyruvate
1.4.1.-
K09758
Sulfur dioxide
STR2...
Thio-sulfate
Sulfite
L-Alanine
STR2...
O-Succinyl-L-homoserine
K00016
Aspartate metabolism
K00651
Sulfur metabolism
Taurine and hypotaurine metabolism
HOM3, BOR1, ...
HOM2, THR2
HOM6, THR6
L-Homoserine
3-Mercaptolactate
L-Aspartate
L-4-Aspartyl-phosphate
L-Aspartate-4-semialdehyde
MET2
L-Cystathionine
STR2...
O-Acetyl-L-homoserine
CYS4, NHS5, ...
IRC7...
K17216
STR2...
K01740
STR2...
K10764
Glycine, serine and threonine metabolism
L-Serine
L-Homocysteine
1.8.4.1
Sulfur metabolism
SAH1
K07173
Homocystine
K00544
MHT1...
K00548
MET6
S-Adenosyl-L-homocysteine
K01243...
S-D-Ribosyl-L-homocysteine
N-Acyl-L-homoserine lactone
L-Methionine S-oxide
1.8.4.13
YKG9
K00558...
K17462
K13060...
S-Adenosyl-L-methionine
S-Methyl-5'-thioadenosine
S-Methyl-5'-thioinosine
N-Formyl-L-methionine
K01450
L-Methionine
SAM2, ETH2...
SPE2
SPE3
SPE4
K12960
S-Adenosyl-methioninamine
K20772...
Aminoacyl-L-methyonine
3.4.13.12
1-Aminocyclopropane-1-carboxylate
K01505
2-Oxobutanoate
Propanoate metabolism
K01243...
K01244
Sulfur metabolism
K01761
Methanethiol
MEU1
K19696
S-Methyl-5-thio-D-ribose
K05933
ARO8
ARO8
3-Methylthio-propionate
K08969
K03334
K00899
Ethylene
Methionine salvage
S-Methyl-5-thio-D-ribose 1-phosphate
ADI1
MRI1
UTR4
4-Methylthio-2-oxobutanoate
ADI1
K08966
K08965
MDE1
1,2-Dihydroxy-3-keto-5-methyl-thiopentene
2-Hydroxy-3-keto-5-methylthiopentenyl-1-phosphate
2,3-Diketo-5-methyl-thiopentyl-1-phosphate
S-Methyl-5-thio-D-ribulose 1-phosphate
00270 5/8/17
(c) Kanehisa Laboratories

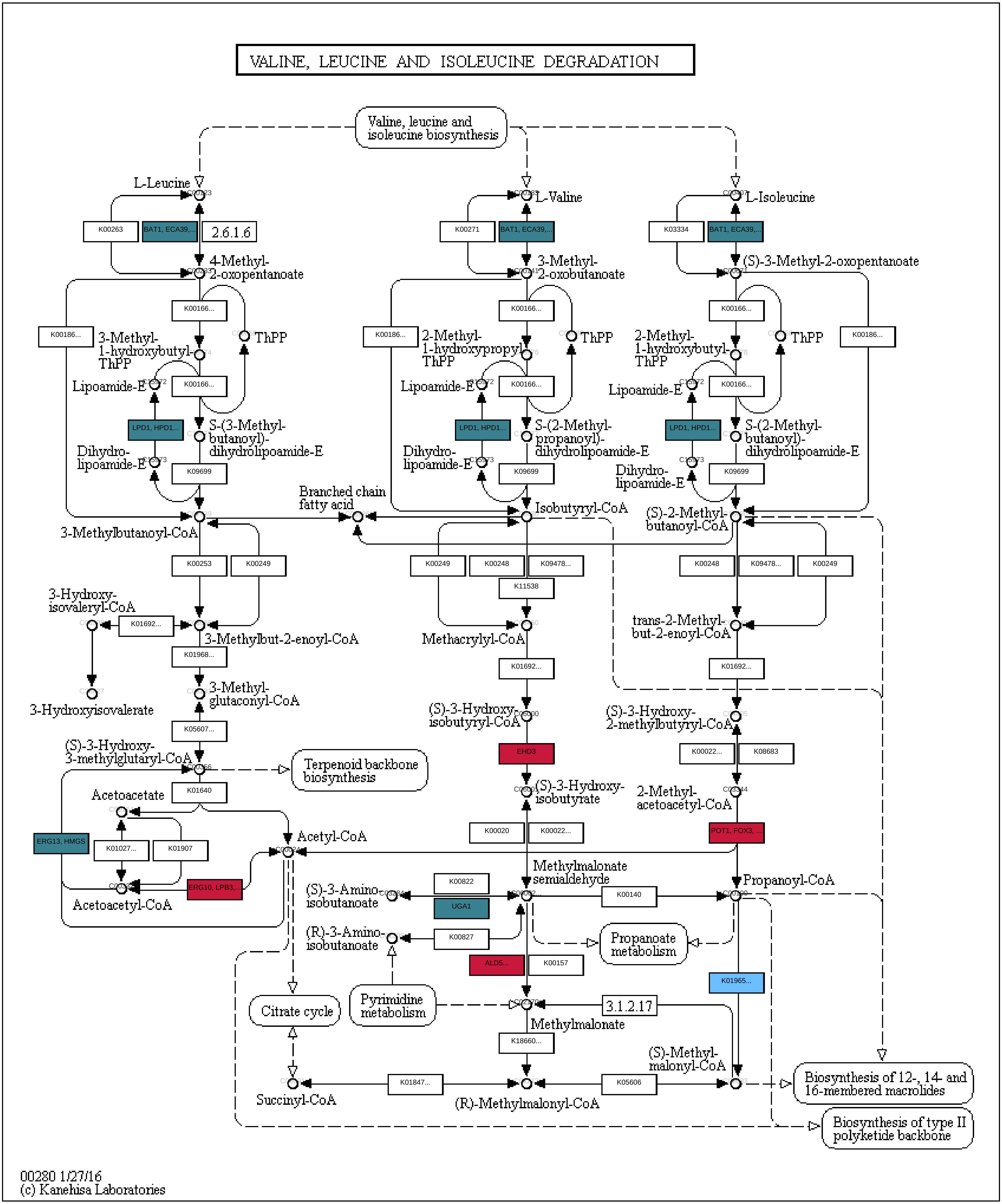

VALINE, LEUCINE AND ISOLEUCINE DEGRADATION
Valine, leucine and isoleucine biosynthesis
L-Leucine
L-Valine
L-Isoleucine
K00263
BAT1, ECA39,...
2.6.1.6
K00271
K03334
4-Methyl-2-oxopentanoate
3-Methyl-2-oxobutanoate
(S)-3-Methyl-2-oxopentanoate
K00166...
K00186...
ThPP
3-Methyl-1-hydroxybutyl-ThPP
2-Methyl-1-hydroxypropyl-ThPP
2-Methyl-1-hydroxybutyl-ThPP
Lipoamide-E
LPD1, HPD1...
S-(3-Methylbutanoyl)-dihydrolipoamide-E
S-(2-Methylpropanoyl)-dihydrolipoamide-E
S-(2-Methylbutanoyl)-dihydrolipoamide-E
Dihydrolipoamide-E
K09699
Branched chain fatty acid
3-Methylbutanoyl-CoA
Isobutyryl-CoA
(S)-2-Methylbutanoyl-CoA
K00253
K00249
K00248
K09478...
K11538
3-Hydroxyisovaleryl-CoA
K01692...
3-Methylbut-2-enoyl-CoA
Methacrylyl-CoA
trans-2-Methylbut-2-enoyl-CoA
K01968...
3-Hydroxyisovalerate
3-Methylglutaconyl-CoA
K05607...
(S)-3-Hydroxyisobutyryl-CoA
(S)-3-Hydroxy-2-methylbutyryl-CoA
(S)-3-Hydroxy-3-methylglutaryl-CoA
Terpenoid backbone biosynthesis
EHD3
K00022...
K08683
K01640
Acetoacetate
(S)-3-Hydroxyisobutyrate
2-Methylacetoacetyl-CoA
ERG13, HMGS
K01027...
K01907
Acetyl-CoA
K00020
POT1, FOX3, ...
ERG10, LPB3,...
Acetoacetyl-CoA
Methylmalonate semialdehyde
Propanoyl-CoA
K00822
UGA1
K00140
(S)-3-Aminoisobutanoate
(R)-3-Aminoisobutanoate
K00827
Propanoate metabolism
ALD5...
K00157
K01965...
Citrate cycle
Pyrimidine metabolism
3.1.2.17
Methylmalonate
K18660...
(S)-Methylmalonyl-CoA
K01847...
K05606
Succinyl-CoA
(R)-Methylmalonyl-CoA
Biosynthesis of 12-, 14- and 16-membered macrolides
Biosynthesis of type II polyketide backbone
00280 1/27/16
(c) Kanehisa Laboratories

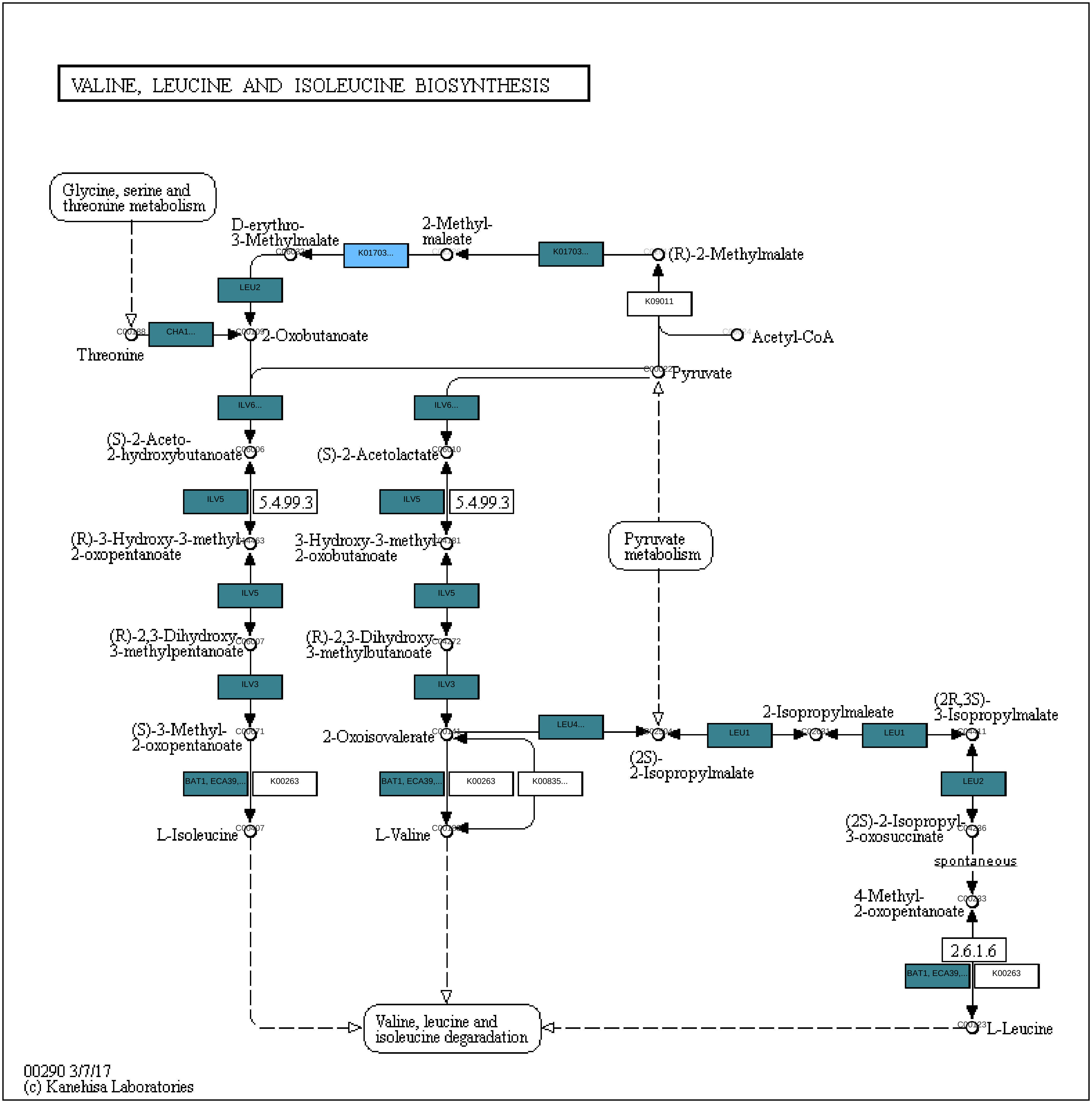
VALINE, LEUCINE AND ISOLEUCINE BIOSYNTHESIS
Glycine, serine and threonine metabolism
D-erythro-3-Methylmalate
K01703...
2-Methyl-maleate
K01703...
(R)-2-Methylmalate
K09011
Acetyl-CoA
LEU2
CHA1...
Threonine
2-Oxobutanoate
Pyruvate
ILV6...
ILV6...
(S)-2-Aceto-2-hydroxybutanoate
(S)-2-Acetolactate
ILV5
5.4.99.3
ILV5
5.4.99.3
(R)-3-Hydroxy-3-methyl-2-oxopentanoate
3-Hydroxy-3-methyl-2-oxobutanoate
Pyruvate metabolism
ILV5
ILV5
(R)-2,3-Dihydroxy-3-methylpentanoate
(R)-2,3-Dihydroxy-3-methylbutanoate
ILV3
ILV3
(S)-3-Methyl-2-oxopentanoate
2-Oxoisovalerate
LEU4...
2-Isopropylmaleate
(2R,3S)-3-Isopropylmalate
LEU1
LEU1
(2S)-2-Isopropylmalate
BAT1, ECA39,...
K00263
BAT1, ECA39,...
K00263
K00835...
LEU2
(2S)-2-Isopropyl-3-oxosuccinate
L-Isoleucine
L-Valine
spontaneous
4-Methyl-2-oxopentanoate
2.6.1.6
BAT1, ECA39,...
K00263
Valine, leucine and isoleucine degaradation
L-Leucine
00290 3/7/17
(c) Kanehisa Laboratories

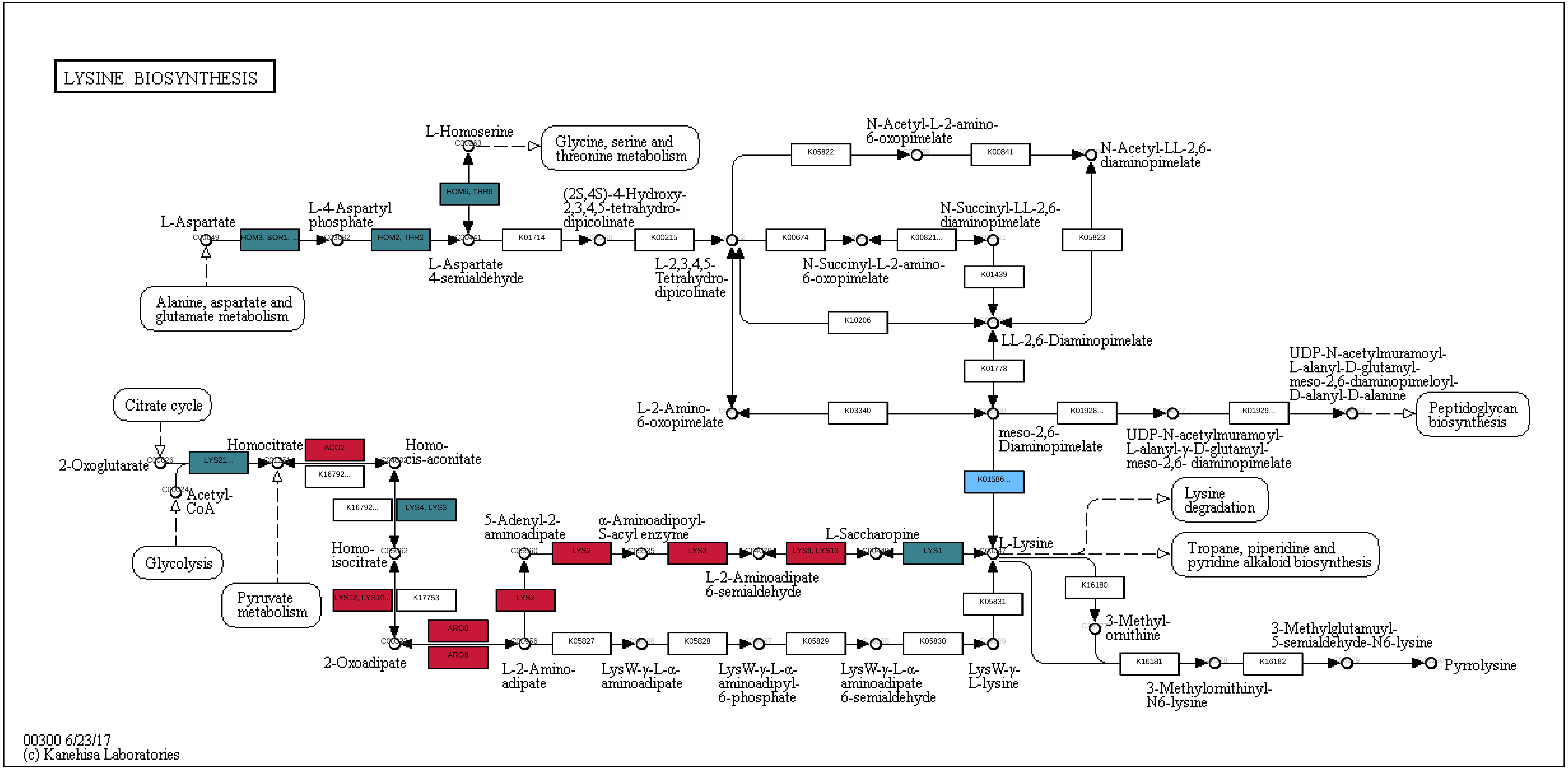
LYSINE BIOSYNTHESIS
L-Homoserine
Glycine, serine and threonine metabolism
HOM6, THR6
L-Aspartate
L-4-Aspartyl phosphate
(2S,4S)-4-Hydroxy-2,3,4,5-tetrahydro-dipicolinate
HOM3, BOR1, ...
HOM2, THR2
K01714
K00215
L-Aspartate 4-semialdehyde
L-2,3,4,5-Tetrahydro-dipicolinate
Alanine, aspartate and glutamate metabolism
N-Acetyl-L-2-amino-6-oxopimelate
K05822
K00841
N-Acetyl-LL-2,6-diaminopimelate
N-Succinyl-LL-2,6-diaminopimelate
K00674
K00821...
K05823
N-Succinyl-L-2-amino-6-oxopimelate
K01439
K10206
LL-2,6-Diaminopimelate
K01778
UDP-N-acetylmuramoyl-L-alanyl-D-glutamyl-meso-2,6-diaminopimeloyl-D-alanyl-D-alanine
L-2-Amino-6-oxopimelate
K03340
K01928...
K01929...
Peptidoglycan biosynthesis
meso-2,6-Diaminopimelate
UDP-N-acetylmuramoyl-L-alanyl-γ-D-glutamyl-meso-2,6- diaminopimelate
K01586...
Citrate cycle
Homocitrate
ACO2
Homo-cis-aconitate
2-Oxoglutarate
LYS21...
K16792...
Acetyl-CoA
K16792...
LYS4, LYS3
Glycolysis
Homo-isocitrate
Pyruvate metabolism
5-Adenyl-2-aminoadipate
α-Aminoadipoyl-S-acyl enzyme
L-Saccharopine
LYS2
LYS2
LYS9, LYS13
LYS1
L-Lysine
Lysine degradation
Tropane, piperidine and pyridine alkaloid biosynthesis
L-2-Aminoadipate 6-semialdehyde
LYS12, LYS10...
K17753
LYS2
K16180
K05831
3-Methyl-ornithine
ARO8
ARO8
K05827
K05828
K05829
K05830
3-Methylglutamyl-5-semialdehyde-N6-lysine
K16181
K16182
Pyrrolysine
2-Oxoadipate
L-2-Amino-adipate
LysW-γ-L-α-aminoadipate
LysW-γ-L-α-aminoadipyl-6-phosphate
LysW-γ-L-α-aminoadipate 6-semialdehyde
LysW-γ-L-lysine
3-Methylornithinyl-N6-lysine
00300 6/23/17
(c) Kanehisa Laboratories

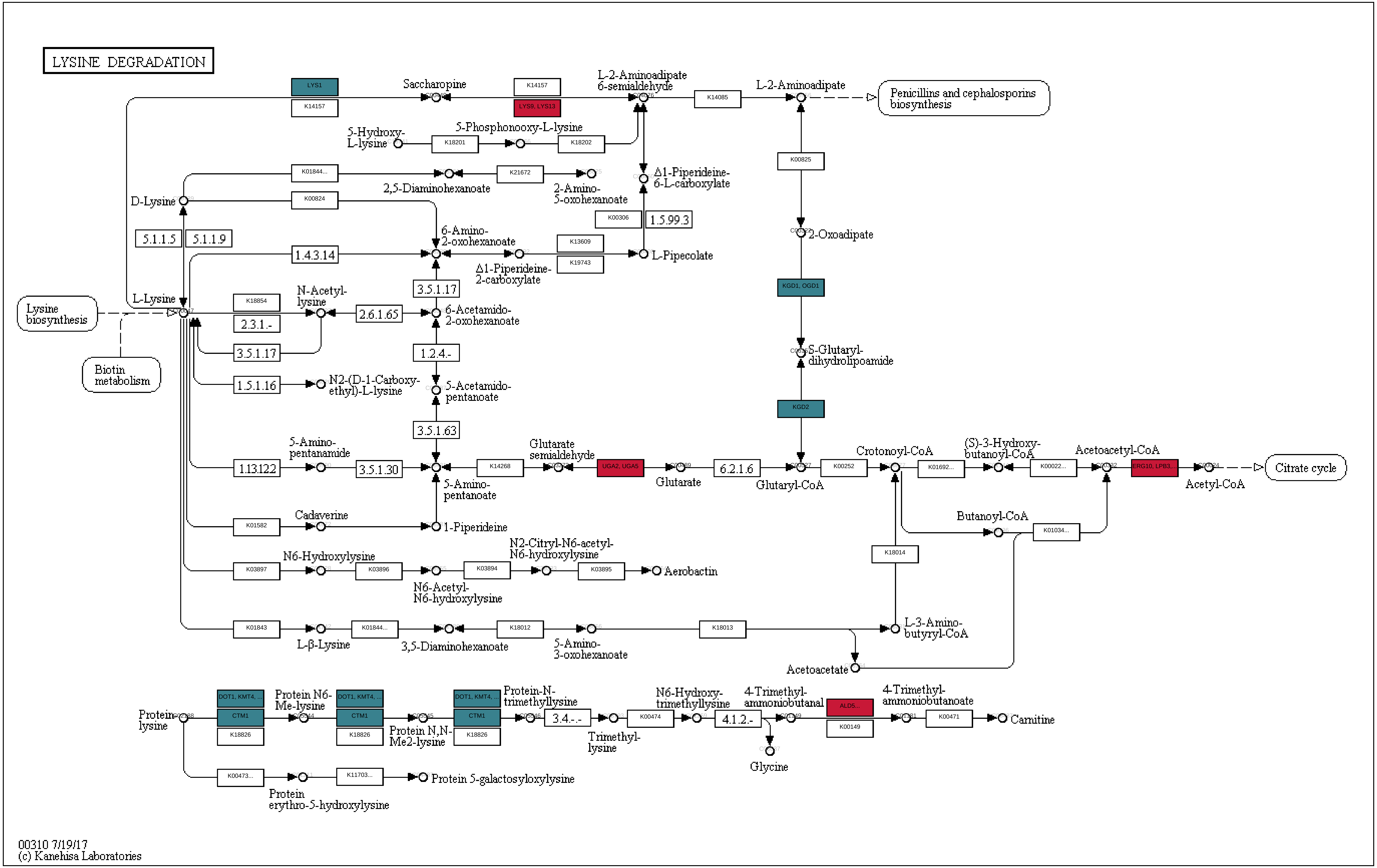
LYSINE DEGRADATION
LYS1
K14157
Saccharopine
K14157
LYS9, LYS13
L-2-Aminoadipate 6-semialdehyde
K14085
L-2-Aminoadipate
Penicillins and cephalosporins biosynthesis
5-Hydroxy-L-lysine
K18201
5-Phosphonooxy-L-lysine
K18202
K01844...
K21672
2,5-Diaminohexanoate
2-Amino-5-oxohexanoate
Δ1-Piperideine-6-L-carboxylate
K00825
D-Lysine
K00824
K00306
1.5.99.3
5.1.1.5
5.1.1.9
1.4.3.14
6-Amino-2-oxohexanoate
K13609
K19743
L-Pipecolate
2-Oxoadipate
Δ1-Piperideine-2-carboxylate
3.5.1.17
KGD1, OGD1
Lysine biosynthesis
L-Lysine
K18854
N-Acetyl-lysine
2.6.1.65
6-Acetamido-2-oxohexanoate
2.3.1.-
3.5.1.17
1.2.4.-
S-Glutaryl-dihydrolipoamide
Biotin metabolism
1.5.1.16
N2-(D-1-Carboxy-ethyl)-L-lysine
5-Acetamido-pentanoate
KGD2
3.5.1.63
5-Amino-pentanamide
Glutarate semialdehyde
1.13.12.2
3.5.1.30
K14268
UGA2, UGA5
6.2.1.6
K00252
Crotonoyl-CoA
K01692...
(S)-3-Hydroxy-butanoyl-CoA
K00022...
Acetoacetyl-CoA
ERG10, LPB3...
Citrate cycle
Glutarate
Glutaryl-CoA
Acetyl-CoA
5-Amino-pentanoate
Cadaverine
K01582
1-Piperideine
Butanoyl-CoA
K01034...
K18014
N6-Hydroxylysine
N2-Citryl-N6-acetyl-N6-hydroxylysine
K03897
K03896
K03894
K03895
Aerobactin
N6-Acetyl-N6-hydroxylysine
K01843
K01844...
K18012
K18013
L-3-Amino-butyryl-CoA
L-β-Lysine
3,5-Diaminohexanoate
5-Amino-3-oxohexanoate
Acetoacetate
Protein-lysine
DOT1, KMT4, ...
CTM1
K18826
Protein N6-Me-lysine
DOT1, KMT4, ...
CTM1
K18826
Protein N,N-Me2-lysine
DOT1, KMT4, ...
CTM1
K18826
Protein-N-trimethyllysine
3.4.-.-
Trimethyl-lysine
K00474
N6-Hydroxy-trimethyllysine
4.1.2.-
Glycine
4-Trimethyl-ammoniobutanal
ALD5...
K00149
4-Trimethyl-ammoniobutanoate
K00471
Carnitine
K00473...
K11703...
Protein 5-galactosyloxylysine
Protein erythro-5-hydroxylysine
00310 7/19/17
(c) Kanehisa Laboratories

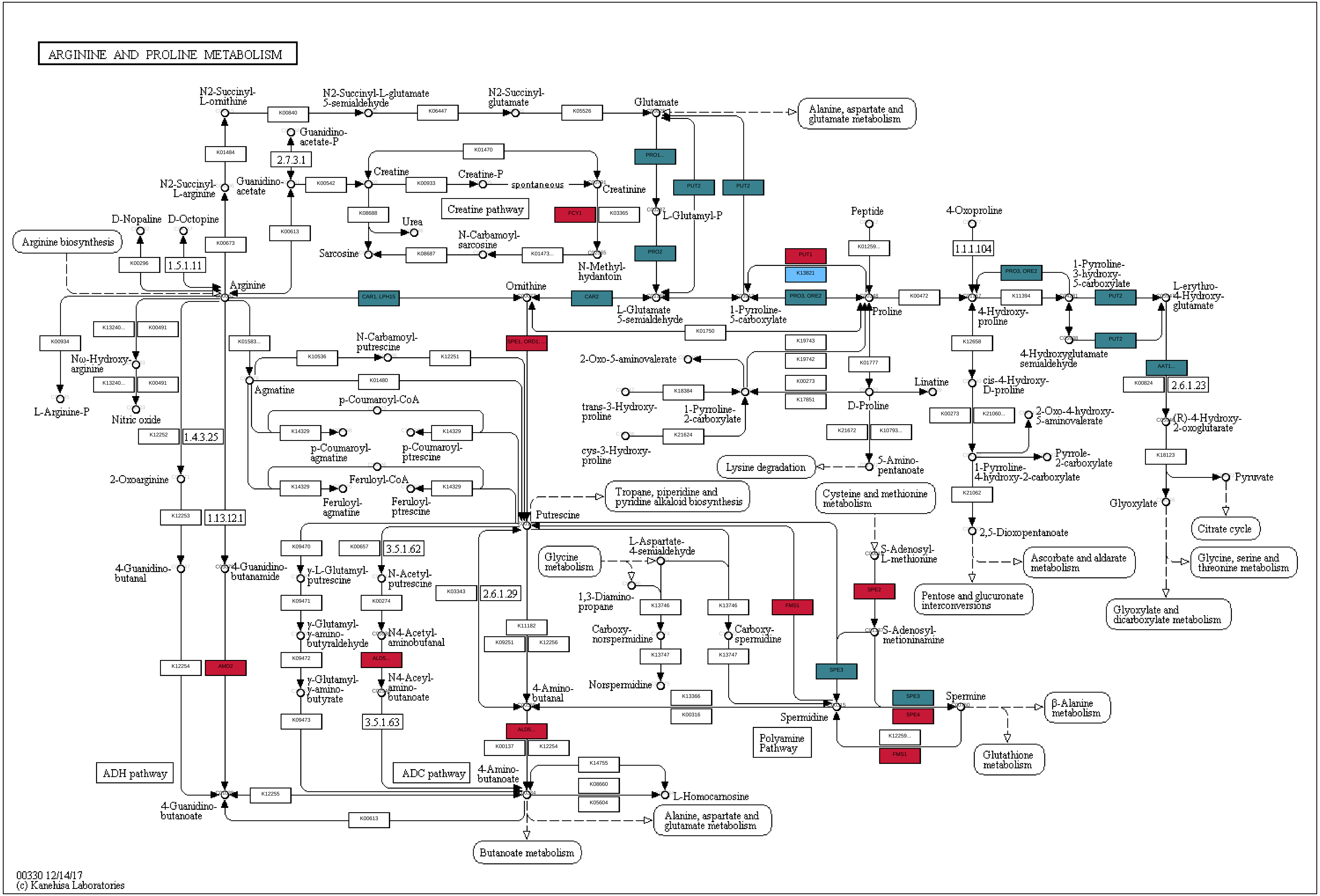
ARGININE AND PROLINE METABOLISM
N2-Succinyl-L-ornithine
N2-Succinyl-L-glutamate 5-semialdehyde
N2-Succinyl-glutamate
Glutamate
Alanine, aspartate and glutamate metabolism
K00840
K06447
K05526
Guanidino-acetate-P
K01484
2.7.3.1
K01470
Creatine
Creatine-P
spontaneous
Creatinine
N2-Succinyl-L-arginine
Guanidino-acetate
K00542
K00933
Creatine pathway
FCY1
K03365
PRO1...
PUT2
L-Glutamyl-P
K08688
Urea
N-Carbamoyl-sarcosine
K00613
K00673
D-Nopaline
D-Octopine
Arginine biosynthesis
Sarcosine
K08687
K01473...
N-Methyl-hydantoin
PRO2
PUT1
K13821
Peptide
4-Oxoproline
K01259...
1.1.1.104
PRO3, ORE2
1-Pyrroline-3-hydroxy-5-carboxylate
L-erythro-4-Hydroxy-glutamate
K00296
1.5.1.11
Arginine
CAR1, LPH15
Ornithine
CAR2
L-Glutamate 5-semialdehyde
1-Pyrroline-5-carboxylate
Proline
K00472
K11394
4-Hydroxy-proline
K01750
K13240...
K00491
K00934
K01583...
SPE1, ORD1...
N-Carbamoyl-putrescine
K10536
K12251
2-Oxo-5-aminovalerate
K19743
K19742
K00273
K17851
K01777
K12658
4-Hydroxyglutamate semialdehyde
AAT1...
Nω-Hydroxy-arginine
L-Arginine-P
Nitric oxide
Agmatine
K01480
p-Coumaroyl-CoA
K18384
trans-3-Hydroxy-proline
1-Pyrroline-2-carboxylate
D-Proline
Linatine
cis-4-Hydroxy-D-proline
K00824
2.6.1.23
K00273
K21060...
2-Oxo-4-hydroxy-5-aminovalerate
(R)-4-Hydroxy-2-oxoglutarate
K12252
1.4.3.25
K14329
p-Coumaroyl-agmatine
p-Coumaroyl-ptrescine
K21624
cys-3-Hydroxy-proline
K21672
K10793...
Pyrrole-2-carboxylate
1-Pyrroline-4-hydroxy-2-carboxylate
K18123
2-Oxoarginine
Feruloyl-CoA
Feruloyl-agmatine
Feruloyl-ptrescine
Lysine degradation
5-Amino-pentanoate
Pyruvate
K21062
Tropane, piperidine and pyridine alkaloid biosynthesis
Cysteine and methionine metabolism
Glyoxylate
Citrate cycle
K12253
1.13.12.1
Putrescine
K09470
K00657
3.5.1.62
Glycine metabolism
L-Aspartate-4-semialdehyde
S-Adenosyl-L-methionine
2,5-Dioxopentanoate
Ascorbate and aldarate metabolism
Glycine, serine and threonine metabolism
4-Guanidino-butanal
4-Guanidino-butanamide
γ-L-Glutamyl-putrescine
N-Acetyl-putrescine
K03343
2.6.1.29
1,3-Diamino-propane
K13746
FMS1
SPE2
Pentose and glucuronate interconversions
Glyoxylate and dicarboxylate metabolism
K09471
K00274
K11182
γ-Glutamyl-γ-amino-butyraldehyde
N4-Acetyl-aminobutanal
K09251
K12256
Carboxy-norspermidine
Carboxy-spermidine
S-Adenosyl-methioninamine
K12254
AMD2
K09472
ALD5...
K13747
SPE3
γ-Glutamyl-γ-amino-butyrate
N4-Aceyl-amino-butanoate
4-Amino-butanal
Norspermidine
K13366
K00316
SPE4
Spermine
β-Alanine metabolism
K09473
3.5.1.63
Spermidine
Polyamine Pathway
K12259...
K00137
ADH pathway
ADC pathway
4-Amino-butanoate
K14755
K08660
K05604
Glutathione metabolism
4-Guanidino-butanoate
K12255
L-Homocarnosine
Alanine, aspartate and glutamate metabolism
Butanoate metabolism
00330 12/14/17
(c) Kanehisa Laboratories

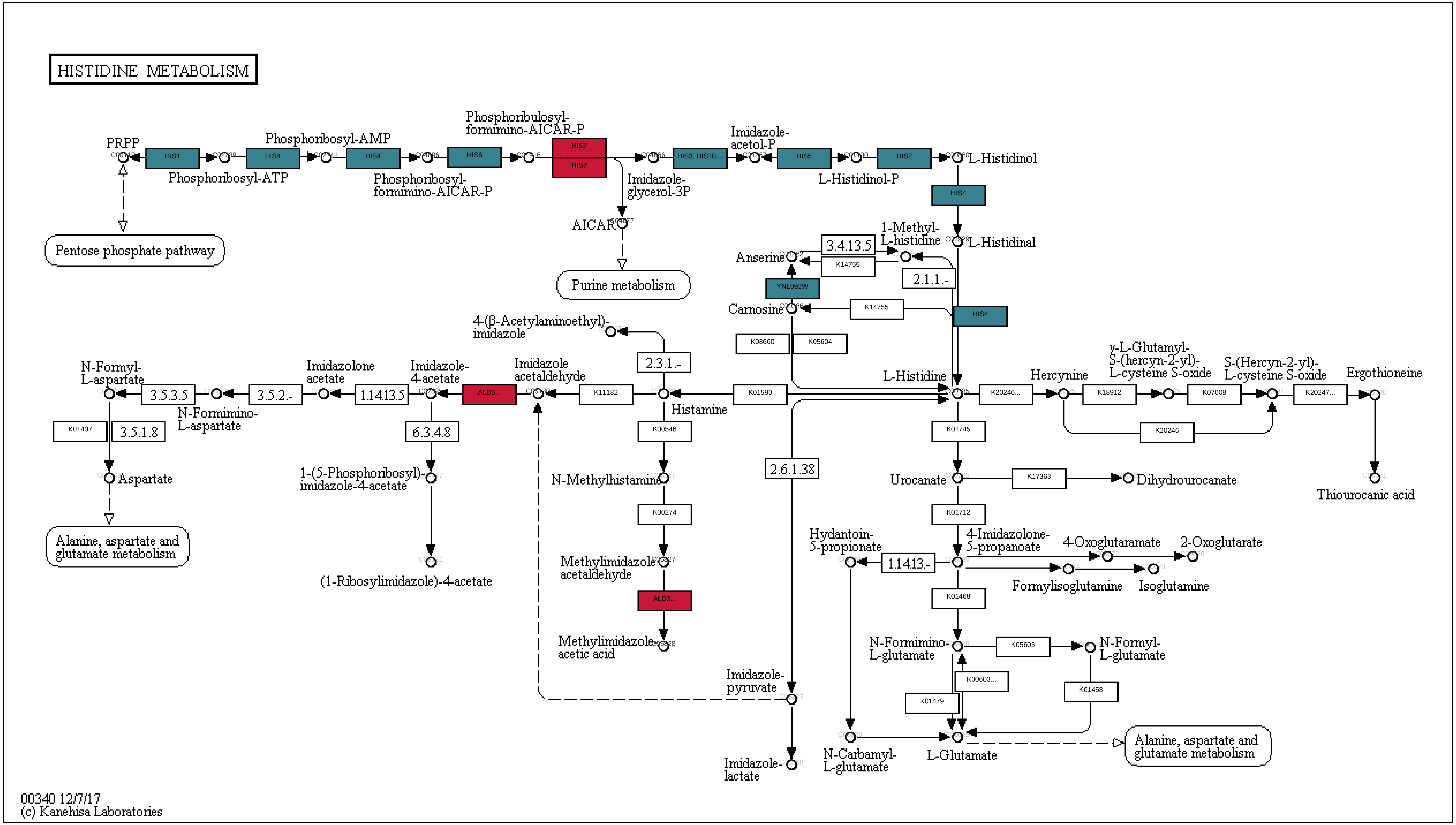
HISTIDINE METABOLISM
PRPP
HIS1
Phosphoribosyl-ATP
HIS4
Phosphoribosyl-AMP
HIS4
Phosphoribosyl-formimino-AICAR-P
HIS6
Phosphoribulosyl-formimino-AICAR-P
HIS7
HIS7
Imidazole-glycerol-3P
AICAR
HIS3, HIS10,...
Imidazole-acetol-P
HIS5
L-Histidinol-P
HIS2
L-Histidinol
HIS4
L-Histidinal
HIS4
Pentose phosphate pathway
Purine metabolism
1-Methyl-L-histidine
3.4.13.5
K14755
Anserine
2.1.1.-
YNL092W
Carnosine
K14755
K08660
K05604
4-(β-Acetylaminoethyl)-imidazole
2.3.1.-
N-Formyl-L-aspartate
3.5.3.5
N-Formimino-L-aspartate
3.5.2.-
Imidazolone acetate
1.14.13.5
Imidazole-4-acetate
ALD5...
Imidazole acetaldehyde
K11182
Histamine
K01590
L-Histidine
K20246...
Hercynine
K18912
γ-L-Glutamyl-S-(hercyn-2-yl)-L-cysteine S-oxide
K07008
S-(Hercyn-2-yl)-L-cysteine S-oxide
K20247...
Ergothioneine
K20246
K01437
3.5.1.8
Aspartate
Alanine, aspartate and glutamate metabolism
6.3.4.8
1-(5-Phosphoribosyl)-imidazole-4-acetate
(1-Ribosylimidazole)-4-acetate
K00546
N-Methylhistamine
K00274
Methylimidazole acetaldehyde
ALD3...
Methylimidazole-acetic acid
2.6.1.38
Imidazole-pyruvate
Imidazole-lactate
K01745
Urocanate
K17363
Dihydrourocanate
Thiourocanic acid
K01712
Hydantoin-5-propionate
1.14.13.-
4-Imidazolone-5-propanoate
4-Oxoglutaramate
2-Oxoglutarate
Formylisoglutamine
Isoglutamine
K01468
N-Formimino-L-glutamate
K05603
N-Formyl-L-glutamate
K00603...
K01479
K01458
N-Carbamyl-L-glutamate
L-Glutamate
Alanine, aspartate and glutamate metabolism
00340 12/7/17
(c) Kanehisa Laboratories

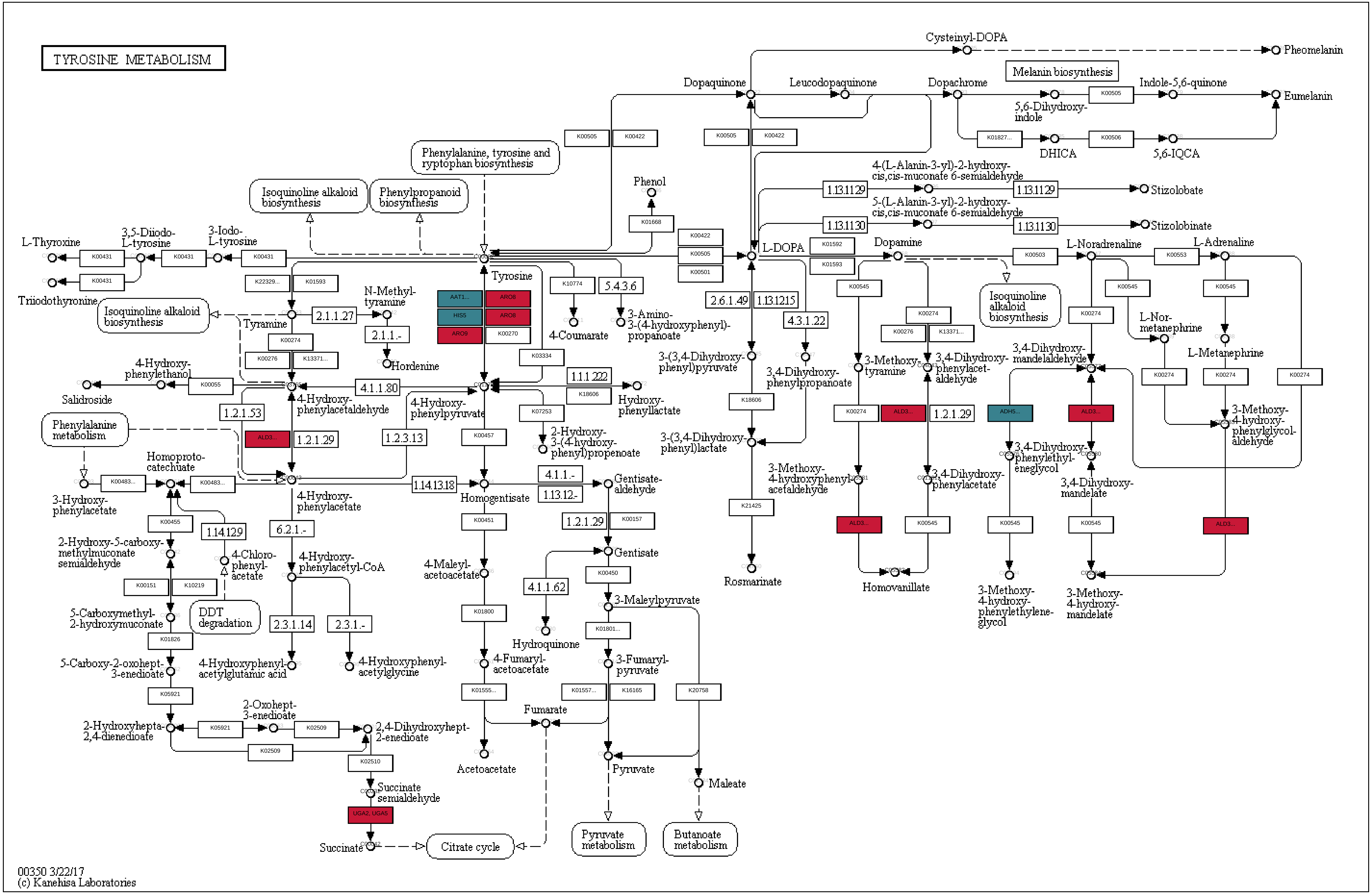
TYROSINE METABOLISM
Cysteinyl-DOPA
Pheomelanin
Melanin biosynthesis
Dopaquinone
Leucodopaquinone
Dopachrome
Indole-5,6-quinone
Eumelanin
5,6-Dihydroxy-indole
K00505
K00422
K00505
K00422
K01827...
DHICA
K00506
5,6-IQCA
Phenylalanine, tyrosine and tryptophan biosynthesis
Isoquinoline alkaloid biosynthesis
Phenylpropanoid biosynthesis
Phenol
K01668
4-(L-Alanin-3-yl)-2-hydroxy-cis,cis-muconate 6-semialdehyde
1.13.11.29
1.13.11.29
Stizolobate
5-(L-Alanin-3-yl)-2-hydroxy-cis,cis-muconate 6-semialdehyde
1.13.11.30
1.13.11.30
Stizolobinate
L-Thyroxine
3,5-Diiodo-L-tyrosine
3-Iodo-L-tyrosine
K00431
K00431
K00431
K00431
Triiodothyronine
K22329...
K01593
K00422
K00505
K00501
L-DOPA
K01592
K01593
Dopamine
K00503
L-Noradrenaline
K00553
L-Adrenaline
Tyrosine
AAT1...
ARO8
HIS5
ARO8
ARO9
K00270
K10774
5.4.3.6
2.6.1.49
1.13.12.15
K00545
K00545
K00545
N-Methyl-tyramine
2.1.1.27
2.1.1.-
Hordenine
Isoquinoline alkaloid biosynthesis
Tyramine
K00274
K00276
K13371...
4-Coumarate
3-Amino-3-(4-hydroxyphenyl)-propanoate
4.3.1.22
K00274
K00276
K13371...
Isoquinoline alkaloid biosynthesis
K00274
L-Nor-metanephrine
L-Metanephrine
4-Hydroxy-phenylethanol
K00055
Salidroside
K03334
4.1.1.80
4-Hydroxy-phenylacetaldehyde
4-Hydroxy-phenylpyruvate
1.1.1.222
K18606
Hydroxy-phenyllactate
3-(3,4-Dihydroxy-phenyl)pyruvate
3,4-Dihydroxy-phenylpropanoate
3-Methoxy-tyramine
3,4-Dihydroxy-phenylacet-aldehyde
3,4-Dihydroxy-mandelaldehyde
K00274
K00274
K00274
1.2.1.53
K07253
K18606
K00274
ALD3...
1.2.1.29
ADH5...
ALD3...
3-Methoxy-4-hydroxy-phenylglycol-aldehyde
Phenylalanine metabolism
ALD3...
1.2.1.29
1.2.3.13
K00457
2-Hydroxy-3-(4-hydroxy-phenyl)propenoate
3-(3,4-Dihydroxy-phenyl)lactate
3,4-Dihydroxy-phenylethyl-eneglycol
Homoproto-catechuate
K00483...
K00483...
1.14.13.18
4.1.1.-
Gentisate-aldehyde
3-Methoxy-4-hydroxyphenyl-acetaldehyde
3,4-Dihydroxy-phenylacetate
3,4-Dihydroxy-mandelate
3-Hydroxy-phenylacetate
4-Hydroxy-phenylacetate
Homogentisate
1.13.12.-
K00455
1.14.12.9
6.2.1.-
K00451
1.2.1.29
K00157
K21425
ALD3...
K00545
K00545
K00545
ALD3...
2-Hydroxy-5-carboxy-methylmuconate semialdehyde
4-Chloro-phenyl-acetate
4-Hydroxy-phenylacetyl-CoA
4-Maleyl-acetoacetate
Gentisate
Rosmarinate
Homovanillate
K00151
K10219
4.1.1.62
K00450
3-Methoxy-4-hydroxy-phenylethylene-glycol
3-Methoxy-4-hydroxy-mandelate
5-Carboxymethyl-2-hydroxymuconate
DDT degradation
2.3.1.14
2.3.1.-
K01800
3-Maleylpyruvate
K01826
K01801...
Hydroquinone
5-Carboxy-2-oxohept-3-enedioate
4-Hydroxyphenyl-acetylglutamic acid
4-Hydroxyphenyl-acetylglycine
4-Fumaryl-acetoacetate
3-Fumaryl-pyruvate
K05921
K01555...
K01557...
K16165
K20758
Fumarate
2-Oxohept-3-enedioate
2-Hydroxyhepta-2,4-dienedioate
K05921
K02509
2,4-Dihydroxyhept-2-enedioate
K02509
K02510
Acetoacetate
Pyruvate
Maleate
Succinate semialdehyde
UGA2, UGA5
Pyruvate metabolism
Butanoate metabolism
Succinate
Citrate cycle
00350 3/22/17
(c) Kanehisa Laboratories

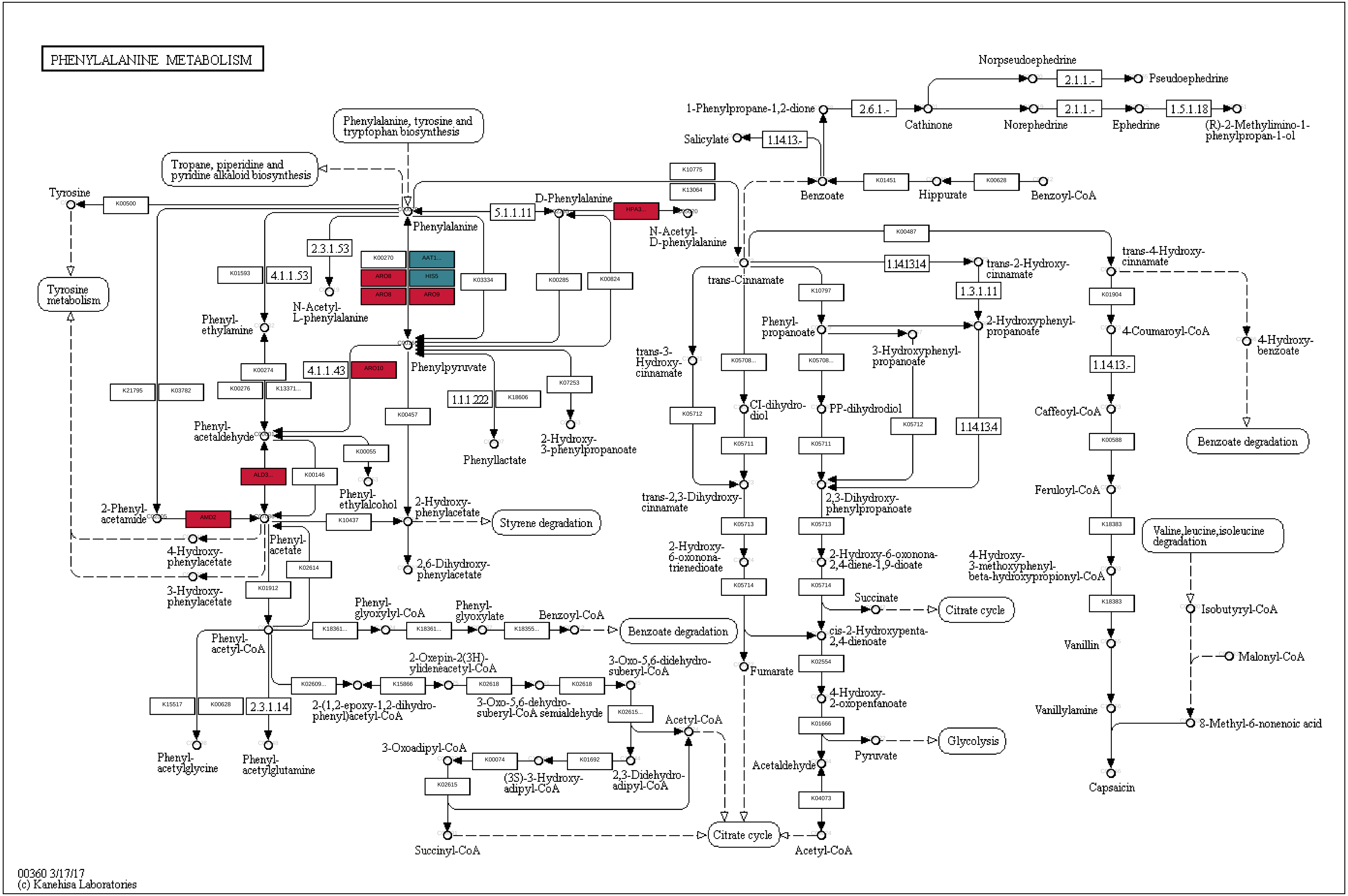
PHENYLALANINE METABOLISM
Phenylalanine, tyrosine and tryptophan biosynthesis
Tropane, piperidine and pyridine alkaloid biosynthesis
Tyrosine
K00500
Tyrosine metabolism
Phenylalanine
5.1.1.11
D-Phenylalanine
HPA3...
N-Acetyl-D-phenylalanine
2.3.1.53
N-Acetyl-L-phenylalanine
K01593
4.1.1.53
K00270
AAT1...
ARO8
HIS5
ARO8
ARO9
K03334
K00285
K00824
Phenyl-ethylamine
K00274
K00276
K13371...
K21795
K03782
4.1.1.43
ARO10
Phenylpyruvate
1.1.1.222
K18606
K07253
Phenyllactate
2-Hydroxy-3-phenylpropanoate
K00457
Phenyl-acetaldehyde
K00055
Phenyl-ethylalcohol
ALD3...
K00146
2-Phenyl-acetamide
AMD2
K10437
2-Hydroxy-phenylacetate
Styrene degradation
4-Hydroxy-phenylacetate
Phenyl-acetate
2,6-Dihydroxy-phenylacetate
3-Hydroxy-phenylacetate
K02614
K01912
Phenyl-acetyl-CoA
K18361...
Phenyl-glyoxylyl-CoA
K18361...
Phenyl-glyoxylate
K18355...
Benzoyl-CoA
Benzoate degradation
2-Oxepin-2(3H)-ylideneacetyl-CoA
K02609...
K15866
K02618
K02618
3-Oxo-5,6-didehydro-suberyl-CoA
2-(1,2-epoxy-1,2-dihydro-phenyl)acetyl-CoA
3-Oxo-5,6-dehydro-suberyl-CoA semialdehyde
K02615...
Acetyl-CoA
K15517
K00628
2.3.1.14
Phenyl-acetylglycine
Phenyl-acetylglutamine
3-Oxoadipyl-CoA
K00074
K01692
(3S)-3-Hydroxy-adipyl-CoA
2,3-Didehydro-adipyl-CoA
K02615
Succinyl-CoA
Citrate cycle
Norpseudoephedrine
2.1.1.-
Pseudoephedrine
1-Phenylpropane-1,2-dione
2.6.1.-
Cathinone
2.1.1.-
Norephedrine
Ephedrine
1.5.1.18
(R)-2-Methylimino-1-phenylpropan-1-ol
Salicylate
1.14.13.-
K10775
K13064
Benzoate
K01451
Hippurate
K00628
Benzoyl-CoA
K00487
trans-Cinnamate
1.14.13.14
trans-2-Hydroxy-cinnamate
trans-4-Hydroxy-cinnamate
K10797
1.3.1.11
K01904
Phenyl-propanoate
2-Hydroxyphenyl-propanoate
3-Hydroxyphenyl-propanoate
4-Coumaroyl-CoA
4-Hydroxy-benzoate
trans-3-Hydroxy-cinnamate
K05708...
K05708...
1.14.13.-
K05712
CI-dihydro-diol
PP-dihydrodiol
K05712
1.14.13.4
Caffeoyl-CoA
Benzoate degradation
K05711
K05711
K00588
trans-2,3-Dihydroxy-cinnamate
2,3-Dihydroxy-phenylpropanoate
Feruloyl-CoA
K05713
K05713
K18383
Valine, leucine, isoleucine degradation
2-Hydroxy-6-oxonona-trienedioate
2-Hydroxy-6-oxonona-2,4-diene-1,9-dioate
4-Hydroxy-3-methoxyphenyl-beta-hydroxypropionyl-CoA
K05714
K05714
Succinate
Citrate cycle
K18383
Isobutyryl-CoA
cis-2-Hydroxypenta-2,4-dienoate
Vanillin
Malonyl-CoA
Fumarate
K02554
4-Hydroxy-2-oxopentanoate
Vanillylamine
8-Methyl-6-nonenoic acid
K01666
Pyruvate
Glycolysis
Acetaldehyde
Capsaicin
K04073
Citrate cycle
Acetyl-CoA
00360 3/17/17
(c) Kanehisa Laboratories

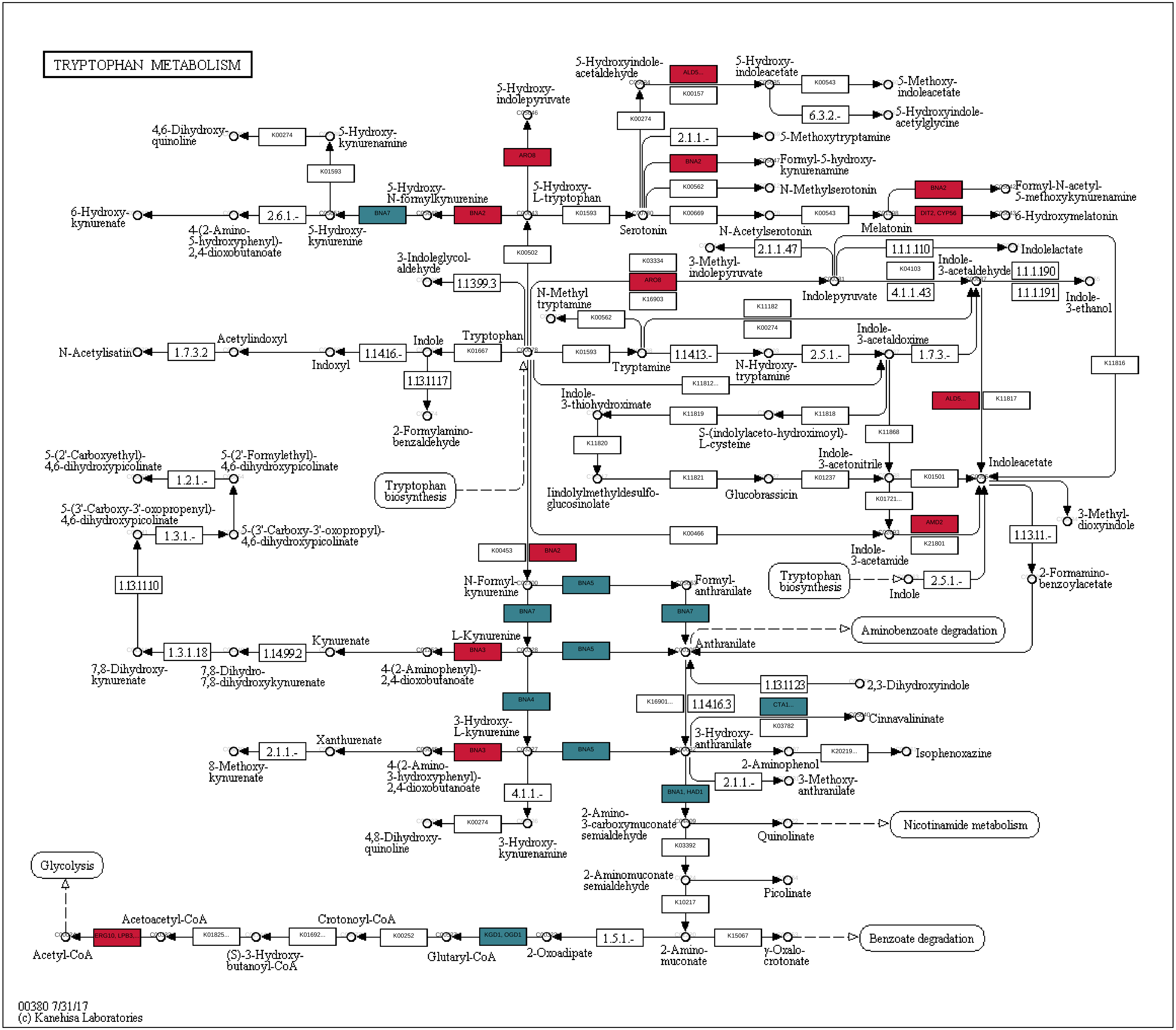
TRYPTOPHAN METABOLISM
5-Hydroxyindole-acetaldehyde
ALD5...
K00157
5-Hydroxy-indoleacetate
K00543
5-Methoxy-indoleacetate
6.3.2.-
5-Hydroxyindole-acetylglycine
5-Hydroxy-indolepyruvate
K00274
2.1.1.-
5-Methoxytryptamine
4,6-Dihydroxy-quinoline
K00274
5-Hydroxy-kynurenamine
ARO8
BNA2
Formyl-5-hydroxy-kynurenamine
K01593
K00562
N-Methylserotonin
BNA2
Formyl-N-acetyl-5-methoxykynurenamine
5-Hydroxy-N-formylkynurenine
5-Hydroxy-L-tryptophan
6-Hydroxy-kynurenine
2.6.1.-
BNA7
BNA2
K01593
K00669
K00543
DIT2, CYP56
6-Hydroxymelatonin
4-(2-Amino-5-hydroxyphenyl)-2,4-dioxobutanoate
5-Hydroxy-kynurenine
Serotonin
N-Acetylserotonin
Melatonin
2.1.1.47
1.1.1.110
Indolelactate
K00502
K03334
3-Methyl-indolepyruvate
K04103
Indole-3-acetaldehyde
1.1.1.190
3-Indoleglycol-aldehyde
1.13.99.3
ARO8
Indolepyruvate
4.1.1.43
1.1.1.191
Indole-3-ethanol
N-Methyl tryptamine
K16903
K11182
K00562
K00274
N-Acetylisatin
1.7.3.2
Acetylindoxyl
1.14.16.-
Indole
K01667
Tryptophan
K01593
Tryptamine
1.14.13.-
N-Hydroxy-tryptamine
2.5.1.-
Indole-3-acetaldoxime
1.7.3.-
K11816
Indoxyl
1.13.11.17
K11812...
Indole-3-thiohydroximate
K11819
K11818
ALD5...
K11817
2-Formylamino-benzaldehyde
S-(indolylaceto-hydroximoyl)-L-cysteine
K11868
5-(2'-Carboxyethyl)-4,6-dihydroxypicolinate
5-(2'-Formylethyl)-4,6-dihydroxypicolinate
1.2.1.-
K11820
Indole-3-acetonitrile
Indoleacetate
Tryptophan biosynthesis
K11821
K01237
K01501
Iindolylmethyldesulfo-glucosinolate
Glucobrassicin
K01721...
3-Methyl-dioxyindole
5-(3'-Carboxy-3'-oxopropenyl)-4,6-dihydroxypicolinate
1.3.1.-
5-(3'-Carboxy-3'-oxopropyl)-4,6-dihydroxypicolinate
K00466
AMD2
K21801
1.13.11.-
K00453
BNA2
Indole-3-acetamide
1.13.11.10
Tryptophan biosynthesis
Indole
2.5.1.-
2-Formamino-benzoylacetate
N-Formyl-kynurenine
BNA5
Formyl-anthranilate
BNA7
BNA7
Aminobenzoate degradation
7,8-Dihydroxy-kynurenate
1.3.1.18
7,8-Dihydro-7,8-dihydroxykynurenate
1.14.99.2
Kynurenate
BNA3
L-Kynurenine
BNA5
Anthranilate
4-(2-Aminophenyl)-2,4-dioxobutanoate
1.13.11.23
2,3-Dihydroxyindole
BNA4
K16901...
1.14.16.3
CTA1...
K03782
Cinnavalininate
3-Hydroxy-L-kynurenine
3-Hydroxy-anthranilate
8-Methoxy-kynurenate
2.1.1.-
Xanthurenate
BNA3
BNA5
K20219...
Isophenoxazine
4-(2-Amino-3-hydroxyphenyl)-2,4-dioxobutanoate
2-Aminophenol
2.1.1.-
3-Methoxy-anthranilate
4.1.1.-
BNA1, HAD1
2-Amino-3-carboxymuconate semialdehyde
Quinolinate
Nicotinamide metabolism
4,8-Dihydroxy-quinoline
K00274
3-Hydroxy-kynurenamine
K03392
Glycolysis
2-Aminomuconate semialdehyde
Picolinate
K10217
Acetoacetyl-CoA
Crotonoyl-CoA
ERG10, LPB3...
K01825...
K01692...
K00252
KGD1, OGD1
1.5.1.-
K15067
Benzoate degradation
Acetyl-CoA
(S)-3-Hydroxy-butanoyl-CoA
Glutaryl-CoA
2-Oxoadipate
2-Amino-muconate
γ-Oxalo-crotonate
00380 7/31/17
(c) Kanehisa Laboratories

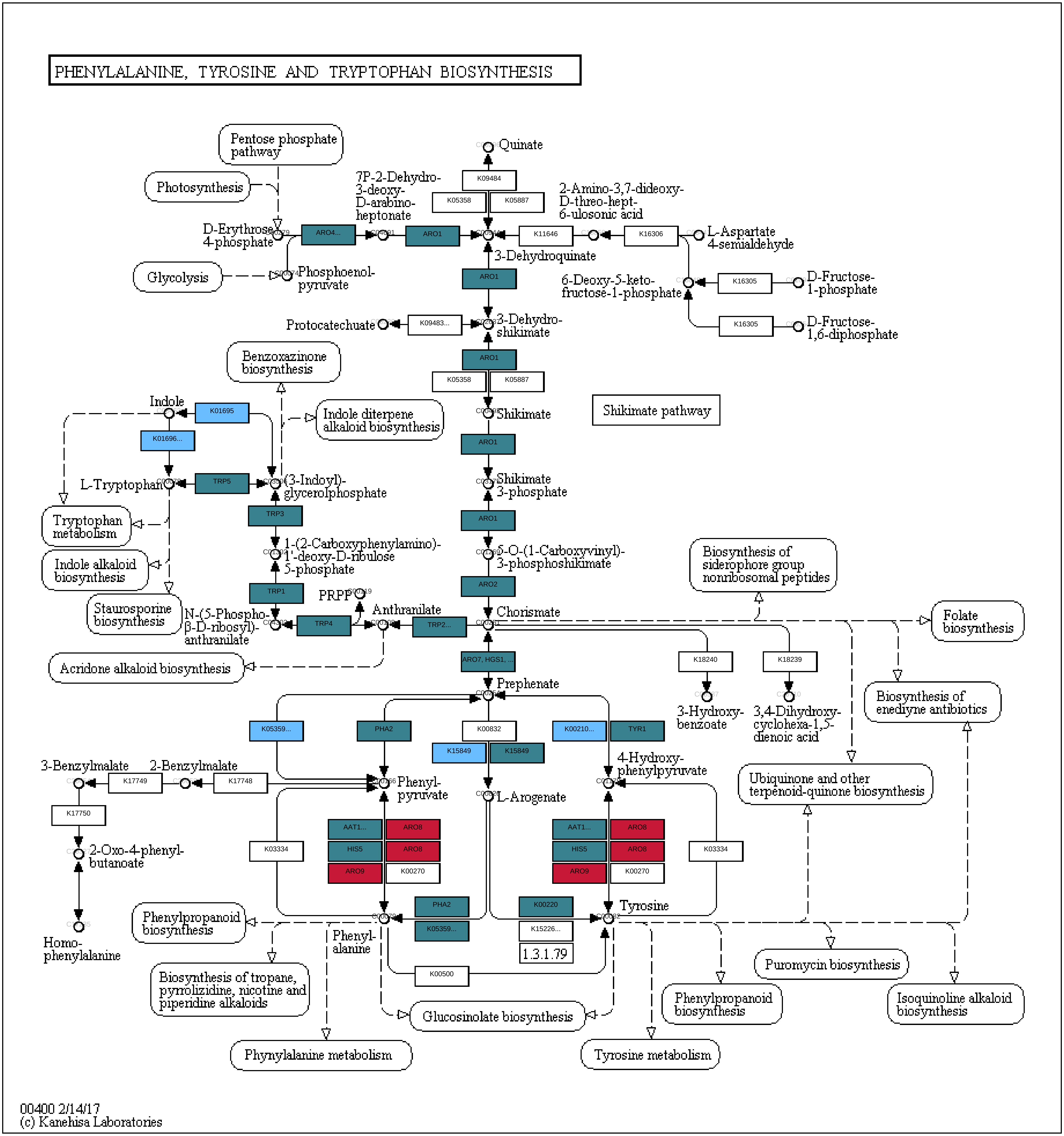
PHENYLALANINE, TYROSINE AND TRYPTOPHAN BIOSYNTHESIS
Pentose phosphate pathway
Photosynthesis
Glycolysis
D-Erythrose 4-phosphate
Phosphoenol-pyruvate
7P-2-Dehydro-3-deoxy-D-arabino-heptonate
Quinate
K09484
K05358
K05887
ARO4...
ARO1
2-Amino-3,7-dideoxy-D-threo-hept-6-ulosonic acid
K11646
K16306
L-Aspartate 4-semialdehyde
3-Dehydroquinate
6-Deoxy-5-keto-fructose-1-phosphate
K16305
D-Fructose-1-phosphate
D-Fructose-1,6-diphosphate
Protocatechuate
K09483...
3-Dehydro-shikimate
Benzoxazinone biosynthesis
Indole diterpene alkaloid biosynthesis
Indole
K01695
K01696...
Shikimate
Shikimate pathway
L-Tryptophan
TRP5
(3-Indoyl)-glycerolphosphate
TRP3
Tryptophan metabolism
Indole alkaloid biosynthesis
Staurosporine biosynthesis
1-(2-Carboxyphenylamino)-1'-deoxy-D-ribulose 5-phosphate
TRP1
PRPP
N-(5-Phospho-β-D-ribosyl)-anthranilate
TRP4
Anthranilate
TRP2...
Shikimate 3-phosphate
5-O-(1-Carboxyvinyl)-3-phosphoshikimate
ARO2
Chorismate
Biosynthesis of siderophore group nonribosomal peptides
Folate biosynthesis
Acridone alkaloid biosynthesis
ARO7, HGS1, ...
K18240
K18239
Prephenate
3-Hydroxy-benzoate
3,4-Dihydroxy-cyclohexa-1,5-dienoic acid
Biosynthesis of enediyne antibiotics
K05359...
PHA2
K00832
K15849
K00210...
TYR1
3-Benzylmalate
2-Benzylmalate
K17749
K17748
K17750
Phenyl-pyruvate
L-Arogenate
4-Hydroxy-phenylpyruvate
Ubiquinone and other terpenoid-quinone biosynthesis
2-Oxo-4-phenyl-butanoate
K03334
AAT1...
ARO8
HIS5
ARO9
K00270
Homo-phenylalanine
Phenylpropanoid biosynthesis
K00220
K15226...
1.3.1.79
Tyrosine
Phenyl-alanine
K00500
Biosynthesis of tropane, pyrrolizidine, nicotine and piperidine alkaloids
Glucosinolate biosynthesis
Puromycin biosynthesis
Isoquinoline alkaloid biosynthesis
Phynylalanine metabolism
Tyrosine metabolism
00400 2/14/17
(c) Kanehisa Laboratories

# 6. Metabolism of other amino acids

| MAP | PATHWAY |
|---|---|
| **410** | beta-Alanine metabolism |
| **430** | Taurine and hypotaurine metabolism |
| **440** | Phosphonate and phosphinate metabolism |
| **450** | Selenocompound metabolism |
| **460** | Cyanoamino acid metabolism |
| **480** | Glutathione metabolism |

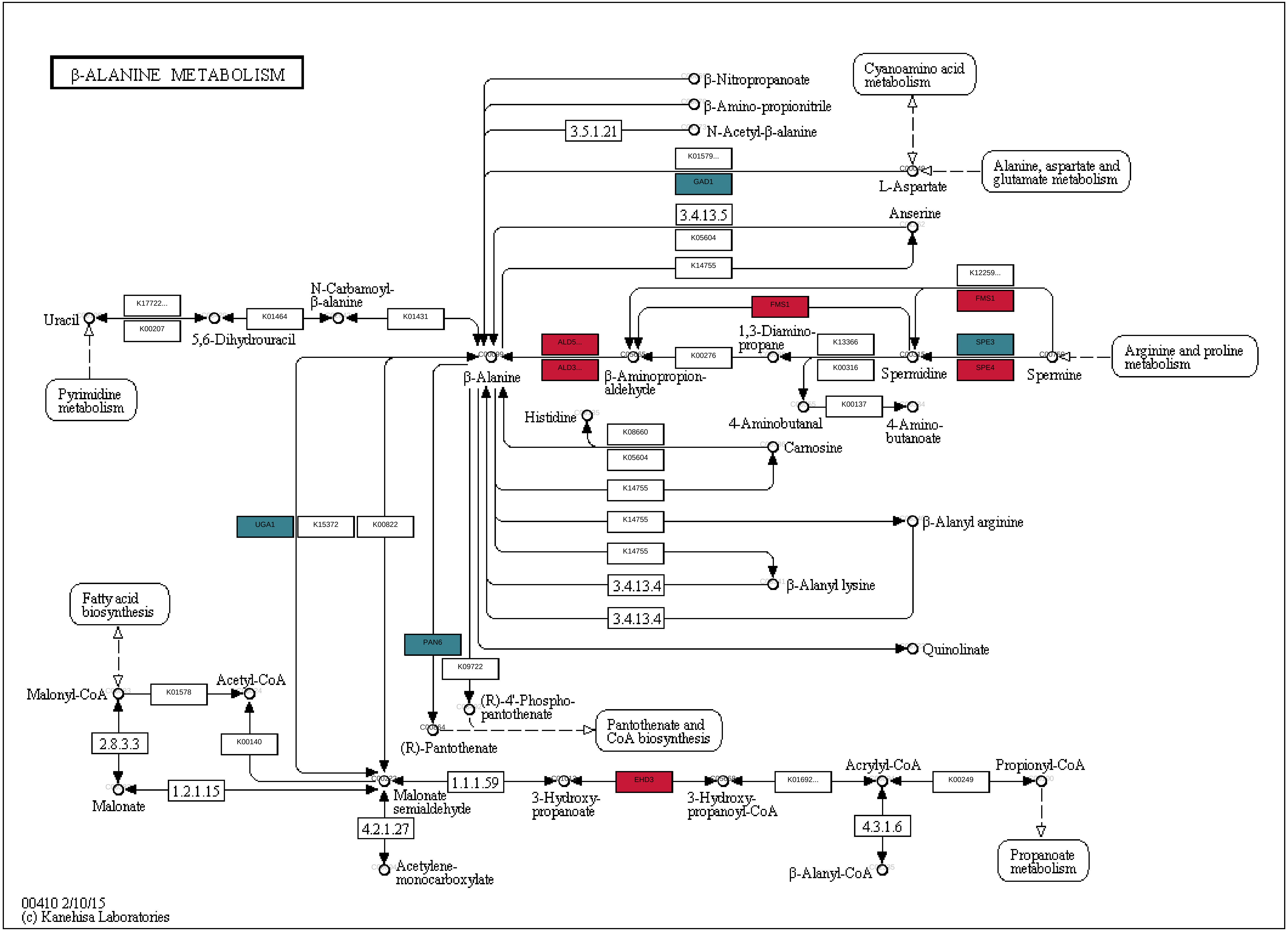
β-ALANINE METABOLISM
β-Nitropropanoate
β-Amino-propionitrile
3.5.1.21
N-Acetyl-β-alanine
Cyanoamino acid metabolism
K01579...
GAD1
L-Aspartate
Alanine, aspartate and glutamate metabolism
3.4.13.5
Anserine
K05604
K14755
K12259...
FMS1
FMS1
Uracil
K17722...
K00207
5,6-Dihydrouracil
K01464
N-Carbamoyl-β-alanine
K01431
1,3-Diamino-propane
K13366
K00316
SPE3
SPE4
ALD5...
ALD3...
β-Alanine
K00276
β-Aminopropion-aldehyde
Spermidine
Spermine
Arginine and proline metabolism
Pyrimidine metabolism
Histidine
K08660
K05604
4-Aminobutanal
K00137
4-Amino-butanoate
Carnosine
K14755
K14755
β-Alanyl arginine
K14755
β-Alanyl lysine
3.4.13.4
3.4.13.4
UGA1
K15372
K00822
Quinolinate
Fatty acid biosynthesis
PAN6
K09722
(R)-4'-Phospho-pantothenate
Pantothenate and CoA biosynthesis
(R)-Pantothenate
Malonyl-CoA
K01578
Acetyl-CoA
2.8.3.3
K00140
Malonate
1.2.1.15
Malonate semialdehyde
1.1.1.59
3-Hydroxy-propanoate
EHD3
3-Hydroxy-propanoyl-CoA
K01692...
Acrylyl-CoA
K00249
Propionyl-CoA
4.2.1.27
Acetylene-monocarboxylate
4.3.1.6
β-Alanyl-CoA
Propanoate metabolism
00410 2/10/15
(c) Kanehisa Laboratories

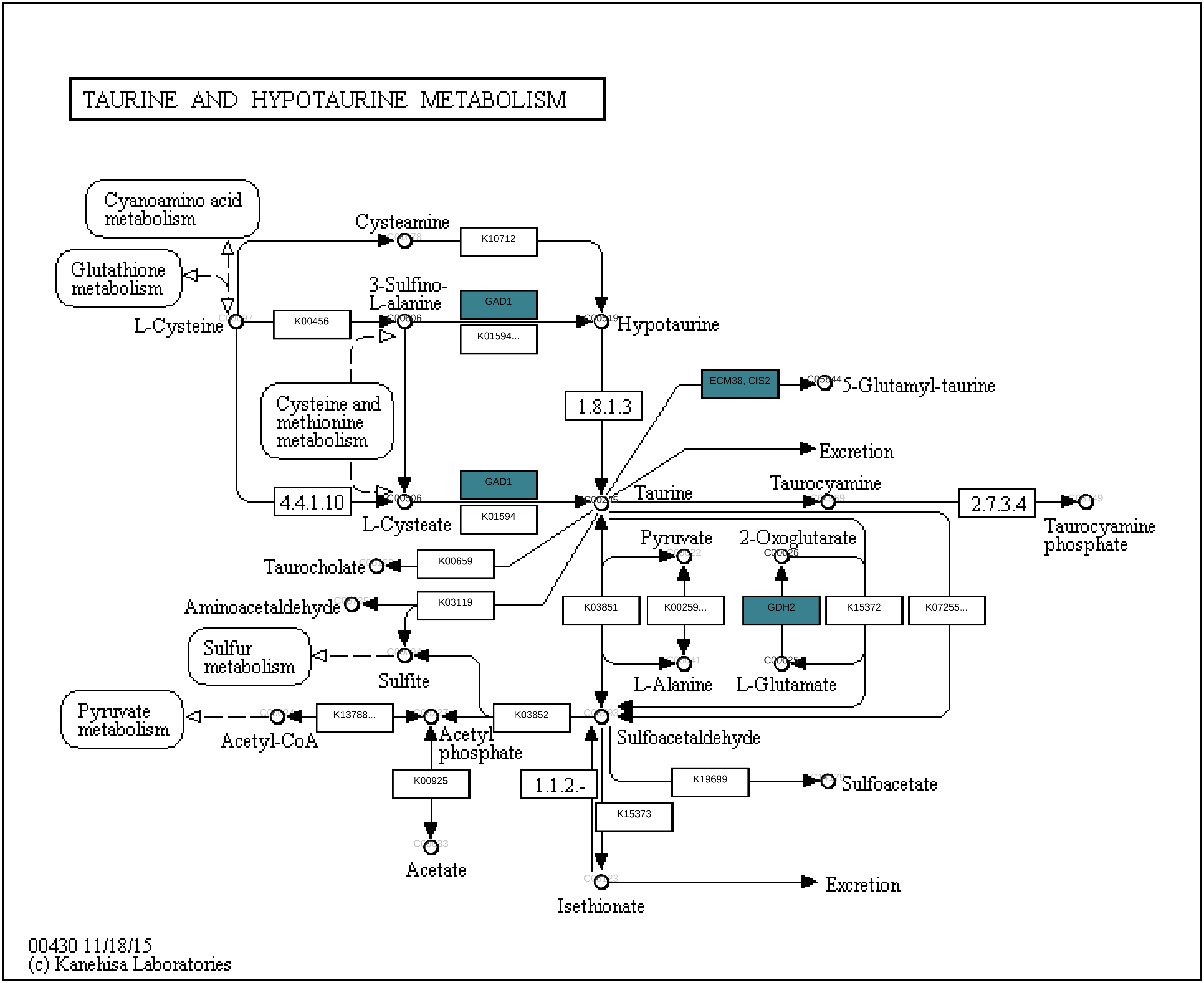

TAURINE AND HYPOTAURINE METABOLISM
Cyanoamino acid metabolism
Glutathione metabolism
Cysteamine
K10712
3-Sulfino-L-alanine
GAD1
K01594...
L-Cysteine
K00456
Hypotaurine
Cysteine and methionine metabolism
1.8.1.3
ECM38, CIS2
5-Glutamyl-taurine
Excretion
4.4.1.10
GAD1
K01594
L-Cysteate
Taurine
Taurocyamine
2.7.3.4
Taurocyamine phosphate
Taurocholate
K00659
Pyruvate
2-Oxoglutarate
Aminoacetaldehyde
K03119
K03851
K00259...
GDH2
K15372
K07255...
Sulfur metabolism
Sulfite
L-Alanine
L-Glutamate
Pyruvate metabolism
K13788...
K03852
Acetyl-CoA
Acetyl phosphate
Sulfoacetaldehyde
K00925
1.1.2.-
K19699
Sulfoacetate
K15373
Acetate
Excretion
Isethionate
00430 11/18/15
(c) Kanehisa Laboratories

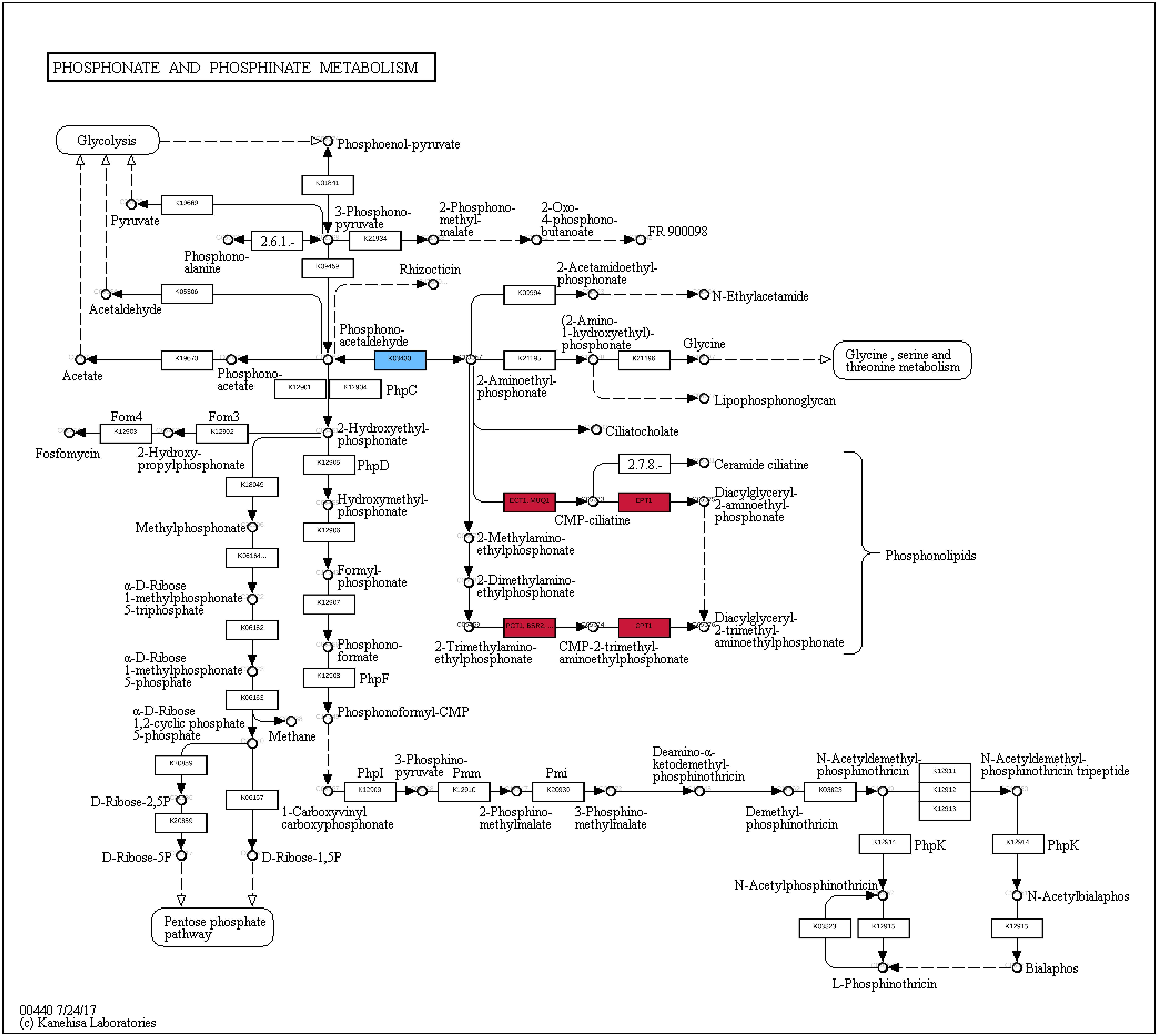
PHOSPHONATE AND PHOSPHINATE METABOLISM
Glycolysis
Phosphoenol-pyruvate
K01841
K19669
Pyruvate
3-Phosphono-pyruvate
2-Phosphono-methyl-malate
2-Oxo-4-phosphono-butanoate
FR 900098
2.6.1.-
K21934
Phosphono-alanine
K09459
Rhizocticin
K05306
Acetaldehyde
2-Acetamidoethyl-phosphonate
K09994
N-Ethylacetamide
Phosphono-acetaldehyde
(2-Amino-1-hydroxyethyl)-phosphonate
Glycine
K19670
K03430
K21195
K21196
Glycine, serine and threonine metabolism
Acetate
Phosphono-acetate
K12901
K12904
PhpC
2-Aminoethyl-phosphonate
Lipophosphonoglycan
Fom4
Fom3
K12903
K12902
2-Hydroxyethyl-phosphonate
Ciliatocholate
Fosfomycin
2-Hydroxy-propylphosphonate
K12905
PhpD
2.7.8.-
Ceramide ciliatine
K18049
Hydroxymethyl-phosphonate
ECT1, MUQ1
EPT1
Diacylglyceryl-2-aminoethyl-phosphonate
CMP-ciliatine
Methylphosphonate
K12906
2-Methylamino-ethylphosphonate
Phosphonolipids
K06164...
Formyl-phosphonate
2-Dimethylamino-ethylphosphonate
α-D-Ribose 1-methylphosphonate 5-triphosphate
K12907
K06162
Phosphono-formate
PCT1, BSR2, ...
CPT1
Diacylglyceryl-2-trimethyl-aminoethylphosphonate
2-Trimethylamino-ethylphosphonate
CMP-2-trimethyl-aminoethylphosphonate
α-D-Ribose 1-methylphosphonate 5-phosphate
K12908
PhpF
K06163
Phosphonoformyl-CMP
α-D-Ribose 1,2-cyclic phosphate 5-phosphate
Methane
Deamino-α-ketodemethyl-phosphinothricin
N-Acetyldemethyl-phosphinothricin
N-Acetyldemethyl-phosphinothricin tripeptide
K20859
PhpI
3-Phosphino-pyruvate
Pmm
Pmi
K12909
K12910
K20930
K03823
K12911
K12912
K12913
D-Ribose-2,5P
K06167
1-Carboxyvinyl carboxyphosphonate
2-Phosphino-methylmalate
3-Phosphino-methylmalate
Demethyl-phosphinothricin
K20859
K12914
PhpK
K12914
PhpK
D-Ribose-5P
D-Ribose-1,5P
N-Acetylphosphinothricin
N-Acetylbialaphos
K03823
K12915
K12915
Pentose phosphate pathway
L-Phosphinothricin
Bialaphos
00440 7/24/17
(c) Kanehisa Laboratories

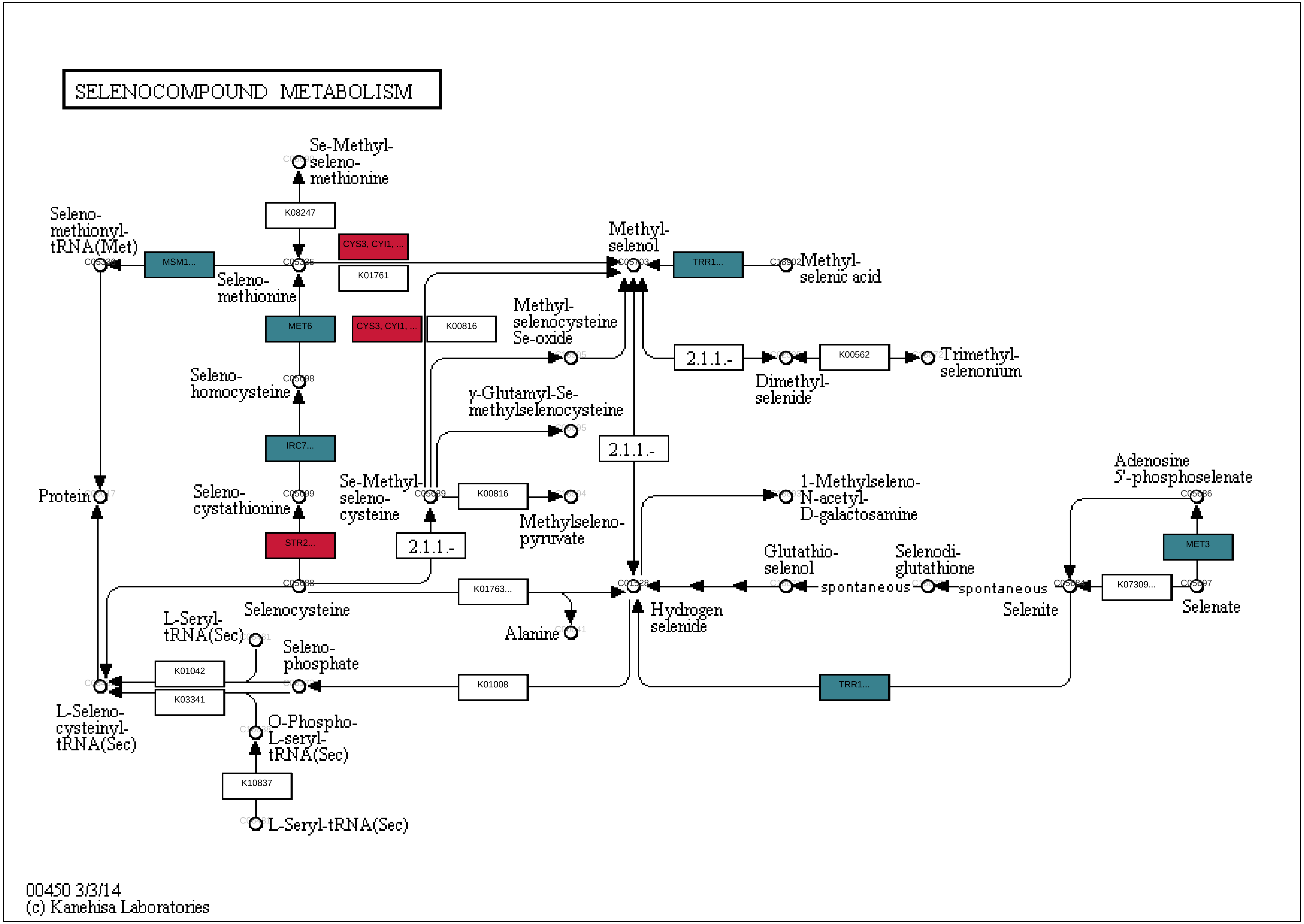
SELENOCOMPOUND METABOLISM
Se-Methyl-selenomethionine
K08247
Seleno-methionyl-tRNA(Met)
C05336
MSM1...
C05335
CYS3, CYI1, ...
K01761
Methyl-selenol
C05703
TRR1...
C18902
Methyl-selenic acid
Seleno-methionine
MET6
CYS3, CYI1, ...
K00816
Methyl-selenocysteine Se-oxide
2.1.1.-
K00562
Trimethyl-selenonium
Dimethyl-selenide
Seleno-homocysteine
C05698
γ-Glutamyl-Se-methylselenocysteine
IRC7...
2.1.1.-
Adenosine 5'-phosphoselenate
C05686
Protein
Seleno-cystathionine
C05699
Se-Methyl-selenocysteine
C05689
K00816
Methylseleno-pyruvate
1-Methylseleno-N-acetyl-D-galactosamine
MET3
STR2...
2.1.1.-
Glutathio-selenol
Selenodi-glutathione
C05688
K01763...
C01528
spontaneous
spontaneous
C05684
K07309...
C05697
Selenocysteine
Hydrogen selenide
Selenite
Selenate
L-Seryl-tRNA(Sec)
Alanine
Seleno-phosphate
K01042
K03341
K01008
TRR1...
L-Seleno-cysteinyl-tRNA(Sec)
O-Phospho-L-seryl-tRNA(Sec)
K10837
L-Seryl-tRNA(Sec)
00450 3/3/14
(c) Kanehisa Laboratories

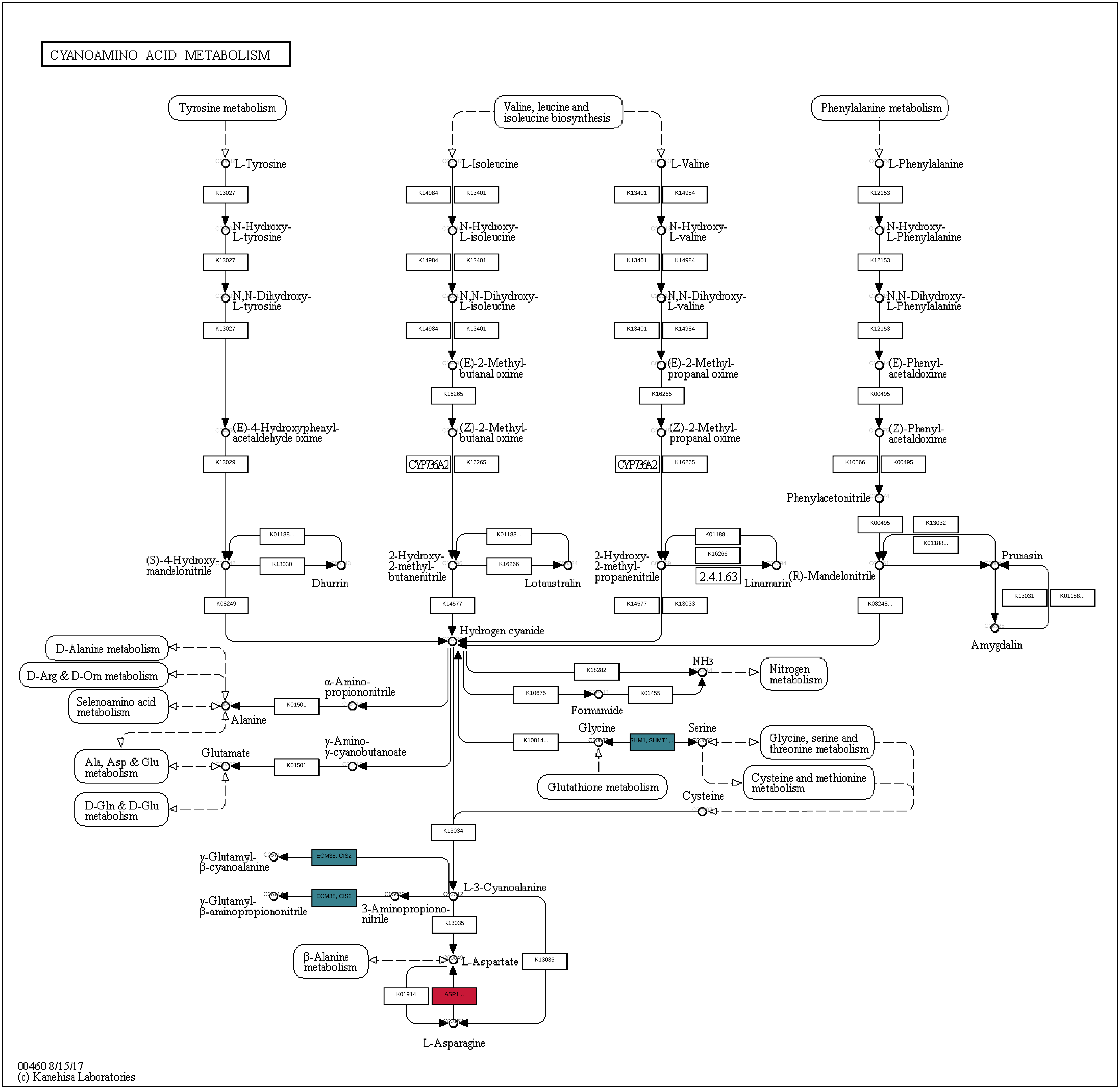
CYANOAMINO ACID METABOLISM
Tyrosine metabolism
Valine, leucine and isoleucine biosynthesis
Phenylalanine metabolism
L-Tyrosine
L-Isoleucine
L-Valine
L-Phenylalanine
K13027
K14984
K13401
K12153
N-Hydroxy-L-tyrosine
N-Hydroxy-L-isoleucine
N-Hydroxy-L-valine
N-Hydroxy-L-Phenylalanine
N,N-Dihydroxy-L-tyrosine
N,N-Dihydroxy-L-isoleucine
N,N-Dihydroxy-L-valine
N,N-Dihydroxy-L-Phenylalanine
(E)-2-Methyl-butanal oxime
(E)-2-Methyl-propanal oxime
(E)-Phenyl-acetaldoxime
K16265
K00495
(E)-4-Hydroxyphenyl-acetaldehyde oxime
(Z)-2-Methyl-butanal oxime
(Z)-2-Methyl-propanal oxime
(Z)-Phenyl-acetaldoxime
K13029
CYP736A2
K10566
Phenylacetonitrile
K01188...
K13030
K16266
K13032
2.4.1.63
(S)-4-Hydroxy-mandelonitrile
Dhurrin
2-Hydroxy-2-methyl-butanenitrile
Lotaustralin
2-Hydroxy-2-methyl-propanenitrile
Linamarin
(R)-Mandelonitrile
Prunasin
K13031
Amygdalin
K08249
K14577
K13033
K08248...
Hydrogen cyanide
D-Alanine metabolism
D-Arg & D-Orn metabolism
Selenoamino acid metabolism
Alanine
K01501
α-Amino-propiononitrile
K18282
NH3
Nitrogen metabolism
K10675
Formamide
K01455
Glutamate
Ala, Asp & Glu metabolism
D-Gln & D-Glu metabolism
γ-Amino-γ-cyanobutanoate
K10814...
Glycine
SHM1, SHMT1...
Serine
Glycine, serine and threonine metabolism
Glutathione metabolism
Cysteine and methionine metabolism
Cysteine
K13034
γ-Glutamyl-β-cyanoalanine
ECM38, CIS2
L-3-Cyanoalanine
γ-Glutamyl-β-aminopropiononitrile
3-Aminopropiono-nitrile
K13035
β-Alanine metabolism
L-Aspartate
K01914
ASP1...
L-Asparagine
00460 8/15/17
(c) Kanehisa Laboratories

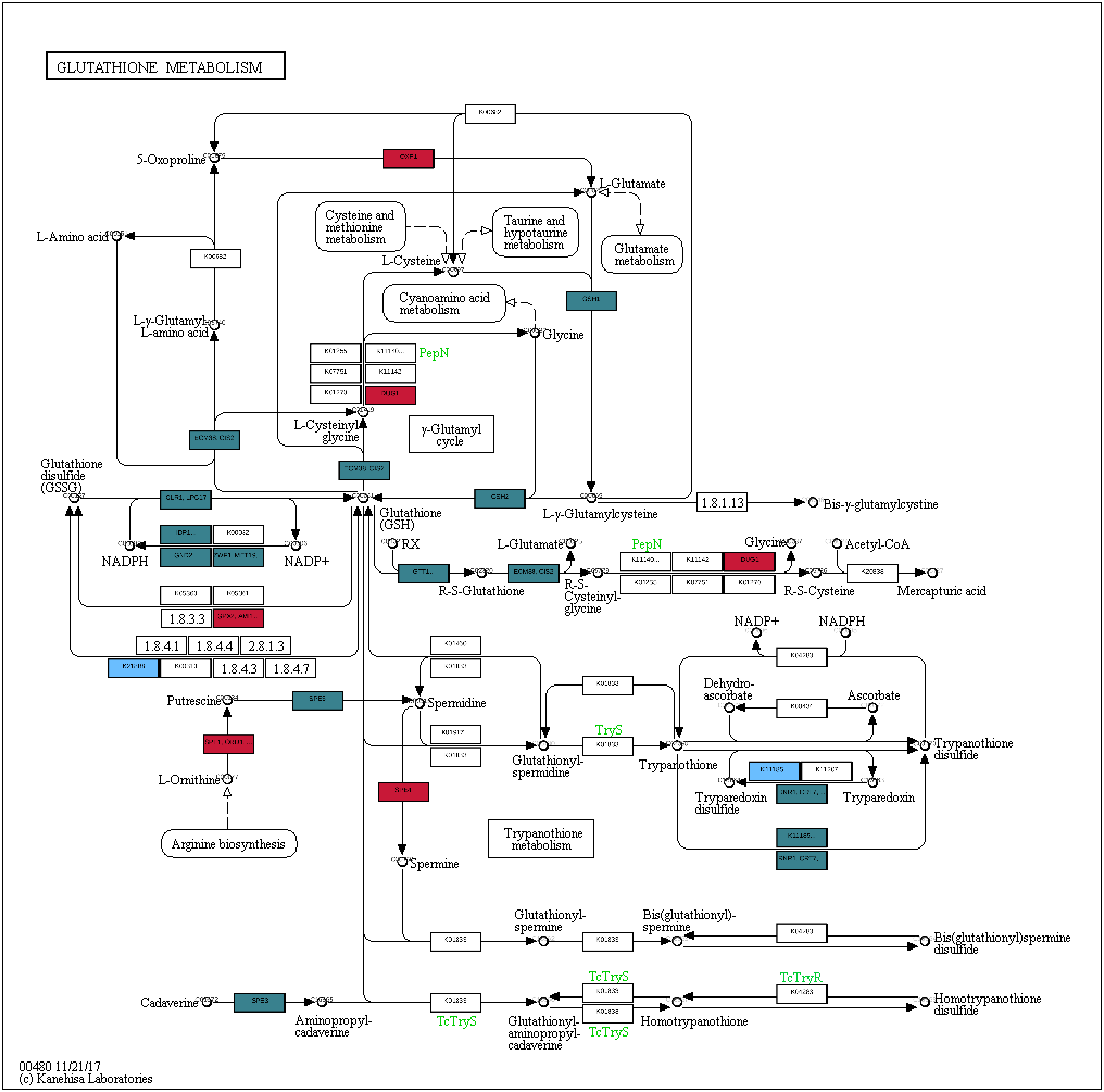

GLUTATHIONE METABOLISM
K00682
5-Oxoproline
OXP1
L-Glutamate
Cysteine and methionine metabolism
Taurine and hypotaurine metabolism
Glutamate metabolism
L-Amino acid
K00682
L-Cysteine
Cyanoamino acid metabolism
GSH1
L-γ-Glutamyl-L-amino acid
Glycine
K01255
K11140...
PepN
K07751
K11142
K01270
DUG1
L-Cysteinyl-glycine
γ-Glutamyl cycle
ECM38, CIS2
ECM38, CIS2
Glutathione disulfide (GSSG)
GLR1, LPG17
GSH2
L-γ-Glutamylcysteine
1.8.1.13
Bis-γ-glutamylcystine
Glutathione (GSH)
IDP1...
K00032
NADPH
GND2...
ZWF1, MET19,...
NADP+
RX
GTT1...
R-S-Glutathione
L-Glutamate
ECM38, CIS2
R-S-Cysteinyl-glycine
PepN
K11140...
K11142
DUG1
K01255
K07751
K01270
Glycine
R-S-Cysteine
Acetyl-CoA
K20838
Mercapturic acid
K05360
K05361
1.8.3.3
GPX2, AMI1...
1.8.4.1
1.8.4.4
2.8.1.3
K21888
K00310
1.8.4.3
1.8.4.7
K01460
K01833
NADP+
NADPH
K04283
Putrescine
SPE3
Spermidine
K01833
Dehydro-ascorbate
K00434
Ascorbate
SPE1, ORD1, ...
K01917...
TryS
K01833
K01833
Glutathionyl-spermidine
Trypanothione
Trypanothione disulfide
L-Ornithine
SPE4
K11185...
K11207
Tryparedoxin disulfide
RNR1, CRT7, ...
Tryparedoxin
Arginine biosynthesis
Trypanothione metabolism
K11185...
RNR1, CRT7, ...
Spermine
Glutathionyl-spermine
Bis(glutathionyl)-spermine
K04283
Bis(glutathionyl)spermine disulfide
K01833
K01833
TcTryS
TcTryR
K01833
K04283
Homotrypanothione disulfide
Cadaverine
SPE3
K01833
TcTryS
K01833
Aminopropyl-cadaverine
Glutathionyl-aminopropyl-cadaverine
TcTryS
Homotrypanothione
00480 11/21/17
(c) Kanehisa Laboratories

# 7. Glycan biosynthesis and metabolism

| MAP | PATHWAY |
|---|---|
| **510** | N-Glycan biosynthesis |
| **513** | Various types of N-glycan biosynthesis |
| **531** | Glycosaminoglycan degradation |
| **563** | Glycosylphosphatidylinositol (GPI)-anchor biosynthesis |

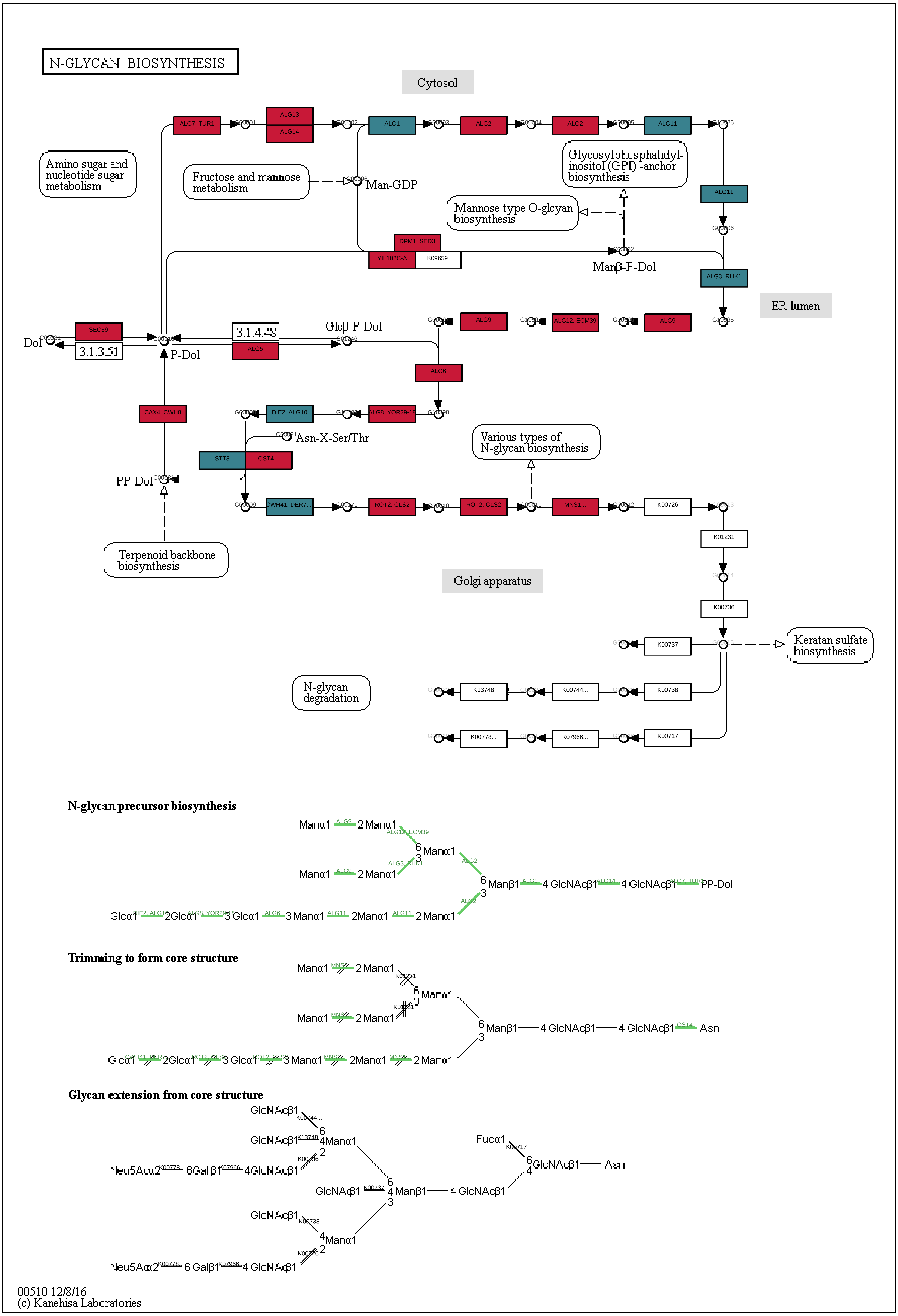

N-GLYCAN BIOSYNTHESIS
Cytosol
ER lumen
Golgi apparatus
Amino sugar and nucleotide sugar metabolism
Fructose and mannose metabolism
Glycosylphosphatidyl-inositol (GPI) -anchor biosynthesis
Mannose type O-glcyan biosynthesis
Various types of N-glycan biosynthesis
Terpenoid backbone biosynthesis
Keratan sulfate biosynthesis
N-glycan degradation
Man-GDP
Manβ-P-Dol
Glcβ-P-Dol
Dol
P-Dol
PP-Dol
Asn-X-Ser/Thr
ALG7, TUR1
ALG13
ALG14
ALG1
ALG2
ALG11
ALG3, RHK1
ALG9
ALG12, ECM39
ALG6
ALG8, YOR29-18
DIE2, ALG10
STT3
OST4...
CWH41, DER7,...
ROT2, GLS2
MNS1...
DPM1, SED3
YIL102C-A
K09659
SEC59
3.1.3.51
3.1.4.48
ALG5
CAX4, CWH8
K00726
K01231
K00736
K00737
K00738
K00744...
K13748
K00717
K07966...
K00778...
N-glycan precursor biosynthesis
Trimming to form core structure
Glycan extension from core structure
00510 12/8/16
(c) Kanehisa Laboratories

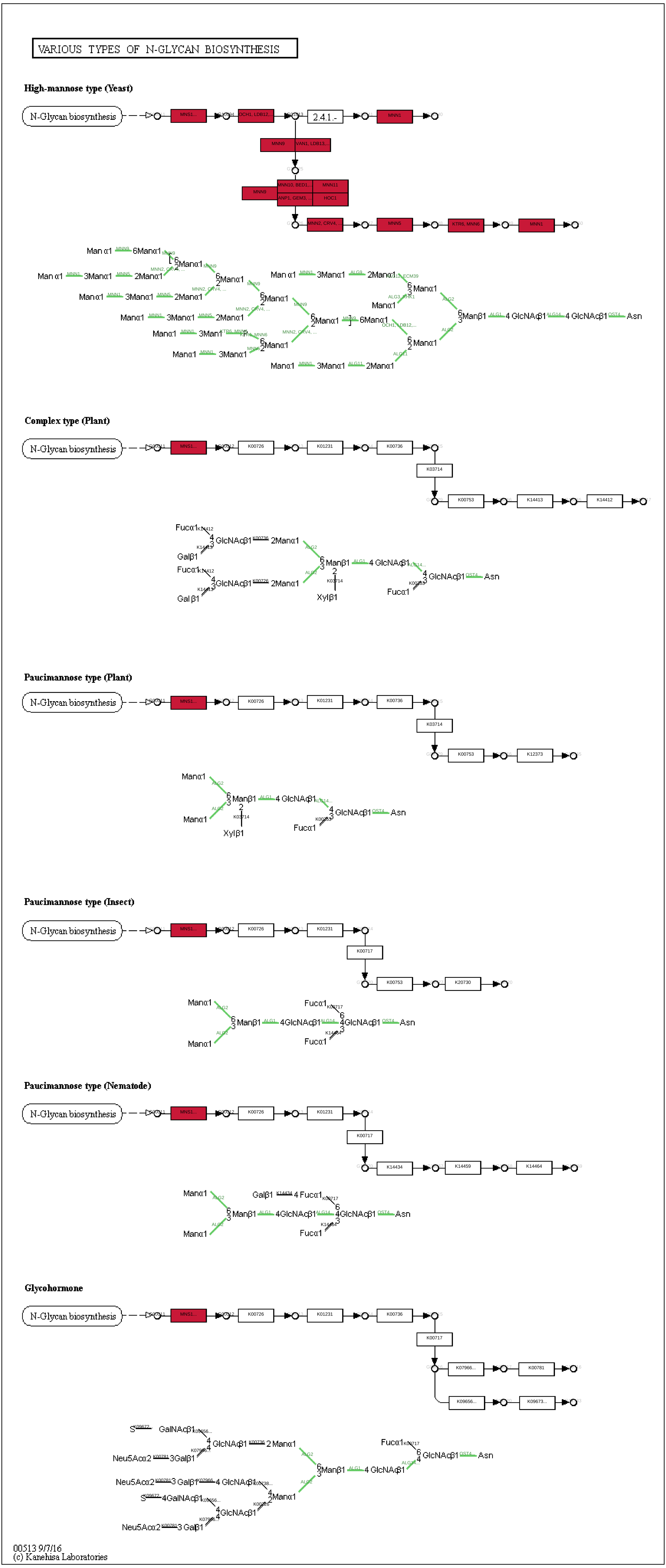
VARIOUS TYPES OF N-GLYCAN BIOSYNTHESIS
High-mannose type (Yeast)
N-Glycan biosynthesis
2.4.1.-
Complex type (Plant)
N-Glycan biosynthesis
Paucimannose type (Plant)
N-Glycan biosynthesis
Paucimannose type (Insect)
N-Glycan biosynthesis
Paucimannose type (Nematode)
N-Glycan biosynthesis
Glycohormone
N-Glycan biosynthesis
00513 9/7/16
(c) Kanehisa Laboratories

**NO REFERENCE PATHWAY AVAILABLE:**

**map 531 Glycosaminoglycan degradation**

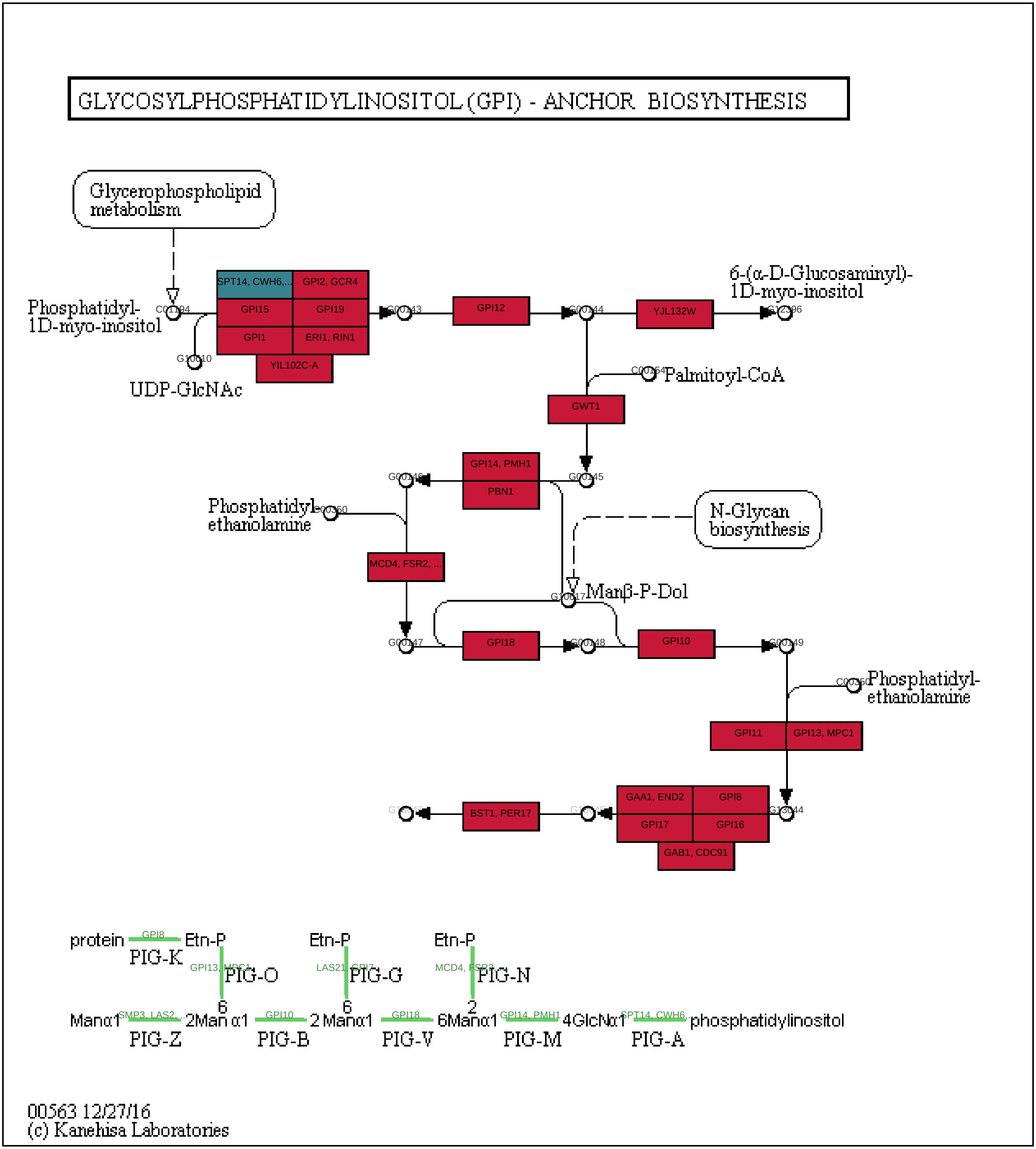
GLYCOSYLPHOSPHATIDYLINOSITOL (GPI) - ANCHOR BIOSYNTHESIS
Glycerophospholipid metabolism
Phosphatidyl-1D-myo-inositol
C01194
SPT14, CWH6,...
GPI2, GCR4
GPI15
GPI19
GPI1
ERI1, RIN1
YIL102C-A
G10610
UDP-GlcNAc
G00143
GPI12
G00144
YJL132W
G12396
6-(α-D-Glucosaminyl)-1D-myo-inositol
C00154
Palmitoyl-CoA
GWT1
G00145
GPI14, PMH1
PBN1
G00146
Phosphatidyl-ethanolamine
C00350
MCD4, FSR2, ...
N-Glycan biosynthesis
Manβ-P-Dol
G10617
G00147
GPI18
G00148
GPI10
G00149
C00350
Phosphatidyl-ethanolamine
GPI11
GPI13, MPC1
GAA1, END2
GPI8
GPI17
GPI16
GAB1, CDC91
G13044
BST1, PER17
protein
GPI8
Etn-P
PIG-K
GPI13, MPC1
PIG-O
Etn-P
LAS21, GPI7
PIG-G
Etn-P
MCD4, FSR2
PIG-N
6
6
2
Manα1
SMP3, LAS2,...
2Man α1
GPI10
2 Manα1
GPI18
6Manα1
GPI14, PMH1
4GlcNα1
SPT14, CWH6,...
phosphatidylinositol
PIG-Z
PIG-B
PIG-V
PIG-M
PIG-A
00563 12/27/16
(c) Kanehisa Laboratories

# 8. Metabolism of cofactors and vitamins

| MAP | PATHWAY |
|---|---|
| **130** | Ubiquinone and other terpenoid-quinone biosynthesis |
| **670** | One carbon pool by folate |
| **730** | Thiamine metabolism |
| **740** | Riboflavin metabolism |
| **750** | Vitamin B6 metabolism |
| **760** | Nicotinate and nicotinamide metabolism |
| **770** | Pantothenate and CoA biosynthesis |
| **780** | Biotin metabolism |
| **785** | Lipoic acid metabolism |
| **790** | Folate biosynthesis |
| **860** | Porphyrin and chlorophyll metabolism |

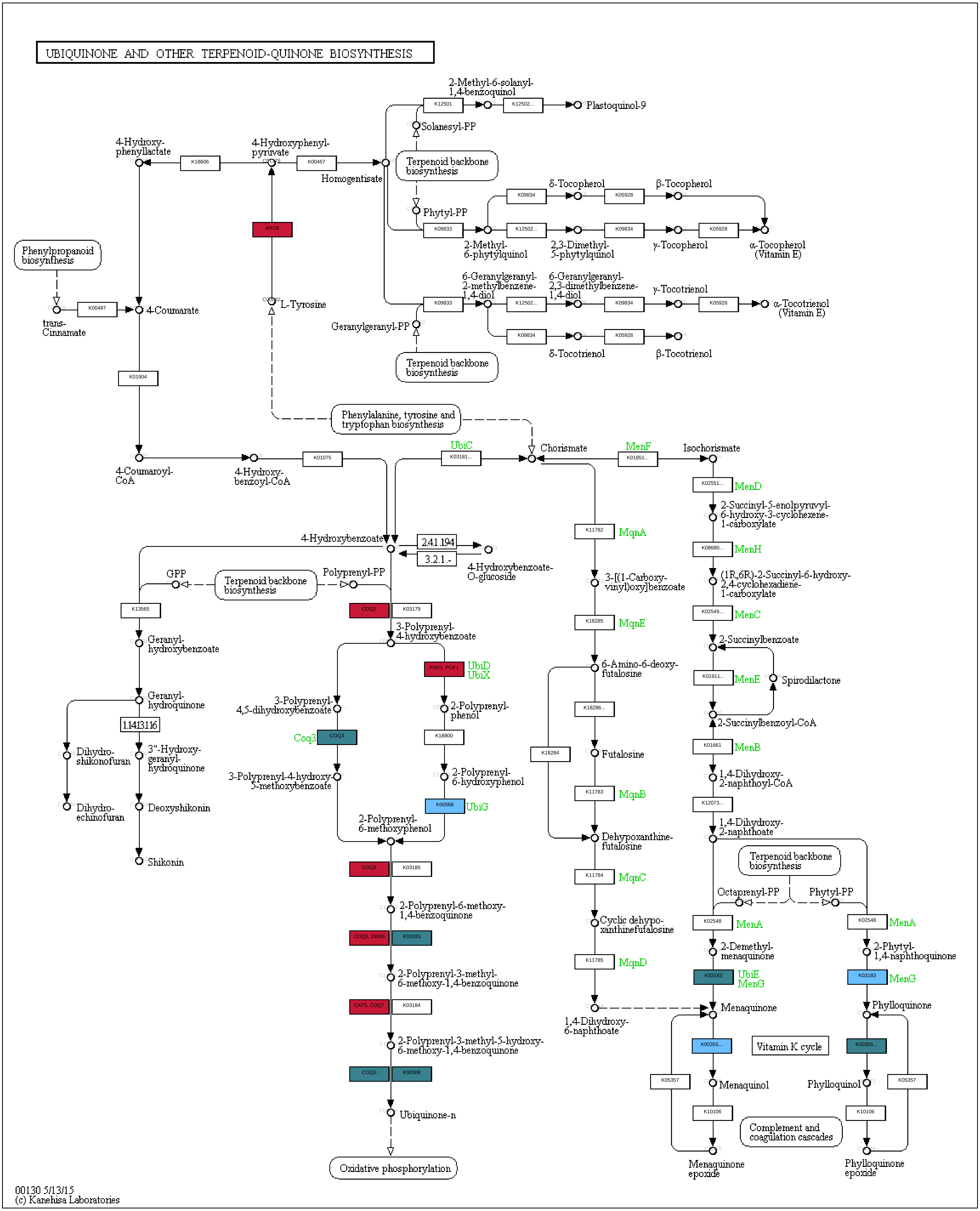
UBIQUINONE AND OTHER TERPENOID-QUINONE BIOSYNTHESIS
2-Methyl-6-solanyl-1,4-benzoquinol
K12501
K12502...
Plastoquinol-9
Solanesyl-PP
4-Hydroxy-phenyllactate
4-Hydroxyphenyl-pyruvate
K18606
K00457
Homogentisate
Terpenoid backbone biosynthesis
δ-Tocopherol
β-Tocopherol
K09834
K05928
Phytyl-PP
ARO8
K09833
K12502...
K09834
K05928
2-Methyl-6-phytylquinol
2,3-Dimethyl-5-phytylquinol
γ-Tocopherol
α-Tocopherol (Vitamin E)
Phenylpropanoid biosynthesis
6-Geranylgeranyl-2-methylbenzene-1,4-diol
6-Geranylgeranyl-2,3-dimethylbenzene-1,4-diol
γ-Tocotrienol
L-Tyrosine
K00487
4-Coumarate
K09833
K12502...
K09834
K05928
α-Tocotrienol (Vitamin E)
trans-Cinnamate
Geranylgeranyl-PP
K09834
K05928
δ-Tocotrienol
β-Tocotrienol
Terpenoid backbone biosynthesis
K01904
Phenylalanine, tyrosine and tryptophan biosynthesis
UbiC
MenF
K01075
K03181...
Chorismate
K01851...
Isochorismate
4-Coumaroyl-CoA
4-Hydroxy-benzoyl-CoA
K02551...
MenD
2-Succinyl-5-enolpyruvyl-6-hydroxy-3-cyclohexene-1-carboxylate
K11782
MqnA
4-Hydroxybenzoate
2.4.1.194
3.2.1.-
K08680...
MenH
4-Hydroxybenzoate-O-glucoside
GPP
Terpenoid backbone biosynthesis
Polyprenyl-PP
3-[(1-Carboxy-vinyl)oxy]benzoate
(1R,6R)-2-Succinyl-6-hydroxy-2,4-cyclohexadiene-1-carboxylate
K13565
COQ2
K03179
K02549...
MenC
K18285
MqnE
Geranyl-hydroxybenzoate
3-Polyprenyl-4-hydroxybenzoate
2-Succinylbenzoate
PAD1, PDF1
UbiD
UbiX
6-Amino-6-deoxy-futalosine
K01911...
MenE
Spirodilactone
Geranyl-hydroquinone
3-Polyprenyl-4,5-dihydroxybenzoate
2-Polyprenyl-phenol
1.14.13.116
K18286...
2-Succinylbenzoyl-CoA
Coq3
COQ3
K18800
3''-Hydroxy-geranyl-hydroquinone
Dihydro-shikonofuran
K18284
Futalosine
K01661
MenB
3-Polyprenyl-4-hydroxy-5-methoxybenzoate
2-Polyprenyl-6-hydroxyphenol
1,4-Dihydroxy-2-naphthoyl-CoA
K11783
MqnB
Dihydro-echinofuran
Deoxyshikonin
K00568
UbiG
K12073...
2-Polyprenyl-6-methoxyphenol
Dehypoxanthine-futalosine
1,4-Dihydroxy-2-naphthoate
Shikonin
COQ6
K03185
Terpenoid backbone biosynthesis
K11784
MqnC
Octaprenyl-PP
Phytyl-PP
2-Polyprenyl-6-methoxy-1,4-benzoquinone
Cyclic dehypo-xanthinefutalosine
K02548
MenA
K02548
MenA
COQ5, DBI56
K03183
2-Demethyl-menaquinone
2-Phytyl-1,4-naphthoquinone
2-Polyprenyl-3-methyl-6-methoxy-1,4-benzoquinone
K11785
MqnD
K03183
UbiE
MenG
K03183
MenG
CAT5, COQ7
K03184
Menaquinone
Phylloquinone
1,4-Dihydroxy-6-naphthoate
2-Polyprenyl-3-methyl-5-hydroxy-6-methoxy-1,4-benzoquinone
K00355...
Vitamin K cycle
K00355...
K05357
K05357
COQ3
K00568
Menaquinol
Phylloquinol
Ubiquinone-n
K10106
K10106
Complement and coagulation cascades
Oxidative phosphorylation
Menaquinone epoxide
Phylloquinone epoxide
00130 5/13/15
(c) Kanehisa Laboratories

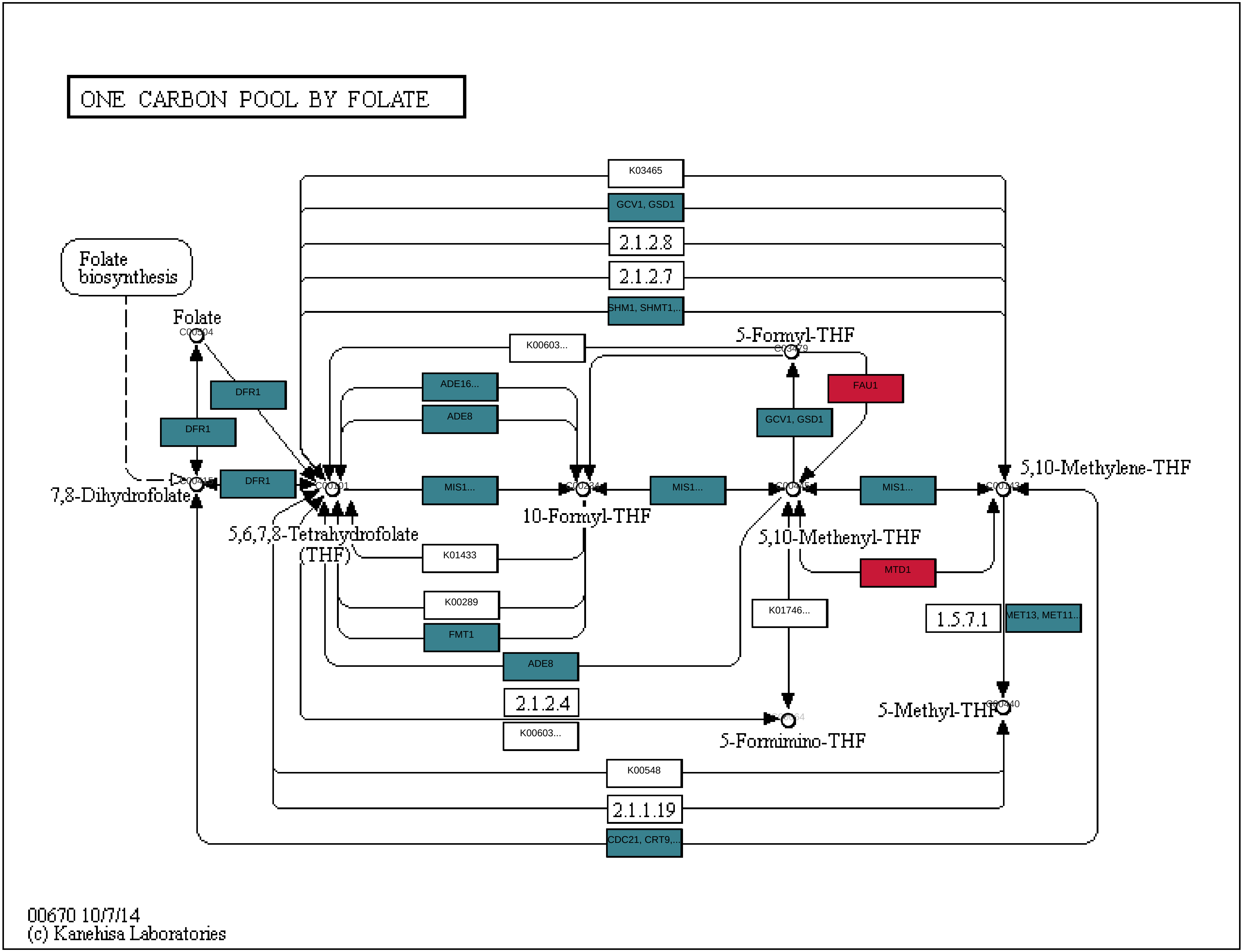
ONE CARBON POOL BY FOLATE
K03465
GCV1, GSD1
2.1.2.8
2.1.2.7
SHM1, SHMT1,...
Folate biosynthesis
Folate
C00504
DFR1
DFR1
DFR1
7,8-Dihydrofolate
K00603...
ADE16...
ADE8
MIS1...
MIS1...
MIS1...
5-Formyl-THF
FAU1
GCV1, GSD1
5,10-Methylene-THF
10-Formyl-THF
5,6,7,8-Tetrahydrofolate (THF)
5,10-Methenyl-THF
K01433
K00289
FMT1
ADE8
2.1.2.4
K00603...
K01746...
MTD1
1.5.7.1
MET13, MET11...
5-Methyl-THF
5-Formimino-THF
K00548
2.1.1.19
CDC21, CRT9,...
00670 10/7/14
(c) Kanehisa Laboratories

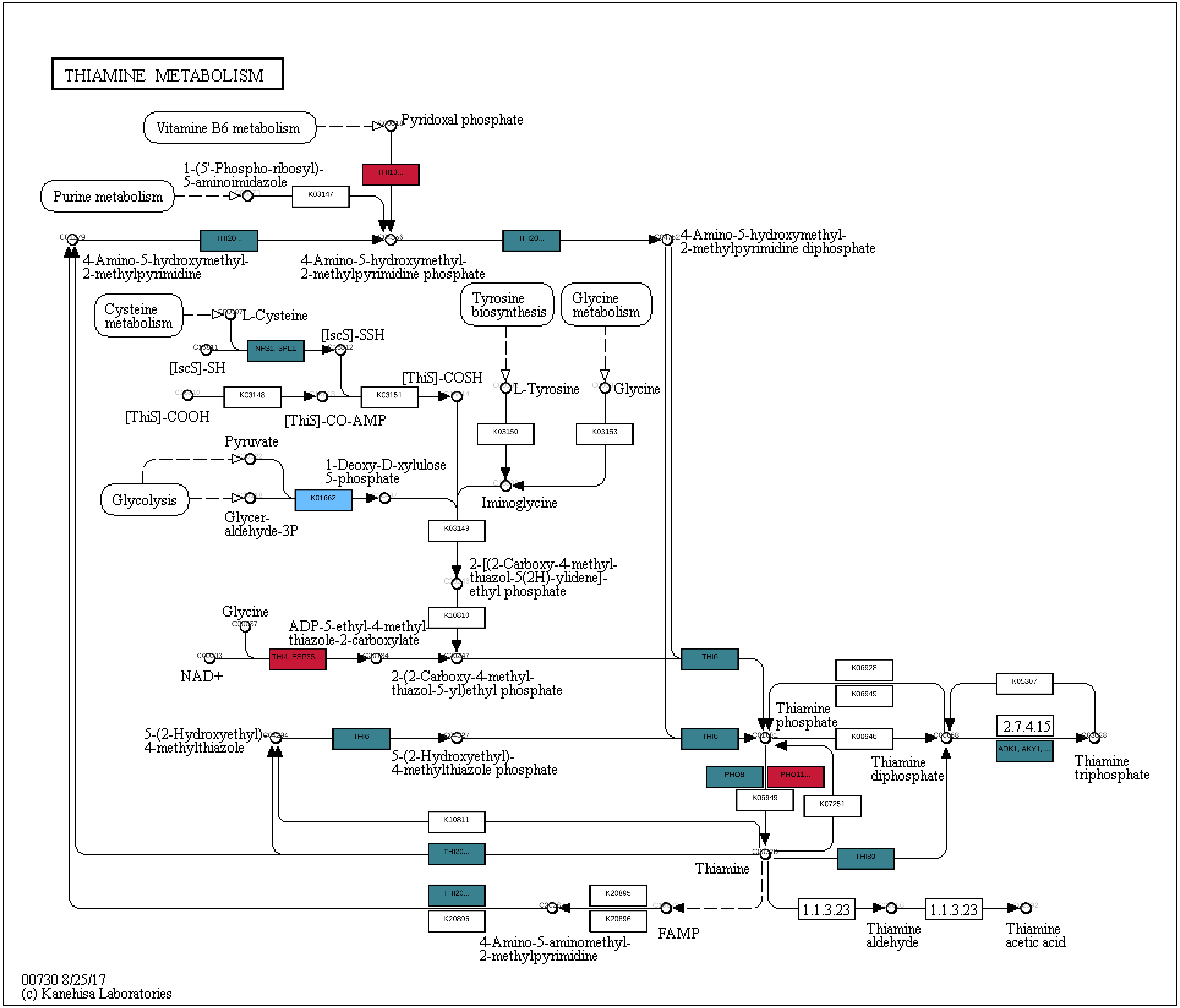
THIAMINE METABOLISM
Vitamine B6 metabolism
Pyridoxal phosphate
THI13...
Purine metabolism
1-(5'-Phospho-ribosyl)-
5-aminoimidazole
K03147
C01279
THI20...
4-Amino-5-hydroxymethyl-
2-methylpyrimidine
C04556
4-Amino-5-hydroxymethyl-
2-methylpyrimidine phosphate
THI20...
C04752
4-Amino-5-hydroxymethyl-
2-methylpyrimidine diphosphate
Cysteine
metabolism
C00097
L-Cysteine
NFS1, SPL1
[IscS]-SSH
[IscS]-SH
Tyrosine
biosynthesis
Glycine
metabolism
[ThiS]-COSH
K03148
K03151
L-Tyrosine
Glycine
[ThiS]-COOH
[ThiS]-CO-AMP
K03150
K03153
Pyruvate
1-Deoxy-D-xylulose
5-phosphate
Glycolysis
K01662
Iminoglycine
Glycer-
aldehyde-3P
K03149
2-[(2-Carboxy-4-methyl-
thiazol-5(2H)-ylidene]-
ethyl phosphate
Glycine
C00037
K10810
ADP-5-ethyl-4-methyl-
thiazole-2-carboxylate
C00003
THI4, ESP35,...
C20784
C20247
THI6
NAD+
2-(2-Carboxy-4-methyl-
thiazol-5-yl)ethyl phosphate
K06928
K06949
K05307
Thiamine
phosphate
5-(2-Hydroxyethyl)-
4-methylthiazole
C04294
THI6
C04327
THI6
C01081
K00946
C00068
2.7.4.15
C03028
ADK1, AKY1, ...
5-(2-Hydroxyethyl)-
4-methylthiazole phosphate
Thiamine
diphosphate
Thiamine
triphosphate
PHO8
PHO11...
K06949
K07251
K10811
THI20...
C00378
THI80
Thiamine
THI20...
K20895
C20267
K20896
K20896
1.1.3.23
1.1.3.23
Thiamine
aldehyde
Thiamine
acetic acid
FAMP
4-Amino-5-aminomethyl-
2-methylpyrimidine
00730 8/25/17
(c) Kanehisa Laboratories

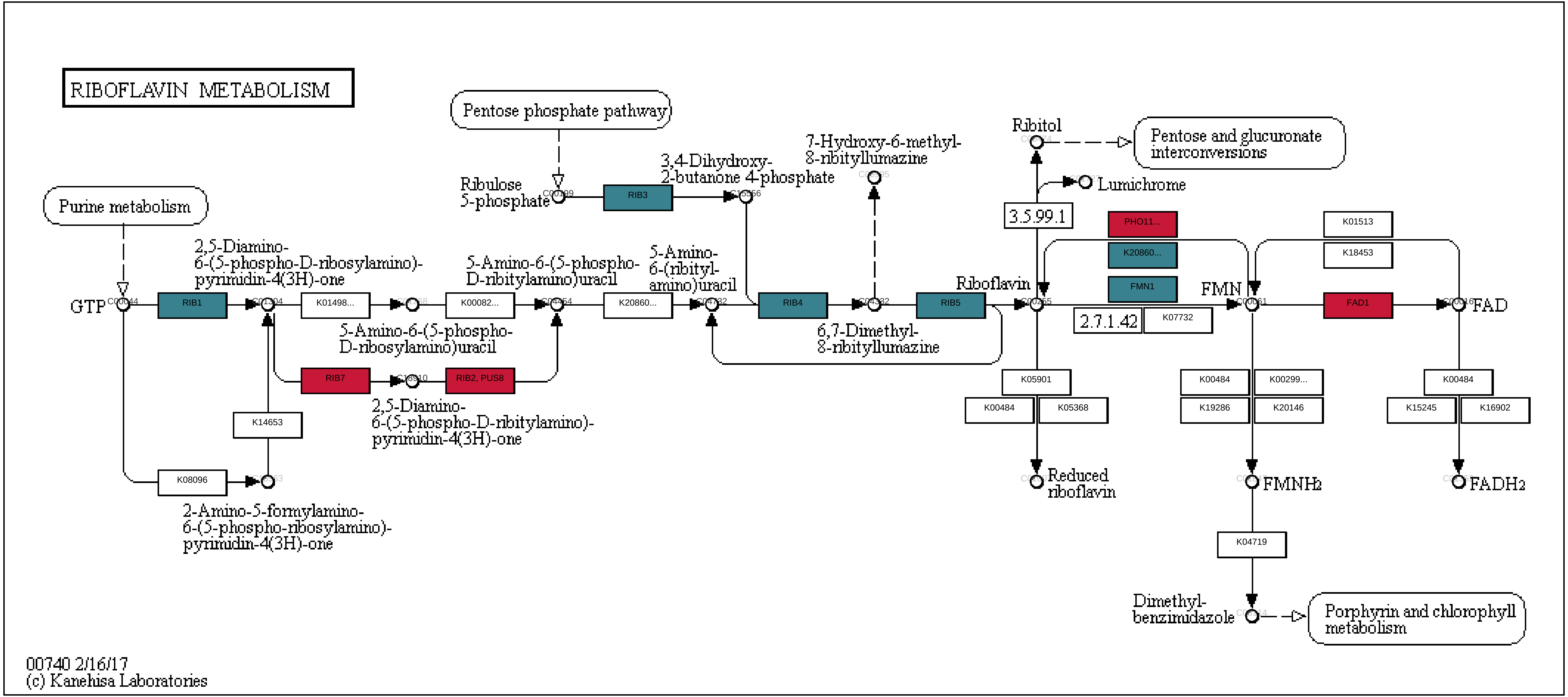

RIBOFLAVIN METABOLISM
Pentose phosphate pathway
Purine metabolism
Ribulose 5-phosphate
3,4-Dihydroxy-2-butanone 4-phosphate
7-Hydroxy-6-methyl-8-ribityllumazine
Ribitol
Pentose and glucuronate interconversions
Lumichrome
3.5.99.1
GTP
RIB1
2,5-Diamino-6-(5-phospho-D-ribosylamino)-pyrimidin-4(3H)-one
K01498...
5-Amino-6-(5-phospho-D-ribitylamino)uracil
K00082...
5-Amino-6-(ribitylamino)uracil
K20860...
RIB3
RIB4
RIB5
Riboflavin
PHO11...
K20860...
FMN1
K01513
K18453
FMN
FAD1
FAD
2.7.1.42
K07732
5-Amino-6-(5-phospho-D-ribosylamino)uracil
6,7-Dimethyl-8-ribityllumazine
RIB7
RIB2, PUS8
2,5-Diamino-6-(5-phospho-D-ribitylamino)-pyrimidin-4(3H)-one
K14653
K08096
2-Amino-5-formylamino-6-(5-phospho-ribosylamino)-pyrimidin-4(3H)-one
K05901
K00484
K05368
Reduced riboflavin
K00484
K00299...
K19286
K20146
FMNH2
K04719
Dimethyl-benzimidazole
Porphyrin and chlorophyll metabolism
K00484
K15245
K16902
FADH2
00740 2/16/17
(c) Kanehisa Laboratories

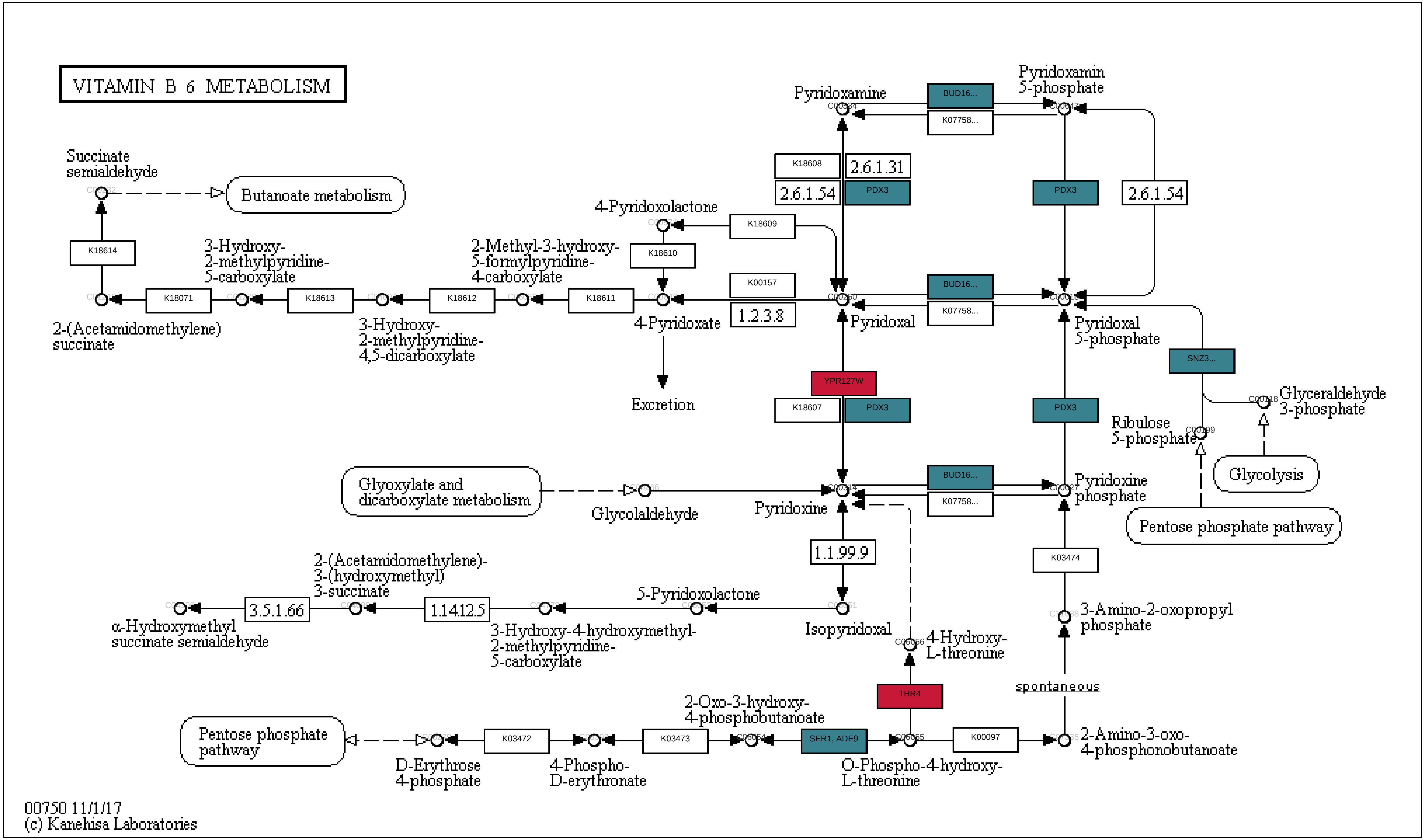
VITAMIN B 6 METABOLISM
Succinate semialdehyde
Butanoate metabolism
K18614
3-Hydroxy-2-methylpyridine-5-carboxylate
2-Methyl-3-hydroxy-5-formylpyridine-4-carboxylate
4-Pyridoxolactone
K18609
K18610
K18071
K18613
K18612
K18611
K00157
1.2.3.8
2-(Acetamidomethylene) succinate
3-Hydroxy-2-methylpyridine-4,5-dicarboxylate
4-Pyridoxate
Excretion
Pyridoxamine
BUD16...
K07758...
Pyridoxamin 5-phosphate
K18608
2.6.1.31
2.6.1.54
PDX3
2.6.1.54
PDX3
BUD16...
K07758...
Pyridoxal
Pyridoxal 5-phosphate
SNZ3...
YPR127W
K18607
PDX3
PDX3
Glyceraldehyde 3-phosphate
Ribulose 5-phosphate
Glycolysis
Glyoxylate and dicarboxylate metabolism
Glycolaldehyde
Pyridoxine
BUD16...
K07758...
Pyridoxine phosphate
Pentose phosphate pathway
1.1.99.9
K03474
2-(Acetamidomethylene)-3-(hydroxymethyl) 3-succinate
5-Pyridoxolactone
3.5.1.66
1.14.12.5
α-Hydroxymethyl succinate semialdehyde
3-Hydroxy-4-hydroxymethyl-2-methylpyridine-5-carboxylate
Isopyridoxal
4-Hydroxy-L-threonine
3-Amino-2-oxopropyl phosphate
spontaneous
THR4
2-Oxo-3-hydroxy-4-phosphobutanoate
Pentose phosphate pathway
K03472
K03473
SER1, ADE9
K00097
2-Amino-3-oxo-4-phosphonobutanoate
D-Erythrose 4-phosphate
4-Phospho-D-erythronate
O-Phospho-4-hydroxy-L-threonine
00750 11/1/17
(c) Kanehisa Laboratories

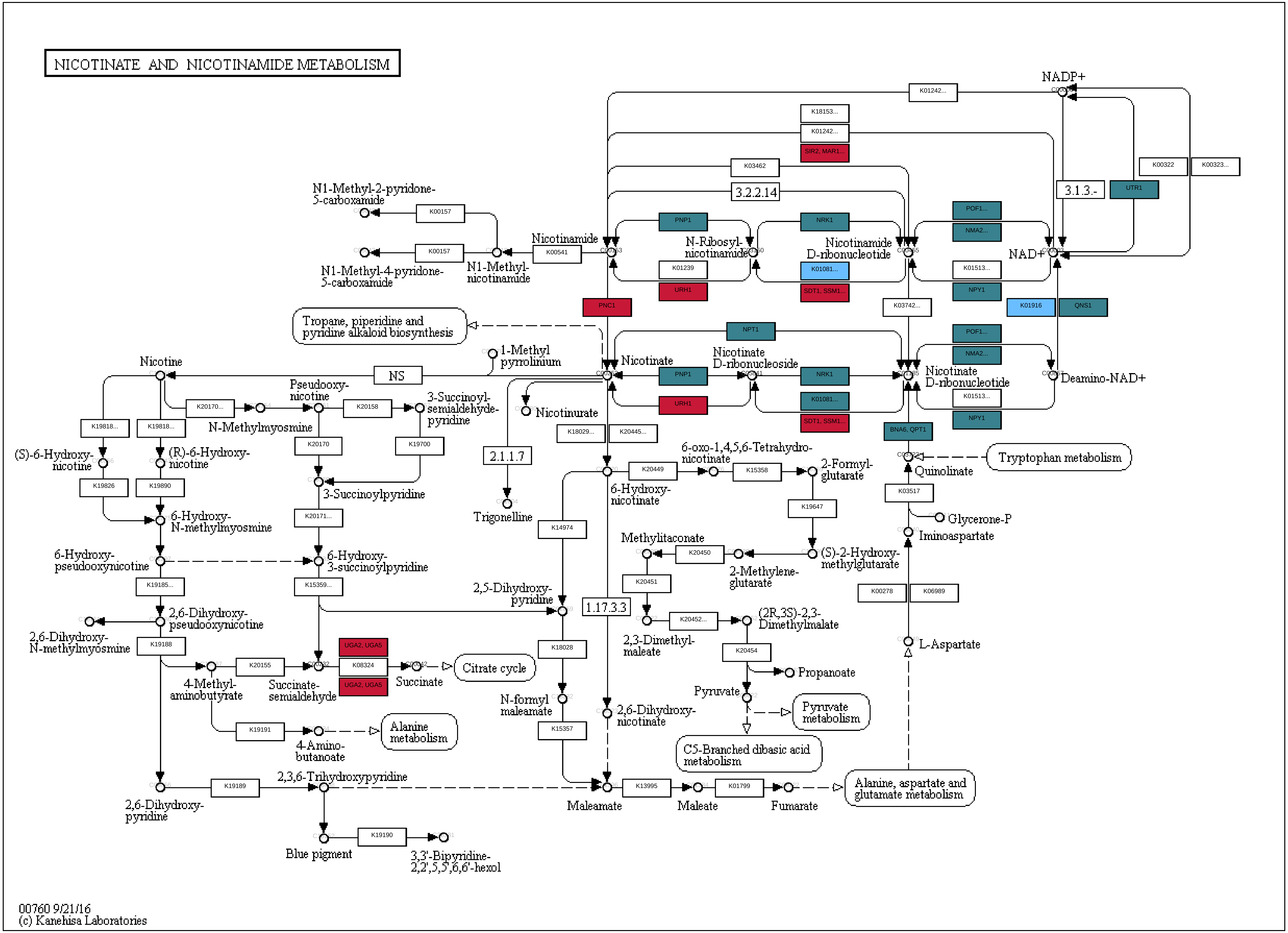

NICOTINATE AND NICOTINAMIDE METABOLISM
NADP+
K01242...
K18153...
K01242...
SIR2, MAR1...
K03462
3.2.2.14
K00322
K00323...
3.1.3.-
UTR1
N1-Methyl-2-pyridone-5-carboxamide
K00157
K00157
N1-Methyl-4-pyridone-5-carboxamide
N1-Methyl-nicotinamide
K00541
Nicotinamide
PNP1
NRK1
POF1...
NMA2...
N-Ribosyl-nicotinamide
Nicotinamide D-ribonucleotide
NAD+
K01239
URH1
K01081...
SDT1, SSM1...
K01513...
NPY1
PNC1
K03742...
K01916
QNS1
Tropane, piperidine and pyridine alkaloid biosynthesis
NPT1
POF1...
NMA2...
1-Methyl pyrrolinium
Nicotine
NS
Nicotinate
PNP1
Nicotinate D-ribonucleoside
NRK1
Nicotinate D-ribonucleotide
Deamino-NAD+
Pseudooxy-nicotine
K20170...
K20158
3-Succinoyl-semialdehyde-pyridine
URH1
K01081...
K01513...
NPY1
Nicotinurate
SDT1, SSM1...
BNA6, QPT1
K19818...
K19818...
N-Methylmyosmine
K20170
K19700
K18029...
K20445...
6-oxo-1,4,5,6-Tetrahydro-nicotinate
2-Formyl-glutarate
(S)-6-Hydroxy-nicotine
(R)-6-Hydroxy-nicotine
2.1.1.7
K20449
K15358
Tryptophan metabolism
Quinolinate
K19826
K19890
3-Succinoylpyridine
6-Hydroxy-nicotinate
K03517
Trigonelline
K19647
Glycerone-P
6-Hydroxy-N-methylmyosmine
K20171...
K14974
Iminoaspartate
Methylitaconate
6-Hydroxy-pseudooxynicotine
6-Hydroxy-3-succinoylpyridine
K20450
(S)-2-Hydroxy-methylglutarate
2-Methylene-glutarate
K19185...
K15359...
K20451
2,5-Dihydroxy-pyridine
K00278
K06989
1.17.3.3
2,6-Dihydroxy-pseudooxynicotine
K20452...
(2R,3S)-2,3-Dimethylmalate
2,6-Dihydroxy-N-methylmyosmine
K19188
UGA2, UGA5
L-Aspartate
2,3-Dimethyl-maleate
K18028
K20454
K20155
K08324
Citrate cycle
Succinate
UGA2, UGA5
Propanoate
4-Methyl-aminobutyrate
Succinate-semialdehyde
Pyruvate
N-formyl maleamate
2,6-Dihydroxy-nicotinate
Pyruvate metabolism
K19191
Alanine metabolism
4-Amino-butanoate
K15357
C5-Branched dibasic acid metabolism
2,3,6-Trihydroxypyridine
K19189
K13995
K01799
Alanine, aspartate and glutamate metabolism
2,6-Dihydroxy-pyridine
Maleamate
Maleate
Fumarate
K19190
Blue pigment
3,3'-Bipyridine-2,2',5,5',6,6'-hexol
00760 9/21/16
(c) Kanehisa Laboratories

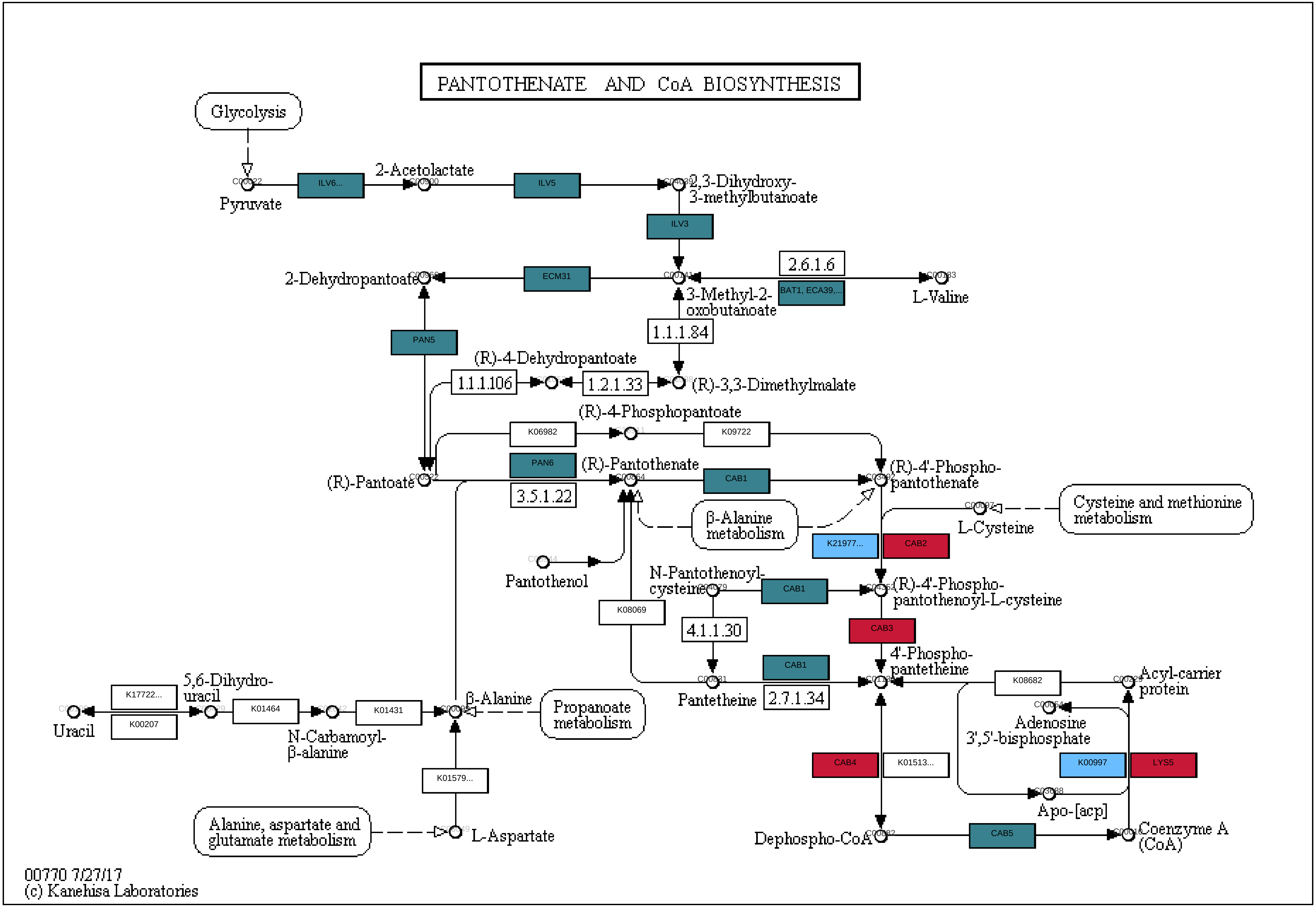
PANTOTHENATE AND CoA BIOSYNTHESIS
Glycolysis
Pyruvate
ILV6...
2-Acetolactate
ILV5
2,3-Dihydroxy-
3-methylbutanoate
ILV3
2.6.1.6
BAT1, ECA39,...
L-Valine
3-Methyl-2-
oxobutanoate
ECM31
2-Dehydropantoate
PAN5
1.1.1.84
(R)-4-Dehydropantoate
1.1.1.106
1.2.1.33
(R)-3,3-Dimethylmalate
(R)-4-Phosphopantoate
K06982
K09722
PAN6
(R)-Pantothenate
(R)-Pantoate
3.5.1.22
CAB1
(R)-4'-Phospho-
pantothenate
β-Alanine
metabolism
Cysteine and methionine
metabolism
L-Cysteine
K21977...
CAB2
Pantothenol
N-Pantothenoyl-
cysteine
CAB1
(R)-4'-Phospho-
pantothenoyl-L-cysteine
K08069
4.1.1.30
CAB3
4'-Phospho-
pantetheine
CAB1
2.7.1.34
Pantetheine
K08682
Acyl-carrier
protein
Adenosine
3',5'-bisphosphate
K17722...
K00207
5,6-Dihydro-
uracil
K01464
K01431
β-Alanine
Propanoate
metabolism
Uracil
N-Carbamoyl-
β-alanine
K01579...
CAB4
K01513...
K00997
LYS5
Apo-[acp]
Alanine, aspartate and
glutamate metabolism
L-Aspartate
Dephospho-CoA
CAB5
Coenzyme A
(CoA)
00770 7/27/17
(c) Kanehisa Laboratories

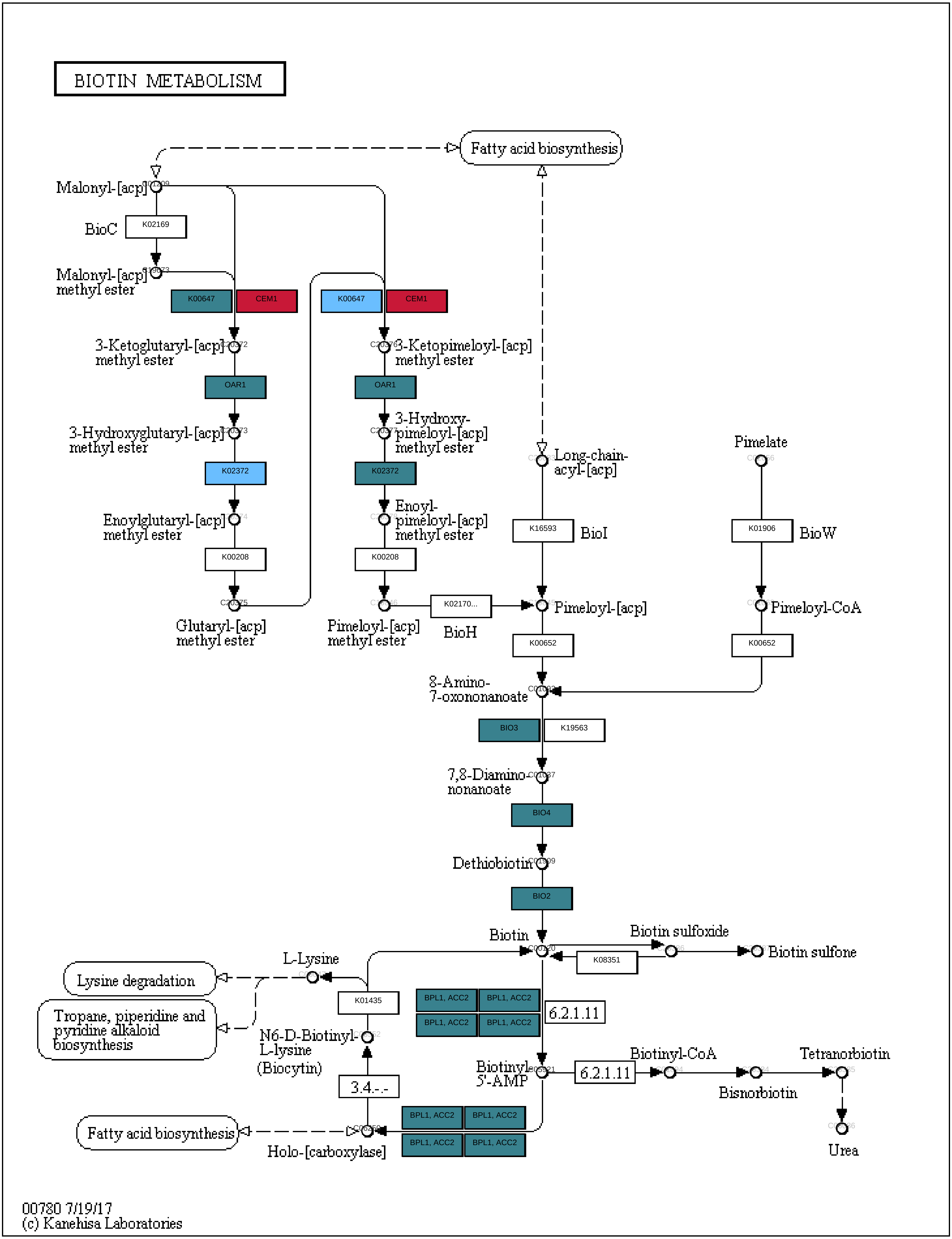

BIOTIN METABOLISM
Fatty acid biosynthesis
Malonyl-[acp]
BioC
K02169
Malonyl-[acp] methyl ester
K00647
CEM1
K00647
CEM1
3-Ketoglutaryl-[acp] methyl ester
3-Ketopimeloyl-[acp] methyl ester
OAR1
OAR1
3-Hydroxyglutaryl-[acp] methyl ester
3-Hydroxy-pimeloyl-[acp] methyl ester
K02372
K02372
Enoylglutaryl-[acp] methyl ester
Enoyl-pimeloyl-[acp] methyl ester
K00208
K00208
Glutaryl-[acp] methyl ester
Pimeloyl-[acp] methyl ester
K02170...
BioH
Long-chain-acyl-[acp]
K16593
BioI
Pimelate
K01906
BioW
Pimeloyl-[acp]
Pimeloyl-CoA
K00652
K00652
8-Amino-7-oxononanoate
BIO3
K19563
7,8-Diamino-nonanoate
BIO4
Dethiobiotin
BIO2
Biotin
Biotin sulfoxide
Biotin sulfone
K08351
L-Lysine
Lysine degradation
Tropane, piperidine and pyridine alkaloid biosynthesis
K01435
BPL1, ACC2
BPL1, ACC2
BPL1, ACC2
BPL1, ACC2
6.2.1.11
N6-D-Biotinyl-L-lysine (Biocytin)
3.4.-.-
Biotinyl-5'-AMP
6.2.1.11
Biotinyl-CoA
Tetranorbiotin
Bisnorbiotin
Urea
BPL1, ACC2
BPL1, ACC2
BPL1, ACC2
BPL1, ACC2
Fatty acid biosynthesis
Holo-[carboxylase]
00780 7/19/17
(c) Kanehisa Laboratories

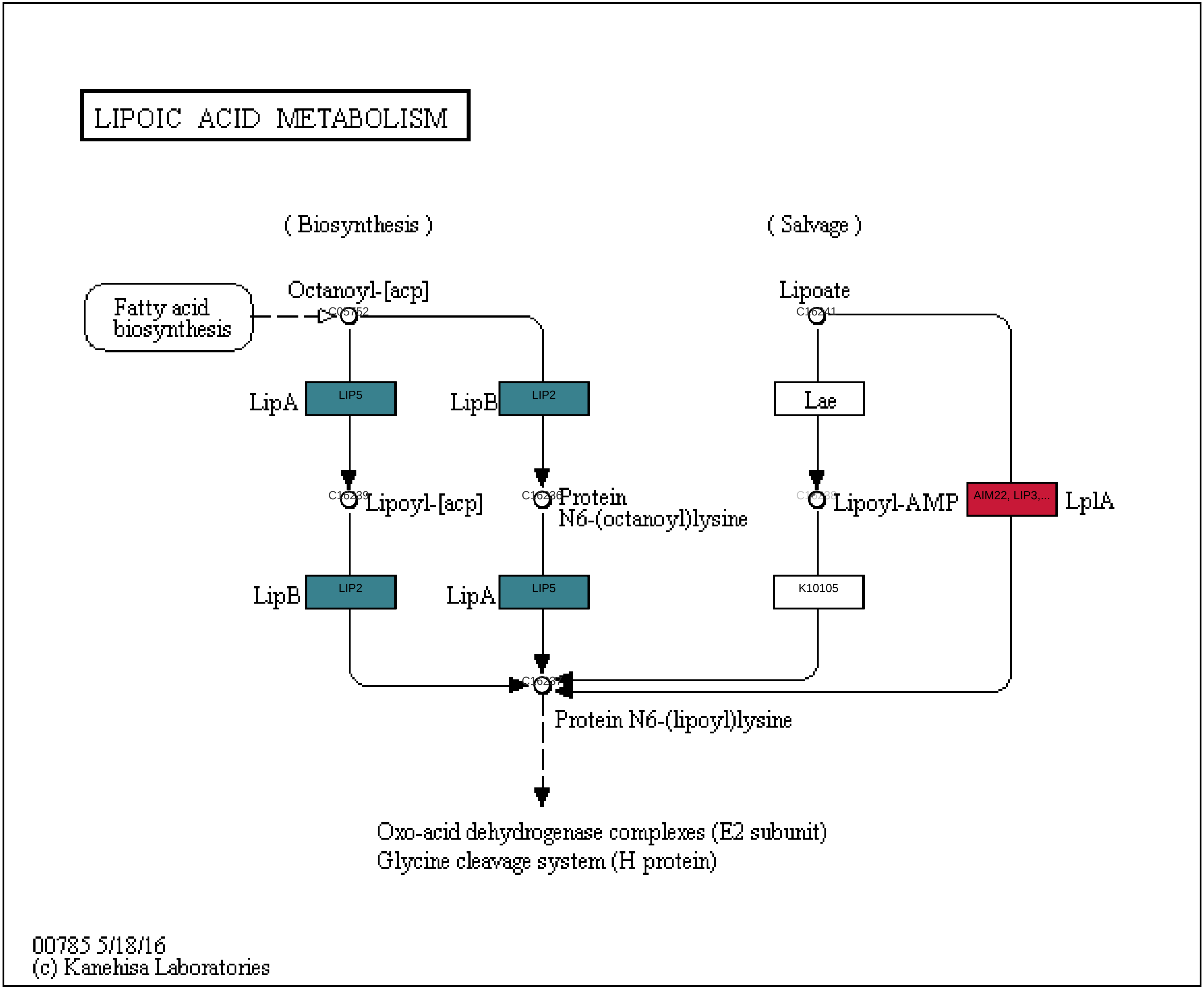
LIPOIC ACID METABOLISM
( Biosynthesis )
( Salvage )
Fatty acid biosynthesis
Octanoyl-[acp]
Lipoate
LipA
LIP5
LipB
LIP2
Lae
C16239
Lipoyl-[acp]
C16236
Protein N6-(octanoyl)lysine
Lipoyl-AMP
AIM22, LIP3,...
LplA
LipB
LIP2
LipA
LIP5
K10105
Protein N6-(lipoyl)lysine
Oxo-acid dehydrogenase complexes (E2 subunit)
Glycine cleavage system (H protein)
00785 5/18/16
(c) Kanehisa Laboratories

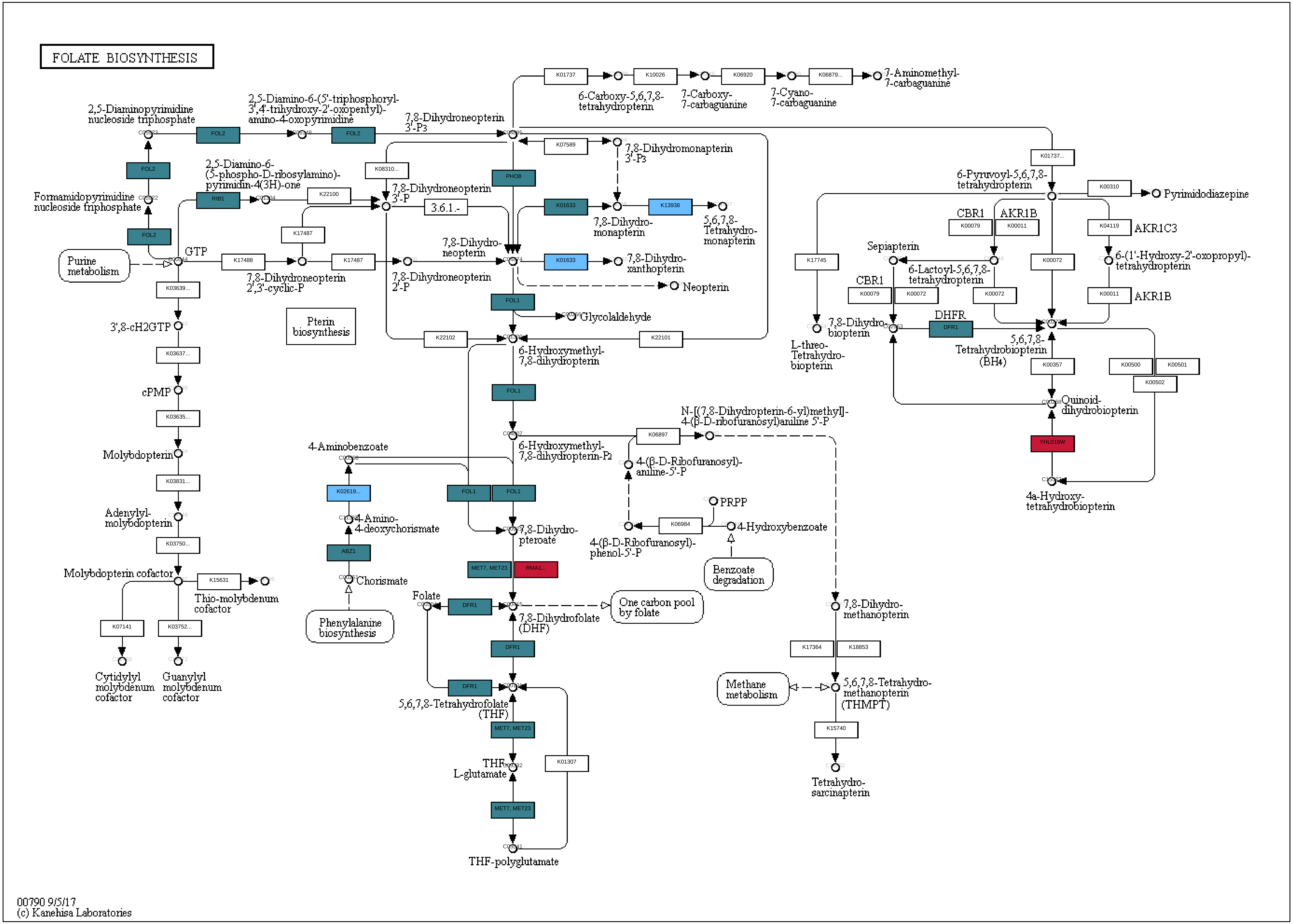
FOLATE BIOSYNTHESIS
2,5-Diaminopyrimidine nucleoside triphosphate
2,5-Diamino-6-(5'-triphosphoryl-3',4'-trihydroxy-2'-oxopentyl)-amino-4-oxopyrimidine
7,8-Dihydroneopterin 3'-P3
FOL2
FOL2
FOL2
FOL2
Formamidopyrimidine nucleoside triphosphate
2,5-Diamino-6-(5-phospho-D-ribosylamino)-pyrimidin-4(3H)-one
RIB1
K22100
K08310...
7,8-Dihydroneopterin 3'-P
3.6.1.-
K17487
Purine metabolism
GTP
K17488
K17487
7,8-Dihydroneopterin 2',3'-cyclic-P
7,8-Dihydroneopterin 2'-P
7,8-Dihydro-neopterin
K03639...
3',8-cH2GTP
K03637...
cPMP
K03635...
Molybdopterin
K03831...
Adenylyl-molybdopterin
K03750...
Molybdopterin cofactor
K15631
Thio-molybdenum cofactor
K07141
K03752...
Cytidylyl molybdenum cofactor
Guanylyl molybdenum cofactor
Pterin biosynthesis
K01737
6-Carboxy-5,6,7,8-tetrahydropterin
K10026
7-Carboxy-7-carbaguanine
K06920
7-Cyano-7-carbaguanine
K06879...
7-Aminomethyl-7-carbaguanine
K07589
7,8-Dihydromonapterin 3'-P3
PHO8
K01633
7,8-Dihydro-monapterin
K13938
5,6,7,8-Tetrahydro-monapterin
K01633
7,8-Dihydro-xanthopterin
Neopterin
FOL1
Glycolaldehyde
K22102
K22101
6-Hydroxymethyl-7,8-dihydropterin
FOL1
6-Hydroxymethyl-7,8-dihydropterin-P2
K06897
N-[(7,8-Dihydropterin-6-yl)methyl]-4-(β-D-ribofuranosyl)aniline 5'-P
4-(β-D-Ribofuranosyl)-aniline-5'-P
PRPP
K06984
4-Hydroxybenzoate
4-(β-D-Ribofuranosyl)-phenol-5'-P
Benzoate degradation
4-Aminobenzoate
K02619...
FOL1
FOL1
4-Amino-4-deoxychorismate
ABZ1
Chorismate
Phenylalanine biosynthesis
7,8-Dihydro-pteroate
MET7, MET23
RMA1...
Folate
DFR1
7,8-Dihydrofolate (DHF)
One carbon pool by folate
DFR1
DFR1
5,6,7,8-Tetrahydrofolate (THF)
MET7, MET23
K01307
THF-L-glutamate
MET7, MET23
THF-polyglutamate
K01737...
6-Pyruvoyl-5,6,7,8-tetrahydropterin
K00310
Pyrimidodiazepine
CBR1
AKR1B
K00079
K00011
K04119
AKR1C3
Sepiapterin
K17745
6-Lactoyl-5,6,7,8-tetrahydropterin
K00072
6-(1'-Hydroxy-2'-oxopropyl)-tetrahydropterin
CBR1
K00079
K00072
K00072
K00011
AKR1B
DHFR
DFR1
7,8-Dihydro-biopterin
L-threo-Tetrahydro-biopterin
5,6,7,8-Tetrahydrobiopterin (BH4)
K00357
K00500
K00501
K00502
Quinoid-dihydrobiopterin
YHL018W
4a-Hydroxy-tetrahydrobiopterin
7,8-Dihydro-methanopterin
K17364
K18853
Methane metabolism
5,6,7,8-Tetrahydro-methanopterin (THMPT)
K15740
Tetrahydro-sarcinapterin
00790 9/5/17
(c) Kanehisa Laboratories

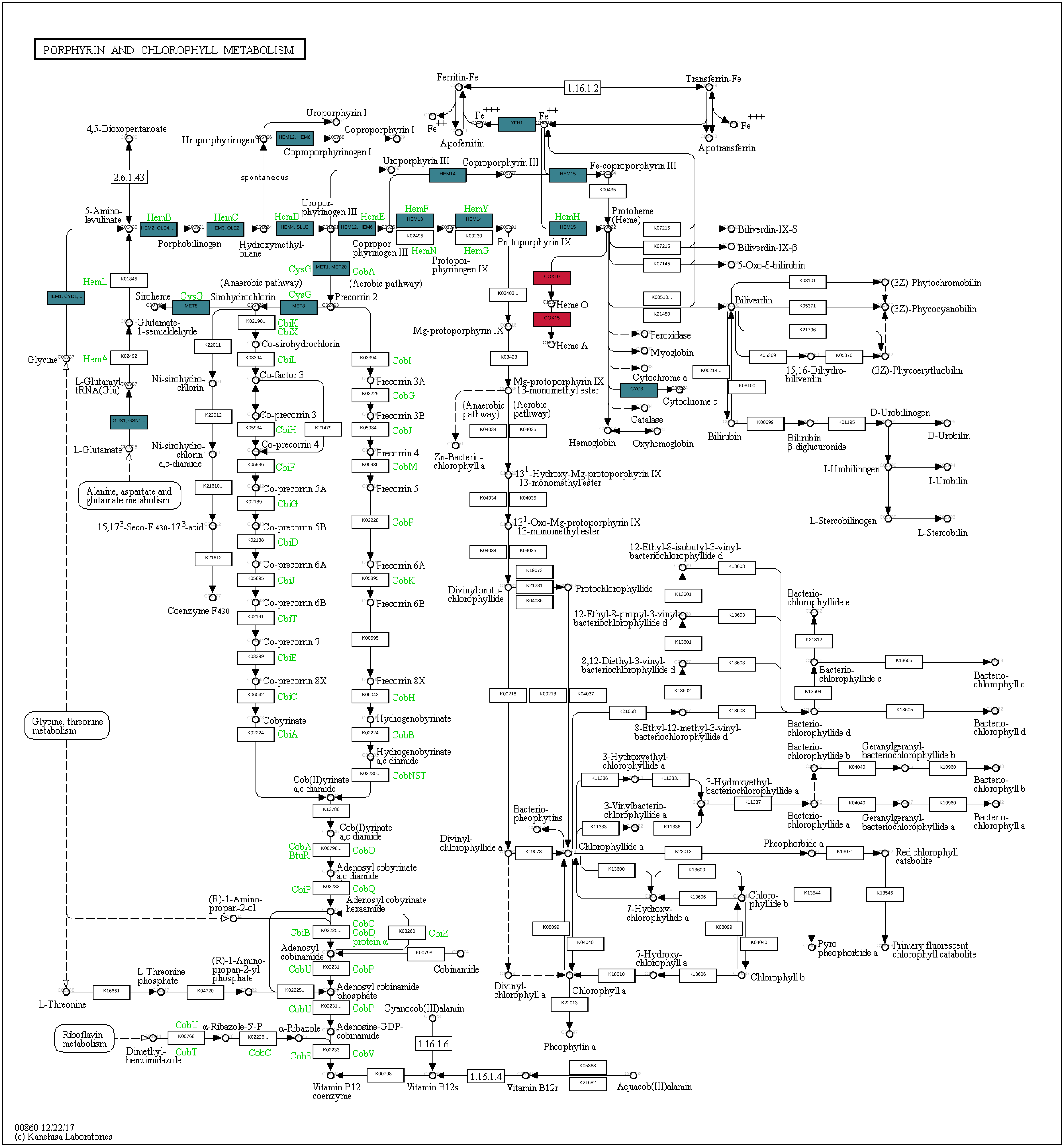
PORPHYRIN AND CHLOROPHYLL METABOLISM
Ferritin-Fe
1.16.1.2
Transferrin-Fe
Fe
Apoferritin
Apotransferrin
YFH1
Uroporphyrin I
Uroporphyrinogen I
Coproporphyrin I
Coproporphyrinogen I
HEM12, HEM6
4,5-Dioxopentanoate
2.6.1.43
spontaneous
Uroporphyrin III
Coproporphyrin III
Fe-coproporphyrin III
HEM14
HEM15
K00435
Protoheme (Heme)
5-Amino-levulinate
HemB
HemC
HemD
HemE
HemF
HemY
HemH
HEM2, OLE4, ...
HEM3, OLE2
HEM4, SLU2
HEM12, HEM6
HEM13
HEM14
HEM15
Porphobilinogen
Hydroxymethyl-bilane
Uropor-phyrinogen III
Copropor-phyrinogen III
HemN
K02495
Protopor-phyrinogen IX
HemG
K00230
Protoporphyrin IX
K07215
Biliverdin-IX-δ
Biliverdin-IX-β
K07145
5-Oxo-δ-bilirubin
HemL
K01845
HEM1, CYD1, ...
CysG
MET1, MET20
CobA
(Anaerobic pathway)
(Aerobic pathway)
Siroheme
MET8
Sirohydrochlorin
Precorrin 2
COX10
Heme O
COX15
Heme A
K03403...
Mg-protoporphyrin IX
K03428
K00510...
K21480
Biliverdin
K08101
(3Z)-Phytochromobilin
K05371
(3Z)-Phycocyanobilin
K21796
Peroxidase
Myoglobin
K05369
K05370
15,16-Dihydro-biliverdin
(3Z)-Phycoerythrobilin
Glutamate-1-semialdehyde
K02190...
CbiK
CbiX
Co-sirohydrochlorin
K22011
K03394...
CbiL
CbiI
HemA
K02492
Glycine
L-Glutamyl-tRNA(Glu)
Ni-sirohydro-chlorin
Co-factor 3
Precorrin 3A
K02229
CobG
Precorrin 3B
K00214...
Cytochrome a
CYC3...
Cytochrome c
K08100
Mg-protoporphyrin IX 13-monomethyl ester
(Anaerobic pathway)
(Aerobic pathway)
K04034
K04035
Zn-Bacterio-chlorophyll a
Catalase
Hemoglobin
Oxyhemoglobin
Bilirubin
K00699
Bilirubin β-diglucuronide
K01195
D-Urobilinogen
D-Urobilin
GUS1, GSN1...
K22012
Co-precorrin 3
K05934...
CbiH
K21479
Co-precorrin 4
CobJ
Precorrin 4
L-Glutamate
Ni-sirohydro-chlorin a,c-diamide
K05936
CbiF
CobM
K21610...
Alanine, aspartate and glutamate metabolism
Co-precorrin 5A
Precorrin 5
13¹-Hydroxy-Mg-protoporphyrin IX 13-monomethyl ester
I-Urobilinogen
I-Urobilin
K02189...
CbiG
K02228
CobF
15,17³-Seco-F 430-17³-acid
Co-precorrin 5B
K02188
CbiD
13¹-Oxo-Mg-protoporphyrin IX 13-monomethyl ester
L-Stercobilinogen
L-Stercobilin
K21612
Co-precorrin 6A
Precorrin 6A
K05895
CbiJ
CobK
12-Ethyl-8-isobutyl-3-vinyl-bacteriochlorophyllide d
K13603
K19073
K21231
K04036
Divinylproto-chlorophyllide
Protochlorophyllide
K13601
Coenzyme F430
Co-precorrin 6B
Precorrin 6B
K02191
CbiT
12-Ethyl-8-propyl-3-vinyl-bacteriochlorophyllide d
Bacterio-chlorophyllide e
K00595
Co-precorrin 7
K03399
CbiE
8,12-Diethyl-3-vinyl-bacteriochlorophyllide d
K21312
K13605
Bacterio-chlorophyllide c
Bacterio-chlorophyll c
Co-precorrin 8X
Precorrin 8X
K06042
CbiC
CobH
K00218
K04037...
K13602
K13604
Glycine, threonine metabolism
Cobyrinate
Hydrogenobyrinate
K02224
CbiA
CobB
K21058
8-Ethyl-12-methyl-3-vinyl-bacteriochlorophyllide d
Bacterio-chlorophyllide d
Bacterio-chlorophyll d
Hydrogenobyrinate a,c diamide
K02230...
CobNST
3-Hydroxyethyl-chlorophyllide a
K11336
K11333...
3-Hydroxyethyl-bacteriochlorophyllide a
Bacterio-chlorophyllide b
Geranylgeranyl-bacteriochlorophyllide b
K04040
K10960
Bacterio-chlorophyll b
Cob(II)yrinate a,c diamide
K13786
Bacterio-pheophytins
3-Vinylbacterio-chlorophyllide a
K11337
Bacterio-chlorophyllide a
Geranylgeranyl-bacteriochlorophyllide a
Bacterio-chlorophyll a
Cob(I)yrinate a,c diamide
CobA
BtuR
K00798...
CobO
Divinyl-chlorophyllide a
Chlorophyllide a
K22013
Pheophorbide a
K13071
Red chlorophyll catabolite
Adenosyl cobyrinate a,c diamide
CbiP
K02232
CobQ
K13600
K13606
Chloro-phyllide b
7-Hydroxy-chlorophyllide a
K13544
K13545
(R)-1-Amino-propan-2-ol
Adenosyl cobyrinate hexaamide
CbiB
K02225...
CobC
CobD
protein α
K08260
CbiZ
K08099
K04040
Pyro-pheophorbide a
Primary fluorescent chlorophyll catabolite
Adenosyl cobinamide
CobU
K02231
CobP
Cobinamide
7-Hydroxy-chlorophyll a
K18010
Chlorophyll b
Divinyl-chlorophyll a
Chlorophyll a
L-Threonine phosphate
(R)-1-Amino-propan-2-yl phosphate
K16651
K04720
Adenosyl cobinamide phosphate
L-Threonine
K02231...
Cyanocob(III)alamin
Riboflavin metabolism
CobU
α-Ribazole-5'-P
α-Ribazole
K00768
K02226...
Adenosine-GDP-cobinamide
1.16.1.6
Pheophytin a
Dimethyl-benzimidazole
CobT
CobC
CobS
K02233
CobV
K00798...
1.16.1.4
K05368
K21682
Vitamin B12 coenzyme
Vitamin B12s
Vitamin B12r
Aquacob(III)alamin
00860 12/22/17
(c) Kanehisa Laboratories

# 9. Metabolism of terpenoids and polyketides

| MAP | PATHWAY |
|---|---|
| **900** | Terpenoid backbone biosynthesis |
| **909** | Sesquiterpenoid and triterpenoid biosynthesis |

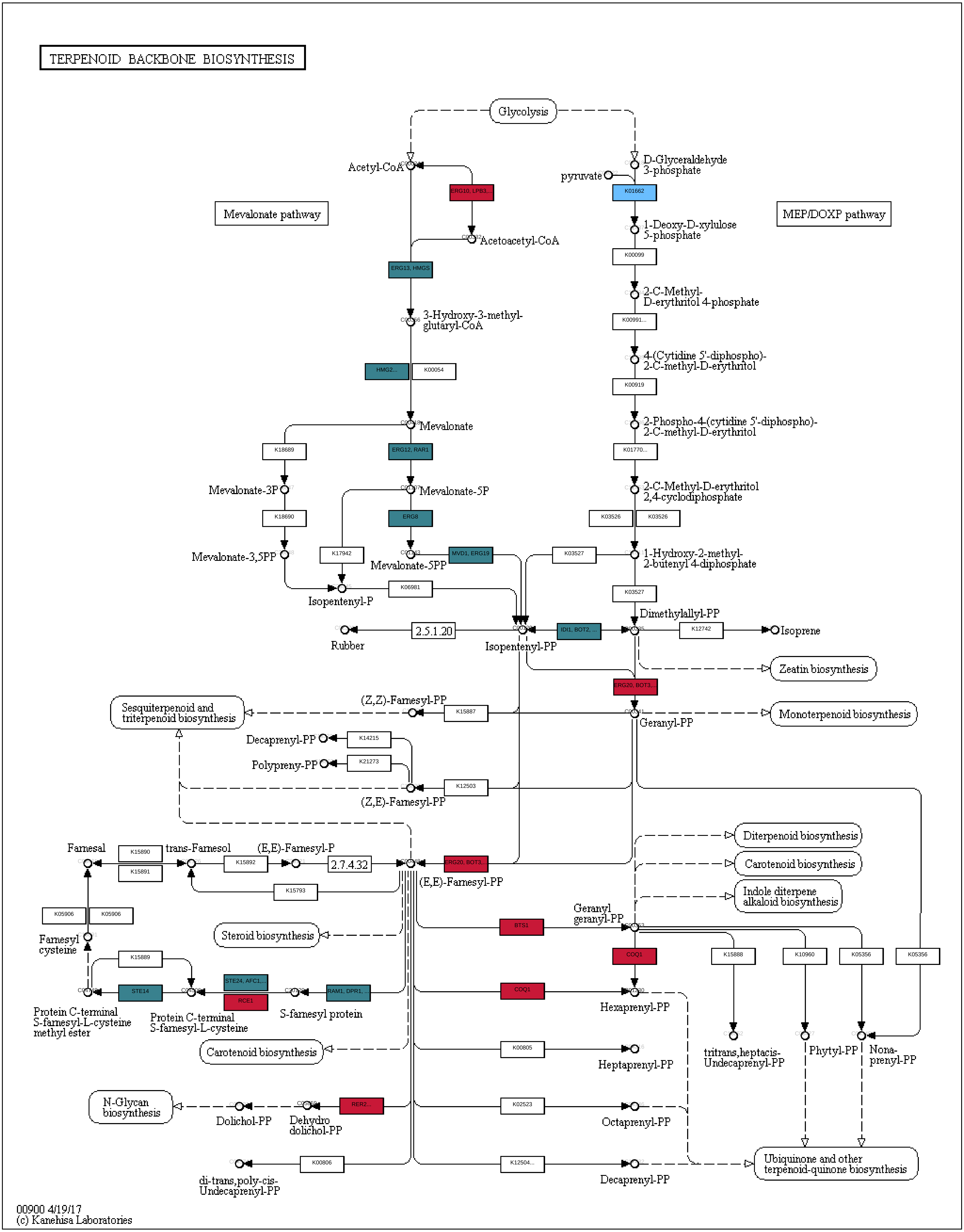

TERPENOID BACKBONE BIOSYNTHESIS
Glycolysis
Acetyl-CoA
ERG10, LPB3...
Acetoacetyl-CoA
ERG13, HMGS
3-Hydroxy-3-methyl-glutaryl-CoA
HMG2...
K00054
Mevalonate
K18689
ERG12, RAR1
Mevalonate-3P
Mevalonate-5P
K18690
ERG8
K17942
Mevalonate-3,5PP
Mevalonate-5PP
MVD1, ERG19
Isopentenyl-P
K06981
Mevalonate pathway
MEP/DOXP pathway
D-Glyceraldehyde 3-phosphate
pyruvate
K01662
1-Deoxy-D-xylulose 5-phosphate
K00099
2-C-Methyl-D-erythritol 4-phosphate
K00991...
4-(Cytidine 5'-diphospho)-2-C-methyl-D-erythritol
K00919
2-Phospho-4-(cytidine 5'-diphospho)-2-C-methyl-D-erythritol
K01770...
2-C-Methyl-D-erythritol 2,4-cyclodiphosphate
K03526
K03526
1-Hydroxy-2-methyl-2-butenyl 4-diphosphate
K03527
K03527
Dimethylallyl-PP
K12742
Isoprene
Rubber
2.5.1.20
Isopentenyl-PP
IDI1, BOT2, ...
Zeatin biosynthesis
ERG20, BOT3,...
Sesquiterpenoid and triterpenoid biosynthesis
(Z,Z)-Farnesyl-PP
K15887
Geranyl-PP
Monoterpenoid biosynthesis
Decaprenyl-PP
K14215
Polypreny-PP
K21273
K12503
(Z,E)-Farnesyl-PP
Diterpenoid biosynthesis
Carotenoid biosynthesis
Indole diterpene alkaloid biosynthesis
Farnesal
K15890
K15891
trans-Farnesol
K15892
(E,E)-Farnesyl-P
2.7.4.32
ERG20, BOT3,...
(E,E)-Farnesyl-PP
K15793
K05906
K05906
Farnesyl cysteine
K15889
Steroid biosynthesis
Geranyl geranyl-PP
BTS1
COQ1
K15888
K10960
K05356
K05356
STE14
STE24, AFC1,...
RCE1
RAM1, DPR1,...
COQ1
Hexaprenyl-PP
Protein C-terminal S-farnesyl-L-cysteine methyl ester
Protein C-terminal S-farnesyl-L-cysteine
S-farnesyl protein
Carotenoid biosynthesis
K00805
Heptaprenyl-PP
tritrans,heptacis-Undecaprenyl-PP
Phytyl-PP
Nona-prenyl-PP
N-Glycan biosynthesis
Dolichol-PP
Dehydro dolichol-PP
RER2...
K02523
Octaprenyl-PP
K00806
K12504...
di-trans,poly-cis-Undecaprenyl-PP
Decaprenyl-PP
Ubiquinone and other terpenoid-quinone biosynthesis
00900 4/19/17
(c) Kanehisa Laboratories

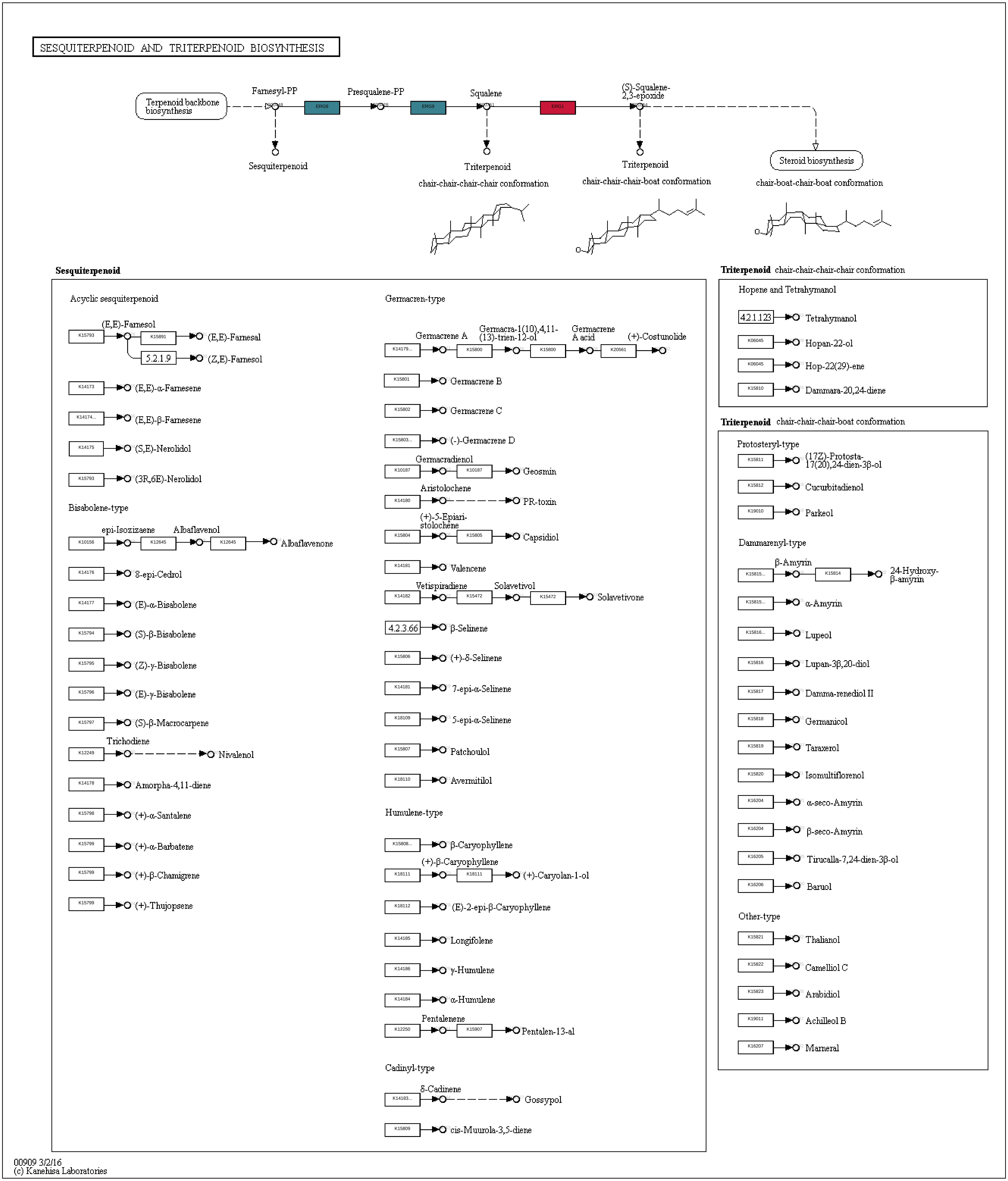

SESQUITERPENOID AND TRITERPENOID BIOSYNTHESIS
Terpenoid backbone biosynthesis
Farnesyl-PP
ERG9
Presqualene-PP
ERG9
Squalene
ERG1
(S)-Squalene-2,3-epoxide
Sesquiterpenoid
Triterpenoid
chair-chair-chair-chair conformation
Triterpenoid
chair-chair-chair-boat conformation
Steroid biosynthesis
chair-boat-chair-boat conformation
Sesquiterpenoid
Acyclic sesquiterpenoid
(E,E)-Farnesol
K15793
K15891
(E,E)-Farnesal
5.2.1.9
(Z,E)-Farnesol
K14173
(E,E)-α-Farnesene
K14174...
(E,E)-β-Farnesene
K14175
(S,E)-Nerolidol
K15793
(3R,6E)-Nerolidol
Bisabolene-type
epi-Isozizaene
Albaflavenol
K10156
K12645
K12645
Albaflavenone
K14176
8-epi-Cedrol
K14177
(E)-α-Bisabolene
K15794
(S)-β-Bisabolene
K15795
(Z)-γ-Bisabolene
K15796
(E)-γ-Bisabolene
K15797
(S)-β-Macrocarpene
Trichodiene
K12249
Nivalenol
K14178
Amorpha-4,11-diene
K15798
(+)-α-Santalene
K15799
(+)-α-Barbatene
K15799
(+)-β-Chamigrene
K15799
(+)-Thujopsene
Germacren-type
Germacrene A
Germacra-1(10),4,11-(13)-trien-12-ol
Germacrene A acid
(+)-Costunolide
K14179...
K15800
K15800
K20561
K15801
Germacrene B
K15802
Germacrene C
K15803...
(-)-Germacrene D
Germacradienol
K10187
K10187
Geosmin
Aristolochene
K14180
PR-toxin
(+)-5-Epiaristolochene
K15804
K15805
Capsidiol
K14181
Valencene
Vetispiradiene
Solavetivol
K14182
K15472
K15472
Solavetivone
4.2.3.66
β-Selinene
K15806
(+)-δ-Selinene
K14181
7-epi-α-Selinene
K18109
5-epi-α-Selinene
K15807
Patchoulol
K18110
Avermitilol
Humulene-type
K15808...
β-Caryophyllene
(+)-β-Caryophyllene
K18111
K18111
(+)-Caryolan-1-ol
K18112
(E)-2-epi-β-Caryophyllene
K14185
Longifolene
K14186
γ-Humulene
K14184
α-Humulene
Pentalenene
K12250
K15907
Pentalen-13-al
Cadinyl-type
δ-Cadinene
K14183...
Gossypol
K15809
cis-Muurola-3,5-diene
Triterpenoid chair-chair-chair-chair conformation
Hopene and Tetrahymanol
4.2.1.123
Tetrahymanol
K06045
Hopan-22-ol
K06045
Hop-22(29)-ene
K15810
Dammara-20,24-diene
Triterpenoid chair-chair-chair-boat conformation
Protosteryl-type
K15811
(17Z)-Protosta-17(20),24-dien-3β-ol
K15812
Cucurbitadienol
K19010
Parkeol
Dammarenyl-type
β-Amyrin
K15815...
K15814
24-Hydroxy-β-amyrin
K15815...
α-Amyrin
K15816...
Lupeol
K15816
Lupan-3β,20-diol
K15817
Damma-renediol II
K15818
Germanicol
K15819
Taraxerol
K15820
Isomultiflorenol
K16204
α-seco-Amyrin
K16204
β-seco-Amyrin
K16205
Tirucalla-7,24-dien-3β-ol
K16206
Baruol
Other-type
K15821
Thalianol
K15822
Camelliol C
K15823
Arabidiol
K19011
Achilleol B
K16207
Marneral
00909 3/2/16
(c) Kanehisa Laboratories

# 10. Biosynthesis of other secondary metabolites

| MAP | PATHWAY |
|---|---|
| **261** | Monobactam biosynthesis |
| **332** | Carbapenem biosynthesis |

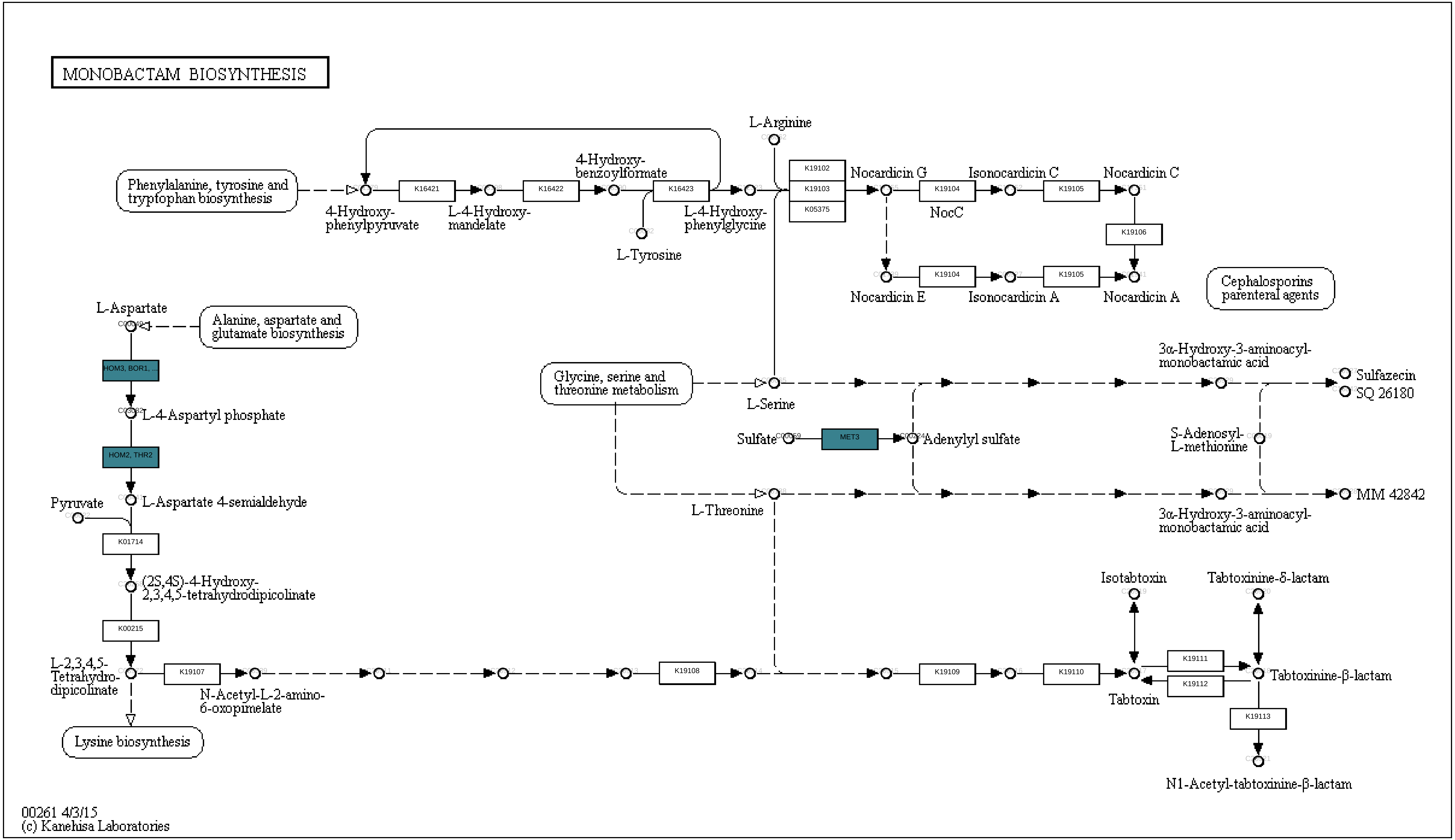

MONOBACTAM BIOSYNTHESIS
Phenylalanine, tyrosine and tryptophan biosynthesis
4-Hydroxy-phenylpyruvate
K16421
L-4-Hydroxy-mandelate
K16422
4-Hydroxy-benzoylformate
K16423
L-Tyrosine
L-4-Hydroxy-phenylglycine
L-Arginine
K19102
K19103
K05375
Nocardicin G
K19104
NocC
Isonocardicin C
K19105
Nocardicin C
K19106
Nocardicin E
K19104
Isonocardicin A
K19105
Nocardicin A
Cephalosporins parenteral agents
L-Aspartate
Alanine, aspartate and glutamate biosynthesis
HOM3, BOR1, ...
L-4-Aspartyl phosphate
HOM2, THR2
L-Aspartate 4-semialdehyde
Pyruvate
K01714
(2S,4S)-4-Hydroxy-2,3,4,5-tetrahydrodipicolinate
K00215
L-2,3,4,5-Tetrahydro-dipicolinate
K19107
N-Acetyl-L-2-amino-6-oxopimelate
Lysine biosynthesis
Glycine, serine and threonine metabolism
L-Serine
Sulfate
MET3
Adenylyl sulfate
L-Threonine
3α-Hydroxy-3-aminoacyl-monobactamic acid
Sulfazecin
SQ 26180
S-Adenosyl-L-methionine
MM 42842
3α-Hydroxy-3-aminoacyl-monobactamic acid
K19108
K19109
K19110
Isotabtoxin
Tabtoxinine-δ-lactam
K19111
K19112
Tabtoxin
Tabtoxinine-β-lactam
K19113
N1-Acetyl-tabtoxinine-β-lactam
00261 4/3/15
(c) Kanehisa Laboratories

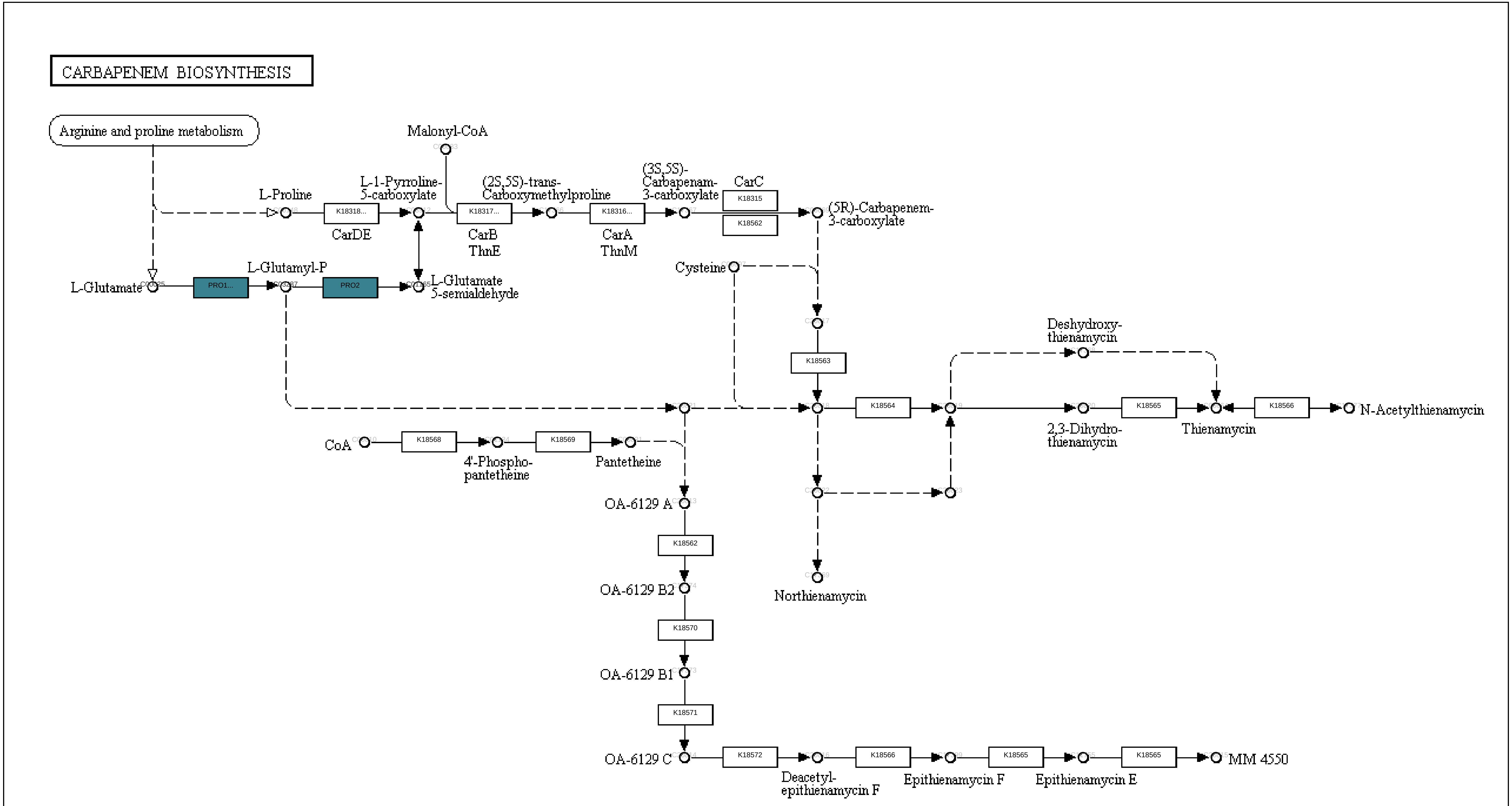


00332 10/1/14

(c) Kanehisa Laboratories

# 11. Xenobiotics biodegradation and metabolism

| MAP | PATHWAY |
|---|---|
| **791** | Atrazine degradation |

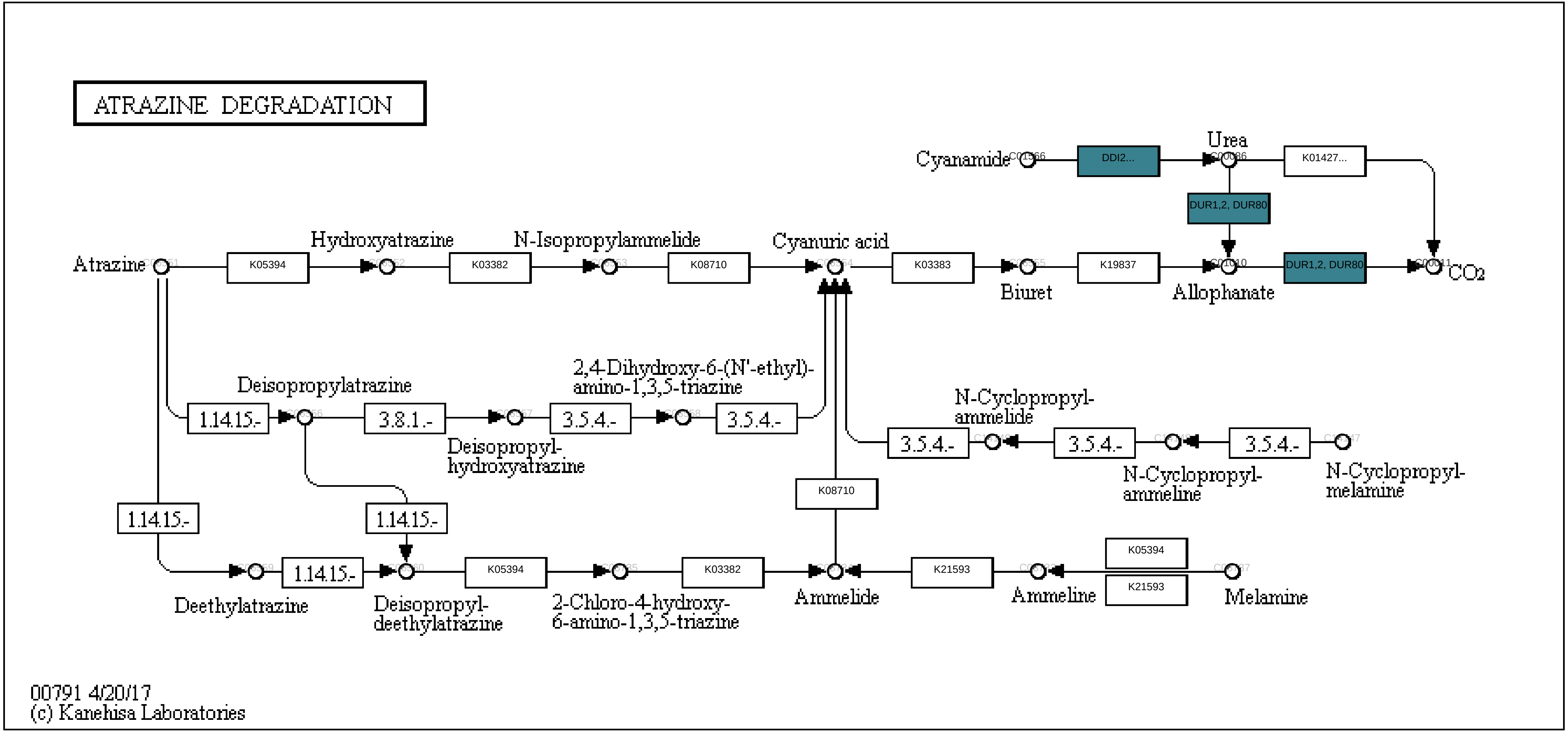
ATRAZINE DEGRADATION
Cyanamide
DDI2...
Urea
K01427...
DUR1,2, DUR80
Atrazine
K05394
Hydroxyatrazine
K03382
N-Isopropylammelide
K08710
Cyanuric acid
K03383
Biuret
K19837
Allophanate
DUR1,2, DUR80
$CO_2$
Deisopropylatrazine
1.14.15.-
3.8.1.-
Deisopropyl-
hydroxyatrazine
2,4-Dihydroxy-6-(N'-ethyl)-
amino-1,3,5-triazine
3.5.4.-
3.5.4.-
N-Cyclopropyl-
ammelide
3.5.4.-
3.5.4.-
3.5.4.-
N-Cyclopropyl-
ammeline
N-Cyclopropyl-
melamine
K08710
1.14.15.-
1.14.15.-
1.14.15.-
Deethylatrazine
Deisopropyl-
deethylatrazine
K05394
2-Chloro-4-hydroxy-
6-amino-1,3,5-triazine
K03382
Ammelide
K21593
Ammeline
K05394
K21593
Melamine
00791 4/20/17
(c) Kanehisa Laboratories

**KEGG pathway analysis**

# *Positive controls*

**Supplementary file 4**

**The following color codes are used:**

| | |
|---|---|
| (teal box) | True positive (TP): element in the reference pathway correctly predicted by Interproscan |
| (white box) | True negative (TN): element in the *not* reference pathway and *not* predicted by Interproscan |
| (red box) | False negative (FN): element in the reference pathway but *not* predicted by Interproscan. |
| (light blue box) | False positive (FP): element in the *not* reference pathway but predicted by Interproscan. Note: could also be a valid new prediction |

**Species:** ***Neurospora crassa***

**Acronym:** **NCR**

**Lifestyle:** **Culturable**

# 1. Carbohydrate metabolism

| MAP | PATHWAY |
|---|---|
| **10** | Glycolysis / Gluconeogenesis |
| **20** | Citrate cycle (TCA cycle) |
| **30** | Pentose phosphate pathway |
| **40** | Pentose and glucuronate interconversions |
| **51** | Fructose and mannose metabolism |
| **52** | Galactose metabolism |
| **53** | Ascorbate and aldarate metabolism |
| **500** | Starch and sucrose metabolism |
| **520** | Amino sugar and nucleotide sugar metabolism |
| **562** | Inositol phosphate metabolism |
| **620** | Pyruvate metabolism |
| **630** | Glyoxylate and dicarboxylate metabolism |
| **640** | Propanoate metabolism |
| **650** | Butanoate metabolism |
| **660** | C5-Branched dibasic acid metabolism |

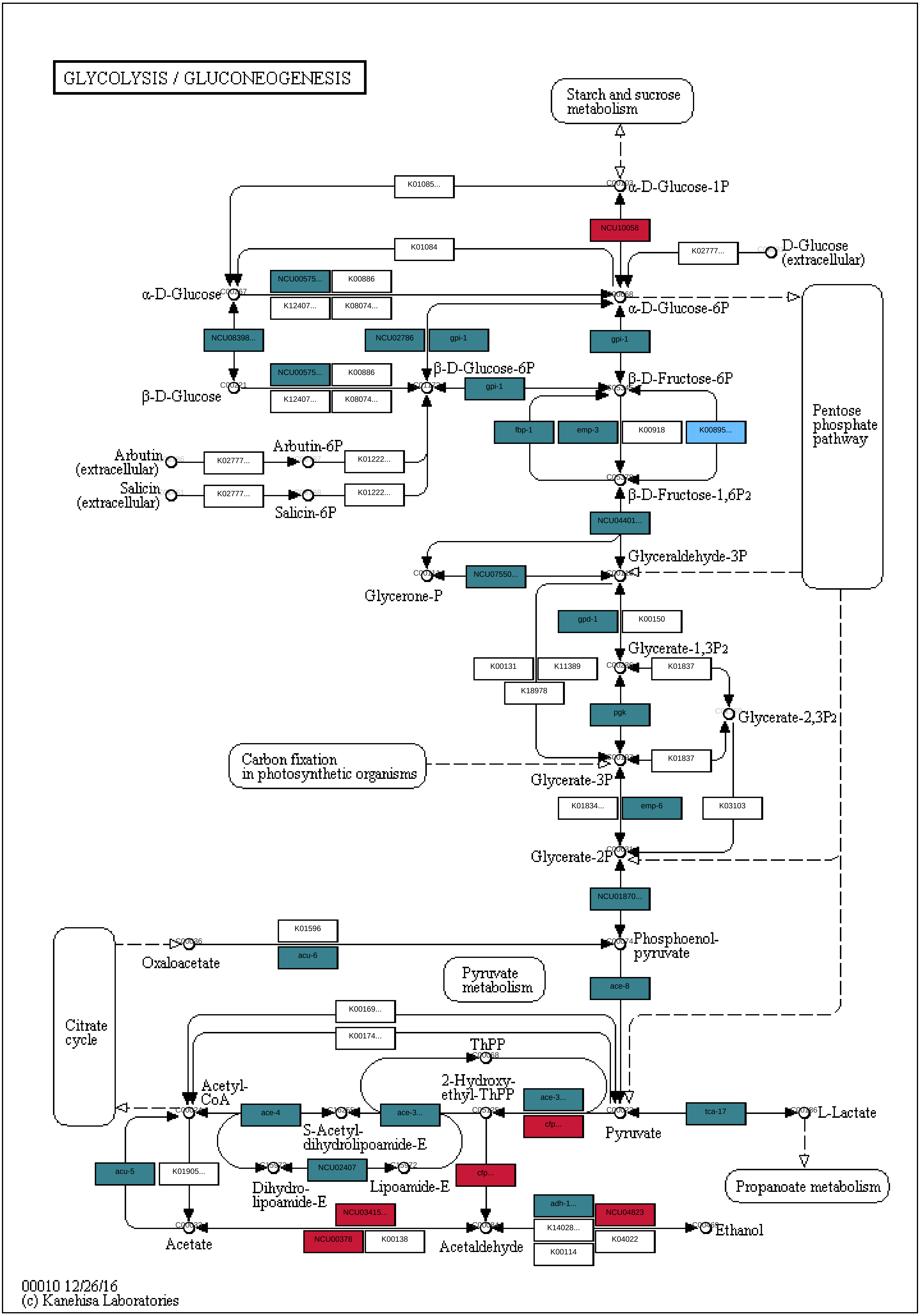

GLYCOLYSIS / GLUCONEOGENESIS
Starch and sucrose metabolism
α-D-Glucose-1P
K01085...
NCU10058
K01084
K02777...
D-Glucose (extracellular)
α-D-Glucose
NCU00575...
K00886
K12407...
K08074...
α-D-Glucose-6P
Pentose phosphate pathway
NCU08398...
NCU02786
gpi-1
β-D-Glucose-6P
β-D-Glucose
β-D-Fructose-6P
fbp-1
emp-3
K00918
K00895...
Arbutin (extracellular)
Arbutin-6P
K01222...
Salicin (extracellular)
Salicin-6P
β-D-Fructose-1,6P2
NCU04401...
Glyceraldehyde-3P
NCU07550...
Glycerone-P
gpd-1
K00150
Glycerate-1,3P2
K00131
K11389
K18978
K01837
pgk
Glycerate-2,3P2
Carbon fixation in photosynthetic organisms
Glycerate-3P
K01834...
emp-6
K03103
Glycerate-2P
NCU01870...
K01596
acu-6
Phosphoenol-pyruvate
Oxaloacetate
Pyruvate metabolism
ace-8
Citrate cycle
K00169...
K00174...
ThPP
2-Hydroxy-ethyl-ThPP
Acetyl-CoA
ace-4
S-Acetyl-dihydrolipoamide-E
ace-3...
cfp...
Pyruvate
tca-17
L-Lactate
Propanoate metabolism
acu-5
K01905...
Dihydro-lipoamide-E
NCU02407
Lipoamide-E
NCU03415...
adh-1...
NCU04823
K14028...
K04022
K00114
NCU00378
K00138
Acetate
Acetaldehyde
Ethanol
00010 12/26/16
(c) Kanehisa Laboratories

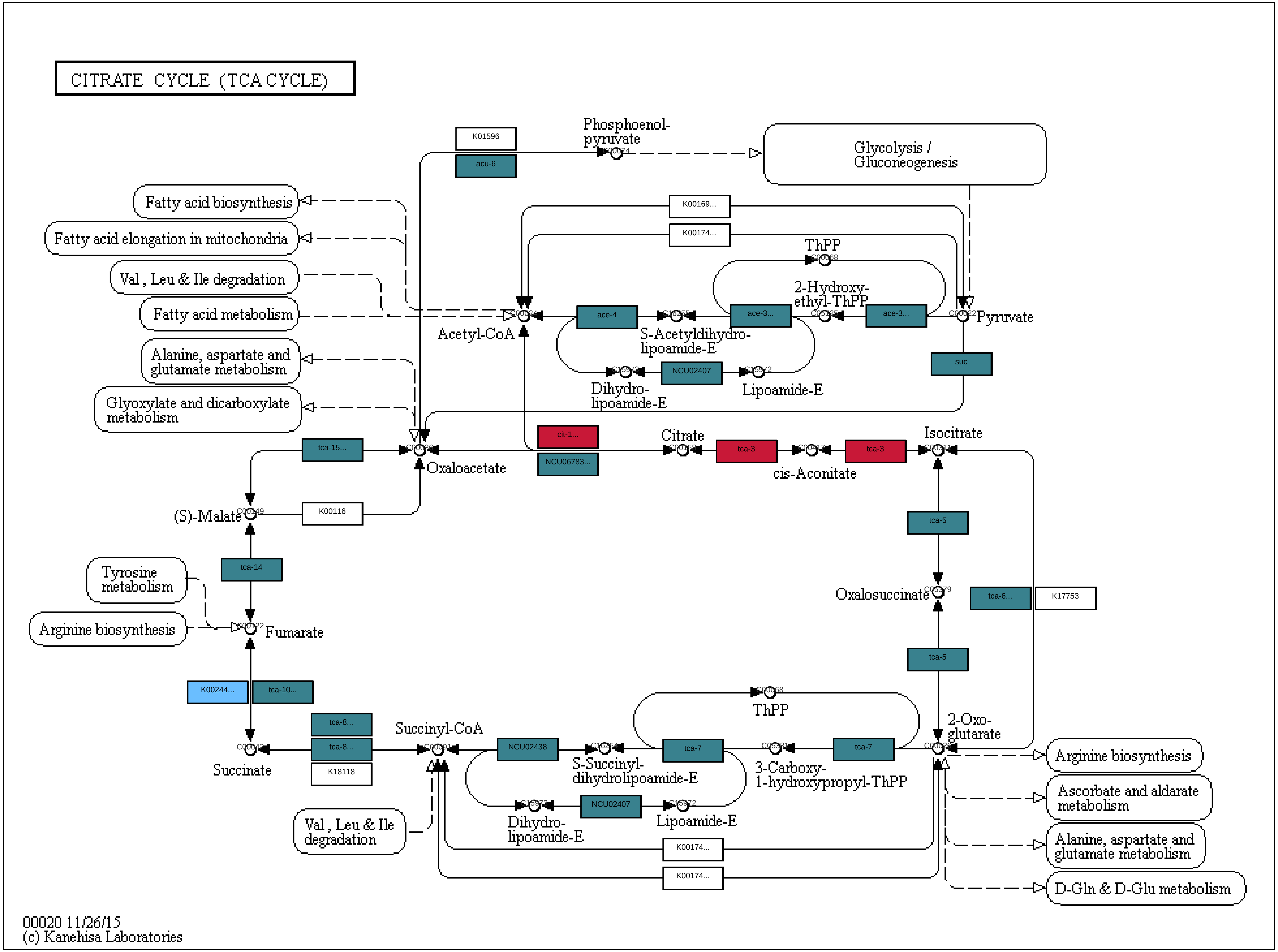

CITRATE CYCLE (TCA CYCLE)
Phosphoenol-pyruvate
Glycolysis / Gluconeogenesis
K01596
acu-6
Fatty acid biosynthesis
Fatty acid elongation in mitochondria
Val, Leu & Ile degradation
Fatty acid metabolism
Alanine, aspartate and glutamate metabolism
Glyoxylate and dicarboxylate metabolism
K00169...
K00174...
ThPP
2-Hydroxy-ethyl-ThPP
ace-4
ace-3...
ace-3...
Acetyl-CoA
S-Acetyldihydro-lipoamide-E
Pyruvate
NCU02407
suc
Dihydro-lipoamide-E
Lipoamide-E
cit-1...
NCU06783...
Citrate
tca-3
tca-3
Isocitrate
cis-Aconitate
tca-15...
Oxaloacetate
(S)-Malate
K00116
tca-14
tca-5
Oxalosuccinate
tca-6...
K17753
tca-5
Tyrosine metabolism
Arginine biosynthesis
Fumarate
K00244...
tca-10...
tca-8...
tca-8...
K18118
Succinate
Succinyl-CoA
NCU02438
S-Succinyl-dihydrolipoamide-E
tca-7
tca-7
3-Carboxy-1-hydroxypropyl-ThPP
2-Oxo-glutarate
NCU02407
Dihydro-lipoamide-E
Lipoamide-E
Val, Leu & Ile degradation
K00174...
K00174...
Arginine biosynthesis
Ascorbate and aldarate metabolism
Alanine, aspartate and glutamate metabolism
D-Gln & D-Glu metabolism
00020 11/26/15
(c) Kanehisa Laboratories

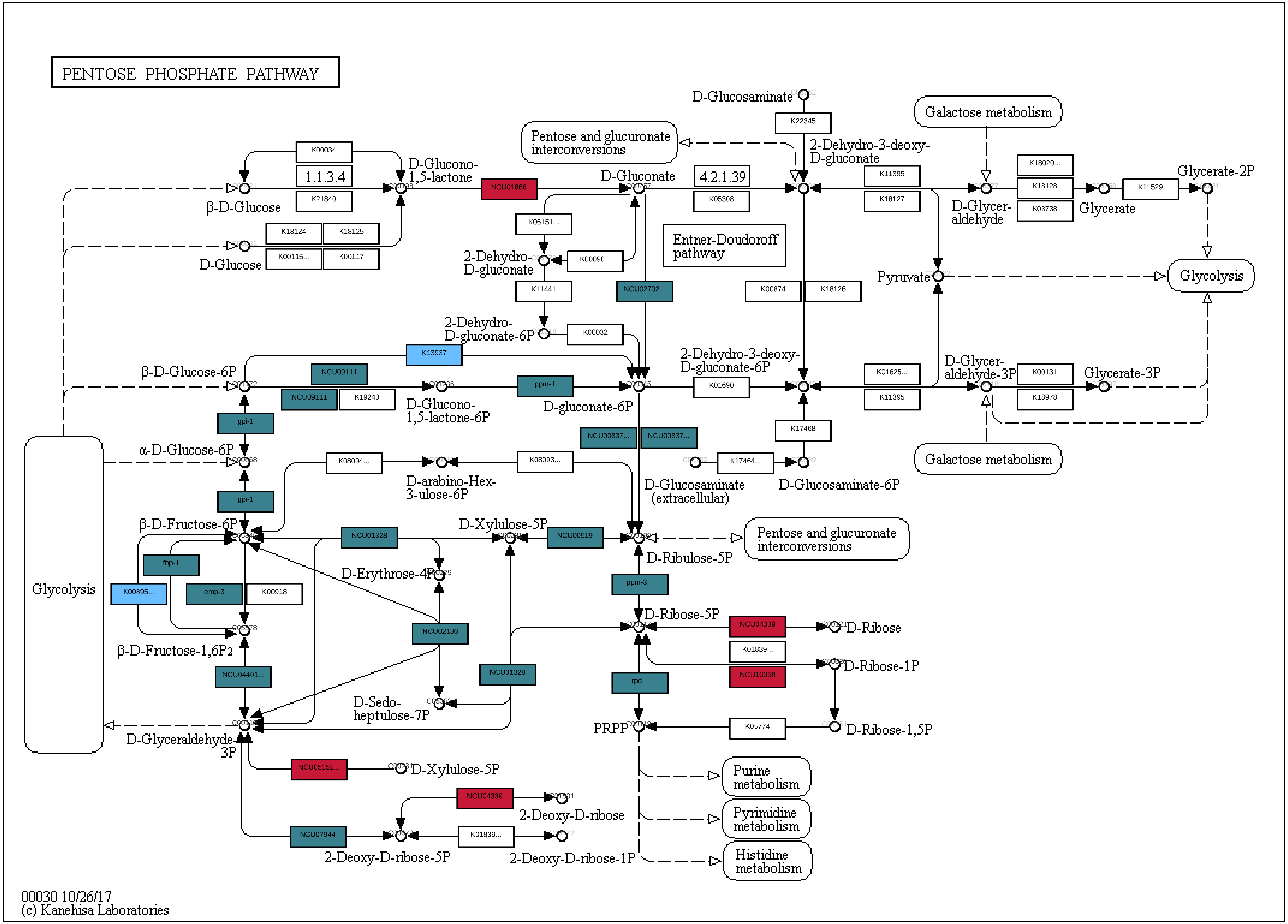
PENTOSE PHOSPHATE PATHWAY
D-Glucosaminate
K22345
Galactose metabolism
Pentose and glucuronate interconversions
2-Dehydro-3-deoxy-D-gluconate
K00034
D-Glucono-1,5-lactone
1.1.3.4
K21840
NCU01866
D-Gluconate
4.2.1.39
K05308
K11395
K18127
K18020...
K18128
K03738
Glycerate
K11529
Glycerate-2P
β-D-Glucose
K18124
K18125
K00115...
K00117
D-Glucose
K06151...
2-Dehydro-D-gluconate
K00090...
Entner-Doudoroff pathway
D-Glyceraldehyde
K11441
NCU02702...
K00874
K18126
Pyruvate
Glycolysis
2-Dehydro-D-gluconate-6P
K00032
K13937
β-D-Glucose-6P
NCU09111
NCU09111
K19243
D-Glucono-1,5-lactone-6P
ppm-1
D-gluconate-6P
2-Dehydro-3-deoxy-D-gluconate-6P
K01690
K01625...
K11395
D-Glyceraldehyde-3P
K00131
K18978
Glycerate-3P
gpi-1
α-D-Glucose-6P
NCU00837...
NCU00837...
K17468
K08094...
D-arabino-Hex-3-ulose-6P
K08093...
K17464...
D-Glucosaminate (extracellular)
D-Glucosamine-6P
Galactose metabolism
gpi-1
β-D-Fructose-6P
NCU01328
D-Xylulose-5P
NCU00519
D-Ribulose-5P
Pentose and glucuronate interconversions
fbp-1
Glycolysis
K00895...
emp-3
K00918
D-Erythrose-4P
ppm-3...
NCU02136
D-Ribose-5P
NCU04339
D-Ribose
K01839...
β-D-Fructose-1,6P2
NCU04401...
NCU01328
NCU10058
D-Ribose-1P
rpd...
D-Sedo-heptulose-7P
PRPP
K05774
D-Ribose-1,5P
D-Glyceraldehyde-3P
NCU05151...
D-Xylulose-5P
Purine metabolism
NCU04339
2-Deoxy-D-ribose
Pyrimidine metabolism
NCU07944
K01839...
2-Deoxy-D-ribose-5P
2-Deoxy-D-ribose-1P
Histidine metabolism
00030 10/26/17
(c) Kanehisa Laboratories

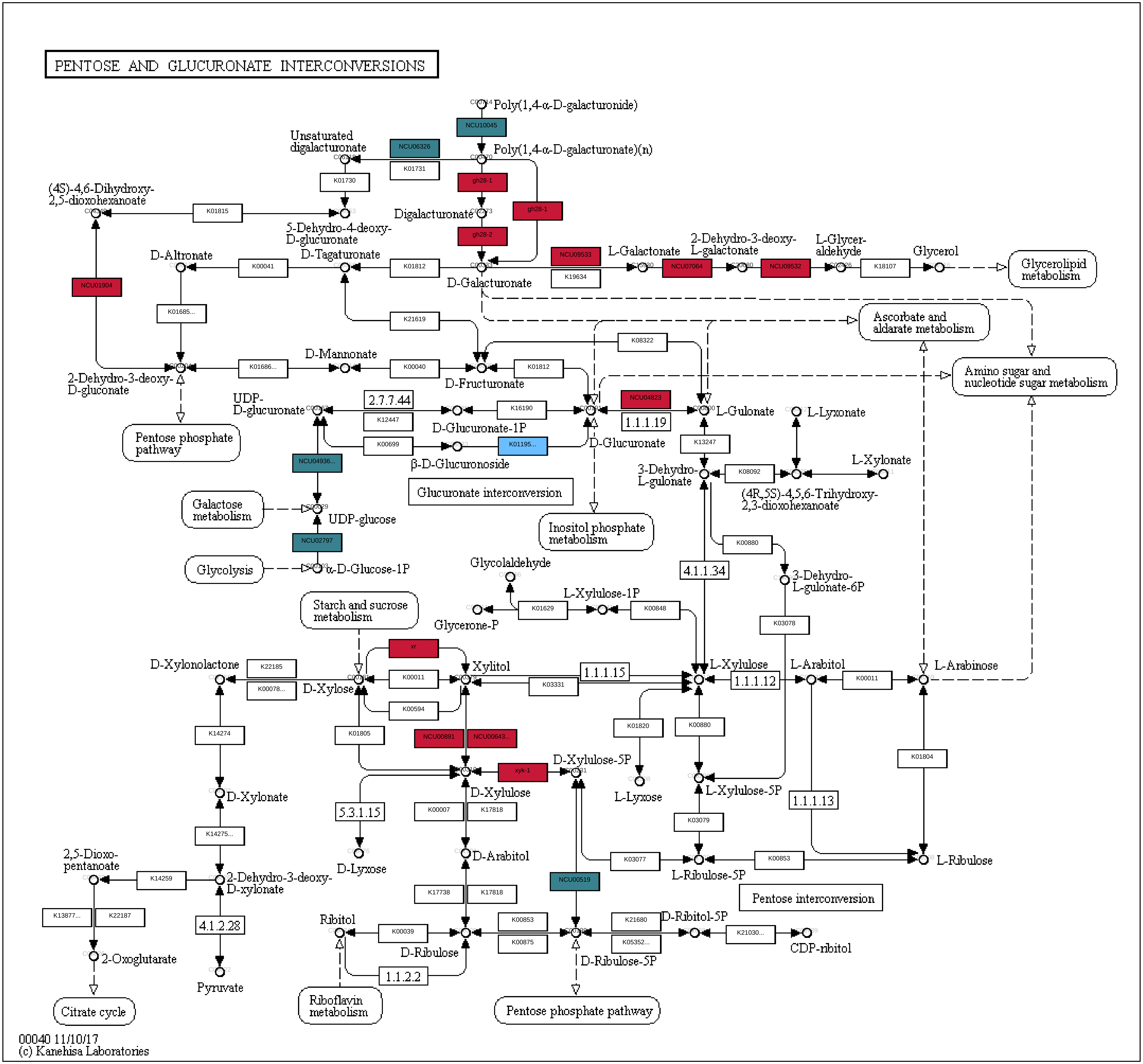
PENTOSE AND GLUCURONATE INTERCONVERSIONS
Poly(1,4-α-D-galacturonide)
NCU10045
Poly(1,4-α-D-galacturonate)(n)
Unsaturated digalacturonate
NCU06326
K01731
K01730
gh28-1
gh28-1
gh28-2
Digalacturonate
(4S)-4,6-Dihydroxy-2,5-dioxohexanoate
K01815
5-Dehydro-4-deoxy-D-glucuronate
D-Tagaturonate
D-Altronate
K00041
K01812
D-Galacturonate
NCU09533
K19634
L-Galactonate
NCU07064
2-Dehydro-3-deoxy-L-galactonate
NCU09532
L-Glyceraldehyde
K18107
Glycerol
Glycerolipid metabolism
NCU01904
K01685...
K21619
Ascorbate and aldarate metabolism
K08322
D-Mannonate
K01686...
K00040
K01812
D-Fructuronate
Amino sugar and nucleotide sugar metabolism
2-Dehydro-3-deoxy-D-gluconate
Pentose phosphate pathway
UDP-D-glucuronate
2.7.7.44
K12447
K16190
D-Glucuronate-1P
NCU04823
1.1.1.19
L-Gulonate
L-Lyxonate
K00699
K01195...
β-D-Glucuronoside
D-Glucuronate
K13247
NCU04936...
3-Dehydro-L-gulonate
K08092
L-Xylonate
(4R,5S)-4,5,6-Trihydroxy-2,3-dioxohexanoate
Glucuronate interconversion
Galactose metabolism
UDP-glucose
Inositol phosphate metabolism
K00880
NCU02797
Glycolysis
α-D-Glucose-1P
Glycolaldehyde
4.1.1.34
3-Dehydro-L-gulonate-6P
L-Xylulose-1P
K01629
K00848
Starch and sucrose metabolism
Glycerone-P
K03078
xr
D-Xylonolactone
K22185
K00078...
K00011
Xylitol
1.1.1.15
L-Xylulose
L-Arabitol
L-Arabinose
D-Xylose
1.1.1.12
K00011
K03331
K00594
K14274
K01805
K01820
K00880
NCU00891
NCU00643...
K01804
D-Xylulose-5P
xyk-1
D-Xylonate
D-Xylulose
L-Lyxose
L-Xylulose-5P
5.3.1.15
K00007
K17818
1.1.1.13
K14275...
D-Lyxose
D-Arabitol
K03079
2,5-Dioxopentanoate
K14259
2-Dehydro-3-deoxy-D-xylonate
K03077
K00853
L-Ribulose
NCU00519
L-Ribulose-5P
Pentose interconversion
K17738
K17818
K13877...
K22187
4.1.2.28
Ribitol
K00039
K00853
K00875
K21680
K05352...
D-Ribitol-5P
K21030...
CDP-ribitol
2-Oxoglutarate
D-Ribulose
D-Ribulose-5P
1.1.2.2
Pyruvate
Citrate cycle
Riboflavin metabolism
Pentose phosphate pathway
00040 11/10/17
(c) Kanehisa Laboratories

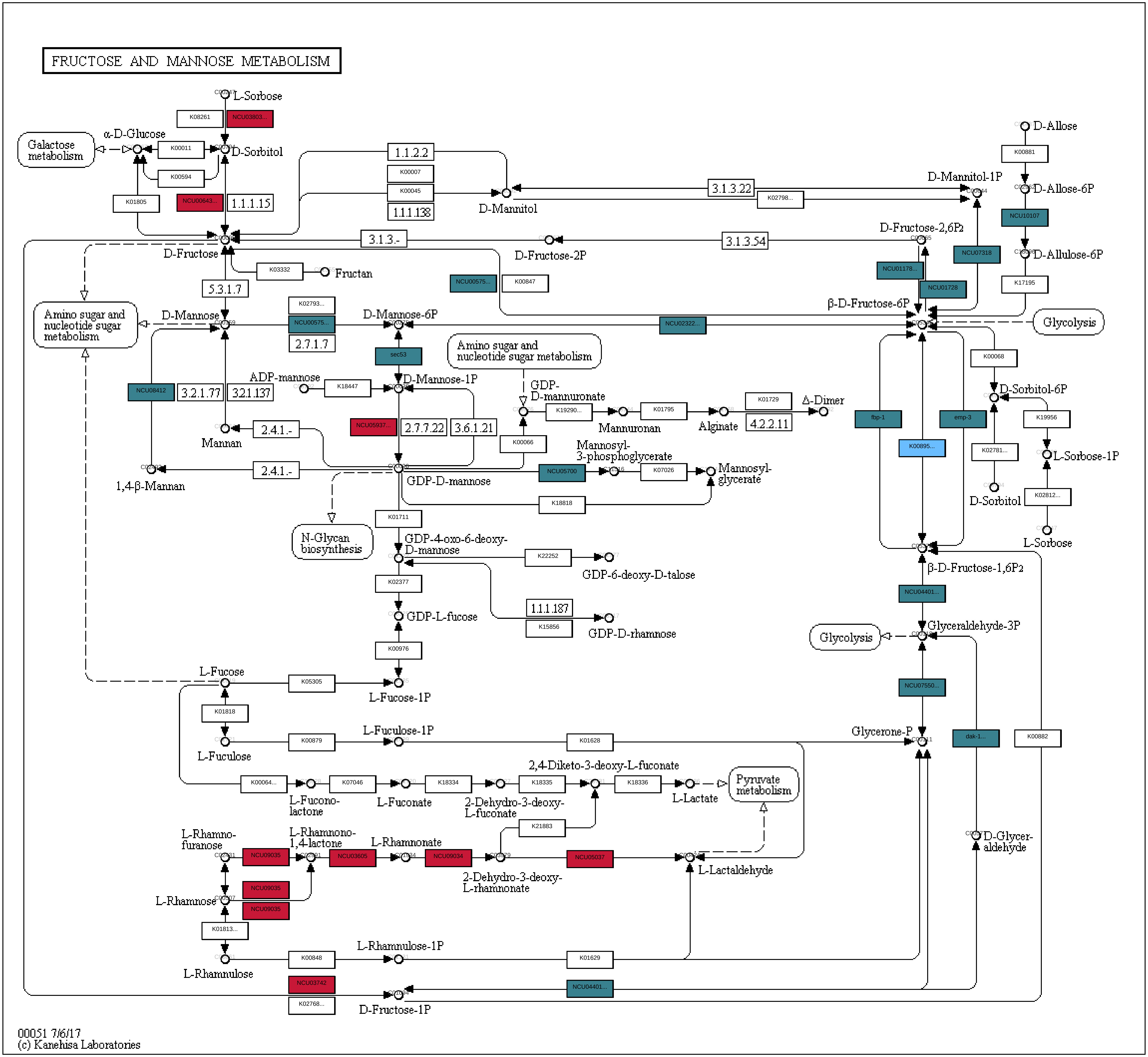
FRUCTOSE AND MANNOSE METABOLISM
L-Sorbose
K08261
NCU03803...
Galactose metabolism
α-D-Glucose
K00011
D-Sorbitol
K00594
K01805
NCU00643...
1.1.1.15
1.1.2.2
K00007
K00045
1.1.1.138
D-Mannitol
3.1.3.22
K02798...
D-Mannitol-1P
D-Allose
K00881
D-Allose-6P
NCU10107
D-Allulose-6P
K17195
3.1.3.-
D-Fructose
D-Fructose-2P
3.1.3.54
D-Fructose-2,6P2
NCU07318
NCU01178...
NCU01728
K03332
Fructan
NCU00575...
K00847
5.3.1.7
Amino sugar and nucleotide sugar metabolism
D-Mannose
K02793...
NCU00575...
2.7.1.7
D-Mannose-6P
NCU02322...
β-D-Fructose-6P
Glycolysis
sec53
Amino sugar and nucleotide sugar metabolism
K00068
NCU08412
3.2.1.77
3.2.1.137
ADP-mannose
K18447
D-Mannose-1P
GDP-D-mannuronate
K19290...
K01795
K01729
Δ-Dimer
Mannuronan
Alginate
4.2.2.11
fbp-1
emp-3
D-Sorbitol-6P
K19956
Mannan
2.4.1.-
NCU05937...
2.7.7.22
3.6.1.21
K00066
K00895...
K02781...
L-Sorbose-1P
Mannosyl-3-phosphoglycerate
NCU05700
K07026
Mannosyl-glycerate
1,4-β-Mannan
2.4.1.-
GDP-D-mannose
K18818
D-Sorbitol
K02812...
N-Glycan biosynthesis
K01711
GDP-4-oxo-6-deoxy-D-mannose
K22252
GDP-6-deoxy-D-talose
L-Sorbose
β-D-Fructose-1,6P2
K02377
NCU04401...
1.1.1.187
K15856
GDP-L-fucose
GDP-D-rhamnose
Glyceraldehyde-3P
Glycolysis
K00976
NCU07550...
L-Fucose
K05305
L-Fucose-1P
K01818
L-Fuculose-1P
Glycerone-P
dak-1...
K00882
K00879
K01628
L-Fuculose
2,4-Diketo-3-deoxy-L-fuconate
K00064...
K07046
K18334
K18335
K18336
Pyruvate metabolism
L-Fucono-lactone
L-Fuconate
2-Dehydro-3-deoxy-L-fuconate
L-Lactate
K21883
L-Rhamno-furanose
L-Rhamnono-1,4-lactone
L-Rhamnonate
NCU09035
NCU03605
NCU09034
NCU05037
D-Glycer-aldehyde
L-Lactaldehyde
2-Dehydro-3-deoxy-L-rhamnonate
NCU09035
L-Rhamnose
NCU09035
K01813...
L-Rhamnulose-1P
K00848
K01629
L-Rhamnulose
NCU03742
NCU04401...
K02768...
D-Fructose-1P
00051 7/6/17
(c) Kanehisa Laboratories

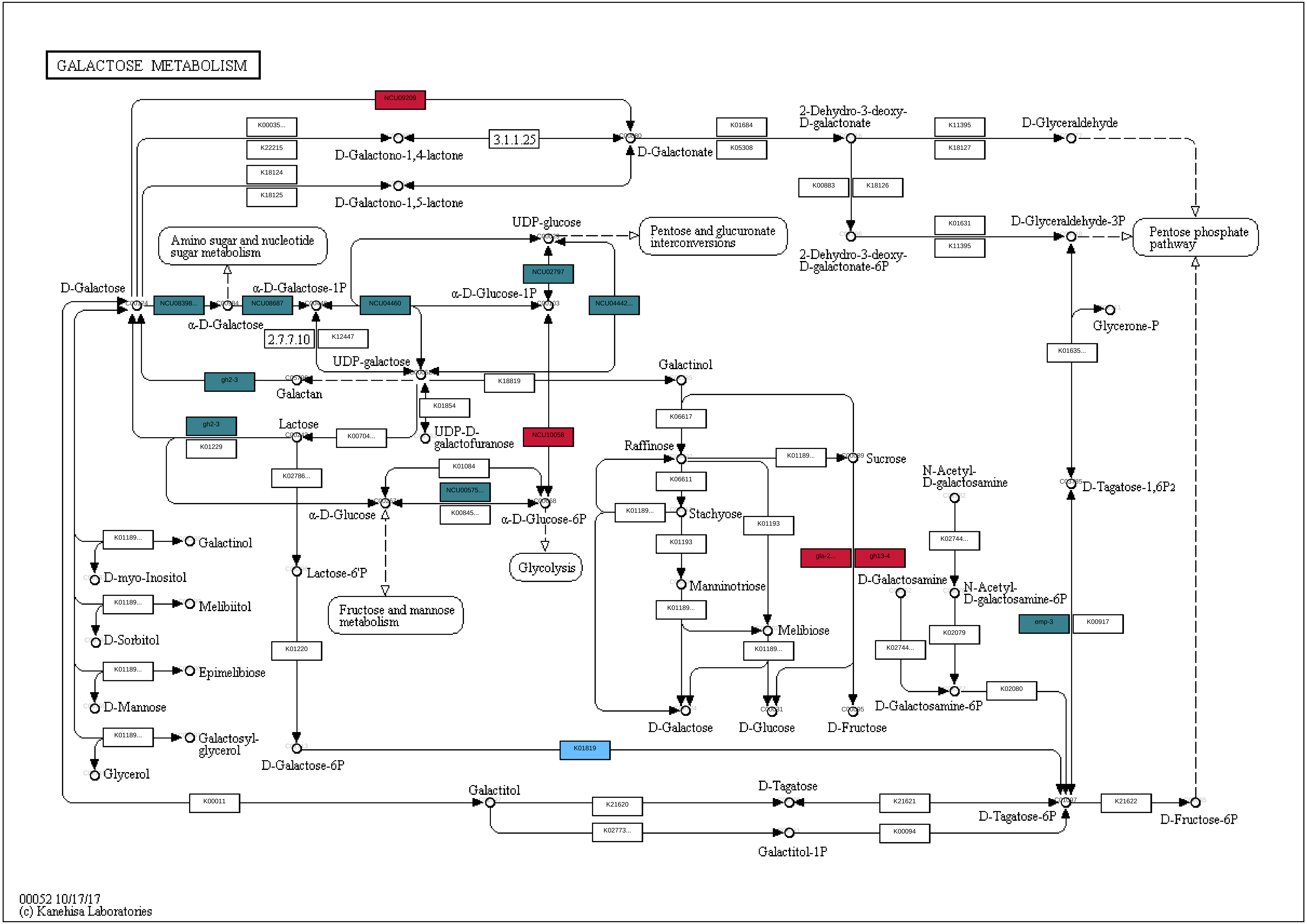
GALACTOSE METABOLISM
NCU09209
K00035...
K22215
K18124
K18125
D-Galactono-1,4-lactone
D-Galactono-1,5-lactone
3.1.1.25
D-Galactonate
K01684
K05308
2-Dehydro-3-deoxy-D-galactonate
K00883
K18126
2-Dehydro-3-deoxy-D-galactonate-6P
K11395
K18127
D-Glyceraldehyde
K01631
K11395
D-Glyceraldehyde-3P
Pentose phosphate pathway
Amino sugar and nucleotide sugar metabolism
UDP-glucose
Pentose and glucuronate interconversions
NCU02797
D-Galactose
NCU08398...
NCU08687
α-D-Galactose-1P
NCU04460
α-D-Glucose-1P
NCU04442...
α-D-Galactose
2.7.7.10
K12447
UDP-galactose
gh2-3
Galactan
K18819
Galactinol
K01854
UDP-D-galactofuranose
NCU10058
Lactose
K00704...
K01229
K02786...
K01084
NCU00575...
K00845...
α-D-Glucose
α-D-Glucose-6P
Glycolysis
K01189...
D-myo-Inositol
Melibiitol
D-Sorbitol
Epimelibiose
D-Mannose
Galactosyl-glycerol
Glycerol
Lactose-6P
Fructose and mannose metabolism
K01220
D-Galactose-6P
K01819
K00011
Galactitol
K21620
K02773...
D-Tagatose
Galactitol-1P
K21621
K00094
D-Tagatose-6P
K21622
D-Fructose-6P
K06617
Raffinose
Sucrose
K06611
Stachyose
K01193
Manninotriose
Melibiose
D-Galactose
D-Glucose
D-Fructose
gla-2...
gh13-4
N-Acetyl-D-galactosamine
K02744...
D-Galactosamine
N-Acetyl-D-galactosamine-6P
K02079
D-Galactosamine-6P
K02080
emp-3
K00917
Glycerone-P
K01635...
D-Tagatose-1,6P2
00052 10/17/17
(c) Kanehisa Laboratories

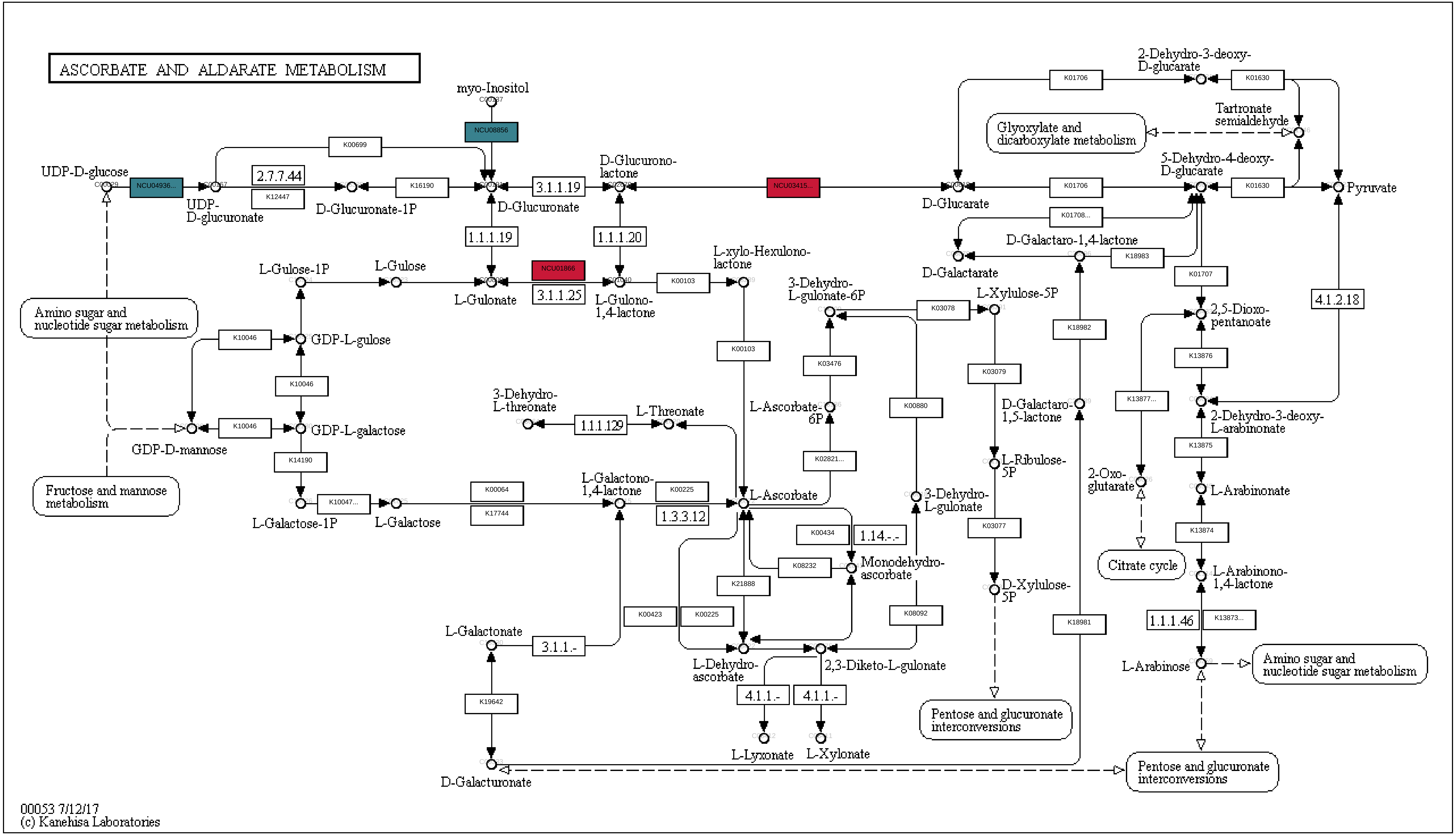
ASCORBATE AND ALDARATE METABOLISM
myo-Inositol
NCU08856
K00699
UDP-D-glucose
NCU04936...
2.7.7.44
K12447
UDP-
D-glucuronate
D-Glucuronate-1P
K16190
D-Glucuronate
3.1.1.19
D-Glucurono-
lactone
NCU03415...
D-Glucarate
K01706
K01630
2-Dehydro-3-deoxy-
D-glucarate
K01706
K01630
Tartronate
semialdehyde
Glyoxylate and
dicarboxylate metabolism
5-Dehydro-4-deoxy-
D-glucarate
Pyruvate
K01708...
D-Galactaro-1,4-lactone
K18983
D-Galactarate
K01707
4.1.2.18
1.1.1.19
1.1.1.20
L-Gulose-1P
L-Gulose
NCU01866
3.1.1.25
L-Gulonate
L-Gulono-
1,4-lactone
K00103
L-xylo-Hexulono-
lactone
3-Dehydro-
L-gulonate-6P
K03078
L-Xylulose-5P
2,5-Dioxo-
pentanoate
Amino sugar and
nucleotide sugar metabolism
K10046
GDP-L-gulose
K10046
K00103
K03476
K18982
K13876
K03079
K00880
K13877...
3-Dehydro-
L-threonate
1.1.1.129
L-Threonate
L-Ascorbate-
6P
D-Galactaro-
1,5-lactone
2-Dehydro-3-deoxy-
L-arabinonate
GDP-D-mannose
K10046
GDP-L-galactose
K14190
K02821...
K13875
L-Ribulose-
5P
2-Oxo-
glutarate
L-Galactono-
1,4-lactone
K00064
K17744
K00225
1.3.3.12
L-Ascorbate
3-Dehydro-
L-gulonate
L-Arabinonate
Fructose and mannose
metabolism
K10047...
L-Galactose-1P
L-Galactose
K00434
1.14.-.-
K03077
K13874
Citrate cycle
K08232
Monodehydro-
ascorbate
K21888
D-Xylulose-
5P
L-Arabinono-
1,4-lactone
K00423
K00225
K08092
K18981
1.1.1.46
K13873...
L-Galactonate
3.1.1.-
L-Dehydro-
ascorbate
2,3-Diketo-L-gulonate
L-Arabinose
Amino sugar and
nucleotide sugar metabolism
K19642
4.1.1.-
4.1.1.-
Pentose and glucuronate
interconversions
L-Lyxonate
L-Xylonate
D-Galacturonate
Pentose and glucuronate
interconversions
00053 7/12/17
(c) Kanehisa Laboratories

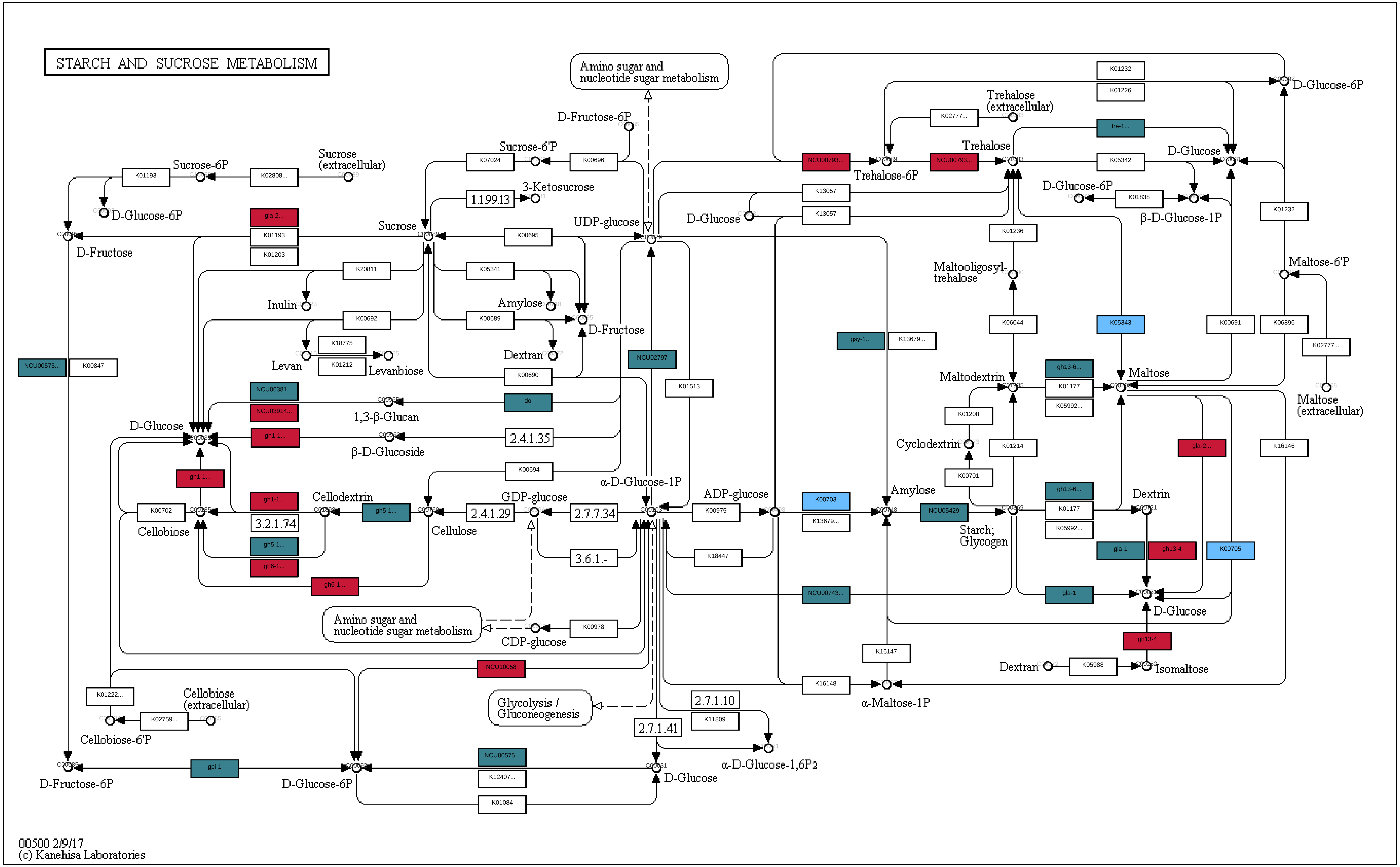
STARCH AND SUCROSE METABOLISM
Amino sugar and nucleotide sugar metabolism
D-Fructose-6P
Sucrose-6'P
K07024
K00696
Sucrose-6P
Sucrose (extracellular)
K01193
K02808...
D-Glucose-6P
1.1.99.13
3-Ketosucrose
gla-2...
K01193
K01203
D-Fructose
Sucrose
K00695
UDP-glucose
D-Glucose
K13057
K13057
K20811
K05341
Inulin
Amylose
K00692
K00689
D-Fructose
K18775
Levan
K01212
Levanbiose
Dextran
K00690
NCU00575...
K00847
NCU06381...
do
NCU03914...
1,3-β-Glucan
D-Glucose
ghl-1...
2.4.1.35
β-D-Glucoside
ghl-1...
K00694
ghl-1...
Cellodextrin
GDP-glucose
α-D-Glucose-1P
ADP-glucose
K00702
3.2.1.74
gh5-1...
2.4.1.29
2.7.7.34
K00975
K00703
Cellobiose
gh5-1...
Cellulose
K13679...
gh6-1...
3.6.1.-
K18447
gh6-1...
Amino sugar and nucleotide sugar metabolism
K00978
CDP-glucose
NCU10058
K01222...
Cellobiose (extracellular)
K02759...
Glycolysis / Gluconeogenesis
2.7.1.10
K11809
2.7.1.41
Cellobiose-6'P
α-D-Glucose-1,6P2
gpi-1
NCU00575...
K12407...
K01084
D-Fructose-6P
D-Glucose-6P
D-Glucose
NCU02797
K01513
K01232
K01226
D-Glucose-6P
Trehalose (extracellular)
K02777...
tre-1...
Trehalose
D-Glucose
NCU00793...
NCU00793...
K05342
Trehalose-6P
D-Glucose-6P
K01838
β-D-Glucose-1P
K01232
K01236
Maltooligosyl-trehalose
Maltose-6'P
K06044
K05343
K00691
K06896
gsy-1...
K13679...
K02777...
Maltodextrin
ghl3-6...
K01177
K05992...
Maltose
Maltose (extracellular)
K01208
Cyclodextrin
K01214
gla-2...
K16146
K00701
Amylose
ghl3-6...
Dextrin
NCU05429
K01177
K05992...
Starch; Glycogen
gla-1
ghl3-4
K00705
NCU00743...
gla-1
D-Glucose
ghl3-4
K16147
Dextran
K05988
Isomaltose
K16148
α-Maltose-1P
00500 2/9/17
(c) Kanehisa Laboratories

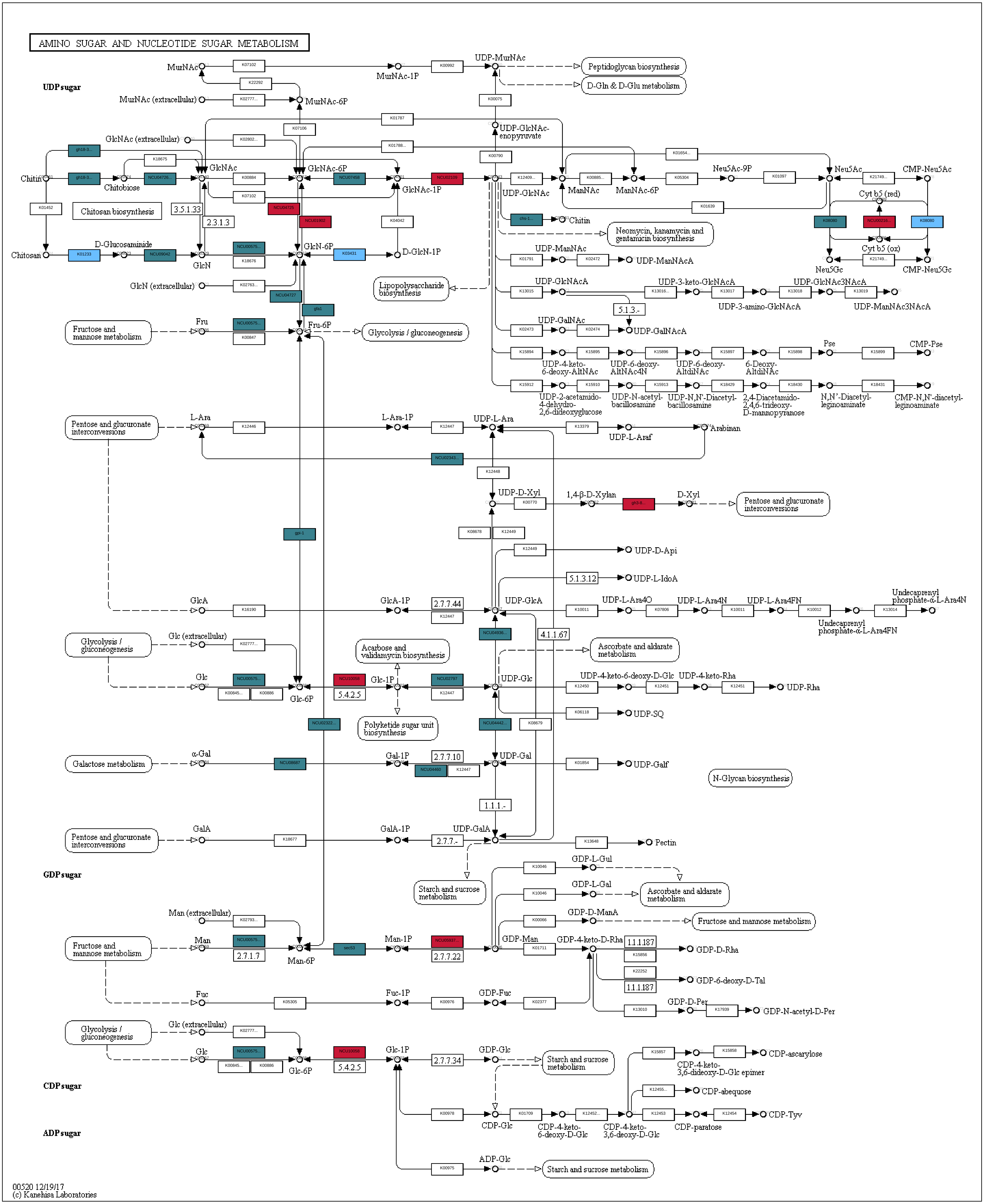

AMINO SUGAR AND NUCLEOTIDE SUGAR METABOLISM
UDP sugar
MurNAc
MurNAc-1P
UDP-MurNAc
Peptidoglycan biosynthesis
D-Gln & D-Glu metabolism
MurNAc (extracellular)
MurNAc-6P
UDP-GlcNAc-enopyruvate
GlcNAc (extracellular)
Chitin
Chitobiose
GlcNAc
GlcNAc-6P
GlcNAc-1P
UDP-GlcNAc
ManNAc
ManNAc-6P
Neu5Ac-9P
Neu5Ac
CMP-Neu5Ac
Cyt b5 (red)
Cyt b5 (ox)
Neu5Gc
CMP-Neu5Gc
Chitosan biosynthesis
3.5.1.33
2.3.1.3
Chitin
Neomycin, kanamycin and gentamicin biosynthesis
Chitosan
D-Glucosaminide
GlcN
GlcN-6P
D-GlcN-1P
UDP-ManNAc
UDP-ManNAcA
GlcN (extracellular)
Lipopolysaccharide biosynthesis
UDP-GlcNAcA
UDP-3-keto-GlcNAcA
UDP-GlcNAc3NAcA
UDP-3-amino-GlcNAcA
UDP-ManNAc3NAcA
5.1.3.-
UDP-GalNAc
UDP-GalNAcA
Fructose and mannose metabolism
Fru
Fru-6P
Glycolysis / gluconeogenesis
Pse
CMP-Pse
UDP-4-keto-6-deoxy-AltNAc
UDP-6-deoxy-AltNAc4N
UDP-6-deoxy-AltdiNAc
6-Deoxy-AltdiNAc
UDP-2-acetamido-4-dehydro-2,6-dideoxyglucose
UDP-N-acetyl-bacillosamine
UDP-N,N'-Diacetyl-bacillosamine
2,4-Diacetamido-2,4,6-trideoxy-D-mannopyranose
N,N'-Diacetyl-legionaminate
CMP-N,N'-diacetyl-legionaminate
Pentose and glucuronate interconversions
L-Ara
L-Ara-1P
UDP-L-Ara
UDP-L-Araf
Arabinan
UDP-D-Xyl
1,4-β-D-Xylan
D-Xyl
Pentose and glucuronate interconversions
UDP-D-Api
5.1.3.12
UDP-L-IdoA
GlcA
GlcA-1P
2.7.7.44
UDP-GlcA
UDP-L-Ara4O
UDP-L-Ara4N
UDP-L-Ara4FN
Undecaprenyl phosphate-α-L-Ara4N
Undecaprenyl phosphate-α-L-Ara4FN
4.1.1.67
Glycolysis / gluconeogenesis
Glc (extracellular)
Acarbose and validamycin biosynthesis
Ascorbate and aldarate metabolism
Glc
Glc-6P
Glc-1P
5.4.2.5
UDP-Glc
UDP-4-keto-6-deoxy-D-Glc
UDP-4-keto-Rha
UDP-Rha
UDP-SQ
Polyketide sugar unit biosynthesis
Galactose metabolism
α-Gal
Gal-1P
2.7.7.10
UDP-Gal
UDP-Galf
N-Glycan biosynthesis
1.1.1.-
Pentose and glucuronate interconversions
GalA
GalA-1P
2.7.7.-
UDP-GalA
Pectin
GDP sugar
Starch and sucrose metabolism
GDP-L-Gul
GDP-L-Gal
Ascorbate and aldarate metabolism
Man (extracellular)
GDP-D-ManA
Fructose and mannose metabolism
Fructose and mannose metabolism
Man
Man-6P
2.7.1.7
Man-1P
2.7.7.22
GDP-Man
GDP-4-keto-D-Rha
1.1.1.187
GDP-D-Rha
GDP-6-deoxy-D-Tal
1.1.1.187
Fuc
Fuc-1P
GDP-Fuc
GDP-D-Per
GDP-N-acetyl-D-Per
Glycolysis / gluconeogenesis
Glc (extracellular)
Glc
Glc-6P
Glc-1P
5.4.2.5
2.7.7.34
GDP-Glc
Starch and sucrose metabolism
CDP-ascarylose
CDP-4-keto-3,6-dideoxy-D-Glc epimer
CDP sugar
CDP-abequose
CDP-Glc
CDP-4-keto-6-deoxy-D-Glc
CDP-4-keto-3,6-deoxy-D-Glc
CDP-paratose
CDP-Tyv
ADP sugar
ADP-Glc
Starch and sucrose metabolism
00520 12/19/17
(c) Kanehisa Laboratories

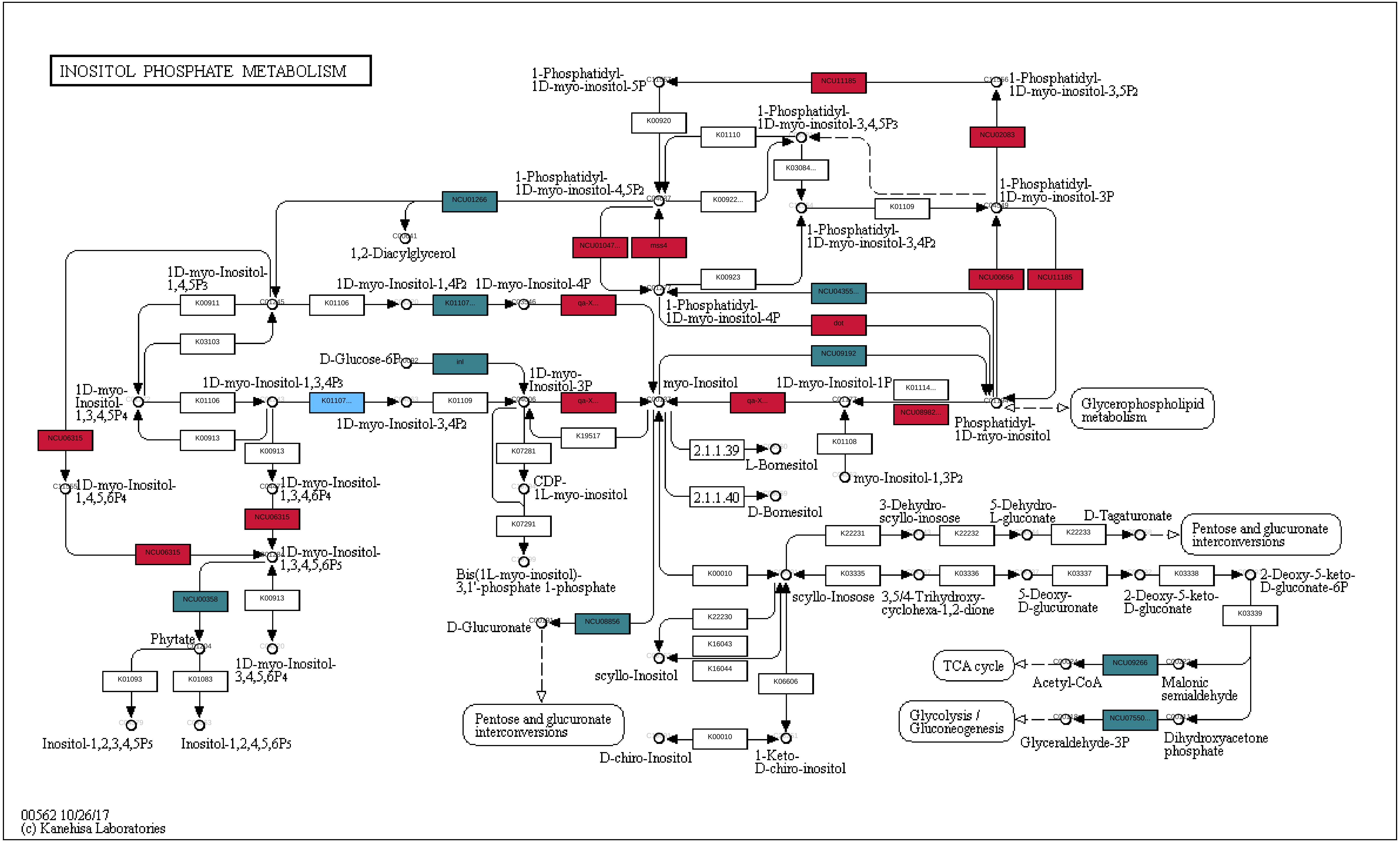

INOSITOL PHOSPHATE METABOLISM
1-Phosphatidyl-1D-myo-inositol-5P
NCU11185
1-Phosphatidyl-1D-myo-inositol-3,5P2
K00920
K01110
1-Phosphatidyl-1D-myo-inositol-3,4,5P3
NCU02083
K03084...
1-Phosphatidyl-1D-myo-inositol-4,5P2
NCU01266
K00922...
1-Phosphatidyl-1D-myo-inositol-3P
K01109
1,2-Diacylglycerol
NCU01047...
mss4
1-Phosphatidyl-1D-myo-inositol-3,4P2
K00923
NCU00656
NCU11185
1D-myo-Inositol-1,4,5P3
1D-myo-Inositol-1,4P2
1D-myo-Inositol-4P
K00911
K01106
K01107...
qa-X...
NCU04355...
1-Phosphatidyl-1D-myo-inositol-4P
dot
K03103
D-Glucose-6P
inl
NCU09192
1D-myo-Inositol-1,3,4P3
1D-myo-Inositol-3P
myo-Inositol
1D-myo-Inositol-1P
K01114...
1D-myo-Inositol-1,3,4,5P4
K01106
K01107...
K01109
qa-X...
qa-X...
NCU08982...
Glycerophospholipid metabolism
1D-myo-Inositol-3,4P2
K19517
Phosphatidyl-1D-myo-inositol
NCU06315
K00913
K00913
K07281
2.1.1.39
K01108
L-Bornesitol
myo-Inositol-1,3P2
1D-myo-Inositol-1,4,5,6P4
1D-myo-Inositol-1,3,4,6P4
CDP-1L-myo-inositol
2.1.1.40
D-Bornesitol
3-Dehydro-scyllo-inosose
5-Dehydro-L-gluconate
D-Tagaturonate
NCU06315
K07291
K22231
K22232
K22233
Pentose and glucuronate interconversions
NCU06315
1D-myo-Inositol-1,3,4,5,6P5
Bis(1L-myo-inositol)-3,1'-phosphate 1-phosphate
K00010
K03335
K03336
K03337
K03338
2-Deoxy-5-keto-D-gluconate-6P
NCU00358
K00913
scyllo-Inosose
3,5/4-Trihydroxy-cyclohexa-1,2-dione
5-Deoxy-D-glucuronate
2-Deoxy-5-keto-D-gluconate
K03339
D-Glucuronate
NCU08856
K22230
Phytate
K16043
TCA cycle
Acetyl-CoA
NCU09266
Malonic semialdehyde
1D-myo-Inositol-3,4,5,6P4
K16044
scyllo-Inositol
K01093
K01083
K06606
Glycolysis / Gluconeogenesis
NCU07550...
Glyceraldehyde-3P
Dihydroxyacetone phosphate
Pentose and glucuronate interconversions
K00010
Inositol-1,2,3,4,5P5
Inositol-1,2,4,5,6P5
D-chiro-Inositol
1-Keto-D-chiro-inositol
00562 10/26/17
(c) Kanehisa Laboratories

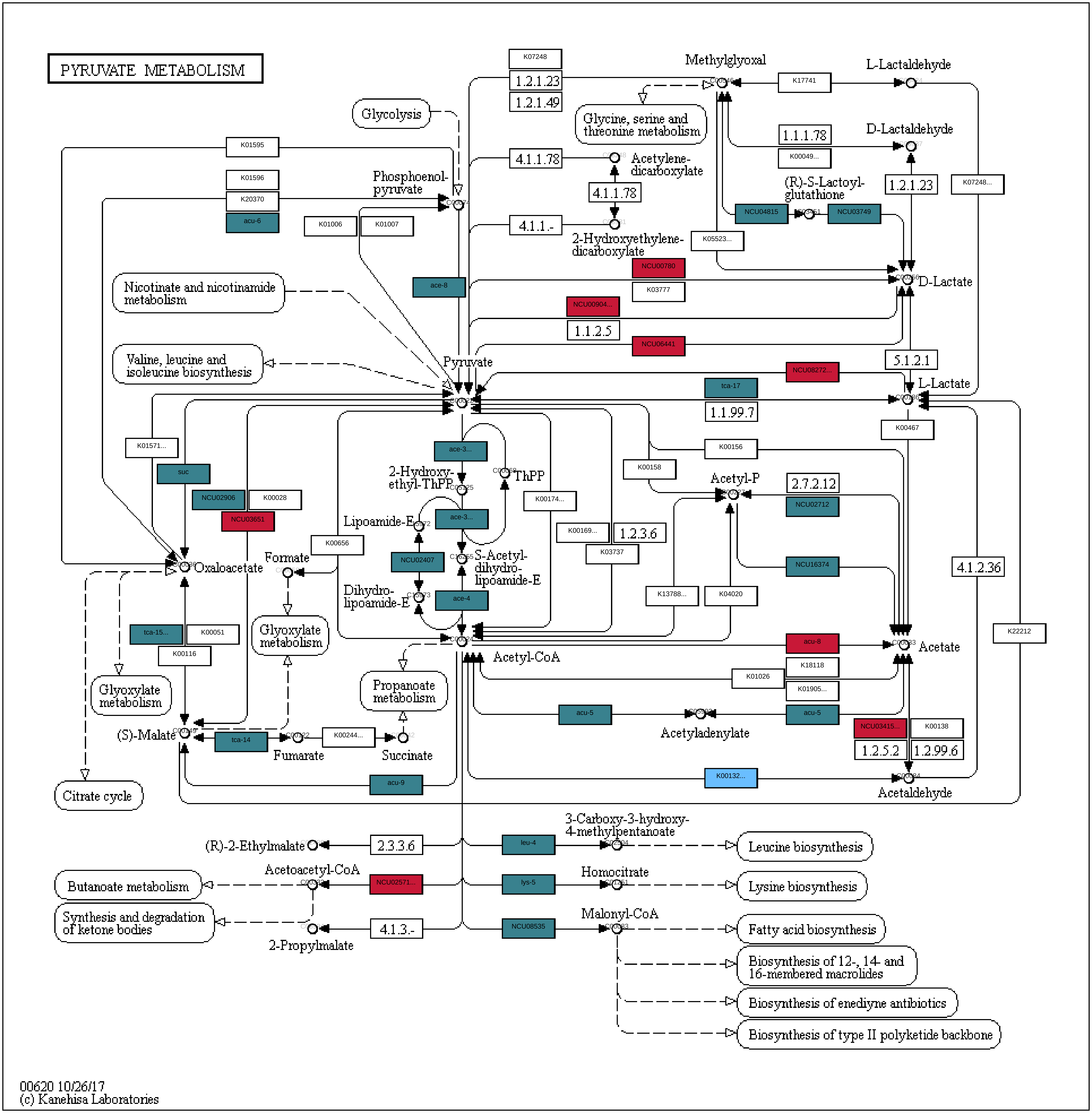
PYRUVATE METABOLISM
Glycolysis
Methylglyoxal
L-Lactaldehyde
D-Lactaldehyde
Glycine, serine and threonine metabolism
Phosphoenol-pyruvate
Acetylene-dicarboxylate
2-Hydroxyethylene-dicarboxylate
(R)-S-Lactoyl-glutathione
D-Lactate
L-Lactate
Pyruvate
Nicotinate and nicotinamide metabolism
Valine, leucine and isoleucine biosynthesis
2-Hydroxy-ethyl-ThPP
ThPP
Lipoamide-E
S-Acetyl-dihydro-lipoamide-E
Dihydro-lipoamide-E
Acetyl-P
Acetyl-CoA
Acetate
Acetyladenylate
Acetaldehyde
Oxaloacetate
Formate
Glyoxylate metabolism
Propanoate metabolism
(S)-Malate
Fumarate
Succinate
Citrate cycle
K07248
1.2.1.23
1.2.1.49
K17741
1.1.1.78
K00049...
4.1.1.78
4.1.1.-
1.2.1.23
K07248...
K01595
K01596
K20370
acu-6
K01006
K01007
ace-8
NCU04815
NCU03749
K05523...
NCU00780
K03777
NCU00904...
1.1.2.5
NCU06441
5.1.2.1
NCU08272...
tca-17
1.1.99.7
K00467
K01571...
suc
NCU02906
K00028
NCU03651
ace-3...
K00156
K00158
2.7.2.12
NCU02712
K00174...
K00169...
1.2.3.6
K00656
NCU02407
ace-4
K03737
NCU16374
4.1.2.36
K13788...
K04020
K22212
tca-15...
K00051
K00116
acu-8
K18118
K01026
K01905...
acu-5
NCU03415...
K00138
1.2.5.2
1.2.99.6
tca-14
K00244...
acu-9
K00132...
(R)-2-Ethylmalate
2.3.3.6
leu-4
3-Carboxy-3-hydroxy-4-methylpentanoate
Leucine biosynthesis
Acetoacetyl-CoA
NCU02571...
lys-5
Homocitrate
Lysine biosynthesis
Butanoate metabolism
Synthesis and degradation of ketone bodies
4.1.3.-
2-Propylmalate
NCU08535
Malonyl-CoA
Fatty acid biosynthesis
Biosynthesis of 12-, 14- and 16-membered macrolides
Biosynthesis of enediyne antibiotics
Biosynthesis of type II polyketide backbone
00620 10/26/17
(c) Kanehisa Laboratories

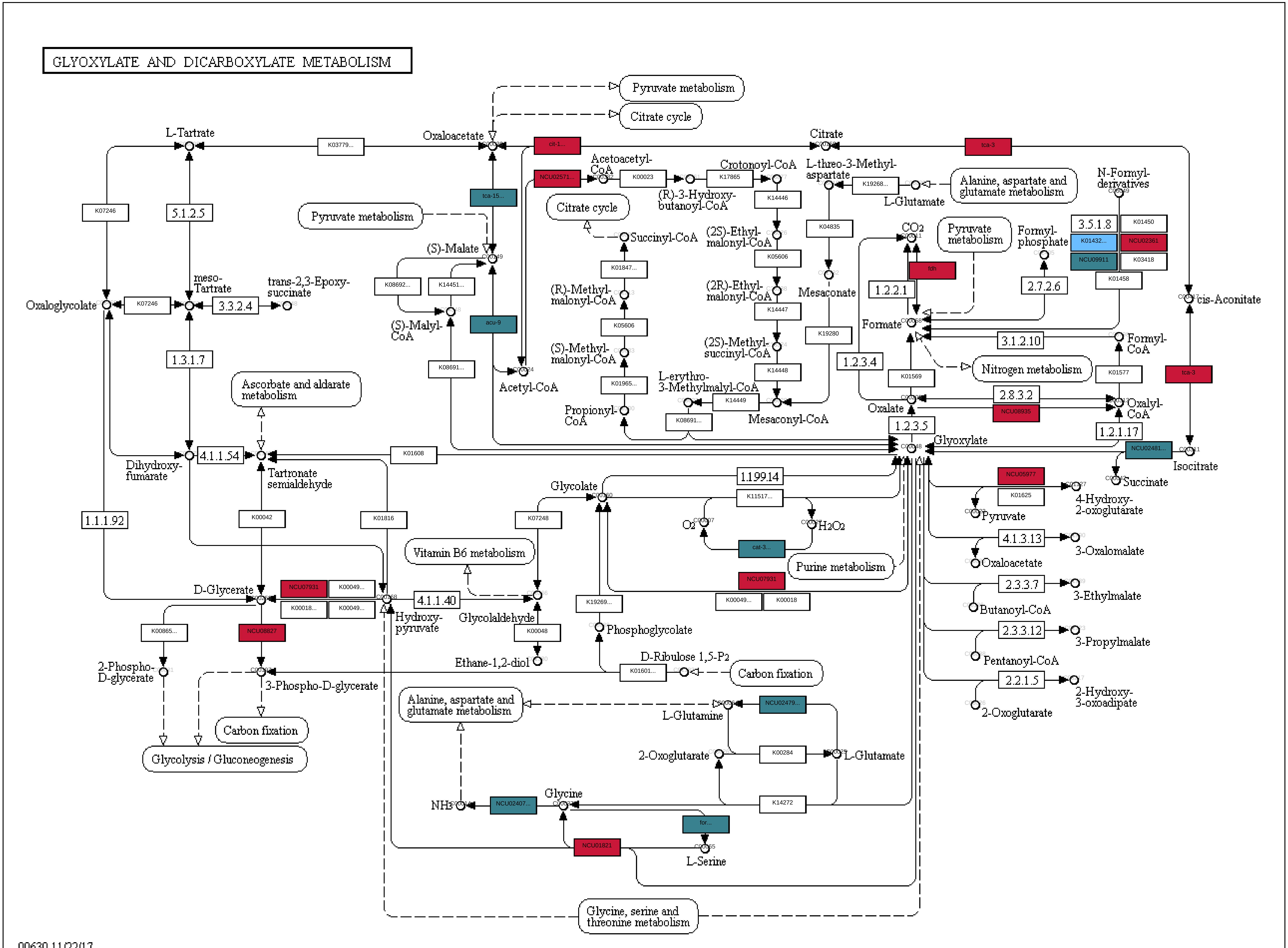


00630 11/22/17
(c) Kanehisa Laboratories

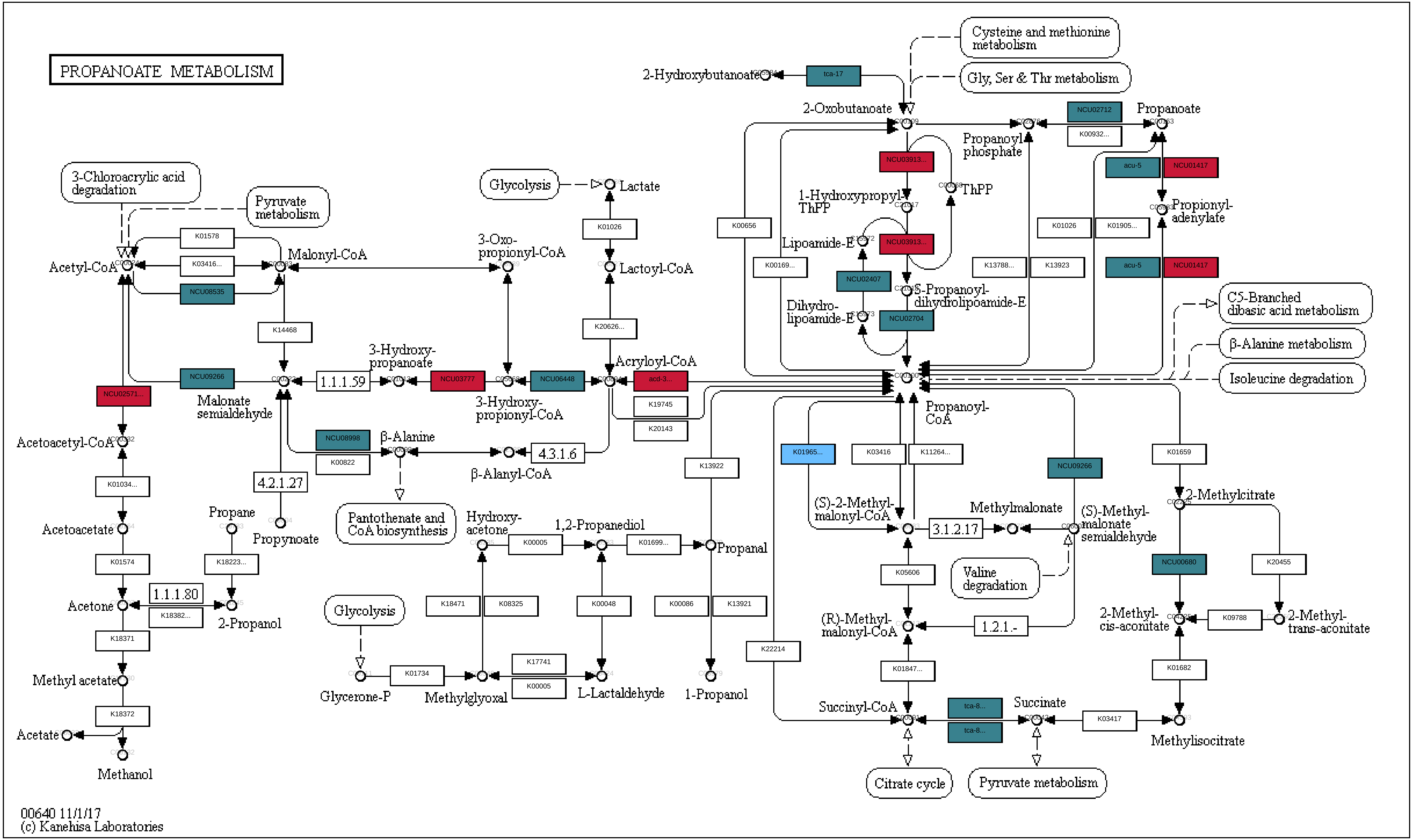
PROPANOATE METABOLISM
3-Chloroacrylic acid degradation
Pyruvate metabolism
Glycolysis
Lactate
K01026
Lactoyl-CoA
K20626...
Acryloyl-CoA
acd-3...
K19745
K20143
3-Oxo-propionyl-CoA
Malonyl-CoA
K01578
K03416...
NCU08535
Acetyl-CoA
K14468
NCU09266
Malonate semialdehyde
1.1.1.59
3-Hydroxy-propanoate
NCU03777
NCU06448
3-Hydroxy-propionyl-CoA
NCU02571...
Acetoacetyl-CoA
K01034...
Acetoacetate
K01574
Acetone
1.1.1.80
K18382...
2-Propanol
Propane
K18223...
K18371
Methyl acetate
K18372
Acetate
Methanol
4.2.1.27
Propynoate
NCU08998
K00822
β-Alanine
4.3.1.6
β-Alanyl-CoA
Pantothenate and CoA biosynthesis
Hydroxy-acetone
K00005
1,2-Propanediol
K01699...
Propanal
K18471
K08325
K00048
K00086
K13921
K13922
Glycolysis
K01734
Glycerone-P
Methylglyoxal
K17741
K00005
L-Lactaldehyde
1-Propanol
2-Hydroxybutanoate
tca-17
Cysteine and methionine metabolism
Gly, Ser & Thr metabolism
2-Oxobutanoate
NCU02712
K00932...
Propanoate
Propanoyl phosphate
NCU03913...
ThPP
1-Hydroxypropyl-ThPP
K00656
Lipoamide-E
NCU03913...
K00169...
NCU02407
S-Propanoyl-dihydrolipoamide-E
Dihydro-lipoamide-E
NCU02704
acu-5
NCU01417
Propionyl-adenylate
K01026
K01905...
K13788...
K13923
acu-5
NCU01417
C5-Branched dibasic acid metabolism
β-Alanine metabolism
Isoleucine degradation
Propanoyl-CoA
K01965...
K03416
K11264...
NCU09266
K01659
2-Methylcitrate
(S)-2-Methyl-malonyl-CoA
3.1.2.17
Methylmalonate
(S)-Methyl-malonate semialdehyde
Valine degradation
K05606
NCU00680
K20455
(R)-Methyl-malonyl-CoA
1.2.1.-
2-Methyl-cis-aconitate
K09788
2-Methyl-trans-aconitate
K22214
K01847...
K01682
Succinyl-CoA
tca-8...
tca-8...
Succinate
K03417
Methylisocitrate
Citrate cycle
Pyruvate metabolism
00640 11/1/17
(c) Kanehisa Laboratories

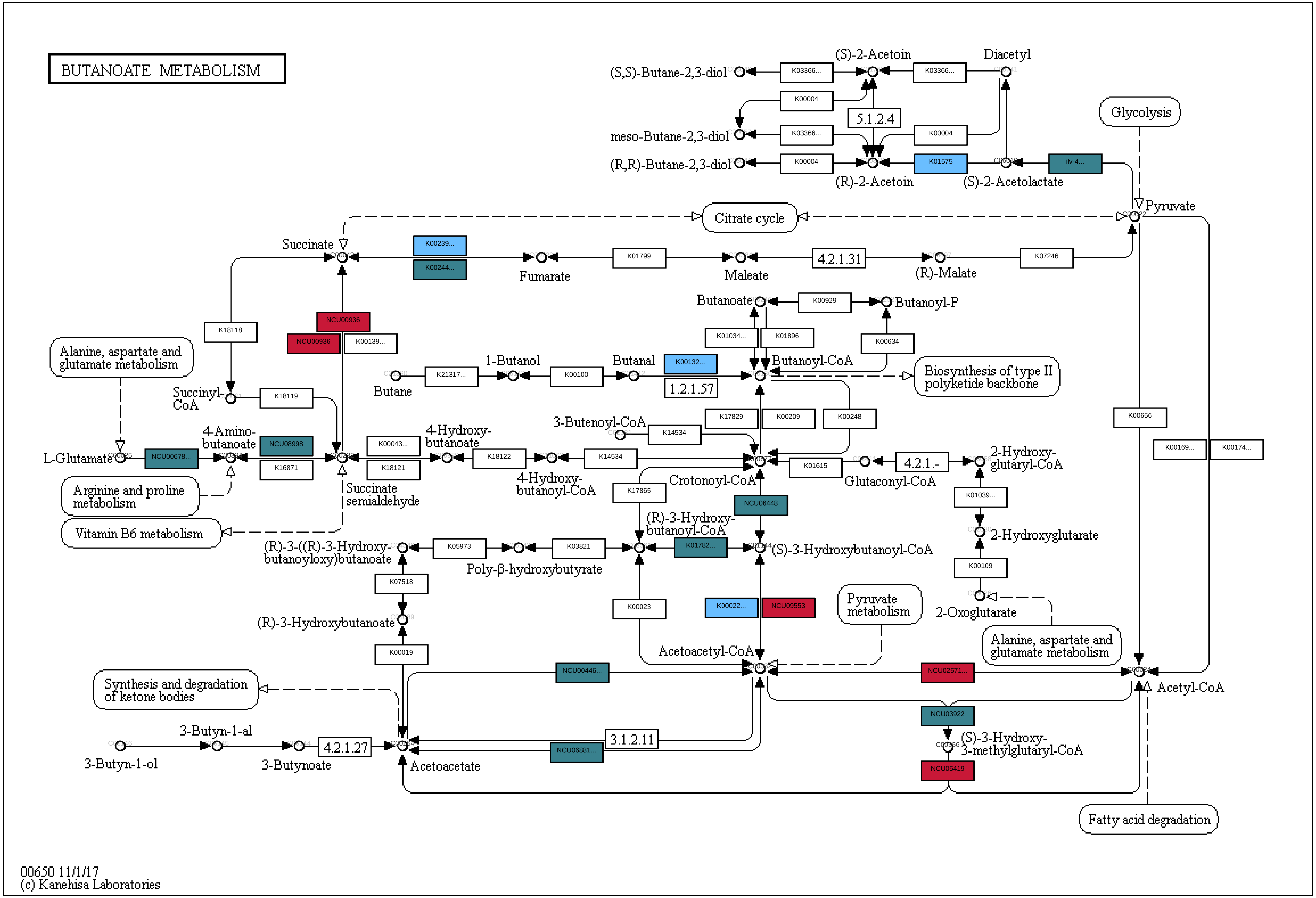
BUTANOATE METABOLISM
(S,S)-Butane-2,3-diol
K03366...
(S)-2-Acetoin
K03366...
Diacetyl
K00004
5.1.2.4
meso-Butane-2,3-diol
K03366...
K00004
Glycolysis
(R,R)-Butane-2,3-diol
K00004
K01575
ilv-4...
(R)-2-Acetoin
(S)-2-Acetolactate
Pyruvate
Citrate cycle
Succinate
K00239...
K00244...
Fumarate
K01799
Maleate
4.2.1.31
(R)-Malate
K07246
Butanoate
K00929
Butanoyl-P
NCU00936
K18118
NCU00936
K00139...
K01034...
K01896
K00634
Alanine, aspartate and glutamate metabolism
K21317...
1-Butanol
K00100
Butanal
K00132...
Butanoyl-CoA
Biosynthesis of type II polyketide backbone
Butane
1.2.1.57
Succinyl-CoA
K18119
4-Amino-butanoate
4-Hydroxy-butanoate
3-Butenoyl-CoA
K17829
K00209
K00248
K00656
L-Glutamate
NCU00678...
NCU08998
K00043...
K18122
K14534
K14534
K01615
4.2.1.-
2-Hydroxy-glutaryl-CoA
K00169...
K00174...
K16871
K18121
4-Hydroxy-butanoyl-CoA
Crotonoyl-CoA
Glutaconyl-CoA
Arginine and proline metabolism
Succinate semialdehyde
K17865
NCU06448
K01039...
(R)-3-Hydroxy-butanoyl-CoA
2-Hydroxyglutarate
Vitamin B6 metabolism
(R)-3-((R)-3-Hydroxy-butanoyloxy)butanoate
K05973
K03821
K01782...
(S)-3-Hydroxybutanoyl-CoA
Poly-β-hydroxybutyrate
K00109
K07518
K00023
K00022...
NCU09553
Pyruvate metabolism
2-Oxoglutarate
(R)-3-Hydroxybutanoate
K00019
Acetoacetyl-CoA
Alanine, aspartate and glutamate metabolism
NCU00446...
NCU02571...
Acetyl-CoA
Synthesis and degradation of ketone bodies
NCU03922
3-Butyn-1-al
3.1.2.11
(S)-3-Hydroxy-3-methylglutaryl-CoA
4.2.1.27
NCU06881...
3-Butyn-1-ol
3-Butynoate
Acetoacetate
NCU05419
Fatty acid degradation
00650 11/1/17
(c) Kanehisa Laboratories

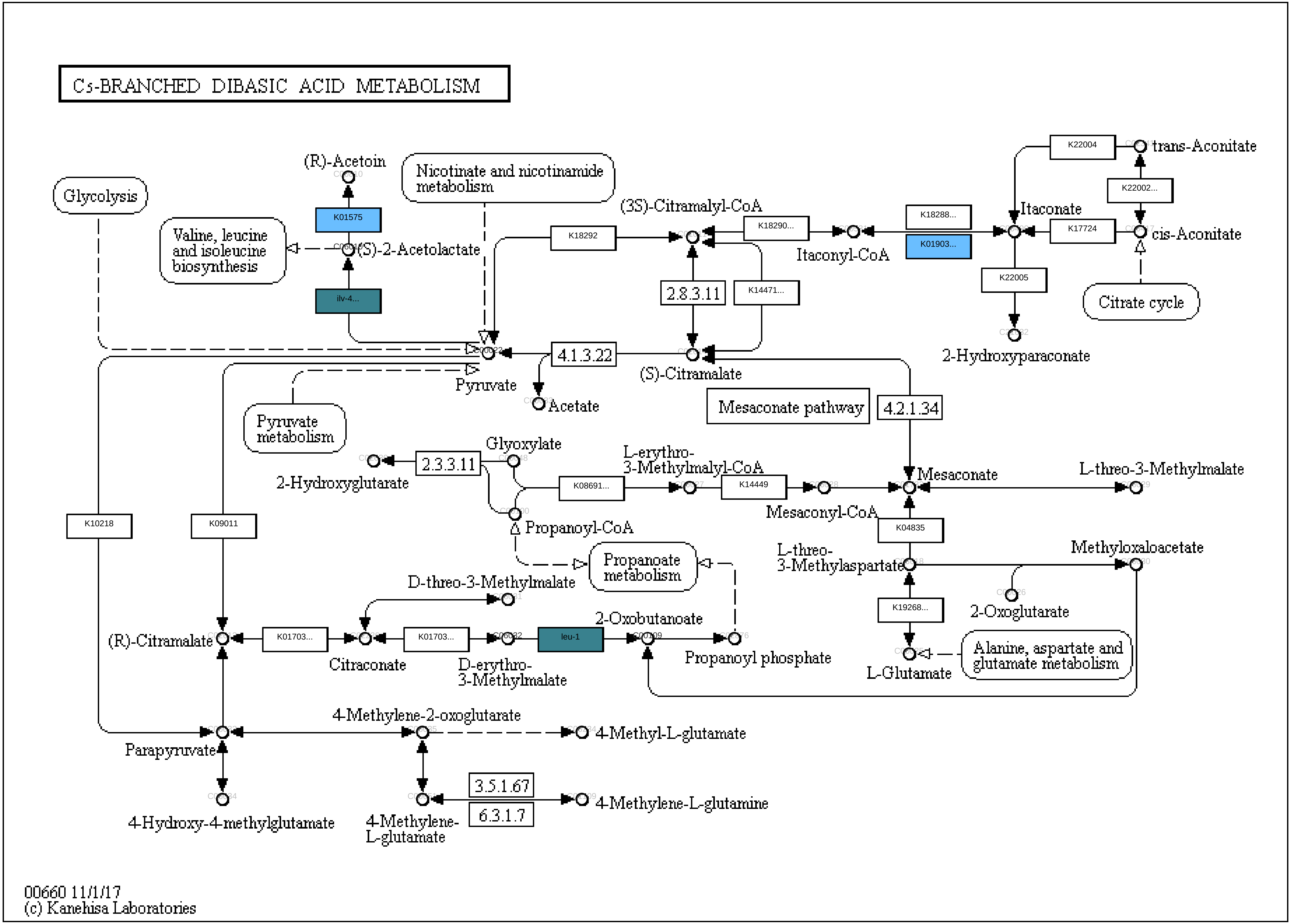
C5-BRANCHED DIBASIC ACID METABOLISM
Glycolysis
(R)-Acetoin
K01575
Nicotinate and nicotinamide metabolism
Valine, leucine and isoleucine biosynthesis
(S)-2-Acetolactate
ilv-4...
(3S)-Citramalyl-CoA
K18292
K18290...
Itaconyl-CoA
K18288...
K01903...
K22004
trans-Aconitate
K22002...
Itaconate
K17724
cis-Aconitate
2.8.3.11
K14471...
K22005
Citrate cycle
2-Hydroxyparaconate
4.1.3.22
Pyruvate
Acetate
(S)-Citramalate
Pyruvate metabolism
Mesaconate pathway
4.2.1.34
Glyoxylate
2.3.3.11
2-Hydroxyglutarate
L-erythro-3-Methylmalyl-CoA
K08691...
K14449
Mesaconate
L-threo-3-Methylmalate
Mesaconyl-CoA
Propanoyl-CoA
K04835
K10218
K09011
Propanoate metabolism
L-threo-3-Methylaspartate
Methyloxaloacetate
D-threo-3-Methylmalate
2-Oxoglutarate
K19268...
2-Oxobutanoate
(R)-Citramalate
K01703...
Citraconate
K01703...
leu-1
Propanoyl phosphate
Alanine, aspartate and glutamate metabolism
L-Glutamate
D-erythro-3-Methylmalate
4-Methylene-2-oxoglutarate
Parapyruvate
4-Methyl-L-glutamate
3.5.1.67
6.3.1.7
4-Methylene-L-glutamine
4-Hydroxy-4-methylglutamate
4-Methylene-L-glutamate
00660 11/1/17
(c) Kanehisa Laboratories

# 2. Energy metabolism

| MAP | PATHWAY |
|---|---|
| **190** | Oxidative phosphorylation |
| **680** | Methane metabolism |
| **910** | Nitrogen metabolism |
| **920** | Sulfur metabolism |

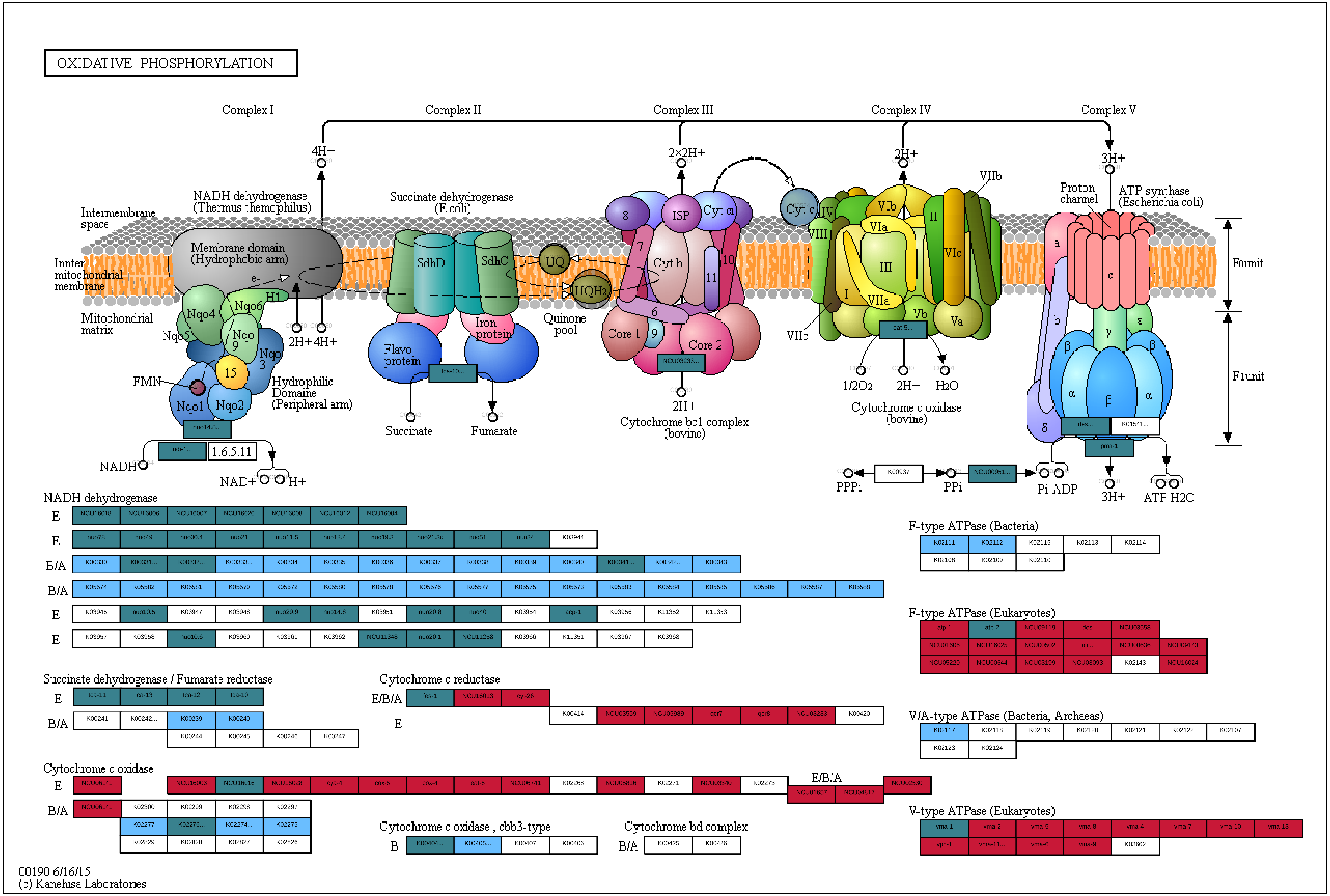
OXIDATIVE PHOSPHORYLATION
Complex I
Complex II
Complex III
Complex IV
Complex V
4H+
2×2H+
2H+
3H+
NADH dehydrogenase
(Thermus themophilus)
Succinate dehydrogenase
(E.coli)
Intermembrane
space
Membrane domain
(Hydrophobic arm)
Innter
mitochondrial
membrane
Mitochondrial
matrix
e-
Nqo4
Nqo6
H1
Nqo5
Nqo
9
Nqo
3
15
FMN
Nqo1
Nqo2
Hydrophilic
Domaine
(Peripheral arm)
nuo14.8...
ndi-1...
1.6.5.11
NADH
NAD+
H+
2H+
4H+
SdhD
SdhC
UQ
UQH2
Flavo
protein
Iron
protein
tca-10...
Succinate
Fumarate
Quinone
pool
8
ISP
Cyt a
Cyt c
7
Cyt b
10
11
6
9
Core 1
Core 2
NCU03233...
Cytochrome bc1 complex
(bovine)
VIIb
IV
VIII
VIb
VIa
II
III
VIc
I
VIIa
Vb
Va
VIIc
eat-5...
1/2O2
H2O
Cytochrome c oxidase
(bovine)
Proton
channel
ATP synthase
(Escherichia coli)
a
c
b
γ
ε
β
α
δ
des...
K01541...
pma-1
Fo unit
F1 unit
K00937
PPPi
PPi
NCU00951...
Pi ADP
ATP H2O
NADH dehydrogenase
E
NCU16018 NCU16006 NCU16007 NCU16020 NCU16008 NCU16012 NCU16004
E
nuo78 nuo49 nuo30.4 nuo21 nuo11.5 nuo18.4 nuo19.3 nuo21.3c nuo51 nuo24 K03944
B/A
K00330 K00331... K00332... K00333... K00334 K00335 K00336 K00337 K00338 K00339 K00340 K00341... K00342... K00343
B/A
K05574 K05582 K05581 K05579 K05572 K05580 K05578 K05576 K05577 K05575 K05573 K05583 K05584 K05585 K05586 K05587 K05588
E
K03945 nuo10.5 K03947 K03948 nuo29.9 nuo14.8 K03951 nuo20.8 nuo40 K03954 acp-1 K03956 K11352 K11353
E
K03957 K03958 nuo10.6 K03960 K03961 K03962 NCU11348 nuo20.1 NCU11258 K03966 K11351 K03967 K03968
Succinate dehydrogenase / Fumarate reductase
E
tca-11 tca-13 tca-12 tca-10
B/A
K00241 K00242... K00239 K00240
K00244 K00245 K00246 K00247
Cytochrome c reductase
E/B/A
fes-1 NCU16013 cyt-26
E
K00414 NCU03559 NCU05989 qcr7 qcr8 NCU03233 K00420
Cytochrome c oxidase
E
NCU06141 NCU16003 NCU16016 NCU16028 cya-4 cox-6 cox-4 eat-5 NCU06741 K02268 NCU05816 K02271 NCU03340 K02273
E/B/A
NCU01657 NCU04817 NCU02530
B/A
NCU06141 K02300 K02299 K02298 K02297
K02277 K02276... K02274... K02275
K02829 K02828 K02827 K02826
Cytochrome c oxidase , cbb3-type
B
K00404... K00405... K00407 K00406
Cytochrome bd complex
B/A
K00425 K00426
F-type ATPase (Bacteria)
K02111 K02112 K02115 K02113 K02114
K02108 K02109 K02110
F-type ATPase (Eukaryotes)
atp-1 atp-2 NCU09119 des NCU03558
NCU01606 NCU16025 NCU00502 oli... NCU00636 NCU09143
NCU05220 NCU00644 NCU03199 NCU08093 K02143 NCU16024
V/A-type ATPase (Bacteria, Archaeas)
K02117 K02118 K02119 K02120 K02121 K02122 K02107
K02123 K02124
V-type ATPase (Eukaryotes)
vma-1 vma-2 vma-5 vma-8 vma-4 vma-7 vma-10 vma-13
vph-1 vma-11... vma-6 vma-9 K03662
00190 6/16/15
(c) Kanehisa Laboratories

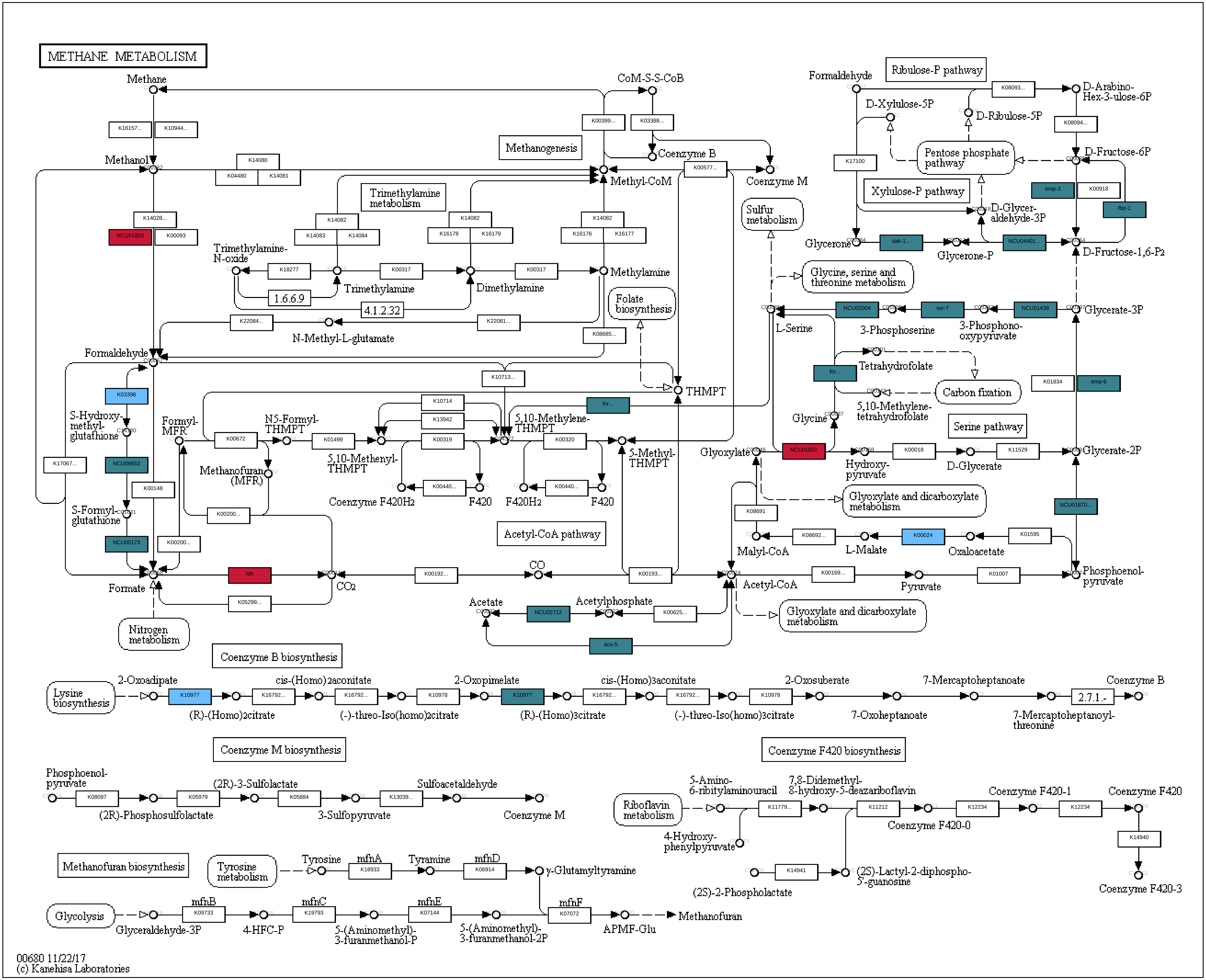

METHANE METABOLISM
Methane
CoM-S-S-CoB
Formaldehyde
Ribulose-P pathway
D-Arabino-Hex-3-ulose-6P
D-Xylulose-5P
D-Ribulose-5P
Methanogenesis
Coenzyme B
Methanol
Methyl-CoM
Coenzyme M
Pentose phosphate pathway
D-Fructose-6P
Trimethylamine metabolism
Sulfur metabolism
Xylulose-P pathway
D-Glyceraldehyde-3P
Glycerone
Glycerone-P
D-Fructose-1,6-P2
Trimethylamine-N-oxide
Trimethylamine
Dimethylamine
Methylamine
Glycine, serine and threonine metabolism
1.6.6.9
4.1.2.32
Folate biosynthesis
N-Methyl-L-glutamate
L-Serine
3-Phosphoserine
3-Phosphonooxypyruvate
Glycerate-3P
Tetrahydrofolate
Carbon fixation
S-Hydroxymethylglutathione
Formyl-MFR
N5-Formyl-THMPT
THMPT
5,10-Methylene-THMPT
5,10-Methylenetetrahydrofolate
Glycine
Serine pathway
Methanofuran (MFR)
5,10-Methenyl-THMPT
5-Methyl-THMPT
Glyoxylate
Hydroxypyruvate
D-Glycerate
Glycerate-2P
Coenzyme F420H2
F420
F420H2
Glyoxylate and dicarboxylate metabolism
S-Formylglutathione
Acetyl-CoA pathway
Malyl-CoA
L-Malate
Oxaloacetate
CO
Formate
CO2
Acetyl-CoA
Pyruvate
Phosphoenolpyruvate
Acetate
Acetylphosphate
Nitrogen metabolism
Coenzyme B biosynthesis
Lysine biosynthesis
2-Oxoadipate
cis-(Homo)2aconitate
2-Oxopimelate
cis-(Homo)3aconitate
2-Oxosuberate
7-Mercaptoheptanoate
(R)-(Homo)2citrate
(-)-threo-Iso(homo)2citrate
(R)-(Homo)3citrate
(-)-threo-Iso(homo)3citrate
7-Oxoheptanoate
7-Mercaptoheptanoylthreonine
2.7.1.-
Coenzyme M biosynthesis
Coenzyme F420 biosynthesis
(2R)-3-Sulfolactate
Sulfoacetaldehyde
(2R)-Phosphosulfolactate
3-Sulfopyruvate
5-Amino-6-ribitylaminouracil
7,8-Didemethyl-8-hydroxy-5-deazariboflavin
Coenzyme F420-1
Coenzyme F420
Riboflavin metabolism
Coenzyme F420-0
4-Hydroxyphenylpyruvate
(2S)-Lactyl-2-diphospho-5'-guanosine
(2S)-2-Phospholactate
Coenzyme F420-3
Methanofuran biosynthesis
Tyrosine metabolism
Tyrosine
mfnA
Tyramine
mfnD
γ-Glutamyltyramine
Glycolysis
Glyceraldehyde-3P
mfnB
4-HFC-P
mfnC
5-(Aminomethyl)-3-furanmethanol-P
mfnE
5-(Aminomethyl)-3-furanmethanol-2P
mfnF
APMF-Glu
Methanofuran
00680 11/22/17
(c) Kanehisa Laboratories

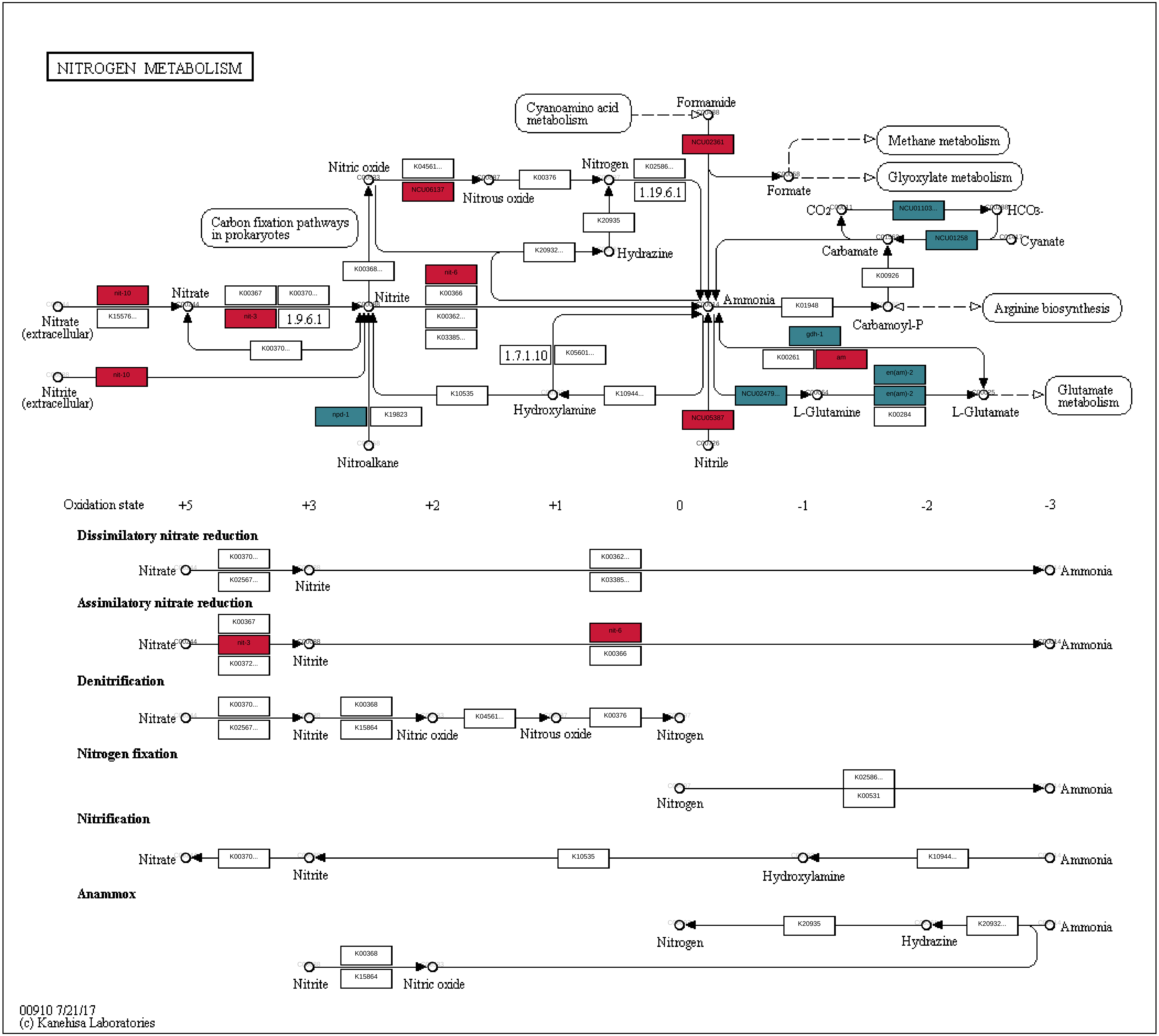
NITROGEN METABOLISM
Cyanoamino acid metabolism
Formamide
NCU02361
Methane metabolism
Glyoxylate metabolism
Formate
Nitric oxide
K04561...
NCU06137
Nitrous oxide
K00376
Nitrogen
K02586...
1.19.6.1
K20935
K20932...
Hydrazine
CO2
NCU01103...
HCO3-
NCU01258
Cyanate
Carbamate
K00926
Carbon fixation pathways in prokaryotes
K00368...
nit-10
K15576...
Nitrate
K00367
K00370...
nit-3
1.9.6.1
Nitrite
nit-6
K00366
K00362...
K03385...
Ammonia
K01948
Carbamoyl-P
Arginine biosynthesis
Nitrate (extracellular)
K00370...
1.7.1.10
K05601...
gdh-1
K00261
am
en(am)-2
Nitrite (extracellular)
K10535
K10944...
NCU02479...
en(am)-2
Glutamate metabolism
npd-1
K19823
Hydroxylamine
NCU05387
L-Glutamine
K00284
L-Glutamate
Nitroalkane
Nitrile
Oxidation state
+5
+3
+2
+1
0
-1
-2
-3
Dissimilatory nitrate reduction
Nitrate
K00370...
K02567...
Nitrite
K00362...
K03385...
Ammonia
Assimilatory nitrate reduction
Nitrate
K00367
nit-3
K00372...
Nitrite
nit-6
K00366
Ammonia
Denitrification
Nitrate
K00370...
K02567...
Nitrite
K00368
K15864
Nitric oxide
K04561...
Nitrous oxide
K00376
Nitrogen
Nitrogen fixation
Nitrogen
K02586...
K00531
Ammonia
Nitrification
Nitrate
K00370...
Nitrite
K10535
Hydroxylamine
K10944...
Ammonia
Anammox
Nitrogen
K20935
Hydrazine
K20932...
Ammonia
Nitrite
K00368
K15864
Nitric oxide
00910 7/21/17
(c) Kanehisa Laboratories

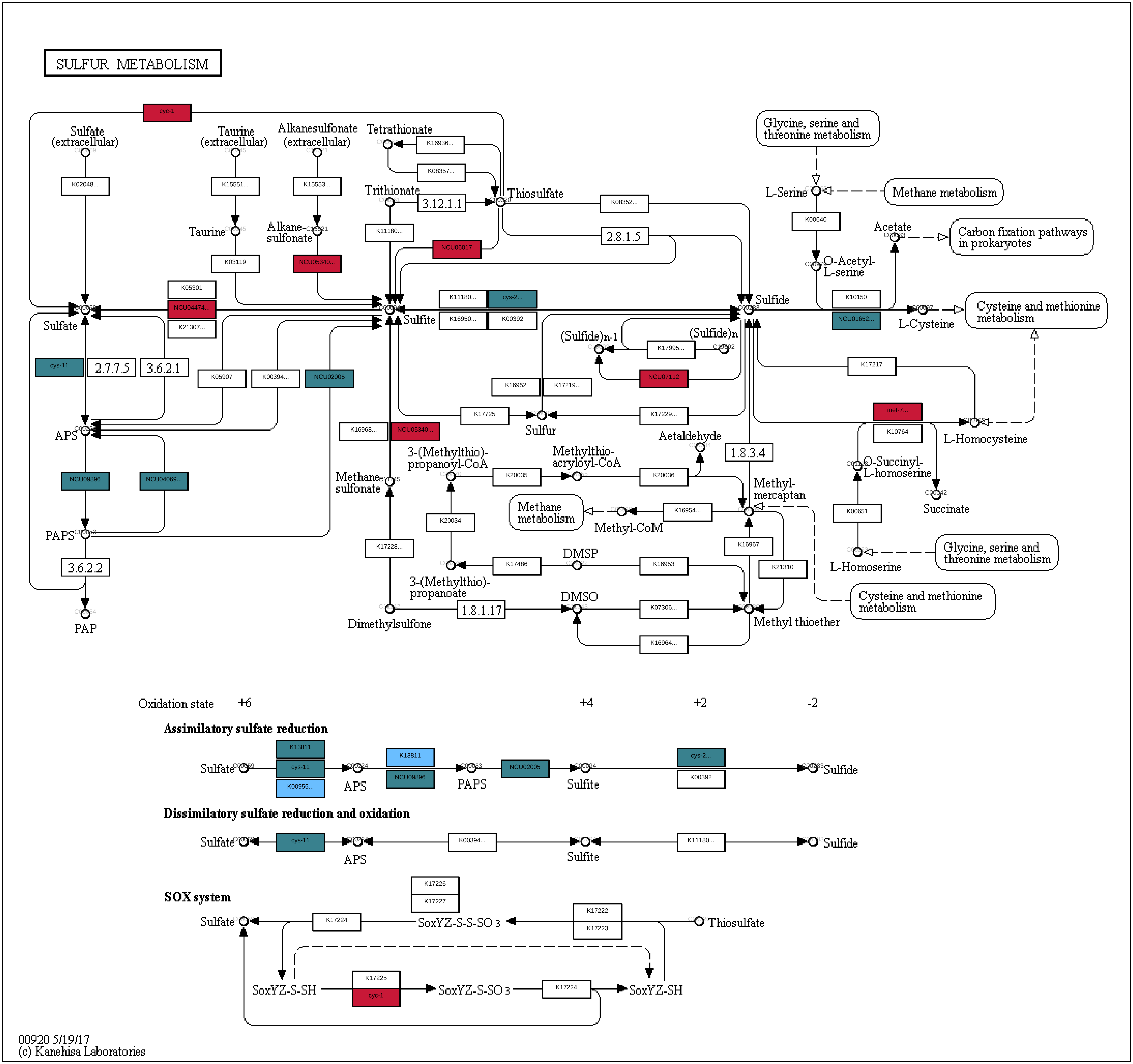
SULFUR METABOLISM
cyc-1
Sulfate (extracellular)
Taurine (extracellular)
Alkanesulfonate (extracellular)
Tetrathionate
K16936...
K08357...
K02048...
K15551...
K15553...
Trithionate
3.12.1.1
Thiosulfate
K08352...
Glycine, serine and threonine metabolism
L-Serine
Methane metabolism
K00640
Acetate
Carbon fixation pathways in prokaryotes
Taurine
Alkane-sulfonate
K11180...
NCU06017
2.8.1.5
O-Acetyl-L-serine
K03119
NCU05340...
K05301
NCU04474...
K21307...
K11180...
cys-2...
K16950...
K00392
Sulfide
K10150
NCU01652...
L-Cysteine
Cysteine and methionine metabolism
Sulfate
Sulfite
(Sulfide)n-1
(Sulfide)n
K17995...
cys-11
2.7.7.5
3.6.2.1
K05907
K00394...
NCU02005
NCU07112
K17217
K16952
K17219...
K17725
K17229...
met-7...
APS
K16968...
NCU05340...
Sulfur
K10764
L-Homocysteine
Aetaldehyde
1.8.3.4
3-(Methylthio)-propanoyl-CoA
Methylthio-acryloyl-CoA
O-Succinyl-L-homoserine
NCU09896
NCU04069...
Methane sulfonate
K20035
K20036
Methyl-mercaptan
Methane metabolism
Methyl-CoM
K16954...
Succinate
K20034
K00651
PAPS
K17228...
K16967
DMSP
K17486
K16953
K21310
Glycine, serine and threonine metabolism
L-Homoserine
3.6.2.2
3-(Methylthio)-propanoate
DMSO
Cysteine and methionine metabolism
1.8.1.17
K07306...
PAP
Dimethylsulfone
Methyl thioether
K16964...
Oxidation state +6 +4 +2 -2
Assimilatory sulfate reduction
K13811
cys-11
K00955...
Sulfate
APS
K13811
NCU09896
PAPS
NCU02005
Sulfite
cys-2...
K00392
Sulfide
Dissimilatory sulfate reduction and oxidation
Sulfate
cys-11
APS
K00394...
Sulfite
K11180...
Sulfide
SOX system
K17226
K17227
Sulfate
K17224
SoxYZ-S-S-SO3
K17222
K17223
Thiosulfate
SoxYZ-S-SH
K17225
cyc-1
SoxYZ-S-S-SO3
K17224
SoxYZ-SH
00920 5/19/17
(c) Kanehisa Laboratories

# 3. Lipid metabolism

| MAP | PATHWAY |
|---|---|
| **61** | Fatty acid biosynthesis |
| **62** | Fatty acid elongation |
| **71** | Fatty acid degradation |
| **72** | Synthesis and degradation of ketone bodies |
| **100** | Steroid biosynthesis |
| **561** | Glycerolipid metabolism |
| **564** | Glycerophospholipid metabolism |
| **565** | Ether lipid metabolism |
| **590** | Arachidonic acid metabolism |
| **592** | alpha-Linolenic acid metabolism |
| **600** | Sphingolipid metabolism |

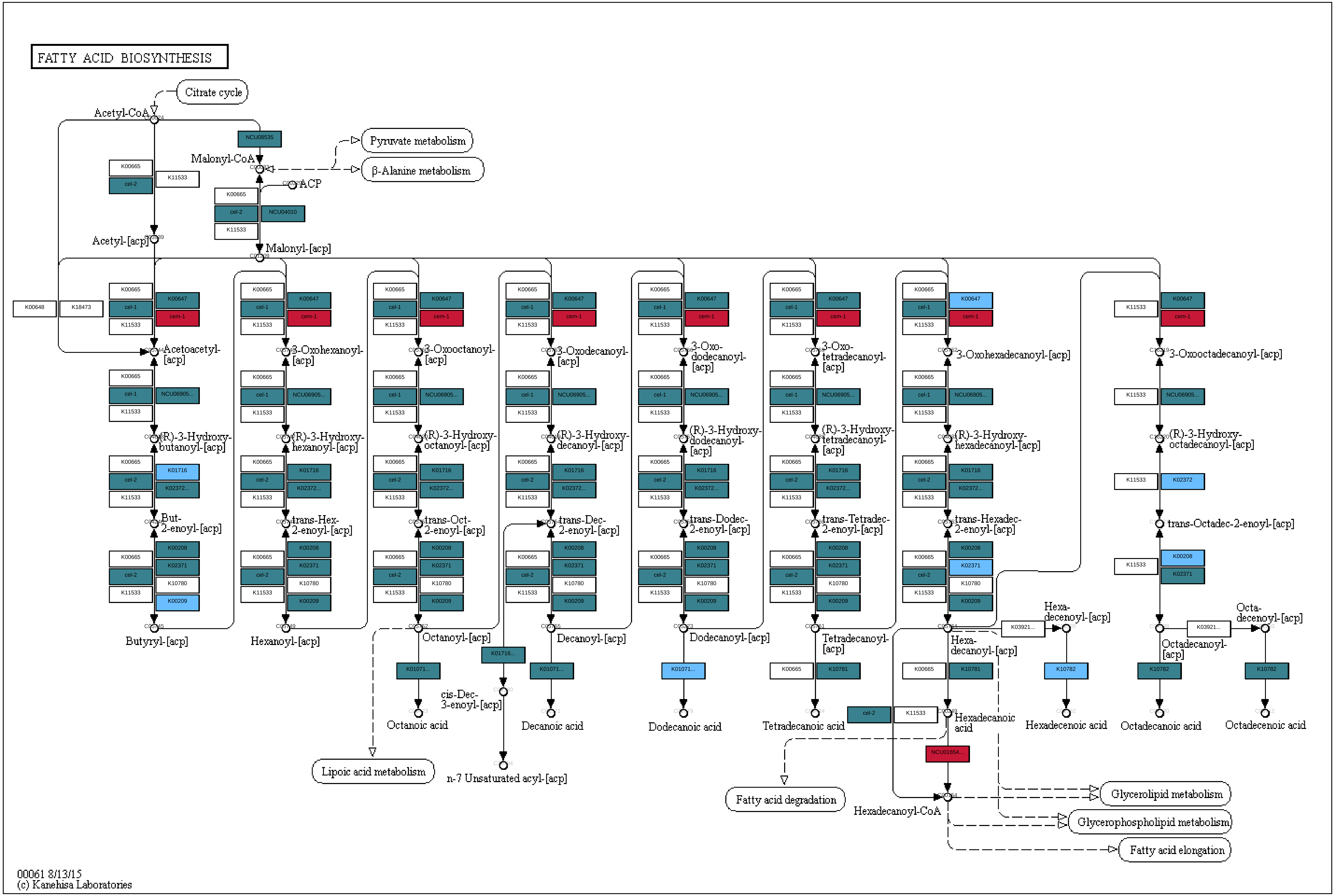
FATTY ACID BIOSYNTHESIS
Citrate cycle
Acetyl-CoA
Malonyl-CoA
Pyruvate metabolism
β-Alanine metabolism
ACP
Acetyl-[acp]
Malonyl-[acp]
NCU08535
NCU04010
K00665
cel-2
K11533
K00648
K18473
K00647
cem-1
cel-1
NCU06905...
K01716
K02372...
K00208
K02371
K10780
K00209
Acetoacetyl-[acp]
3-Oxohexanoyl-[acp]
3-Oxooctanoyl-[acp]
3-Oxodecanoyl-[acp]
3-Oxo-dodecanoyl-[acp]
3-Oxo-tetradecanoyl-[acp]
3-Oxohexadecanoyl-[acp]
3-Oxooctadecanoyl-[acp]
(R)-3-Hydroxy-butanoyl-[acp]
(R)-3-Hydroxy-hexanoyl-[acp]
(R)-3-Hydroxy-octanoyl-[acp]
(R)-3-Hydroxy-decanoyl-[acp]
(R)-3-Hydroxy-dodecanoyl-[acp]
(R)-3-Hydroxy-tetradecanoyl-[acp]
(R)-3-Hydroxy-hexadecanoyl-[acp]
(R)-3-Hydroxy-octadecanoyl-[acp]
But-2-enoyl-[acp]
trans-Hex-2-enoyl-[acp]
trans-Oct-2-enoyl-[acp]
trans-Dec-2-enoyl-[acp]
trans-Dodec-2-enoyl-[acp]
trans-Tetradec-2-enoyl-[acp]
trans-Hexadec-2-enoyl-[acp]
trans-Octadec-2-enoyl-[acp]
Butyryl-[acp]
Hexanoyl-[acp]
Octanoyl-[acp]
Decanoyl-[acp]
Dodecanoyl-[acp]
Tetradecanoyl-[acp]
Hexa-decanoyl-[acp]
Hexa-decenoyl-[acp]
Octadecanoyl-[acp]
Octa-decenoyl-[acp]
K03921...
K01071...
K01716...
K10781
K10782
NCU01654...
cis-Dec-3-enoyl-[acp]
Octanoic acid
Decanoic acid
Dodecanoic acid
Tetradecanoic acid
Hexadecanoic acid
Hexadecenoic acid
Octadecanoic acid
Octadecenoic acid
Lipoic acid metabolism
n-7 Unsaturated acyl-[acp]
Fatty acid degradation
Hexadecanoyl-CoA
Glycerolipid metabolism
Glycerophospholipid metabolism
Fatty acid elongation
00061 8/13/15
(c) Kanehisa Laboratories

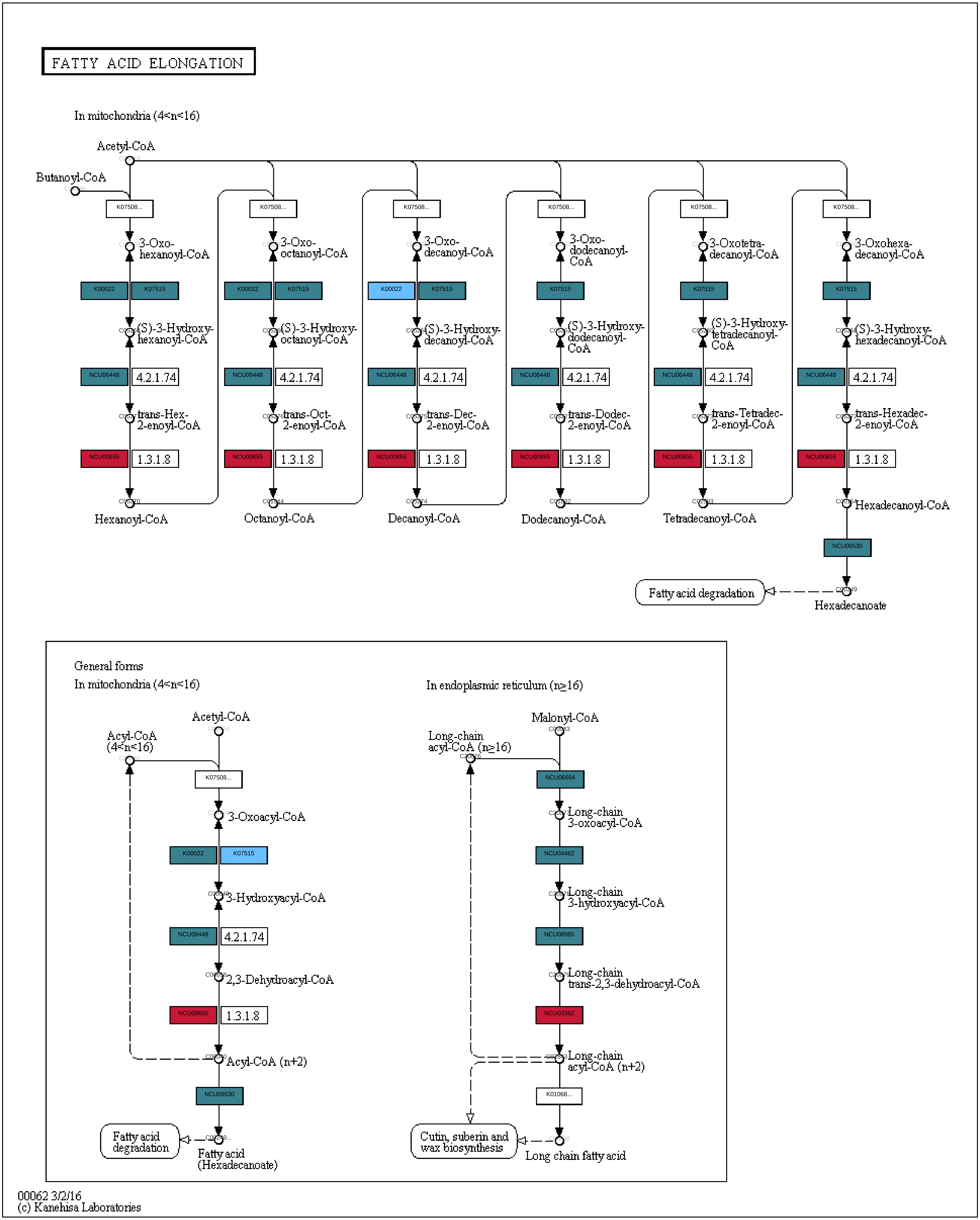
FATTY ACID ELONGATION
In mitochondria (4<n<16)
Acetyl-CoA
Butanoyl-CoA
K07508...
3-Oxo-hexanoyl-CoA
3-Oxo-octanoyl-CoA
3-Oxo-decanoyl-CoA
3-Oxo-dodecanoyl-CoA
3-Oxotetra-decanoyl-CoA
3-Oxohexa-decanoyl-CoA
K00022
K07515
(S)-3-Hydroxy-hexanoyl-CoA
(S)-3-Hydroxy-octanoyl-CoA
(S)-3-Hydroxy-decanoyl-CoA
(S)-3-Hydroxy-dodecanoyl-CoA
(S)-3-Hydroxy-tetradecanoyl-CoA
(S)-3-Hydroxy-hexadecanoyl-CoA
NCU06448
4.2.1.74
trans-Hex-2-enoyl-CoA
trans-Oct-2-enoyl-CoA
trans-Dec-2-enoyl-CoA
trans-Dodec-2-enoyl-CoA
trans-Tetradec-2-enoyl-CoA
trans-Hexadec-2-enoyl-CoA
NCU00655
1.3.1.8
Hexanoyl-CoA
Octanoyl-CoA
Decanoyl-CoA
Dodecanoyl-CoA
Tetradecanoyl-CoA
Hexadecanoyl-CoA
NCU06530
Fatty acid degradation
Hexadecanoate
General forms
In mitochondria (4<n<16)
Acetyl-CoA
Acyl-CoA (4<n<16)
K07508...
3-Oxoacyl-CoA
K00022
K07515
3-Hydroxyacyl-CoA
NCU06448
4.2.1.74
2,3-Dehydroacyl-CoA
NCU00655
1.3.1.8
Acyl-CoA (n+2)
NCU06530
Fatty acid degradation
Fatty acid (Hexadecanoate)
In endoplasmic reticulum (n≥16)
Malonyl-CoA
Long-chain acyl-CoA (n≥16)
NCU06694
Long-chain 3-oxoacyl-CoA
NCU04462
Long-chain 3-hydroxyacyl-CoA
NCU08985
Long-chain trans-2,3-dehydroacyl-CoA
NCU03362
Long-chain acyl-CoA (n+2)
K01068...
Cutin, suberin and wax biosynthesis
Long chain fatty acid
00062 3/2/16
(c) Kanehisa Laboratories

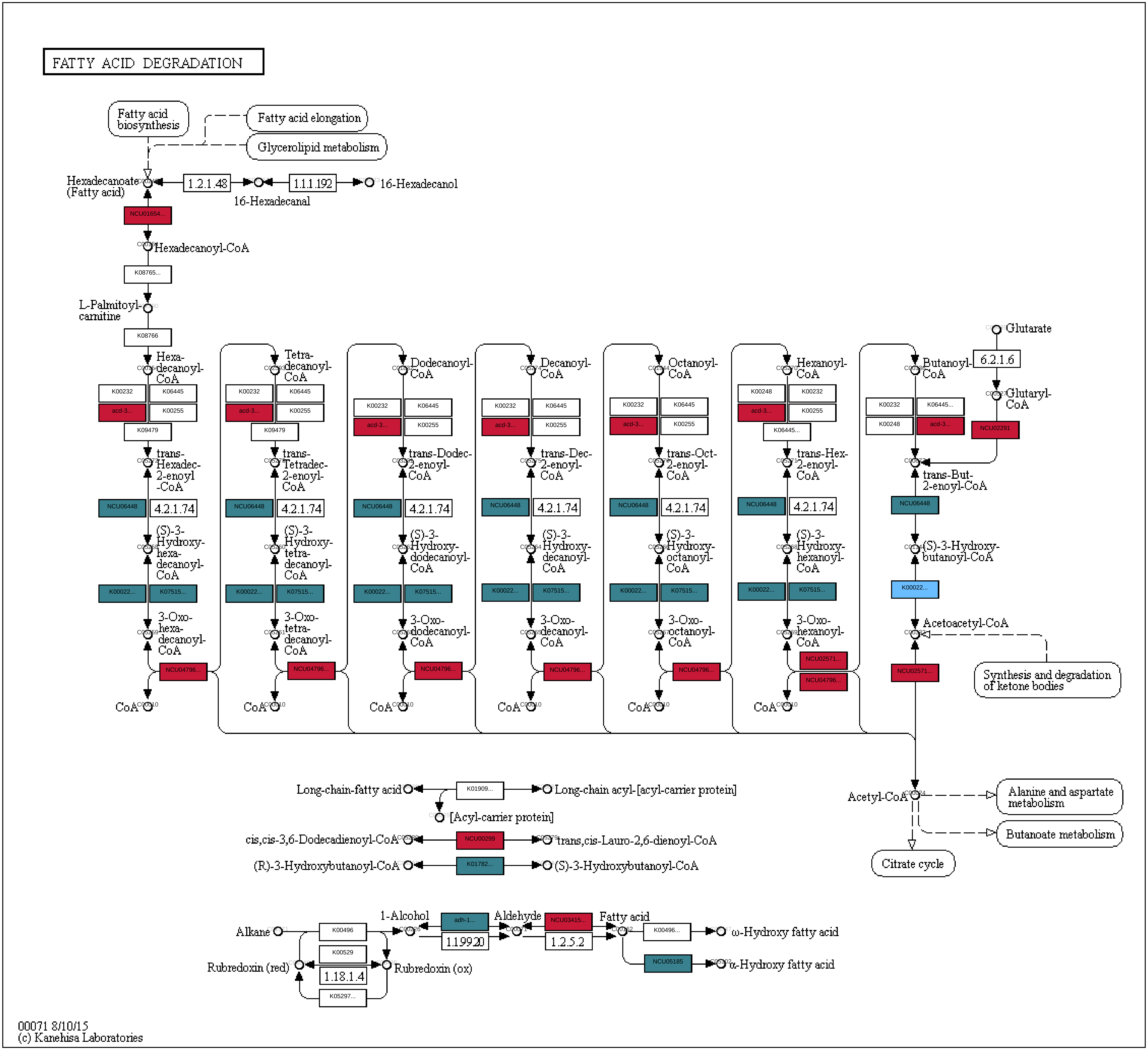
FATTY ACID DEGRADATION
Fatty acid biosynthesis
Fatty acid elongation
Glycerolipid metabolism
Hexadecanoate (Fatty acid)
1.2.1.48
16-Hexadecanal
1.1.1.192
16-Hexadecanol
NCU01654...
Hexadecanoyl-CoA
K08765...
L-Palmitoyl-carnitine
K08766
Hexa-decanoyl-CoA
Tetra-decanoyl-CoA
Dodecanoyl-CoA
Decanoyl-CoA
Octanoyl-CoA
Hexanoyl-CoA
Butanoyl-CoA
Glutarate
6.2.1.6
Glutaryl-CoA
NCU02291
K00232
K06445
acd-3...
K00255
K09479
K00248
K06445...
trans-Hexadec-2-enoyl-CoA
trans-Tetradec-2-enoyl-CoA
trans-Dodec-2-enoyl-CoA
trans-Dec-2-enoyl-CoA
trans-Oct-2-enoyl-CoA
trans-Hex-2-enoyl-CoA
trans-But-2-enoyl-CoA
NCU06448
4.2.1.74
(S)-3-Hydroxy-hexa-decanoyl-CoA
(S)-3-Hydroxy-tetra-decanoyl-CoA
(S)-3-Hydroxy-dodecanoyl-CoA
(S)-3-Hydroxy-decanoyl-CoA
(S)-3-Hydroxy-octanoyl-CoA
(S)-3-Hydroxy-hexanoyl-CoA
(S)-3-Hydroxy-butanoyl-CoA
K00022...
K07515...
3-Oxo-hexa-decanoyl-CoA
3-Oxo-tetra-decanoyl-CoA
3-Oxo-dodecanoyl-CoA
3-Oxo-decanoyl-CoA
3-Oxo-octanoyl-CoA
3-Oxo-hexanoyl-CoA
Acetoacetyl-CoA
NCU04796...
NCU02571...
Synthesis and degradation of ketone bodies
CoA
Acetyl-CoA
Alanine and aspartate metabolism
Butanoate metabolism
Citrate cycle
Long-chain-fatty acid
K01909...
Long-chain acyl-[acyl-carrier protein]
[Acyl-carrier protein]
cis,cis-3,6-Dodecadienoyl-CoA
NCU00299
trans,cis-Lauro-2,6-dienoyl-CoA
(R)-3-Hydroxybutanoyl-CoA
K01782...
(S)-3-Hydroxybutanoyl-CoA
Alkane
K00496
K00529
1.18.1.4
K05297...
Rubredoxin (red)
Rubredoxin (ox)
1-Alcohol
adh-1...
1.1.99.20
Aldehyde
NCU03415...
1.2.5.2
Fatty acid
K00496...
ω-Hydroxy fatty acid
NCU05185
α-Hydroxy fatty acid
00071 8/10/15
(c) Kanehisa Laboratories

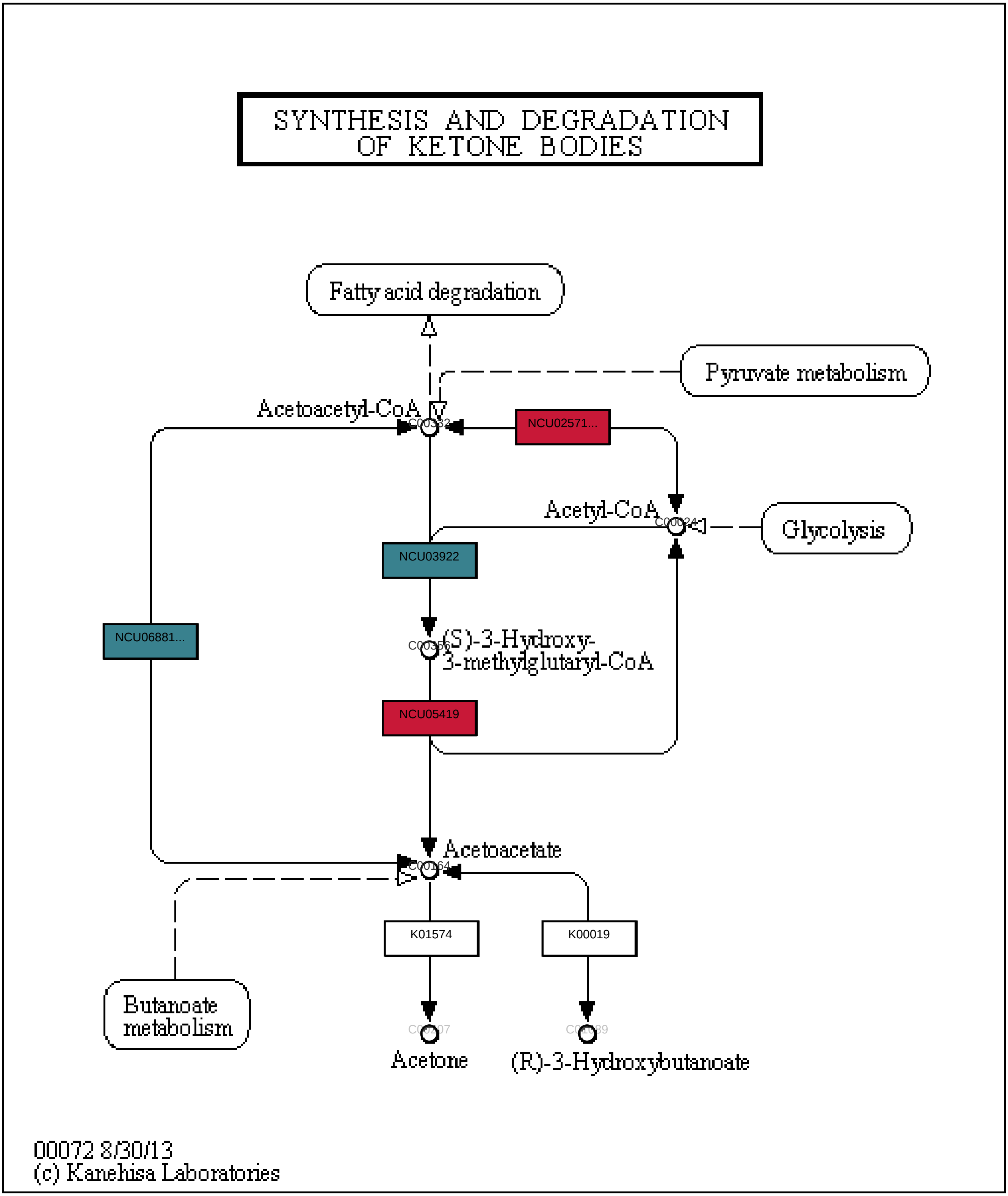
SYNTHESIS AND DEGRADATION OF KETONE BODIES
Fatty acid degradation
Pyruvate metabolism
Acetoacetyl-CoA
NCU02571...
Acetyl-CoA
Glycolysis
NCU03922
NCU06881...
(S)-3-Hydroxy-3-methylglutaryl-CoA
NCU05419
Acetoacetate
K01574
K00019
Butanoate metabolism
Acetone
(R)-3-Hydroxybutanoate
00072 8/30/13
(c) Kanehisa Laboratories

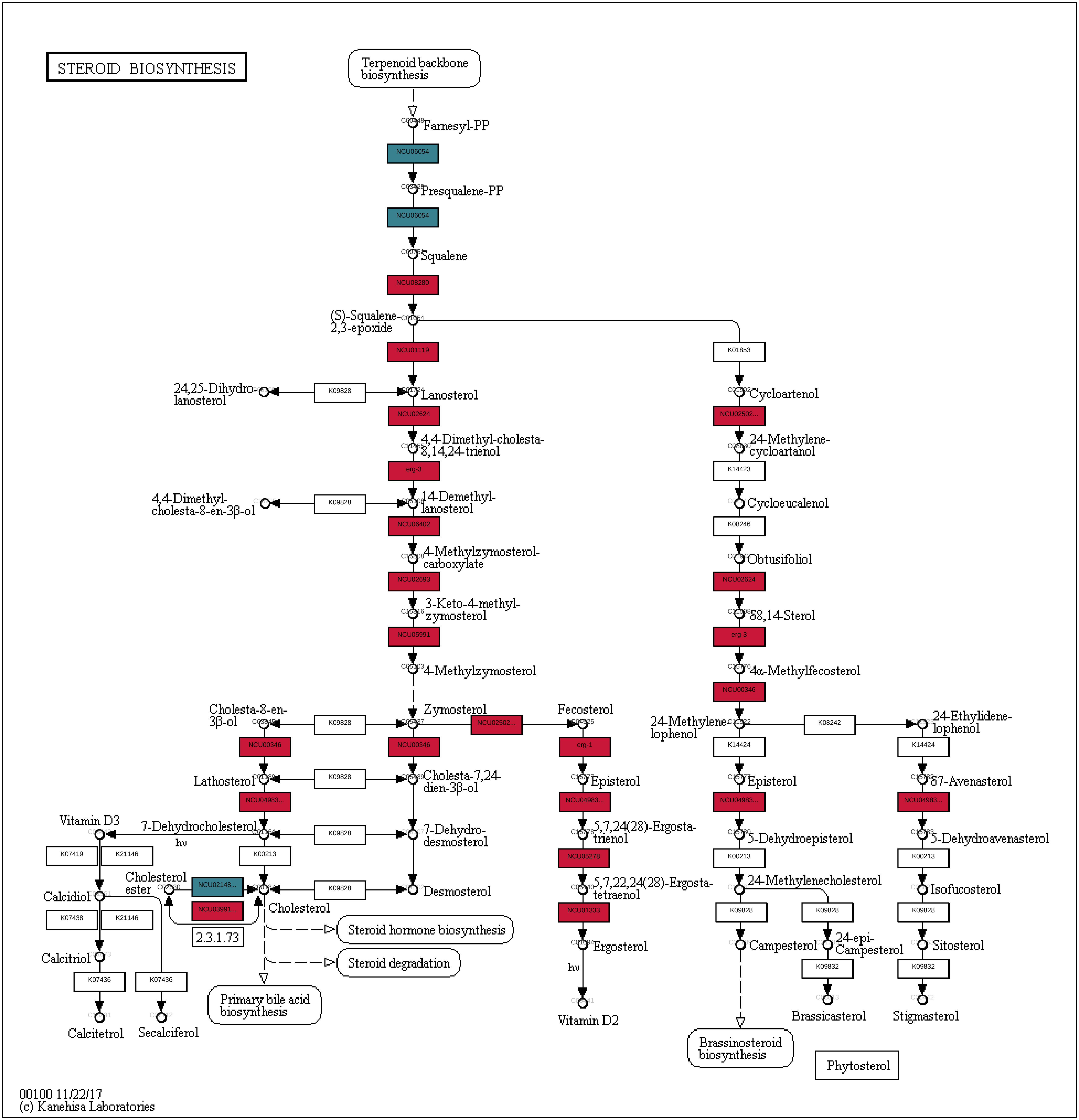
STEROID BIOSYNTHESIS
Terpenoid backbone biosynthesis
Farnesyl-PP
NCU06054
Presqualene-PP
NCU06054
Squalene
NCU08280
(S)-Squalene-2,3-epoxide
NCU01119
K01853
24,25-Dihydro-lanosterol
K09828
Lanosterol
Cycloartenol
NCU02624
NCU02502...
4,4-Dimethyl-cholesta-8,14,24-trienol
24-Methylene-cycloartanol
erg-3
K14423
4,4-Dimethyl-cholesta-8-en-3β-ol
K09828
14-Demethyl-lanosterol
Cycloeucalenol
NCU06402
K08246
4-Methylzymosterol-carboxylate
Obtusifoliol
NCU02693
NCU02624
3-Keto-4-methyl-zymosterol
δ8,14-Sterol
NCU05991
erg-3
4-Methylzymosterol
4α-Methylfecosterol
NCU00346
Cholesta-8-en-3β-ol
K09828
Zymosterol
NCU02502...
Fecosterol
24-Methylene-lophenol
K08242
24-Ethylidene-lophenol
NCU00346
NCU00346
erg-1
K14424
K14424
Lathosterol
K09828
Cholesta-7,24-dien-3β-ol
Episterol
Episterol
δ7-Avenasterol
NCU04983...
NCU04983...
NCU04983...
NCU04983...
Vitamin D3
7-Dehydrocholesterol
K09828
7-Dehydro-desmosterol
5,7,24(28)-Ergosta-trienol
5-Dehydroepisterol
5-Dehydroavenasterol
hν
K07419
K21146
K00213
NCU05278
K00213
K00213
Cholesterol ester
NCU02148...
K09828
Desmosterol
5,7,22,24(28)-Ergosta-tetraenol
24-Methylenecholesterol
Isofucosterol
Calcidiol
NCU03991...
Cholesterol
NCU01333
K09828
K09828
K09828
K07438
K21146
2.3.1.73
Steroid hormone biosynthesis
Campesterol
24-epi-Campesterol
Sitosterol
Calcitriol
Steroid degradation
Ergosterol
K09832
K09832
K07436
K07436
hν
Primary bile acid biosynthesis
Brassicasterol
Stigmasterol
Calcitetrol
Secalciferol
Vitamin D2
Brassinosteroid biosynthesis
Phytosterol
00100 11/22/17
(c) Kanehisa Laboratories

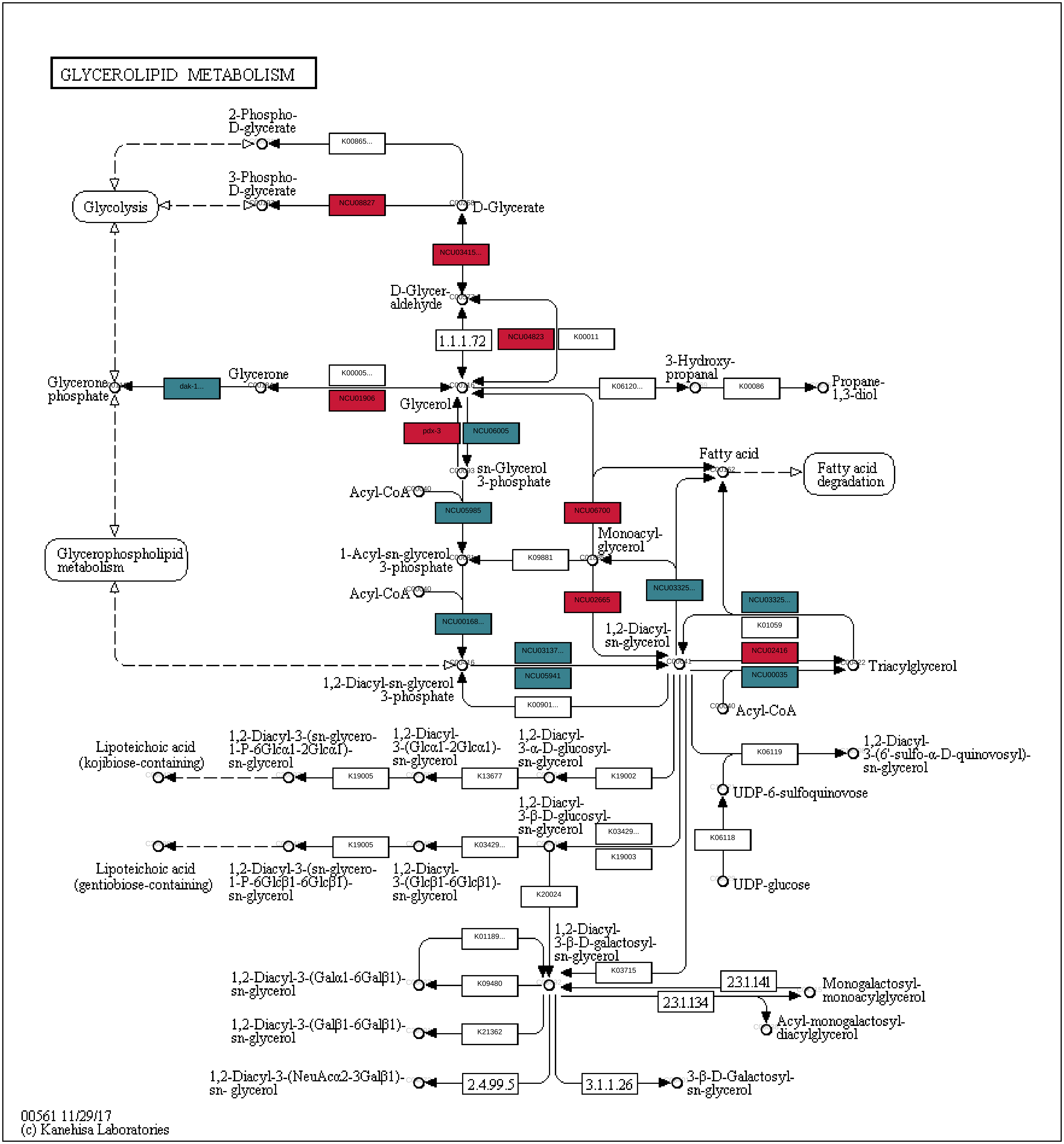
GLYCEROLIPID METABOLISM
2-Phospho-D-glycerate
K00865...
3-Phospho-D-glycerate
NCU08827
D-Glycerate
Glycolysis
NCU03415...
D-Glyceraldehyde
1.1.1.72
NCU04823
K00011
Glycerone phosphate
dak-1...
Glycerone
K00005...
NCU01906
Glycerol
K06120...
3-Hydroxypropanal
K00086
Propane-1,3-diol
pdx-3
NCU06005
sn-Glycerol 3-phosphate
Fatty acid
Fatty acid degradation
Acyl-CoA
NCU05985
NCU06700
Monoacylglycerol
Glycerophospholipid metabolism
1-Acyl-sn-glycerol 3-phosphate
K09881
NCU03325...
NCU02665
NCU00168...
1,2-Diacyl-sn-glycerol
NCU03325...
K01059
NCU02416
NCU03137...
NCU05941
NCU00035
Triacylglycerol
1,2-Diacyl-sn-glycerol 3-phosphate
K00901...
Acyl-CoA
Lipoteichoic acid (kojibiose-containing)
1,2-Diacyl-3-(sn-glycero-1-P-6Glcα1-2Glcα1)-sn-glycerol
1,2-Diacyl-3-(Glcα1-2Glcα1)-sn-glycerol
1,2-Diacyl-3-α-D-glucosyl-sn-glycerol
K19005
K13677
K19002
K06119
1,2-Diacyl-3-(6'-sulfo-α-D-quinovosyl)-sn-glycerol
UDP-6-sulfoquinovose
1,2-Diacyl-3-β-D-glucosyl-sn-glycerol
K19005
K03429...
K03429...
K19003
K06118
Lipoteichoic acid (gentiobiose-containing)
1,2-Diacyl-3-(sn-glycero-1-P-6Glcβ1-6Glcβ1)-sn-glycerol
1,2-Diacyl-3-(Glcβ1-6Glcβ1)-sn-glycerol
K20024
UDP-glucose
K01189...
1,2-Diacyl-3-β-D-galactosyl-sn-glycerol
K03715
1,2-Diacyl-3-(Galα1-6Galβ1)-sn-glycerol
K09480
2.3.1.141
Monogalactosylmonoacylglycerol
2.3.1.134
Acyl-monogalactosyl-diacylglycerol
1,2-Diacyl-3-(Galβ1-6Galβ1)-sn-glycerol
K21362
1,2-Diacyl-3-(NeuAcα2-3Galβ1)-sn- glycerol
2.4.99.5
3.1.1.26
3-β-D-Galactosyl-sn-glycerol
00561 11/29/17
(c) Kanehisa Laboratories

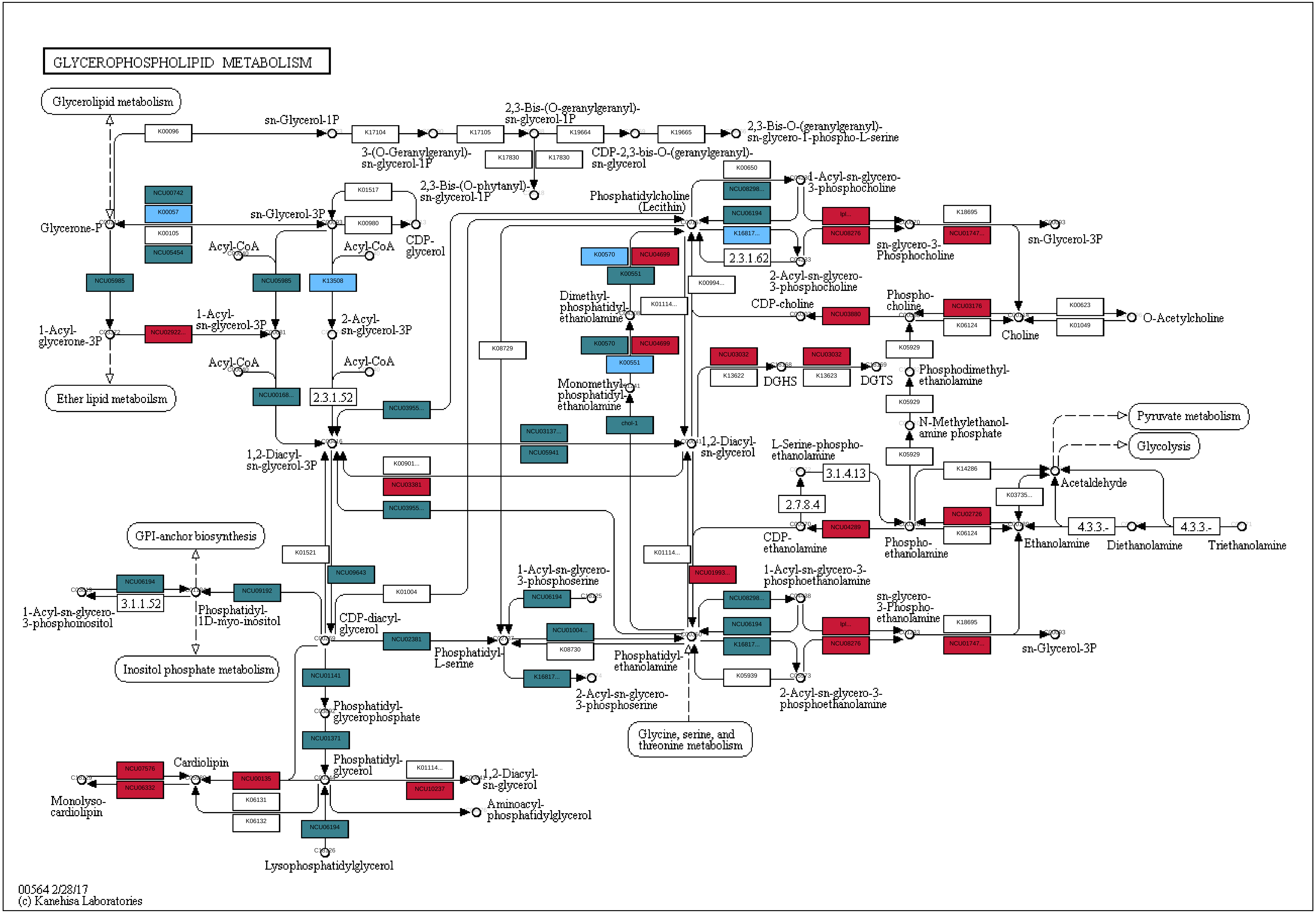
GLYCEROPHOSPHOLIPID METABOLISM
Glycerolipid metabolism
Glycerone-P
1-Acyl-glycerone-3P
Ether lipid metaboilsm
sn-Glycerol-1P
3-(O-Geranylgeranyl)-sn-glycerol-1P
2,3-Bis-(O-geranylgeranyl)-sn-glycerol-1P
2,3-Bis-O-(geranylgeranyl)-sn-glycero-1-phospho-L-serine
CDP-2,3-bis-O-(geranylgeranyl)-sn-glycerol
2,3-Bis-(O-phytanyl)-sn-glycerol-1P
sn-Glycerol-3P
Acyl-CoA
CDP-glycerol
1-Acyl-sn-glycerol-3P
2-Acyl-sn-glycerol-3P
2.3.1.52
1,2-Diacyl-sn-glycerol-3P
Phosphatidylcholine (Lecithin)
1-Acyl-sn-glycero-3-phosphocholine
sn-glycero-3-Phosphocholine
2-Acyl-sn-glycero-3-phosphocholine
2.3.1.62
Dimethyl-phosphatidyl-ethanolamine
Monomethyl-phosphatidyl-ethanolamine
CDP-choline
Phospho-choline
Choline
O-Acetylcholine
DGHS
DGTS
Phosphodimethyl-ethanolamine
N-Methylethanol-amine phosphate
1,2-Diacyl-sn-glycerol
L-Serine-phospho-ethanolamine
3.1.4.13
2.7.8.4
Pyruvate metabolism
Glycolysis
Acetaldehyde
CDP-ethanolamine
Phospho-ethanolamine
Ethanolamine
4.3.3.-
Diethanolamine
Triethanolamine
GPI-anchor biosynthesis
1-Acyl-sn-glycero-3-phosphoinositol
3.1.1.52
Phosphatidyl-1D-myo-inositol
Inositol phosphate metabolism
CDP-diacyl-glycerol
1-Acyl-sn-glycero-3-phosphoserine
Phosphatidyl-L-serine
2-Acyl-sn-glycero-3-phosphoserine
Phosphatidyl-ethanolamine
1-Acyl-sn-glycero-3-phosphoethanolamine
sn-glycero-3-Phospho-ethanolamine
2-Acyl-sn-glycero-3-phosphoethanolamine
Glycine, serine, and threonine metabolism
Phosphatidyl-glycerophosphate
Phosphatidyl-glycerol
Cardiolipin
Monolyso-cardiolipin
1,2-Diacyl-sn-glycerol
Aminoacyl-phosphatidylglycerol
Lysophosphatidylglycerol
K00096
K17104
K17105
K19664
K19665
K17830
K00650
NCU00742
K00057
K00105
NCU05454
K01517
K00980
NCU05985
K13508
NCU02922...
NCU00168...
NCU03955...
NCU08298...
NCU06194
K16817...
lpl...
NCU08276
K18695
NCU01747...
K00570
NCU04699
K00551
K00994...
K01114...
NCU03880
NCU03176
K06124
K00623
K01049
NCU03032
K13622
K13623
K05929
chol-1
NCU03137...
NCU05941
K08729
K00901...
NCU03381
K14286
K03735...
NCU02726
NCU04289
K01521
NCU09643
NCU09192
K01004
NCU01993...
NCU02381
NCU01004...
K08730
K05939
NCU01141
NCU01371
NCU07576
NCU06332
NCU00135
K06131
K06132
NCU10237
00564 2/28/17
(c) Kanehisa Laboratories

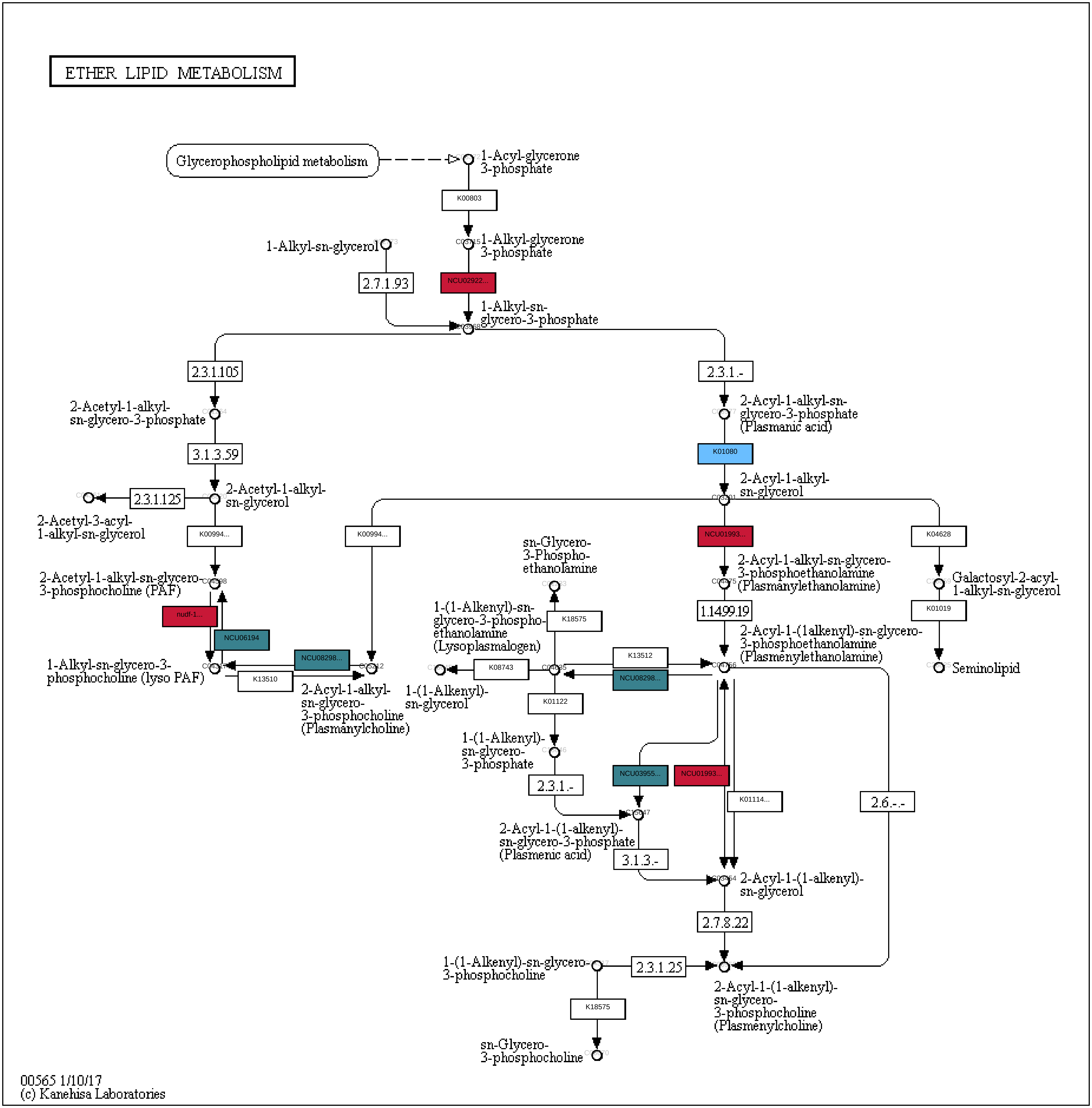

ETHER LIPID METABOLISM
Glycerophospholipid metabolism
1-Acyl-glycerone 3-phosphate
K00803
1-Alkyl-sn-glycerol
1-Alkyl-glycerone 3-phosphate
2.7.1.93
NCU02922...
1-Alkyl-sn-glycero-3-phosphate
2.3.1.105
2.3.1.-
2-Acetyl-1-alkyl-sn-glycero-3-phosphate
2-Acyl-1-alkyl-sn-glycero-3-phosphate (Plasmanic acid)
3.1.3.59
K01080
2.3.1.125
2-Acetyl-1-alkyl-sn-glycerol
2-Acyl-1-alkyl-sn-glycerol
2-Acetyl-3-acyl-1-alkyl-sn-glycerol
K00994...
K00994...
NCU01993...
K04628
sn-Glycero-3-Phospho-ethanolamine
2-Acyl-1-alkyl-sn-glycero-3-phosphoethanolamine (Plasmanylethanolamine)
2-Acetyl-1-alkyl-sn-glycero-3-phosphocholine (PAF)
Galactosyl-2-acyl-1-alkyl-sn-glycerol
1-(1-Alkenyl)-sn-glycero-3-phospho-ethanolamine (Lysoplasmalogen)
K18575
1.14.99.19
K01019
nudf-1...
NCU06194
2-Acyl-1-(1alkenyl)-sn-glycero-3-phosphoethanolamine (Plasmenylethanolamine)
NCU08298...
K13512
1-Alkyl-sn-glycero-3-phosphocholine (lyso PAF)
K13510
K08743
NCU08298...
Seminolipid
2-Acyl-1-alkyl-sn-glycero-3-phosphocholine (Plasmanylcholine)
1-(1-Alkenyl)-sn-glycerol
K01122
1-(1-Alkenyl)-sn-glycero-3-phosphate
2.3.1.-
NCU03955...
NCU01993...
K01114...
2.6.-.-
2-Acyl-1-(1-alkenyl)-sn-glycero-3-phosphate (Plasmenic acid)
3.1.3.-
2-Acyl-1-(1-alkenyl)-sn-glycerol
2.7.8.22
1-(1-Alkenyl)-sn-glycero-3-phosphocholine
2.3.1.25
2-Acyl-1-(1-alkenyl)-sn-glycero-3-phosphocholine (Plasmenylcholine)
K18575
sn-Glycero-3-phosphocholine
00565 1/10/17
(c) Kanehisa Laboratories

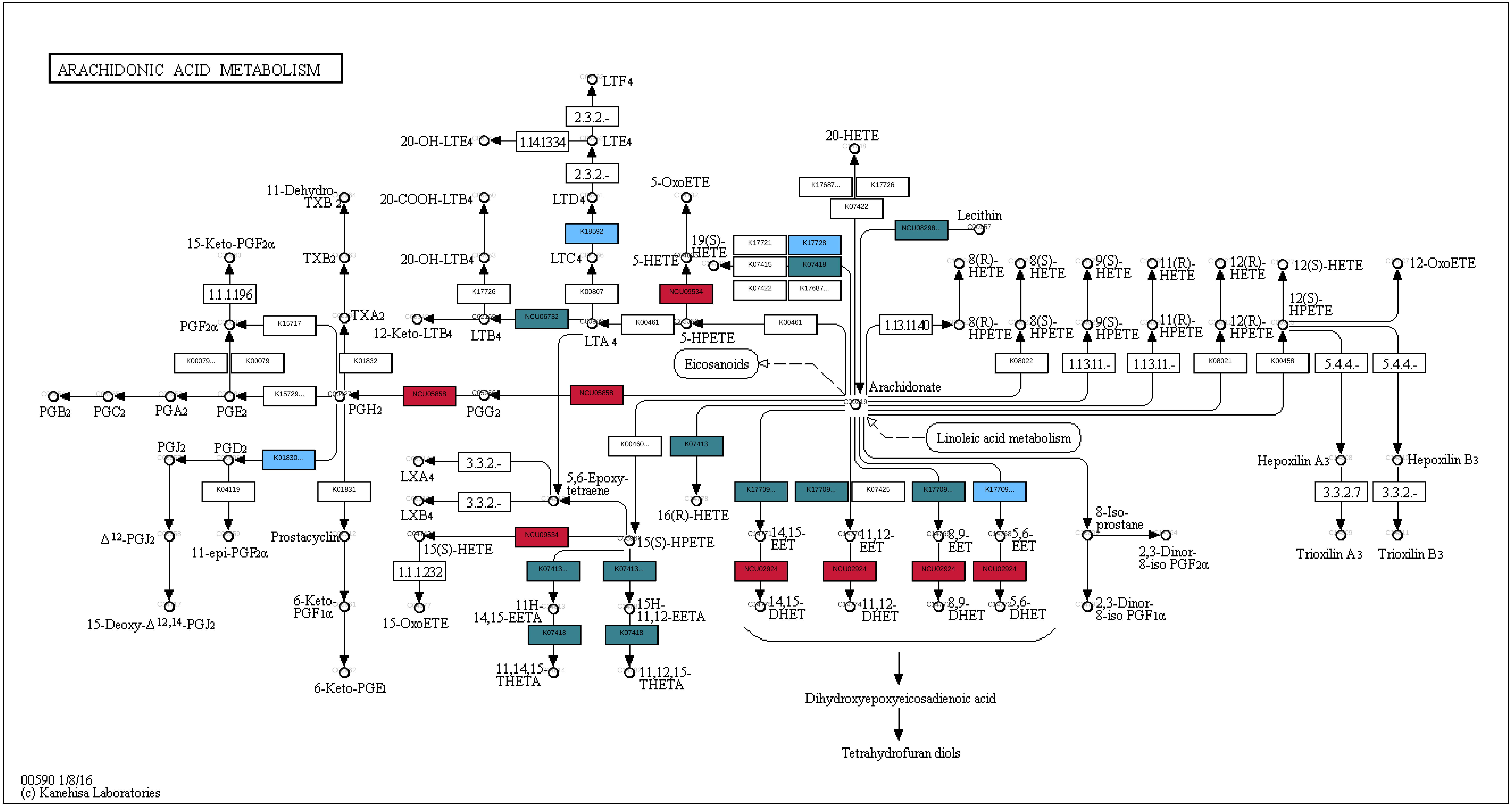
ARACHIDONIC ACID METABOLISM
LTF4
2.3.2.-
20-OH-LTE4
1.14.13.34
LTE4
2.3.2.-
20-HETE
K17687...
K17726
K07422
5-OxoETE
11-Dehydro-TXB 2
20-COOH-LTB4
LTD4
Lecithin
NCU08298...
K18592
19(S)-HETE
K17721
K17728
15-Keto-PGF2α
TXB2
20-OH-LTB4
LTC4
5-HETE
K07415
K07418
8(R)-HETE
8(S)-HETE
9(S)-HETE
11(R)-HETE
12(R)-HETE
12(S)-HETE
12-OxoETE
K17726
K00807
NCU09534
K07422
K17687...
1.1.1.196
12(S)-HPETE
TXA2
NCU06732
PGF2α
K15717
12-Keto-LTB4
LTB4
LTA 4
K00461
5-HPETE
K00461
1.13.11.40
8(R)-HPETE
8(S)-HPETE
9(S)-HPETE
11(R)-HPETE
12(R)-HPETE
K00079...
K00079
K01832
Eicosanoids
K08022
1.13.11.-
1.13.11.-
K08021
K00458
5.4.4.-
5.4.4.-
PGB2
PGC2
PGA2
PGE2
K15729...
NCU05858
PGH2
PGG2
NCU05858
Arachidonate
PGJ2
PGD2
K01830...
K00460...
K07413
Linoleic acid metabolism
Hepoxilin A3
Hepoxilin B3
LXA4
3.3.2.-
5,6-Epoxy-tetraene
K04119
K01831
K17709...
K17709...
K07425
K17709...
K17709...
3.3.2.7
3.3.2.-
LXB4
3.3.2.-
16(R)-HETE
8-Iso-prostane
Δ12-PGJ2
11-epi-PGF2α
Prostacyclin
NCU09534
15(S)-HETE
15(S)-HPETE
14,15-EET
11,12-EET
8,9-EET
5,6-EET
2,3-Dinor-8-iso PGF2α
Trioxilin A3
Trioxilin B3
1.1.1.232
K07413...
K07413...
NCU02924
NCU02924
NCU02924
NCU02924
6-Keto-PGF1α
11H-14,15-EETA
15H-11,12-EETA
14,15-DHET
11,12-DHET
8,9-DHET
5,6-DHET
2,3-Dinor-8-iso PGF1α
15-Deoxy-Δ12,14-PGJ2
15-OxoETE
K07418
K07418
11,14,15-THETA
11,12,15-THETA
6-Keto-PGE1
Dihydroxyepoxyeicosadienoic acid
Tetrahydrofuran diols
00590 1/8/16
(c) Kanehisa Laboratories

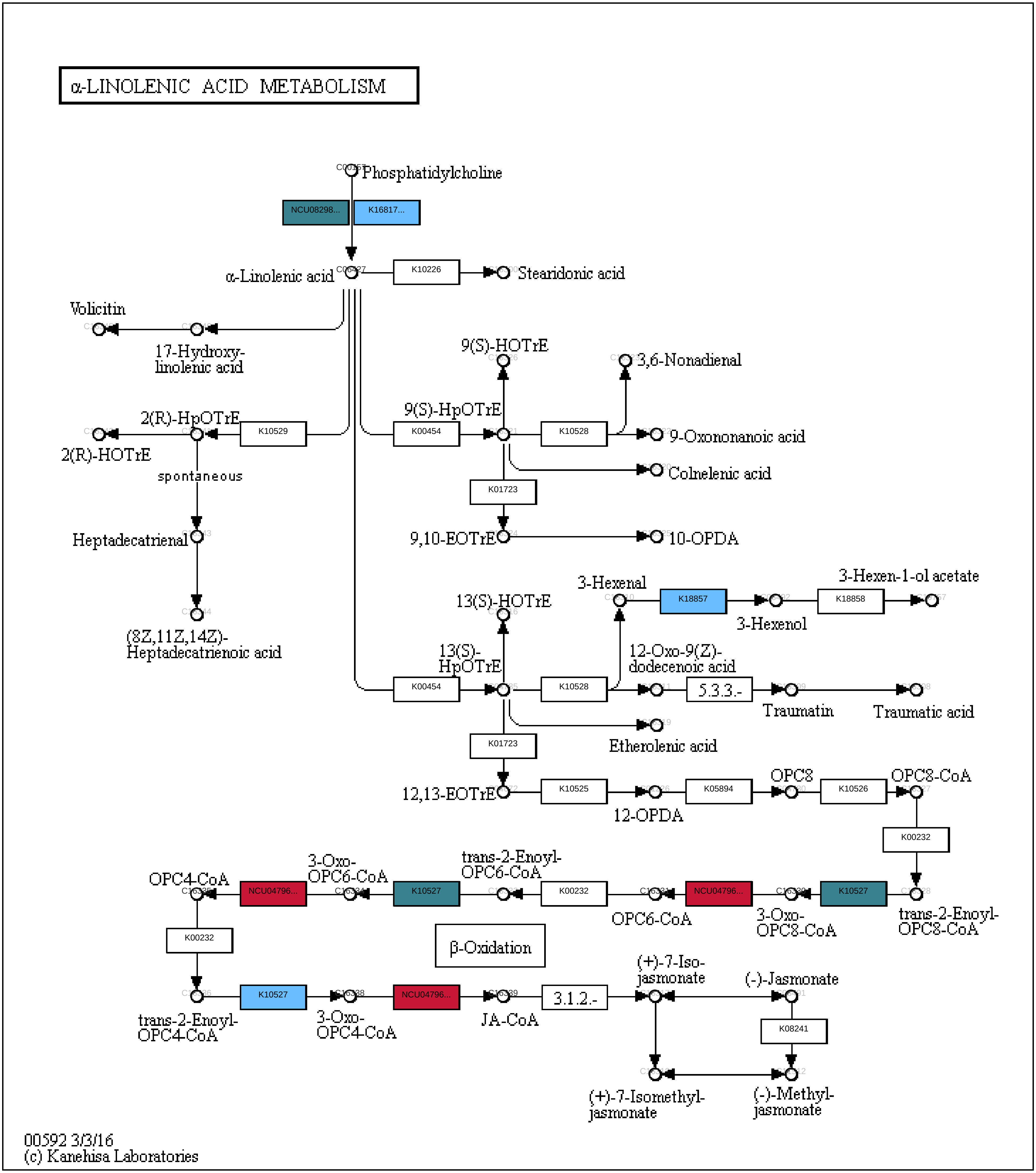
α-LINOLENIC ACID METABOLISM
Phosphatidylcholine
NCU08298...
K16817...
α-Linolenic acid
K10226
Stearidonic acid
Volicitin
17-Hydroxy-linolenic acid
9(S)-HOTrE
3,6-Nonadienal
9(S)-HpOTrE
2(R)-HpOTrE
K10529
2(R)-HOTrE
K00454
K10528
9-Oxononanoic acid
spontaneous
Colnelenic acid
K01723
Heptadecatrienal
9,10-EOTrE
10-OPDA
3-Hexenal
K18857
3-Hexen-1-ol acetate
K18858
13(S)-HOTrE
3-Hexenol
(8Z,11Z,14Z)-Heptadecatrienoic acid
13(S)-HpOTrE
12-Oxo-9(Z)-dodecenoic acid
K00454
K10528
5.3.3.-
Traumatin
Traumatic acid
Etherolenic acid
K01723
12,13-EOTrE
K10525
K05894
OPC8
K10526
OPC8-CoA
12-OPDA
K00232
OPC4-CoA
3-Oxo-OPC6-CoA
trans-2-Enoyl-OPC6-CoA
NCU04796...
K10527
K00232
NCU04796...
K10527
OPC6-CoA
3-Oxo-OPC8-CoA
trans-2-Enoyl-OPC8-CoA
K00232
β-Oxidation
(+)-7-Iso-jasmonate
(-)-Jasmonate
K10527
NCU04796...
3.1.2.-
K08241
trans-2-Enoyl-OPC4-CoA
3-Oxo-OPC4-CoA
JA-CoA
(+)-7-Isomethyl-jasmonate
(-)-Methyl-jasmonate
00592 3/3/16
(c) Kanehisa Laboratories

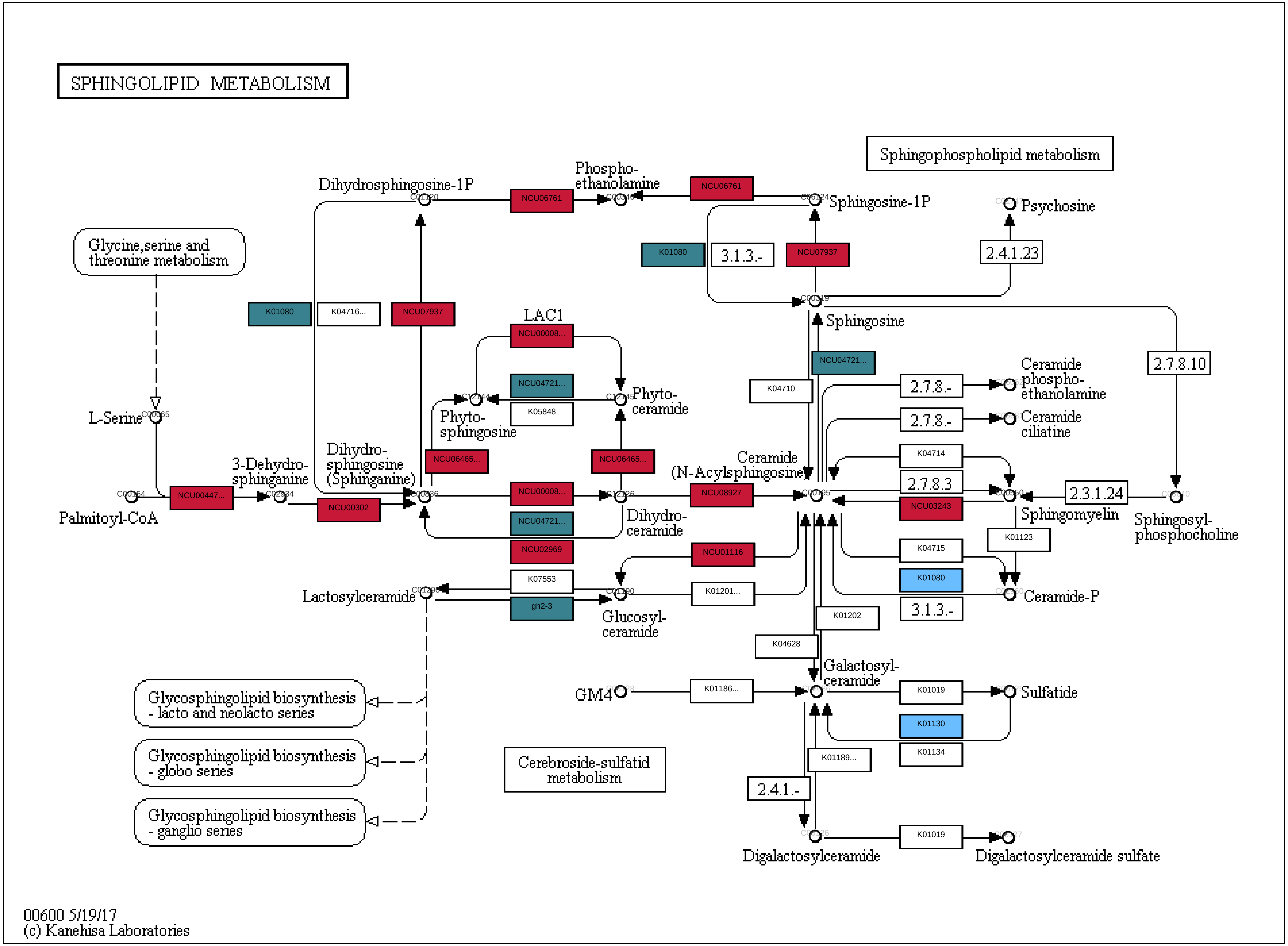
SPHINGOLIPID METABOLISM
Sphingophospholipid metabolism
Glycine,serine and threonine metabolism
Dihydrosphingosine-1P
Phospho-ethanolamine
NCU06761
NCU06761
Sphingosine-1P
Psychosine
K01080
K04716...
NCU07937
K01080
3.1.3.-
NCU07937
2.4.1.23
LAC1
NCU00008...
Sphingosine
NCU04721...
NCU04721...
Phyto-ceramide
K05848
Phyto-sphingosine
K04710
2.7.8.-
Ceramide phospho-ethanolamine
2.7.8.-
Ceramide ciliatine
2.7.8.10
L-Serine
3-Dehydro-sphinganine
Dihydro-sphingosine (Sphinganine)
NCU06465...
NCU06465...
Ceramide (N-Acylsphingosine)
K04714
2.7.8.3
NCU03243
2.3.1.24
Palmitoyl-CoA
NCU00447...
NCU00302
NCU00008...
NCU04721...
NCU08927
Dihydro-ceramide
Sphingomyelin
Sphingosyl-phosphocholine
NCU02969
K01123
NCU01116
K04715
K01080
K07553
Lactosylceramide
gh2-3
K01201...
Glucosyl-ceramide
Ceramide-P
3.1.3.-
K01202
K04628
Galactosyl-ceramide
GM4
K01186...
K01019
Sulfatide
K01130
K01134
K01189...
Glycosphingolipid biosynthesis - lacto and neolacto series
Glycosphingolipid biosynthesis - globo series
Glycosphingolipid biosynthesis - ganglio series
Cerebroside-sulfatid metabolism
2.4.1.-
Digalactosylceramide
K01019
Digalactosylceramide sulfate
00600 5/19/17
(c) Kanehisa Laboratories

# 4. Nucleotide metabolism

| MAP | PATHWAY |
|---|---|
| **230** | Purine metabolism |
| **240** | Pyrimidine metabolism |

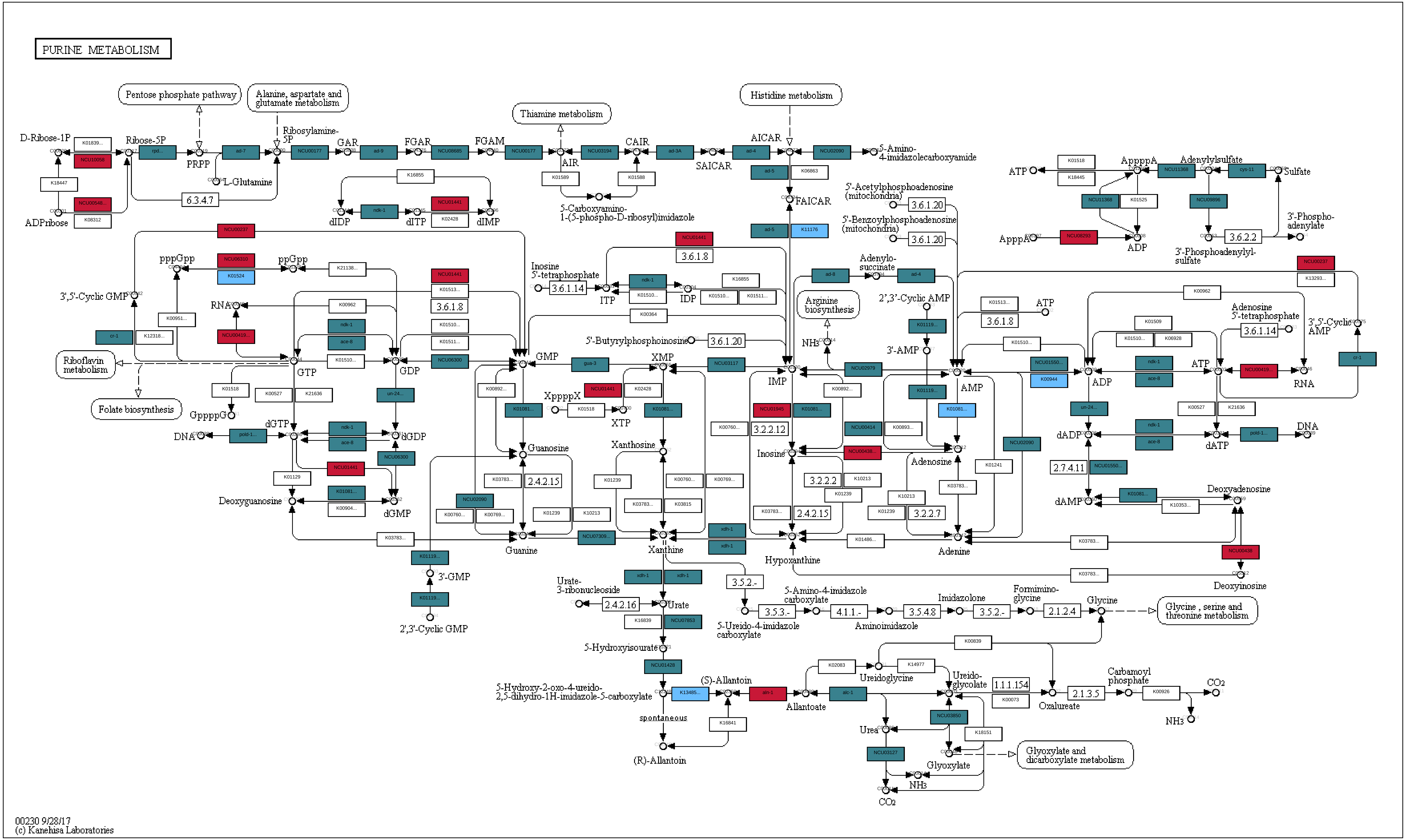
PURINE METABOLISM
Pentose phosphate pathway
Alanine, aspartate and glutamate metabolism
Thiamine metabolism
Histidine metabolism
D-Ribose-1P
Ribose-5P
PRPP
Ribosylamine-5P
L-Glutamine
ADPRibose
GAR
FGAR
FGAM
AIR
CAIR
SAICAR
AICAR
5-Amino-4-imidazolecarboxyamide
FAICAR
5-Carboxyamino-1-(5-phospho-D-ribosyl)imidazole
dIDP
dITP
dIMP
ATP
ApppA
Adenylylsulfate
Sulfate
ADP
AppppA
5'-Acetylphosphoadenosine (mitochondria)
5'-Benzoylphosphoadenosine (mitochondria)
3'-Phospho-adenylate
3'-Phosphoadenylyl-sulfate
pppGpp
ppGpp
3',5'-Cyclic GMP
RNA
Riboflavin metabolism
Folate biosynthesis
GppppG
GMP
GDP
GTP
dGTP
dGDP
dGMP
DNA
Deoxyguanosine
Guanosine
Guanine
3'-GMP
2',3'-Cyclic GMP
Inosine 5'-tetraphosphate
ITP
IDP
XppppX
XTP
XMP
Xanthosine
Xanthine
5'-Butyrylphosphoinosine
IMP
Inosine
Hypoxanthine
NH3
Arginine biosynthesis
Adenylo-succinate
2',3'-Cyclic AMP
3'-AMP
AMP
Adenosine
Adenine
Adenosine 5'-tetraphosphate
3',5'-Cyclic AMP
dADP
dATP
dAMP
Deoxyadenosine
Deoxyinosine
Urate-3-ribonucleoside
Urate
5-Hydroxyisourate
5-Hydroxy-2-oxo-4-ureido-2,5-dihydro-1H-imidazole-5-carboxylate
spontaneous
(S)-Allantoin
(R)-Allantoin
Allantoate
5-Amino-4-imidazole carboxylate
5-Ureido-4-imidazole carboxylate
Aminoimidazole
Imidazolone
Formimino-glycine
Glycine
Glycine, serine and threonine metabolism
Ureidoglycine
Ureido-glycolate
Oxalureate
Carbamoyl phosphate
CO2
Urea
Glyoxylate
Glyoxylate and dicarboxylate metabolism
00230 9/28/17
(c) Kanehisa Laboratories

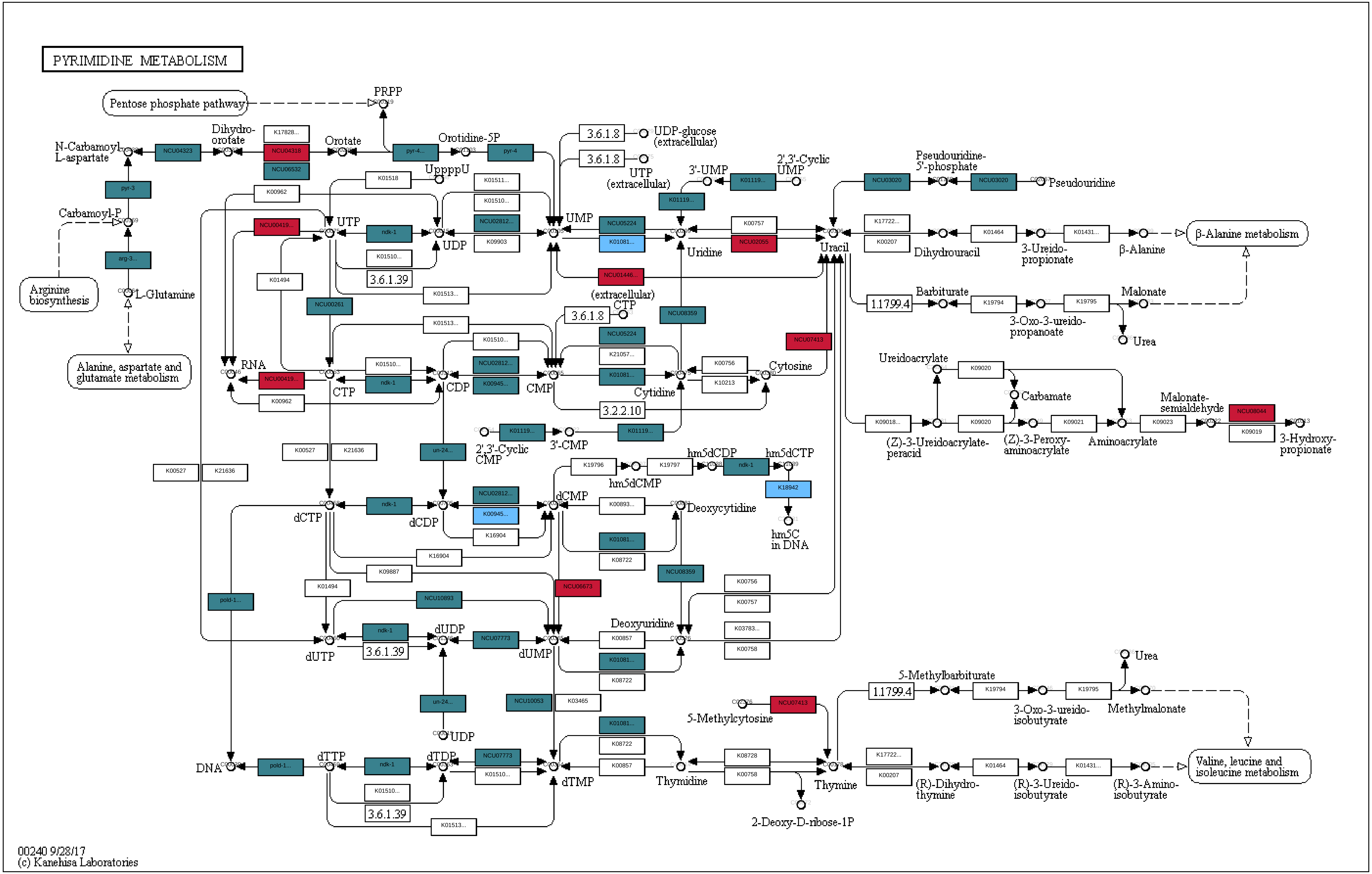
PYRIMIDINE METABOLISM
Pentose phosphate pathway
PRPP
N-Carbamoyl-L-aspartate
Dihydro-orotate
Orotate
Orotidine-5P
NCU04323
K17828...
NCU04318
NCU06532
pyr-4...
pyr-4
pyr-3
Carbamoyl-P
arg-3...
Arginine biosynthesis
L-Glutamine
Alanine, aspartate and glutamate metabolism
3.6.1.8
UDP-glucose (extracellular)
UTP (extracellular)
UppppU
K01518
K00962
K01511...
K01510...
NCU02812...
K09903
NCU00419...
ndk-1
UTP
UDP
UMP
K01510...
3.6.1.39
K01494
K01513...
NCU00261
3'-UMP
2',3'-Cyclic UMP
NCU01119...
NCU05224
K01081...
K00757
NCU02055
Uridine
NCU01446...
(extracellular)
Pseudouridine-5'-phosphate
NCU03020
Pseudouridine
Uracil
K17722...
K00207
Dihydrouracil
K01464
3-Ureido-propionate
K01431...
β-Alanine
β-Alanine metabolism
1.17.99.4
Barbiturate
K19794
3-Oxo-3-ureido-propanoate
K19795
Malonate
Urea
CTP
NCU08359
NCU07413
K21057...
K00756
K10213
Cytosine
Cytidine
3.2.2.10
RNA
CTP
CDP
CMP
K00945...
K01510...
K00962
Ureidoacrylate
K09020
Carbamate
K09018...
K09021
Aminoacrylate
K09023
Malonate-semialdehyde
NCU08044
K09019
3-Hydroxy-propionate
(Z)-3-Ureidoacrylate-peracid
(Z)-3-Peroxy-aminoacrylate
2',3'-Cyclic CMP
3'-CMP
K00527
K21636
un-24...
K19796
K19797
hm5dCDP
hm5dCTP
hm5dCMP
K18942
hm5C in DNA
dCMP
dCTP
dCDP
K16904
K00893...
Deoxycytidine
K08722
K09887
K01494
NCU06673
NCU10893
pold-1...
dUDP
dUTP
dUMP
NCU07773
Deoxyuridine
K00857
K03783...
K00758
NCU10053
K03465
UDP
5-Methylcytosine
5-Methylbarbiturate
Methylmalonate
3-Oxo-3-ureido-isobutyrate
Urea
DNA
dTTP
dTDP
dTMP
K08728
Thymidine
Thymine
2-Deoxy-D-ribose-1P
(R)-Dihydro-thymine
(R)-3-Ureido-isobutyrate
(R)-3-Amino-isobutyrate
Valine, leucine and isoleucine metabolism
00240 9/28/17
(c) Kanehisa Laboratories

# 5. Amino Acid metabolism

| MAP | PATHWAY |
|---|---|
| **220** | Arginine biosynthesis |
| **250** | Alanine, aspartate and glutamate metabolism |
| **260** | Glycine, serine and threonine metabolism |
| **270** | Cysteine and methionine metabolism |
| **280** | Valine, leucine and isoleucine degradation |
| **290** | Valine, leucine and isoleucine biosynthesis |
| **300** | Lysine biosynthesis |
| **310** | Lysine degradation |
| **330** | Arginine and proline metabolism |
| **340** | Histidine metabolism |
| **350** | Tyrosine metabolism |
| **360** | Phenylalanine metabolism |
| **380** | Tryptophan metabolism |
| **400** | Phenylalanine, tyrosine and tryptophan biosynthesis |

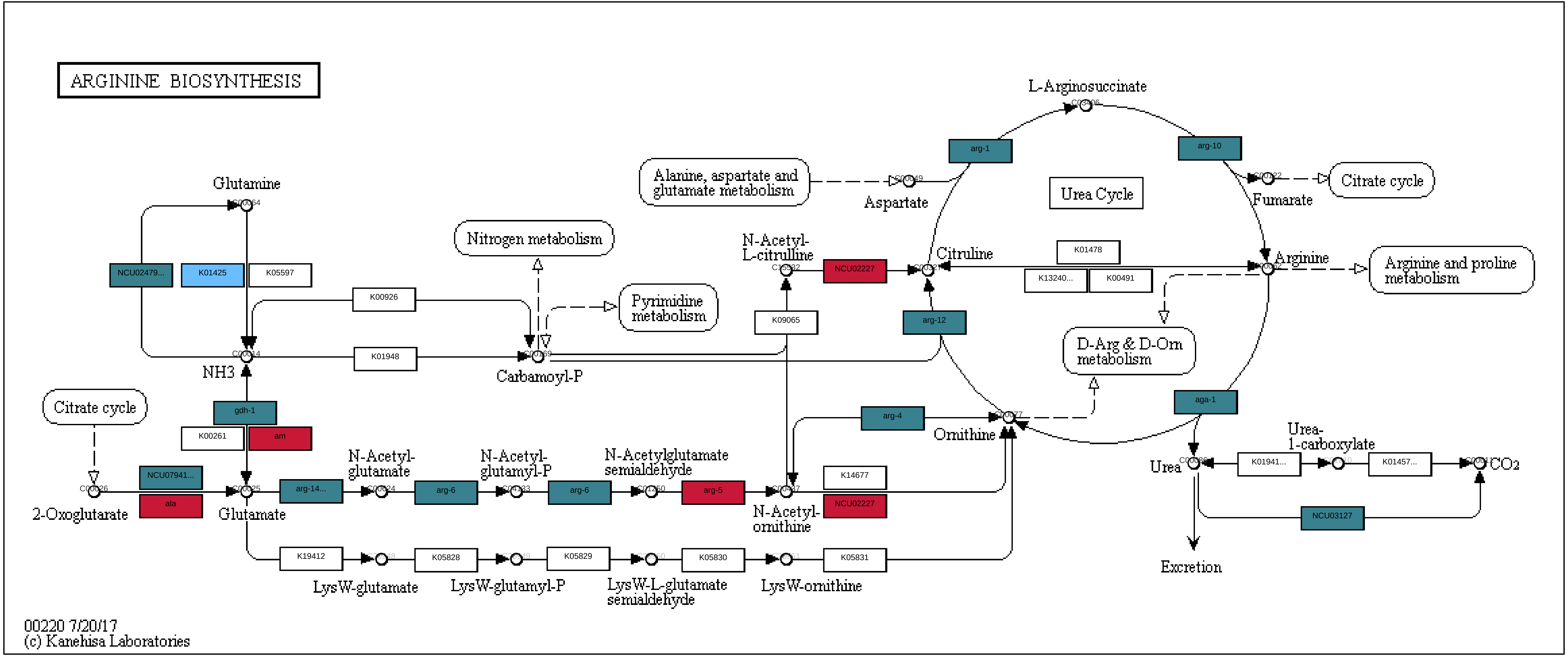
ARGININE BIOSYNTHESIS
L-Arginosuccinate
arg-1
arg-10
Alanine, aspartate and glutamate metabolism
Aspartate
Urea Cycle
Citrate cycle
Fumarate
Glutamine
Nitrogen metabolism
N-Acetyl-L-citrulline
Citruline
K01478
Arginine
Arginine and proline metabolism
NCU02479...
K01425
K05597
NCU02227
K13240...
K00491
K00926
Pyrimidine metabolism
K09065
arg-12
D-Arg & D-Orn metabolism
K01948
NH3
Carbamoyl-P
Citrate cycle
gdh-1
arg-4
aga-1
K00261
am
Ornithine
Urea-1-carboxylate
NCU07941...
N-Acetyl-glutamate
N-Acetyl-glutamyl-P
N-Acetylglutamate semialdehyde
K14677
Urea
K01941...
K01457...
CO2
arg-14...
arg-6
arg-6
arg-5
ala
NCU02227
2-Oxoglutarate
Glutamate
N-Acetyl-ornithine
NCU03127
Excretion
K19412
K05828
K05829
K05830
K05831
LysW-glutamate
LysW-glutamyl-P
LysW-L-glutamate semialdehyde
LysW-ornithine
00220 7/20/17
(c) Kanehisa Laboratories

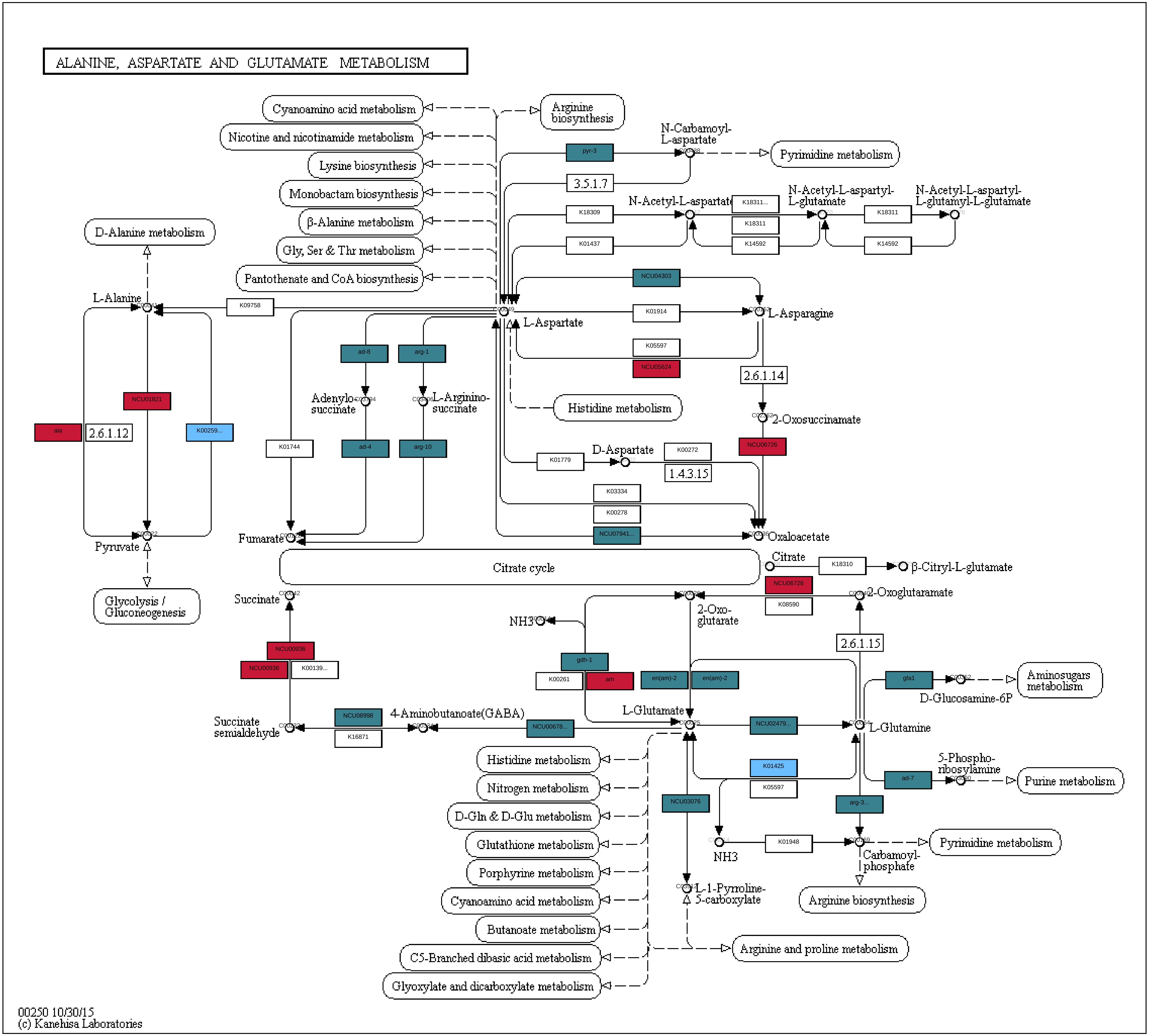
ALANINE, ASPARTATE AND GLUTAMATE METABOLISM
Cyanoamino acid metabolism
Nicotine and nicotinamide metabolism
Lysine biosynthesis
Monobactam biosynthesis
β-Alanine metabolism
Gly, Ser & Thr metabolism
Pantothenate and CoA biosynthesis
Arginine biosynthesis
N-Carbamoyl-L-aspartate
Pyrimidine metabolism
pyr-3
3.5.1.7
N-Acetyl-L-aspartate
N-Acetyl-L-aspartyl-L-glutamate
N-Acetyl-L-aspartyl-L-glutamyl-L-glutamate
K18309
K18311...
K18311
K14592
K01437
D-Alanine metabolism
L-Alanine
K09758
NCU04303
K01914
L-Aspartate
L-Asparagine
K05597
NCU05624
ad-8
arg-1
Adenylo-succinate
L-Arginino-succinate
Histidine metabolism
2.6.1.14
2-Oxosuccinamate
NCU01821
ala
2.6.1.12
K00259...
K01744
ad-4
arg-10
NCU06726
D-Aspartate
K01779
K00272
1.4.3.15
K03334
K00278
NCU07941...
Oxaloacetate
Fumarate
Pyruvate
Citrate cycle
Citrate
K18310
β-Citryl-L-glutamate
Glycolysis / Gluconeogenesis
Succinate
NCU06726
K08590
2-Oxoglutaramate
NH3
2-Oxo-glutarate
2.6.1.15
NCU00936
NCU00936
K00139...
gdh-1
K00261
am
en(am)-2
en(am)-2
gfa1
Aminosugars metabolism
D-Glucosamine-6P
Succinate semialdehyde
NCU08998
K16871
4-Aminobutanoate(GABA)
NCU00678...
L-Glutamate
NCU02479...
L-Glutamine
5-Phospho-ribosylamine
ad-7
Purine metabolism
Histidine metabolism
Nitrogen metabolism
D-Gln & D-Glu metabolism
Glutathione metabolism
Porphyrine metabolism
Cyanoamino acid metabolism
Butanoate metabolism
C5-Branched dibasic acid metabolism
Glyoxylate and dicarboxylate metabolism
K01425
K05597
NCU03076
arg-3...
NH3
K01948
Carbamoyl-phosphate
Pyrimidine metabolism
L-1-Pyrroline-5-carboxylate
Arginine biosynthesis
Arginine and proline metabolism
00250 10/30/15
(c) Kanehisa Laboratories

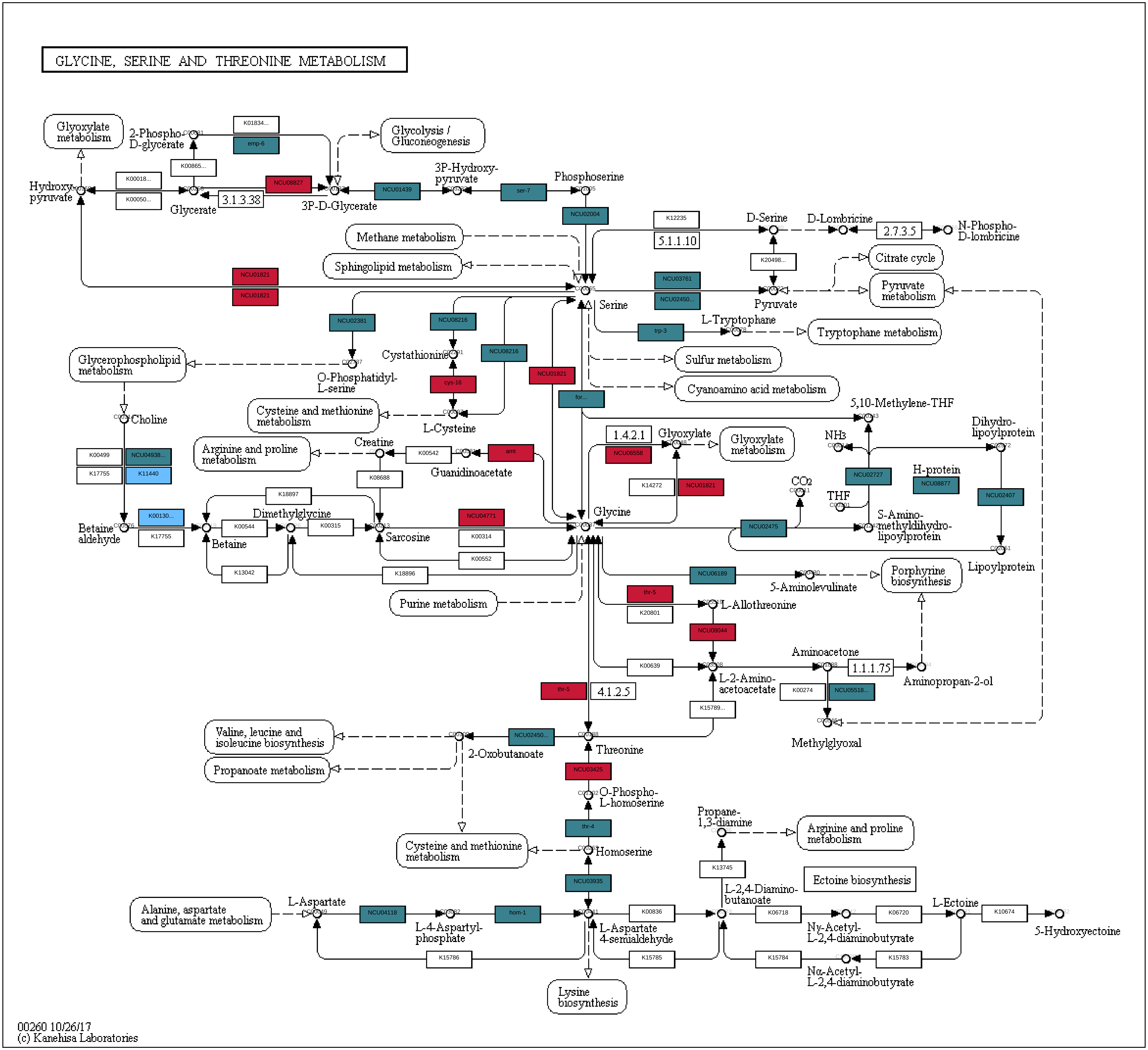
GLYCINE, SERINE AND THREONINE METABOLISM
Glyoxylate metabolism
2-Phospho-D-glycerate
K01834...
emp-6
Glycolysis / Gluconeogenesis
K00865...
K00018...
K00050...
NCU08827
Hydroxy-pyruvate
Glycerate
3.1.3.38
3P-D-Glycerate
NCU01439
3P-Hydroxy-pyruvate
ser-7
Phosphoserine
NCU02004
K12235
5.1.1.10
D-Serine
D-Lombricine
2.7.3.5
N-Phospho-D-lombricine
K20498...
Citrate cycle
Methane metabolism
Sphingolipid metabolism
NCU01821
NCU01821
NCU03761
NCU02450...
Pyruvate
Pyruvate metabolism
Serine
L-Tryptophane
trp-3
Tryptophane metabolism
NCU02381
NCU08216
NCU08216
Cystathionine
NCU01821
Glycerophospholipid metabolism
O-Phosphatidyl-L-serine
cys-16
Sulfur metabolism
for...
Cyanoamino acid metabolism
Cysteine and methionine metabolism
L-Cysteine
5,10-Methylene-THF
Choline
Creatine
Arginine and proline metabolism
K00542
amt
1.4.2.1
Glyoxylate
Glyoxylate metabolism
NCU06558
NH3
Dihydro-lipoylprotein
K00499
NCU04938...
K17755
K11440
K08688
Guanidinoacetate
K14272
NCU01821
NCU02727
H-protein
NCU08877
CO2
THF
NCU02407
K18897
Dimethylglycine
K00130...
K00544
K00315
NCU04771
Glycine
S-Amino-methyldihydro-lipoylprotein
Betaine aldehyde
K17755
Betaine
Sarcosine
K00314
NCU02475
K00552
K13042
K18896
NCU06189
5-Aminolevulinate
Porphyrine biosynthesis
Lipoylprotein
Purine metabolism
thr-5
K20801
L-Allothreonine
NCU08044
Aminoacetone
K00639
1.1.1.75
Aminopropan-2-ol
thr-5
4.1.2.5
L-2-Amino-acetoacetate
K00274
NCU05518...
K15789...
Methylglyoxal
Valine, leucine and isoleucine biosynthesis
NCU02450...
2-Oxobutanoate
Threonine
Propanoate metabolism
NCU03425
O-Phospho-L-homoserine
Propane-1,3-diamine
Arginine and proline metabolism
thr-4
Cysteine and methionine metabolism
Homoserine
K13745
Ectoine biosynthesis
NCU03935
L-2,4-Diamino-butanoate
Alanine, aspartate and glutamate metabolism
L-Aspartate
NCU04118
hom-1
K00836
K06718
K06720
L-Ectoine
K10674
L-4-Aspartyl-phosphate
L-Aspartate 4-semialdehyde
Nγ-Acetyl-L-2,4-diaminobutyrate
5-Hydroxyectoine
K15786
K15785
K15784
K15783
Nα-Acetyl-L-2,4-diaminobutyrate
Lysine biosynthesis
00260 10/26/17
(c) Kanehisa Laboratories

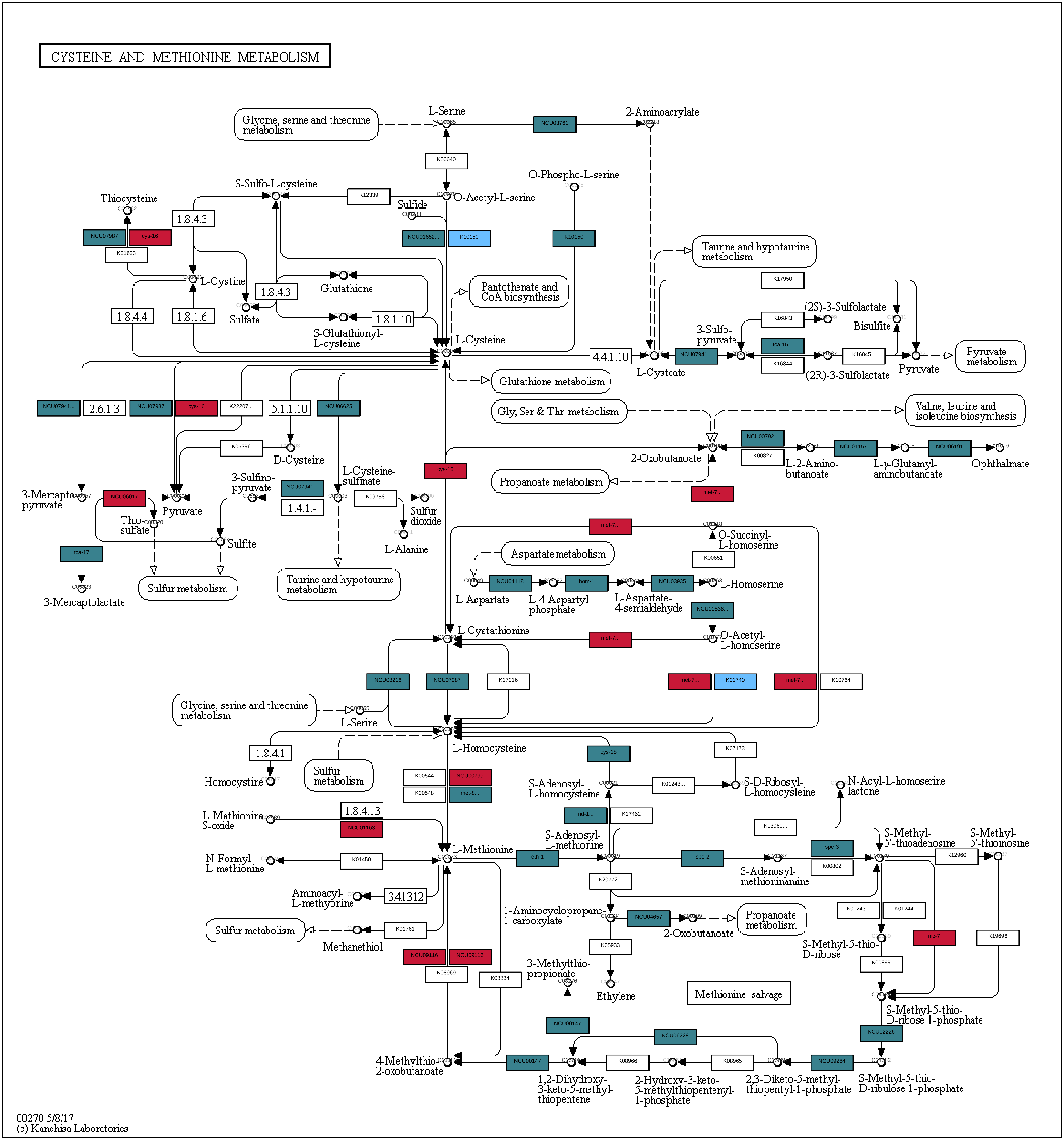
CYSTEINE AND METHIONINE METABOLISM
Glycine, serine and threonine metabolism
L-Serine
NCU03761
2-Aminoacrylate
K00640
O-Phospho-L-serine
S-Sulfo-L-cysteine
K12339
O-Acetyl-L-serine
Thiocysteine
1.8.4.3
Sulfide
NCU07987
cys-16
NCU01652...
K10150
K10150
K21623
Taurine and hypotaurine metabolism
L-Cystine
Glutathione
1.8.4.3
Pantothenate and CoA biosynthesis
K17950
Sulfate
(2S)-3-Sulfolactate
1.8.4.4
1.8.1.6
S-Glutathionyl-L-cysteine
1.8.1.10
K16843
Bisulfite
3-Sulfo-pyruvate
L-Cysteine
tca-15...
4.4.1.10
NCU07941...
K16845...
Pyruvate metabolism
K16844
Pyruvate
L-Cysteate
(2R)-3-Sulfolactate
Glutathione metabolism
NCU07941...
2.6.1.3
NCU07987
cys-16
K22207...
5.1.1.10
NCU06625
Gly, Ser & Thr metabolism
Valine, leucine and isoleucine biosynthesis
NCU00792...
NCU01157...
NCU06191
K05396
K00827
D-Cysteine
2-Oxobutanoate
L-2-Amino-butanoate
L-γ-Glutamyl-aminobutanoate
Ophthalmate
cys-16
Propanoate metabolism
L-Cysteine-sulfinate
3-Sulfino-pyruvate
NCU07941...
3-Mercapto-pyruvate
NCU06017
K09758
met-7...
Pyruvate
1.4.1.-
Sulfur dioxide
Thio-sulfate
met-7...
Sulfite
O-Succinyl-L-homoserine
L-Alanine
K00651
Aspartate metabolism
tca-17
NCU04118
hom-1
NCU03935
L-Homoserine
Sulfur metabolism
Taurine and hypotaurine metabolism
L-Aspartate
L-4-Aspartyl-phosphate
L-Aspartate-4-semialdehyde
3-Mercaptolactate
NCU00536...
L-Cystathionine
O-Acetyl-L-homoserine
met-7...
NCU08216
NCU07987
K17216
met-7...
K01740
met-7...
K10764
Glycine, serine and threonine metabolism
L-Serine
L-Homocysteine
1.8.4.1
cys-18
K07173
Sulfur metabolism
Homocystine
K00544
NCU00799
S-Adenosyl-L-homocysteine
K01243...
S-D-Ribosyl-L-homocysteine
N-Acyl-L-homoserine lactone
K00548
met-8...
1.8.4.13
L-Methionine S-oxide
rid-1...
K17462
NCU01163
K13060...
S-Adenosyl-L-methionine
S-Methyl-5'-thioadenosine
S-Methyl-5'-thioinosine
L-Methionine
N-Formyl-L-methionine
K01450
eth-1
spe-2
spe-3
K12960
K00802
S-Adenosyl-methioninamine
K20772...
Aminoacyl-L-methyonine
3.4.13.12
1-Aminocyclopropane-1-carboxylate
NCU04657
Propanoate metabolism
K01243...
K01244
2-Oxobutanoate
Sulfur metabolism
K01761
nic-7
K19696
Methanethiol
S-Methyl-5-thio-D-ribose
K05933
NCU09116
NCU09116
K00899
3-Methylthio-propionate
K08969
K03334
Ethylene
Methionine salvage
S-Methyl-5-thio-D-ribose 1-phosphate
NCU00147
NCU02226
NCU06228
4-Methylthio-2-oxobutanoate
NCU00147
K08966
K08965
NCU09264
1,2-Dihydroxy-3-keto-5-methyl-thiopentene
2-Hydroxy-3-keto-5-methylthiopentenyl-1-phosphate
2,3-Diketo-5-methyl-thiopentyl-1-phosphate
S-Methyl-5-thio-D-ribulose 1-phosphate
00270 5/8/17
(c) Kanehisa Laboratories

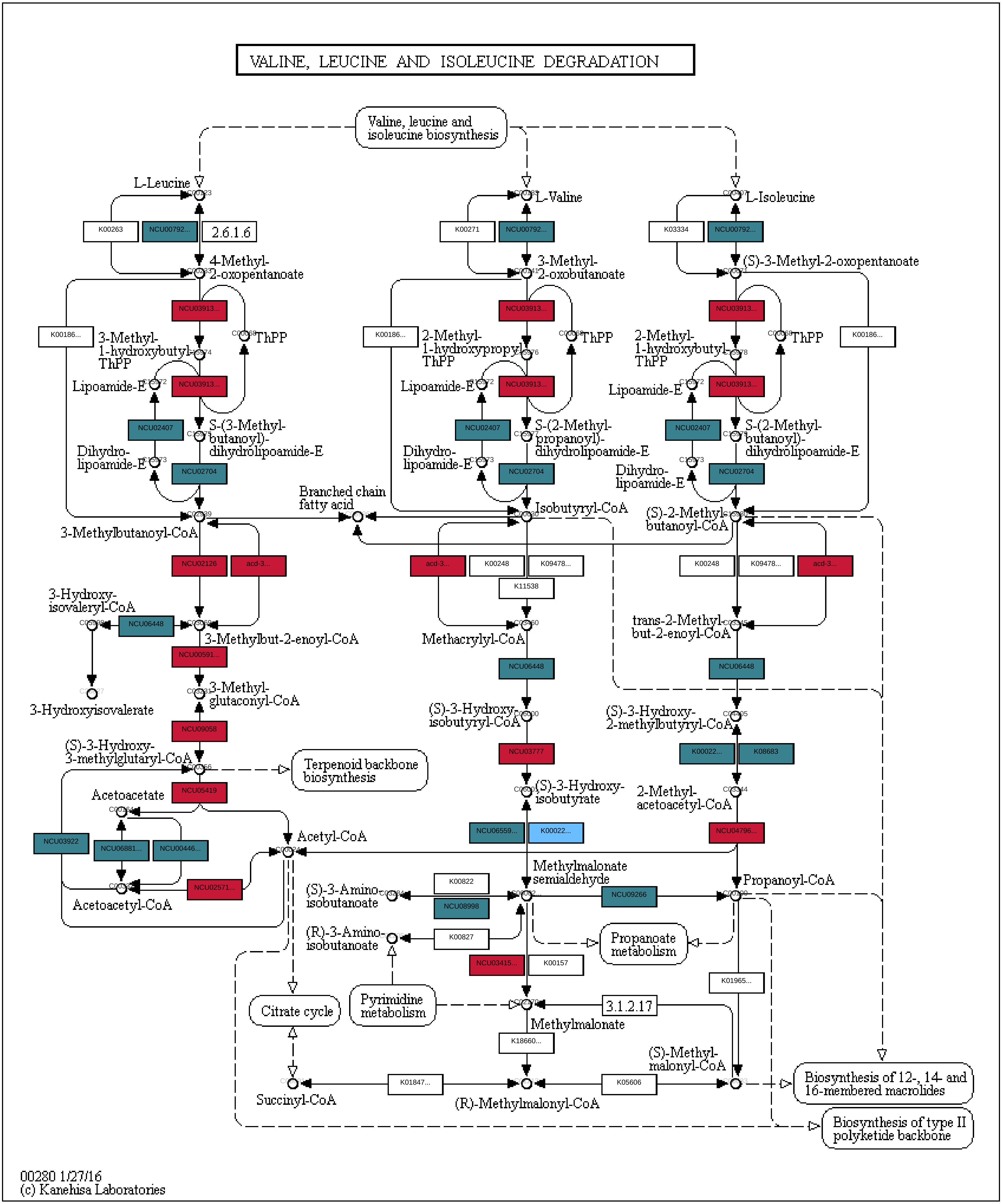
VALINE, LEUCINE AND ISOLEUCINE DEGRADATION
Valine, leucine and isoleucine biosynthesis
L-Leucine
C00123
K00263
NCU00792...
2.6.1.6
4-Methyl-2-oxopentanoate
NCU03913...
K00186...
3-Methyl-1-hydroxybutyl-ThPP
ThPP
Lipoamide-E
NCU02407
S-(3-Methylbutanoyl)-dihydrolipoamide-E
Dihydrolipoamide-E
NCU02704
3-Methylbutanoyl-CoA
Branched chain fatty acid
NCU02126
acd-3...
3-Hydroxyisovaleryl-CoA
NCU06448
3-Methylbut-2-enoyl-CoA
NCU00591...
3-Hydroxyisovalerate
3-Methylglutaconyl-CoA
NCU09058
(S)-3-Hydroxy-3-methylglutaryl-CoA
Terpenoid backbone biosynthesis
NCU05419
Acetoacetate
NCU03922
NCU06881...
NCU00446...
NCU02571...
Acetoacetyl-CoA
Acetyl-CoA
L-Valine
K00271
3-Methyl-2-oxobutanoate
2-Methyl-1-hydroxypropyl-ThPP
S-(2-Methylpropanoyl)-dihydrolipoamide-E
Isobutyryl-CoA
K00248
K09478...
K11538
Methacrylyl-CoA
(S)-3-Hydroxyisobutyryl-CoA
NCU03777
(S)-3-Hydroxyisobutyrate
NCU06559...
K00022...
Methylmalonate semialdehyde
(S)-3-Aminoisobutanoate
K00822
NCU08998
NCU09266
(R)-3-Aminoisobutanoate
K00827
NCU03415...
K00157
Propanoate metabolism
Citrate cycle
Pyrimidine metabolism
3.1.2.17
Methylmalonate
K18660...
Succinyl-CoA
K01847...
(R)-Methylmalonyl-CoA
K05606
(S)-Methylmalonyl-CoA
L-Isoleucine
K03334
(S)-3-Methyl-2-oxopentanoate
2-Methyl-1-hydroxybutyl-ThPP
S-(2-Methylbutanoyl)-dihydrolipoamide-E
(S)-2-Methylbutanoyl-CoA
trans-2-Methylbut-2-enoyl-CoA
(S)-3-Hydroxy-2-methylbutyryl-CoA
K08683
2-Methylacetoacetyl-CoA
NCU04796...
Propanoyl-CoA
K01965...
Biosynthesis of 12-, 14- and 16-membered macrolides
Biosynthesis of type II polyketide backbone
00280 1/27/16
(c) Kanehisa Laboratories

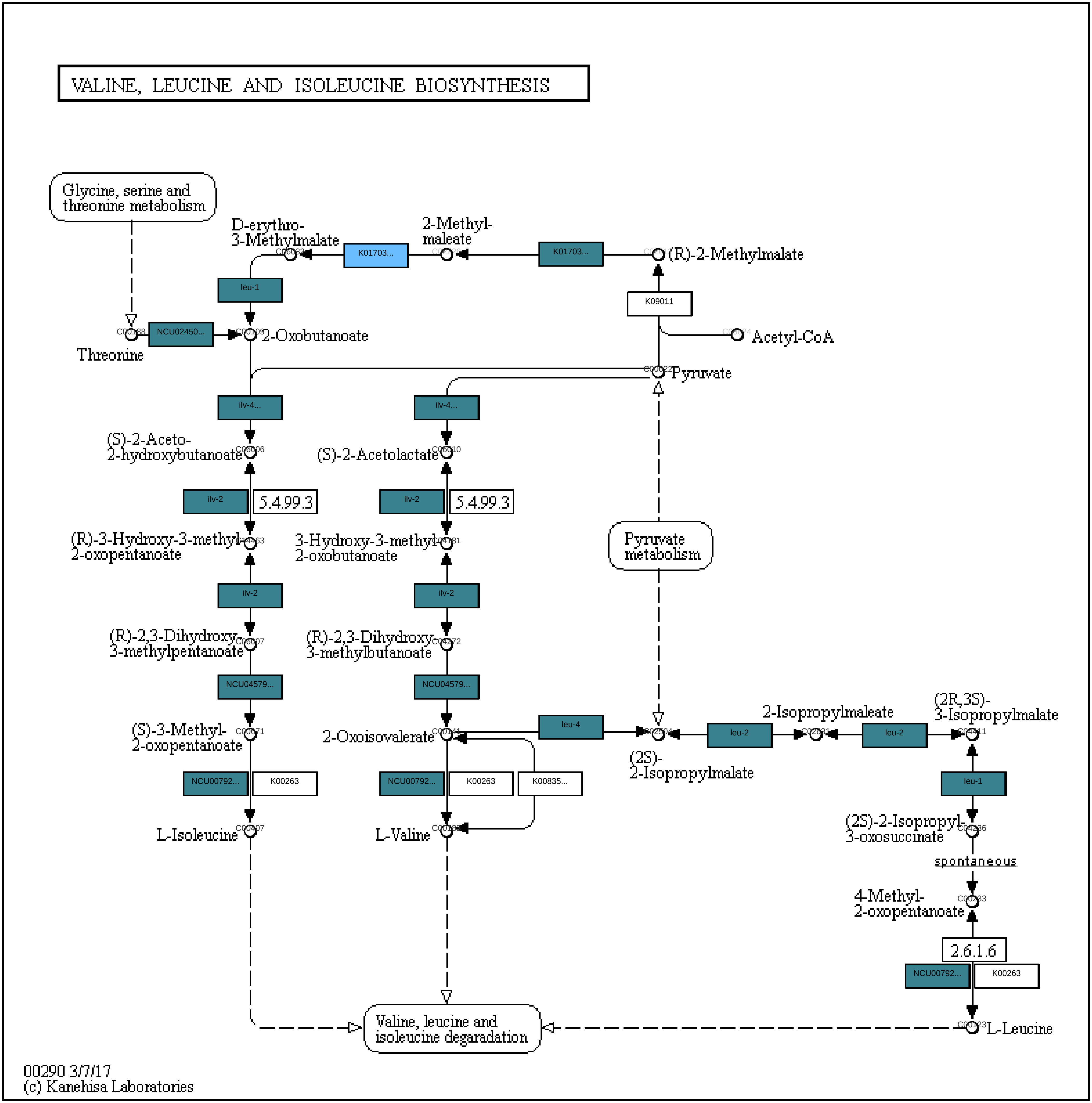
VALINE, LEUCINE AND ISOLEUCINE BIOSYNTHESIS
Glycine, serine and threonine metabolism
D-erythro-3-Methylmalate
K01703...
2-Methyl-maleate
K01703...
(R)-2-Methylmalate
leu-1
K09011
C00188
NCU02450...
2-Oxobutanoate
Threonine
Acetyl-CoA
C00022
Pyruvate
ilv-4...
ilv-4...
(S)-2-Aceto-2-hydroxybutanoate
C06006
(S)-2-Acetolactate
C06010
ilv-2
5.4.99.3
ilv-2
5.4.99.3
(R)-3-Hydroxy-3-methyl-2-oxopentanoate
C14463
3-Hydroxy-3-methyl-2-oxobutanoate
C04181
Pyruvate metabolism
ilv-2
ilv-2
(R)-2,3-Dihydroxy-3-methylpentanoate
C06007
(R)-2,3-Dihydroxy-3-methylbutanoate
C04272
NCU04579...
NCU04579...
(S)-3-Methyl-2-oxopentanoate
C00671
2-Oxoisovalerate
leu-4
(2S)-2-Isopropylmalate
leu-2
2-Isopropylmaleate
leu-2
(2R,3S)-3-Isopropylmalate
C04411
NCU00792...
K00263
NCU00792...
K00263
K00835...
leu-1
L-Isoleucine
C00407
L-Valine
(2S)-2-Isopropyl-3-oxosuccinate
C04236
spontaneous
4-Methyl-2-oxopentanoate
C00233
2.6.1.6
NCU00792...
K00263
Valine, leucine and isoleucine degaradation
C00123
L-Leucine
00290 3/7/17
(c) Kanehisa Laboratories

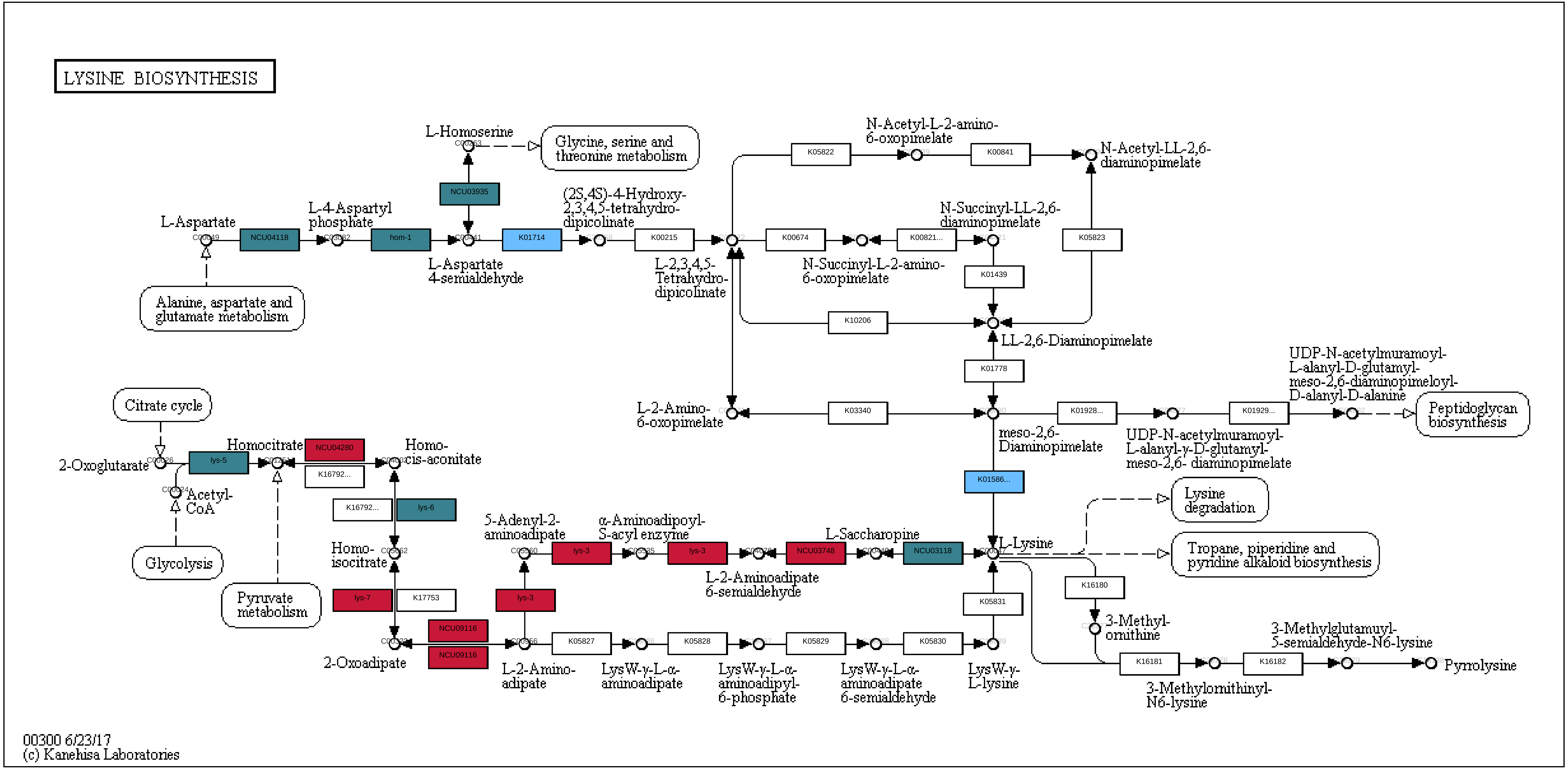

LYSINE BIOSYNTHESIS
L-Homoserine
Glycine, serine and threonine metabolism
NCU03935
L-Aspartate
L-4-Aspartyl phosphate
NCU04118
hom-1
K01714
(2S,4S)-4-Hydroxy-2,3,4,5-tetrahydro-dipicolinate
K00215
L-Aspartate 4-semialdehyde
L-2,3,4,5-Tetrahydro-dipicolinate
Alanine, aspartate and glutamate metabolism
K05822
N-Acetyl-L-2-amino-6-oxopimelate
K00841
N-Acetyl-LL-2,6-diaminopimelate
K00674
K00821...
N-Succinyl-LL-2,6-diaminopimelate
K05823
N-Succinyl-L-2-amino-6-oxopimelate
K01439
K10206
LL-2,6-Diaminopimelate
K01778
L-2-Amino-6-oxopimelate
K03340
K01928...
K01929...
UDP-N-acetylmuramoyl-L-alanyl-D-glutamyl-meso-2,6-diaminopimeloyl-D-alanyl-D-alanine
Peptidoglycan biosynthesis
meso-2,6-Diaminopimelate
UDP-N-acetylmuramoyl-L-alanyl-γ-D-glutamyl-meso-2,6- diaminopimelate
K01586...
Citrate cycle
2-Oxoglutarate
lys-5
Homocitrate
NCU04280
K16792...
Homo-cis-aconitate
Acetyl-CoA
K16792...
lys-6
Glycolysis
Pyruvate metabolism
Homo-isocitrate
lys-7
K17753
5-Adenyl-2-aminoadipate
α-Aminoadipoyl-S-acyl enzyme
lys-3
lys-3
L-Saccharopine
NCU03748
NCU03118
L-Lysine
Lysine degradation
Tropane, piperidine and pyridine alkaloid biosynthesis
L-2-Aminoadipate 6-semialdehyde
lys-3
NCU09116
NCU09116
2-Oxoadipate
K05827
K05828
K05829
K05830
K05831
K16180
3-Methyl-ornithine
K16181
K16182
3-Methylglutamuyl-5-semialdehyde-N6-lysine
Pyrrolysine
L-2-Amino-adipate
LysW-γ-L-α-aminoadipate
LysW-γ-L-α-aminoadipyl-6-phosphate
LysW-γ-L-α-aminoadipate 6-semialdehyde
LysW-γ-L-lysine
3-Methylornithinyl-N6-lysine
00300 6/23/17
(c) Kanehisa Laboratories

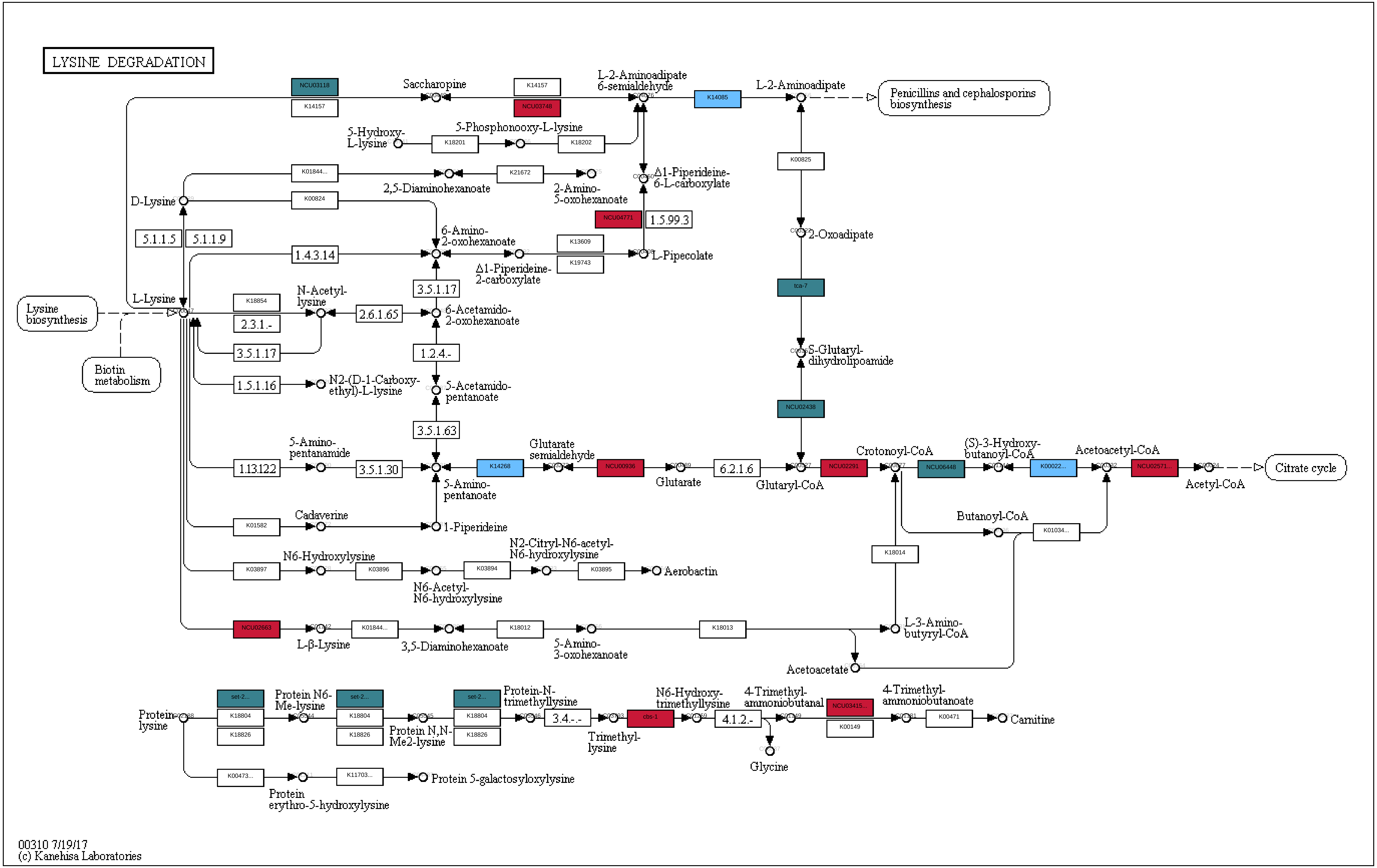

LYSINE DEGRADATION
NCU03118
K14157
Saccharopine
K14157
NCU03748
L-2-Aminoadipate 6-semialdehyde
K14085
L-2-Aminoadipate
Penicillins and cephalosporins biosynthesis
5-Hydroxy-L-lysine
K18201
5-Phosphonooxy-L-lysine
K18202
K00825
K01844...
K21672
2,5-Diaminohexanoate
2-Amino-5-oxohexanoate
Δ1-Piperideine-6-L-carboxylate
D-Lysine
K00824
NCU04771
1.5.99.3
5.1.1.5
5.1.1.9
6-Amino-2-oxohexanoate
2-Oxoadipate
1.4.3.14
K13609
K19743
L-Pipecolate
Δ1-Piperideine-2-carboxylate
tca-7
3.5.1.17
L-Lysine
K18854
N-Acetyl-lysine
2.6.1.65
6-Acetamido-2-oxohexanoate
Lysine biosynthesis
2.3.1.-
3.5.1.17
1.2.4.-
S-Glutaryl-dihydrolipoamide
Biotin metabolism
1.5.1.16
N2-(D-1-Carboxy-ethyl)-L-lysine
5-Acetamido-pentanoate
NCU02438
3.5.1.63
5-Amino-pentanamide
Glutarate semialdehyde
Crotonoyl-CoA
(S)-3-Hydroxy-butanoyl-CoA
Acetoacetyl-CoA
1.13.12.2
3.5.1.30
K14268
NCU00936
6.2.1.6
NCU02291
NCU06448
K00022...
NCU02571...
Citrate cycle
5-Amino-pentanoate
Glutarate
Glutaryl-CoA
Acetyl-CoA
Cadaverine
K01582
1-Piperideine
Butanoyl-CoA
K01034...
K18014
N6-Hydroxylysine
N2-Citryl-N6-acetyl-N6-hydroxylysine
K03897
K03896
K03894
K03895
Aerobactin
N6-Acetyl-N6-hydroxylysine
L-3-Amino-butyryl-CoA
NCU02663
K01844...
K18012
K18013
L-β-Lysine
3,5-Diaminohexanoate
5-Amino-3-oxohexanoate
Acetoacetate
set-2...
Protein N6-Me-lysine
set-2...
set-2...
Protein-N-trimethyllysine
N6-Hydroxy-trimethyllysine
4-Trimethyl-ammoniobutanal
4-Trimethyl-ammoniobutanoate
Protein-lysine
K18804
K18826
K18804
K18826
K18804
K18826
3.4.-.-
cbs-1
4.1.2.-
NCU03415...
K00149
K00471
Carnitine
Protein N,N-Me2-lysine
Trimethyl-lysine
Glycine
K00473...
K11703...
Protein 5-galactosyloxylysine
Protein erythro-5-hydroxylysine
00310 7/19/17
(c) Kanehisa Laboratories

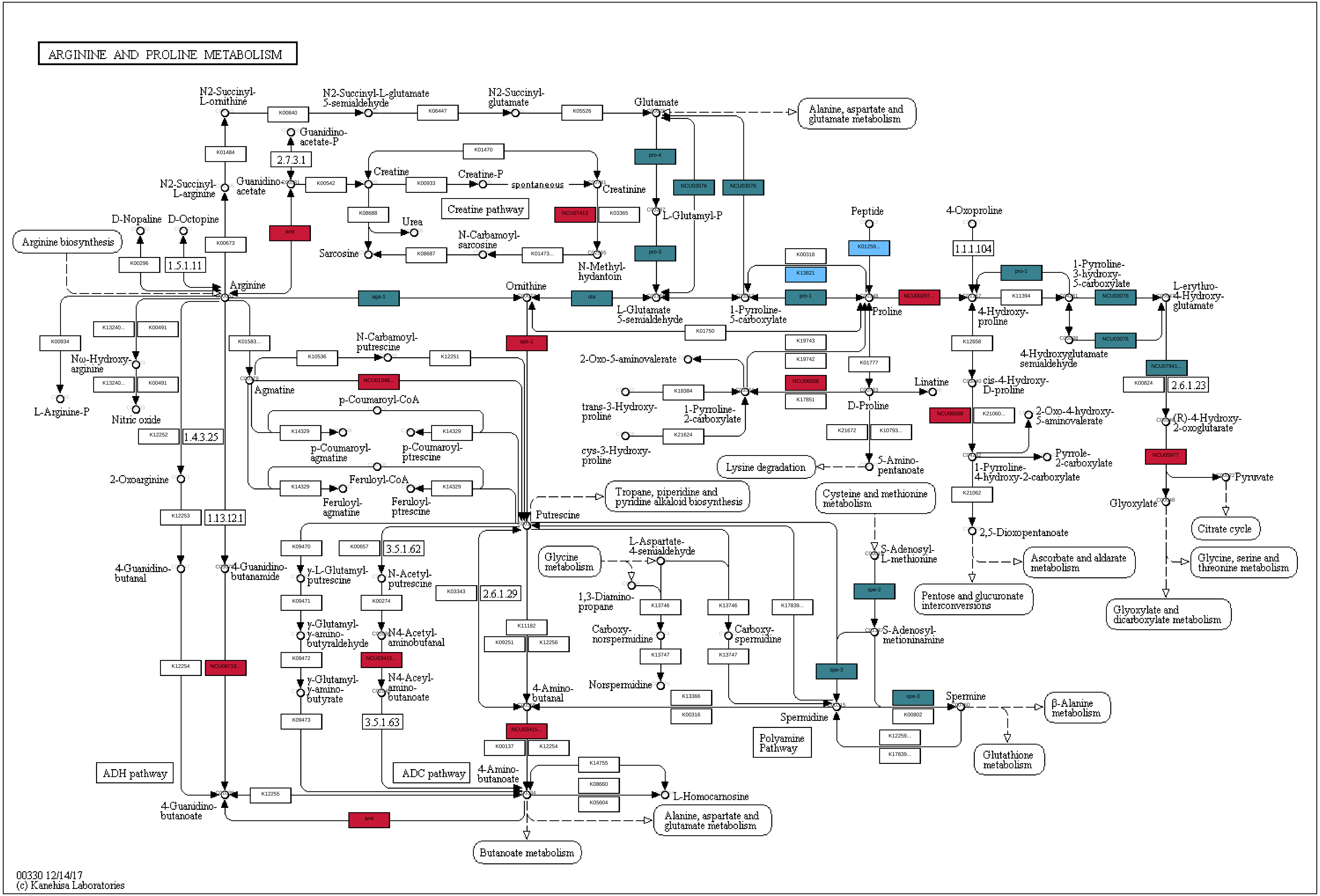
ARGININE AND PROLINE METABOLISM
N2-Succinyl-L-ornithine
N2-Succinyl-L-glutamate 5-semialdehyde
N2-Succinyl-glutamate
Glutamate
Alanine, aspartate and glutamate metabolism
K00840
K06447
K05526
Guanidino-acetate-P
2.7.3.1
K01470
K01484
N2-Succinyl-L-arginine
Guanidino-acetate
K00542
Creatine
K00933
Creatine-P
spontaneous
Creatinine
pro-4
NCU03076
NCU03076
Creatine pathway
NCU07413
K03365
L-Glutamyl-P
K08688
Urea
D-Nopaline
D-Octopine
Arginine biosynthesis
K00673
amt
Sarcosine
K08687
N-Carbamoyl-sarcosine
K01473...
N-Methyl-hydantoin
pro-3
Peptide
4-Oxoproline
K01259...
1.1.1.104
K00318
K13821
K00296
1.5.1.11
pro-1
1-Pyrroline-3-hydroxy-5-carboxylate
Arginine
aga-1
Ornithine
ota
L-Glutamate 5-semialdehyde
1-Pyrroline-5-carboxylate
pro-1
NCU00207...
Proline
K11394
NCU03076
L-erythro-4-Hydroxy-glutamate
4-Hydroxy-proline
K01750
K00934
K13240...
K00491
K01583...
spe-1
K19743
K12658
NCU03076
4-Hydroxyglutamate semialdehyde
Nω-Hydroxy-arginine
K10536
N-Carbamoyl-putrescine
K12251
2-Oxo-5-aminovalerate
K19742
K01777
NCU07941...
K13240...
K00491
Agmatine
NCU01348...
NCU06558
Linatine
cis-4-Hydroxy-D-proline
K00824
2.6.1.23
L-Arginine-P
Nitric oxide
p-Coumaroyl-CoA
K18384
K17851
D-Proline
trans-3-Hydroxy-proline
1-Pyrroline-2-carboxylate
NCU06558
K21060...
2-Oxo-4-hydroxy-5-aminovalerate
(R)-4-Hydroxy-2-oxoglutarate
K12252
1.4.3.25
K14329
K14329
K21624
K21672
K10793...
p-Coumaroyl-agmatine
p-Coumaroyl-ptrescine
cys-3-Hydroxy-proline
5-Amino-pentanoate
Lysine degradation
Pyrrole-2-carboxylate
1-Pyrroline-4-hydroxy-2-carboxylate
NCU05977
2-Oxoarginine
Feruloyl-CoA
K14329
K14329
Pyruvate
Feruloyl-agmatine
Feruloyl-ptrescine
Tropane, piperidine and pyridine alkaloid biosynthesis
Cysteine and methionine metabolism
K21062
Glyoxylate
Putrescine
Citrate cycle
K12253
1.13.12.1
K09470
K00657
3.5.1.62
2,5-Dioxopentanoate
L-Aspartate-4-semialdehyde
Glycine metabolism
S-Adenosyl-L-methionine
Ascorbate and aldarate metabolism
Glycine, serine and threonine metabolism
4-Guanidino-butanal
4-Guanidino-butanamide
γ-L-Glutamyl-putrescine
N-Acetyl-putrescine
K03343
2.6.1.29
1,3-Diamino-propane
spe-2
Pentose and glucuronate interconversions
K09471
K00274
K13746
K13746
K17839...
Glyoxylate and dicarboxylate metabolism
K11182
γ-Glutamyl-γ-amino-butyraldehyde
N4-Acetyl-aminobutanal
Carboxy-norspermidine
Carboxy-spermidine
S-Adenosyl-metioninamine
K09251
K12256
K09472
NCU03415...
K13747
K13747
K12254
NCU08719...
spe-3
γ-Glutamyl-γ-amino-butyrate
N4-Aceyl-amino-butanoate
4-Amino-butanal
Norspermidine
K13366
spe-3
Spermine
β-Alanine metabolism
K00316
K00802
K09473
3.5.1.63
NCU03415...
Spermidine
Polyamine Pathway
K12259...
K00137
K12254
K17839...
Glutathione metabolism
ADH pathway
ADC pathway
4-Amino-butanoate
K14755
K08660
L-Homocarnosine
K12255
K05604
4-Guanidino-butanoate
amt
Alanine, aspartate and glutamate metabolism
Butanoate metabolism
00330 12/14/17
(c) Kanehisa Laboratories

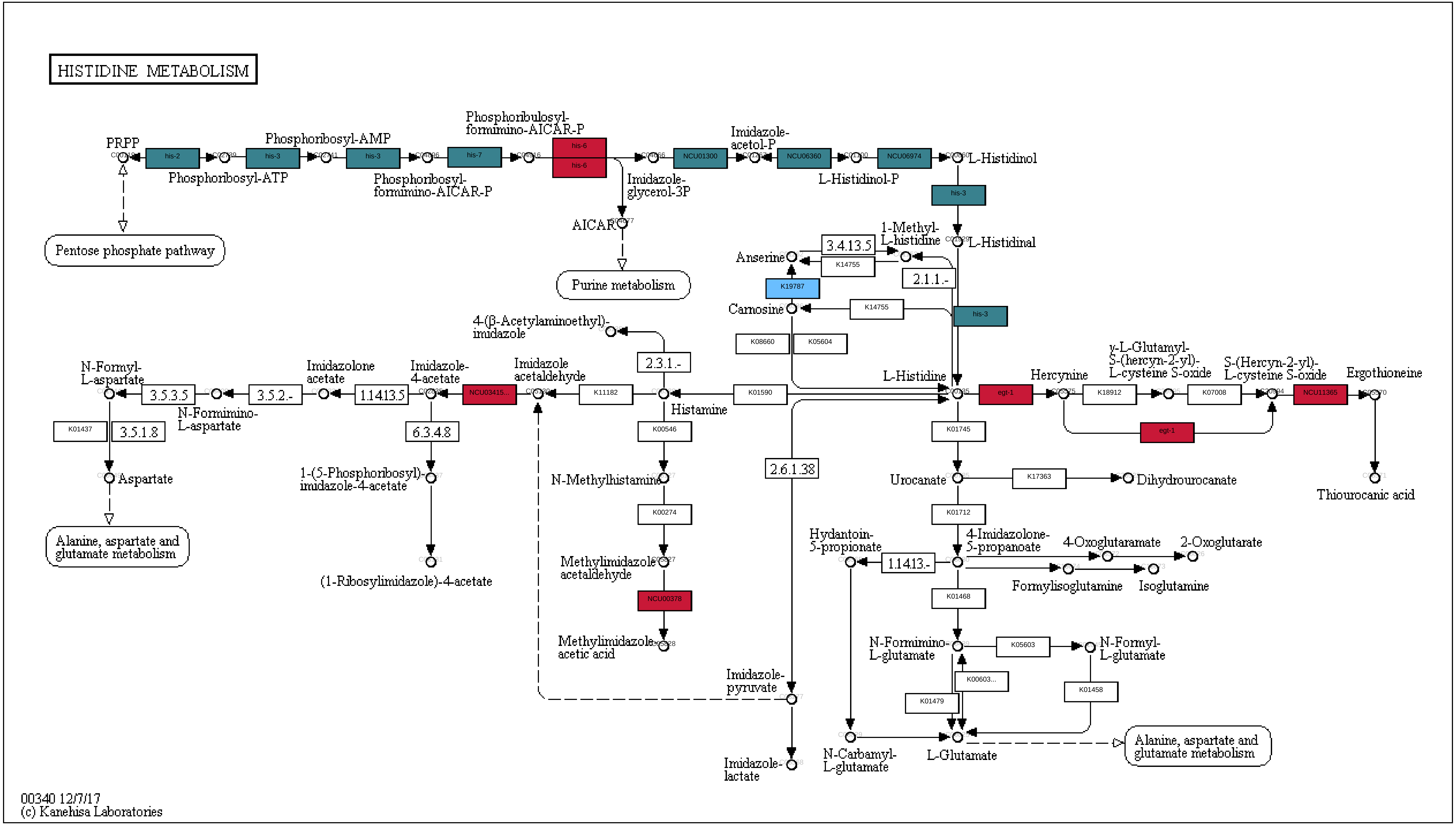
HISTIDINE METABOLISM
PRPP
his-2
Phosphoribosyl-ATP
his-3
Phosphoribosyl-AMP
his-3
Phosphoribosyl-formimino-AICAR-P
his-7
Phosphoribulosyl-formimino-AICAR-P
his-6
his-6
Imidazole-glycerol-3P
AICAR
Purine metabolism
NCU01300
Imidazole-acetol-P
NCU06360
L-Histidinol-P
NCU06974
L-Histidinol
his-3
L-Histidinal
his-3
Pentose phosphate pathway
1-Methyl-L-histidine
3.4.13.5
Anserine
K14755
2.1.1.-
K19787
Carnosine
K14755
K08660
K05604
L-Histidine
egt-1
Hercynine
K18912
γ-L-Glutamyl-S-(hercyn-2-yl)-L-cysteine S-oxide
K07008
S-(Hercyn-2-yl)-L-cysteine S-oxide
NCU11365
Ergothioneine
egt-1
Thiourocanic acid
4-(β-Acetylaminoethyl)-imidazole
2.3.1.-
K01590
Histamine
K11182
Imidazole acetaldehyde
NCU03415...
Imidazole-4-acetate
1.14.13.5
Imidazolone acetate
3.5.2.-
N-Formimino-L-aspartate
3.5.3.5
N-Formyl-L-aspartate
K01437
3.5.1.8
Aspartate
Alanine, aspartate and glutamate metabolism
6.3.4.8
1-(5-Phosphoribosyl)-imidazole-4-acetate
(1-Ribosylimidazole)-4-acetate
K00546
N-Methylhistamine
K00274
Methylimidazole acetaldehyde
NCU00378
Methylimidazole-acetic acid
2.6.1.38
Imidazole-pyruvate
Imidazole-lactate
K01745
Urocanate
K17363
Dihydrourocanate
K01712
4-Imidazolone-5-propanoate
4-Oxoglutaramate
2-Oxoglutarate
Formylisoglutamine
Isoglutamine
1.14.13.-
Hydantoin-5-propionate
K01468
N-Formimino-L-glutamate
K05603
N-Formyl-L-glutamate
K00603...
K01479
K01458
N-Carbamyl-L-glutamate
L-Glutamate
Alanine, aspartate and glutamate metabolism
00340 12/7/17
(c) Kanehisa Laboratories

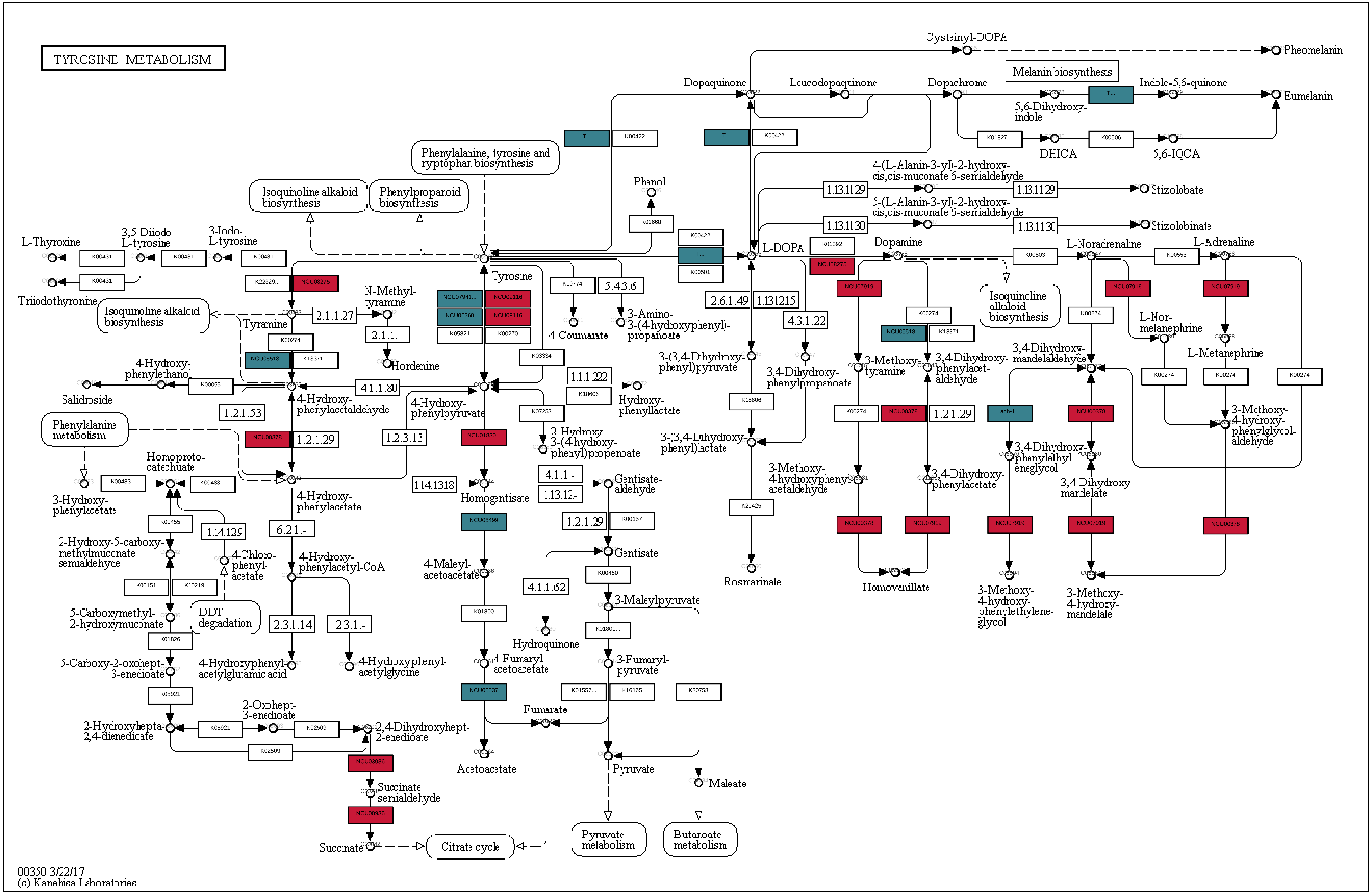
TYROSINE METABOLISM
Cysteinyl-DOPA
Pheomelanin
Melanin biosynthesis
Dopaquinone
Leucodopaquinone
Dopachrome
Indole-5,6-quinone
Eumelanin
5,6-Dihydroxy-indole
DHICA
5,6-IQCA
Phenylalanine, tyrosine and ryptophan biosynthesis
Isoquinoline alkaloid biosynthesis
Phenylpropanoid biosynthesis
Phenol
4-(L-Alanin-3-yl)-2-hydroxy-cis,cis-muconate 6-semialdehyde
Stizolobate
5-(L-Alanin-3-yl)-2-hydroxy-cis,cis-muconate 6-semialdehyde
Stizolobinate
1.13.11.29
1.13.11.30
L-Thyroxine
3,5-Diiodo-L-tyrosine
3-Iodo-L-tyrosine
Triiodothyronine
L-DOPA
Dopamine
L-Noradrenaline
L-Adrenaline
Tyrosine
N-Methyl-tyramine
Tyramine
2.1.1.27
2.1.1.-
Hordenine
4-Coumarate
5.4.3.6
3-Amino-3-(4-hydroxyphenyl)-propanoate
2.6.1.49
1.13.12.15
4.3.1.22
L-Nor-metanephrine
L-Metanephrine
4-Hydroxy-phenylethanol
Salidroside
4.1.1.80
4-Hydroxy-phenylacetaldehyde
4-Hydroxy-phenylpyruvate
1.1.1.222
Hydroxy-phenyllactate
3-(3,4-Dihydroxy-phenyl)pyruvate
3,4-Dihydroxy-phenylpropanoate
3-Methoxy-tyramine
3,4-Dihydroxy-phenylacet-aldehyde
3,4-Dihydroxy-mandelaldehyde
1.2.1.53
Phenylalanine metabolism
1.2.1.29
1.2.3.13
2-Hydroxy-3-(4-hydroxy-phenyl)propenoate
3-(3,4-Dihydroxy-phenyl)lactate
3-Methoxy-4-hydroxy-phenylglycol-aldehyde
3,4-Dihydroxy-phenylethyl-eneglycol
Homoproto-catechuate
1.14.13.18
4.1.1.-
Gentisate-aldehyde
3-Methoxy-4-hydroxyphenyl-acetaldehyde
3,4-Dihydroxy-phenylacetate
3,4-Dihydroxy-mandelate
3-Hydroxy-phenylacetate
4-Hydroxy-phenylacetate
Homogentisate
1.13.12.-
2-Hydroxy-5-carboxy-methylmuconate semialdehyde
1.14.12.9
6.2.1.-
1.2.1.29
Gentisate
4-Chloro-phenyl-acetate
4-Hydroxy-phenylacetyl-CoA
4-Maleyl-acetoacetate
Rosmarinate
Homovanillate
3-Methoxy-4-hydroxy-phenylethylene-glycol
3-Methoxy-4-hydroxy-mandelate
4.1.1.62
5-Carboxymethyl-2-hydroxymuconate
DDT degradation
2.3.1.14
2.3.1.-
3-Maleylpyruvate
Hydroquinone
5-Carboxy-2-oxohept-3-enedioate
4-Hydroxyphenyl-acetylglutamic acid
4-Hydroxyphenyl-acetylglycine
4-Fumaryl-acetoacetate
3-Fumaryl-pyruvate
2-Oxohept-3-enedioate
Fumarate
2-Hydroxyhepta-2,4-dienedioate
2,4-Dihydroxyhept-2-enedioate
Acetoacetate
Pyruvate
Maleate
Succinate semialdehyde
Pyruvate metabolism
Butanoate metabolism
Succinate
Citrate cycle
00350 3/22/17
(c) Kanehisa Laboratories

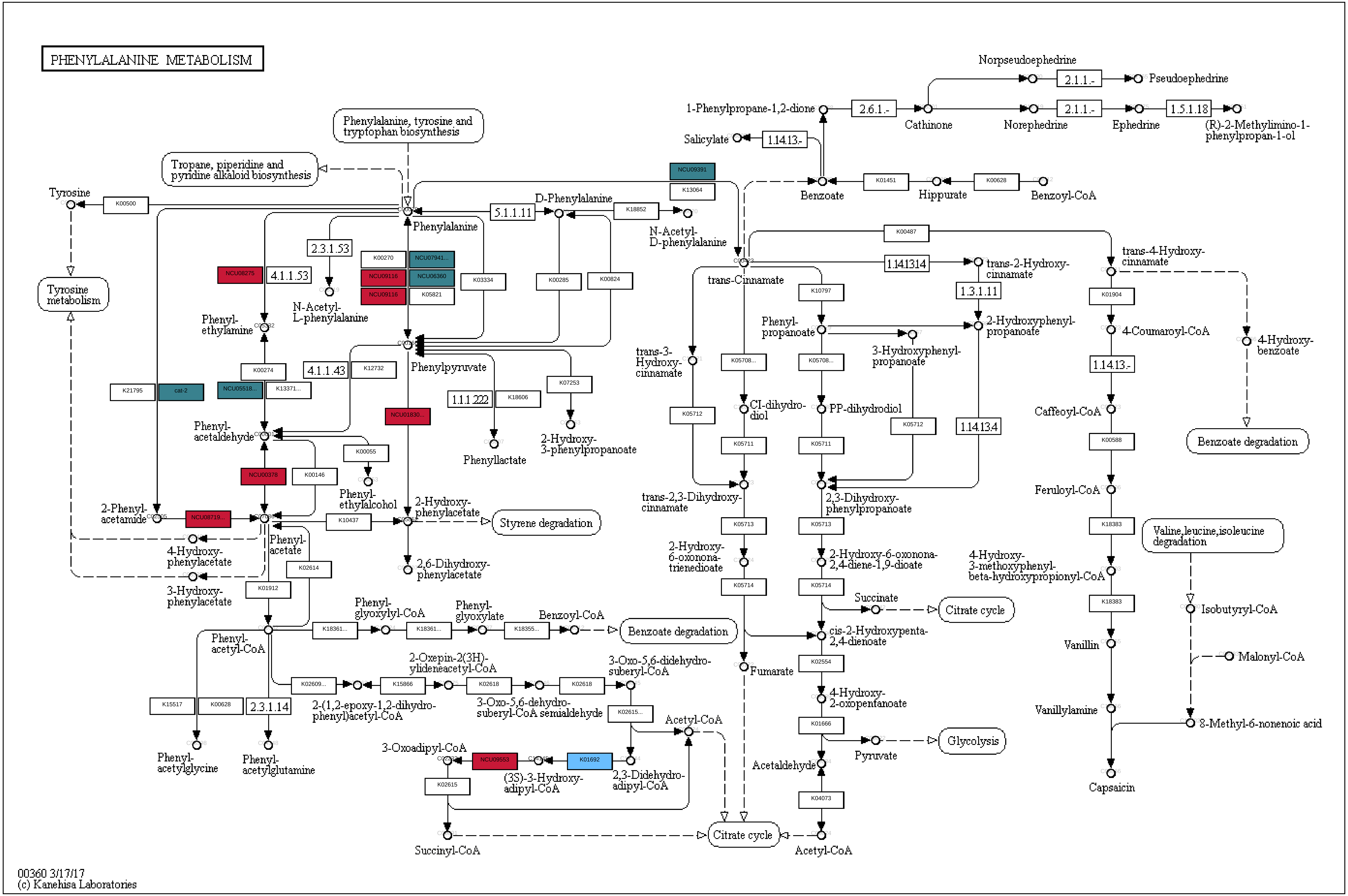
PHENYLALANINE METABOLISM
Phenylalanine, tyrosine and tryptophan biosynthesis
Tropane, piperidine and pyridine alkaloid biosynthesis
Tyrosine
K00500
Tyrosine metabolism
Phenylalanine
5.1.1.11
D-Phenylalanine
K18852
N-Acetyl-D-phenylalanine
2.3.1.53
N-Acetyl-L-phenylalanine
NCU08275
4.1.1.53
K00270
NCU07941...
NCU09116
NCU06360
NCU09116
K05821
K03334
K00285
K00824
NCU09391
K13064
Phenyl-ethylamine
Phenylpyruvate
4.1.1.43
K12732
K00274
K21795
cat-2
NCU05518...
K13371...
NCU01830...
1.1.1.222
K18606
K07253
Phenyllactate
2-Hydroxy-3-phenylpropanoate
Phenyl-acetaldehyde
K00055
Phenyl-ethylalcohol
NCU00378
K00146
2-Phenyl-acetamide
NCU08719...
K10437
2-Hydroxy-phenylacetate
Styrene degradation
Phenyl-acetate
4-Hydroxy-phenylacetate
3-Hydroxy-phenylacetate
2,6-Dihydroxy-phenylacetate
K02614
K01912
Phenyl-acetyl-CoA
K18361...
Phenyl-glyoxylyl-CoA
K18361...
Phenyl-glyoxylate
K18355...
Benzoyl-CoA
Benzoate degradation
K15517
K00628
2.3.1.14
Phenyl-acetylglycine
Phenyl-acetylglutamine
K02609...
2-(1,2-epoxy-1,2-dihydro-phenyl)acetyl-CoA
K15866
2-Oxepin-2(3H)-ylideneacetyl-CoA
K02618
3-Oxo-5,6-dehydro-suberyl-CoA semialdehyde
K02618
3-Oxo-5,6-didehydro-suberyl-CoA
K02615...
Acetyl-CoA
3-Oxoadipyl-CoA
NCU09553
K01692
(3S)-3-Hydroxy-adipyl-CoA
2,3-Didehydro-adipyl-CoA
K02615
Succinyl-CoA
Citrate cycle
1-Phenylpropane-1,2-dione
2.6.1.-
Cathinone
Norpseudoephedrine
2.1.1.-
Pseudoephedrine
2.1.1.-
Norephedrine
Ephedrine
1.5.1.18
(R)-2-Methylimino-1-phenylpropan-1-ol
Salicylate
1.14.13.-
Benzoate
K01451
Hippurate
K00628
Benzoyl-CoA
K00487
trans-Cinnamate
1.14.13.14
trans-2-Hydroxy-cinnamate
1.3.1.11
trans-4-Hydroxy-cinnamate
K10797
Phenyl-propanoate
2-Hydroxyphenyl-propanoate
3-Hydroxyphenyl-propanoate
trans-3-Hydroxy-cinnamate
K05708...
K05708...
Cl-dihydro-diol
PP-dihydrodiol
K05712
K05712
1.14.13.4
K05711
K05711
trans-2,3-Dihydroxy-cinnamate
2,3-Dihydroxy-phenylpropanoate
K05713
K05713
2-Hydroxy-6-oxonona-trienedioate
2-Hydroxy-6-oxonona-2,4-diene-1,9-dioate
K05714
K05714
Succinate
Citrate cycle
cis-2-Hydroxypenta-2,4-dienoate
Fumarate
K02554
4-Hydroxy-2-oxopentanoate
K01666
Pyruvate
Glycolysis
Acetaldehyde
K04073
Acetyl-CoA
Citrate cycle
K01904
4-Coumaroyl-CoA
4-Hydroxy-benzoate
1.14.13.-
Caffeoyl-CoA
Benzoate degradation
K00588
Feruloyl-CoA
K18383
Valine, leucine, isoleucine degradation
4-Hydroxy-3-methoxyphenyl-beta-hydroxypropionyl-CoA
K18383
Isobutyryl-CoA
Vanillin
Malonyl-CoA
Vanillylamine
8-Methyl-6-nonenoic acid
Capsaicin
00360 3/17/17
(c) Kanehisa Laboratories

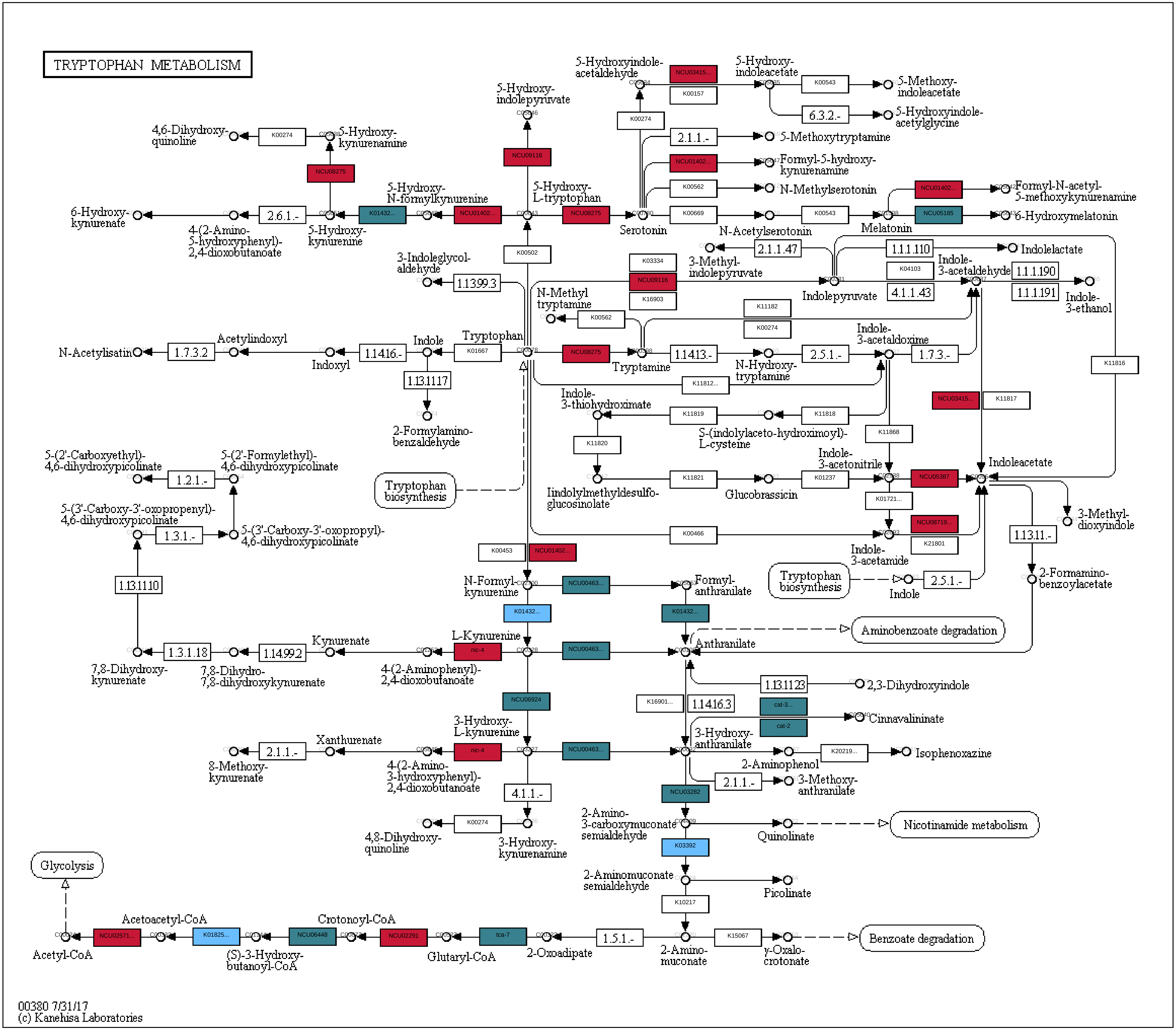
TRYPTOPHAN METABOLISM
5-Hydroxyindole-acetaldehyde
NCU03415...
K00157
5-Hydroxy-indoleacetate
K00543
5-Methoxy-indoleacetate
6.3.2.-
5-Hydroxyindole-acetylglycine
5-Hydroxy-indolepyruvate
K00274
2.1.1.-
5-Methoxytryptamine
4,6-Dihydroxy-quinoline
K00274
5-Hydroxy-kynurenamine
NCU09116
NCU01402...
Formyl-5-hydroxy-kynurenamine
NCU08275
K00562
N-Methylserotonin
5-Hydroxy-N-formylkynurenine
5-Hydroxy-L-tryptophan
NCU01402...
Formyl-N-acetyl-5-methoxykynurenamine
6-Hydroxy-kynurenate
2.6.1.-
K01432...
NCU01402...
NCU08275
K00669
K00543
NCU05185
6-Hydroxymelatonin
4-(2-Amino-5-hydroxyphenyl)-2,4-dioxobutanoate
5-Hydroxy-kynurenine
Serotonin
N-Acetylserotonin
Melatonin
K00502
2.1.1.47
1.1.1.110
Indolelactate
3-Indoleglycol-aldehyde
K03334
3-Methyl-indolepyruvate
K04103
Indole-3-acetaldehyde
1.1.1.190
1.13.99.3
NCU09116
Indolepyruvate
4.1.1.43
1.1.1.191
Indole-3-ethanol
N-Methyl tryptamine
K16903
K11182
K00562
K00274
Indole-3-acetaldoxime
N-Acetylisatin
1.7.3.2
Acetylindoxyl
Indoxyl
1.14.16.-
Indole
K01667
Tryptophan
NCU08275
Tryptamine
1.14.13.-
N-Hydroxy-tryptamine
2.5.1.-
1.7.3.-
K11816
1.13.11.17
K11812...
Indole-3-thiohydroximate
K11819
K11818
S-(indolylaceto-hydroximoyl)-L-cysteine
NCU03415...
K11817
2-Formylamino-benzaldehyde
K11820
K11868
5-(2'-Carboxyethyl)-4,6-dihydroxypicolinate
5-(2'-Formylethyl)-4,6-dihydroxypicolinate
1.2.1.-
Tryptophan biosynthesis
Iindolylmethyldesulfo-glucosinolate
K11821
Glucobrassicin
K01237
Indole-3-acetonitrile
NCU05387
Indoleacetate
K01721...
3-Methyl-dioxyindole
5-(3'-Carboxy-3'-oxopropenyl)-4,6-dihydroxypicolinate
1.3.1.-
5-(3'-Carboxy-3'-oxopropyl)-4,6-dihydroxypicolinate
K00466
NCU08719...
K21801
Indole-3-acetamide
1.13.11.-
K00453
NCU01402...
Tryptophan biosynthesis
Indole
2.5.1.-
2-Formamino-benzoylacetate
1.13.11.10
N-Formyl-kynurenine
NCU00463...
Formyl-anthranilate
K01432...
K01432...
Aminobenzoate degradation
L-Kynurenine
Anthranilate
7,8-Dihydroxy-kynurenate
1.3.1.18
7,8-Dihydro-7,8-dihydroxykynurenate
1.14.99.2
Kynurenate
4-(2-Aminophenyl)-2,4-dioxobutanoate
nic-4
NCU00463...
1.13.11.23
2,3-Dihydroxyindole
NCU06924
K16901...
1.14.16.3
cat-3...
Cinnavalininate
cat-2
3-Hydroxy-L-kynurenine
3-Hydroxy-anthranilate
8-Methoxy-kynurenate
2.1.1.-
Xanthurenate
nic-4
NCU00463...
K20219...
Isophenoxazine
2-Aminophenol
4-(2-Amino-3-hydroxyphenyl)-2,4-dioxobutanoate
4.1.1.-
2.1.1.-
3-Methoxy-anthranilate
NCU03282
K00274
2-Amino-3-carboxymuconate semialdehyde
Quinolinate
Nicotinamide metabolism
4,8-Dihydroxy-quinoline
3-Hydroxy-kynurenamine
K03392
Glycolysis
2-Aminomuconate semialdehyde
Picolinate
K10217
Acetoacetyl-CoA
Crotonoyl-CoA
NCU02571...
K01825...
NCU06448
NCU02291
tca-7
1.5.1.-
K15067
Benzoate degradation
Acetyl-CoA
(S)-3-Hydroxy-butanoyl-CoA
Glutaryl-CoA
2-Oxoadipate
2-Amino-muconate
γ-Oxalo-crotonate
00380 7/31/17
(c) Kanehisa Laboratories

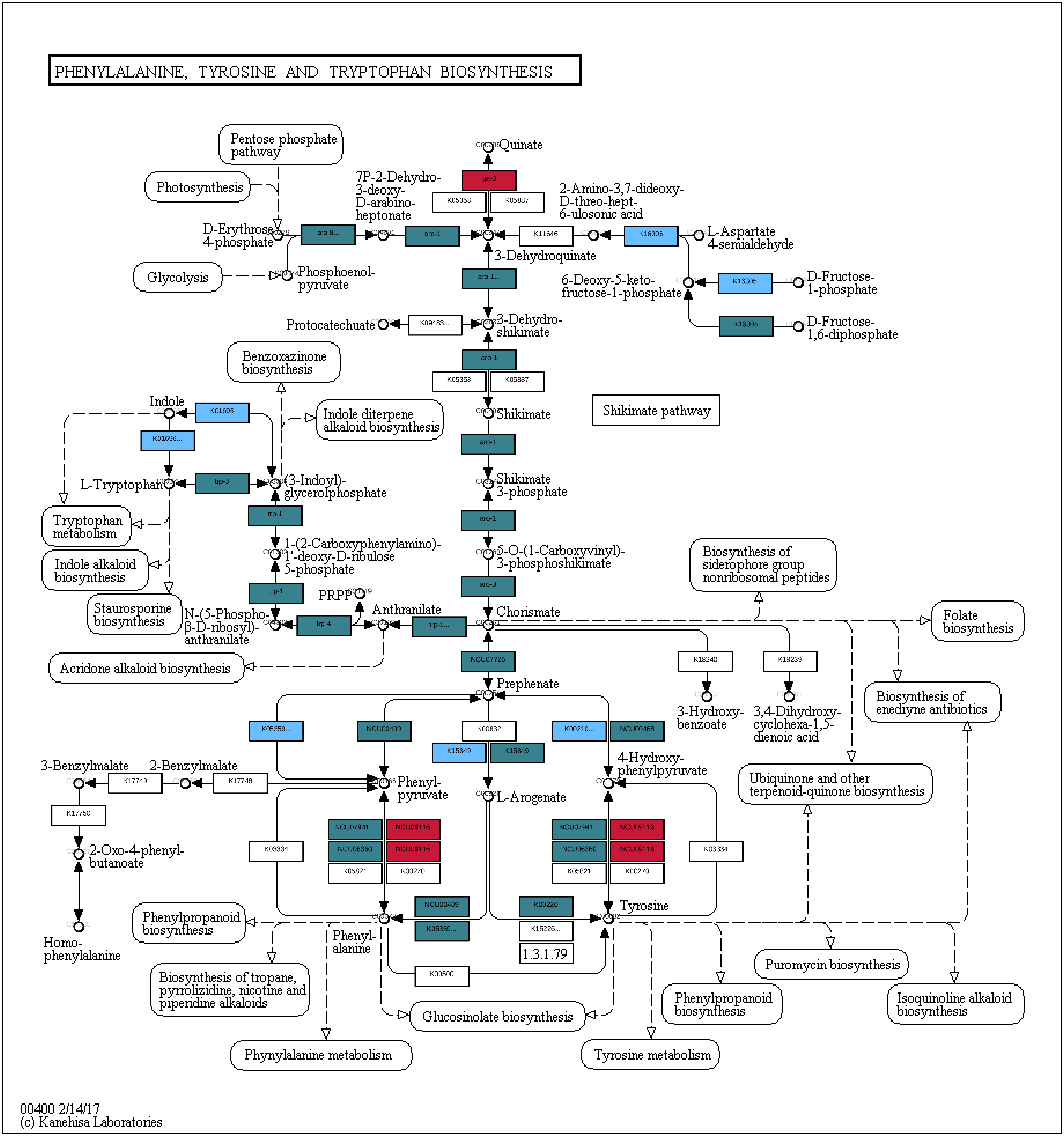
PHENYLALANINE, TYROSINE AND TRYPTOPHAN BIOSYNTHESIS
Pentose phosphate pathway
Photosynthesis
Glycolysis
D-Erythrose 4-phosphate
Phosphoenol-pyruvate
7P-2-Dehydro-3-deoxy-D-arabino-heptonate
Quinate
qa-3
K05358
K05887
aro-8...
aro-1
K11646
K16306
2-Amino-3,7-dideoxy-D-threo-hept-6-ulosonic acid
L-Aspartate 4-semialdehyde
3-Dehydroquinate
aro-1...
6-Deoxy-5-keto-fructose-1-phosphate
K16305
D-Fructose-1-phosphate
D-Fructose-1,6-diphosphate
Protocatechuate
K09483...
3-Dehydro-shikimate
Benzoxazinone biosynthesis
Shikimate
Shikimate pathway
Indole
K01695
K01696...
Indole diterpene alkaloid biosynthesis
L-Tryptophan
trp-3
(3-Indoyl)-glycerolphosphate
Shikimate 3-phosphate
Tryptophan metabolism
trp-1
1-(2-Carboxyphenylamino)-1'-deoxy-D-ribulose 5-phosphate
aro-3
5-O-(1-Carboxyvinyl)-3-phosphoshikimate
Indole alkaloid biosynthesis
Biosynthesis of siderophore group nonribosomal peptides
PRPP
Staurosporine biosynthesis
N-(5-Phospho-β-D-ribosyl)-anthranilate
trp-4
Anthranilate
trp-1...
Chorismate
Folate biosynthesis
Acridone alkaloid biosynthesis
NCU07725
K18240
K18239
Prephenate
3-Hydroxy-benzoate
3,4-Dihydroxy-cyclohexa-1,5-dienoic acid
Biosynthesis of enediyne antibiotics
K05359...
NCU00409
K00832
K00210...
NCU00468
K15849
4-Hydroxy-phenylpyruvate
Ubiquinone and other terpenoid-quinone biosynthesis
3-Benzylmalate
2-Benzylmalate
K17749
K17748
Phenyl-pyruvate
L-Arogenate
K17750
K03334
NCU07941...
NCU09116
NCU06360
K05821
K00270
2-Oxo-4-phenyl-butanoate
Tyrosine
K00220
K15226...
1.3.1.79
Phenylpropanoid biosynthesis
Homo-phenylalanine
Phenyl-alanine
K00500
Puromycin biosynthesis
Biosynthesis of tropane, pyrrolizidine, nicotine and piperidine alkaloids
Glucosinolate biosynthesis
Isoquinoline alkaloid biosynthesis
Phynylalanine metabolism
Tyrosine metabolism
00400 2/14/17
(c) Kanehisa Laboratories

# 6. Metabolism of other amino acids

| MAP | PATHWAY |
|---|---|
| **410** | beta-Alanine metabolism |
| **430** | Taurine and hypotaurine metabolism |
| **440** | Phosphonate and phosphinate metabolism |
| **450** | Selenocompound metabolism |
| **460** | Cyanoamino acid metabolism |
| **480** | Glutathione metabolism |

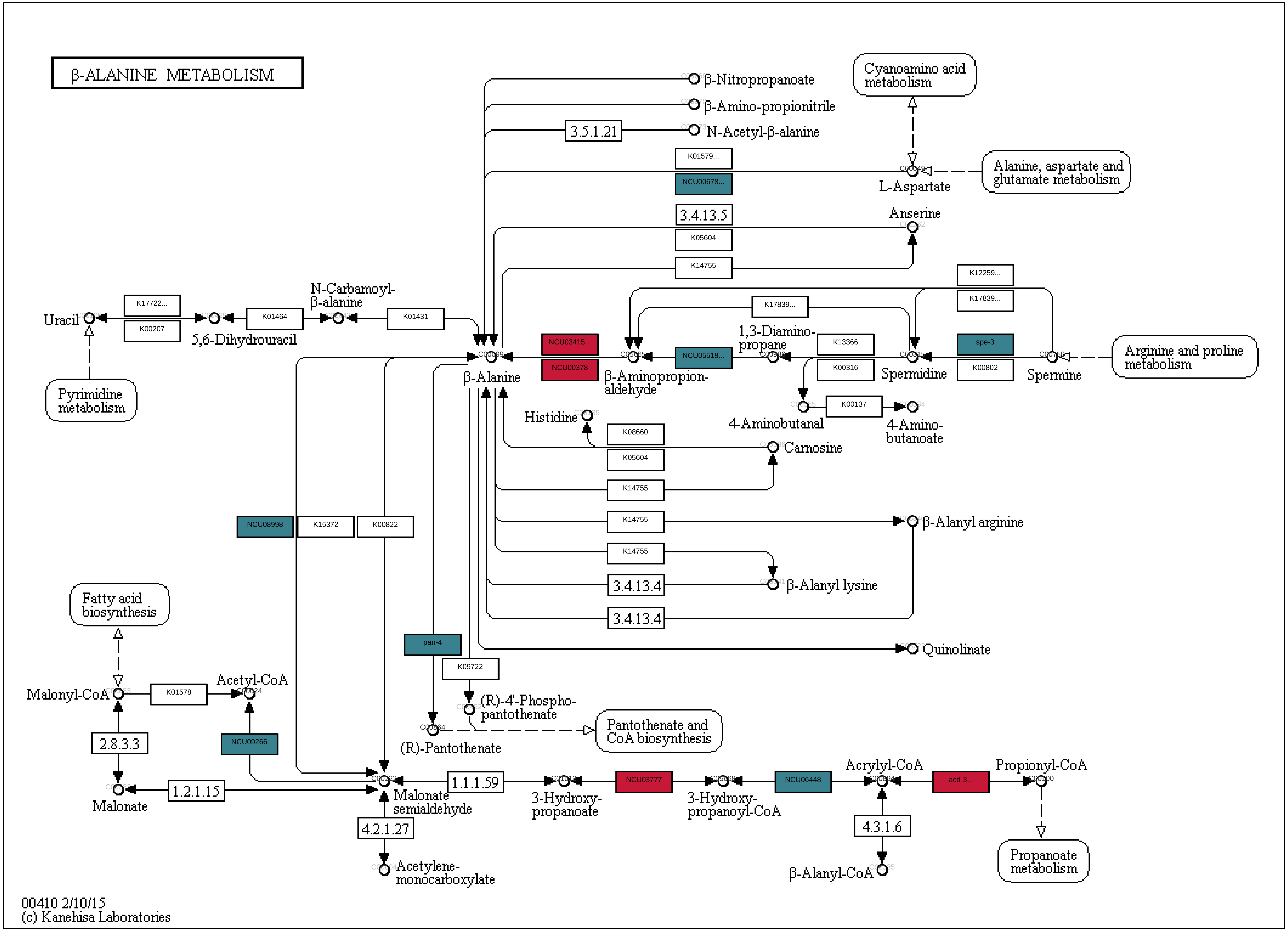

β-ALANINE METABOLISM
β-Nitropropanoate
β-Amino-propionitrile
3.5.1.21
N-Acetyl-β-alanine
Cyanoamino acid metabolism
K01579...
NCU00678...
L-Aspartate
Alanine, aspartate and glutamate metabolism
3.4.13.5
Anserine
K05604
K14755
K12259...
K17839...
K17839...
N-Carbamoyl-β-alanine
K17722...
K00207
K01464
K01431
Uracil
5,6-Dihydrouracil
Pyrimidine metabolism
NCU03415...
NCU00378
NCU05518...
1,3-Diamino-propane
K13366
K00316
spe-3
K00802
Arginine and proline metabolism
β-Alanine
β-Aminopropion-aldehyde
Spermidine
Spermine
Histidine
4-Aminobutanal
K00137
4-Amino-butanoate
K08660
K05604
Carnosine
K14755
K14755
β-Alanyl arginine
NCU08998
K15372
K00822
K14755
3.4.13.4
β-Alanyl lysine
3.4.13.4
pan-4
Quinolinate
K09722
Fatty acid biosynthesis
Malonyl-CoA
K01578
Acetyl-CoA
(R)-4'-Phospho-pantothenate
Pantothenate and CoA biosynthesis
(R)-Pantothenate
2.8.3.3
NCU09266
1.1.1.59
NCU03777
NCU06448
Acrylyl-CoA
acd-3...
Propionyl-CoA
Malonate
1.2.1.15
Malonate semialdehyde
3-Hydroxy-propanoate
3-Hydroxy-propanoyl-CoA
4.2.1.27
4.3.1.6
Propanoate metabolism
Acetylene-monocarboxylate
β-Alanyl-CoA
00410 2/10/15
(c) Kanehisa Laboratories

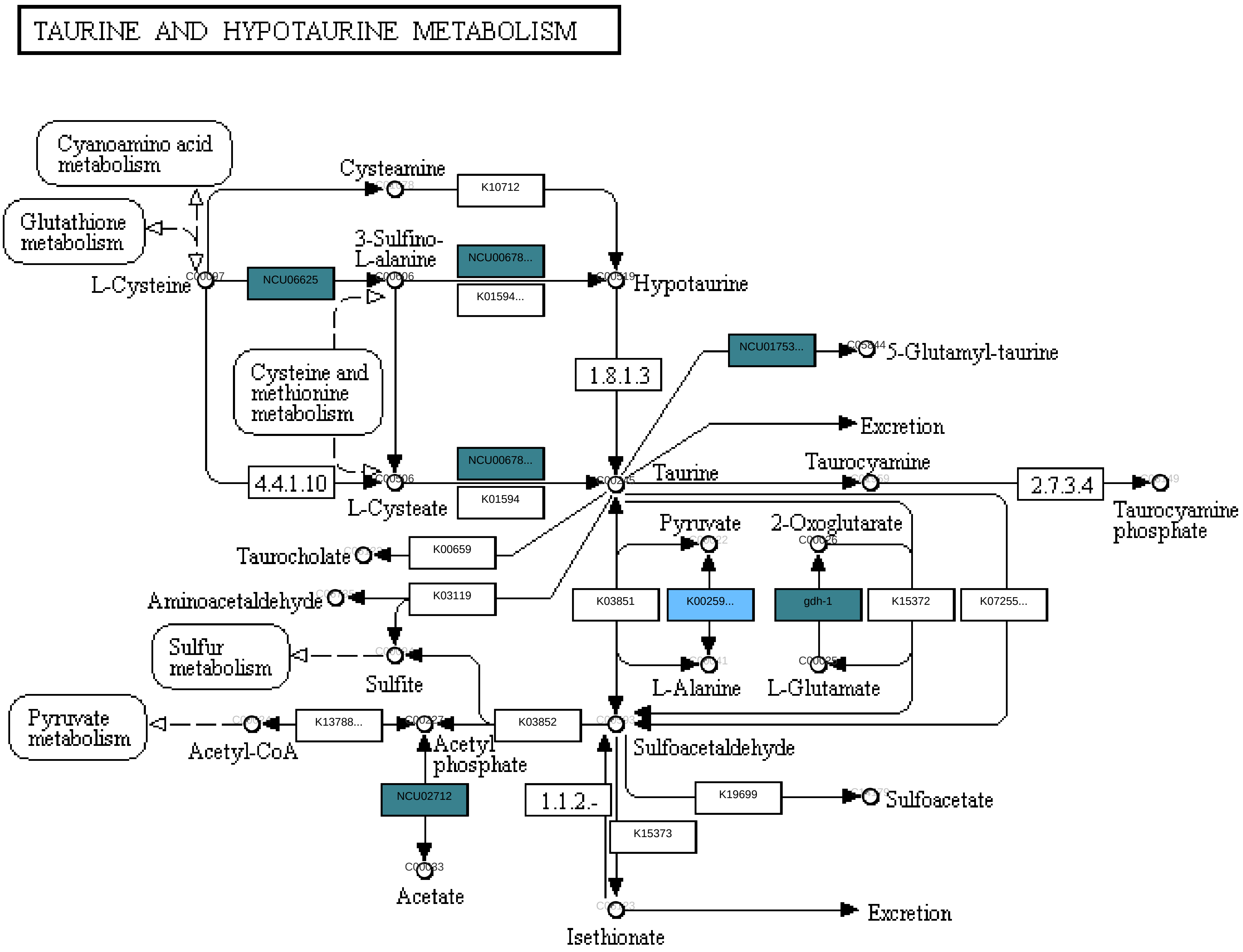


00430 11/18/15
(c) Kanehisa Laboratories

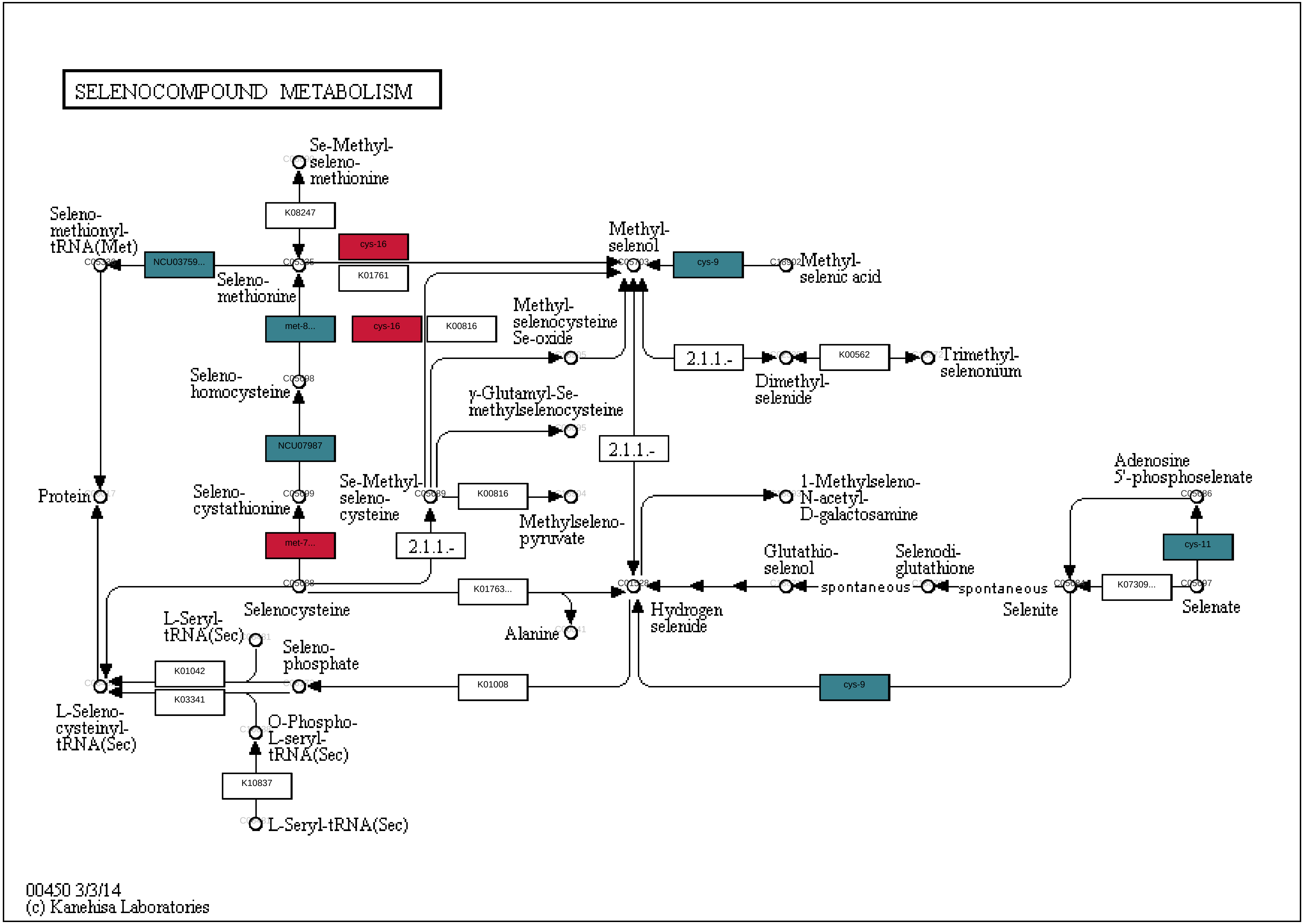
SELENOCOMPOUND METABOLISM
Se-Methyl-seleno-methionine
K08247
Seleno-methionyl-tRNA(Met)
C05336
NCU03759...
C05335
cys-16
K01761
Seleno-methionine
Methyl-selenol
C05703
cys-9
C18902
Methyl-selenic acid
met-8...
cys-16
K00816
Methyl-selenocysteine Se-oxide
2.1.1.-
K00562
Trimethyl-selenonium
Dimethyl-selenide
Seleno-homocysteine
C05698
γ-Glutamyl-Se-methylselenocysteine
NCU07987
2.1.1.-
Adenosine 5'-phosphoselenate
C05686
Protein
Seleno-cystathionine
C05699
Se-Methyl-seleno-cysteine
C05689
K00816
Methylseleno-pyruvate
1-Methylseleno-N-acetyl-D-galactosamine
met-7...
2.1.1.-
cys-11
Glutathio-selenol
Selenodi-glutathione
C05688
K01763...
C01528
spontaneous
spontaneous
C05684
K07309...
C05697
Selenocysteine
Hydrogen selenide
Selenite
Selenate
L-Seryl-tRNA(Sec)
Alanine
Seleno-phosphate
K01042
K03341
K01008
cys-9
L-Seleno-cysteinyl-tRNA(Sec)
O-Phospho-L-seryl-tRNA(Sec)
K10837
L-Seryl-tRNA(Sec)
00450 3/3/14
(c) Kanehisa Laboratories

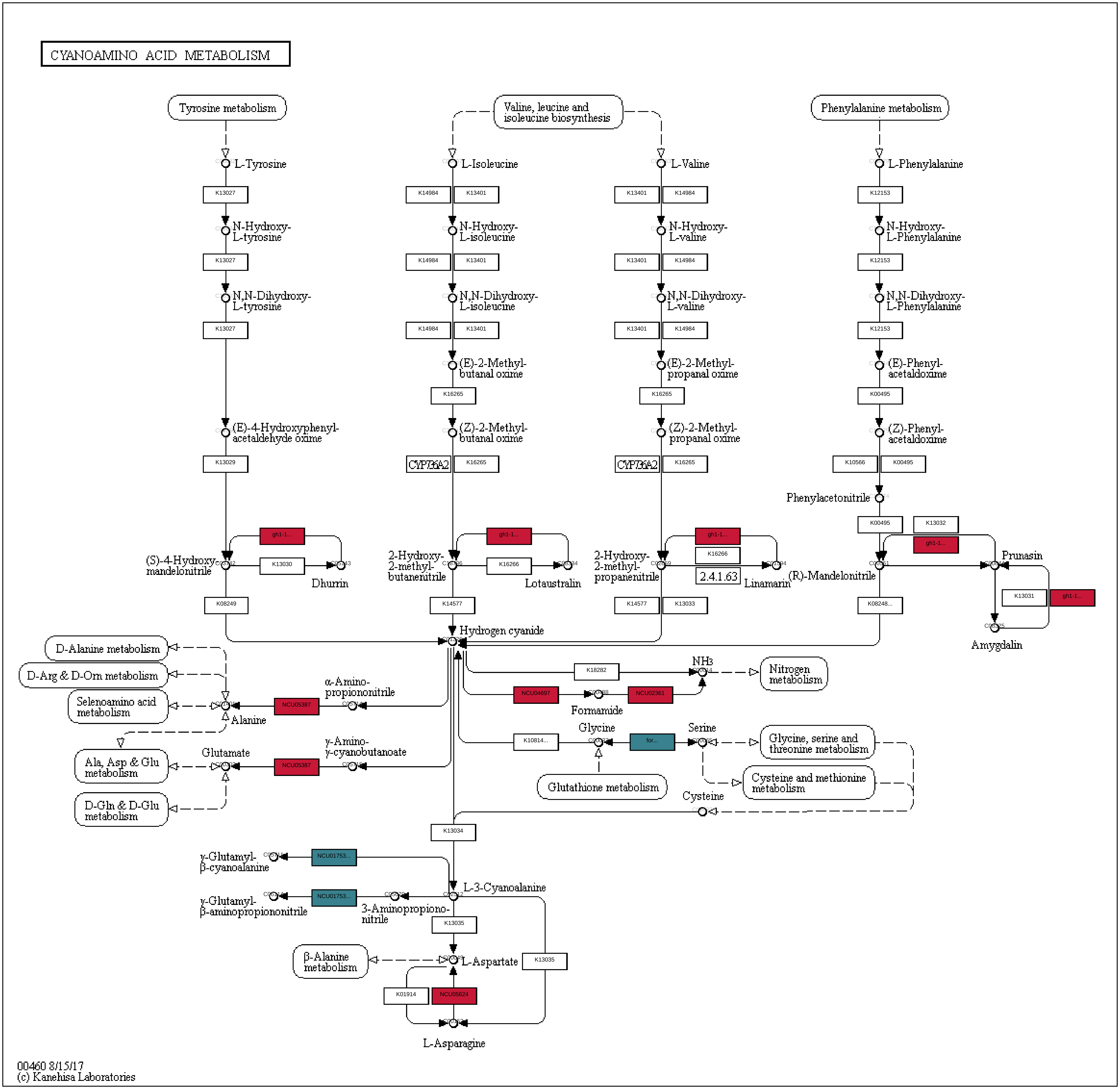

CYANOAMINO ACID METABOLISM
Tyrosine metabolism
Valine, leucine and isoleucine biosynthesis
Phenylalanine metabolism
L-Tyrosine
L-Isoleucine
L-Valine
L-Phenylalanine
K13027
K14984
K13401
K12153
N-Hydroxy-L-tyrosine
N-Hydroxy-L-isoleucine
N-Hydroxy-L-valine
N-Hydroxy-L-Phenylalanine
N,N-Dihydroxy-L-tyrosine
N,N-Dihydroxy-L-isoleucine
N,N-Dihydroxy-L-valine
N,N-Dihydroxy-L-Phenylalanine
(E)-2-Methyl-butanal oxime
(E)-2-Methyl-propanal oxime
(E)-Phenyl-acetaldoxime
K16265
K00495
(E)-4-Hydroxyphenyl-acetaldehyde oxime
(Z)-2-Methyl-butanal oxime
(Z)-2-Methyl-propanal oxime
(Z)-Phenyl-acetaldoxime
K13029
CYP736A2
K10566
Phenylacetonitrile
K13032
ghl-1...
K13030
K16266
2.4.1.63
(S)-4-Hydroxy-mandelonitrile
Dhurrin
2-Hydroxy-2-methyl-butanenitrile
Lotaustralin
2-Hydroxy-2-methyl-propanenitrile
Linamarin
(R)-Mandelonitrile
Prunasin
K13031
Amygdalin
K08249
K14577
K13033
K08248...
Hydrogen cyanide
D-Alanine metabolism
D-Arg & D-Orn metabolism
Selenoamino acid metabolism
NCU05387
α-Amino-propiononitrile
Alanine
K18282
NH3
Nitrogen metabolism
NCU04697
NCU02361
Formamide
Glutamate
γ-Amino-γ-cyanobutanoate
Ala, Asp & Glu metabolism
D-Gln & D-Glu metabolism
K10814...
Glycine
for...
Serine
Glycine, serine and threonine metabolism
Glutathione metabolism
Cysteine and methionine metabolism
Cysteine
K13034
γ-Glutamyl-β-cyanoalanine
NCU01753...
L-3-Cyanoalanine
γ-Glutamyl-β-aminopropiononitrile
3-Aminopropiono-nitrile
K13035
β-Alanine metabolism
L-Aspartate
K01914
NCU05624
L-Asparagine
00460 8/15/17
(c) Kanehisa Laboratories

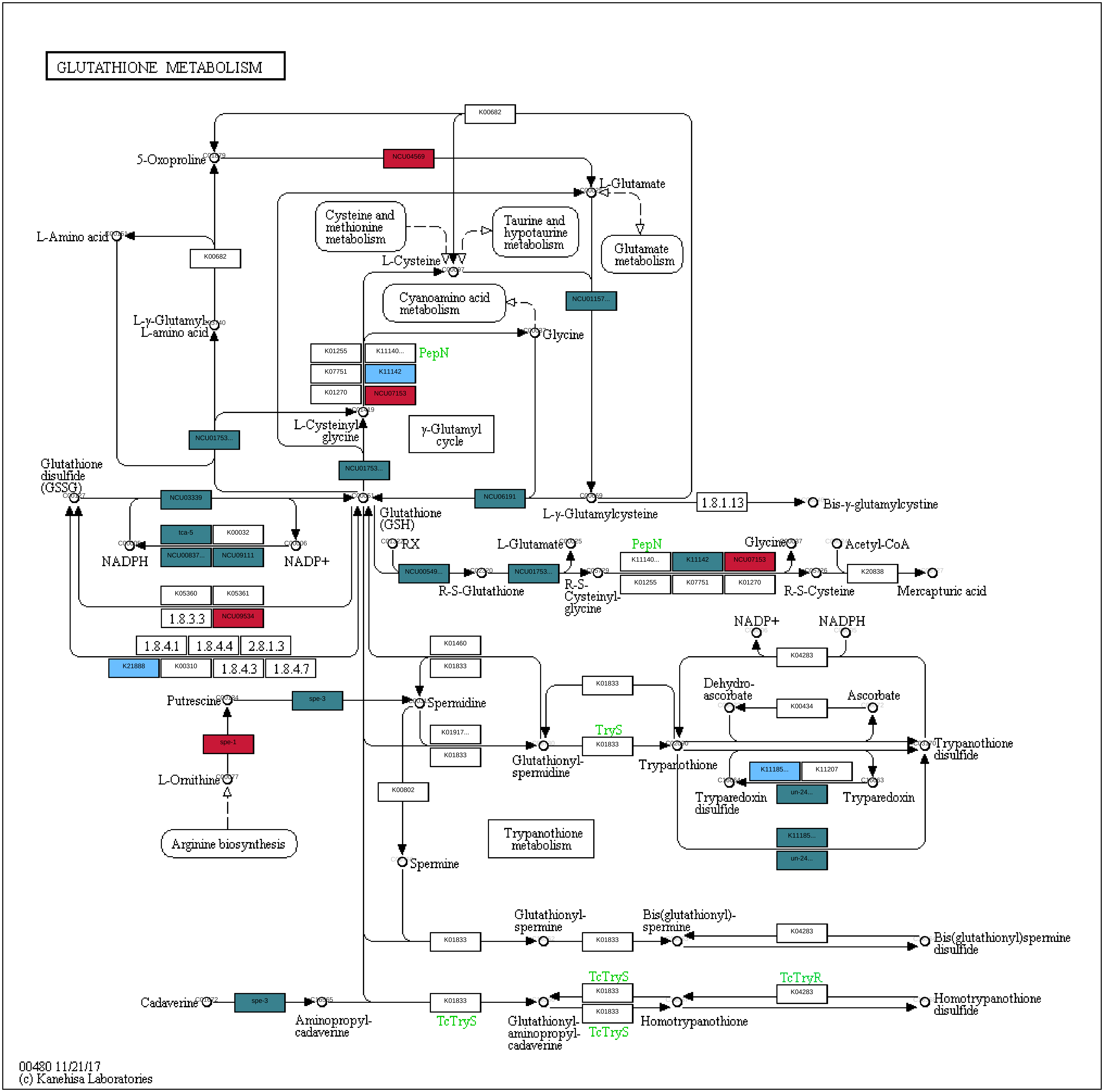
GLUTATHIONE METABOLISM
K00682
5-Oxoproline
NCU04569
L-Glutamate
Cysteine and methionine metabolism
Taurine and hypotaurine metabolism
Glutamate metabolism
L-Amino acid
K00682
L-Cysteine
Cyanoamino acid metabolism
NCU01157...
L-γ-Glutamyl-L-amino acid
Glycine
K01255
K11140...
PepN
K07751
K11142
K01270
NCU07153
L-Cysteinyl-glycine
γ-Glutamyl cycle
NCU01753...
NCU01753...
Glutathione disulfide (GSSG)
NCU03339
NCU06191
Glutathione (GSH)
L-γ-Glutamylcysteine
1.8.1.13
Bis-γ-glutamylcystine
tca-5
K00032
NCU00837...
NCU09111
NADPH
NADP+
RX
L-Glutamate
PepN
Glycine
Acetyl-CoA
NCU00549...
NCU01753...
K11140...
K11142
NCU07153
K20838
K01255
K07751
K01270
R-S-Glutathione
R-S-Cysteinyl-glycine
R-S-Cysteine
Mercapturic acid
K05360
K05361
1.8.3.3
NCU09534
1.8.4.1
1.8.4.4
2.8.1.3
K21888
K00310
1.8.4.3
1.8.4.7
K01460
K01833
NADP+
NADPH
K04283
Putrescine
spe-3
Spermidine
K01833
Dehydro-ascorbate
K00434
Ascorbate
spe-1
K01917...
TryS
K01833
Trypanothione disulfide
K01833
Glutathionyl-spermidine
Trypanothione
K11185...
K11207
L-Ornithine
K00802
un-24...
Tryparedoxin disulfide
Tryparedoxin
Arginine biosynthesis
Trypanothione metabolism
K11185...
un-24...
Spermine
Glutathionyl-spermine
Bis(glutathionyl)-spermine
K04283
K01833
K01833
Bis(glutathionyl)spermine disulfide
TcTryS
TcTryR
K01833
K04283
Cadaverine
spe-3
K01833
Homotrypanothione disulfide
K01833
Aminopropyl-cadaverine
TcTryS
Glutathionyl-aminopropyl-cadaverine
TcTryS
Homotrypanothione
00480 11/21/17
(c) Kanehisa Laboratories

# 7. Glycan biosynthesis and metabolism

| MAP | PATHWAY |
|---|---|
| **510** | N-Glycan biosynthesis |
| **513** | Various types of N-glycan biosynthesis |
| **531** | Glycosaminoglycan degradation |
| **563** | Glycosylphosphatidylinositol (GPI)-anchor biosynthesis |

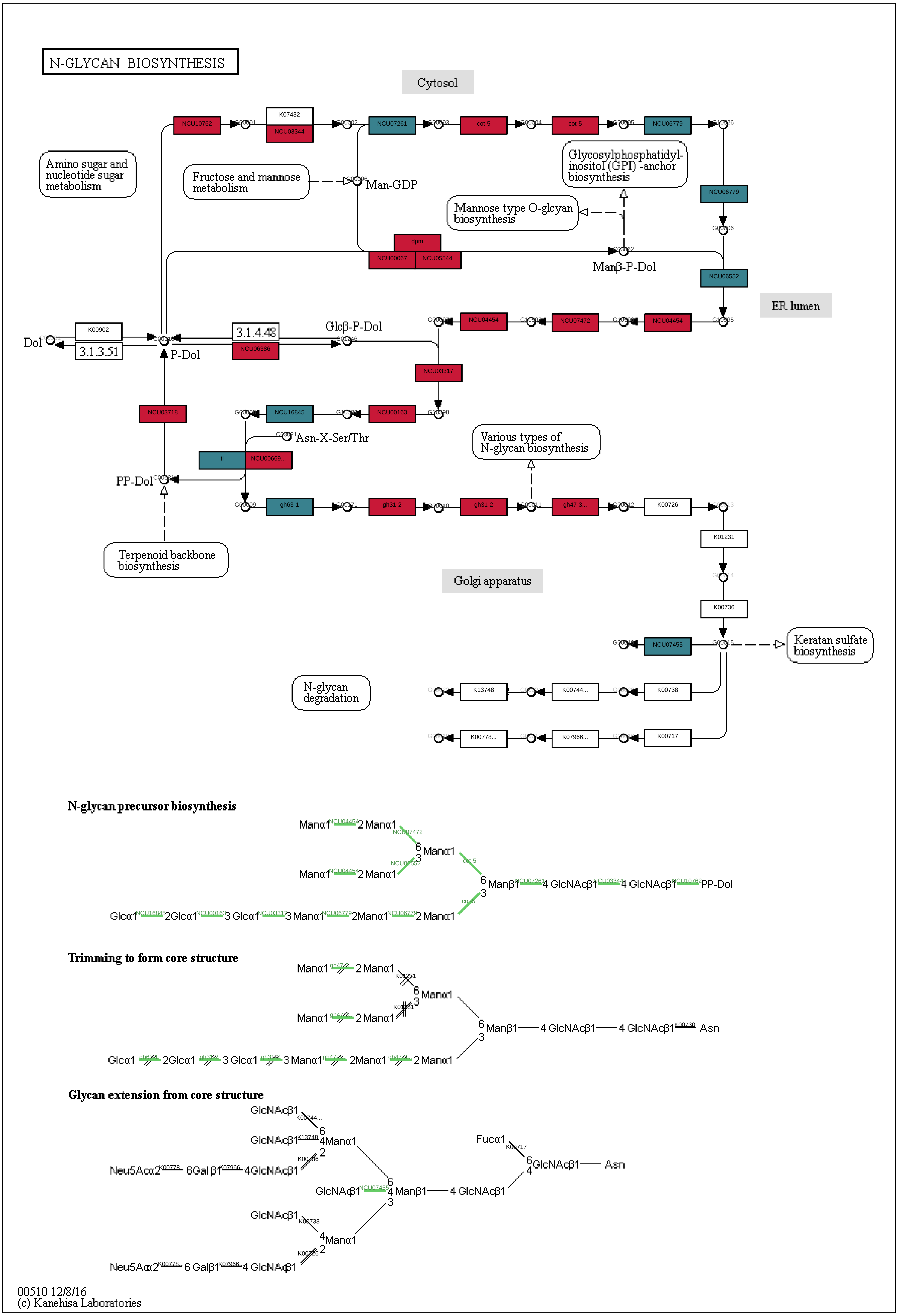
N-GLYCAN BIOSYNTHESIS
Cytosol
ER lumen
Golgi apparatus
Amino sugar and nucleotide sugar metabolism
Fructose and mannose metabolism
Man-GDP
Glycosylphosphatidyl-inositol (GPI) -anchor biosynthesis
Mannose type O-glcyan biosynthesis
Manβ-P-Dol
Dol
P-Dol
PP-Dol
Glcβ-P-Dol
Asn-X-Ser/Thr
K00902
3.1.3.51
3.1.4.48
K07432
NCU10762
NCU03344
NCU07261
cot-5
NCU06779
NCU06552
dpm
NCU00067
NCU05544
NCU06386
NCU04454
NCU07472
NCU03317
NCU03718
NCU16845
NCU00163
ti
NCU00669...
gh63-1
gh31-2
gh47-3...
K00726
K01231
K00736
NCU07455
K13748
K00744...
K00738
K00778...
K07966...
K00717
Various types of N-glycan biosynthesis
Terpenoid backbone biosynthesis
Keratan sulfate biosynthesis
N-glycan degradation
N-glycan precursor biosynthesis
Trimming to form core structure
Glycan extension from core structure
Manα1 2 Manα1
6 Manα1 3
6 Manβ1 4 GlcNAcβ1 4 GlcNAcβ1 PP-Dol
Glcα1 2Glcα1 3 Glcα1 3 Manα1 2Manα1 2 Manα1
Manβ1 4 GlcNAcβ1 4 GlcNAcβ1 Asn
GlcNAcβ1
GlcNAcβ1 4Manα1
Neu5Acα2 6Gal β1 4GlcNAcβ1
GlcNAcβ1 4 Manβ1 4 GlcNAcβ1
Fucα1
6GlcNAcβ1 Asn
Neu5Acα2 6 Galβ1 4 GlcNAcβ1
00510 12/8/16
(c) Kanehisa Laboratories

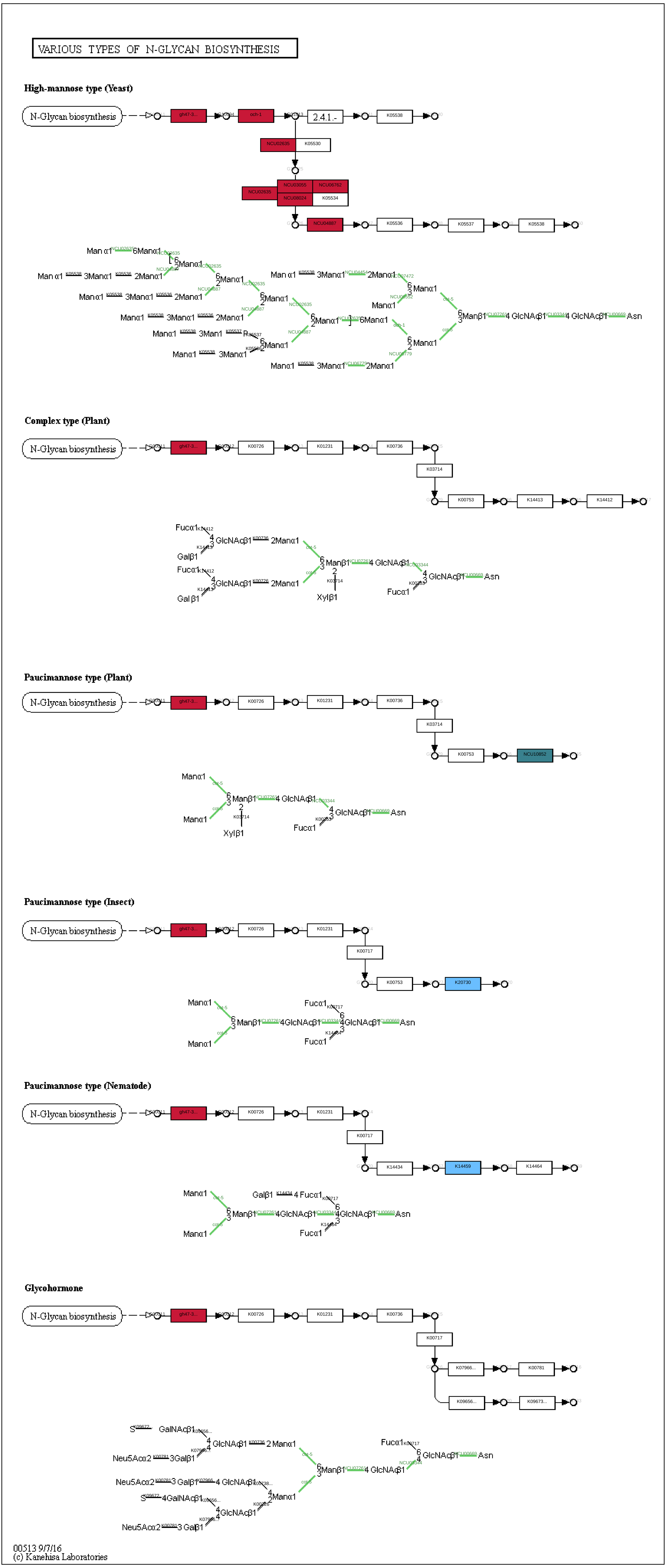
VARIOUS TYPES OF N-GLYCAN BIOSYNTHESIS
High-mannose type (Yeast)
N-Glycan biosynthesis
gh47-3...
och-1
2.4.1.-
K05538
NCU02635
K05530
NCU03055
NCU06762
NCU08024
K05534
NCU04887
K05536
K05537
Complex type (Plant)
K00726
K01231
K00736
K03714
K00753
K14413
K14412
Paucimannose type (Plant)
NCU10852
Paucimannose type (Insect)
K00717
K20730
Paucimannose type (Nematode)
K14434
K14459
K14464
Glycohormone
K07966...
K00781
K09656...
K09673...
00513 9/7/16
(c) Kanehisa Laboratories

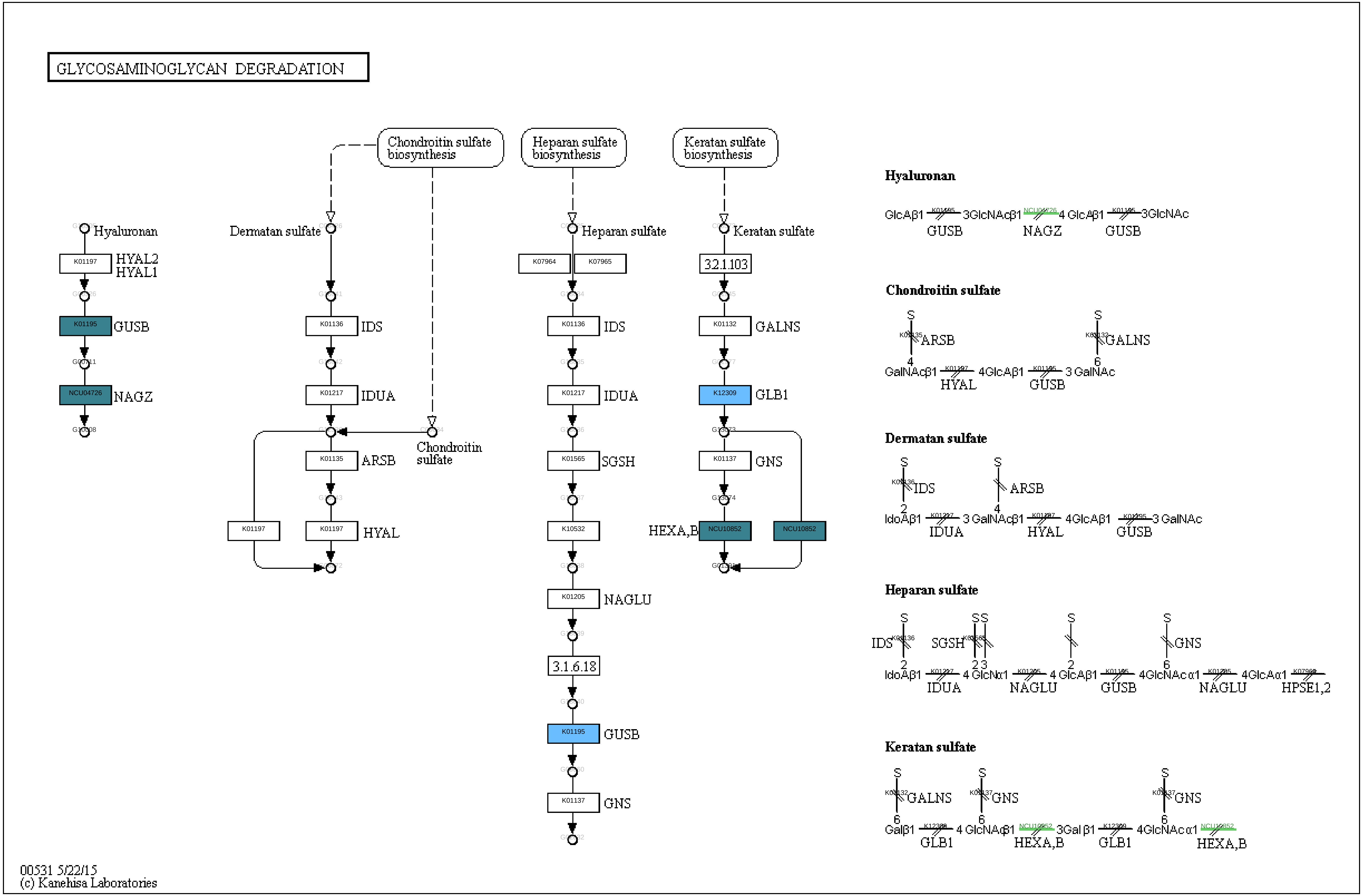
GLYCOSAMINOGLYCAN DEGRADATION
Chondroitin sulfate biosynthesis
Heparan sulfate biosynthesis
Keratan sulfate biosynthesis
Hyaluronan
K01197
HYAL2
HYAL1
K01195
GUSB
NCU04726
NAGZ
Dermatan sulfate
K01136
IDS
K01217
IDUA
K01135
ARSB
K01197
K01197
HYAL
Chondroitin sulfate
Heparan sulfate
K07964
K07965
K01136
IDS
K01217
IDUA
K01565
SGSH
K10532
K01205
NAGLU
3.1.6.18
K01195
GUSB
K01137
GNS
Keratan sulfate
3.2.1.103
K01132
GALNS
K12309
GLB1
K01137
GNS
HEXA,B
NCU10852
NCU10852
Hyaluronan
GlcAβ1 3GlcNAcβ1 4 GlcAβ1 3GlcNAc
GUSB
NAGZ
GUSB
Chondroitin sulfate
S
ARSB
S
GALNS
4
6
GalNAcβ1 4GlcAβ1 3 GalNAc
HYAL
GUSB
Dermatan sulfate
S
IDS
S
ARSB
2
4
IdoAβ1 3 GalNAcβ1 4GlcAβ1 3 GalNAc
IDUA
HYAL
GUSB
Heparan sulfate
S
SS
S
S
IDS
SGSH
GNS
2
2 3
2
6
IdoAβ1 4 GlcNα1 4 GlcAβ1 4GlcNAcα1 4GlcAα1
IDUA
NAGLU
GUSB
NAGLU
HPSE1,2
Keratan sulfate
S
S
S
GALNS
GNS
GNS
6
6
6
Galβ1 4 GlcNAcβ1 3Gal β1 4GlcNAcα1
GLB1
HEXA,B
GLB1
HEXA,B
00531 5/22/15
(c) Kanehisa Laboratories

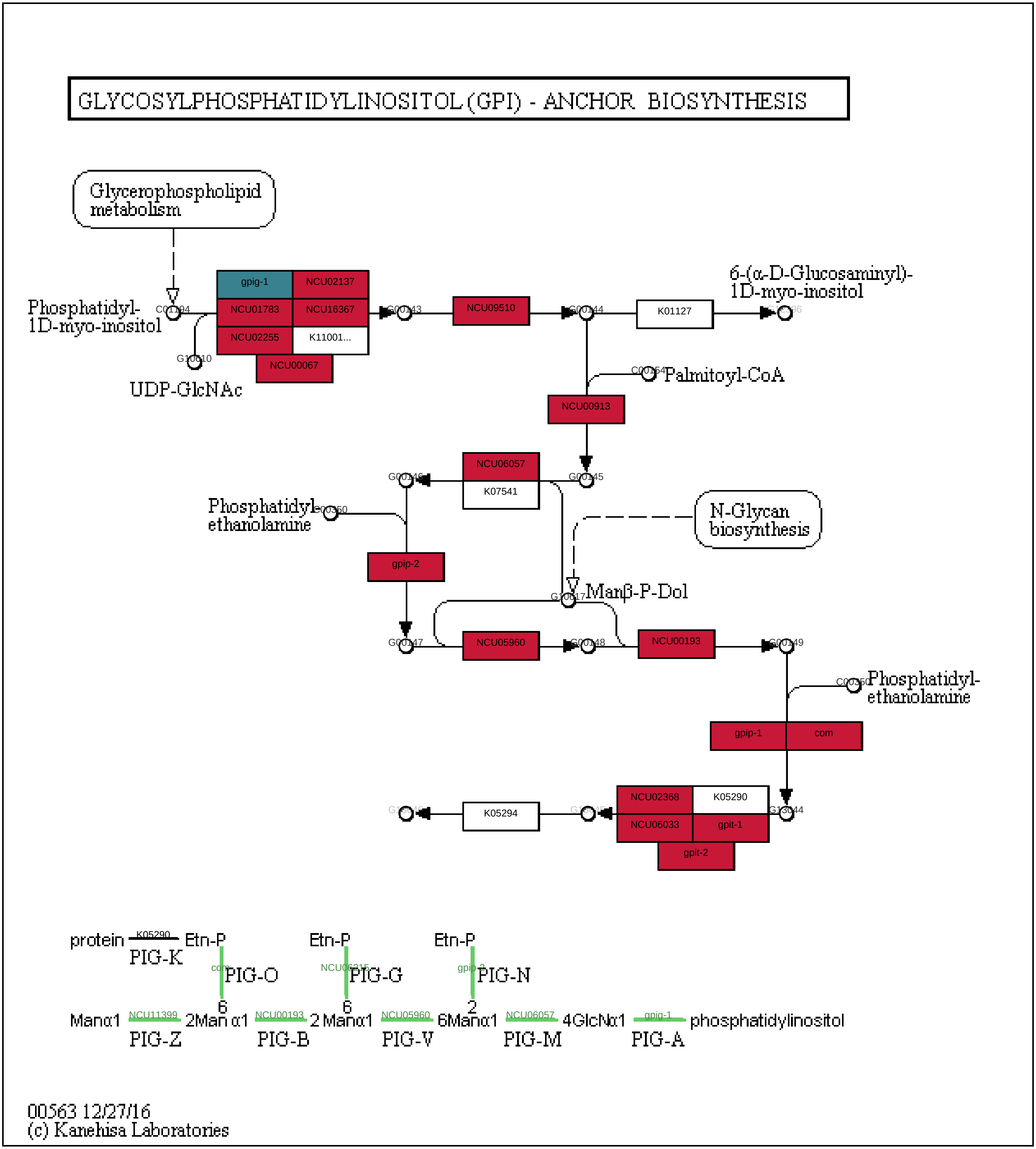
GLYCOSYLPHOSPHATIDYLINOSITOL (GPI) - ANCHOR BIOSYNTHESIS
Glycerophospholipid metabolism
Phosphatidyl-1D-myo-inositol
C01194
gpig-1
NCU02137
NCU01783
NCU16367
NCU02255
K11001...
NCU00067
G10610
UDP-GlcNAc
G00143
NCU09510
G00144
K01127
6-(α-D-Glucosaminyl)-1D-myo-inositol
C00154
Palmitoyl-CoA
NCU00913
NCU06057
K07541
G00146
G00145
Phosphatidylethanolamine
C00350
N-Glycan biosynthesis
gpip-2
Manβ-P-Dol
G10617
G00147
NCU05960
G00148
NCU00193
G00149
C00350
Phosphatidylethanolamine
gpip-1
com
NCU02368
K05290
NCU06033
gpit-1
gpit-2
G13044
K05294
protein
K05290
PIG-K
Etn-P
com
PIG-O
Etn-P
NCU06215
PIG-G
Etn-P
gpip-2
PIG-N
6
6
2
Manα1
NCU11399
2Man α1
NCU00193
2 Manα1
NCU05960
6Manα1
NCU06057
4GlcNα1
gpig-1
phosphatidylinositol
PIG-Z
PIG-B
PIG-V
PIG-M
PIG-A
00563 12/27/16
(c) Kanehisa Laboratories

# 8. Metabolism of cofactors and vitamins

| MAP | PATHWAY |
|---|---|
| **130** | Ubiquinone and other terpenoid-quinone biosynthesis |
| **670** | One carbon pool by folate |
| **730** | Thiamine metabolism |
| **740** | Riboflavin metabolism |
| **750** | Vitamin B6 metabolism |
| **760** | Nicotinate and nicotinamide metabolism |
| **770** | Pantothenate and CoA biosynthesis |
| **780** | Biotin metabolism |
| **785** | Lipoic acid metabolism |
| **790** | Folate biosynthesis |
| **860** | Porphyrin and chlorophyll metabolism |

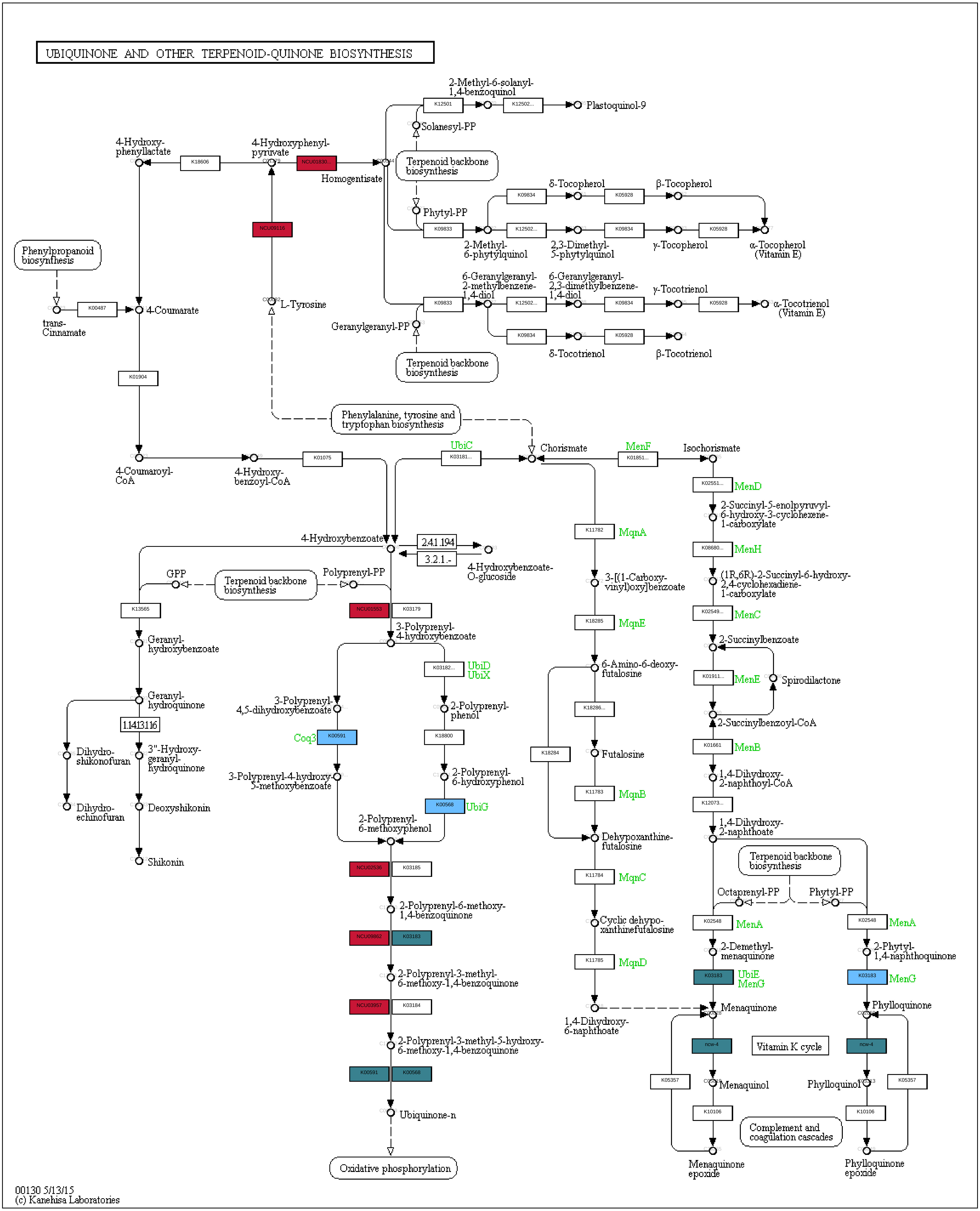
UBIQUINONE AND OTHER TERPENOID-QUINONE BIOSYNTHESIS
2-Methyl-6-solanyl-1,4-benzoquinol
K12501
K12502...
Plastoquinol-9
Solanesyl-PP
4-Hydroxy-phenyllactate
K18606
4-Hydroxyphenyl-pyruvate
NCU01830...
Homogentisate
Terpenoid backbone biosynthesis
δ-Tocopherol
β-Tocopherol
K09834
K05928
Phytyl-PP
K09833
K12502...
K09834
K05928
2-Methyl-6-phytylquinol
2,3-Dimethyl-5-phytylquinol
γ-Tocopherol
α-Tocopherol (Vitamin E)
NCU09116
Phenylpropanoid biosynthesis
6-Geranylgeranyl-2-methylbenzene-1,4-diol
6-Geranylgeranyl-2,3-dimethylbenzene-1,4-diol
γ-Tocotrienol
K00487
4-Coumarate
L-Tyrosine
K09833
K12502...
K09834
K05928
α-Tocotrienol (Vitamin E)
trans-Cinnamate
Geranylgeranyl-PP
K09834
K05928
δ-Tocotrienol
β-Tocotrienol
Terpenoid backbone biosynthesis
K01904
Phenylalanine, tyrosine and tryptophan biosynthesis
UbiC
K03181...
Chorismate
MenF
K01851...
Isochorismate
4-Coumaroyl-CoA
4-Hydroxy-benzoyl-CoA
K01075
K02551...
MenD
2-Succinyl-5-enolpyruvyl-6-hydroxy-3-cyclohexene-1-carboxylate
K11782
MqnA
4-Hydroxybenzoate
2.4.1.194
3.2.1.-
4-Hydroxybenzoate-O-glucoside
K08680...
MenH
GPP
Terpenoid backbone biosynthesis
Polyprenyl-PP
3-[(1-Carboxy-vinyl)oxy]benzoate
(1R,6R)-2-Succinyl-6-hydroxy-2,4-cyclohexadiene-1-carboxylate
K13565
NCU01553
K03179
K18285
MqnE
K02549...
MenC
Geranyl-hydroxybenzoate
3-Polyprenyl-4-hydroxybenzoate
2-Succinylbenzoate
K03182...
UbiD
UbiX
6-Amino-6-deoxy-futalosine
K01911...
MenE
Spirodilactone
Geranyl-hydroquinone
3-Polyprenyl-4,5-dihydroxybenzoate
2-Polyprenyl-phenol
1.14.13.116
K18286...
2-Succinylbenzoyl-CoA
Coq3
K00591
K18800
K18284
Dihydro-shikonofuran
3''-Hydroxy-geranyl-hydroquinone
Futalosine
K01661
MenB
3-Polyprenyl-4-hydroxy-5-methoxybenzoate
2-Polyprenyl-6-hydroxyphenol
1,4-Dihydroxy-2-naphthoyl-CoA
K11783
MqnB
Dihydro-echinofuran
Deoxyshikonin
K00568
UbiG
K12073...
2-Polyprenyl-6-methoxyphenol
1,4-Dihydroxy-2-naphthoate
Dehypoxanthine-futalosine
Shikonin
Terpenoid backbone biosynthesis
NCU02536
K03185
K11784
MqnC
Octaprenyl-PP
Phytyl-PP
2-Polyprenyl-6-methoxy-1,4-benzoquinone
Cyclic dehypo-xanthinefutalosine
K02548
MenA
K02548
MenA
NCU09862
K03183
2-Demethyl-menaquinone
2-Phytyl-1,4-naphthoquinone
2-Polyprenyl-3-methyl-6-methoxy-1,4-benzoquinone
K11785
MqnD
K03183
UbiE
MenG
K03183
MenG
NCU03957
K03184
1,4-Dihydroxy-6-naphthoate
Menaquinone
Phylloquinone
2-Polyprenyl-3-methyl-5-hydroxy-6-methoxy-1,4-benzoquinone
ncw-4
Vitamin K cycle
ncw-4
K00591
K00568
K05357
Menaquinol
Phylloquinol
K05357
Ubiquinone-n
K10106
Complement and coagulation cascades
K10106
Oxidative phosphorylation
Menaquinone epoxide
Phylloquinone epoxide
00130 5/13/15
(c) Kanehisa Laboratories

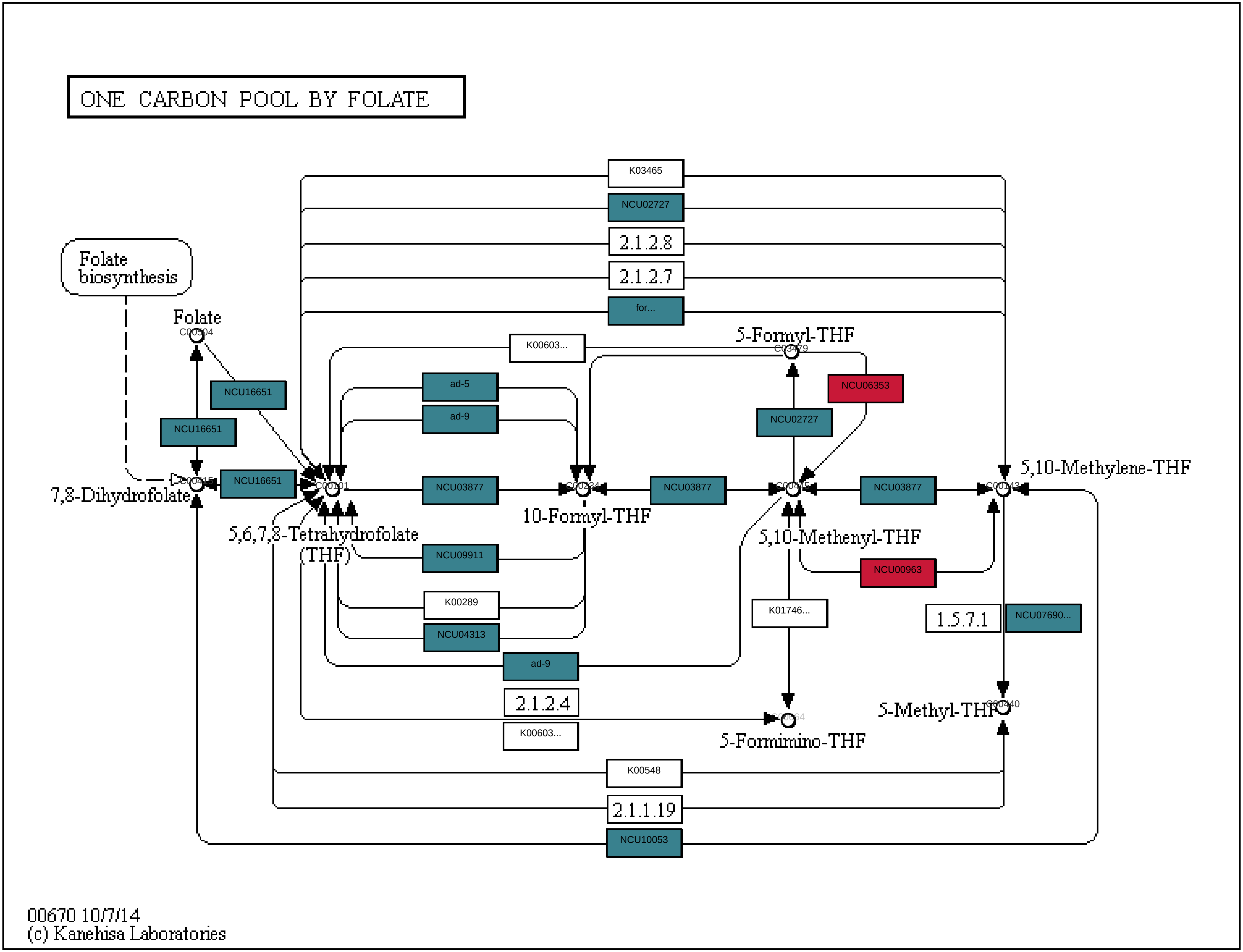
ONE CARBON POOL BY FOLATE
K03465
NCU02727
2.1.2.8
2.1.2.7
for...
Folate biosynthesis
Folate
C00504
5-Formyl-THF
C03479
K00603...
NCU16651
NCU16651
ad-5
ad-9
NCU06353
NCU02727
7,8-Dihydrofolate
C00415
NCU16651
C00101
NCU03877
C00234
NCU03877
C00445
NCU03877
C00143
5,10-Methylene-THF
10-Formyl-THF
5,6,7,8-Tetrahydrofolate (THF)
5,10-Methenyl-THF
NCU09911
NCU00963
K00289
K01746...
1.5.7.1
NCU07690...
NCU04313
ad-9
2.1.2.4
K00603...
5-Methyl-THF
C00440
5-Formimino-THF
K00548
2.1.1.19
NCU10053
00670 10/7/14
(c) Kanehisa Laboratories

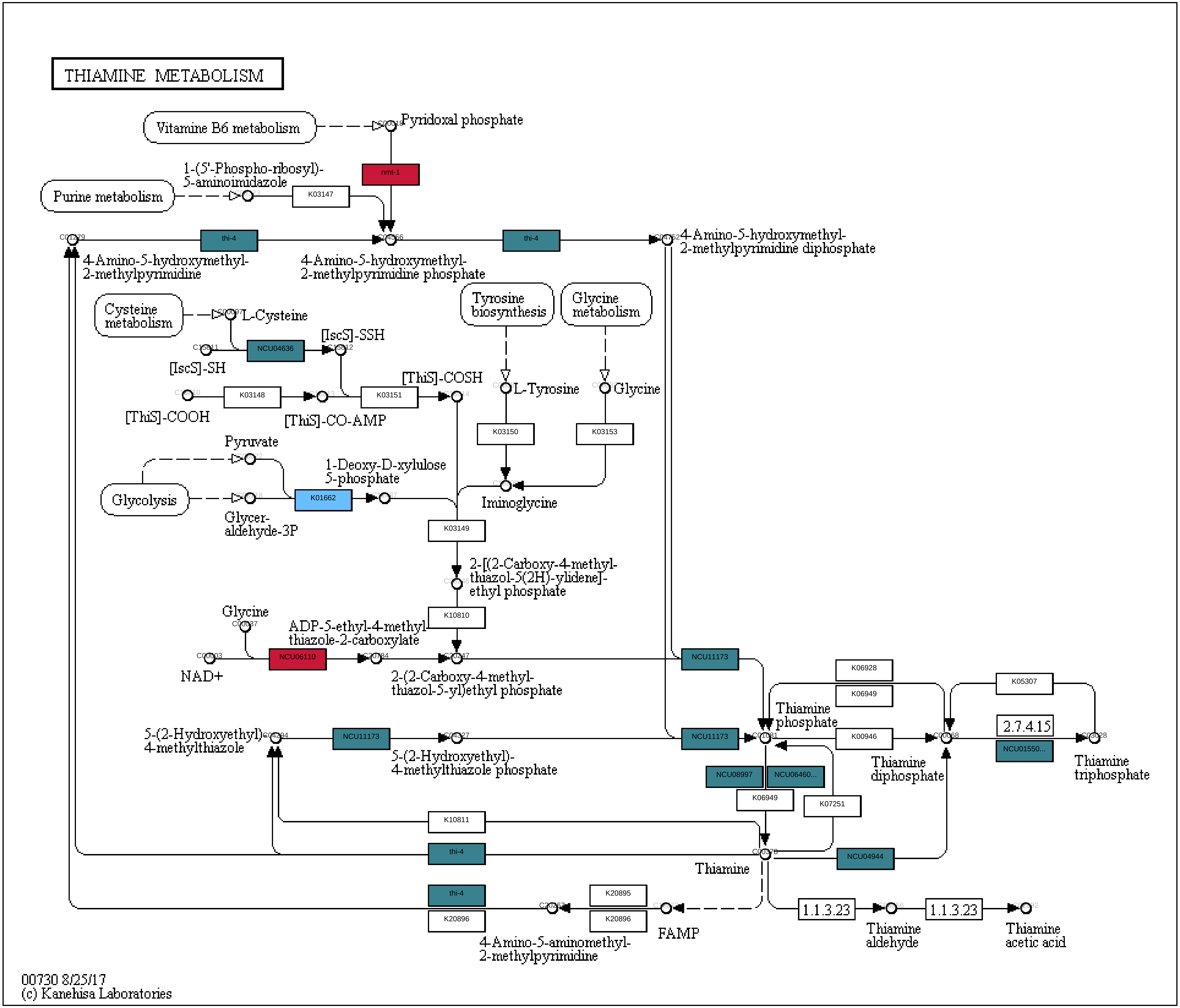
THIAMINE METABOLISM
Vitamine B6 metabolism
Pyridoxal phosphate
nmt-1
1-(5'-Phospho-ribosyl)-
5-aminoimidazole
Purine metabolism
K03147
thi-4
thi-4
4-Amino-5-hydroxymethyl-
2-methylpyrimidine diphosphate
4-Amino-5-hydroxymethyl-
2-methylpyrimidine
4-Amino-5-hydroxymethyl-
2-methylpyrimidine phosphate
Cysteine
metabolism
L-Cysteine
Tyrosine
biosynthesis
Glycine
metabolism
NCU04636
[IscS]-SSH
[IscS]-SH
[ThiS]-COSH
K03148
K03151
L-Tyrosine
Glycine
[ThiS]-COOH
[ThiS]-CO-AMP
K03150
K03153
Pyruvate
1-Deoxy-D-xylulose
5-phosphate
Glycolysis
K01662
Iminoglycine
Glycer-
aldehyde-3P
K03149
2-[(2-Carboxy-4-methyl-
thiazol-5(2H)-ylidene]-
ethyl phosphate
Glycine
K10810
ADP-5-ethyl-4-methyl-
thiazole-2-carboxylate
NCU06110
NAD+
NCU11173
2-(2-Carboxy-4-methyl-
thiazol-5-yl)ethyl phosphate
K06928
K06949
K05307
Thiamine
phosphate
2.7.4.15
5-(2-Hydroxyethyl)-
4-methylthiazole
NCU11173
NCU11173
K00946
5-(2-Hydroxyethyl)-
4-methylthiazole phosphate
NCU01550...
Thiamine
diphosphate
Thiamine
triphosphate
NCU08997
NCU06460...
K06949
K07251
K10811
thi-4
NCU04944
Thiamine
thi-4
K20895
1.1.3.23
1.1.3.23
K20896
K20896
FAMP
Thiamine
aldehyde
Thiamine
acetic acid
4-Amino-5-aminomethyl-
2-methylpyrimidine
00730 8/25/17
(c) Kanehisa Laboratories

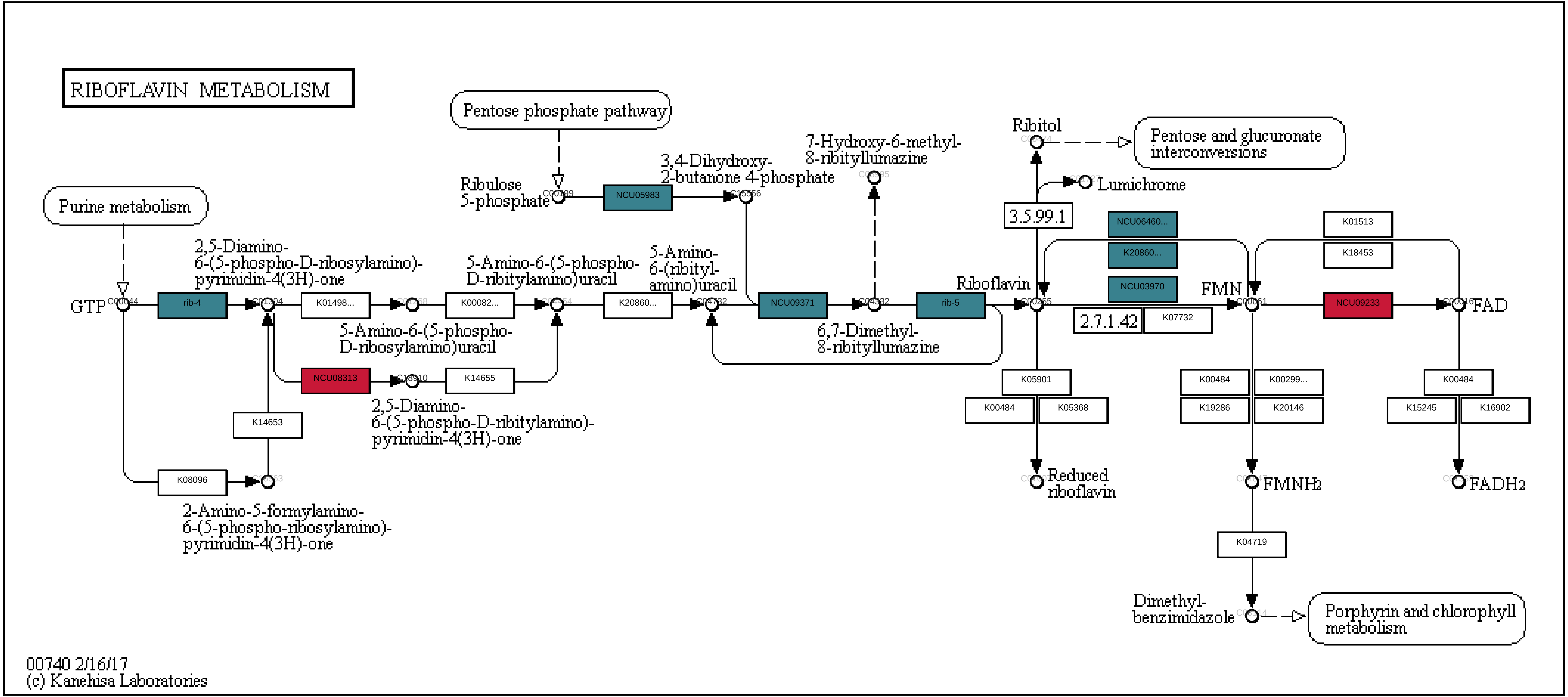

RIBOFLAVIN METABOLISM
Pentose phosphate pathway
Purine metabolism
Ribulose 5-phosphate
NCU05983
3,4-Dihydroxy-2-butanone 4-phosphate
7-Hydroxy-6-methyl-8-ribityllumazine
Ribitol
Pentose and glucuronate interconversions
Lumichrome
3.5.99.1
2,5-Diamino-6-(5-phospho-D-ribosylamino)-pyrimidin-4(3H)-one
5-Amino-6-(5-phospho-D-ribitylamino)uracil
5-Amino-6-(ribitylamino)uracil
GTP
rib-4
K01498...
K00082...
K20860...
NCU09371
rib-5
Riboflavin
NCU06460...
K20860...
NCU03970
2.7.1.42
K07732
FMN
K01513
K18453
NCU09233
FAD
5-Amino-6-(5-phospho-D-ribosylamino)uracil
6,7-Dimethyl-8-ribityllumazine
NCU08313
K14655
2,5-Diamino-6-(5-phospho-D-ribitylamino)-pyrimidin-4(3H)-one
K14653
K08096
2-Amino-5-formylamino-6-(5-phospho-ribosylamino)-pyrimidin-4(3H)-one
K05901
K00484
K05368
Reduced riboflavin
K00484
K00299...
K19286
K20146
FMNH2
K04719
Dimethylbenzimidazole
Porphyrin and chlorophyll metabolism
K00484
K15245
K16902
FADH2
00740 2/16/17
(c) Kanehisa Laboratories

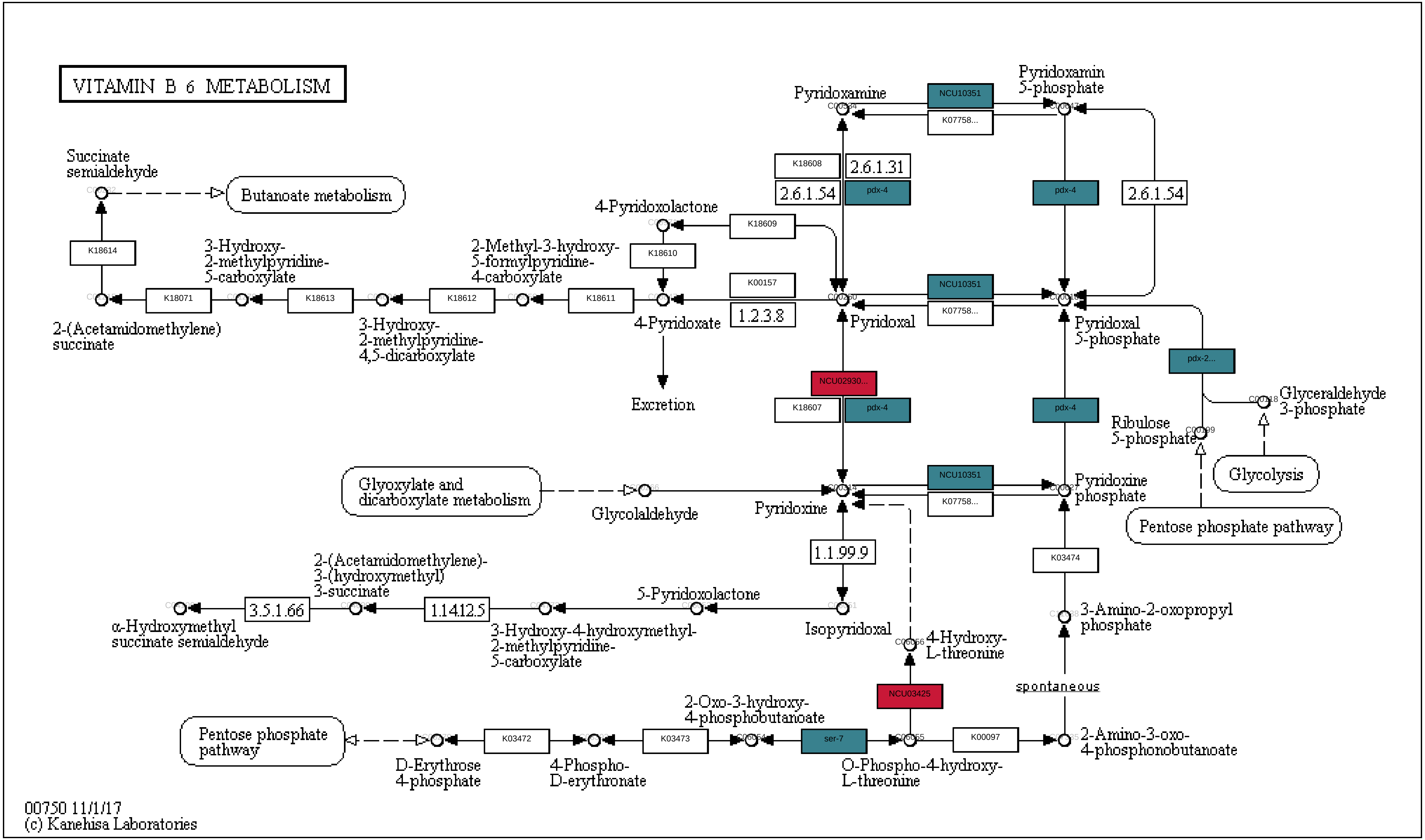
VITAMIN B 6 METABOLISM
Succinate semialdehyde
Butanoate metabolism
K18614
3-Hydroxy-2-methylpyridine-5-carboxylate
2-Methyl-3-hydroxy-5-formylpyridine-4-carboxylate
4-Pyridoxolactone
K18609
K18610
K18071
K18613
K18612
K18611
K00157
1.2.3.8
2-(Acetamidomethylene) succinate
3-Hydroxy-2-methylpyridine-4,5-dicarboxylate
4-Pyridoxate
Excretion
Pyridoxamine
NCU10351
K07758...
Pyridoxamin 5-phosphate
K18608
2.6.1.31
2.6.1.54
pdx-4
Pyridoxal
Pyridoxal 5-phosphate
pdx-2...
NCU02930...
K18607
Glyceraldehyde 3-phosphate
Ribulose 5-phosphate
Glycolysis
Glyoxylate and dicarboxylate metabolism
Glycolaldehyde
Pyridoxine
Pyridoxine phosphate
Pentose phosphate pathway
1.1.99.9
K03474
2-(Acetamidomethylene)-3-(hydroxymethyl)-3-succinate
5-Pyridoxolactone
3.5.1.66
1.14.12.5
α-Hydroxymethyl succinate semialdehyde
3-Hydroxy-4-hydroxymethyl-2-methylpyridine-5-carboxylate
Isopyridoxal
4-Hydroxy-L-threonine
3-Amino-2-oxopropyl phosphate
spontaneous
NCU03425
2-Oxo-3-hydroxy-4-phosphobutanoate
K03472
K03473
ser-7
K00097
D-Erythrose 4-phosphate
4-Phospho-D-erythronate
O-Phospho-4-hydroxy-L-threonine
2-Amino-3-oxo-4-phosphonobutanoate
00750 11/1/17
(c) Kanehisa Laboratories

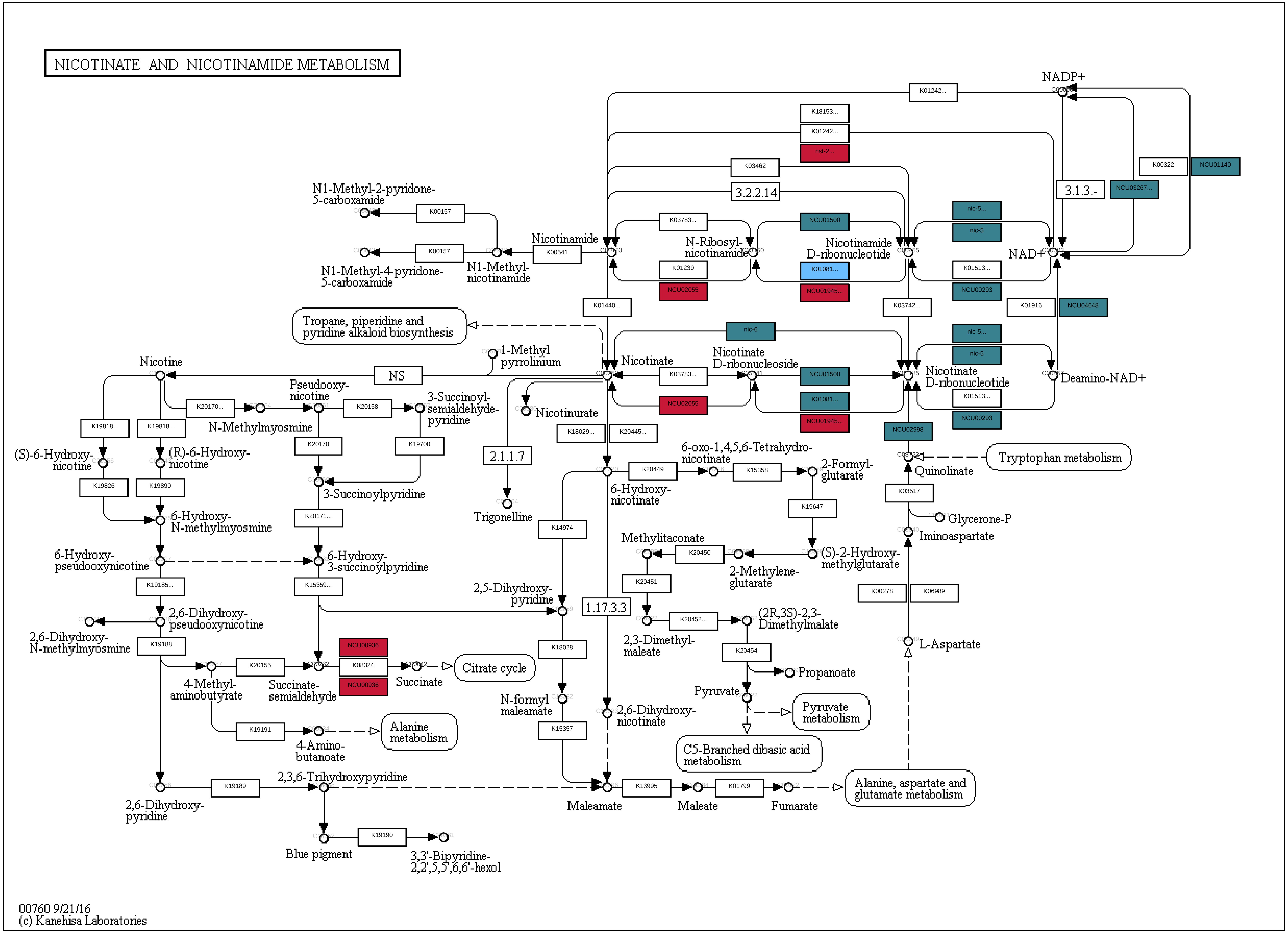
NICOTINATE AND NICOTINAMIDE METABOLISM
NADP+
K01242...
K18153...
K01242...
nst-2...
K03462
3.2.2.14
K00322
NCU01140
3.1.3.-
NCU03267...
N1-Methyl-2-pyridone-5-carboxamide
K00157
K00157
N1-Methyl-4-pyridone-5-carboxamide
K00541
N1-Methyl-nicotinamide
Nicotinamide
K03783...
N-Ribosyl-nicotinamide
NCU01500
Nicotinamide D-ribonucleotide
nic-5...
nic-5
NAD+
K01239
NCU02055
K01081...
NCU01945...
K01513...
NCU00293
K01440...
K03742...
K01916
NCU04648
Tropane, piperidine and pyridine alkaloid biosynthesis
1-Methyl pyrrolinium
nic-6
Nicotinate
Nicotinate D-ribonucleoside
nic-5...
nic-5
Nicotinate D-ribonucleotide
Deamino-NAD+
Nicotine
NS
K03783...
NCU01500
K01513...
NCU02055
K01081...
NCU00293
NCU01945...
NCU02998
K20170...
Pseudooxy-nicotine
K20158
3-Succinoyl-semialdehyde-pyridine
Nicotinurate
K19818...
K19818...
N-Methylmyosmine
K20170
K19700
K18029...
K20445...
(S)-6-Hydroxy-nicotine
(R)-6-Hydroxy-nicotine
2.1.1.7
6-oxo-1,4,5,6-Tetrahydro-nicotinate
2-Formyl-glutarate
Tryptophan metabolism
K20449
K15358
Quinolinate
K19826
K19890
3-Succinoylpyridine
6-Hydroxy-nicotinate
K03517
Trigonelline
K19647
6-Hydroxy-N-methylmyosmine
K20171...
K14974
Glycerone-P
Methylitaconate
Iminoaspartate
6-Hydroxy-pseudooxynicotine
6-Hydroxy-3-succinoylpyridine
K20450
(S)-2-Hydroxy-methylglutarate
2-Methylene-glutarate
K20451
K00278
K06989
K19185...
K15359...
2,5-Dihydroxy-pyridine
1.17.3.3
2,6-Dihydroxy-pseudooxynicotine
K20452...
(2R,3S)-2,3-Dimethylmalate
2,6-Dihydroxy-N-methylmyosmine
K19188
NCU00936
K18028
2,3-Dimethyl-maleate
L-Aspartate
K20154
K20155
K08324
Citrate cycle
NCU00936
K20454
4-Methyl-aminobutyrate
Succinate-semialdehyde
Succinate
Propanoate
Pyruvate
N-formyl maleamate
2,6-Dihydroxy-nicotinate
Pyruvate metabolism
K19191
Alanine metabolism
K15357
4-Amino-butanoate
C5-Branched dibasic acid metabolism
2,3,6-Trihydroxypyridine
K19189
K13995
K01799
Alanine, aspartate and glutamate metabolism
2,6-Dihydroxy-pyridine
Maleamate
Maleate
Fumarate
K19190
Blue pigment
3,3'-Bipyridine-2,2',5,5',6,6'-hexol
00760 9/21/16
(c) Kanehisa Laboratories

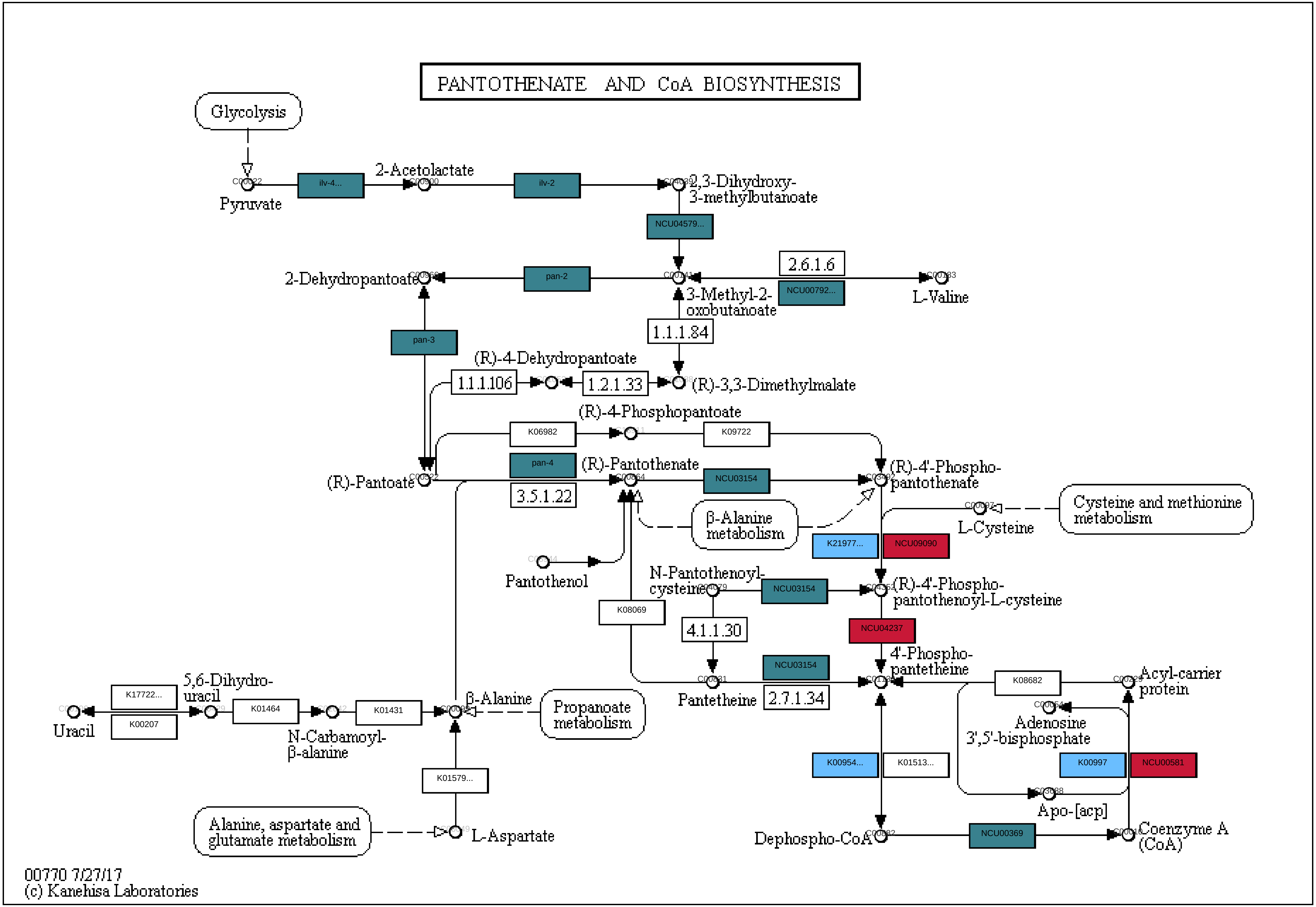

PANTOTHENATE AND CoA BIOSYNTHESIS
Glycolysis
Pyruvate
C00022
ilv-4...
2-Acetolactate
ilv-2
2,3-Dihydroxy-
3-methylbutanoate
NCU04579...
2.6.1.6
NCU00792...
L-Valine
2-Dehydropantoate
pan-2
3-Methyl-2-
oxobutanoate
1.1.1.84
pan-3
(R)-4-Dehydropantoate
1.1.1.106
1.2.1.33
(R)-3,3-Dimethylmalate
(R)-4-Phosphopantoate
K06982
K09722
pan-4
(R)-Pantothenate
(R)-Pantoate
NCU03154
(R)-4'-Phospho-
pantothenate
3.5.1.22
β-Alanine
metabolism
Cysteine and methionine
metabolism
L-Cysteine
K21977...
NCU09090
Pantothenol
N-Pantothenoyl-
cysteine
NCU03154
(R)-4'-Phospho-
pantothenoyl-L-cysteine
K08069
4.1.1.30
NCU04237
4'-Phospho-
pantetheine
NCU03154
Pantetheine
2.7.1.34
K08682
Acyl-carrier
protein
Adenosine
3',5'-bisphosphate
K17722...
K00207
5,6-Dihydro-
uracil
K01464
K01431
β-Alanine
Propanoate
metabolism
Uracil
N-Carbamoyl-
β-alanine
K00954...
K01513...
K00997
NCU00581
K01579...
Apo-[acp]
Alanine, aspartate and
glutamate metabolism
L-Aspartate
Dephospho-CoA
NCU00369
Coenzyme A
(CoA)
00770 7/27/17
(c) Kanehisa Laboratories

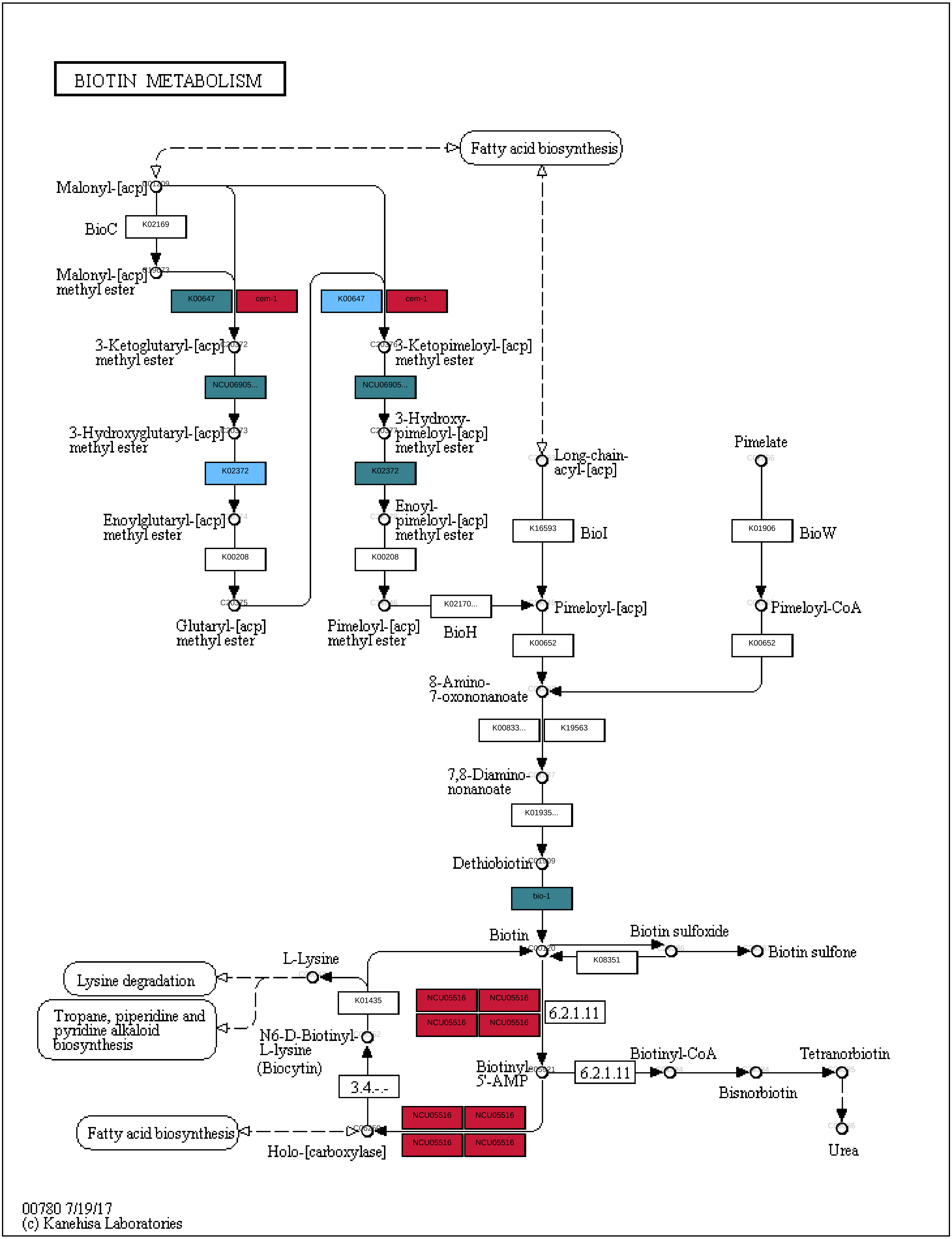
BIOTIN METABOLISM
Fatty acid biosynthesis
Malonyl-[acp]
BioC
K02169
Malonyl-[acp] methyl ester
K00647
cem-1
K00647
cem-1
3-Ketoglutaryl-[acp] methyl ester
3-Ketopimeloyl-[acp] methyl ester
NCU06905...
NCU06905...
3-Hydroxyglutaryl-[acp] methyl ester
3-Hydroxy-pimeloyl-[acp] methyl ester
K02372
K02372
Enoylglutaryl-[acp] methyl ester
Enoyl-pimeloyl-[acp] methyl ester
K00208
K00208
Glutaryl-[acp] methyl ester
Pimeloyl-[acp] methyl ester
K02170...
BioH
Long-chain-acyl-[acp]
K16593
BioI
Pimelate
K01906
BioW
Pimeloyl-[acp]
Pimeloyl-CoA
K00652
K00652
8-Amino-7-oxononanoate
K00833...
K19563
7,8-Diamino-nonanoate
K01935...
Dethiobiotin
bio-1
Biotin
Biotin sulfoxide
Biotin sulfone
K08351
L-Lysine
Lysine degradation
Tropane, piperidine and pyridine alkaloid biosynthesis
K01435
NCU05516
NCU05516
NCU05516
NCU05516
6.2.1.11
N6-D-Biotinyl-L-lysine (Biocytin)
3.4.-.-
Biotinyl-5'-AMP
6.2.1.11
Biotinyl-CoA
Tetranorbiotin
Bisnorbiotin
Urea
NCU05516
NCU05516
NCU05516
NCU05516
Fatty acid biosynthesis
Holo-[carboxylase]
00780 7/19/17
(c) Kanehisa Laboratories

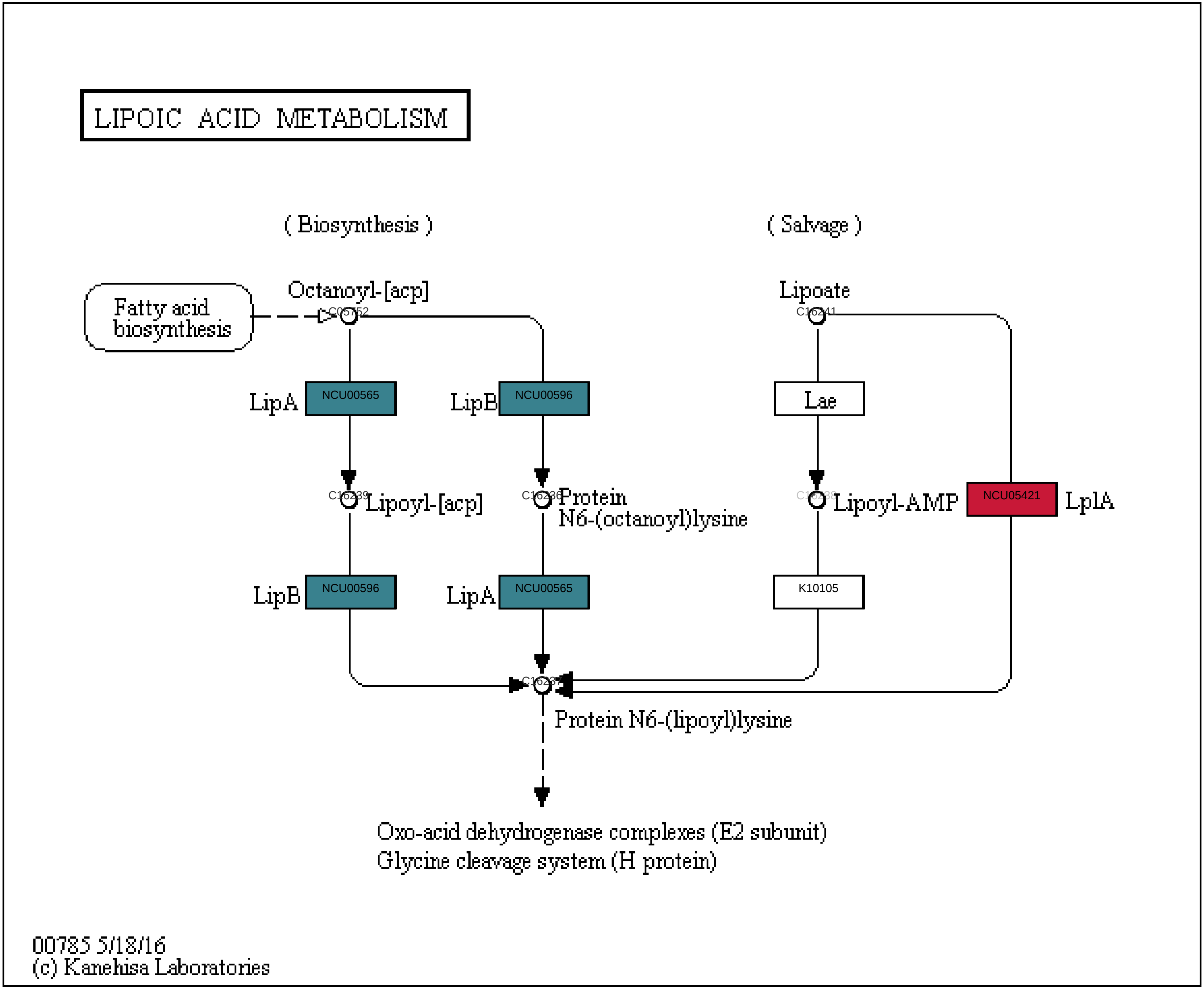
LIPOIC ACID METABOLISM
( Biosynthesis )
( Salvage )
Fatty acid biosynthesis
Octanoyl-[acp]
C05752
Lipoate
C16241
LipA
NCU00565
LipB
NCU00596
Lae
C16239
Lipoyl-[acp]
C16236
Protein N6-(octanoyl)lysine
Lipoyl-AMP
NCU05421
LplA
LipB
NCU00596
LipA
NCU00565
K10105
C16237
Protein N6-(lipoyl)lysine
Oxo-acid dehydrogenase complexes (E2 subunit)
Glycine cleavage system (H protein)
00785 5/18/16
(c) Kanehisa Laboratories

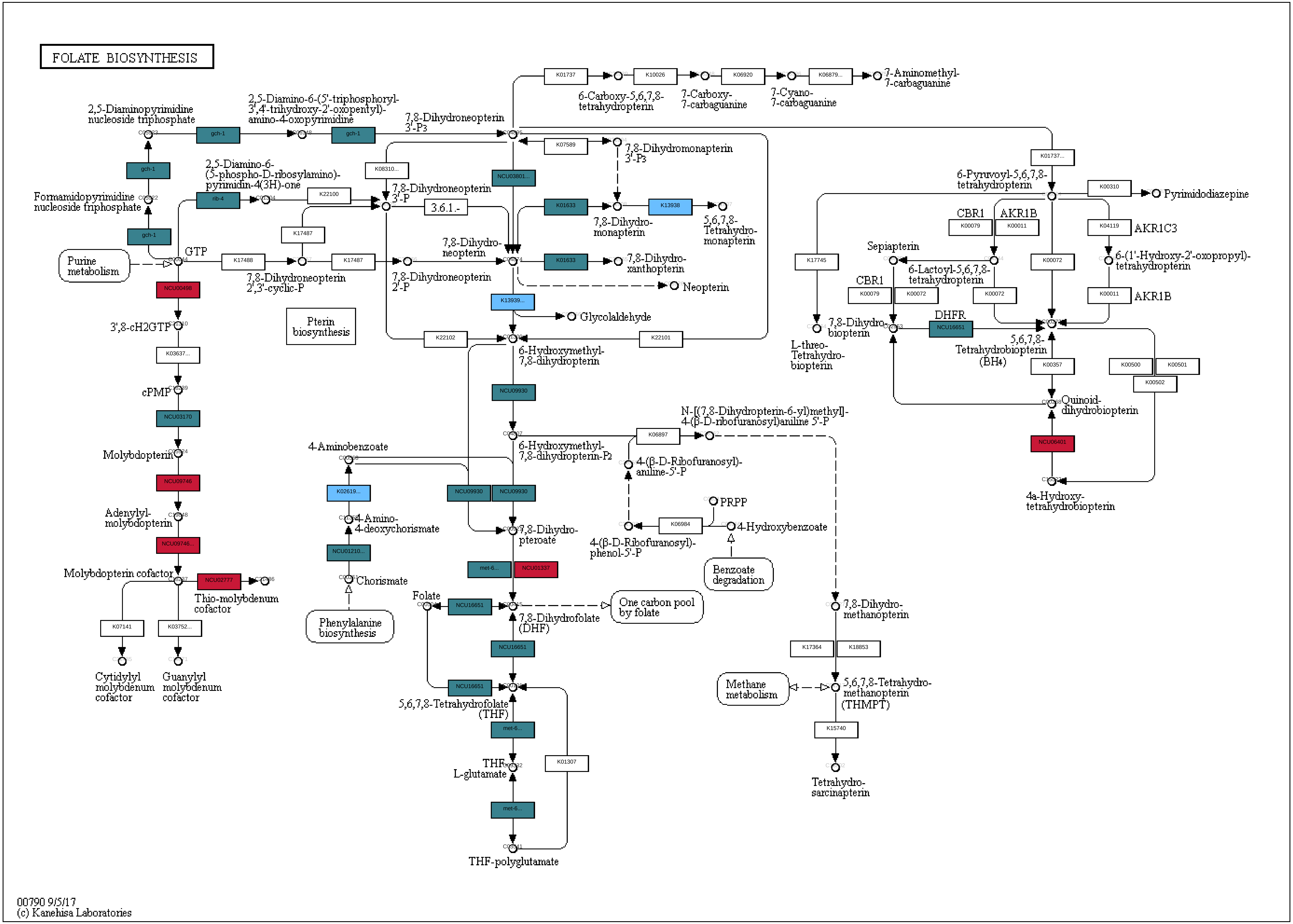
FOLATE BIOSYNTHESIS
2,5-Diaminopyrimidine nucleoside triphosphate
2,5-Diamino-6-(5'-triphosphoryl-3',4'-trihydroxy-2'-oxopentyl)-amino-4-oxopyrimidine
7,8-Dihydroneopterin 3'-P3
Formamidopyrimidine nucleoside triphosphate
2,5-Diamino-6-(5'-phospho-D-ribosylamino)-pyrimidin-4(3H)-one
7,8-Dihydroneopterin 3'-P
3.6.1.-
GTP
Purine metabolism
7,8-Dihydroneopterin 2',3'-cyclic-P
7,8-Dihydroneopterin 2'-P
7,8-Dihydro-neopterin
3',8-cH2GTP
cPMP
Molybdopterin
Adenylyl-molybdopterin
Molybdopterin cofactor
Thio-molybdenum cofactor
Cytidylyl molybdenum cofactor
Guanylyl molybdenum cofactor
Pterin biosynthesis
7,8-Dihydromonapterin 3'-P3
7,8-Dihydro-monapterin
5,6,7,8-Tetrahydro-monapterin
7,8-Dihydro-xanthopterin
Neopterin
Glycolaldehyde
6-Hydroxymethyl-7,8-dihydropterin
6-Hydroxymethyl-7,8-dihydropterin-P2
4-Aminobenzoate
4-Amino-4-deoxychorismate
Chorismate
Phenylalanine biosynthesis
7,8-Dihydro-pteroate
Folate
7,8-Dihydrofolate (DHF)
One carbon pool by folate
5,6,7,8-Tetrahydrofolate (THF)
THF-L-glutamate
THF-polyglutamate
6-Carboxy-5,6,7,8-tetrahydropterin
7-Carboxy-7-carbaguanine
7-Cyano-7-carbaguanine
7-Aminomethyl-7-carbaguanine
6-Pyruvoyl-5,6,7,8-tetrahydropterin
Pyrimidodiazepine
CBR1
AKR1B
AKR1C3
Sepiapterin
6-Lactoyl-5,6,7,8-tetrahydropterin
6-(1'-Hydroxy-2'-oxopropyl)-tetrahydropterin
CBR1
AKR1B
DHFR
7,8-Dihydro-biopterin
L-threo-Tetrahydro-biopterin
5,6,7,8-Tetrahydrobiopterin (BH4)
Quinoid-dihydrobiopterin
4a-Hydroxy-tetrahydrobiopterin
N-[(7,8-Dihydropterin-6-yl)methyl]-4-(β-D-ribofuranosyl)aniline 5'-P
4-(β-D-Ribofuranosyl)-aniline-5'-P
PRPP
4-Hydroxybenzoate
4-(β-D-Ribofuranosyl)-phenol-5'-P
Benzoate degradation
7,8-Dihydro-methanopterin
5,6,7,8-Tetrahydro-methanopterin (THMPT)
Methane metabolism
Tetrahydro-sarcinapterin
gch-1
rib-4
K22100
K08310...
K17487
K17488
K22102
K22101
NCU03801...
K07589
K01633
K13938
K13939...
NCU09930
NCU00498
K03637...
NCU03170
NCU09746
NCU09746...
NCU02777
K07141
K03752...
K02619...
NCU01210...
met-6...
NCU01337
NCU16651
K01307
K06897
K06984
K01737
K10026
K06920
K06879...
K01737...
K00310
K00079
K00011
K04119
K17745
K00072
K00357
K00500
K00501
K00502
NCU06401
K17364
K18853
K15740
00790 9/5/17
(c) Kanehisa Laboratories

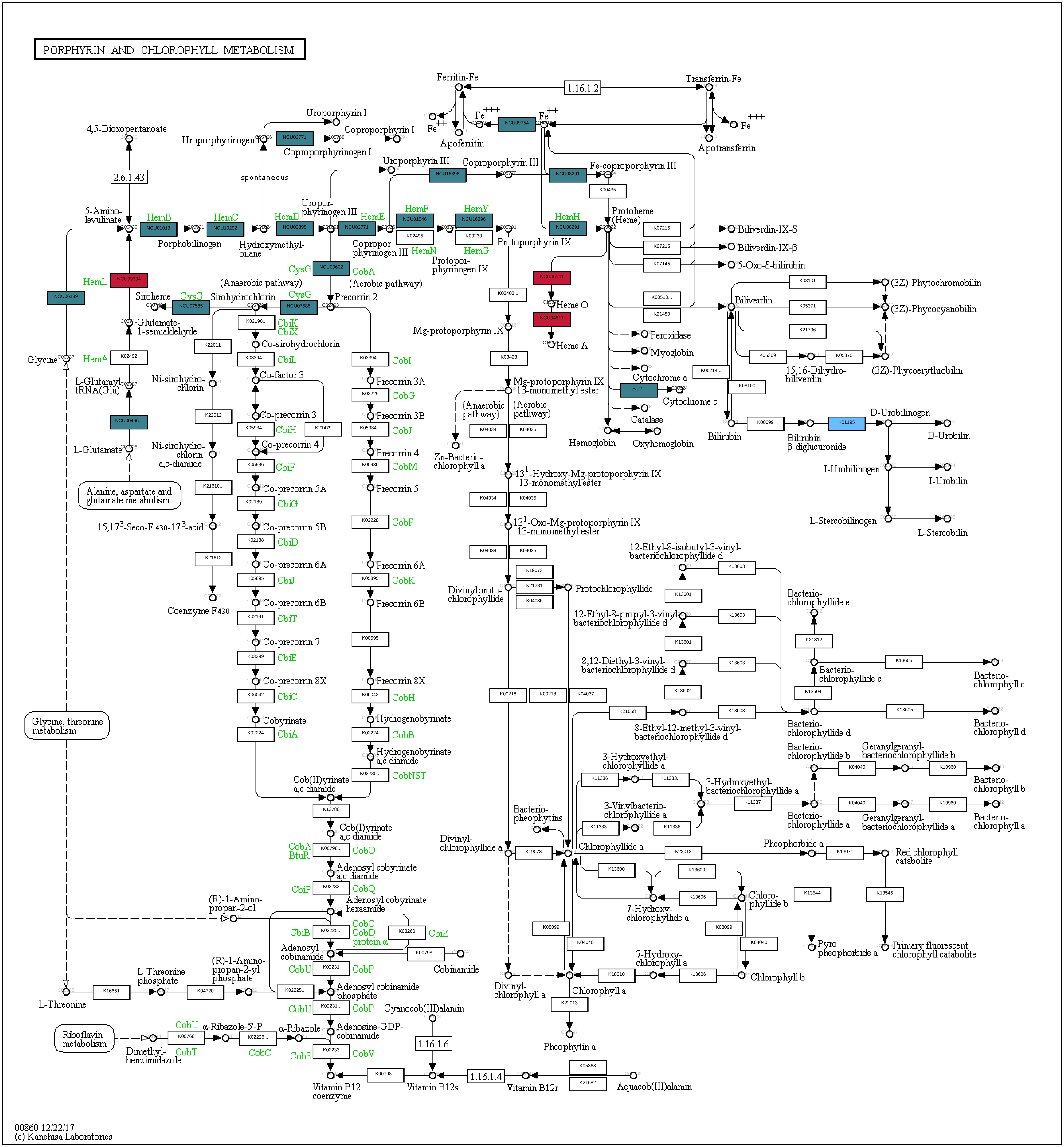

PORPHYRIN AND CHLOROPHYLL METABOLISM
Ferritin-Fe
1.16.1.2
Transferrin-Fe
Fe++
Fe+++
Fe++
Fe+++
Apoferritin
Apotransferrin
NCU09754
Uroporphyrin I
Coproporphyrin I
Uroporphyrinogen I
NCU02771
Coproporphyrinogen I
4,5-Dioxopentanoate
2.6.1.43
spontaneous
Uroporphyrin III
Coproporphyrin III
NCU16396
NCU08291
Fe-coproporphyrin III
K00435
5-Amino-levulinate
HemB
NCU01013
Porphobilinogen
HemC
NCU10292
Hydroxymethyl-bilane
HemD
NCU02395
Uropor-phyrinogen III
HemE
NCU02771
Copropor-phyrinogen III
HemF
NCU01546
K02495
HemN
HemY
NCU16396
K00230
Protopor-phyrinogen IX
HemG
Protoporphyrin IX
HemH
NCU08291
Protoheme (Heme)
K07215
Biliverdin-IX-δ
K07215
Biliverdin-IX-β
K07145
5-Oxo-δ-bilirubin
K08101
(3Z)-Phytochromobilin
K00510...
Biliverdin
K05371
(3Z)-Phycocyanobilin
K21480
K21796
NCU06141
Heme O
NCU04817
Heme A
Peroxidase
Myoglobin
K00214...
Cytochrome a
cyt-2...
Cytochrome c
Catalase
Hemoglobin
Oxyhemoglobin
K05369
15,16-Dihydro-biliverdin
K05370
(3Z)-Phycoerythrobilin
K08100
Bilirubin
K00699
Bilirubin β-diglucuronide
K01195
D-Urobilinogen
D-Urobilin
I-Urobilinogen
I-Urobilin
L-Stercobilinogen
L-Stercobilin
HemL
NCU09304
NCU06189
CysG
NCU00602
CobA
(Anaerobic pathway)
(Aerobic pathway)
Siroheme
CysG
NCU07585
Sirohydrochlorin
CysG
NCU07585
Precorrin 2
Glutamate-1-semialdehyde
HemA
K02492
Glycine
L-Glutamyl-tRNA(Glu)
NCU00466...
L-Glutamate
Alanine, aspartate and glutamate metabolism
K22011
Ni-sirohydro-chlorin
K22012
Ni-sirohydro-chlorin a,c-diamide
K21610...
15,17³-Seco-F 430-17³-acid
K21612
Coenzyme F430
K02190...
CbiK
CbiX
Co-sirohydrochlorin
K03394...
CbiL
Co-factor 3
Co-precorrin 3
K05934...
CbiH
K21479
Co-precorrin 4
K05936
CbiF
Co-precorrin 5A
K02189...
CbiG
Co-precorrin 5B
K02188
CbiD
Co-precorrin 6A
K05895
CbiJ
Co-precorrin 6B
K02191
CbiT
Co-precorrin 7
K03399
CbiE
Co-precorrin 8X
K06042
CbiC
Cobyrinate
K02224
CbiA
K03394...
CobI
Precorrin 3A
K02229
CobG
Precorrin 3B
K05934...
CobJ
Precorrin 4
K05936
CobM
Precorrin 5
K02228
CobF
Precorrin 6A
K05895
CobK
Precorrin 6B
K00595
Precorrin 8X
K06042
CobH
Hydrogenobyrinate
K02224
CobB
Hydrogenobyrinate a,c diamide
K02230...
CobNST
Cob(II)yrinate a,c diamide
K13786
Cob(I)yrinate a,c diamide
CobA
BtuR
K00798...
CobO
Adenosyl cobyrinate a,c diamide
CbiP
K02232
CobQ
Adenosyl cobyrinate hexaamide
(R)-1-Amino-propan-2-ol
CbiB
K02225...
CobC
CobD
protein α
K08260
CbiZ
Adenosyl cobinamide
K00798...
Cobinamide
CobU
K02231
CobP
Glycine, threonine metabolism
L-Threonine phosphate
(R)-1-Amino-propan-2-yl phosphate
K16651
K04720
K02225...
Adenosyl cobinamide phosphate
L-Threonine
CobU
K02231...
CobP
Cyanocob(III)alamin
Riboflavin metabolism
CobU
α-Ribazole-5'-P
α-Ribazole
Adenosine-GDP-cobinamide
K00768
K02226...
Dimethyl-benzimidazole
CobT
CobC
CobS
K02233
CobV
1.16.1.6
Vitamin B12 coenzyme
K00798...
Vitamin B12s
1.16.1.4
Vitamin B12r
K05368
K21682
Aquacob(III)alamin
K03403...
Mg-protoporphyrin IX
K03428
Mg-protoporphyrin IX 13-monomethyl ester
(Anaerobic pathway)
(Aerobic pathway)
K04034
K04035
Zn-Bacterio-chlorophyll a
13¹-Hydroxy-Mg-protoporphyrin IX 13-monomethyl ester
K04034
K04035
13¹-Oxo-Mg-protoporphyrin IX 13-monomethyl ester
K04034
K04035
K19073
K21231
K04036
Divinylproto-chlorophyllide
Protochlorophyllide
K00218
K00218
K04037...
12-Ethyl-8-isobutyl-3-vinyl-bacteriochlorophyllide d
K13603
K13601
12-Ethyl-8-propyl-3-vinyl-bacteriochlorophyllide d
K13603
K13601
8,12-Diethyl-3-vinyl-bacteriochlorophyllide d
K13603
K13602
K21058
K13603
8-Ethyl-12-methyl-3-vinyl-bacteriochlorophyllide d
Bacterio-chlorophyllide e
K21312
Bacterio-chlorophyllide c
K13605
Bacterio-chlorophyll c
K13604
Bacterio-chlorophyllide d
K13605
Bacterio-chlorophyll d
3-Hydroxyethyl-chlorophyllide a
K11336
K11333...
3-Hydroxyethyl-bacteriochlorophyllide a
K11337
3-Vinylbacterio-chlorophyllide a
K11333...
K11336
Bacterio-chlorophyllide b
Geranylgeranyl-bacteriochlorophyllide b
K04040
K10960
Bacterio-chlorophyll b
Bacterio-chlorophyllide a
Geranylgeranyl-bacteriochlorophyllide a
K04040
K10960
Bacterio-chlorophyll a
Bacterio-pheophytins
Divinyl-chlorophyllide a
K19073
Chlorophyllide a
K22013
Pheophorbide a
K13071
Red chlorophyll catabolite
K13600
K13600
K13606
Chloro-phyllide b
7-Hydroxy-chlorophyllide a
K13544
K13545
K08099
K04040
K08099
K04040
Pyro-pheophorbide a
Primary fluorescent chlorophyll catabolite
7-Hydroxy-chlorophyll a
K18010
K13606
Chlorophyll b
Divinyl-chlorophyll a
Chlorophyll a
K22013
Pheophytin a
00860 12/22/17
(c) Kanehisa Laboratories

# 9. Metabolism of terpenoids and polyketides

| MAP | PATHWAY |
|---|---|
| **900** | Terpenoid backbone biosynthesis |
| **909** | Sesquiterpenoid and triterpenoid biosynthesis |

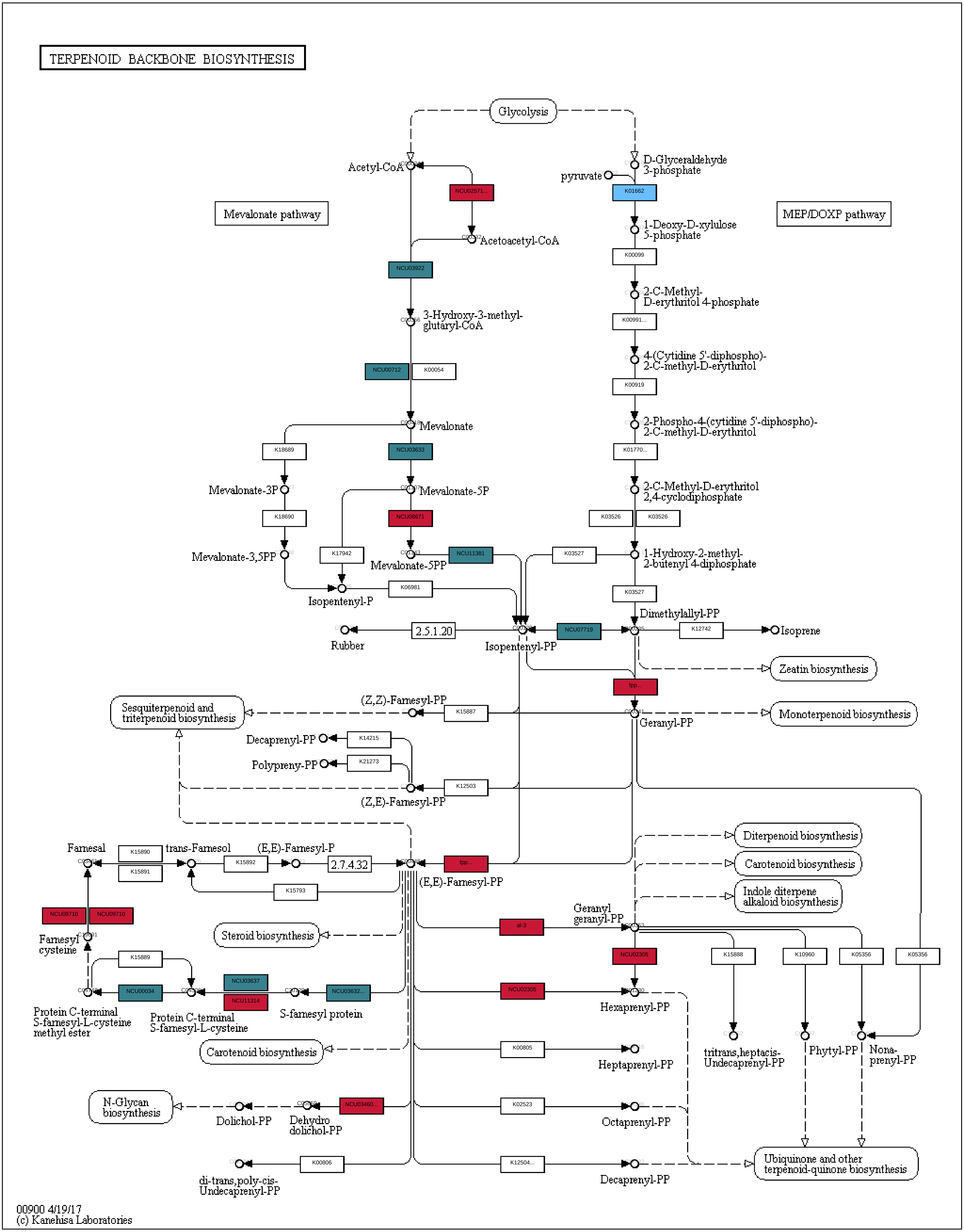

TERPENOID BACKBONE BIOSYNTHESIS
Glycolysis
Acetyl-CoA
D-Glyceraldehyde 3-phosphate
pyruvate
NCU02571...
K01662
Mevalonate pathway
MEP/DOXP pathway
Acetoacetyl-CoA
1-Deoxy-D-xylulose 5-phosphate
NCU03922
K00099
2-C-Methyl-D-erythritol 4-phosphate
3-Hydroxy-3-methyl-glutaryl-CoA
K00991...
NCU00712
K00054
4-(Cytidine 5'-diphospho)-2-C-methyl-D-erythritol
K00919
Mevalonate
2-Phospho-4-(cytidine 5'-diphospho)-2-C-methyl-D-erythritol
K18689
NCU03633
K01770...
Mevalonate-3P
Mevalonate-5P
2-C-Methyl-D-erythritol 2,4-cyclodiphosphate
K18690
NCU08871
K03526
K03526
K17942
Mevalonate-3,5PP
NCU11381
K03527
1-Hydroxy-2-methyl-2-butenyl 4-diphosphate
Mevalonate-5PP
K06981
Isopentenyl-P
K03527
Dimethylallyl-PP
2.5.1.20
NCU07719
K12742
Isoprene
Rubber
Isopentenyl-PP
Zeatin biosynthesis
fpp...
(Z,Z)-Farnesyl-PP
Sesquiterpenoid and triterpenoid biosynthesis
K15887
Monoterpenoid biosynthesis
Geranyl-PP
Decaprenyl-PP
K14215
Polypreny-PP
K21273
K12503
(Z,E)-Farnesyl-PP
Diterpenoid biosynthesis
Farnesal
K15890
trans-Farnesol
(E,E)-Farnesyl-P
K15891
K15892
2.7.4.32
fpp...
(E,E)-Farnesyl-PP
Carotenoid biosynthesis
K15793
Indole diterpene alkaloid biosynthesis
NCU09710
NCU09710
Geranyl geranyl-PP
al-3
Farnesyl cysteine
Steroid biosynthesis
K15889
NCU02305
K15888
K10960
K05356
K05356
NCU03637
NCU00034
NCU11314
NCU03632...
NCU02305
Hexaprenyl-PP
Protein C-terminal S-farnesyl-L-cysteine methyl ester
Protein C-terminal S-farnesyl-L-cysteine
S-farnesyl protein
Carotenoid biosynthesis
K00805
tritrans,heptacis-Undecaprenyl-PP
Phytyl-PP
Nona-prenyl-PP
Heptaprenyl-PP
N-Glycan biosynthesis
NCU03460...
K02523
Dolichol-PP
Dehydro dolichol-PP
Octaprenyl-PP
Ubiquinone and other terpenoid-quinone biosynthesis
K00806
K12504...
di-trans,poly-cis-Undecaprenyl-PP
Decaprenyl-PP
00900 4/19/17
(c) Kanehisa Laboratories

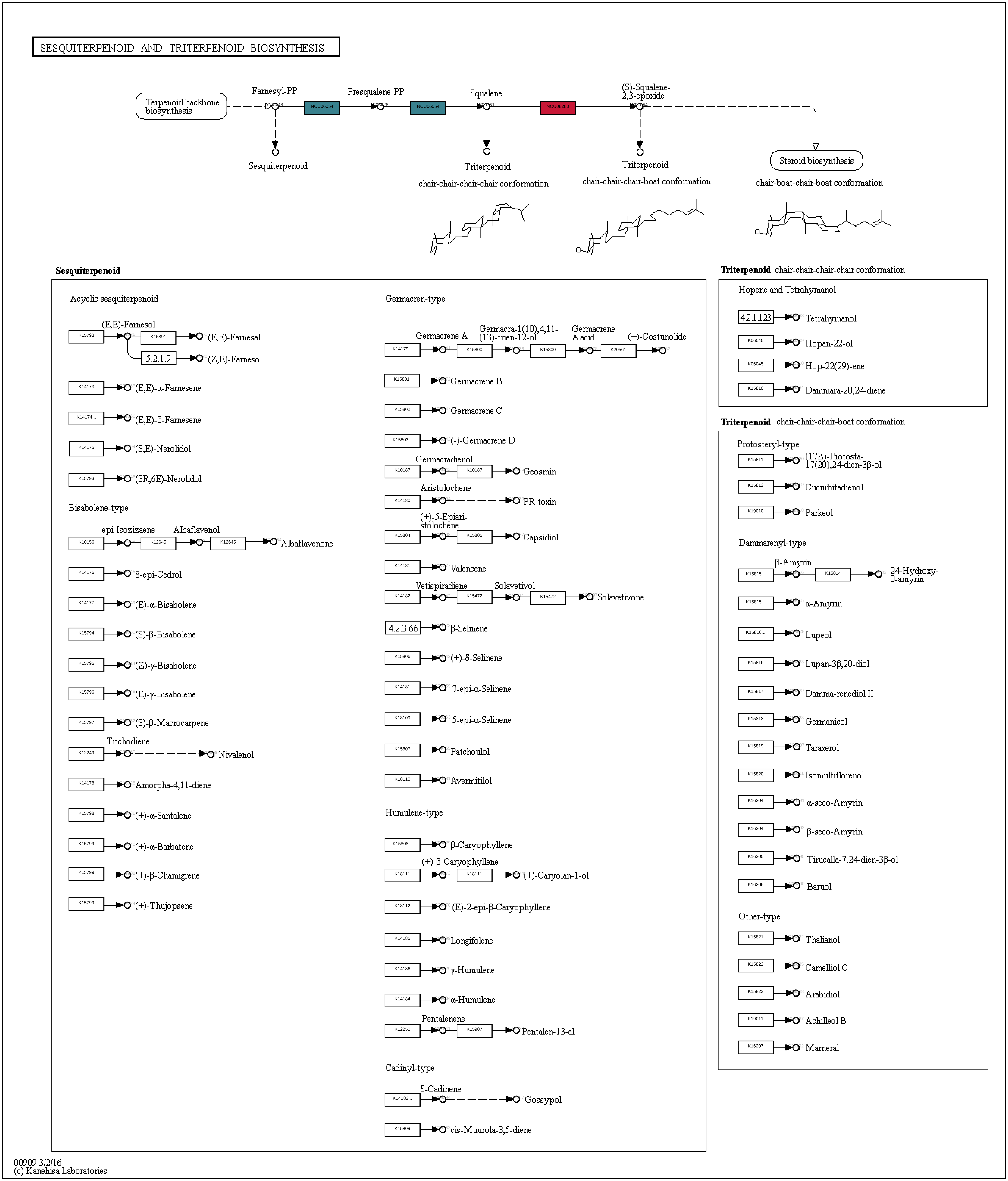
SESQUITERPENOID AND TRITERPENOID BIOSYNTHESIS
Terpenoid backbone biosynthesis
Farnesyl-PP
NCU06054
Presqualene-PP
NCU06054
Squalene
NCU08280
(S)-Squalene-2,3-epoxide
Sesquiterpenoid
Triterpenoid
chair-chair-chair-chair conformation
Triterpenoid
chair-chair-chair-boat conformation
Steroid biosynthesis
chair-boat-chair-boat conformation
Sesquiterpenoid
Acyclic sesquiterpenoid
(E,E)-Farnesol
K15793
K15891
(E,E)-Farnesal
5.2.1.9
(Z,E)-Farnesol
K14173
(E,E)-α-Farnesene
K14174...
(E,E)-β-Farnesene
K14175
(S,E)-Nerolidol
K15793
(3R,6E)-Nerolidol
Bisabolene-type
epi-Isozizaene
Albaflavenol
K10156
K12645
K12645
Albaflavenone
K14176
8-epi-Cedrol
K14177
(E)-α-Bisabolene
K15794
(S)-β-Bisabolene
K15795
(Z)-γ-Bisabolene
K15796
(E)-γ-Bisabolene
K15797
(S)-β-Macrocarpene
Trichodiene
K12249
Nivalenol
K14178
Amorpha-4,11-diene
K15798
(+)-α-Santalene
K15799
(+)-α-Barbatene
K15799
(+)-β-Chamigrene
K15799
(+)-Thujopsene
Germacren-type
Germacrene A
Germacra-1(10),4,11-(13)-trien-12-ol
Germacrene A acid
(+)-Costunolide
K14179...
K15800
K15800
K20561
K15801
Germacrene B
K15802
Germacrene C
K15803...
(-)-Germacrene D
Germacradienol
K10187
K10187
Geosmin
Aristolochene
K14180
PR-toxin
(+)-5-Epiaristolochene
K15804
K15805
Capsidiol
K14181
Valencene
Vetispiradiene
Solavetivol
K14182
K15472
K15472
Solavetivone
4.2.3.66
β-Selinene
K15806
(+)-δ-Selinene
K14181
7-epi-α-Selinene
K18109
5-epi-α-Selinene
K15807
Patchoulol
K18110
Avermitilol
Humulene-type
K15808...
β-Caryophyllene
(+)-β-Caryophyllene
K18111
K18111
(+)-Caryolan-1-ol
K18112
(E)-2-epi-β-Caryophyllene
K14185
Longifolene
K14186
γ-Humulene
K14184
α-Humulene
Pentalenene
K12250
K15907
Pentalen-13-al
Cadinyl-type
δ-Cadinene
K14183...
Gossypol
K15809
cis-Muurola-3,5-diene
Triterpenoid chair-chair-chair-chair conformation
Hopene and Tetrahymanol
4.2.1.123
Tetrahymanol
K06045
Hopan-22-ol
K06045
Hop-22(29)-ene
K15810
Dammara-20,24-diene
Triterpenoid chair-chair-chair-boat conformation
Protosteryl-type
K15811
(17Z)-Protosta-17(20),24-dien-3β-ol
K15812
Cucurbitadienol
K19010
Parkeol
Dammarenyl-type
β-Amyrin
K15815...
K15814
24-Hydroxy-β-amyrin
K15815...
α-Amyrin
K15816...
Lupeol
K15816
Lupan-3β,20-diol
K15817
Damma-renediol II
K15818
Germanicol
K15819
Taraxerol
K15820
Isomultiflorenol
K16204
α-seco-Amyrin
K16204
β-seco-Amyrin
K16205
Tirucalla-7,24-dien-3β-ol
K16206
Baruol
Other-type
K15821
Thalianol
K15822
Camelliol C
K15823
Arabidiol
K19011
Achilleol B
K16207
Marneral
00909 3/2/16
(c) Kanehisa Laboratories

# 10. Biosynthesis of other secondary metabolites

| MAP | PATHWAY |
|---|---|
| **261** | Monobactam biosynthesis |
| **332** | Carbapenem biosynthesis |

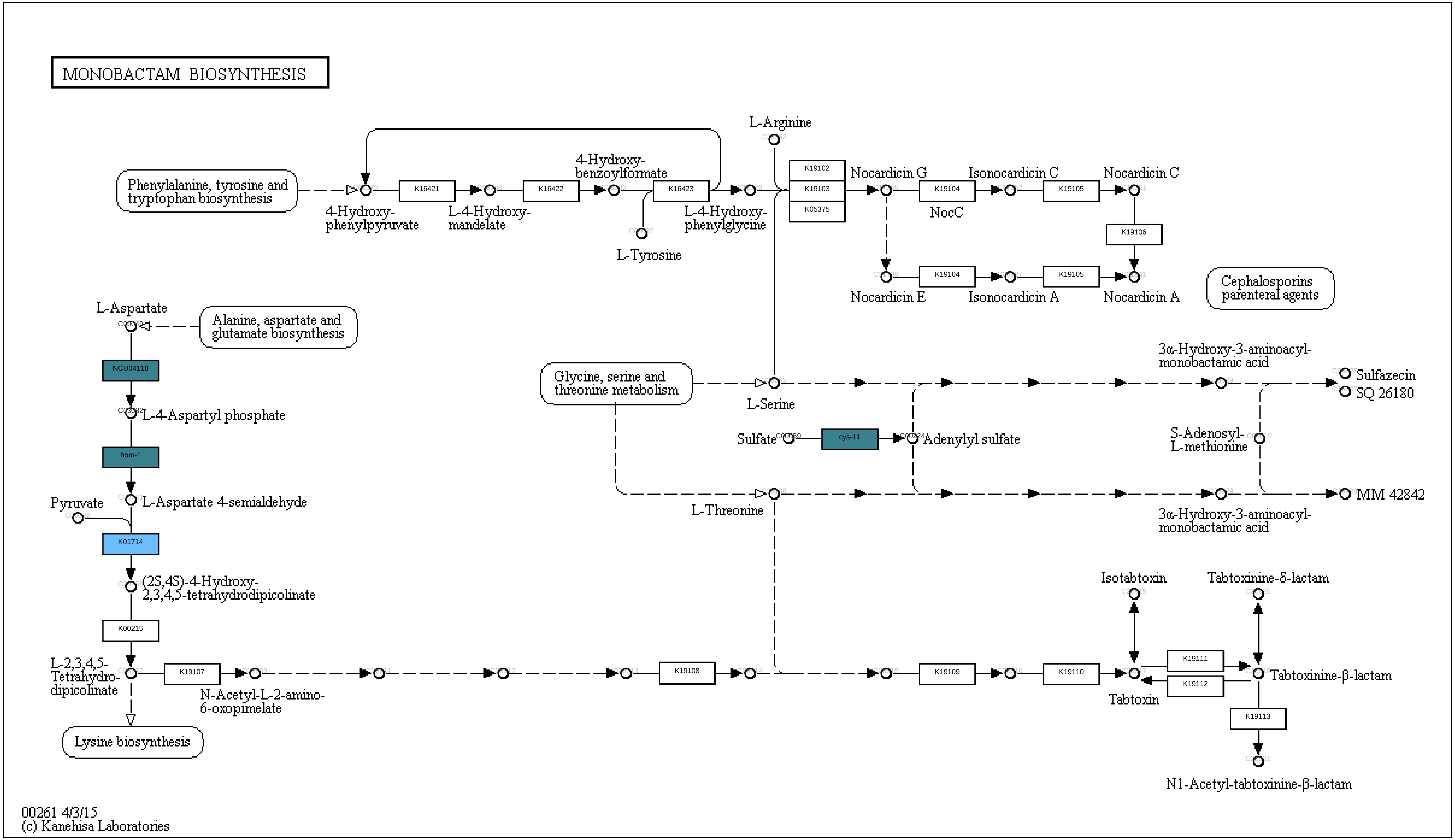
MONOBACTAM BIOSYNTHESIS
Phenylalanine, tyrosine and tryptophan biosynthesis
4-Hydroxy-phenylpyruvate
K16421
L-4-Hydroxy-mandelate
K16422
4-Hydroxy-benzoylformate
K16423
L-Tyrosine
L-4-Hydroxy-phenylglycine
L-Arginine
K19102
K19103
K05375
Nocardicin G
K19104
NocC
Isonocardicin C
K19105
Nocardicin C
K19106
Nocardicin E
K19104
Isonocardicin A
K19105
Nocardicin A
Cephalosporins parenteral agents
L-Aspartate
C00049
Alanine, aspartate and glutamate biosynthesis
NCU04118
C03082
L-4-Aspartyl phosphate
hom-1
L-Aspartate 4-semialdehyde
Pyruvate
K01714
(2S,4S)-4-Hydroxy-2,3,4,5-tetrahydrodipicolinate
K00215
L-2,3,4,5-Tetrahydro-dipicolinate
K19107
N-Acetyl-L-2-amino-6-oxopimelate
Lysine biosynthesis
Glycine, serine and threonine metabolism
L-Serine
Sulfate
C00059
cys-11
C00224
Adenylyl sulfate
L-Threonine
3α-Hydroxy-3-aminoacyl-monobactamic acid
Sulfazecin
SQ 26180
S-Adenosyl-L-methionine
MM 42842
3α-Hydroxy-3-aminoacyl-monobactamic acid
K19108
K19109
K19110
Isotabtoxin
Tabtoxinine-δ-lactam
K19111
K19112
Tabtoxin
Tabtoxinine-β-lactam
K19113
N1-Acetyl-tabtoxinine-β-lactam
00261 4/3/15
(c) Kanehisa Laboratories

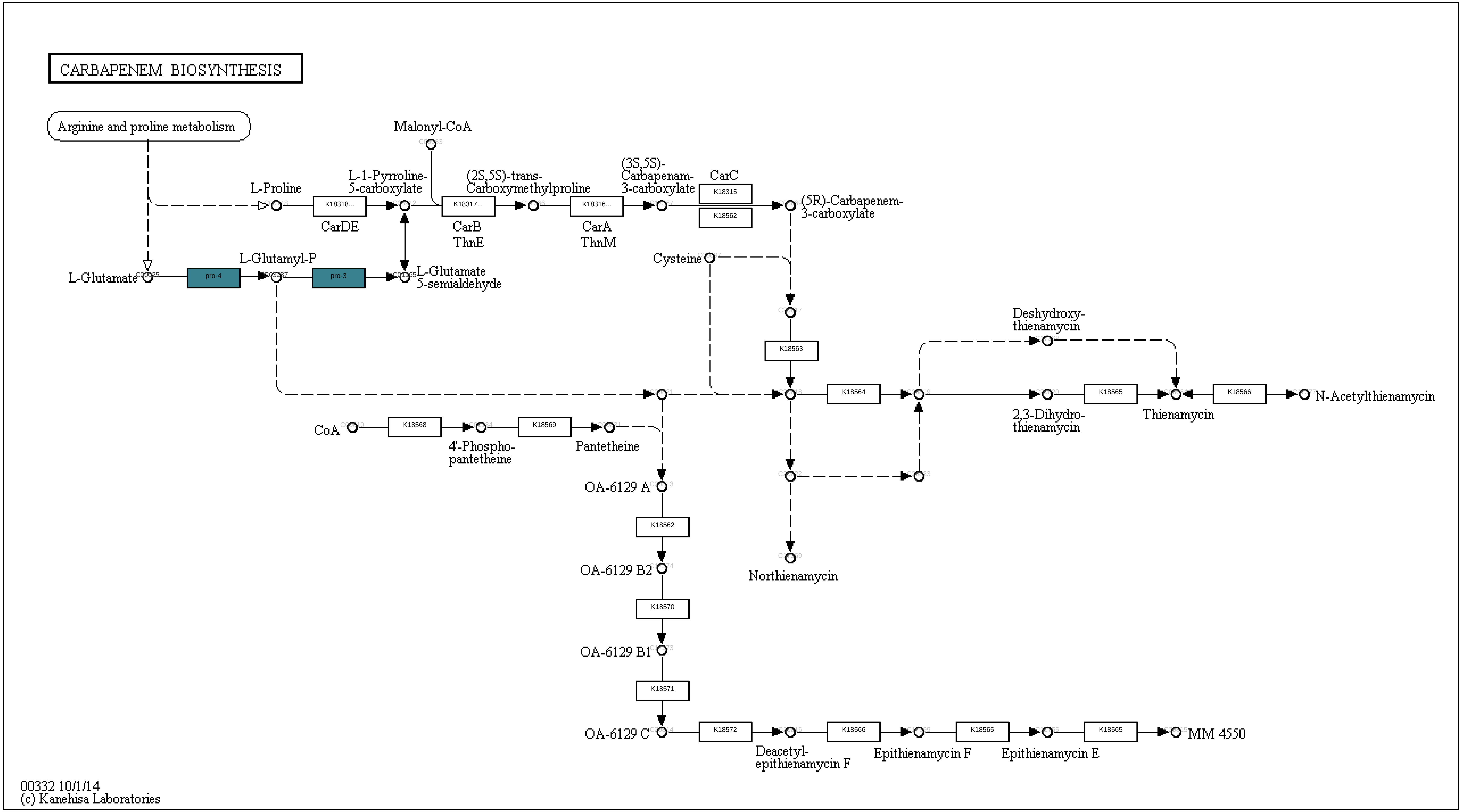
CARBAPENEM BIOSYNTHESIS
Arginine and proline metabolism
Malonyl-CoA
L-Proline
L-1-Pyrroline-5-carboxylate
(2S,5S)-trans-Carboxymethylproline
(3S,5S)-Carbapenam-3-carboxylate
CarC
K18315
K18562
(5R)-Carbapenem-3-carboxylate
K18318...
CarDE
K18317...
CarB
ThnE
K18316...
CarA
ThnM
L-Glutamyl-P
L-Glutamate
pro-4
pro-3
L-Glutamate 5-semialdehyde
Cysteine
K18563
Deshydroxy-thienamycin
K18564
K18565
K18566
N-Acetylthienamycin
2,3-Dihydro-thienamycin
Thienamycin
CoA
K18568
4'-Phospho-pantetheine
K18569
Pantetheine
OA-6129 A
K18562
OA-6129 B2
K18570
OA-6129 B1
K18571
OA-6129 C
K18572
Deacetyl-epithienamycin F
K18566
Epithienamycin F
K18565
Epithienamycin E
K18565
MM 4550
Northienamycin
00332 10/1/14
(c) Kanehisa Laboratories

# 11. Xenobiotics biodegradation and metabolism

| MAP | PATHWAY |
|---|---|
| **791** | Atrazine degradation |

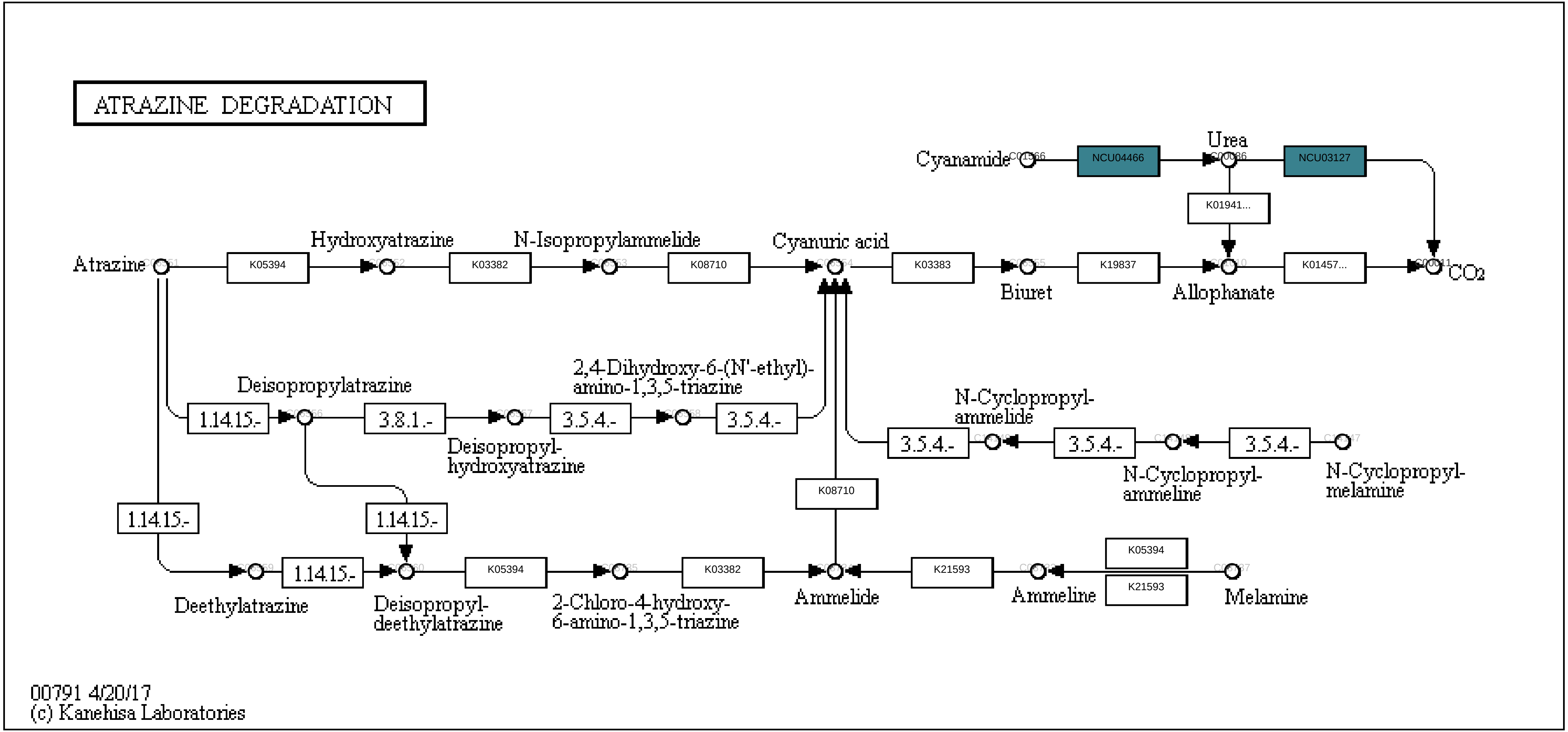
ATRAZINE DEGRADATION
Cyanamide
NCU04466
Urea
NCU03127
K01941...
Atrazine
K05394
Hydroxyatrazine
K03382
N-Isopropylammelide
K08710
Cyanuric acid
K03383
Biuret
K19837
Allophanate
K01457...
CO2
Deisopropylatrazine
1.14.15.-
3.8.1.-
2,4-Dihydroxy-6-(N'-ethyl)-amino-1,3,5-triazine
3.5.4.-
3.5.4.-
Deisopropyl-hydroxyatrazine
N-Cyclopropyl-ammelide
3.5.4.-
3.5.4.-
3.5.4.-
N-Cyclopropyl-ammeline
N-Cyclopropyl-melamine
K08710
1.14.15.-
1.14.15.-
1.14.15.-
Deethylatrazine
Deisopropyl-deethylatrazine
K05394
2-Chloro-4-hydroxy-6-amino-1,3,5-triazine
K03382
Ammelide
K21593
Ammeline
K05394
K21593
Melamine
00791 4/20/17
(c) Kanehisa Laboratories

**KEGG pathway analysis**

# *Positive controls*

**Supplementary file 4**

**The following color codes are used:**

True positive (TP): element in the reference pathway correctly predicted by Interproscan

True negative (TN): element in the *not* reference pathway and *not* predicted by Interproscan

False negative (FN): element in the reference pathway but *not* predicted by Interproscan.

False positive (FP): element in the *not* reference pathway but predicted by Interproscan. Note: could also be a valid new prediction

**Species:** ***Cryptococcus neoformans***

**Acronym:** **CNE**

**Lifestyle:** **Culturable**

# 1. Carbohydrate metabolism

| MAP | PATHWAY |
|---|---|
| **10** | Glycolysis / Gluconeogenesis |
| **20** | Citrate cycle (TCA cycle) |
| **30** | Pentose phosphate pathway |
| **40** | Pentose and glucuronate interconversions |
| **51** | Fructose and mannose metabolism |
| **52** | Galactose metabolism |
| **53** | Ascorbate and aldarate metabolism |
| **500** | Starch and sucrose metabolism |
| **520** | Amino sugar and nucleotide sugar metabolism |
| **562** | Inositol phosphate metabolism |
| **620** | Pyruvate metabolism |
| **630** | Glyoxylate and dicarboxylate metabolism |
| **640** | Propanoate metabolism |
| **650** | Butanoate metabolism |
| **660** | C5-Branched dibasic acid metabolism |

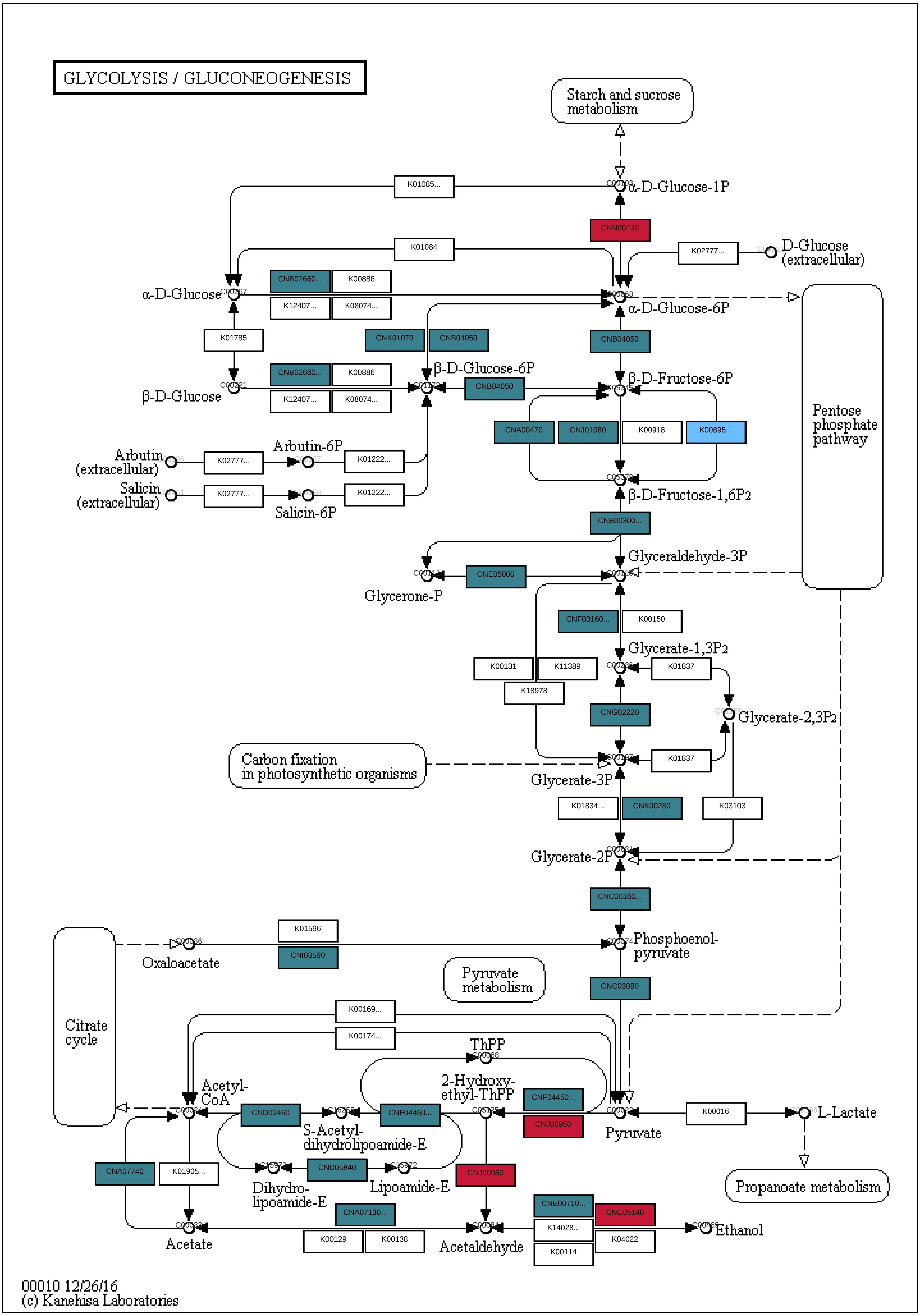
GLYCOLYSIS / GLUCONEOGENESIS
Starch and sucrose metabolism
α-D-Glucose-1P
CNN00430
K01085...
K01084
K02777...
D-Glucose (extracellular)
CNB02660...
K00886
K12407...
K08074...
α-D-Glucose
α-D-Glucose-6P
K01785
CNK01070
CNB04050
β-D-Glucose
β-D-Glucose-6P
β-D-Fructose-6P
CNA00470
CNJ01080
K00918
K00895...
Arbutin (extracellular)
Arbutin-6P
K01222...
Salicin (extracellular)
Salicin-6P
β-D-Fructose-1,6P2
CNB00300...
Pentose phosphate pathway
Glyceraldehyde-3P
CNE05000
Glycerone-P
CNF03160...
K00150
Glycerate-1,3P2
K00131
K11389
K18978
K01837
CNG02220
Glycerate-2,3P2
Carbon fixation in photosynthetic organisms
Glycerate-3P
K01834...
CNK00280
K03103
Glycerate-2P
CNC00160...
K01596
CNI03590
Oxaloacetate
Phosphoenol-pyruvate
Citrate cycle
Pyruvate metabolism
CNC03080
K00169...
K00174...
ThPP
2-Hydroxy-ethyl-ThPP
Acetyl-CoA
CND02450
S-Acetyl-dihydrolipoamide-E
CNF04450...
CNJ00950
Pyruvate
K00016
L-Lactate
Propanoate metabolism
CNA07740
K01905...
CND05840
Dihydro-lipoamide-E
Lipoamide-E
CNA07130...
K00129
K00138
Acetate
Acetaldehyde
CNE00710...
CNC05140
K14028...
K04022
K00114
Ethanol
00010 12/26/16
(c) Kanehisa Laboratories

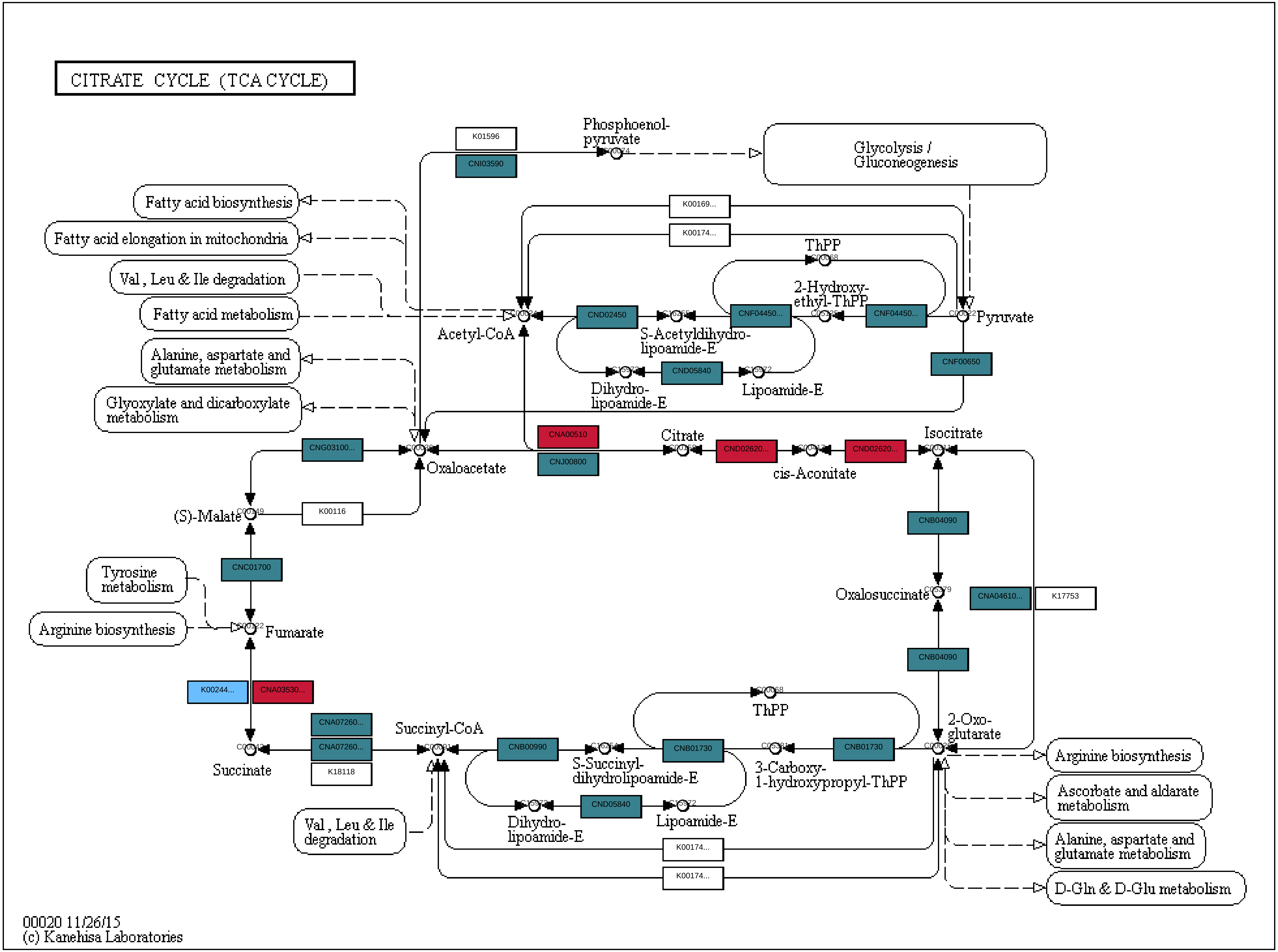
CITRATE CYCLE (TCA CYCLE)
Phosphoenol-pyruvate
Glycolysis / Gluconeogenesis
K01596
CNI03590
Fatty acid biosynthesis
Fatty acid elongation in mitochondria
Val, Leu & Ile degradation
Fatty acid metabolism
Alanine, aspartate and glutamate metabolism
Glyoxylate and dicarboxylate metabolism
K00169...
K00174...
ThPP
2-Hydroxy-ethyl-ThPP
CND02450
CNF04450...
CNF04450...
Pyruvate
Acetyl-CoA
S-Acetyldihydro-lipoamide-E
CNF00650
CND05840
Dihydro-lipoamide-E
Lipoamide-E
CNA00510
CNJ00800
Citrate
CND02620...
CND02620...
Isocitrate
cis-Aconitate
CNG03100...
Oxaloacetate
(S)-Malate
K00116
CNB04090
CNC01700
Tyrosine metabolism
Arginine biosynthesis
Fumarate
Oxalosuccinate
CNA04610...
K17753
CNB04090
K00244...
CNA03530...
CNA07260...
CNA07260...
K18118
Succinate
Succinyl-CoA
CNB00990
ThPP
CNB01730
CNB01730
2-Oxo-glutarate
S-Succinyl-dihydrolipoamide-E
3-Carboxy-1-hydroxypropyl-ThPP
CND05840
Dihydro-lipoamide-E
Lipoamide-E
Val, Leu & Ile degradation
K00174...
K00174...
Arginine biosynthesis
Ascorbate and aldarate metabolism
Alanine, aspartate and glutamate metabolism
D-Gln & D-Glu metabolism
00020 11/26/15
(c) Kanehisa Laboratories

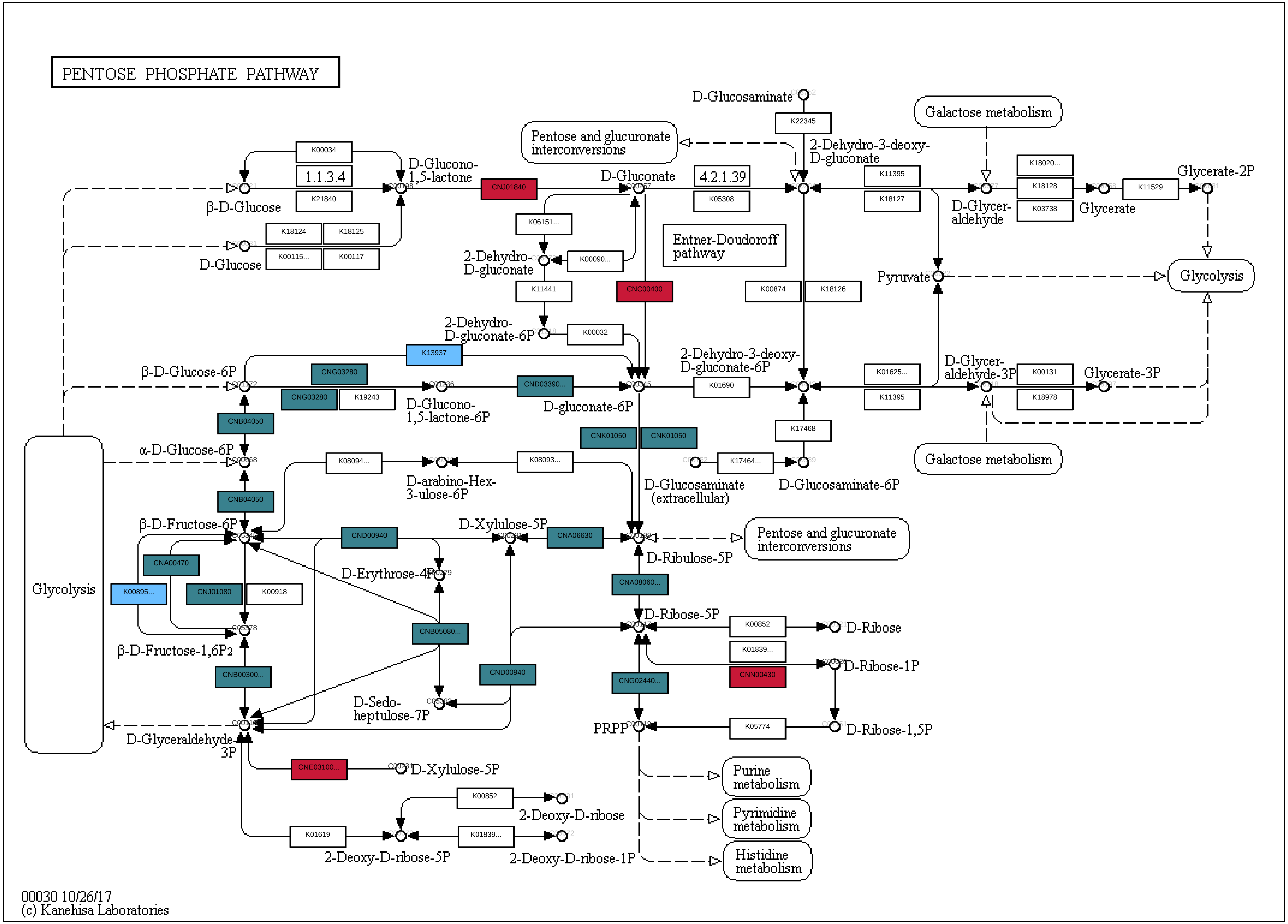
PENTOSE PHOSPHATE PATHWAY
D-Glucosaminate
K22345
Galactose metabolism
Pentose and glucuronate interconversions
2-Dehydro-3-deoxy-D-gluconate
K00034
D-Glucono-1,5-lactone
1.1.3.4
K21840
β-D-Glucose
CNJ01840
D-Gluconate
4.2.1.39
K05308
K11395
K18127
K18020...
K18128
K03738
D-Glyceraldehyde
Glycerate
K11529
Glycerate-2P
K18124
K18125
K00115...
K00117
D-Glucose
K06151...
2-Dehydro-D-gluconate
K00090...
K11441
CNC00400
Entner-Doudoroff pathway
K00874
K18126
Pyruvate
Glycolysis
2-Dehydro-D-gluconate-6P
K00032
K13937
CNG03280
β-D-Glucose-6P
K19243
D-Glucono-1,5-lactone-6P
CND03390...
D-gluconate-6P
2-Dehydro-3-deoxy-D-gluconate-6P
K01690
K01625...
K11395
D-Glyceraldehyde-3P
K00131
K18978
Glycerate-3P
CNB04050
α-D-Glucose-6P
CNK01050
K17468
K08094...
D-arabino-Hex-3-ulose-6P
K08093...
K17464...
D-Glucosaminate (extracellular)
D-Glucosaminate-6P
β-D-Fructose-6P
CND00940
D-Xylulose-5P
CNA06630
D-Ribulose-5P
CNA00470
K00895...
CNJ01080
K00918
D-Erythrose-4P
CNA08060...
D-Ribose-5P
K00852
D-Ribose
K01839...
CNN00430
D-Ribose-1P
CNB05080...
β-D-Fructose-1,6P2
CNB00300...
CND00940
CNG02440...
D-Sedo-heptulose-7P
PRPP
K05774
D-Ribose-1,5P
D-Glyceraldehyde-3P
CNE03100...
D-Xylulose-5P
Purine metabolism
K00852
2-Deoxy-D-ribose
Pyrimidine metabolism
K01619
K01839...
2-Deoxy-D-ribose-5P
2-Deoxy-D-ribose-1P
Histidine metabolism
00030 10/26/17
(c) Kanehisa Laboratories

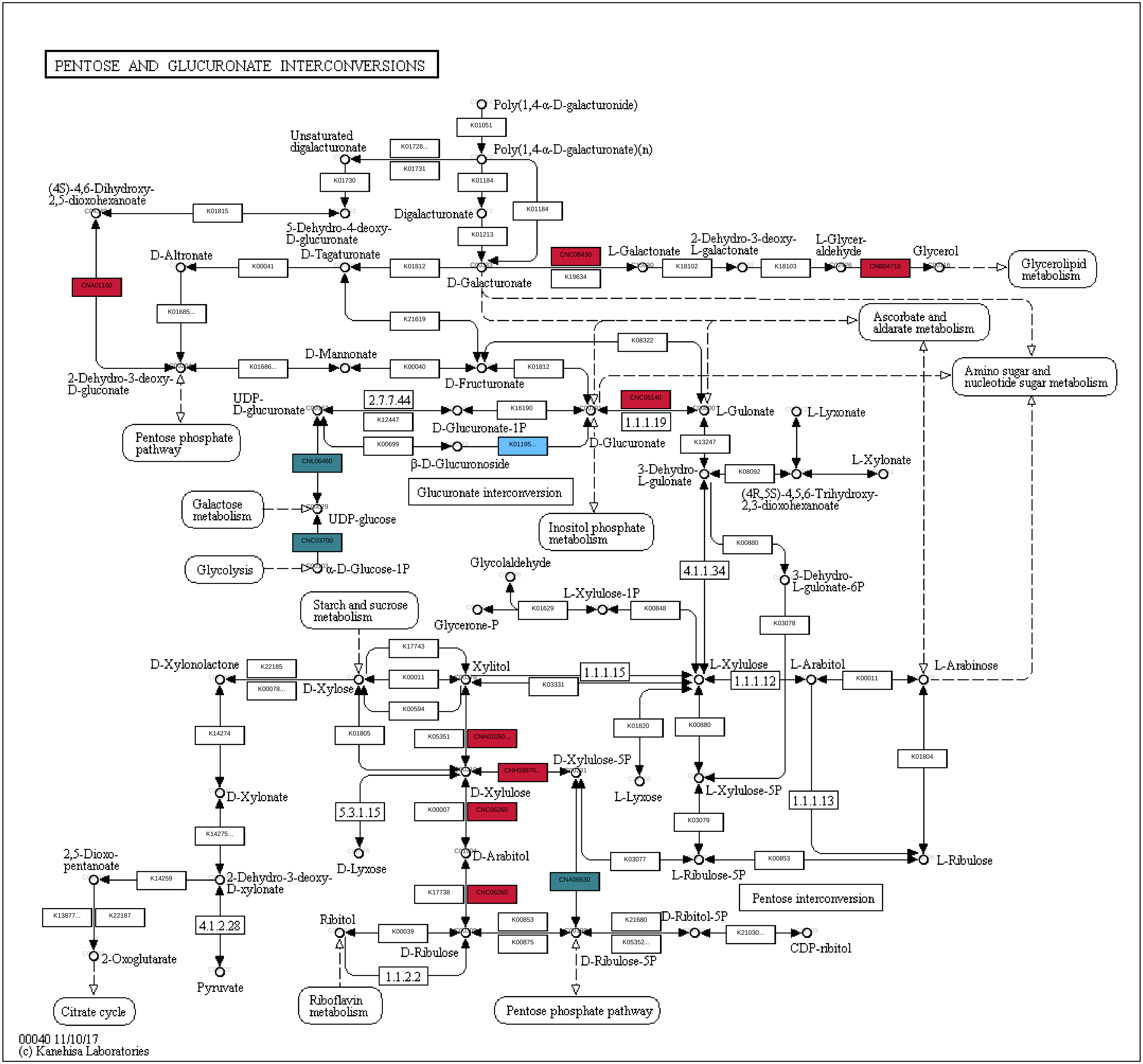
PENTOSE AND GLUCURONATE INTERCONVERSIONS
Poly(1,4-α-D-galacturonide)
K01051
Poly(1,4-α-D-galacturonate)(n)
Unsaturated digalacturonate
K01728...
K01731
K01730
K01184
K01184
Digalacturonate
K01213
(4S)-4,6-Dihydroxy-2,5-dioxohexanoate
K01815
5-Dehydro-4-deoxy-D-glucuronate
D-Tagaturonate
D-Altronate
K00041
K01812
CNC06430
K19634
L-Galactonate
K18102
2-Dehydro-3-deoxy-L-galactonate
K18103
L-Glycer-aldehyde
CNB04710
Glycerol
Glycerolipid metabolism
D-Galacturonate
CNA01160
K01685...
K21619
Ascorbate and aldarate metabolism
K08322
D-Mannonate
K01686...
K00040
K01812
2-Dehydro-3-deoxy-D-gluconate
D-Fructuronate
Amino sugar and nucleotide sugar metabolism
UDP-D-glucuronate
2.7.7.44
K12447
K16190
D-Glucuronate-1P
CNC05140
1.1.1.19
L-Gulonate
L-Lyxonate
Pentose phosphate pathway
K00699
K01195...
β-D-Glucuronoside
D-Glucuronate
K13247
CNL06460
3-Dehydro-L-gulonate
K08092
L-Xylonate
(4R,5S)-4,5,6-Trihydroxy-2,3-dioxohexanoate
Glucuronate interconversion
Galactose metabolism
UDP-glucose
Inositol phosphate metabolism
CNC03700
K00880
Glycolysis
α-D-Glucose-1P
Glycolaldehyde
4.1.1.34
3-Dehydro-L-gulonate-6P
L-Xylulose-1P
K01629
K00848
Glycerone-P
K03078
Starch and sucrose metabolism
K17743
Xylitol
1.1.1.15
L-Xylulose
L-Arabitol
L-Arabinose
D-Xylonolactone
K22185
K00078...
D-Xylose
K00011
K03331
1.1.1.12
K00011
K00594
K14274
K01805
K05351
CNA01050...
K01820
K00880
K01804
D-Xylulose-5P
CNH03870...
L-Lyxose
D-Xylonate
5.3.1.15
K00007
CNC06260
D-Xylulose
L-Xylulose-5P
1.1.1.13
K14275...
K03079
D-Lyxose
D-Arabitol
2,5-Dioxo-pentanoate
K14259
2-Dehydro-3-deoxy-D-xylonate
K03077
K00853
L-Ribulose
L-Ribulose-5P
K17738
CNC06260
CNA06630
Pentose interconversion
K13877...
K22187
4.1.2.28
Ribitol
K00039
K00853
K00875
K21680
K05352...
D-Ribitol-5P
K21030...
CDP-ribitol
2-Oxoglutarate
D-Ribulose
D-Ribulose-5P
1.1.2.2
Pyruvate
Citrate cycle
Riboflavin metabolism
Pentose phosphate pathway
00040 11/10/17
(c) Kanehisa Laboratories

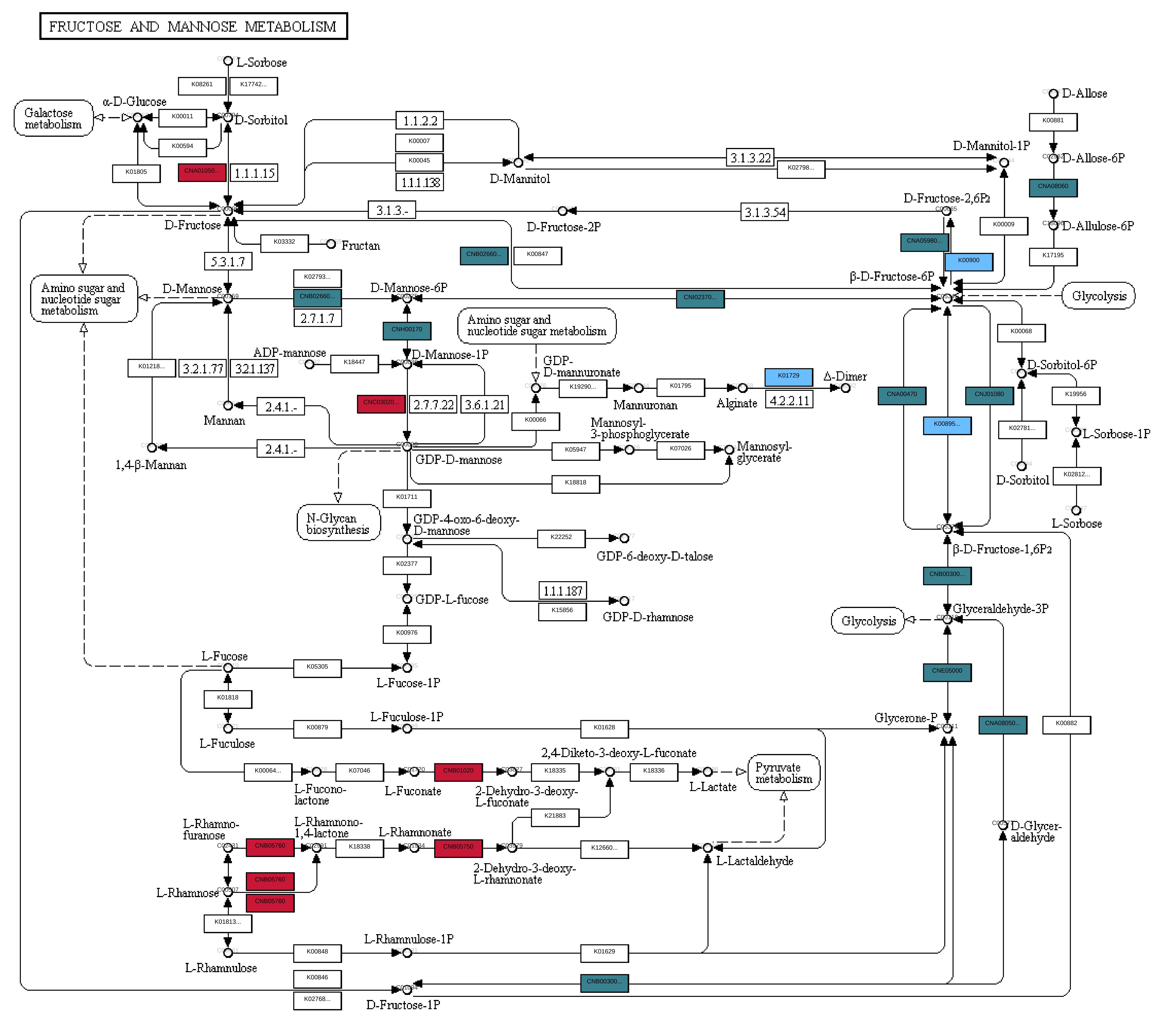


00051 7/6/17
(c) Kanehisa Laboratories

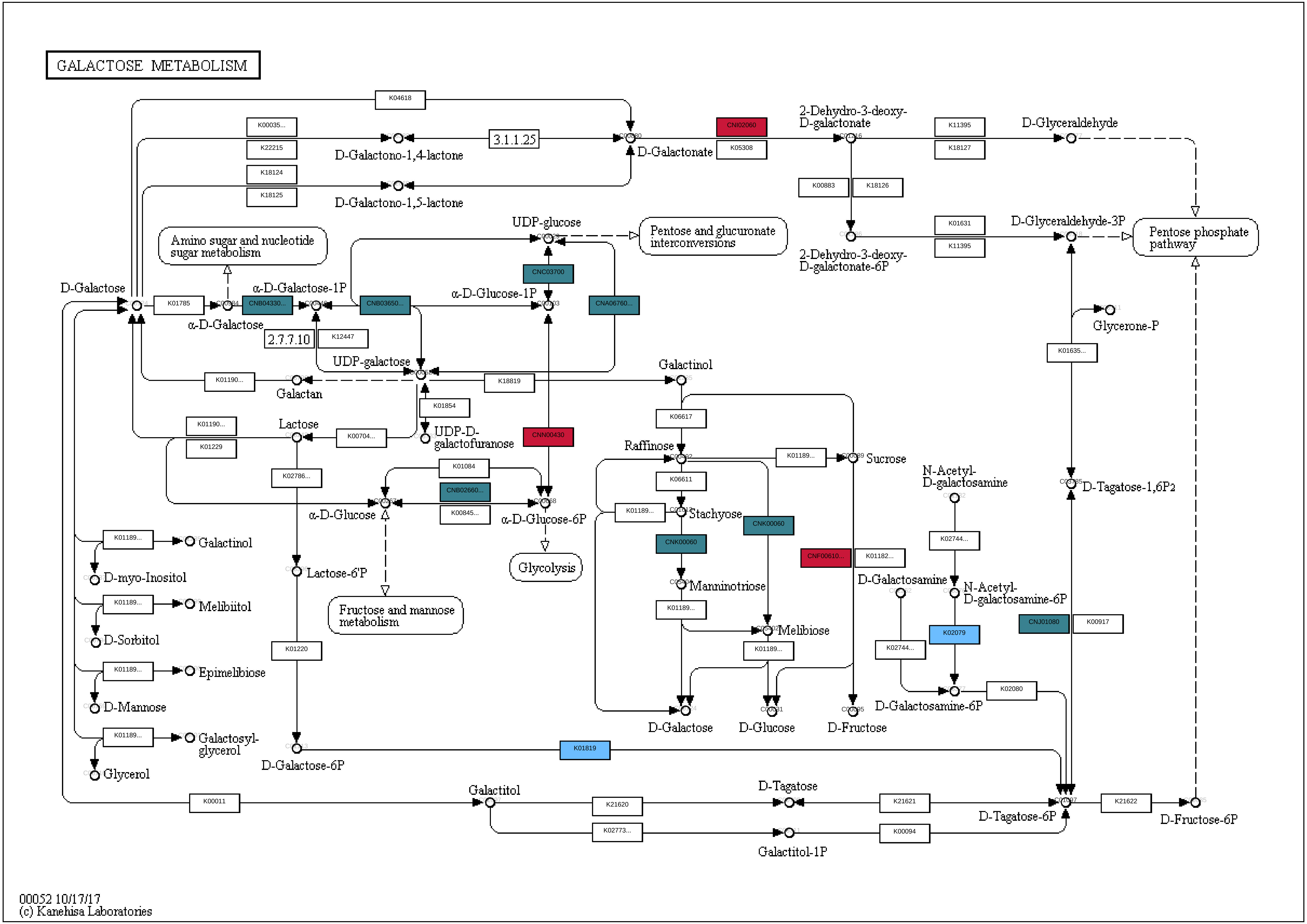
GALACTOSE METABOLISM
K04618
K00035...
K22215
K18124
K18125
3.1.1.25
D-Galactono-1,4-lactone
D-Galactono-1,5-lactone
D-Galactonate
CNI02060
K05308
2-Dehydro-3-deoxy-
D-galactonate
K11395
K18127
D-Glyceraldehyde
K00883
K18126
K01631
K11395
D-Glyceraldehyde-3P
2-Dehydro-3-deoxy-
D-galactonate-6P
Pentose phosphate
pathway
Pentose and glucuronate
interconversions
UDP-glucose
Amino sugar and nucleotide
sugar metabolism
D-Galactose
K01785
CNB04330...
α-D-Galactose-1P
CNB03650...
CNC03700
α-D-Glucose-1P
CNA06760...
α-D-Galactose
2.7.7.10
K12447
UDP-galactose
K01190...
Galactan
K18819
Galactinol
K01854
UDP-D-
galactofuranose
Glycerone-P
K01635...
K01190...
K01229
Lactose
K00704...
CNN00430
K06617
Raffinose
K01189...
Sucrose
K02786...
K01084
CNB02660...
K00845...
α-D-Glucose
α-D-Glucose-6P
K06611
K01189...
Stachyose
CNK00060
CNK00060
N-Acetyl-
D-galactosamine
D-Tagatose-1,6P2
K01189...
Galactinol
D-myo-Inositol
Glycolysis
CNF00610...
K01182...
K02744...
Lactose-6P
Mannninotriose
D-Galactosamine
N-Acetyl-
D-galactosamine-6P
K01189...
Melibiitol
D-Sorbitol
Fructose and mannose
metabolism
K01189...
Melibiose
K02079
CNJ01080
K00917
K01220
K01189...
K02744...
K01189...
Epimelibiose
D-Mannose
K02080
D-Galactosamine-6P
D-Galactose
D-Glucose
D-Fructose
K01189...
Galactosyl-
glycerol
Glycerol
D-Galactose-6P
K01819
K00011
Galactitol
K21620
D-Tagatose
K21621
K21622
D-Tagatose-6P
D-Fructose-6P
K02773...
Galactitol-1P
K00094
00052 10/17/17
(c) Kanehisa Laboratories

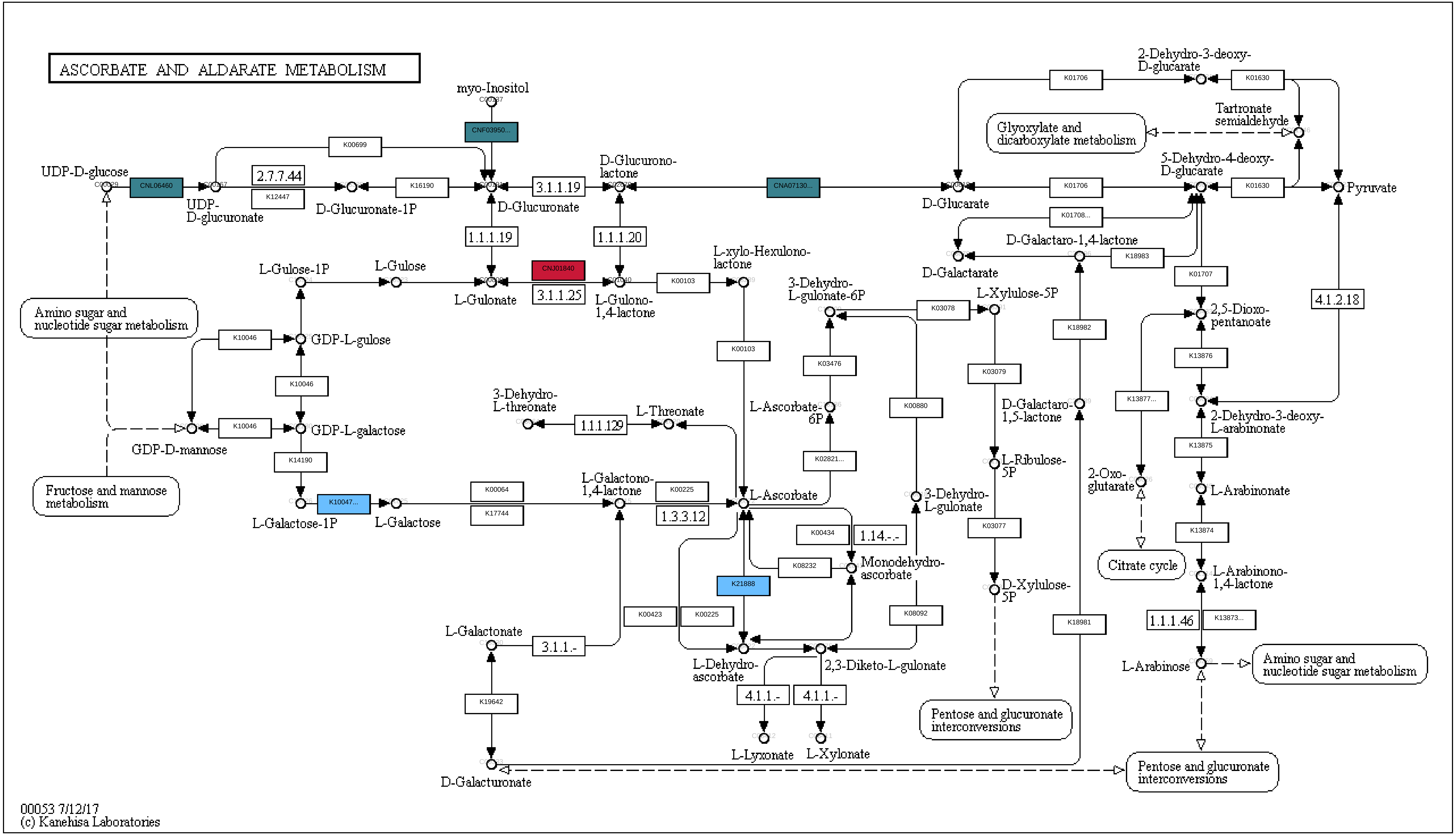

ASCORBATE AND ALDARATE METABOLISM
myo-Inositol
CNF03950...
K00699
UDP-D-glucose
CNL06460
2.7.7.44
K12447
UDP-D-glucuronate
D-Glucuronate-1P
K16190
D-Glucuronate
3.1.1.19
D-Glucurono-lactone
CNA07130...
D-Glucarate
K01706
K01630
2-Dehydro-3-deoxy-D-glucarate
Tartronate semialdehyde
Glyoxylate and dicarboxylate metabolism
5-Dehydro-4-deoxy-D-glucarate
Pyruvate
K01708...
D-Galactaro-1,4-lactone
K18983
D-Galactarate
K01707
4.1.2.18
2,5-Dioxo-pentanoate
K13876
K13877...
2-Dehydro-3-deoxy-L-arabinonate
K13875
L-Arabinonate
2-Oxo-glutarate
Citrate cycle
K13874
L-Arabinono-1,4-lactone
1.1.1.46
K13873...
L-Arabinose
Amino sugar and nucleotide sugar metabolism
Pentose and glucuronate interconversions
1.1.1.19
1.1.1.20
CNJ01840
3.1.1.25
L-Gulose-1P
L-Gulose
L-Gulonate
L-Gulono-1,4-lactone
K00103
L-xylo-Hexulono-lactone
3-Dehydro-L-gulonate-6P
K03078
L-Xylulose-5P
K18982
Amino sugar and nucleotide sugar metabolism
K10046
GDP-L-gulose
K10046
K00103
K03476
K00880
K03079
D-Galactaro-1,5-lactone
L-Ascorbate-6P
K02821...
L-Ribulose-5P
3-Dehydro-L-threonate
1.1.1.129
L-Threonate
K10046
GDP-D-mannose
GDP-L-galactose
K14190
Fructose and mannose metabolism
K10047...
L-Galactose-1P
L-Galactose
K00064
K17744
L-Galactono-1,4-lactone
K00225
1.3.3.12
L-Ascorbate
3-Dehydro-L-gulonate
K03077
K00434
1.14.-.-
K08232
Monodehydro-ascorbate
D-Xylulose-5P
K21888
K00423
K00225
K08092
K18981
L-Galactonate
3.1.1.-
L-Dehydro-ascorbate
2,3-Diketo-L-gulonate
4.1.1.-
4.1.1.-
K19642
L-Lyxonate
L-Xylonate
Pentose and glucuronate interconversions
D-Galacturonate
Pentose and glucuronate interconversions
00053 7/12/17
(c) Kanehisa Laboratories

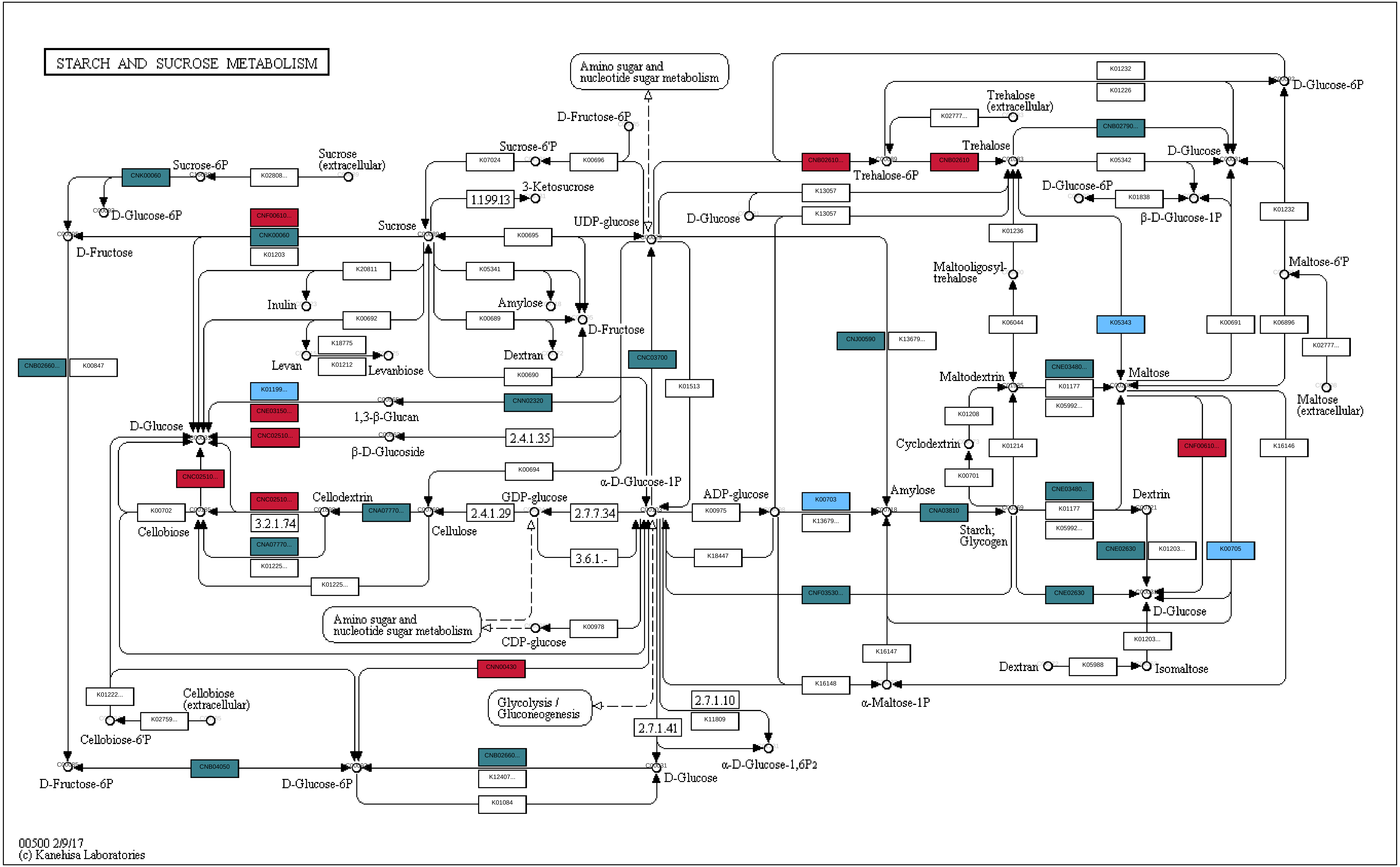
STARCH AND SUCROSE METABOLISM
Amino sugar and nucleotide sugar metabolism
D-Fructose-6P
Sucrose-6'P
Sucrose-6P
Sucrose (extracellular)
K07024
K00696
K02808...
CNK00060
D-Glucose-6P
3-Ketosucrose
1.1.99.13
CNF00610...
CNK00060
K01203
D-Fructose
Sucrose
K00695
UDP-glucose
D-Glucose
K13057
K13057
K20811
K05341
Inulin
Amylose
K00692
K00689
D-Fructose
K18775
Levan
K01212
Levanbiose
Dextran
K00690
CNB02660...
K00847
K01199...
CNN02320
CNE03150...
1,3-β-Glucan
D-Glucose
CNC02510...
2.4.1.35
β-D-Glucoside
CNC02510...
K00694
α-D-Glucose-1P
CNC02510...
Cellodextrin
GDP-glucose
ADP-glucose
K00702
CNA07770...
2.4.1.29
2.7.7.34
K00975
K00703
Cellobiose
3.2.1.74
Cellulose
K13679...
CNA07770...
3.6.1.-
K18447
K01225...
K01225...
CNF03530...
Amino sugar and nucleotide sugar metabolism
K00978
CDP-glucose
CNN00430
K01222...
Cellobiose (extracellular)
Glycolysis / Gluconeogenesis
2.7.1.10
K02759...
K11809
Cellobiose-6'P
2.7.1.41
α-D-Glucose-1,6P2
CNB04050
CNB02660...
D-Fructose-6P
D-Glucose-6P
K12407...
D-Glucose
K01084
CNC03700
K01513
K01232
K01226
D-Glucose-6P
Trehalose (extracellular)
K02777...
CNB02790...
CNB02610...
CNB02610
Trehalose
K05342
D-Glucose
Trehalose-6P
D-Glucose-6P
K01838
β-D-Glucose-1P
K01232
K01236
Maltooligosyl-trehalose
Maltose-6'P
CNJ00590
K13679...
K06044
K05343
K00691
K06896
K02777...
Maltodextrin
CNE03480...
Maltose
K01177
Maltose (extracellular)
K05992...
K01208
Cyclodextrin
K01214
CNF00610...
K16146
K00701
Amylose
CNE03480...
Dextrin
CNA03810
K01177
Starch; Glycogen
K05992...
CNE02630
K01203...
K00705
CNE02630
D-Glucose
K16147
K01203...
Dextran
K05988
Isomaltose
K16148
α-Maltose-1P
00500 2/9/17
(c) Kanehisa Laboratories

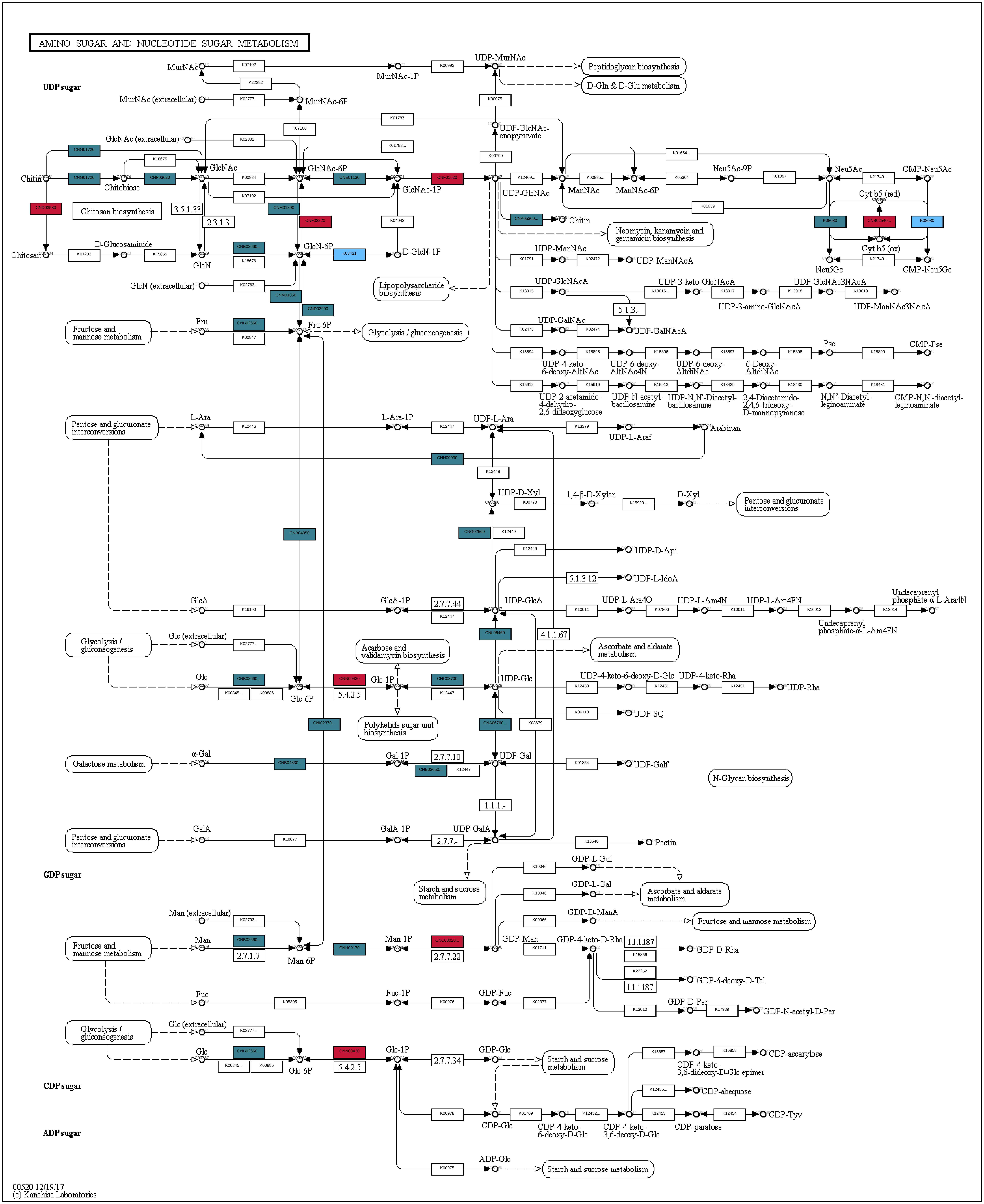
AMINO SUGAR AND NUCLEOTIDE SUGAR METABOLISM
UDP sugar
MurNAc
MurNAc-1P
UDP-MurNAc
Peptidoglycan biosynthesis
D-Gln & D-Glu metabolism
MurNAc (extracellular)
MurNAc-6P
UDP-GlcNAc-enopyruvate
GlcNAc (extracellular)
Chitin
Chitobiose
GlcNAc
GlcNAc-6P
GlcNAc-1P
UDP-GlcNAc
ManNAc
ManNAc-6P
Neu5Ac-9P
Neu5Ac
CMP-Neu5Ac
Cyt b5 (red)
Cyt b5 (ox)
Neu5Gc
CMP-Neu5Gc
Chitosan biosynthesis
3.5.1.33
2.3.1.3
Chitin
Neomycin, kanamycin and gentamicin biosynthesis
Chitosan
D-Glucosaminide
GlcN
GlcN-6P
D-GlcN-1P
UDP-ManNAc
UDP-ManNAcA
GlcN (extracellular)
Lipopolysaccharide biosynthesis
UDP-GlcNAcA
UDP-3-keto-GlcNAcA
UDP-GlcNAc3NAcA
UDP-3-amino-GlcNAcA
UDP-ManNAc3NAcA
5.1.3.-
UDP-GalNAc
UDP-GalNAcA
Fructose and mannose metabolism
Fru
Fru-6P
Glycolysis / gluconeogenesis
Pse
CMP-Pse
UDP-4-keto-6-deoxy-AltNAc
UDP-6-deoxy-AltNAc4N
UDP-6-deoxy-AltdiNAc
6-Deoxy-AltdiNAc
UDP-2-acetamido-4-dehydro-2,6-dideoxyglucose
UDP-N-acetyl-bacillosamine
UDP-N,N'-Diacetyl-bacillosamine
2,4-Diacetamido-2,4,6-trideoxy-D-mannopyranose
N,N'-Diacetyl-legionaminate
CMP-N,N'-diacetyl-legionaminate
Pentose and glucuronate interconversions
L-Ara
L-Ara-1P
UDP-L-Ara
UDP-L-Araf
Arabinan
UDP-D-Xyl
1,4-β-D-Xylan
D-Xyl
Pentose and glucuronate interconversions
UDP-D-Api
5.1.3.12
UDP-L-IdoA
GlcA
GlcA-1P
2.7.7.44
UDP-GlcA
UDP-L-Ara4O
UDP-L-Ara4N
UDP-L-Ara4FN
Undecaprenyl phosphate-α-L-Ara4N
Undecaprenyl phosphate-α-L-Ara4FN
4.1.1.67
Glycolysis / gluconeogenesis
Glc (extracellular)
Acarbose and validamycin biosynthesis
Ascorbate and aldarate metabolism
Glc
Glc-6P
Glc-1P
5.4.2.5
UDP-Glc
UDP-4-keto-6-deoxy-D-Glc
UDP-4-keto-Rha
UDP-Rha
UDP-SQ
Polyketide sugar unit biosynthesis
Galactose metabolism
α-Gal
Gal-1P
2.7.7.10
UDP-Gal
UDP-Galf
N-Glycan biosynthesis
1.1.1.-
Pentose and glucuronate interconversions
GalA
GalA-1P
2.7.7.-
UDP-GalA
Pectin
GDP sugar
GDP-L-Gul
Starch and sucrose metabolism
GDP-L-Gal
Ascorbate and aldarate metabolism
GDP-D-ManA
Fructose and mannose metabolism
Man (extracellular)
Fructose and mannose metabolism
Man
2.7.1.7
Man-6P
Man-1P
2.7.7.22
GDP-Man
GDP-4-keto-D-Rha
1.1.1.187
GDP-D-Rha
GDP-6-deoxy-D-Tal
1.1.1.187
Fuc
Fuc-1P
GDP-Fuc
GDP-D-Per
GDP-N-acetyl-D-Per
Glycolysis / gluconeogenesis
Glc (extracellular)
Glc
Glc-6P
5.4.2.5
Glc-1P
2.7.7.34
GDP-Glc
Starch and sucrose metabolism
CDP-ascarylose
CDP-4-keto-3,6-dideoxy-D-Glc epimer
CDP sugar
CDP-abequose
CDP-Glc
CDP-4-keto-6-deoxy-D-Glc
CDP-4-keto-3,6-deoxy-D-Glc
CDP-paratose
CDP-Tyv
ADP sugar
ADP-Glc
Starch and sucrose metabolism
00520 12/19/17
(c) Kanehisa Laboratories

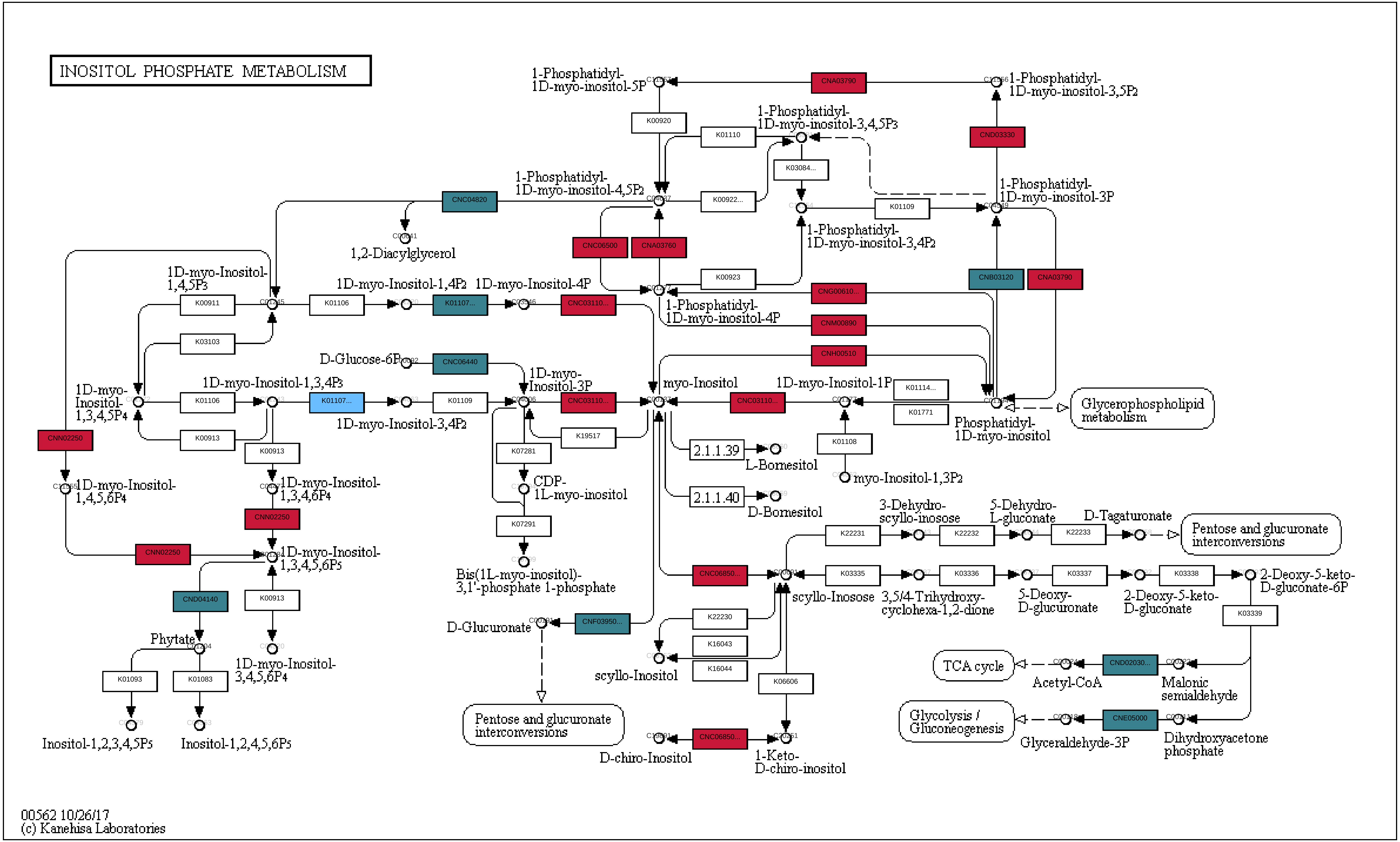
INOSITOL PHOSPHATE METABOLISM
1-Phosphatidyl-1D-myo-inositol-5P
CNA03790
1-Phosphatidyl-1D-myo-inositol-3,5P2
K00920
K01110
1-Phosphatidyl-1D-myo-inositol-3,4,5P3
CND03330
K03084...
1-Phosphatidyl-1D-myo-inositol-4,5P2
CNC04820
K00922...
1-Phosphatidyl-1D-myo-inositol-3P
K01109
1,2-Diacylglycerol
CNC06500
CNA03760
1-Phosphatidyl-1D-myo-inositol-3,4P2
K00923
CNB03120
CNA03790
1D-myo-Inositol-1,4,5P3
1D-myo-Inositol-1,4P2
1D-myo-Inositol-4P
K00911
K01106
CNK01107...
CNC03110...
CNG00610...
1-Phosphatidyl-1D-myo-inositol-4P
K03103
CNM00890
D-Glucose-6P
CNC06440
CNH00510
1D-myo-Inositol-3P
1D-myo-Inositol-1,3,4P3
myo-Inositol
1D-myo-Inositol-1P
1D-myo-Inositol-1,3,4,5P4
K01106
K01107...
K01109
CNC03110...
CNC03110...
K01114...
K01771
Glycerophospholipid metabolism
Phosphatidyl-1D-myo-inositol
1D-myo-Inositol-3,4P2
K19517
K00913
K07281
CNN02250
K00913
2.1.1.39
L-Bornesitol
K01108
myo-Inositol-1,3P2
1D-myo-Inositol-1,4,5,6P4
1D-myo-Inositol-1,3,4,6P4
CDP-1L-myo-inositol
CNN02250
2.1.1.40
D-Bornesitol
3-Dehydro-scyllo-inosose
5-Dehydro-L-gluconate
D-Tagaturonate
K07291
K22231
K22232
K22233
Pentose and glucuronate interconversions
CNN02250
1D-myo-Inositol-1,3,4,5,6P5
Bis(1L-myo-inositol)-3,1'-phosphate 1-phosphate
CNC06850...
K03335
K03336
K03337
K03338
2-Deoxy-5-keto-D-gluconate-6P
CND04140
K00913
scyllo-Inosose
3,5/4-Trihydroxy-cyclohexa-1,2-dione
5-Deoxy-D-glucuronate
2-Deoxy-5-keto-D-gluconate
K03339
D-Glucuronate
CNF03950...
K22230
Phytate
K16043
1D-myo-Inositol-3,4,5,6P4
scyllo-Inositol
K16044
TCA cycle
CND02030...
Acetyl-CoA
Malonic semialdehyde
K01093
K01083
K06606
Pentose and glucuronate interconversions
Glycolysis / Gluconeogenesis
CNE05000
Glyceraldehyde-3P
Dihydroxyacetone phosphate
Inositol-1,2,3,4,5P5
Inositol-1,2,4,5,6P5
CNC06850...
D-chiro-Inositol
1-Keto-D-chiro-inositol
00562 10/26/17
(c) Kanehisa Laboratories

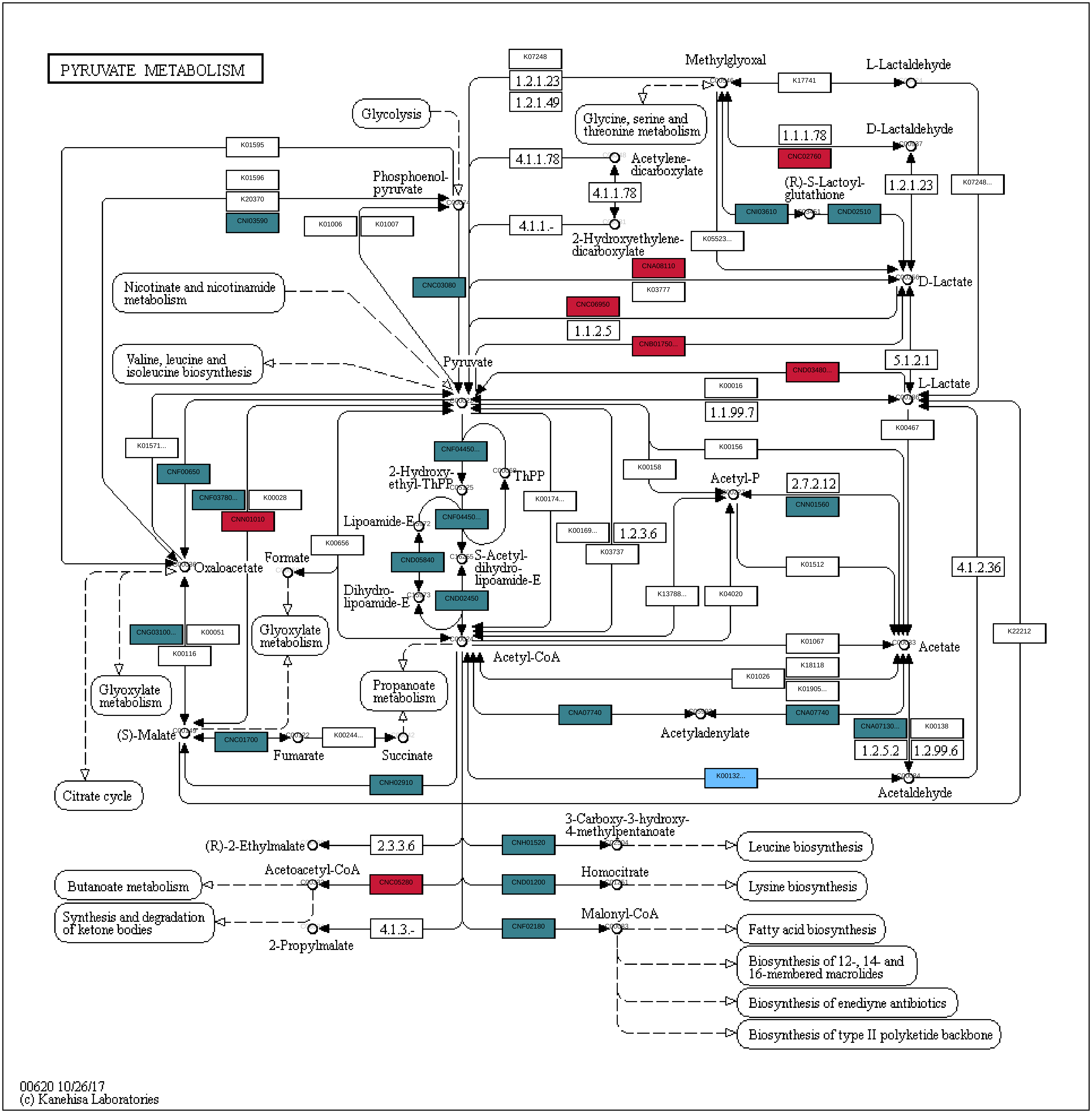
PYRUVATE METABOLISM
Glycolysis
Phosphoenol-pyruvate
Methylglyoxal
L-Lactaldehyde
D-Lactaldehyde
Glycine, serine and threonine metabolism
Acetylene-dicarboxylate
2-Hydroxyethylene-dicarboxylate
(R)-S-Lactoyl-glutathione
D-Lactate
L-Lactate
Pyruvate
Nicotinate and nicotinamide metabolism
Valine, leucine and isoleucine biosynthesis
Oxaloacetate
Formate
Glyoxylate metabolism
2-Hydroxy-ethyl-ThPP
ThPP
Lipoamide-E
S-Acetyl-dihydro-lipoamide-E
Dihydro-lipoamide-E
Acetyl-P
Acetyl-CoA
Acetate
Acetyladenylate
Acetaldehyde
Propanoate metabolism
(S)-Malate
Fumarate
Succinate
Citrate cycle
1.2.1.23
1.2.1.49
4.1.1.78
4.1.1.-
1.1.1.78
1.2.1.23
1.1.2.5
5.1.2.1
1.1.99.7
2.7.2.12
1.2.3.6
4.1.2.36
1.2.5.2
1.2.99.6
2.3.3.6
4.1.3.-
K07248
K17741
K01595
K01596
K20370
K01006
K01007
K07248...
K05523...
K03777
K00016
K00467
K00156
K00158
K00174...
K00169...
K03737
K01512
K13788...
K04020
K01571...
K00028
K00656
K00051
K00116
K00244...
K01067
K18118
K01026
K01905...
K00138
K22212
CNC02760
CNI03610
CND02510
CNI03590
CNC03080
CNA08110
CNC06950
CNB01750...
CND03480...
CNF04450...
CNF00650
CNF03780...
CNN01010
CND05840
CND02450
CNN01560
CNG03100...
CNC01700
CNH02910
CNA07740
CNA07130...
K00132...
(R)-2-Ethylmalate
3-Carboxy-3-hydroxy-4-methylpentanoate
Leucine biosynthesis
Acetoacetyl-CoA
Homocitrate
Butanoate metabolism
Lysine biosynthesis
Synthesis and degradation of ketone bodies
2-Propylmalate
Malonyl-CoA
Fatty acid biosynthesis
Biosynthesis of 12-, 14- and 16-membered macrolides
Biosynthesis of enediyne antibiotics
Biosynthesis of type II polyketide backbone
CNH01520
CNC05280
CND01200
CNF02180
00620 10/26/17
(c) Kanehisa Laboratories

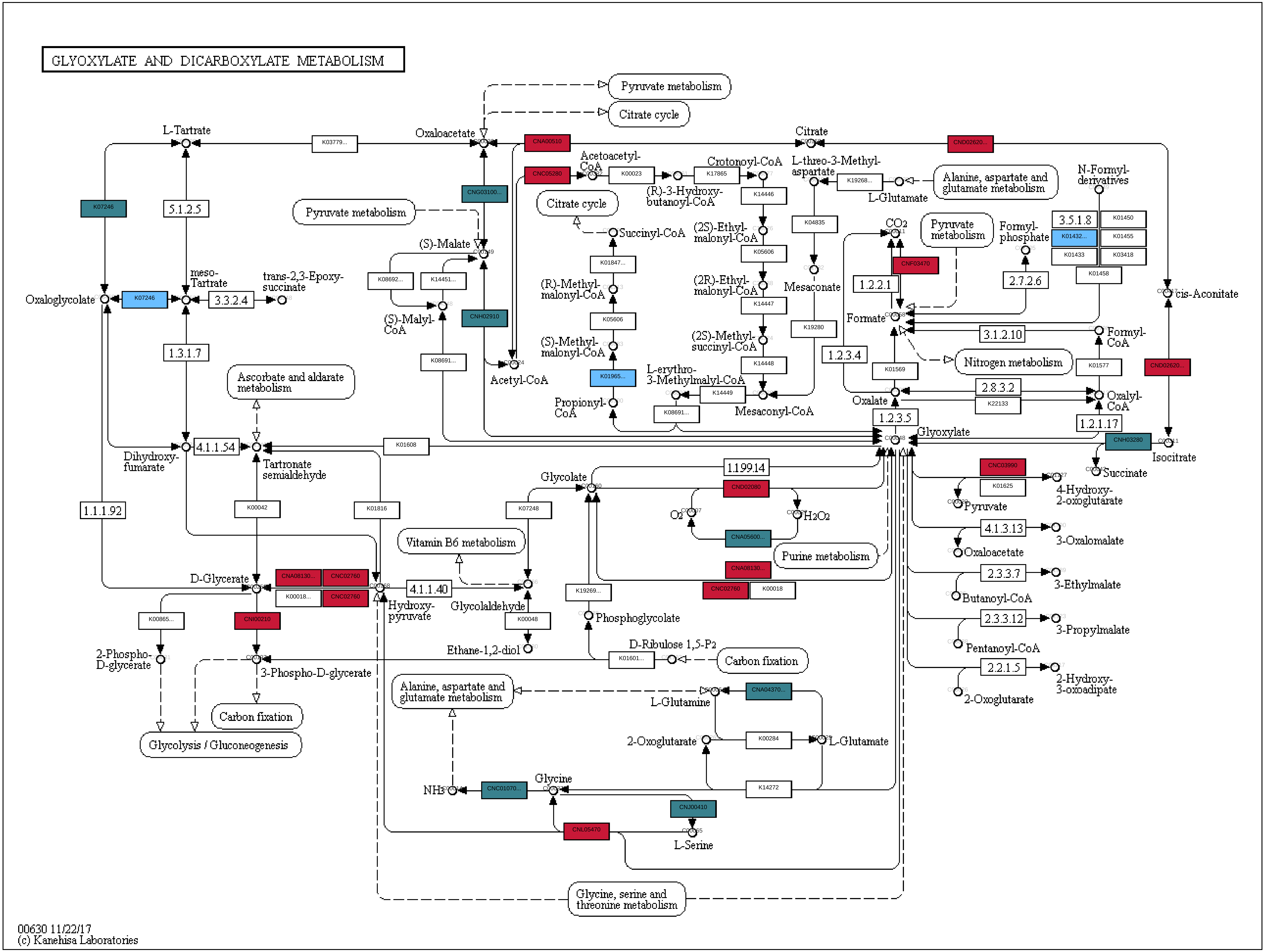
GLYOXYLATE AND DICARBOXYLATE METABOLISM
Pyruvate metabolism
Citrate cycle
L-Tartrate
K03779...
Oxaloacetate
CNA00510
Citrate
CND02620...
Acetoacetyl-CoA
CNC05280
K00023
Crotonoyl-CoA
K17865
L-threo-3-Methyl-aspartate
K19268...
Alanine, aspartate and glutamate metabolism
N-Formyl-derivatives
K07246
5.1.2.5
CNG03100...
Pyruvate metabolism
Citrate cycle
(R)-3-Hydroxy-butanoyl-CoA
K14446
L-Glutamate
K04835
Succinyl-CoA
(2S)-Ethyl-malonyl-CoA
CO2
Pyruvate metabolism
Formyl-phosphate
3.5.1.8
K01450
K01432...
K01455
K01433
K03418
K01458
(S)-Malate
K08692...
K14451...
meso-Tartrate
trans-2,3-Epoxy-succinate
K01847...
K05606
CNF03470
Oxaloglycolate
K07246
3.3.2.4
(R)-Methyl-malonyl-CoA
(2R)-Ethyl-malonyl-CoA
K14447
Mesaconate
1.2.2.1
2.7.2.6
cis-Aconitate
CNH02910
(S)-Malyl-CoA
K05606
K19280
Formate
3.1.2.10
Formyl-CoA
1.3.1.7
K08691...
(S)-Methyl-malonyl-CoA
(2S)-Methyl-succinyl-CoA
1.2.3.4
Nitrogen metabolism
Ascorbate and aldarate metabolism
Acetyl-CoA
K01965...
K14448
L-erythro-3-Methylmalyl-CoA
K01569
K01577
CND02620...
Propionyl-CoA
K14449
K08691...
Mesaconyl-CoA
Oxalate
2.8.3.2
K22133
Oxalyl-CoA
1.2.3.5
1.2.1.17
Glyoxylate
4.1.1.54
K01608
CNH03280
Isocitrate
Dihydroxy-fumarate
Tartronate semialdehyde
CNC03990
Succinate
Glycolate
1.1.99.14
K01625
4-Hydroxy-2-oxoglutarate
1.1.1.92
K00042
K01816
K07248
CND02080
Pyruvate
O2
H2O2
4.1.3.13
Vitamin B6 metabolism
CNA05600...
3-Oxalomalate
Purine metabolism
Oxaloacetate
CNA08130...
D-Glycerate
CNA08130...
CNC02760
2.3.3.7
K00018...
CNC02760
4.1.1.40
K19269...
CNC02760
K00018
3-Ethylmalate
Hydroxy-pyruvate
Glycolaldehyde
Butanoyl-CoA
K00865...
CNI00210
K00048
Phosphoglycolate
2.3.3.12
3-Propylmalate
2-Phospho-D-glycerate
Ethane-1,2-diol
D-Ribulose 1,5-P2
Pentanoyl-CoA
K01601...
Carbon fixation
3-Phospho-D-glycerate
2.2.1.5
2-Hydroxy-3-oxoadipate
Alanine, aspartate and glutamate metabolism
CNA04370...
L-Glutamine
2-Oxoglutarate
Carbon fixation
Glycolysis / Gluconeogenesis
2-Oxoglutarate
K00284
L-Glutamate
Glycine
NH3
CNC01070...
K14272
CNJ00410
CNL05470
L-Serine
Glycine, serine and threonine metabolism
00630 11/22/17
(c) Kanehisa Laboratories

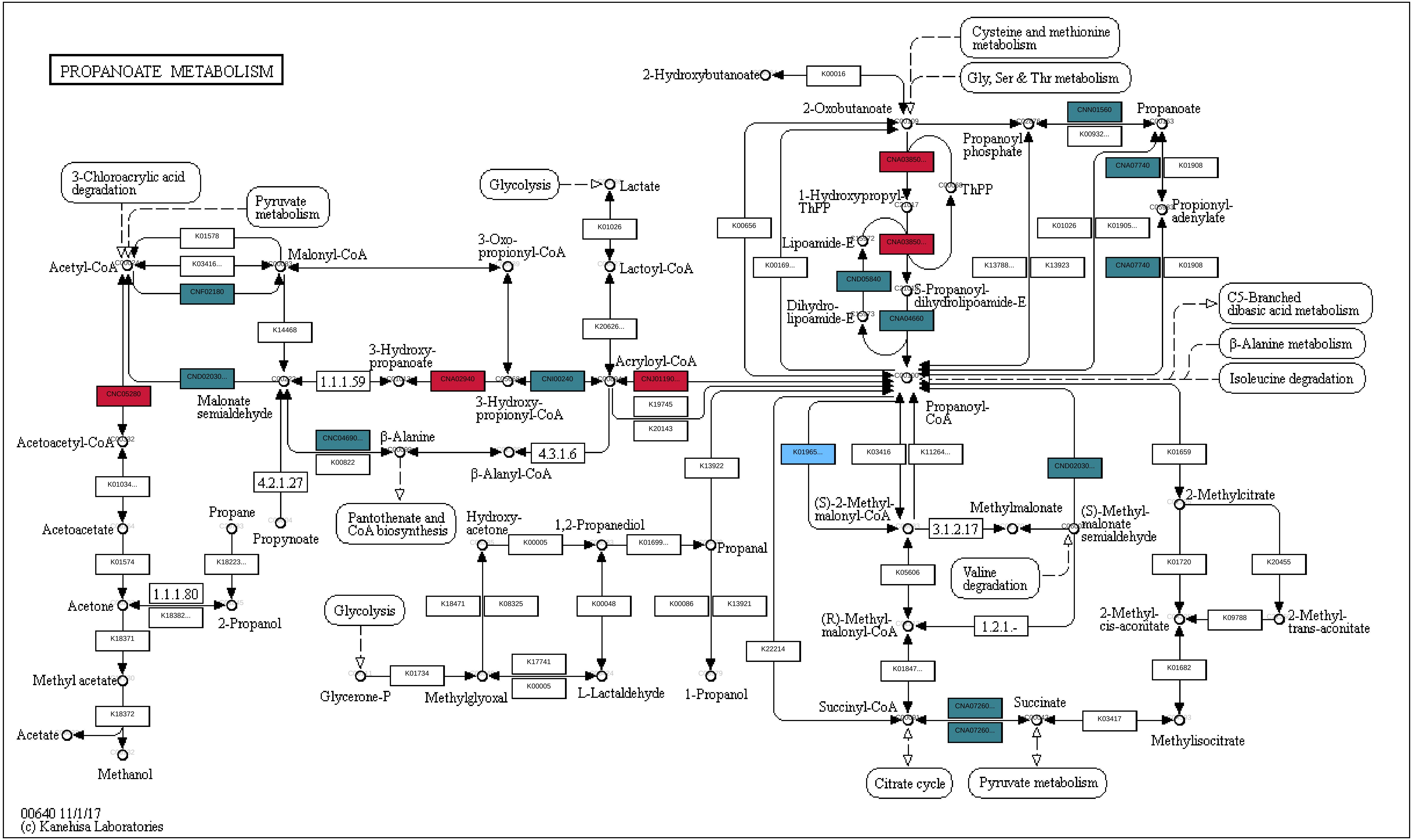
PROPANOATE METABOLISM
2-Hydroxybutanoate
K00016
Cysteine and methionine metabolism
Gly, Ser & Thr metabolism
2-Oxobutanoate
Propanoate
CNN01560
K00932...
Propanoyl phosphate
CNA03850...
ThPP
1-Hydroxypropyl-ThPP
Lipoamide-E
CNA03850...
K00656
K00169...
CND05840
S-Propanoyl-dihydrolipoamide-E
Dihydro-lipoamide-E
CNA04660
CNA07740
K01908
Propionyl-adenylate
K01026
K01905...
K13788...
K13923
CNA07740
K01908
C5-Branched dibasic acid metabolism
β-Alanine metabolism
Isoleucine degradation
Propanoyl-CoA
3-Chloroacrylic acid degradation
Pyruvate metabolism
K01578
K03416...
CNF02180
Acetyl-CoA
Malonyl-CoA
Glycolysis
Lactate
K01026
Lactoyl-CoA
3-Oxo-propionyl-CoA
K14468
K20626...
3-Hydroxy-propanoate
Acryloyl-CoA
CND02030...
1.1.1.59
CNA02940
CNI00240
CNJ01190...
K19745
K20143
CNC05280
Malonate semialdehyde
3-Hydroxy-propionyl-CoA
Acetoacetyl-CoA
CNC04690...
K00822
β-Alanine
β-Alanyl-CoA
4.3.1.6
K13922
K01965...
K03416
K11264...
CND02030...
K01659
K01034...
4.2.1.27
Pantothenate and CoA biosynthesis
(S)-2-Methyl-malonyl-CoA
Methylmalonate
3.1.2.17
(S)-Methyl-malonate semialdehyde
2-Methylcitrate
Acetoacetate
Propane
Propynoate
Hydroxy-acetone
1,2-Propanediol
K00005
K01699...
Propanal
K01574
K18223...
K05606
Valine degradation
K01720
K20455
1.1.1.80
Acetone
K18382...
2-Propanol
K18471
K08325
K00048
K00086
K13921
Glycolysis
(R)-Methyl-malonyl-CoA
1.2.1.-
2-Methyl-cis-aconitate
K09788
2-Methyl-trans-aconitate
K18371
K22214
Methyl acetate
K01734
K17741
K00005
K01847...
K01682
Glycerone-P
Methylglyoxal
L-Lactaldehyde
1-Propanol
Succinyl-CoA
CNA07260...
CNA07260...
Succinate
K03417
Methylisocitrate
K18372
Acetate
Methanol
Citrate cycle
Pyruvate metabolism
00640 11/1/17
(c) Kanehisa Laboratories

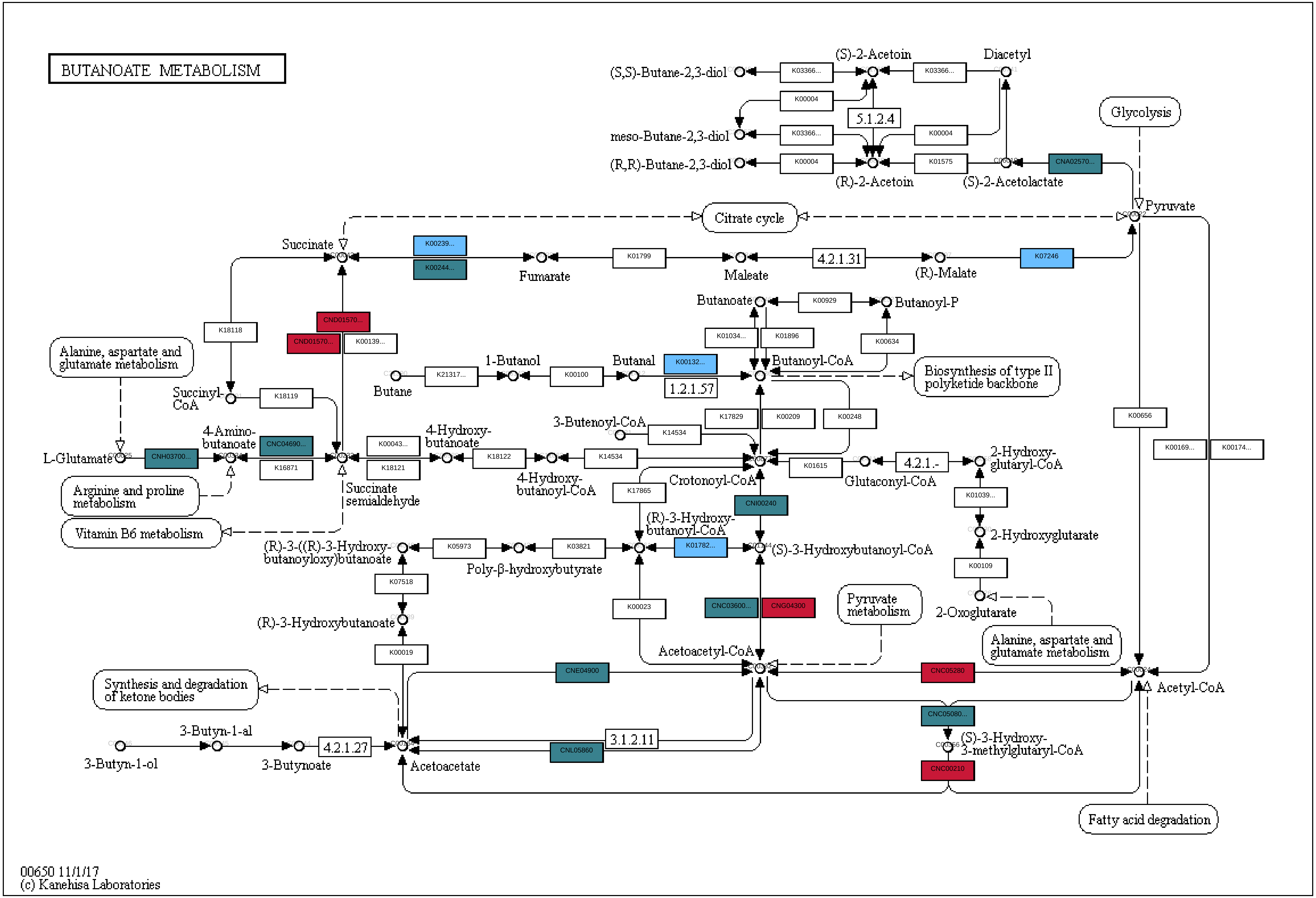

BUTANOATE METABOLISM
(S,S)-Butane-2,3-diol
(S)-2-Acetoin
Diacetyl
K03366...
K03366...
K00004
5.1.2.4
meso-Butane-2,3-diol
K03366...
K00004
(R,R)-Butane-2,3-diol
K00004
K01575
CNA02570...
(R)-2-Acetoin
(S)-2-Acetolactate
Glycolysis
Pyruvate
Citrate cycle
Succinate
K00239...
K00244...
Fumarate
K01799
Maleate
4.2.1.31
(R)-Malate
K07246
Butanoate
K00929
Butanoyl-P
K01034...
K01896
K00634
K18118
CND01570...
CND01570...
K00139...
Alanine, aspartate and glutamate metabolism
1-Butanol
Butanal
K00132...
Butanoyl-CoA
Biosynthesis of type II polyketide backbone
K21317...
K00100
1.2.1.57
Butane
Succinyl-CoA
K18119
K17829
K00209
K00248
K00656
3-Butenoyl-CoA
K14534
4-Amino-butanoate
CNC04690...
4-Hydroxy-butanoate
K00043...
L-Glutamate
CNH03700...
K16871
K18121
K18122
K14534
K01615
Glutaconyl-CoA
4.2.1.-
2-Hydroxy-glutaryl-CoA
K00169...
K00174...
4-Hydroxy-butanoyl-CoA
Crotonoyl-CoA
Arginine and proline metabolism
Succinate semialdehyde
K17865
CNI00240
K01039...
Vitamin B6 metabolism
(R)-3-Hydroxy-butanoyl-CoA
2-Hydroxyglutarate
(R)-3-((R)-3-Hydroxy-butanoyloxy)butanoate
K05973
K03821
K01782...
(S)-3-Hydroxybutanoyl-CoA
Poly-β-hydroxybutyrate
K00109
K07518
K00023
CNC03600...
CNG04300
Pyruvate metabolism
2-Oxoglutarate
(R)-3-Hydroxybutanoate
Alanine, aspartate and glutamate metabolism
K00019
Acetoacetyl-CoA
Synthesis and degradation of ketone bodies
CNE04900
CNC05280
Acetyl-CoA
CNC05080...
3-Butyn-1-al
(S)-3-Hydroxy-3-methylglutaryl-CoA
4.2.1.27
3.1.2.11
3-Butyn-1-ol
3-Butynoate
CNL05860
Acetoacetate
CNC00210
Fatty acid degradation
00650 11/1/17
(c) Kanehisa Laboratories

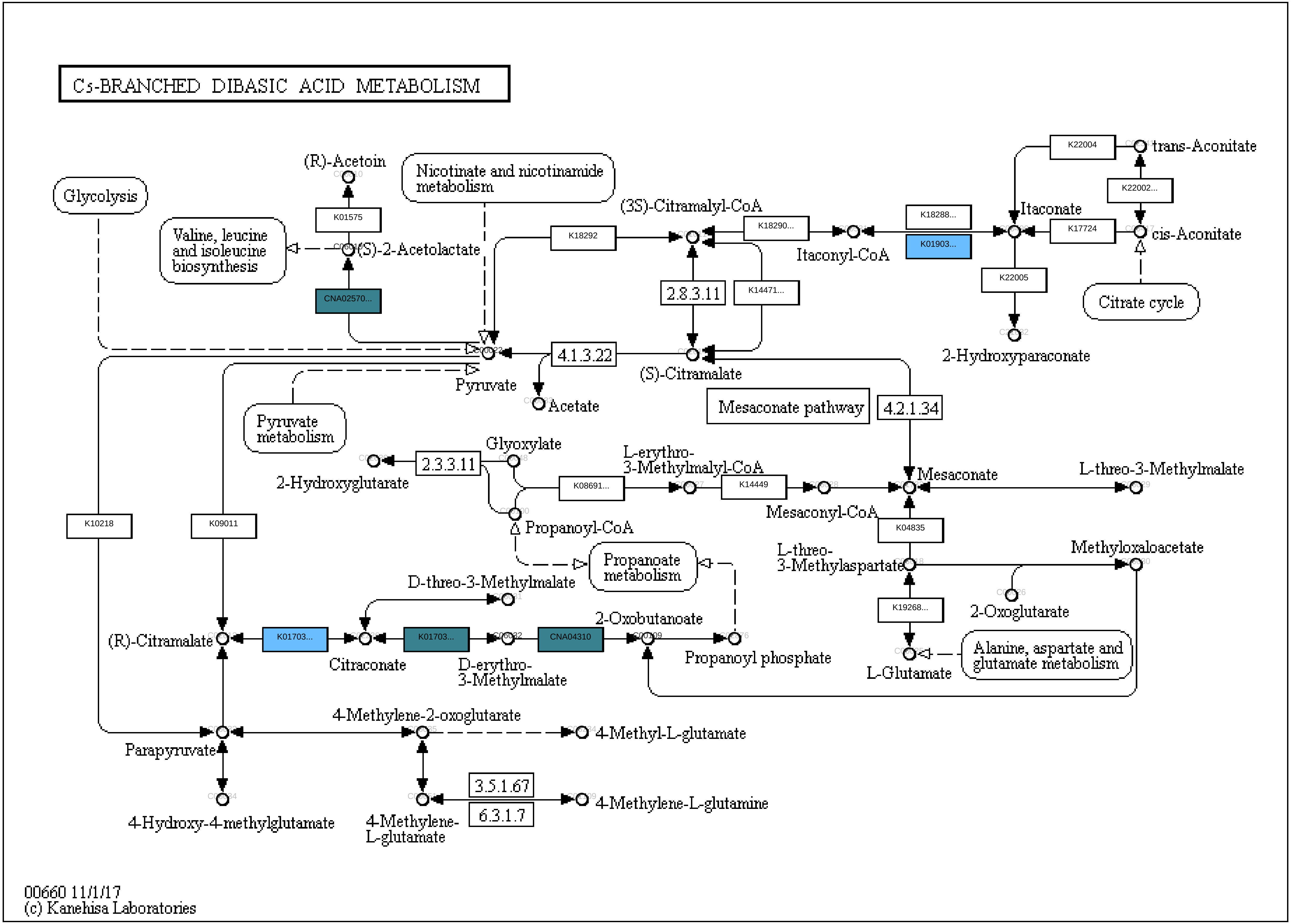

C5-BRANCHED DIBASIC ACID METABOLISM
(R)-Acetoin
Nicotinate and nicotinamide metabolism
(3S)-Citramalyl-CoA
K22004
trans-Aconitate
Glycolysis
K01575
K22002...
Valine, leucine and isoleucine biosynthesis
(S)-2-Acetolactate
K18292
K18290...
K18288...
Itaconate
K17724
cis-Aconitate
K01903...
Itaconyl-CoA
CNA02570...
2.8.3.11
K14471...
K22005
Citrate cycle
4.1.3.22
Pyruvate
(S)-Citramalate
2-Hydroxyparaconate
Acetate
Mesaconate pathway
4.2.1.34
Pyruvate metabolism
Glyoxylate
2.3.3.11
L-erythro-3-Methylmalyl-CoA
2-Hydroxyglutarate
K08691...
K14449
Mesaconate
L-threo-3-Methylmalate
Mesaconyl-CoA
K10218
K09011
Propanoyl-CoA
K04835
L-threo-3-Methylaspartate
Methyloxaloacetate
Propanoate metabolism
D-threo-3-Methylmalate
2-Oxoglutarate
K19268...
2-Oxobutanoate
(R)-Citramalate
K01703...
K01703...
CNA04310
Alanine, aspartate and glutamate metabolism
Citraconate
D-erythro-3-Methylmalate
Propanoyl phosphate
L-Glutamate
4-Methylene-2-oxoglutarate
4-Methyl-L-glutamate
Parapyruvate
3.5.1.67
6.3.1.7
4-Methylene-L-glutamine
4-Hydroxy-4-methylglutamate
4-Methylene-L-glutamate
00660 11/1/17
(c) Kanehisa Laboratories

# 2. Energy metabolism

| MAP | PATHWAY |
|---|---|
| **190** | Oxidative phosphorylation |
| **680** | Methane metabolism |
| **910** | Nitrogen metabolism |
| **920** | Sulfur metabolism |

# OXIDATIVE PHOSPHORYLATION

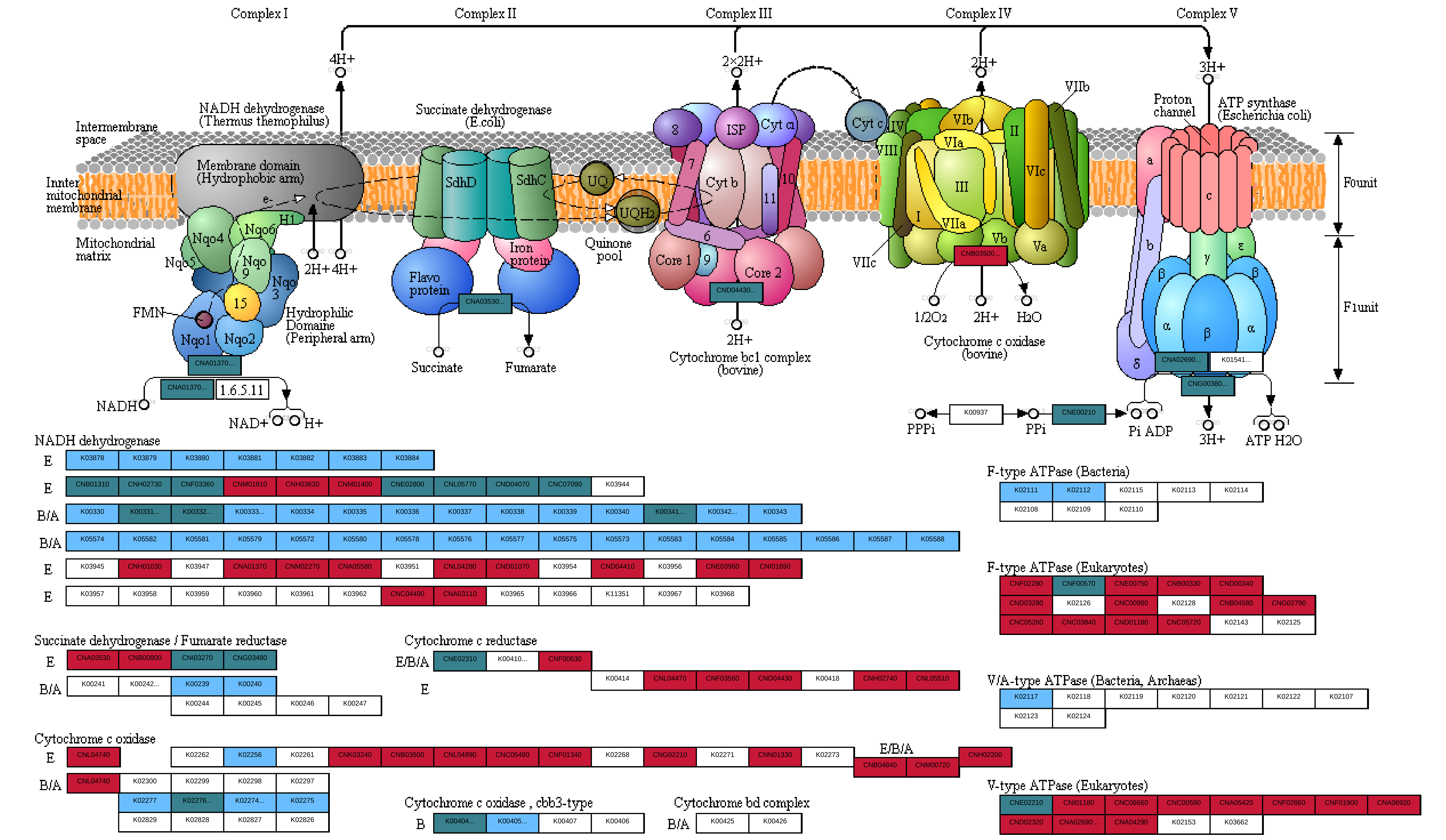


00190 6/16/15

(c) Kanehisa Laboratories

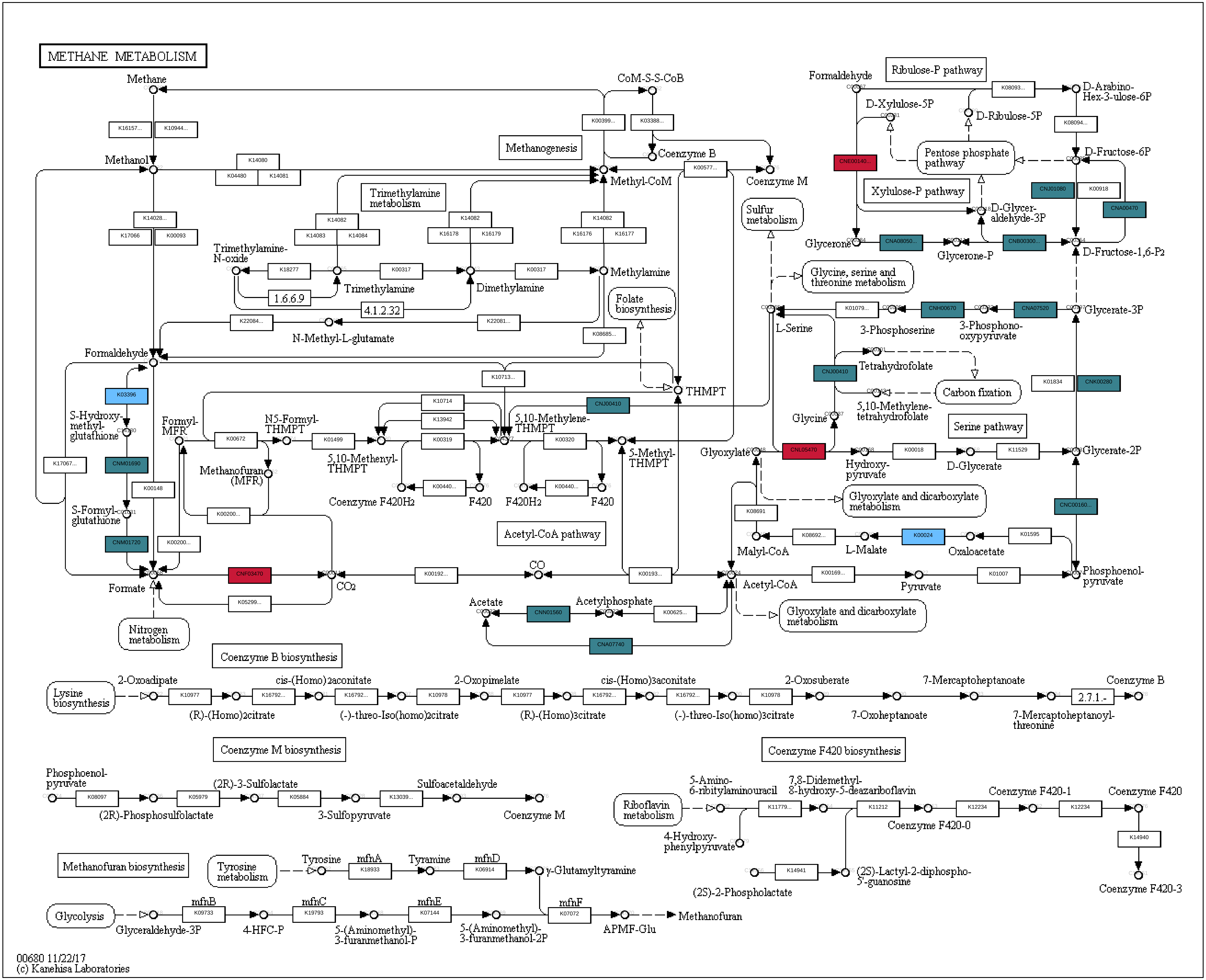
METHANE METABOLISM
Methane
CoM-S-S-CoB
K16157...
K10944...
K00399...
K03388...
Methanogenesis
Methanol
K14080
K04480
K14081
Coenzyme B
K00577...
Coenzyme M
Methyl-CoM
Trimethylamine metabolism
Sulfur metabolism
K14028...
K14082
K14082
K14082
K17066
K00093
K14083
K14084
K16178
K16179
K16176
K16177
Trimethylamine-N-oxide
K18277
K00317
K00317
Methylamine
1.6.6.9
Trimethylamine
Dimethylamine
4.1.2.32
Folate biosynthesis
K22084...
K22081...
N-Methyl-L-glutamate
K08685...
Formaldehyde
K10713...
THMPT
K03396
K10714
CNJ00410
K13942
S-Hydroxy-methyl-glutathione
Formyl-MFR
N5-Formyl-THMPT
5,10-Methylene-THMPT
K00672
K01499
K00319
K00320
5-Methyl-THMPT
K17067...
CNM01690
Methanofuran (MFR)
5,10-Methenyl-THMPT
K00148
K00440...
K00440...
Coenzyme F420H2
F420
F420H2
F420
K00200...
S-Formyl-glutathione
Acetyl-CoA pathway
CNM01720
K00200...
CO
CNF03470
K00192...
K00193...
Formate
CO2
K05299...
Acetate
Acetylphosphate
CNN01560
K00625...
Nitrogen metabolism
CNA07740
Formaldehyde
Ribulose-P pathway
K08093...
D-Arabino-Hex-3-ulose-6P
D-Xylulose-5P
D-Ribulose-5P
K08094...
CNE00140...
Pentose phosphate pathway
D-Fructose-6P
Xylulose-P pathway
CNJ01080
K00918
CNA00470
D-Glycer-aldehyde-3P
Glycerone
CNA08050...
CNB00300...
Glycerone-P
D-Fructose-1,6-P2
Glycine, serine and threonine metabolism
K01079...
CNH00670
CNA07520
Glycerate-3P
L-Serine
3-Phosphoserine
3-Phosphono-oxypyruvate
Tetrahydrofolate
CNJ00410
Carbon fixation
K01834
CNK00280
5,10-Methylene-tetrahydrofolate
Glycine
Serine pathway
Glyoxylate
CNL05470
K00018
K11529
Glycerate-2P
Hydroxy-pyruvate
D-Glycerate
Glyoxylate and dicarboxylate metabolism
K08691
CNC00160...
K08692...
K00024
K01595
Malyl-CoA
L-Malate
Oxaloacetate
K00169...
K01007
Phosphoenol-pyruvate
Acetyl-CoA
Pyruvate
Glyoxylate and dicarboxylate metabolism
Coenzyme B biosynthesis
Lysine biosynthesis
2-Oxoadipate
K10977
(R)-(Homo)2citrate
cis-(Homo)2aconitate
K16792...
K16792...
(-)-threo-Iso(homo)2citrate
2-Oxopimelate
K10978
K10977
(R)-(Homo)3citrate
cis-(Homo)3aconitate
K16792...
K16792...
(-)-threo-Iso(homo)3citrate
2-Oxosuberate
K10978
7-Oxoheptanoate
7-Mercaptoheptanoate
7-Mercaptoheptanoyl-threonine
2.7.1.-
Coenzyme B
Coenzyme M biosynthesis
Coenzyme F420 biosynthesis
Phosphoenol-pyruvate
K08097
(2R)-Phosphosulfolactate
K05979
(2R)-3-Sulfolactate
K05884
3-Sulfopyruvate
K13039...
Sulfoacetaldehyde
Coenzyme M
Riboflavin metabolism
5-Amino-6-ribitylaminouracil
K11779...
7,8-Didemethyl-8-hydroxy-5-deazariboflavin
K11212
Coenzyme F420-0
K12234
Coenzyme F420-1
K12234
Coenzyme F420
4-Hydroxy-phenylpyruvate
K14940
K14941
(2S)-2-Phospholactate
(2S)-Lactyl-2-diphospho-5'-guanosine
Coenzyme F420-3
Methanofuran biosynthesis
Tyrosine metabolism
Tyrosine
mfnA
K18933
Tyramine
mfnD
K06914
γ-Glutamyltyramine
Glycolysis
Glyceraldehyde-3P
mfnB
K09733
4-HFC-P
mfnC
K19793
5-(Aminomethyl)-3-furanmethanol-P
mfnE
K07144
5-(Aminomethyl)-3-furanmethanol-2P
mfnF
K07072
APMF-Glu
Methanofuran
00680 11/22/17
(c) Kanehisa Laboratories

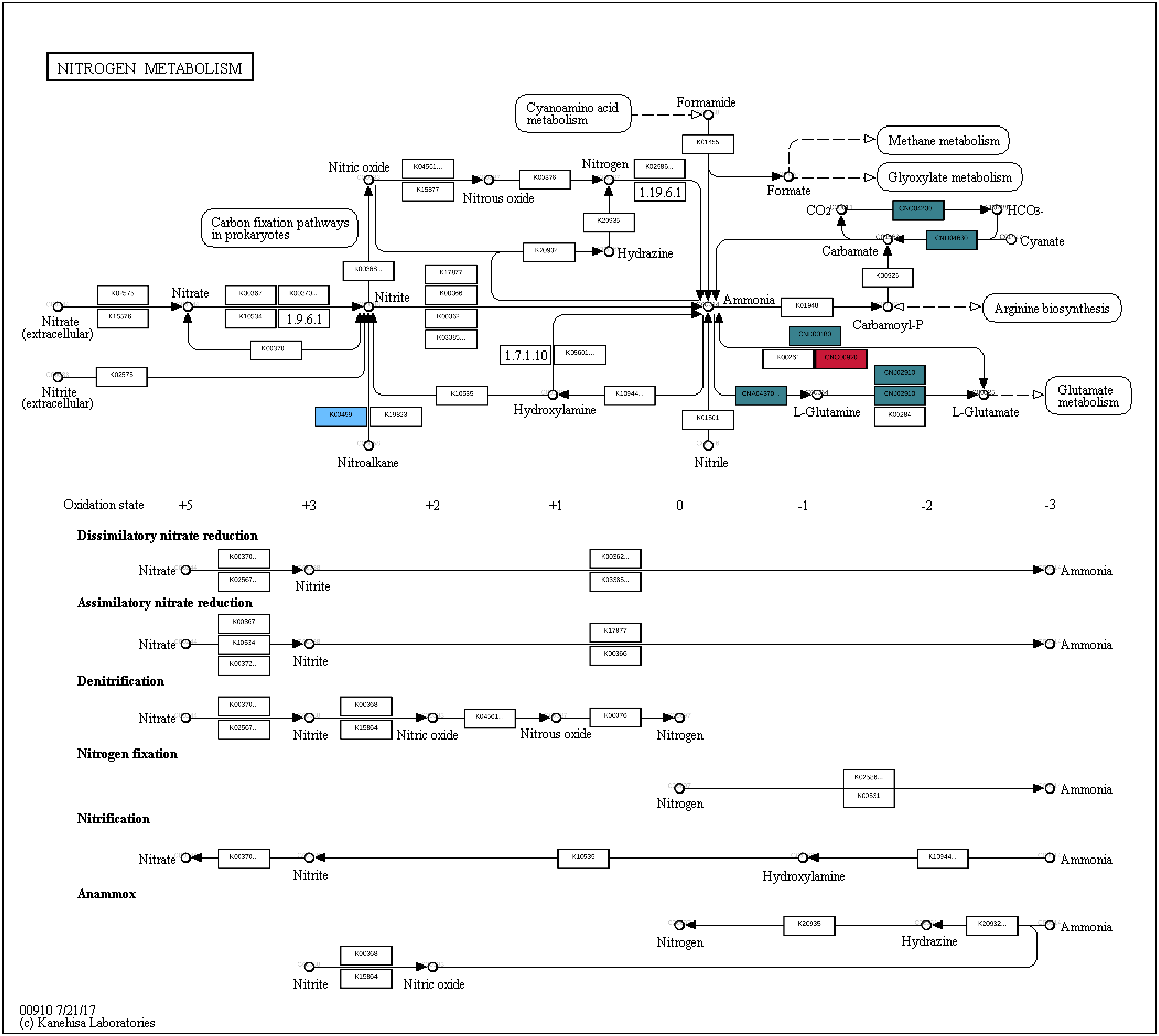
NITROGEN METABOLISM
Cyanoamino acid metabolism
Formamide
K01455
Methane metabolism
Glyoxylate metabolism
Formate
Nitric oxide
K04561...
K15877
Nitrous oxide
K00376
Nitrogen
K02586...
1.19.6.1
K20935
K20932...
Hydrazine
Carbon fixation pathways in prokaryotes
CO2
CNC04230...
HCO3-
CND04630
Cyanate
Carbamate
K00926
K00368...
K17877
K00366
K00362...
K03385...
Nitrate
Nitrite
K02575
K15576...
K00367
K00370...
K10534
1.9.6.1
K00370...
Nitrate (extracellular)
Nitrite (extracellular)
K02575
Ammonia
K01948
Carbamoyl-P
Arginine biosynthesis
CND00180
K00261
CNC00920
CNJ02910
CNA04370...
CNJ02910
K00284
L-Glutamine
L-Glutamate
Glutamate metabolism
1.7.1.10
K05601...
K10535
K10944...
Hydroxylamine
K00459
K19823
Nitroalkane
K01501
Nitrile
Oxidation state
+5
+3
+2
+1
0
-1
-2
-3
Dissimilatory nitrate reduction
Nitrate
K00370...
K02567...
Nitrite
K00362...
K03385...
Ammonia
Assimilatory nitrate reduction
Nitrate
K00367
K10534
K00372...
Nitrite
K17877
K00366
Ammonia
Denitrification
Nitrate
K00370...
K02567...
Nitrite
K00368
K15864
Nitric oxide
K04561...
Nitrous oxide
K00376
Nitrogen
Nitrogen fixation
Nitrogen
K02586...
K00531
Ammonia
Nitrification
Nitrate
K00370...
Nitrite
K10535
Hydroxylamine
K10944...
Ammonia
Anammox
Nitrogen
K20935
Hydrazine
K20932...
Ammonia
Nitrite
K00368
K15864
Nitric oxide
00910 7/21/17
(c) Kanehisa Laboratories

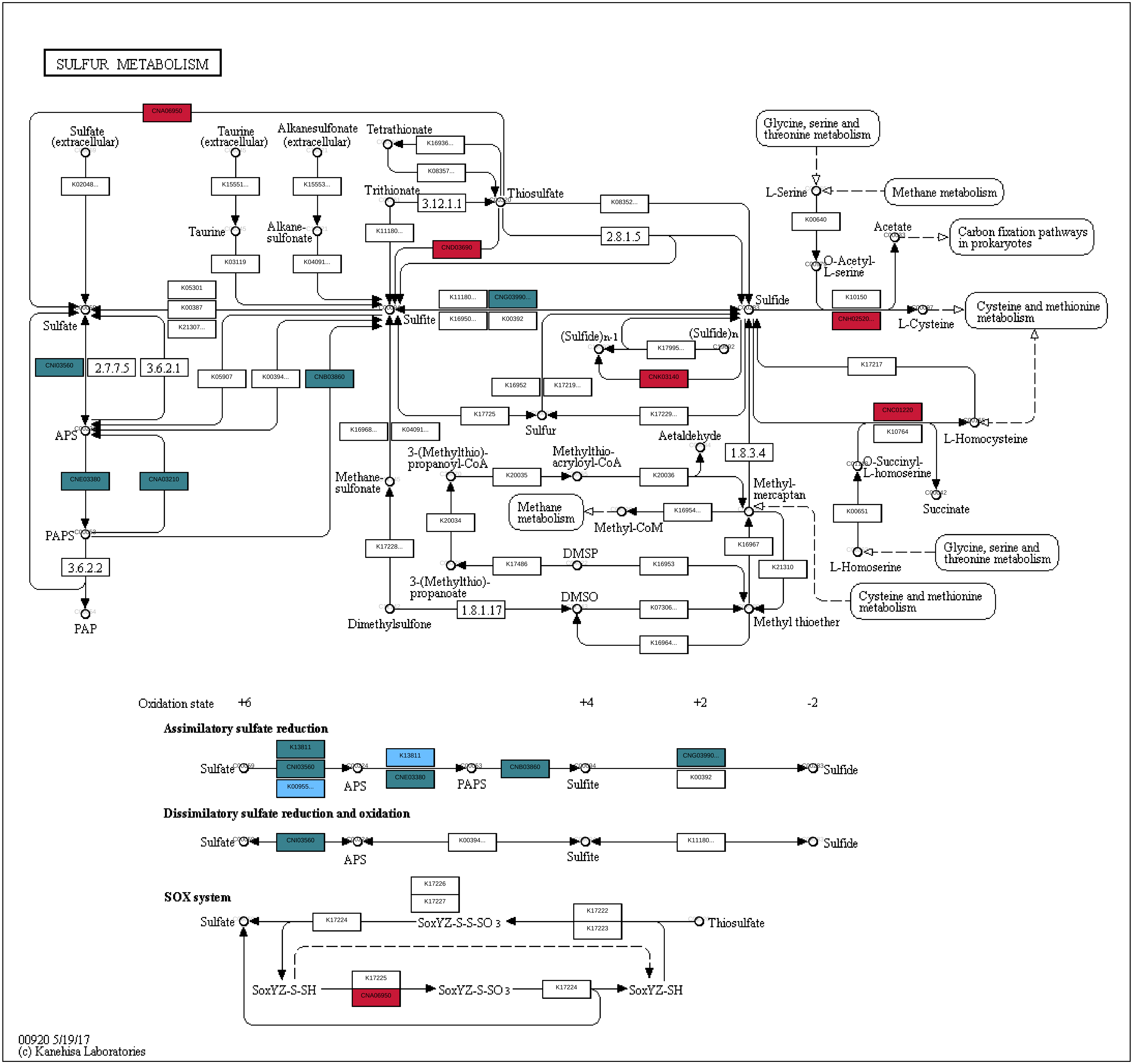
SULFUR METABOLISM
CNA06950
Sulfate (extracellular)
Taurine (extracellular)
Alkanesulfonate (extracellular)
Tetrathionate
K16936...
K08357...
Trithionate
3.12.1.1
Thiosulfate
K08352...
2.8.1.5
Glycine, serine and threonine metabolism
L-Serine
Methane metabolism
K00640
Acetate
Carbon fixation pathways in prokaryotes
O-Acetyl-L-serine
K02048...
K15551...
K15553...
Taurine
Alkane-sulfonate
K11180...
CND03690
K03119
K04091...
K05301
K00387
K21307...
Sulfate
K11180...
CNG03990...
K16950...
K00392
Sulfite
Sulfide
K10150
CNH02520...
L-Cysteine
Cysteine and methionine metabolism
(Sulfide)n-1
(Sulfide)n
K17995...
CNK03140
K17217
CNI03560
2.7.7.5
3.6.2.1
K05907
K00394...
CNB03860
K16952
K17219...
APS
K17725
Sulfur
K17229...
CNC01220
K10764
L-Homocysteine
K16968...
K04091...
Aetaldehyde
1.8.3.4
3-(Methylthio)-propanoyl-CoA
Methylthio-acryloyl-CoA
K20035
K20036
O-Succinyl-L-homoserine
CNE03380
CNA03210
Methane-sulfonate
Methyl-mercaptan
Succinate
Methane metabolism
Methyl-CoM
K16954...
K00651
K20034
PAPS
K17228...
K16967
DMSP
K17486
K16953
K21310
Glycine, serine and threonine metabolism
L-Homoserine
3.6.2.2
3-(Methylthio)-propanoate
DMSO
Cysteine and methionine metabolism
1.8.1.17
K07306...
PAP
Dimethylsulfone
Methyl thioether
K16964...
Oxidation state +6 +4 +2 -2
Assimilatory sulfate reduction
Sulfate
K13811
CNI03560
K00955...
APS
K13811
CNE03380
PAPS
CNB03860
Sulfite
CNG03990...
K00392
Sulfide
Dissimilatory sulfate reduction and oxidation
Sulfate
CNI03560
APS
K00394...
Sulfite
K11180...
Sulfide
SOX system
K17226
K17227
Sulfate
K17224
SoxYZ-S-S-SO 3
K17222
K17223
Thiosulfate
SoxYZ-S-SH
K17225
CNA06950
SoxYZ-S-SO 3
K17224
SoxYZ-SH
00920 5/19/17
(c) Kanehisa Laboratories

# 3. Lipid metabolism

| MAP | PATHWAY |
|---|---|
| **61** | Fatty acid biosynthesis |
| **62** | Fatty acid elongation |
| **71** | Fatty acid degradation |
| **72** | Synthesis and degradation of ketone bodies |
| **100** | Steroid biosynthesis |
| **561** | Glycerolipid metabolism |
| **564** | Glycerophospholipid metabolism |
| **565** | Ether lipid metabolism |
| **590** | Arachidonic acid metabolism |
| **592** | alpha-Linolenic acid metabolism |
| **600** | Sphingolipid metabolism |

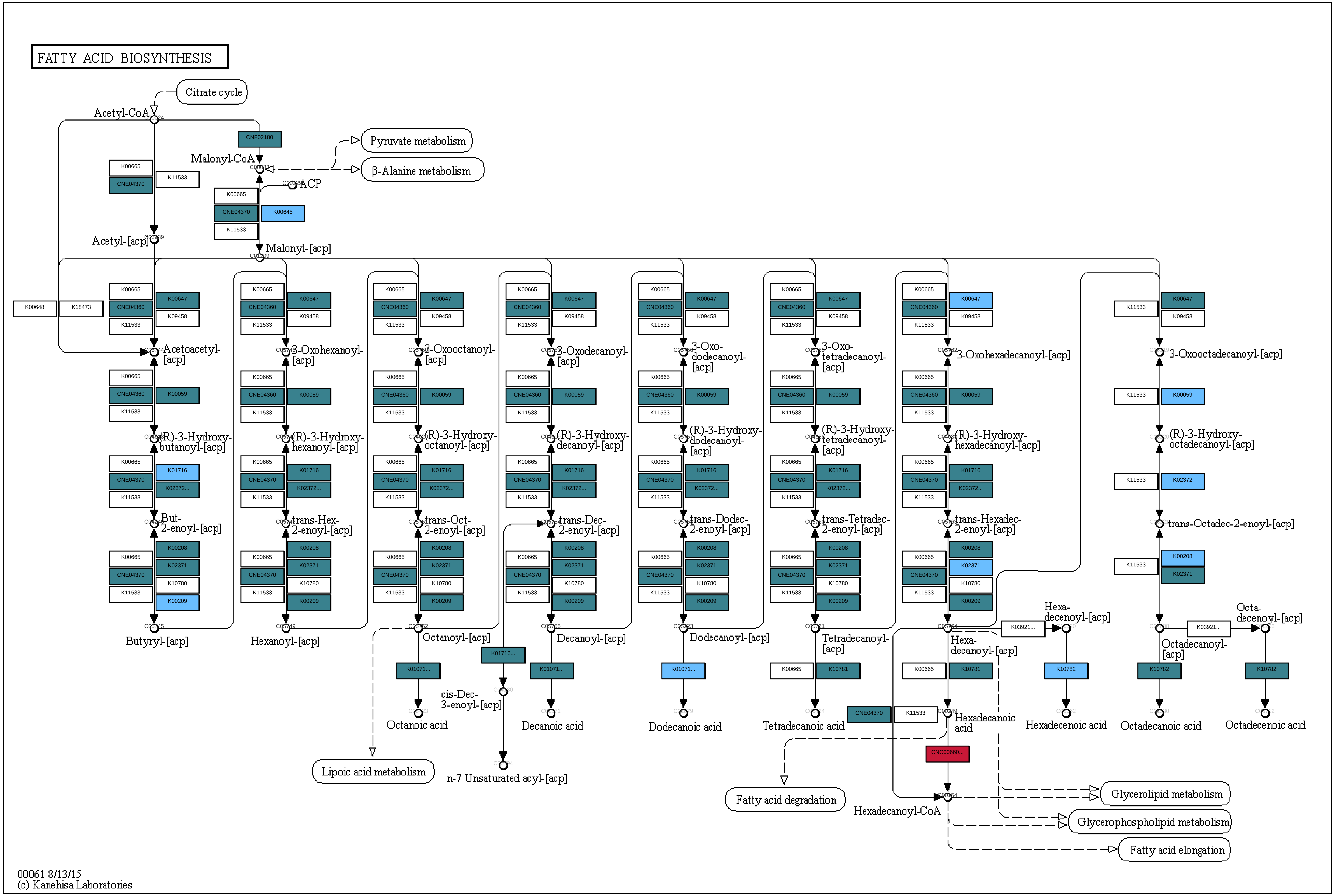

FATTY ACID BIOSYNTHESIS
Citrate cycle
Acetyl-CoA
CNF02180
Malonyl-CoA
Pyruvate metabolism
β-Alanine metabolism
ACP
K00665
CNE04370
K11533
K00645
Acetyl-[acp]
Malonyl-[acp]
K00648
K18473
K00647
CNE04360
K09458
Acetoacetyl-[acp]
3-Oxohexanoyl-[acp]
3-Oxooctanoyl-[acp]
3-Oxodecanoyl-[acp]
3-Oxo-dodecanoyl-[acp]
3-Oxo-tetradecanoyl-[acp]
3-Oxohexadecanoyl-[acp]
3-Oxooctadecanoyl-[acp]
K00059
(R)-3-Hydroxy-butanoyl-[acp]
(R)-3-Hydroxy-hexanoyl-[acp]
(R)-3-Hydroxy-octanoyl-[acp]
(R)-3-Hydroxy-decanoyl-[acp]
(R)-3-Hydroxy-dodecanoyl-[acp]
(R)-3-Hydroxy-tetradecanoyl-[acp]
(R)-3-Hydroxy-hexadecanoyl-[acp]
(R)-3-Hydroxy-octadecanoyl-[acp]
K01716
K02372...
K02372
But-2-enoyl-[acp]
trans-Hex-2-enoyl-[acp]
trans-Oct-2-enoyl-[acp]
trans-Dec-2-enoyl-[acp]
trans-Dodec-2-enoyl-[acp]
trans-Tetradec-2-enoyl-[acp]
trans-Hexadec-2-enoyl-[acp]
trans-Octadec-2-enoyl-[acp]
K00208
K02371
K10780
K00209
Butyryl-[acp]
Hexanoyl-[acp]
Octanoyl-[acp]
Decanoyl-[acp]
Dodecanoyl-[acp]
Tetradecanoyl-[acp]
Hexa-decanoyl-[acp]
Octadecanoyl-[acp]
K01716...
K01071...
K10781
K03921...
Hexa-decenoyl-[acp]
Octa-decenoyl-[acp]
K10782
cis-Dec-3-enoyl-[acp]
Octanoic acid
Decanoic acid
Dodecanoic acid
Tetradecanoic acid
Hexadecanoic acid
Hexadecenoic acid
Octadecanoic acid
Octadecenoic acid
n-7 Unsaturated acyl-[acp]
Lipoic acid metabolism
Fatty acid degradation
CNC00660...
Hexadecanoyl-CoA
Glycerolipid metabolism
Glycerophospholipid metabolism
Fatty acid elongation
00061 8/13/15
(c) Kanehisa Laboratories

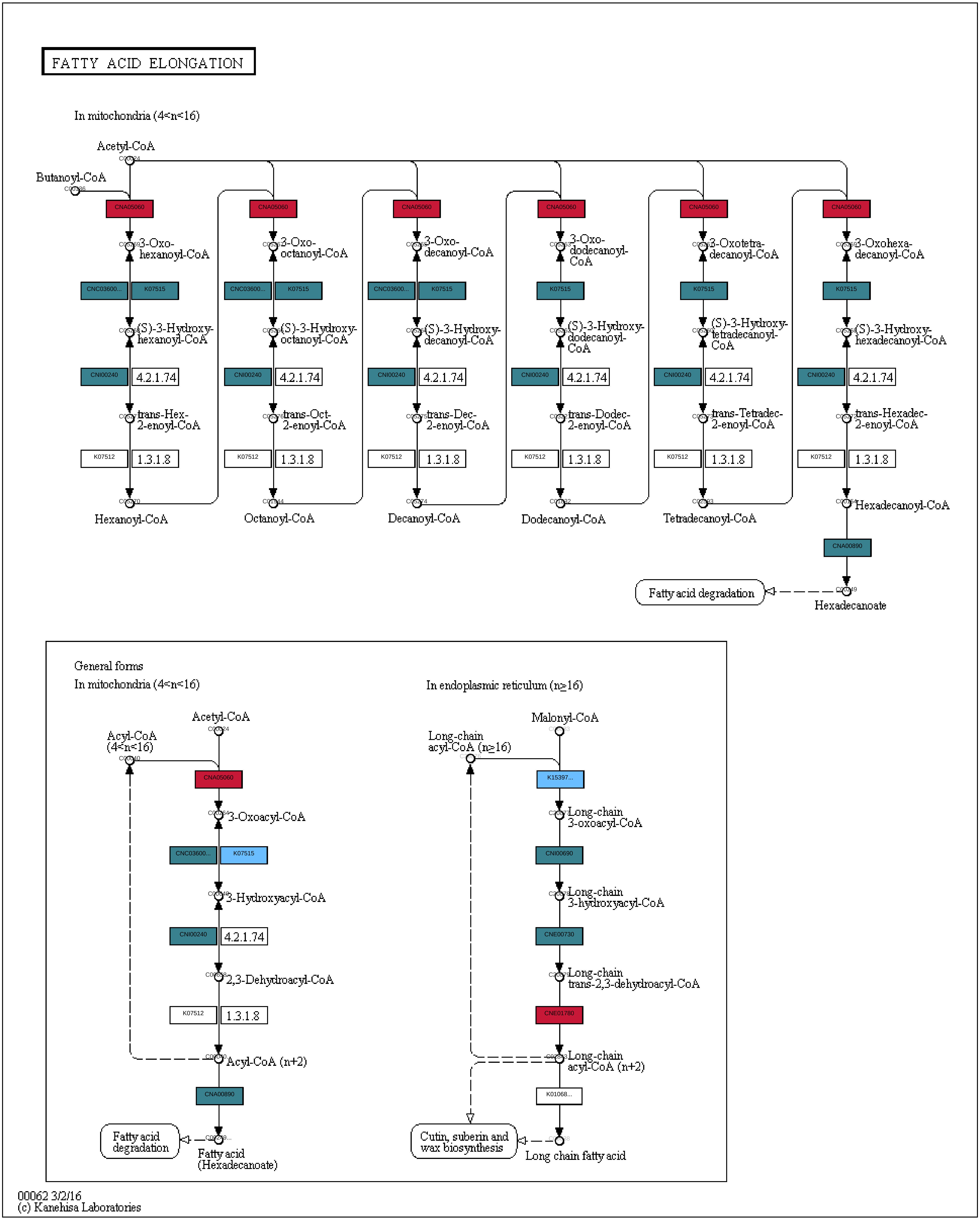

FATTY ACID ELONGATION
In mitochondria (4<n<16)
Acetyl-CoA
Butanoyl-CoA
CNA05060
3-Oxo-hexanoyl-CoA
3-Oxo-octanoyl-CoA
3-Oxo-decanoyl-CoA
3-Oxo-dodecanoyl-CoA
3-Oxotetra-decanoyl-CoA
3-Oxohexa-decanoyl-CoA
CNC03600...
K07515
(S)-3-Hydroxy-hexanoyl-CoA
(S)-3-Hydroxy-octanoyl-CoA
(S)-3-Hydroxy-decanoyl-CoA
(S)-3-Hydroxy-dodecanoyl-CoA
(S)-3-Hydroxy-tetradecanoyl-CoA
(S)-3-Hydroxy-hexadecanoyl-CoA
CNI00240
4.2.1.74
trans-Hex-2-enoyl-CoA
trans-Oct-2-enoyl-CoA
trans-Dec-2-enoyl-CoA
trans-Dodec-2-enoyl-CoA
trans-Tetradec-2-enoyl-CoA
trans-Hexadec-2-enoyl-CoA
K07512
1.3.1.8
Hexanoyl-CoA
Octanoyl-CoA
Decanoyl-CoA
Dodecanoyl-CoA
Tetradecanoyl-CoA
Hexadecanoyl-CoA
CNA00890
Fatty acid degradation
Hexadecanoate
General forms
In mitochondria (4<n<16)
Acyl-CoA (4<n<16)
3-Oxoacyl-CoA
3-Hydroxyacyl-CoA
2,3-Dehydroacyl-CoA
Acyl-CoA (n+2)
Fatty acid degradation
Fatty acid (Hexadecanoate)
In endoplasmic reticulum (n≥16)
Malonyl-CoA
Long-chain acyl-CoA (n≥16)
K15397...
Long-chain 3-oxoacyl-CoA
CNI00690
Long-chain 3-hydroxyacyl-CoA
CNE00730
Long-chain trans-2,3-dehydroacyl-CoA
CNE01780
Long-chain acyl-CoA (n+2)
K01068...
Cutin, suberin and wax biosynthesis
Long chain fatty acid
00062 3/2/16
(c) Kanehisa Laboratories

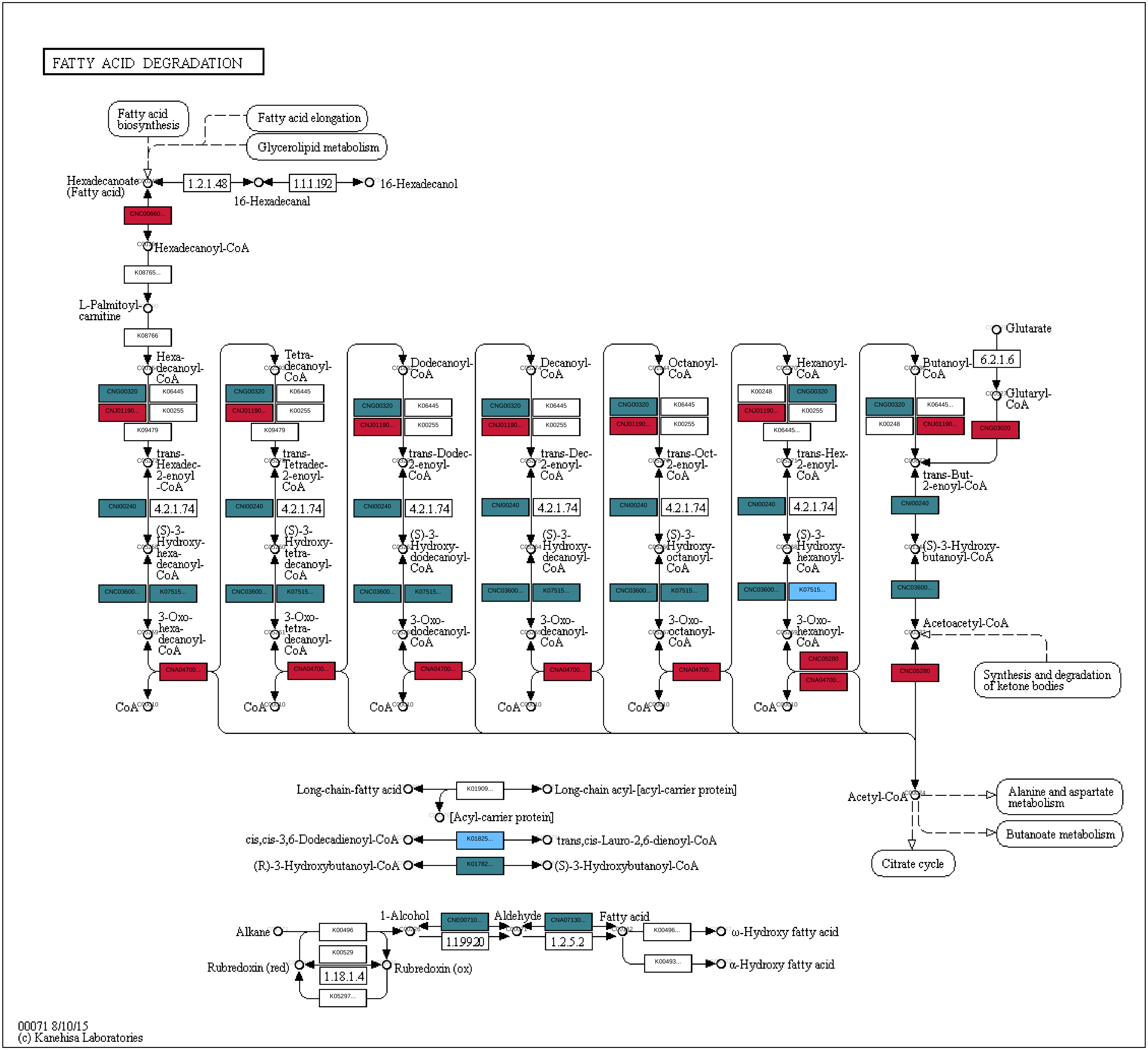
FATTY ACID DEGRADATION
Fatty acid biosynthesis
Fatty acid elongation
Glycerolipid metabolism
Hexadecanoate (Fatty acid)
1.2.1.48
1.1.1.192
16-Hexadecanal
16-Hexadecanol
CNC00660...
Hexadecanoyl-CoA
K08765...
L-Palmitoyl-carnitine
K08766
Hexa-decanoyl-CoA
Tetra-decanoyl-CoA
Dodecanoyl-CoA
Decanoyl-CoA
Octanoyl-CoA
Hexanoyl-CoA
Butanoyl-CoA
Glutarate
6.2.1.6
Glutaryl-CoA
CNG03020
CNG00320
K06445
CNJ01190...
K00255
K09479
K00248
K06445...
trans-Hexadec-2-enoyl-CoA
trans-Tetradec-2-enoyl-CoA
trans-Dodec-2-enoyl-CoA
trans-Dec-2-enoyl-CoA
trans-Oct-2-enoyl-CoA
trans-Hex-2-enoyl-CoA
trans-But-2-enoyl-CoA
CNI00240
4.2.1.74
(S)-3-Hydroxy-hexa-decanoyl-CoA
(S)-3-Hydroxy-tetra-decanoyl-CoA
(S)-3-Hydroxy-dodecanoyl-CoA
(S)-3-Hydroxy-decanoyl-CoA
(S)-3-Hydroxy-octanoyl-CoA
(S)-3-Hydroxy-hexanoyl-CoA
(S)-3-Hydroxy-butanoyl-CoA
CNC03600...
K07515...
3-Oxo-hexa-decanoyl-CoA
3-Oxo-tetra-decanoyl-CoA
3-Oxo-dodecanoyl-CoA
3-Oxo-decanoyl-CoA
3-Oxo-octanoyl-CoA
3-Oxo-hexanoyl-CoA
Acetoacetyl-CoA
CNA04700...
CNC05280
CoA
Synthesis and degradation of ketone bodies
Acetyl-CoA
Alanine and aspartate metabolism
Butanoate metabolism
Citrate cycle
Long-chain-fatty acid
K01909...
Long-chain acyl-[acyl-carrier protein]
[Acyl-carrier protein]
cis,cis-3,6-Dodecadienoyl-CoA
K01825...
trans,cis-Lauro-2,6-dienoyl-CoA
(R)-3-Hydroxybutanoyl-CoA
K01782...
(S)-3-Hydroxybutanoyl-CoA
Alkane
K00496
K00529
1.18.1.4
K05297...
Rubredoxin (red)
Rubredoxin (ox)
1-Alcohol
CNE00710...
1.1.99.20
Aldehyde
CNA07130...
1.2.5.2
Fatty acid
K00496...
K00493...
ω-Hydroxy fatty acid
α-Hydroxy fatty acid
00071 8/10/15
(c) Kanehisa Laboratories

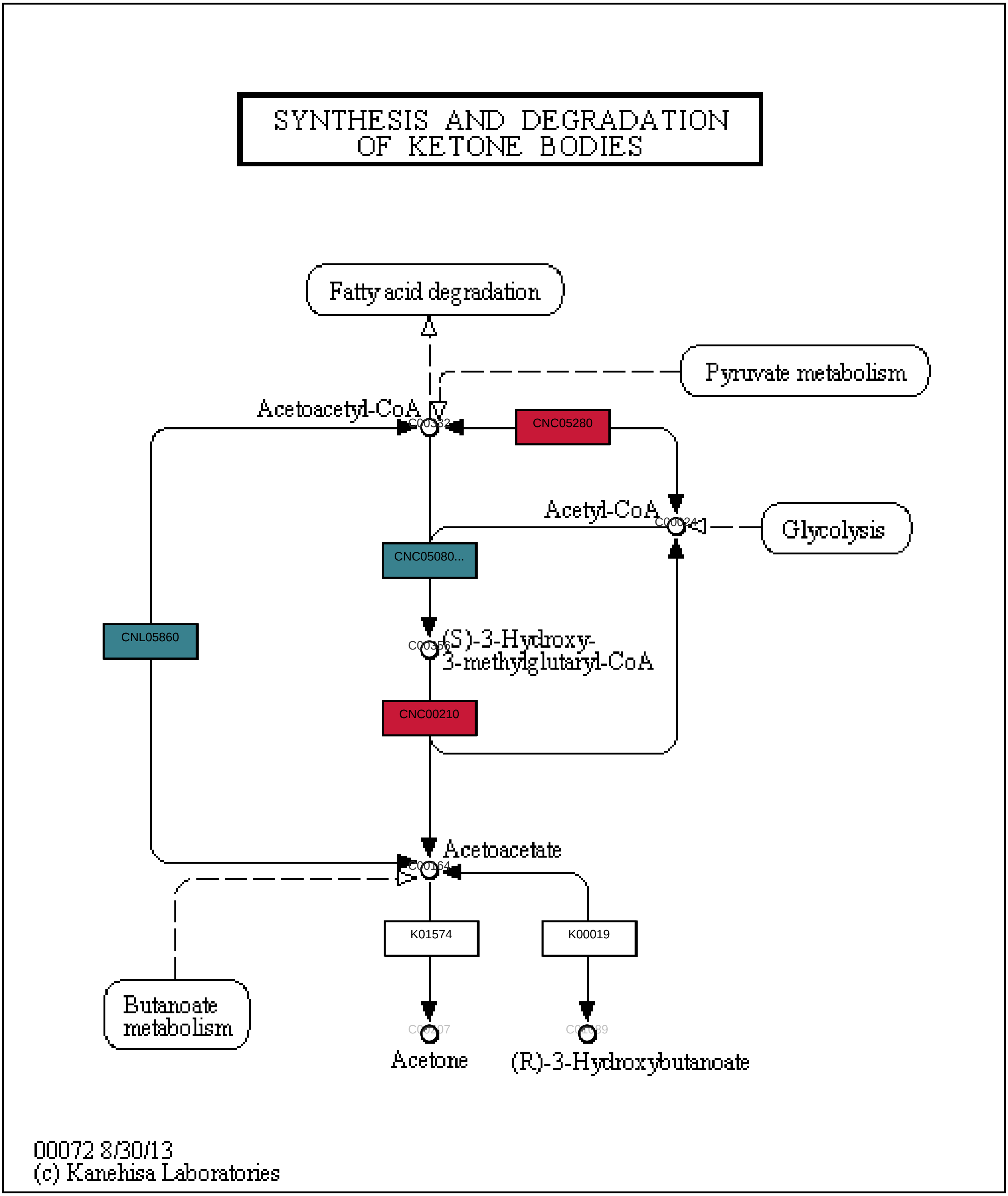
SYNTHESIS AND DEGRADATION
OF KETONE BODIES
Fatty acid degradation
Pyruvate metabolism
Acetoacetyl-CoA
C00332
CNC05280
Acetyl-CoA
C00024
Glycolysis
CNC05080...
CNL05860
C00356
(S)-3-Hydroxy-
3-methylglutaryl-CoA
CNC00210
Acetoacetate
C00164
K01574
K00019
Butanoate
metabolism
Acetone
(R)-3-Hydroxybutanoate
00072 8/30/13
(c) Kanehisa Laboratories

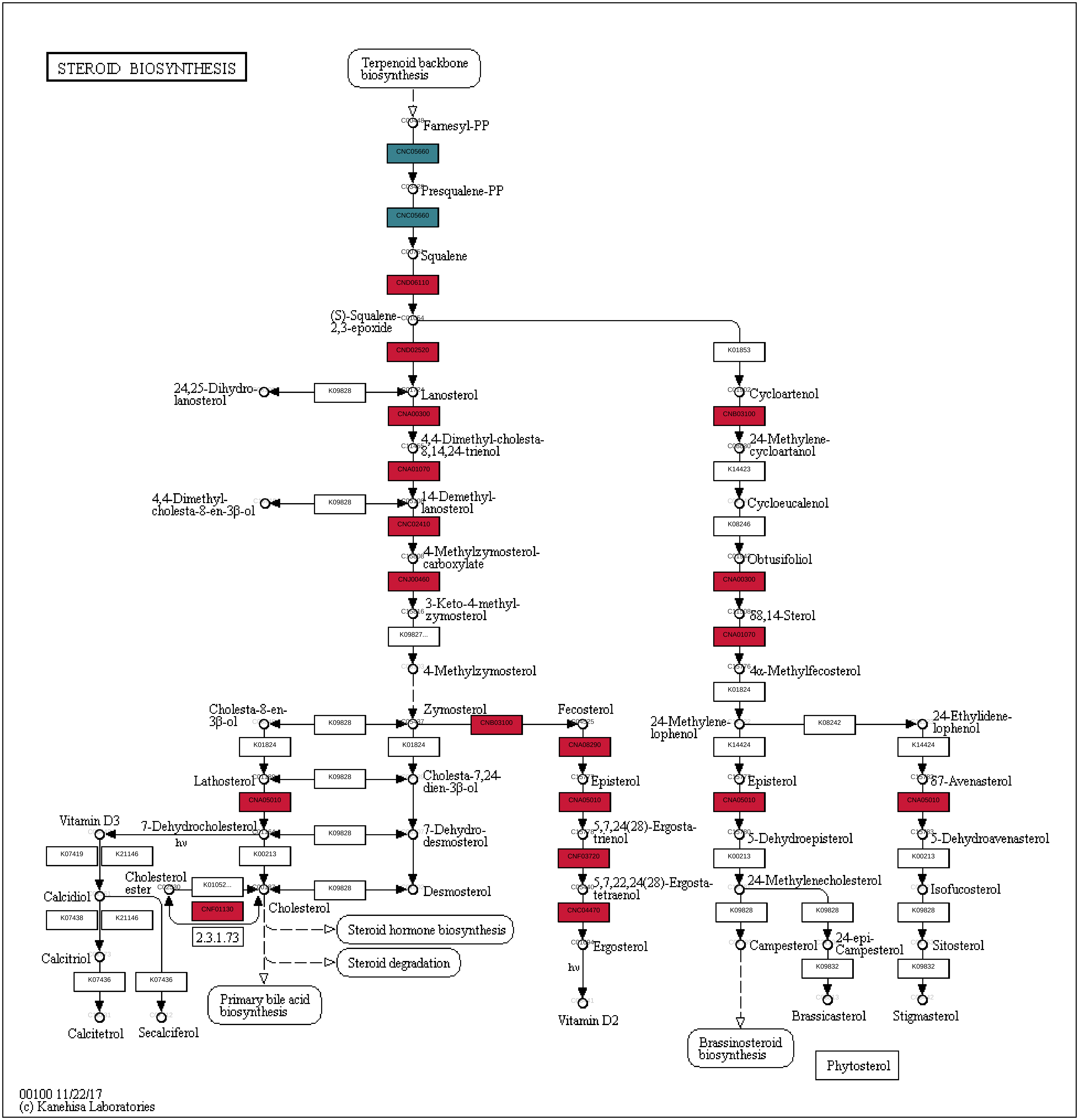
STEROID BIOSYNTHESIS
Terpenoid backbone biosynthesis
Farnesyl-PP
CNC05660
Presqualene-PP
CNC05660
Squalene
CND06110
(S)-Squalene-2,3-epoxide
CND02520
K01853
24,25-Dihydro-lanosterol
K09828
Lanosterol
Cycloartenol
CNA00300
CNB03100
4,4-Dimethyl-cholesta-8,14,24-trienol
24-Methylene-cycloartanol
CNA01070
K14423
4,4-Dimethyl-cholesta-8-en-3β-ol
K09828
14-Demethyl-lanosterol
Cycloeucalenol
CNC02410
K08246
4-Methylzymosterol-carboxylate
Obtusifoliol
CNJ00460
CNA00300
3-Keto-4-methyl-zymosterol
δ8,14-Sterol
K09827...
CNA01070
4-Methylzymosterol
4α-Methylfecosterol
K01824
Cholesta-8-en-3β-ol
K09828
Zymosterol
CNB03100
Fecosterol
24-Methylene-lophenol
K08242
24-Ethylidene-lophenol
K01824
K01824
CNA08290
K14424
K14424
Lathosterol
K09828
Cholesta-7,24-dien-3β-ol
Episterol
Episterol
δ7-Avenasterol
CNA05010
CNA05010
CNA05010
CNA05010
Vitamin D3
7-Dehydrocholesterol
K09828
7-Dehydro-desmosterol
5,7,24(28)-Ergosta-trienol
5-Dehydroepisterol
5-Dehydroavenasterol
hν
K07419
K21146
K00213
CNF03720
K00213
K00213
Cholesterol ester
K01052...
K09828
Desmosterol
5,7,22,24(28)-Ergosta-tetraenol
24-Methylenecholesterol
Isofucosterol
Calcidiol
CNF01130
Cholesterol
CNC04470
K09828
K09828
K09828
K07438
K21146
2.3.1.73
Steroid hormone biosynthesis
Campesterol
24-epi-Campesterol
Sitosterol
Calcitriol
Steroid degradation
Ergosterol
K07436
K07436
hν
K09832
K09832
Primary bile acid biosynthesis
Calcitetrol
Secalciferol
Vitamin D2
Brassicasterol
Stigmasterol
Brassinosteroid biosynthesis
Phytosterol
00100 11/22/17
(c) Kanehisa Laboratories

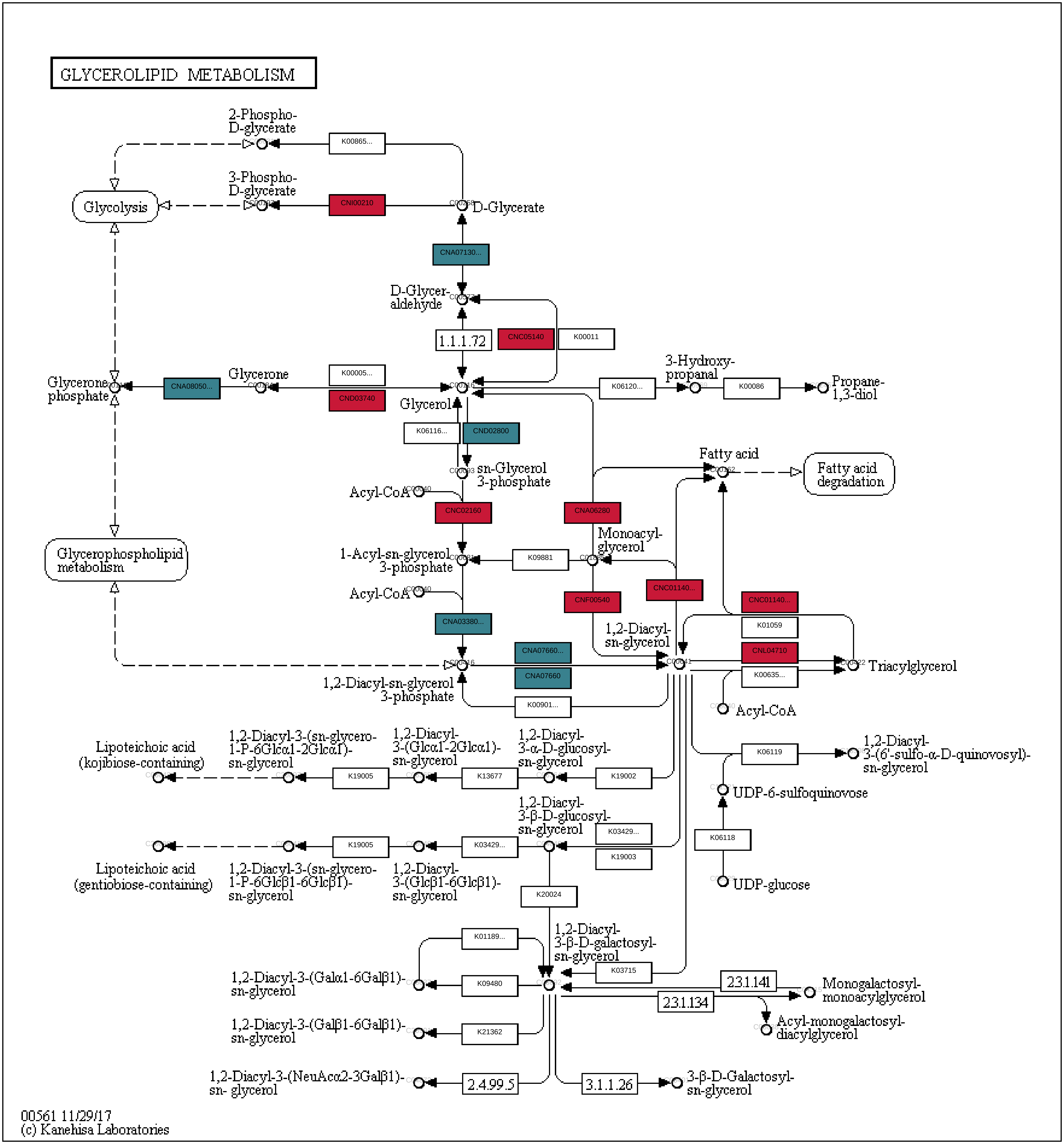
GLYCEROLIPID METABOLISM
2-Phospho-D-glycerate
K00865...
3-Phospho-D-glycerate
CNI00210
D-Glycerate
Glycolysis
CNA07130...
D-Glyceraldehyde
1.1.1.72
CNC05140
K00011
Glycerone phosphate
CNA08050...
Glycerone
K00005...
CND03740
Glycerol
K06120...
3-Hydroxypropanal
K00086
Propane-1,3-diol
K06116...
CND02800
sn-Glycerol 3-phosphate
Fatty acid
Fatty acid degradation
Acyl-CoA
CNC02160
CNA06280
Glycerophospholipid metabolism
Monoacylglycerol
1-Acyl-sn-glycerol 3-phosphate
K09881
CNC01140...
CNF00540
CNA03380...
1,2-Diacyl-sn-glycerol
K01059
CNL04710
CNA07660...
CNA07660
Triacylglycerol
K00635...
1,2-Diacyl-sn-glycerol 3-phosphate
K00901...
Lipoteichoic acid (kojibiose-containing)
1,2-Diacyl-3-(sn-glycero-1-P-6Glcα1-2Glcα1)-sn-glycerol
1,2-Diacyl-3-(Glcα1-2Glcα1)-sn-glycerol
1,2-Diacyl-3-α-D-glucosyl-sn-glycerol
K19005
K13677
K19002
K06119
1,2-Diacyl-3-(6'-sulfo-α-D-quinovosyl)-sn-glycerol
UDP-6-sulfoquinovose
1,2-Diacyl-3-β-D-glucosyl-sn-glycerol
K03429...
K19003
K06118
Lipoteichoic acid (gentiobiose-containing)
1,2-Diacyl-3-(sn-glycero-1-P-6Glcβ1-6Glcβ1)-sn-glycerol
1,2-Diacyl-3-(Glcβ1-6Glcβ1)-sn-glycerol
UDP-glucose
K20024
K01189...
1,2-Diacyl-3-β-D-galactosyl-sn-glycerol
K03715
1,2-Diacyl-3-(Galα1-6Galβ1)-sn-glycerol
K09480
2.3.1.141
Monogalactosylmonoacylglycerol
2.3.1.134
Acyl-monogalactosyl-diacylglycerol
1,2-Diacyl-3-(Galβ1-6Galβ1)-sn-glycerol
K21362
1,2-Diacyl-3-(NeuAcα2-3Galβ1)-sn- glycerol
2.4.99.5
3.1.1.26
3-β-D-Galactosyl-sn-glycerol
00561 11/29/17
(c) Kanehisa Laboratories

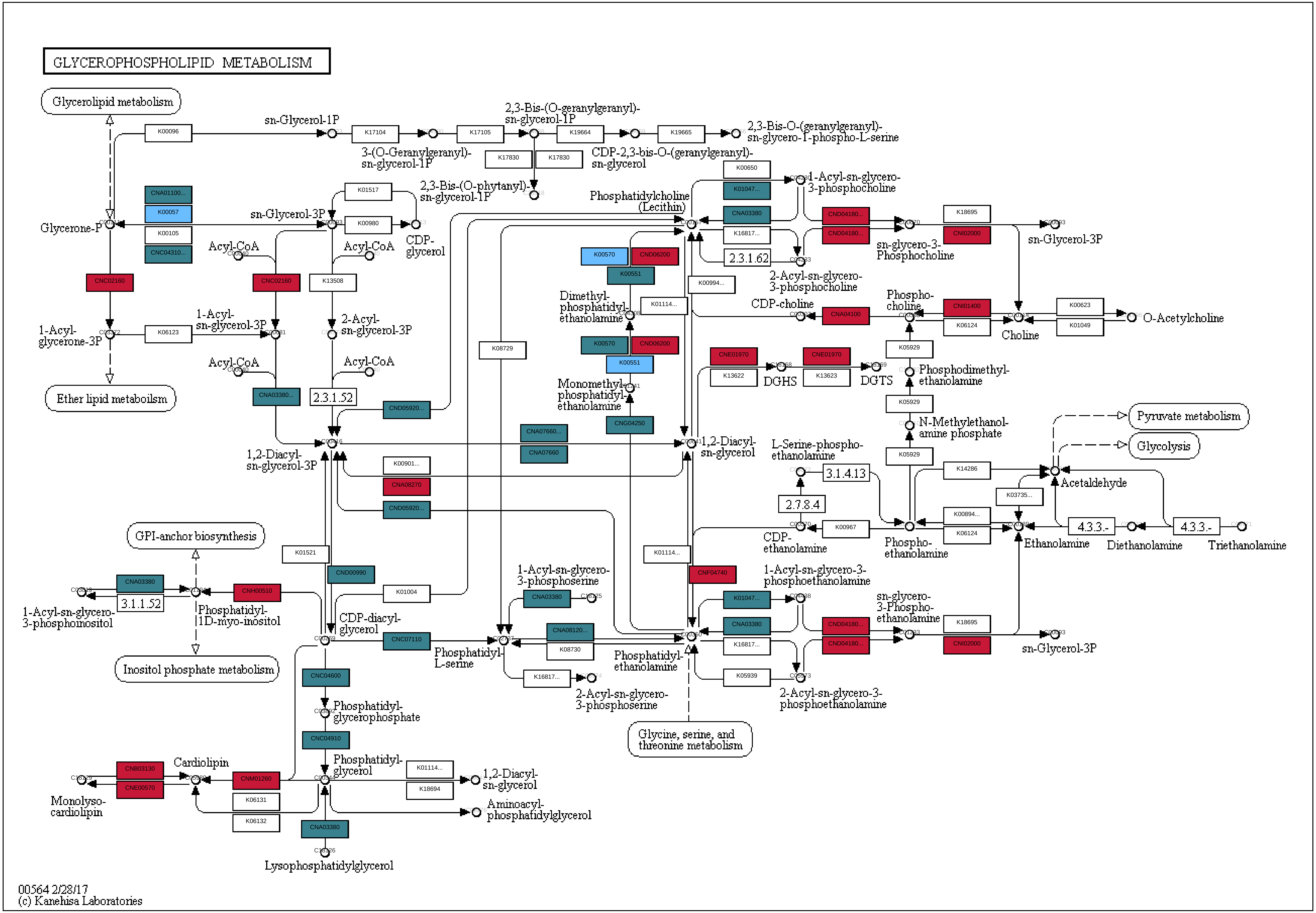
GLYCEROPHOSPHOLIPID METABOLISM
Glycerolipid metabolism
sn-Glycerol-1P
2,3-Bis-(O-geranylgeranyl)-sn-glycerol-1P
2,3-Bis-O-(geranylgeranyl)-sn-glycero-1-phospho-L-serine
3-(O-Geranylgeranyl)-sn-glycerol-1P
CDP-2,3-bis-O-(geranylgeranyl)-sn-glycerol
2,3-Bis-(O-phytanyl)-sn-glycerol-1P
K00096
K17104
K17105
K19664
K19665
K17830
K17830
Glycerone-P
CNA01100...
K00057
K00105
CNC04310...
sn-Glycerol-3P
K01517
K00980
CDP-glycerol
Acyl-CoA
Acyl-CoA
CNC02160
CNC02160
K13508
1-Acyl-glycerone-3P
K06123
1-Acyl-sn-glycerol-3P
2-Acyl-sn-glycerol-3P
Acyl-CoA
Acyl-CoA
Ether lipid metaboilsm
CNA03380...
2.3.1.52
CND05920...
1,2-Diacyl-sn-glycerol-3P
K00901...
CNA08270
CND05920...
Phosphatidylcholine (Lecithin)
K00650
K01047...
CNA03380
K16817...
2.3.1.62
K00994...
1-Acyl-sn-glycero-3-phosphocholine
2-Acyl-sn-glycero-3-phosphocholine
CND04180...
CND04180...
K18695
CNI02000
sn-Glycerol-3P
sn-glycero-3-Phosphocholine
K00570
CND06200
K00551
Dimethyl-phosphatidyl-ethanolamine
K01114...
CDP-choline
CNA04100
Phospho-choline
CNI01400
K06124
Choline
K00623
K01049
O-Acetylcholine
K08729
K00570
CND06200
K00551
Monomethyl-phosphatidyl-ethanolamine
CNG04250
CNA07660...
CNA07660
1,2-Diacyl-sn-glycerol
CNE01970
K13622
DGHS
CNE01970
K13623
DGTS
K05929
Phosphodimethyl-ethanolamine
K05929
N-Methylethanol-amine phosphate
K05929
Pyruvate metabolism
Glycolysis
L-Serine-phospho-ethanolamine
3.1.4.13
2.7.8.4
K14286
Acetaldehyde
K03735...
K00894...
K06124
CDP-ethanolamine
K00967
Phospho-ethanolamine
Ethanolamine
4.3.3.-
Diethanolamine
4.3.3.-
Triethanolamine
GPI-anchor biosynthesis
K01521
CND00990
K01004
K01114...
CNF04740
CNA03380
CNH00510
3.1.1.52
1-Acyl-sn-glycero-3-phosphoinositol
Phosphatidyl-1D-myo-inositol
Inositol phosphate metabolism
CDP-diacyl-glycerol
CNC07110
1-Acyl-sn-glycero-3-phosphoserine
CNA03380
CNA08120...
K08730
Phosphatidyl-L-serine
K16817...
2-Acyl-sn-glycero-3-phosphoserine
Phosphatidyl-ethanolamine
1-Acyl-sn-glycero-3-phosphoethanolamine
K01047...
CNA03380
K16817...
K05939
sn-glycero-3-Phospho-ethanolamine
CND04180...
CND04180...
K18695
CNI02000
sn-Glycerol-3P
2-Acyl-sn-glycero-3-phosphoethanolamine
Glycine, serine, and threonine metabolism
CNC04600
Phosphatidyl-glycerophosphate
CNC04910
Phosphatidyl-glycerol
K01114...
K18694
1,2-Diacyl-sn-glycerol
Aminoacyl-phosphatidylglycerol
Cardiolipin
CNB03130
CNE00570
CNM01260
K06131
K06132
Monolyso-cardiolipin
CNA03380
Lysophosphatidylglycerol
00564 2/28/17
(c) Kanehisa Laboratories

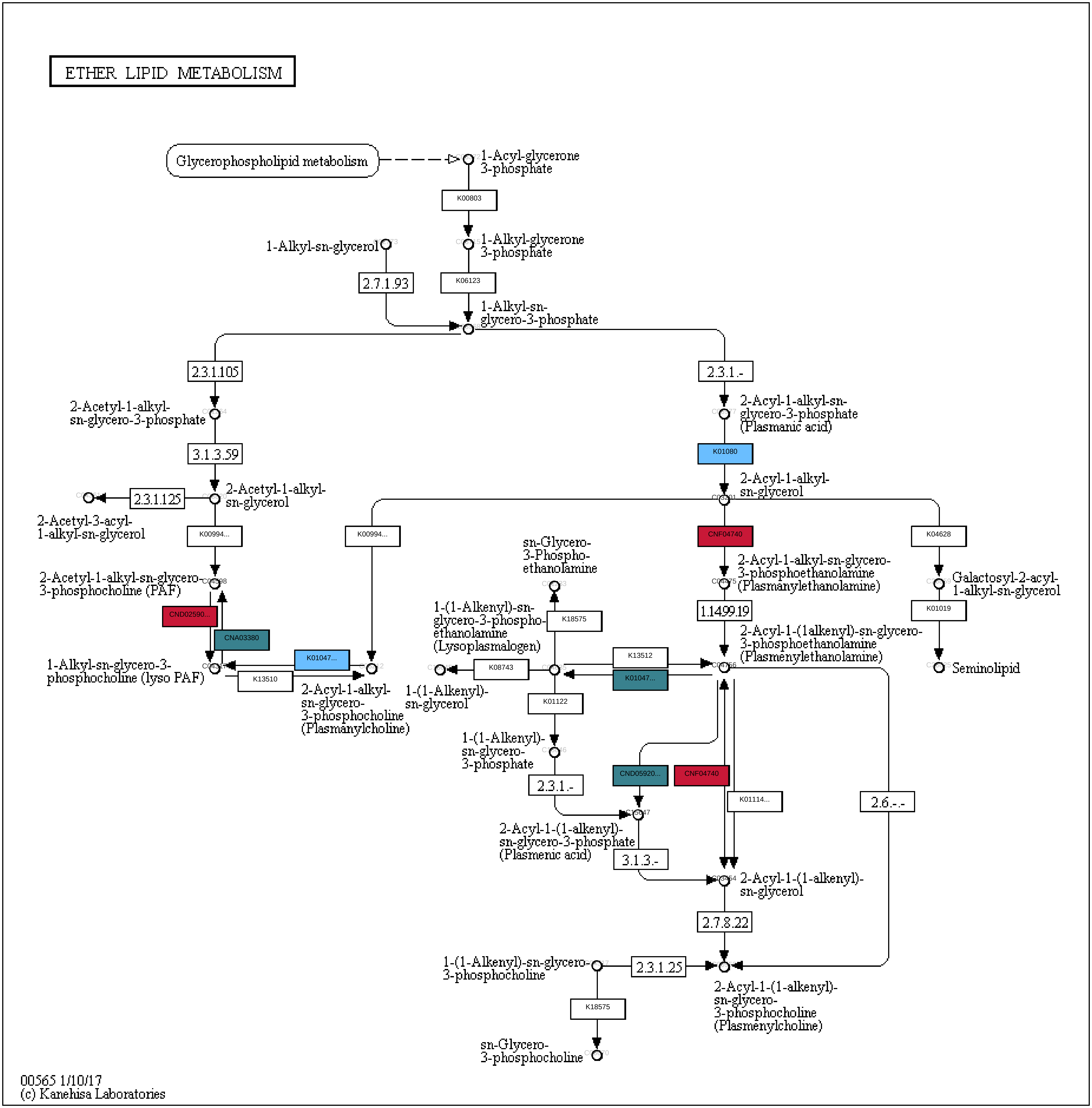
ETHER LIPID METABOLISM
Glycerophospholipid metabolism
1-Acyl-glycerone 3-phosphate
K00803
1-Alkyl-sn-glycerol
1-Alkyl-glycerone 3-phosphate
2.7.1.93
K06123
1-Alkyl-sn-glycero-3-phosphate
2.3.1.105
2.3.1.-
2-Acetyl-1-alkyl-sn-glycero-3-phosphate
2-Acyl-1-alkyl-sn-glycero-3-phosphate (Plasmanic acid)
3.1.3.59
K01080
2.3.1.125
2-Acetyl-1-alkyl-sn-glycerol
2-Acyl-1-alkyl-sn-glycerol
2-Acetyl-3-acyl-1-alkyl-sn-glycerol
K00994...
K00994...
CNF04740
K04628
sn-Glycero-3-Phospho-ethanolamine
2-Acyl-1-alkyl-sn-glycero-3-phosphoethanolamine (Plasmanylethanolamine)
2-Acetyl-1-alkyl-sn-glycero-3-phosphocholine (PAF)
Galactosyl-2-acyl-1-alkyl-sn-glycerol
CND02590...
K18575
1-(1-Alkenyl)-sn-glycero-3-phospho-ethanolamine (Lysoplasmalogen)
1.14.99.19
K01019
CNA03380
2-Acyl-1-(1alkenyl)-sn-glycero-3-phosphoethanolamine (Plasmenylethanolamine)
K01047...
K13512
1-Alkyl-sn-glycero-3-phosphocholine (lyso PAF)
K08743
K01047...
Seminolipid
K13510
2-Acyl-1-alkyl-sn-glycero-3-phosphocholine (Plasmanylcholine)
1-(1-Alkenyl)-sn-glycerol
K01122
1-(1-Alkenyl)-sn-glycero-3-phosphate
2.3.1.-
CND05920...
CNF04740
K01114...
2.6.-.-
2-Acyl-1-(1-alkenyl)-sn-glycero-3-phosphate (Plasmenic acid)
3.1.3.-
2-Acyl-1-(1-alkenyl)-sn-glycerol
2.7.8.22
1-(1-Alkenyl)-sn-glycero-3-phosphocholine
2.3.1.25
2-Acyl-1-(1-alkenyl)-sn-glycero-3-phosphocholine (Plasmenylcholine)
K18575
sn-Glycero-3-phosphocholine
00565 1/10/17
(c) Kanehisa Laboratories

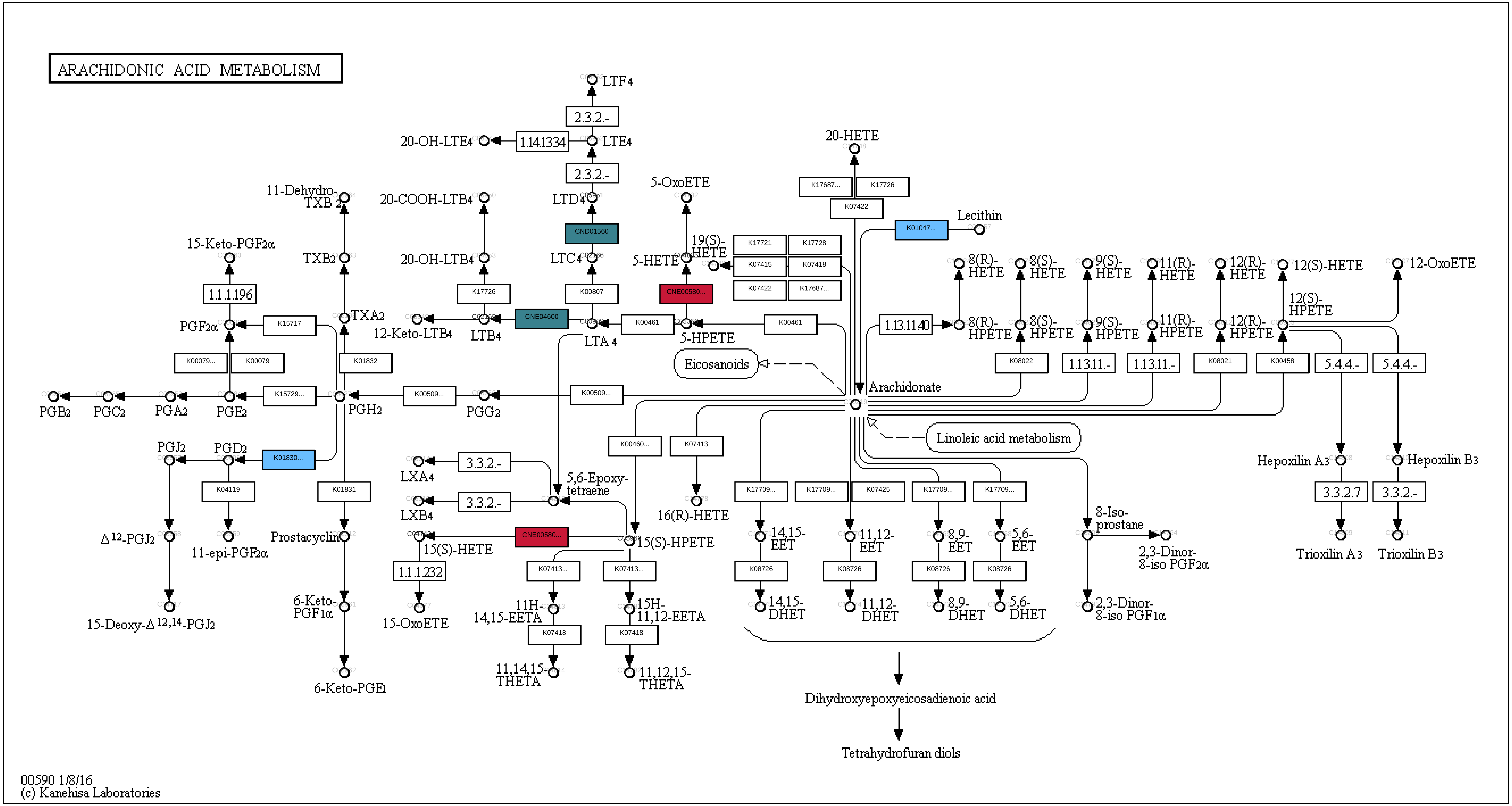
ARACHIDONIC ACID METABOLISM
LTF4
2.3.2.-
20-OH-LTE4
1.14.13.34
LTE4
2.3.2.-
20-HETE
K17687...
K17726
K07422
5-OxoETE
11-Dehydro-TXB 2
20-COOH-LTB4
LTD4
CND01560
Lecithin
K01047...
19(S)-HETE
K17721
K17728
K07415
K07418
K07422
K17687...
15-Keto-PGF2α
TXB2
20-OH-LTB4
LTC4
5-HETE
8(R)-HETE
8(S)-HETE
9(S)-HETE
11(R)-HETE
12(R)-HETE
12(S)-HETE
12-OxoETE
1.1.1.196
K17726
K00807
CNE00580...
12(S)-HPETE
TXA2
CNE04600
12-Keto-LTB4
LTB4
K00461
K00461
1.13.11.40
8(R)-HPETE
8(S)-HPETE
9(S)-HPETE
11(R)-HPETE
12(R)-HPETE
PGF2α
K15717
LTA 4
5-HPETE
K00079...
K00079
K01832
Eicosanoids
K08022
1.13.11.-
1.13.11.-
K08021
K00458
5.4.4.-
5.4.4.-
K15729...
K00509...
K00509...
Arachidonate
PGB2
PGC2
PGA2
PGE2
PGH2
PGG2
Linoleic acid metabolism
PGJ2
PGD2
K01830...
K00460...
K07413
3.3.2.-
LXA4
5,6-Epoxy-tetraene
Hepoxilin A3
Hepoxilin B3
K04119
K01831
K17709...
K17709...
K07425
K17709...
K17709...
3.3.2.7
3.3.2.-
3.3.2.-
LXB4
16(R)-HETE
8-Iso-prostane
Δ12-PGJ2
11-epi-PGF2α
Prostacyclin
CNE00580...
15(S)-HPETE
14,15-EET
11,12-EET
8,9-EET
5,6-EET
2,3-Dinor-8-iso PGF2α
Trioxilin A3
Trioxilin B3
15(S)-HETE
1.1.1.232
K07413...
K07413...
K08726
K08726
K08726
K08726
6-Keto-PGF1α
11H-14,15-EETA
15H-11,12-EETA
14,15-DHET
11,12-DHET
8,9-DHET
5,6-DHET
2,3-Dinor-8-iso PGF1α
15-Deoxy-Δ12,14-PGJ2
15-OxoETE
K07418
K07418
11,14,15-THETA
11,12,15-THETA
6-Keto-PGE1
Dihydroxyepoxyeicosadienoic acid
Tetrahydrofuran diols
00590 1/8/16
(c) Kanehisa Laboratories

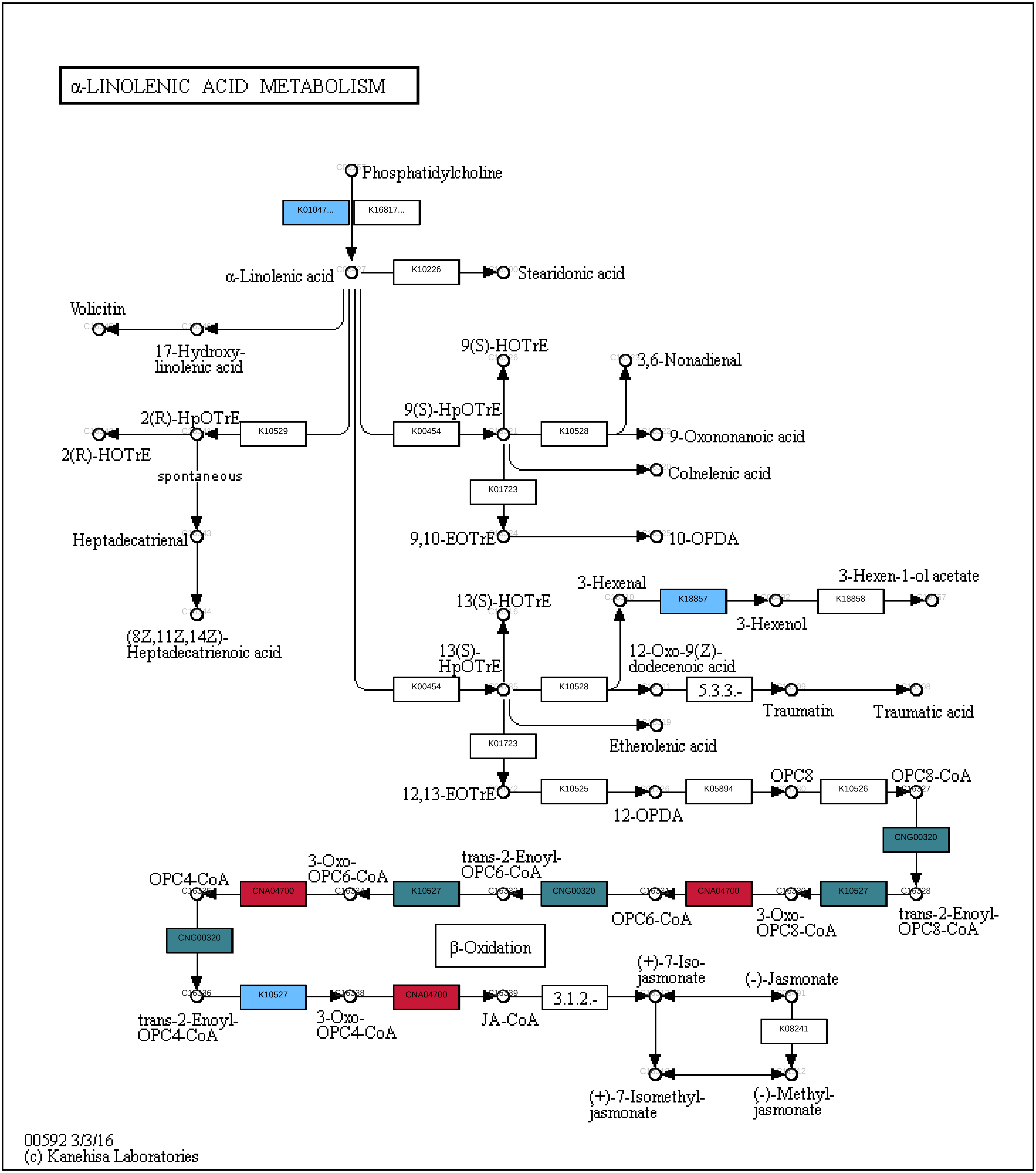
α-LINOLENIC ACID METABOLISM
Phosphatidylcholine
K01047...
K16817...
α-Linolenic acid
K10226
Stearidonic acid
Volicitin
17-Hydroxy-linolenic acid
2(R)-HpOTrE
K10529
2(R)-HOTrE
spontaneous
Heptadecatrienal
(8Z,11Z,14Z)-Heptadecatrienoic acid
9(S)-HOTrE
3,6-Nonadienal
9(S)-HpOTrE
K00454
K10528
9-Oxononanoic acid
Colnelenic acid
K01723
9,10-EOTrE
10-OPDA
3-Hexenal
K18857
K18858
3-Hexen-1-ol acetate
3-Hexenol
13(S)-HOTrE
13(S)-HpOTrE
K00454
K10528
12-Oxo-9(Z)-dodecenoic acid
5.3.3.-
Traumatin
Traumatic acid
Etherolenic acid
K01723
12,13-EOTrE
K10525
12-OPDA
K05894
OPC8
K10526
OPC8-CoA
CNG00320
trans-2-Enoyl-OPC8-CoA
K10527
3-Oxo-OPC8-CoA
CNA04700
OPC6-CoA
CNG00320
trans-2-Enoyl-OPC6-CoA
K10527
3-Oxo-OPC6-CoA
CNA04700
OPC4-CoA
C16335
CNG00320
β-Oxidation
C16336
trans-2-Enoyl-OPC4-CoA
K10527
C16338
3-Oxo-OPC4-CoA
CNA04700
C16339
JA-CoA
3.1.2.-
(+)-7-Iso-jasmonate
(-)-Jasmonate
K08241
(+)-7-Isomethyl-jasmonate
(-)-Methyl-jasmonate
00592 3/3/16
(c) Kanehisa Laboratories

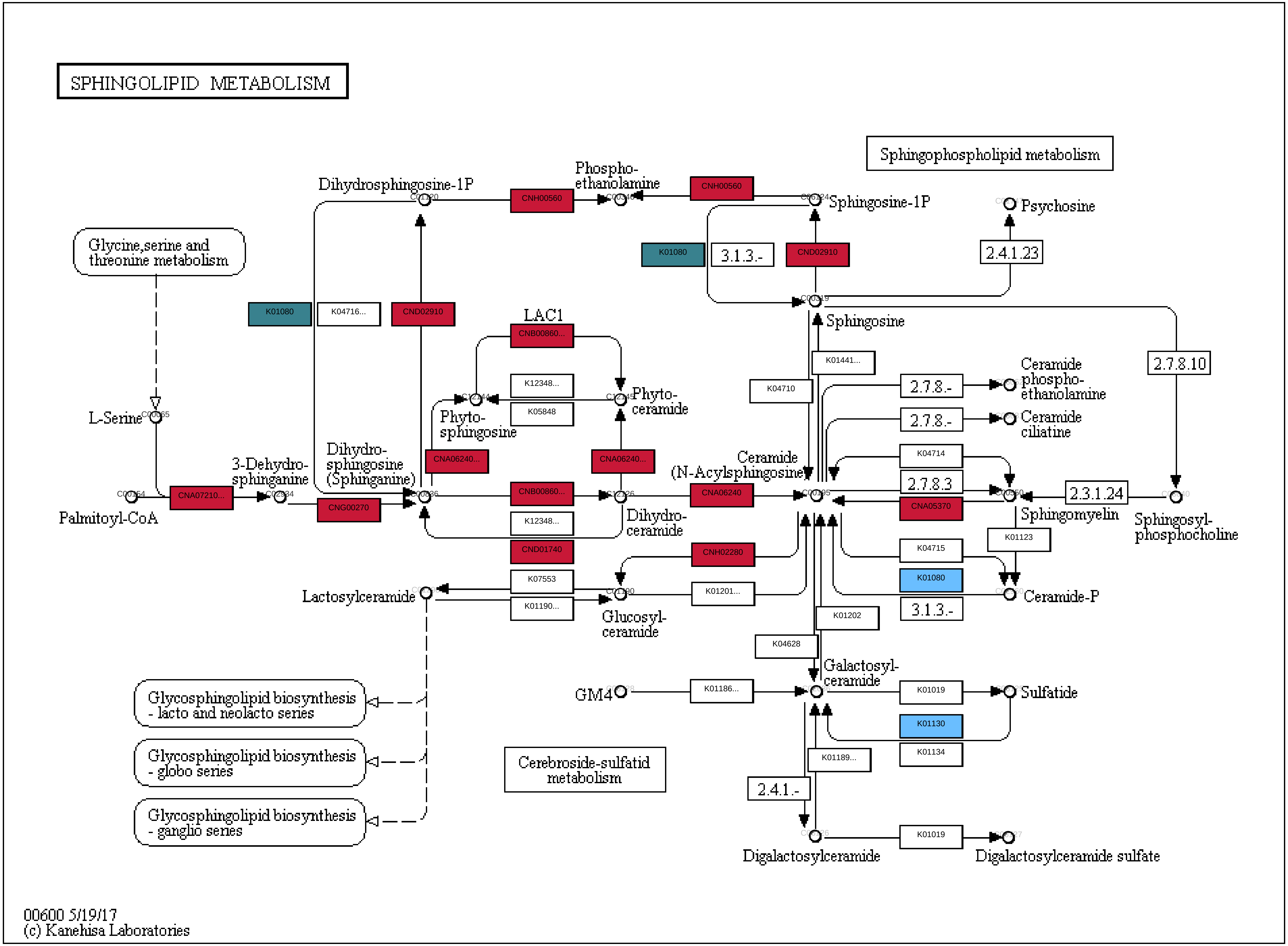

SPHINGOLIPID METABOLISM
Sphingophospholipid metabolism
Glycine,serine and threonine metabolism
Dihydrosphingosine-1P
Phospho-ethanolamine
Sphingosine-1P
Psychosine
CNH00560
CNH00560
K01080
3.1.3.-
CND02910
2.4.1.23
K01080
K04716...
CND02910
LAC1
CNB00860...
Sphingosine
K01441...
2.7.8.10
K12348...
K05848
K04710
2.7.8.-
Ceramide phospho-ethanolamine
2.7.8.-
Ceramide ciliatine
Phyto-ceramide
Phyto-sphingosine
L-Serine
3-Dehydro-sphinganine
Dihydro-sphingosine (Sphinganine)
CNA06240...
CNA06240...
Ceramide (N-Acylsphingosine)
K04714
2.7.8.3
2.3.1.24
Palmitoyl-CoA
CNA07210...
CNG00270
CNB00860...
K12348...
CNA06240
CNA05370
Sphingomyelin
Sphingosyl-phosphocholine
Dihydro-ceramide
CND01740
CNH02280
K04715
K01123
K07553
K01080
Lactosylceramide
K01190...
K01201...
Ceramide-P
Glucosyl-ceramide
K01202
3.1.3.-
K04628
Galactosyl-ceramide
GM4
K01186...
K01019
Sulfatide
K01130
Glycosphingolipid biosynthesis - lacto and neolacto series
Glycosphingolipid biosynthesis - globo series
Cerebroside-sulfatid metabolism
K01189...
K01134
2.4.1.-
Glycosphingolipid biosynthesis - ganglio series
K01019
Digalactosylceramide
Digalactosylceramide sulfate
00600 5/19/17
(c) Kanehisa Laboratories

# 4. Nucleotide metabolism

| MAP | PATHWAY |
|---|---|
| **230** | Purine metabolism |
| **240** | Pyrimidine metabolism |

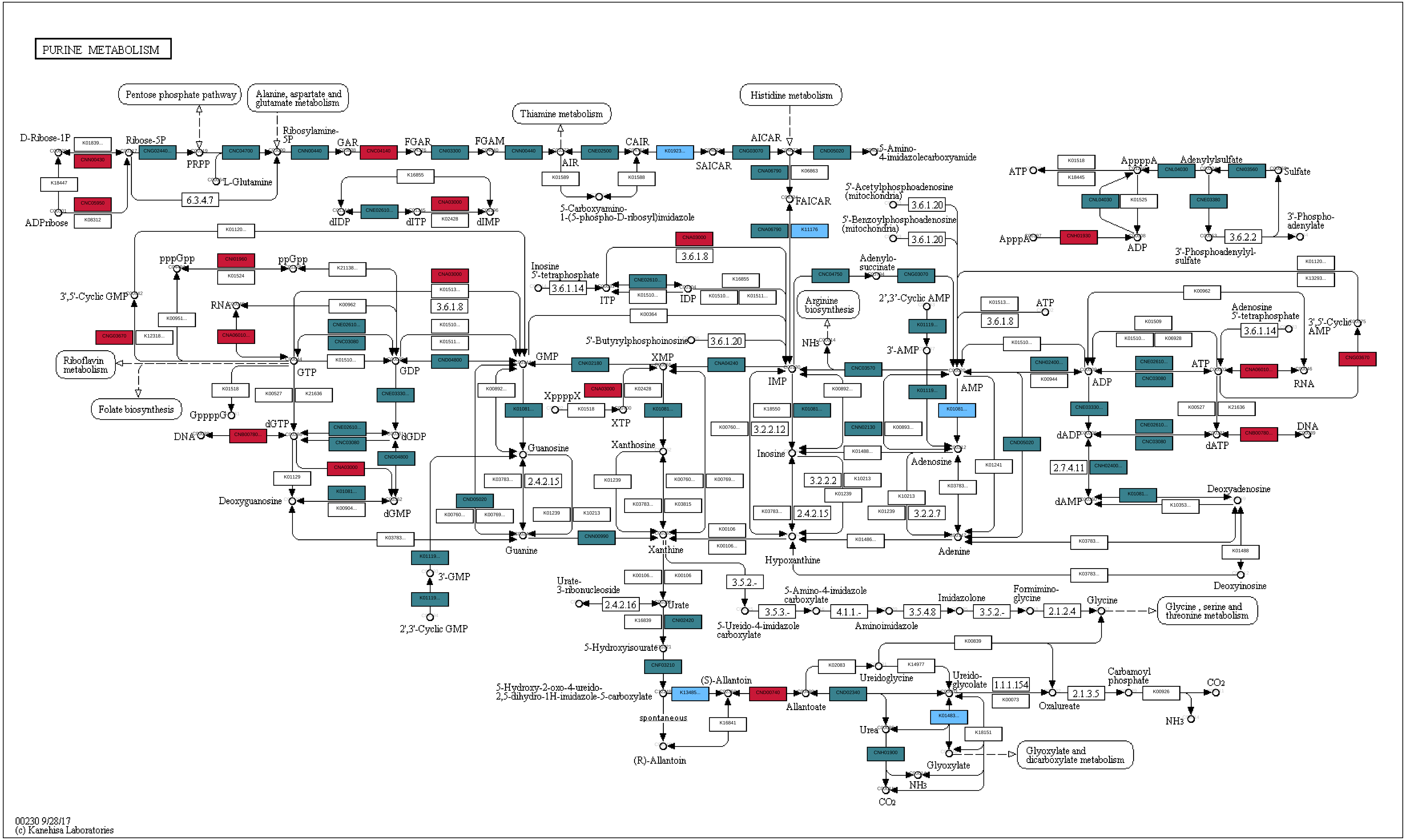
PURINE METABOLISM
Pentose phosphate pathway
Alanine, aspartate and glutamate metabolism
Thiamine metabolism
Histidine metabolism
D-Ribose-1P
Ribose-5P
PRPP
Ribosylamine-5P
L-Glutamine
GAR
FGAR
FGAM
AIR
CAIR
SAICAR
AICAR
5-Amino-4-imidazolecarboxyamide
FAICAR
5-Carboxyamino-1-(5-phospho-D-ribosyl)imidazole
ADPribose
6.3.4.7
dIDP
dITP
dIMP
3',5'-Cyclic GMP
pppGpp
ppGpp
RNA
Riboflavin metabolism
Folate biosynthesis
GTP
GDP
GMP
GppppG
dGTP
dGDP
dGMP
DNA
Deoxyguanosine
Guanosine
Guanine
3'-GMP
2',3'-Cyclic GMP
Inosine 5'-tetraphosphate
3.6.1.14
ITP
IDP
3.6.1.8
5'-Butyrylphosphoinosine
3.6.1.20
XMP
XppppX
XTP
Xanthosine
Xanthine
2.4.2.15
IMP
Inosine
Hypoxanthine
3.2.2.12
3.2.2.2
3.2.2.7
Arginine biosynthesis
NH3
Adenylo-succinate
2',3'-Cyclic AMP
3'-AMP
AMP
Adenosine
Adenine
5'-Acetylphosphoadenosine (mitochondria)
5'-Benzoylphosphoadenosine (mitochondria)
ATP
ApppA
AppppA
ADP
Adenylylsulfate
Sulfate
3'-Phospho-adenylate
3'-Phosphoadenylyl-sulfate
3.6.2.2
Adenosine 5'-tetraphosphate
3',5'-Cyclic AMP
dADP
dATP
dAMP
2.7.4.11
Deoxyadenosine
Deoxyinosine
Urate-3-ribonucleoside
2.4.2.16
Urate
5-Hydroxyisourate
5-Hydroxy-2-oxo-4-ureido-2,5-dihydro-1H-imidazole-5-carboxylate
spontaneous
(S)-Allantoin
(R)-Allantoin
Allantoate
Ureidoglycine
Ureido-glycolate
1.1.1.154
Urea
Glyoxylate
Glyoxylate and dicarboxylate metabolism
CO2
Oxalureate
2.1.3.5
Carbamoyl phosphate
3.5.2.-
5-Ureido-4-imidazole carboxylate
3.5.3.-
5-Amino-4-imidazole carboxylate
4.1.1.-
Aminoimidazole
3.5.4.8
Imidazolone
Formimino-glycine
2.1.2.4
Glycine
Glycine, serine and threonine metabolism
00230 9/28/17
(c) Kanehisa Laboratories

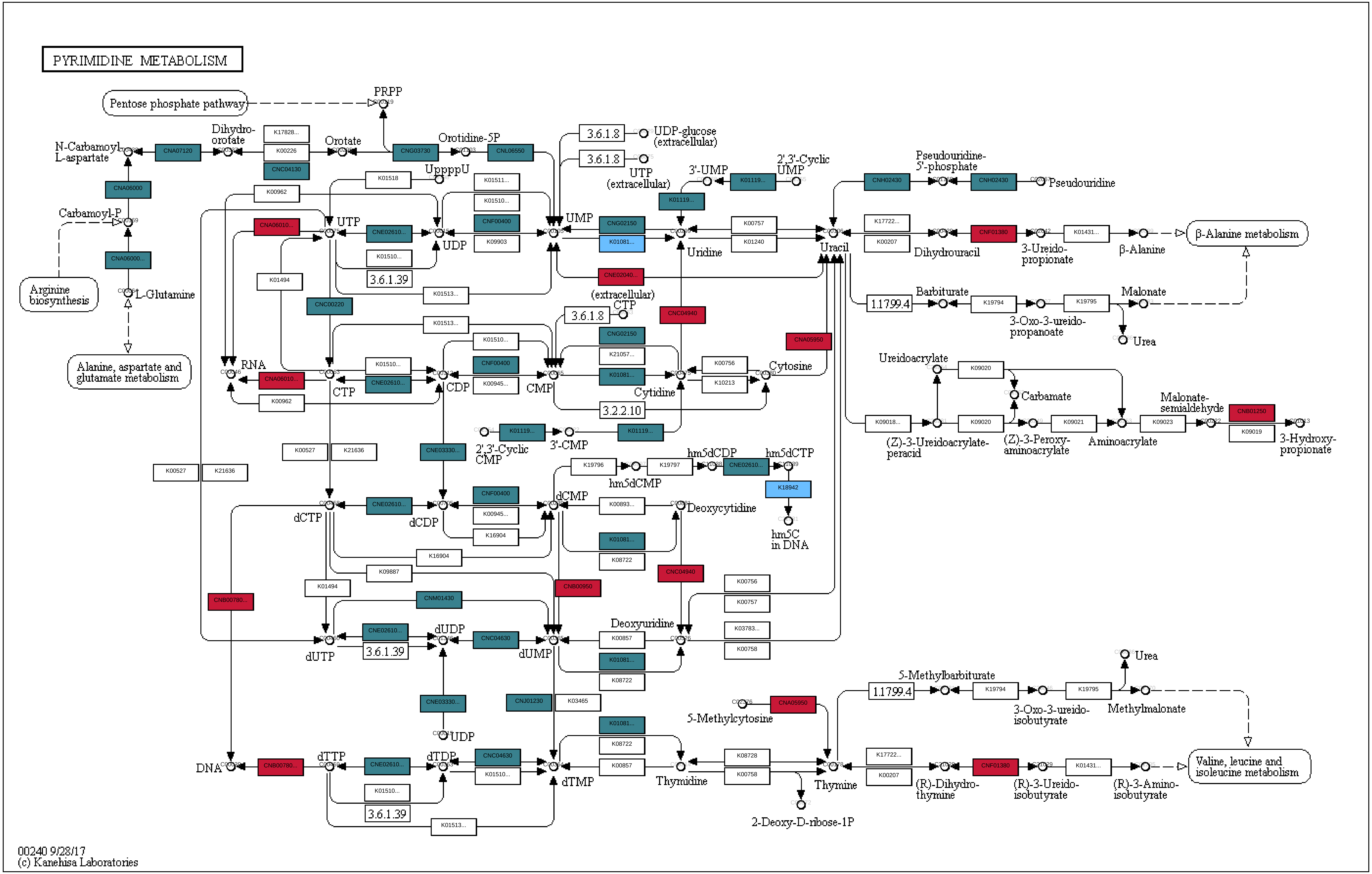
PYRIMIDINE METABOLISM
Pentose phosphate pathway
PRPP
N-Carbamoyl-L-aspartate
CNA07120
Dihydro-orotate
K17828...
K00226
CNC04130
Orotate
CNG03730
Orotidine-5P
CNL06550
CNA06000
Carbamoyl-P
CNA06000...
L-Glutamine
Arginine biosynthesis
Alanine, aspartate and glutamate metabolism
K01518
UppppU
K00962
K01511...
K01510...
CNF00400
K09903
CNA06010...
UTP
CNE02610...
UDP
K01510...
3.6.1.39
K01494
K01513...
CNC00220
UMP
3.6.1.8
UDP-glucose (extracellular)
3.6.1.8
UTP (extracellular)
3'-UMP
K01119...
2',3'-Cyclic UMP
CNK01119...
CNG02150
K01081...
CNE02040...
(extracellular) CTP
3.6.1.8
K00757
K01240
Uridine
Uracil
Pseudouridine-5'-phosphate
CNH02430
CNH02430
Pseudouridine
K17722...
K00207
CNF01380
K01431...
Dihydrouracil
3-Ureido-propionate
β-Alanine
β-Alanine metabolism
CNC04940
CNA05950
1.17.99.4
Barbiturate
K19794
K19795
Malonate
3-Oxo-3-ureido-propanoate
Urea
K01513...
K01510...
CNG02150
K21057...
K00756
K10213
Cytosine
RNA
CNA06010...
K01510...
CNE02610...
CNF00400
K00945...
CTP
CDP
CMP
K01081...
Cytidine
K00962
3.2.2.10
Ureidoacrylate
K09020
Carbamate
K09018...
K09020
K09021
K09023
CNB01250
K09019
Malonate-semialdehyde
(Z)-3-Ureidoacrylate-peracid
(Z)-3-Peroxy-aminoacrylate
Aminoacrylate
3-Hydroxy-propionate
K01119...
K01119...
2',3'-Cyclic CMP
3'-CMP
K00527
K21636
CNE03330...
K00527
K21636
K19796
K19797
hm5dCDP
CNE02610...
hm5dCTP
hm5dCMP
K18942
hm5C in DNA
CNE02610...
CNF00400
K00945...
K16904
dCTP
dCDP
dCMP
K00893...
Deoxycytidine
K01081...
K08722
K16904
K09887
CNC04940
CNB00950
K01494
CNB00780...
CNM01430
K00756
K00757
K03783...
K00758
CNE02610...
dUDP
CNC04630
Deoxyuridine
K00857
dUTP
3.6.1.39
dUMP
K01081...
K08722
Urea
5-Methylbarbiturate
1.17.99.4
K19794
K19795
CNE03330...
CNJ01230
K03465
3-Oxo-3-ureido-isobutyrate
Methylmalonate
CNA05950
5-Methylcytosine
UDP
K01081...
K08722
DNA
CNB00780...
dTTP
CNE02610...
dTDP
CNC04630
K01510...
K00857
K08728
K00758
K17722...
K00207
CNF01380
K01431...
Valine, leucine and isoleucine metabolism
K01510...
3.6.1.39
K01513...
dTMP
Thymidine
Thymine
(R)-Dihydro-thymine
(R)-3-Ureido-isobutyrate
(R)-3-Amino-isobutyrate
2-Deoxy-D-ribose-1P
00240 9/28/17
(c) Kanehisa Laboratories

# 5. Amino Acid metabolism

| MAP | PATHWAY |
|---|---|
| **220** | Arginine biosynthesis |
| **250** | Alanine, aspartate and glutamate metabolism |
| **260** | Glycine, serine and threonine metabolism |
| **270** | Cysteine and methionine metabolism |
| **280** | Valine, leucine and isoleucine degradation |
| **290** | Valine, leucine and isoleucine biosynthesis |
| **300** | Lysine biosynthesis |
| **310** | Lysine degradation |
| **330** | Arginine and proline metabolism |
| **340** | Histidine metabolism |
| **350** | Tyrosine metabolism |
| **360** | Phenylalanine metabolism |
| **380** | Tryptophan metabolism |
| **400** | Phenylalanine, tyrosine and tryptophan biosynthesis |

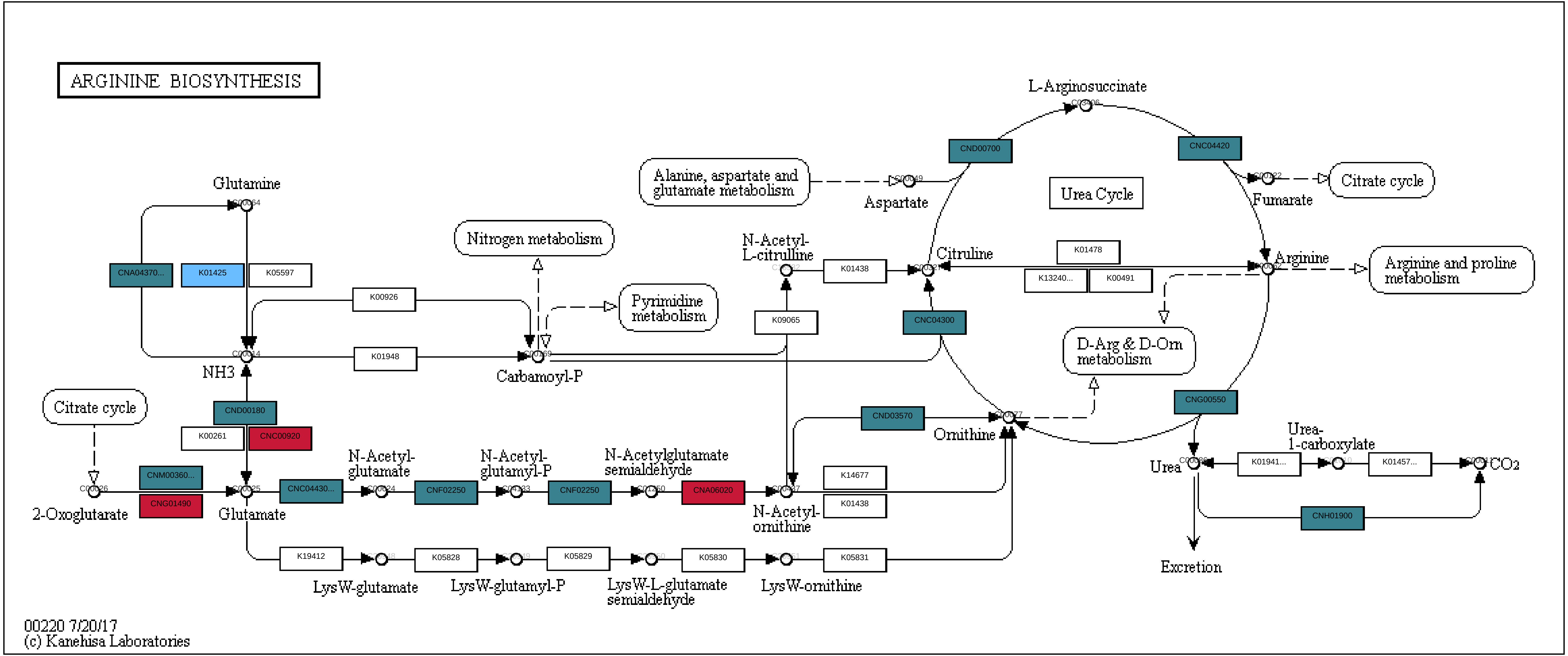

ARGININE BIOSYNTHESIS
L-Arginosuccinate
CND00700
CNC04420
Alanine, aspartate and glutamate metabolism
Aspartate
Urea Cycle
Citrate cycle
Fumarate
Glutamine
CNA04370...
K01425
K05597
Nitrogen metabolism
N-Acetyl-L-citrulline
K01438
Citruline
K01478
K13240...
K00491
Arginine
Arginine and proline metabolism
K00926
Pyrimidine metabolism
K09065
CNC04300
D-Arg & D-Orn metabolism
K01948
NH3
Carbamoyl-P
Citrate cycle
CND00180
K00261
CNC00920
CND03570
Ornithine
CNG00550
Urea-1-carboxylate
N-Acetyl-glutamate
N-Acetyl-glutamyl-P
N-Acetylglutamate semialdehyde
K14677
Urea
K01941...
K01457...
CO2
CNM00360...
CNG01490
2-Oxoglutarate
Glutamate
CNC04430...
CNF02250
CNF02250
CNA06020
N-Acetyl-ornithine
K01438
CNH01900
Excretion
K19412
K05828
K05829
K05830
K05831
LysW-glutamate
LysW-glutamyl-P
LysW-L-glutamate semialdehyde
LysW-ornithine
00220 7/20/17
(c) Kanehisa Laboratories

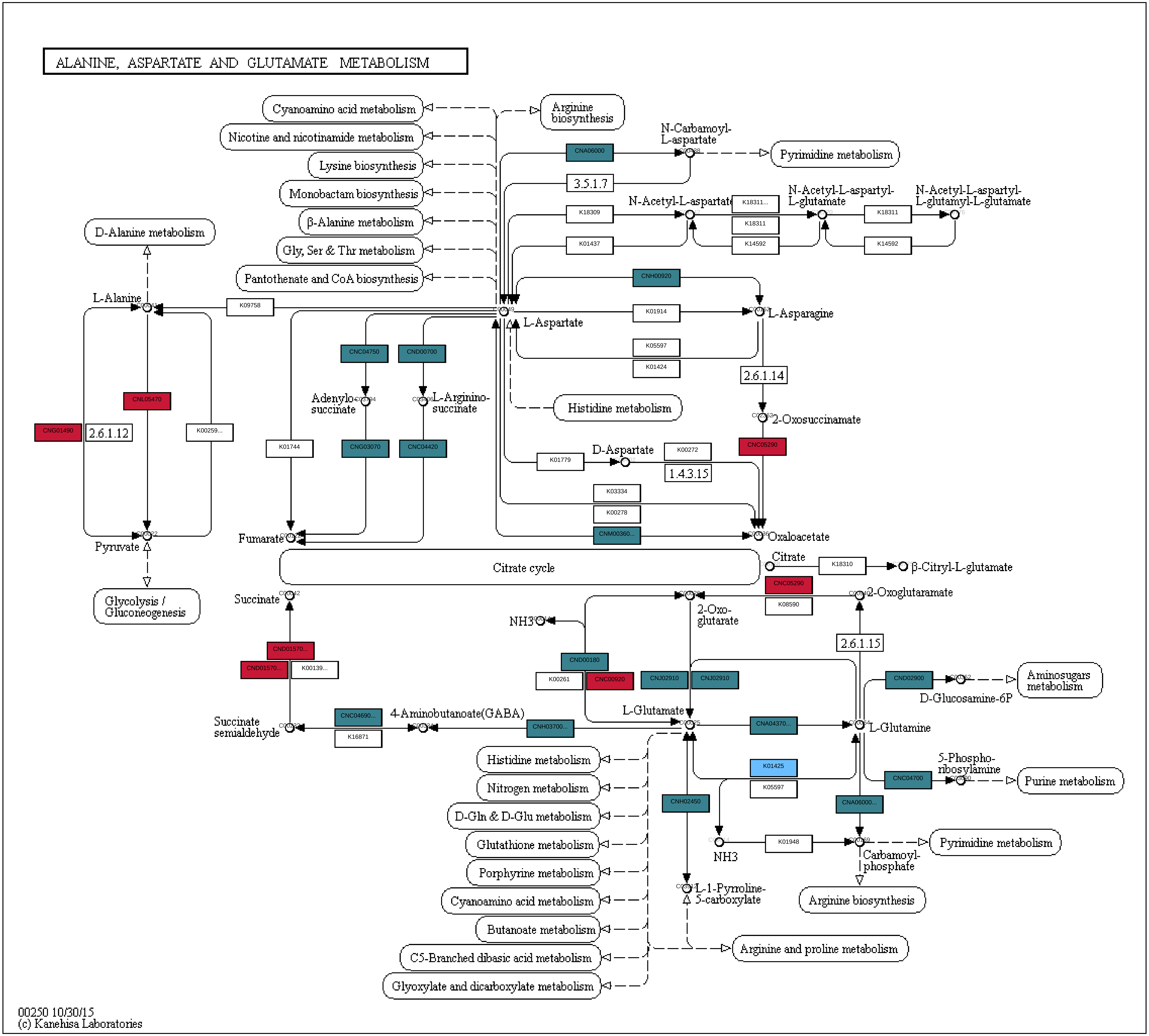
ALANINE, ASPARTATE AND GLUTAMATE METABOLISM
Cyanoamino acid metabolism
Nicotine and nicotinamide metabolism
Lysine biosynthesis
Monobactam biosynthesis
β-Alanine metabolism
Gly, Ser & Thr metabolism
Pantothenate and CoA biosynthesis
Arginine biosynthesis
D-Alanine metabolism
N-Carbamoyl-L-aspartate
Pyrimidine metabolism
CNA06000
3.5.1.7
N-Acetyl-L-aspartate
N-Acetyl-L-aspartyl-L-glutamate
N-Acetyl-L-aspartyl-L-glutamyl-L-glutamate
K18309
K18311...
K18311
K14592
K01437
CNH00920
L-Alanine
K09758
L-Aspartate
K01914
L-Asparagine
K05597
K01424
CNC04750
CND00700
Adenylo-succinate
L-Argino-succinate
Histidine metabolism
2.6.1.14
2-Oxosuccinamate
CNL05470
CNG01490
2.6.1.12
K00259...
K01744
CNG03070
CNC04420
CNC05290
D-Aspartate
K00272
K01779
1.4.3.15
K03334
K00278
CNM00360...
Pyruvate
Fumarate
Oxaloacetate
Citrate cycle
Citrate
K18310
β-Citryl-L-glutamate
Glycolysis / Gluconeogenesis
Succinate
CNC05290
K08590
2-Oxoglutaramate
NH3
2-Oxo-glutarate
2.6.1.15
CND01570...
CND01570...
K00139...
CND00180
K00261
CNC00920
CNJ02910
CNJ02910
CND02900
Aminosugars metabolism
D-Glucosamine-6P
Succinate semialdehyde
CNC04690...
K16871
4-Aminobutanoate(GABA)
CNH03700...
L-Glutamate
CNA04370...
L-Glutamine
5-Phospho-ribosylamine
CNC04700
Purine metabolism
Histidine metabolism
Nitrogen metabolism
D-Gln & D-Glu metabolism
Glutathione metabolism
Porphyrine metabolism
Cyanoamino acid metabolism
Butanoate metabolism
C5-Branched dibasic acid metabolism
Glyoxylate and dicarboxylate metabolism
K01425
K05597
CNH02450
CNA06000...
NH3
K01948
Carbamoyl-phosphate
Pyrimidine metabolism
L-1-Pyrroline-5-carboxylate
Arginine biosynthesis
Arginine and proline metabolism
00250 10/30/15
(c) Kanehisa Laboratories

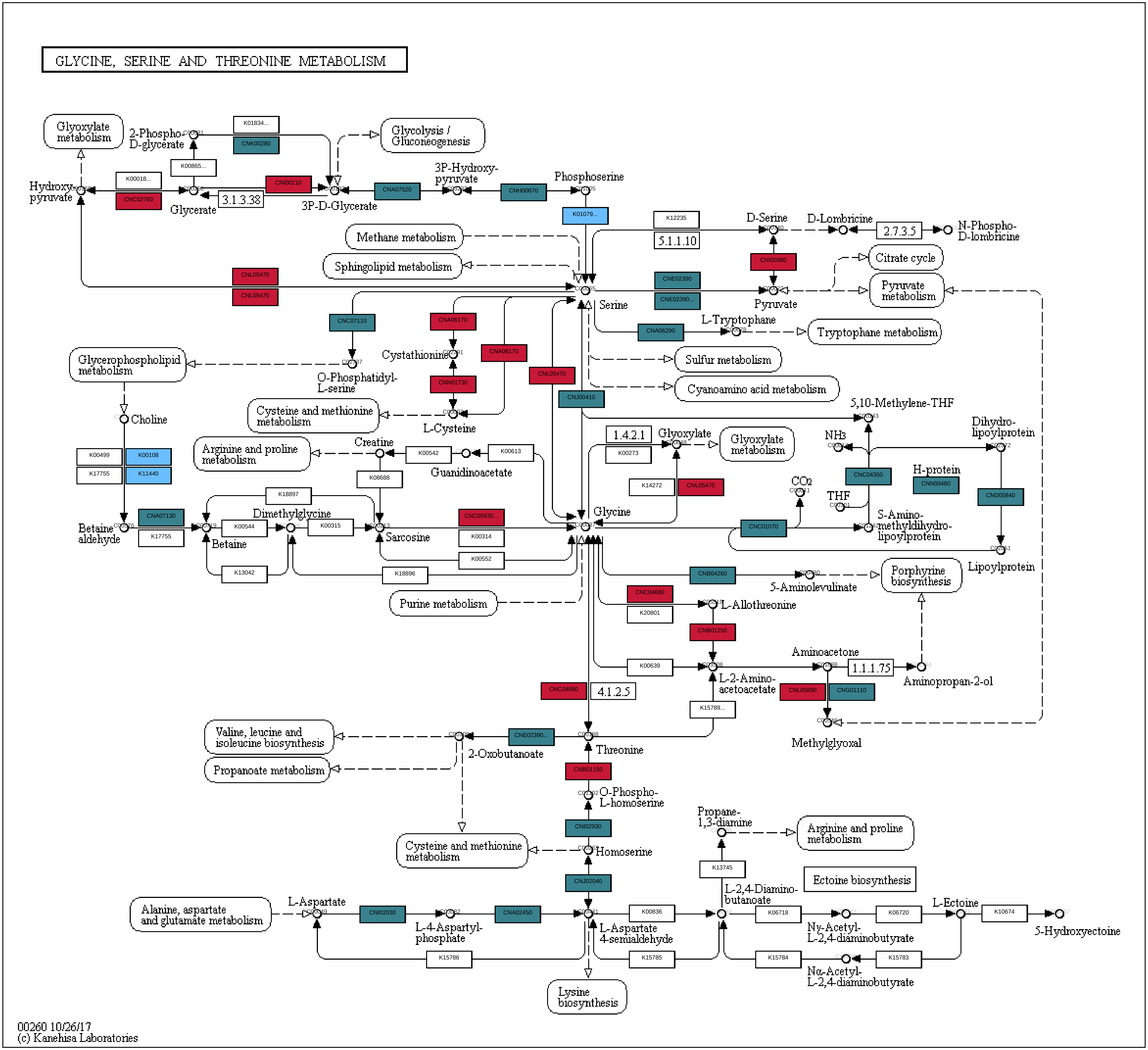
GLYCINE, SERINE AND THREONINE METABOLISM
Glyoxylate metabolism
2-Phospho-D-glycerate
K01834...
CNK00280
Glycolysis / Gluconeogenesis
K00865...
K00018...
Hydroxy-pyruvate
CNC02760
CNI00210
3.1.3.38
Glycerate
3P-D-Glycerate
CNA07520
3P-Hydroxy-pyruvate
CNH00670
Phosphoserine
K01079...
K12235
5.1.1.10
D-Serine
D-Lombricine
2.7.3.5
N-Phospho-D-lombricine
Methane metabolism
Sphingolipid metabolism
CNI00380
Citrate cycle
CNL05470
CNL05470
CNE02390
CNE02390...
Pyruvate metabolism
Serine
Pyruvate
CNC07110
CNA06170
CNA06290
L-Tryptophane
Tryptophane metabolism
Glycerophospholipid metabolism
Cystathionine
CNA06170
CNL05470
Sulfur metabolism
O-Phosphatidyl-L-serine
CNN01730
CNJ00410
Cyanoamino acid metabolism
Choline
Cysteine and methionine metabolism
L-Cysteine
5,10-Methylene-THF
Dihydro-lipoylprotein
Arginine and proline metabolism
Creatine
K00542
K00613
1.4.2.1
Glyoxylate
Glyoxylate metabolism
NH3
K00499
K00108
K17755
K11440
Guanidinoacetate
K08688
K00273
CNC04350
H-protein
CNN00460
K14272
CNL05470
CO2
THF
CND05840
K18897
Dimethylglycine
CNC05930...
Glycine
S-Amino-methyldihydro-lipoylprotein
Betaine aldehyde
CNA07130
K00544
K00315
K00314
CNC01070
K17755
Betaine
Sarcosine
K00552
Lipoylprotein
K13042
K18896
CNB04260
Porphyrine biosynthesis
5-Aminolevulinate
Purine metabolism
CNC04680
L-Allothreonine
K20801
CNB01250
Aminoacetone
K00639
1.1.1.75
Aminopropan-2-ol
CNC04680
4.1.2.5
L-2-Amino-acetoacetate
CNL05890
CNG01110
K15789...
Methylglyoxal
Valine, leucine and isoleucine biosynthesis
CNE02390...
2-Oxobutanoate
Threonine
Propanoate metabolism
CNB01150
O-Phospho-L-homoserine
Propane-1,3-diamine
Arginine and proline metabolism
CNI02930
Cysteine and methionine metabolism
Homoserine
K13745
Ectoine biosynthesis
CNJ02040
L-2,4-Diamino-butanoate
L-Aspartate
L-Ectoine
Alanine, aspartate and glutamate metabolism
CNI02030
CNA02450
K00836
K06718
K06720
K10674
L-4-Aspartyl-phosphate
L-Aspartate 4-semialdehyde
Nγ-Acetyl-L-2,4-diaminobutyrate
5-Hydroxyectoine
K15786
K15785
K15784
K15783
Nα-Acetyl-L-2,4-diaminobutyrate
Lysine biosynthesis
00260 10/26/17
(c) Kanehisa Laboratories

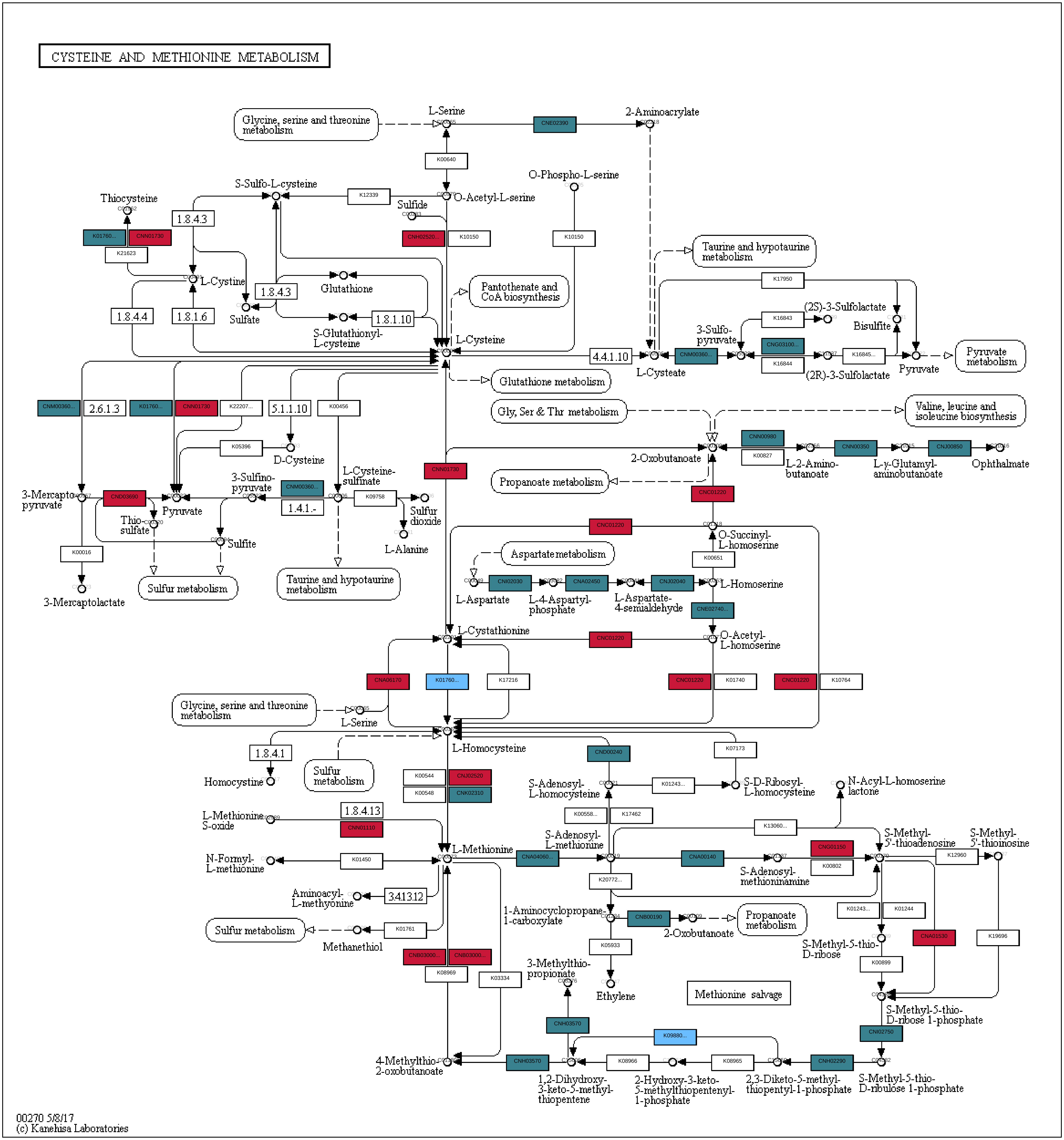
CYSTEINE AND METHIONINE METABOLISM
Glycine, serine and threonine metabolism
L-Serine
CNE02390
2-Aminoacrylate
K00640
O-Phospho-L-serine
S-Sulfo-L-cysteine
K12339
O-Acetyl-L-serine
Thiocysteine
1.8.4.3
Sulfide
K01760...
CNN01730
CNH02520...
K10150
K10150
K21623
Taurine and hypotaurine metabolism
L-Cystine
1.8.4.3
Glutathione
Pantothenate and CoA biosynthesis
K17950
1.8.4.4
1.8.1.6
Sulfate
1.8.1.10
S-Glutathionyl-L-cysteine
L-Cysteine
(2S)-3-Sulfolactate
K16843
Bisulfite
3-Sulfo-pyruvate
CNG03100...
4.4.1.10
CNM00360...
K16844
K16845...
Pyruvate
Pyruvate metabolism
L-Cysteate
(2R)-3-Sulfolactate
Glutathione metabolism
CNM00360...
2.6.1.3
K01760...
CNN01730
K22207...
5.1.1.10
K00456
Gly, Ser & Thr metabolism
Valine, leucine and isoleucine biosynthesis
K05396
D-Cysteine
CNN00980
K00827
CNN00350
CNJ00850
2-Oxobutanoate
L-2-Amino-butanoate
L-γ-Glutamyl-aminobutanoate
Ophthalmate
CNN01730
L-Cysteine-sulfinate
Propanoate metabolism
3-Sulfino-pyruvate
CNM00360...
3-Mercapto-pyruvate
CND03690
K09758
CNC01220
Pyruvate
1.4.1.-
Sulfur dioxide
Thio-sulfate
Sulfite
CNC01220
O-Succinyl-L-homoserine
K00016
L-Alanine
Aspartate metabolism
K00651
Sulfur metabolism
Taurine and hypotaurine metabolism
CNI02030
CNA02450
CNJ02040
L-Homoserine
3-Mercaptolactate
L-Aspartate
L-4-Aspartyl-phosphate
L-Aspartate-4-semialdehyde
CNE02740...
L-Cystathionine
CNC01220
O-Acetyl-L-homoserine
CNA06170
K01760...
K17216
CNC01220
K01740
CNC01220
K10764
Glycine, serine and threonine metabolism
L-Serine
L-Homocysteine
1.8.4.1
CND00240
K07173
Sulfur metabolism
Homocystine
K00544
CNJ02520
K00548
CNK02310
S-Adenosyl-L-homocysteine
K01243...
S-D-Ribosyl-L-homocysteine
N-Acyl-L-homoserine lactone
1.8.4.13
L-Methionine S-oxide
CNN01110
K00558...
K17462
K13060...
S-Methyl-5'-thioadenosine
S-Methyl-5'-thioinosine
L-Methionine
S-Adenosyl-L-methionine
CNA04060...
CNA00140
CNG01150
K12960
N-Formyl-L-methionine
K01450
K00802
S-Adenosyl-methioninamine
K20772...
Aminoacyl-L-methyonine
3.4.13.12
1-Aminocyclopropane-1-carboxylate
CNB00190
Propanoate metabolism
K01243...
K01244
2-Oxobutanoate
Sulfur metabolism
K01761
S-Methyl-5-thio-D-ribose
CNA01530
K19696
Methanethiol
K05933
CNB03000...
CNB03000...
K00899
K08969
K03334
3-Methylthio-propionate
Ethylene
Methionine salvage
S-Methyl-5-thio-D-ribose 1-phosphate
CNH03570
CNI02750
K09880...
4-Methylthio-2-oxobutanoate
CNH03570
K08966
K08965
CNH02290
1,2-Dihydroxy-3-keto-5-methyl-thiopentene
2-Hydroxy-3-keto-5-methylthiopentenyl-1-phosphate
2,3-Diketo-5-methyl-thiopentyl-1-phosphate
S-Methyl-5-thio-D-ribulose 1-phosphate
00270 5/8/17
(c) Kanehisa Laboratories

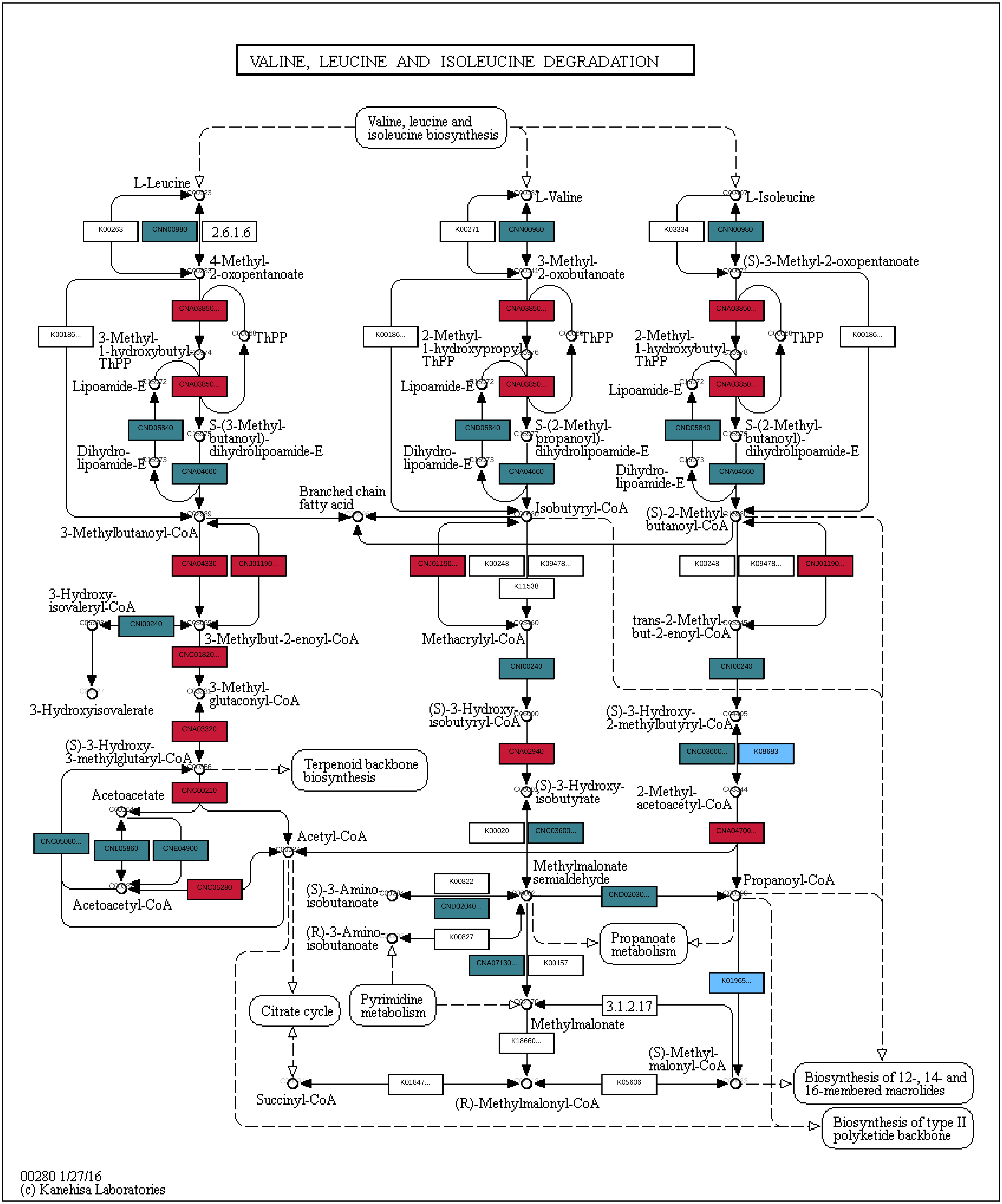
VALINE, LEUCINE AND ISOLEUCINE DEGRADATION
Valine, leucine and isoleucine biosynthesis
L-Leucine
C00123
K00263
CNN00980
2.6.1.6
4-Methyl-2-oxopentanoate
CNA03850...
K00186...
3-Methyl-1-hydroxybutyl-ThPP
ThPP
Lipoamide-E
CND05840
S-(3-Methylbutanoyl)-dihydrolipoamide-E
Dihydro-lipoamide-E
CNA04660
Branched chain fatty acid
3-Methylbutanoyl-CoA
CNA04330
CNJ01190...
3-Hydroxy-isovaleryl-CoA
CNI00240
3-Methylbut-2-enoyl-CoA
CNC01820...
3-Hydroxyisovalerate
3-Methyl-glutaconyl-CoA
CNA03320
(S)-3-Hydroxy-3-methylglutaryl-CoA
Terpenoid backbone biosynthesis
CNC00210
Acetoacetate
CNC05080...
CNL05860
CNE04900
CNC05280
Acetoacetyl-CoA
Acetyl-CoA
L-Valine
K00271
3-Methyl-2-oxobutanoate
2-Methyl-1-hydroxypropyl-ThPP
S-(2-Methylpropanoyl)-dihydrolipoamide-E
Isobutyryl-CoA
K00248
K09478...
K11538
Methacrylyl-CoA
(S)-3-Hydroxy-isobutyryl-CoA
CNA02940
(S)-3-Hydroxy-isobutyrate
K00020
CNC03600...
Methylmalonate semialdehyde
(S)-3-Amino-isobutanoate
K00822
CND02040...
CND02030...
(R)-3-Amino-isobutanoate
K00827
CNA07130...
K00157
Propanoate metabolism
Pyrimidine metabolism
Citrate cycle
3.1.2.17
Methylmalonate
K18660...
Succinyl-CoA
K01847...
(R)-Methylmalonyl-CoA
K05606
(S)-Methyl-malonyl-CoA
L-Isoleucine
K03334
(S)-3-Methyl-2-oxopentanoate
2-Methyl-1-hydroxybutyl-ThPP
S-(2-Methylbutanoyl)-dihydrolipoamide-E
(S)-2-Methyl-butanoyl-CoA
trans-2-Methyl-but-2-enoyl-CoA
(S)-3-Hydroxy-2-methylbutyryl-CoA
K08683
2-Methyl-acetoacetyl-CoA
CNA04700...
Propanoyl-CoA
K01965...
Biosynthesis of 12-, 14- and 16-membered macrolides
Biosynthesis of type II polyketide backbone
00280 1/27/16
(c) Kanehisa Laboratories

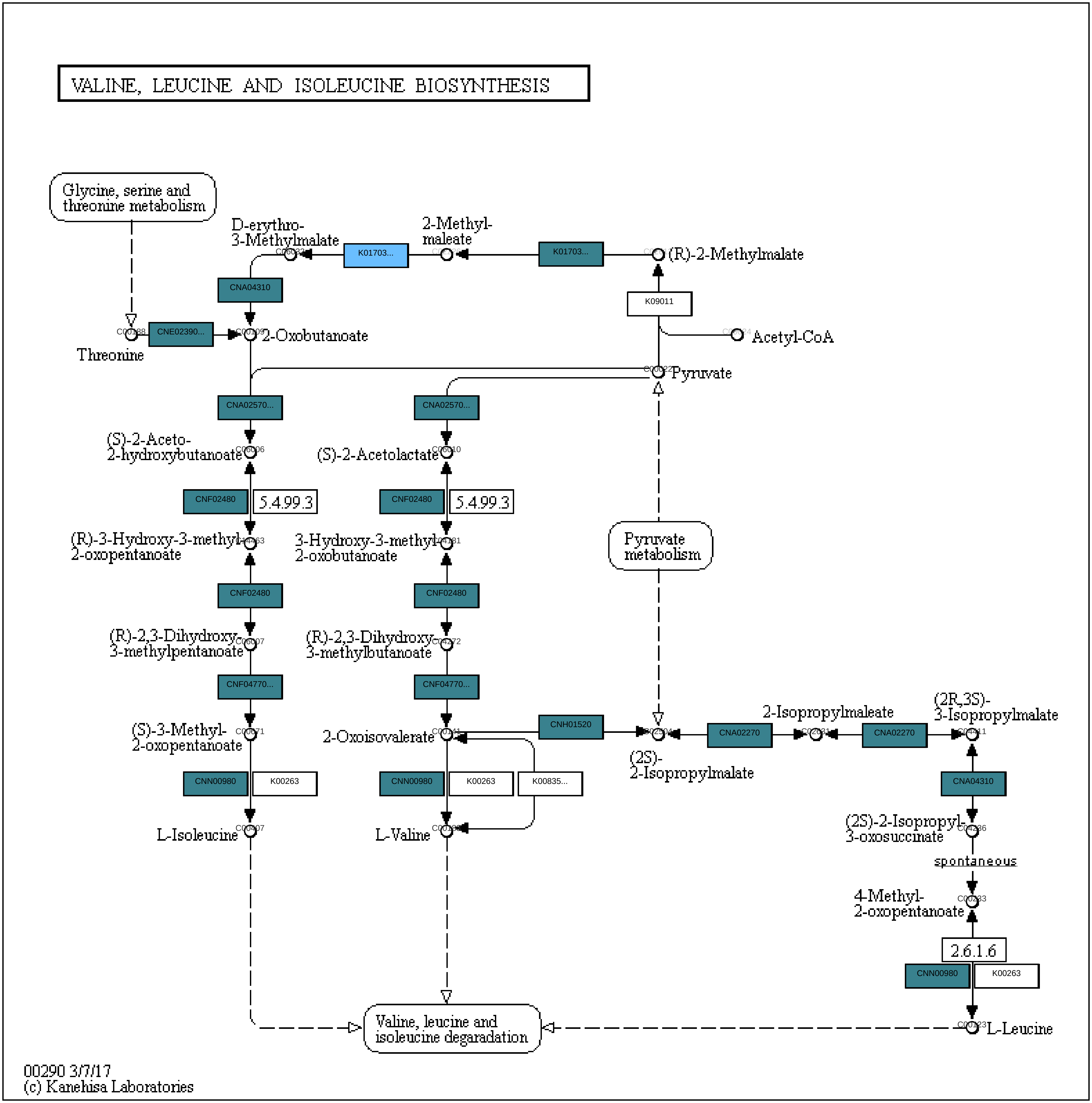

VALINE, LEUCINE AND ISOLEUCINE BIOSYNTHESIS
Glycine, serine and threonine metabolism
D-erythro-3-Methylmalate
K01703...
2-Methyl-maleate
K01703...
(R)-2-Methylmalate
CNA04310
K09011
Acetyl-CoA
CNE02390...
2-Oxobutanoate
Threonine
Pyruvate
CNA02570...
CNA02570...
(S)-2-Aceto-2-hydroxybutanoate
(S)-2-Acetolactate
CNF02480
5.4.99.3
CNF02480
5.4.99.3
(R)-3-Hydroxy-3-methyl-2-oxopentanoate
3-Hydroxy-3-methyl-2-oxobutanoate
Pyruvate metabolism
CNF02480
CNF02480
(R)-2,3-Dihydroxy-3-methylpentanoate
(R)-2,3-Dihydroxy-3-methylbutanoate
CNF04770...
CNF04770...
CNH01520
(S)-3-Methyl-2-oxopentanoate
2-Oxoisovalerate
CNA02270
2-Isopropylmaleate
CNA02270
(2R,3S)-3-Isopropylmalate
(2S)-2-Isopropylmalate
CNN00980
K00263
CNN00980
K00263
K00835...
CNA04310
L-Isoleucine
L-Valine
(2S)-2-Isopropyl-3-oxosuccinate
spontaneous
4-Methyl-2-oxopentanoate
2.6.1.6
CNN00980
K00263
Valine, leucine and isoleucine degaradation
L-Leucine
00290 3/7/17
(c) Kanehisa Laboratories

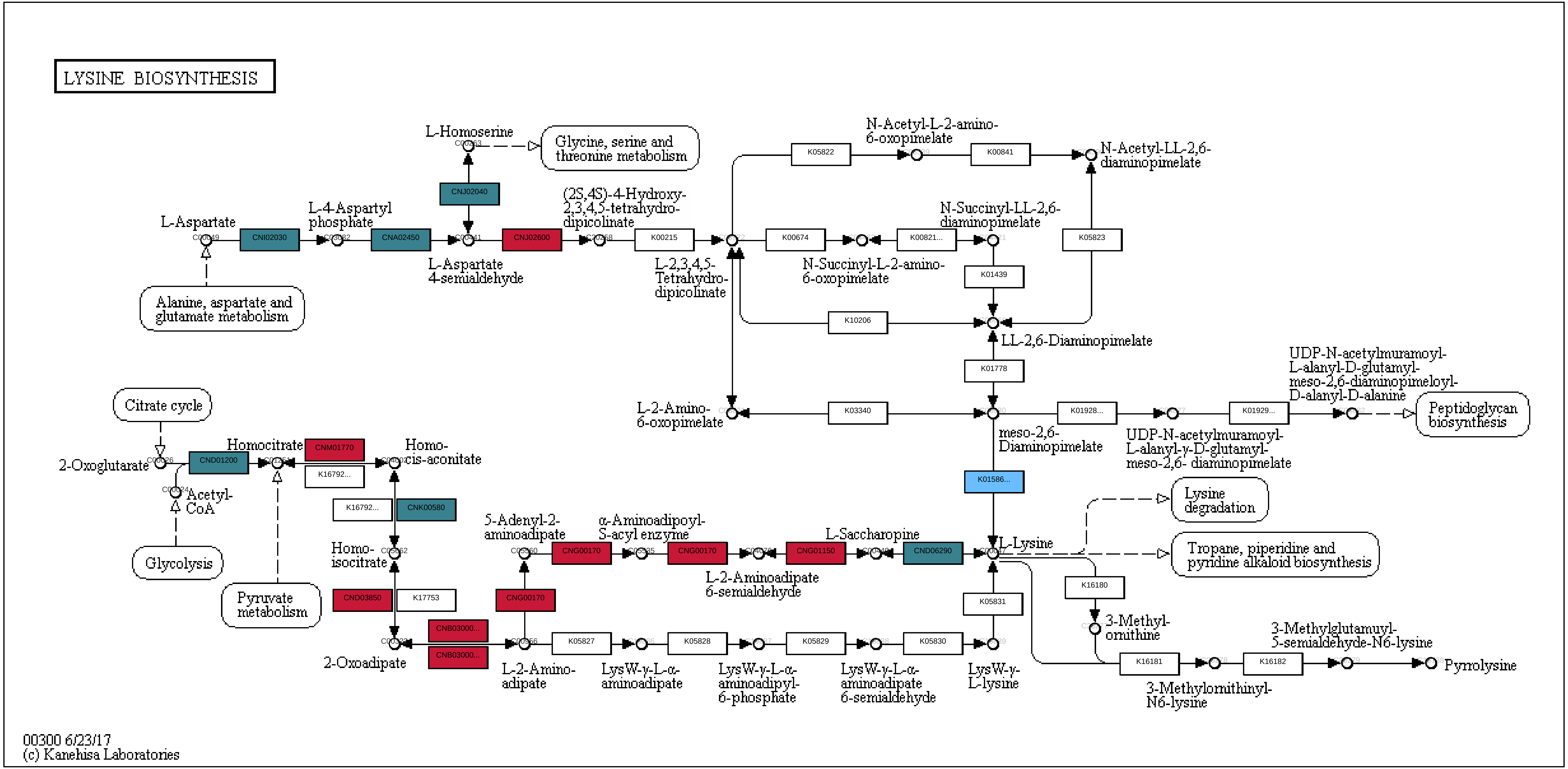
LYSINE BIOSYNTHESIS
L-Homoserine
Glycine, serine and threonine metabolism
CNJ02040
L-Aspartate
CNI02030
L-4-Aspartyl phosphate
CNA02450
CNJ02600
(2S,4S)-4-Hydroxy-2,3,4,5-tetrahydro-dipicolinate
K00215
L-Aspartate 4-semialdehyde
L-2,3,4,5-Tetrahydro-dipicolinate
Alanine, aspartate and glutamate metabolism
N-Acetyl-L-2-amino-6-oxopimelate
K05822
K00841
N-Acetyl-LL-2,6-diaminopimelate
K00674
K00821...
N-Succinyl-LL-2,6-diaminopimelate
K05823
N-Succinyl-L-2-amino-6-oxopimelate
K01439
K10206
LL-2,6-Diaminopimelate
K01778
L-2-Amino-6-oxopimelate
K03340
K01928...
K01929...
meso-2,6-Diaminopimelate
UDP-N-acetylmuramoyl-L-alanyl-D-glutamyl-meso-2,6-diaminopimeloyl-D-alanyl-D-alanine
UDP-N-acetylmuramoyl-L-alanyl-γ-D-glutamyl-meso-2,6- diaminopimelate
Peptidoglycan biosynthesis
K01586...
Citrate cycle
2-Oxoglutarate
CND01200
Homocitrate
CNM01770
K16792...
Homo-cis-aconitate
Acetyl-CoA
K16792...
CNK00580
Glycolysis
Pyruvate metabolism
Homo-isocitrate
CND03850
K17753
5-Adenyl-2-aminoadipate
α-Aminoadipoyl-S-acyl enzyme
CNG00170
CNG00170
L-Saccharopine
CNG01150
CND06290
L-Lysine
Lysine degradation
Tropane, piperidine and pyridine alkaloid biosynthesis
L-2-Aminoadipate 6-semialdehyde
CNG00170
CNB03000...
CNB03000...
2-Oxoadipate
L-2-Amino-adipate
K05827
K05828
K05829
K05830
K05831
K16180
3-Methyl-ornithine
LysW-γ-L-α-aminoadipate
LysW-γ-L-α-aminoadipyl-6-phosphate
LysW-γ-L-α-aminoadipate 6-semialdehyde
LysW-γ-L-lysine
K16181
K16182
3-Methylglutamuyl-5-semialdehyde-N6-lysine
3-Methylornithinyl-N6-lysine
Pyrrolysine
00300 6/23/17
(c) Kanehisa Laboratories

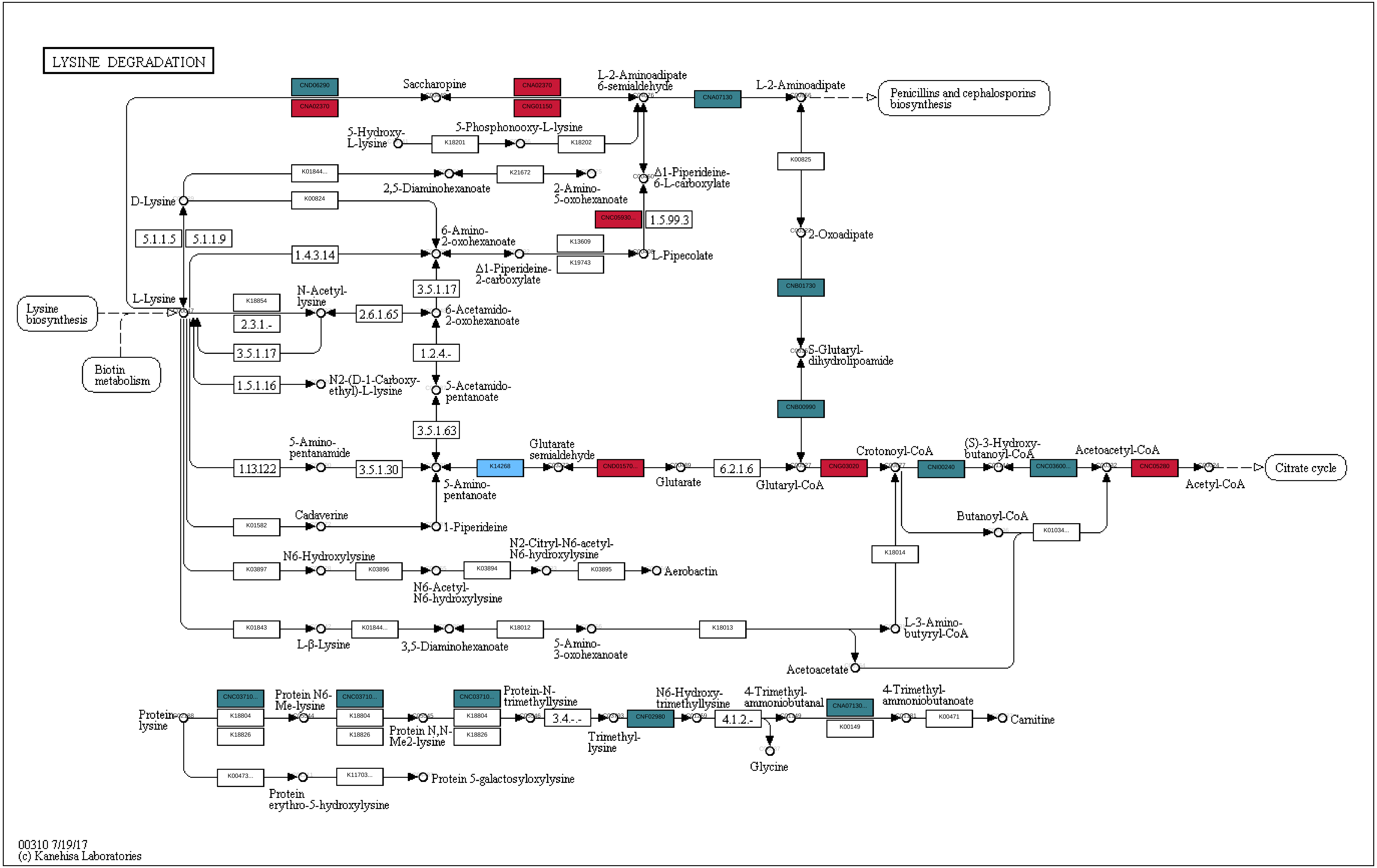
LYSINE DEGRADATION
CND06290
CNA02370
Saccharopine
CNA02370
CNG01150
L-2-Aminoadipate 6-semialdehyde
CNA07130
L-2-Aminoadipate
Penicillins and cephalosporins biosynthesis
5-Hydroxy-L-lysine
K18201
5-Phosphonooxy-L-lysine
K18202
K01844...
K21672
2,5-Diaminohexanoate
2-Amino-5-oxohexanoate
Δ1-Piperideine-6-L-carboxylate
K00825
D-Lysine
K00824
CNC05930...
1.5.99.3
5.1.1.5
5.1.1.9
6-Amino-2-oxohexanoate
2-Oxoadipate
1.4.3.14
K13609
K19743
L-Pipecolate
Δ1-Piperideine-2-carboxylate
3.5.1.17
CNB01730
Lysine biosynthesis
L-Lysine
K18854
N-Acetyl-lysine
2.6.1.65
6-Acetamido-2-oxohexanoate
2.3.1.-
3.5.1.17
1.2.4.-
S-Glutaryl-dihydrolipoamide
Biotin metabolism
1.5.1.16
N2-(D-1-Carboxy-ethyl)-L-lysine
5-Acetamido-pentanoate
3.5.1.63
CNB00990
5-Amino-pentanamide
Glutarate semialdehyde
Crotonoyl-CoA
(S)-3-Hydroxy-butanoyl-CoA
Acetoacetyl-CoA
1.13.12.2
3.5.1.30
K14268
CND01570...
6.2.1.6
CNG03020
CNI00240
CNC03600...
CNC05280
Citrate cycle
5-Amino-pentanoate
Glutarate
Glutaryl-CoA
Acetyl-CoA
Cadaverine
K01582
1-Piperideine
Butanoyl-CoA
K01034...
K18014
N2-Citryl-N6-acetyl-N6-hydroxylysine
N6-Hydroxylysine
K03897
K03896
K03894
K03895
Aerobactin
N6-Acetyl-N6-hydroxylysine
K01843
K01844...
K18012
K18013
L-3-Amino-butyryl-CoA
L-β-Lysine
3,5-Diaminohexanoate
5-Amino-3-oxohexanoate
Acetoacetate
CNC03710...
Protein N6-Me-lysine
CNC03710...
CNC03710...
Protein-N-trimethyllysine
N6-Hydroxy-trimethyllysine
4-Trimethyl-ammoniobutanal
CNA07130...
4-Trimethyl-ammoniobutanoate
Protein-lysine
K18804
K18826
K18804
K18826
K18804
K18826
Protein N,N-Me2-lysine
3.4.-.-
CNF02980
4.1.2.-
K00149
K00471
Carnitine
Trimethyl-lysine
Glycine
K00473...
K11703...
Protein 5-galactosyloxylysine
Protein erythro-5-hydroxylysine
00310 7/19/17
(c) Kanehisa Laboratories

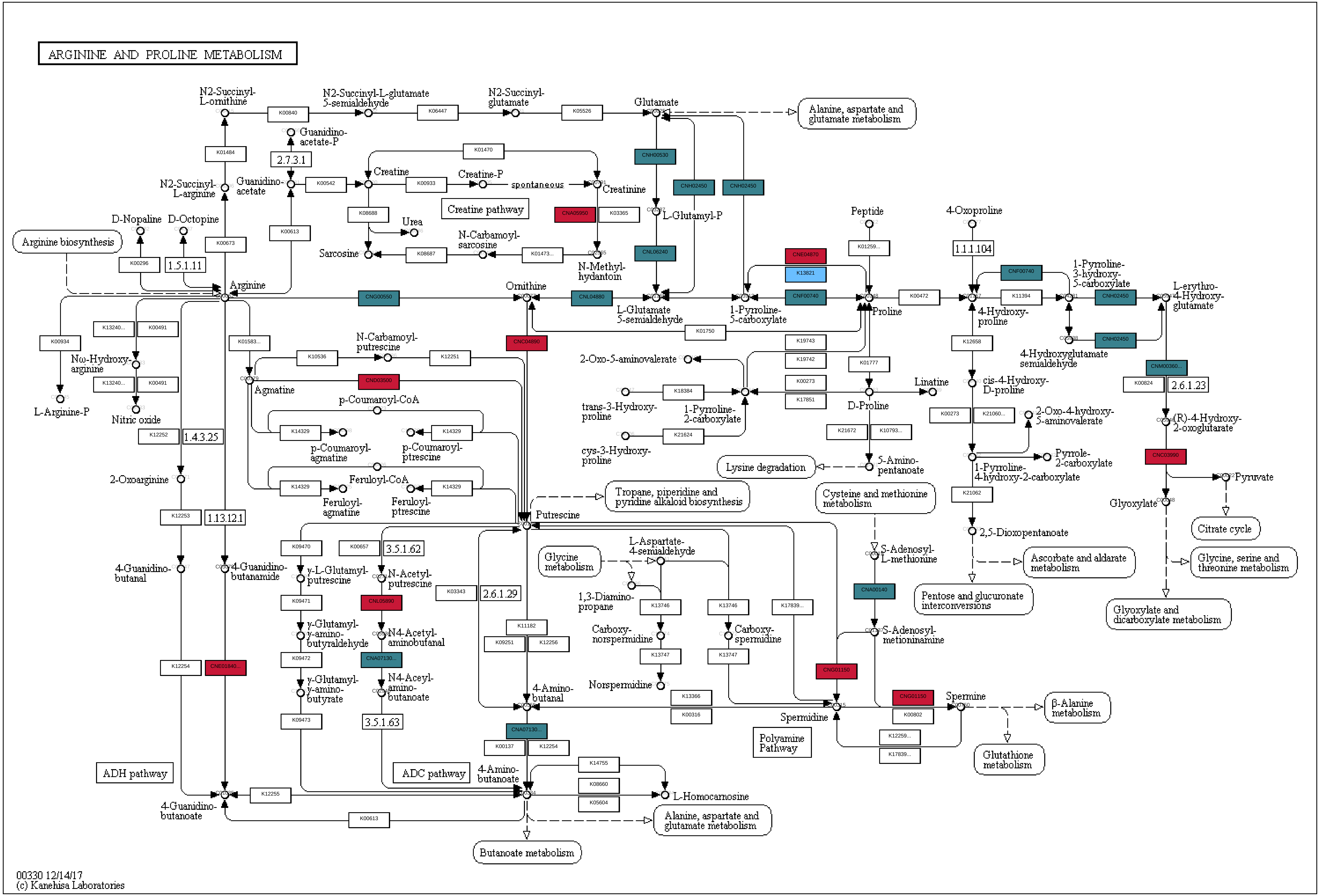

ARGININE AND PROLINE METABOLISM
N2-Succinyl-L-ornithine
N2-Succinyl-L-glutamate 5-semialdehyde
N2-Succinyl-glutamate
Glutamate
Alanine, aspartate and glutamate metabolism
Guanidino-acetate-P
N2-Succinyl-L-arginine
Guanidino-acetate
Creatine
Creatine-P
spontaneous
Creatinine
Creatine pathway
L-Glutamyl-P
Urea
D-Nopaline
D-Octopine
Arginine biosynthesis
Sarcosine
N-Carbamoyl-sarcosine
N-Methyl-hydantoin
Peptide
4-Oxoproline
Arginine
Ornithine
L-Glutamate 5-semialdehyde
1-Pyrroline-5-carboxylate
Proline
4-Hydroxy-proline
1-Pyrroline-3-hydroxy-5-carboxylate
L-erythro-4-Hydroxy-glutamate
4-Hydroxyglutamate semialdehyde
Nω-Hydroxy-arginine
N-Carbamoyl-putrescine
2-Oxo-5-aminovalerate
Agmatine
Linatine
cis-4-Hydroxy-D-proline
L-Arginine-P
Nitric oxide
p-Coumaroyl-CoA
trans-3-Hydroxy-proline
1-Pyrroline-2-carboxylate
D-Proline
2-Oxo-4-hydroxy-5-aminovalerate
(R)-4-Hydroxy-2-oxoglutarate
p-Coumaroyl-agmatine
p-Coumaroyl-ptrescine
cys-3-Hydroxy-proline
Lysine degradation
5-Amino-pentanoate
Pyrrole-2-carboxylate
1-Pyrroline-4-hydroxy-2-carboxylate
2-Oxoarginine
Feruloyl-CoA
Feruloyl-agmatine
Feruloyl-ptrescine
Putrescine
Tropane, piperidine and pyridine alkaloid biosynthesis
Cysteine and methionine metabolism
Pyruvate
Glyoxylate
Citrate cycle
2,5-Dioxopentanoate
4-Guanidino-butanal
4-Guanidino-butanamide
γ-L-Glutamyl-putrescine
N-Acetyl-putrescine
Glycine metabolism
L-Aspartate-4-semialdehyde
S-Adenosyl-L-methionine
Ascorbate and aldarate metabolism
Glycine, serine and threonine metabolism
1,3-Diamino-propane
Pentose and glucuronate interconversions
Glyoxylate and dicarboxylate metabolism
γ-Glutamyl-γ-amino-butyraldehyde
N4-Acetyl-aminobutanal
Carboxy-norspermidine
Carboxy-spermidine
S-Adenosyl-metioninamine
γ-Glutamyl-γ-amino-butyrate
N4-Aceyl-amino-butanoate
Norspermidine
4-Amino-butanal
Spermine
Spermidine
β-Alanine metabolism
Polyamine Pathway
Glutathione metabolism
ADH pathway
ADC pathway
4-Amino-butanoate
L-Homocarnosine
4-Guanidino-butanoate
Alanine, aspartate and glutamate metabolism
Butanoate metabolism
00330 12/14/17
(c) Kanehisa Laboratories

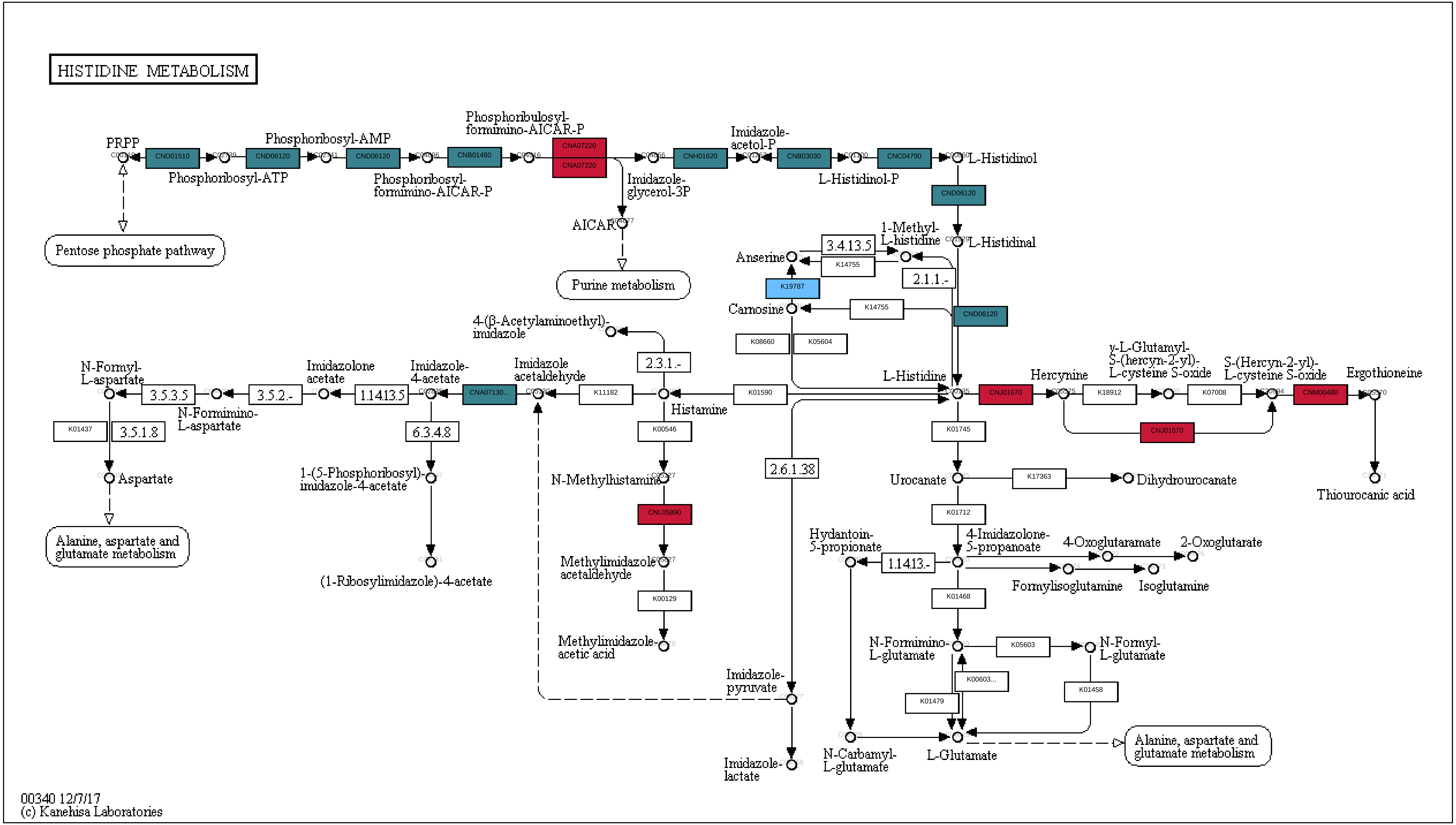

HISTIDINE METABOLISM
PRPP
CND01510
Phosphoribosyl-ATP
CND06120
Phosphoribosyl-AMP
CND06120
Phosphoribosyl-formimino-AICAR-P
CNB01460
Phosphoribulosyl-formimino-AICAR-P
CNA07220
CNA07220
AICAR
Imidazole-glycerol-3P
CNH01620
Imidazole-acetol-P
CNB03030
L-Histidinol-P
CNC04790
L-Histidinol
CND06120
L-Histidinal
Pentose phosphate pathway
Purine metabolism
1-Methyl-L-histidine
3.4.13.5
Anserine
K14755
2.1.1.-
K19787
Carnosine
K14755
CND06120
K08660
K05604
4-(β-Acetylaminoethyl)-imidazole
2.3.1.-
N-Formyl-L-aspartate
3.5.3.5
N-Formimino-L-aspartate
3.5.2.-
Imidazolone acetate
1.14.13.5
Imidazole-4-acetate
CNA07130...
Imidazole acetaldehyde
K11182
Histamine
K01590
L-Histidine
CNJ01670
Hercynine
K18912
γ-L-Glutamyl-S-(hercyn-2-yl)-L-cysteine S-oxide
K07008
S-(Hercyn-2-yl)-L-cysteine S-oxide
CNM00480
Ergothioneine
CNJ01670
K01437
3.5.1.8
6.3.4.8
K00546
K01745
Aspartate
1-(5-Phosphoribosyl)-imidazole-4-acetate
N-Methylhistamine
2.6.1.38
Urocanate
K17363
Dihydrourocanate
Thiourocanic acid
CNL05890
K01712
Alanine, aspartate and glutamate metabolism
Hydantoin-5-propionate
1.14.13.-
4-Imidazolone-5-propanoate
4-Oxoglutaramate
2-Oxoglutarate
Methylimidazole acetaldehyde
(1-Ribosylimidazole)-4-acetate
Formylisoglutamine
Isoglutamine
K00129
K01468
Methylimidazole-acetic acid
N-Formimino-L-glutamate
K05603
N-Formyl-L-glutamate
K00603...
Imidazole-pyruvate
K01479
K01458
N-Carbamyl-L-glutamate
L-Glutamate
Alanine, aspartate and glutamate metabolism
Imidazole-lactate
00340 12/7/17
(c) Kanehisa Laboratories

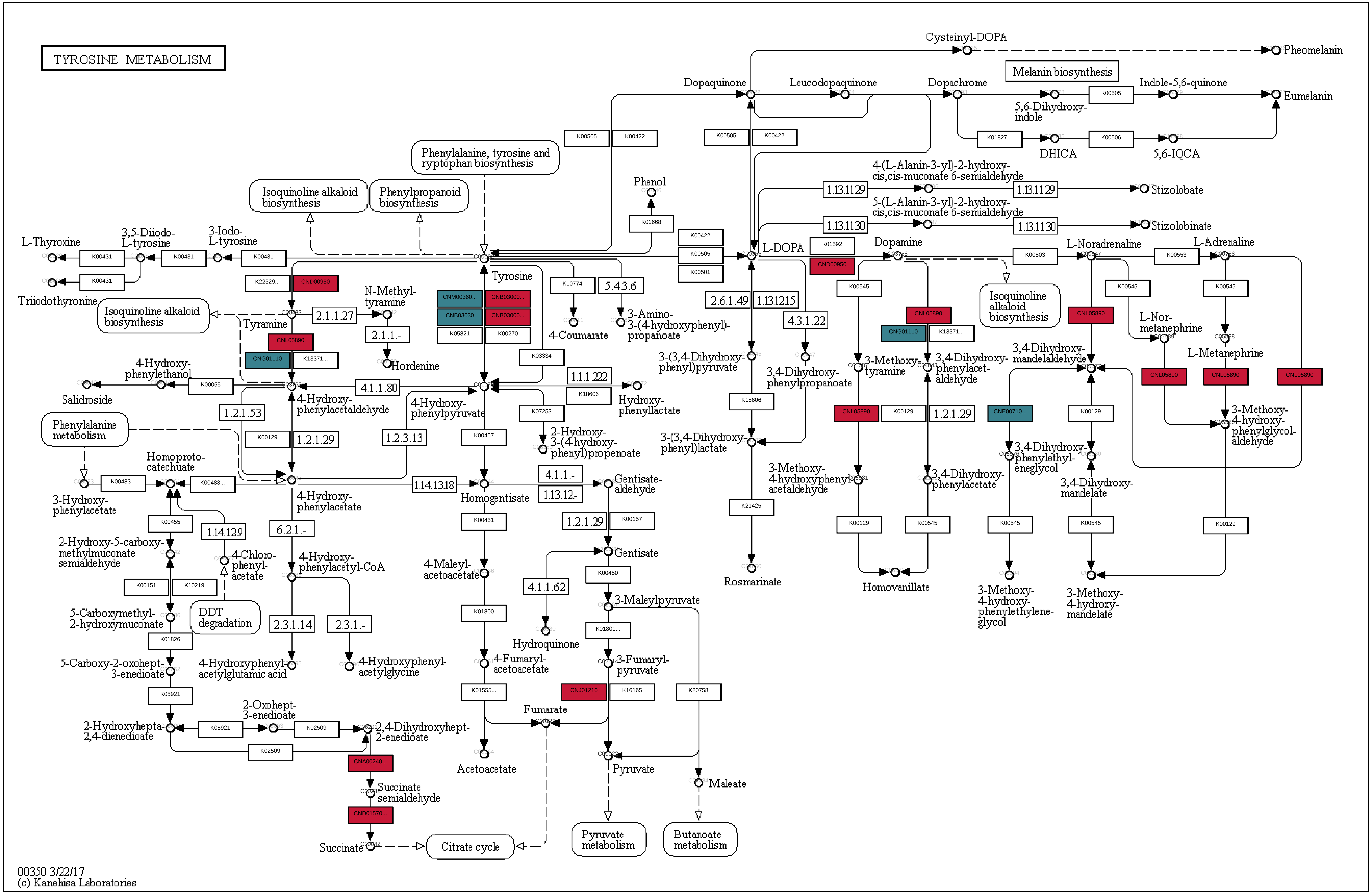
TYROSINE METABOLISM
Cysteinyl-DOPA
Pheomelanin
Melanin biosynthesis
Dopaquinone
Leucodopaquinone
Dopachrome
Indole-5,6-quinone
Eumelanin
5,6-Dihydroxy-indole
DHICA
5,6-IQCA
K00505
K00422
K01827...
K00506
Phenylalanine, tyrosine and ryptophan biosynthesis
Isoquinoline alkaloid biosynthesis
Phenylpropanoid biosynthesis
Phenol
K01668
4-(L-Alanin-3-yl)-2-hydroxy-cis,cis-muconate 6-semialdehyde
5-(L-Alanin-3-yl)-2-hydroxy-cis,cis-muconate 6-semialdehyde
1.13.11.29
1.13.11.30
Stizolobate
Stizolobinate
L-Thyroxine
3,5-Diiodo-L-tyrosine
3-Iodo-L-tyrosine
K00431
Triiodothyronine
L-DOPA
K01592
Dopamine
L-Noradrenaline
L-Adrenaline
K00503
K00553
K00501
CND00950
Tyrosine
K22329...
N-Methyl-tyramine
2.1.1.27
2.1.1.-
Tyramine
Hordenine
CNM00360...
CNB03000...
CNB03030
K05821
K00270
K10774
5.4.3.6
4-Coumarate
3-Amino-3-(4-hydroxyphenyl)-propanoate
2.6.1.49
1.13.12.15
4.3.1.22
K00545
CNL05890
CNG01110
K13371...
Isoquinoline alkaloid biosynthesis
L-Nor-metanephrine
L-Metanephrine
3-(3,4-Dihydroxy-phenyl)pyruvate
3,4-Dihydroxy-phenylpropanoate
3-Methoxy-tyramine
3,4-Dihydroxy-phenylacet-aldehyde
3,4-Dihydroxy-mandelaldehyde
4-Hydroxy-phenylethanol
K00055
Salidroside
K03334
4.1.1.80
1.1.1.222
K18606
Hydroxy-phenyllactate
4-Hydroxy-phenylacetaldehyde
4-Hydroxy-phenylpyruvate
K07253
2-Hydroxy-3-(4-hydroxy-phenyl)propenoate
3-(3,4-Dihydroxy-phenyl)lactate
K00129
1.2.1.29
CNE00710...
3-Methoxy-4-hydroxy-phenylglycol-aldehyde
3,4-Dihydroxy-phenylethyl-eneglycol
3,4-Dihydroxy-mandelate
3,4-Dihydroxy-phenylacetate
3-Methoxy-4-hydroxyphenyl-acetaldehyde
1.2.1.53
Phenylalanine metabolism
1.2.3.13
K00457
Homoproto-catechuate
K00483...
3-Hydroxy-phenylacetate
1.14.13.18
Homogentisate
4.1.1.-
1.13.12.-
Gentisate-aldehyde
4-Hydroxy-phenylacetate
K21425
K00455
1.14.12.9
K00451
1.2.1.29
K00157
Gentisate
Rosmarinate
Homovanillate
3-Methoxy-4-hydroxy-phenylethylene-glycol
3-Methoxy-4-hydroxy-mandelate
2-Hydroxy-5-carboxy-methylmuconate semialdehyde
4-Chloro-phenyl-acetate
6.2.1.-
4-Hydroxy-phenylacetyl-CoA
4-Maleyl-acetoacetate
K00450
4.1.1.62
3-Maleylpyruvate
K00151
K10219
DDT degradation
5-Carboxymethyl-2-hydroxymuconate
K01826
2.3.1.14
2.3.1.-
K01800
Hydroquinone
K01801...
5-Carboxy-2-oxohept-3-enedioate
4-Hydroxyphenyl-acetylglutamic acid
4-Hydroxyphenyl-acetylglycine
4-Fumaryl-acetoacetate
3-Fumaryl-pyruvate
K05921
K01555...
CNJ01210
K16165
K20758
Fumarate
2-Oxohept-3-enedioate
2-Hydroxyhepta-2,4-dienedioate
K02509
2,4-Dihydroxyhept-2-enedioate
CNA00240...
Acetoacetate
Pyruvate
Maleate
Succinate semialdehyde
CND01570...
Succinate
Citrate cycle
Pyruvate metabolism
Butanoate metabolism
00350 3/22/17
(c) Kanehisa Laboratories

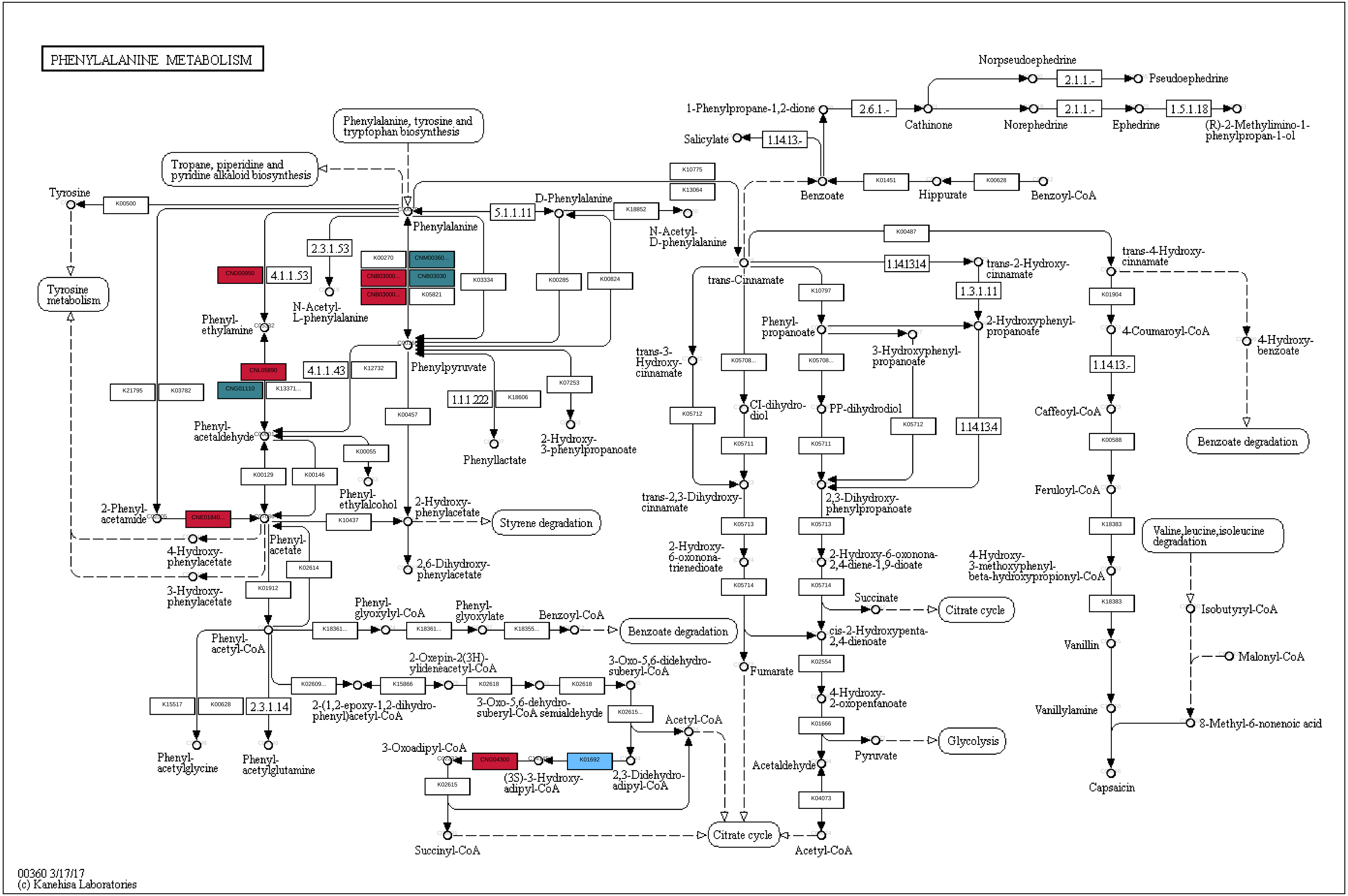
PHENYLALANINE METABOLISM
Norpseudoephedrine
2.1.1.-
Pseudoephedrine
1-Phenylpropane-1,2-dione
2.6.1.-
Cathinone
Norephedrine
2.1.1.-
Ephedrine
1.5.1.18
(R)-2-Methylimino-1-phenylpropan-1-ol
Salicylate
1.14.13.-
Phenylalanine, tyrosine and tryptophan biosynthesis
Tropane, piperidine and pyridine alkaloid biosynthesis
K10775
K13064
Benzoate
K01451
Hippurate
K00628
Benzoyl-CoA
Tyrosine
K00500
5.1.1.11
D-Phenylalanine
K18852
N-Acetyl-D-phenylalanine
Phenylalanine
2.3.1.53
K00270
CNM00360...
K00487
trans-4-Hydroxy-cinnamate
1.14.13.14
trans-2-Hydroxy-cinnamate
Tyrosine metabolism
CND00950
4.1.1.53
CNB03000...
CNB03030
K03334
K00285
K00824
trans-Cinnamate
CNB03000...
K05821
N-Acetyl-L-phenylalanine
K10797
1.3.1.11
K01904
Phenyl-ethylamine
Phenyl-propanoate
2-Hydroxyphenyl-propanoate
4-Coumaroyl-CoA
3-Hydroxyphenyl-propanoate
4-Hydroxy-benzoate
Phenylpyruvate
trans-3-Hydroxy-cinnamate
CNL05890
4.1.1.43
K12732
K07253
K05708...
K05708...
1.14.13.-
K21795
K03782
CNG01110
K13371...
1.1.1.222
K18606
K00457
K05712
CI-dihydro-diol
PP-dihydrodiol
K05712
1.14.13.4
Caffeoyl-CoA
Phenyl-acetaldehyde
2-Hydroxy-3-phenylpropanoate
Benzoate degradation
Phenyllactate
K00055
K05711
K05711
K00588
K00129
K00146
Phenyl-ethylalcohol
trans-2,3-Dihydroxy-cinnamate
2,3-Dihydroxy-phenylpropanoate
Feruloyl-CoA
2-Phenyl-acetamide
CNE01840...
K10437
2-Hydroxy-phenylacetate
Styrene degradation
K05713
K05713
K18383
Valine, leucine, isoleucine degradation
4-Hydroxy-phenylacetate
Phenyl-acetate
2,6-Dihydroxy-phenylacetate
2-Hydroxy-6-oxonona-trienedioate
2-Hydroxy-6-oxonona-2,4-diene-1,9-dioate
4-Hydroxy-3-methoxyphenyl-beta-hydroxypropionyl-CoA
K02614
3-Hydroxy-phenylacetate
K01912
K05714
K05714
Succinate
Citrate cycle
K18383
Isobutyryl-CoA
Phenyl-glyoxylyl-CoA
Phenyl-glyoxylate
Benzoyl-CoA
Phenyl-acetyl-CoA
K18361...
K18361...
K18355...
Benzoate degradation
cis-2-Hydroxypenta-2,4-dienoate
Vanillin
Malonyl-CoA
2-Oxepin-2(3H)-ylideneacetyl-CoA
3-Oxo-5,6-didehydro-suberyl-CoA
Fumarate
K02554
K02609...
K15866
K02618
K02618
K15517
K00628
2.3.1.14
2-(1,2-epoxy-1,2-dihydro-phenyl)acetyl-CoA
3-Oxo-5,6-dehydro-suberyl-CoA semialdehyde
K02615...
Acetyl-CoA
4-Hydroxy-2-oxopentanoate
Vanillylamine
8-Methyl-6-nonenoic acid
K01666
Pyruvate
Glycolysis
Phenyl-acetylglycine
Phenyl-acetylglutamine
3-Oxoadipyl-CoA
CNG04300
K01692
Acetaldehyde
(3S)-3-Hydroxy-adipyl-CoA
2,3-Didehydro-adipyl-CoA
Capsaicin
K02615
K04073
Succinyl-CoA
Citrate cycle
Acetyl-CoA
00360 3/17/17
(c) Kanehisa Laboratories

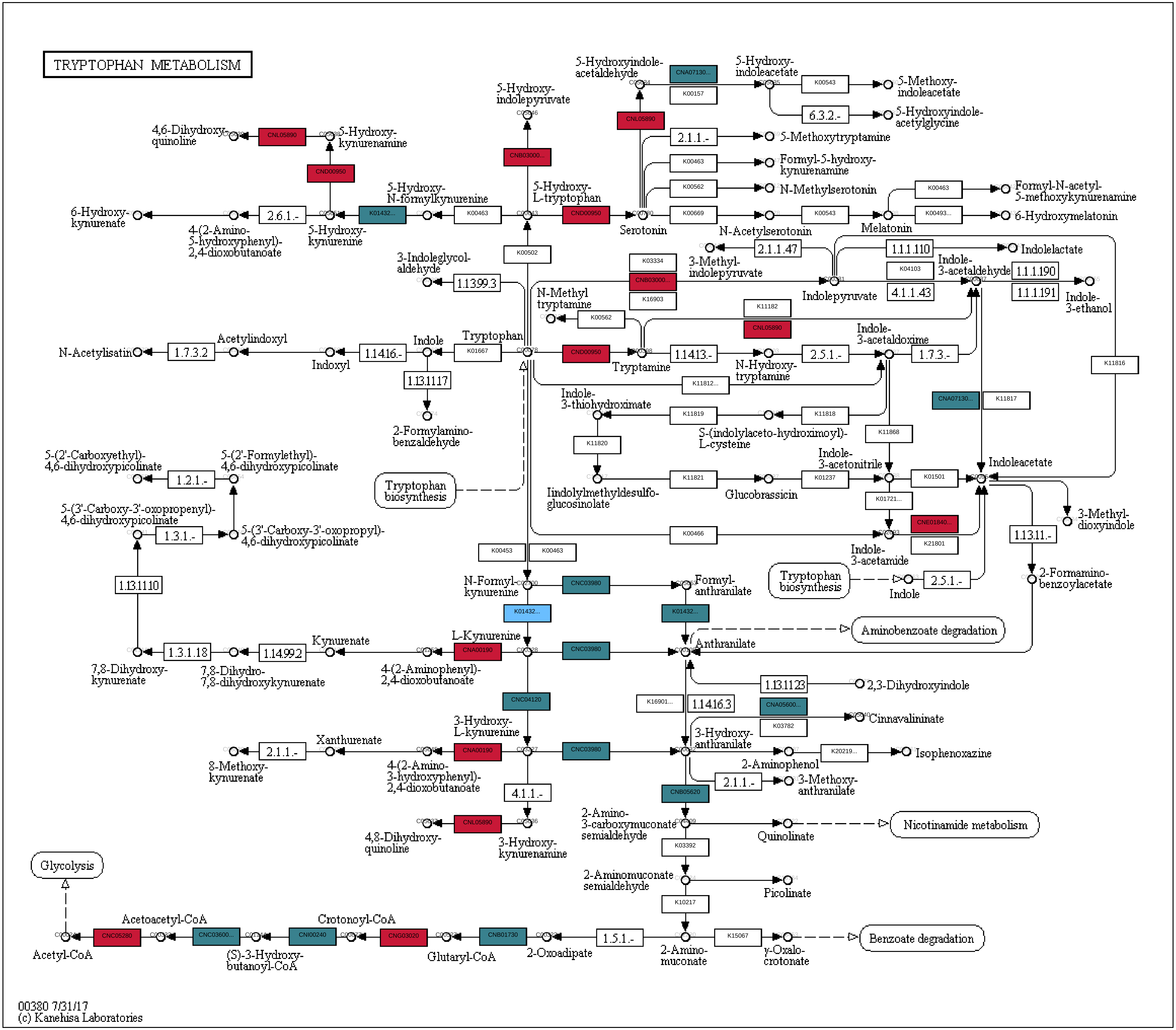
TRYPTOPHAN METABOLISM
5-Hydroxyindole-acetaldehyde
CNA07130...
K00157
5-Hydroxy-indoleacetate
K00543
5-Methoxy-indoleacetate
6.3.2.-
5-Hydroxyindole-acetylglycine
5-Hydroxy-indolepyruvate
CNL05890
2.1.1.-
5-Methoxytryptamine
K00463
Formyl-5-hydroxy-kynurenamine
K00562
N-Methylserotonin
K00669
K00543
K00463
Formyl-N-acetyl-5-methoxykynurenamine
K00493...
6-Hydroxymelatonin
Melatonin
N-Acetylserotonin
Serotonin
4,6-Dihydroxy-quinoline
CNL05890
5-Hydroxy-kynurenamine
CNB03000...
CND00950
5-Hydroxy-N-formylkynurenine
5-Hydroxy-L-tryptophan
6-Hydroxy-kynurenate
2.6.1.-
K01432...
K00463
CND00950
4-(2-Amino-5-hydroxyphenyl)-2,4-dioxobutanoate
5-Hydroxy-kynurenine
K00502
3-Indoleglycol-aldehyde
1.1.3.99.3
K03334
CNB03000...
K16903
3-Methyl-indolepyruvate
2.1.1.47
1.1.1.110
Indolelactate
K04103
Indole-3-acetaldehyde
1.1.1.190
4.1.1.43
1.1.1.191
Indolepyruvate
Indole-3-ethanol
N-Methyl tryptamine
K00562
K11182
CNL05890
Acetylindoxyl
Indole
Tryptophan
N-Acetylisatin
1.7.3.2
1.14.16.-
K01667
CND00950
1.14.13.-
2.5.1.-
Indole-3-acetaldoxime
1.7.3.-
K11816
Indoxyl
1.13.11.17
Tryptamine
N-Hydroxy-tryptamine
K11812...
2-Formylamino-benzaldehyde
Indole-3-thiohydroximate
K11819
K11818
S-(indolylaceto-hydroximoyl)-L-cysteine
CNA07130...
K11817
K11868
K11820
5-(2'-Carboxyethyl)-4,6-dihydroxypicolinate
1.2.1.-
5-(2'-Formylethyl)-4,6-dihydroxypicolinate
Tryptophan biosynthesis
Iindolylmethyldesulfo-glucosinolate
K11821
Glucobrassicin
K01237
Indole-3-acetonitrile
K01501
Indoleacetate
K01721...
CNE01840...
3-Methyl-dioxyindole
5-(3'-Carboxy-3'-oxopropenyl)-4,6-dihydroxypicolinate
1.3.1.-
5-(3'-Carboxy-3'-oxopropyl)-4,6-dihydroxypicolinate
K00466
K21801
Indole-3-acetamide
K00453
K00463
1.13.11.-
1.13.11.10
N-Formyl-kynurenine
CNC03980
Formyl-anthranilate
Tryptophan biosynthesis
2.5.1.-
Indole
2-Formamino-benzoylacetate
K01432...
K01432...
Aminobenzoate degradation
Anthranilate
7,8-Dihydroxy-kynurenate
1.3.1.18
7,8-Dihydro-7,8-dihydroxykynurenate
1.14.99.2
Kynurenate
CNA00190
L-Kynurenine
CNC03980
4-(2-Aminophenyl)-2,4-dioxobutanoate
1.13.11.23
2,3-Dihydroxyindole
K16901...
1.14.16.3
CNA05600...
CNC04120
Cinnavalininate
K03782
3-Hydroxy-L-kynurenine
3-Hydroxy-anthranilate
8-Methoxy-kynurenate
2.1.1.-
Xanthurenate
CNA00190
CNC03980
K20219...
Isophenoxazine
2-Aminophenol
4-(2-Amino-3-hydroxyphenyl)-2,4-dioxobutanoate
4.1.1.-
2.1.1.-
3-Methoxy-anthranilate
CNB05620
CNL05890
4,8-Dihydroxy-quinoline
3-Hydroxy-kynurenamine
2-Amino-3-carboxymuconate semialdehyde
Quinolinate
Nicotinamide metabolism
K03392
Glycolysis
2-Aminomuconate semialdehyde
Picolinate
K10217
Acetoacetyl-CoA
Crotonoyl-CoA
CNC05280
CNC03600...
CNI00240
CNG03020
CNB01730
1.5.1.-
K15067
Benzoate degradation
Acetyl-CoA
(S)-3-Hydroxy-butanoyl-CoA
Glutaryl-CoA
2-Oxoadipate
2-Amino-muconate
γ-Oxalo-crotonate
00380 7/31/17
(c) Kanehisa Laboratories

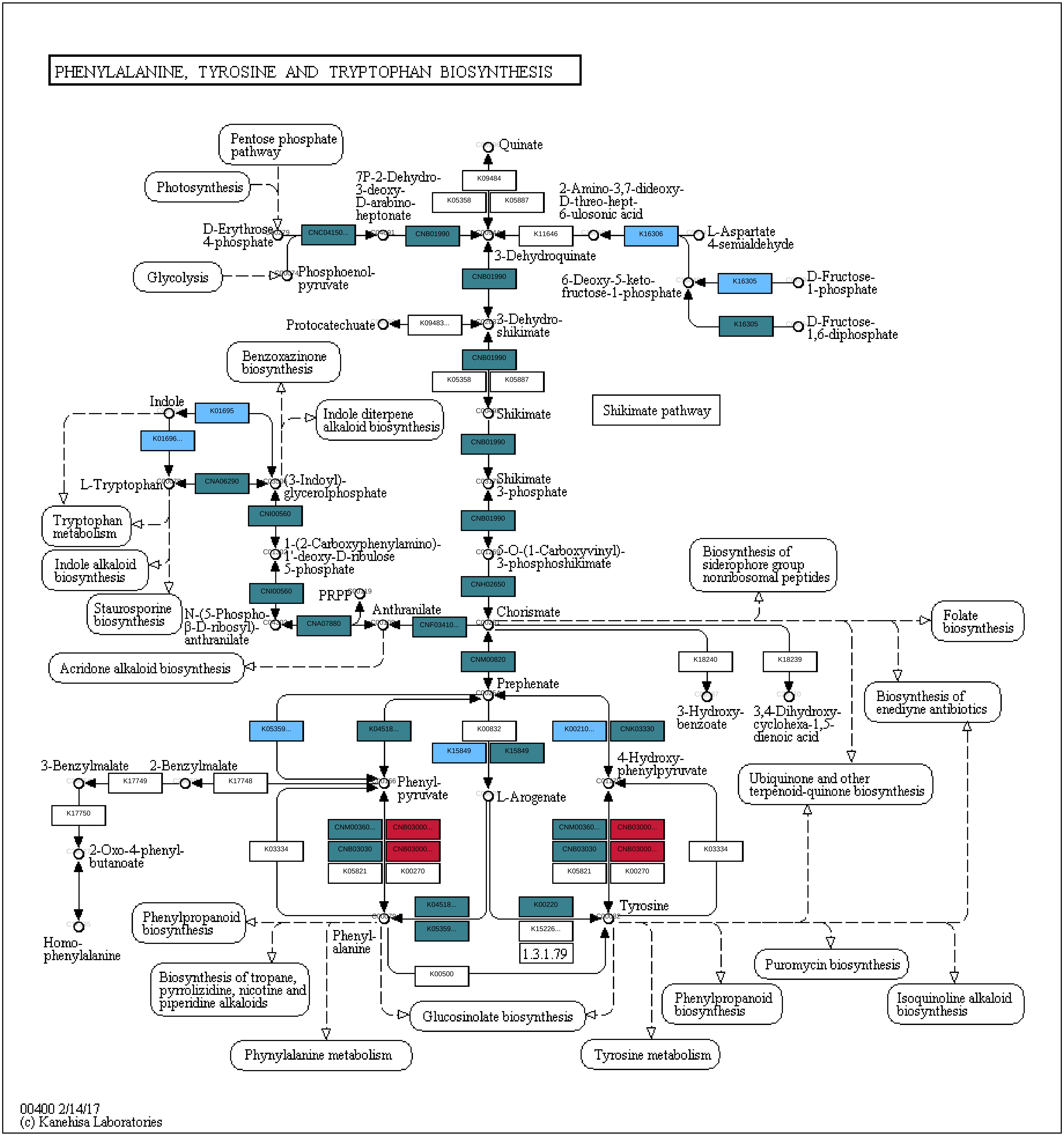
PHENYLALANINE, TYROSINE AND TRYPTOPHAN BIOSYNTHESIS
Pentose phosphate pathway
Photosynthesis
Glycolysis
D-Erythrose 4-phosphate
Phosphoenol-pyruvate
CNC04150...
7P-2-Dehydro-3-deoxy-D-arabino-heptonate
CNB01990
Quinate
K09484
K05358
K05887
3-Dehydroquinate
K11646
2-Amino-3,7-dideoxy-D-threo-hept-6-ulosonic acid
K16306
L-Aspartate 4-semialdehyde
6-Deoxy-5-keto-fructose-1-phosphate
K16305
D-Fructose-1-phosphate
D-Fructose-1,6-diphosphate
CNB01990
Protocatechuate
K09483...
3-Dehydro-shikimate
CNB01990
K05358
K05887
Shikimate
Shikimate pathway
Benzoxazinone biosynthesis
Indole diterpene alkaloid biosynthesis
Indole
K01695
K01696...
L-Tryptophan
CNA06290
(3-Indoyl)-glycerolphosphate
CNI00560
1-(2-Carboxyphenylamino)-1'-deoxy-D-ribulose 5-phosphate
CNI00560
Tryptophan metabolism
Indole alkaloid biosynthesis
Staurosporine biosynthesis
N-(5-Phospho-β-D-ribosyl)-anthranilate
CNA07880
PRPP
Anthranilate
CNF03410...
Acridone alkaloid biosynthesis
CNB01990
Shikimate 3-phosphate
CNB01990
5-O-(1-Carboxyvinyl)-3-phosphoshikimate
CNH02650
Chorismate
Biosynthesis of siderophore group nonribosomal peptides
Folate biosynthesis
CNM00820
K18240
K18239
Prephenate
3-Hydroxy-benzoate
3,4-Dihydroxy-cyclohexa-1,5-dienoic acid
Biosynthesis of enediyne antibiotics
K05359...
K04518...
K00832
K15849
K15849
K00210...
CNK03330
4-Hydroxy-phenylpyruvate
Ubiquinone and other terpenoid-quinone biosynthesis
3-Benzylmalate
2-Benzylmalate
K17749
K17748
K17750
Phenyl-pyruvate
L-Arogenate
2-Oxo-4-phenyl-butanoate
Homo-phenylalanine
K03334
CNM00360...
CNB03000...
CNB03030
CNB03000...
K05821
K00270
CNM00360...
CNB03000...
CNB03030
CNB03000...
K05821
K00270
K03334
K04518...
K05359...
K00220
K15226...
1.3.1.79
Tyrosine
Phenylpropanoid biosynthesis
Phenyl-alanine
K00500
Biosynthesis of tropane, pyrrolizidine, nicotine and piperidine alkaloids
Glucosinolate biosynthesis
Phynylalanine metabolism
Tyrosine metabolism
Phenylpropanoid biosynthesis
Puromycin biosynthesis
Isoquinoline alkaloid biosynthesis
00400 2/14/17
(c) Kanehisa Laboratories

# 6. Metabolism of other amino acids

| MAP | PATHWAY |
|---|---|
| **410** | beta-Alanine metabolism |
| **430** | Taurine and hypotaurine metabolism |
| **440** | Phosphonate and phosphinate metabolism |
| **450** | Selenocompound metabolism |
| **460** | Cyanoamino acid metabolism |
| **480** | Glutathione metabolism |

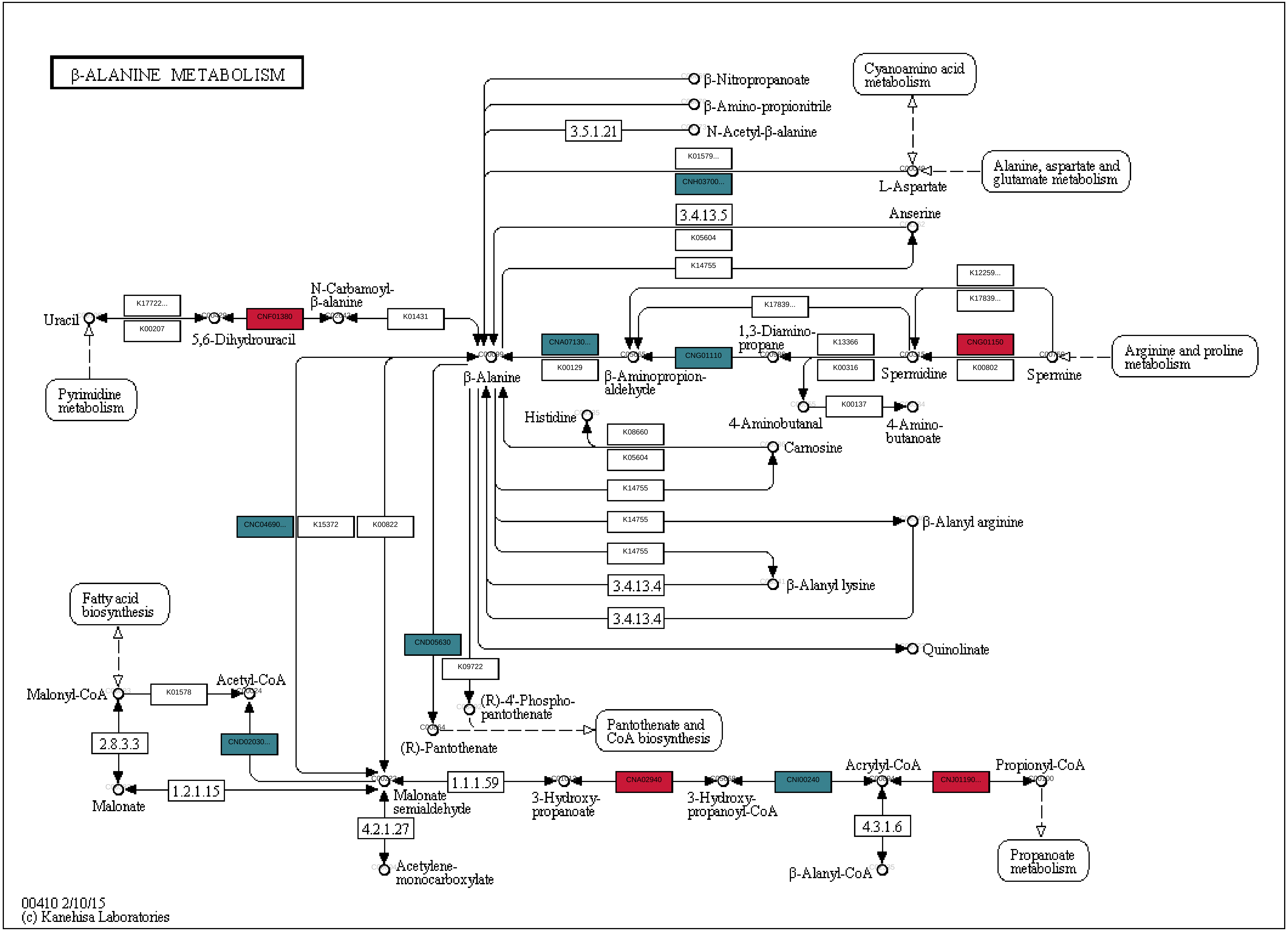

β-ALANINE METABOLISM
β-Nitropropanoate
β-Amino-propionitrile
3.5.1.21
N-Acetyl-β-alanine
Cyanoamino acid metabolism
K01579...
CNH03700...
L-Aspartate
Alanine, aspartate and glutamate metabolism
3.4.13.5
Anserine
K05604
K14755
K12259...
K17839...
K17839...
Uracil
K17722...
K00207
CNF01380
N-Carbamoyl-β-alanine
K01431
5,6-Dihydrouracil
CNA07130...
K00129
CNG01110
1,3-Diamino-propane
K13366
K00316
CNG01150
K00802
β-Alanine
β-Aminopropion-aldehyde
Spermidine
Spermine
Arginine and proline metabolism
Pyrimidine metabolism
Histidine
K08660
K05604
4-Aminobutanal
K00137
4-Amino-butanoate
Carnosine
K14755
K14755
β-Alanyl arginine
K14755
CNC04690...
K15372
K00822
3.4.13.4
β-Alanyl lysine
3.4.13.4
Quinolinate
Fatty acid biosynthesis
CND05630
K09722
(R)-4'-Phospho-pantothenate
Malonyl-CoA
K01578
Acetyl-CoA
Pantothenate and CoA biosynthesis
(R)-Pantothenate
2.8.3.3
CND02030...
1.2.1.15
Malonate
1.1.1.59
Malonate semialdehyde
CNA02940
3-Hydroxy-propanoate
CNI00240
3-Hydroxy-propanoyl-CoA
Acrylyl-CoA
CNJ01190...
Propionyl-CoA
4.2.1.27
4.3.1.6
Acetylene-monocarboxylate
β-Alanyl-CoA
Propanoate metabolism
00410 2/10/15
(c) Kanehisa Laboratories

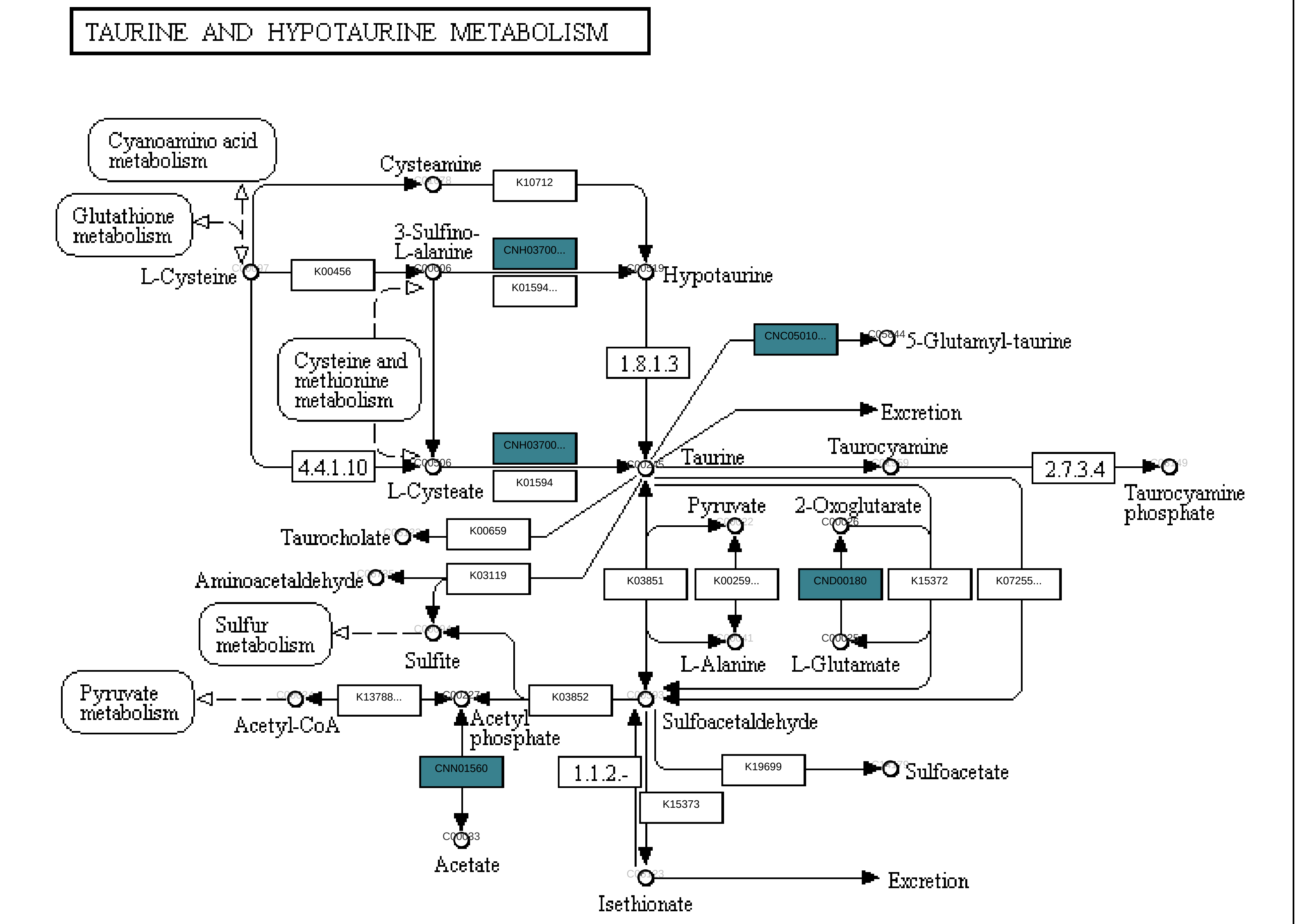


00430 11/18/15
(c) Kanehisa Laboratories

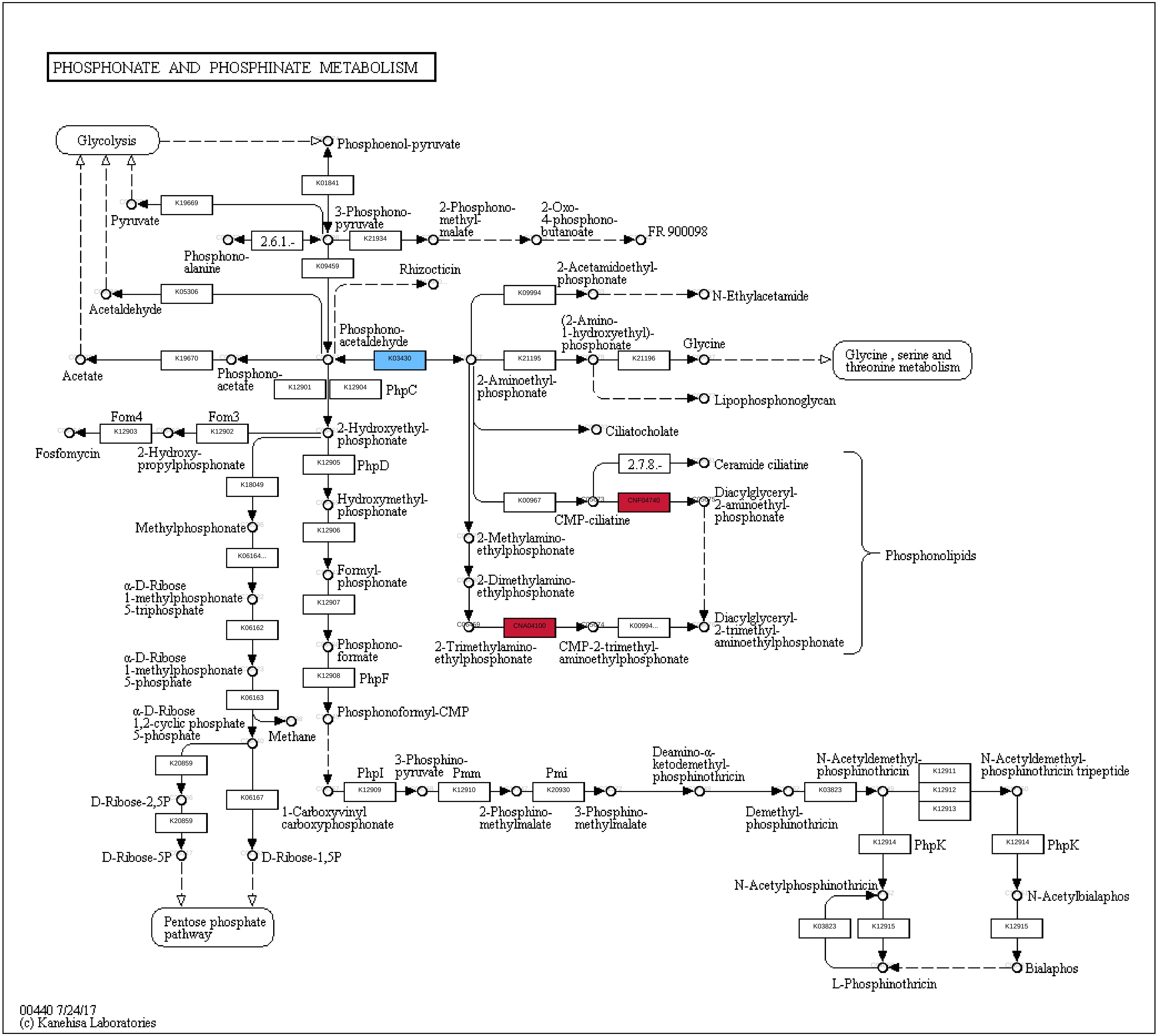
PHOSPHONATE AND PHOSPHINATE METABOLISM
Glycolysis
Phosphoenol-pyruvate
K01841
K19669
Pyruvate
3-Phosphono-pyruvate
2-Phosphono-methyl-malate
2-Oxo-4-phosphono-butanoate
FR 900098
2.6.1.-
K21934
Phosphono-alanine
K09459
Rhizocticin
K05306
Acetaldehyde
2-Acetamidoethyl-phosphonate
K09994
N-Ethylacetamide
Phosphono-acetaldehyde
(2-Amino-1-hydroxyethyl)-phosphonate
Glycine
Glycine , serine and threonine metabolism
K19670
K03430
K21195
K21196
Acetate
Phosphono-acetate
K12901
K12904
PhpC
2-Aminoethyl-phosphonate
Lipophosphonoglycan
Fom4
Fom3
K12903
K12902
2-Hydroxyethyl-phosphonate
Ciliatocholate
Fosfomycin
2-Hydroxy-propylphosphonate
K12905
PhpD
2.7.8.-
Ceramide ciliatine
K18049
Hydroxymethyl-phosphonate
K00967
CNF04740
Diacylglyceryl-2-aminoethyl-phosphonate
CMP-ciliatine
Methylphosphonate
K12906
2-Methylamino-ethylphosphonate
Phospholipids
K06164...
Formyl-phosphonate
2-Dimethylamino-ethylphosphonate
α-D-Ribose 1-methylphosphonate 5-triphosphate
K12907
K06162
Phosphono-formate
CNA04100
K00994...
Diacylglyceryl-2-trimethyl-aminoethylphosphonate
2-Trimethylamino-ethylphosphonate
CMP-2-trimethyl-aminoethylphosphonate
α-D-Ribose 1-methylphosphonate 5-phosphate
K12908
PhpF
K06163
Phosphonoformyl-CMP
α-D-Ribose 1,2-cyclic phosphate 5-phosphate
Methane
3-Phosphino-pyruvate
Deamino-α-ketodemethyl-phosphinothricin
N-Acetyldemethyl-phosphinothricin
N-Acetyldemethyl-phosphinothricin tripeptide
K20859
PhpI
Prnn
Pmi
K12909
K12910
K20930
K03823
K12911
K12912
K12913
D-Ribose-2,5P
K06167
1-Carboxyvinyl carboxyphosphonate
2-Phosphino-methylmalate
3-Phosphino-methylmalate
Demethyl-phosphinothricin
K20859
K12914
PhpK
K12914
PhpK
D-Ribose-5P
D-Ribose-1,5P
N-Acetylphosphinothricin
N-Acetylbialaphos
K03823
K12915
K12915
Pentose phosphate pathway
L-Phosphinothricin
Bialaphos
00440 7/24/17
(c) Kanehisa Laboratories

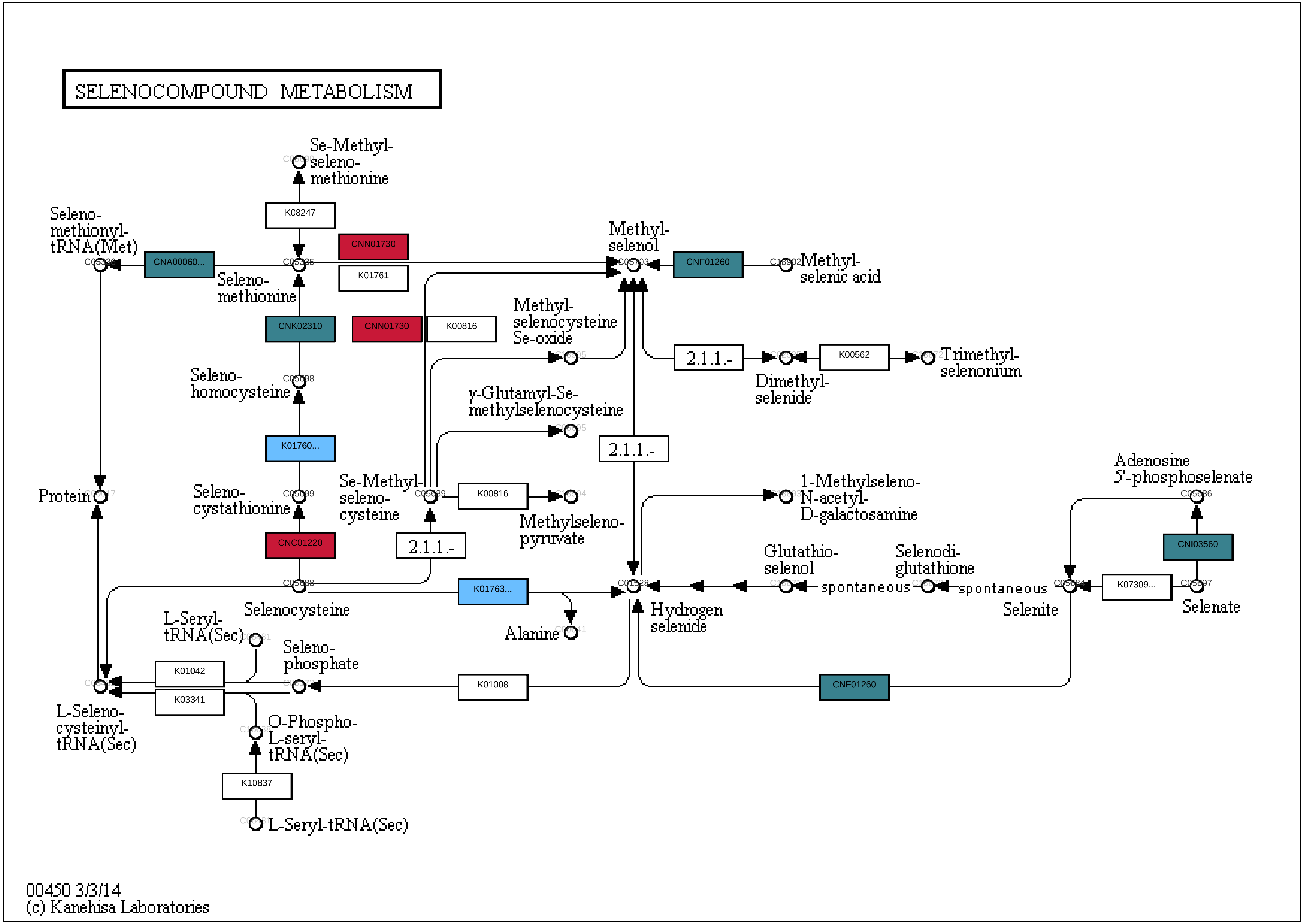
SELENOCOMPOUND METABOLISM
Se-Methyl-selenomethionine
K08247
Seleno-methionyl-tRNA(Met)
CNA00060...
Selenomethionine
C05335
CNN01730
K01761
Methyl-selenol
C05703
CNF01260
C18902
Methyl-selenic acid
CNK02310
CNN01730
K00816
Methyl-selenocysteine Se-oxide
2.1.1.-
K00562
Dimethyl-selenide
Trimethyl-selenonium
Seleno-homocysteine
C05698
γ-Glutamyl-Se-methylselenocysteine
K01760...
2.1.1.-
Adenosine 5'-phosphoselenate
C05696
Seleno-cystathionine
C05699
Se-Methyl-selenocysteine
C05689
K00816
Methylseleno-pyruvate
1-Methylseleno-N-acetyl-D-galactosamine
Protein
CNC01220
2.1.1.-
CNI03560
Glutathio-selenol
Selenodi-glutathione
C05688
K01763...
C01528
spontaneous
spontaneous
C05684
K07309...
C05697
Selenocysteine
Hydrogen selenide
Selenite
Selenate
L-Seryl-tRNA(Sec)
Alanine
Seleno-phosphate
K01042
K03341
K01008
CNF01260
L-Seleno-cysteinyl-tRNA(Sec)
O-Phospho-L-seryl-tRNA(Sec)
K10837
L-Seryl-tRNA(Sec)
00450 3/3/14
(c) Kanehisa Laboratories

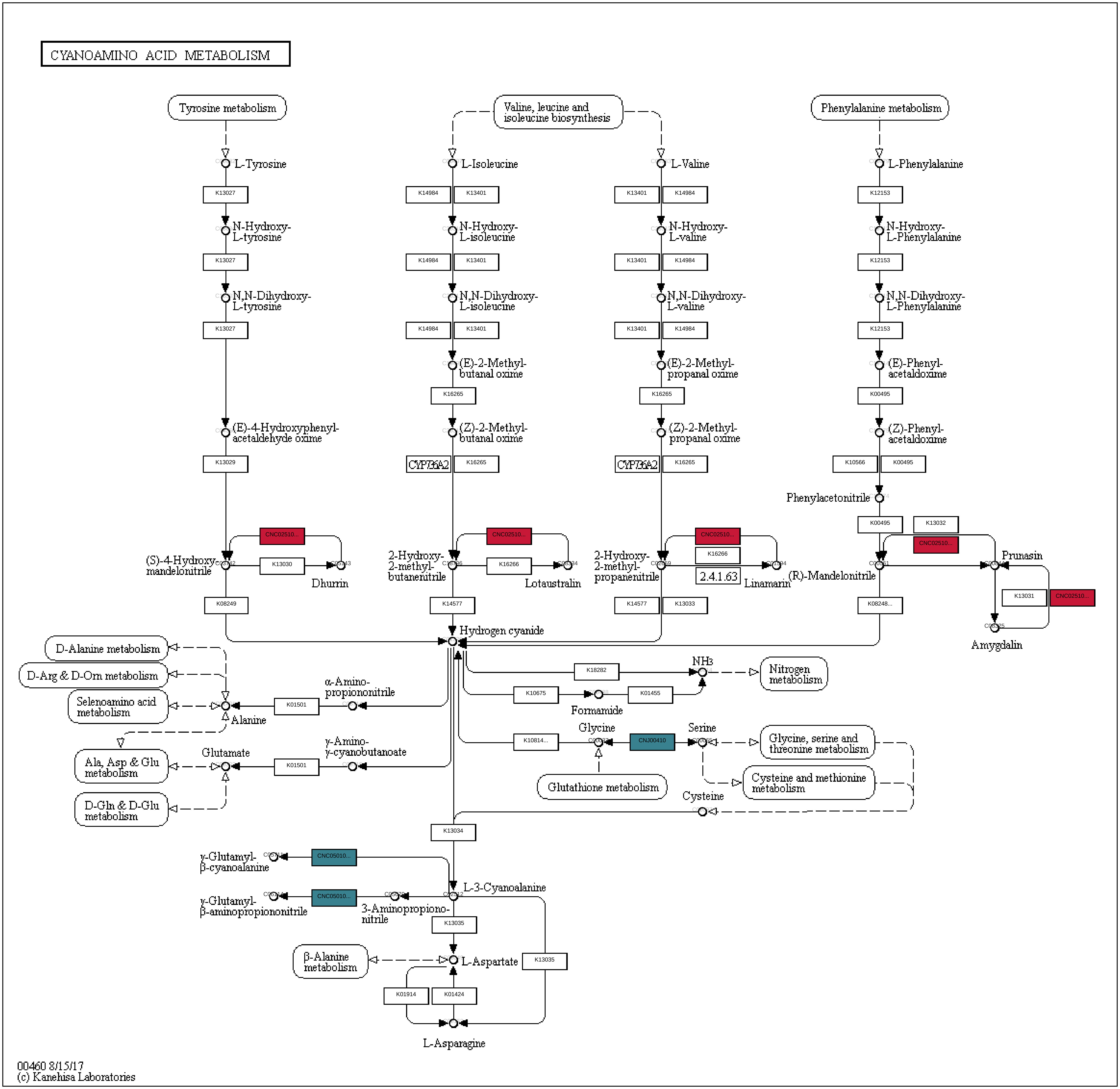
CYANOAMINO ACID METABOLISM
Tyrosine metabolism
Valine, leucine and isoleucine biosynthesis
Phenylalanine metabolism
L-Tyrosine
L-Isoleucine
L-Valine
L-Phenylalanine
K13027
K14984
K13401
K12153
N-Hydroxy-L-tyrosine
N-Hydroxy-L-isoleucine
N-Hydroxy-L-valine
N-Hydroxy-L-Phenylalanine
N,N-Dihydroxy-L-tyrosine
N,N-Dihydroxy-L-isoleucine
N,N-Dihydroxy-L-valine
N,N-Dihydroxy-L-Phenylalanine
(E)-2-Methyl-butanal oxime
(E)-2-Methyl-propanal oxime
(E)-Phenyl-acetaldoxime
K16265
K00495
(E)-4-Hydroxyphenyl-acetaldehyde oxime
(Z)-2-Methyl-butanal oxime
(Z)-2-Methyl-propanal oxime
(Z)-Phenyl-acetaldoxime
K13029
CYP736A2
K10566
Phenylacetonitrile
K13032
CNC02510...
K13030
K16266
2.4.1.63
(S)-4-Hydroxy-mandelonitrile
Dhurrin
2-Hydroxy-2-methyl-butanenitrile
Lotaustralin
2-Hydroxy-2-methyl-propanenitrile
Linamarin
(R)-Mandelonitrile
Prunasin
K13031
Amygdalin
K08249
K14577
K13033
K08248...
Hydrogen cyanide
D-Alanine metabolism
D-Arg & D-Orn metabolism
Selenoamino acid metabolism
α-Amino-propiononitrile
K01501
Alanine
K18282
NH3
Nitrogen metabolism
K10675
K01455
Formamide
Glycine
Serine
K10814...
CNJ00410
Glycine, serine and threonine metabolism
Glutamate
γ-Amino-γ-cyanobutanoate
Ala, Asp & Glu metabolism
D-Gln & D-Glu metabolism
Glutathione metabolism
Cysteine and methionine metabolism
Cysteine
K13034
γ-Glutamyl-β-cyanoalanine
CNC05010...
L-3-Cyanoalanine
γ-Glutamyl-β-aminopropiononitrile
3-Aminopropiono-nitrile
K13035
β-Alanine metabolism
L-Aspartate
K01914
K01424
L-Asparagine
00460 8/15/17
(c) Kanehisa Laboratories

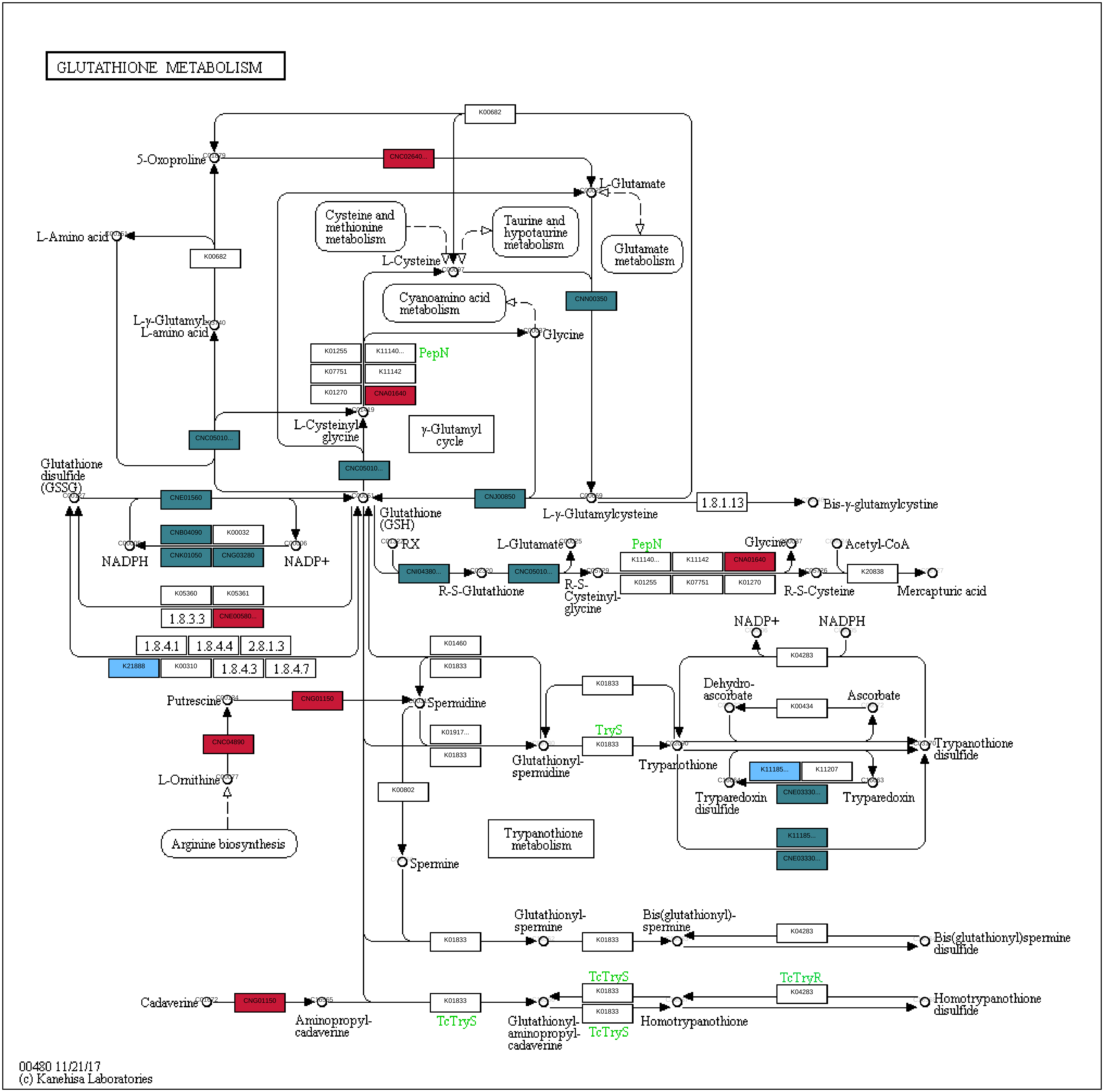
GLUTATHIONE METABOLISM
K00682
5-Oxoproline
CNC02640...
L-Glutamate
Cysteine and methionine metabolism
Taurine and hypotaurine metabolism
Glutamate metabolism
L-Amino acid
K00682
L-Cysteine
Cyanoamino acid metabolism
CNN00350
L-γ-Glutamyl-L-amino acid
Glycine
K01255
K11140...
PepN
K07751
K11142
K01270
CNA01640
L-Cysteinyl-glycine
γ-Glutamyl cycle
CNC05010...
CNC05010...
Glutathione disulfide (GSSG)
CNE01560
CNJ00850
L-γ-Glutamylcysteine
1.8.1.13
Bis-γ-glutamylcystine
Glutathione (GSH)
RX
L-Glutamate
PepN
Glycine
Acetyl-CoA
CNB04090
K00032
CNK01050
CNG03280
NADPH
NADP+
CNI04380...
CNC05010...
K11140...
K11142
CNA01640
K20838
R-S-Glutathione
R-S-Cysteinyl-glycine
K01255
K07751
K01270
R-S-Cysteine
Mercapturic acid
K05360
K05361
1.8.3.3
CNE00580...
NADP+
NADPH
1.8.4.1
1.8.4.4
2.8.1.3
K01460
K04283
K21888
K00310
1.8.4.3
1.8.4.7
K01833
Putrescine
CNG01150
Spermidine
K01833
Dehydro-ascorbate
K00434
Ascorbate
CNC04890
TryS
K01917...
K01833
Trypanothione disulfide
K01833
Glutathionyl-spermidine
Trypanothione
K11185...
K11207
L-Ornithine
K00802
CNE03330...
Trypanothione metabolism
Tryparedoxin disulfide
Tryparedoxin
Arginine biosynthesis
K11185...
CNE03330...
Spermine
Glutathionyl-spermine
Bis(glutathionyl)-spermine
K04283
K01833
K01833
Bis(glutathionyl)spermine disulfide
TcTryS
TcTryR
K01833
K04283
Cadaverine
CNG01150
K01833
Homotrypanothione disulfide
K01833
Aminopropyl-cadaverine
TcTryS
Glutathionyl-aminopropyl-cadaverine
TcTryS
Homotrypanothione
00480 11/21/17
(c) Kanehisa Laboratories

# 7. Glycan biosynthesis and metabolism

| MAP | PATHWAY |
|---|---|
| **510** | N-Glycan biosynthesis |
| **513** | Various types of N-glycan biosynthesis |
| **531** | Glycosaminoglycan degradation |
| **563** | Glycosylphosphatidylinositol (GPI)-anchor biosynthesis |

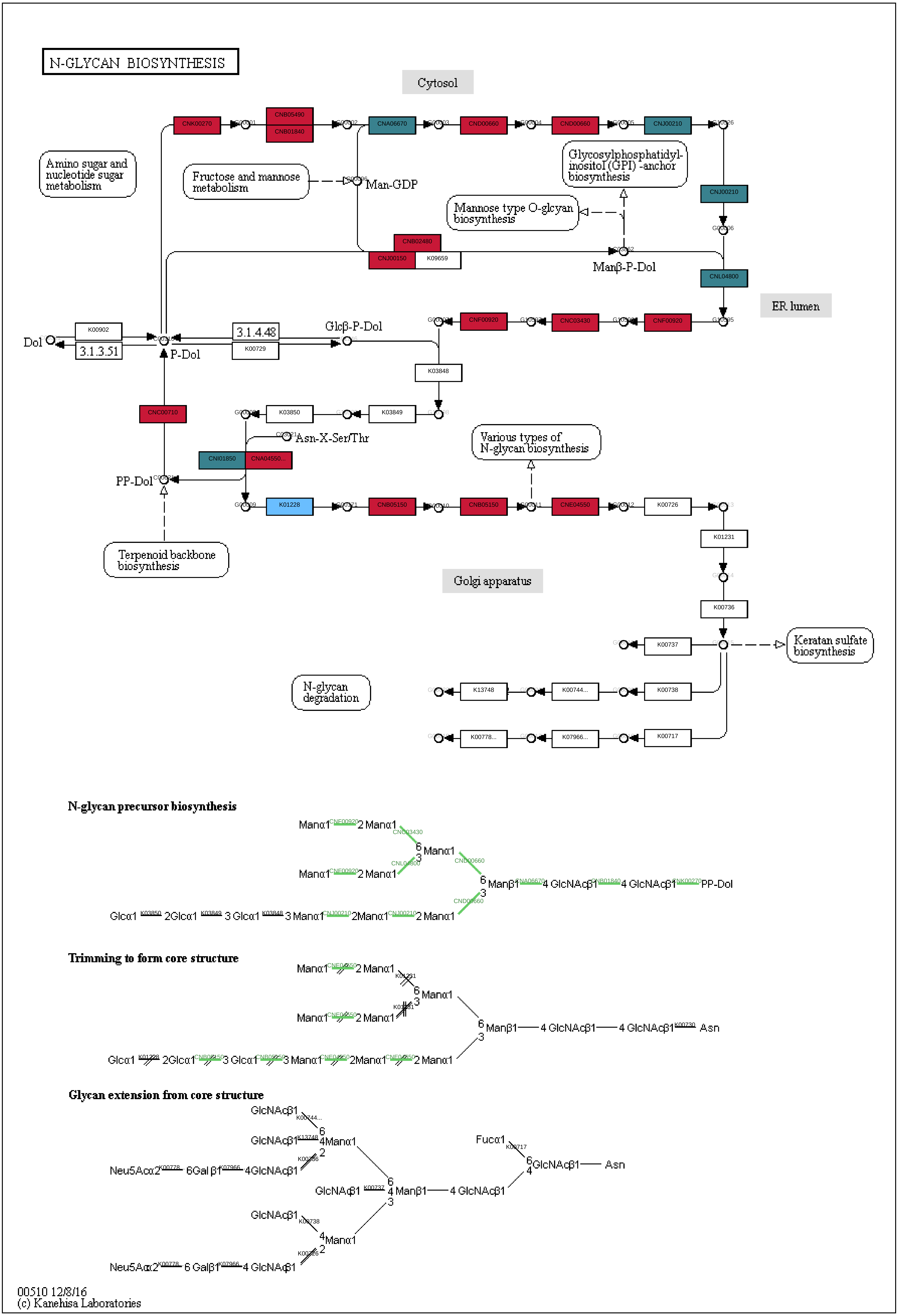

N-GLYCAN BIOSYNTHESIS
Cytosol
ER lumen
Golgi apparatus
Amino sugar and nucleotide sugar metabolism
Fructose and mannose metabolism
Glycosylphosphatidyl-inositol (GPI) -anchor biosynthesis
Mannose type O-glcyan biosynthesis
Various types of N-glycan biosynthesis
Terpenoid backbone biosynthesis
Keratan sulfate biosynthesis
N-glycan degradation
Man-GDP
Manβ-P-Dol
Glcβ-P-Dol
Dol
P-Dol
PP-Dol
Asn-X-Ser/Thr
3.1.4.48
3.1.3.51
CNK00270
CNB05490
CNB01840
CNA06670
CND00660
CNJ00210
CNB02480
CNJ00150
K09659
CNL04800
CNF00920
CNC03430
K00902
K00729
K03848
K03850
K03849
CNC00710
CNI01850
CNA04550...
K01228
CNB05150
CNE04550
K00726
K01231
K00736
K00737
K00738
K00744...
K13748
K00717
K07966...
K00778...
N-glycan precursor biosynthesis
Trimming to form core structure
Glycan extension from core structure
00510 12/8/16
(c) Kanehisa Laboratories

# VARIOUS TYPES OF N-GLYCAN BIOSYNTHESIS

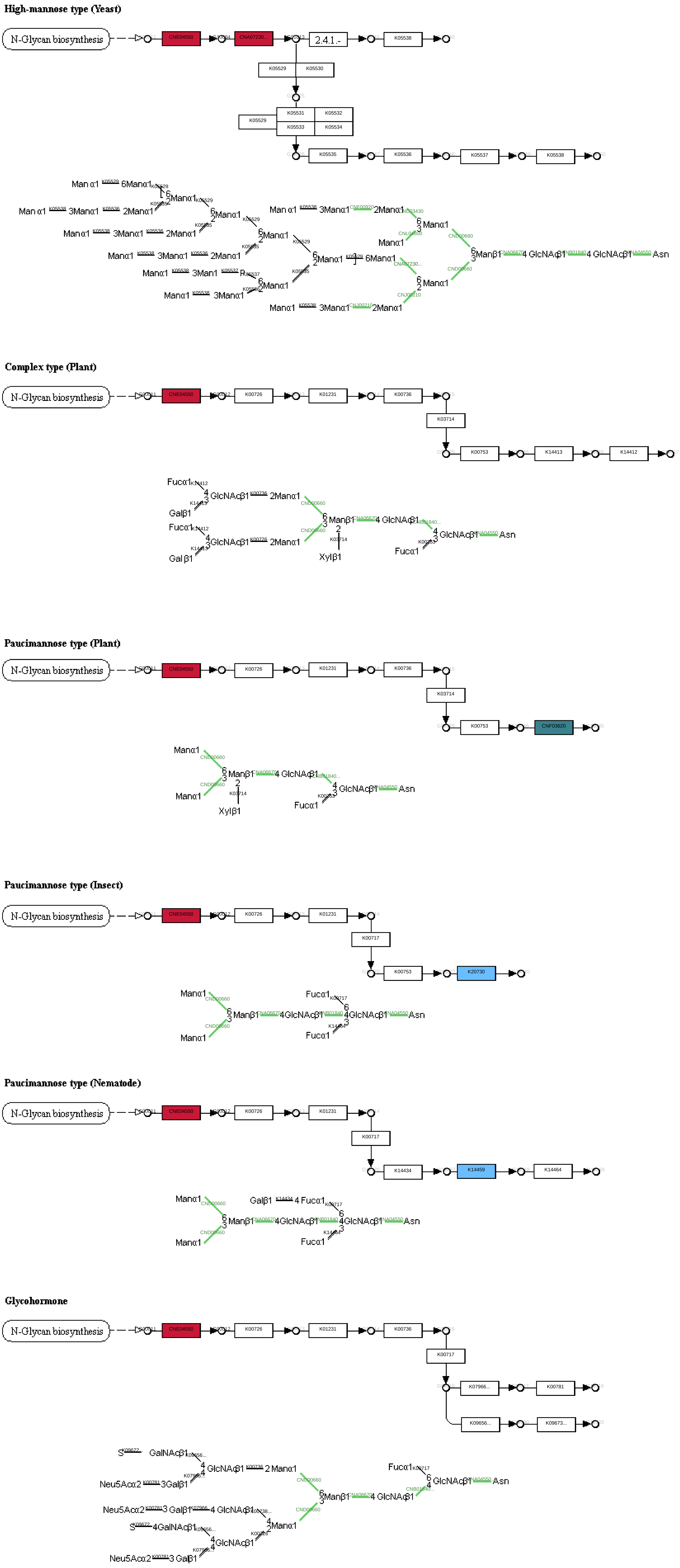


00513 9/7/16
(c) Kanehisa Laboratories

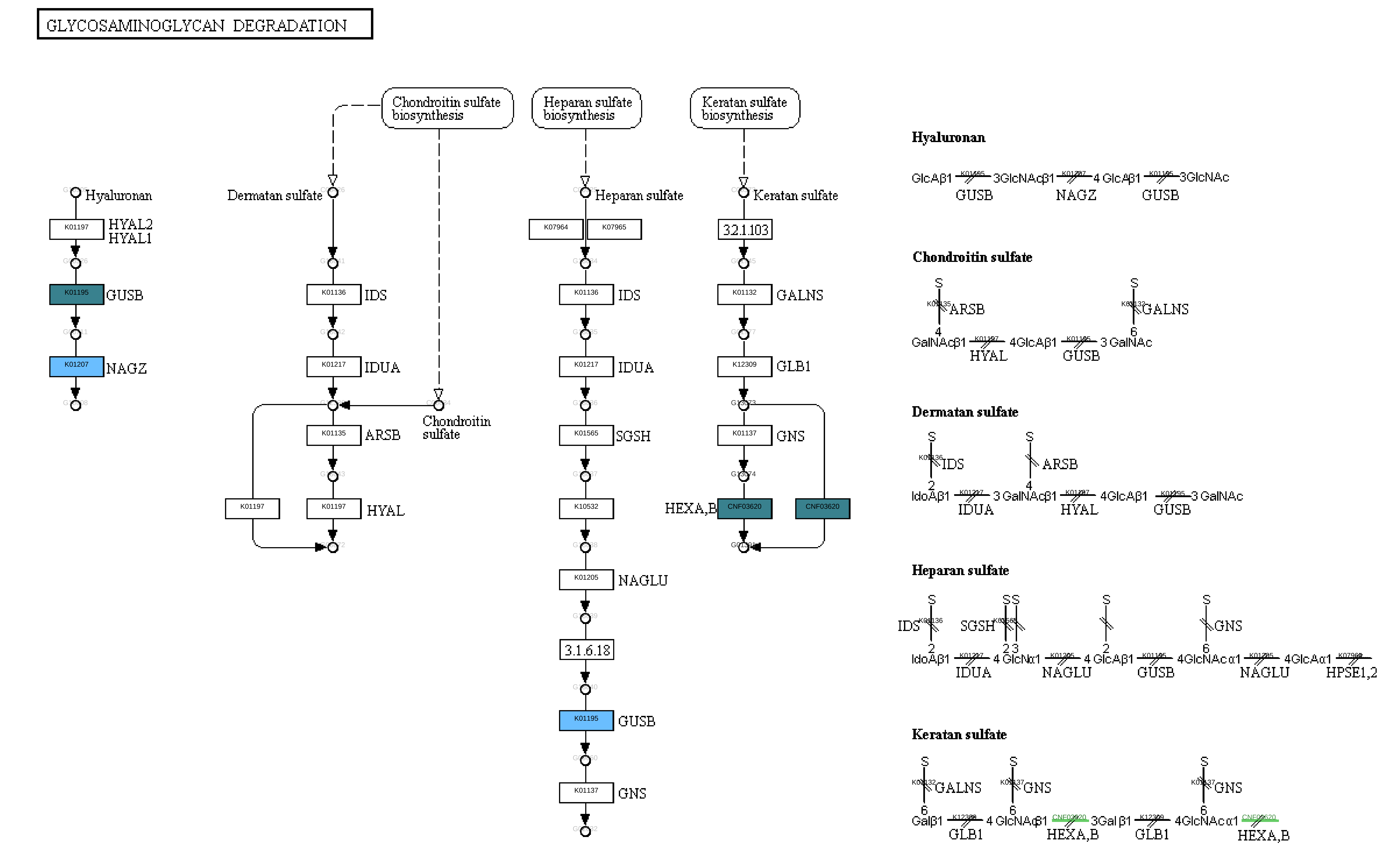


00531 5/22/15

(c) Kanehisa Laboratories

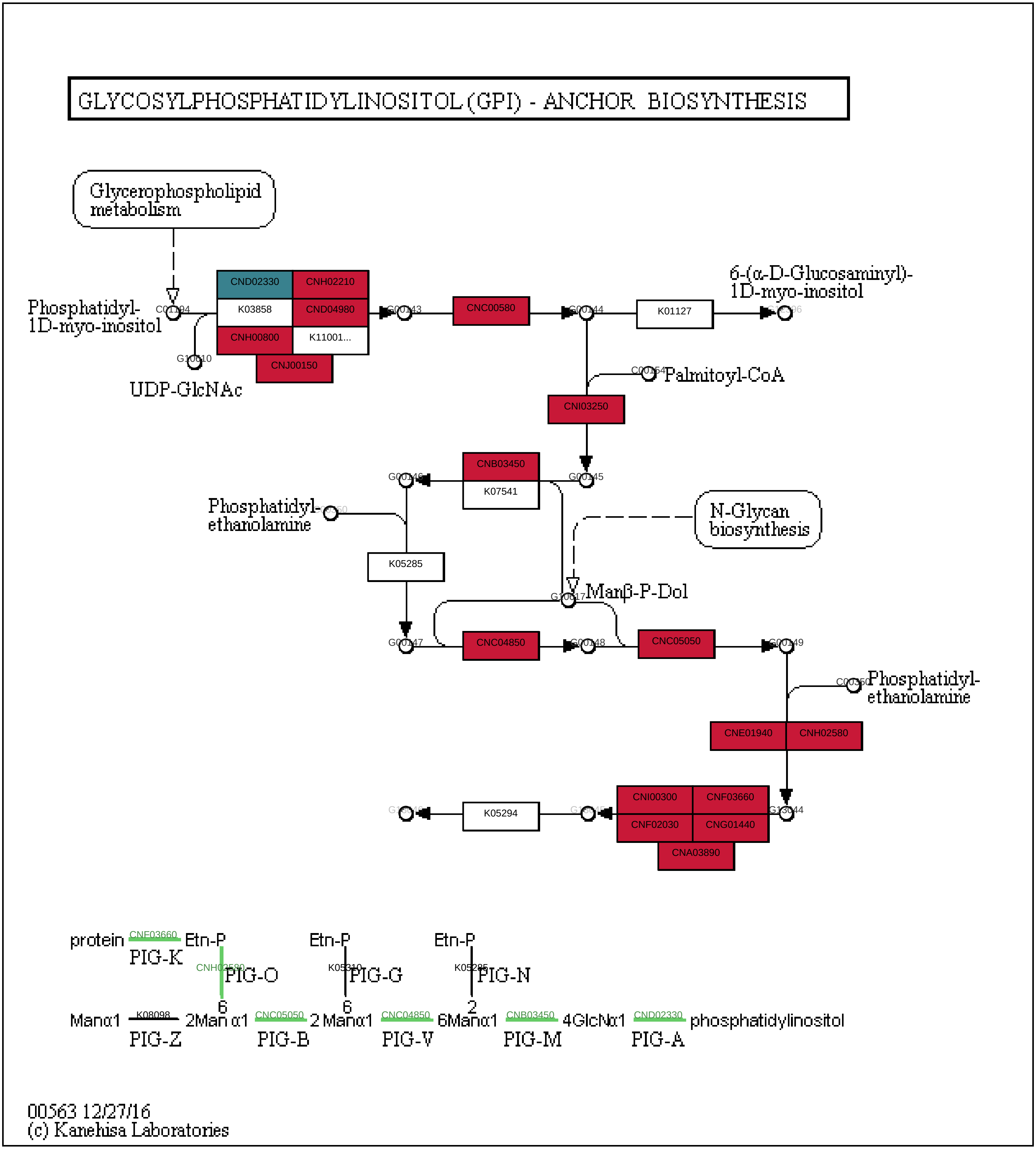
GLYCOSYLPHOSPHATIDYLINOSITOL (GPI) - ANCHOR BIOSYNTHESIS
Glycerophospholipid metabolism
Phosphatidyl-1D-myo-inositol
C01194
G10610
UDP-GlcNAc
CND02330
CNH02210
K03858
CND04980
CNH00800
K11001...
CNJ00150
G00143
CNC00580
G00144
K01127
6-(α-D-Glucosaminyl)-1D-myo-inositol
C00154
Palmitoyl-CoA
CNI03250
CNB03450
K07541
G00146
G00145
Phosphatidyl-ethanolamine
N-Glycan biosynthesis
K05285
Manβ-P-Dol
G10617
G00147
CNC04850
G00148
CNC05050
G00149
C00350
Phosphatidyl-ethanolamine
CNE01940
CNH02580
CNI00300
CNF03660
CNF02030
CNG01440
CNA03890
G13044
K05294
protein
CNF03660
Etn-P
PIG-K
CNH02580
PIG-O
Etn-P
K05310
PIG-G
Etn-P
K05285
PIG-N
6
6
2
Manα1
K08098
2Man α1
CNC05050
2 Manα1
CNC04850
6Manα1
CNB03450
4GlcNα1
CND02330
phosphatidylinositol
PIG-Z
PIG-B
PIG-V
PIG-M
PIG-A
00563 12/27/16
(c) Kanehisa Laboratories

# 8. Metabolism of cofactors and vitamins

| MAP | PATHWAY |
|---|---|
| **130** | Ubiquinone and other terpenoid-quinone biosynthesis |
| **670** | One carbon pool by folate |
| **730** | Thiamine metabolism |
| **740** | Riboflavin metabolism |
| **750** | Vitamin B6 metabolism |
| **760** | Nicotinate and nicotinamide metabolism |
| **770** | Pantothenate and CoA biosynthesis |
| **780** | Biotin metabolism |
| **785** | Lipoic acid metabolism |
| **790** | Folate biosynthesis |
| **860** | Porphyrin and chlorophyll metabolism |

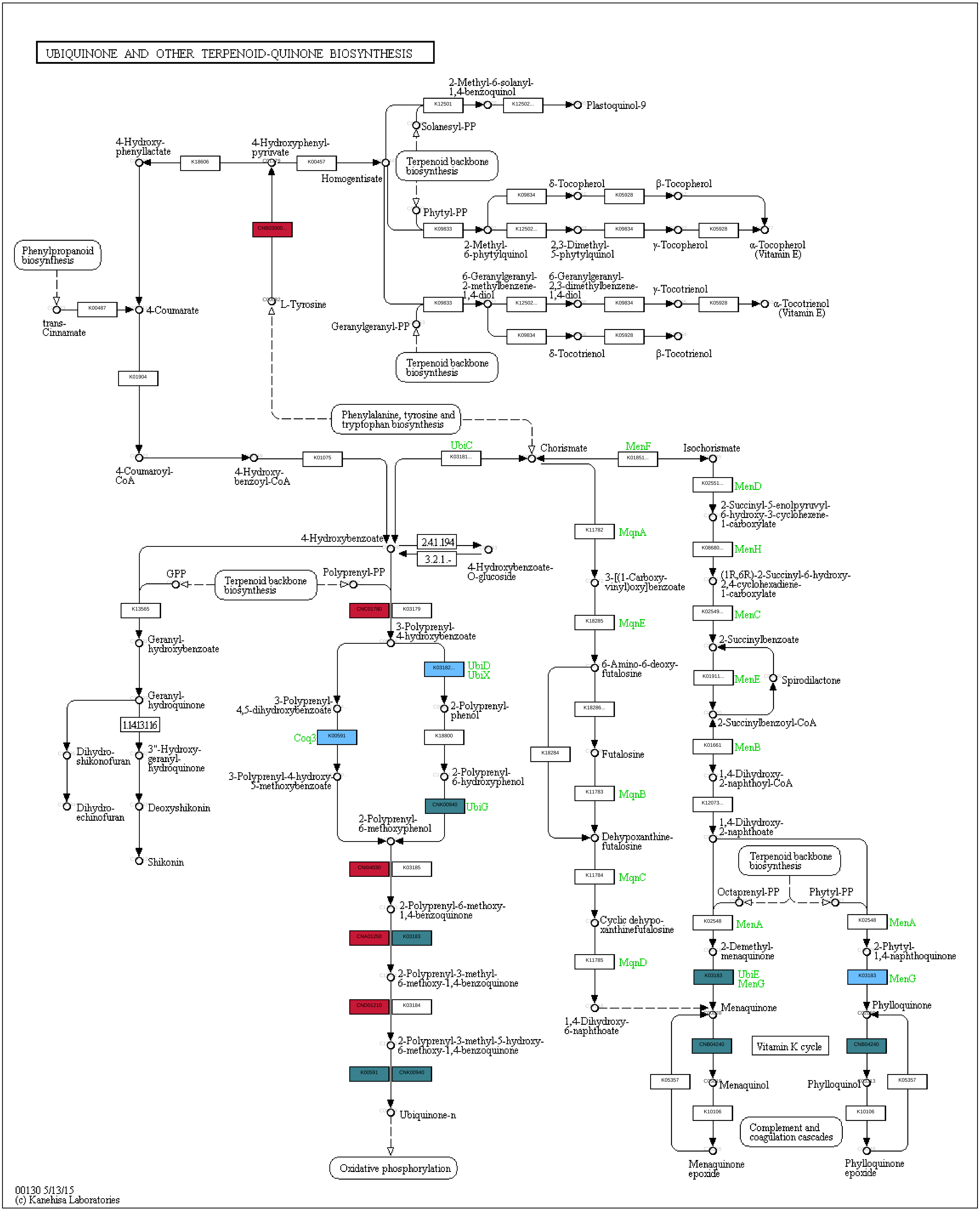
UBIQUINONE AND OTHER TERPENOID-QUINONE BIOSYNTHESIS
2-Methyl-6-solanyl-1,4-benzoquinol
K12501
K12502...
Plastoquinol-9
Solanesyl-PP
4-Hydroxy-phenyllactate
K18606
4-Hydroxyphenyl-pyruvate
K00457
Homogentisate
Terpenoid backbone biosynthesis
δ-Tocopherol
β-Tocopherol
K09834
K05928
Phytyl-PP
K09833
K12502...
K09834
K05928
2-Methyl-6-phytylquinol
2,3-Dimethyl-5-phytylquinol
γ-Tocopherol
α-Tocopherol (Vitamin E)
CNB03000...
Phenylpropanoid biosynthesis
6-Geranylgeranyl-2-methylbenzene-1,4-diol
6-Geranylgeranyl-2,3-dimethylbenzene-1,4-diol
γ-Tocotrienol
K00487
4-Coumarate
L-Tyrosine
K09833
K12502...
K09834
K05928
α-Tocotrienol (Vitamin E)
trans-Cinnamate
Geranylgeranyl-PP
K09834
K05928
δ-Tocotrienol
β-Tocotrienol
Terpenoid backbone biosynthesis
K01904
Phenylalanine, tyrosine and tryptophan biosynthesis
UbiC
Chorismate
MenF
Isochorismate
K01075
K03181...
K01851...
4-Coumaroyl-CoA
4-Hydroxy-benzoyl-CoA
K02551...
MenD
2-Succinyl-5-enolpyruvyl-6-hydroxy-3-cyclohexene-1-carboxylate
4-Hydroxybenzoate
2.4.1.194
3.2.1.-
K11782
MqnA
K08680...
MenH
4-Hydroxybenzoate-O-glucoside
GPP
Terpenoid backbone biosynthesis
Polyprenyl-PP
3-[(1-Carboxy-vinyl)oxy]benzoate
(1R,6R)-2-Succinyl-6-hydroxy-2,4-cyclohexadiene-1-carboxylate
K13565
CNC01780
K03179
K18285
MqnE
K02549...
MenC
Geranyl-hydroxybenzoate
3-Polyprenyl-4-hydroxybenzoate
2-Succinylbenzoate
K03182...
UbiD
UbiX
6-Amino-6-deoxy-futalosine
K01911...
MenE
Spirodilactone
Geranyl-hydroquinone
1.14.13.116
3-Polyprenyl-4,5-dihydroxybenzoate
2-Polyprenyl-phenol
K18286...
2-Succinylbenzoyl-CoA
Coq3
K00591
K18800
K18284
K01661
MenB
Dihydro-shikonofuran
3''-Hydroxy-geranyl-hydroquinone
Futalosine
1,4-Dihydroxy-2-naphthoyl-CoA
3-Polyprenyl-4-hydroxy-5-methoxybenzoate
2-Polyprenyl-6-hydroxyphenol
K11783
MqnB
K12073...
Dihydro-echinofuran
Deoxyshikonin
CNK00940
UbiG
1,4-Dihydroxy-2-naphthoate
2-Polyprenyl-6-methoxyphenol
Dehypoxanthine-futalosine
Terpenoid backbone biosynthesis
Shikonin
CNI04030
K03185
K11784
MqnC
Octaprenyl-PP
Phytyl-PP
2-Polyprenyl-6-methoxy-1,4-benzoquinone
Cyclic dehypo-xanthinefutalosine
K02548
MenA
K02548
MenA
CNA01250
K03183
2-Demethyl-menaquinone
2-Phytyl-1,4-naphthoquinone
2-Polyprenyl-3-methyl-6-methoxy-1,4-benzoquinone
K11785
MqnD
K03183
UbiE
MenG
K03183
MenG
CND01210
K03184
1,4-Dihydroxy-6-naphthoate
Menaquinone
Phylloquinone
2-Polyprenyl-3-methyl-5-hydroxy-6-methoxy-1,4-benzoquinone
CNB04240
Vitamin K cycle
CNB04240
K00591
CNK00940
K05357
Menaquinol
Phylloquinol
K05357
Ubiquinone-n
K10106
Complement and coagulation cascades
K10106
Menaquinone epoxide
Phylloquinone epoxide
Oxidative phosphorylation
00130 5/13/15
(c) Kanehisa Laboratories

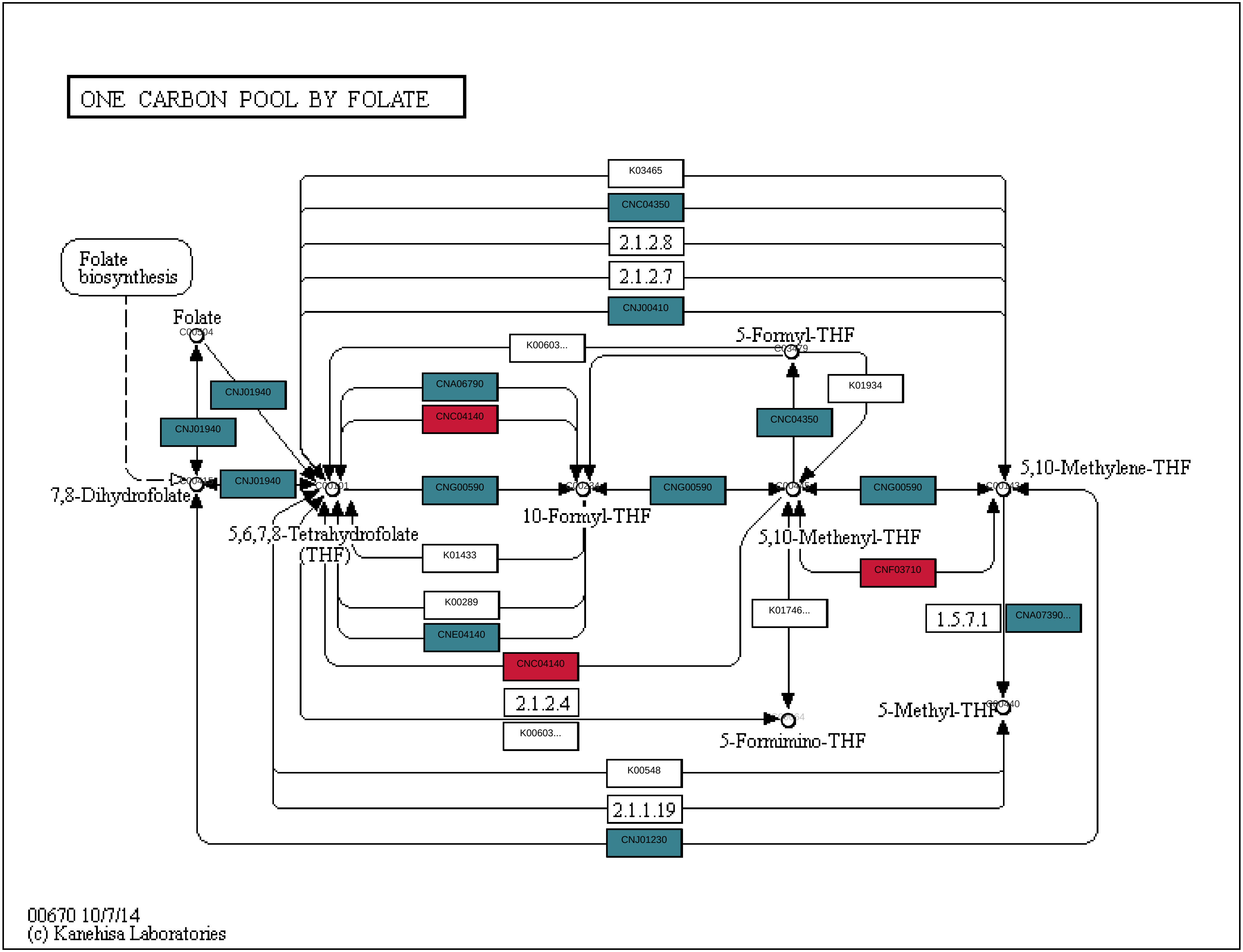
ONE CARBON POOL BY FOLATE
Folate biosynthesis
Folate
C00504
7,8-Dihydrofolate
C00415
5,6,7,8-Tetrahydrofolate (THF)
C00101
10-Formyl-THF
C00234
5-Formyl-THF
C03479
5,10-Methenyl-THF
C00445
5,10-Methylene-THF
C00143
5-Methyl-THF
C00440
5-Formimino-THF
K03465
CNC04350
2.1.2.8
2.1.2.7
CNJ00410
K00603...
CNA06790
CNC04140
K01934
CNC04350
CNJ01940
CNJ01940
CNJ01940
CNG00590
CNG00590
CNG00590
K01433
CNF03710
K00289
CNE04140
K01746...
1.5.7.1
CNA07390...
CNC04140
2.1.2.4
K00603...
K00548
2.1.1.19
CNJ01230
00670 10/7/14
(c) Kanehisa Laboratories

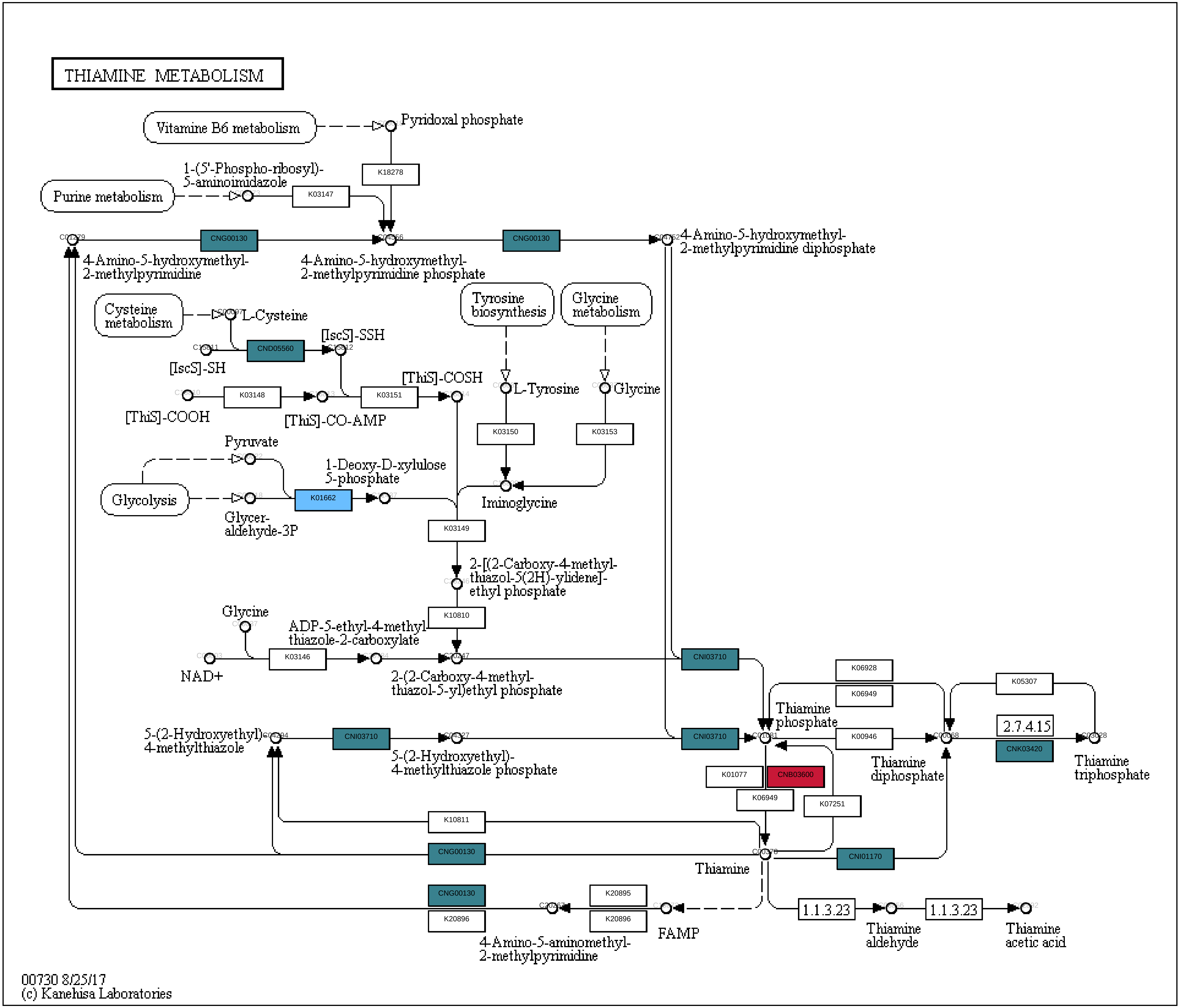
THIAMINE METABOLISM
Vitamine B6 metabolism
Pyridoxal phosphate
K18278
Purine metabolism
1-(5'-Phospho-ribosyl)-
5-aminoimidazole
K03147
CNG00130
CNG00130
4-Amino-5-hydroxymethyl-
2-methylpyrimidine diphosphate
4-Amino-5-hydroxymethyl-
2-methylpyrimidine
4-Amino-5-hydroxymethyl-
2-methylpyrimidine phosphate
Cysteine metabolism
L-Cysteine
CND05560
[IscS]-SSH
[IscS]-SH
Tyrosine biosynthesis
Glycine metabolism
K03148
K03151
[ThiS]-COSH
[ThiS]-COOH
[ThiS]-CO-AMP
L-Tyrosine
Glycine
K03150
K03153
Pyruvate
1-Deoxy-D-xylulose
5-phosphate
Glycolysis
K01662
Iminoglycine
Glycer-
aldehyde-3P
K03149
2-[(2-Carboxy-4-methyl-
thiazol-5(2H)-ylidene]-
ethyl phosphate
K10810
Glycine
ADP-5-ethyl-4-methyl-
thiazole-2-carboxylate
K03146
NAD+
2-(2-Carboxy-4-methyl-
thiazol-5-yl)ethyl phosphate
CNI03710
K06928
K06949
K05307
Thiamine
phosphate
5-(2-Hydroxyethyl)-
4-methylthiazole
CNI03710
5-(2-Hydroxyethyl)-
4-methylthiazole phosphate
CNI03710
K00946
2.7.4.15
CNK03420
Thiamine
diphosphate
Thiamine
triphosphate
K01077
CNB03600
K06949
K07251
K10811
CNG00130
CNI01170
Thiamine
CNG00130
K20895
K20896
K20896
1.1.3.23
1.1.3.23
FAMP
Thiamine
aldehyde
Thiamine
acetic acid
4-Amino-5-aminomethyl-
2-methylpyrimidine
00730 8/25/17
(c) Kanehisa Laboratories

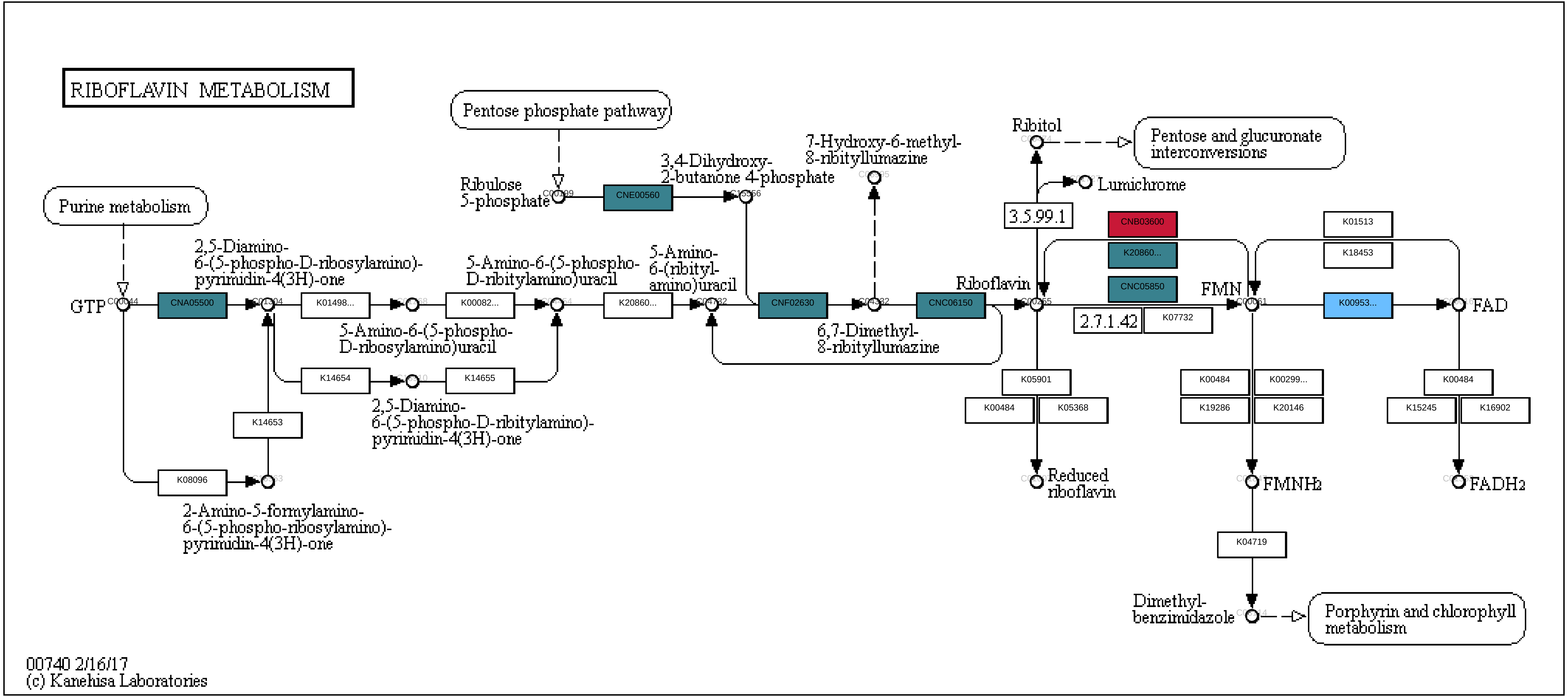
RIBOFLAVIN METABOLISM
Pentose phosphate pathway
Purine metabolism
Ribulose 5-phosphate
C00199
CNE00560
3,4-Dihydroxy-2-butanone 4-phosphate
7-Hydroxy-6-methyl-8-ribityllumazine
Ribitol
Pentose and glucuronate interconversions
Lumichrome
3.5.99.1
CNB03600
K20860...
CNC05850
K01513
K18453
2,5-Diamino-6-(5-phospho-D-ribosylamino)-pyrimidin-4(3H)-one
5-Amino-6-(5-phospho-D-ribitylamino)uracil
5-Amino-6-(ribitylamino)uracil
GTP
C00044
CNA05500
C01304
K01498...
K00082...
K20860...
C04732
CNF02630
C04332
CNC06150
Riboflavin
C00255
FMN
C00061
K00953...
FAD
2.7.1.42
K07732
5-Amino-6-(5-phospho-D-ribosylamino)uracil
6,7-Dimethyl-8-ribityllumazine
K14654
K14655
2,5-Diamino-6-(5-phospho-D-ribitylamino)-pyrimidin-4(3H)-one
K14653
K08096
2-Amino-5-formylamino-6-(5-phospho-ribosylamino)-pyrimidin-4(3H)-one
K05901
K00484
K05368
K00484
K00299...
K19286
K20146
K00484
K15245
K16902
Reduced riboflavin
FMNH2
FADH2
K04719
Dimethyl-benzimidazole
Porphyrin and chlorophyll metabolism
00740 2/16/17
(c) Kanehisa Laboratories

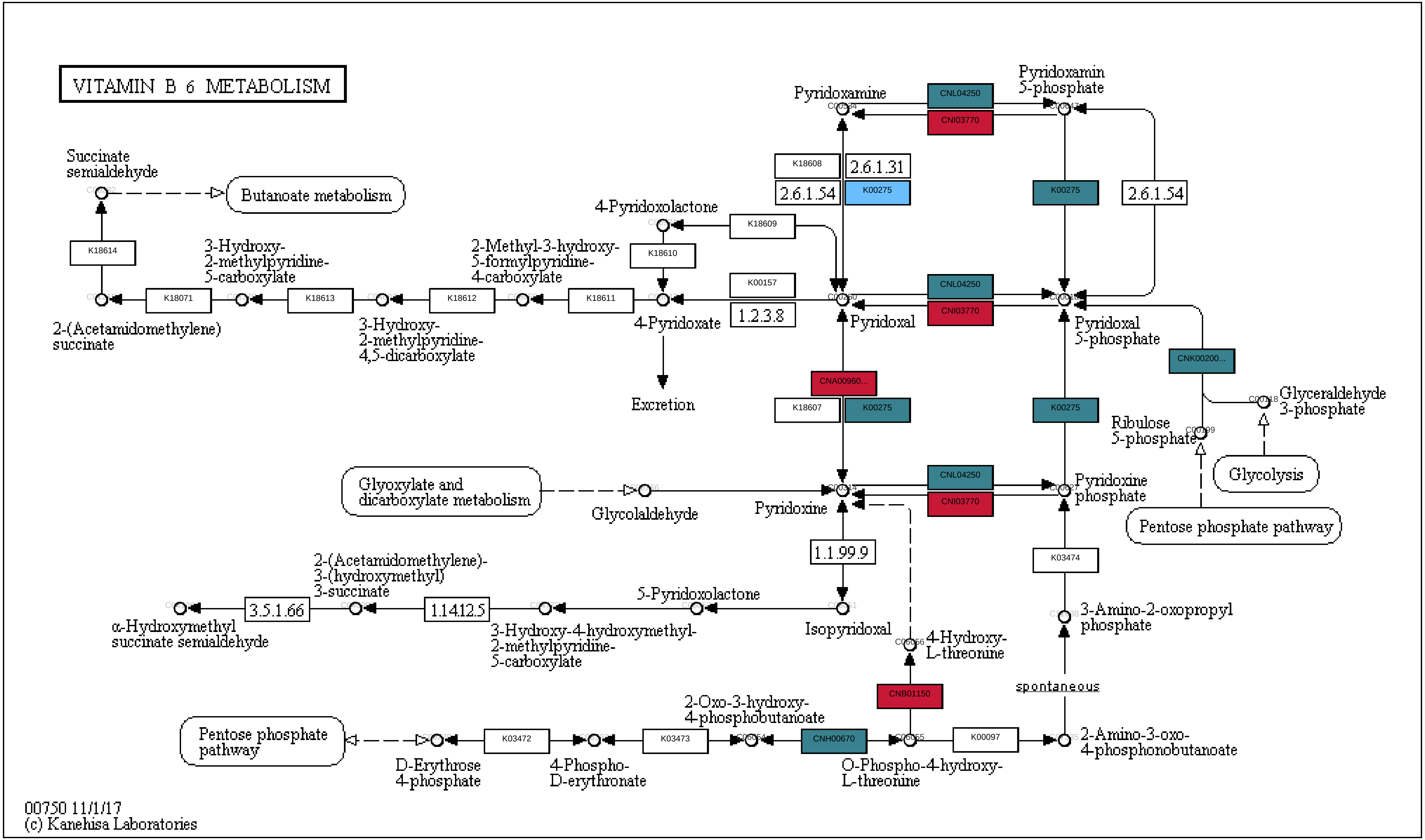
VITAMIN B 6 METABOLISM
Succinate semialdehyde
Butanoate metabolism
K18614
3-Hydroxy-2-methylpyridine-5-carboxylate
2-Methyl-3-hydroxy-5-formylpyridine-4-carboxylate
4-Pyridoxolactone
K18609
K18610
K18071
K18613
K18612
K18611
K00157
1.2.3.8
2-(Acetamidomethylene) succinate
3-Hydroxy-2-methylpyridine-4,5-dicarboxylate
4-Pyridoxate
Excretion
Pyridoxamine
Pyridoxamin 5-phosphate
CNL04250
CNI03770
K18608
2.6.1.31
2.6.1.54
K00275
K00275
2.6.1.54
CNL04250
CNI03770
Pyridoxal
Pyridoxal 5-phosphate
CNK00200...
CNA00960...
K18607
K00275
K00275
Glyceraldehyde 3-phosphate
Ribulose 5-phosphate
Glycolysis
Glyoxylate and dicarboxylate metabolism
Glycolaldehyde
Pyridoxine
CNL04250
CNI03770
Pyridoxine phosphate
Pentose phosphate pathway
1.1.99.9
K03474
2-(Acetamidomethylene)-3-(hydroxymethyl)3-succinate
5-Pyridoxolactone
3.5.1.66
1.14.12.5
Isopyridoxal
3-Amino-2-oxopropyl phosphate
α-Hydroxymethyl succinate semialdehyde
3-Hydroxy-4-hydroxymethyl-2-methylpyridine-5-carboxylate
4-Hydroxy-L-threonine
CNB01150
spontaneous
2-Oxo-3-hydroxy-4-phosphobutanoate
Pentose phosphate pathway
K03472
K03473
CNH00670
K00097
2-Amino-3-oxo-4-phosphonobutanoate
D-Erythrose 4-phosphate
4-Phospho-D-erythronate
O-Phospho-4-hydroxy-L-threonine
00750 11/1/17
(c) Kanehisa Laboratories

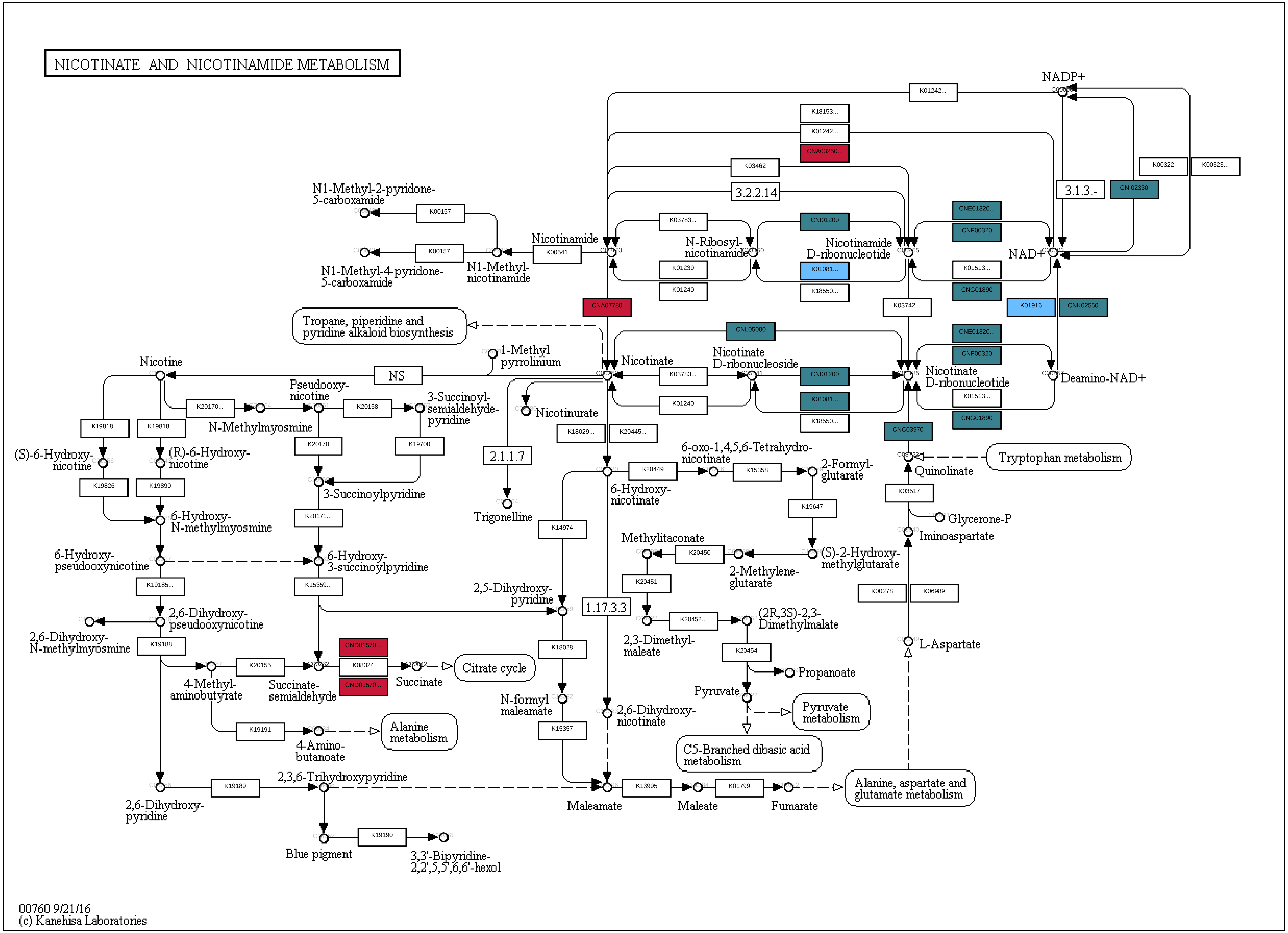
NICOTINATE AND NICOTINAMIDE METABOLISM
NADP+
K01242...
K18153...
K01242...
CNA03250...
K03462
3.2.2.14
3.1.3.-
CNI02330
K00322
K00323...
N1-Methyl-2-pyridone-5-carboxamide
K00157
K00157
N1-Methyl-4-pyridone-5-carboxamide
N1-Methyl-nicotinamide
K00541
Nicotinamide
K03783...
N-Ribosyl-nicotinamide
CNI01200
Nicotinamide D-ribonucleotide
CNE01320...
CNF00320
NAD+
K01239
K01240
K01081...
K18550...
K01513...
CNG01890
CNA07780
K03742...
K01916
CNK02550
Tropane, piperidine and pyridine alkaloid biosynthesis
CNL05000
1-Methyl pyrrolinium
Nicotine
NS
Nicotinate
K03783...
Nicotinate D-ribonucleoside
CNI01200
CNE01320...
CNF00320
Nicotinate D-ribonucleotide
Deamino-NAD+
K20170...
Pseudooxy-nicotine
K20158
3-Succinoyl-semialdehyde-pyridine
Nicotinurate
K01240
K01081...
K01513...
K18550...
CNG01890
CNC03970
K19818...
K19818...
N-Methylmyosmine
K20170
K19700
K18029...
K20445...
6-oxo-1,4,5,6-Tetrahydro-nicotinate
2-Formyl-glutarate
Tryptophan metabolism
(S)-6-Hydroxy-nicotine
(R)-6-Hydroxy-nicotine
2.1.1.7
K20449
K15358
Quinolinate
K19826
K19890
3-Succinoylpyridine
6-Hydroxy-nicotinate
K03517
Trigonelline
K19647
Glycerone-P
6-Hydroxy-N-methylmyosmine
K20171...
K14974
Methylitaconate
Iminoaspartate
6-Hydroxy-pseudooxynicotine
6-Hydroxy-3-succinoylpyridine
K20450
(S)-2-Hydroxy-methylglutarate
2-Methylene-glutarate
K20451
K00278
K06989
K19185...
K15359...
2,5-Dihydroxy-pyridine
1.17.3.3
2,6-Dihydroxy-pseudooxynicotine
K20452...
(2R,3S)-2,3-Dimethylmalate
2,6-Dihydroxy-N-methylmyosmine
K19188
CND01570...
K18028
2,3-Dimethyl-maleate
L-Aspartate
K20155
K08324
Citrate cycle
K20454
4-Methyl-aminobutyrate
Succinate-semialdehyde
CND01570...
Succinate
Propanoate
N-formyl maleamate
Pyruvate
2,6-Dihydroxy-nicotinate
Pyruvate metabolism
K19191
Alanine metabolism
4-Amino-butanoate
K15357
C5-Branched dibasic acid metabolism
2,3,6-Trihydroxypyridine
K19189
Alanine, aspartate and glutamate metabolism
2,6-Dihydroxy-pyridine
K13995
K01799
Maleamate
Maleate
Fumarate
K19190
Blue pigment
3,3'-Bipyridine-2,2',5,5',6,6'-hexol
00760 9/21/16
(c) Kanehisa Laboratories

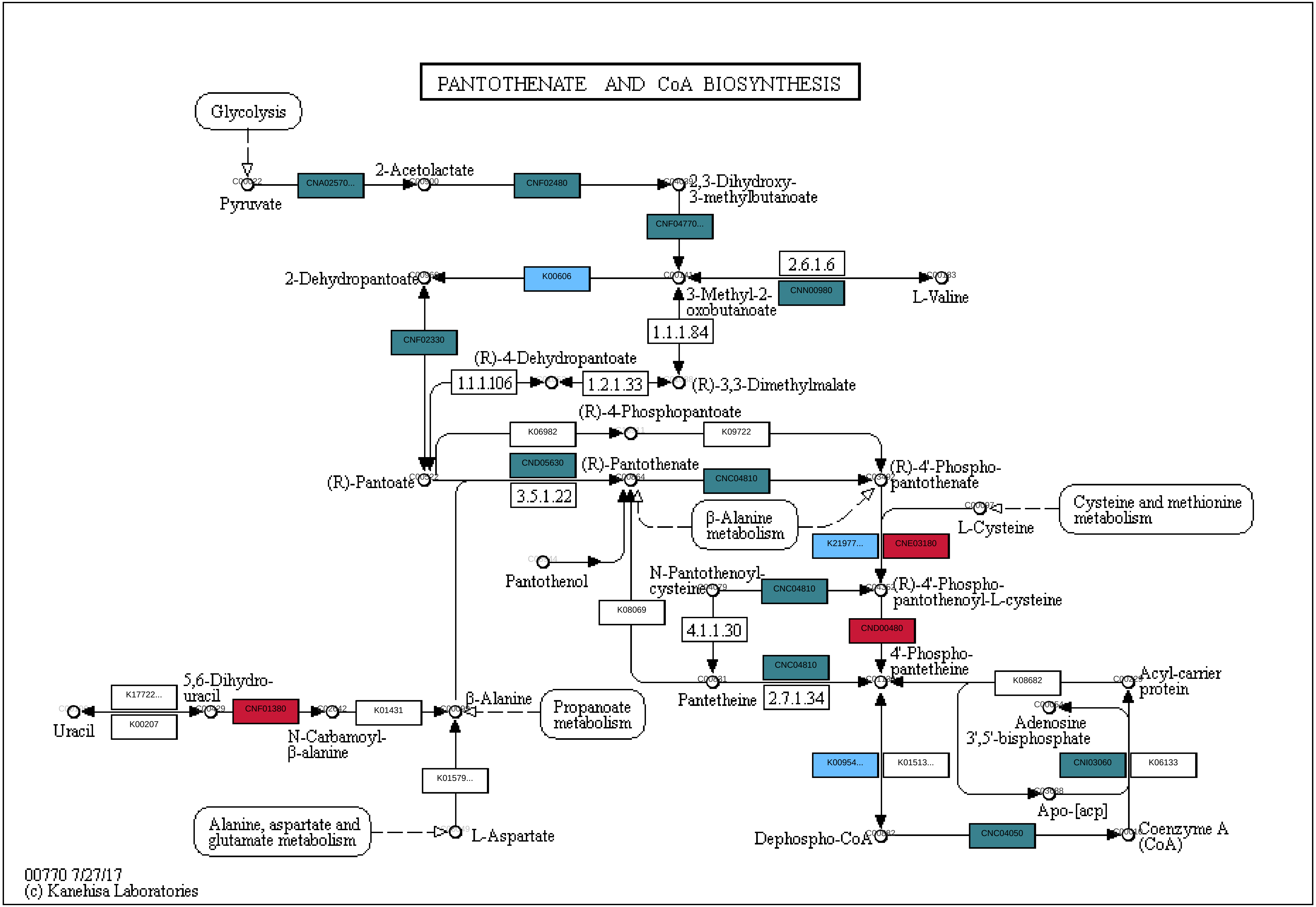
PANTOTHENATE AND CoA BIOSYNTHESIS
Glycolysis
Pyruvate
CNA02570...
2-Acetolactate
CNF02480
2,3-Dihydroxy-
3-methylbutanoate
CNF04770...
2-Dehydropantoate
K00606
2.6.1.6
CNN00980
L-Valine
3-Methyl-2-
oxobutanoate
1.1.1.84
CNF02330
(R)-4-Dehydropantoate
1.1.1.106
1.2.1.33
(R)-3,3-Dimethylmalate
(R)-4-Phosphopantoate
K06982
K09722
(R)-Pantoate
CND05630
(R)-Pantothenate
CNC04810
(R)-4'-Phospho-
pantothenate
3.5.1.22
β-Alanine
metabolism
Cysteine and methionine
metabolism
L-Cysteine
K21977...
CNE03180
Pantothenol
N-Pantothenoyl-
cysteine
CNC04810
(R)-4'-Phospho-
pantothenoyl-L-cysteine
K08069
4.1.1.30
CND00480
4'-Phospho-
pantetheine
CNC04810
Pantetheine
2.7.1.34
K08682
Acyl-carrier
protein
Adenosine
3',5'-bisphosphate
K17722...
K00207
5,6-Dihydro-
uracil
CNF01380
K01431
β-Alanine
Propanoate
metabolism
Uracil
N-Carbamoyl-
β-alanine
K01579...
K00954...
K01513...
CNI03060
K06133
Apo-[acp]
Alanine, aspartate and
glutamate metabolism
L-Aspartate
Dephospho-CoA
CNC04050
Coenzyme A
(CoA)
00770 7/27/17
(c) Kanehisa Laboratories

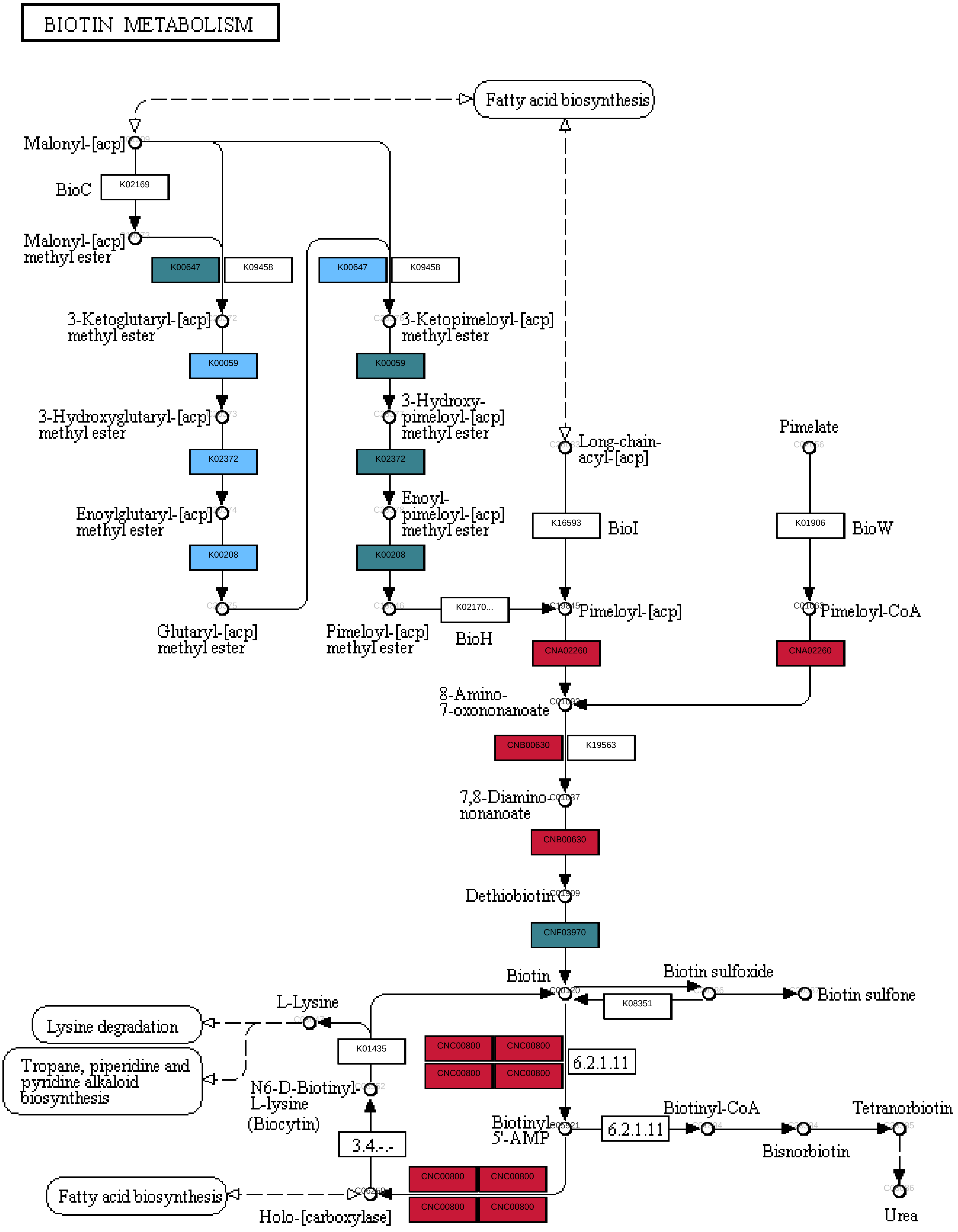


00780 7/19/17
(c) Kanehisa Laboratories

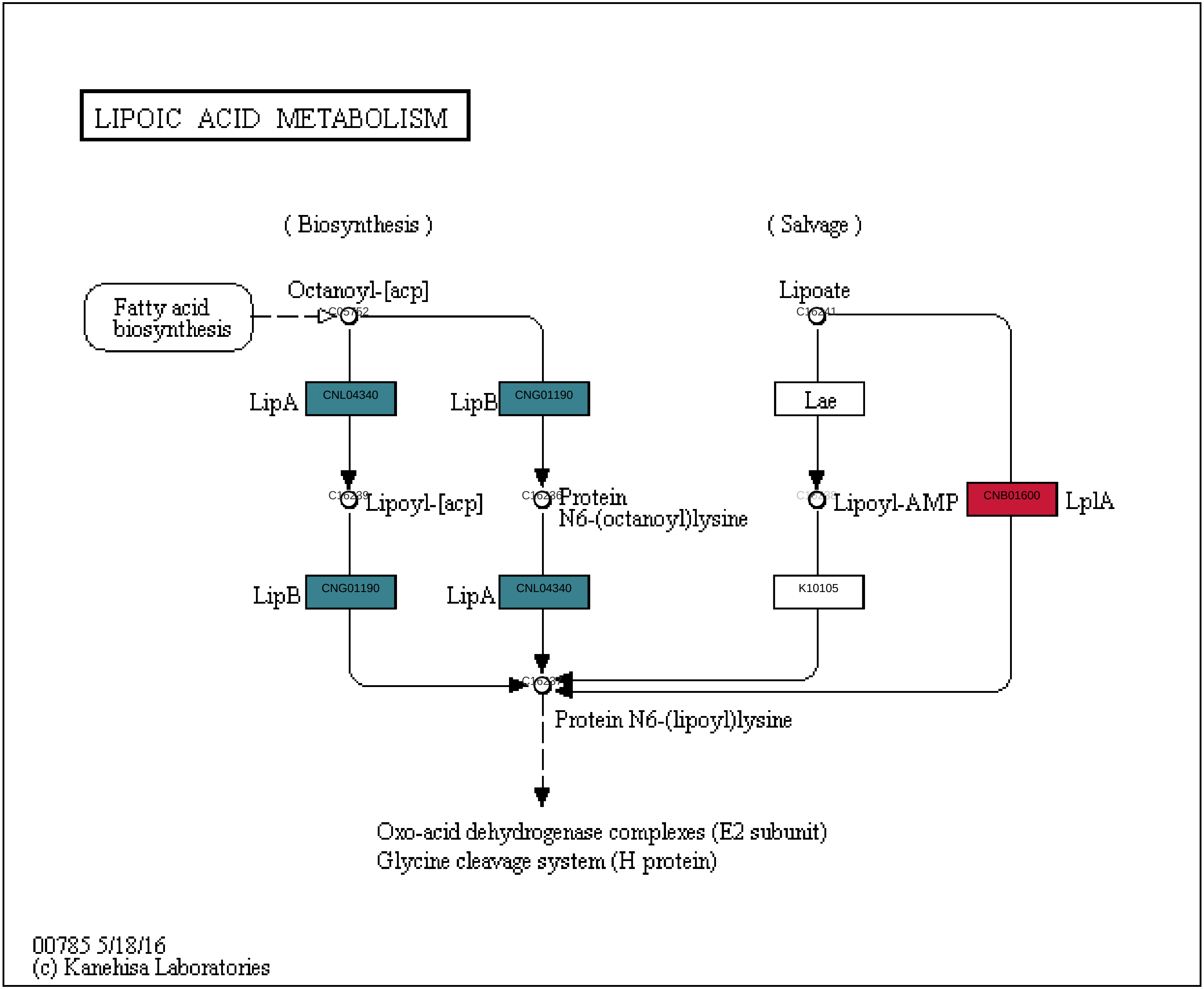
LIPOIC ACID METABOLISM
( Biosynthesis )
( Salvage )
Fatty acid biosynthesis
Octanoyl-[acp]
Lipoate
LipA
CNL04340
LipB
CNG01190
Lae
C16239
Lipoyl-[acp]
C16236
Protein N6-(octanoyl)lysine
Lipoyl-AMP
CNB01600
LplA
LipB
CNG01190
LipA
CNL04340
K10105
Protein N6-(lipoyl)lysine
Oxo-acid dehydrogenase complexes (E2 subunit)
Glycine cleavage system (H protein)
00785 5/18/16
(c) Kanehisa Laboratories

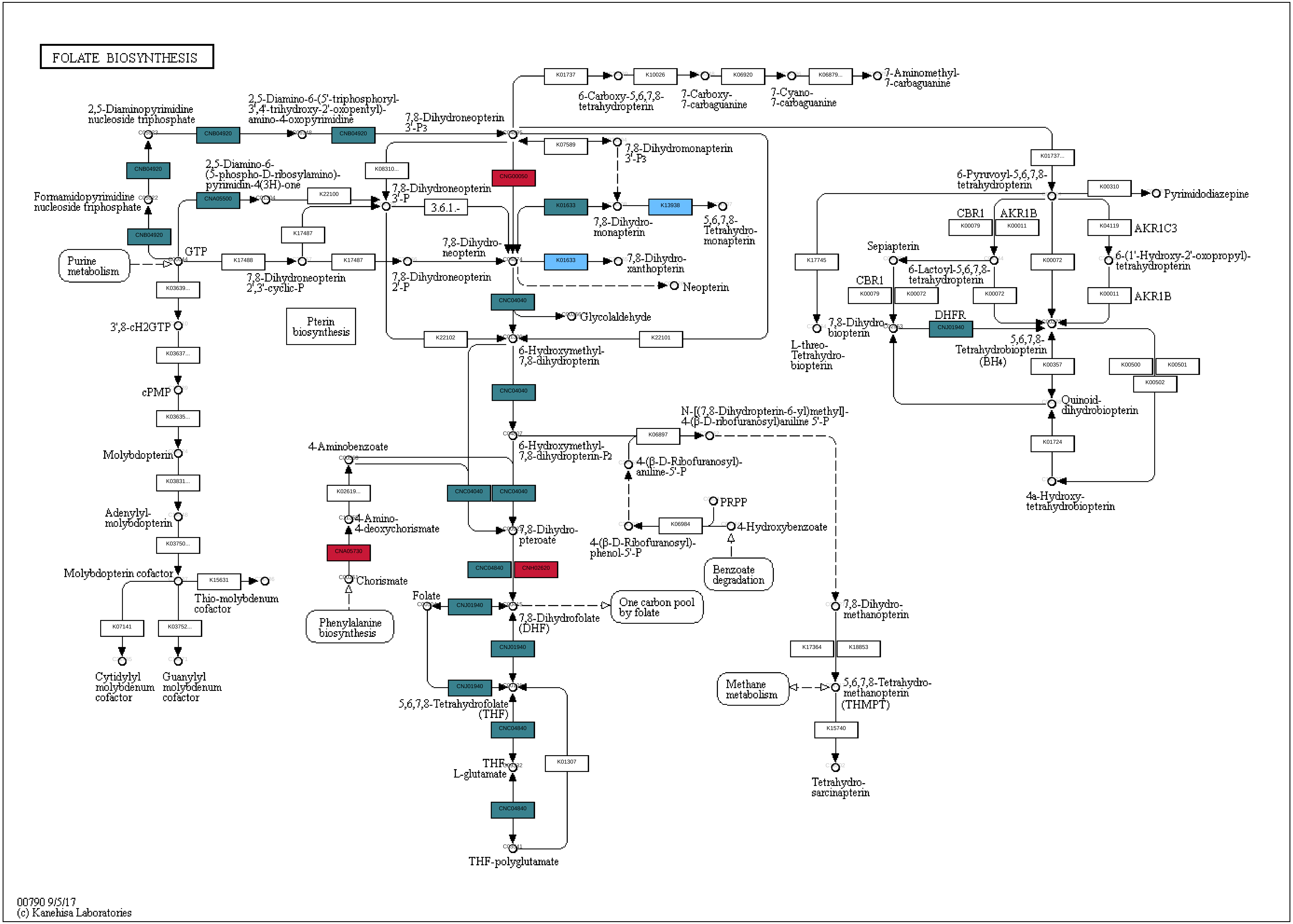

00790 9/5/17
(c) Kanehisa Laboratories

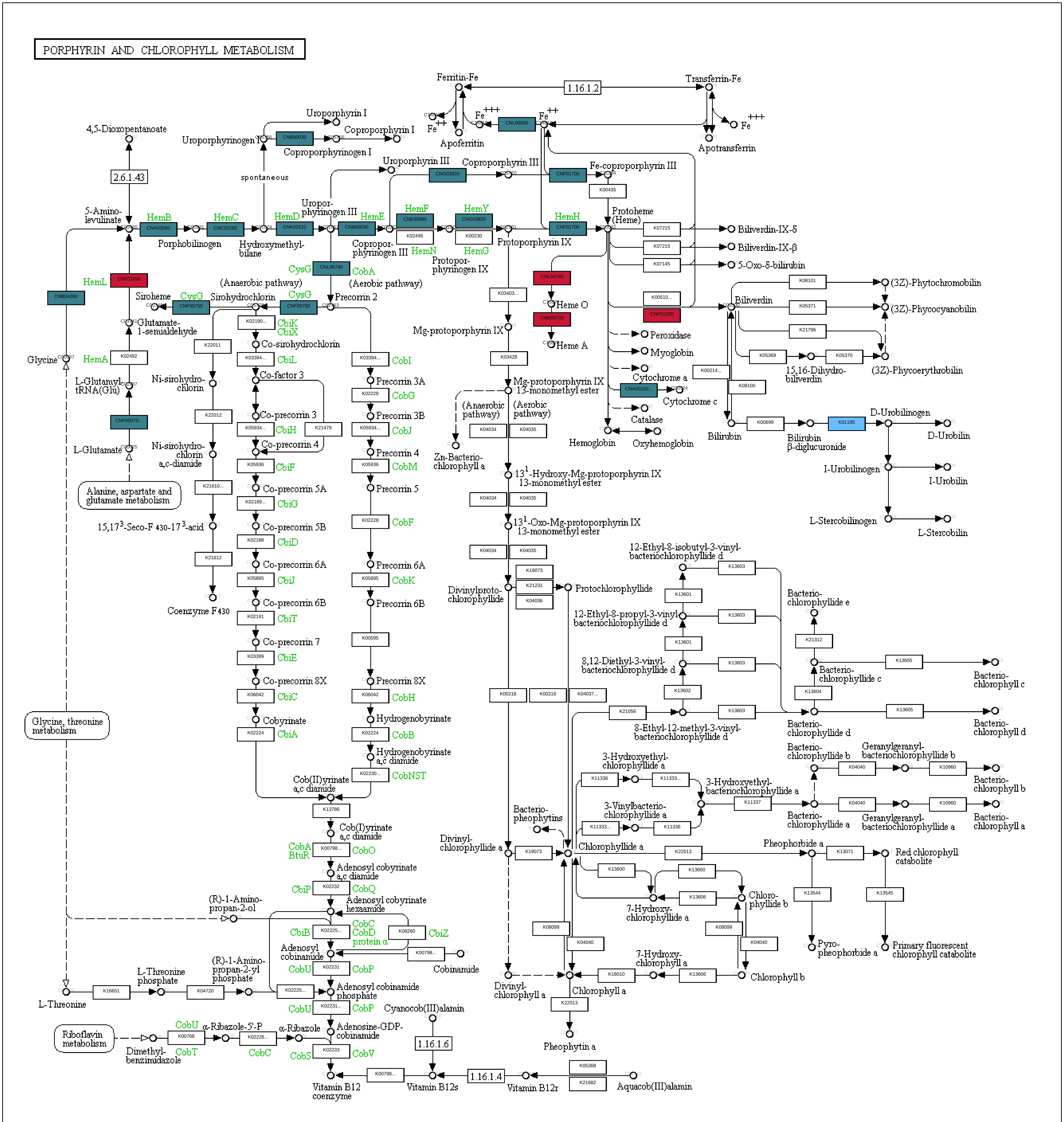


00860 12/22/17
(c) Kanehisa Laboratories

# 9. Metabolism of terpenoids and polyketides

| MAP | PATHWAY |
|---|---|
| **900** | Terpenoid backbone biosynthesis |
| **909** | Sesquiterpenoid and triterpenoid biosynthesis |

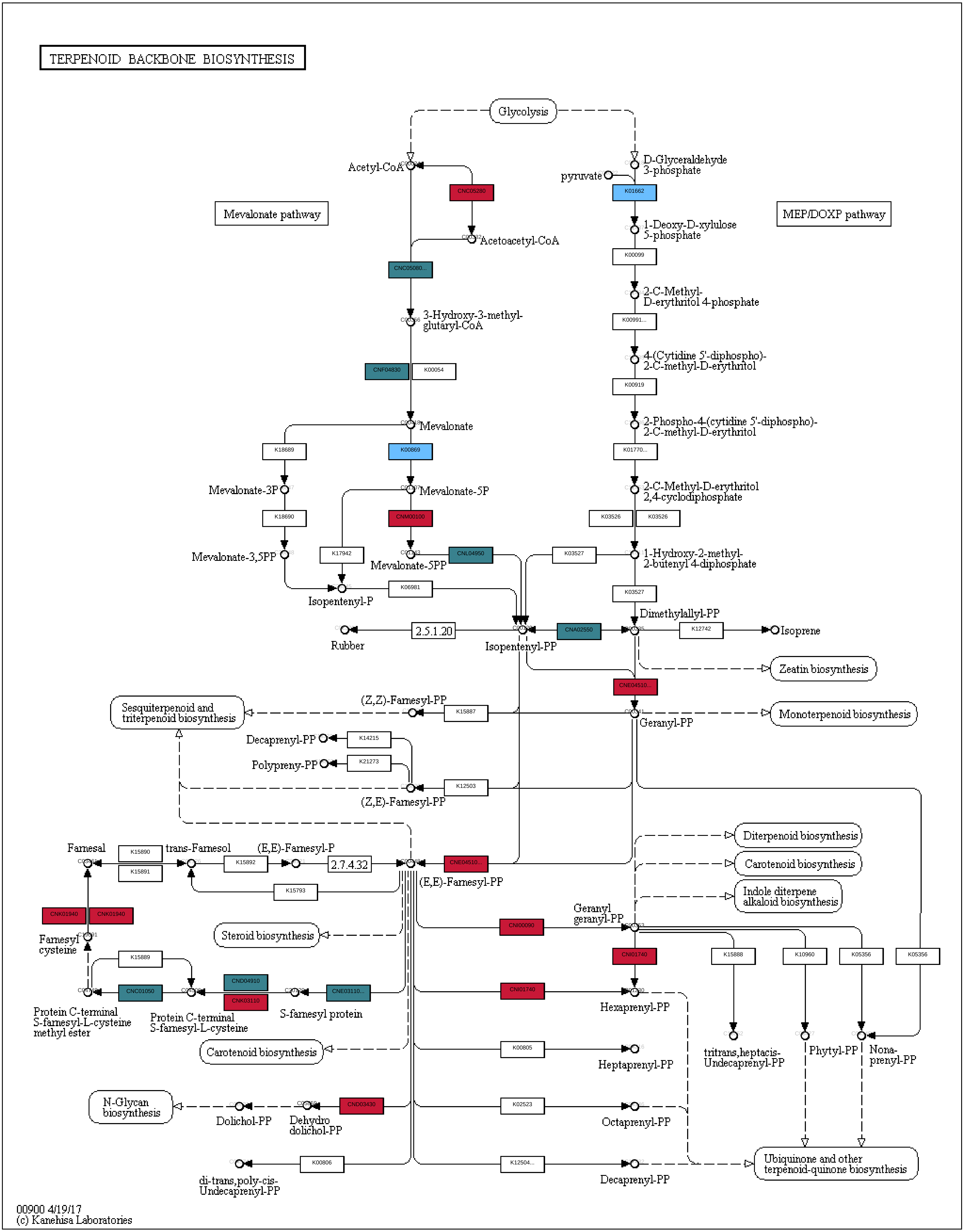
TERPENOID BACKBONE BIOSYNTHESIS
Glycolysis
Acetyl-CoA
CNC05280
D-Glyceraldehyde 3-phosphate
pyruvate
K01662
Mevalonate pathway
MEP/DOXP pathway
Acetoacetyl-CoA
1-Deoxy-D-xylulose 5-phosphate
CNC05080...
K00099
2-C-Methyl-D-erythritol 4-phosphate
3-Hydroxy-3-methyl-glutaryl-CoA
K00991...
CNF04830
K00054
4-(Cytidine 5'-diphospho)-2-C-methyl-D-erythritol
K00919
Mevalonate
2-Phospho-4-(cytidine 5'-diphospho)-2-C-methyl-D-erythritol
K18689
K00869
K01770...
Mevalonate-3P
Mevalonate-5P
2-C-Methyl-D-erythritol 2,4-cyclodiphosphate
K18690
CNM00100
K03526
K03526
Mevalonate-3,5PP
K17942
CNL04950
K03527
1-Hydroxy-2-methyl-2-butenyl 4-diphosphate
Mevalonate-5PP
Isopentenyl-P
K06981
K03527
Dimethylallyl-PP
Rubber
2.5.1.20
CNA02550
K12742
Isoprene
Isopentenyl-PP
Zeatin biosynthesis
CNE04510...
(Z,Z)-Farnesyl-PP
Sesquiterpenoid and triterpenoid biosynthesis
K15887
Monoterpenoid biosynthesis
Geranyl-PP
Decaprenyl-PP
K14215
Polypreny-PP
K21273
K12503
(Z,E)-Farnesyl-PP
Diterpenoid biosynthesis
Farnesal
K15890
trans-Farnesol
(E,E)-Farnesyl-P
K15892
2.7.4.32
CNE04510...
Carotenoid biosynthesis
K15891
(E,E)-Farnesyl-PP
K15793
Indole diterpene alkaloid biosynthesis
CNK01940
CNK01940
Geranyl geranyl-PP
Farnesyl cysteine
Steroid biosynthesis
CNI00090
CNI01740
K15888
K10960
K05356
K05356
K15889
CND04910
CNC01050
CNE03110...
CNK03110
CNI01740
Hexaprenyl-PP
Protein C-terminal S-farnesyl-L-cysteine methyl ester
Protein C-terminal S-farnesyl-L-cysteine
S-farnesyl protein
K00805
Carotenoid biosynthesis
Heptaprenyl-PP
tritrans,heptacis-Undecaprenyl-PP
Phytyl-PP
Nona-prenyl-PP
N-Glycan biosynthesis
CND03430
K02523
Dolichol-PP
Dehydro dolichol-PP
Octaprenyl-PP
Ubiquinone and other terpenoid-quinone biosynthesis
K00806
K12504...
di-trans,poly-cis-Undecaprenyl-PP
Decaprenyl-PP
00900 4/19/17
(c) Kanehisa Laboratories

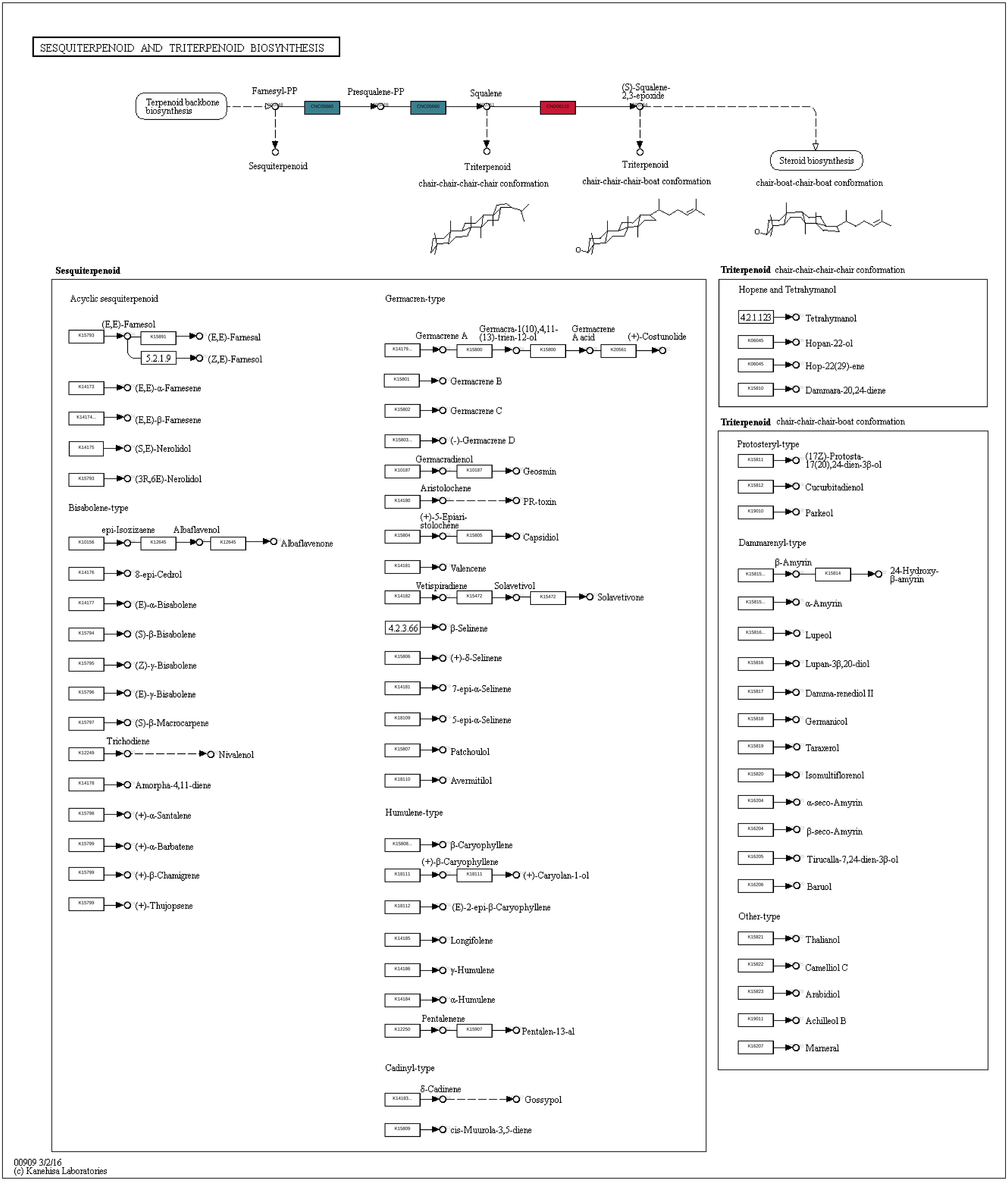

SESQUITERPENOID AND TRITERPENOID BIOSYNTHESIS
Terpenoid backbone biosynthesis
Farnesyl-PP
CNC05660
Presqualene-PP
CNC05660
Squalene
CND06110
(S)-Squalene-2,3-epoxide
Sesquiterpenoid
Triterpenoid
chair-chair-chair-chair conformation
Triterpenoid
chair-chair-chair-boat conformation
Steroid biosynthesis
chair-boat-chair-boat conformation
Sesquiterpenoid
Acyclic sesquiterpenoid
K15793
(E,E)-Farnesol
K15891
(E,E)-Farnesal
5.2.1.9
(Z,E)-Farnesol
K14173
(E,E)-α-Farnesene
K14174...
(E,E)-β-Farnesene
K14175
(S,E)-Nerolidol
K15793
(3R,6E)-Nerolidol
Bisabolene-type
K10156
epi-Isozizaene
K12645
Albaflavenol
K12645
Albaflavenone
K14176
8-epi-Cedrol
K14177
(E)-α-Bisabolene
K15794
(S)-β-Bisabolene
K15795
(Z)-γ-Bisabolene
K15796
(E)-γ-Bisabolene
K15797
(S)-β-Macrocarpene
K12249
Trichodiene
Nivalenol
K14178
Amorpha-4,11-diene
K15798
(+)-α-Santalene
K15799
(+)-α-Barbatene
K15799
(+)-β-Chamigrene
K15799
(+)-Thujopsene
Germacren-type
K14179...
Germacrene A
K15800
Germacra-1(10),4,11-(13)-trien-12-ol
K15800
Germacrene A acid
K20561
(+)-Costunolide
K15801
Germacrene B
K15802
Germacrene C
K15803...
(-)-Germacrene D
K10187
Germacradienol
K10187
Geosmin
K14180
Aristolochene
PR-toxin
K15804
(+)-5-Epiaristolochene
K15805
Capsidiol
K14181
Valencene
K14182
Vetispiradiene
K15472
Solavetivol
K15472
Solavetivone
4.2.3.66
β-Selinene
K15806
(+)-δ-Selinene
K14181
7-epi-α-Selinene
K18109
5-epi-α-Selinene
K15807
Patchoulol
K18110
Avermitilol
Humulene-type
K15808...
β-Caryophyllene
K18111
(+)-β-Caryophyllene
K18111
(+)-Caryolan-1-ol
K18112
(E)-2-epi-β-Caryophyllene
K14185
Longifolene
K14186
γ-Humulene
K14184
α-Humulene
K12250
Pentalenene
K15907
Pentalen-13-al
Cadinyl-type
K14183...
δ-Cadinene
Gossypol
K15809
cis-Muurola-3,5-diene
Triterpenoid chair-chair-chair-chair conformation
Hopene and Tetrahymanol
4.2.1.123
Tetrahymanol
K06045
Hopan-22-ol
K06045
Hop-22(29)-ene
K15810
Dammara-20,24-diene
Triterpenoid chair-chair-chair-boat conformation
Protosteryl-type
K15811
(17Z)-Protosta-17(20),24-dien-3β-ol
K15812
Cucurbitadienol
K19010
Parkeol
Dammarenyl-type
K15815...
β-Amyrin
K15814
24-Hydroxy-β-amyrin
K15815...
α-Amyrin
K15816...
Lupeol
K15816
Lupan-3β,20-diol
K15817
Damma-renediol II
K15818
Germanicol
K15819
Taraxerol
K15820
Isomultiflorenol
K16204
α-seco-Amyrin
K16204
β-seco-Amyrin
K16205
Tirucalla-7,24-dien-3β-ol
K16206
Baruol
Other-type
K15821
Thalianol
K15822
Camelliol C
K15823
Arabidiol
K19011
Achilleol B
K16207
Marneral
00909 3/2/16
(c) Kanehisa Laboratories

# 10. Biosynthesis of other secondary metabolites

| MAP | PATHWAY |
|---|---|
| **261** | Monobactam biosynthesis |
| **332** | Carbapenem biosynthesis |

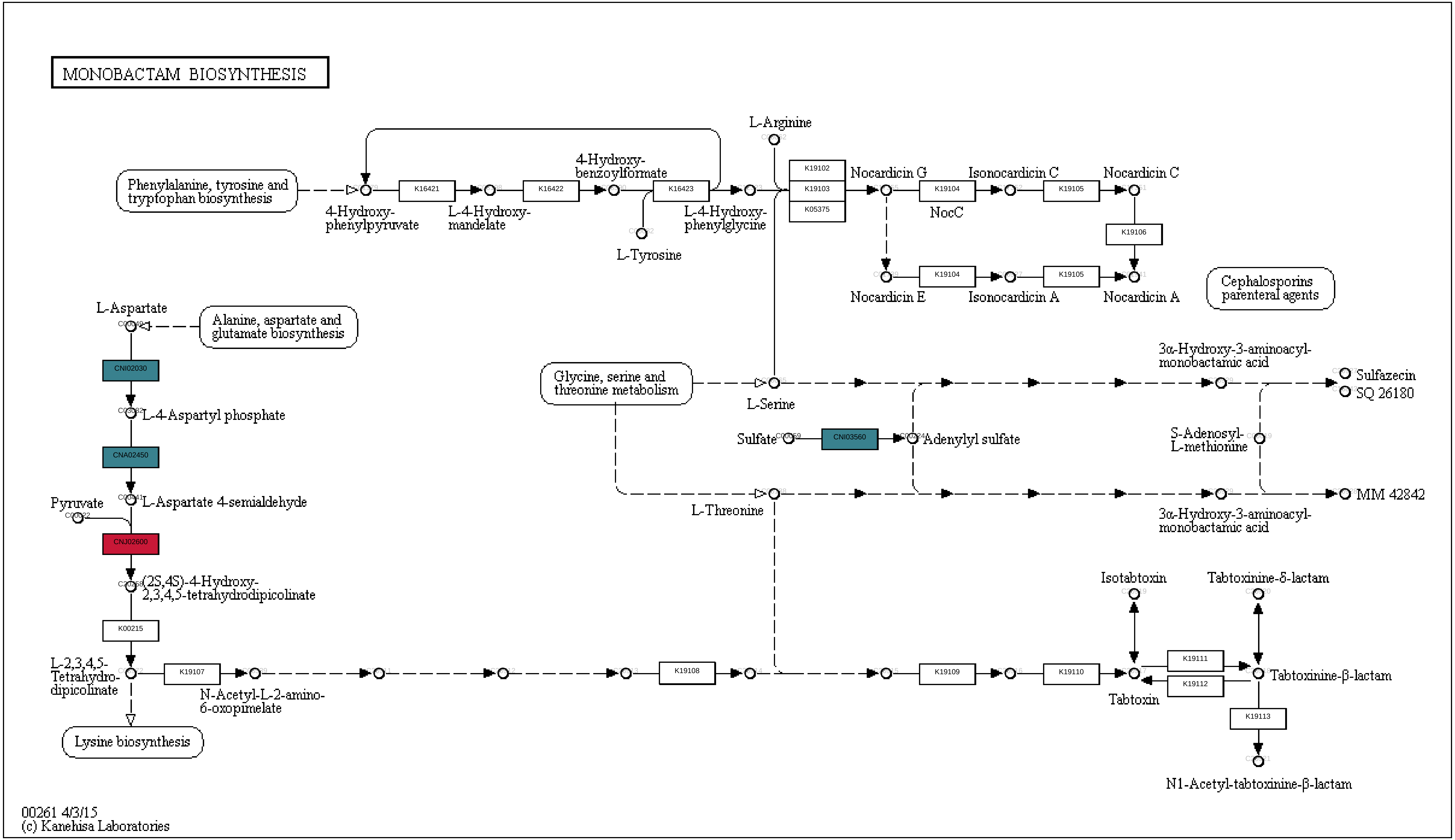
MONOBACTAM BIOSYNTHESIS
Phenylalanine, tyrosine and tryptophan biosynthesis
4-Hydroxy-phenylpyruvate
K16421
L-4-Hydroxy-mandelate
K16422
4-Hydroxy-benzoylformate
K16423
L-Tyrosine
L-4-Hydroxy-phenylglycine
L-Arginine
K19102
K19103
K05375
Nocardicin G
K19104
NocC
Isonocardicin C
K19105
Nocardicin C
K19106
Nocardicin E
K19104
Isonocardicin A
K19105
Nocardicin A
Cephalosporins parenteral agents
L-Aspartate
Alanine, aspartate and glutamate biosynthesis
CNI02030
L-4-Aspartyl phosphate
CNA02450
L-Aspartate 4-semialdehyde
Pyruvate
CNJ02600
(2S,4S)-4-Hydroxy-2,3,4,5-tetrahydrodipicolinate
K00215
L-2,3,4,5-Tetrahydro-dipicolinate
K19107
N-Acetyl-L-2-amino-6-oxopimelate
Lysine biosynthesis
Glycine, serine and threonine metabolism
L-Serine
Sulfate
CNI03560
Adenylyl sulfate
L-Threonine
3α-Hydroxy-3-aminoacyl-monobactamic acid
Sulfazecin
SQ 26180
S-Adenosyl-L-methionine
MM 42842
3α-Hydroxy-3-aminoacyl-monobactamic acid
K19108
K19109
K19110
Isotabtoxin
Tabtoxinine-δ-lactam
K19111
K19112
Tabtoxin
Tabtoxinine-β-lactam
K19113
N1-Acetyl-tabtoxinine-β-lactam
00261 4/3/15
(c) Kanehisa Laboratories

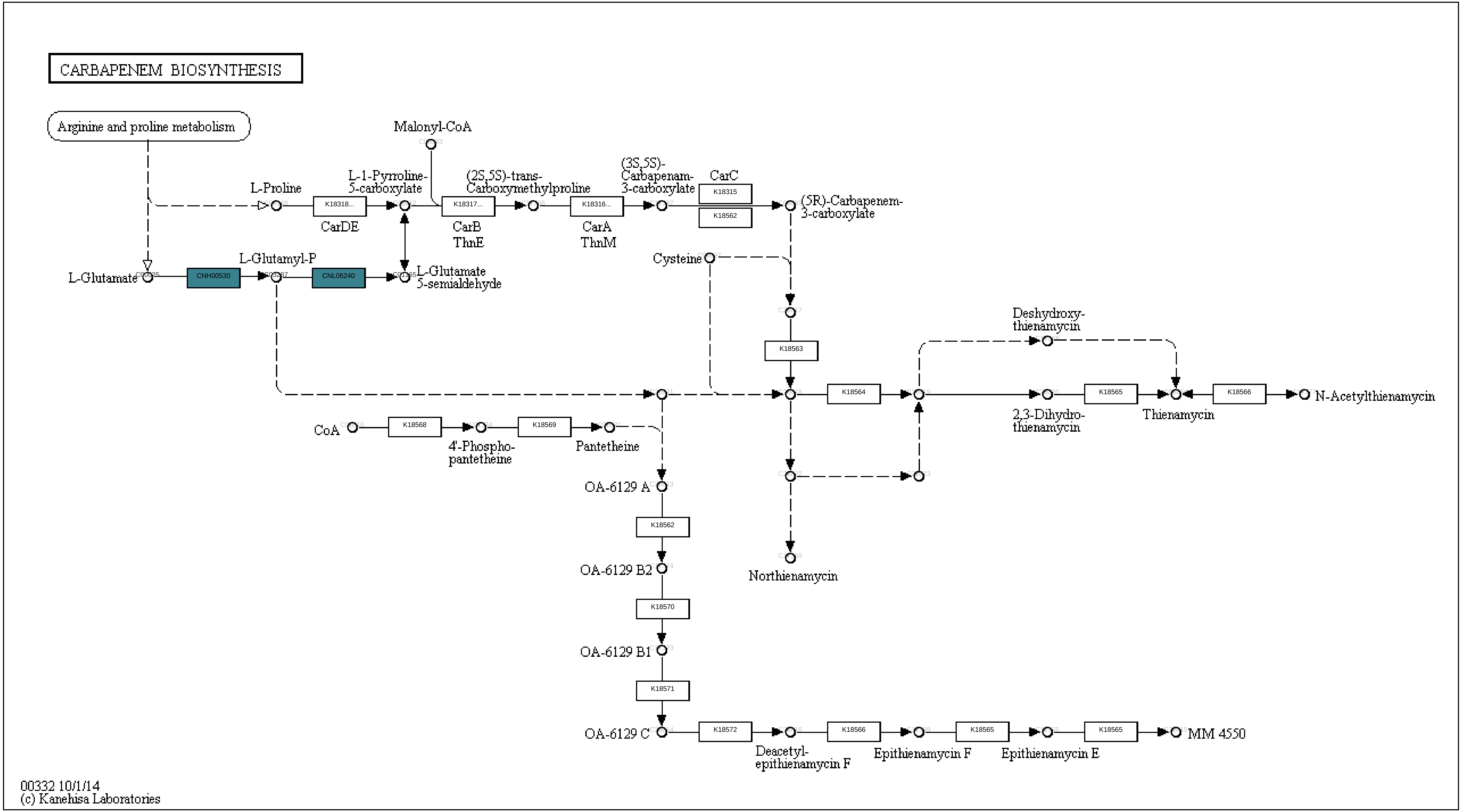


00332 10/1/14

(c) Kanehisa Laboratories

# 11. Xenobiotics biodegradation and metabolism

| MAP | PATHWAY |
|---|---|
| **791** | Atrazine degradation |

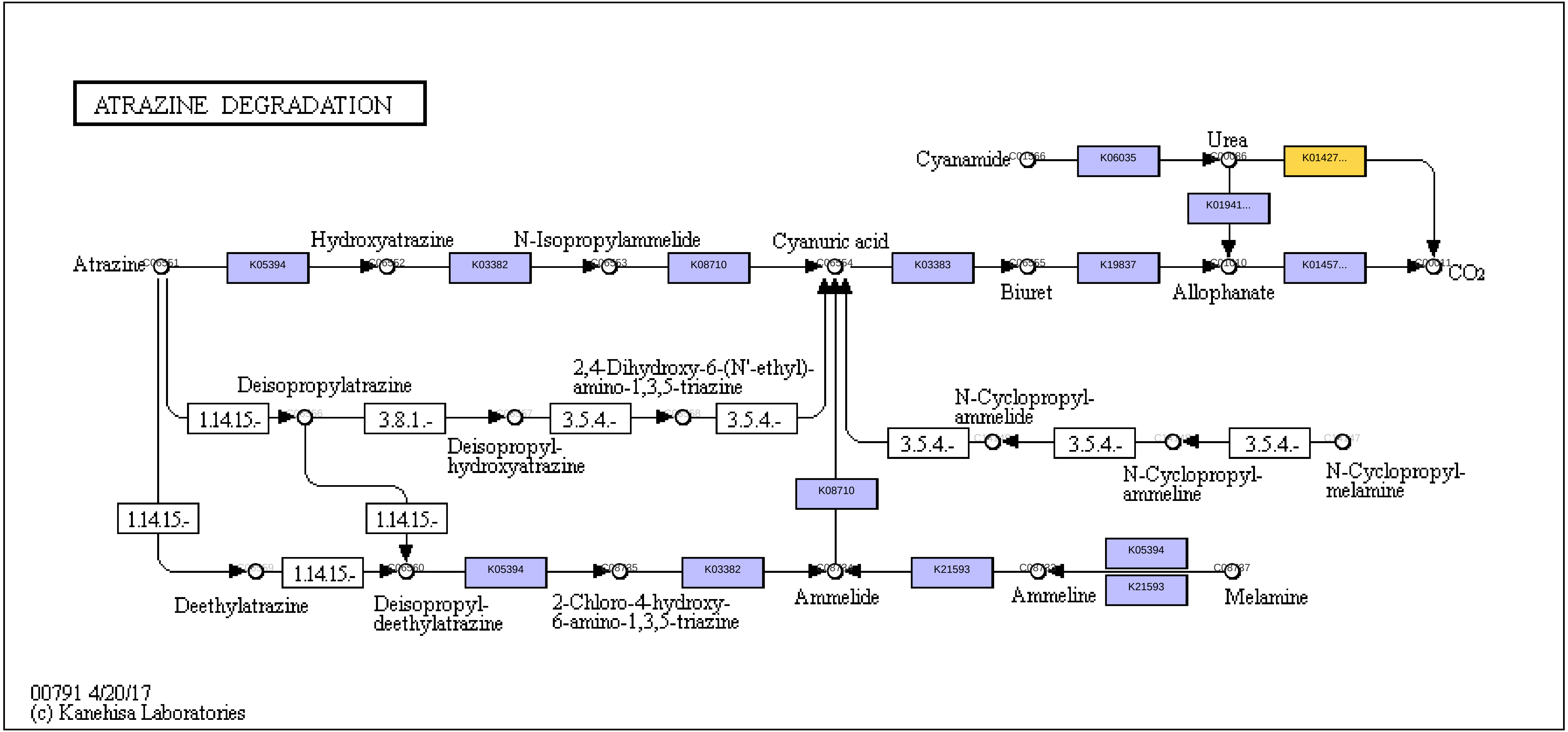
ATRAZINE DEGRADATION
Cyanamide
K06035
Urea
K01427...
K01941...
Atrazine
K05394
Hydroxyatrazine
K03382
N-Isopropylammelide
K08710
Cyanuric acid
K03383
Biuret
K19837
Allophanate
K01457...
CO2
Deisopropylatrazine
1.14.15.-
3.8.1.-
Deisopropyl-
hydroxyatrazine
2,4-Dihydroxy-6-(N'-ethyl)-
amino-1,3,5-triazine
3.5.4.-
3.5.4.-
N-Cyclopropyl-
ammelide
3.5.4.-
3.5.4.-
3.5.4.-
N-Cyclopropyl-
ammeline
N-Cyclopropyl-
melamine
K08710
1.14.15.-
1.14.15.-
1.14.15.-
Deethylatrazine
Deisopropyl-
deethylatrazine
K05394
2-Chloro-4-hydroxy-
6-amino-1,3,5-triazine
K03382
Ammelide
K21593
Ammeline
K05394
K21593
Melamine
00791 4/20/17
(c) Kanehisa Laboratories

**KEGG pathway analysis**

# *Positive controls*

**Supplementary file 4**

**The following color codes are used:**

True positive (TP): element in the reference pathway correctly predicted by Interproscan

True negative (TN): element in the *not* reference pathway and *not* predicted by Interproscan

False negative (FN): element in the reference pathway but *not* predicted by Interproscan.

False positive (FP): element in the *not* reference pathway but predicted by Interproscan. Note: could also be a valid new prediction

**Species:** ***Ustilago maydis***

**Acronym:** **UMA**

**Lifestyle:** **Facultative biotrophic (smut)**

# 1. Carbohydrate metabolism

| MAP | PATHWAY |
|---|---|
| **10** | Glycolysis / Gluconeogenesis |
| **20** | Citrate cycle (TCA cycle) |
| **30** | Pentose phosphate pathway |
| **40** | Pentose and glucuronate interconversions |
| **51** | Fructose and mannose metabolism |
| **52** | Galactose metabolism |
| **53** | Ascorbate and aldarate metabolism |
| **500** | Starch and sucrose metabolism |
| **520** | Amino sugar and nucleotide sugar metabolism |
| **562** | Inositol phosphate metabolism |
| **620** | Pyruvate metabolism |
| **630** | Glyoxylate and dicarboxylate metabolism |
| **640** | Propanoate metabolism |
| **650** | Butanoate metabolism |
| **660** | C5-Branched dibasic acid metabolism |

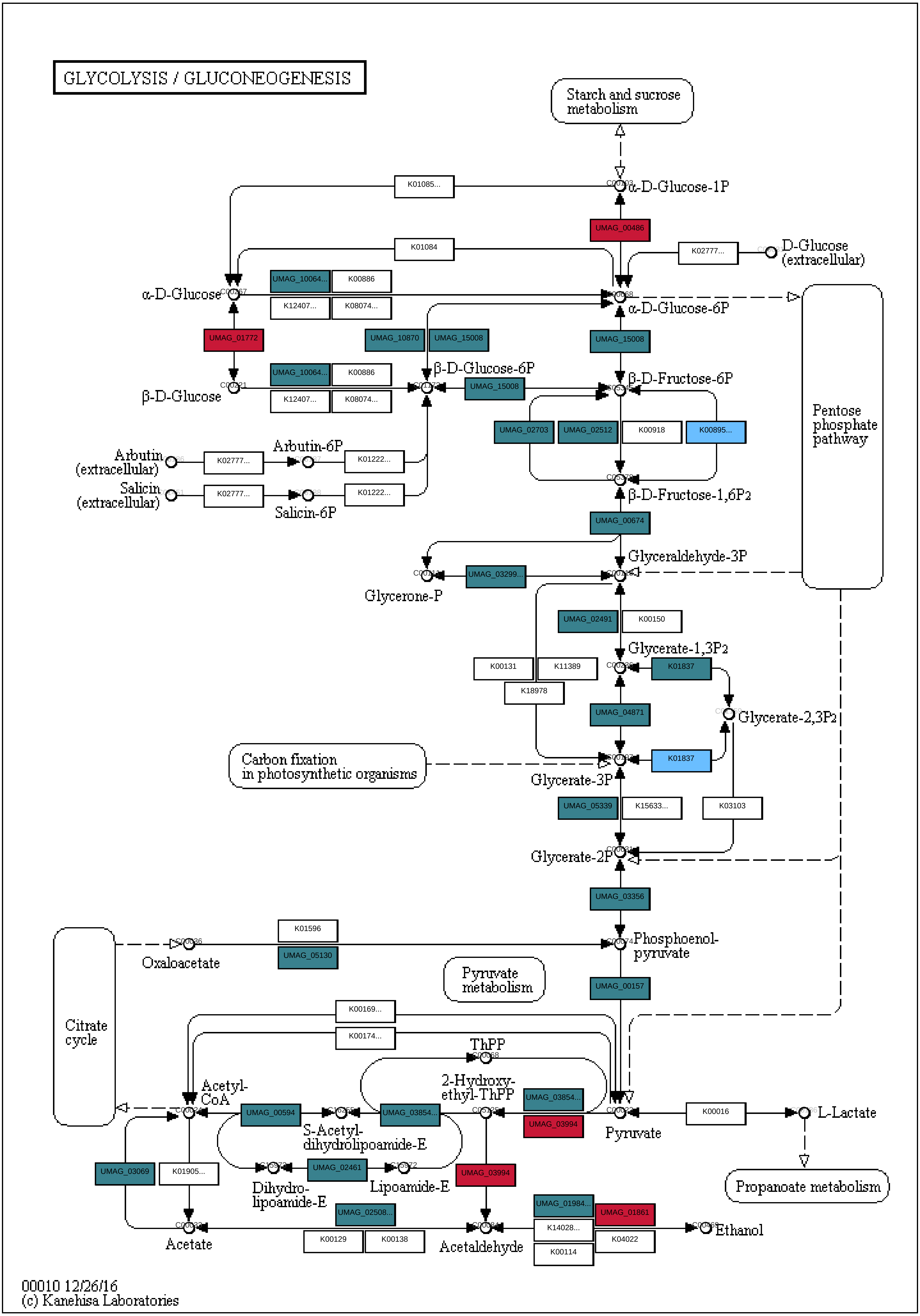
GLYCOLYSIS / GLUCONEOGENESIS
Starch and sucrose metabolism
α-D-Glucose-1P
K01085...
UMAG_00486
K01084
K02777...
D-Glucose (extracellular)
α-D-Glucose
UMAG_10064...
K00886
K12407...
K08074...
α-D-Glucose-6P
UMAG_01772
UMAG_10870
UMAG_15008
UMAG_15008
β-D-Glucose-6P
UMAG_10064...
K00886
UMAG_15008
β-D-Fructose-6P
β-D-Glucose
K12407...
K08074...
Pentose phosphate pathway
UMAG_02703
UMAG_02512
K00918
K00895...
Arbutin (extracellular)
K02777...
Arbutin-6P
K01222...
Salicin (extracellular)
K02777...
K01222...
Salicin-6P
β-D-Fructose-1,6P2
UMAG_00674
Glyceraldehyde-3P
UMAG_03299...
Glycerone-P
UMAG_02491
K00150
Glycerate-1,3P2
K00131
K11389
K01837
K18978
UMAG_04871
Glycerate-2,3P2
Carbon fixation in photosynthetic organisms
K01837
Glycerate-3P
UMAG_05339
K15633...
K03103
Glycerate-2P
UMAG_03356
K01596
UMAG_05130
Phosphoenol-pyruvate
Oxaloacetate
Pyruvate metabolism
UMAG_00157
Citrate cycle
K00169...
K00174...
ThPP
2-Hydroxy-ethyl-ThPP
Acetyl-CoA
UMAG_00594
UMAG_03854...
UMAG_03854...
UMAG_03994
K00016
L-Lactate
Pyruvate
S-Acetyl-dihydrolipoamide-E
UMAG_03069
K01905...
UMAG_02461
UMAG_03994
Propanoate metabolism
Dihydro-lipoamide-E
Lipoamide-E
UMAG_02508...
UMAG_01984...
UMAG_01861
K14028...
K04022
Ethanol
K00129
K00138
K00114
Acetate
Acetaldehyde
00010 12/26/16
(c) Kanehisa Laboratories

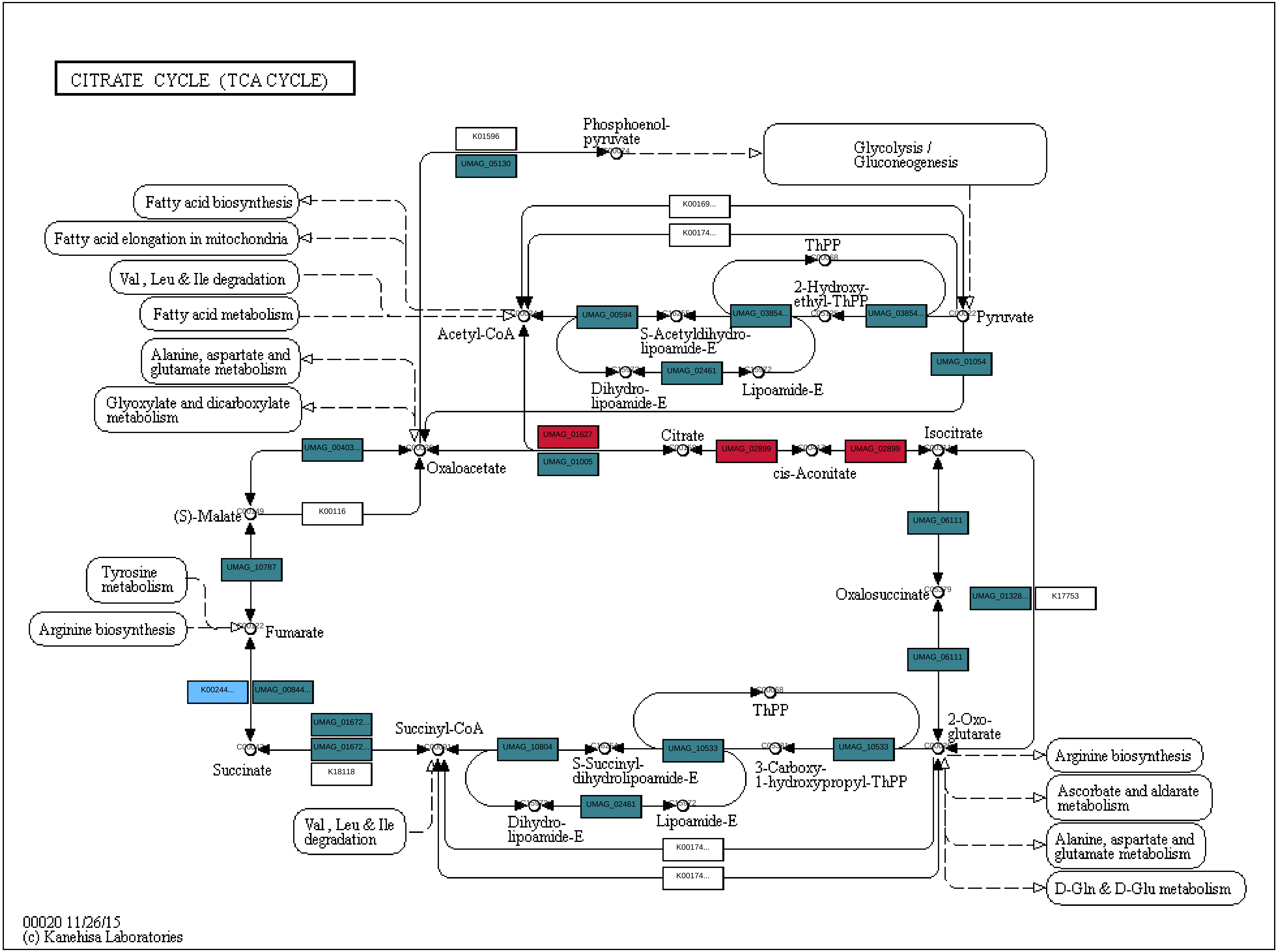
CITRATE CYCLE (TCA CYCLE)
K01596
UMAG_05130
Phosphoenol-
pyruvate
Glycolysis /
Gluconeogenesis
Fatty acid biosynthesis
Fatty acid elongation in mitochondria
Val , Leu & Ile degradation
Fatty acid metabolism
Alanine, aspartate and
glutamate metabolism
Glyoxylate and dicarboxylate
metabolism
K00169...
K00174...
ThPP
2-Hydroxy-
ethyl-ThPP
UMAG_00594
UMAG_03854...
UMAG_03854...
Pyruvate
Acetyl-CoA
S-Acetyldihydro-
lipoamide-E
UMAG_02461
Dihydro-
lipoamide-E
Lipoamide-E
UMAG_01054
UMAG_01627
UMAG_01005
Citrate
UMAG_02899
UMAG_02899
cis-Aconitate
Isocitrate
UMAG_00403...
Oxaloacetate
(S)-Malate
K00116
UMAG_10787
UMAG_06111
Oxalosuccinate
UMAG_01328...
K17753
UMAG_06111
Tyrosine
metabolism
Arginine biosynthesis
Fumarate
K00244...
UMAG_00844...
UMAG_01672...
UMAG_01672...
K18118
Succinate
Succinyl-CoA
UMAG_10804
S-Succinyl-
dihydrolipoamide-E
UMAG_10533
UMAG_10533
ThPP
3-Carboxy-
1-hydroxypropyl-ThPP
2-Oxo-
glutarate
UMAG_02461
Dihydro-
lipoamide-E
Lipoamide-E
Val , Leu & Ile
degradation
K00174...
K00174...
Arginine biosynthesis
Ascorbate and aldarate
metabolism
Alanine, aspartate and
glutamate metabolism
D-Gln & D-Glu metabolism
00020 11/26/15
(c) Kanehisa Laboratories

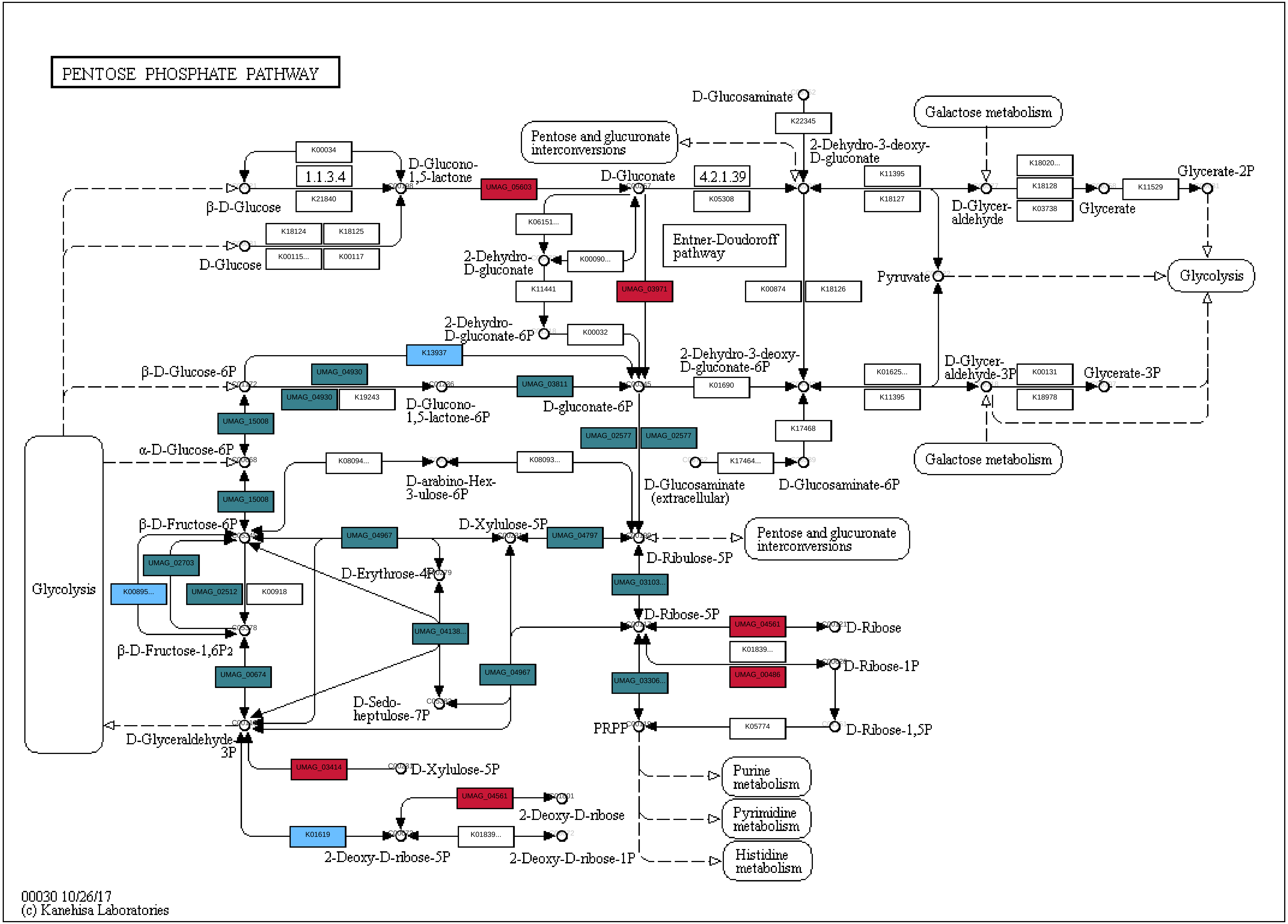

PENTOSE PHOSPHATE PATHWAY
D-Glucosaminate
K22345
Galactose metabolism
Pentose and glucuronate interconversions
2-Dehydro-3-deoxy-D-gluconate
K00034
D-Glucono-1,5-lactone
1.1.3.4
D-Gluconate
4.2.1.39
K18020...
K11395
Glycerate-2P
UMAG_05603
K18128
K11529
K21840
K05308
K18127
D-Glyceraldehyde
Glycerate
β-D-Glucose
K03738
K18124
K18125
K06151...
Entner-Doudoroff pathway
D-Glucose
K00115...
K00117
2-Dehydro-D-gluconate
K00090...
Glycolysis
Pyruvate
K11441
UMAG_03971
K00874
K18126
2-Dehydro-D-gluconate-6P
K00032
K13937
2-Dehydro-3-deoxy-D-gluconate-6P
β-D-Glucose-6P
UMAG_04930
K01625...
D-Glyceraldehyde-3P
K00131
Glycerate-3P
K19243
UMAG_03811
K01690
K11395
K18978
D-Glucono-1,5-lactone-6P
D-gluconate-6P
UMAG_15008
UMAG_02577
K17468
α-D-Glucose-6P
K08094...
K08093...
K17464...
D-arabino-Hex-3-ulose-6P
D-Glucosaminate (extracellular)
D-Glucosaminate-6P
β-D-Fructose-6P
D-Xylulose-5P
UMAG_04967
UMAG_04797
D-Ribulose-5P
UMAG_02703
D-Erythrose-4P
K00895...
UMAG_02512
K00918
UMAG_03103...
D-Ribose-5P
UMAG_04138...
UMAG_04561
D-Ribose
β-D-Fructose-1,6P2
K01839...
D-Ribose-1P
UMAG_00674
UMAG_00486
UMAG_03306...
D-Sedo-heptulose-7P
PRPP
K05774
D-Ribose-1,5P
D-Glyceraldehyde-3P
UMAG_03414
Purine metabolism
2-Deoxy-D-ribose
Pyrimidine metabolism
K01619
2-Deoxy-D-ribose-5P
2-Deoxy-D-ribose-1P
Histidine metabolism
00030 10/26/17
(c) Kanehisa Laboratories

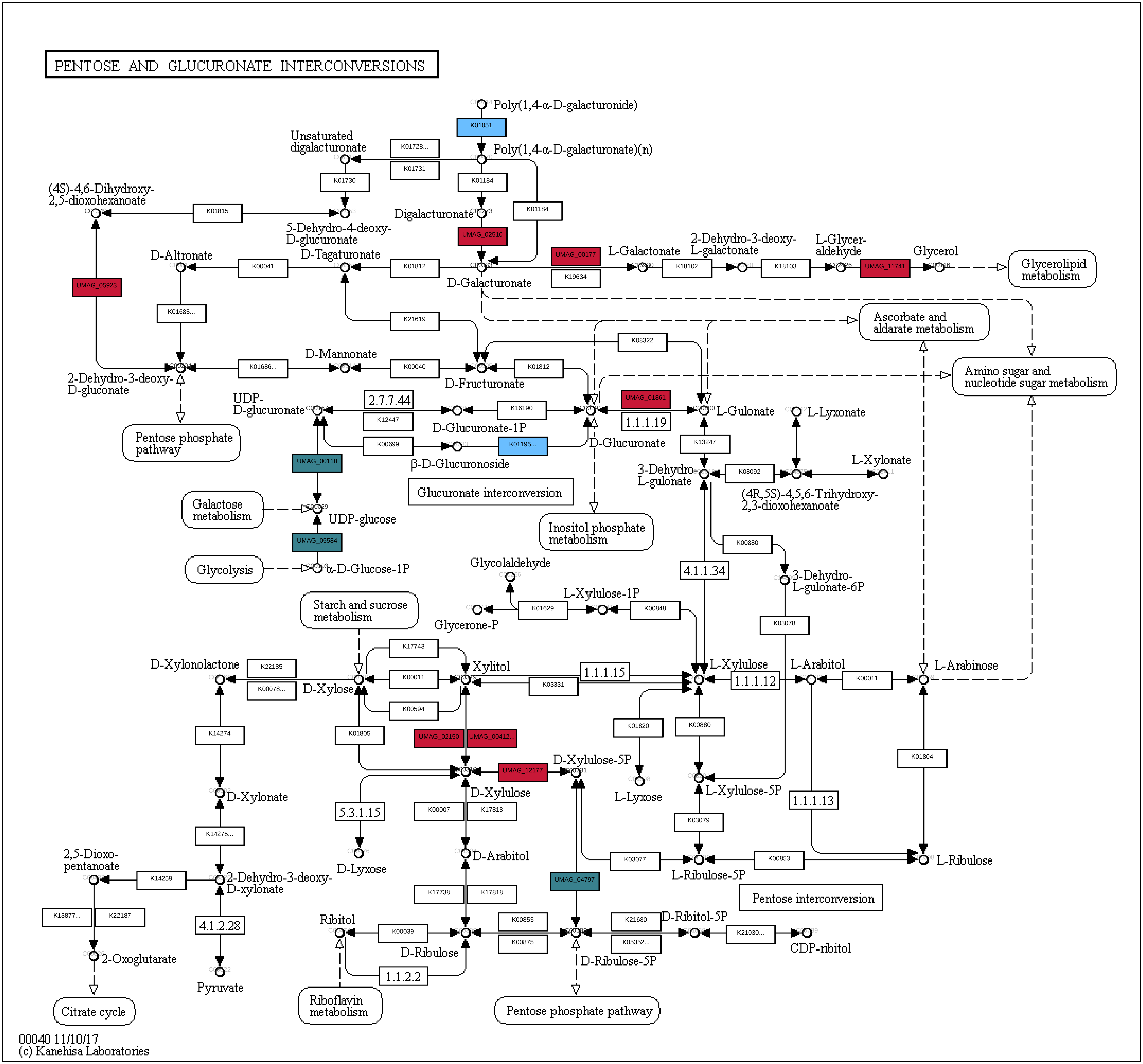
PENTOSE AND GLUCURONATE INTERCONVERSIONS
Poly(1,4-α-D-galacturonide)
K01051
Poly(1,4-α-D-galacturonate)(n)
Unsaturated digalacturonate
K01728...
K01731
K01730
K01184
K01184
(4S)-4,6-Dihydroxy-2,5-dioxohexanoate
K01815
5-Dehydro-4-deoxy-D-glucuronate
Digalacturonate
UMAG_02510
UMAG_00177
K19634
L-Galactonate
2-Dehydro-3-deoxy-L-galactonate
L-Glycer-aldehyde
Glycerol
K18102
K18103
UMAG_11741
Glycerolipid metabolism
D-Altronate
K00041
D-Tagaturonate
K01812
D-Galacturonate
UMAG_05923
K01685...
K21619
Ascorbate and aldarate metabolism
K08322
Amino sugar and nucleotide sugar metabolism
D-Mannonate
K01686...
K00040
K01812
2-Dehydro-3-deoxy-D-gluconate
D-Fructuronate
UDP-D-glucuronate
2.7.7.44
K12447
K16190
D-Glucuronate-1P
UMAG_01861
1.1.1.19
L-Gulonate
L-Lyxonate
Pentose phosphate pathway
K00699
K01195...
β-D-Glucuronoside
D-Glucuronate
K13247
UMAG_00118
L-Xylonate
3-Dehydro-L-gulonate
K08092
(4R,5S)-4,5,6-Trihydroxy-2,3-dioxohexanoate
Glucuronate interconversion
Galactose metabolism
UDP-glucose
Inositol phosphate metabolism
UMAG_05584
K00880
Glycolysis
α-D-Glucose-1P
Glycolaldehyde
4.1.1.34
3-Dehydro-L-gulonate-6P
L-Xylulose-1P
Starch and sucrose metabolism
K01629
K00848
Glycerone-P
K03078
K17743
D-Xylonolactone
K22185
Xylitol
1.1.1.15
L-Xylulose
L-Arabitol
L-Arabinose
K00078...
D-Xylose
K00011
K03331
1.1.1.12
K00011
K00594
K14274
K01805
UMAG_02150
UMAG_00412...
K01820
K00880
K01804
UMAG_12177
D-Xylulose-5P
D-Xylonate
D-Xylulose
L-Lyxose
L-Xylulose-5P
5.3.1.15
K00007
K17818
1.1.1.13
K14275...
K03079
D-Lyxose
D-Arabitol
2,5-Dioxo-pentanoate
K14259
2-Dehydro-3-deoxy-D-xylonate
UMAG_04797
K03077
K00853
L-Ribulose
L-Ribulose-5P
K17738
K17818
Pentose interconversion
K13877...
K22187
4.1.2.28
Ribitol
K00039
K00853
K21680
D-Ribitol-5P
K00875
K05352...
K21030...
CDP-ribitol
2-Oxoglutarate
D-Ribulose
D-Ribulose-5P
1.1.2.2
Pyruvate
Citrate cycle
Riboflavin metabolism
Pentose phosphate pathway
00040 11/10/17
(c) Kanehisa Laboratories

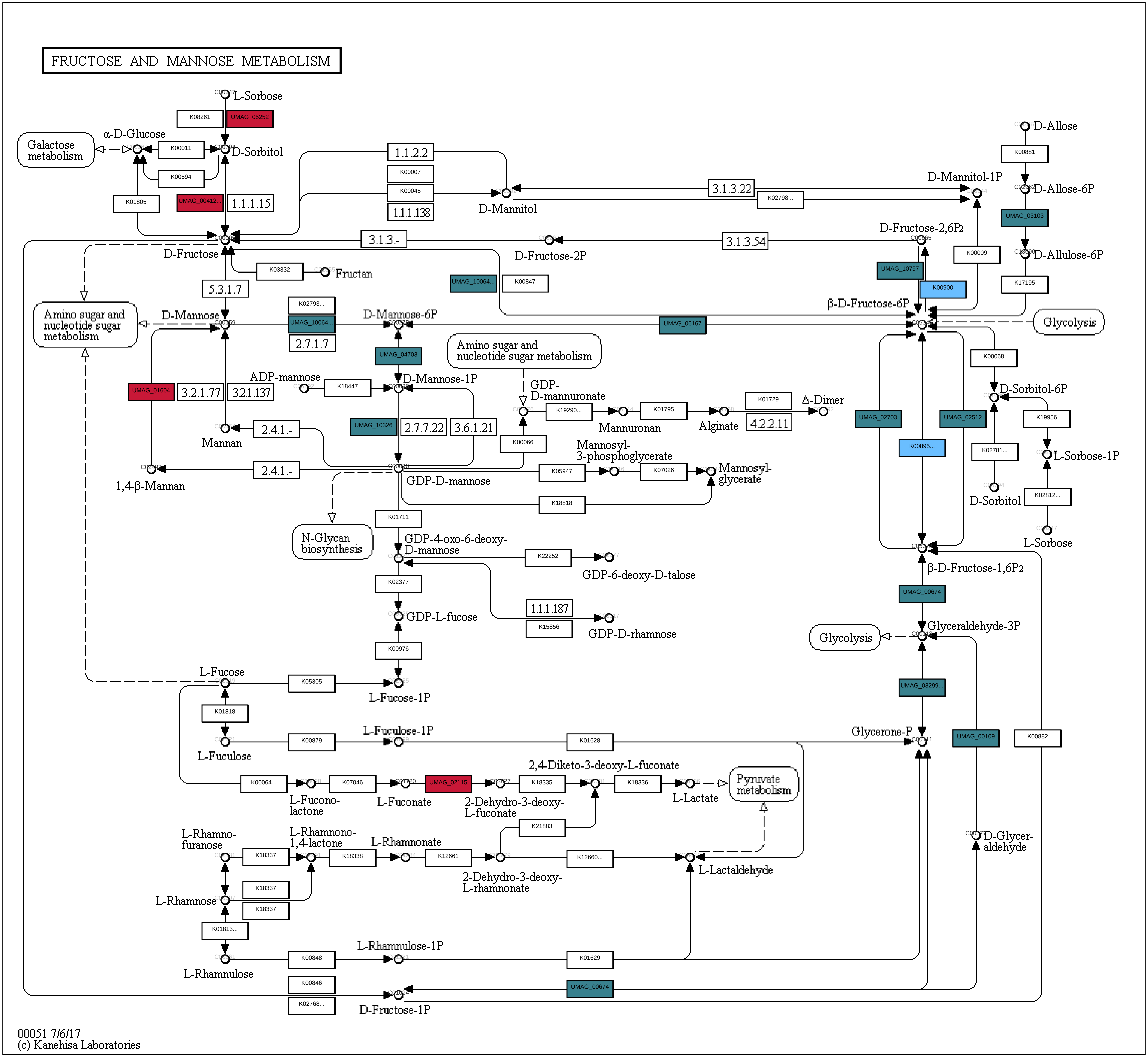
FRUCTOSE AND MANNOSE METABOLISM
L-Sorbose
K08261
UMAG_05252
Galactose metabolism
α-D-Glucose
K00011
D-Sorbitol
K00594
1.1.2.2
K00007
K00045
1.1.1.138
K01805
UMAG_00412...
1.1.1.15
D-Mannitol
3.1.3.22
K02798...
D-Mannitol-1P
D-Allose
K00881
D-Allose-6P
UMAG_03103
D-Allulose-6P
K17195
3.1.3.-
D-Fructose-2P
3.1.3.54
D-Fructose-2,6P2
K00009
D-Fructose
K03332
Fructan
UMAG_10064...
K00847
UMAG_10797
K00900
5.3.1.7
Amino sugar and nucleotide sugar metabolism
D-Mannose
K02793...
UMAG_10064...
2.7.1.7
D-Mannose-6P
UMAG_06167
β-D-Fructose-6P
Glycolysis
UMAG_04703
Amino sugar and nucleotide sugar metabolism
K00068
D-Sorbitol-6P
UMAG_01604
3.2.1.77
3.2.1.137
ADP-mannose
K18447
D-Mannose-1P
GDP-D-mannuronate
K19290...
Mannuronan
K01795
Alginate
K01729
4.2.2.11
Δ-Dimer
UMAG_02703
UMAG_02512
K19956
UMAG_10326
2.7.7.22
3.6.1.21
K00066
Mannan
2.4.1.-
Mannosyl-3-phosphoglycerate
K05947
K07026
Mannosyl-glycerate
K00895...
K02781...
L-Sorbose-1P
1,4-β-Mannan
2.4.1.-
GDP-D-mannose
K18818
D-Sorbitol
K02812...
N-Glycan biosynthesis
K01711
GDP-4-oxo-6-deoxy-D-mannose
K22252
GDP-6-deoxy-D-talose
L-Sorbose
β-D-Fructose-1,6P2
UMAG_00674
K02377
1.1.1.187
K15856
GDP-D-rhamnose
GDP-L-fucose
Glycolysis
Glyceraldehyde-3P
K00976
UMAG_03299...
L-Fucose
K05305
L-Fucose-1P
K01818
L-Fuculose-1P
L-Fuculose
K00879
K01628
Glycerone-P
UMAG_00109
K00882
2,4-Diketo-3-deoxy-L-fuconate
K00064...
K07046
UMAG_02115
K18335
K18336
Pyruvate metabolism
L-Fucono-lactone
L-Fuconate
2-Dehydro-3-deoxy-L-fuconate
L-Lactate
K21883
L-Rhamno-furanose
K18337
L-Rhamnono-1,4-lactone
K18338
L-Rhamnonate
K12661
K12660...
D-Glycer-aldehyde
2-Dehydro-3-deoxy-L-rhamnonate
L-Lactaldehyde
K18337
L-Rhamnose
K18337
K01813...
L-Rhamnulose-1P
K00848
K01629
L-Rhamnulose
K00846
UMAG_00674
K02768...
D-Fructose-1P
00051 7/6/17
(c) Kanehisa Laboratories

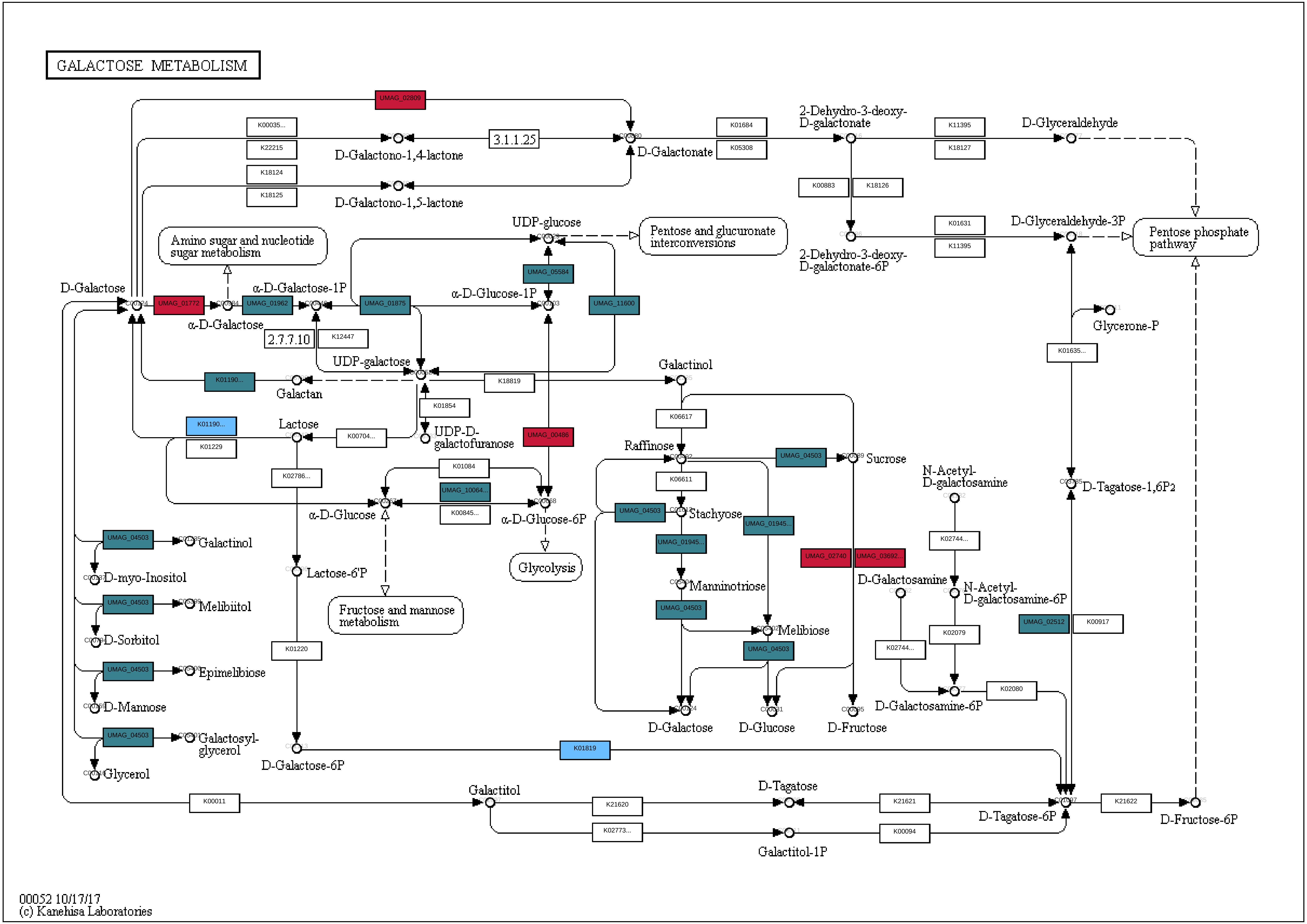
GALACTOSE METABOLISM
UMAG_02809
K00035...
K22215
K18124
K18125
D-Galactono-1,4-lactone
D-Galactono-1,5-lactone
3.1.1.25
D-Galactonate
K01684
K05308
2-Dehydro-3-deoxy-D-galactonate
K00883
K18126
2-Dehydro-3-deoxy-D-galactonate-6P
K11395
K18127
D-Glyceraldehyde
K01631
K11395
D-Glyceraldehyde-3P
Pentose phosphate pathway
Amino sugar and nucleotide sugar metabolism
UDP-glucose
Pentose and glucuronate interconversions
UMAG_05584
D-Galactose
UMAG_01772
UMAG_01962
α-D-Galactose-1P
UMAG_01875
α-D-Glucose-1P
UMAG_11600
α-D-Galactose
2.7.7.10
K12447
UDP-galactose
Glycerone-P
K01635...
K01190...
Galactan
K18819
Galactinol
K01854
UDP-D-galactofuranose
UMAG_00486
K01190...
K01229
Lactose
K00704...
K06617
Raffinose
UMAG_04503
Sucrose
K02786...
K01084
UMAG_10064...
K00845...
α-D-Glucose
α-D-Glucose-6P
K06611
UMAG_04503
Stachyose
UMAG_01945...
UMAG_01945...
N-Acetyl-D-galactosamine
D-Tagatose-1,6P2
UMAG_04503
Galactinol
D-myo-Inositol
Lactose-6P
Glycolysis
Manninotriose
UMAG_02740
UMAG_03692...
K02744...
D-Galactosamine
N-Acetyl-D-galactosamine-6P
UMAG_04503
Melibiitol
D-Sorbitol
Fructose and mannose metabolism
UMAG_04503
Melibiose
UMAG_04503
K02079
K02744...
UMAG_02512
K00917
K01220
UMAG_04503
Epimelibiose
D-Mannose
K02080
D-Galactosamine-6P
D-Galactose
D-Glucose
D-Fructose
UMAG_04503
Galactosyl-glycerol
Glycerol
D-Galactose-6P
K01819
K00011
Galactitol
K21620
D-Tagatose
K21621
K21622
K02773...
K00094
D-Tagatose-6P
D-Fructose-6P
Galactitol-1P
00052 10/17/17
(c) Kanehisa Laboratories

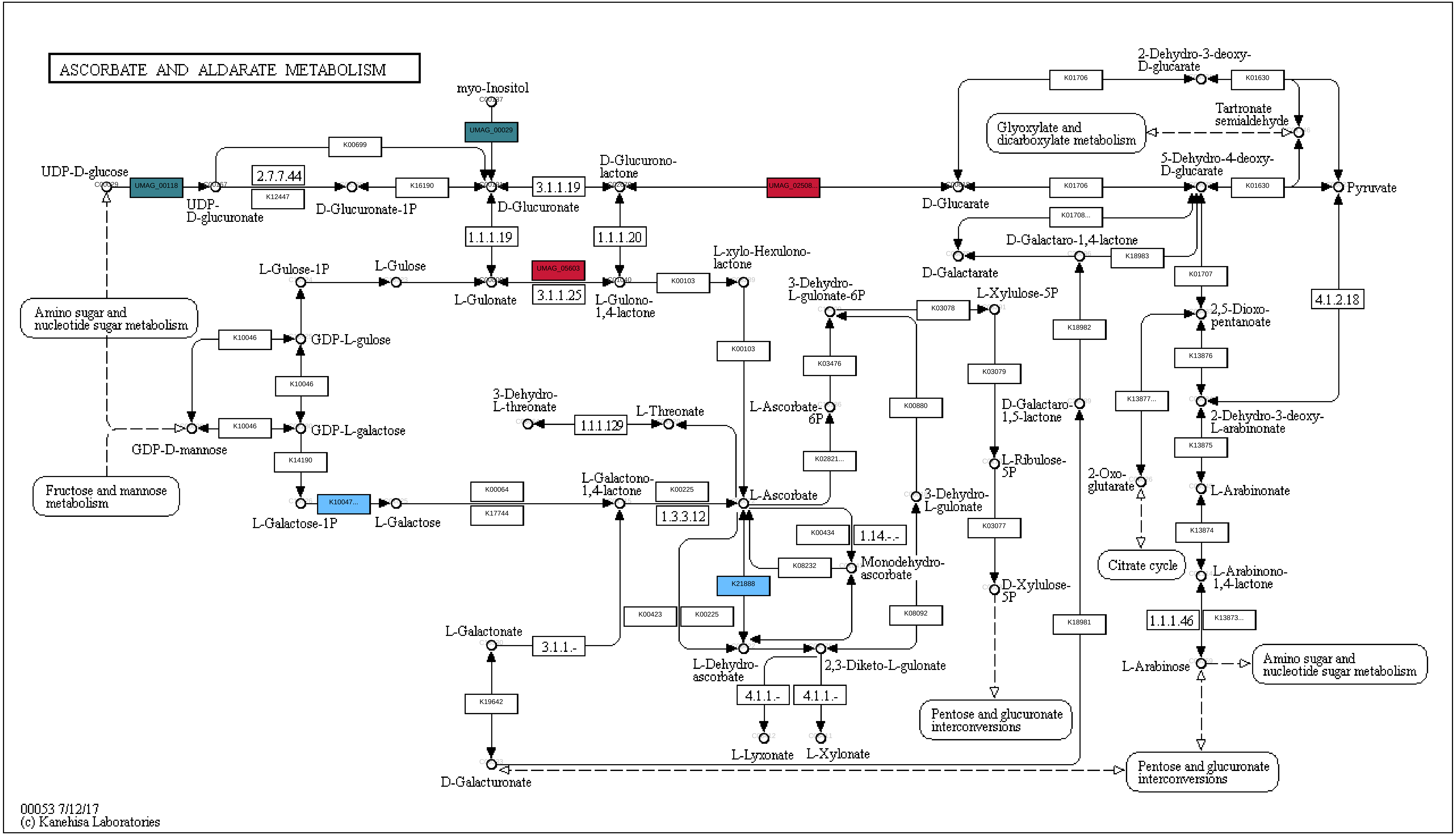
ASCORBATE AND ALDARATE METABOLISM
myo-Inositol
UMAG_00029
K00699
UDP-D-glucose
UMAG_00118
2.7.7.44
K12447
UDP-D-glucuronate
D-Glucuronate-1P
K16190
D-Glucuronate
3.1.1.19
D-Glucurono-lactone
UMAG_02508...
D-Glucarate
K01706
5-Dehydro-4-deoxy-D-glucarate
K01630
Pyruvate
2-Dehydro-3-deoxy-D-glucarate
Tartronate semialdehyde
Glyoxylate and dicarboxylate metabolism
K01708...
D-Galactaro-1,4-lactone
K18983
D-Galactarate
K01707
2,5-Dioxo-pentanoate
4.1.2.18
1.1.1.19
1.1.1.20
UMAG_05603
3.1.1.25
L-Gulose-1P
L-Gulose
L-Gulonate
L-Gulono-1,4-lactone
K00103
L-xylo-Hexulono-lactone
3-Dehydro-L-gulonate-6P
K03078
L-Xylulose-5P
Amino sugar and nucleotide sugar metabolism
K10046
GDP-L-gulose
K00103
K03476
K18982
K13876
K13877...
K10046
K00880
K03079
D-Galactaro-1,5-lactone
2-Dehydro-3-deoxy-L-arabinonate
3-Dehydro-L-threonate
1.11.129
L-Threonate
L-Ascorbate-6P
GDP-L-galactose
K10046
GDP-D-mannose
K14190
K02821...
L-Ribulose-5P
K13875
2-Oxo-glutarate
L-Arabinonate
Fructose and mannose metabolism
K10047...
L-Galactose-1P
L-Galactose
K00064
K17744
L-Galactono-1,4-lactone
K00225
1.3.3.12
L-Ascorbate
3-Dehydro-L-gulonate
K03077
K13874
K00434
1.14.-.-
K08232
Monodehydro-ascorbate
Citrate cycle
L-Arabinono-1,4-lactone
K21888
D-Xylulose-5P
K00423
K00225
K08092
K18981
1.1.1.46
K13873...
L-Galactonate
3.1.1.-
L-Dehydro-ascorbate
2,3-Diketo-L-gulonate
L-Arabinose
Amino sugar and nucleotide sugar metabolism
K19642
4.1.1.-
4.1.1.-
Pentose and glucuronate interconversions
L-Lyxonate
L-Xylonate
D-Galacturonate
Pentose and glucuronate interconversions
00053 7/12/17
(c) Kanehisa Laboratories

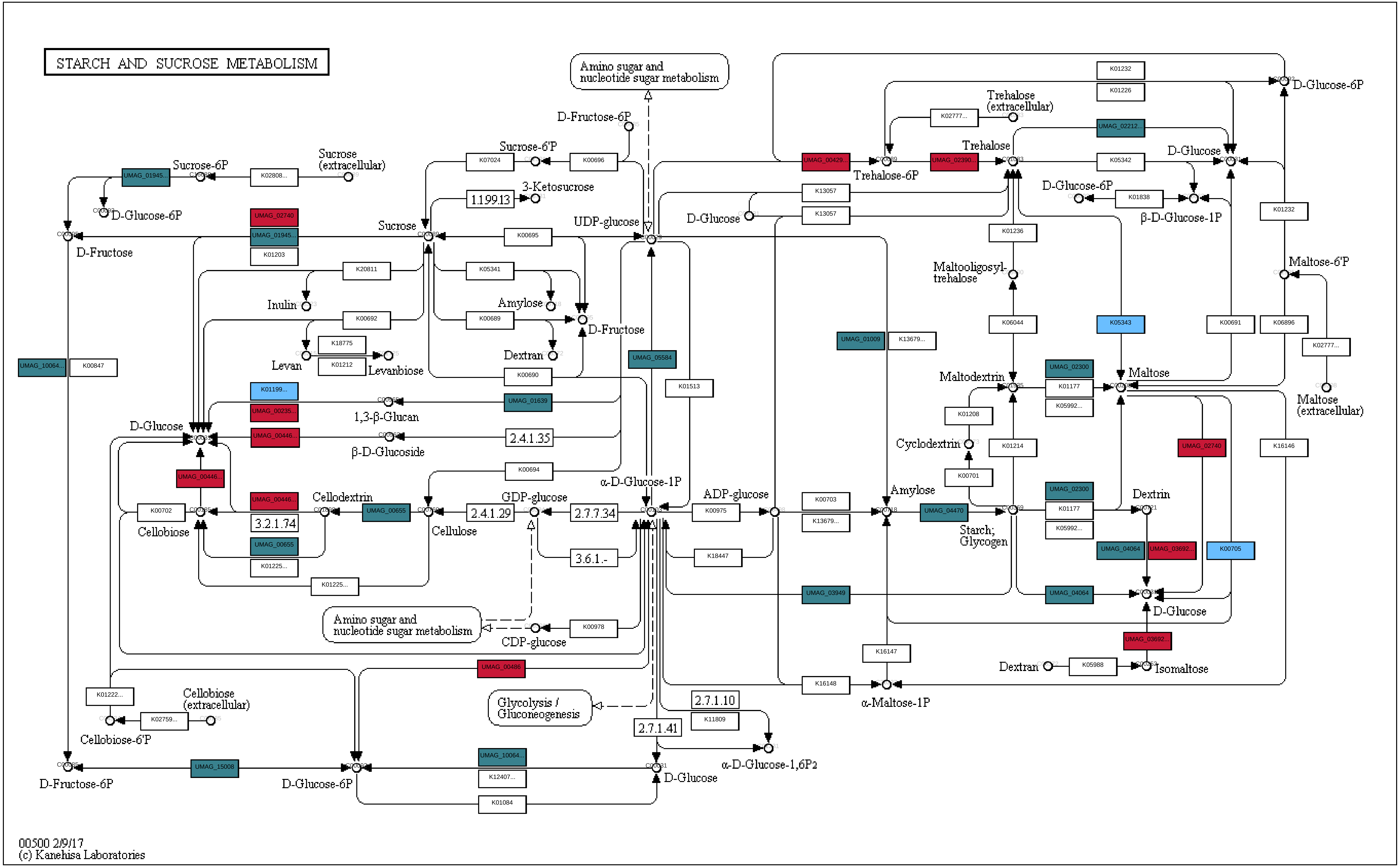

STARCH AND SUCROSE METABOLISM
Amino sugar and nucleotide sugar metabolism
D-Fructose-6P
Sucrose-6'P
K07024
K00696
Sucrose (extracellular)
Sucrose-6P
UMAG_01945...
K02808...
D-Glucose-6P
3-Ketosucrose
1.1.99.13
UMAG_02740
UMAG_01945...
K01203
D-Fructose
Sucrose
K00695
UDP-glucose
K20811
K05341
Inulin
Amylose
K00692
K00689
D-Fructose
K18775
Levan
K01212
Levanbiose
Dextran
K00690
UMAG_10064...
K00847
UMAG_05584
K01513
K01199...
UMAG_01639
UMAG_00235...
1,3-β-Glucan
D-Glucose
UMAG_00446...
2.4.1.35
β-D-Glucoside
K00694
UMAG_00446...
α-D-Glucose-1P
UMAG_00446...
Cellodextrin
GDP-glucose
K00702
UMAG_00655
2.4.1.29
2.7.7.34
Cellobiose
3.2.1.74
Cellulose
UMAG_00655
3.6.1.-
K01225...
K01225...
Amino sugar and nucleotide sugar metabolism
K00978
CDP-glucose
UMAG_00486
K01222...
Cellobiose (extracellular)
Glycolysis / Gluconeogenesis
2.7.1.10
K02759...
K11809
Cellobiose-6'P
2.7.1.41
α-D-Glucose-1,6P2
UMAG_15008
UMAG_10064...
K12407...
D-Fructose-6P
D-Glucose-6P
D-Glucose
K01084
K01232
D-Glucose-6P
K01226
Trehalose (extracellular)
K02777...
UMAG_02212...
Trehalose
D-Glucose
UMAG_00429...
UMAG_02390...
K05342
Trehalose-6P
K13057
D-Glucose-6P
K01838
D-Glucose
K13057
β-D-Glucose-1P
K01232
K01236
Maltooligosyl-trehalose
Maltose-6'P
UMAG_01009
K13679...
K06044
K05343
K00691
K06896
K02777...
UMAG_02300
Maltose
Maltodextrin
K01177
K05992...
Maltose (extracellular)
K01208
Cyclodextrin
K01214
UMAG_02740
K16146
K00701
UMAG_02300
Amylose
ADP-glucose
Dextrin
K00703
K00975
UMAG_04470
K01177
K13679...
Starch; Glycogen
K05992...
UMAG_04064
UMAG_03692...
K00705
K18447
UMAG_03949
UMAG_04064
D-Glucose
UMAG_03692...
K16147
Dextran
K05988
Isomaltose
K16148
α-Maltose-1P
00500 2/9/17
(c) Kanehisa Laboratories

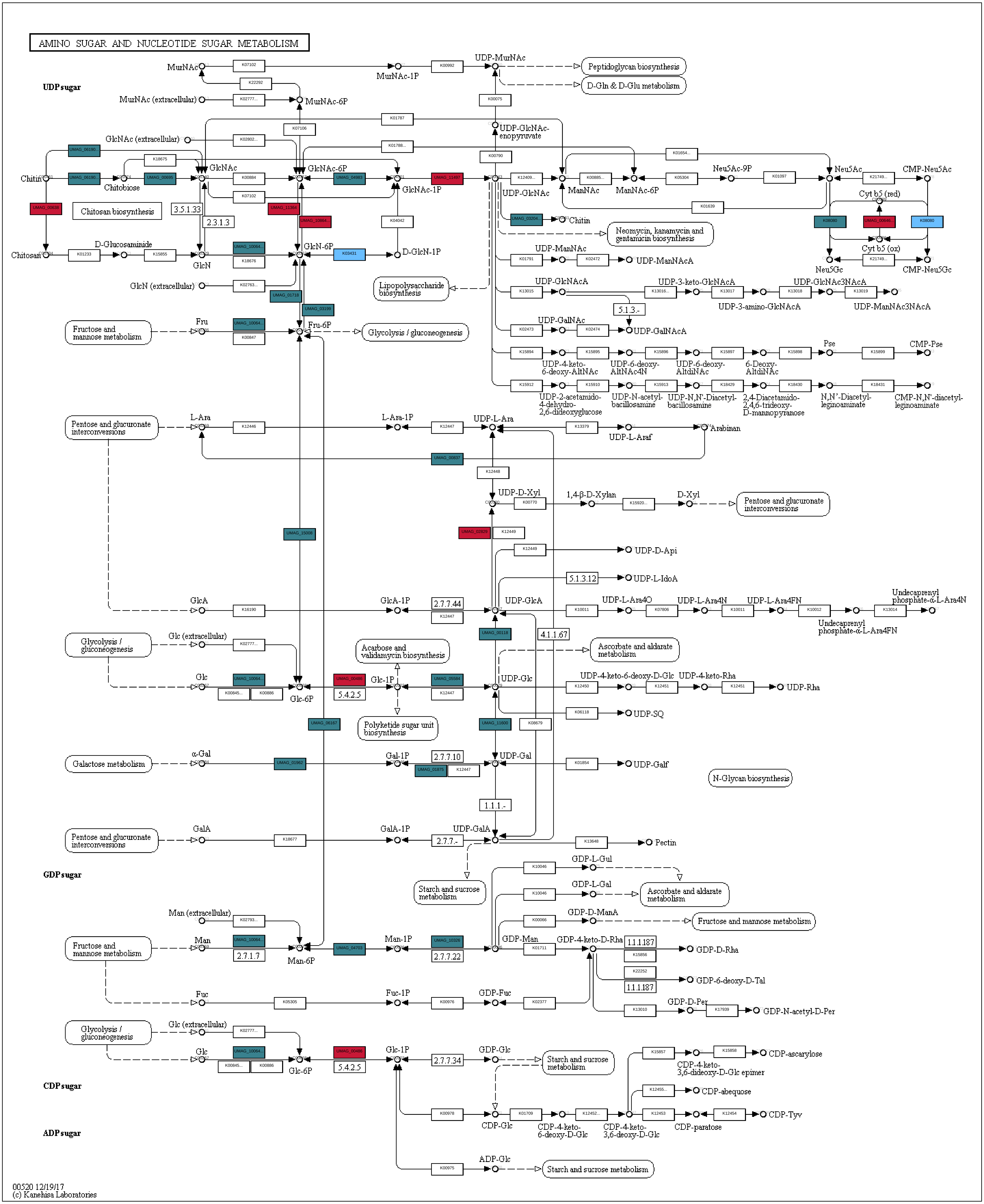

AMINO SUGAR AND NUCLEOTIDE SUGAR METABOLISM
UDP sugar
MurNAc
K07102
MurNAc-1P
K00992
UDP-MurNAc
Peptidoglycan biosynthesis
D-Gln & D-Glu metabolism
K22292
MurNAc (extracellular)
K02777...
MurNAc-6P
K00075
K01787
K07106
UDP-GlcNAc-enopyruvate
GlcNAc (extracellular)
K02802...
K01788...
K00790
UMAG_06190...
K18675
GlcNAc
GlcNAc-6P
K01654...
Neu5Ac-9P
Neu5Ac
CMP-Neu5Ac
Chitin
UMAG_06190...
Chitobiose
UMAG_00695
K00884
UMAG_04983
UMAG_11497
GlcNAc-1P
K12409...
UDP-GlcNAc
K00885...
ManNAc
ManNAc-6P
K05304
K01097
K21749...
K07102
K01639
Cyt b5 (red)
UMAG_00638
Chitosan biosynthesis
3.5.1.33
2.3.1.3
UMAG_11364
UMAG_10864...
K04042
UMAG_03204...
Chitin
Neomycin, kanamycin and gentamicin biosynthesis
K08080
UMAG_00646...
K08080
Cyt b5 (ox)
D-Glucosaminide
Chitosan
K01233
K15855
GlcN
UMAG_10064...
K18676
GlcN-6P
K03431
D-GlcN-1P
UDP-ManNAc
K01791
K02472
UDP-ManNAcA
Neu5Gc
K21749...
CMP-Neu5Gc
GlcN (extracellular)
K02763...
UMAG_01718
UMAG_03199
Lipopolysaccharide biosynthesis
UDP-GlcNAcA
K13015
UDP-3-keto-GlcNAcA
K13016...
K13017
UDP-3-amino-GlcNAcA
UDP-GlcNAc3NAcA
K13018
K13019
UDP-ManNAc3NAcA
5.1.3.-
Fructose and mannose metabolism
Fru
UMAG_10064...
K00847
Fru-6P
Glycolysis / gluconeogenesis
UDP-GalNAc
K02473
K02474
UDP-GalNAcA
K15894
K15895
K15896
K15897
K15898
Pse
K15899
CMP-Pse
UDP-4-keto-6-deoxy-AltNAc
UDP-6-deoxy-AltNAc4N
UDP-6-deoxy-AltdiNAc
6-Deoxy-AltdiNAc
K15912
K15910
K15913
K18429
K18430
K18431
UDP-2-acetamido-4-dehydro-2,6-dideoxyglucose
UDP-N-acetyl-bacillosamine
UDP-N,N'-Diacetyl-bacillosamine
2,4-Diacetamido-2,4,6-trideoxy-D-mannopyranose
N,N'-Diacetyl-legionaminate
CMP-N,N'-diacetyl-legionaminate
Pentose and glucuronate interconversions
L-Ara
K12446
L-Ara-1P
K12447
UDP-L-Ara
K13379
UDP-L-Araf
Arabinan
UMAG_00837
K12448
UDP-D-Xyl
K00770
1,4-β-D-Xylan
K15920...
D-Xyl
Pentose and glucuronate interconversions
UMAG_15008
UMAG_02829
K12449
K12449
UDP-D-Api
5.1.3.12
UDP-L-IdoA
GlcA
K16190
GlcA-1P
2.7.7.44
K12447
UDP-GlcA
K10011
UDP-L-Ara4O
K07806
UDP-L-Ara4N
K10011
UDP-L-Ara4FN
K10012
K13014
Undecaprenyl phosphate-α-L-Ara4N
Undecaprenyl phosphate-α-L-Ara4FN
UMAG_00118
4.1.1.67
Glycolysis / gluconeogenesis
Glc (extracellular)
K02777...
Acarbose and validamycin biosynthesis
Ascorbate and aldarate metabolism
Glc
UMAG_10064...
K00845...
K00886
Glc-6P
UMAG_00486
5.4.2.5
Glc-1P
UMAG_05584
K12447
UDP-Glc
UDP-4-keto-6-deoxy-D-Glc
K12450
UDP-4-keto-Rha
K12451
K12451
UDP-Rha
K06118
UDP-SQ
UMAG_06167
Polyketide sugar unit biosynthesis
UMAG_11600
K08679
Galactose metabolism
α-Gal
UMAG_01962
Gal-1P
UMAG_01875
2.7.7.10
K12447
UDP-Gal
K01854
UDP-Galf
N-Glycan biosynthesis
1.1.1.-
Pentose and glucuronate interconversions
GalA
K18677
GalA-1P
2.7.7.-
UDP-GalA
K13648
Pectin
GDP sugar
K10046
GDP-L-Gul
Starch and sucrose metabolism
K10046
GDP-L-Gal
Ascorbate and aldarate metabolism
Man (extracellular)
K02793...
K00066
GDP-D-ManA
Fructose and mannose metabolism
Fructose and mannose metabolism
Man
UMAG_10064...
2.7.1.7
Man-6P
UMAG_04703
Man-1P
UMAG_10326
2.7.7.22
GDP-Man
K01711
GDP-4-keto-D-Rha
1.1.1.187
K15856
GDP-D-Rha
K22252
1.1.1.187
GDP-6-deoxy-D-Tal
Fuc
K05305
Fuc-1P
K00976
GDP-Fuc
K02377
K13010
GDP-D-Per
K17939
GDP-N-acetyl-D-Per
Glycolysis / gluconeogenesis
Glc (extracellular)
K02777...
Glc
UMAG_10064...
K00845...
K00886
Glc-6P
UMAG_00486
5.4.2.5
Glc-1P
2.7.7.34
GDP-Glc
Starch and sucrose metabolism
K15857
K15858
CDP-ascarylose
CDP-4-keto-3,6-dideoxy-D-Glc epimer
K12455...
CDP-abequose
CDP sugar
K00978
CDP-Glc
K01709
CDP-4-keto-6-deoxy-D-Glc
K12452...
CDP-4-keto-3,6-deoxy-D-Glc
K12453
CDP-paratose
K12454
CDP-Tyv
ADP sugar
K00975
ADP-Glc
Starch and sucrose metabolism
00520 12/19/17
(c) Kanehisa Laboratories

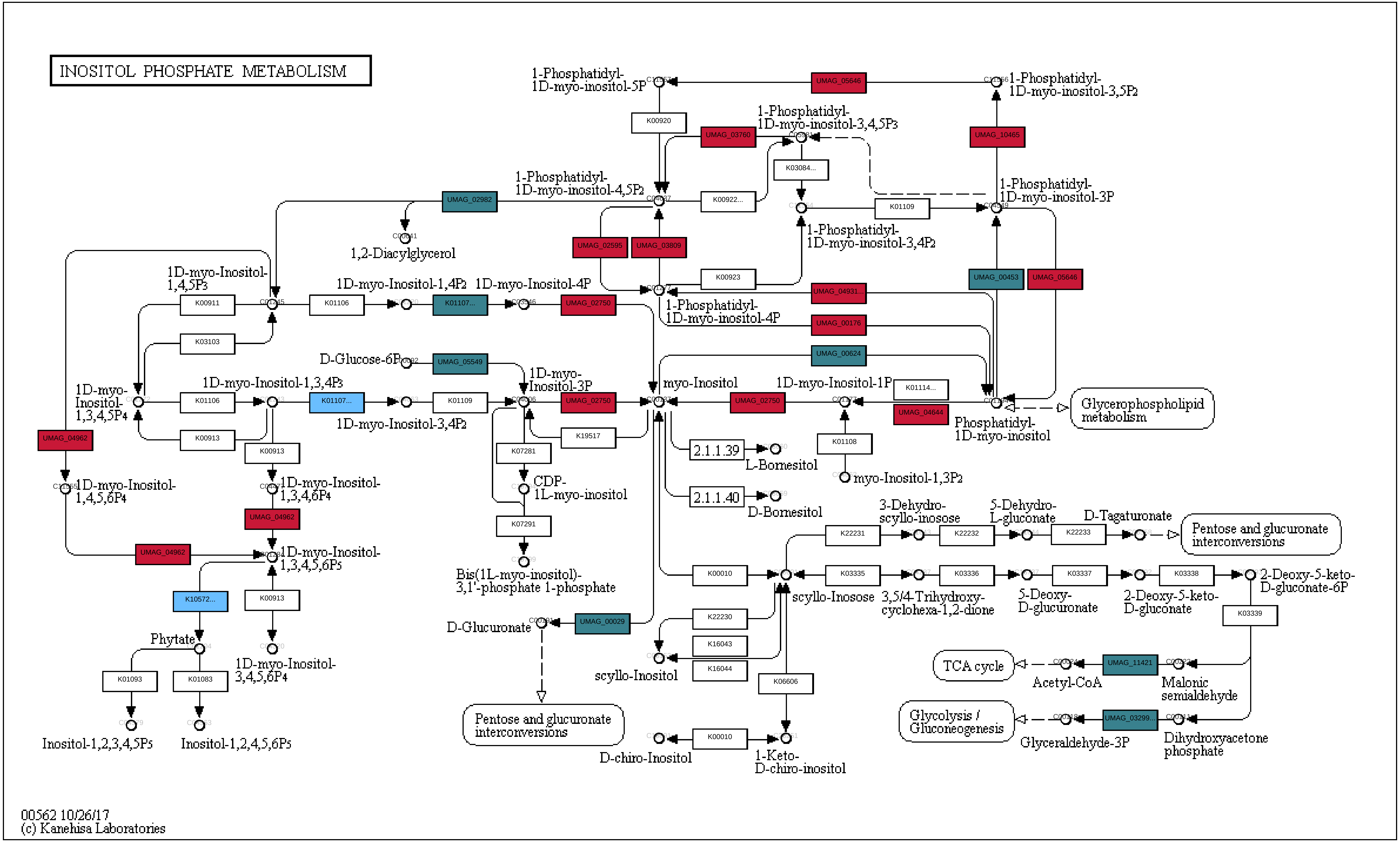
INOSITOL PHOSPHATE METABOLISM
1-Phosphatidyl-1D-myo-inositol-5P
UMAG_05646
1-Phosphatidyl-1D-myo-inositol-3,5P2
K00920
UMAG_03760
1-Phosphatidyl-1D-myo-inositol-3,4,5P3
UMAG_10465
K03084...
1-Phosphatidyl-1D-myo-inositol-4,5P2
UMAG_02982
K00922...
K01109
1-Phosphatidyl-1D-myo-inositol-3P
1,2-Diacylglycerol
UMAG_02595
UMAG_03809
1-Phosphatidyl-1D-myo-inositol-3,4P2
1D-myo-Inositol-1,4,5P3
K00923
UMAG_00453
UMAG_05646
1D-myo-Inositol-1,4P2
1D-myo-Inositol-4P
K00911
K01106
K01107...
UMAG_02750
UMAG_04931...
1-Phosphatidyl-1D-myo-inositol-4P
UMAG_00176
K03103
D-Glucose-6P
UMAG_05549
UMAG_00624
1D-myo-Inositol-3P
myo-Inositol
1D-myo-Inositol-1P
K01114...
1D-myo-Inositol-1,3,4P3
1D-myo-Inositol-1,3,4,5P4
K01106
K01107...
K01109
UMAG_02750
UMAG_02750
UMAG_04644
Glycerophospholipid metabolism
1D-myo-Inositol-3,4P2
Phosphatidyl-1D-myo-inositol
UMAG_04962
K00913
K19517
K00913
K07281
K01108
2.1.1.39
L-Bornesitol
myo-Inositol-1,3P2
1D-myo-Inositol-1,4,5,6P4
1D-myo-Inositol-1,3,4,6P4
CDP-1L-myo-inositol
2.1.1.40
D-Bornesitol
UMAG_04962
K07291
3-Dehydro-scyllo-inosose
5-Dehydro-L-gluconate
D-Tagaturonate
K22231
K22232
K22233
Pentose and glucuronate interconversions
UMAG_04962
1D-myo-Inositol-1,3,4,5,6P5
Bis(1L-myo-inositol)-3,1'-phosphate 1-phosphate
K00010
K03335
K03336
K03337
K03338
2-Deoxy-5-keto-D-gluconate-6P
K10572...
K00913
scyllo-Inosose
3,5/4-Trihydroxy-cyclohexa-1,2-dione
5-Deoxy-D-glucuronate
2-Deoxy-5-keto-D-gluconate
K03339
K22230
D-Glucuronate
UMAG_00029
Phytate
K16043
K16044
TCA cycle
UMAG_11421
Acetyl-CoA
Malonic semialdehyde
1D-myo-Inositol-3,4,5,6P4
scyllo-Inositol
K06606
K01093
K01083
Pentose and glucuronate interconversions
Glycolysis / Gluconeogenesis
UMAG_03299...
Glyceraldehyde-3P
Dihydroxyacetone phosphate
K00010
Inositol-1,2,3,4,5P5
Inositol-1,2,4,5,6P5
D-chiro-Inositol
1-Keto-D-chiro-inositol
00562 10/26/17
(c) Kanehisa Laboratories

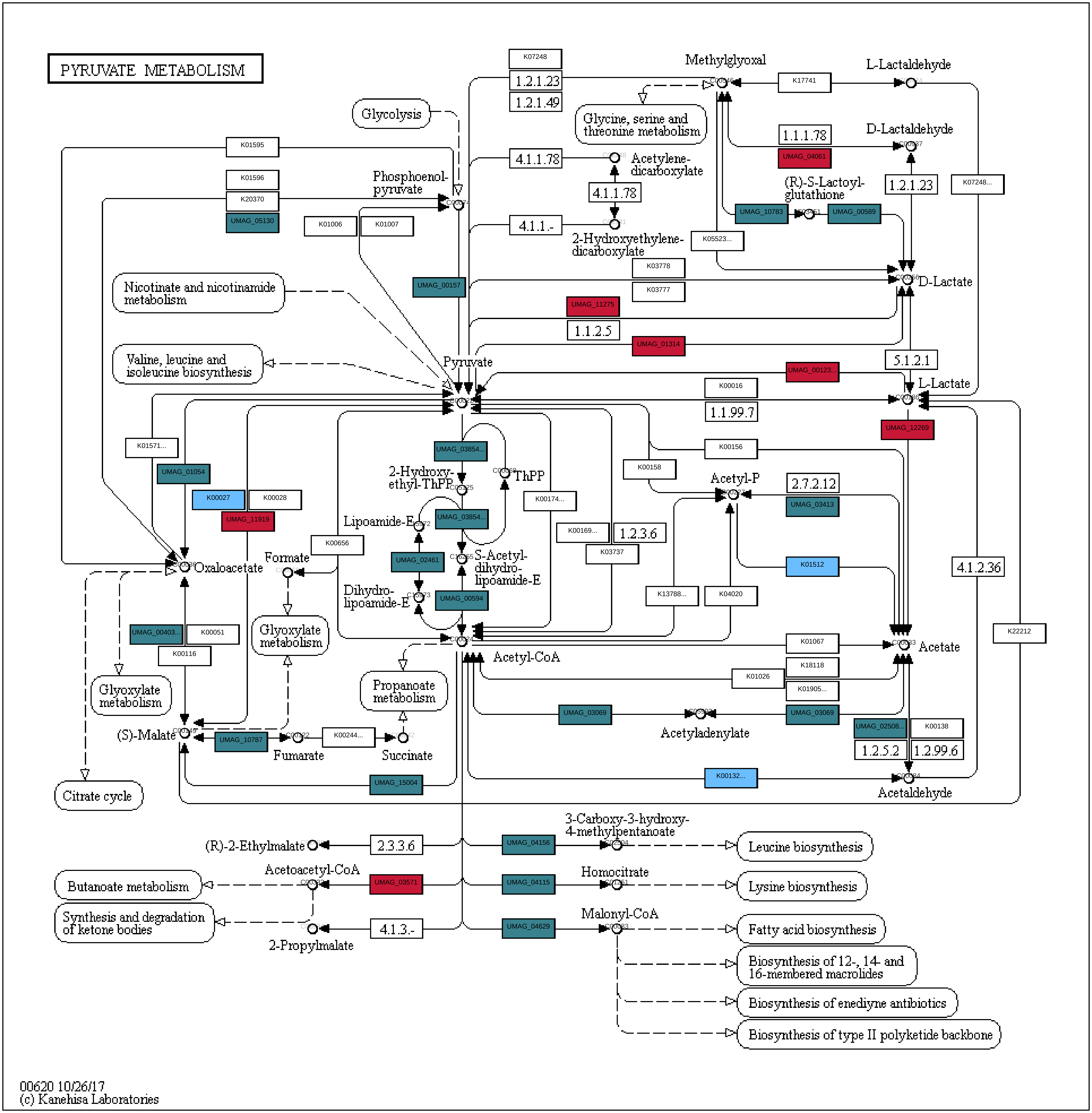
PYRUVATE METABOLISM
Glycolysis
Methylglyoxal
L-Lactaldehyde
K07248
1.2.1.23
1.2.1.49
K17741
Glycine, serine and threonine metabolism
D-Lactaldehyde
1.1.1.78
UMAG_04061
K01595
K01596
K20370
UMAG_05130
Phosphoenol-pyruvate
4.1.1.78
Acetylene-dicarboxylate
4.1.1.78
(R)-S-Lactoyl-glutathione
1.2.1.23
K07248...
UMAG_10783
UMAG_00589
K01006
K01007
4.1.1.-
2-Hydroxyethylene-dicarboxylate
K05523...
K03778
D-Lactate
K03777
UMAG_00157
Nicotinate and nicotinamide metabolism
UMAG_11275
1.1.2.5
UMAG_01314
Valine, leucine and isoleucine biosynthesis
Pyruvate
5.1.2.1
UMAG_00123...
L-Lactate
K00016
1.1.99.7
UMAG_12269
K01571...
UMAG_03854...
K00156
UMAG_01054
2-Hydroxy-ethyl-ThPP
ThPP
K00158
Acetyl-P
2.7.2.12
K00027
K00028
K00174...
UMAG_03413
UMAG_11919
Lipoamide-E
UMAG_03854...
K00169...
1.2.3.6
K00656
UMAG_02461
S-Acetyl-dihydro-lipoamide-E
K03737
Formate
Oxaloacetate
K01512
4.1.2.36
Dihydro-lipoamide-E
UMAG_00594
K13788...
K04020
Glyoxylate metabolism
UMAG_00403...
K00051
K00116
Acetyl-CoA
K01067
Acetate
K22212
K18118
K01026
K01905...
Propanoate metabolism
Glyoxylate metabolism
UMAG_03069
UMAG_03069
UMAG_02508...
K00138
Acetyladenylate
(S)-Malate
UMAG_10787
K00244...
1.2.5.2
1.2.99.6
Fumarate
Succinate
UMAG_15004
K00132...
Citrate cycle
Acetaldehyde
3-Carboxy-3-hydroxy-4-methylpentanoate
(R)-2-Ethylmalate
2.3.3.6
UMAG_04156
Leucine biosynthesis
Acetoacetyl-CoA
Homocitrate
Butanoate metabolism
UMAG_03571
UMAG_04115
Lysine biosynthesis
Synthesis and degradation of ketone bodies
4.1.3.-
UMAG_04629
Malonyl-CoA
Fatty acid biosynthesis
2-Propylmalate
Biosynthesis of 12-, 14- and 16-membered macrolides
Biosynthesis of enediyne antibiotics
Biosynthesis of type II polyketide backbone
00620 10/26/17
(c) Kanehisa Laboratories

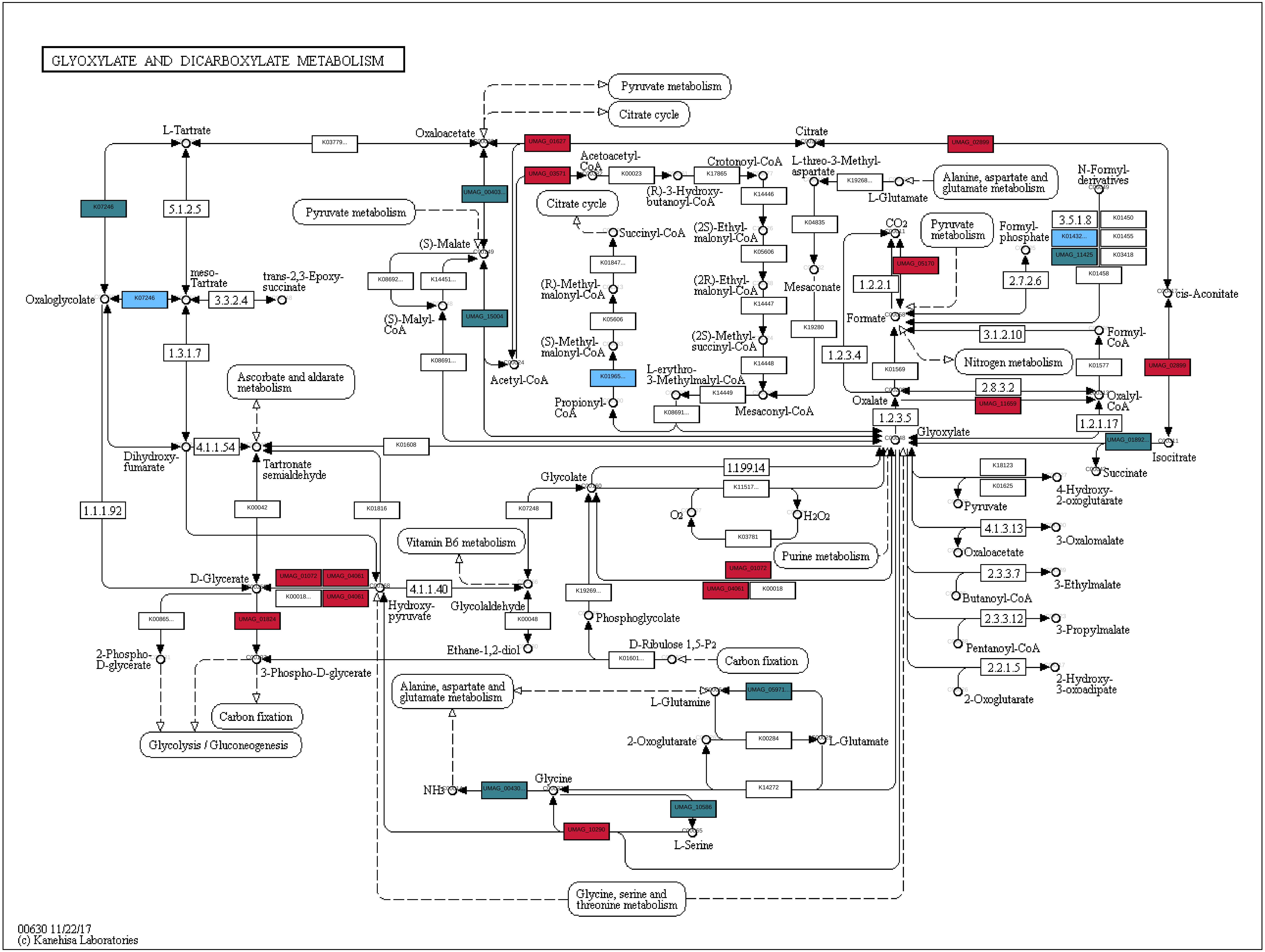
GLYOXYLATE AND DICARBOXYLATE METABOLISM
Pyruvate metabolism
Citrate cycle
L-Tartrate
K03779...
Oxaloacetate
UMAG_01627
Citrate
UMAG_02899
K07246
5.1.2.5
UMAG_03571
Acetoacetyl-CoA
K00023
Crotonoyl-CoA
K17865
L-threo-3-Methyl-aspartate
K19268...
Alanine, aspartate and glutamate metabolism
N-Formyl-derivatives
UMAG_00403...
(R)-3-Hydroxy-butanoyl-CoA
K14446
L-Glutamate
Pyruvate metabolism
Citrate cycle
3.5.1.8
K01450
K01432...
K01455
UMAG_11425
K03418
Succinyl-CoA
(2S)-Ethyl-malonyl-CoA
K04835
CO2
Pyruvate metabolism
Formyl-phosphate
(S)-Malate
K05606
UMAG_05170
K01458
K08692...
K14451...
K01847...
meso-Tartrate
trans-2,3-Epoxy-succinate
(2R)-Ethyl-malonyl-CoA
Mesaconate
1.2.2.1
2.7.2.6
cis-Aconitate
Oxaloglycolate
K07246
3.3.2.4
(R)-Methyl-malonyl-CoA
UMAG_15004
K14447
(S)-Malyl-CoA
K05606
K19280
Formate
3.1.2.10
Formyl-CoA
(S)-Methyl-malonyl-CoA
(2S)-Methyl-succinyl-CoA
1.3.1.7
K08691...
Acetyl-CoA
K14448
1.2.3.4
Nitrogen metabolism
UMAG_02899
K01569
K01577
Ascorbate and aldarate metabolism
K01965...
L-erythro-3-Methylmalyl-CoA
2.8.3.2
Oxalate
Oxalyl-CoA
Propionyl-CoA
K14449
UMAG_11659
K08691...
Mesaconyl-CoA
1.2.3.5
1.2.1.17
Glyoxylate
UMAG_01892...
Isocitrate
4.1.1.54
K01608
Dihydroxy-fumarate
Tartronate semialdehyde
1.1.99.14
K18123
Succinate
Glycolate
K11517...
4-Hydroxy-2-oxoglutarate
K01625
Pyruvate
1.1.1.92
K00042
K01816
K07248
O2
H2O2
4.1.3.13
3-Oxalomalate
K03781
Oxaloacetate
Vitamin B6 metabolism
Purine metabolism
UMAG_01072
2.3.3.7
3-Ethylmalate
D-Glycerate
UMAG_01072
UMAG_04061
4.1.1.40
K19269...
UMAG_04061
K00018
Butanoyl-CoA
K00018...
UMAG_04061
Hydroxy-pyruvate
Glycolaldehyde
2.3.3.12
K00865...
UMAG_01824
K00048
Phosphoglycolate
3-Propylmalate
Pentanoyl-CoA
2-Phospho-D-glycerate
Ethane-1,2-diol
D-Ribulose 1,5-P2
K01601...
Carbon fixation
2.2.1.5
3-Phospho-D-glycerate
2-Hydroxy-3-oxoadipate
Alanine, aspartate and glutamate metabolism
UMAG_05971...
L-Glutamine
2-Oxoglutarate
Carbon fixation
Glycolysis / Gluconeogenesis
2-Oxoglutarate
K00284
L-Glutamate
Glycine
K14272
NH3
UMAG_00430...
UMAG_10586
UMAG_10290
L-Serine
Glycine, serine and threonine metabolism
00630 11/22/17
(c) Kanehisa Laboratories

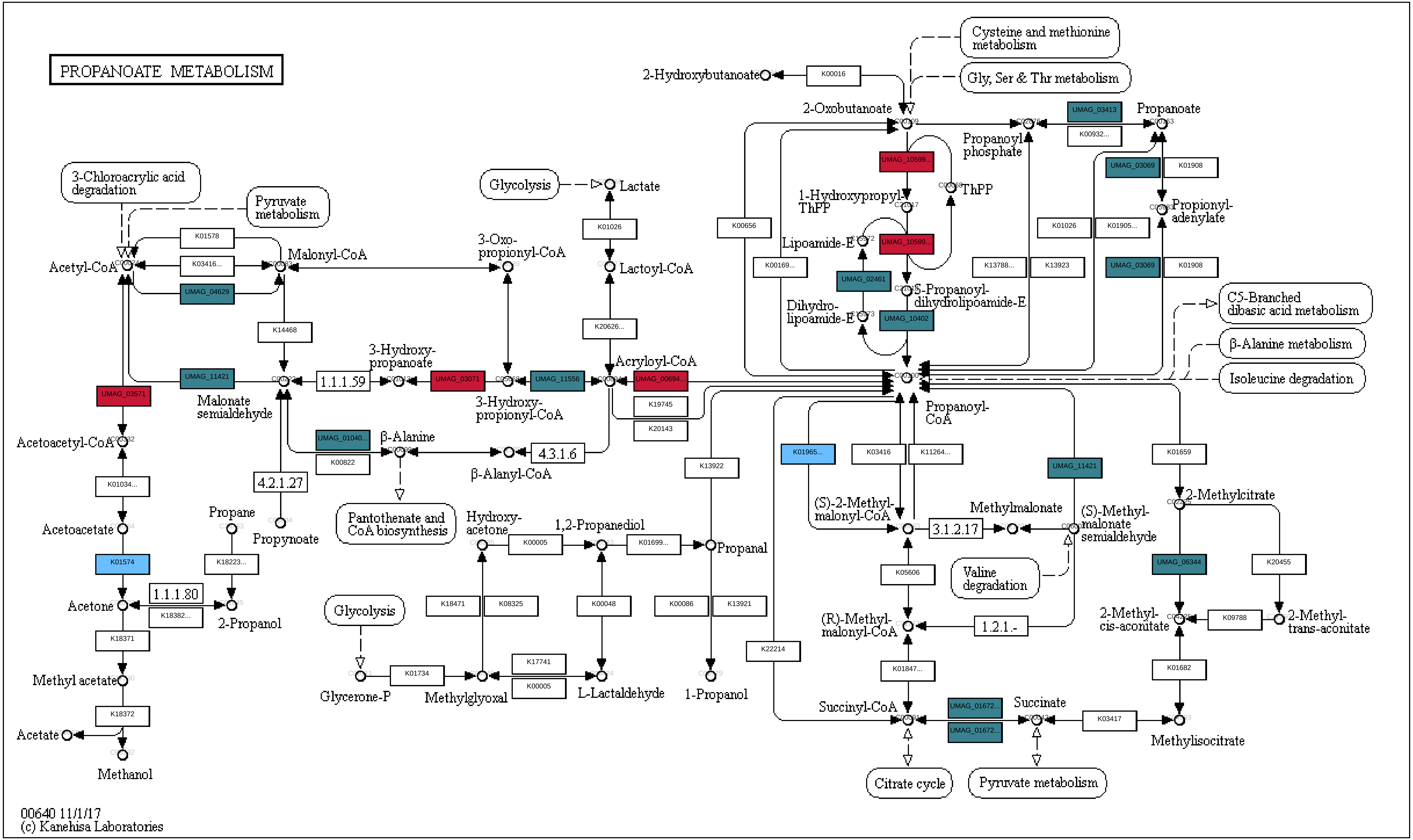
PROPANOATE METABOLISM
2-Hydroxybutanoate
K00016
Cysteine and methionine metabolism
Gly, Ser & Thr metabolism
2-Oxobutanoate
UMAG_03413
K00932...
Propanoate
Propanoyl phosphate
UMAG_10599...
1-Hydroxypropyl-ThPP
ThPP
UMAG_03069
K01908
Propionyl-adenylate
K00656
Lipoamide-E
UMAG_10599...
K01026
K01905...
K00169...
K13788...
K13923
UMAG_03069
K01908
UMAG_02461
S-Propanoyl-dihydrolipoamide-E
Dihydro-lipoamide-E
UMAG_10402
C5-Branched dibasic acid metabolism
β-Alanine metabolism
Isoleucine degradation
3-Chloroacrylic acid degradation
Pyruvate metabolism
Glycolysis
Lactate
K01026
K01578
K03416...
UMAG_04629
Acetyl-CoA
Malonyl-CoA
3-Oxo-propionyl-CoA
Lactoyl-CoA
K14468
K20626...
3-Hydroxy-propanoate
Acryloyl-CoA
UMAG_11421
1.1.1.59
UMAG_03071
UMAG_11556
UMAG_00694...
UMAG_03571
Malonate semialdehyde
3-Hydroxy-propionyl-CoA
K19745
K20143
Propanoyl-CoA
Acetoacetyl-CoA
UMAG_01040...
K00822
β-Alanine
4.3.1.6
β-Alanyl-CoA
K01965...
K03416
K11264...
UMAG_11421
K01659
K13922
K01034...
4.2.1.27
Pantothenate and CoA biosynthesis
(S)-2-Methyl-malonyl-CoA
Methylmalonate
3.1.2.17
(S)-Methyl-malonate semialdehyde
2-Methylcitrate
Acetoacetate
Propane
Propynoate
Hydroxy-acetone
1,2-Propanediol
K00005
K01699...
Propanal
K01574
K18223...
K05606
Valine degradation
UMAG_06344
K20455
1.1.1.80
Acetone
K18382...
2-Propanol
Glycolysis
K18471
K08325
K00048
K00086
K13921
(R)-Methyl-malonyl-CoA
1.2.1.-
2-Methyl-cis-aconitate
K09788
2-Methyl-trans-aconitate
K18371
K22214
K17741
K01734
K00005
K01847...
K01682
Methyl acetate
Glycerone-P
Methylglyoxal
L-Lactaldehyde
1-Propanol
Succinyl-CoA
UMAG_01672...
UMAG_01672...
Succinate
K03417
Methylisocitrate
K18372
Acetate
Methanol
Citrate cycle
Pyruvate metabolism
00640 11/1/17
(c) Kanehisa Laboratories

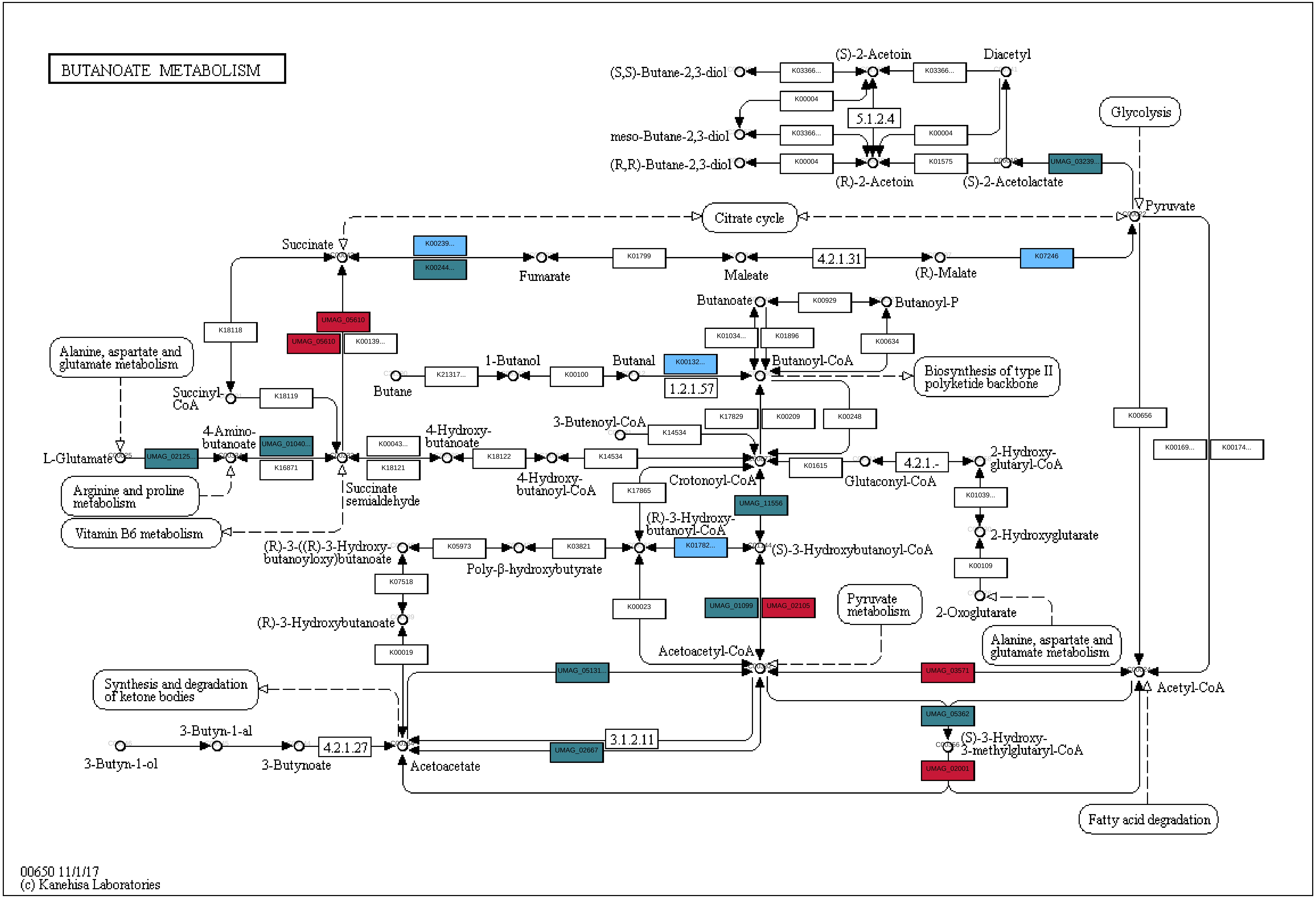
BUTANOATE METABOLISM
(S,S)-Butane-2,3-diol
K03366...
(S)-2-Acetoin
K03366...
Diacetyl
K00004
5.1.2.4
meso-Butane-2,3-diol
K03366...
K00004
(R,R)-Butane-2,3-diol
K00004
K01575
UMAG_03239...
(R)-2-Acetoin
(S)-2-Acetolactate
Glycolysis
Pyruvate
Citrate cycle
Succinate
K00239...
K00244...
Fumarate
K01799
Maleate
4.2.1.31
(R)-Malate
K07246
Butanoate
K00929
Butanoyl-P
K01034...
K01896
K00634
UMAG_05610
UMAG_05610
K00139...
K18118
Alanine, aspartate and glutamate metabolism
K21317...
1-Butanol
K00100
Butanal
K00132...
Butanoyl-CoA
Biosynthesis of type II polyketide backbone
Butane
1.2.1.57
Succinyl-CoA
K18119
K17829
K00209
K00248
K00656
3-Butenoyl-CoA
K14534
4-Amino-butanoate
UMAG_01040...
K00043...
4-Hydroxy-butanoate
L-Glutamate
UMAG_02125...
K16871
K18121
K18122
K14534
K01615
4.2.1.-
2-Hydroxy-glutaryl-CoA
K00169...
K00174...
Arginine and proline metabolism
Succinate semialdehyde
4-Hydroxy-butanoyl-CoA
Crotonoyl-CoA
Glutaconyl-CoA
K17865
UMAG_11556
K01039...
(R)-3-Hydroxy-butanoyl-CoA
2-Hydroxyglutarate
Vitamin B6 metabolism
(R)-3-((R)-3-Hydroxy-butanoyloxy)butanoate
K05973
K03821
K01782...
(S)-3-Hydroxybutanoyl-CoA
Poly-β-hydroxybutyrate
K00109
K07518
K00023
UMAG_01099
UMAG_02105
Pyruvate metabolism
2-Oxoglutarate
(R)-3-Hydroxybutanoate
Alanine, aspartate and glutamate metabolism
K00019
Acetoacetyl-CoA
UMAG_05131...
UMAG_03571
Acetyl-CoA
Synthesis and degradation of ketone bodies
UMAG_05362
3-Butyn-1-al
3.1.2.11
(S)-3-Hydroxy-3-methylglutaryl-CoA
3-Butyn-1-ol
3-Butynoate
4.2.1.27
UMAG_02667
Acetoacetate
UMAG_02001
Fatty acid degradation
00650 11/1/17
(c) Kanehisa Laboratories

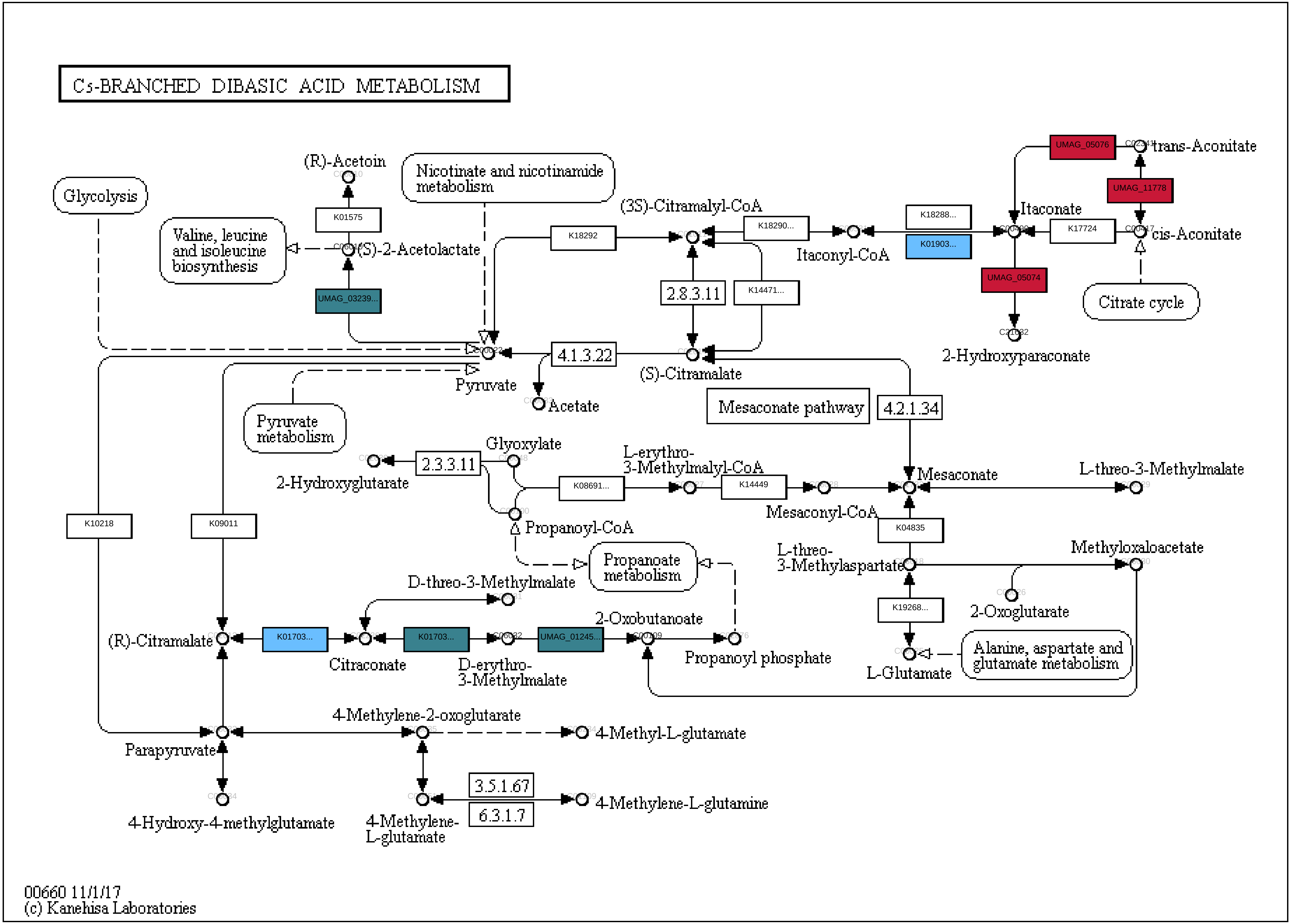
C5-BRANCHED DIBASIC ACID METABOLISM
Glycolysis
(R)-Acetoin
K01575
Nicotinate and nicotinamide metabolism
Valine, leucine and isoleucine biosynthesis
(S)-2-Acetolactate
UMAG_03239...
K18292
(3S)-Citramalyl-CoA
K18290...
Itaconyl-CoA
K18288...
K01903...
UMAG_05076
trans-Aconitate
UMAG_11778
Itaconate
K17724
cis-Aconitate
UMAG_05074
Citrate cycle
2-Hydroxyparaconate
2.8.3.11
K14471...
4.1.3.22
Pyruvate
Acetate
(S)-Citramalate
Mesaconate pathway
4.2.1.34
Pyruvate metabolism
Glyoxylate
2.3.3.11
2-Hydroxyglutarate
L-erythro-3-Methylmalyl-CoA
K08691...
K14449
Mesaconate
L-threo-3-Methylmalate
Mesaconyl-CoA
K04835
Propanoyl-CoA
K10218
K09011
Propanoate metabolism
L-threo-3-Methylaspartate
Methyloxaloacetate
D-threo-3-Methylmalate
2-Oxoglutarate
K19268...
2-Oxobutanoate
(R)-Citramalate
K01703...
Citraconate
K01703...
D-erythro-3-Methylmalate
UMAG_01245...
C00109
Propanoyl phosphate
L-Glutamate
Alanine, aspartate and glutamate metabolism
4-Methylene-2-oxoglutarate
4-Methyl-L-glutamate
Parapyruvate
3.5.1.67
6.3.1.7
4-Hydroxy-4-methylglutamate
4-Methylene-L-glutamate
4-Methylene-L-glutamine
00660 11/1/17
(c) Kanehisa Laboratories

# 2. Energy metabolism

| MAP | PATHWAY |
|---|---|
| **190** | Oxidative phosphorylation |
| **680** | Methane metabolism |
| **910** | Nitrogen metabolism |
| **920** | Sulfur metabolism |

# OXIDATIVE PHOSPHORYLATION

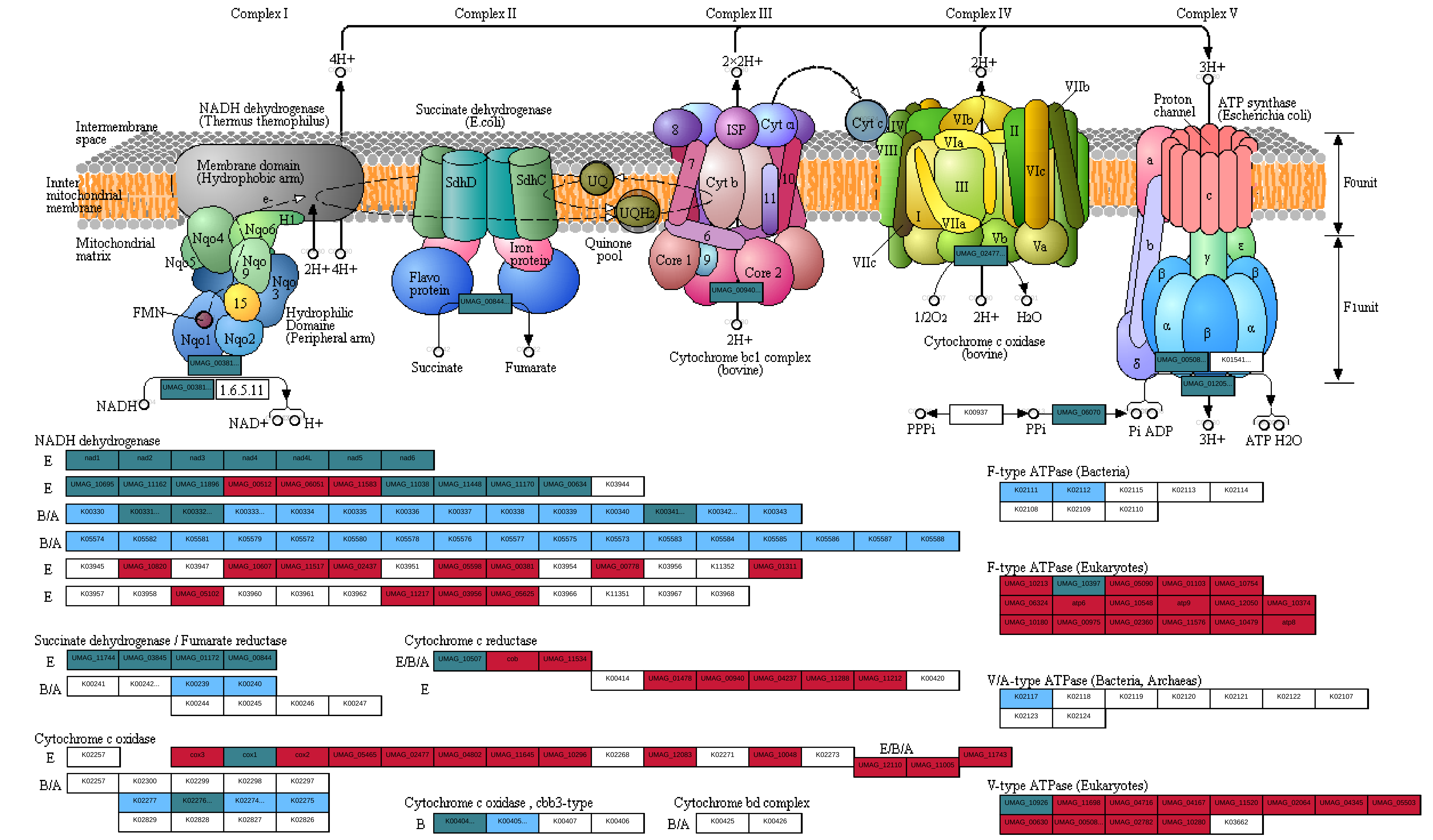


00190 6/16/15

(c) Kanehisa Laboratories

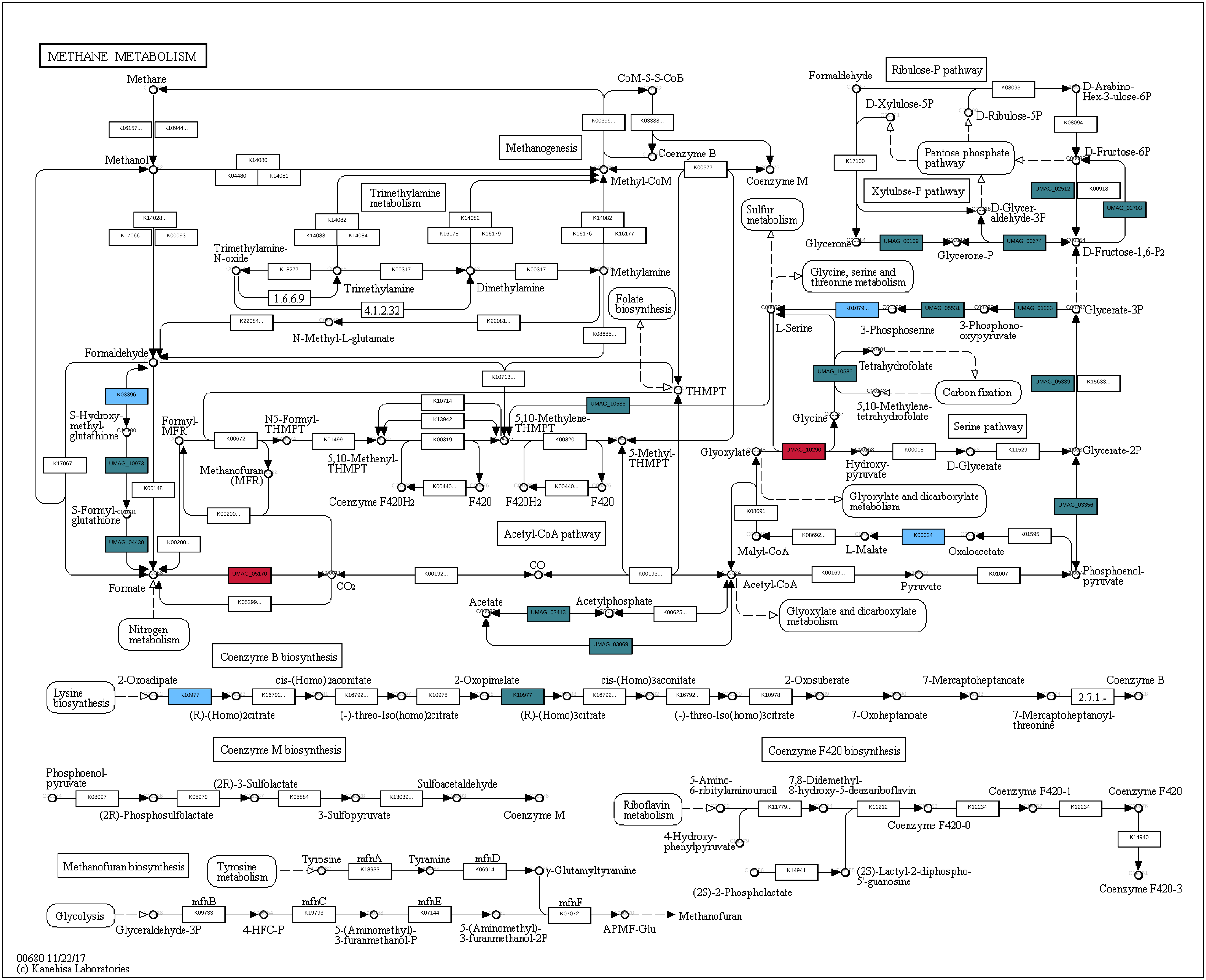
METHANE METABOLISM
Methane
CoM-S-S-CoB
K16157...
K10944...
K00399...
K03388...
Coenzyme B
Methanogenesis
Methanol
K14080
K04480
K14081
K00577...
Methyl-CoM
Coenzyme M
Trimethylamine metabolism
K14028...
K17066
K00093
K14082
K14083
K14084
K16178
K16179
K16176
K16177
Trimethylamine-N-oxide
K18277
K00317
Methylamine
Trimethylamine
Dimethylamine
1.6.6.9
4.1.2.32
Folate biosynthesis
K22084...
K22081...
N-Methyl-L-glutamate
K08685...
Formaldehyde
K10713...
THMPT
K03396
S-Hydroxy-methyl-glutathione
K10714
K13942
K00319
K00320
UMAG_10586
Formyl-MFR
N5-Formyl-THMPT
5,10-Methylene-THMPT
K00672
K01499
5,10-Methenyl-THMPT
5-Methyl-THMPT
K17067...
UMAG_10973
Methanofuran (MFR)
K00440...
Coenzyme F420H2
F420
F420H2
K00148
S-Formyl-glutathione
K00200...
Acetyl-CoA pathway
UMAG_04430
UMAG_05170
K00192...
CO
K00193...
Formate
CO2
K05299...
Acetate
Acetylphosphate
UMAG_03413
K00625...
UMAG_03069
Nitrogen metabolism
Formaldehyde
Ribulose-P pathway
K08093...
D-Arabino-Hex-3-ulose-6P
D-Xylulose-5P
D-Ribulose-5P
K08094...
K17100
Pentose phosphate pathway
D-Fructose-6P
Xylulose-P pathway
UMAG_02512
K00918
UMAG_02703
Sulfur metabolism
D-Glyceraldehyde-3P
Glycerone
UMAG_00109
Glycerone-P
UMAG_00674
D-Fructose-1,6-P2
Glycine, serine and threonine metabolism
K01079...
UMAG_05531
UMAG_01233
Glycerate-3P
L-Serine
3-Phosphoserine
3-Phosphono-oxypyruvate
Tetrahydrofolate
UMAG_10586
Carbon fixation
UMAG_05339
K15633...
5,10-Methylene-tetrahydrofolate
Glycine
Serine pathway
Glyoxylate
UMAG_10290
K00018
K11529
Glycerate-2P
Hydroxy-pyruvate
D-Glycerate
Glyoxylate and dicarboxylate metabolism
K08691
UMAG_03356
K08692...
K00024
K01595
Malyl-CoA
L-Malate
Oxaloacetate
K00169...
K01007
Acetyl-CoA
Pyruvate
Phosphoenol-pyruvate
Glyoxylate and dicarboxylate metabolism
Coenzyme B biosynthesis
Lysine biosynthesis
2-Oxoadipate
K10977
(R)-(Homo)2citrate
cis-(Homo)2aconitate
K16792...
(-)-threo-Iso(homo)2citrate
2-Oxopimelate
K10978
K10977
(R)-(Homo)3citrate
cis-(Homo)3aconitate
(-)-threo-Iso(homo)3citrate
2-Oxosuberate
7-Oxoheptanoate
7-Mercaptoheptanoate
7-Mercaptoheptanoyl-threonine
2.7.1.-
Coenzyme B
Coenzyme M biosynthesis
Coenzyme F420 biosynthesis
Phosphoenol-pyruvate
K08097
(2R)-Phosphosulfolactate
K05979
(2R)-3-Sulfolactate
K05884
3-Sulfopyruvate
K13039...
Sulfoacetaldehyde
Coenzyme M
Riboflavin metabolism
5-Amino-6-ribitylaminouracil
7,8-Didemethyl-8-hydroxy-5-deazariboflavin
K11779...
K11212
Coenzyme F420-0
K12234
Coenzyme F420-1
Coenzyme F420
4-Hydroxy-phenylpyruvate
K14940
K14941
(2S)-Lactyl-2-diphospho-5'-guanosine
(2S)-2-Phospholactate
Coenzyme F420-3
Methanofuran biosynthesis
Tyrosine metabolism
Tyrosine
mfnA
K18933
Tyramine
mfnD
K06914
γ-Glutamyltyramine
Glycolysis
Glyceraldehyde-3P
mfnB
K09733
4-HFC-P
mfnC
K19793
5-(Aminomethyl)-3-furanmethanol-P
mfnE
K07144
5-(Aminomethyl)-3-furanmethanol-2P
mfnF
K07072
APMF-Glu
Methanofuran
00680 11/22/17
(c) Kanehisa Laboratories

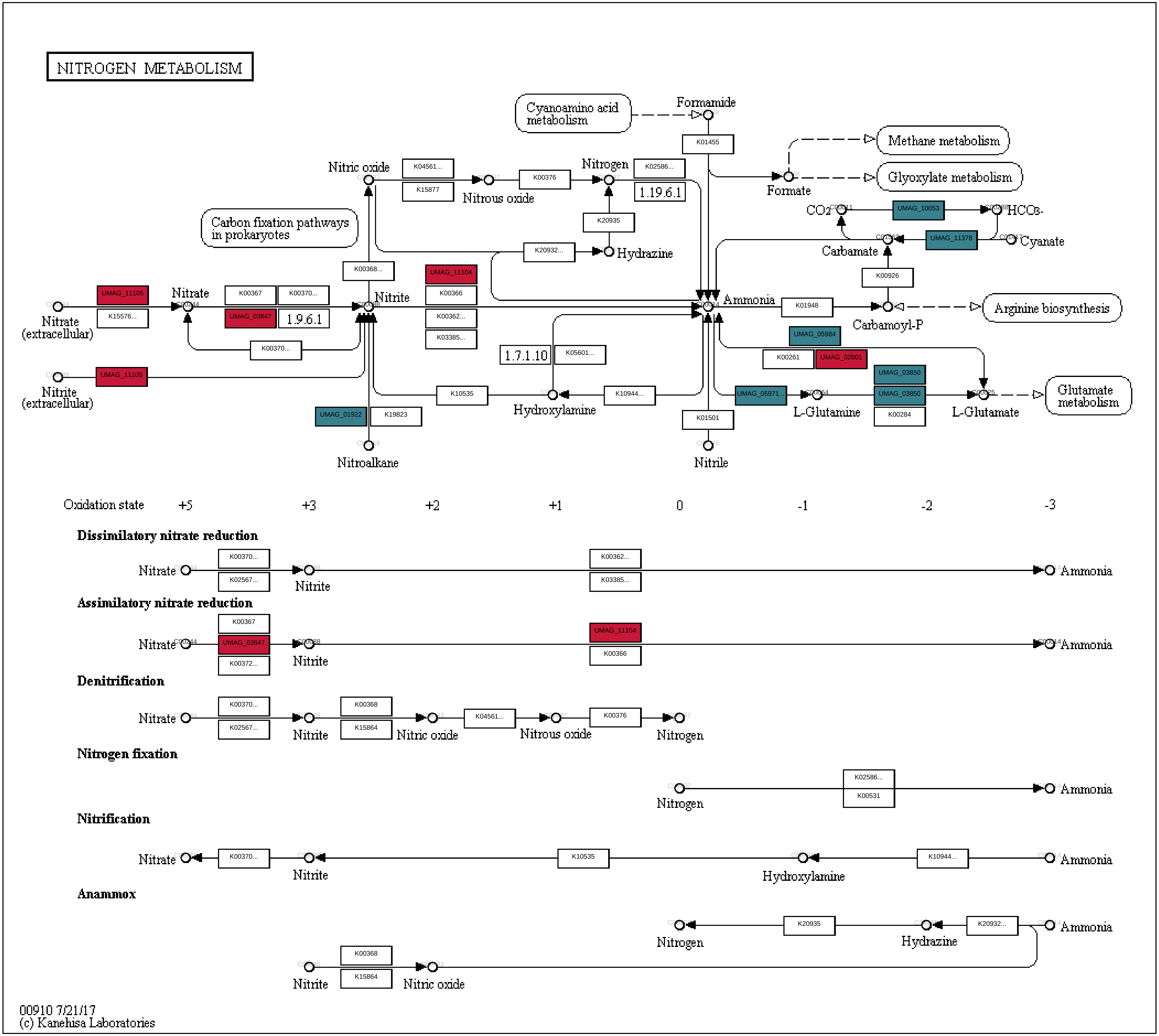
NITROGEN METABOLISM
Cyanoamino acid metabolism
Formamide
K01455
Methane metabolism
Glyoxylate metabolism
Formate
Nitric oxide
K04561...
K15877
Nitrous oxide
K00376
Nitrogen
K02586...
1.19.6.1
K20935
K20932...
Hydrazine
Carbon fixation pathways in prokaryotes
CO2
UMAG_10053
HCO3-
UMAG_11378
Cyanate
Carbamate
K00926
K00368...
UMAG_11104
K00366
K00362...
K03385...
UMAG_11105
K15576...
Nitrate
K00367
K00370...
UMAG_03847
1.9.6.1
Nitrite
Ammonia
K01948
Carbamoyl-P
Arginine biosynthesis
Nitrate (extracellular)
K00370...
1.7.1.10
K05601...
UMAG_05984
K00261
UMAG_02801
UMAG_03850
Nitrite (extracellular)
UMAG_11105
K10535
Hydroxylamine
K10944...
UMAG_05971...
L-Glutamine
UMAG_03850
K00284
L-Glutamate
Glutamate metabolism
UMAG_01922
K19823
K01501
Nitroalkane
Nitrile
Oxidation state
+5
+3
+2
+1
0
-1
-2
-3
Dissimilatory nitrate reduction
Nitrate
K00370...
K02567...
Nitrite
K00362...
K03385...
Ammonia
Assimilatory nitrate reduction
Nitrate
K00367
UMAG_03847
K00372...
Nitrite
UMAG_11104
K00366
Ammonia
Denitrification
Nitrate
K00370...
K02567...
Nitrite
K00368
K15864
Nitric oxide
K04561...
Nitrous oxide
K00376
Nitrogen
Nitrogen fixation
Nitrogen
K02586...
K00531
Ammonia
Nitrification
Nitrate
K00370...
Nitrite
K10535
Hydroxylamine
K10944...
Ammonia
Anammox
Nitrogen
K20935
Hydrazine
K20932...
Ammonia
Nitrite
K00368
K15864
Nitric oxide
00910 7/21/17
(c) Kanehisa Laboratories

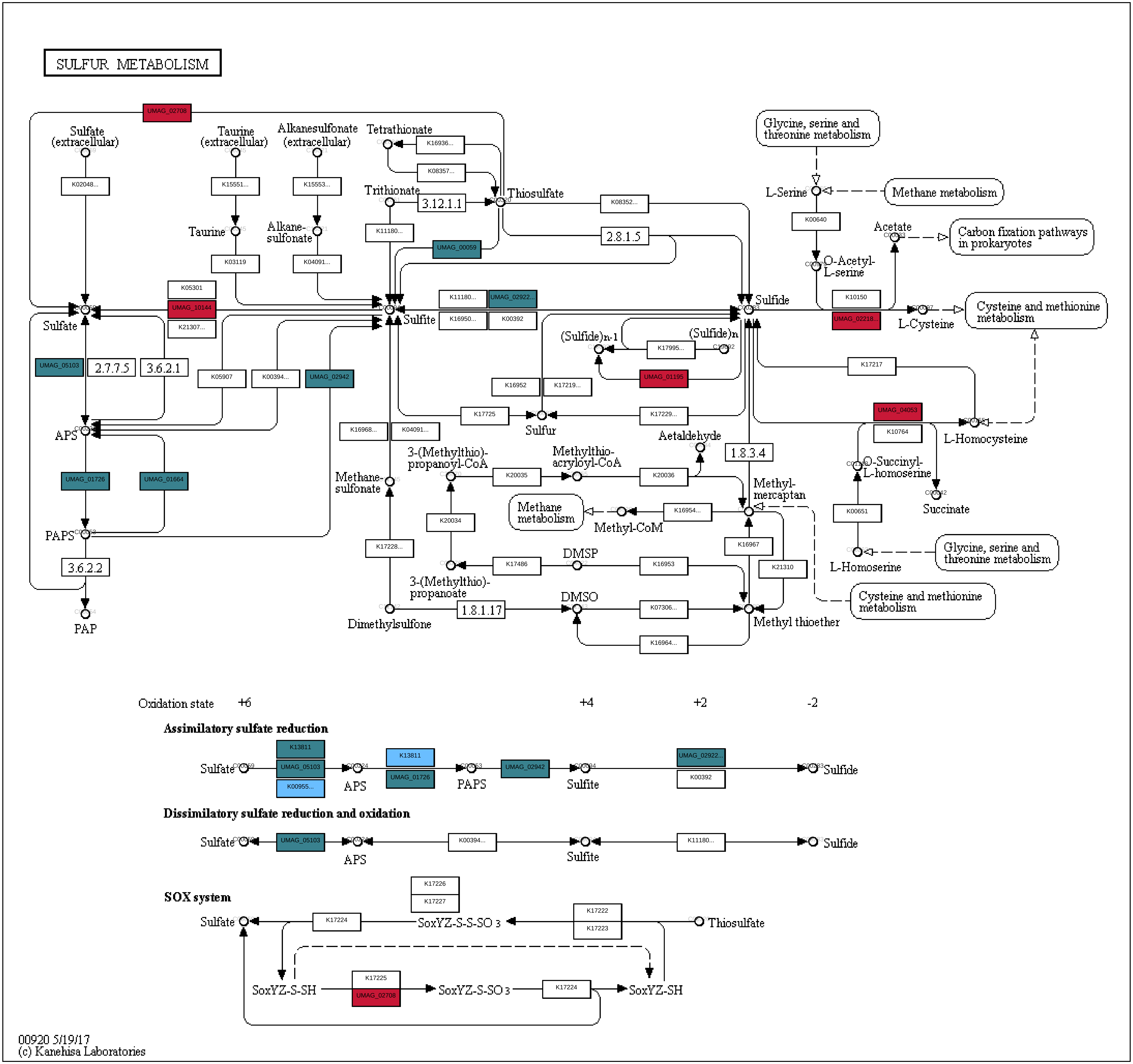
SULFUR METABOLISM
UMAG_02708
Sulfate (extracellular)
Taurine (extracellular)
Alkanesulfonate (extracellular)
Tetrathionate
K16936...
K08357...
K02048...
K15551...
K15553...
Trithionate
3.12.1.1
Thiosulfate
K08352...
Glycine, serine and threonine metabolism
L-Serine
Methane metabolism
K00640
Acetate
Carbon fixation pathways in prokaryotes
O-Acetyl-L-serine
Taurine
Alkane-sulfonate
K11180...
UMAG_00059
2.8.1.5
K03119
K04091...
K05301
UMAG_10144
K21307...
K11180...
UMAG_02922...
K16950...
K00392
Sulfite
Sulfide
K10150
UMAG_02218...
L-Cysteine
Cysteine and methionine metabolism
Sulfate
(Sulfide)n-1
(Sulfide)n
K17995...
UMAG_05103
2.7.7.5
3.6.2.1
K05907
K00394...
UMAG_02942
UMAG_01195
K17217
K16952
K17219...
UMAG_04053
K17725
Sulfur
K17229...
K10764
L-Homocysteine
APS
K16968...
K04091...
Aetaldehyde
1.8.3.4
3-(Methylthio)-propanoyl-CoA
Methylthio-acryloyl-CoA
O-Succinyl-L-homoserine
UMAG_01726
UMAG_01664
Methane-sulfonate
K20035
K20036
Methyl-mercaptan
Succinate
Methane metabolism
K16954...
Methyl-CoM
K00651
K20034
PAPS
K17228...
K16967
Glycine, serine and threonine metabolism
K17486
DMSP
K16953
K21310
L-Homoserine
3.6.2.2
3-(Methylthio)-propanoate
Cysteine and methionine metabolism
DMSO
1.8.1.17
K07306...
Dimethylsulfone
Methyl thioether
PAP
K16964...
Oxidation state +6 +4 +2 -2
Assimilatory sulfate reduction
K13811
UMAG_05103
K00955...
K13811
UMAG_01726
UMAG_02942
UMAG_02922...
K00392
Sulfate
APS
PAPS
Sulfite
Sulfide
Dissimilatory sulfate reduction and oxidation
UMAG_05103
K00394...
K11180...
Sulfate
APS
Sulfite
Sulfide
SOX system
K17226
K17227
K17224
K17222
K17223
Sulfate
SoxYZ-S-S-SO3
Thiosulfate
K17225
UMAG_02708
K17224
SoxYZ-S-SH
SoxYZ-S-S-SO3
SoxYZ-SH
00920 5/19/17
(c) Kanehisa Laboratories

# 3. Lipid metabolism

| MAP | PATHWAY |
|---|---|
| **61** | Fatty acid biosynthesis |
| **62** | Fatty acid elongation |
| **71** | Fatty acid degradation |
| **72** | Synthesis and degradation of ketone bodies |
| **100** | Steroid biosynthesis |
| **561** | Glycerolipid metabolism |
| **564** | Glycerophospholipid metabolism |
| **565** | Ether lipid metabolism |
| **590** | Arachidonic acid metabolism |
| **592** | alpha-Linolenic acid metabolism |
| **600** | Sphingolipid metabolism |

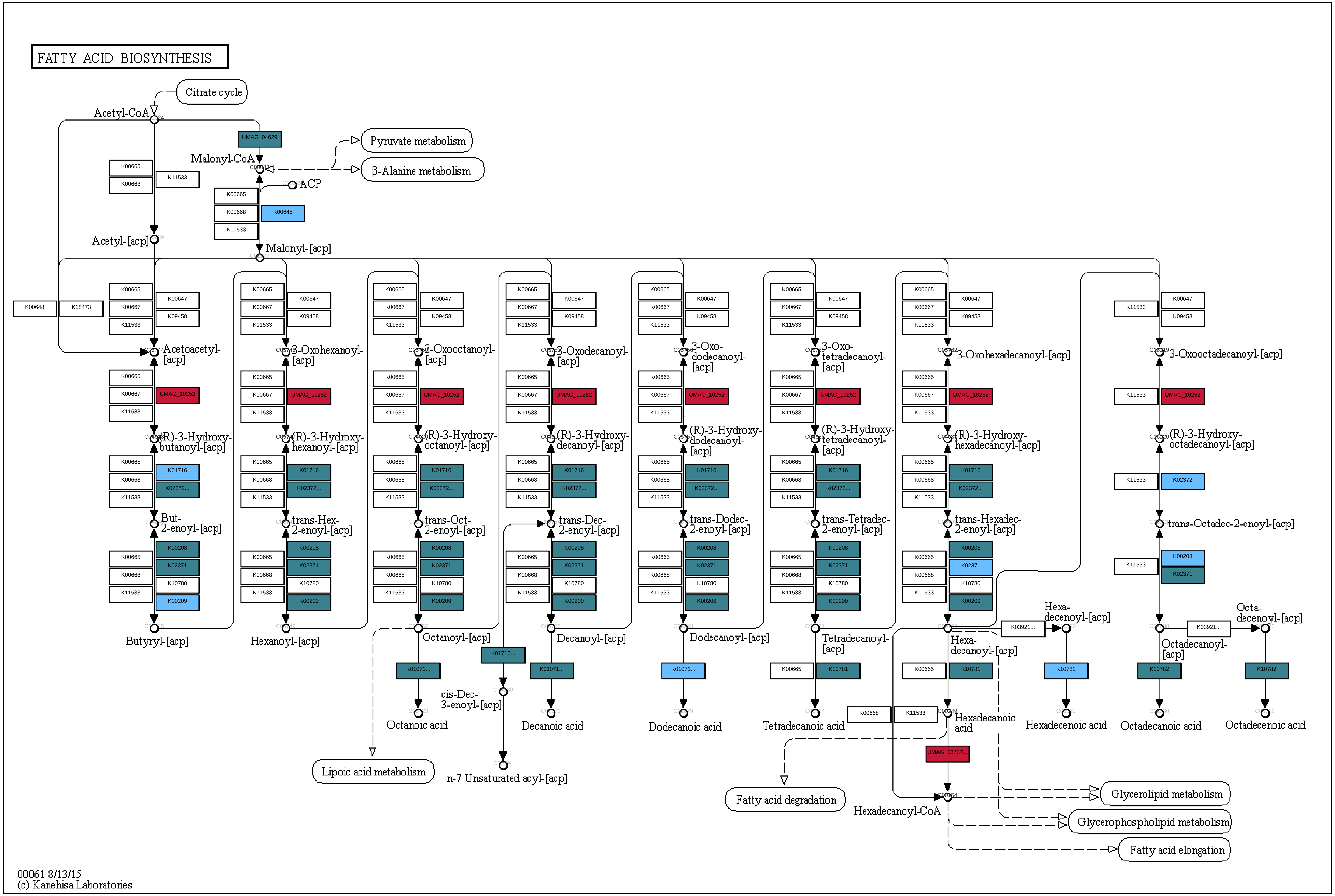
FATTY ACID BIOSYNTHESIS
Citrate cycle
Acetyl-CoA
Malonyl-CoA
UMAG_04629
Pyruvate metabolism
β-Alanine metabolism
ACP
K00665
K00668
K11533
K00645
Acetyl-[acp]
Malonyl-[acp]
K00648
K18473
K00667
K00647
K09458
Acetoacetyl-[acp]
3-Oxohexanoyl-[acp]
3-Oxooctanoyl-[acp]
3-Oxodecanoyl-[acp]
3-Oxo-dodecanoyl-[acp]
3-Oxo-tetradecanoyl-[acp]
3-Oxohexadecanoyl-[acp]
3-Oxooctadecanoyl-[acp]
UMAG_10252
(R)-3-Hydroxy-butanoyl-[acp]
(R)-3-Hydroxy-hexanoyl-[acp]
(R)-3-Hydroxy-octanoyl-[acp]
(R)-3-Hydroxy-decanoyl-[acp]
(R)-3-Hydroxy-dodecanoyl-[acp]
(R)-3-Hydroxy-tetradecanoyl-[acp]
(R)-3-Hydroxy-hexadecanoyl-[acp]
(R)-3-Hydroxy-octadecanoyl-[acp]
K01716
K02372...
K02372
But-2-enoyl-[acp]
trans-Hex-2-enoyl-[acp]
trans-Oct-2-enoyl-[acp]
trans-Dec-2-enoyl-[acp]
trans-Dodec-2-enoyl-[acp]
trans-Tetradec-2-enoyl-[acp]
trans-Hexadec-2-enoyl-[acp]
trans-Octadec-2-enoyl-[acp]
K00208
K02371
K10780
K00209
Butyryl-[acp]
Hexanoyl-[acp]
Octanoyl-[acp]
Decanoyl-[acp]
Dodecanoyl-[acp]
Tetradecanoyl-[acp]
Hexa-decanoyl-[acp]
Hexa-decenoyl-[acp]
Octadecanoyl-[acp]
Octa-decenoyl-[acp]
K01071...
K01716...
K10781
K10782
K03921...
cis-Dec-3-enoyl-[acp]
Octanoic acid
Decanoic acid
Dodecanoic acid
Tetradecanoic acid
Hexadecanoic acid
Hexadecenoic acid
Octadecanoic acid
Octadecenoic acid
n-7 Unsaturated acyl-[acp]
Lipoic acid metabolism
Fatty acid degradation
UMAG_03737...
Hexadecanoyl-CoA
Glycerolipid metabolism
Glycerophospholipid metabolism
Fatty acid elongation
00061 8/13/15
(c) Kanehisa Laboratories

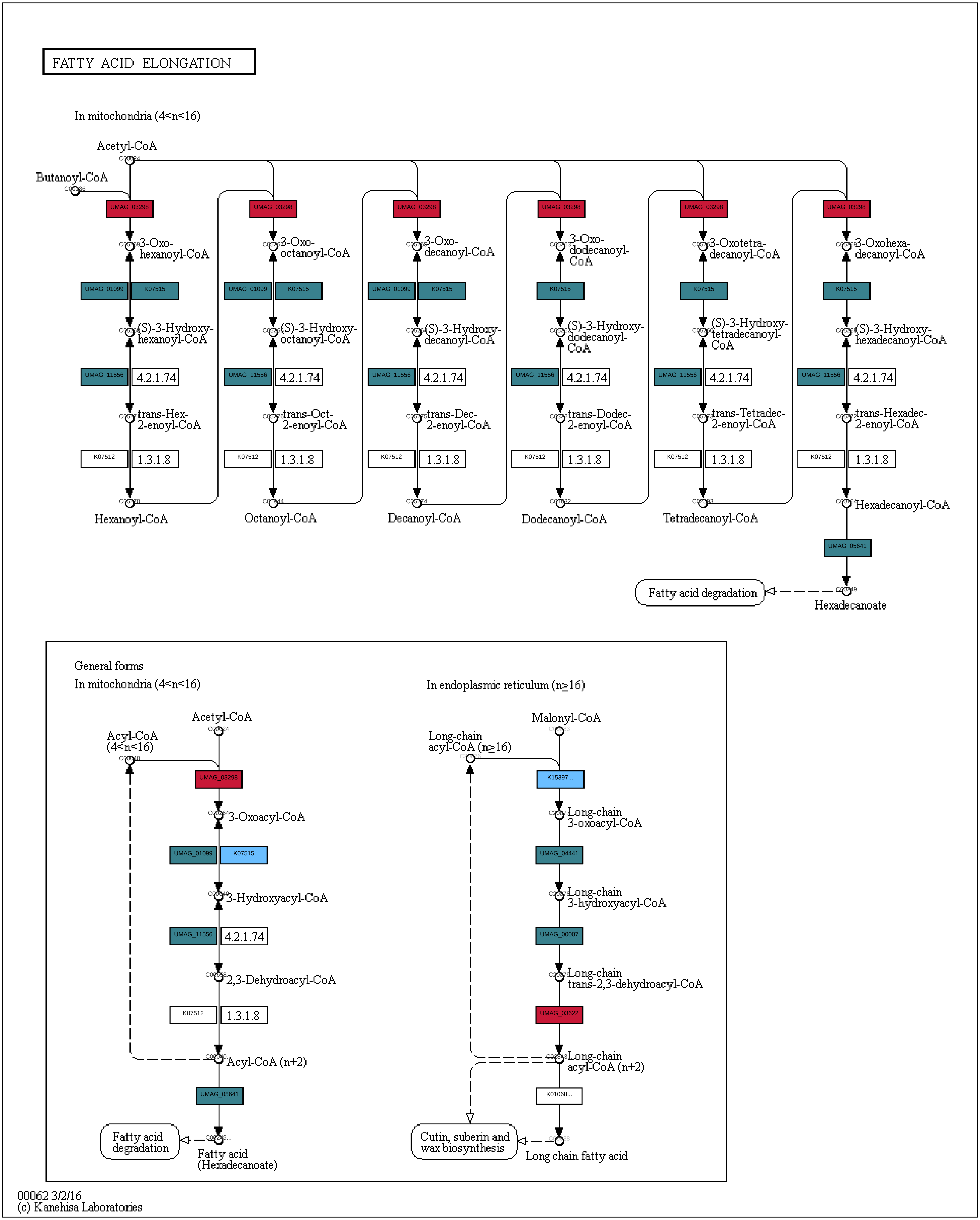
FATTY ACID ELONGATION
In mitochondria (4<n<16)
Acetyl-CoA
Butanoyl-CoA
UMAG_03298
3-Oxo-hexanoyl-CoA
3-Oxo-octanoyl-CoA
3-Oxo-decanoyl-CoA
3-Oxo-dodecanoyl-CoA
3-Oxotetra-decanoyl-CoA
3-Oxohexa-decanoyl-CoA
UMAG_01099
K07515
(S)-3-Hydroxy-hexanoyl-CoA
(S)-3-Hydroxy-octanoyl-CoA
(S)-3-Hydroxy-decanoyl-CoA
(S)-3-Hydroxy-dodecanoyl-CoA
(S)-3-Hydroxy-tetradecanoyl-CoA
(S)-3-Hydroxy-hexadecanoyl-CoA
UMAG_11556
4.2.1.74
trans-Hex-2-enoyl-CoA
trans-Oct-2-enoyl-CoA
trans-Dec-2-enoyl-CoA
trans-Dodec-2-enoyl-CoA
trans-Tetradec-2-enoyl-CoA
trans-Hexadec-2-enoyl-CoA
K07512
1.3.1.8
Hexanoyl-CoA
Octanoyl-CoA
Decanoyl-CoA
Dodecanoyl-CoA
Tetradecanoyl-CoA
Hexadecanoyl-CoA
UMAG_05641
Fatty acid degradation
Hexadecanoate
General forms
In mitochondria (4<n<16)
Acetyl-CoA
Acyl-CoA (4<n<16)
UMAG_03298
3-Oxoacyl-CoA
UMAG_01099
K07515
3-Hydroxyacyl-CoA
UMAG_11556
4.2.1.74
2,3-Dehydroacyl-CoA
K07512
1.3.1.8
Acyl-CoA (n+2)
UMAG_05641
Fatty acid degradation
Fatty acid (Hexadecanoate)
In endoplasmic reticulum (n≥16)
Malonyl-CoA
Long-chain acyl-CoA (n≥16)
K15397...
Long-chain 3-oxoacyl-CoA
UMAG_04441
Long-chain 3-hydroxyacyl-CoA
UMAG_00007
Long-chain trans-2,3-dehydroacyl-CoA
UMAG_03622
Long-chain acyl-CoA (n+2)
K01068...
Cutin, suberin and wax biosynthesis
Long chain fatty acid
00062 3/2/16
(c) Kanehisa Laboratories

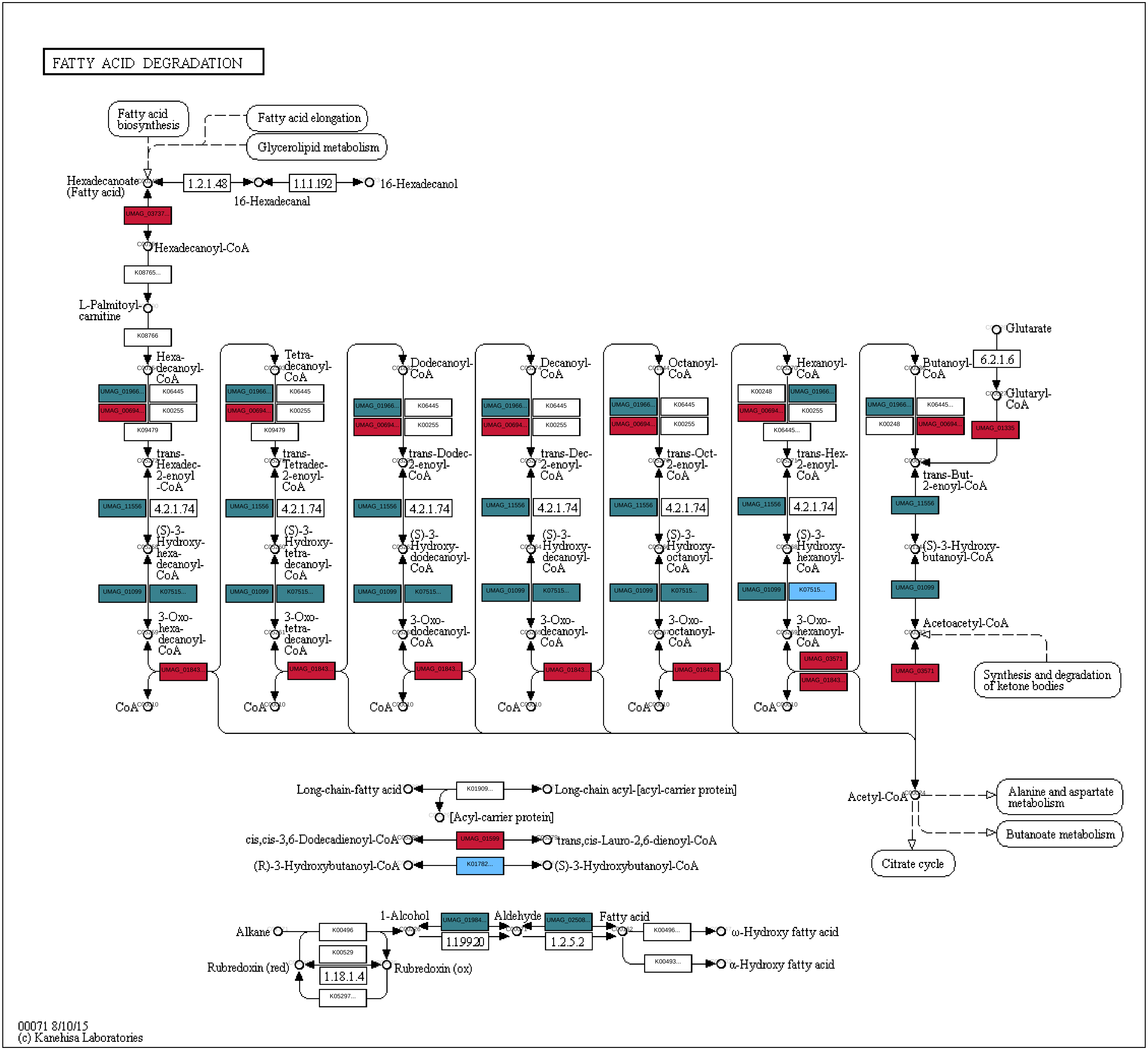

FATTY ACID DEGRADATION
Fatty acid biosynthesis
Fatty acid elongation
Glycerolipid metabolism
Hexadecanoate (Fatty acid)
1.2.1.48
16-Hexadecanal
1.1.1.192
16-Hexadecanol
UMAG_03737...
Hexadecanoyl-CoA
K08765...
L-Palmitoyl-carnitine
K08766
Hexa-decanoyl-CoA
UMAG_01966...
K06445
UMAG_00694...
K00255
K09479
trans-Hexadec-2-enoyl-CoA
UMAG_11556
4.2.1.74
(S)-3-Hydroxy-hexa-decanoyl-CoA
UMAG_01099
K07515...
3-Oxo-hexa-decanoyl-CoA
UMAG_01843...
CoA
Tetra-decanoyl-CoA
trans-Tetradec-2-enoyl-CoA
(S)-3-Hydroxy-tetra-decanoyl-CoA
3-Oxo-tetra-decanoyl-CoA
Dodecanoyl-CoA
trans-Dodec-2-enoyl-CoA
(S)-3-Hydroxy-dodecanoyl-CoA
3-Oxo-dodecanoyl-CoA
Decanoyl-CoA
trans-Dec-2-enoyl-CoA
(S)-3-Hydroxy-decanoyl-CoA
3-Oxo-decanoyl-CoA
Octanoyl-CoA
trans-Oct-2-enoyl-CoA
(S)-3-Hydroxy-octanoyl-CoA
3-Oxo-octanoyl-CoA
Hexanoyl-CoA
K00248
K06445...
trans-Hex-2-enoyl-CoA
(S)-3-Hydroxy-hexanoyl-CoA
3-Oxo-hexanoyl-CoA
UMAG_03571
Glutarate
Butanoyl-CoA
6.2.1.6
Glutaryl-CoA
UMAG_01335
trans-But-2-enoyl-CoA
(S)-3-Hydroxy-butanoyl-CoA
Acetoacetyl-CoA
Synthesis and degradation of ketone bodies
Acetyl-CoA
Alanine and aspartate metabolism
Butanoate metabolism
Citrate cycle
Long-chain-fatty acid
K01909...
Long-chain acyl-[acyl-carrier protein]
[Acyl-carrier protein]
cis,cis-3,6-Dodecadienoyl-CoA
UMAG_01599
trans,cis-Lauro-2,6-dienoyl-CoA
(R)-3-Hydroxybutanoyl-CoA
K01782...
(S)-3-Hydroxybutanoyl-CoA
1-Alcohol
UMAG_01984...
Aldehyde
UMAG_02508...
Fatty acid
Alkane
K00496
1.1.99.20
1.2.5.2
K00496...
ω-Hydroxy fatty acid
K00529
Rubredoxin (red)
1.18.1.4
Rubredoxin (ox)
K00493...
α-Hydroxy fatty acid
K05297...
00071 8/10/15
(c) Kanehisa Laboratories

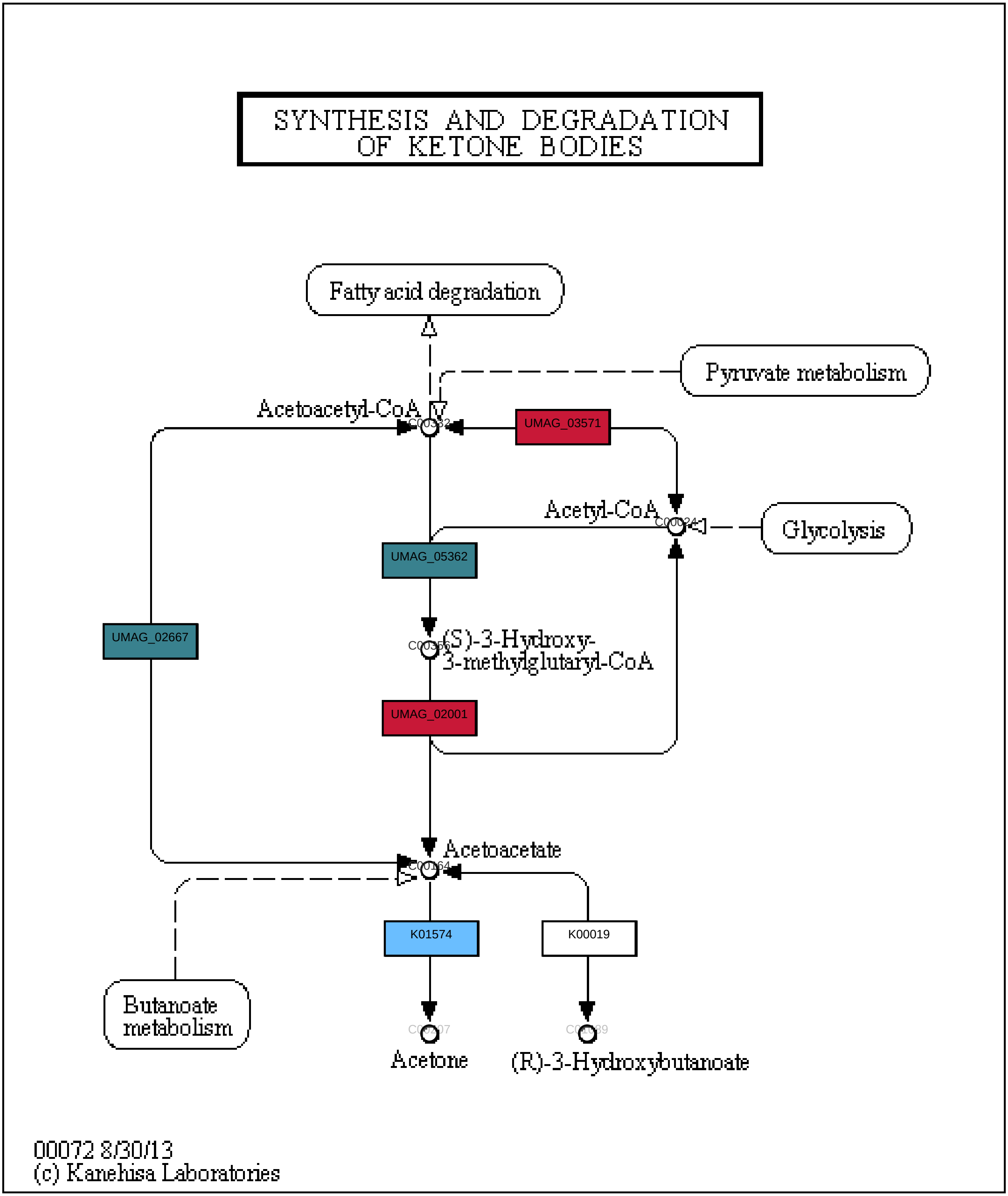
SYNTHESIS AND DEGRADATION OF KETONE BODIES
Fatty acid degradation
Pyruvate metabolism
Acetoacetyl-CoA
C00332
UMAG_03571
Acetyl-CoA
C00024
Glycolysis
UMAG_05362
UMAG_02667
C00356
(S)-3-Hydroxy-3-methylglutaryl-CoA
UMAG_02001
Acetoacetate
C00164
K01574
K00019
Butanoate metabolism
Acetone
(R)-3-Hydroxybutanoate
00072 8/30/13
(c) Kanehisa Laboratories

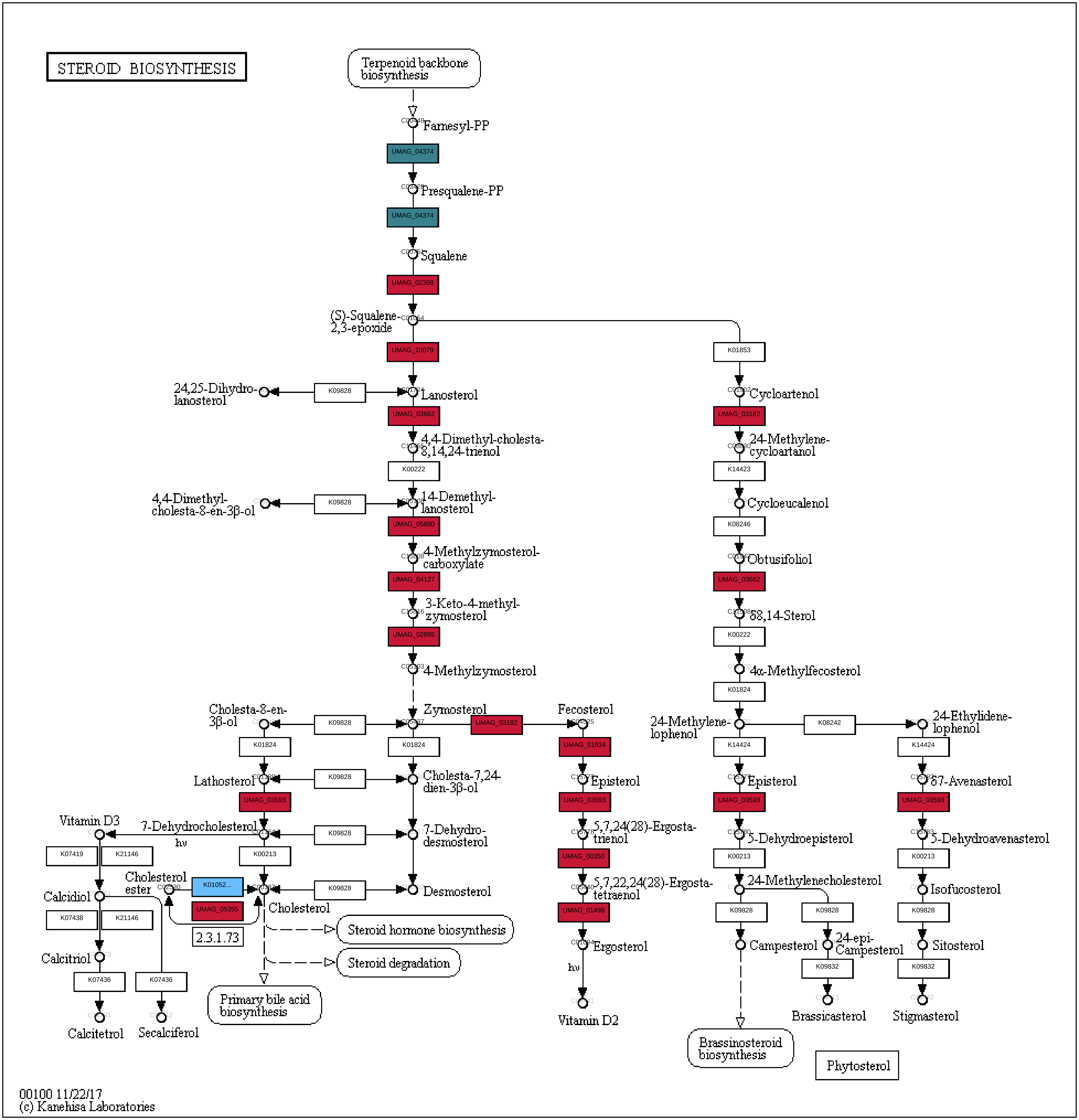
STEROID BIOSYNTHESIS
Terpenoid backbone biosynthesis
Farnesyl-PP
UMAG_04374
Presqualene-PP
UMAG_04374
Squalene
UMAG_02398
(S)-Squalene-2,3-epoxide
UMAG_10079
K01853
24,25-Dihydro-lanosterol
K09828
Lanosterol
Cycloartenol
UMAG_03662
UMAG_03182
4,4-Dimethyl-cholesta-8,14,24-trienol
24-Methylene-cycloartanol
K00222
K14423
4,4-Dimethyl-cholesta-8-en-3β-ol
K09828
14-Demethyl-lanosterol
Cycloeucalenol
UMAG_05880
K08246
4-Methylzymosterol-carboxylate
Obtusifoliol
UMAG_04127
UMAG_03662
3-Keto-4-methyl-zymosterol
δ8,14-Sterol
UMAG_02895
K00222
4-Methylzymosterol
4α-Methylfecosterol
K01824
Cholesta-8-en-3β-ol
K09828
Zymosterol
UMAG_03182
Fecosterol
24-Methylene-lophenol
K08242
24-Ethylidene-lophenol
K01824
K01824
UMAG_01934
K14424
K14424
Lathosterol
K09828
Cholesta-7,24-dien-3β-ol
Episterol
Episterol
δ7-Avenasterol
UMAG_03593
UMAG_03593
UMAG_03593
UMAG_03593
Vitamin D3
7-Dehydrocholesterol
K09828
7-Dehydro-desmosterol
5,7,24(28)-Ergosta-trienol
5-Dehydroepisterol
5-Dehydroavenasterol
hν
K07419
K21146
K00213
UMAG_00350
K00213
K00213
Cholesterol ester
K01052...
K09828
Desmosterol
5,7,22,24(28)-Ergosta-tetraenol
24-Methylenecholesterol
Isofucosterol
Calcidiol
UMAG_05355
Cholesterol
UMAG_01498
K09828
K09828
K09828
K07438
K21146
2.3.1.73
Steroid hormone biosynthesis
24-epi-Campesterol
Campesterol
Sitosterol
Calcitriol
Steroid degradation
Ergosterol
K09832
K09832
K07436
K07436
hν
Primary bile acid biosynthesis
Brassicasterol
Stigmasterol
Calcitetrol
Secalciferol
Vitamin D2
Brassinosteroid biosynthesis
Phytosterol
00100 11/22/17
(c) Kanehisa Laboratories

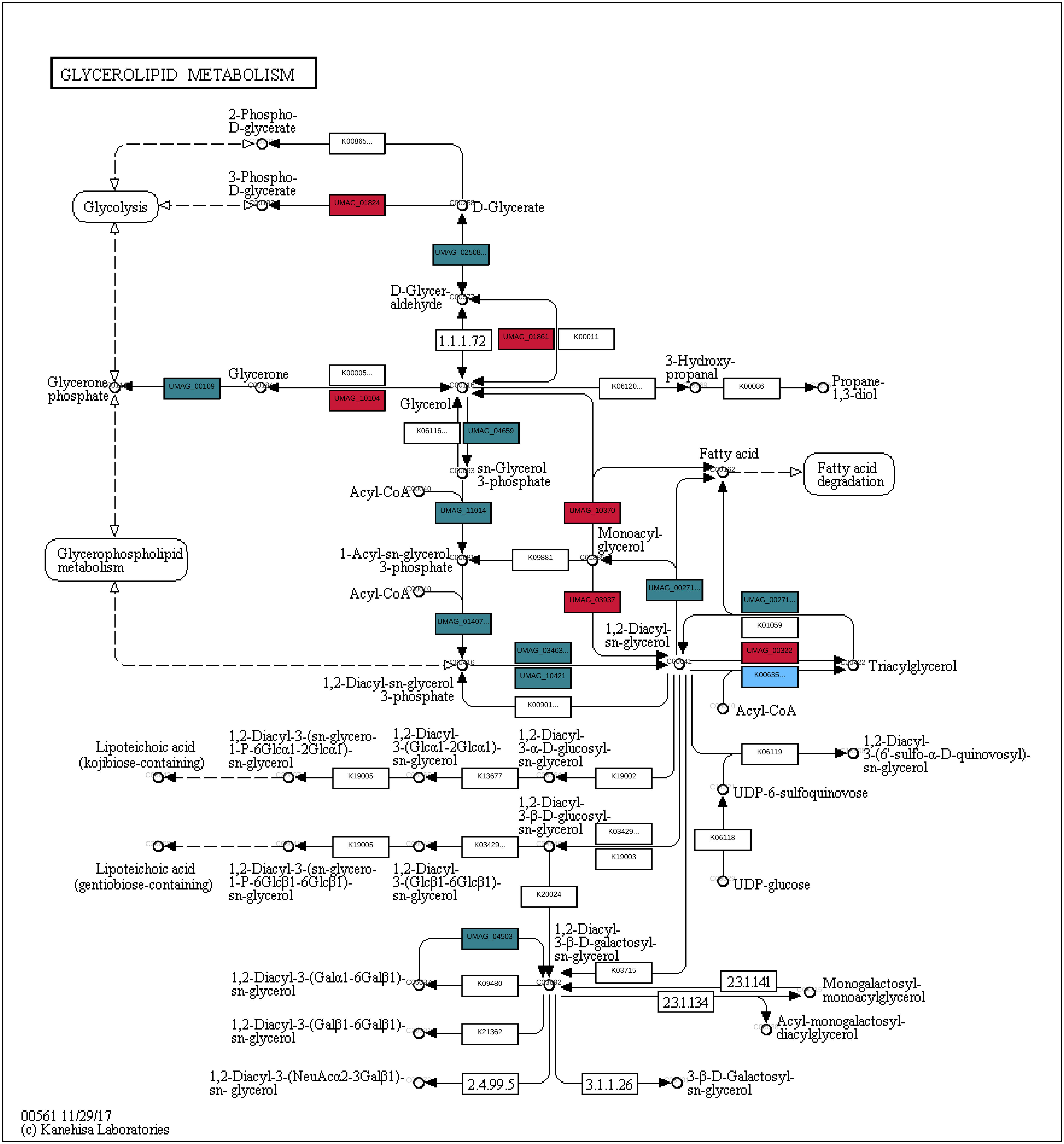
GLYCEROLIPID METABOLISM
2-Phospho-D-glycerate
K00865...
3-Phospho-D-glycerate
UMAG_01824
D-Glycerate
Glycolysis
UMAG_02508...
D-Glyceraldehyde
1.1.1.72
UMAG_01861
K00011
Glycerone phosphate
UMAG_00109
Glycerone
K00005...
UMAG_10104
Glycerol
K06120...
3-Hydroxypropanal
K00086
Propane-1,3-diol
K06116...
UMAG_04659
sn-Glycerol 3-phosphate
Acyl-CoA
UMAG_11014
Fatty acid
Fatty acid degradation
UMAG_10370
Monoacylglycerol
Glycerophospholipid metabolism
1-Acyl-sn-glycerol 3-phosphate
K09881
UMAG_00271...
UMAG_03937
UMAG_00271...
Acyl-CoA
UMAG_01407...
K01059
1,2-Diacyl-sn-glycerol
UMAG_03463...
UMAG_00322
UMAG_10421
K00635...
Triacylglycerol
1,2-Diacyl-sn-glycerol 3-phosphate
K00901...
Acyl-CoA
Lipoteichoic acid (kojibiose-containing)
1,2-Diacyl-3-(sn-glycero-1-P-6Glcα1-2Glcα1)-sn-glycerol
1,2-Diacyl-3-(Glcα1-2Glcα1)-sn-glycerol
1,2-Diacyl-3-α-D-glucosyl-sn-glycerol
K19005
K13677
K19002
K06119
1,2-Diacyl-3-(6'-sulfo-α-D-quinovosyl)-sn-glycerol
UDP-6-sulfoquinovose
1,2-Diacyl-3-β-D-glucosyl-sn-glycerol
K03429...
K19005
K03429...
K19003
K06118
Lipoteichoic acid (gentiobiose-containing)
1,2-Diacyl-3-(sn-glycero-1-P-6Glcβ1-6Glcβ1)-sn-glycerol
1,2-Diacyl-3-(Glcβ1-6Glcβ1)-sn-glycerol
K20024
UDP-glucose
UMAG_04503
1,2-Diacyl-3-β-D-galactosyl-sn-glycerol
1,2-Diacyl-3-(Galα1-6Galβ1)-sn-glycerol
K09480
K03715
2.3.1.141
Monogalactosylmonoacylglycerol
2.3.1.134
Acyl-monogalactosyldiacylglycerol
1,2-Diacyl-3-(Galβ1-6Galβ1)-sn-glycerol
K21362
1,2-Diacyl-3-(NeuAcα2-3Galβ1)-sn- glycerol
2.4.99.5
3.1.1.26
3-β-D-Galactosyl-sn-glycerol
00561 11/29/17
(c) Kanehisa Laboratories

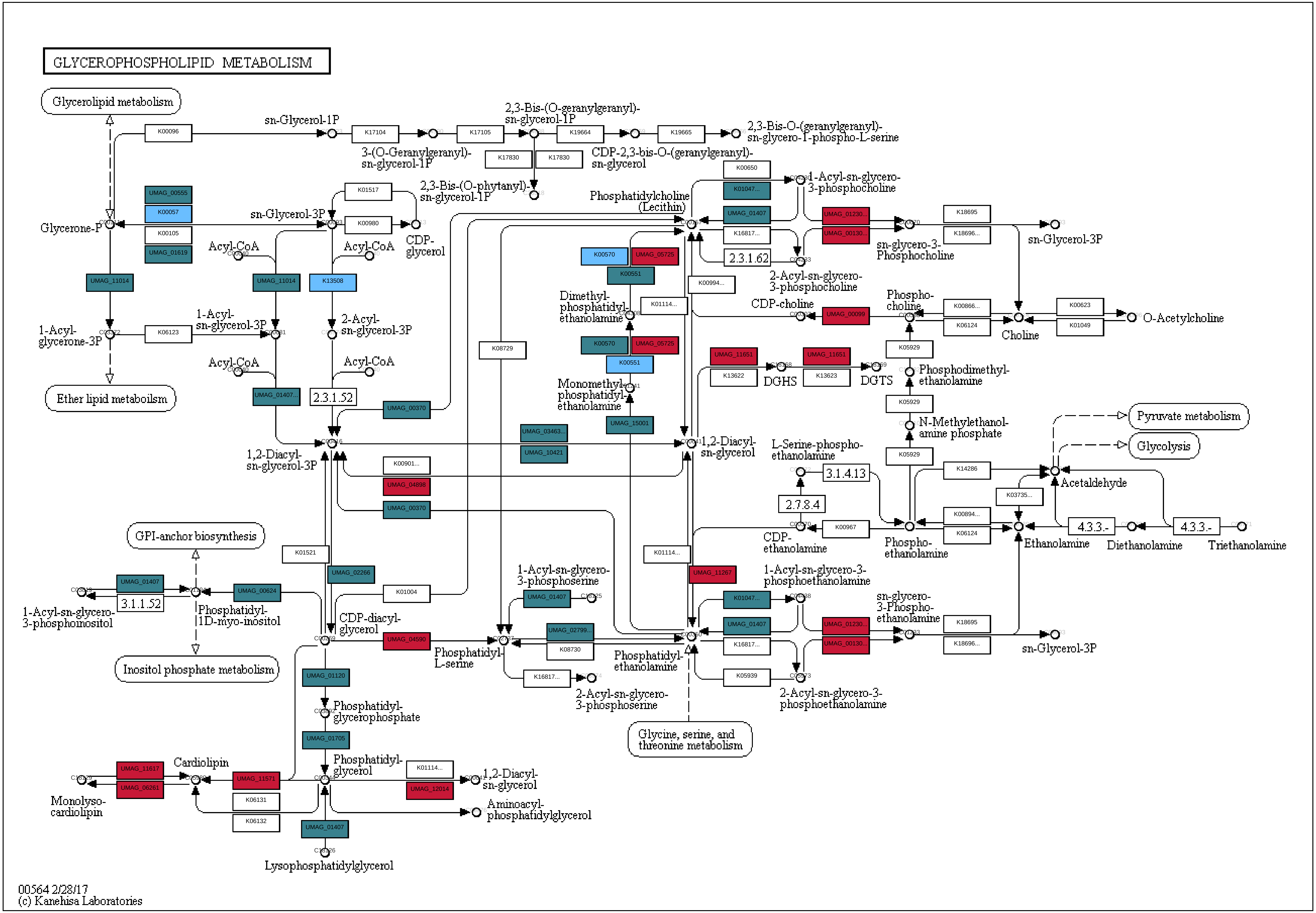
GLYCEROPHOSPHOLIPID METABOLISM
Glycerolipid metabolism
Ether lipid metaboilsm
GPI-anchor biosynthesis
Inositol phosphate metabolism
Glycine, serine, and threonine metabolism
Pyruvate metabolism
Glycolysis
sn-Glycerol-1P
3-(O-Geranylgeranyl)-sn-glycerol-1P
2,3-Bis-(O-geranylgeranyl)-sn-glycerol-1P
CDP-2,3-bis-(O-(geranylgeranyl)-sn-glycerol
2,3-Bis-O-(geranylgeranyl)-sn-glycero-1-phospho-L-serine
2,3-Bis-(O-phytanyl)-sn-glycerol-1P
Glycerone-P
sn-Glycerol-3P
CDP-glycerol
Acyl-CoA
1-Acyl-glycerone-3P
1-Acyl-sn-glycerol-3P
2-Acyl-sn-glycerol-3P
1,2-Diacyl-sn-glycerol-3P
1,2-Diacyl-sn-glycerol
CDP-diacyl-glycerol
Phosphatidyl-1D-myo-inositol
1-Acyl-sn-glycero-3-phosphoinositol
Phosphatidyl-glycerophosphate
Phosphatidyl-glycerol
Cardiolipin
Monolyso-cardiolipin
Aminoacyl-phosphatidylglycerol
Lysophosphatidylglycerol
Phosphatidyl-L-serine
1-Acyl-sn-glycero-3-phosphoserine
2-Acyl-sn-glycero-3-phosphoserine
Phosphatidyl-ethanolamine
Phosphatidylcholine (Lecithin)
Dimethyl-phosphatidyl-ethanolamine
Monomethyl-phosphatidyl-ethanolamine
1-Acyl-sn-glycero-3-phosphocholine
2-Acyl-sn-glycero-3-phosphocholine
sn-glycero-3-Phosphocholine
CDP-choline
Phospho-choline
Choline
O-Acetylcholine
DGHS
DGTS
Phosphodimethyl-ethanolamine
N-Methylethanol-amine phosphate
L-Serine-phospho-ethanolamine
Acetaldehyde
CDP-ethanolamine
Phospho-ethanolamine
Ethanolamine
Diethanolamine
Triethanolamine
1-Acyl-sn-glycero-3-phosphoethanolamine
2-Acyl-sn-glycero-3-phosphoethanolamine
sn-glycero-3-Phospho-ethanolamine
K00096
K17104
K17105
K19664
K19665
K17830
K00650
K01517
K00980
K00057
K00105
K06123
K13508
2.3.1.52
2.3.1.62
3.1.4.13
2.7.8.4
4.3.3.-
3.1.1.52
K00570
K00551
K00994...
K01114...
K08729
K18695
K18696...
K00866...
K06124
K00623
K01049
K05929
K13622
K13623
K14286
K03735...
K00894...
K00967
K00901...
K01521
K01004
K08730
K16817...
K01047...
K05939
K06131
K06132
UMAG_00555
UMAG_01619
UMAG_11014
UMAG_01407
UMAG_00370
UMAG_04898
UMAG_02266
UMAG_00624
UMAG_04590
UMAG_01120
UMAG_01705
UMAG_11617
UMAG_06261
UMAG_11571
UMAG_12014
UMAG_03463...
UMAG_10421
UMAG_02799...
UMAG_15001
UMAG_05725
UMAG_01230...
UMAG_00130...
UMAG_00099
UMAG_11651
UMAG_11267
00564 2/28/17
(c) Kanehisa Laboratories

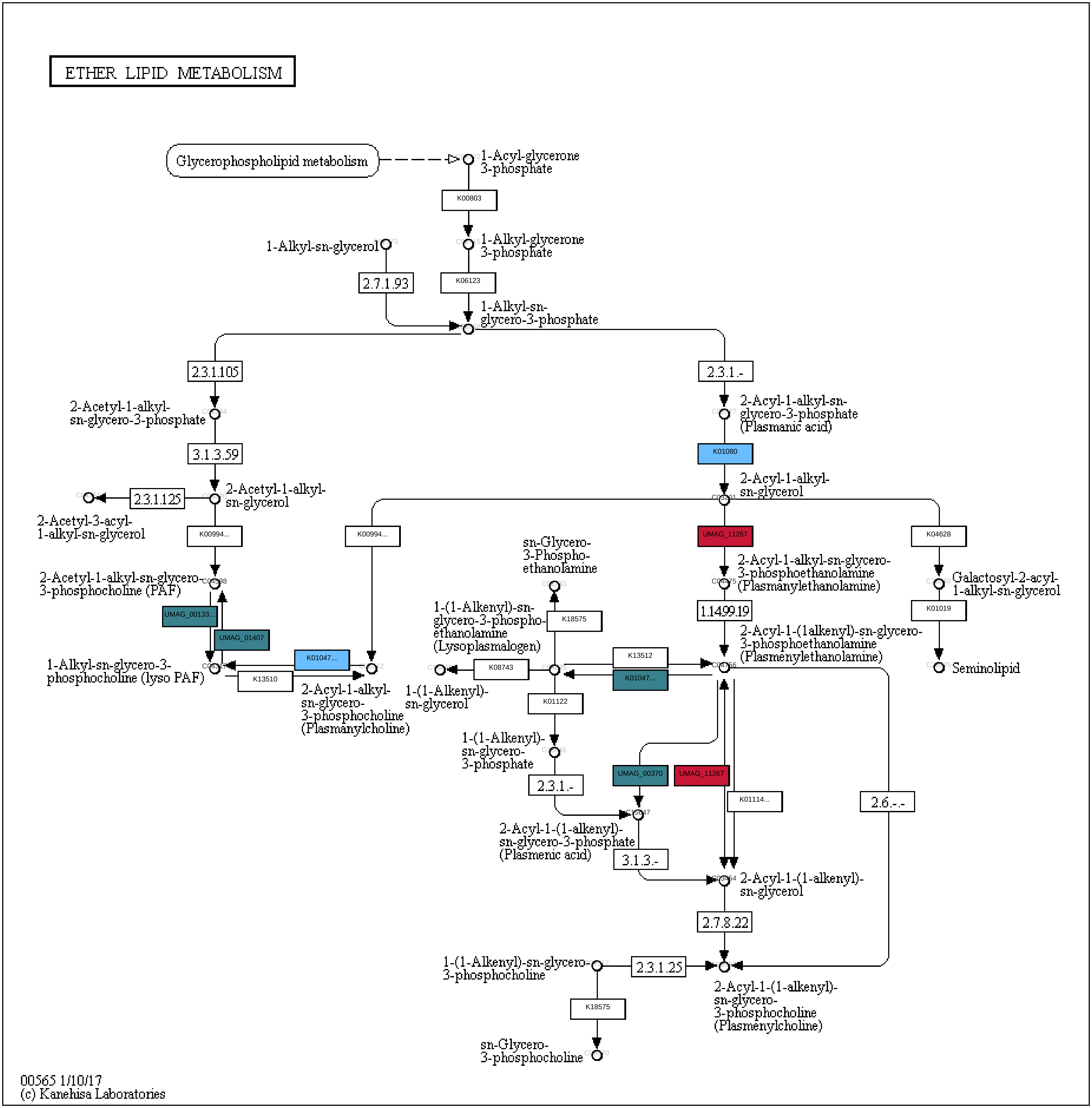
ETHER LIPID METABOLISM
Glycerophospholipid metabolism
1-Acyl-glycerone 3-phosphate
K00803
1-Alkyl-sn-glycerol
1-Alkyl-glycerone 3-phosphate
2.7.1.93
K06123
1-Alkyl-sn-glycero-3-phosphate
2.3.1.105
2.3.1.-
2-Acetyl-1-alkyl-sn-glycero-3-phosphate
2-Acyl-1-alkyl-sn-glycero-3-phosphate (Plasmanic acid)
3.1.3.59
K01080
2.3.1.125
2-Acetyl-1-alkyl-sn-glycerol
2-Acyl-1-alkyl-sn-glycerol
2-Acetyl-3-acyl-1-alkyl-sn-glycerol
K00994...
K00994...
UMAG_11267
K04628
sn-Glycero-3-Phospho-ethanolamine
2-Acetyl-1-alkyl-sn-glycero-3-phosphocholine (PAF)
2-Acyl-1-alkyl-sn-glycero-3-phosphoethanolamine (Plasmanylethanolamine)
Galactosyl-2-acyl-1-alkyl-sn-glycerol
1-(1-Alkenyl)-sn-glycero-3-phospho-ethanolamine (Lysoplasmalogen)
K18575
1.14.99.19
K01019
UMAG_00133...
UMAG_01407
2-Acyl-1-(1alkenyl)-sn-glycero-3-phosphoethanolamine (Plasmenylethanolamine)
K13512
K01047...
1-Alkyl-sn-glycero-3-phosphocholine (lyso PAF)
K13510
K08743
K01047...
Seminolipid
2-Acyl-1-alkyl-sn-glycero-3-phosphocholine (Plasmanylcholine)
1-(1-Alkenyl)-sn-glycerol
K01122
1-(1-Alkenyl)-sn-glycero-3-phosphate
2.3.1.-
UMAG_00370
UMAG_11267
K01114...
2.6.-.-
2-Acyl-1-(1-alkenyl)-sn-glycero-3-phosphate (Plasmenic acid)
3.1.3.-
2-Acyl-1-(1-alkenyl)-sn-glycerol
2.7.8.22
1-(1-Alkenyl)-sn-glycero-3-phosphocholine
2.3.1.25
2-Acyl-1-(1-alkenyl)-sn-glycero-3-phosphocholine (Plasmenylcholine)
K18575
sn-Glycero-3-phosphocholine
00565 1/10/17
(c) Kanehisa Laboratories

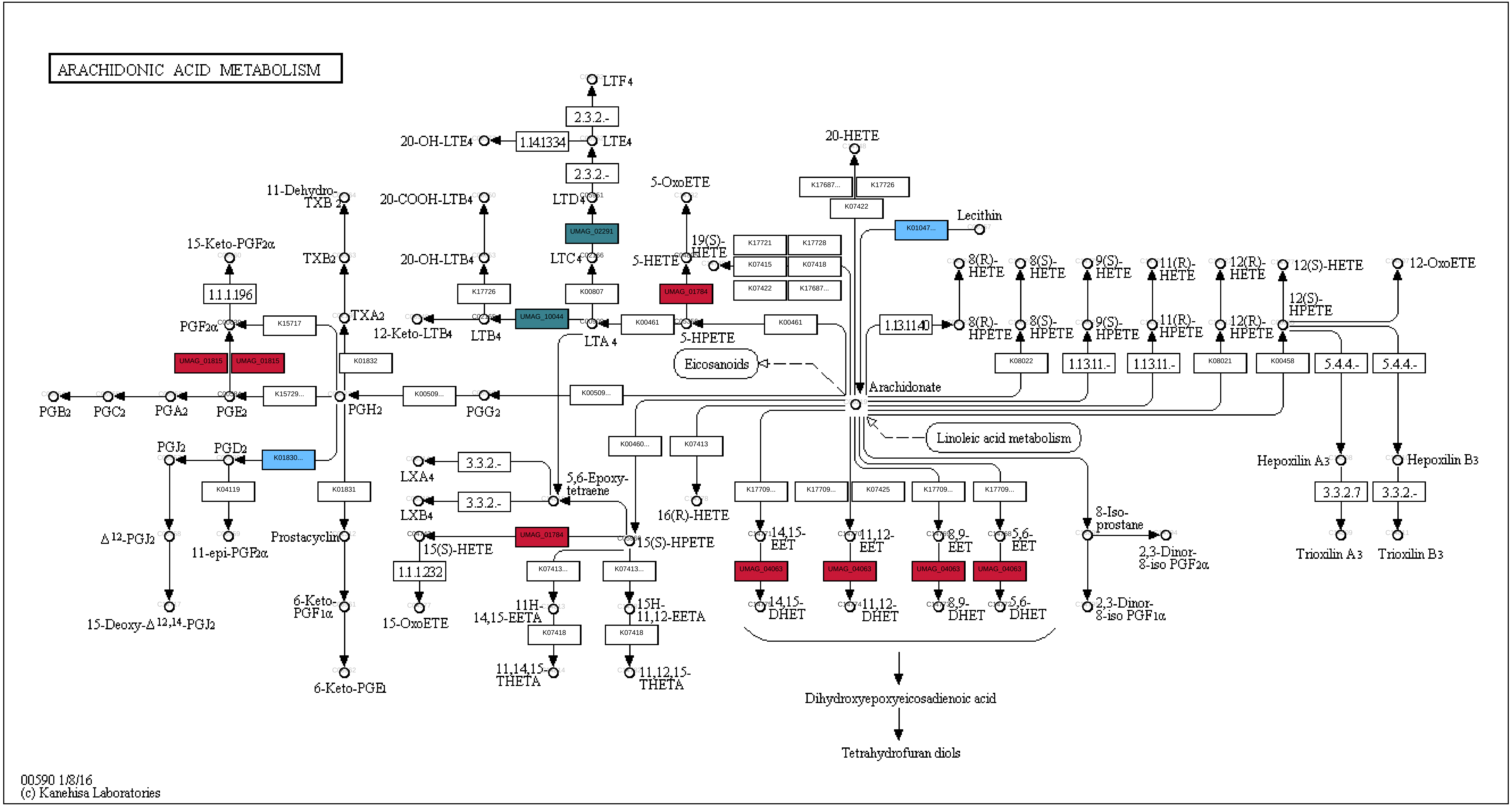
ARACHIDONIC ACID METABOLISM
LTF4
2.3.2.-
20-OH-LTE4
1.14.13.34
LTE4
2.3.2.-
LTD4
UMAG_02291
LTC4
K00807
LTA 4
K00461
5-HPETE
K00461
5-OxoETE
19(S)-HETE
5-HETE
UMAG_01784
Eicosanoids
20-HETE
K17687...
K17726
K07422
K17721
K17728
K07415
K07418
K07422
K17687...
Lecithin
K01047...
Arachidonate
Linoleic acid metabolism
11-Dehydro-TXB 2
TXB2
TXA2
K01832
K01831
Prostacyclin
6-Keto-PGF1α
6-Keto-PGE1
20-COOH-LTB4
20-OH-LTB4
K17726
UMAG_10044
12-Keto-LTB4
LTB4
15-Keto-PGF2α
1.1.1.196
PGF2α
K15717
UMAG_01815
UMAG_01815
PGB2
PGC2
PGA2
PGE2
K15729...
PGH2
K00509...
PGG2
K00509...
PGJ2
PGD2
K01830...
K04119
Δ12-PGJ2
11-epi-PGF2α
15-Deoxy-Δ12,14-PGJ2
LXA4
3.3.2.-
LXB4
3.3.2.-
5,6-Epoxy-tetraene
UMAG_01784
15(S)-HETE
15(S)-HPETE
1.1.1.232
15-OxoETE
K07413...
K07413...
11H-14,15-EETA
15H-11,12-EETA
K07418
K07418
11,14,15-THETA
11,12,15-THETA
K00460...
K07413
16(R)-HETE
K17709...
K17709...
K07425
K17709...
K17709...
14,15-EET
11,12-EET
8,9-EET
5,6-EET
UMAG_04063
UMAG_04063
UMAG_04063
UMAG_04063
14,15-DHET
11,12-DHET
8,9-DHET
5,6-DHET
Dihydroxyepoxyeicosadienoic acid
Tetrahydrofuran diols
8-Iso-prostane
2,3-Dinor-8-iso PGF2α
2,3-Dinor-8-iso PGF1α
8(R)-HETE
8(S)-HETE
9(S)-HETE
11(R)-HETE
12(R)-HETE
12(S)-HETE
12-OxoETE
1.13.11.40
8(R)-HPETE
8(S)-HPETE
9(S)-HPETE
11(R)-HPETE
12(R)-HPETE
12(S)-HPETE
K08022
1.13.11.-
1.13.11.-
K08021
K00458
5.4.4.-
5.4.4.-
Hepoxilin A3
Hepoxilin B3
3.3.2.7
3.3.2.-
Trioxilin A3
Trioxilin B3
00590 1/8/16
(c) Kanehisa Laboratories

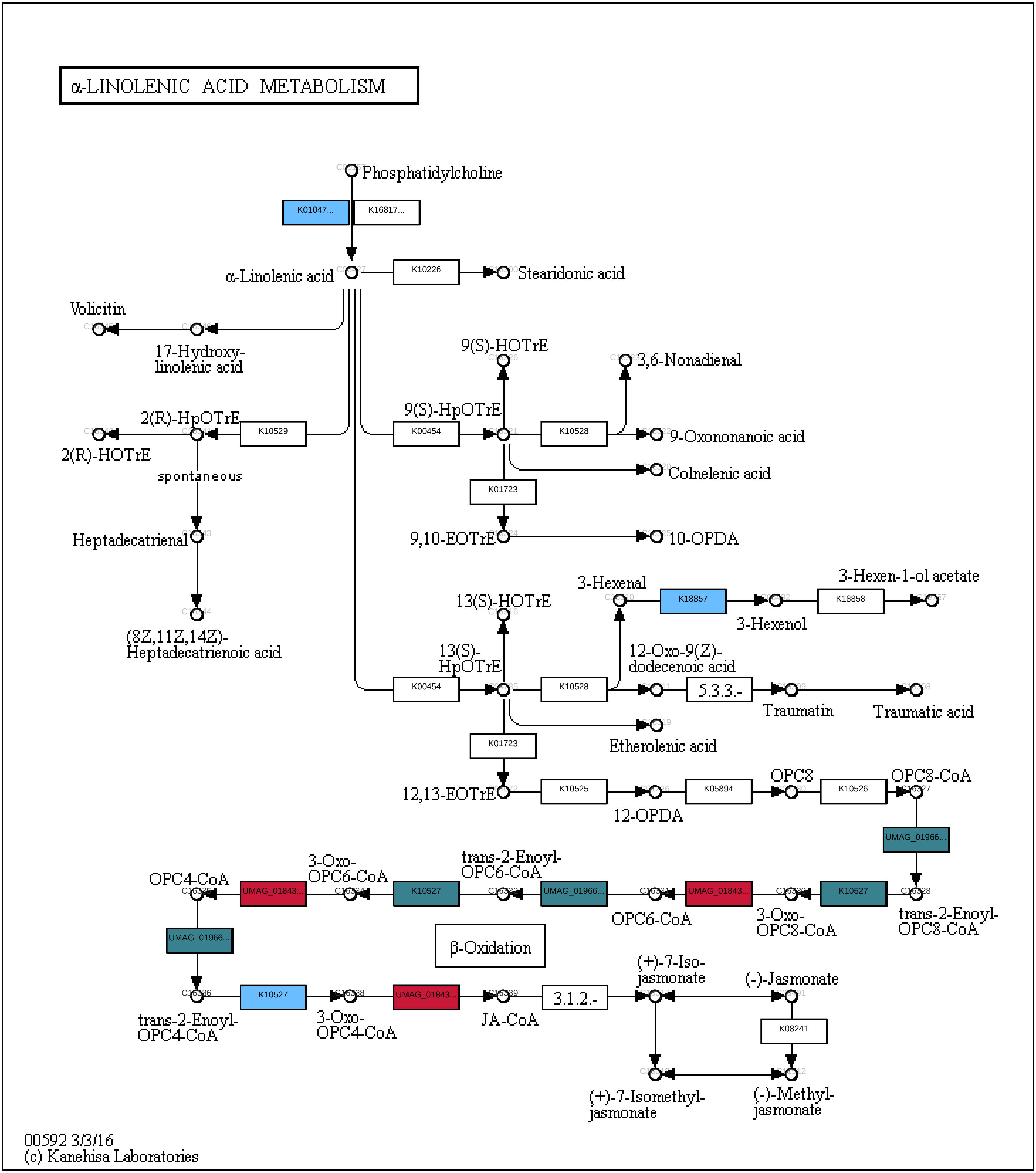
α-LINOLENIC ACID METABOLISM
Phosphatidylcholine
K01047...
K16817...
α-Linolenic acid
K10226
Stearidonic acid
Volicitin
17-Hydroxy-linolenic acid
9(S)-HOTrE
3,6-Nonadienal
2(R)-HpOTrE
K10529
9(S)-HpOTrE
K00454
K10528
2(R)-HOTrE
9-Oxononanoic acid
spontaneous
Colnelenic acid
K01723
Heptadecatrienal
9,10-EOTrE
10-OPDA
3-Hexenal
K18857
K18858
3-Hexen-1-ol acetate
13(S)-HOTrE
3-Hexenol
(8Z,11Z,14Z)-Heptadecatrienoic acid
13(S)-HpOTrE
12-Oxo-9(Z)-dodecenoic acid
K00454
K10528
5.3.3.-
Traumatin
Traumatic acid
Etherolenic acid
K01723
12,13-EOTrE
K10525
K05894
OPC8
K10526
OPC8-CoA
12-OPDA
UMAG_01966...
3-Oxo-OPC6-CoA
trans-2-Enoyl-OPC6-CoA
OPC4-CoA
UMAG_01843...
K10527
UMAG_01966...
UMAG_01843...
K10527
OPC6-CoA
3-Oxo-OPC8-CoA
trans-2-Enoyl-OPC8-CoA
UMAG_01966...
β-Oxidation
(+)-7-Iso-jasmonate
(-)-Jasmonate
K10527
UMAG_01843...
3.1.2.-
K08241
trans-2-Enoyl-OPC4-CoA
3-Oxo-OPC4-CoA
JA-CoA
(+)-7-Isomethyl-jasmonate
(-)-Methyl-jasmonate
00592 3/3/16
(c) Kanehisa Laboratories

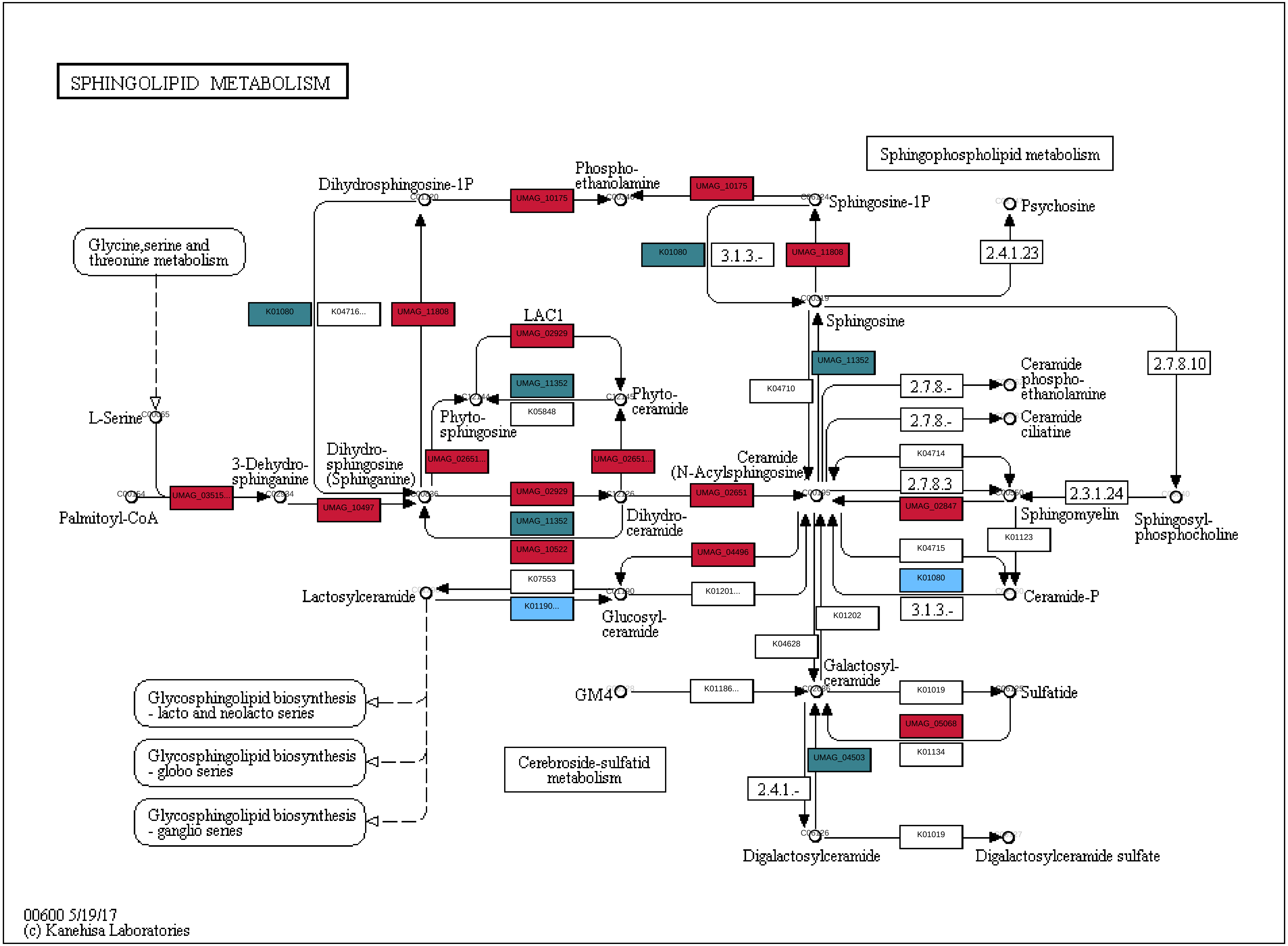
SPHINGOLIPID METABOLISM
Sphingophospholipid metabolism
Glycine, serine and threonine metabolism
L-Serine
Palmitoyl-CoA
UMAG_03515...
3-Dehydro-sphinganine
UMAG_10497
Dihydro-sphingosine (Sphinganine)
K01080
K04716...
UMAG_11808
Dihydrosphingosine-1P
UMAG_10175
Phospho-ethanolamine
UMAG_10175
Sphingosine-1P
K01080
3.1.3.-
UMAG_11808
Psychosine
2.4.1.23
Sphingosine
LAC1
UMAG_02929
UMAG_11352
K05848
Phyto-sphingosine
Phyto-ceramide
UMAG_02651...
UMAG_02651...
UMAG_02929
UMAG_11352
UMAG_10522
Dihydro-ceramide
UMAG_02651
Ceramide (N-Acylsphingosine)
UMAG_11352
K04710
2.7.8.-
Ceramide phospho-ethanolamine
2.7.8.-
Ceramide ciliatine
2.7.8.10
K04714
2.7.8.3
UMAG_02847
2.3.1.24
Sphingomyelin
Sphingosyl-phosphocholine
K04715
K01123
K01080
Ceramide-P
3.1.3.-
UMAG_04496
K07553
K01190...
K01201...
Lactosylceramide
Glucosyl-ceramide
K01202
K04628
Galactosyl-ceramide
GM4
K01186...
K01019
Sulfatide
UMAG_05068
K01134
UMAG_04503
2.4.1.-
Digalactosylceramide
K01019
Digalactosylceramide sulfate
Glycosphingolipid biosynthesis - lacto and neolacto series
Glycosphingolipid biosynthesis - globo series
Glycosphingolipid biosynthesis - ganglio series
Cerebroside-sulfatid metabolism
00600 5/19/17
(c) Kanehisa Laboratories

# 4. Nucleotide metabolism

| MAP | PATHWAY |
|---|---|
| **230** | Purine metabolism |
| **240** | Pyrimidine metabolism |

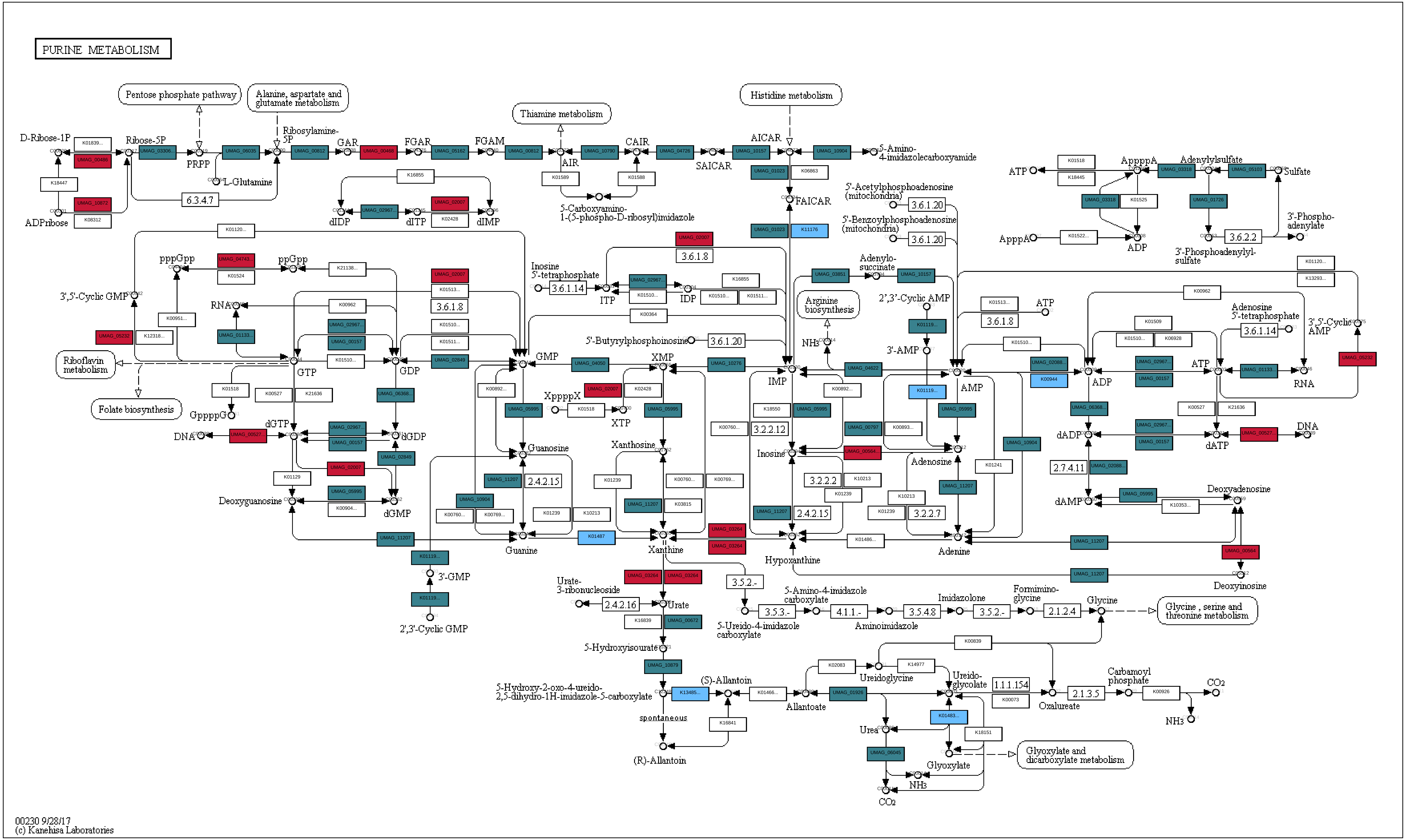

PURINE METABOLISM
Pentose phosphate pathway
Alanine, aspartate and glutamate metabolism
Thiamine metabolism
Histidine metabolism
D-Ribose-1P
Ribose-5P
PRPP
Ribosylamine-5P
GAR
FGAR
FGAM
AIR
CAIR
SAICAR
AICAR
5-Amino-4-imidazolecarboxyamide
FAICAR
L-Glutamine
ADPribose
6.3.4.7
5-Carboxyamino-1-(5-phospho-D-ribosyl)imidazole
dIDP
dITP
dIMP
UMAG_00486
UMAG_10872
UMAG_03306...
UMAG_06035
UMAG_00812
UMAG_00468
UMAG_05162
UMAG_10790
UMAG_04726
UMAG_10157
UMAG_10904
UMAG_01023
UMAG_02967...
UMAG_02007
K01839...
K18447
K08312
K16855
K02428
K01589
K01588
K06863
K11176
ATP
ApppA
AppppA
ADP
Adenylylsulfate
Sulfate
3'-Phospho-adenylate
3'-Phosphoadenylyl-sulfate
3.6.2.2
5'-Acetylphosphoadenosine (mitochondria)
5'-Benzoylphosphoadenosine (mitochondria)
3.6.1.20
UMAG_03318
UMAG_05103
UMAG_01726
K01518
K18445
K01525
K01522...
pppGpp
ppGpp
3',5'-Cyclic GMP
RNA
GMP
GTP
GDP
GpppG
dGTP
dGDP
dGMP
DNA
Deoxyguanosine
Guanosine
Guanine
Riboflavin metabolism
Folate biosynthesis
3'-GMP
2',3'-Cyclic GMP
Inosine 5'-tetraphosphate
3.6.1.14
ITP
IDP
XMP
XTP
XppppX
Xanthosine
Xanthine
5'-Butyrylphosphoinosine
3.6.1.8
2.4.2.15
IMP
Inosine
Hypoxanthine
Adenylo-succinate
Arginine biosynthesis
NH3
2',3'-Cyclic AMP
3'-AMP
AMP
Adenosine
Adenine
3.2.2.12
3.2.2.2
3.2.2.7
Adenosine 5'-tetraphosphate
3',5'-Cyclic AMP
dADP
dATP
dAMP
Deoxyadenosine
Deoxyinosine
2.7.4.11
UMAG_04743...
UMAG_05232
UMAG_01133...
UMAG_00157
UMAG_02849
UMAG_06368...
UMAG_00527...
UMAG_05995
UMAG_04050
UMAG_10276
UMAG_04622
UMAG_03851
UMAG_00797
UMAG_00564...
UMAG_11207
UMAG_02088...
UMAG_00564
UMAG_01119...
UMAG_03264
UMAG_00672
UMAG_10879
UMAG_01926
UMAG_06045
K01120...
K01524
K21138...
K00951...
K12318...
K00962
K01513...
K01510...
K01511...
K01518
K00527
K21636
K01129
K00904...
K00892...
K00760...
K00769...
K01239
K10213
K01487
K02428
K03815
K00364
K18550
K00893...
K01241
K01509
K06928
K13293...
K00944
K10353...
K01486...
Urate-3-ribonucleoside
2.4.2.16
Urate
K16839
5-Hydroxyisourate
5-Hydroxy-2-oxo-4-ureido-2,5-dihydro-1H-imidazole-5-carboxylate
K13485...
(S)-Allantoin
spontaneous
K16841
(R)-Allantoin
3.5.2.-
5-Amino-4-imidazolone carboxylate
3.5.3.-
4.1.1.-
3.5.4.8
Imidazolone
Formimino-glycine
2.1.2.4
Glycine
Glycine, serine and threonine metabolism
5-Ureido-4-imidazole carboxylate
Aminoimidazolone
K00839
K02083
K14977
K01466...
Allantoate
Ureidoglycine
Ureido-glycolate
1.1.1.154
K00073
Oxalureate
2.1.3.5
Carbamoyl phosphate
K00926
CO2
NH3
K01483...
K18151
Urea
Glyoxylate
Glyoxylate and dicarboxylate metabolism
CO2
00230 9/28/17
(c) Kanehisa Laboratories

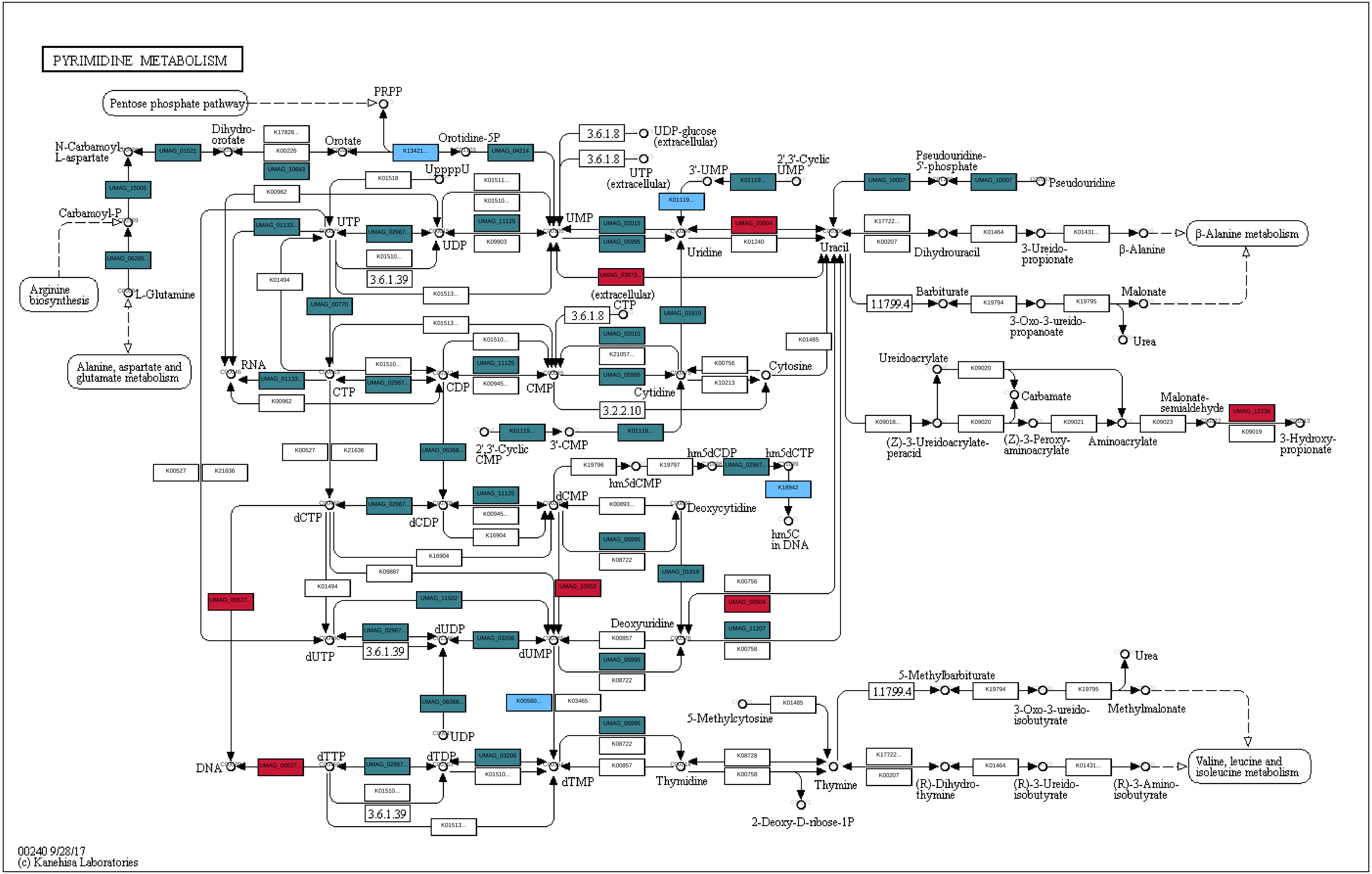
PYRIMIDINE METABOLISM
Pentose phosphate pathway
PRPP
N-Carbamoyl-L-aspartate
UMAG_01521
Dihydro-orotate
K17828...
K00226
UMAG_10643
Orotate
K13421...
Orotidine-5P
UMAG_04214
UMAG_15005
Carbamoyl-P
UMAG_06285...
L-Glutamine
Arginine biosynthesis
Alanine, aspartate and glutamate metabolism
K01518
UppppU
K00962
K01511...
K01510...
UMAG_11125
K09903
UMAG_01133...
UTP
UMAG_02967...
UDP
K01510...
3.6.1.39
K01494
K01513...
UMP
3.6.1.8
UDP-glucose (extracellular)
3.6.1.8
UTP (extracellular)
3'-UMP
UMAG_01119...
2',3'-Cyclic UMP
K01119...
UMAG_02015
UMAG_05995
UMAG_03504
K01240
Uridine
UMAG_03873...
(extracellular)
Uracil
Pseudouridine-5'-phosphate
UMAG_10007
UMAG_10007
Pseudouridine
K17722...
K00207
Dihydrouracil
K01464
3-Ureido-propionate
K01431...
β-Alanine
β-Alanine metabolism
1.17.99.4
Barbiturate
K19794
3-Oxo-3-ureido-propanoate
K19795
Malonate
Urea
UMAG_00770
K01513...
3.6.1.8
CTP
UMAG_01919
K01510...
UMAG_02015
K21057...
UMAG_05995
K01485
K00756
Cytosine
K10213
RNA
UMAG_01133...
CTP
K01510...
UMAG_02967...
CDP
UMAG_11125
K00945...
CMP
Cytidine
K00962
3.2.2.10
K01119...
2',3'-Cyclic CMP
3'-CMP
K01119...
Ureidoacrylate
K09020
Carbamate
K09018...
K09020
K09021
K09023
Malonate-semialdehyde
UMAG_12236
K09019
(Z)-3-Ureidoacrylate-peracid
(Z)-3-Peroxy-aminoacrylate
Aminoacrylate
3-Hydroxy-propionate
K00527
K21636
UMAG_06368...
K00527
K21636
K19796
hm5dCMP
K19797
hm5dCDP
UMAG_02967...
hm5dCTP
K18942
hm5C in DNA
dCTP
UMAG_02967...
dCDP
UMAG_11125
K00945...
K16904
dCMP
K00893...
Deoxycytidine
UMAG_05995
K08722
K16904
K09887
K01494
UMAG_10652
UMAG_01919
K00756
UMAG_03504
UMAG_00527...
UMAG_11502
dUDP
UMAG_02967...
dUTP
3.6.1.39
UMAG_03206
dUMP
Deoxyuridine
K00857
UMAG_05995
K08722
UMAG_11207
K00758
UMAG_06368...
K00560...
K03465
UDP
UMAG_05995
K08722
5-Methylcytosine
K01485
5-Methylbarbiturate
1.17.99.4
K19794
3-Oxo-3-ureido-isobutyrate
K19795
Urea
Methylmalonate
Valine, leucine and isoleucine metabolism
DNA
UMAG_00527...
dTTP
UMAG_02967...
dTDP
UMAG_03206
K01510...
dTMP
K00857
Thymidine
K08728
K00758
Thymine
K17722...
K00207
(R)-Dihydro-thymine
K01464
(R)-3-Ureido-isobutyrate
K01431...
(R)-3-Amino-isobutyrate
K01510...
3.6.1.39
K01513...
2-Deoxy-D-ribose-1P
00240 9/28/17
(c) Kanehisa Laboratories

# 5. Amino Acid metabolism

| MAP | PATHWAY |
|---|---|
| **220** | Arginine biosynthesis |
| **250** | Alanine, aspartate and glutamate metabolism |
| **260** | Glycine, serine and threonine metabolism |
| **270** | Cysteine and methionine metabolism |
| **280** | Valine, leucine and isoleucine degradation |
| **290** | Valine, leucine and isoleucine biosynthesis |
| **300** | Lysine biosynthesis |
| **310** | Lysine degradation |
| **330** | Arginine and proline metabolism |
| **340** | Histidine metabolism |
| **350** | Tyrosine metabolism |
| **360** | Phenylalanine metabolism |
| **380** | Tryptophan metabolism |
| **400** | Phenylalanine, tyrosine and tryptophan biosynthesis |

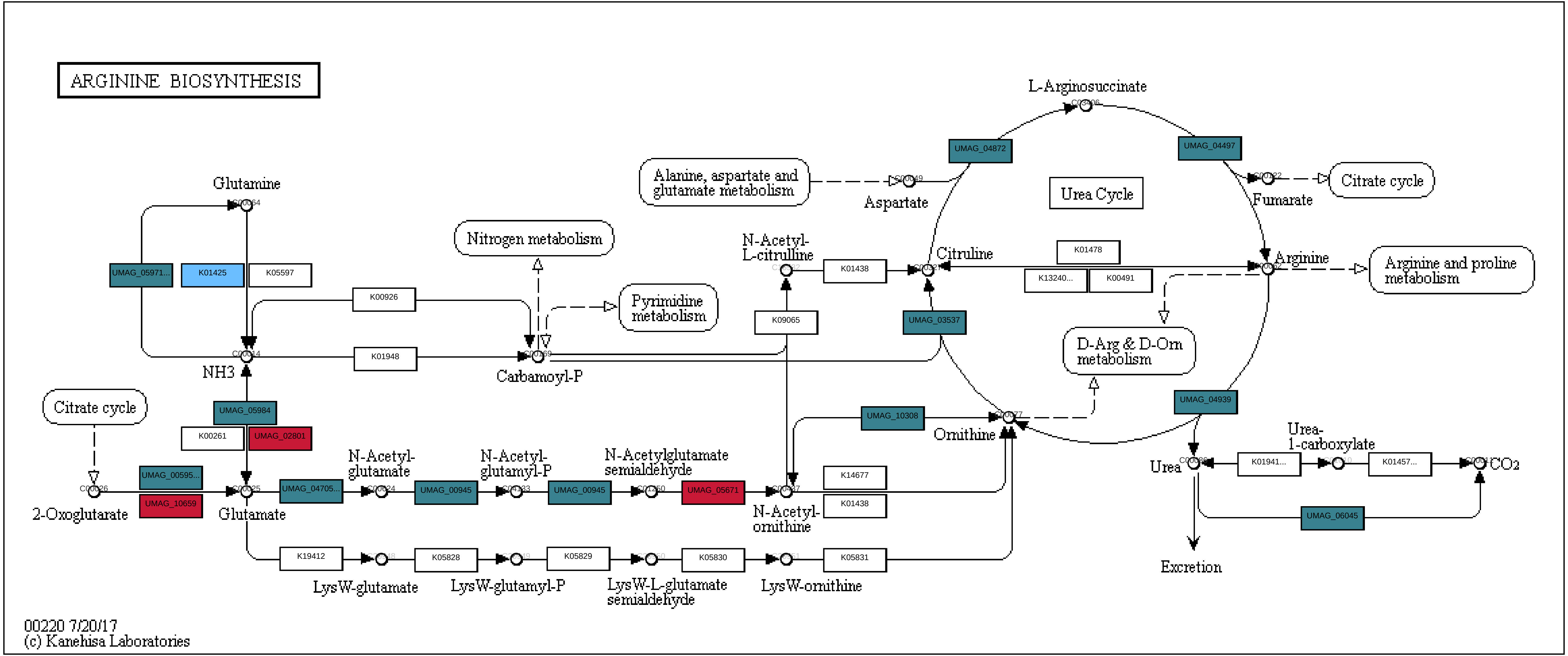
ARGININE BIOSYNTHESIS
Glutamine
UMAG_05971...
K01425
K05597
K00926
Nitrogen metabolism
Pyrimidine metabolism
K01948
NH3
Carbamoyl-P
Citrate cycle
UMAG_05984
K00261
UMAG_02801
UMAG_00595...
UMAG_10659
2-Oxoglutarate
Glutamate
UMAG_04705...
N-Acetyl-glutamate
UMAG_00945
N-Acetyl-glutamyl-P
UMAG_00945
N-Acetylglutamate semialdehyde
UMAG_05671
N-Acetyl-ornithine
K14677
K01438
K19412
LysW-glutamate
K05828
LysW-glutamyl-P
K05829
LysW-L-glutamate semialdehyde
K05830
LysW-ornithine
K05831
Alanine, aspartate and glutamate metabolism
Aspartate
N-Acetyl-L-citrulline
K01438
K09065
Citrulline
UMAG_03537
UMAG_04872
L-Arginosuccinate
UMAG_04497
Urea Cycle
Fumarate
Citrate cycle
K01478
K13240...
K00491
Arginine
Arginine and proline metabolism
D-Arg & D-Orn metabolism
UMAG_10308
Ornithine
UMAG_04939
Urea
Urea-1-carboxylate
K01941...
K01457...
CO2
UMAG_06045
Excretion
00220 7/20/17
(c) Kanehisa Laboratories

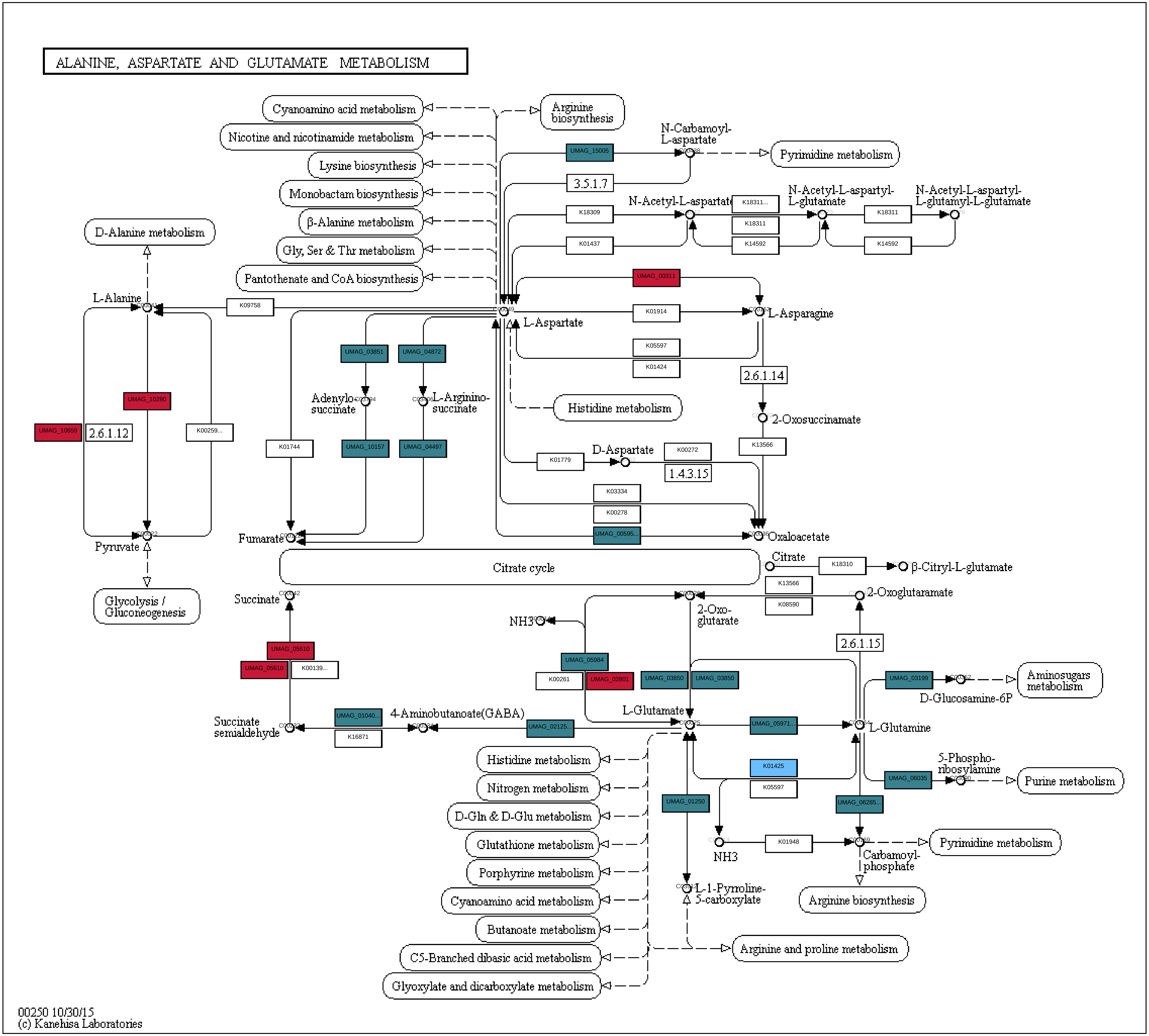
ALANINE, ASPARTATE AND GLUTAMATE METABOLISM
Cyanoamino acid metabolism
Nicotine and nicotinamide metabolism
Lysine biosynthesis
Monobactam biosynthesis
β-Alanine metabolism
Gly, Ser & Thr metabolism
Pantothenate and CoA biosynthesis
Arginine biosynthesis
N-Carbamoyl-L-aspartate
Pyrimidine metabolism
UMAG_15005
3.5.1.7
N-Acetyl-L-aspartate
N-Acetyl-L-aspartyl-L-glutamate
N-Acetyl-L-aspartyl-L-glutamyl-L-glutamate
K18309
K18311...
K18311
K14592
K01437
K18311
K14592
D-Alanine metabolism
L-Alanine
K09758
UMAG_00311
L-Aspartate
K01914
L-Asparagine
K05597
K01424
2.6.1.14
UMAG_03851
UMAG_04872
Adenylo-succinate
L-Arginino-succinate
Histidine metabolism
2-Oxosuccinamate
UMAG_10290
UMAG_10659
2.6.1.12
K00259...
K01744
UMAG_10157
UMAG_04497
D-Aspartate
K01779
K00272
1.4.3.15
K13566
K03334
K00278
UMAG_00595...
Oxaloacetate
Fumarate
Pyruvate
Glycolysis / Gluconeogenesis
Citrate cycle
Citrate
K18310
β-Citryl-L-glutamate
K13566
K08590
Succinate
2-Oxoglutaramate
NH3
2-Oxo-glutarate
2.6.1.15
UMAG_05610
UMAG_05610
K00139...
UMAG_05984
K00261
UMAG_02801
UMAG_03850
UMAG_03850
UMAG_03199
Aminosugars metabolism
D-Glucosamine-6P
4-Aminobutanoate(GABA)
UMAG_01040...
K16871
UMAG_02125...
Succinate semialdehyde
L-Glutamate
UMAG_05971...
L-Glutamine
Histidine metabolism
Nitrogen metabolism
D-Gln & D-Glu metabolism
Glutathione metabolism
Porphyrine metabolism
Cyanoamino acid metabolism
Butanoate metabolism
C5-Branched dibasic acid metabolism
Glyoxylate and dicarboxylate metabolism
K01425
K05597
UMAG_01250
UMAG_06285...
UMAG_06035
5-Phospho-ribosylamine
Purine metabolism
NH3
K01948
Carbamoyl-phosphate
Pyrimidine metabolism
L-1-Pyrroline-5-carboxylate
Arginine biosynthesis
Arginine and proline metabolism
00250 10/30/15
(c) Kanehisa Laboratories

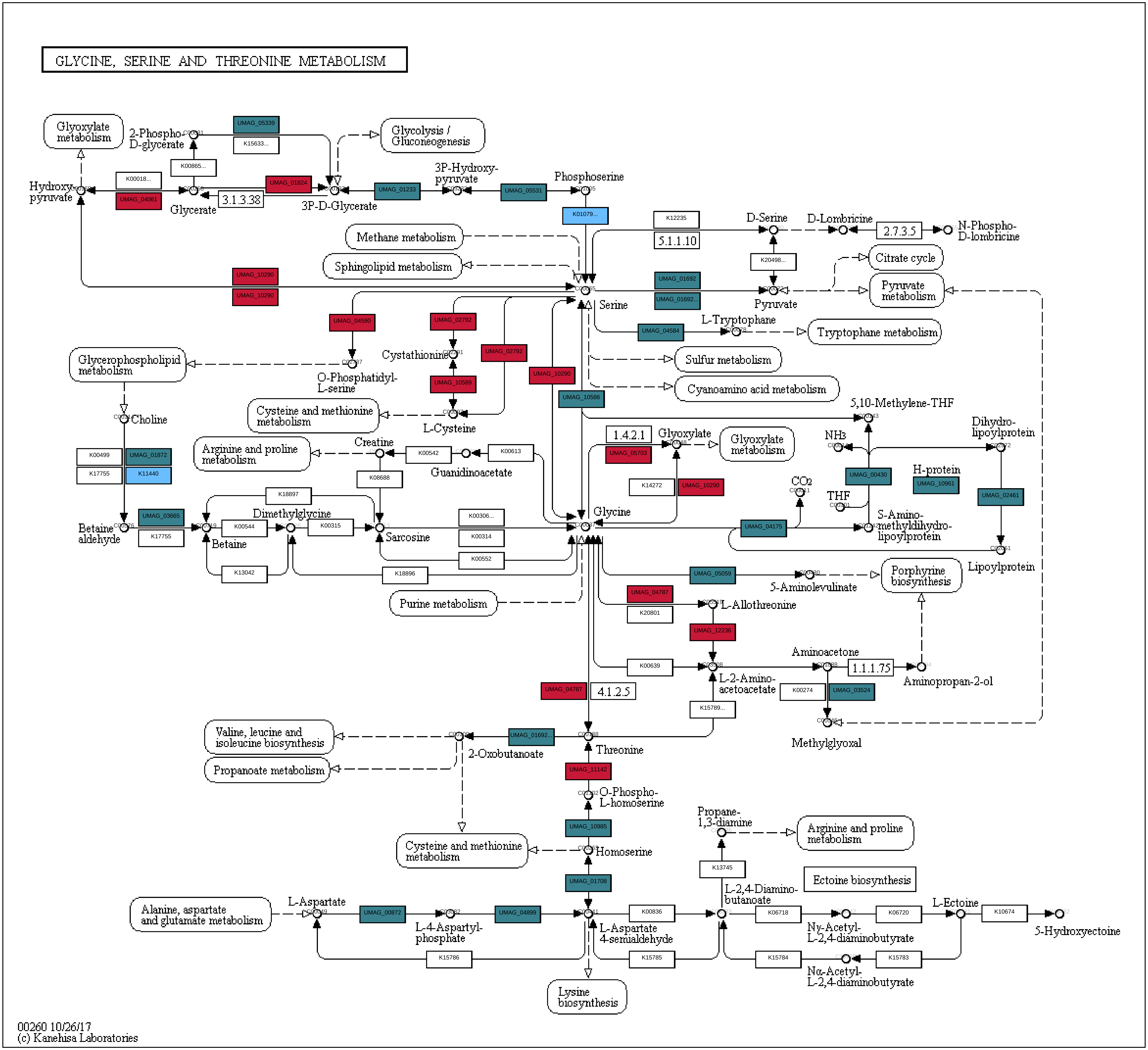
GLYCINE, SERINE AND THREONINE METABOLISM
Glyoxylate metabolism
2-Phospho-D-glycerate
UMAG_05339
K15633...
Glycolysis / Gluconeogenesis
K00865...
K00018...
UMAG_01824
UMAG_01233
3P-Hydroxy-pyruvate
UMAG_05531
Phosphoserine
Hydroxy-pyruvate
UMAG_04061
Glycerate
3.1.3.38
3P-D-Glycerate
K01079...
K12235
5.1.1.10
D-Serine
D-Lombricine
2.7.3.5
N-Phospho-D-lombricine
Methane metabolism
Sphingolipid metabolism
K20498...
Citrate cycle
UMAG_10290
UMAG_10290
UMAG_01692
UMAG_01692...
Pyruvate metabolism
Serine
Pyruvate
UMAG_04590
UMAG_02792
UMAG_04584
L-Tryptophane
Tryptophane metabolism
Glycerophospholipid metabolism
Cystathionine
UMAG_02792
UMAG_10290
Sulfur metabolism
O-Phosphatidyl-L-serine
UMAG_10589
Cyanoamino acid metabolism
UMAG_10586
Cysteine and methionine metabolism
L-Cysteine
5,10-Methylene-THF
Choline
1.4.2.1
Glyoxylate
Glyoxylate metabolism
Dihydro-lipoylprotein
Arginine and proline metabolism
Creatine
K00542
K00613
NH3
UMAG_05703
K00499
UMAG_01872
Guanidinoacetate
UMAG_00430
H-protein
K17755
K11440
K08688
UMAG_10961
K14272
UMAG_10290
CO2
THF
UMAG_02461
K18897
Dimethylglycine
Glycine
S-Amino-methyldihydro-lipoylprotein
Betaine aldehyde
UMAG_03665
K00544
K00315
K00306...
UMAG_04175
K17755
Betaine
Sarcosine
K00314
K00552
Lipoylprotein
K13042
K18896
UMAG_05059
Porphyrine biosynthesis
5-Aminolevulinate
Purine metabolism
UMAG_04787
K20801
L-Allothreonine
UMAG_12236
Aminoacetone
K00639
1.1.1.75
Aminopropan-2-ol
UMAG_04787
4.1.2.5
L-2-Amino-acetoacetate
K00274
UMAG_03524
K15789...
Valine, leucine and isoleucine biosynthesis
UMAG_01692...
2-Oxobutanoate
Threonine
Methylglyoxal
Propanoate metabolism
UMAG_11142
O-Phospho-L-homoserine
Propane-1,3-diamine
Arginine and proline metabolism
UMAG_10985
Cysteine and methionine metabolism
Homoserine
K13745
Ectoine biosynthesis
UMAG_01708
L-2,4-Diamino-butanoate
Alanine, aspartate and glutamate metabolism
L-Aspartate
UMAG_00872
UMAG_04899
K00836
K06718
K06720
L-Ectoine
K10674
L-4-Aspartyl-phosphate
L-Aspartate 4-semialdehyde
Nγ-Acetyl-L-2,4-diaminobutyrate
5-Hydroxyectoine
K15786
K15785
K15784
K15783
Nα-Acetyl-L-2,4-diaminobutyrate
Lysine biosynthesis
00260 10/26/17
(c) Kanehisa Laboratories

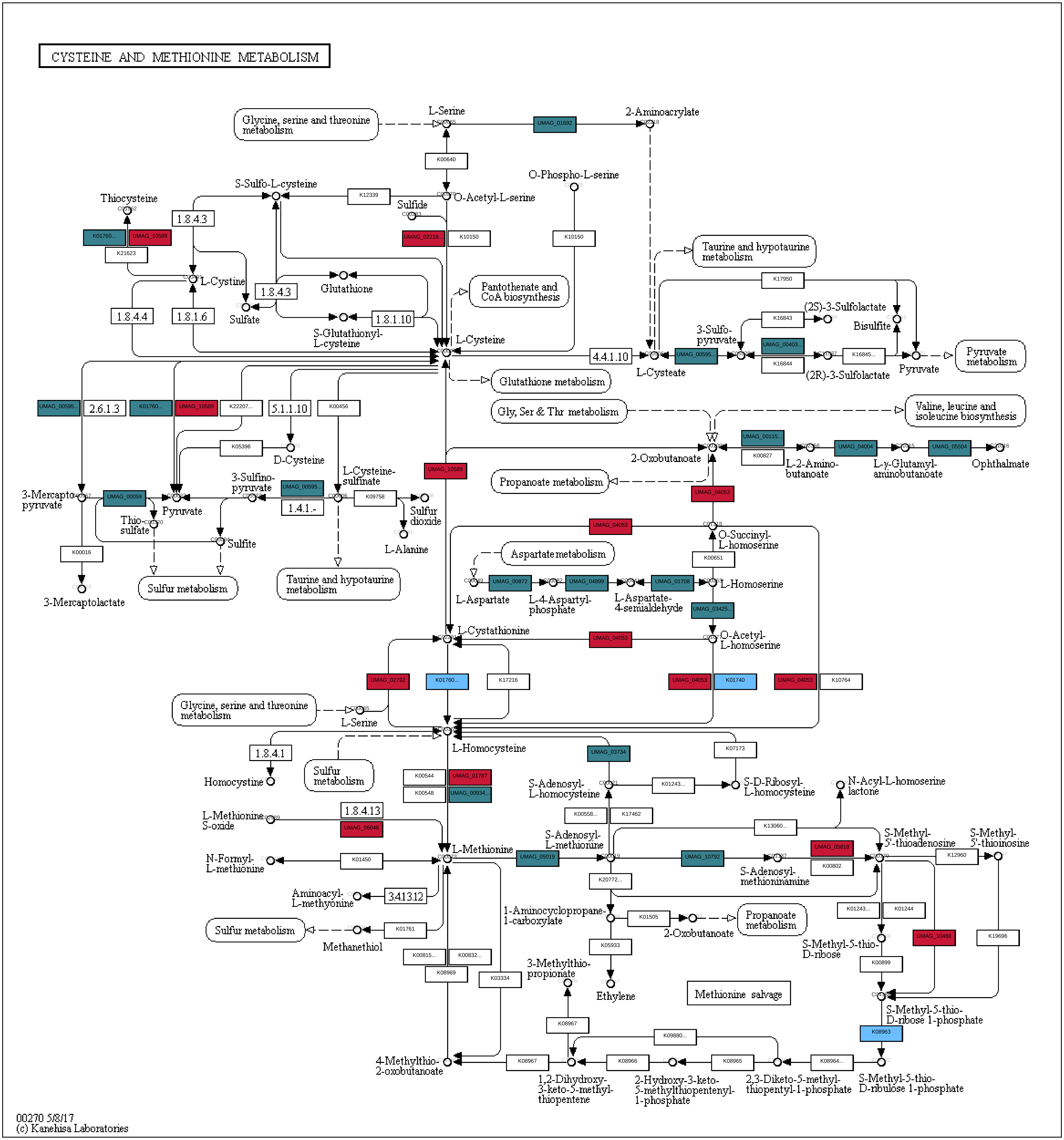
CYSTEINE AND METHIONINE METABOLISM
Glycine, serine and threonine metabolism
L-Serine
UMAG_01692
2-Aminoacrylate
K00640
O-Phospho-L-serine
S-Sulfo-L-cysteine
K12339
Thiocysteine
1.8.4.3
Sulfide
O-Acetyl-L-serine
K01760...
UMAG_10589
UMAG_02218...
K10150
K10150
K21623
Taurine and hypotaurine metabolism
L-Cystine
1.8.4.3
Glutathione
Pantothenate and CoA biosynthesis
K17950
Sulfate
1.8.4.4
1.8.1.6
S-Glutathionyl-L-cysteine
1.8.1.10
L-Cysteine
(2S)-3-Sulfolactate
K16843
Bisulfite
3-Sulfo-pyruvate
UMAG_00403...
4.4.1.10
UMAG_00595...
K16845...
K16844
Pyruvate
Pyruvate metabolism
L-Cysteate
(2R)-3-Sulfolactate
Glutathione metabolism
UMAG_00595...
2.6.1.3
K01760...
UMAG_10589
K22207...
5.1.1.10
K00456
Gly, Ser & Thr metabolism
Valine, leucine and isoleucine biosynthesis
K05396
D-Cysteine
UMAG_00115...
UMAG_04004
UMAG_05504
K00827
2-Oxobutanoate
L-2-Amino-butanoate
L-γ-Glutamyl-aminobutanoate
Ophthalmate
UMAG_10589
Propanoate metabolism
3-Sulfino-pyruvate
UMAG_00595...
L-Cysteine-sulfinate
UMAG_04053
3-Mercapto-pyruvate
UMAG_00059
Pyruvate
K09758
1.4.1.-
Sulfur dioxide
Thio-sulfate
Sulfite
L-Alanine
UMAG_04053
O-Succinyl-L-homoserine
K00016
K00651
Aspartate metabolism
Sulfur metabolism
Taurine and hypotaurine metabolism
UMAG_00872
UMAG_04899
UMAG_01708
L-Homoserine
3-Mercaptolactate
L-Aspartate
L-4-Aspartyl-phosphate
L-Aspartate-4-semialdehyde
UMAG_03425...
L-Cystathionine
UMAG_04053
O-Acetyl-L-homoserine
UMAG_02792
K01760...
K17216
UMAG_04053
K01740
UMAG_04053
K10764
Glycine, serine and threonine metabolism
L-Serine
L-Homocysteine
UMAG_03734
K07173
1.8.4.1
Sulfur metabolism
Homocystine
K00544
UMAG_01787
K00548
UMAG_00934...
S-Adenosyl-L-homocysteine
K01243...
S-D-Ribosyl-L-homocysteine
N-Acyl-L-homoserine lactone
L-Methionine S-oxide
1.8.4.13
UMAG_06048
K00558...
K17462
K13060...
S-Adenosyl-L-methionine
S-Methyl-5'-thioadenosine
S-Methyl-5'-thioinosine
N-Formyl-L-methionine
K01450
L-Methionine
UMAG_05019
UMAG_10792
UMAG_05818
K12960
K00802
S-Adenosyl-methioninamine
K20772...
Aminoacyl-L-methyonine
3.4.13.12
1-Aminocyclopropane-1-carboxylate
K01505
Propanoate metabolism
K01243...
K01244
Sulfur metabolism
K01761
2-Oxobutanoate
S-Methyl-5-thio-D-ribose
UMAG_10466
K19696
Methanethiol
K05933
K00899
K00815...
K00832...
3-Methylthio-propionate
K08969
K03334
Ethylene
Methionine salvage
S-Methyl-5-thio-D-ribose 1-phosphate
K08967
K08963
K09880...
4-Methylthio-2-oxobutanoate
K08967
K08966
K08965
K08964...
1,2-Dihydroxy-3-keto-5-methyl-thiopentene
2-Hydroxy-3-keto-5-methylthiopentenyl-1-phosphate
2,3-Diketo-5-methyl-thiopentyl-1-phosphate
S-Methyl-5-thio-D-ribulose 1-phosphate
00270 5/8/17
(c) Kanehisa Laboratories

# VALINE, LEUCINE AND ISOLEUCINE DEGRADATION

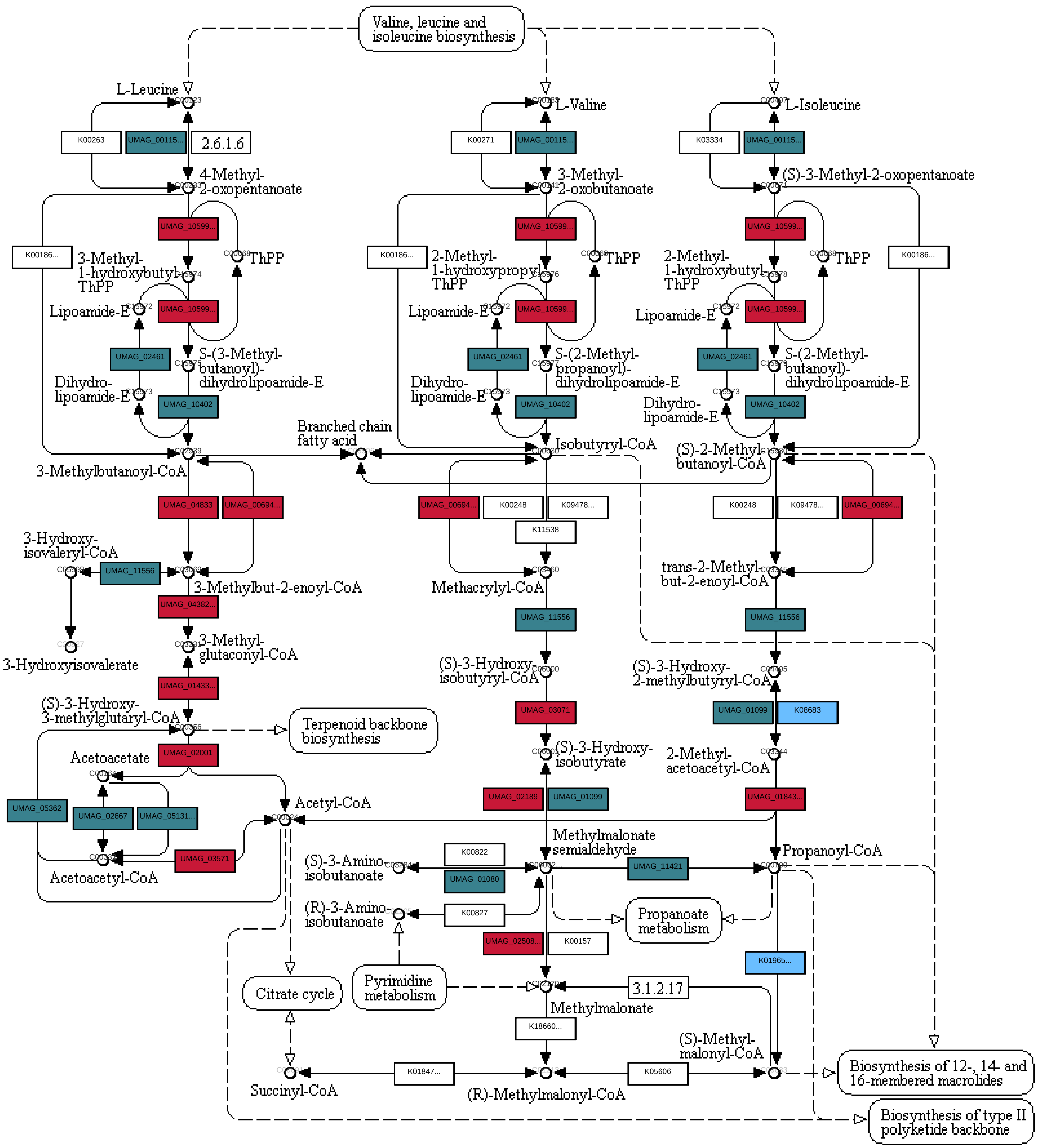


00280 1/27/16

(c) Kanehisa Laboratories

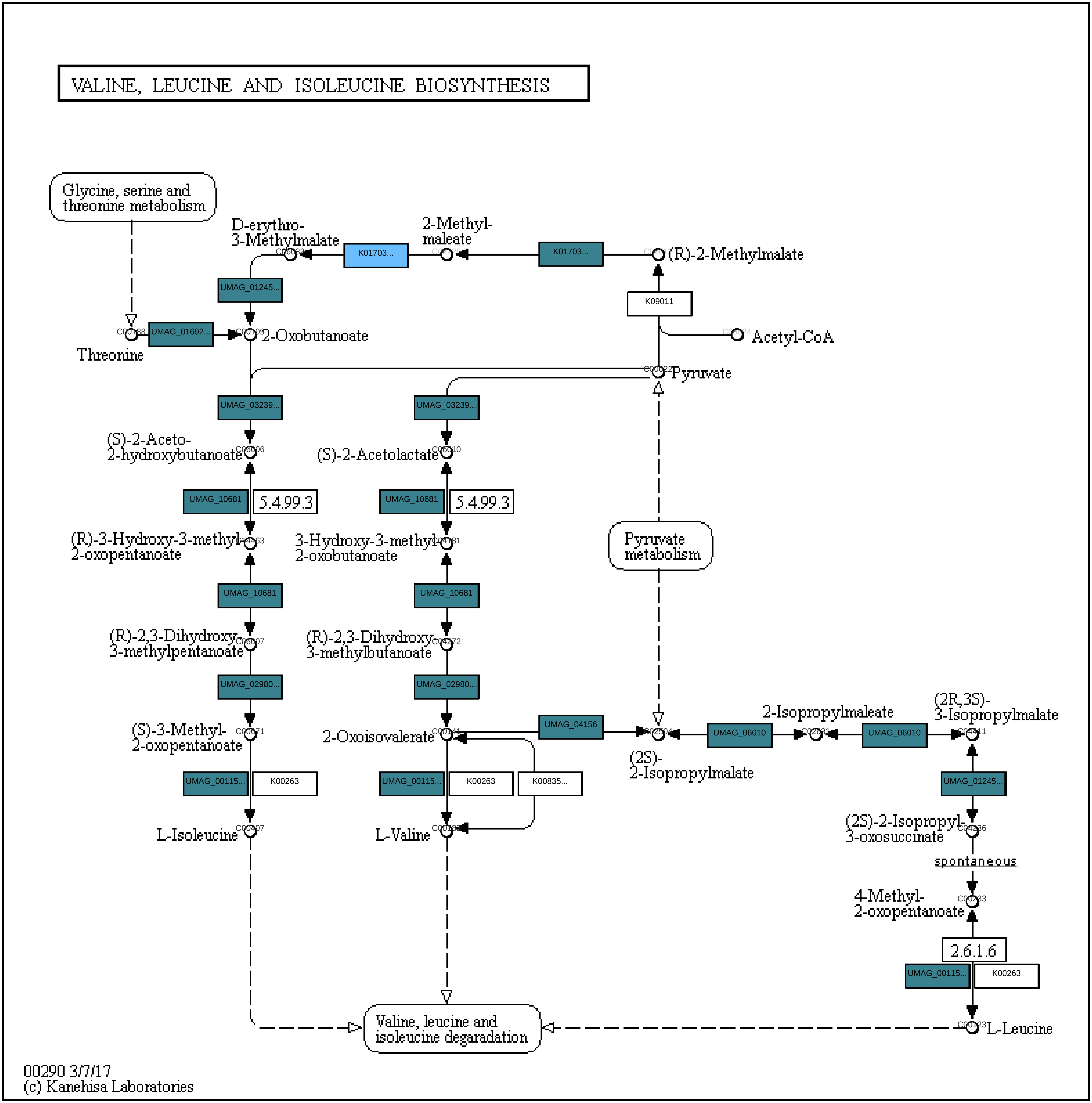

VALINE, LEUCINE AND ISOLEUCINE BIOSYNTHESIS
Glycine, serine and threonine metabolism
D-erythro-3-Methylmalate
K01703...
2-Methyl-maleate
K01703...
(R)-2-Methylmalate
K09011
Acetyl-CoA
UMAG_01245...
Threonine
UMAG_01692...
2-Oxobutanoate
Pyruvate
UMAG_03239...
UMAG_03239...
(S)-2-Aceto-2-hydroxybutanoate
(S)-2-Acetolactate
UMAG_10681
5.4.99.3
UMAG_10681
5.4.99.3
(R)-3-Hydroxy-3-methyl-2-oxopentanoate
3-Hydroxy-3-methyl-2-oxobutanoate
Pyruvate metabolism
UMAG_10681
UMAG_10681
(R)-2,3-Dihydroxy-3-methylpentanoate
(R)-2,3-Dihydroxy-3-methylbutanoate
UMAG_02980...
UMAG_02980...
(S)-3-Methyl-2-oxopentanoate
2-Oxoisovalerate
UMAG_04156
(2S)-2-Isopropylmalate
UMAG_06010
2-Isopropylmaleate
UMAG_06010
(2R,3S)-3-Isopropylmalate
UMAG_00115...
K00263
UMAG_00115...
K00263
K00835...
UMAG_01245...
L-Isoleucine
L-Valine
(2S)-2-Isopropyl-3-oxosuccinate
spontaneous
4-Methyl-2-oxopentanoate
2.6.1.6
UMAG_00115...
K00263
Valine, leucine and isoleucine degaradation
L-Leucine
00290 3/7/17
(c) Kanehisa Laboratories

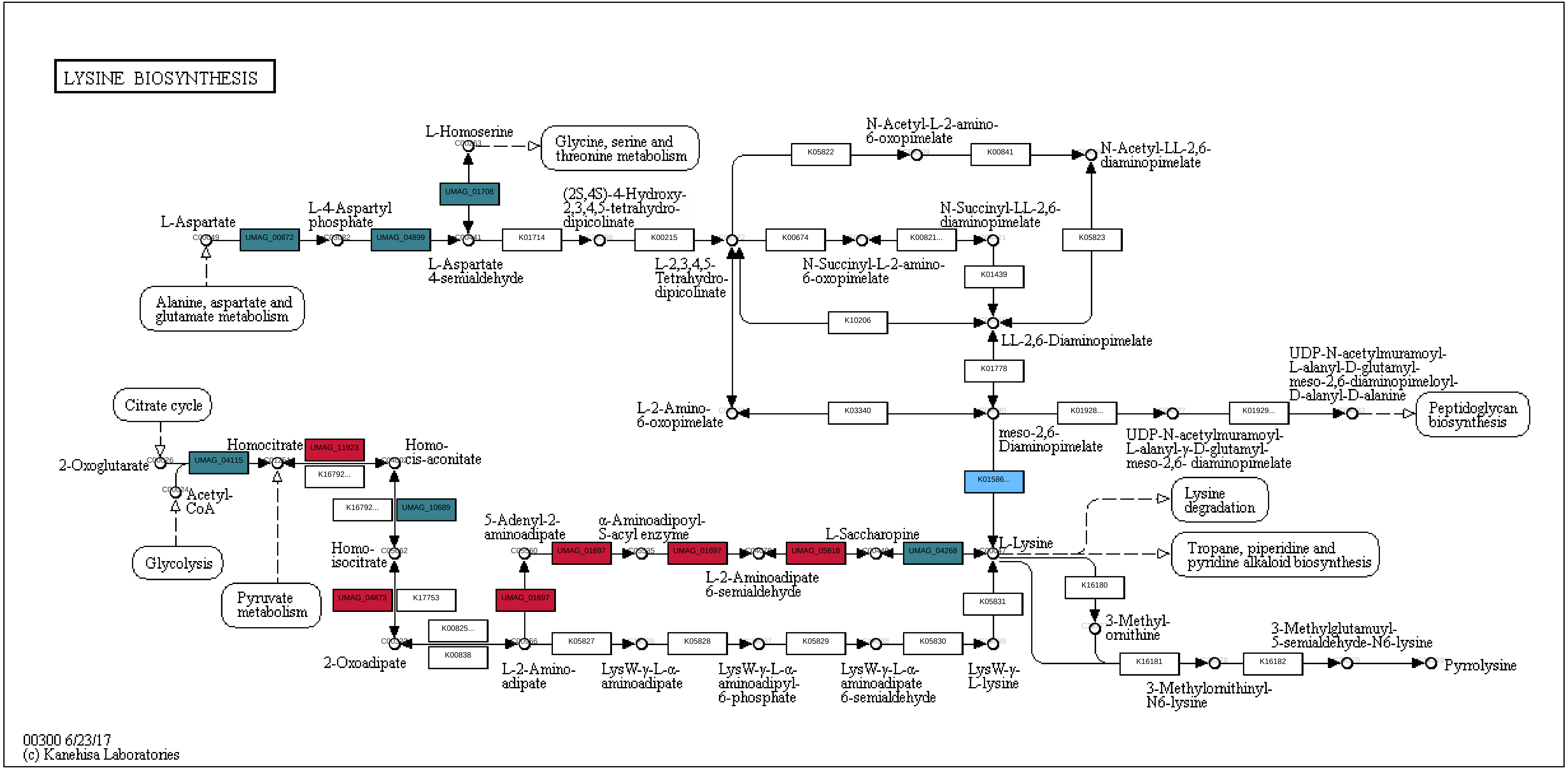
LYSINE BIOSYNTHESIS
L-Homoserine
Glycine, serine and threonine metabolism
UMAG_01708
L-Aspartate
L-4-Aspartyl phosphate
UMAG_00872
UMAG_04899
K01714
(2S,4S)-4-Hydroxy-2,3,4,5-tetrahydro-dipicolinate
K00215
L-Aspartate 4-semialdehyde
L-2,3,4,5-Tetrahydro-dipicolinate
Alanine, aspartate and glutamate metabolism
N-Acetyl-L-2-amino-6-oxopimelate
K05822
K00841
N-Acetyl-LL-2,6-diaminopimelate
N-Succinyl-LL-2,6-diaminopimelate
K00674
K00821...
K05823
N-Succinyl-L-2-amino-6-oxopimelate
K01439
K10206
LL-2,6-Diaminopimelate
K01778
L-2-Amino-6-oxopimelate
K03340
meso-2,6-Diaminopimelate
K01928...
K01929...
UDP-N-acetylmuramoyl-L-alanyl-D-glutamyl-meso-2,6-diaminopimeloyl-D-alanyl-D-alanine
Peptidoglycan biosynthesis
UDP-N-acetylmuramoyl-L-alanyl-γ-D-glutamyl-meso-2,6- diaminopimelate
K01586...
Citrate cycle
2-Oxoglutarate
UMAG_04115
Homocitrate
UMAG_11923
K16792...
Homo-cis-aconitate
Acetyl-CoA
K16792...
UMAG_10689
Glycolysis
Pyruvate metabolism
Homo-isocitrate
UMAG_04873
K17753
K00825...
K00838
2-Oxoadipate
5-Adenyl-2-aminoadipate
UMAG_01697
α-Aminoadipoyl-S-acyl enzyme
UMAG_01697
L-Saccharopine
UMAG_05818
UMAG_04268
L-Lysine
Lysine degradation
Tropane, piperidine and pyridine alkaloid biosynthesis
L-2-Aminoadipate 6-semialdehyde
UMAG_01697
K05827
K05828
K05829
K05830
K05831
K16180
3-Methyl-ornithine
L-2-Amino-adipate
LysW-γ-L-α-aminoadipate
LysW-γ-L-α-aminoadipyl-6-phosphate
LysW-γ-L-α-aminoadipate 6-semialdehyde
LysW-γ-L-lysine
K16181
K16182
3-Methylglutamuyl-5-semialdehyde-N6-lysine
Pyrrolysine
3-Methylornithinyl-N6-lysine
00300 6/23/17
(c) Kanehisa Laboratories

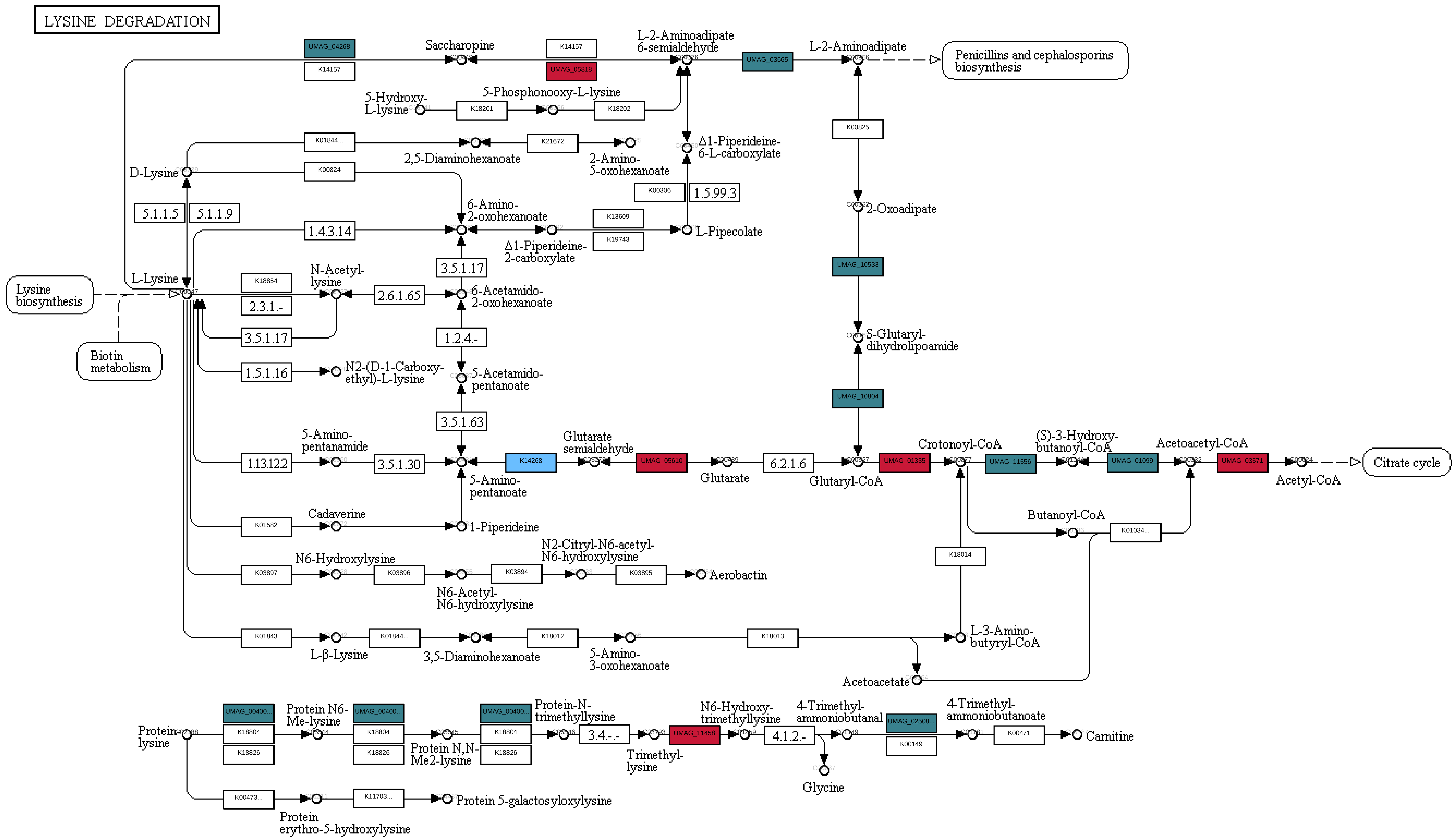

00310 7/19/17
(c) Kanehisa Laboratories

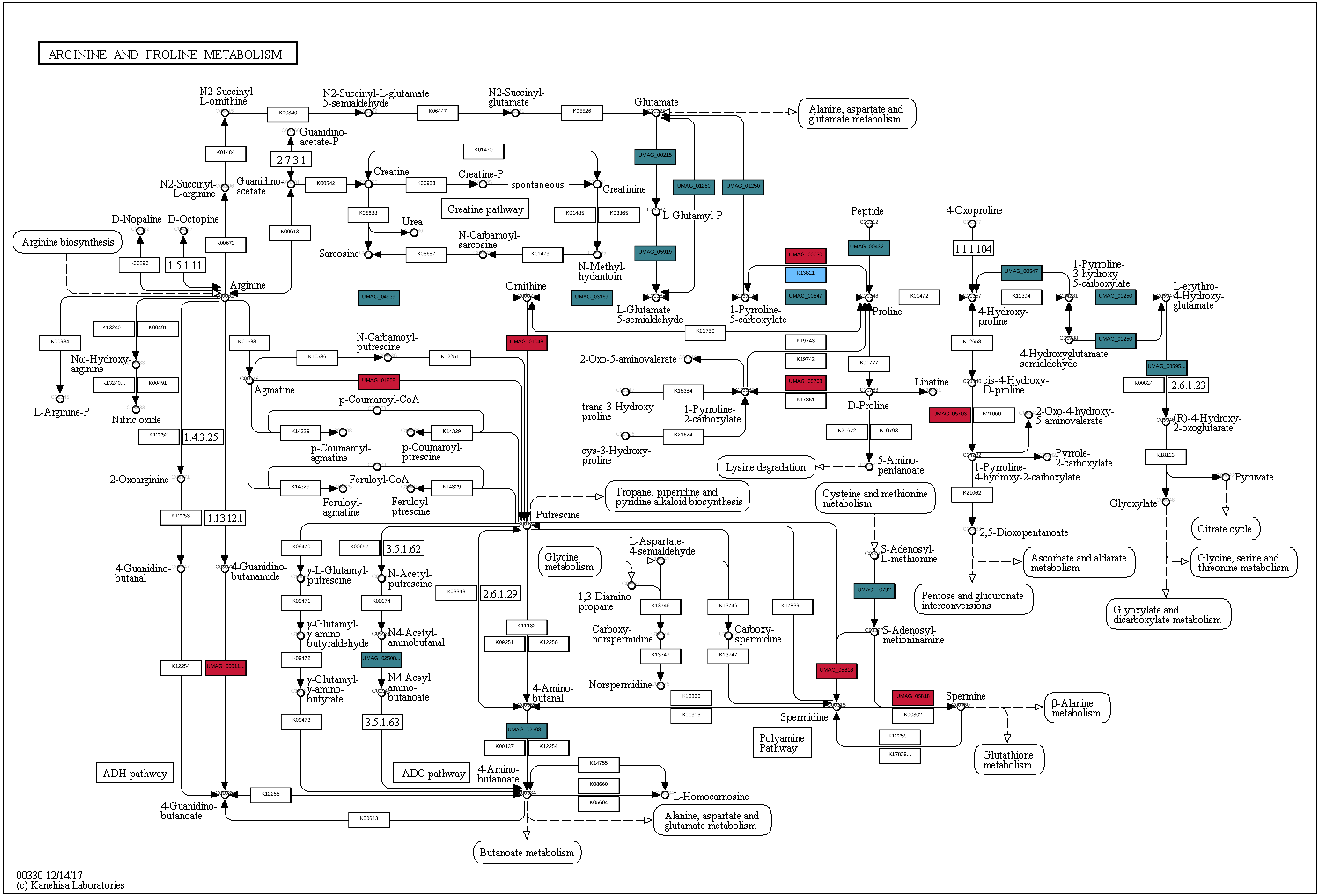

ARGININE AND PROLINE METABOLISM
N2-Succinyl-L-ornithine
N2-Succinyl-L-glutamate 5-semialdehyde
N2-Succinyl-glutamate
Glutamate
Alanine, aspartate and glutamate metabolism
K00840
K06447
K05526
Guanidino-acetate-P
2.7.3.1
K01484
K01470
Creatine
Creatine-P
spontaneous
Creatinine
N2-Succinyl-L-arginine
Guanidino-acetate
K00542
K00933
Creatine pathway
K01485
K03365
UMAG_00215
UMAG_01250
UMAG_01250
L-Glutamyl-P
K08688
Urea
D-Nopaline
D-Octopine
K00673
K00613
N-Carbamoyl-sarcosine
Peptide
4-Oxoproline
Arginine biosynthesis
Sarcosine
K08687
K01473...
UMAG_05919
UMAG_00030
UMAG_00432...
1.1.1.104
K00296
1.5.1.11
N-Methyl-hydantoin
K13821
UMAG_00547
1-Pyrroline-3-hydroxy-5-carboxylate
Arginine
UMAG_04939
Ornithine
UMAG_03169
UMAG_00547
K00472
K11394
UMAG_01250
L-erythro-4-Hydroxy-glutamate
L-Glutamate 5-semialdehyde
1-Pyrroline-5-carboxylate
Proline
4-Hydroxy-proline
K13240...
K00491
K00934
K01583...
K01750
UMAG_01048
K19743
K12658
UMAG_01250
N-Carbamoyl-putrescine
K10536
K12251
2-Oxo-5-aminovalerate
K19742
K01777
4-Hydroxyglutamate semialdehyde
Nω-Hydroxy-arginine
UMAG_00595...
Agmatine
UMAG_01858
UMAG_05703
Linatine
cis-4-Hydroxy-D-proline
K13240...
K00491
K18384
D-Proline
K00824
2.6.1.23
L-Arginine-P
p-Coumaroyl-CoA
trans-3-Hydroxy-proline
1-Pyrroline-2-carboxylate
K17851
UMAG_05703
K21060...
2-Oxo-4-hydroxy-5-aminovalerate
(R)-4-Hydroxy-2-oxoglutarate
Nitric oxide
K14329
K14329
K21624
K21672
K10793...
K12252
1.4.3.25
p-Coumaroyl-agmatine
p-Coumaroyl-ptrescine
cys-3-Hydroxy-proline
Pyrrole-2-carboxylate
1-Pyrroline-4-hydroxy-2-carboxylate
K18123
Lysine degradation
5-Amino-pentanoate
Pyruvate
Feruloyl-CoA
2-Oxoarginine
K14329
K14329
Feruloyl-agmatine
Feruloyl-ptrescine
Tropane, piperidine and pyridine alkaloid biosynthesis
Cysteine and methionine metabolism
K21062
Glyoxylate
Citrate cycle
Putrescine
K12253
1.13.12.1
2,5-Dioxopentanoate
K09470
K00657
3.5.1.62
L-Aspartate-4-semialdehyde
Glycine metabolism
S-Adenosyl-L-methionine
Ascorbate and aldarate metabolism
Glycine, serine and threonine metabolism
4-Guanidino-butanal
4-Guanidino-butanamide
γ-L-Glutamyl-putrescine
N-Acetyl-putrescine
K03343
2.6.1.29
1,3-Diamino-propane
UMAG_10792
Pentose and glucuronate interconversions
Glyoxylate and dicarboxylate metabolism
K09471
K00274
K13746
K13746
K17839...
γ-Glutamyl-γ-amino-butyraldehyde
N4-Acetyl-aminobutanal
K11182
Carboxy-norspermidine
Carboxy-spermidine
S-Adenosyl-methioninamine
K09251
K12256
K12254
UMAG_00011...
K09472
UMAG_02508...
K13747
K13747
UMAG_05818
γ-Glutamyl-γ-amino-butyrate
N4-Acetyl-amino-butanoate
4-Amino-butanal
Norspermidine
K13366
UMAG_05818
Spermine
β-Alanine metabolism
K09473
3.5.1.63
K00316
K00802
Spermidine
UMAG_02508...
K12259...
K00137
K12254
Polyamine Pathway
K17839...
Glutathione metabolism
ADH pathway
ADC pathway
4-Amino-butanoate
K14755
K08660
L-Homocarnosine
4-Guanidino-butanoate
K12255
K05604
Alanine, aspartate and glutamate metabolism
K00613
Butanoate metabolism
00330 12/14/17
(c) Kanehisa Laboratories

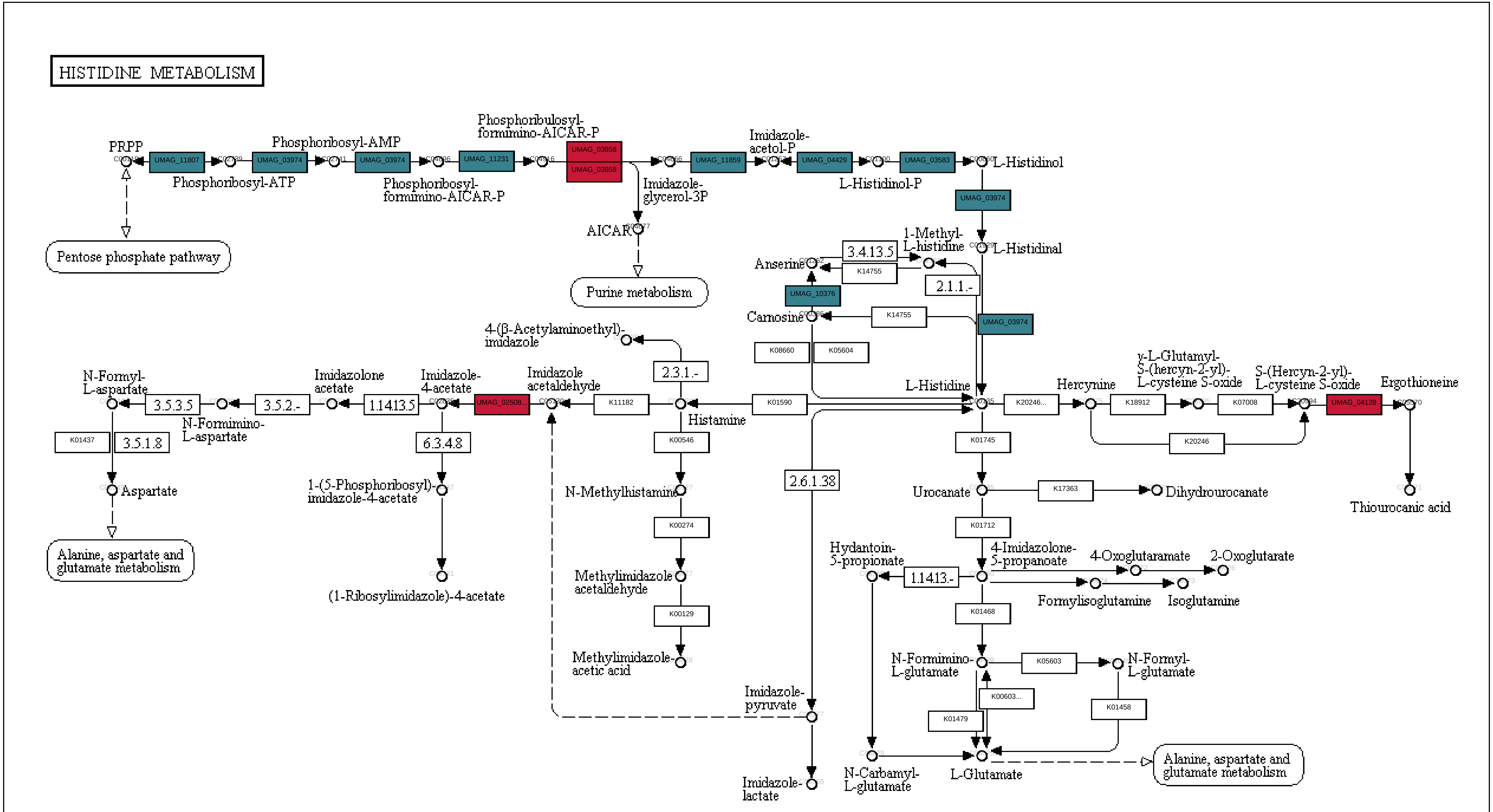


00340 12/7/17
(c) Kanehisa Laboratories

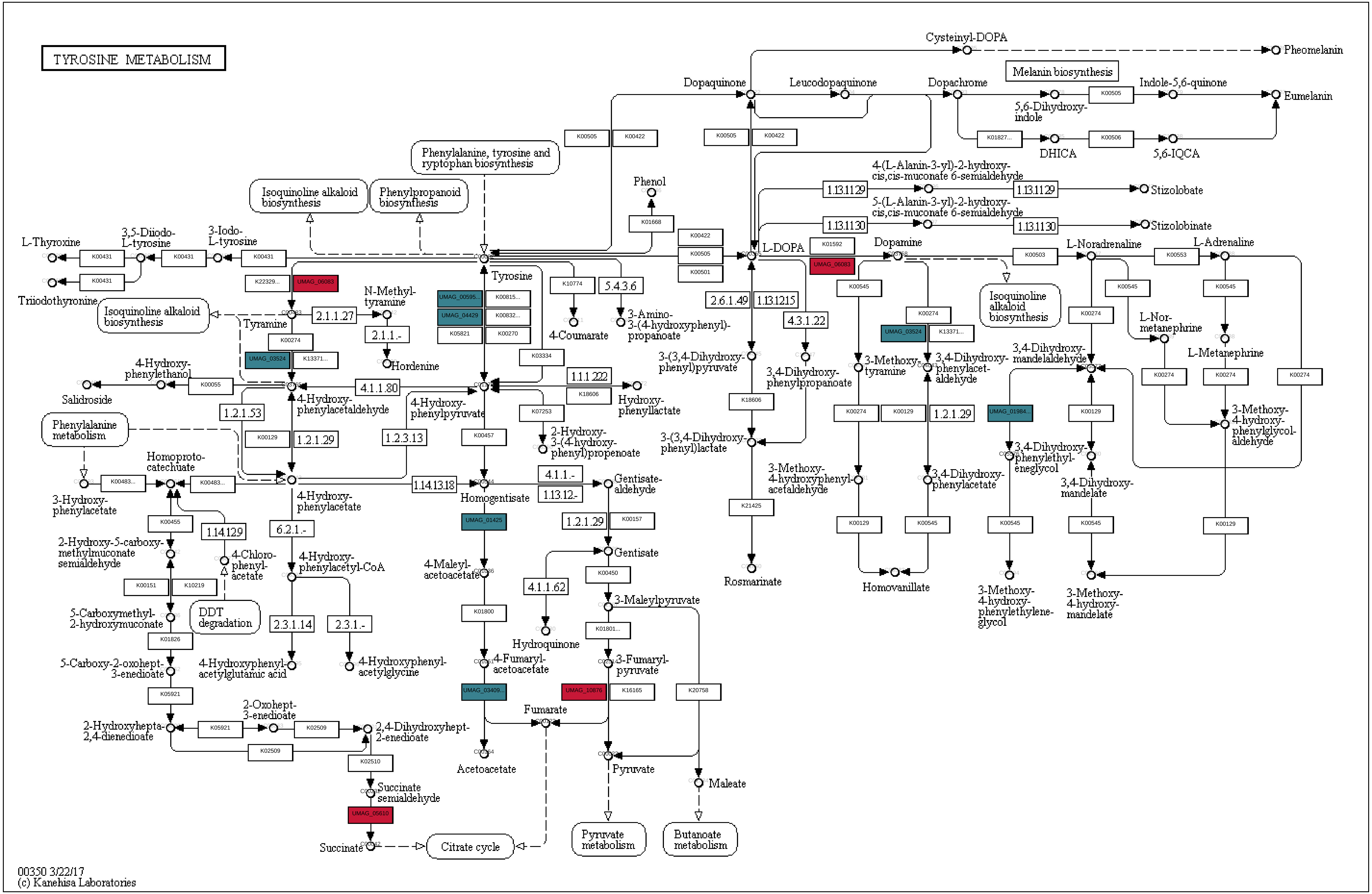
TYROSINE METABOLISM
Cysteinyl-DOPA
Pheomelanin
Melanin biosynthesis
Dopaquinone
Leucodopaquinone
Dopachrome
Indole-5,6-quinone
Eumelanin
5,6-Dihydroxy-indole
DHICA
5,6-IQCA
K00505
K00422
K01827...
K00506
Phenylalanine, tyrosine and tryptophan biosynthesis
Isoquinoline alkaloid biosynthesis
Phenylpropanoid biosynthesis
Phenol
K01668
4-(L-Alanin-3-yl)-2-hydroxy-cis,cis-muconate 6-semialdehyde
5-(L-Alanin-3-yl)-2-hydroxy-cis,cis-muconate 6-semialdehyde
1.13.11.29
1.13.11.30
Stizolobate
Stizolobinate
L-Thyroxine
3,5-Diiodo-L-tyrosine
3-Iodo-L-tyrosine
K00431
Triiodothyronine
K22329...
UMAG_06083
Tyrosine
K00501
L-DOPA
K01592
Dopamine
K00503
L-Noradrenaline
K00553
L-Adrenaline
N-Methyl-tyramine
2.1.1.27
2.1.1.-
Hordenine
Tyramine
K00274
UMAG_03524
K13371...
UMAG_00595...
K00815...
UMAG_04429
K00832...
K05821
K00270
K10774
5.4.3.6
4-Coumarate
3-Amino-3-(4-hydroxyphenyl)-propanoate
2.6.1.49
1.13.12.15
4.3.1.22
K00545
L-Nor-metanephrine
L-Metanephrine
3,4-Dihydroxy-mandelaldehyde
4-Hydroxy-phenylethanol
K00055
Salidroside
K03334
3-(3,4-Dihydroxy-phenyl)pyruvate
3,4-Dihydroxy-phenylpropanoate
3-Methoxy-tyramine
3,4-Dihydroxy-phenylacet-aldehyde
4.1.1.80
4-Hydroxy-phenylacetaldehyde
4-Hydroxy-phenylpyruvate
1.1.1.222
K18606
Hydroxy-phenyllactate
1.2.1.53
K00129
1.2.1.29
1.2.3.13
K00457
K07253
2-Hydroxy-3-(4-hydroxy-phenyl)propenoate
3-(3,4-Dihydroxy-phenyl)lactate
UMAG_01984...
3-Methoxy-4-hydroxy-phenylglycol-aldehyde
3,4-Dihydroxy-phenylethyl-eneglycol
Phenylalanine metabolism
Homoproto-catechuate
K00483...
3-Hydroxy-phenylacetate
1.14.13.18
4.1.1.-
1.13.12.-
Gentisate-aldehyde
Homogentisate
4-Hydroxy-phenylacetate
3-Methoxy-4-hydroxyphenyl-acetaldehyde
3,4-Dihydroxy-phenylacetate
3,4-Dihydroxy-mandelate
K00455
1.14.12.9
6.2.1.-
UMAG_01425
K00157
K21425
2-Hydroxy-5-carboxy-methylmuconate semialdehyde
4-Chloro-phenyl-acetate
4-Hydroxy-phenylacetyl-CoA
4-Maleyl-acetoacetate
Gentisate
Rosmarinate
Homovanillate
K00151
K10219
K00450
4.1.1.62
3-Methoxy-4-hydroxy-phenylethylene-glycol
3-Methoxy-4-hydroxy-mandelate
5-Carboxymethyl-2-hydroxymuconate
DDT degradation
2.3.1.14
2.3.1.-
K01800
3-Maleylpyruvate
Hydroquinone
K01826
K01801...
5-Carboxy-2-oxohept-3-enedioate
4-Hydroxyphenyl-acetylglutamic acid
4-Hydroxyphenyl-acetylglycine
4-Fumaryl-acetoacetate
3-Fumaryl-pyruvate
K05921
UMAG_03409...
UMAG_10876
K16165
K20758
Fumarate
2-Oxohept-3-enedioate
2,4-Dihydroxyhept-2-enedioate
2-Hydroxyhepta-2,4-dienedioate
K02509
K02510
Acetoacetate
Pyruvate
Maleate
Succinate semialdehyde
UMAG_05610
Succinate
Citrate cycle
Pyruvate metabolism
Butanoate metabolism
00350 3/22/17
(c) Kanehisa Laboratories

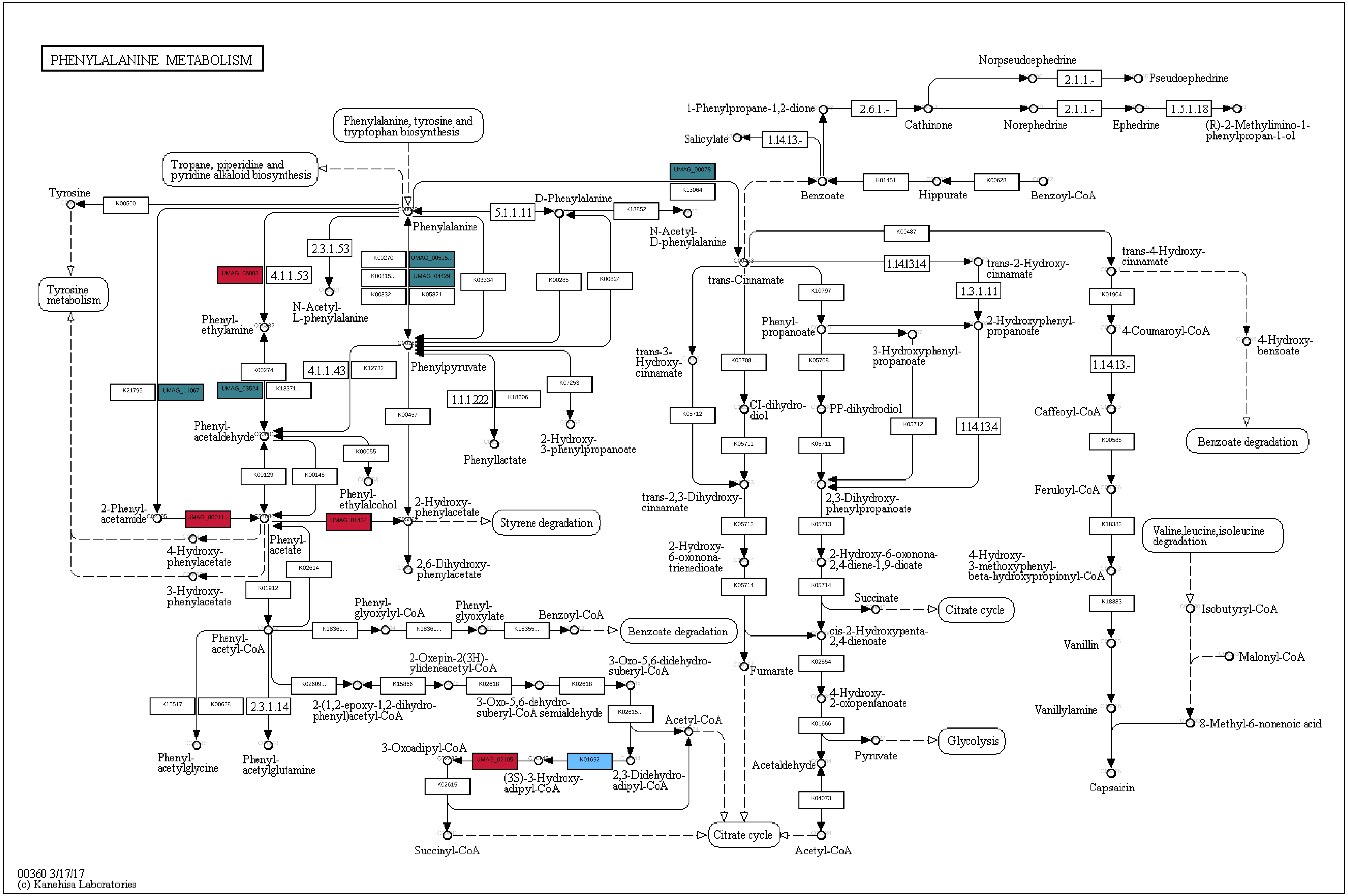
PHENYLALANINE METABOLISM
Phenylalanine, tyrosine and tryptophan biosynthesis
Tropane, piperidine and pyridine alkaloid biosynthesis
Tyrosine
K00500
Tyrosine metabolism
Phenylalanine
5.1.1.11
D-Phenylalanine
K18852
N-Acetyl-D-phenylalanine
2.3.1.53
N-Acetyl-L-phenylalanine
UMAG_06083
4.1.1.53
K00270
UMAG_00595...
K00815...
UMAG_04429
K00832...
K05821
K03334
K00285
K00824
Phenyl-ethylamine
K00274
UMAG_03524
K13371...
4.1.1.43
K12732
Phenylpyruvate
K21795
UMAG_11067
1.1.1.222
K18606
K07253
K00457
Phenyl-acetaldehyde
K00055
Phenyllactate
2-Hydroxy-3-phenylpropanoate
K00129
K00146
Phenyl-ethylalcohol
2-Phenyl-acetamide
UMAG_00011...
UMAG_01424
2-Hydroxy-phenylacetate
Styrene degradation
4-Hydroxy-phenylacetate
Phenyl-acetate
2,6-Dihydroxy-phenylacetate
K02614
3-Hydroxy-phenylacetate
K01912
Phenyl-glyoxylyl-CoA
Phenyl-glyoxylate
Benzoyl-CoA
Phenyl-acetyl-CoA
K18361...
K18361...
K18355...
Benzoate degradation
2-Oxepin-2(3H)-ylideneacetyl-CoA
3-Oxo-5,6-didehydro-suberyl-CoA
K02609...
K15866
K02618
K02618
K15517
K00628
2.3.1.14
2-(1,2-epoxy-1,2-dihydro-phenyl)acetyl-CoA
3-Oxo-5,6-dehydro-suberyl-CoA semialdehyde
K02615...
Acetyl-CoA
Phenyl-acetylglycine
Phenyl-acetylglutamine
3-Oxoadipyl-CoA
UMAG_02105
K01692
(3S)-3-Hydroxy-adipyl-CoA
2,3-Didehydro-adipyl-CoA
K02615
Succinyl-CoA
Citrate cycle
UMAG_00078
K13064
1-Phenylpropane-1,2-dione
2.6.1.-
Cathinone
Norpseudoephedrine
2.1.1.-
Pseudoephedrine
Norephedrine
2.1.1.-
Ephedrine
1.5.1.18
(R)-2-Methylimino-1-phenylpropan-1-ol
Salicylate
1.14.13.-
Benzoate
K01451
Hippurate
K00628
Benzoyl-CoA
K00487
trans-Cinnamate
1.14.13.14
trans-2-Hydroxy-cinnamate
trans-4-Hydroxy-cinnamate
K10797
1.3.1.11
Phenyl-propanoate
2-Hydroxyphenyl-propanoate
3-Hydroxyphenyl-propanoate
K01904
4-Coumaroyl-CoA
4-Hydroxy-benzoate
trans-3-Hydroxy-cinnamate
K05708...
K05708...
1.14.13.-
CI-dihydro-diol
PP-dihydrodiol
K05712
K05712
1.14.13.4
Caffeoyl-CoA
K00588
Benzoate degradation
K05711
K05711
trans-2,3-Dihydroxy-cinnamate
2,3-Dihydroxy-phenylpropanoate
Feruloyl-CoA
K05713
K05713
K18383
Valine, leucine, isoleucine degradation
2-Hydroxy-6-oxonona-trienedioate
2-Hydroxy-6-oxonona-2,4-diene-1,9-dioate
4-Hydroxy-3-methoxyphenyl-beta-hydroxypropionyl-CoA
K05714
K05714
Succinate
Citrate cycle
K18383
Isobutyryl-CoA
cis-2-Hydroxypenta-2,4-dienoate
Vanillin
Malonyl-CoA
Fumarate
K02554
4-Hydroxy-2-oxopentanoate
Vanillylamine
8-Methyl-6-nonenoic acid
K01666
Pyruvate
Glycolysis
Acetaldehyde
Capsaicin
K04073
Acetyl-CoA
00360 3/17/17
(c) Kanehisa Laboratories

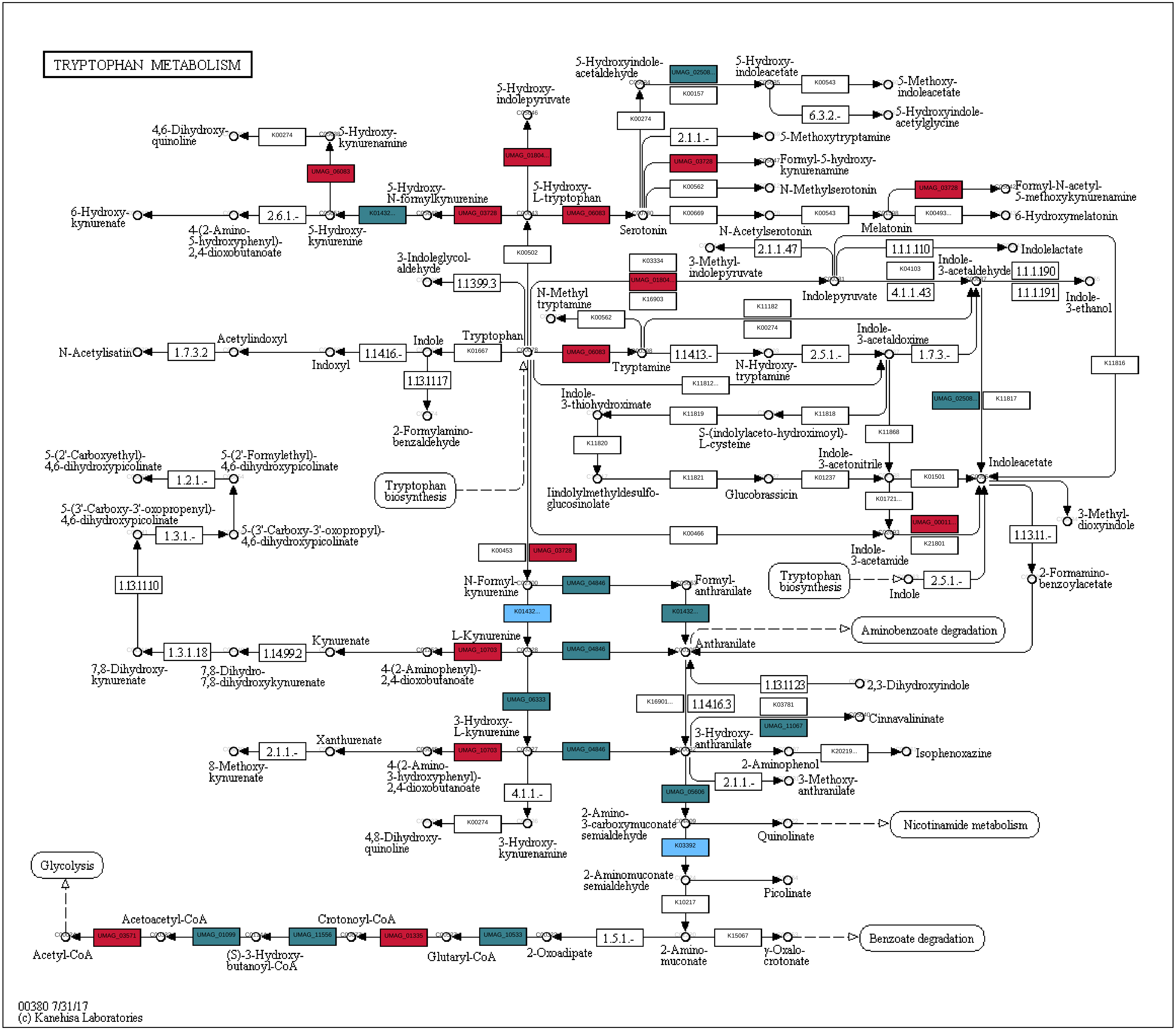
TRYPTOPHAN METABOLISM
5-Hydroxyindole-acetaldehyde
UMAG_02508...
K00157
5-Hydroxy-indoleacetate
K00543
5-Methoxy-indoleacetate
6.3.2.-
5-Hydroxyindole-acetylglycine
5-Hydroxy-indolepyruvate
K00274
2.1.1.-
5-Methoxytryptamine
4,6-Dihydroxy-quinoline
K00274
5-Hydroxy-kynurenamine
UMAG_01804...
UMAG_03728
Formyl-5-hydroxy-kynurenamine
UMAG_06083
K00562
N-Methylserotonin
UMAG_03728
Formyl-N-acetyl-5-methoxykynurenamine
5-Hydroxy-N-formylkynurenine
5-Hydroxy-L-tryptophan
6-Hydroxy-kynurenate
2.6.1.-
K01432...
UMAG_03728
UMAG_06083
K00669
K00543
K00493...
6-Hydroxymelatonin
4-(2-Amino-5-hydroxyphenyl)-2,4-dioxobutanoate
5-Hydroxy-kynurenine
Serotonin
N-Acetylserotonin
Melatonin
K00502
2.1.1.47
1.1.1.110
Indolelactate
3-Indoleglycol-aldehyde
K03334
3-Methyl-indolepyruvate
Indole-3-acetaldehyde
K04103
1.1.1.190
1.13.99.3
UMAG_01804...
Indolepyruvate
4.1.1.43
1.1.1.191
Indole-3-ethanol
N-Methyl tryptamine
K16903
K11182
K00562
K00274
Indole-3-acetaldoxime
Acetylindoxyl
Indole
Tryptophan
N-Acetylisatin
1.7.3.2
1.14.16.-
K01667
UMAG_06083
1.14.13.-
2.5.1.-
1.7.3.-
K11816
Indoxyl
1.13.11.17
Tryptamine
N-Hydroxy-tryptamine
K11812...
Indole-3-thiohydroximate
UMAG_02508...
K11817
K11819
K11818
2-Formylamino-benzaldehyde
S-(indolylaceto-hydroximoyl)-L-cysteine
K11868
K11820
5-(2'-Carboxyethyl)-4,6-dihydroxypicolinate
5-(2'-Formylethyl)-4,6-dihydroxypicolinate
Indole-3-acetonitrile
Indoleacetate
1.2.1.-
Tryptophan biosynthesis
K11821
K01237
K01501
Indolylmethyldesulfo-glucosinolate
Glucobrassicin
K01721...
5-(3'-Carboxy-3'-oxopropenyl)-4,6-dihydroxypicolinate
3-Methyl-dioxyindole
1.3.1.-
5-(3'-Carboxy-3'-oxopropyl)-4,6-dihydroxypicolinate
K00466
UMAG_00011...
K21801
1.13.11.-
K00453
UMAG_03728
Indole-3-acetamide
1.13.11.10
Tryptophan biosynthesis
2.5.1.-
N-Formyl-kynurenine
UMAG_04846
Formyl-anthranilate
Indole
2-Formamino-benzoylacetate
K01432...
K01432...
Aminobenzoate degradation
Kynurenate
L-Kynurenine
Anthranilate
1.3.1.18
1.14.99.2
UMAG_10703
UMAG_04846
7,8-Dihydroxy-kynurenate
7,8-Dihydro-7,8-dihydroxykynurenate
4-(2-Aminophenyl)-2,4-dioxobutanoate
1.13.11.23
2,3-Dihydroxyindole
K16901...
1.14.16.3
UMAG_06333
K03781
Cinnavalininate
UMAG_11067
3-Hydroxy-L-kynurenine
3-Hydroxy-anthranilate
Xanthurenate
2.1.1.-
UMAG_10703
UMAG_04846
K20219...
Isophenoxazine
8-Methoxy-kynurenate
2-Aminophenol
4-(2-Amino-3-hydroxyphenyl)-2,4-dioxobutanoate
2.1.1.-
3-Methoxy-anthranilate
4.1.1.-
UMAG_05606
2-Amino-3-carboxymuconate semialdehyde
K00274
Quinolinate
Nicotinamide metabolism
4,8-Dihydroxy-quinoline
3-Hydroxy-kynurenamine
K03392
Glycolysis
2-Aminomuconate semialdehyde
Picolinate
K10217
Acetoacetyl-CoA
Crotonoyl-CoA
UMAG_03571
UMAG_01099
UMAG_11556
UMAG_01335
UMAG_10533
1.5.1.-
K15067
Benzoate degradation
Acetyl-CoA
(S)-3-Hydroxy-butanoyl-CoA
Glutaryl-CoA
2-Oxoadipate
2-Amino-muconate
γ-Oxalo-crotonate
00380 7/31/17
(c) Kanehisa Laboratories

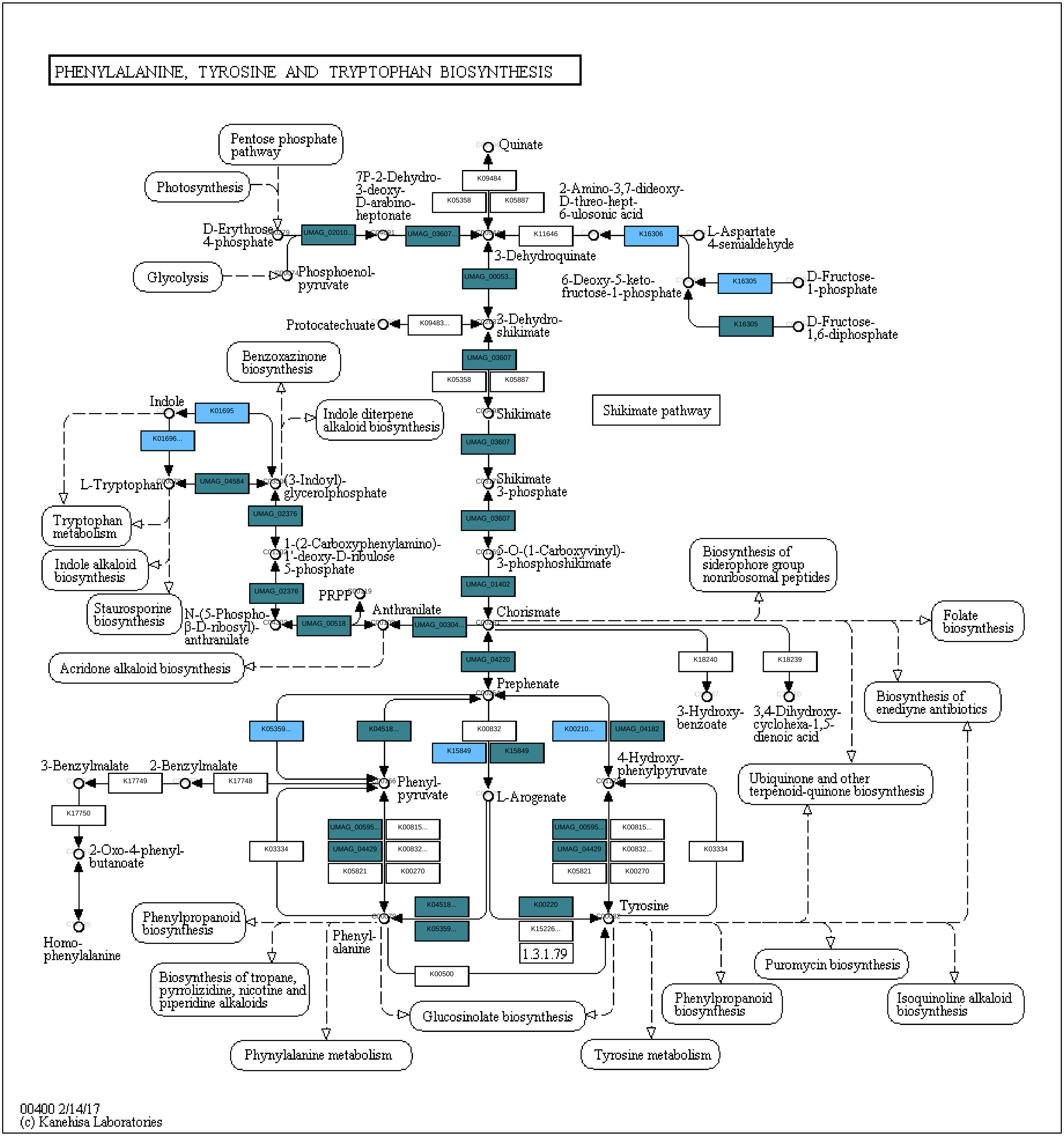
PHENYLALANINE, TYROSINE AND TRYPTOPHAN BIOSYNTHESIS
Pentose phosphate pathway
Photosynthesis
Glycolysis
D-Erythrose 4-phosphate
Phosphoenol-pyruvate
7P-2-Dehydro-3-deoxy-D-arabino-heptonate
Quinate
K09484
K05358
K05887
2-Amino-3,7-dideoxy-D-threo-hept-6-ulosonic acid
L-Aspartate 4-semialdehyde
UMAG_02010...
UMAG_03607...
K11646
K16306
3-Dehydroquinate
UMAG_00053...
6-Deoxy-5-keto-fructose-1-phosphate
K16305
D-Fructose-1-phosphate
D-Fructose-1,6-diphosphate
Protocatechuate
K09483...
3-Dehydro-shikimate
UMAG_03607
Benzoxazinone biosynthesis
Indole diterpene alkaloid biosynthesis
Shikimate
Shikimate pathway
Indole
K01695
K01696...
L-Tryptophan
UMAG_04584
(3-Indoyl)-glycerolphosphate
Shikimate 3-phosphate
Tryptophan metabolism
UMAG_02376
1-(2-Carboxyphenylamino)-1'-deoxy-D-ribulose 5-phosphate
5-O-(1-Carboxyvinyl)-3-phosphoshikimate
Indole alkaloid biosynthesis
Biosynthesis of siderophore group nonribosomal peptides
UMAG_01402
PRPP
Staurosporine biosynthesis
N-(5-Phospho-β-D-ribosyl)-anthranilate
UMAG_00518
Anthranilate
UMAG_00304...
Chorismate
Folate biosynthesis
Acridone alkaloid biosynthesis
UMAG_04220
K18240
K18239
Prephenate
3-Hydroxy-benzoate
3,4-Dihydroxy-cyclohexa-1,5-dienoic acid
Biosynthesis of enediyne antibiotics
K05359...
K04518...
K00832
K00210...
UMAG_04182
K15849
3-Benzylmalate
2-Benzylmalate
K17749
K17748
Phenyl-pyruvate
4-Hydroxy-phenylpyruvate
L-Arogenate
Ubiquinone and other terpenoid-quinone biosynthesis
K17750
K03334
UMAG_00595...
K00815...
UMAG_04429
K00832...
K05821
K00270
2-Oxo-4-phenyl-butanoate
K00220
Tyrosine
K15226...
1.3.1.79
Homo-phenylalanine
Phenylpropanoid biosynthesis
Phenyl-alanine
K00500
Puromycin biosynthesis
Biosynthesis of tropane, pyrrolizidine, nicotine and piperidine alkaloids
Glucosinolate biosynthesis
Isoquinoline alkaloid biosynthesis
Phynylalanine metabolism
Tyrosine metabolism
00400 2/14/17
(c) Kanehisa Laboratories

# 6. Metabolism of other amino acids

| MAP | PATHWAY |
|---|---|
| **410** | beta-Alanine metabolism |
| **430** | Taurine and hypotaurine metabolism |
| **440** | Phosphonate and phosphinate metabolism |
| **450** | Selenocompound metabolism |
| **460** | Cyanoamino acid metabolism |
| **480** | Glutathione metabolism |

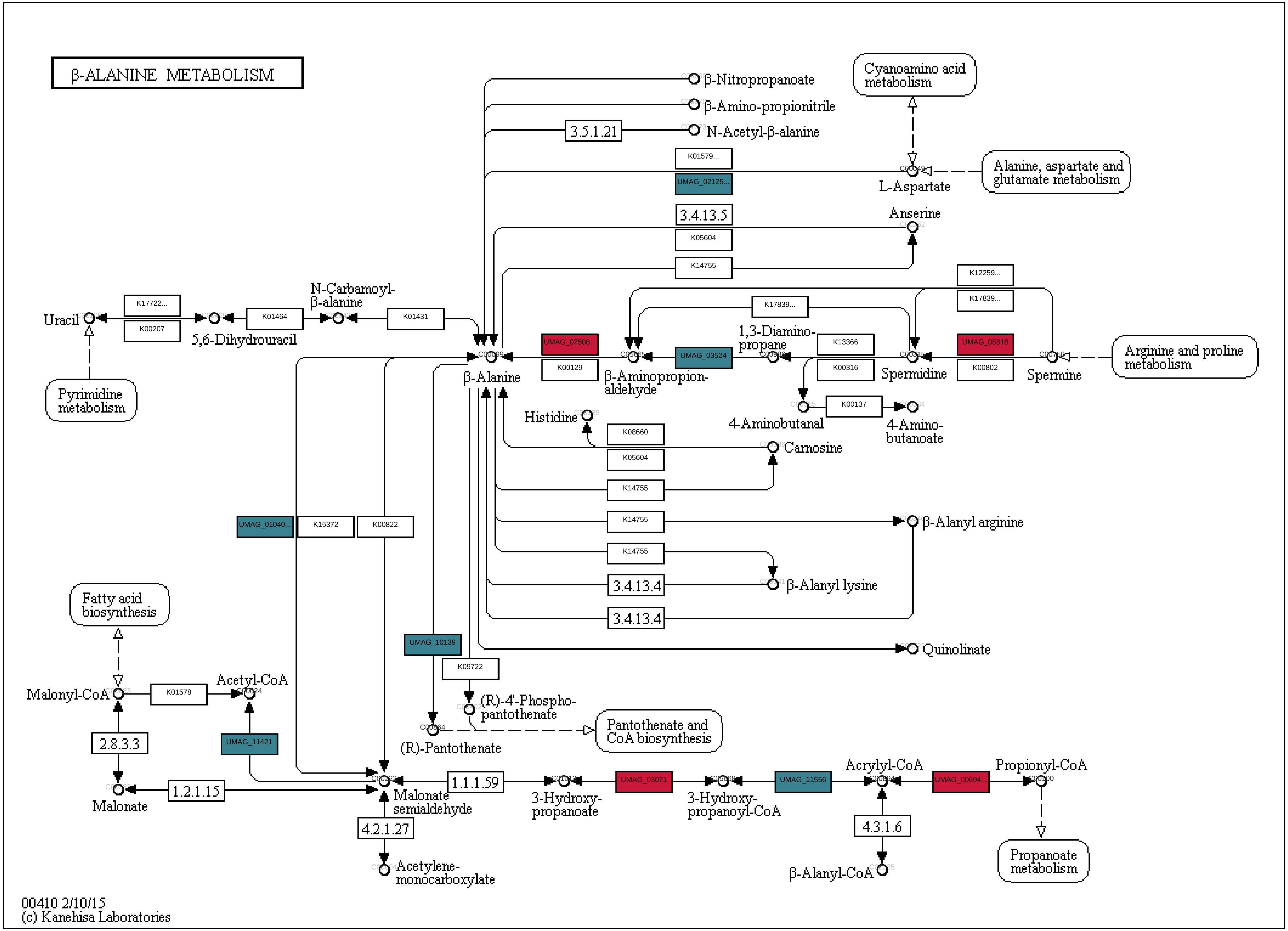
β-ALANINE METABOLISM
β-Nitropropanoate
β-Amino-propionitrile
3.5.1.21
N-Acetyl-β-alanine
Cyanoamino acid metabolism
K01579...
UMAG_02125...
L-Aspartate
Alanine, aspartate and glutamate metabolism
3.4.13.5
K05604
K14755
Anserine
K12259...
K17839...
K17839...
Uracil
K17722...
K00207
5,6-Dihydrouracil
K01464
N-Carbamoyl-β-alanine
K01431
UMAG_02508...
K00129
β-Alanine
β-Aminopropion-aldehyde
UMAG_03524
1,3-Diamino-propane
K13366
K00316
Spermidine
UMAG_05818
K00802
Spermine
Arginine and proline metabolism
Pyrimidine metabolism
Histidine
K08660
K05604
4-Aminobutanal
K00137
4-Amino-butanoate
Carnosine
K14755
K14755
β-Alanyl arginine
UMAG_01040...
K15372
K00822
K14755
β-Alanyl lysine
3.4.13.4
3.4.13.4
Quinolinate
Fatty acid biosynthesis
UMAG_10139
K09722
Malonyl-CoA
K01578
Acetyl-CoA
(R)-4'-Phospho-pantothenate
Pantothenate and CoA biosynthesis
(R)-Pantothenate
2.8.3.3
UMAG_11421
Malonate
1.2.1.15
1.1.1.59
Malonate semialdehyde
3-Hydroxy-propanoate
UMAG_03071
3-Hydroxy-propanoyl-CoA
UMAG_11556
Acrylyl-CoA
UMAG_00694...
Propionyl-CoA
4.2.1.27
Acetylene-monocarboxylate
4.3.1.6
β-Alanyl-CoA
Propanoate metabolism
00410 2/10/15
(c) Kanehisa Laboratories

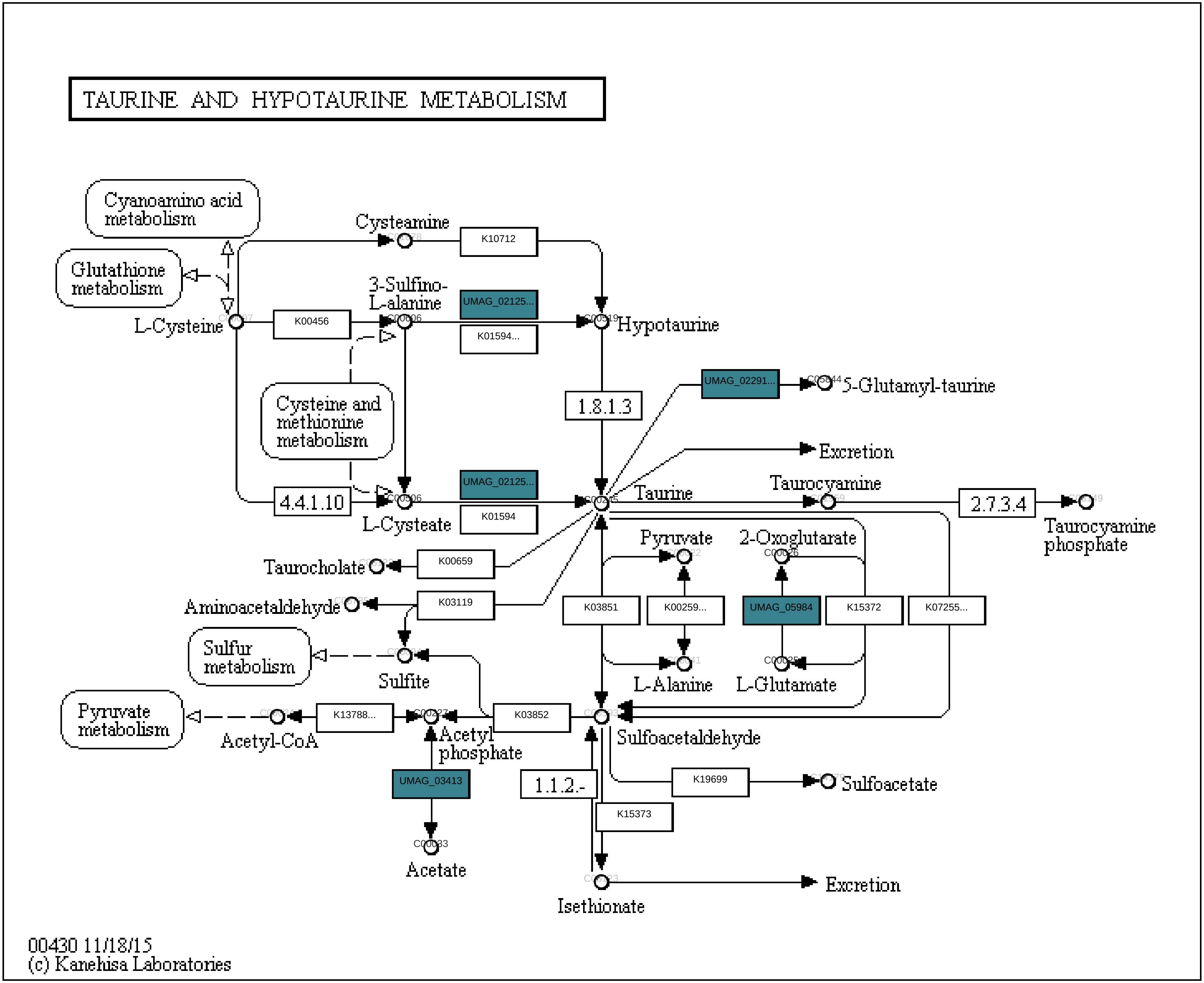
TAURINE AND HYPOTAURINE METABOLISM
Cyanoamino acid metabolism
Glutathione metabolism
L-Cysteine
Cysteamine
K10712
3-Sulfino-L-alanine
K00456
UMAG_02125...
K01594...
Hypotaurine
Cysteine and methionine metabolism
1.8.1.3
UMAG_02291...
5-Glutamyl-taurine
Excretion
4.4.1.10
UMAG_02125...
K01594
L-Cysteate
Taurine
Taurocyamine
2.7.3.4
Taurocyamine phosphate
Taurocholate
K00659
Pyruvate
2-Oxoglutarate
Aminoacetaldehyde
K03119
K03851
K00259...
UMAG_05984
K15372
K07255...
Sulfur metabolism
Sulfite
L-Alanine
L-Glutamate
Pyruvate metabolism
K13788...
K03852
Acetyl-CoA
Acetyl phosphate
Sulfoacetaldehyde
UMAG_03413
1.1.2.-
K19699
Sulfoacetate
K15373
C00033
Acetate
Excretion
Isethionate
00430 11/18/15
(c) Kanehisa Laboratories

# PHOSPHONATE AND PHOSPHINATE METABOLISM

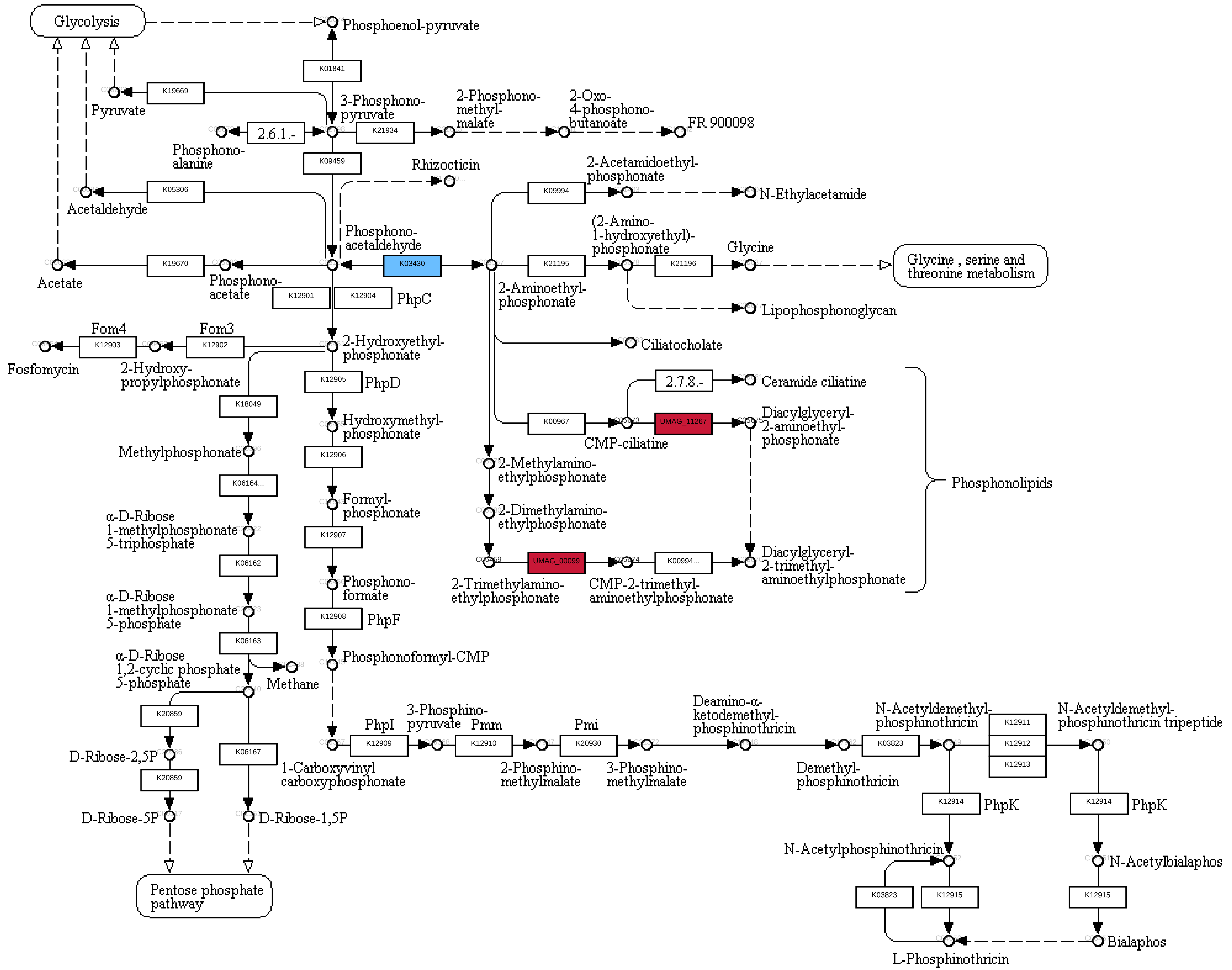


00440 7/24/17

(c) Kanehisa Laboratories

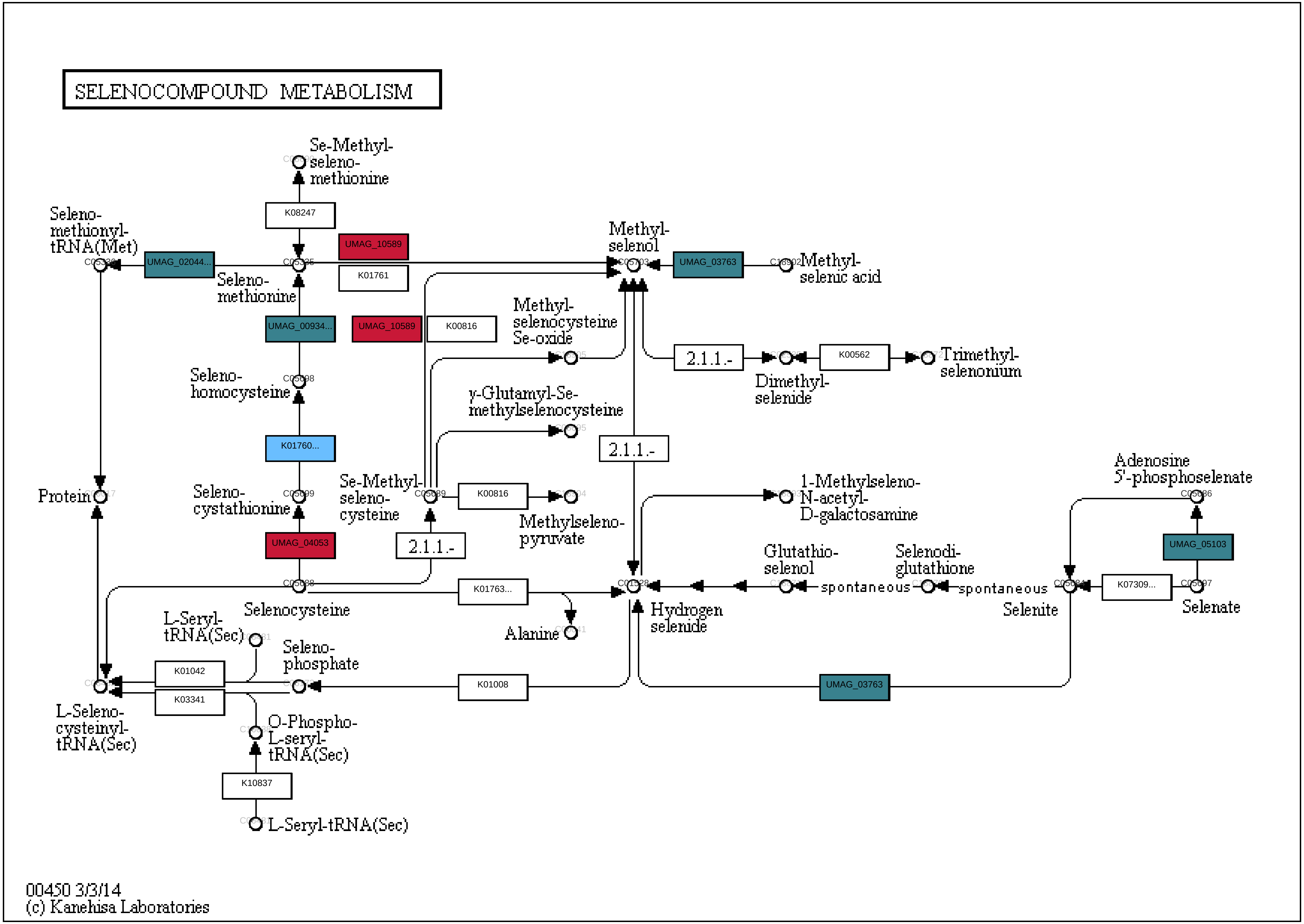
SELENOCOMPOUND METABOLISM
Se-Methyl-seleno-methionine
K08247
Seleno-methionyl-tRNA(Met)
C05336
UMAG_02044...
C05335
UMAG_10589
K01761
Seleno-methionine
Methyl-selenol
C05703
UMAG_03763
Methyl-selenic acid
UMAG_00934...
UMAG_10589
K00816
Methyl-selenocysteine Se-oxide
2.1.1.-
K00562
Trimethyl-selenonium
Dimethyl-selenide
Seleno-homocysteine
C05698
γ-Glutamyl-Se-methylselenocysteine
K01760...
2.1.1.-
Adenosine 5'-phosphoselenate
C05686
Protein
Seleno-cystathionine
C05699
Se-Methyl-seleno-cysteine
C05689
K00816
Methylseleno-pyruvate
1-Methylseleno-N-acetyl-D-galactosamine
UMAG_04053
2.1.1.-
UMAG_05103
Glutathio-selenol
Selenodi-glutathione
C05688
K01763...
C01528
spontaneous
spontaneous
C05684
K07309...
C05697
Selenocysteine
Hydrogen selenide
Selenite
Selenate
L-Seryl-tRNA(Sec)
Alanine
Seleno-phosphate
K01042
K03341
K01008
UMAG_03763
L-Seleno-cysteinyl-tRNA(Sec)
O-Phospho-L-seryl-tRNA(Sec)
K10837
L-Seryl-tRNA(Sec)
00450 3/3/14
(c) Kanehisa Laboratories

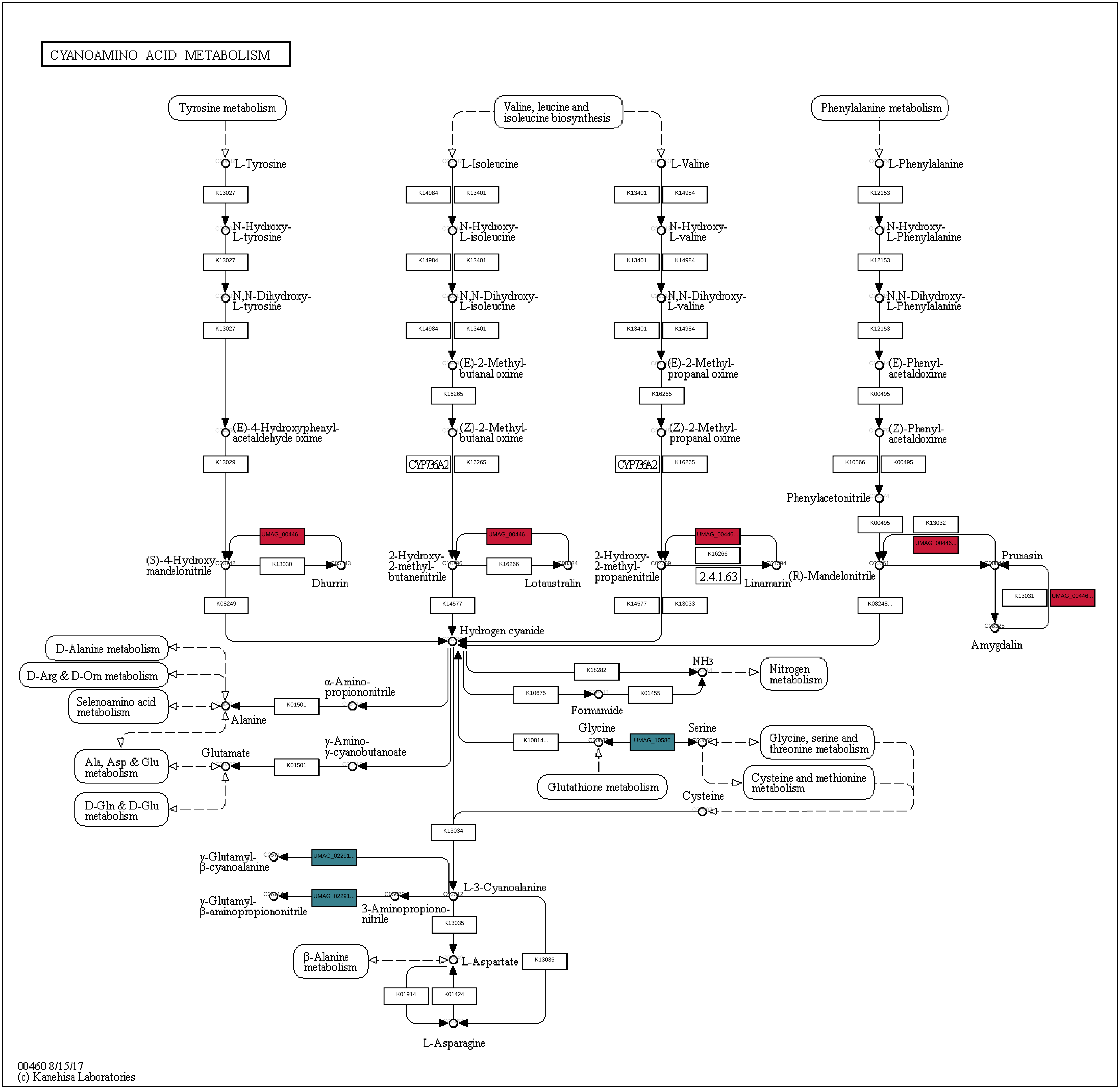
CYANOAMINO ACID METABOLISM
Tyrosine metabolism
Valine, leucine and isoleucine biosynthesis
Phenylalanine metabolism
L-Tyrosine
L-Isoleucine
L-Valine
L-Phenylalanine
K13027
K14984
K13401
K12153
N-Hydroxy-L-tyrosine
N-Hydroxy-L-isoleucine
N-Hydroxy-L-valine
N-Hydroxy-L-Phenylalanine
N,N-Dihydroxy-L-tyrosine
N,N-Dihydroxy-L-isoleucine
N,N-Dihydroxy-L-valine
N,N-Dihydroxy-L-Phenylalanine
(E)-2-Methyl-butanal oxime
(E)-2-Methyl-propanal oxime
(E)-Phenyl-acetaldoxime
K16265
K00495
(E)-4-Hydroxyphenyl-acetaldehyde oxime
(Z)-2-Methyl-butanal oxime
(Z)-2-Methyl-propanal oxime
(Z)-Phenyl-acetaldoxime
K13029
CYP736A2
K10566
Phenylacetonitrile
K13032
UMAG_00446...
K13030
K16266
2.4.1.63
(S)-4-Hydroxy-mandelonitrile
Dhurrin
2-Hydroxy-2-methyl-butanenitrile
Lotaustralin
2-Hydroxy-2-methyl-propanenitrile
Linamarin
(R)-Mandelonitrile
Prunasin
K13031
Amygdalin
K08249
K14577
K13033
K08248...
Hydrogen cyanide
D-Alanine metabolism
D-Arg & D-Orn metabolism
Selenoamino acid metabolism
K18282
NH3
Nitrogen metabolism
K10675
K01455
Formamide
α-Amino-propiononitrile
K01501
Alanine
Glycine
Serine
K10814...
UMAG_10586
Glycine, serine and threonine metabolism
Glutamate
γ-Amino-γ-cyanobutanoate
Ala, Asp & Glu metabolism
D-Gln & D-Glu metabolism
Glutathione metabolism
Cysteine and methionine metabolism
Cysteine
K13034
γ-Glutamyl-β-cyanoalanine
UMAG_02291...
L-3-Cyanoalanine
γ-Glutamyl-β-aminopropiononitrile
3-Aminopropiono-nitrile
K13035
β-Alanine metabolism
L-Aspartate
K01914
K01424
L-Asparagine
00460 8/15/17
(c) Kanehisa Laboratories

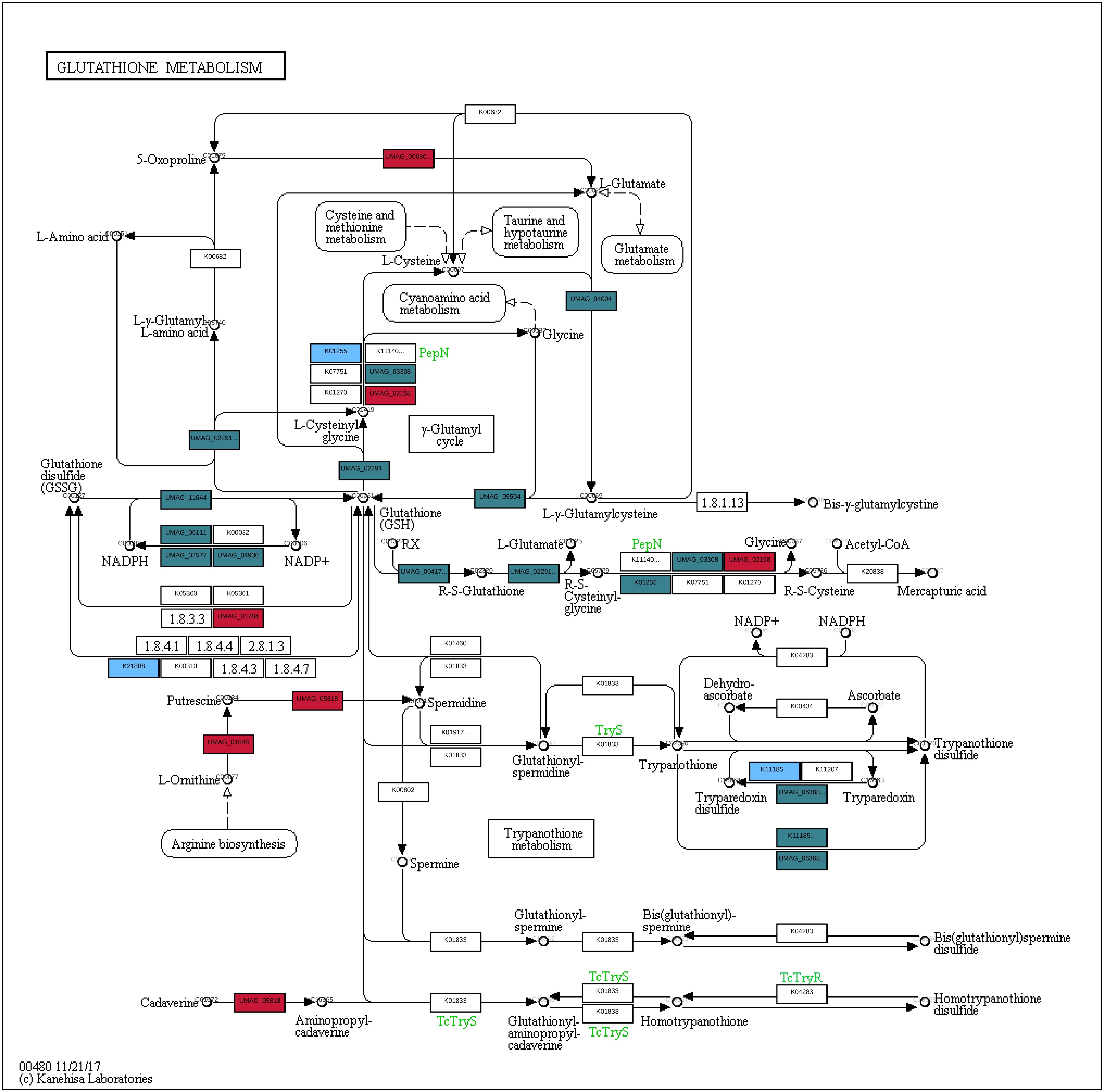
GLUTATHIONE METABOLISM
K00682
5-Oxoproline
UMAG_00080...
L-Glutamate
L-Amino acid
K00682
Cysteine and methionine metabolism
Taurine and hypotaurine metabolism
Glutamate metabolism
L-Cysteine
Cyanoamino acid metabolism
UMAG_04004
L-γ-Glutamyl-L-amino acid
Glycine
K01255
K11140...
PepN
K07751
UMAG_03308
K01270
UMAG_02158
L-Cysteinyl-glycine
γ-Glutamyl cycle
UMAG_02291...
UMAG_02291...
Glutathione disulfide (GSSG)
UMAG_11644
UMAG_05504
Glutathione (GSH)
L-γ-Glutamylcysteine
1.8.1.13
Bis-γ-glutamylcystine
UMAG_06111
K00032
UMAG_02577
UMAG_04930
NADPH
NADP+
RX
L-Glutamate
PepN
Glycine
Acetyl-CoA
K11140...
UMAG_03308
UMAG_02158
UMAG_00417...
UMAG_02291...
K01255
K07751
K01270
K20838
R-S-Glutathione
R-S-Cysteinyl-glycine
R-S-Cysteine
Mercapturic acid
K05360
K05361
1.8.3.3
UMAG_01784
1.8.4.1
1.8.4.4
2.8.1.3
K21888
K00310
1.8.4.3
1.8.4.7
NADP+
NADPH
K01460
K04283
K01833
Putrescine
UMAG_05818
Spermidine
K01833
Dehydro-ascorbate
K00434
Ascorbate
UMAG_01048
K01917...
TryS
K01833
K01833
Trypanothione disulfide
Glutathionyl-spermidine
Trypanothione
K11185...
K11207
L-Ornithine
K00802
UMAG_06368...
Tryparedoxin disulfide
Tryparedoxin
Arginine biosynthesis
Trypanothione metabolism
K11185...
UMAG_06368...
Spermine
Glutathionyl-spermine
Bis(glutathionyl)-spermine
K04283
K01833
K01833
Bis(glutathionyl)spermine disulfide
TcTryS
TcTryR
K01833
K04283
Cadaverine
UMAG_05818
K01833
K01833
Homotrypanothione disulfide
Aminopropyl-cadaverine
TcTryS
Glutathionyl-aminopropyl-cadaverine
TcTryS
Homotrypanothione
00480 11/21/17
(c) Kanehisa Laboratories

# 7. Glycan biosynthesis and metabolism

| MAP | PATHWAY |
|---|---|
| **510** | N-Glycan biosynthesis |
| **513** | Various types of N-glycan biosynthesis |
| **531** | Glycosaminoglycan degradation |
| **563** | Glycosylphosphatidylinositol (GPI)-anchor biosynthesis |

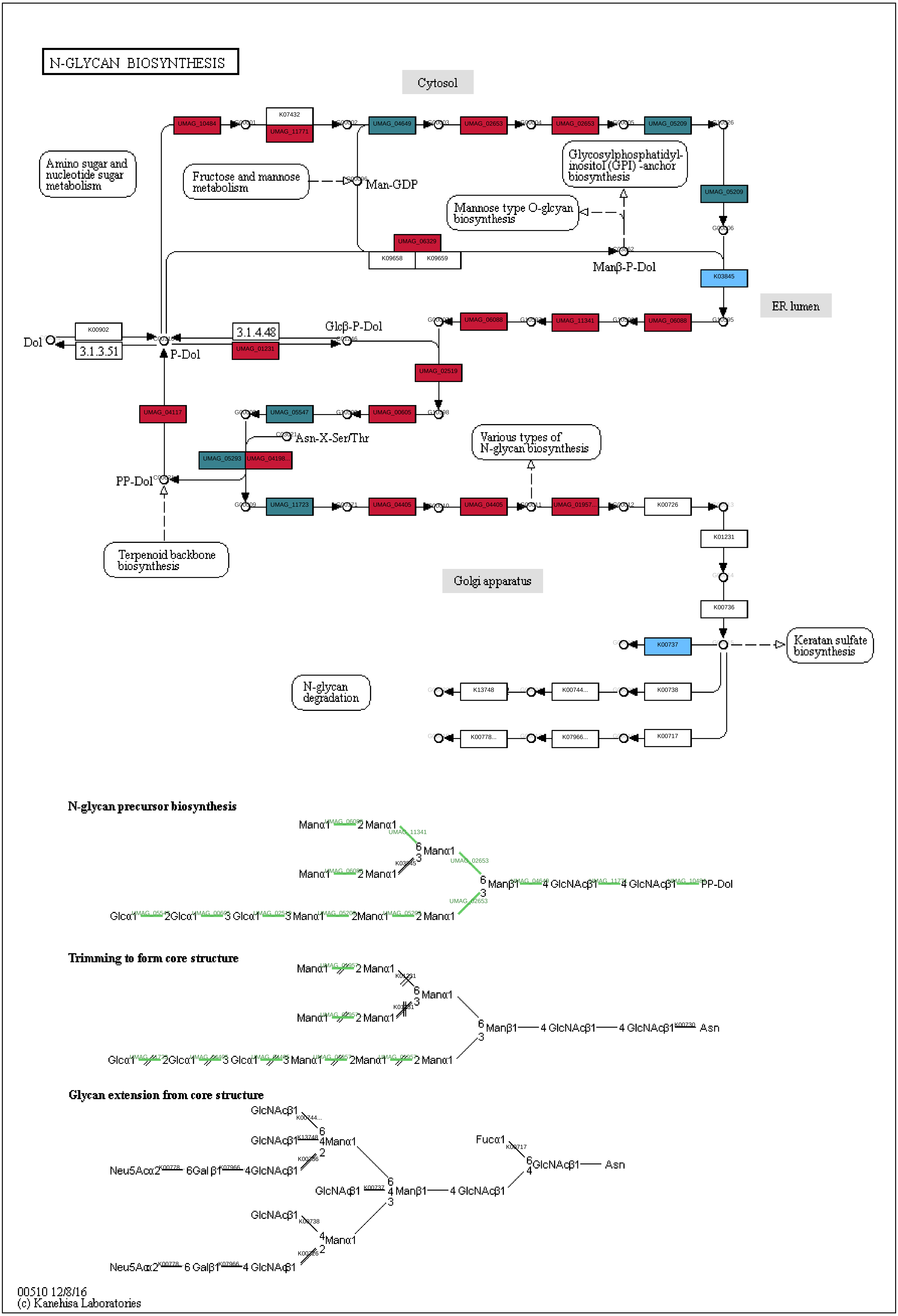
N-GLYCAN BIOSYNTHESIS
Cytosol
ER lumen
Golgi apparatus
Amino sugar and nucleotide sugar metabolism
Fructose and mannose metabolism
Man-GDP
Glycosylphosphatidyl-inositol (GPI) -anchor biosynthesis
Mannose type O-glcyan biosynthesis
Manβ-P-Dol
Dol
P-Dol
PP-Dol
Glcβ-P-Dol
Asn-X-Ser/Thr
Various types of N-glycan biosynthesis
Terpenoid backbone biosynthesis
Keratan sulfate biosynthesis
N-glycan degradation
3.1.4.48
3.1.3.51
K00902
K07432
K09658
K09659
K03845
K00726
K01231
K00736
K00737
K13748
K00744...
K00738
K00778...
K07966...
K00717
UMAG_10484
UMAG_11771
UMAG_04649
UMAG_02653
UMAG_05209
UMAG_06329
UMAG_06088
UMAG_11341
UMAG_01231
UMAG_02519
UMAG_04117
UMAG_05547
UMAG_00605
UMAG_05293
UMAG_04198...
UMAG_11723
UMAG_04405
UMAG_01957...
N-glycan precursor biosynthesis
Trimming to form core structure
Glycan extension from core structure
00510 12/8/16
(c) Kanehisa Laboratories

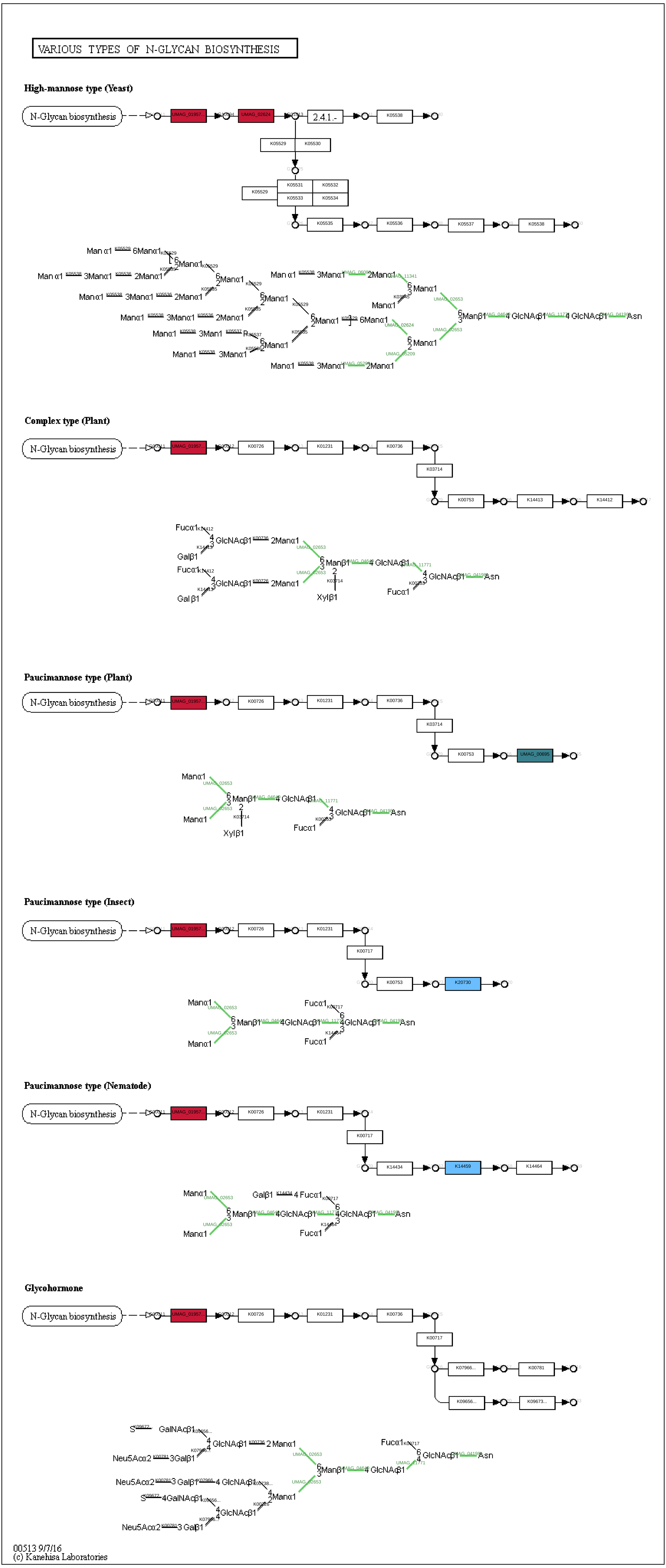
VARIOUS TYPES OF N-GLYCAN BIOSYNTHESIS
High-mannose type (Yeast)
N-Glycan biosynthesis
UMAG_01957
UMAG_02624
2.4.1.-
K05538
K05529
K05530
K05531
K05532
K05533
K05534
K05535
K05536
K05537
Complex type (Plant)
K00726
K01231
K00736
K03714
K00753
K14413
K14412
Paucimannose type (Plant)
UMAG_00695
Paucimannose type (Insect)
K00717
K20730
Paucimannose type (Nematode)
K14434
K14459
K14464
Glycohormone
K07966...
K00781
K09656...
K09673...
Asn
00513 9/7/16
(c) Kanehisa Laboratories

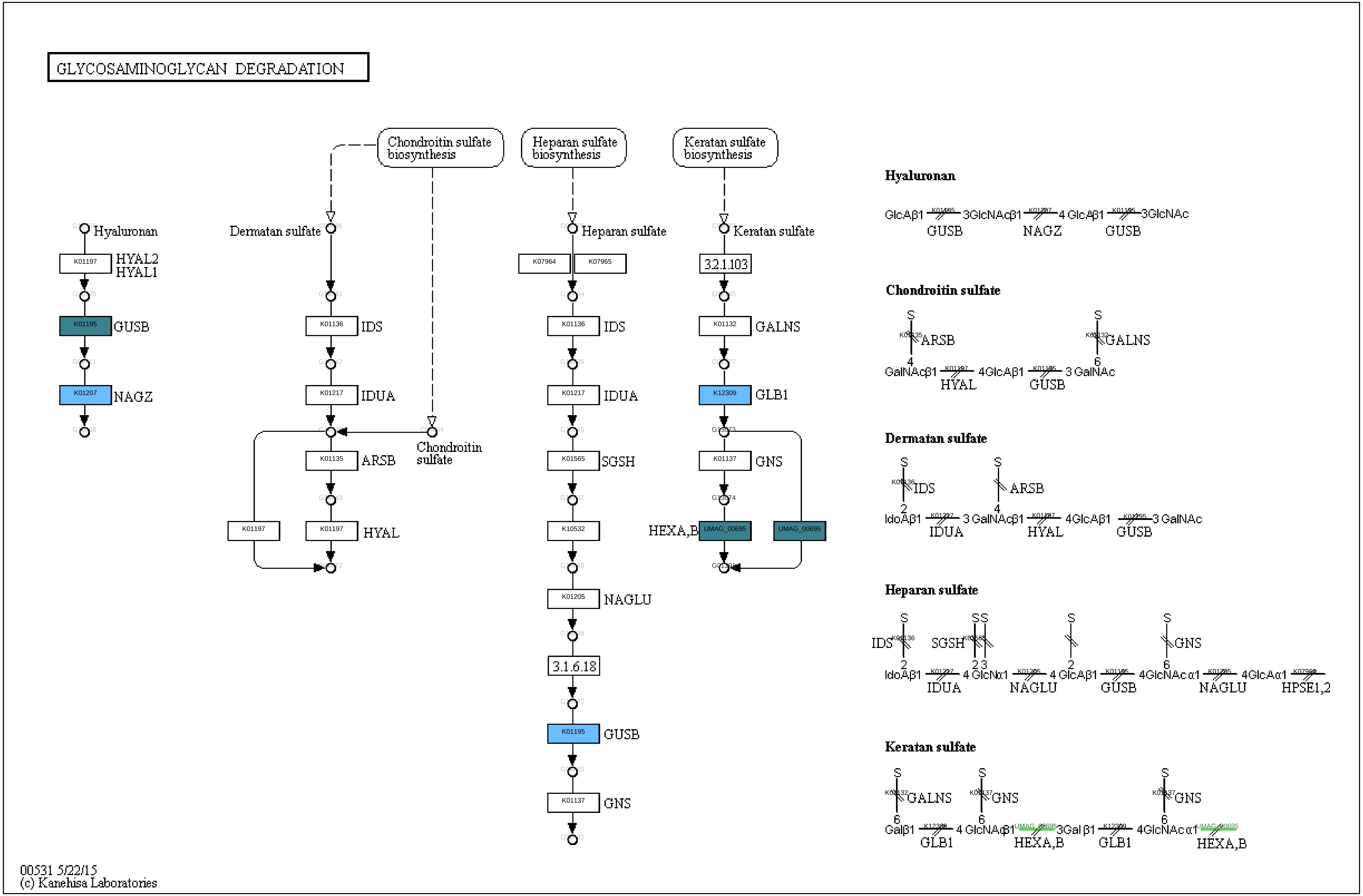
GLYCOSAMINOGLYCAN DEGRADATION
Chondroitin sulfate biosynthesis
Heparan sulfate biosynthesis
Keratan sulfate biosynthesis
Hyaluronan
K01197 HYAL2 HYAL1
K01195 GUSB
K01207 NAGZ
Dermatan sulfate
K01136 IDS
K01217 IDUA
Chondroitin sulfate
K01135 ARSB
K01197
K01197 HYAL
Heparan sulfate
K07964
K07965
K01136 IDS
K01217 IDUA
K01565 SGSH
K10532
K01205 NAGLU
3.1.6.18
K01195 GUSB
K01137 GNS
Keratan sulfate
3.2.1.103
K01132 GALNS
K12309 GLB1
K01137 GNS
HEXA,B UMAG_00695
UMAG_00695
Hyaluronan
GlcAβ1 3GlcNAcβ1 4 GlcAβ1 3GlcNAc
GUSB NAGZ GUSB
Chondroitin sulfate
ARSB GALNS
GalNAcβ1 4GlcAβ1 3 GalNAc
HYAL GUSB
Dermatan sulfate
IDS ARSB
IdoAβ1 3 GalNAcβ1 4GlcAβ1 3 GalNAc
IDUA HYAL GUSB
Heparan sulfate
IDS SGSH GNS
IdoAβ1 4 GlcNα1 4 GlcAβ1 4GlcNAcα1 4GlcAα1
IDUA NAGLU GUSB NAGLU HPSE1,2
Keratan sulfate
GALNS GNS GNS
Galβ1 4 GlcNAcβ1 3Gal β1 4GlcNAcα1
GLB1 HEXA,B GLB1 HEXA,B
00531 5/22/15
(c) Kanehisa Laboratories

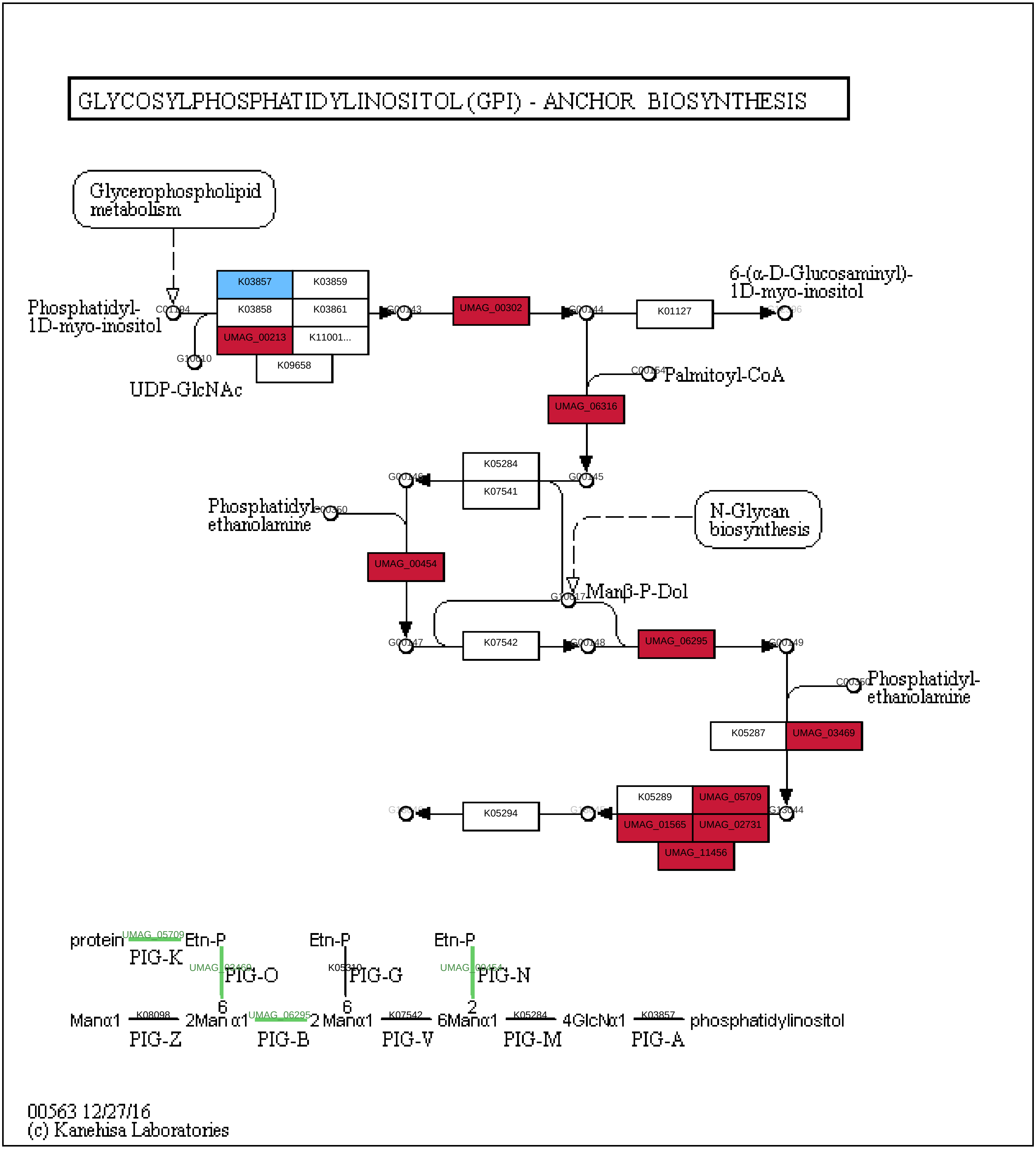
GLYCOSYLPHOSPHATIDYLINOSITOL (GPI) - ANCHOR BIOSYNTHESIS
Glycerophospholipid metabolism
Phosphatidyl-1D-myo-inositol
C01194
K03857
K03859
K03858
K03861
UMAG_00213
K11001...
K09658
G10610
UDP-GlcNAc
G00143
UMAG_00302
G00144
K01127
6-(α-D-Glucosaminyl)-1D-myo-inositol
C00154
Palmitoyl-CoA
UMAG_06316
G00145
K05284
K07541
G00146
Phosphatidylethanolamine
C00350
UMAG_00454
N-Glycan biosynthesis
Manβ-P-Dol
G10617
G00147
K07542
G00148
UMAG_06295
G00149
C00350
Phosphatidylethanolamine
K05287
UMAG_03469
K05289
UMAG_05709
UMAG_01565
UMAG_02731
UMAG_11456
G13044
K05294
protein
UMAG_05709
Etn-P
PIG-K
UMAG_03469
PIG-O
Etn-P
K05310
PIG-G
Etn-P
UMAG_00454
PIG-N
6
6
2
Manα1
K08098
2Man α1
UMAG_06295
2 Manα1
K07542
6Manα1
K05284
4GlcNα1
K03857
phosphatidylinositol
PIG-Z
PIG-B
PIG-V
PIG-M
PIG-A
00563 12/27/16
(c) Kanehisa Laboratories

# 8. Metabolism of cofactors and vitamins

| MAP | PATHWAY |
|---|---|
| **130** | Ubiquinone and other terpenoid-quinone biosynthesis |
| **670** | One carbon pool by folate |
| **730** | Thiamine metabolism |
| **740** | Riboflavin metabolism |
| **750** | Vitamin B6 metabolism |
| **760** | Nicotinate and nicotinamide metabolism |
| **770** | Pantothenate and CoA biosynthesis |
| **780** | Biotin metabolism |
| **785** | Lipoic acid metabolism |
| **790** | Folate biosynthesis |
| **860** | Porphyrin and chlorophyll metabolism |

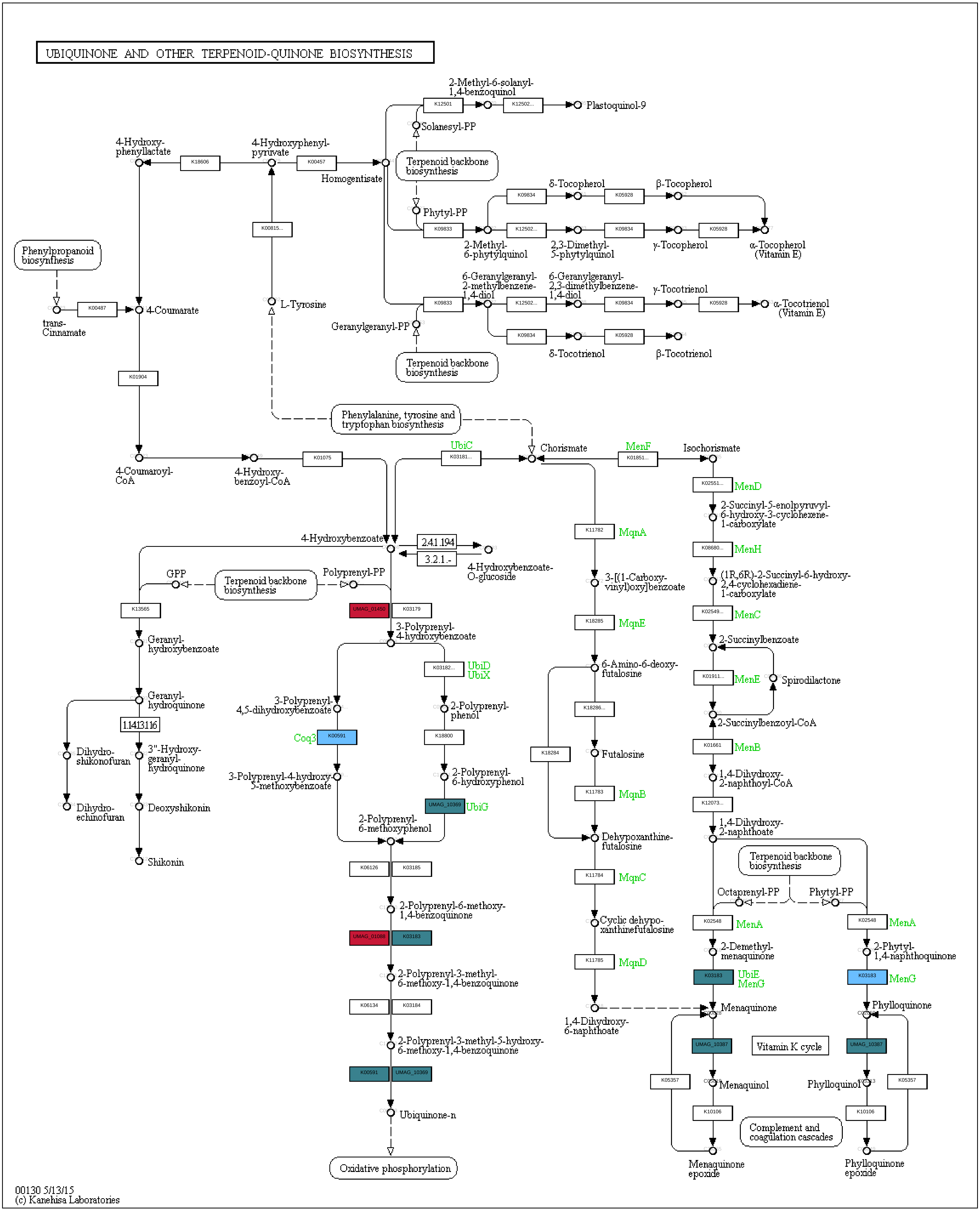
UBIQUINONE AND OTHER TERPENOID-QUINONE BIOSYNTHESIS
2-Methyl-6-solanyl-1,4-benzoquinol
K12501
K12502...
Plastoquinol-9
Solanesyl-PP
Terpenoid backbone biosynthesis
4-Hydroxy-phenyllactate
K18606
4-Hydroxyphenyl-pyruvate
K00457
Homogentisate
Phytyl-PP
δ-Tocopherol
β-Tocopherol
K09834
K05928
K09833
K12502...
K09834
K05928
2-Methyl-6-phytylquinol
2,3-Dimethyl-5-phytylquinol
γ-Tocopherol
α-Tocopherol (Vitamin E)
K00815...
Phenylpropanoid biosynthesis
6-Geranylgeranyl-2-methylbenzene-1,4-diol
6-Geranylgeranyl-2,3-dimethylbenzene-1,4-diol
γ-Tocotrienol
α-Tocotrienol (Vitamin E)
K00487
4-Coumarate
L-Tyrosine
Geranylgeranyl-PP
trans-Cinnamate
δ-Tocotrienol
β-Tocotrienol
Terpenoid backbone biosynthesis
K01904
Phenylalanine, tyrosine and tryptophan biosynthesis
UbiC
K03181...
Chorismate
MenF
K01851...
Isochorismate
4-Coumaroyl-CoA
4-Hydroxy-benzoyl-CoA
K01075
K02551...
MenD
2-Succinyl-5-enolpyruvyl-6-hydroxy-3-cyclohexene-1-carboxylate
K11782
MqnA
K08680...
MenH
4-Hydroxybenzoate
2.4.1.194
3.2.1.-
4-Hydroxybenzoate-O-glucoside
GPP
Terpenoid backbone biosynthesis
Polyprenyl-PP
3-[(1-Carboxy-vinyl)oxy]benzoate
(1R,6R)-2-Succinyl-6-hydroxy-2,4-cyclohexadiene-1-carboxylate
K13565
UMAG_01450
K03179
K18285
MqnE
K02549...
MenC
Geranyl-hydroxybenzoate
3-Polyprenyl-4-hydroxybenzoate
2-Succinylbenzoate
K03182...
UbiD
UbiX
6-Amino-6-deoxy-futalosine
Geranyl-hydroquinone
K01911...
MenE
Spirodilactone
1.14.13.116
3-Polyprenyl-4,5-dihydroxybenzoate
2-Polyprenyl-phenol
K18286...
2-Succinylbenzoyl-CoA
Dihydro-shikonofuran
3''-Hydroxy-geranyl-hydroquinone
Coq3
K00591
K18800
K18284
Futalosine
K01661
MenB
3-Polyprenyl-4-hydroxy-5-methoxybenzoate
2-Polyprenyl-6-hydroxyphenol
K11783
MqnB
1,4-Dihydroxy-2-naphthoyl-CoA
Dihydro-echinofuran
Deoxyshikonin
UMAG_10369
UbiG
K12073...
2-Polyprenyl-6-methoxyphenol
1,4-Dihydroxy-2-naphthoate
Dehypoxanthine-futalosine
Shikonin
K06126
K03185
Terpenoid backbone biosynthesis
K11784
MqnC
Octaprenyl-PP
Phytyl-PP
2-Polyprenyl-6-methoxy-1,4-benzoquinone
Cyclic dehypo-xanthinefutalosine
K02548
MenA
K02548
MenA
UMAG_01088
K03183
2-Demethyl-menaquinone
2-Phytyl-1,4-naphthoquinone
2-Polyprenyl-3-methyl-6-methoxy-1,4-benzoquinone
K11785
MqnD
K03183
UbiE
MenG
K03183
MenG
K06134
K03184
1,4-Dihydroxy-6-naphthoate
Menaquinone
Phylloquinone
2-Polyprenyl-3-methyl-5-hydroxy-6-methoxy-1,4-benzoquinone
UMAG_10387
Vitamin K cycle
UMAG_10387
K00591
UMAG_10369
K05357
Menaquinol
Phylloquinol
K05357
Ubiquinone-n
K10106
K10106
Complement and coagulation cascades
Oxidative phosphorylation
Menaquinone epoxide
Phylloquinone epoxide
00130 5/13/15
(c) Kanehisa Laboratories

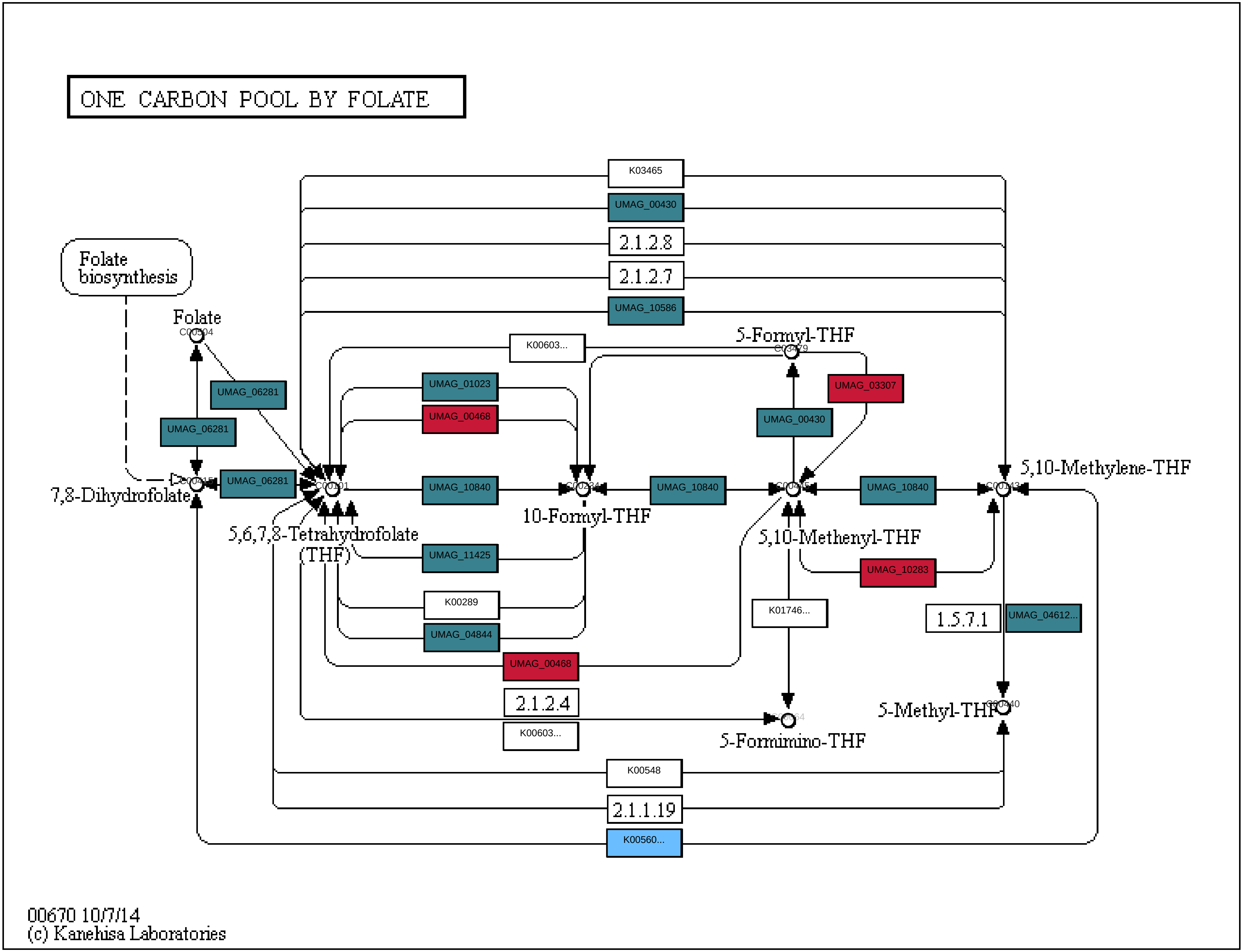
ONE CARBON POOL BY FOLATE
Folate biosynthesis
Folate
C00504
7,8-Dihydrofolate
5,6,7,8-Tetrahydrofolate (THF)
C00101
10-Formyl-THF
C00234
5-Formyl-THF
C03479
5,10-Methenyl-THF
C00445
5,10-Methylene-THF
C00143
5-Methyl-THF
C00440
5-Formimino-THF
K03465
UMAG_00430
2.1.2.8
2.1.2.7
UMAG_10586
K00603...
UMAG_06281
UMAG_06281
UMAG_06281
UMAG_01023
UMAG_00468
UMAG_03307
UMAG_00430
UMAG_10840
UMAG_10840
UMAG_10840
UMAG_11425
UMAG_10283
K00289
UMAG_04844
K01746...
1.5.7.1
UMAG_04612...
UMAG_00468
2.1.2.4
K00603...
K00548
2.1.1.19
K00560...
00670 10/7/14
(c) Kanehisa Laboratories

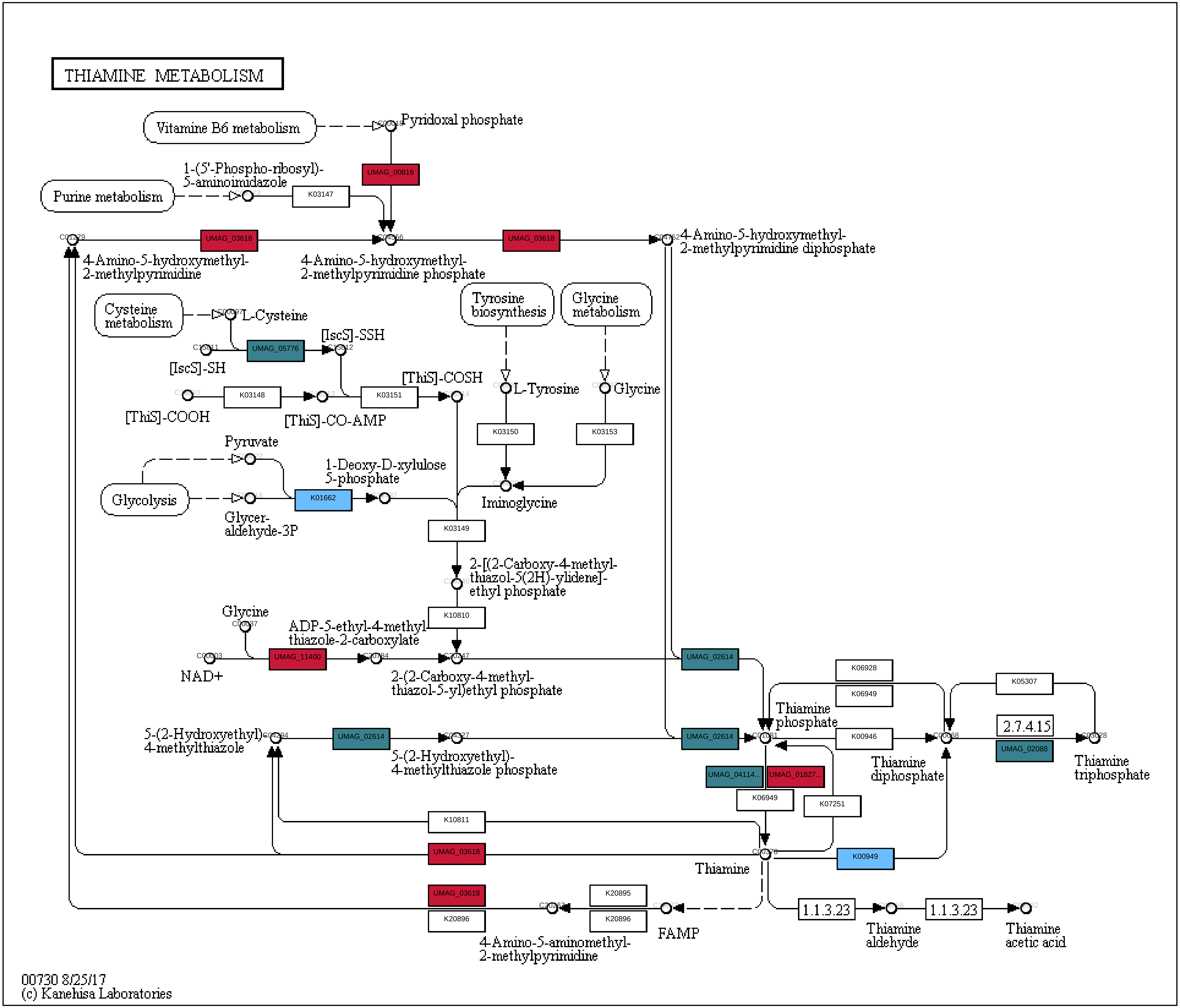

THIAMINE METABOLISM
Vitamine B6 metabolism
Pyridoxal phosphate
UMAG_00816
Purine metabolism
1-(5'-Phospho-ribosyl)-
5-aminoimidazole
K03147
C01279
UMAG_03618
UMAG_03618
C04556
C04752
4-Amino-5-hydroxymethyl-
2-methylpyrimidine diphosphate
4-Amino-5-hydroxymethyl-
2-methylpyrimidine
4-Amino-5-hydroxymethyl-
2-methylpyrimidine phosphate
Cysteine
metabolism
C00097
L-Cysteine
Tyrosine
biosynthesis
Glycine
metabolism
C15811
UMAG_05776
C15812
[IscS]-SSH
[IscS]-SH
[ThiS]-COSH
K03148
K03151
L-Tyrosine
Glycine
[ThiS]-COOH
[ThiS]-CO-AMP
K03150
K03153
Pyruvate
1-Deoxy-D-xylulose
5-phosphate
Glycolysis
K01662
Iminoglycine
Glycer-
aldehyde-3P
K03149
2-[(2-Carboxy-4-methyl-
thiazol-5(2H)-ylidene]-
ethyl phosphate
Glycine
C00037
K10810
ADP-5-ethyl-4-methyl-
thiazole-2-carboxylate
C00003
UMAG_11400
C20784
C20247
UMAG_02614
NAD+
2-(2-Carboxy-4-methyl-
thiazol-5-yl)ethyl phosphate
K06928
K06949
K05307
Thiamine
phosphate
5-(2-Hydroxyethyl)-
4-methylthiazole
C04294
UMAG_02614
C04327
UMAG_02614
C01081
K00946
C00068
2.7.4.15
C03028
UMAG_02088
5-(2-Hydroxyethyl)-
4-methylthiazole phosphate
Thiamine
diphosphate
Thiamine
triphosphate
UMAG_04114...
UMAG_01827...
K06949
K07251
K10811
UMAG_03618
C00378
K00949
Thiamine
UMAG_03618
K20895
K20896
K20896
1.1.3.23
1.1.3.23
FAMP
Thiamine
aldehyde
Thiamine
acetic acid
4-Amino-5-aminomethyl-
2-methylpyrimidine
00730 8/25/17
(c) Kanehisa Laboratories

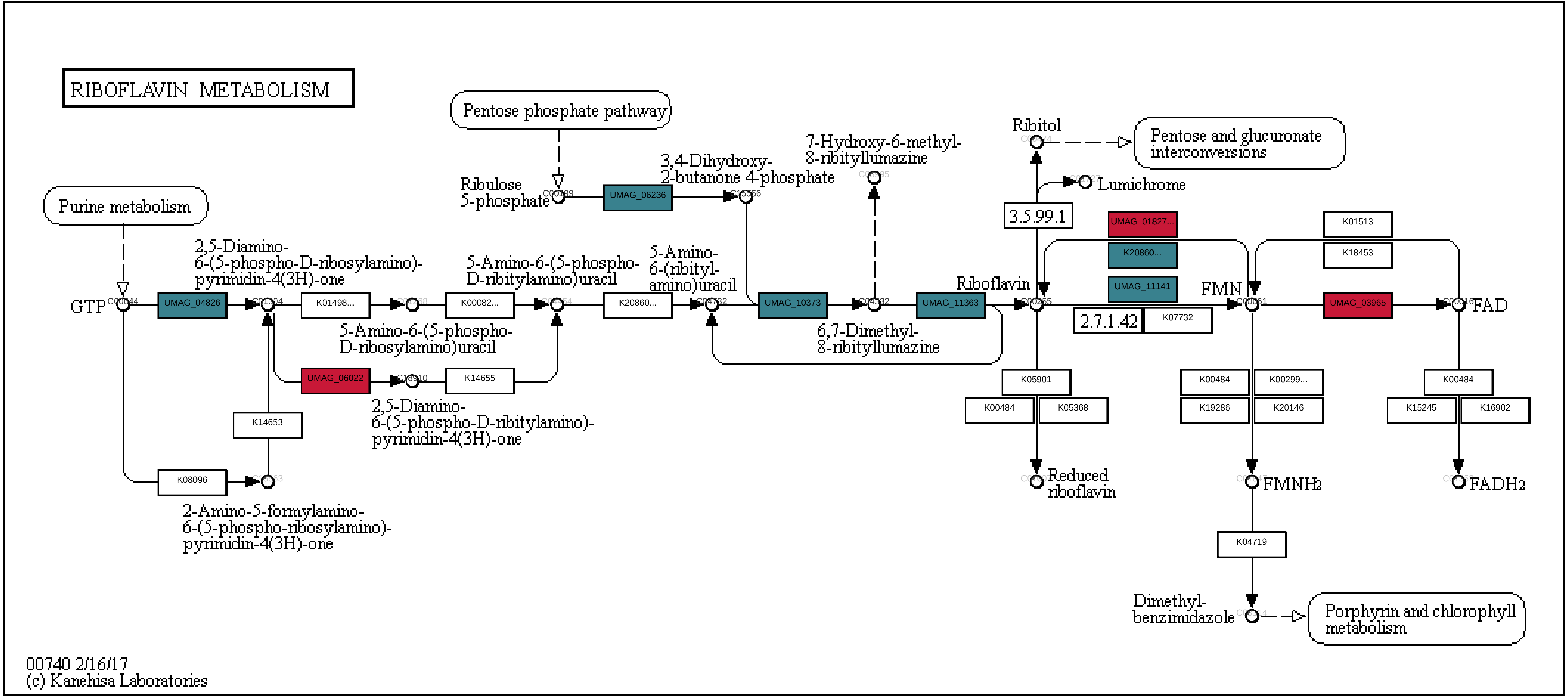

RIBOFLAVIN METABOLISM
Pentose phosphate pathway
Purine metabolism
Ribulose 5-phosphate
UMAG_06236
3,4-Dihydroxy-2-butanone 4-phosphate
7-Hydroxy-6-methyl-8-ribityllumazine
Ribitol
Pentose and glucuronate interconversions
Lumichrome
3.5.99.1
UMAG_01827...
K20860...
UMAG_11141
K01513
K18453
GTP
UMAG_04826
2,5-Diamino-6-(5-phospho-D-ribosylamino)-pyrimidin-4(3H)-one
K01498...
5-Amino-6-(5-phospho-D-ribitylamino)uracil
K00082...
K20860...
5-Amino-6-(ribitylamino)uracil
UMAG_10373
UMAG_11363
Riboflavin
FMN
UMAG_03965
FAD
5-Amino-6-(5-phospho-D-ribosylamino)uracil
6,7-Dimethyl-8-ribityllumazine
2.7.1.42
K07732
UMAG_06022
K14655
2,5-Diamino-6-(5-phospho-D-ribitylamino)-pyrimidin-4(3H)-one
K14653
K08096
2-Amino-5-formylamino-6-(5-phospho-ribosylamino)-pyrimidin-4(3H)-one
K05901
K00484
K05368
Reduced riboflavin
K00484
K00299...
K19286
K20146
FMNH2
K04719
Dimethyl-benzimidazole
Porphyrin and chlorophyll metabolism
K00484
K15245
K16902
FADH2
00740 2/16/17
(c) Kanehisa Laboratories

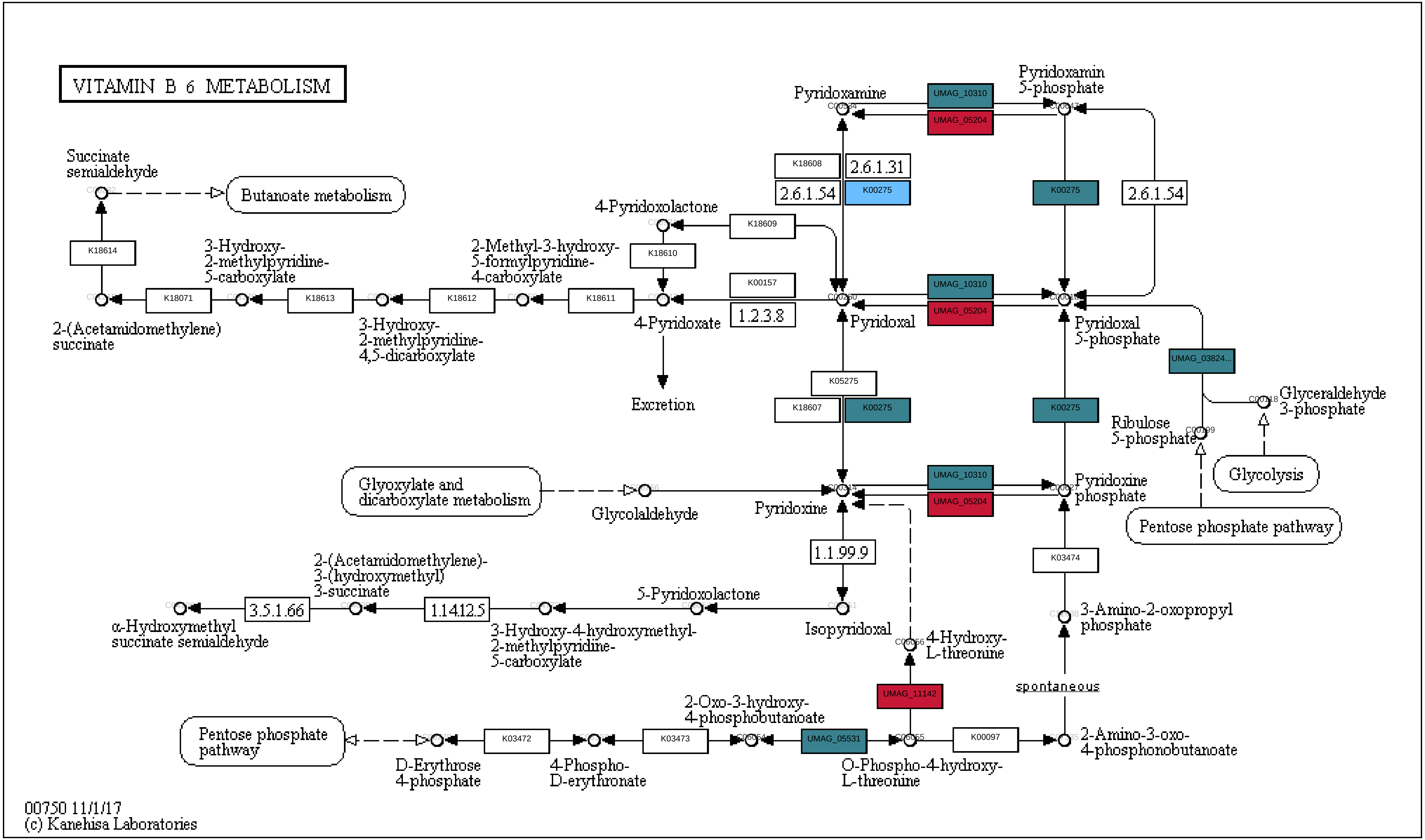
VITAMIN B 6 METABOLISM
Succinate semialdehyde
Butanoate metabolism
K18614
3-Hydroxy-2-methylpyridine-5-carboxylate
2-Methyl-3-hydroxy-5-formylpyridine-4-carboxylate
4-Pyridoxolactone
K18609
K18610
K18071
K18613
K18612
K18611
K00157
1.2.3.8
2-(Acetamidomethylene) succinate
3-Hydroxy-2-methylpyridine-4,5-dicarboxylate
4-Pyridoxate
Excretion
Pyridoxamine
Pyridoxamin 5-phosphate
UMAG_10310
UMAG_05204
K18608
2.6.1.31
2.6.1.54
K00275
Pyridoxal
Pyridoxal 5-phosphate
UMAG_03824...
Glyceraldehyde 3-phosphate
Ribulose 5-phosphate
Glycolysis
K05275
K18607
Glyoxylate and dicarboxylate metabolism
Glycolaldehyde
Pyridoxine
Pyridoxine phosphate
Pentose phosphate pathway
1.1.99.9
K03474
2-(Acetamidomethylene)-3-(hydroxymethyl)3-succinate
5-Pyridoxolactone
3.5.1.66
1.14.12.5
α-Hydroxymethyl succinate semialdehyde
3-Hydroxy-4-hydroxymethyl-2-methylpyridine-5-carboxylate
Isopyridoxal
4-Hydroxy-L-threonine
3-Amino-2-oxopropyl phosphate
spontaneous
UMAG_11142
2-Oxo-3-hydroxy-4-phosphobutanoate
K03472
K03473
UMAG_05531
K00097
D-Erythrose 4-phosphate
4-Phospho-D-erythronate
O-Phospho-4-hydroxy-L-threonine
2-Amino-3-oxo-4-phosphonobutanoate
00750 11/1/17
(c) Kanehisa Laboratories

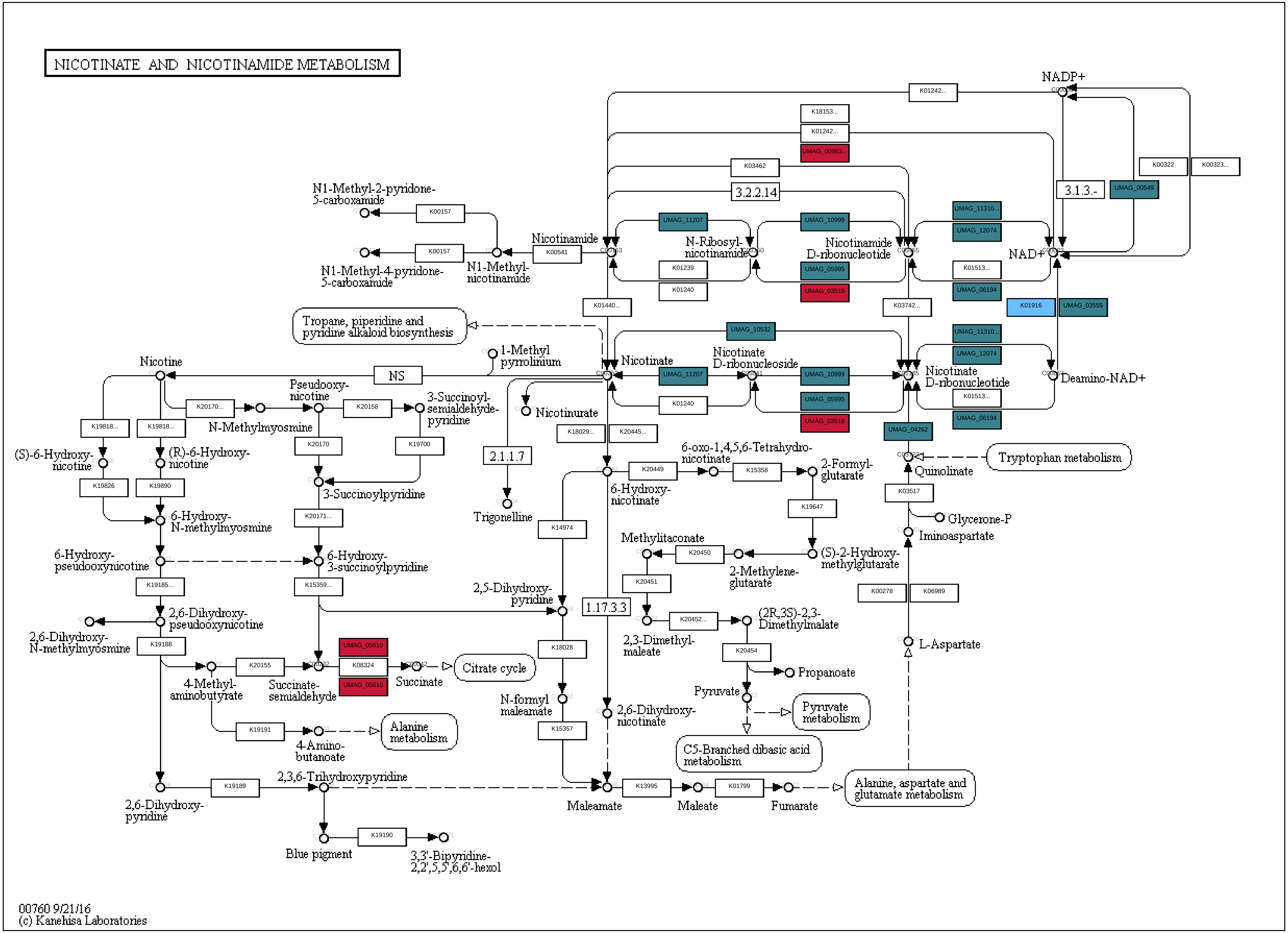
NICOTINATE AND NICOTINAMIDE METABOLISM
NADP+
K01242...
K18153...
K01242...
UMAG_00963...
K03462
3.2.2.14
K00322
K00323...
3.1.3.-
UMAG_00549
N1-Methyl-2-pyridone-5-carboxamide
K00157
K00157
N1-Methyl-4-pyridone-5-carboxamide
Nicotinamide
K00541
N1-Methyl-nicotinamide
UMAG_11207
N-Ribosyl-nicotinamide
K01239
K01240
UMAG_10999
Nicotinamide D-ribonucleotide
UMAG_05995
UMAG_03518
UMAG_11310...
UMAG_12074
NAD+
K01513...
UMAG_06194
K01440...
K03742...
K01916
UMAG_03555
Tropane, piperidine and pyridine alkaloid biosynthesis
UMAG_10532
1-Methyl pyrrolinium
Nicotine
NS
Nicotinate
Nicotinate D-ribonucleoside
UMAG_11207
UMAG_10999
UMAG_11310...
UMAG_12074
Nicotinate D-ribonucleotide
Deamino-NAD+
K01240
UMAG_05995
UMAG_03518
K01513...
UMAG_06194
UMAG_04262
Pseudooxy-nicotine
K20170...
K20158
3-Succinoyl-semialdehyde-pyridine
N-Methylmyosmine
Nicotinurate
K19818...
K19818...
K20170
K19700
K18029...
K20445...
(S)-6-Hydroxy-nicotine
(R)-6-Hydroxy-nicotine
2.1.1.7
6-oxo-1,4,5,6-Tetrahydro-nicotinate
K20449
K15358
2-Formyl-glutarate
Tryptophan metabolism
Quinolinate
K19826
K19890
3-Succinoylpyridine
6-Hydroxy-nicotinate
K03517
Trigonelline
K19647
6-Hydroxy-N-methylmyosmine
K20171...
K14974
Glycerone-P
Iminoaspartate
Methylitaconate
K20450
(S)-2-Hydroxy-methylglutarate
6-Hydroxy-pseudooxynicotine
6-Hydroxy-3-succinoylpyridine
2-Methylene-glutarate
K20451
K00278
K06989
K19185...
K15359...
2,5-Dihydroxy-pyridine
1.17.3.3
2,6-Dihydroxy-pseudooxynicotine
K20452...
(2R,3S)-2,3-Dimethylmalate
2,6-Dihydroxy-N-methylmyosmine
K19188
UMAG_05610
K18028
2,3-Dimethyl-maleate
L-Aspartate
K20454
K20155
K08324
Citrate cycle
UMAG_05610
Propanoate
4-Methyl-aminobutyrate
Succinate-semialdehyde
Succinate
N-formyl maleamate
Pyruvate
2,6-Dihydroxy-nicotinate
Pyruvate metabolism
K19191
Alanine metabolism
K15357
4-Amino-butanoate
C5-Branched dibasic acid metabolism
2,3,6-Trihydroxypyridine
K19189
K13995
K01799
Alanine, aspartate and glutamate metabolism
2,6-Dihydroxy-pyridine
Maleamate
Maleate
Fumarate
K19190
Blue pigment
3,3'-Bipyridine-2,2',5,5',6,6'-hexol
00760 9/21/16
(c) Kanehisa Laboratories

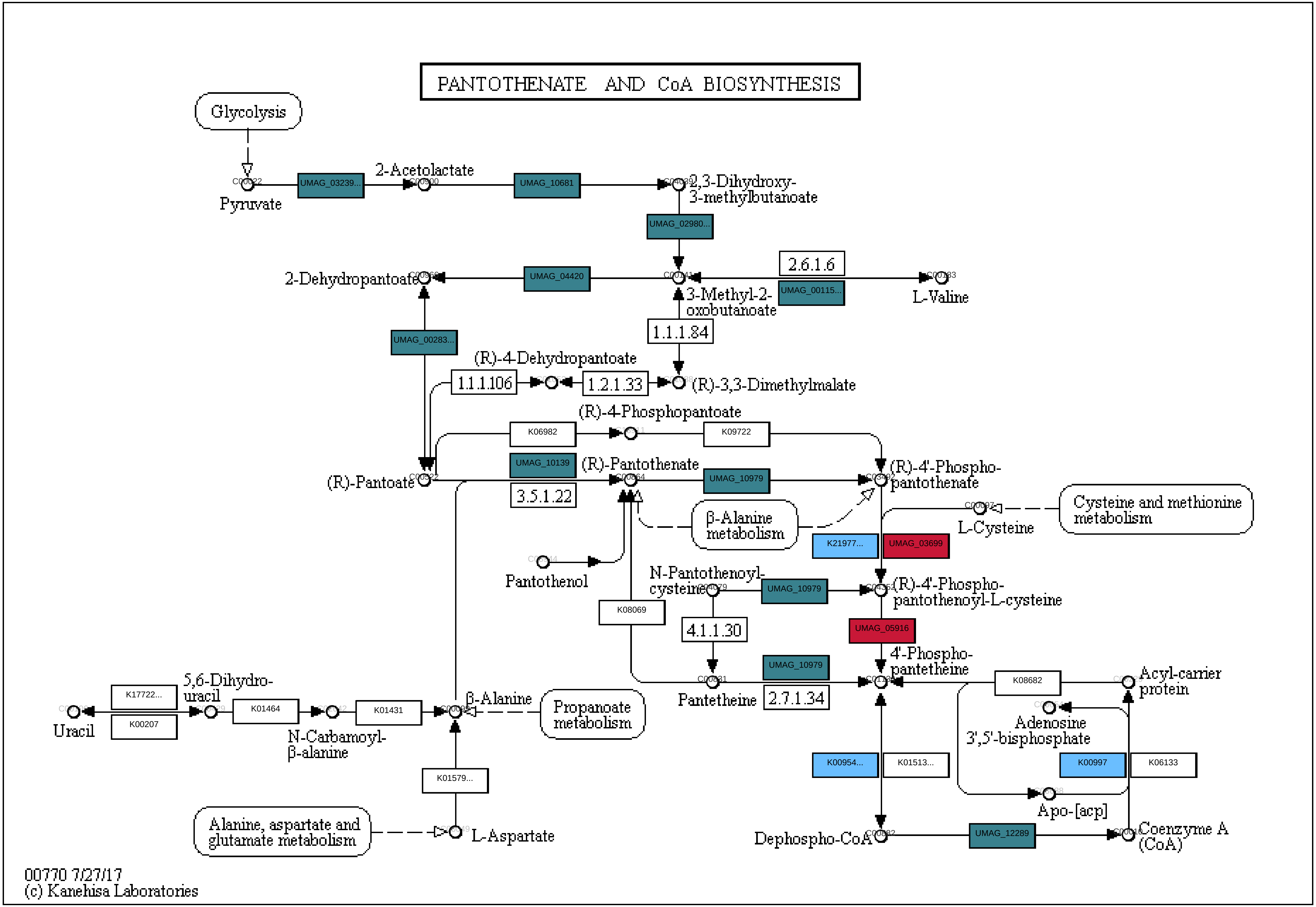

PANTOTHENATE AND CoA BIOSYNTHESIS
Glycolysis
Pyruvate
UMAG_03239...
2-Acetolactate
UMAG_10681
2,3-Dihydroxy-3-methylbutanoate
UMAG_02980...
2-Dehydropantoate
UMAG_04420
2.6.1.6
UMAG_00115...
L-Valine
3-Methyl-2-oxobutanoate
1.1.1.84
UMAG_00283...
(R)-4-Dehydropantoate
1.1.1.106
1.2.1.33
(R)-3,3-Dimethylmalate
(R)-4-Phosphopantoate
K06982
K09722
UMAG_10139
(R)-Pantothenate
UMAG_10979
(R)-4'-Phospho-pantothenate
(R)-Pantoate
3.5.1.22
β-Alanine metabolism
Cysteine and methionine metabolism
L-Cysteine
K21977...
UMAG_03699
Pantothenol
N-Pantothenoyl-cysteine
UMAG_10979
(R)-4'-Phospho-pantothenoyl-L-cysteine
K08069
4.1.1.30
UMAG_05916
4'-Phospho-pantetheine
UMAG_10979
K08682
Acyl-carrier protein
Pantetheine
2.7.1.34
5,6-Dihydro-uracil
K17722...
K00207
Uracil
K01464
K01431
N-Carbamoyl-β-alanine
β-Alanine
Propanoate metabolism
Adenosine 3',5'-bisphosphate
K00954...
K01513...
K00997
K06133
K01579...
Apo-[acp]
Alanine, aspartate and glutamate metabolism
L-Aspartate
Dephospho-CoA
UMAG_12289
Coenzyme A (CoA)
00770 7/27/17
(c) Kanehisa Laboratories

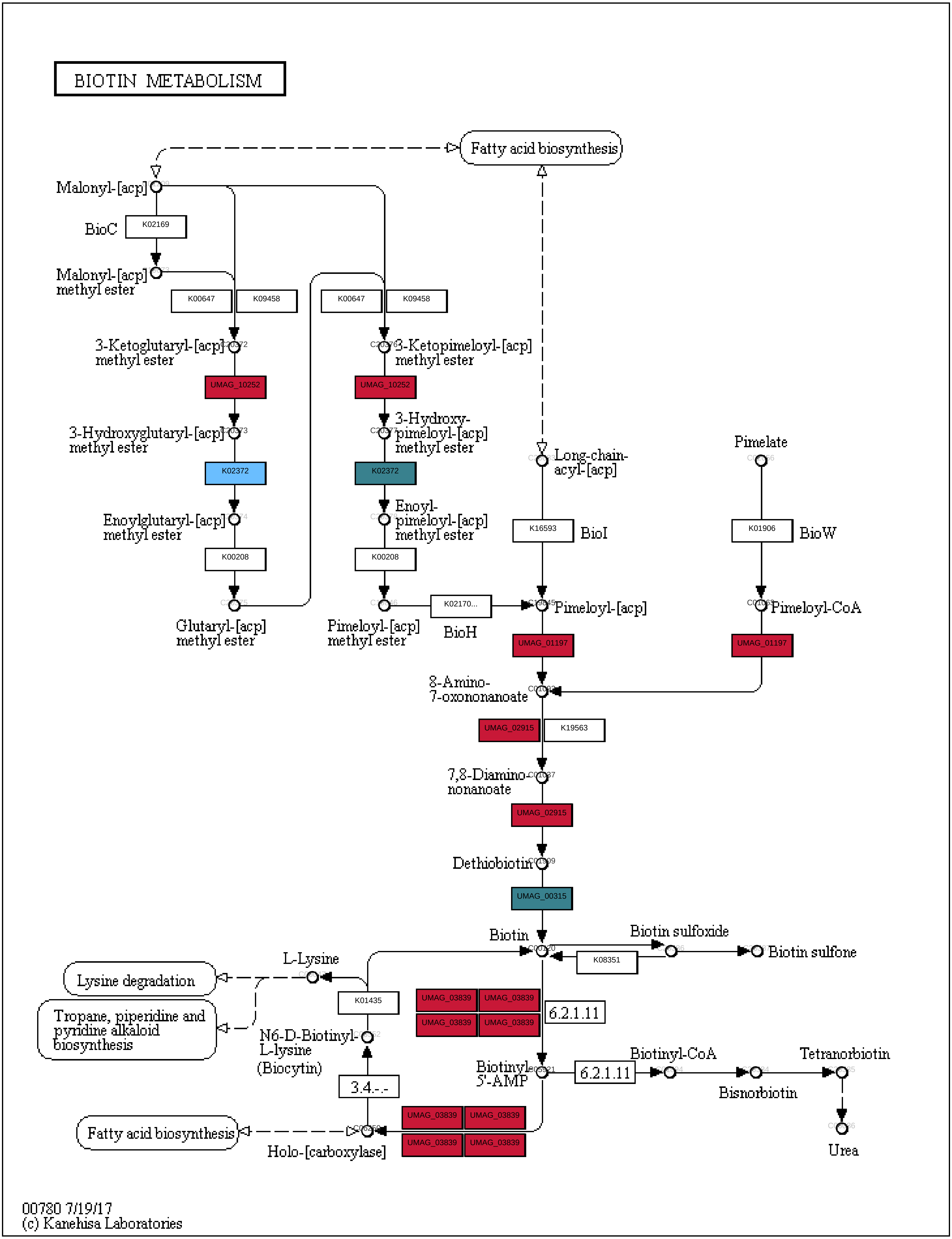
BIOTIN METABOLISM
Fatty acid biosynthesis
Malonyl-[acp]
BioC
K02169
Malonyl-[acp] methyl ester
K00647
K09458
K00647
K09458
3-Ketoglutaryl-[acp] methyl ester
C20372
3-Ketopimeloyl-[acp] methyl ester
UMAG_10252
UMAG_10252
3-Hydroxyglutaryl-[acp] methyl ester
C20373
3-Hydroxy-pimeloyl-[acp] methyl ester
K02372
K02372
Enoylglutaryl-[acp] methyl ester
Enoyl-pimeloyl-[acp] methyl ester
K00208
K00208
Glutaryl-[acp] methyl ester
Pimeloyl-[acp] methyl ester
K02170...
BioH
Long-chain-acyl-[acp]
K16593
BioI
Pimeloyl-[acp]
UMAG_01197
Pimelate
K01906
BioW
Pimeloyl-CoA
UMAG_01197
8-Amino-7-oxononanoate
UMAG_02915
K19563
7,8-Diamino-nonanoate
UMAG_02915
Dethiobiotin
C01909
UMAG_00315
Biotin
Biotin sulfoxide
K08351
Biotin sulfone
L-Lysine
Lysine degradation
Tropane, piperidine and pyridine alkaloid biosynthesis
K01435
UMAG_03839
UMAG_03839
UMAG_03839
UMAG_03839
6.2.1.11
N6-D-Biotinyl-L-lysine (Biocytin)
3.4.-.-
Biotinyl-5'-AMP
6.2.1.11
Biotinyl-CoA
Bisnorbiotin
Tetranorbiotin
Urea
UMAG_03839
UMAG_03839
UMAG_03839
UMAG_03839
Fatty acid biosynthesis
Holo-[carboxylase]
00780 7/19/17
(c) Kanehisa Laboratories

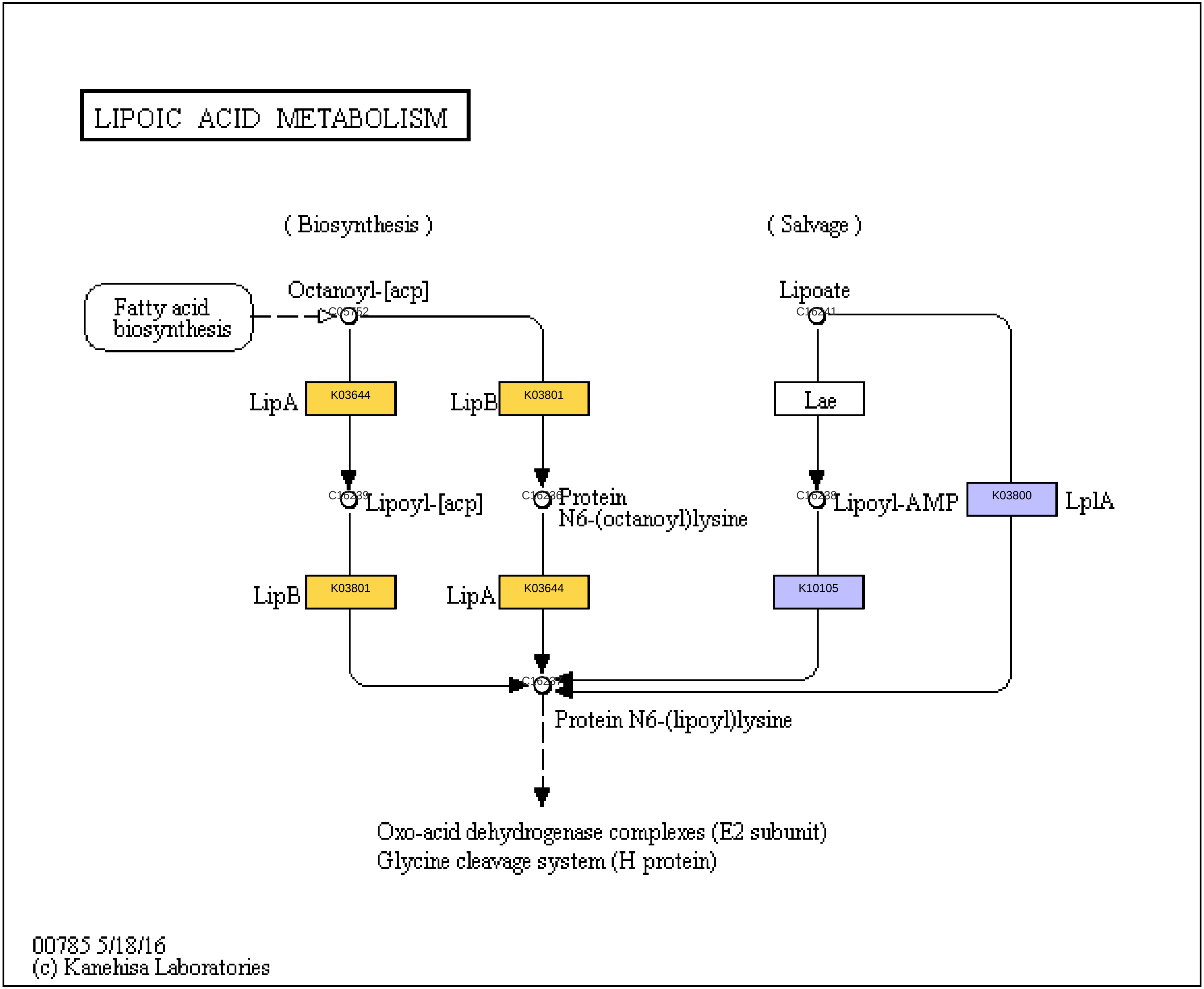
LIPOIC ACID METABOLISM
( Biosynthesis )
( Salvage )
Fatty acid biosynthesis
Octanoyl-[acp]
Lipoate
LipA
K03644
LipB
K03801
Lae
K03800
LplA
C16239
Lipoyl-[acp]
C16236
Protein N6-(octanoyl)lysine
C16238
Lipoyl-AMP
LipB
K03801
LipA
K03644
K10105
Protein N6-(lipoyl)lysine
Oxo-acid dehydrogenase complexes (E2 subunit)
Glycine cleavage system (H protein)
00785 5/18/16
(c) Kanehisa Laboratories

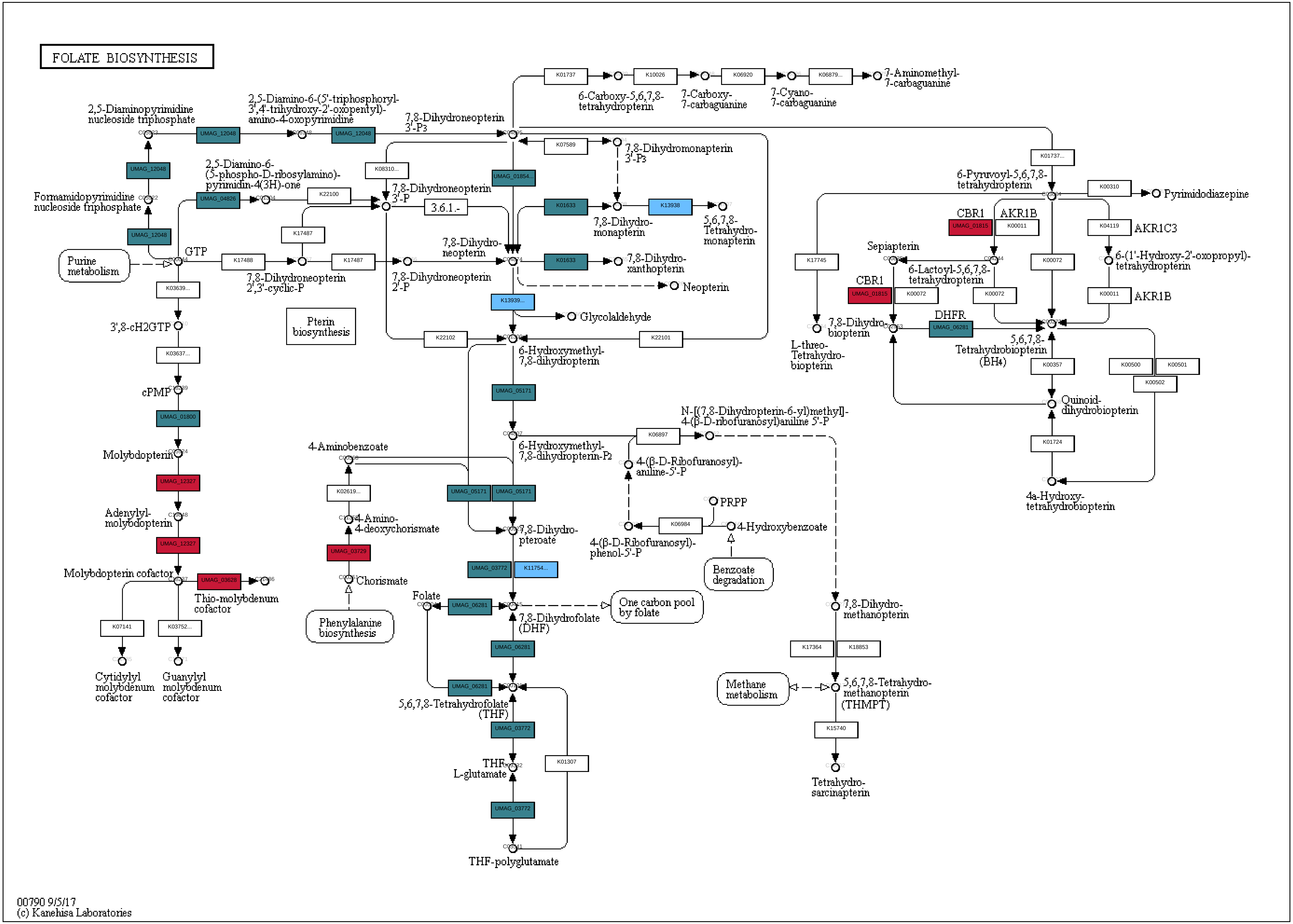
FOLATE BIOSYNTHESIS
2,5-Diaminopyrimidine nucleoside triphosphate
2,5-Diamino-6-(5'-triphosphoryl-3',4'-trihydroxy-2'-oxopentyl)-amino-4-oxopyrimidine
7,8-Dihydroneopterin 3'-P3
Formamidopyrimidine nucleoside triphosphate
2,5-Diamino-6-(5-phospho-D-ribosylamino)-pyrimidin-4(3H)-one
7,8-Dihydroneopterin 3'-P
3.6.1.-
Purine metabolism
GTP
7,8-Dihydroneopterin 2',3'-cyclic-P
7,8-Dihydroneopterin 2'-P
7,8-Dihydro-neopterin
Pterin biosynthesis
3',8-cH2GTP
cPMP
Molybdopterin
Adenylyl-molybdopterin
Molybdopterin cofactor
Thio-molybdenum cofactor
Cytidylyl molybdenum cofactor
Guanylyl molybdenum cofactor
6-Carboxy-5,6,7,8-tetrahydropterin
7-Carboxy-7-carbaguanine
7-Cyano-7-carbaguanine
7-Aminomethyl-7-carbaguanine
7,8-Dihydromonapterin 3'-P3
7,8-Dihydro-monapterin
5,6,7,8-Tetrahydro-monapterin
7,8-Dihydro-xanthopterin
Neopterin
Glycolaldehyde
6-Hydroxymethyl-7,8-dihydropterin
6-Hydroxymethyl-7,8-dihydropterin-P2
4-Aminobenzoate
4-Amino-4-deoxychorismate
Chorismate
Phenylalanine biosynthesis
7,8-Dihydro-pteroate
Folate
7,8-Dihydrofolate (DHF)
One carbon pool by folate
5,6,7,8-Tetrahydrofolate (THF)
THF-L-glutamate
THF-polyglutamate
N-[(7,8-Dihydropterin-6-yl)methyl]-4-(β-D-ribofuranosyl)aniline 5'-P
4-(β-D-Ribofuranosyl)-aniline-5'-P
PRPP
4-Hydroxybenzoate
4-(β-D-Ribofuranosyl)-phenol-5'-P
Benzoate degradation
7,8-Dihydro-methanopterin
5,6,7,8-Tetrahydro-methanopterin (THMPT)
Methane metabolism
Tetrahydro-sarcinapterin
6-Pyruvoyl-5,6,7,8-tetrahydropterin
Pyrimidodiazepine
CBR1
AKR1B
AKR1C3
Sepiapterin
6-Lactoyl-5,6,7,8-tetrahydropterin
6-(1'-Hydroxy-2'-oxopropyl)-tetrahydropterin
AKR1B
CBR1
DHFR
7,8-Dihydro-biopterin
L-threo-Tetrahydro-biopterin
5,6,7,8-Tetrahydrobiopterin (BH4)
Quinoid-dihydrobiopterin
4a-Hydroxy-tetrahydrobiopterin
K01737
K10026
K06920
K06879...
K07589
K08310...
K22100
K17487
K17488
K17487
K03639...
K03637...
K07141
K03752...
K01737...
K00310
K04119
K17745
K00011
K00072
K00072
K00072
K00011
K00357
K00500
K00501
K00502
K01724
K13938
K13939...
K22102
K22101
K06897
K06984
K02619...
K11754...
K01307
K17364
K18853
K15740
UMAG_12048
UMAG_04826
UMAG_01854...
K01633
UMAG_05171
UMAG_01800
UMAG_12327
UMAG_03628
UMAG_03729
UMAG_03772
UMAG_06281
UMAG_01815
00790 9/5/17
(c) Kanehisa Laboratories

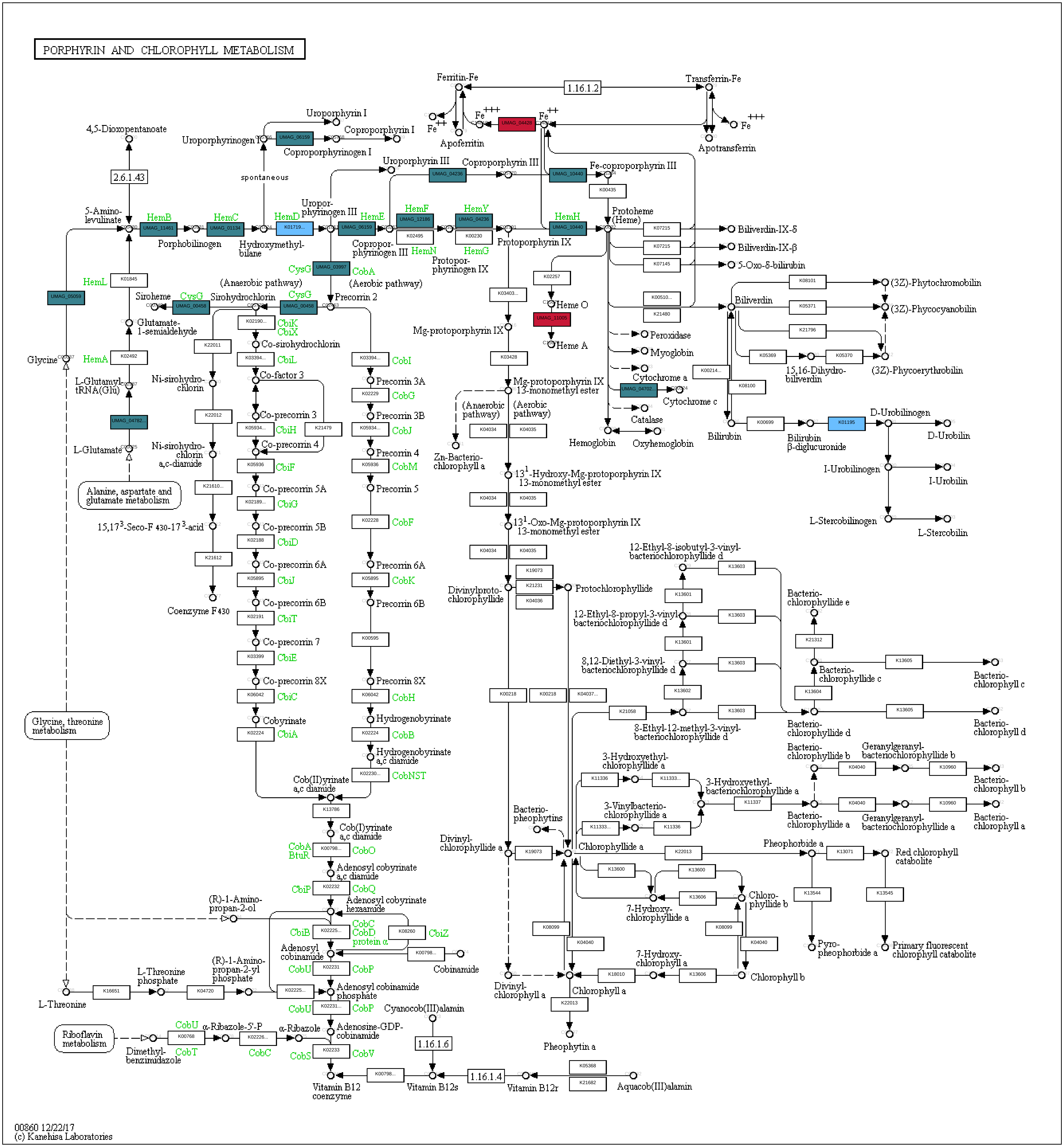

PORPHYRIN AND CHLOROPHYLL METABOLISM
Ferritin-Fe
1.16.1.2
Transferrin-Fe
Apoferritin
Apotransferrin
UMAG_04428
Uroporphyrin I
Uroporphyrinogen I
Coproporphyrin I
Coproporphyrinogen I
UMAG_06159
4,5-Dioxopentanoate
2.6.1.43
spontaneous
Uroporphyrin III
Coproporphyrin III
UMAG_04236
UMAG_10440
Fe-coproporphyrin III
K00435
5-Amino-levulinate
HemB
HemC
HemD
HemE
HemF
HemY
HemH
UMAG_11461
UMAG_01134
K01719...
UMAG_06159
UMAG_12186
UMAG_04236
UMAG_10440
K02495
K00230
Porphobilinogen
Hydroxymethyl-bilane
Uropor-phyrinogen III
Coprопor-phyrinogen III
HemN
HemG
Protopor-phyrinogen IX
Protoporphyrin IX
Protoheme (Heme)
K07215
Biliverdin-IX-δ
Biliverdin-IX-β
K07145
5-Oxo-δ-bilirubin
K08101
(3Z)-Phytochromobilin
(3Z)-Phycocyanobilin
K05371
Biliverdin
K00510...
K21480
K21796
HemL
K01845
UMAG_05059
CysG
UMAG_03997
CobA
(Anaerobic pathway)
(Aerobic pathway)
Siroheme
Sirohydrochlorin
Precorrin 2
UMAG_00458
K02257
Heme O
UMAG_11005
Heme A
K03403...
Mg-protoporphyrin IX
K03428
Glutamate-1-semialdehyde
K02190...
CbiK
CbiX
Co-sirohydrochlorin
K22011
Glycine
HemA
K02492
K03394...
CbiL
K03394...
CobI
Peroxidase
Myoglobin
K00214...
Cytochrome a
UMAG_04702
Cytochrome c
K05369
K05370
15,16-Dihydro-biliverdin
(3Z)-Phycoerythrobilin
K08100
L-Glutamyl-tRNA(Glu)
UMAG_04782...
Ni-sirohydro-chlorin
Co-factor 3
Precorrin 3A
K02229
CobG
Mg-protoporphyrin IX 13-monomethyl ester
(Anaerobic pathway)
(Aerobic pathway)
K04034
K04035
Catalase
Hemoglobin
Oxyhemoglobin
Bilirubin
K00699
Bilirubin β-diglucuronide
K01195
D-Urobilinogen
D-Urobilin
K22012
Co-precorrin 3
K05934...
CbiH
K21479
K05934...
CobJ
Precorrin 3B
Precorrin 4
Co-precorrin 4
L-Glutamate
Ni-sirohydro-chlorin a,c-diamide
K05936
CbiF
K05936
CobM
Zn-Bacterio-chlorophyll a
$13^1$-Hydroxy-Mg-protoporphyrin IX 13-monomethyl ester
I-Urobilinogen
I-Urobilin
Alanine, aspartate and glutamate metabolism
K21610...
Co-precorrin 5A
K02189...
CbiG
Precorrin 5
K04034
K04035
$15,17^3$-Seco-F 430-$17^3$-acid
Co-precorrin 5B
K02188
CbiD
K02228
CobF
$13^1$-Oxo-Mg-protoporphyrin IX 13-monomethyl ester
L-Stercobilinogen
L-Stercobilin
K21612
Co-precorrin 6A
K05895
CbiJ
Precorrin 6A
K05895
CobK
K04034
K04035
12-Ethyl-8-isobutyl-3-vinyl-bacteriochlorophyllide d
K13603
K19073
K21231
K04036
Divinylproto-chlorophyllide
Protochlorophyllide
K13601
Bacterio-chlorophyllide e
Coenzyme F430
Co-precorrin 6B
K02191
CbiT
Precorrin 6B
12-Ethyl-8-propyl-3-vinyl-bacteriochlorophyllide d
K13603
K00595
K21312
Co-precorrin 7
K03399
CbiE
K13601
8,12-Diethyl-3-vinyl-bacteriochlorophyllide d
K13603
Bacterio-chlorophyllide c
K13605
Bacterio-chlorophyll c
Co-precorrin 8X
K06042
CbiC
Precorrin 8X
K06042
CobH
Hydrogenobyrinate
K13602
K13604
K00218
K00218
K04037...
K21058
K13603
K13605
Glycine, threonine metabolism
Cobyrinate
K02224
CbiA
K02224
CobB
8-Ethyl-12-methyl-3-vinyl-bacteriochlorophyllide d
Bacterio-chlorophyllide d
Bacterio-chlorophyll d
Hydrogenobyrinate a,c diamide
K02230...
CobNST
3-Hydroxyethyl-chlorophyllide a
Bacterio-chlorophyllide b
Geranylgeranyl-bacteriochlorophyllide b
K04040
K10960
Bacterio-chlorophyll b
Cob(II)yrinate a,c diamide
K11336
K11333...
3-Hydroxyethyl-bacteriochlorophyllide a
K13786
Bacterio-pheophytins
3-Vinylbacterio-chlorophyllide a
K11337
K04040
K10960
Bacterio-chlorophyllide a
Geranylgeranyl-bacteriochlorophyllide a
Bacterio-chlorophyll a
Cob(I)yrinate a,c diamide
K11333...
K11336
CobA
BtuR
K00798...
CobO
Divinyl-chlorophyllide a
K19073
Chlorophyllide a
K22013
Pheophorbide a
K13071
Red chlorophyll catabolite
Adenosyl cobyrinate a,c diamide
K13600
K13600
CbiP
K02232
CobQ
(R)-1-Amino-propan-2-ol
Adenosyl cobyrinate hexaamide
K13606
Chloro-phyllide b
7-Hydroxy-chlorophyllide a
K13544
K13545
CbiB
K02225...
CobC
CobD
protein α
K08260
CbiZ
K08099
K08099
Adenosyl cobinamide
K00798...
Cobinamide
K04040
K04040
Pyro-pheophorbide a
Primary fluorescent chlorophyll catabolite
K02231
CobU
CobP
7-Hydroxy-chlorophyll a
L-Threonine phosphate
(R)-1-Amino-propan-2-yl phosphate
K16651
K04720
K02225...
Adenosyl cobinamide phosphate
Divinyl-chlorophyll a
K18010
K13606
Chlorophyll b
L-Threonine
Chlorophyll a
K22013
CobU
K02231...
CobP
Cyanocob(III)alamin
Riboflavin metabolism
α-Ribazole-5'-P
α-Ribazole
K00768
K02226...
Adenosine-GDP-cobinamide
1.16.1.6
Dimethyl-benzimidazole
CobT
CobC
CobS
K02233
CobV
Pheophytin a
K05368
K21682
K00798...
1.16.1.4
Vitamin B12 coenzyme
Vitamin B12s
Vitamin B12r
Aquacob(III)alamin
00860 12/22/17
(c) Kanehisa Laboratories

# 9. Metabolism of terpenoids and polyketides

| MAP | PATHWAY |
|---|---|
| **900** | Terpenoid backbone biosynthesis |
| **909** | Sesquiterpenoid and triterpenoid biosynthesis |

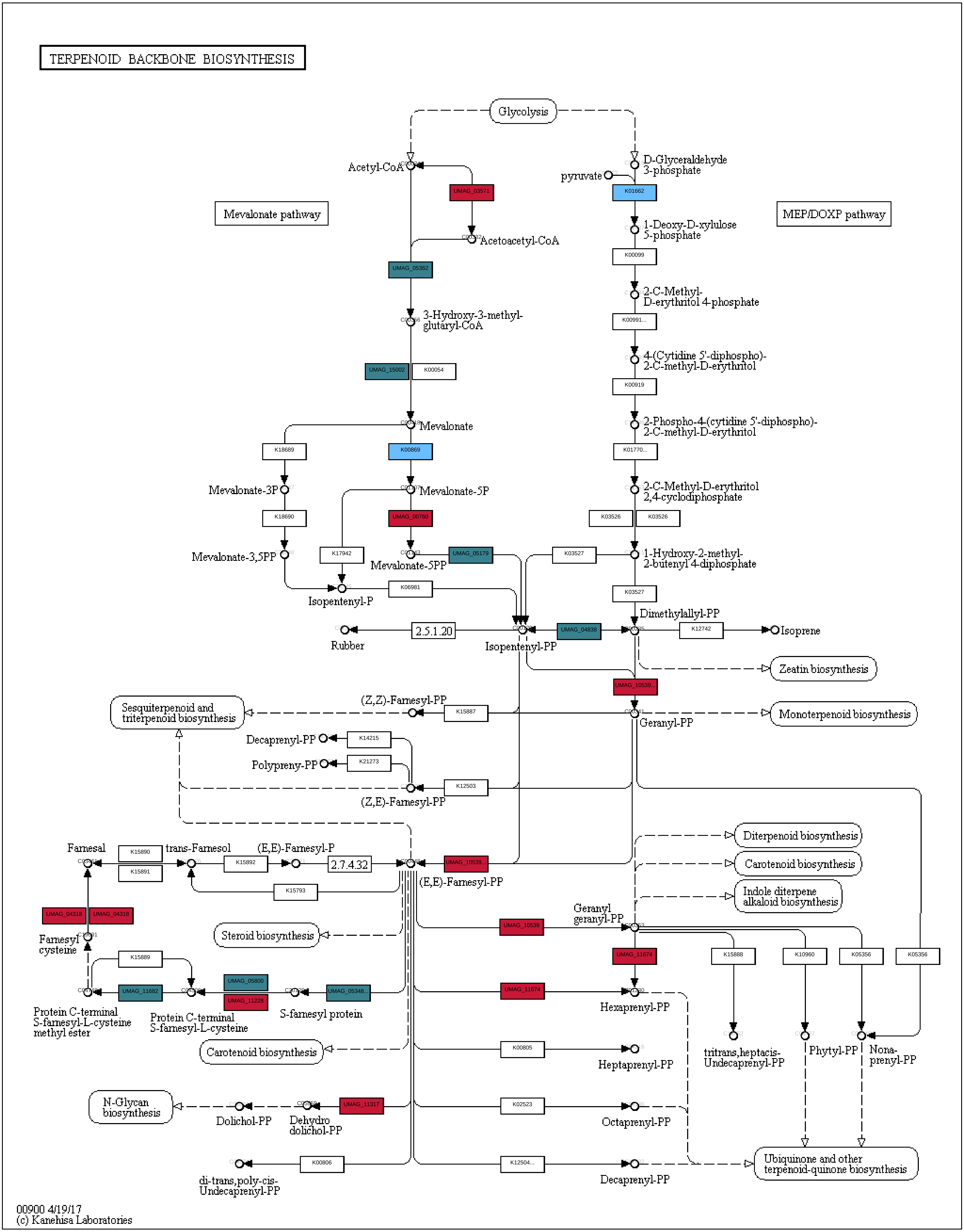
TERPENOID BACKBONE BIOSYNTHESIS
Glycolysis
Acetyl-CoA
UMAG_03571
Acetoacetyl-CoA
UMAG_05362
3-Hydroxy-3-methyl-
glutaryl-CoA
UMAG_15002
K00054
Mevalonate
Mevalonate pathway
K18689
K00869
Mevalonate-3P
Mevalonate-5P
K18690
UMAG_00760
K17942
Mevalonate-3,5PP
Mevalonate-5PP
UMAG_05179
Isopentenyl-P
K06981
D-Glyceraldehyde
3-phosphate
pyruvate
K01662
1-Deoxy-D-xylulose
5-phosphate
K00099
2-C-Methyl-
D-erythritol 4-phosphate
K00991...
4-(Cytidine 5'-diphospho)-
2-C-methyl-D-erythritol
K00919
2-Phospho-4-(cytidine 5'-diphospho)-
2-C-methyl-D-erythritol
K01770...
2-C-Methyl-D-erythritol
2,4-cyclodiphosphate
K03526
K03526
1-Hydroxy-2-methyl-
2-butenyl 4-diphosphate
K03527
K03527
MEP/DOXP pathway
Dimethylallyl-PP
Rubber
2.5.1.20
Isopentenyl-PP
UMAG_04838
K12742
Isoprene
Zeatin biosynthesis
UMAG_10539...
Monoterpenoid biosynthesis
Geranyl-PP
Sesquiterpenoid and
triterpenoid biosynthesis
(Z,Z)-Farnesyl-PP
K15887
Decaprenyl-PP
K14215
Polypreny-PP
K21273
K12503
(Z,E)-Farnesyl-PP
Farnesal
K15890
K15891
trans-Farnesol
K15892
(E,E)-Farnesyl-P
2.7.4.32
UMAG_10539...
(E,E)-Farnesyl-PP
K15793
Diterpenoid biosynthesis
Carotenoid biosynthesis
Indole diterpene
alkaloid biosynthesis
UMAG_04318
UMAG_04318
Farnesyl
cysteine
Steroid biosynthesis
UMAG_10539
Geranyl
geranyl-PP
K15889
UMAG_11682
UMAG_05800
UMAG_11228
UMAG_05348...
UMAG_11674
K15888
K10960
K05356
K05356
UMAG_11674
Protein C-terminal
S-farnesyl-L-cysteine
methyl ester
Protein C-terminal
S-farnesyl-L-cysteine
S-farnesyl protein
Hexaprenyl-PP
Carotenoid biosynthesis
K00805
Heptaprenyl-PP
tritrans,heptacis-
Undecaprenyl-PP
Phytyl-PP
Nona-
prenyl-PP
N-Glycan
biosynthesis
UMAG_11317
Dolichol-PP
Dehydro
dolichol-PP
K02523
Octaprenyl-PP
Ubiquinone and other
terpenoid-quinone biosynthesis
K00806
K12504...
di-trans,poly-cis-
Undecaprenyl-PP
Decaprenyl-PP
00900 4/19/17
(c) Kanehisa Laboratories

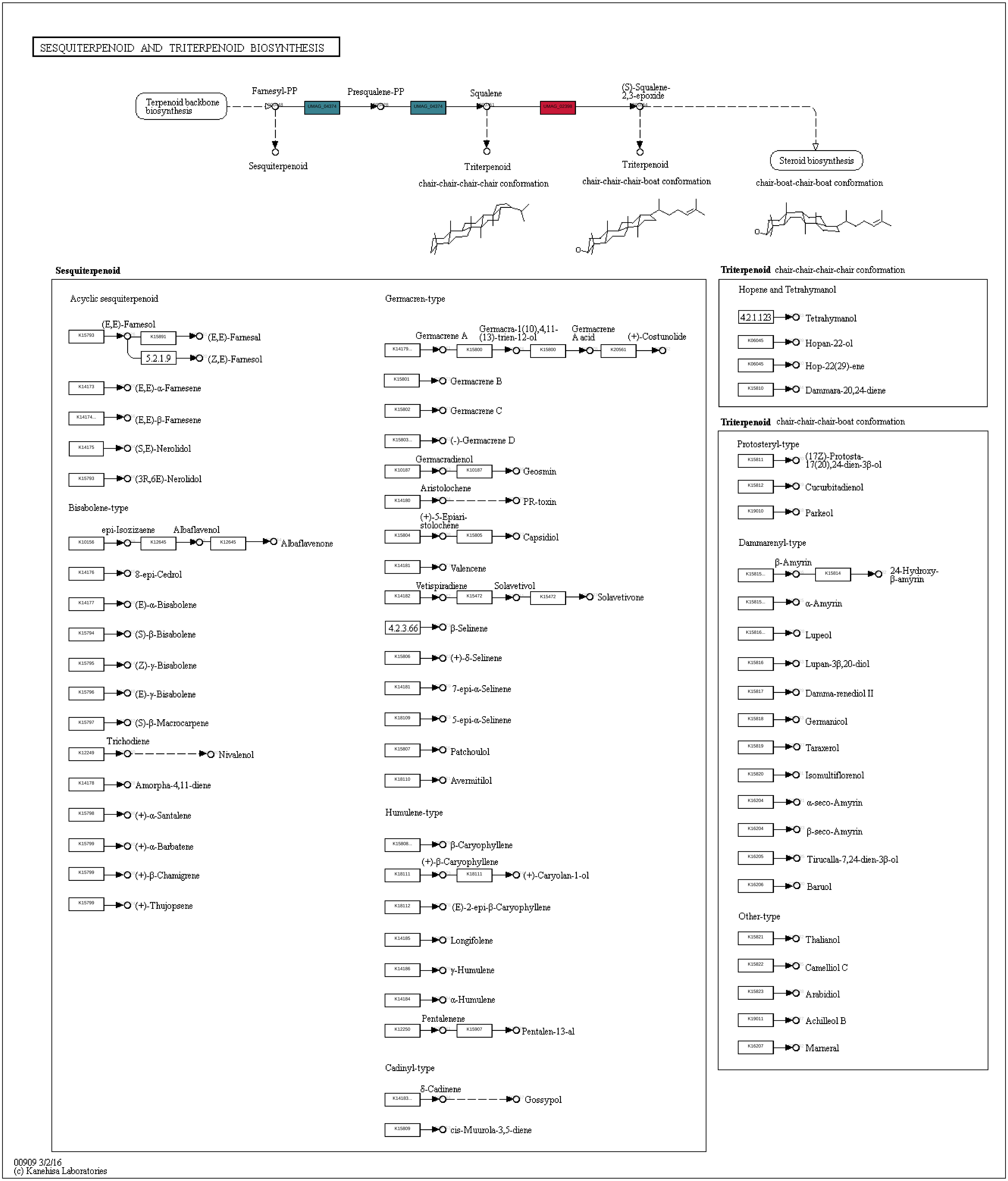
SESQUITERPENOID AND TRITERPENOID BIOSYNTHESIS
Terpenoid backbone biosynthesis
Farnesyl-PP
UMAG_04374
Presqualene-PP
UMAG_04374
Squalene
UMAG_02398
(S)-Squalene-2,3-epoxide
Sesquiterpenoid
Triterpenoid
chair-chair-chair-chair conformation
Triterpenoid
chair-chair-chair-boat conformation
Steroid biosynthesis
chair-boat-chair-boat conformation
Sesquiterpenoid
Acyclic sesquiterpenoid
(E,E)-Farnesol
K15793
K15891
(E,E)-Farnesal
5.2.1.9
(Z,E)-Farnesol
K14173
(E,E)-α-Farnesene
K14174...
(E,E)-β-Farnesene
K14175
(S,E)-Nerolidol
K15793
(3R,6E)-Nerolidol
Bisabolene-type
epi-Isozizaene
Albaflavenol
K10156
K12645
K12645
Albaflavenone
K14176
8-epi-Cedrol
K14177
(E)-α-Bisabolene
K15794
(S)-β-Bisabolene
K15795
(Z)-γ-Bisabolene
K15796
(E)-γ-Bisabolene
K15797
(S)-β-Macrocarpene
Trichodiene
K12249
Nivalenol
K14178
Amorpha-4,11-diene
K15798
(+)-α-Santalene
K15799
(+)-α-Barbatene
K15799
(+)-β-Chamigrene
K15799
(+)-Thujopsene
Germacren-type
Germacrene A
Germacra-1(10),4,11-(13)-trien-12-ol
Germacrene A acid
(+)-Costunolide
K14179...
K15800
K15800
K20561
K15801
Germacrene B
K15802
Germacrene C
K15803...
(-)-Germacrene D
Germacradienol
K10187
K10187
Geosmin
Aristolochene
K14180
PR-toxin
(+)-5-Epiaristolochene
K15804
K15805
Capsidiol
K14181
Valencene
Vetispiradiene
Solavetivol
K14182
K15472
K15472
Solavetivone
4.2.3.66
β-Selinene
K15806
(+)-δ-Selinene
K14181
7-epi-α-Selinene
K18109
5-epi-α-Selinene
K15807
Patchoulol
K18110
Avermitilol
Humulene-type
K15808...
β-Caryophyllene
(+)-β-Caryophyllene
K18111
K18111
(+)-Caryolan-1-ol
K18112
(E)-2-epi-β-Caryophyllene
K14185
Longifolene
K14186
γ-Humulene
K14184
α-Humulene
Pentalenene
K12250
K15907
Pentalen-13-al
Cadinyl-type
δ-Cadinene
K14183...
Gossypol
K15809
cis-Muurola-3,5-diene
Triterpenoid chair-chair-chair-chair conformation
Hopene and Tetrahymanol
4.2.1.123
Tetrahymanol
K06045
Hopan-22-ol
K06045
Hop-22(29)-ene
K15810
Dammara-20,24-diene
Triterpenoid chair-chair-chair-boat conformation
Protosteryl-type
K15811
(17Z)-Protosta-17(20),24-dien-3β-ol
K15812
Cucurbitadienol
K19010
Parkeol
Dammarenyl-type
β-Amyrin
K15815...
K15814
24-Hydroxy-β-amyrin
K15815...
α-Amyrin
K15816...
Lupeol
K15816
Lupan-3β,20-diol
K15817
Damma-renediol II
K15818
Germanicol
K15819
Taraxerol
K15820
Isomultiflorenol
K16204
α-seco-Amyrin
K16204
β-seco-Amyrin
K16205
Tirucalla-7,24-dien-3β-ol
K16206
Baruol
Other-type
K15821
Thalianol
K15822
Camelliol C
K15823
Arabidiol
K19011
Achilleol B
K16207
Marneral
00909 3/2/16
(c) Kanehisa Laboratories

# 10. Biosynthesis of other secondary metabolites

| MAP | PATHWAY |
|---|---|
| **261** | Monobactam biosynthesis |
| **332** | Carbapenem biosynthesis |

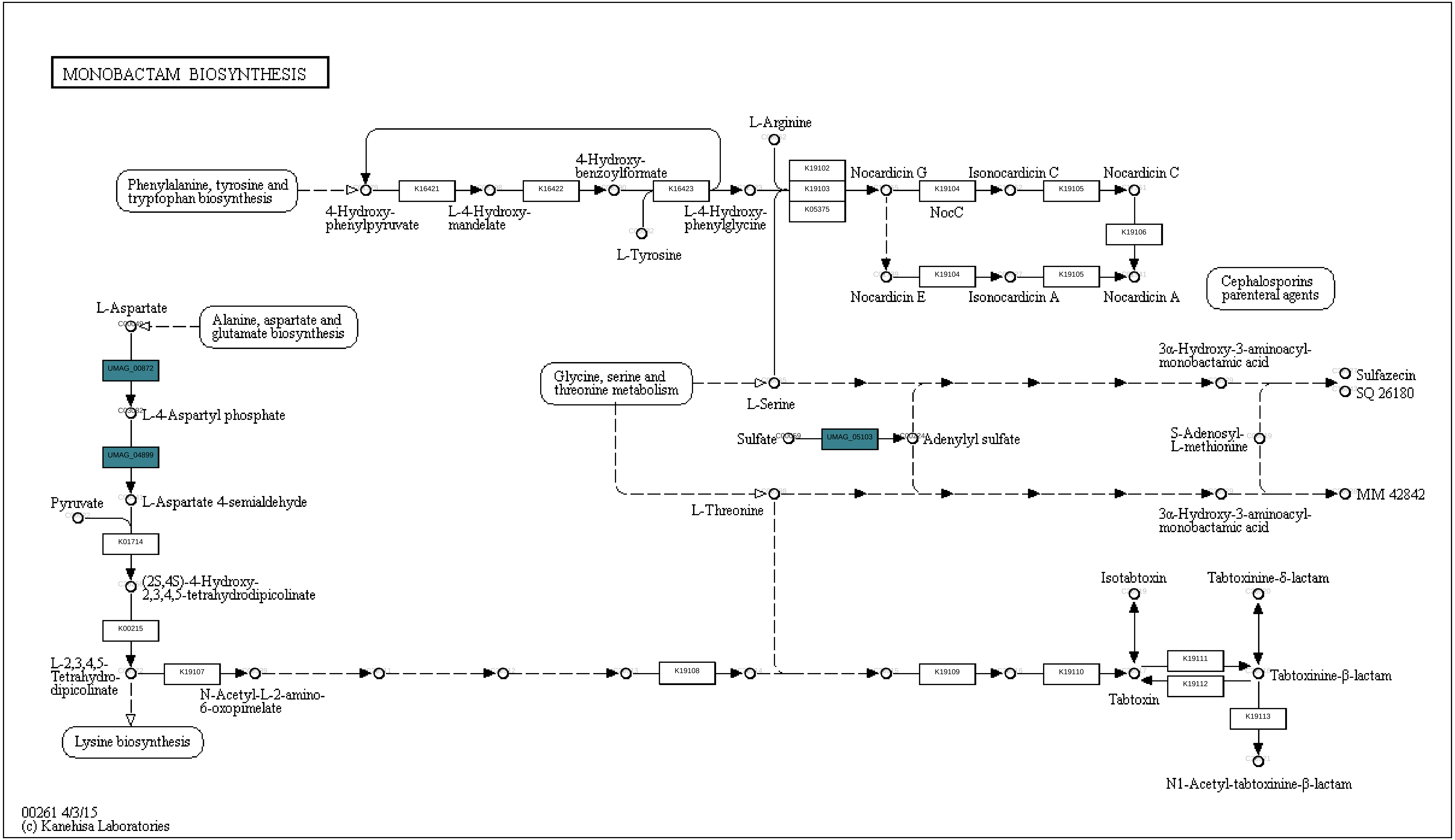

MONOBACTAM BIOSYNTHESIS
Phenylalanine, tyrosine and tryptophan biosynthesis
4-Hydroxy-phenylpyruvate
K16421
L-4-Hydroxy-mandelate
K16422
4-Hydroxy-benzoylformate
K16423
L-Tyrosine
L-4-Hydroxy-phenylglycine
L-Arginine
K19102
K19103
K05375
Nocardicin G
K19104
NocC
Isonocardicin C
K19105
Nocardicin C
K19106
Nocardicin E
Isonocardicin A
Nocardicin A
Cephalosporins parenteral agents
L-Aspartate
Alanine, aspartate and glutamate biosynthesis
UMAG_00872
L-4-Aspartyl phosphate
UMAG_04899
L-Aspartate 4-semialdehyde
Pyruvate
K01714
(2S,4S)-4-Hydroxy-2,3,4,5-tetrahydrodipicolinate
K00215
L-2,3,4,5-Tetrahydro-dipicolinate
K19107
N-Acetyl-L-2-amino-6-oxopimelate
Lysine biosynthesis
Glycine, serine and threonine metabolism
L-Serine
Sulfate
UMAG_05103
Adenylyl sulfate
L-Threonine
3α-Hydroxy-3-aminoacyl-monobactamic acid
Sulfazecin
SQ 26180
S-Adenosyl-L-methionine
MM 42842
3α-Hydroxy-3-aminoacyl-monobactamic acid
K19108
K19109
K19110
Isotabtoxin
Tabtoxinine-δ-lactam
K19111
K19112
Tabtoxin
Tabtoxinine-β-lactam
K19113
N1-Acetyl-tabtoxinine-β-lactam
00261 4/3/15
(c) Kanehisa Laboratories

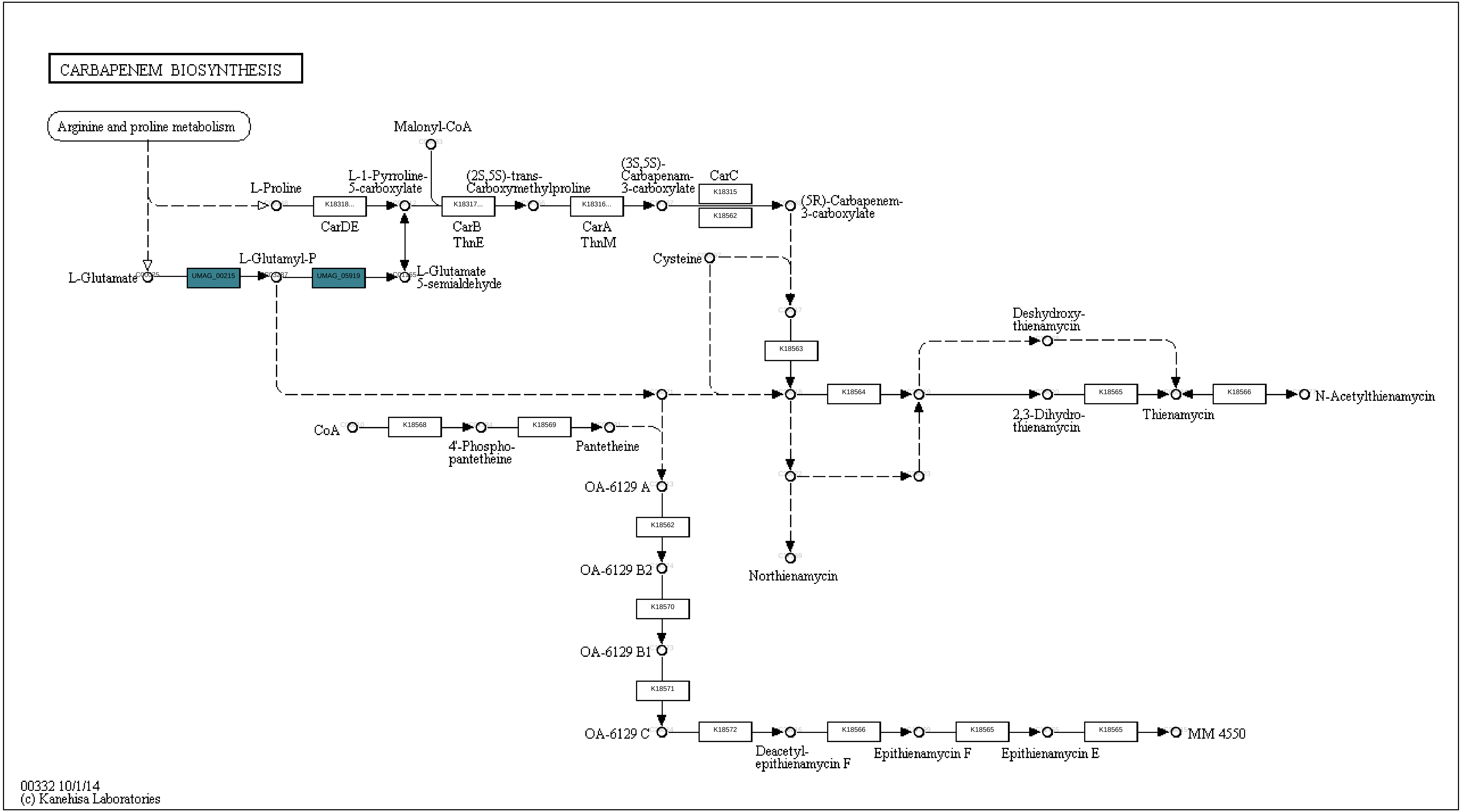

CARBAPENEM BIOSYNTHESIS
Arginine and proline metabolism
Malonyl-CoA
L-Proline
L-1-Pyrroline-5-carboxylate
(2S,5S)-trans-Carboxymethylproline
(3S,5S)-Carbapenam-3-carboxylate
CarC
K18315
K18562
(5R)-Carbapenem-3-carboxylate
K18318...
K18317...
K18316...
CarDE
CarB
ThnE
CarA
ThnM
L-Glutamyl-P
L-Glutamate
UMAG_00215
UMAG_05919
L-Glutamate 5-semialdehyde
Cysteine
K18563
Deshydroxy-thienamycin
K18564
K18565
K18566
N-Acetylthienamycin
Thienamycin
2,3-Dihydro-thienamycin
CoA
K18568
K18569
4'-Phospho-pantetheine
Pantetheine
OA-6129 A
K18562
OA-6129 B2
K18570
OA-6129 B1
K18571
OA-6129 C
K18572
K18566
K18565
K18565
Northienamycin
Deacetyl-epithienamycin F
Epithienamycin F
Epithienamycin E
MM 4550
00332 10/1/14
(c) Kanehisa Laboratories

# 11. Xenobiotics biodegradation and metabolism

| MAP | PATHWAY |
|---|---|
| **791** | Atrazine degradation |

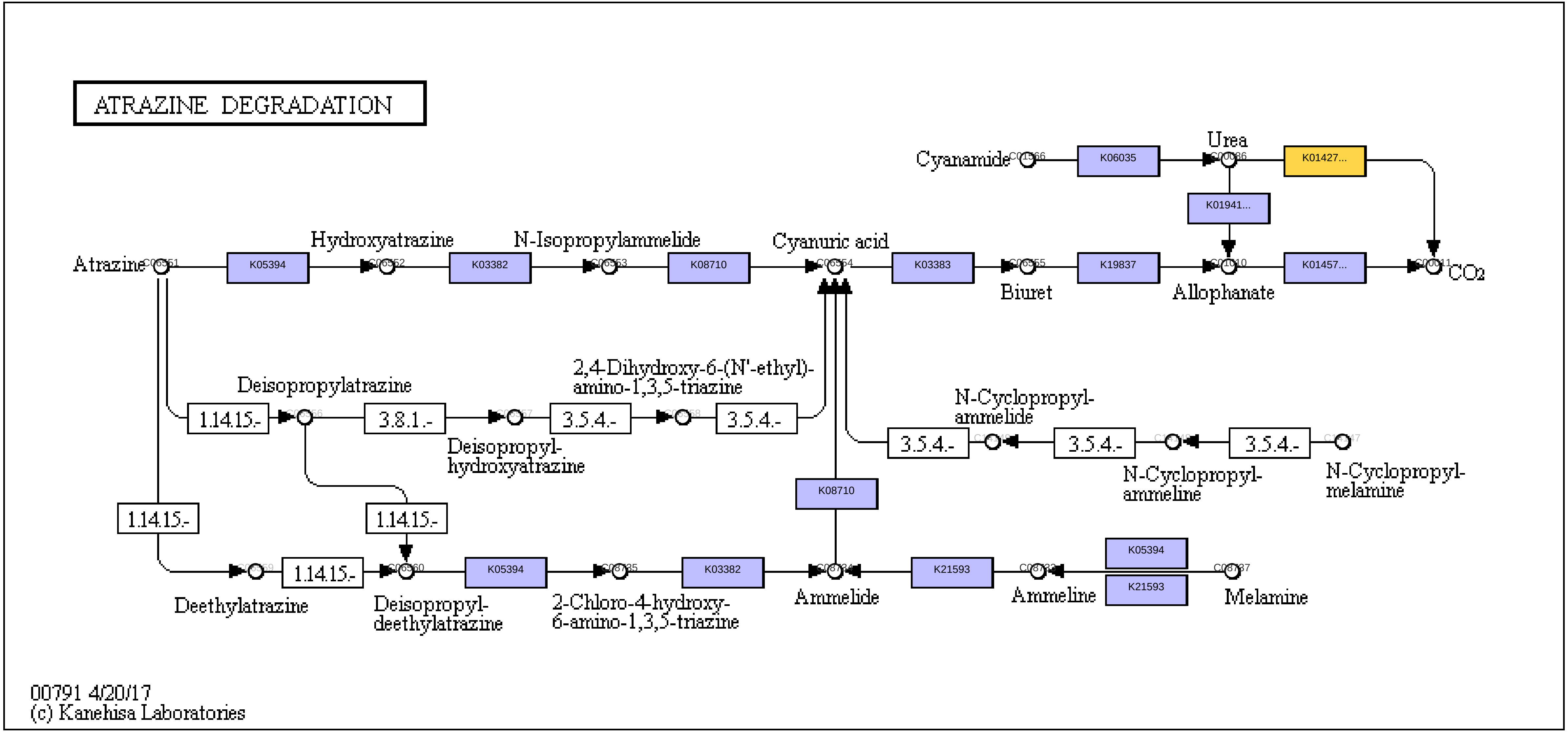

ATRAZINE DEGRADATION
Cyanamide
K06035
Urea
K01427...
K01941...
Atrazine
K05394
Hydroxyatrazine
K03382
N-Isopropylammelide
K08710
Cyanuric acid
K03383
Biuret
K19837
Allophanate
K01457...
CO2
Deisopropylatrazine
1.14.15.-
3.8.1.-
Deisopropyl-
hydroxyatrazine
2,4-Dihydroxy-6-(N'-ethyl)-
amino-1,3,5-triazine
3.5.4.-
3.5.4.-
N-Cyclopropyl-
ammelide
3.5.4.-
3.5.4.-
3.5.4.-
N-Cyclopropyl-
ammeline
N-Cyclopropyl-
melamine
K08710
1.14.15.-
1.14.15.-
1.14.15.-
Deethylatrazine
Deisopropyl-
deethylatrazine
K05394
2-Chloro-4-hydroxy-
6-amino-1,3,5-triazine
K03382
Ammelide
K21593
Ammeline
K05394
K21593
Melamine
00791 4/20/17
(c) Kanehisa Laboratories

**KEGG pathway analysis**

# *Positive controls*

**Supplementary file 4**

**The following color codes are used:**

| Color | Meaning |
|---|---|
| Dark teal | True positive (TP): element in the reference pathway correctly predicted by Interproscan |
| White | True negative (TN): element in the *not* reference pathway and *not* predicted by Interproscan |
| Red | False negative (FN): element in the reference pathway but *not* predicted by Interproscan. |
| Light blue | False positive (FP): element in the *not* reference pathway but predicted by Interproscan. Note: could also be a valid new prediction |

**Species:** ***Puccinia graminis f.sp. tritici***

**Acronym:** **PGR**

**Lifestyle:** **Obligate biotrophic (rust)**

# 1. Carbohydrate metabolism

| MAP | PATHWAY |
|---|---|
| **10** | Glycolysis / Gluconeogenesis |
| **20** | Citrate cycle (TCA cycle) |
| **30** | Pentose phosphate pathway |
| **40** | Pentose and glucuronate interconversions |
| **51** | Fructose and mannose metabolism |
| **52** | Galactose metabolism |
| **53** | Ascorbate and aldarate metabolism |
| **500** | Starch and sucrose metabolism |
| **520** | Amino sugar and nucleotide sugar metabolism |
| **562** | Inositol phosphate metabolism |
| **620** | Pyruvate metabolism |
| **630** | Glyoxylate and dicarboxylate metabolism |
| **640** | Propanoate metabolism |
| **650** | Butanoate metabolism |
| **660** | C5-Branched dibasic acid metabolism |

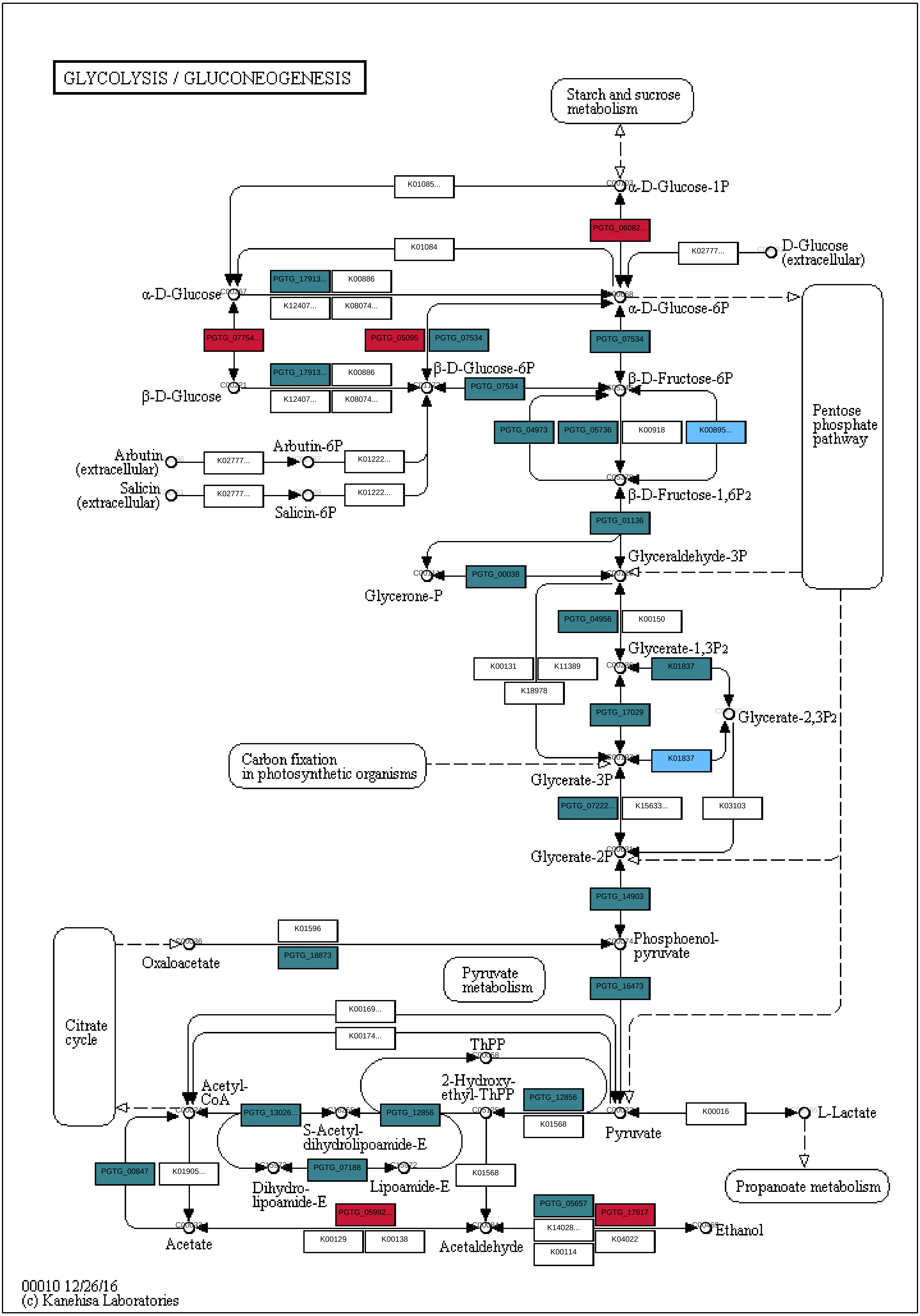
GLYCOLYSIS / GLUCONEOGENESIS
Starch and sucrose metabolism
α-D-Glucose-1P
K01085...
PGTG_06082...
K01084
K02777...
D-Glucose (extracellular)
α-D-Glucose
PGTG_17913...
K00886
K12407...
K08074...
α-D-Glucose-6P
PGTG_07754...
PGTG_05095
PGTG_07534
β-D-Glucose-6P
β-D-Glucose
β-D-Fructose-6P
PGTG_04973
PGTG_05736
K00918
K00895...
Pentose phosphate pathway
Arbutin (extracellular)
Arbutin-6P
K01222...
Salicin (extracellular)
Salicin-6P
β-D-Fructose-1,6P2
PGTG_01136
Glyceraldehyde-3P
PGTG_00038
Glycerone-P
PGTG_04956
K00150
Glycerate-1,3P2
K00131
K11389
K18978
K01837
PGTG_17029
Glycerate-2,3P2
Carbon fixation in photosynthetic organisms
Glycerate-3P
PGTG_07222...
K15633...
K03103
Glycerate-2P
PGTG_14903
K01596
PGTG_18873
Phosphoenol-pyruvate
Oxaloacetate
Citrate cycle
Pyruvate metabolism
PGTG_16473
K00169...
K00174...
ThPP
2-Hydroxy-ethyl-ThPP
Acetyl-CoA
PGTG_13026...
PGTG_12856
K01568
K00016
L-Lactate
Pyruvate
S-Acetyl-dihydrolipoamide-E
PGTG_00847
K01905...
PGTG_07188
Dihydro-lipoamide-E
Lipoamide-E
Propanoate metabolism
PGTG_05992...
PGTG_05657
PGTG_17917
K14028...
K04022
K00129
K00138
K00114
Acetate
Acetaldehyde
Ethanol
00010 12/26/16
(c) Kanehisa Laboratories

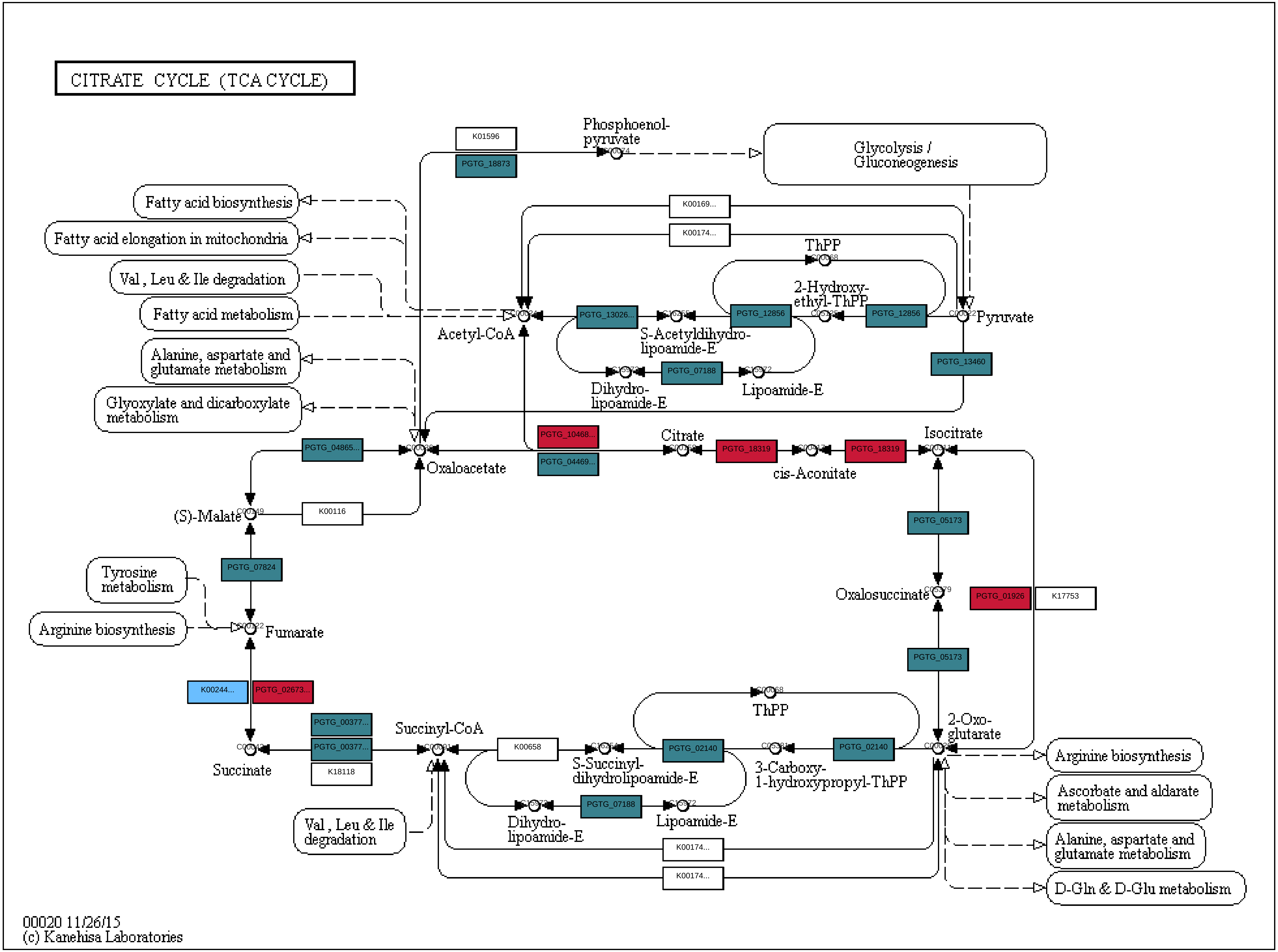
CITRATE CYCLE (TCA CYCLE)
Phosphoenol-pyruvate
K01596
PGTG_18873
Glycolysis / Gluconeogenesis
Fatty acid biosynthesis
Fatty acid elongation in mitochondria
Val, Leu & Ile degradation
Fatty acid metabolism
Alanine, aspartate and glutamate metabolism
Glyoxylate and dicarboxylate metabolism
K00169...
K00174...
ThPP
2-Hydroxy-ethyl-ThPP
PGTG_13026...
PGTG_12856
PGTG_12856
Acetyl-CoA
S-Acetyldihydro-lipoamide-E
Pyruvate
PGTG_07188
PGTG_13460
Dihydro-lipoamide-E
Lipoamide-E
PGTG_10468...
PGTG_04469...
PGTG_04865...
Oxaloacetate
Citrate
PGTG_18319
PGTG_18319
cis-Aconitate
Isocitrate
(S)-Malate
K00116
PGTG_05173
Tyrosine metabolism
PGTG_07824
Oxalosuccinate
PGTG_01926
K17753
Arginine biosynthesis
Fumarate
PGTG_05173
K00244...
PGTG_02673...
PGTG_00377...
PGTG_00377...
K18118
Succinate
Succinyl-CoA
K00658
ThPP
PGTG_02140
PGTG_02140
2-Oxo-glutarate
S-Succinyl-dihydrolipoamide-E
3-Carboxy-1-hydroxypropyl-ThPP
PGTG_07188
Dihydro-lipoamide-E
Lipoamide-E
Val, Leu & Ile degradation
K00174...
K00174...
Arginine biosynthesis
Ascorbate and aldarate metabolism
Alanine, aspartate and glutamate metabolism
D-Gln & D-Glu metabolism
00020 11/26/15
(c) Kanehisa Laboratories

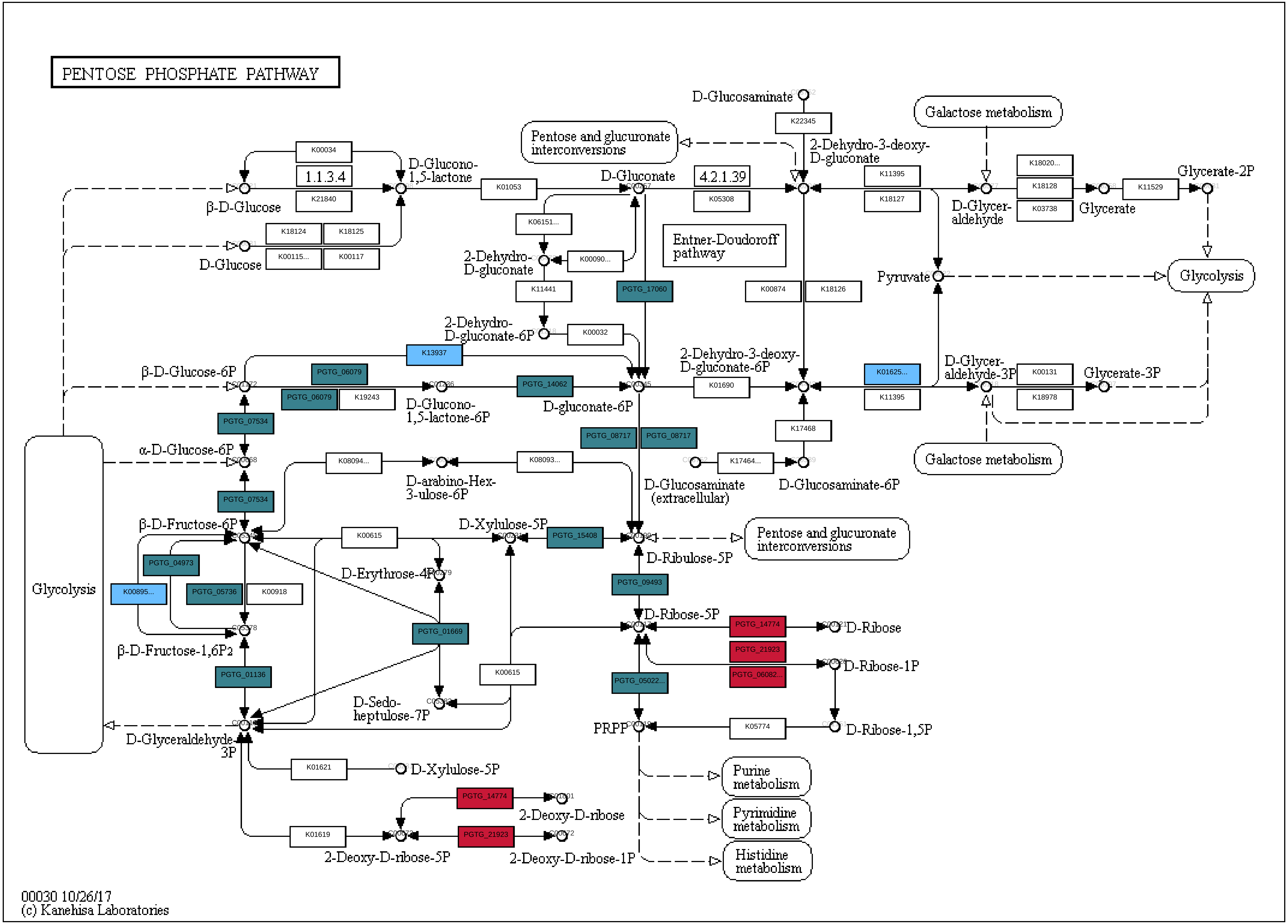
PENTOSE PHOSPHATE PATHWAY
D-Glucosaminate
K22345
Galactose metabolism
Pentose and glucuronate interconversions
2-Dehydro-3-deoxy-D-gluconate
K00034
D-Glucono-1,5-lactone
1.1.3.4
K21840
K01053
D-Gluconate
4.2.1.39
K05308
K11395
K18127
K18020...
K18128
K03738
K11529
Glycerate-2P
β-D-Glucose
D-Glycer-aldehyde
Glycerate
K18124
K18125
K06151...
Entner-Doudoroff pathway
K00115...
K00117
D-Glucose
2-Dehydro-D-gluconate
K00090...
Pyruvate
Glycolysis
K11441
PGTG_17060
K00874
K18126
2-Dehydro-D-gluconate-6P
K00032
K13937
2-Dehydro-3-deoxy-D-gluconate-6P
β-D-Glucose-6P
PGTG_06079
PGTG_14062
K01690
K01625...
D-Glycer-aldehyde-3P
K00131
Glycerate-3P
PGTG_06079
K19243
D-Glucono-1,5-lactone-6P
D-gluconate-6P
K11395
K18978
PGTG_07534
K17468
PGTG_08717
PGTG_08717
α-D-Glucose-6P
K08094...
K08093...
K17464...
Galactose metabolism
D-arabino-Hex-3-ulose-6P
D-Glucosaminate (extracellular)
D-Glucosaminate-6P
Glycolysis
PGTG_07534
β-D-Fructose-6P
D-Xylulose-5P
K00615
PGTG_15408
Pentose and glucuronate interconversions
D-Ribulose-5P
PGTG_04973
D-Erythrose-4P
K00895...
PGTG_05736
K00918
PGTG_09493
D-Ribose-5P
PGTG_14774
D-Ribose
PGTG_01669
β-D-Fructose-1,6P2
PGTG_21923
D-Ribose-1P
PGTG_01136
K00615
PGTG_05022...
PGTG_06082...
D-Sedo-heptulose-7P
PRPP
K05774
D-Ribose-1,5P
D-Glyceraldehyde-3P
K01621
D-Xylulose-5P
Purine metabolism
PGTG_14774
2-Deoxy-D-ribose
Pyrimidine metabolism
K01619
PGTG_21923
2-Deoxy-D-ribose-5P
2-Deoxy-D-ribose-1P
Histidine metabolism
00030 10/26/17
(c) Kanehisa Laboratories

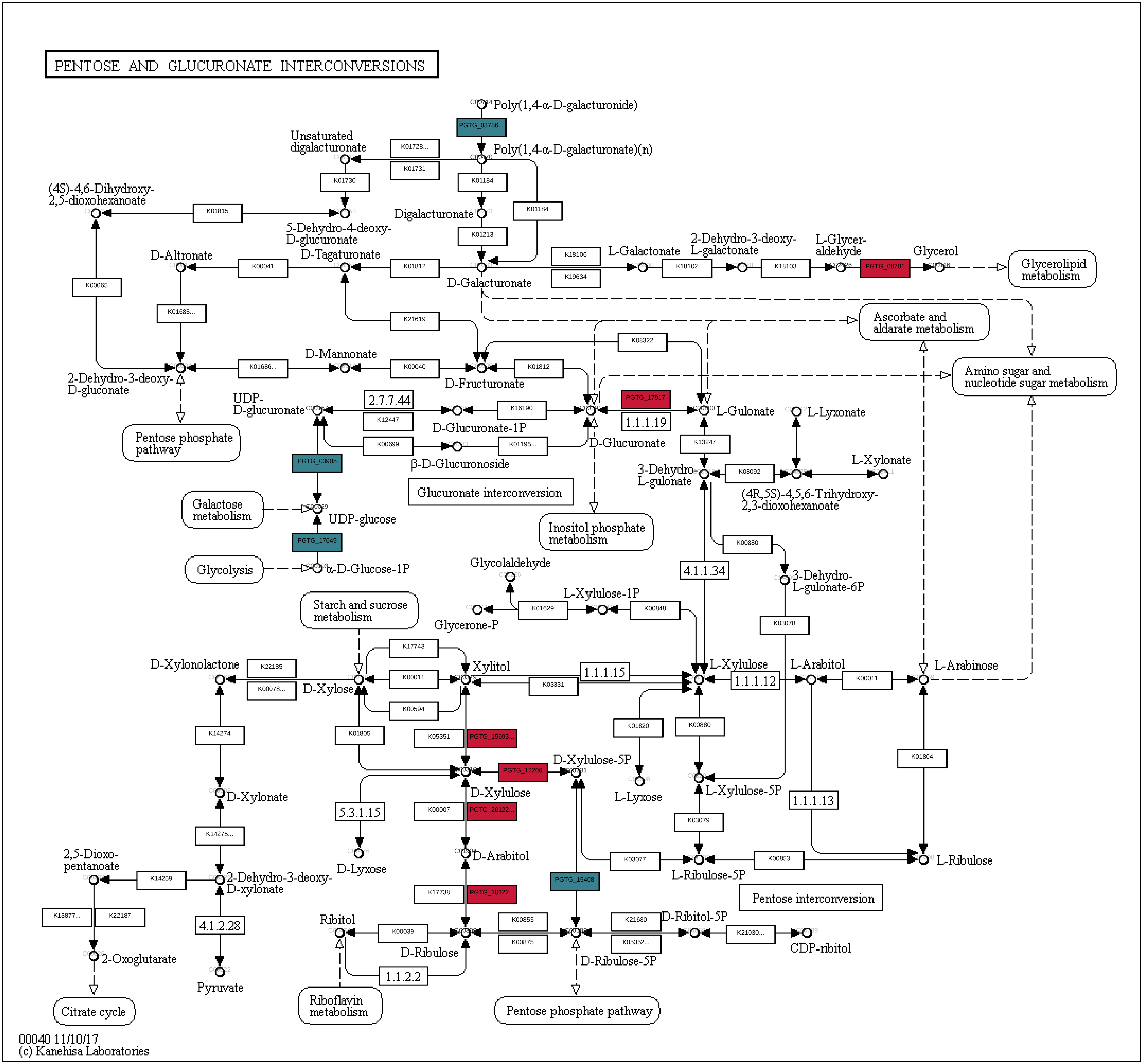

PENTOSE AND GLUCURONATE INTERCONVERSIONS
Poly(1,4-α-D-galacturonide)
PGTG_03786...
Poly(1,4-α-D-galacturonate)(n)
Unsaturated digalacturonate
K01728...
K01731
K01730
K01184
(4S)-4,6-Dihydroxy-2,5-dioxohexanoate
K01815
5-Dehydro-4-deoxy-D-glucuronate
Digalacturonate
K01184
K01213
D-Altronate
K00041
D-Tagaturonate
K01812
D-Galacturonate
K18106
K19634
L-Galactonate
K18102
2-Dehydro-3-deoxy-L-galactonate
K18103
L-Glyceraldehyde
PGTG_08701
Glycerol
Glycerolipid metabolism
K00065
K01685...
K21619
Ascorbate and aldarate metabolism
K08322
D-Mannonate
K01686...
K00040
K01812
D-Fructuronate
Amino sugar and nucleotide sugar metabolism
2-Dehydro-3-deoxy-D-gluconate
UDP-D-glucuronate
2.7.7.44
K12447
K16190
D-Glucuronate-1P
PGTG_17917
1.1.1.19
L-Gulonate
L-Lyxonate
Pentose phosphate pathway
K00699
K01195...
β-D-Glucuronoside
D-Glucuronate
K13247
PGTG_03905
3-Dehydro-L-gulonate
K08092
L-Xylonate
(4R,5S)-4,5,6-Trihydroxy-2,3-dioxohexanoate
Glucuronate interconversion
Galactose metabolism
UDP-glucose
Inositol phosphate metabolism
PGTG_17649
K00880
Glycolysis
α-D-Glucose-1P
Glycolaldehyde
4.1.1.34
3-Dehydro-L-gulonate-6P
L-Xylulose-1P
K01629
K00848
Glycerone-P
Starch and sucrose metabolism
K03078
K17743
D-Xylonolactone
K22185
K00078...
D-Xylose
K00011
Xylitol
1.1.1.15
K03331
L-Xylulose
1.1.1.12
L-Arabitol
K00011
L-Arabinose
K00594
K14274
K01805
K05351
PGTG_15693...
K01820
K00880
K01804
PGTG_12206
D-Xylulose-5P
L-Lyxose
D-Xylonate
D-Xylulose
L-Xylulose-5P
5.3.1.15
K00007
PGTG_20122...
1.1.1.13
K14275...
K03079
D-Lyxose
D-Arabitol
2,5-Dioxopentanoate
K14259
2-Dehydro-3-deoxy-D-xylonate
PGTG_15408
K03077
K00853
L-Ribulose
L-Ribulose-5P
K17738
PGTG_20122...
Pentose interconversion
K13877...
K22187
4.1.2.28
Ribitol
K00039
K00853
K00875
K21680
K05352...
D-Ribitol-5P
K21030...
CDP-ribitol
D-Ribulose
D-Ribulose-5P
2-Oxoglutarate
1.1.2.2
Pyruvate
Citrate cycle
Riboflavin metabolism
Pentose phosphate pathway
00040 11/10/17
(c) Kanehisa Laboratories

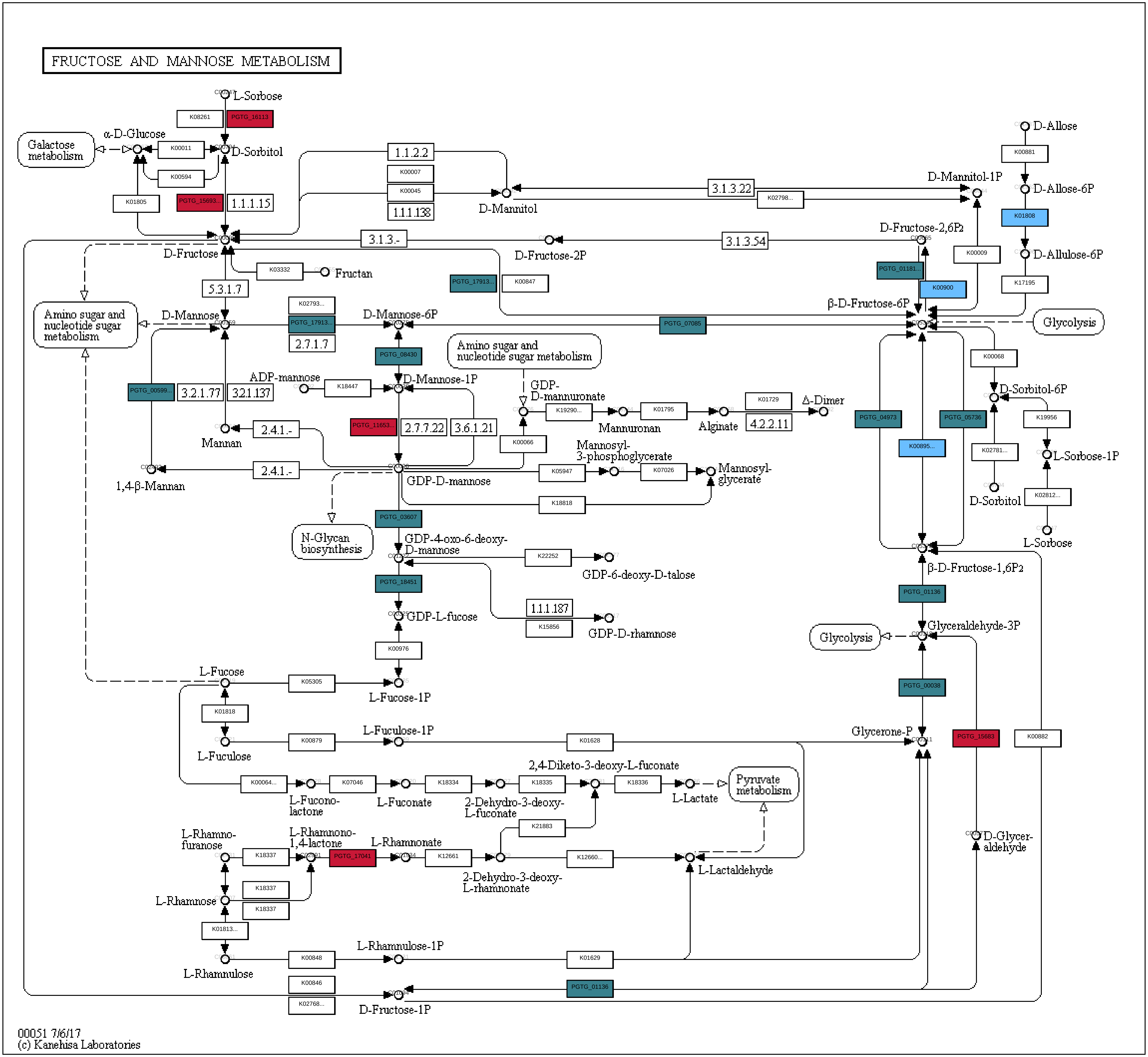
FRUCTOSE AND MANNOSE METABOLISM
L-Sorbose
K08261
PGTG_16113
Galactose metabolism
α-D-Glucose
K00011
D-Sorbitol
K00594
K01805
PGTG_15693...
1.1.1.15
1.1.2.2
K00007
K00045
1.1.1.138
D-Mannitol
3.1.3.22
K02798...
D-Mannitol-1P
D-Allose
K00881
D-Allose-6P
K01808
D-Allulose-6P
K17195
3.1.3.-
D-Fructose-2P
3.1.3.54
D-Fructose-2,6P2
K00009
D-Fructose
K03332
Fructan
5.3.1.7
PGTG_17913...
K00847
PGTG_01181...
K00900
β-D-Fructose-6P
Glycolysis
Amino sugar and nucleotide sugar metabolism
D-Mannose
K02793...
PGTG_17913...
2.7.1.7
D-Mannose-6P
PGTG_07085
PGTG_08430
Amino sugar and nucleotide sugar metabolism
K00068
D-Sorbitol-6P
PGTG_00599...
3.2.1.77
3.2.1.137
ADP-mannose
K18447
D-Mannose-1P
GDP-D-mannuronate
K19290...
Mannuronan
K01795
Alginate
K01729
4.2.2.11
Δ-Dimer
PGTG_04973
PGTG_05736
K19956
K00895...
K02781...
L-Sorbose-1P
Mannan
2.4.1.-
PGTG_11653...
2.7.7.22
3.6.1.21
K00066
Mannosyl-3-phosphoglycerate
K05947
K07026
Mannosyl-glycerate
D-Sorbitol
K02812...
1,4-β-Mannan
2.4.1.-
GDP-D-mannose
K18818
L-Sorbose
N-Glycan biosynthesis
PGTG_03607
GDP-4-oxo-6-deoxy-D-mannose
K22252
GDP-6-deoxy-D-talose
β-D-Fructose-1,6P2
PGTG_18451
1.1.1.187
K15856
GDP-D-rhamnose
PGTG_01136
GDP-L-fucose
Glyceraldehyde-3P
Glycolysis
K00976
L-Fucose
K05305
L-Fucose-1P
PGTG_00038
K01818
L-Fuculose-1P
Glycerone-P
PGTG_15683
K00882
L-Fuculose
K00879
K01628
2,4-Diketo-3-deoxy-L-fuconate
K00064...
K07046
K18334
K18335
K18336
Pyruvate metabolism
L-Fucono-lactone
L-Fuconate
2-Dehydro-3-deoxy-L-fuconate
L-Lactate
K21883
L-Rhamno-furanose
K18337
L-Rhamnono-1,4-lactone
PGTG_17041
L-Rhamnonate
K12661
K12660...
D-Glycer-aldehyde
L-Lactaldehyde
2-Dehydro-3-deoxy-L-rhamnonate
K18337
L-Rhamnose
K18337
K01813...
L-Rhamnulose-1P
K00848
K01629
L-Rhamnulose
K00846
PGTG_01136
K02768...
D-Fructose-1P
00051 7/6/17
(c) Kanehisa Laboratories

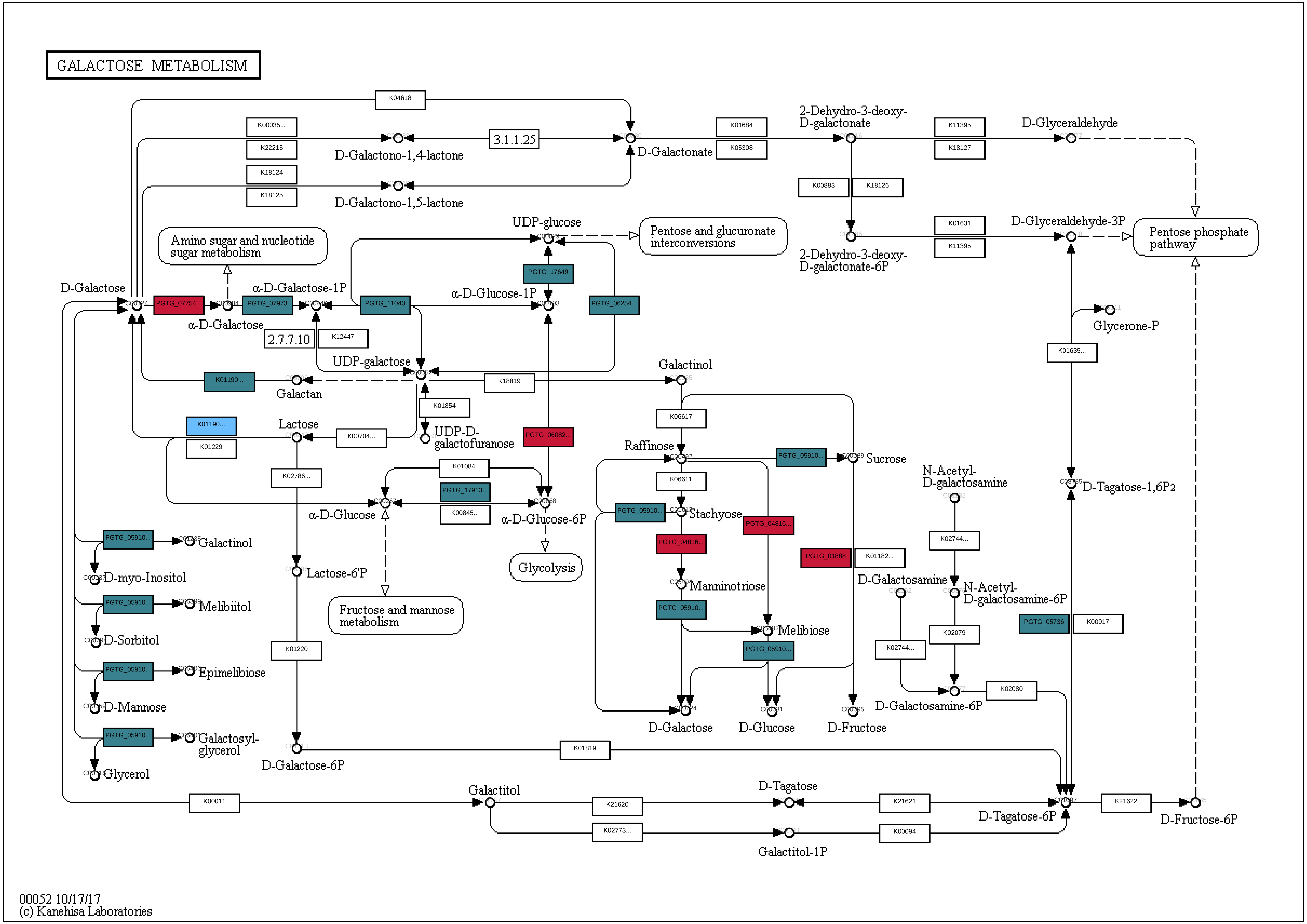
GALACTOSE METABOLISM
K04618
K00035...
K22215
K18124
K18125
D-Galactono-1,4-lactone
3.1.1.25
D-Galactonate
K01684
K05308
2-Dehydro-3-deoxy-
D-galactonate
K11395
K18127
D-Glyceraldehyde
D-Galactono-1,5-lactone
K00883
K18126
K01631
K11395
D-Glyceraldehyde-3P
2-Dehydro-3-deoxy-
D-galactonate-6P
Pentose phosphate
pathway
UDP-glucose
Pentose and glucuronate
interconversions
Amino sugar and nucleotide
sugar metabolism
PGTG_17649
D-Galactose
PGTG_07754...
α-D-Galactose-1P
PGTG_07973
PGTG_11040
α-D-Glucose-1P
PGTG_06254...
α-D-Galactose
2.7.7.10
K12447
Glycerone-P
K01635...
UDP-galactose
Galactinol
K01190...
Galactan
K18819
K01854
K06617
K01190...
K01229
Lactose
K00704...
UDP-D-
galactofuranose
PGTG_06082...
Raffinose
PGTG_05910...
Sucrose
K01084
K02786...
PGTG_17913...
K00845...
K06611
N-Acetyl-
D-galactosamine
D-Tagatose-1,6P2
α-D-Glucose
α-D-Glucose-6P
PGTG_05910...
Stachyose
PGTG_04816...
PGTG_05910...
Galactinol
PGTG_04816...
PGTG_01888
K01182...
K02744...
D-myo-Inositol
Lactose-6P
Glycolysis
Manninotriose
D-Galactosamine
N-Acetyl-
D-galactosamine-6P
PGTG_05910...
Melibiitol
Fructose and mannose
metabolism
PGTG_05910...
Melibiose
PGTG_05736
K00917
K02079
D-Sorbitol
K01220
K02744...
PGTG_05910...
PGTG_05910...
Epimelibiose
K02080
D-Mannose
D-Galactose
D-Glucose
D-Fructose
D-Galactosamine-6P
PGTG_05910...
Galactosyl-
glycerol
K01819
D-Galactose-6P
Glycerol
Galactitol
D-Tagatose
K00011
K21620
K21621
K21622
D-Tagatose-6P
D-Fructose-6P
K02773...
K00094
Galactitol-1P
00052 10/17/17
(c) Kanehisa Laboratories

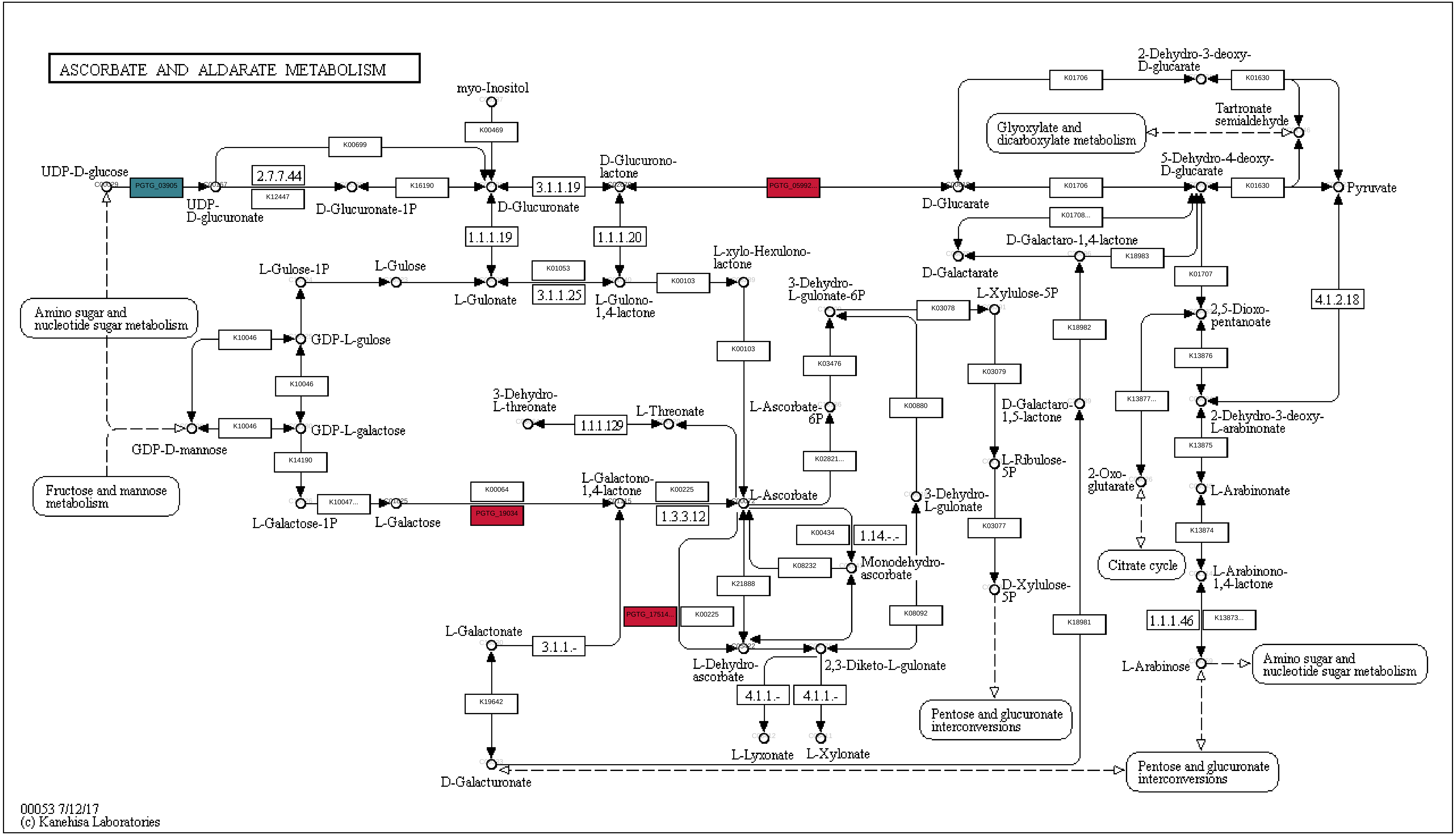

ASCORBATE AND ALDARATE METABOLISM
myo-Inositol
K00469
K00699
UDP-D-glucose
PGTG_03905
2.7.7.44
K12447
UDP-D-glucuronate
K16190
D-Glucuronate-1P
3.1.1.19
D-Glucuronate
D-Glucurono-lactone
PGTG_05992...
D-Glucarate
K01706
2-Dehydro-3-deoxy-D-glucarate
K01630
Tartronate semialdehyde
Glyoxylate and dicarboxylate metabolism
5-Dehydro-4-deoxy-D-glucarate
K01706
K01630
Pyruvate
K01708...
D-Galactaro-1,4-lactone
K18983
D-Galactarate
K01707
4.1.2.18
1.1.1.19
1.1.1.20
L-Gulose-1P
L-Gulose
K01053
L-Gulonate
3.1.1.25
L-Gulono-1,4-lactone
K00103
L-xylo-Hexulono-lactone
3-Dehydro-L-gulonate-6P
K03078
L-Xylulose-5P
2,5-Dioxo-pentanoate
Amino sugar and nucleotide sugar metabolism
K10046
GDP-L-gulose
K10046
K00103
K03476
K18982
K13876
K03079
K00880
K13877...
2-Dehydro-3-deoxy-L-arabinonate
3-Dehydro-L-threonate
1.1.1.129
L-Threonate
L-Ascorbate-6P
D-Galactaro-1,5-lactone
K10046
GDP-L-galactose
GDP-D-mannose
K14190
K02821...
L-Ribulose-5P
K13875
2-Oxo-glutarate
L-Arabinonate
Fructose and mannose metabolism
K10047...
L-Galactose-1P
L-Galactose
K00064
PGTG_19034
L-Galactono-1,4-lactone
K00225
1.3.3.12
L-Ascorbate
3-Dehydro-L-gulonate
K03077
K13874
K00434
1.14.-.-
K08232
Monodehydro-ascorbate
Citrate cycle
D-Xylulose-5P
L-Arabinono-1,4-lactone
K21888
PGTG_17514...
K00225
K08092
K18981
1.1.1.46
K13873...
L-Galactonate
3.1.1.-
L-Dehydro-ascorbate
2,3-Diketo-L-gulonate
L-Arabinose
Amino sugar and nucleotide sugar metabolism
K19642
4.1.1.-
4.1.1.-
Pentose and glucuronate interconversions
L-Lyxonate
L-Xylonate
D-Galacturonate
Pentose and glucuronate interconversions
00053 7/12/17
(c) Kanehisa Laboratories

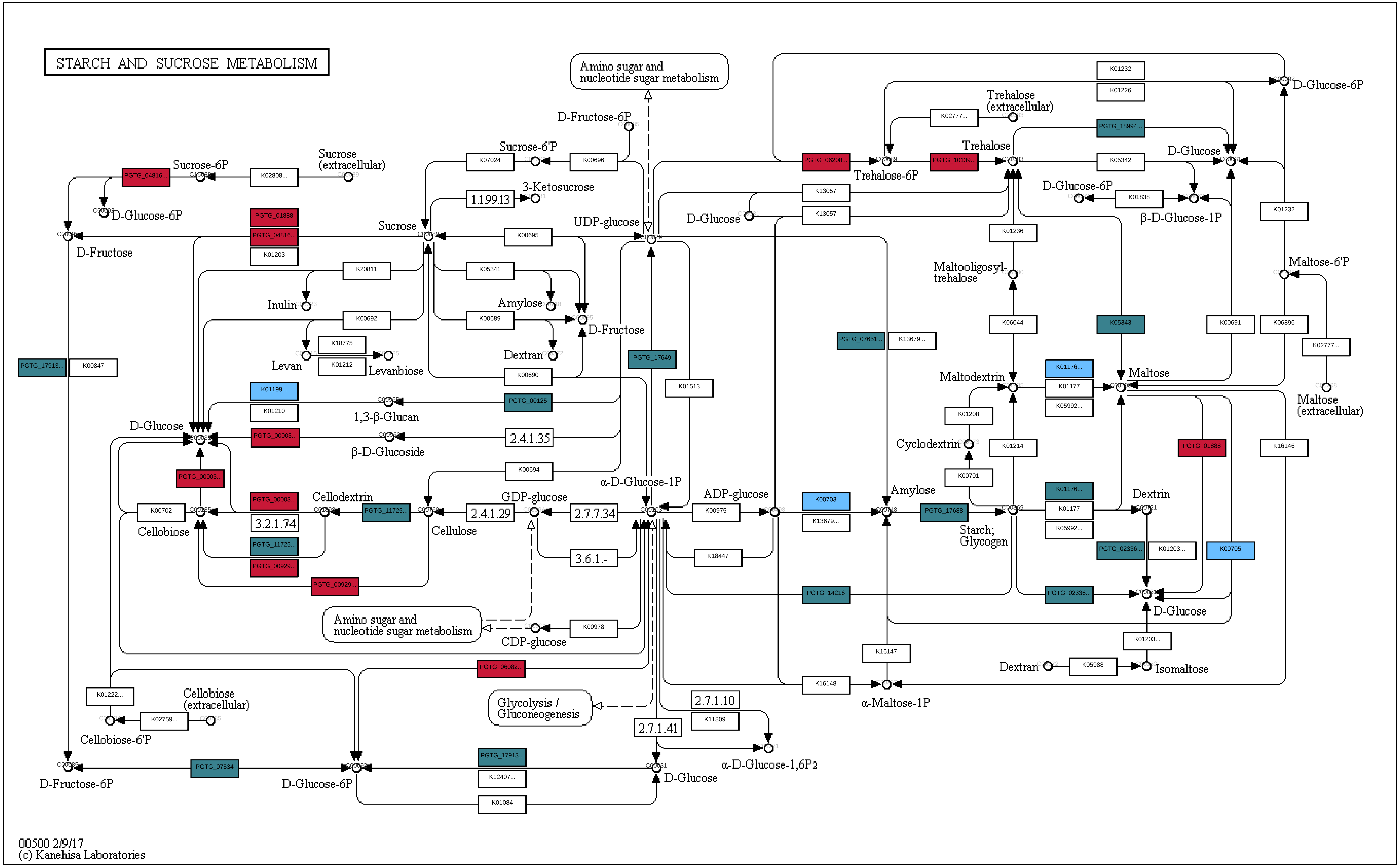

STARCH AND SUCROSE METABOLISM
Amino sugar and nucleotide sugar metabolism
D-Fructose-6P
Sucrose-6'P
K07024
K00696
Sucrose-6P
Sucrose (extracellular)
PGTG_04816...
K02808...
D-Glucose-6P
D-Fructose
PGTG_01888
PGTG_04816...
K01203
1.1.99.13
3-Ketosucrose
Sucrose
K00695
UDP-glucose
K20811
K05341
Inulin
Amylose
K00692
K00689
D-Fructose
K18775
Levan
K01212
Levanbiose
Dextran
K00690
PGTG_17913...
K00847
K01199...
K01210
PGTG_00125
1,3-β-Glucan
D-Glucose
PGTG_00003...
2.4.1.35
β-D-Glucoside
K00694
PGTG_00003...
K00702
Cellobiose
PGTG_00003...
Cellodextrin
3.2.1.74
PGTG_11725...
PGTG_11725...
PGTG_00929...
PGTG_00929...
Cellulose
GDP-glucose
2.4.1.29
2.7.7.34
3.6.1.-
α-D-Glucose-1P
PGTG_17649
K01513
Amino sugar and nucleotide sugar metabolism
K00978
CDP-glucose
PGTG_06082...
K01222...
Cellobiose (extracellular)
K02759...
Cellobiose-6'P
Glycolysis / Gluconeogenesis
2.7.1.10
K11809
2.7.1.41
α-D-Glucose-1,6P2
D-Fructose-6P
PGTG_07534
D-Glucose-6P
PGTG_17913...
K12407...
K01084
D-Glucose
K01232
K01226
D-Glucose-6P
Trehalose (extracellular)
K02777...
PGTG_18994...
Trehalose
D-Glucose
PGTG_06208...
PGTG_10139...
K05342
Trehalose-6P
K13057
K13057
D-Glucose
D-Glucose-6P
K01838
β-D-Glucose-1P
K01232
K01236
Maltooligosyl-trehalose
Maltose-6'P
PGTG_07651...
K13679...
K06044
K05343
K00691
K06896
K02777...
Maltodextrin
K01176...
K01177
K05992...
Maltose
Maltose (extracellular)
K01208
Cyclodextrin
K01214
PGTG_01888
K16146
K00701
ADP-glucose
K00975
K13679...
K00703
Amylose
PGTG_17688
Starch; Glycogen
K01176...
K01177
K05992...
Dextrin
PGTG_02336...
K01203...
K00705
K18447
PGTG_14216
PGTG_02336...
D-Glucose
K16147
K01203...
Dextran
K05988
Isomaltose
K16148
α-Maltose-1P
00500 2/9/17
(c) Kanehisa Laboratories

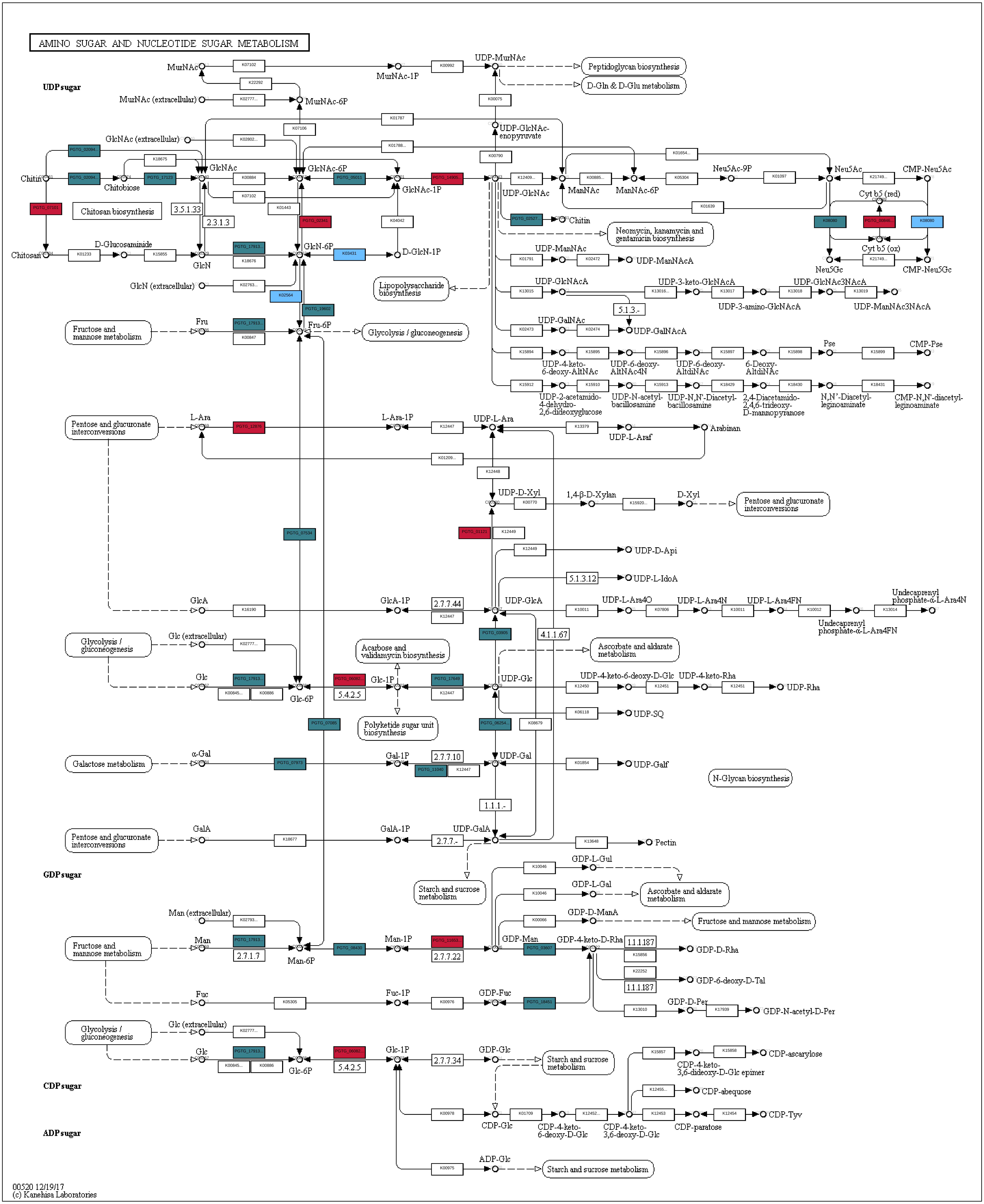
AMINO SUGAR AND NUCLEOTIDE SUGAR METABOLISM
UDP sugar
GDP sugar
CDP sugar
ADP sugar
MurNAc
MurNAc (extracellular)
MurNAc-6P
MurNAc-1P
UDP-MurNAc
Peptidoglycan biosynthesis
D-Gln & D-Glu metabolism
UDP-GlcNAc-enopyruvate
GlcNAc (extracellular)
GlcNAc
GlcNAc-6P
GlcNAc-1P
UDP-GlcNAc
Chitin
Chitobiose
Chitosan
Chitosan biosynthesis
3.5.1.33
2.3.1.3
D-Glucosamide
GlcN
GlcN-6P
D-GlcN-1P
GlcN (extracellular)
Lipopolysaccharide biosynthesis
Neomycin, kanamycin and gentamicin biosynthesis
ManNAc
ManNAc-6P
Neu5Ac-9P
Neu5Ac
CMP-Neu5Ac
Cyt b5 (red)
Cyt b5 (ox)
Neu5Gc
CMP-Neu5Gc
UDP-ManNAc
UDP-ManNAcA
UDP-GlcNAcA
UDP-3-keto-GlcNAcA
UDP-GlcNAc3NAcA
UDP-3-amino-GlcNAcA
UDP-ManNAc3NAcA
5.1.3.-
UDP-GalNAc
UDP-GalNAcA
Fructose and mannose metabolism
Fru
Fru-6P
Glycolysis / gluconeogenesis
UDP-4-keto-6-deoxy-AltNAc
UDP-6-deoxy-AltNAc4N
UDP-6-deoxy-AltdiNAc
6-Deoxy-AltdiNAc
Pse
CMP-Pse
UDP-2-acetamido-4-dehydro-2,6-dideoxyglucose
UDP-N-acetyl-bacillosamine
UDP-N,N'-Diacetyl-bacillosamine
2,4-Diacetamido-2,4,6-trideoxy-D-mannopyranose
N,N'-Diacetyl-legionaminate
CMP-N,N'-diacetyl-legionaminate
Pentose and glucuronate interconversions
L-Ara
L-Ara-1P
UDP-L-Ara
UDP-L-Araf
Arabinan
UDP-D-Xyl
1,4-β-D-Xylan
D-Xyl
Pentose and glucuronate interconversions
UDP-D-Api
5.1.3.12
UDP-L-IdoA
GlcA
GlcA-1P
2.7.7.44
UDP-GlcA
UDP-L-Ara4O
UDP-L-Ara4N
UDP-L-Ara4FN
Undecaprenyl phosphate-α-L-Ara4N
Undecaprenyl phosphate-α-L-Ara4FN
4.1.1.67
Ascorbate and aldarate metabolism
Glycolysis / gluconeogenesis
Glc (extracellular)
Acarbose and validamycin biosynthesis
Glc
Glc-6P
Glc-1P
5.4.2.5
UDP-Glc
UDP-4-keto-6-deoxy-D-Glc
UDP-4-keto-Rha
UDP-Rha
UDP-SQ
Polyketide sugar unit biosynthesis
Galactose metabolism
α-Gal
Gal-1P
2.7.7.10
UDP-Gal
UDP-Galf
N-Glycan biosynthesis
1.1.1.-
Pentose and glucuronate interconversions
GalA
GalA-1P
2.7.7.-
UDP-GalA
Pectin
GDP-L-Gul
GDP-L-Gal
Ascorbate and aldarate metabolism
Starch and sucrose metabolism
GDP-D-ManA
Fructose and mannose metabolism
Man (extracellular)
Fructose and mannose metabolism
Man
2.7.1.7
Man-6P
Man-1P
2.7.7.22
GDP-Man
GDP-4-keto-D-Rha
1.1.1.187
GDP-D-Rha
GDP-6-deoxy-D-Tal
1.1.1.187
Fuc
Fuc-1P
GDP-Fuc
GDP-D-Per
GDP-N-acetyl-D-Per
Glycolysis / gluconeogenesis
Glc (extracellular)
Glc
Glc-6P
5.4.2.5
Glc-1P
2.7.7.34
GDP-Glc
Starch and sucrose metabolism
CDP-ascarylose
CDP-4-keto-3,6-dideoxy-D-Glc epimer
CDP-abequose
CDP-Glc
CDP-4-keto-6-deoxy-D-Glc
CDP-4-keto-3,6-deoxy-D-Glc
CDP-paratose
CDP-Tyv
ADP-Glc
Starch and sucrose metabolism
00520 12/19/17
(c) Kanehisa Laboratories

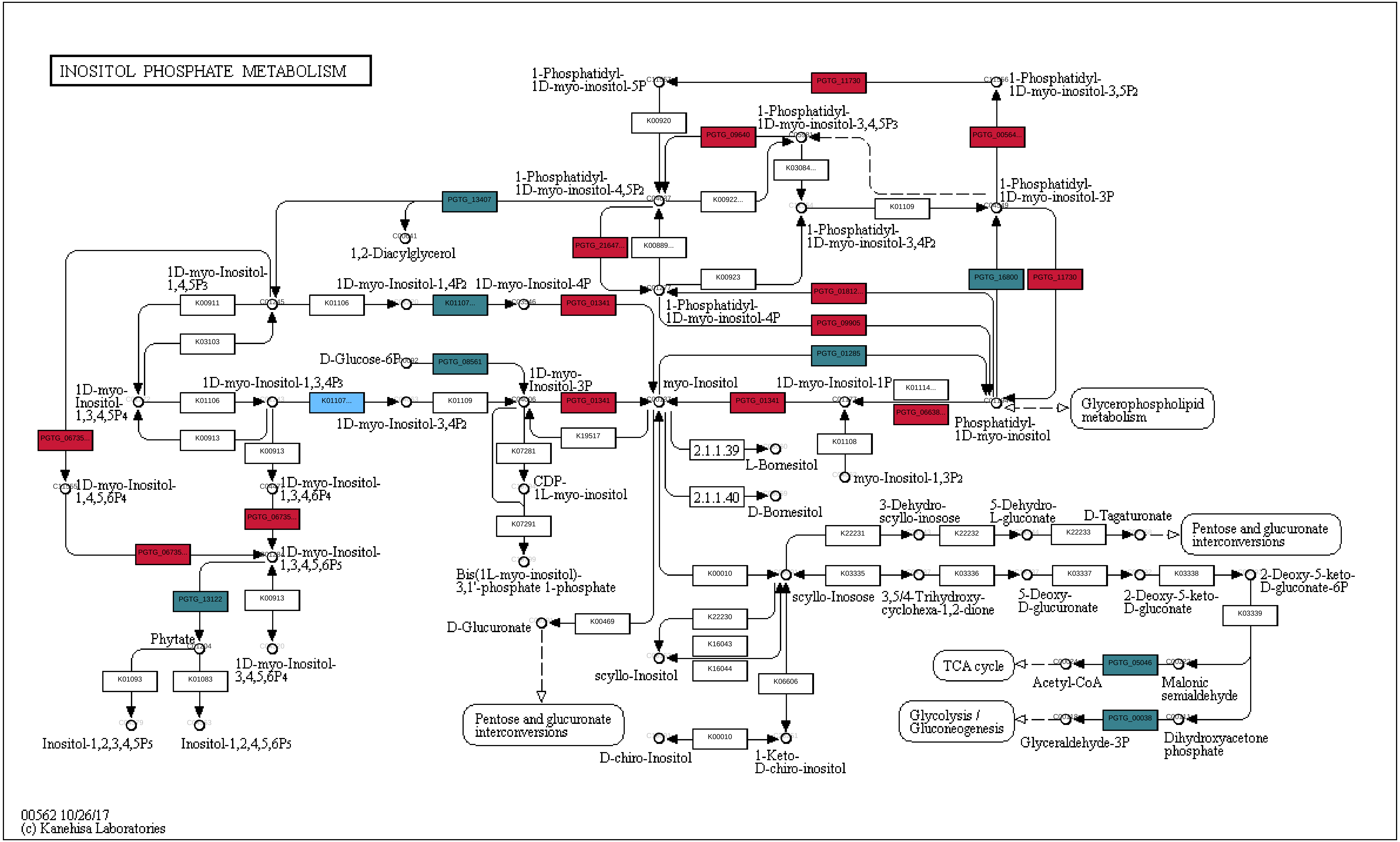
INOSITOL PHOSPHATE METABOLISM
1-Phosphatidyl-1D-myo-inositol-5P
PGTG_11730
1-Phosphatidyl-1D-myo-inositol-3,5P2
K00920
PGTG_09640
1-Phosphatidyl-1D-myo-inositol-3,4,5P3
PGTG_00564...
K03084...
1-Phosphatidyl-1D-myo-inositol-4,5P2
PGTG_13407
K00922...
K01109
1-Phosphatidyl-1D-myo-inositol-3P
1,2-Diacylglycerol
PGTG_21647...
K00889...
1-Phosphatidyl-1D-myo-inositol-3,4P2
K00923
PGTG_16800
PGTG_11730
1D-myo-Inositol-1,4,5P3
1D-myo-Inositol-1,4P2
1D-myo-Inositol-4P
K00911
K01106
K01107...
PGTG_01341
PGTG_01812...
1-Phosphatidyl-1D-myo-inositol-4P
PGTG_09905
K03103
D-Glucose-6P
PGTG_08561
PGTG_01285
1D-myo-Inositol-3P
myo-Inositol
1D-myo-Inositol-1P
K01114...
1D-myo-Inositol-1,3,4,5P4
1D-myo-Inositol-1,3,4P3
K01106
K01107...
K01109
PGTG_01341
PGTG_01341
PGTG_06638...
Glycerophospholipid metabolism
Phosphatidyl-1D-myo-inositol
1D-myo-Inositol-3,4P2
K00913
K19517
K01108
PGTG_06735...
K00913
K07281
2.1.1.39
L-Bornesitol
myo-Inositol-1,3P2
1D-myo-Inositol-1,4,5,6P4
1D-myo-Inositol-1,3,4,6P4
CDP-1L-myo-inositol
2.1.1.40
D-Bornesitol
3-Dehydro-scyllo-inosose
5-Dehydro-L-gluconate
D-Tagaturonate
PGTG_06735...
K07291
K22231
K22232
K22233
Pentose and glucuronate interconversions
PGTG_06735...
1D-myo-Inositol-1,3,4,5,6P5
Bis(1L-myo-inositol)-3,1'-phosphate 1-phosphate
K00010
K03335
K03336
K03337
K03338
2-Deoxy-5-keto-D-gluconate-6P
PGTG_13122
K00913
scyllo-Inosose
3,5/4-Trihydroxy-cyclohexa-1,2-dione
5-Deoxy-D-glucuronate
2-Deoxy-5-keto-D-gluconate
K03339
D-Glucuronate
K00469
K22230
Phytate
1D-myo-Inositol-3,4,5,6P4
K16043
TCA cycle
PGTG_05046
scyllo-Inositol
K16044
Acetyl-CoA
Malonic semialdehyde
K01093
K01083
K06606
Glycolysis / Gluconeogenesis
PGTG_00038
Pentose and glucuronate interconversions
K00010
Glyceraldehyde-3P
Dihydroxyacetone phosphate
Inositol-1,2,3,4,5P5
Inositol-1,2,4,5,6P5
D-chiro-Inositol
1-Keto-D-chiro-inositol
00562 10/26/17
(c) Kanehisa Laboratories

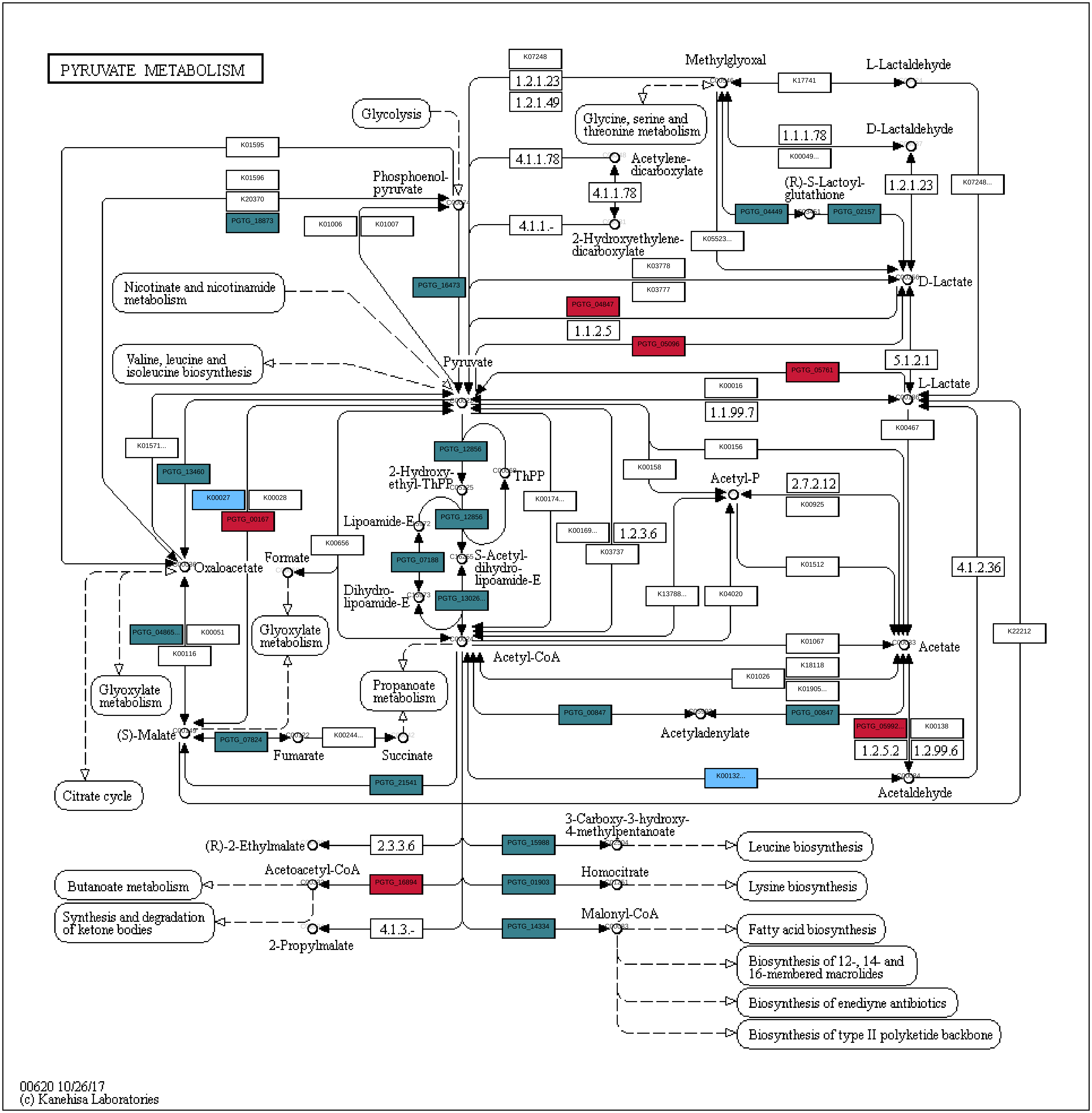
PYRUVATE METABOLISM
Methylglyoxal
L-Lactaldehyde
D-Lactaldehyde
Glycolysis
Glycine, serine and threonine metabolism
Acetylene-dicarboxylate
2-Hydroxyethylene-dicarboxylate
Phosphoenol-pyruvate
(R)-S-Lactoyl-glutathione
D-Lactate
L-Lactate
Nicotinate and nicotinamide metabolism
Valine, leucine and isoleucine biosynthesis
Pyruvate
2-Hydroxy-ethyl-ThPP
ThPP
Lipoamide-E
S-Acetyl-dihydro-lipoamide-E
Dihydro-lipoamide-E
Acetyl-P
Acetyl-CoA
Acetate
Acetyladenylate
Acetaldehyde
Oxaloacetate
Formate
Glyoxylate metabolism
Propanoate metabolism
(S)-Malate
Fumarate
Succinate
Citrate cycle
(R)-2-Ethylmalate
3-Carboxy-3-hydroxy-4-methylpentanoate
Leucine biosynthesis
Acetoacetyl-CoA
Homocitrate
Lysine biosynthesis
Butanoate metabolism
Synthesis and degradation of ketone bodies
2-Propylmalate
Malonyl-CoA
Fatty acid biosynthesis
Biosynthesis of 12-, 14- and 16-membered macrolides
Biosynthesis of enediyne antibiotics
Biosynthesis of type II polyketide backbone
K07248
1.2.1.23
1.2.1.49
K17741
1.1.1.78
K00049...
1.2.1.23
K07248...
4.1.1.78
4.1.1.78
4.1.1.-
K01595
K01596
K20370
PGTG_18873
K01006
K01007
PGTG_04449
PGTG_02157
K05523...
K03778
K03777
PGTG_16473
PGTG_04847
1.1.2.5
PGTG_05096
5.1.2.1
PGTG_05761
K00016
1.1.99.7
K00467
K01571...
K00156
PGTG_13460
PGTG_12856
K00158
2.7.2.12
K00027
K00028
K00925
PGTG_00167
K00174...
PGTG_12856
K00656
K00169...
1.2.3.6
PGTG_07188
K03737
K01512
4.1.2.36
PGTG_13026...
K13788...
K04020
PGTG_04865...
K00051
K00116
K01067
K22212
K18118
K01026
K01905...
PGTG_00847
PGTG_00847
PGTG_05992...
K00138
1.2.5.2
1.2.99.6
PGTG_07824
K00244...
PGTG_21541
K00132...
2.3.3.6
PGTG_15988
PGTG_16894
PGTG_01903
4.1.3.-
PGTG_14334
00620 10/26/17
(c) Kanehisa Laboratories

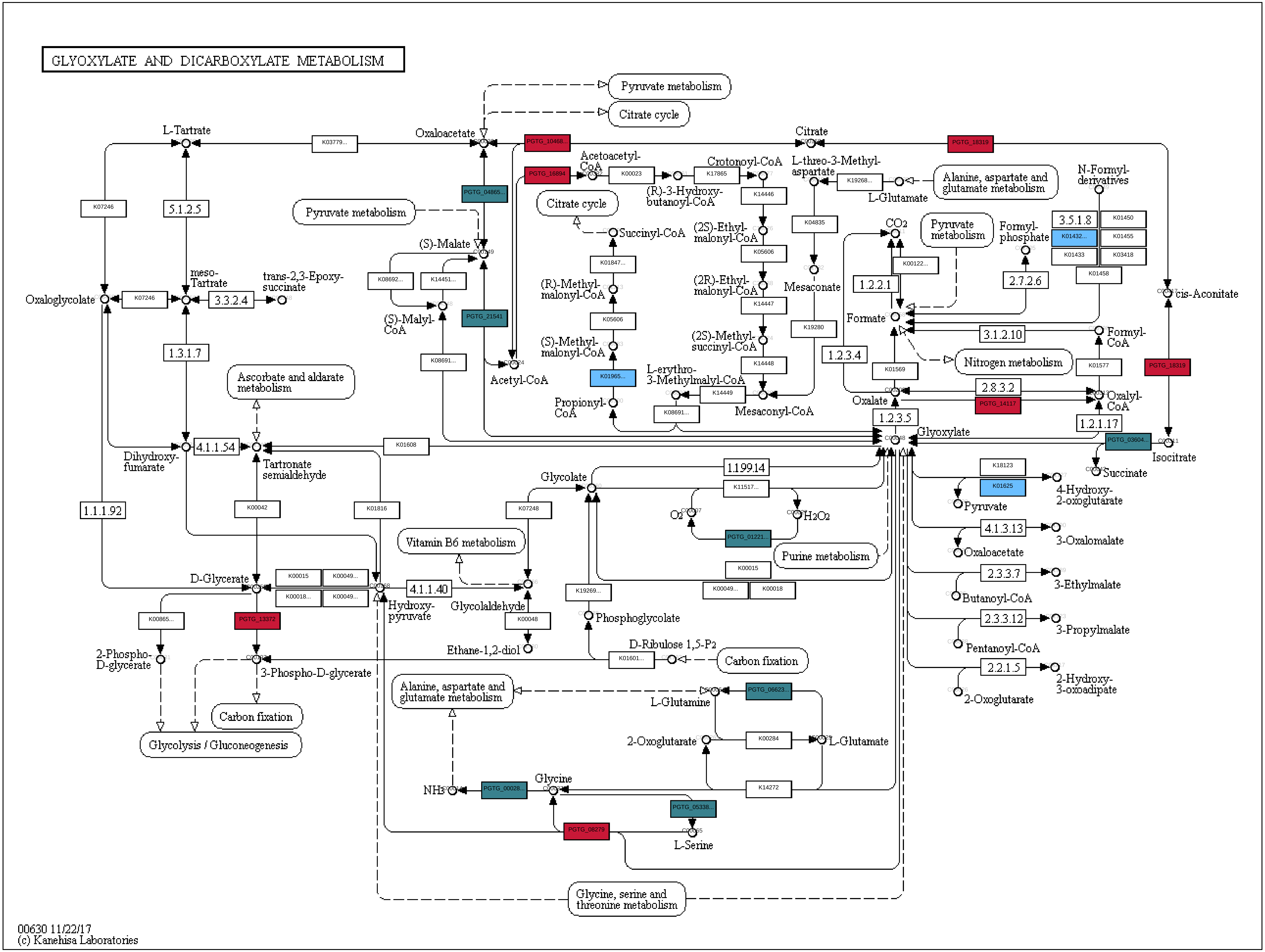
GLYOXYLATE AND DICARBOXYLATE METABOLISM
Pyruvate metabolism
Citrate cycle
L-Tartrate
Oxaloacetate
Citrate
Acetoacetyl-CoA
Crotonoyl-CoA
L-threo-3-Methyl-aspartate
Alanine, aspartate and glutamate metabolism
N-Formyl-derivatives
(R)-3-Hydroxy-butanoyl-CoA
L-Glutamate
Succinyl-CoA
(2S)-Ethyl-malonyl-CoA
CO2
Formyl-phosphate
(S)-Malate
meso-Tartrate
trans-2,3-Epoxy-succinate
Oxaloglycolate
(R)-Methyl-malonyl-CoA
(2R)-Ethyl-malonyl-CoA
Mesaconate
cis-Aconitate
(S)-Malyl-CoA
Formate
Formyl-CoA
(S)-Methyl-malonyl-CoA
(2S)-Methyl-succinyl-CoA
Nitrogen metabolism
Acetyl-CoA
Ascorbate and aldarate metabolism
L-erythro-3-Methylmalyl-CoA
Propionyl-CoA
Mesaconyl-CoA
Oxalate
Oxalyl-CoA
Glyoxylate
Dihydroxy-fumarate
Tartronate semialdehyde
Isocitrate
Succinate
Glycolate
4-Hydroxy-2-oxoglutarate
Pyruvate
O2
H2O2
Vitamin B6 metabolism
Purine metabolism
Oxaloacetate
3-Oxalomalate
D-Glycerate
3-Ethylmalate
Hydroxy-pyruvate
Glycolaldehyde
Butanoyl-CoA
Phosphoglycolate
3-Propylmalate
2-Phospho-D-glycerate
Ethane-1,2-diol
D-Ribulose 1,5-P2
Pentanoyl-CoA
3-Phospho-D-glycerate
Carbon fixation
2-Hydroxy-3-oxoadipate
Alanine, aspartate and glutamate metabolism
L-Glutamine
2-Oxoglutarate
Carbon fixation
Glycolysis / Gluconeogenesis
2-Oxoglutarate
L-Glutamate
NH3
Glycine
L-Serine
Glycine, serine and threonine metabolism
00630 11/22/17
(c) Kanehisa Laboratories

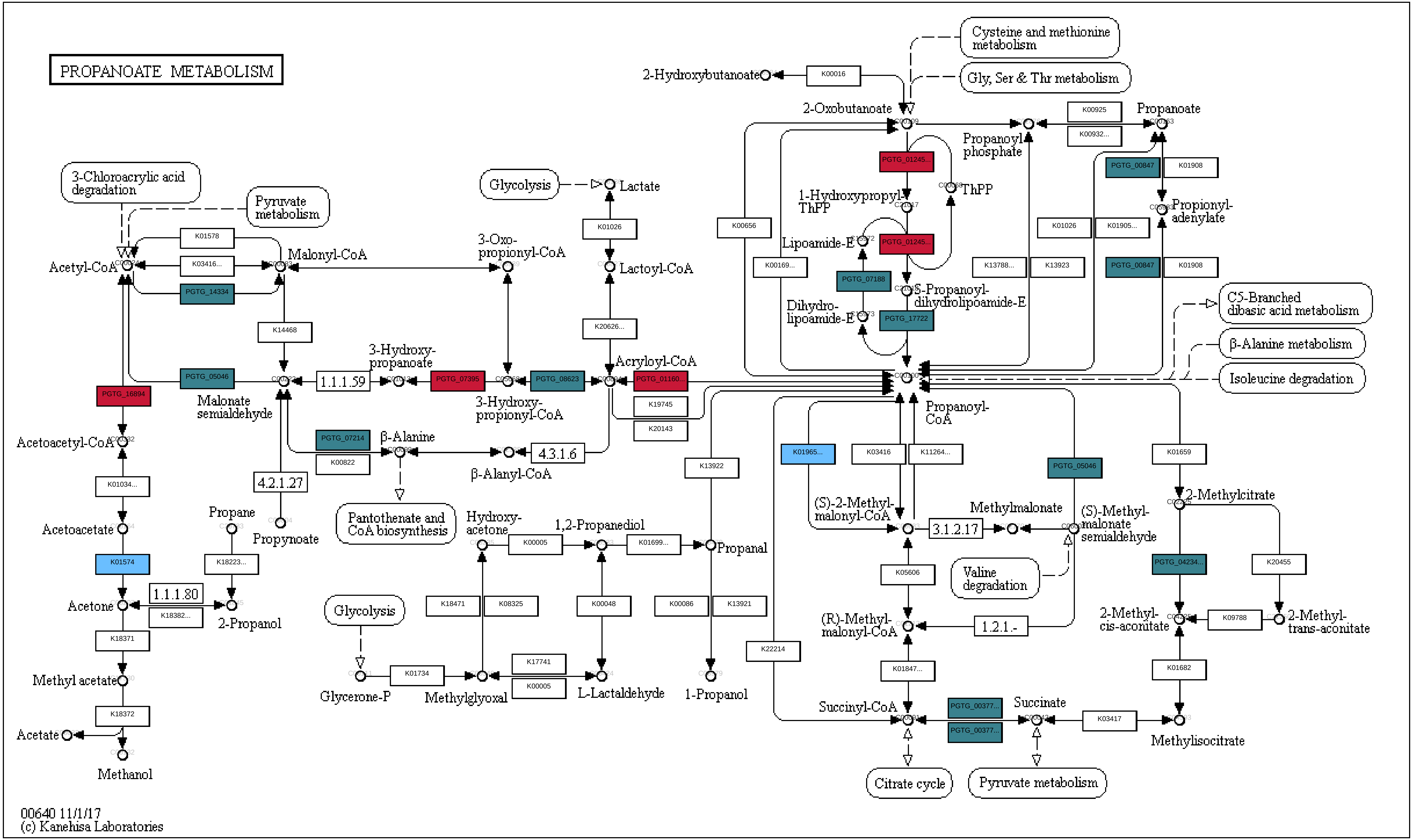
PROPANOATE METABOLISM
3-Chloroacrylic acid degradation
Pyruvate metabolism
Glycolysis
Lactate
K01026
Lactoyl-CoA
K20626...
Acryloyl-CoA
PGTG_01160...
K19745
K20143
K01578
K03416...
PGTG_14334
Acetyl-CoA
Malonyl-CoA
3-Oxo-propionyl-CoA
K14468
PGTG_05046
Malonate semialdehyde
1.1.1.59
3-Hydroxy-propanoate
PGTG_07395
PGTG_08623
3-Hydroxy-propionyl-CoA
PGTG_16894
Acetoacetyl-CoA
K01034...
Acetoacetate
K01574
Acetone
1.1.1.80
K18382...
K18371
Methyl acetate
K18372
Acetate
Methanol
Propane
K18223...
2-Propanol
4.2.1.27
Propynoate
PGTG_07214
K00822
β-Alanine
4.3.1.6
β-Alanyl-CoA
Pantothenate and CoA biosynthesis
Hydroxy-acetone
K00005
1,2-Propanediol
K01699...
Propanal
K18471
K08325
K00048
K00086
K13921
K13922
Glycolysis
Glycerone-P
K01734
Methylglyoxal
K17741
K00005
L-Lactaldehyde
1-Propanol
Cysteine and methionine metabolism
Gly, Ser & Thr metabolism
2-Hydroxybutanoate
K00016
2-Oxobutanoate
K00925
K00932...
Propanoate
Propanoyl phosphate
PGTG_01245...
1-Hydroxypropyl-ThPP
ThPP
K00656
Lipoamide-E
PGTG_01245...
K00169...
PGTG_07188
S-Propanoyl-dihydrolipoamide-E
Dihydro-lipoamide-E
PGTG_17722
K13788...
K13923
PGTG_00847
K01908
K01026
K01905...
Propionyl-adenylate
PGTG_00847
K01908
C5-Branched dibasic acid metabolism
β-Alanine metabolism
Isoleucine degradation
Propanoyl-CoA
K01965...
K03416
K11264...
PGTG_05046
K01659
(S)-2-Methyl-malonyl-CoA
3.1.2.17
Methylmalonate
(S)-Methyl-malonate semialdehyde
2-Methylcitrate
Valine degradation
K05606
(R)-Methyl-malonyl-CoA
1.2.1.-
K01847...
K22214
PGTG_04234...
K20455
2-Methyl-cis-aconitate
K09788
2-Methyl-trans-aconitate
K01682
Succinyl-CoA
PGTG_00377...
PGTG_00377...
Succinate
K03417
Methylisocitrate
Citrate cycle
Pyruvate metabolism
00640 11/1/17
(c) Kanehisa Laboratories

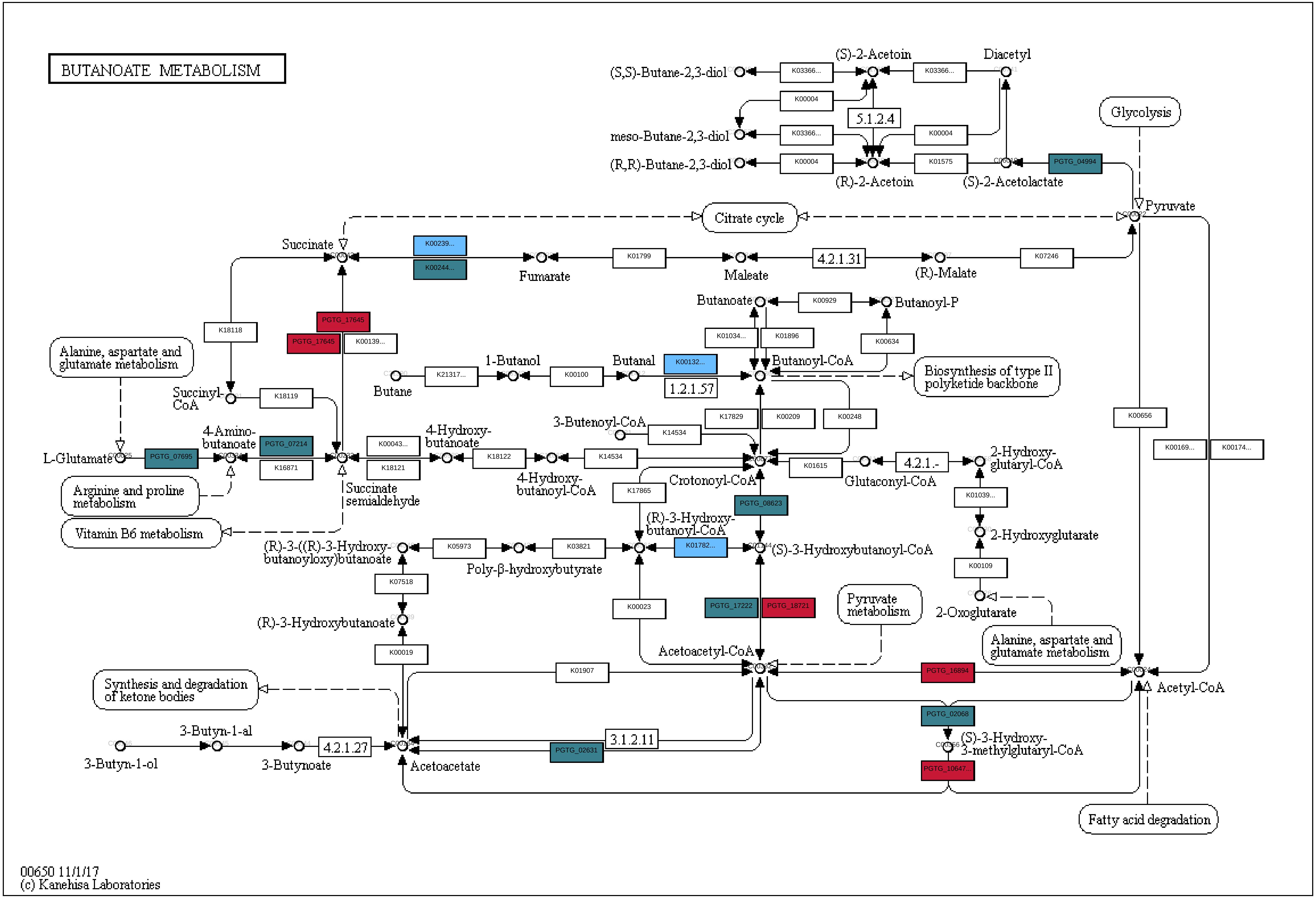
BUTANOATE METABOLISM
(S)-2-Acetoin
Diacetyl
(S,S)-Butane-2,3-diol
K03366...
K03366...
K00004
5.1.2.4
meso-Butane-2,3-diol
K03366...
K00004
Glycolysis
(R,R)-Butane-2,3-diol
K00004
K01575
PGTG_04994
(R)-2-Acetoin
(S)-2-Acetolactate
Pyruvate
Citrate cycle
Succinate
K00239...
K00244...
K01799
4.2.1.31
K07246
Fumarate
Maleate
(R)-Malate
Butanoate
K00929
Butanoyl-P
PGTG_17645
K18118
PGTG_17645
K00139...
K01034...
K01896
K00634
Alanine, aspartate and glutamate metabolism
1-Butanol
Butanal
K00132...
Butanoyl-CoA
K21317...
K00100
Biosynthesis of type II polyketide backbone
Butane
1.2.1.57
Succinyl-CoA
K18119
K17829
K00209
K00248
K00656
3-Butenoyl-CoA
4-Amino-butanoate
4-Hydroxy-butanoate
K00043...
K14534
K00169...
K00174...
L-Glutamate
PGTG_07695
PGTG_07214
K18122
K14534
2-Hydroxy-glutaryl-CoA
K16871
K18121
K01615
4.2.1.-
Arginine and proline metabolism
Succinate semialdehyde
4-Hydroxy-butanoyl-CoA
Crotonoyl-CoA
Glutaconyl-CoA
K17865
PGTG_08623
K01039...
(R)-3-Hydroxy-butanoyl-CoA
Vitamin B6 metabolism
2-Hydroxyglutarate
(R)-3-((R)-3-Hydroxy-butanoyloxy)butanoate
K05973
K03821
K01782...
(S)-3-Hydroxybutanoyl-CoA
Poly-β-hydroxybutyrate
K07518
K00109
K00023
PGTG_17222
PGTG_18721
Pyruvate metabolism
2-Oxoglutarate
(R)-3-Hydroxybutanoate
Alanine, aspartate and glutamate metabolism
K00019
Acetoacetyl-CoA
K01907
PGTG_16894
Acetyl-CoA
Synthesis and degradation of ketone bodies
PGTG_02068
3-Butyn-1-al
(S)-3-Hydroxy-3-methylglutaryl-CoA
4.2.1.27
3.1.2.11
3-Butyn-1-ol
3-Butynoate
PGTG_02631
Acetoacetate
PGTG_10647...
Fatty acid degradation
00650 11/1/17
(c) Kanehisa Laboratories

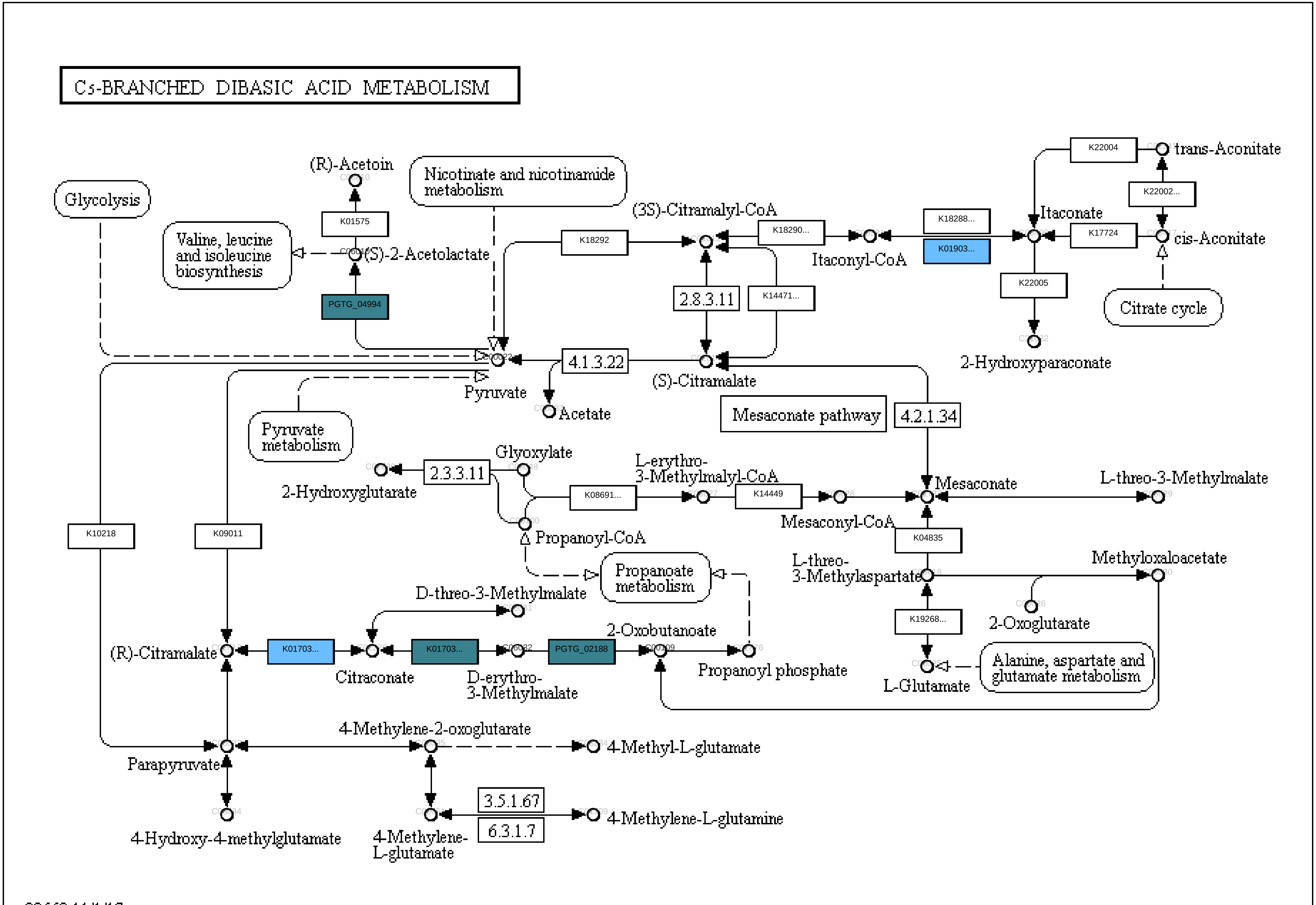


00660 11/1/17

(c) Kanehisa Laboratories

# 2. Energy metabolism

| MAP | PATHWAY |
|---|---|
| **190** | Oxidative phosphorylation |
| **680** | Methane metabolism |
| **910** | Nitrogen metabolism |
| **920** | Sulfur metabolism |

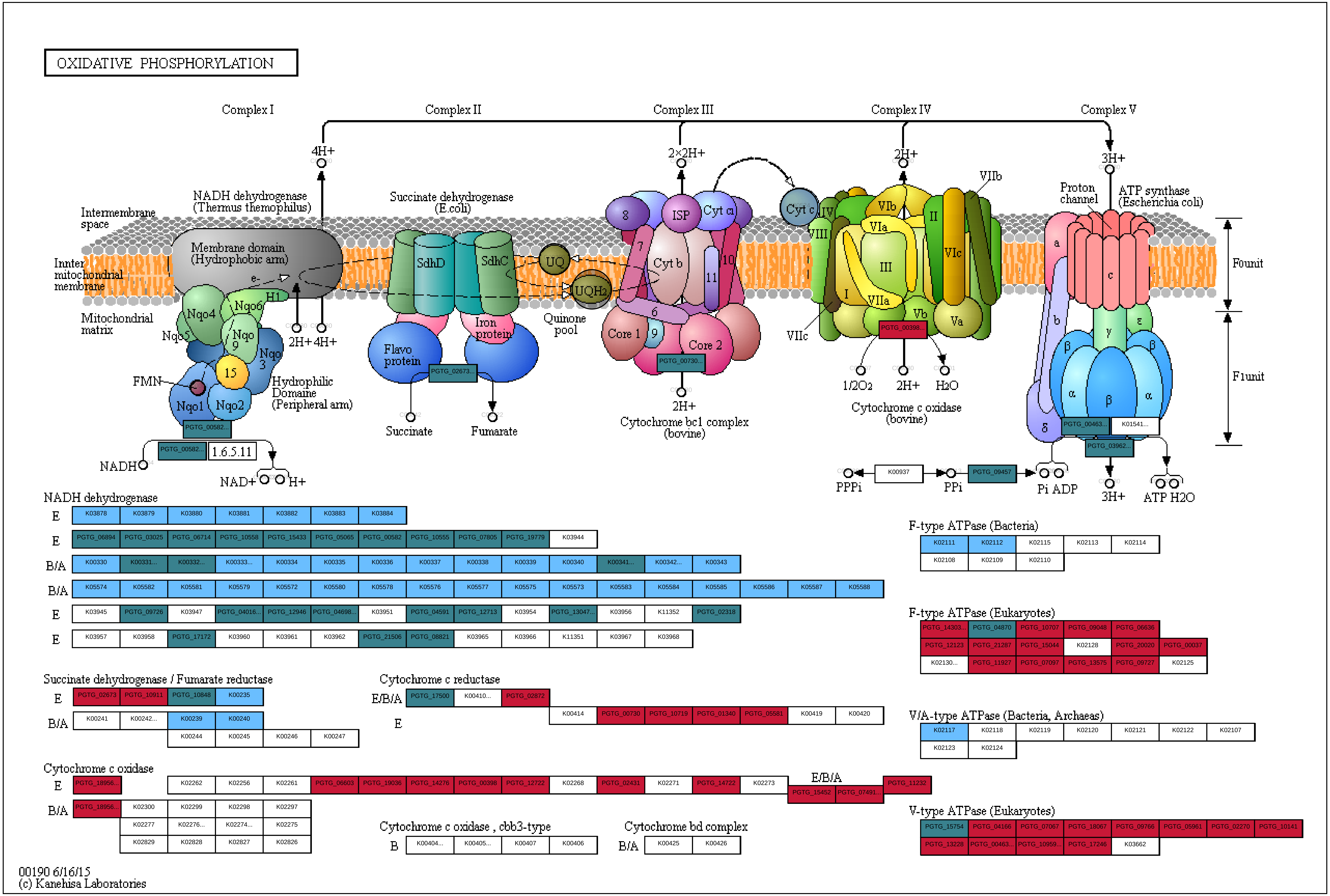

OXIDATIVE PHOSPHORYLATION
Complex I
Complex II
Complex III
Complex IV
Complex V
4H+
2×2H+
2H+
3H+
NADH dehydrogenase (Thermus themophilus)
Intermembrane space
Innter mitochondrial membrane
Mitochondrial matrix
Membrane domain (Hydrophobic arm)
e-
H1
Nqo4
Nqo6
Nqo5
Nqo 9
Nqo 3
15
FMN
Nqo1
Nqo2
Hydrophilic Domaine (Peripheral arm)
2H+ 4H+
PGTG_00582...
PGTG_00582...
1.6.5.11
NADH
NAD+
H+
Succinate dehydrogenase (E.coli)
SdhD
SdhC
Iron protein
Flavo protein
PGTG_02673...
Succinate
Fumarate
UQ
UQH2
Quinone pool
8
ISP
Cyt a
Cyt c
7
Cyt b
10
11
6
Core 1
9
Core 2
PGTG_00730...
2H+
Cytochrome bc1 complex (bovine)
IV
VIII
VIb
VIa
II
VIc
III
I
VIIa
Vb
Va
VIIb
VIIc
PGTG_00398...
1/2O2
2H+
H2O
Cytochrome c oxidase (bovine)
Proton channel
ATP synthase (Escherichia coli)
a
c
b
γ
ε
β
α
β
α
δ
Founit
F1unit
PGTG_00463...
K01541...
PGTG_03962...
PPPi
K00937
PPi
PGTG_09457
Pi ADP
3H+
ATP H2O
NADH dehydrogenase
E K03878 K03879 K03880 K03881 K03882 K03883 K03884
E PGTG_06894 PGTG_03025 PGTG_06714 PGTG_10558 PGTG_15433 PGTG_05065 PGTG_00582 PGTG_10555 PGTG_07805 PGTG_19779 K03944
B/A K00330 K00331... K00332... K00333... K00334 K00335 K00336 K00337 K00338 K00339 K00340 K00341... K00342... K00343
B/A K05574 K05582 K05581 K05579 K05572 K05580 K05578 K05576 K05577 K05575 K05573 K05583 K05584 K05585 K05586 K05587 K05588
E K03945 PGTG_09726 K03947 PGTG_04016... PGTG_12946 PGTG_04698... K03951 PGTG_04591 PGTG_12713 K03954 PGTG_13047... K03956 K11352 PGTG_02318
E K03957 K03958 PGTG_17172 K03960 K03961 K03962 PGTG_21506 PGTG_08821 K03965 K03966 K11351 K03967 K03968
Succinate dehydrogenase / Fumarate reductase
E PGTG_02673 PGTG_10911 PGTG_10848 K00235
B/A K00241 K00242... K00239 K00240
K00244 K00245 K00246 K00247
Cytochrome c reductase
E/B/A PGTG_17500 K00410... PGTG_02872
E K00414 PGTG_00730 PGTG_10719 PGTG_01340 PGTG_05581 K00419 K00420
Cytochrome c oxidase
E PGTG_18956... K02262 K02256 K02261 PGTG_06603 PGTG_19036 PGTG_14276 PGTG_00398 PGTG_12722 K02268 PGTG_02431 K02271 PGTG_14722 K02273
E/B/A PGTG_15452 PGTG_07491... PGTG_11232
B/A PGTG_18956... K02300 K02299 K02298 K02297
K02277 K02276... K02274... K02275
K02829 K02828 K02827 K02826
Cytochrome c oxidase , cbb3-type
B K00404... K00405... K00407 K00406
Cytochrome bd complex
B/A K00425 K00426
F-type ATPase (Bacteria)
K02111 K02112 K02115 K02113 K02114
K02108 K02109 K02110
F-type ATPase (Eukaryotes)
PGTG_14303... PGTG_04870 PGTG_10707 PGTG_09048 PGTG_06636
PGTG_12123 PGTG_21287 PGTG_15044 K02128 PGTG_20020 PGTG_00037
K02130... PGTG_11927 PGTG_07097 PGTG_13575 PGTG_09727 K02125
V/A-type ATPase (Bacteria, Archaeas)
K02117 K02118 K02119 K02120 K02121 K02122 K02107
K02123 K02124
V-type ATPase (Eukaryotes)
PGTG_15754 PGTG_04166 PGTG_07067 PGTG_18067 PGTG_09766 PGTG_05961 PGTG_02270 PGTG_10141
PGTG_13228 PGTG_00463... PGTG_10959... PGTG_17246 K03662
00190 6/16/15
(c) Kanehisa Laboratories

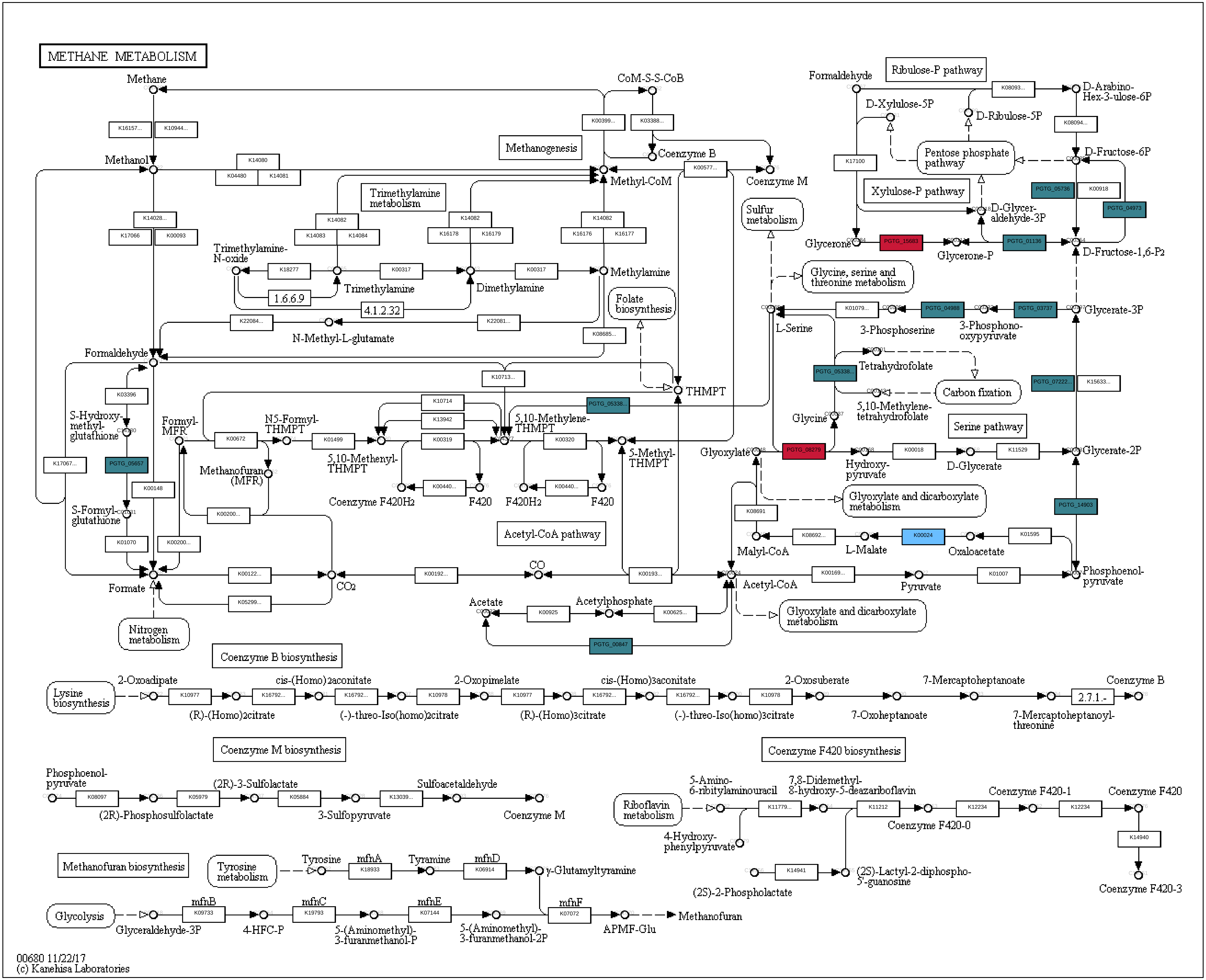
METHANE METABOLISM
Methane
Methanol
Formaldehyde
Formate
CO2
CO
CoM-S-S-CoB
Coenzyme B
Coenzyme M
Methyl-CoM
Methanogenesis
Trimethylamine metabolism
Trimethylamine-N-oxide
Trimethylamine
Dimethylamine
Methylamine
N-Methyl-L-glutamate
Folate biosynthesis
THMPT
Sulfur metabolism
S-Hydroxy-methyl-glutathione
S-Formyl-glutathione
Formyl-MFR
Methanofuran (MFR)
N5-Formyl-THMPT
5,10-Methenyl-THMPT
5,10-Methylene-THMPT
5-Methyl-THMPT
Coenzyme F420H2
F420
F420H2
Acetyl-CoA pathway
Acetate
Acetylphosphate
Acetyl-CoA
Nitrogen metabolism
Ribulose-P pathway
D-Arabino-Hex-3-ulose-6P
D-Xylulose-5P
D-Ribulose-5P
Pentose phosphate pathway
D-Fructose-6P
Xylulose-P pathway
D-Glyceraldehyde-3P
Glycerone
Glycerone-P
D-Fructose-1,6-P2
Glycine, serine and threonine metabolism
L-Serine
3-Phosphoserine
3-Phosphonooxypyruvate
Glycerate-3P
Tetrahydrofolate
5,10-Methylenetetrahydrofolate
Carbon fixation
Glycine
Serine pathway
Glyoxylate
Hydroxypyruvate
D-Glycerate
Glycerate-2P
Glyoxylate and dicarboxylate metabolism
Malyl-CoA
L-Malate
Oxaloacetate
Phosphoenolpyruvate
Pyruvate
Coenzyme B biosynthesis
Lysine biosynthesis
2-Oxoadipate
(R)-(Homo)2citrate
cis-(Homo)2aconitate
(-)-threo-Iso(homo)2citrate
2-Oxopimelate
(R)-(Homo)3citrate
cis-(Homo)3aconitate
(-)-threo-Iso(homo)3citrate
2-Oxosuberate
7-Oxoheptanoate
7-Mercaptoheptanoate
7-Mercaptoheptanoyl-threonine
2.7.1.-
Coenzyme M biosynthesis
Phosphoenolpyruvate
(2R)-Phosphosulfolactate
(2R)-3-Sulfolactate
3-Sulfopyruvate
Sulfoacetaldehyde
Coenzyme M
Coenzyme F420 biosynthesis
Riboflavin metabolism
5-Amino-6-ribitylaminouracil
4-Hydroxyphenylpyruvate
7,8-Didemethyl-8-hydroxy-5-deazariboflavin
Coenzyme F420-0
Coenzyme F420-1
Coenzyme F420
Coenzyme F420-3
(2S)-2-Phospholactate
(2S)-Lactyl-2-diphospho-5'-guanosine
Methanofuran biosynthesis
Tyrosine metabolism
Tyrosine
Tyramine
γ-Glutamyltyramine
mfnA
mfnD
Glycolysis
Glyceraldehyde-3P
mfnB
4-HFC-P
mfnC
5-(Aminomethyl)-3-furanmethanol-P
mfnE
5-(Aminomethyl)-3-furanmethanol-2P
mfnF
APMF-Glu
Methanofuran
1.6.6.9
4.1.2.32
PGTG_15683
PGTG_01136
PGTG_05736
PGTG_04973
PGTG_04988
PGTG_03737
PGTG_05338...
PGTG_07222...
PGTG_08279
PGTG_14903
PGTG_05657
PGTG_00847
00680 11/22/17
(c) Kanehisa Laboratories

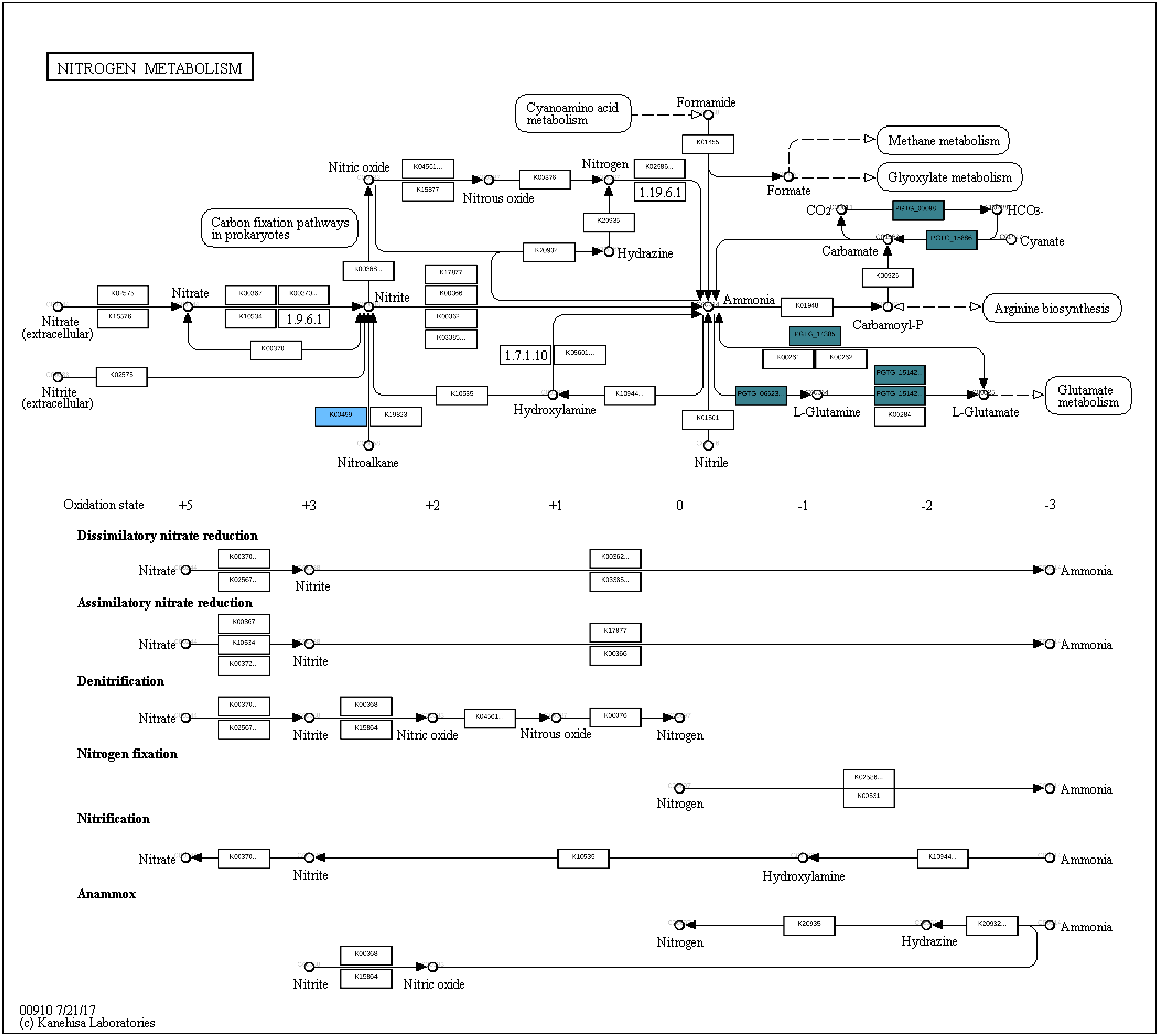
NITROGEN METABOLISM
Cyanoamino acid metabolism
Formamide
K01455
Methane metabolism
Glyoxylate metabolism
Formate
Nitric oxide
K04561...
K15877
Nitrous oxide
K00376
Nitrogen
K02586...
1.19.6.1
K20935
K20932...
Hydrazine
CO2
PGTG_00098...
HCO3-
PGTG_15886
Cyanate
Carbamate
Carbon fixation pathways in prokaryotes
K00368...
K17877
K00366
K00362...
K03385...
K00926
Nitrate
K02575
K15576...
K00367
K00370...
K10534
1.9.6.1
Nitrite
Ammonia
K01948
Carbamoyl-P
Arginine biosynthesis
Nitrate (extracellular)
K00370...
PGTG_14385
1.7.1.10
K05601...
K00261
K00262
PGTG_15142...
Nitrite (extracellular)
K02575
K10535
K10944...
PGTG_06623...
PGTG_15142...
Glutamate metabolism
Hydroxylamine
L-Glutamine
L-Glutamate
K00459
K19823
K01501
K00284
Nitroalkane
Nitrile
Oxidation state
+5
+3
+2
+1
0
-1
-2
-3
Dissimilatory nitrate reduction
Nitrate
K00370...
K02567...
Nitrite
K00362...
K03385...
Ammonia
Assimilatory nitrate reduction
Nitrate
K00367
K10534
K00372...
Nitrite
K17877
K00366
Ammonia
Denitrification
Nitrate
K00370...
K02567...
Nitrite
K00368
K15864
Nitric oxide
K04561...
Nitrous oxide
K00376
Nitrogen
Nitrogen fixation
Nitrogen
K02586...
K00531
Ammonia
Nitrification
Nitrate
K00370...
Nitrite
K10535
Hydroxylamine
K10944...
Ammonia
Anammox
Nitrogen
K20935
Hydrazine
K20932...
Ammonia
Nitrite
K00368
K15864
Nitric oxide
00910 7/21/17
(c) Kanehisa Laboratories

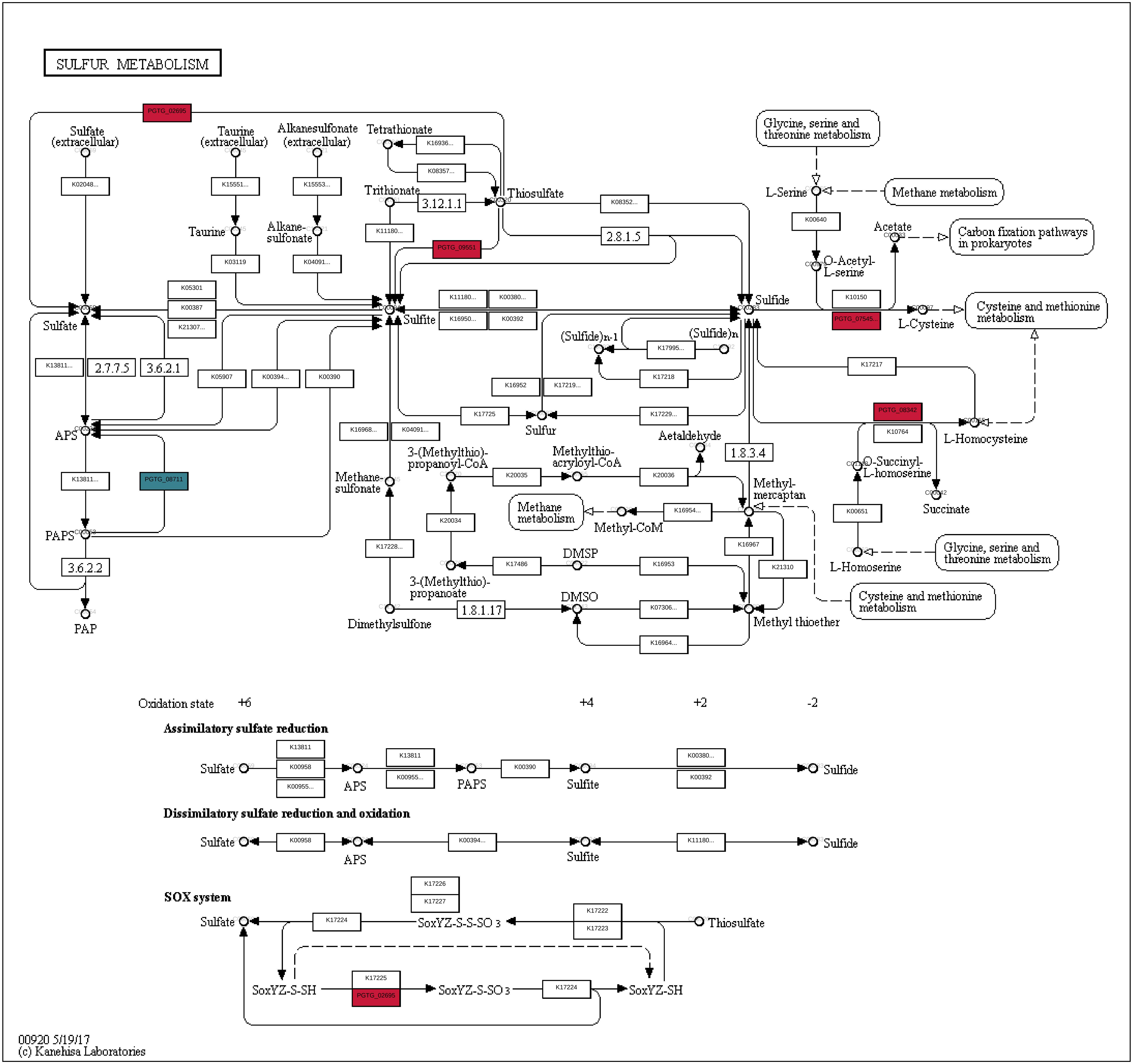
SULFUR METABOLISM
PGTG_02695
Sulfate (extracellular)
Taurine (extracellular)
Alkanesulfonate (extracellular)
Tetrathionate
K16936...
K08357...
Trithionate
3.12.1.1
Thiosulfate
K08352...
2.8.1.5
K02048...
K15551...
K15553...
Taurine
Alkane-sulfonate
K03119
K04091...
K11180...
PGTG_09551
Glycine, serine and threonine metabolism
L-Serine
Methane metabolism
K00640
Acetate
Carbon fixation pathways in prokaryotes
O-Acetyl-L-serine
K10150
PGTG_07545...
L-Cysteine
Cysteine and methionine metabolism
K05301
K00387
K21307...
Sulfate
K11180...
K00380...
K16950...
K00392
Sulfite
Sulfide
(Sulfide)n-1
(Sulfide)n
K17995...
K17218
K17217
K13811...
2.7.7.5
3.6.2.1
K05907
K00394...
K00390
K16952
K17219...
K17725
Sulfur
K17229...
PGTG_08342
K10764
L-Homocysteine
APS
K16968...
K04091...
Aetaldehyde
1.8.3.4
3-(Methylthio)-propanoyl-CoA
Methylthio-acryloyl-CoA
K20035
K20036
O-Succinyl-L-homoserine
K13811...
PGTG_08711
Methane-sulfonate
Methyl-mercaptan
Methane metabolism
Methyl-CoM
K16954...
Succinate
K20034
K00651
PAPS
K17228...
K16967
K17486
DMSP
K16953
K21310
Glycine, serine and threonine metabolism
L-Homoserine
3.6.2.2
3-(Methylthio)-propanoate
Cysteine and methionine metabolism
DMSO
1.8.1.17
K07306...
PAP
Dimethylsulfone
Methyl thioether
K16964...
Oxidation state +6 +4 +2 -2
Assimilatory sulfate reduction
Sulfate
K13811
K00958
K00955...
APS
K13811
K00955...
PAPS
K00390
Sulfite
K00380...
K00392
Sulfide
Dissimilatory sulfate reduction and oxidation
Sulfate
K00958
APS
K00394...
Sulfite
K11180...
Sulfide
SOX system
K17226
K17227
Sulfate
K17224
SoxYZ-S-S-SO3
K17222
K17223
Thiosulfate
SoxYZ-S-SH
K17225
PGTG_02695
SoxYZ-S-SO3
K17224
SoxYZ-SH
00920 5/19/17
(c) Kanehisa Laboratories

# 3. Lipid metabolism

| MAP | PATHWAY |
|---|---|
| **61** | Fatty acid biosynthesis |
| **62** | Fatty acid elongation |
| **71** | Fatty acid degradation |
| **72** | Synthesis and degradation of ketone bodies |
| **100** | Steroid biosynthesis |
| **561** | Glycerolipid metabolism |
| **564** | Glycerophospholipid metabolism |
| **565** | Ether lipid metabolism |
| **590** | Arachidonic acid metabolism |
| **592** | alpha-Linolenic acid metabolism |
| **600** | Sphingolipid metabolism |

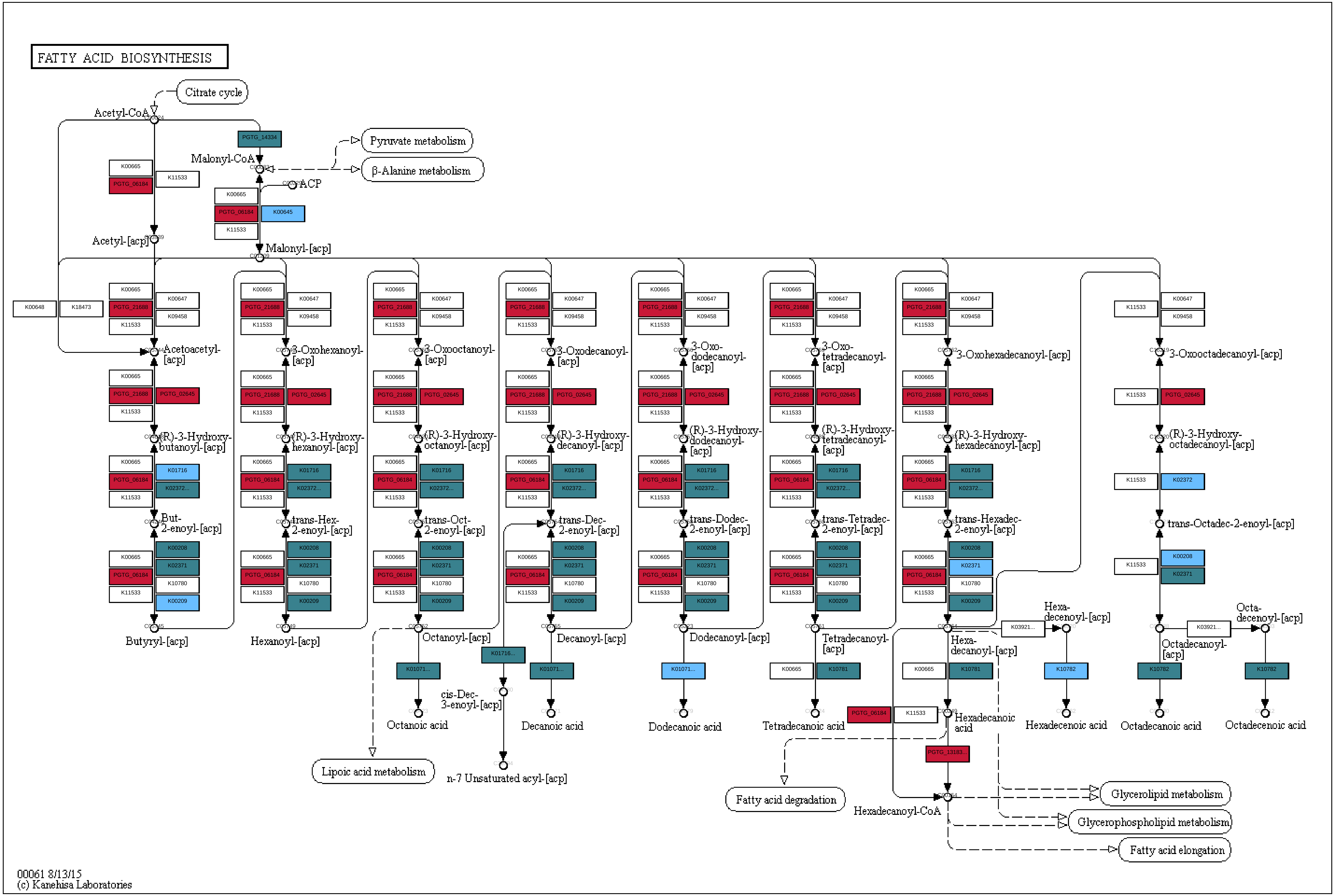
FATTY ACID BIOSYNTHESIS
Citrate cycle
Acetyl-CoA
Malonyl-CoA
Pyruvate metabolism
β-Alanine metabolism
ACP
Acetyl-[acp]
Malonyl-[acp]
PGTG_14334
K00665
PGTG_06184
K11533
K00645
K00647
K09458
PGTG_21688
PGTG_02645
K01716
K02372...
K00208
K02371
K10780
K00209
K00648
K18473
Acetoacetyl-[acp]
3-Oxohexanoyl-[acp]
3-Oxooctanoyl-[acp]
3-Oxodecanoyl-[acp]
3-Oxo-dodecanoyl-[acp]
3-Oxo-tetradecanoyl-[acp]
3-Oxohexadecanoyl-[acp]
3-Oxooctadecanoyl-[acp]
(R)-3-Hydroxy-butanoyl-[acp]
(R)-3-Hydroxy-hexanoyl-[acp]
(R)-3-Hydroxy-octanoyl-[acp]
(R)-3-Hydroxy-decanoyl-[acp]
(R)-3-Hydroxy-dodecanoyl-[acp]
(R)-3-Hydroxy-tetradecanoyl-[acp]
(R)-3-Hydroxy-hexadecanoyl-[acp]
(R)-3-Hydroxy-octadecanoyl-[acp]
But-2-enoyl-[acp]
trans-Hex-2-enoyl-[acp]
trans-Oct-2-enoyl-[acp]
trans-Dec-2-enoyl-[acp]
trans-Dodec-2-enoyl-[acp]
trans-Tetradec-2-enoyl-[acp]
trans-Hexadec-2-enoyl-[acp]
trans-Octadec-2-enoyl-[acp]
Butyryl-[acp]
Hexanoyl-[acp]
Octanoyl-[acp]
Decanoyl-[acp]
Dodecanoyl-[acp]
Tetradecanoyl-[acp]
Hexa-decanoyl-[acp]
Octadecanoyl-[acp]
Hexa-decenoyl-[acp]
Octa-decenoyl-[acp]
K03921...
K01071...
K01716...
K10781
K10782
PGTG_13183...
cis-Dec-3-enoyl-[acp]
Octanoic acid
Decanoic acid
Dodecanoic acid
Tetradecanoic acid
Hexadecanoic acid
Hexadecenoic acid
Octadecanoic acid
Octadecenoic acid
Lipoic acid metabolism
n-7 Unsaturated acyl-[acp]
Fatty acid degradation
Hexadecanoyl-CoA
Glycerolipid metabolism
Glycerophospholipid metabolism
Fatty acid elongation
00061 8/13/15
(c) Kanehisa Laboratories

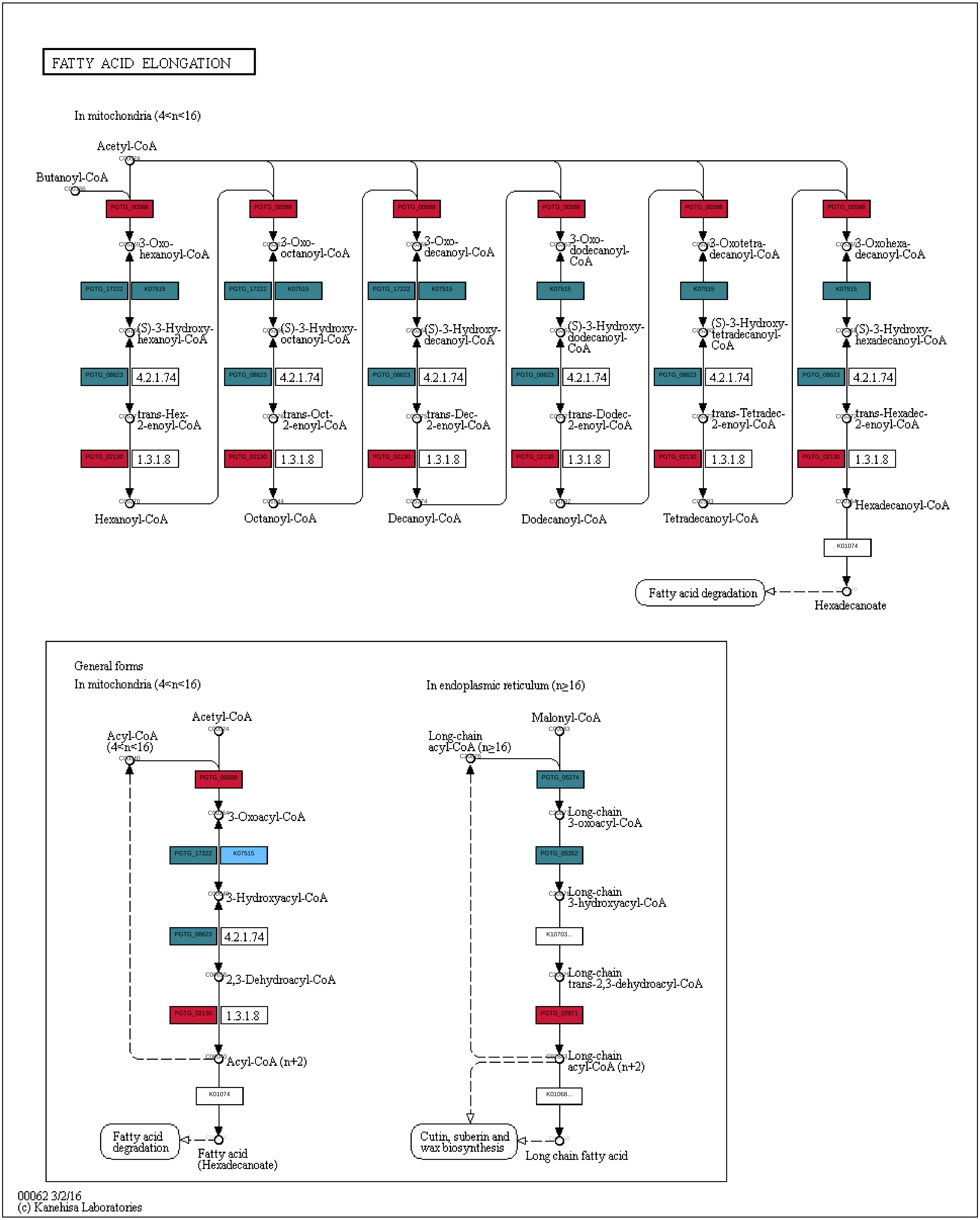
FATTY ACID ELONGATION
In mitochondria (4<n<16)
Acetyl-CoA
Butanoyl-CoA
PGTG_00588
3-Oxo-hexanoyl-CoA
3-Oxo-octanoyl-CoA
3-Oxo-decanoyl-CoA
3-Oxo-dodecanoyl-CoA
3-Oxotetra-decanoyl-CoA
3-Oxohexa-decanoyl-CoA
PGTG_17222
K07515
(S)-3-Hydroxy-hexanoyl-CoA
(S)-3-Hydroxy-octanoyl-CoA
(S)-3-Hydroxy-decanoyl-CoA
(S)-3-Hydroxy-dodecanoyl-CoA
(S)-3-Hydroxy-tetradecanoyl-CoA
(S)-3-Hydroxy-hexadecanoyl-CoA
PGTG_08623
4.2.1.74
trans-Hex-2-enoyl-CoA
trans-Oct-2-enoyl-CoA
trans-Dec-2-enoyl-CoA
trans-Dodec-2-enoyl-CoA
trans-Tetradec-2-enoyl-CoA
trans-Hexadec-2-enoyl-CoA
PGTG_02130
1.3.1.8
Hexanoyl-CoA
Octanoyl-CoA
Decanoyl-CoA
Dodecanoyl-CoA
Tetradecanoyl-CoA
Hexadecanoyl-CoA
K01074
Fatty acid degradation
Hexadecanoate
General forms
In mitochondria (4<n<16)
Acetyl-CoA
Acyl-CoA (4<n<16)
3-Oxoacyl-CoA
3-Hydroxyacyl-CoA
2,3-Dehydroacyl-CoA
Acyl-CoA (n+2)
Fatty acid degradation
Fatty acid (Hexadecanoate)
In endoplasmic reticulum (n≥16)
Malonyl-CoA
Long-chain acyl-CoA (n≥16)
PGTG_05274
Long-chain 3-oxoacyl-CoA
PGTG_05352
Long-chain 3-hydroxyacyl-CoA
K10703...
Long-chain trans-2,3-dehydroacyl-CoA
PGTG_02971
Long-chain acyl-CoA (n+2)
K01068...
Cutin, suberin and wax biosynthesis
Long chain fatty acid
00062 3/2/16
(c) Kanehisa Laboratories

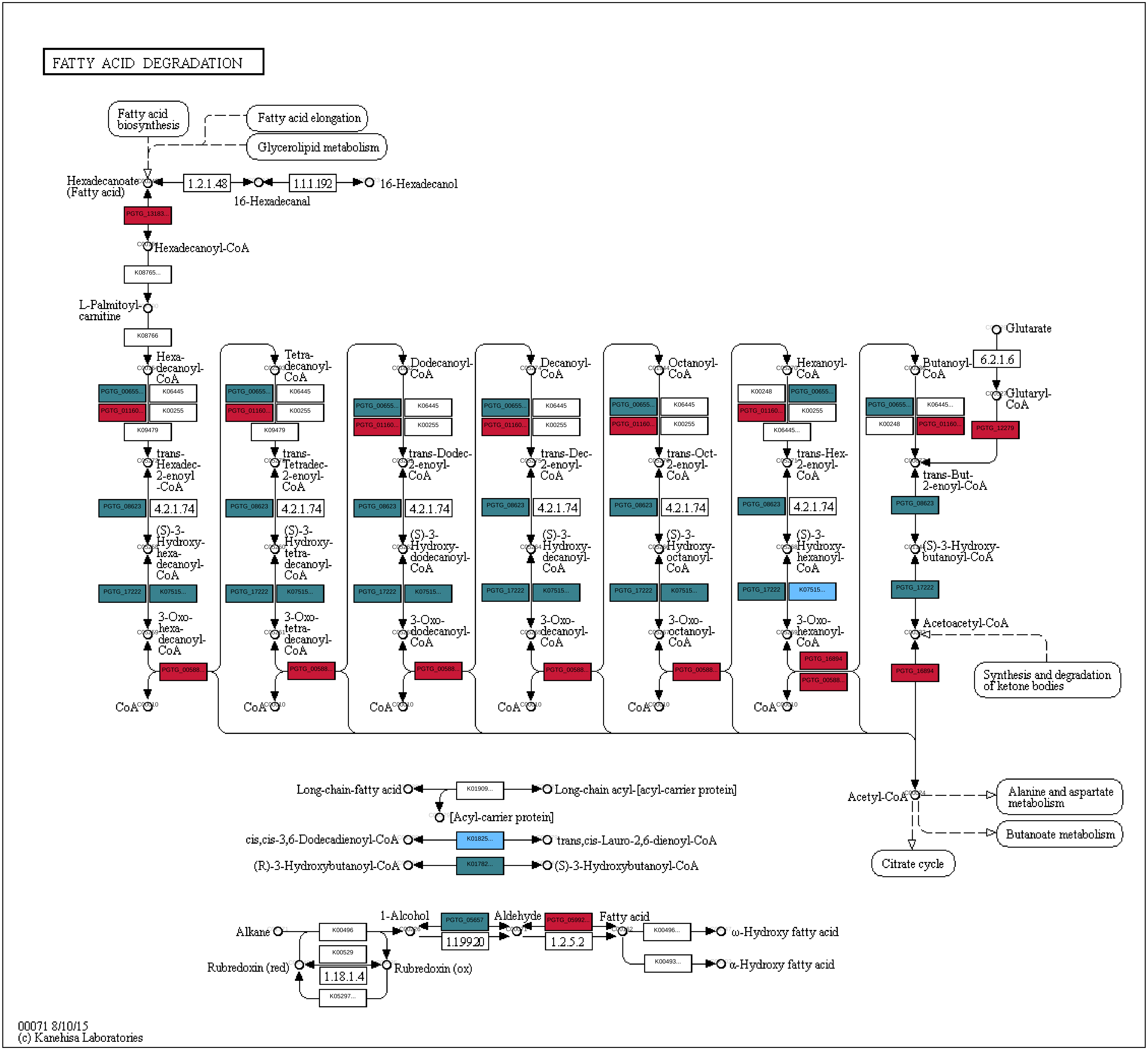

FATTY ACID DEGRADATION
Fatty acid biosynthesis
Fatty acid elongation
Glycerolipid metabolism
Hexadecanoate (Fatty acid)
1.2.1.48
16-Hexadecanal
1.1.1.192
16-Hexadecanol
PGTG_13183...
Hexadecanoyl-CoA
K08765...
L-Palmitoyl-carnitine
K08766
Hexa-decanoyl-CoA
PGTG_00655...
K06445
PGTG_01160...
K00255
K09479
trans-Hexadec-2-enoyl-CoA
PGTG_08623
4.2.1.74
(S)-3-Hydroxy-hexa-decanoyl-CoA
PGTG_17222
K07515...
3-Oxo-hexa-decanoyl-CoA
PGTG_00588...
CoA
Tetra-decanoyl-CoA
trans-Tetradec-2-enoyl-CoA
(S)-3-Hydroxy-tetra-decanoyl-CoA
3-Oxo-tetra-decanoyl-CoA
Dodecanoyl-CoA
trans-Dodec-2-enoyl-CoA
(S)-3-Hydroxy-dodecanoyl-CoA
3-Oxo-dodecanoyl-CoA
Decanoyl-CoA
trans-Dec-2-enoyl-CoA
(S)-3-Hydroxy-decanoyl-CoA
3-Oxo-decanoyl-CoA
Octanoyl-CoA
trans-Oct-2-enoyl-CoA
(S)-3-Hydroxy-octanoyl-CoA
3-Oxo-octanoyl-CoA
Hexanoyl-CoA
K00248
K06445...
trans-Hex-2-enoyl-CoA
(S)-3-Hydroxy-hexanoyl-CoA
3-Oxo-hexanoyl-CoA
PGTG_16894
Glutarate
6.2.1.6
Butanoyl-CoA
Glutaryl-CoA
PGTG_12279
trans-But-2-enoyl-CoA
(S)-3-Hydroxy-butanoyl-CoA
Acetoacetyl-CoA
Synthesis and degradation of ketone bodies
Acetyl-CoA
Alanine and aspartate metabolism
Butanoate metabolism
Citrate cycle
Long-chain-fatty acid
K01909...
Long-chain acyl-[acyl-carrier protein]
[Acyl-carrier protein]
cis,cis-3,6-Dodecadienoyl-CoA
K01825...
trans,cis-Lauro-2,6-dienoyl-CoA
(R)-3-Hydroxybutanoyl-CoA
K01782...
(S)-3-Hydroxybutanoyl-CoA
Alkane
K00496
K00529
1.18.1.4
K05297...
Rubredoxin (red)
Rubredoxin (ox)
1-Alcohol
PGTG_05657
1.1.99.20
Aldehyde
PGTG_05992...
1.2.5.2
Fatty acid
K00496...
ω-Hydroxy fatty acid
K00493...
α-Hydroxy fatty acid
00071 8/10/15
(c) Kanehisa Laboratories

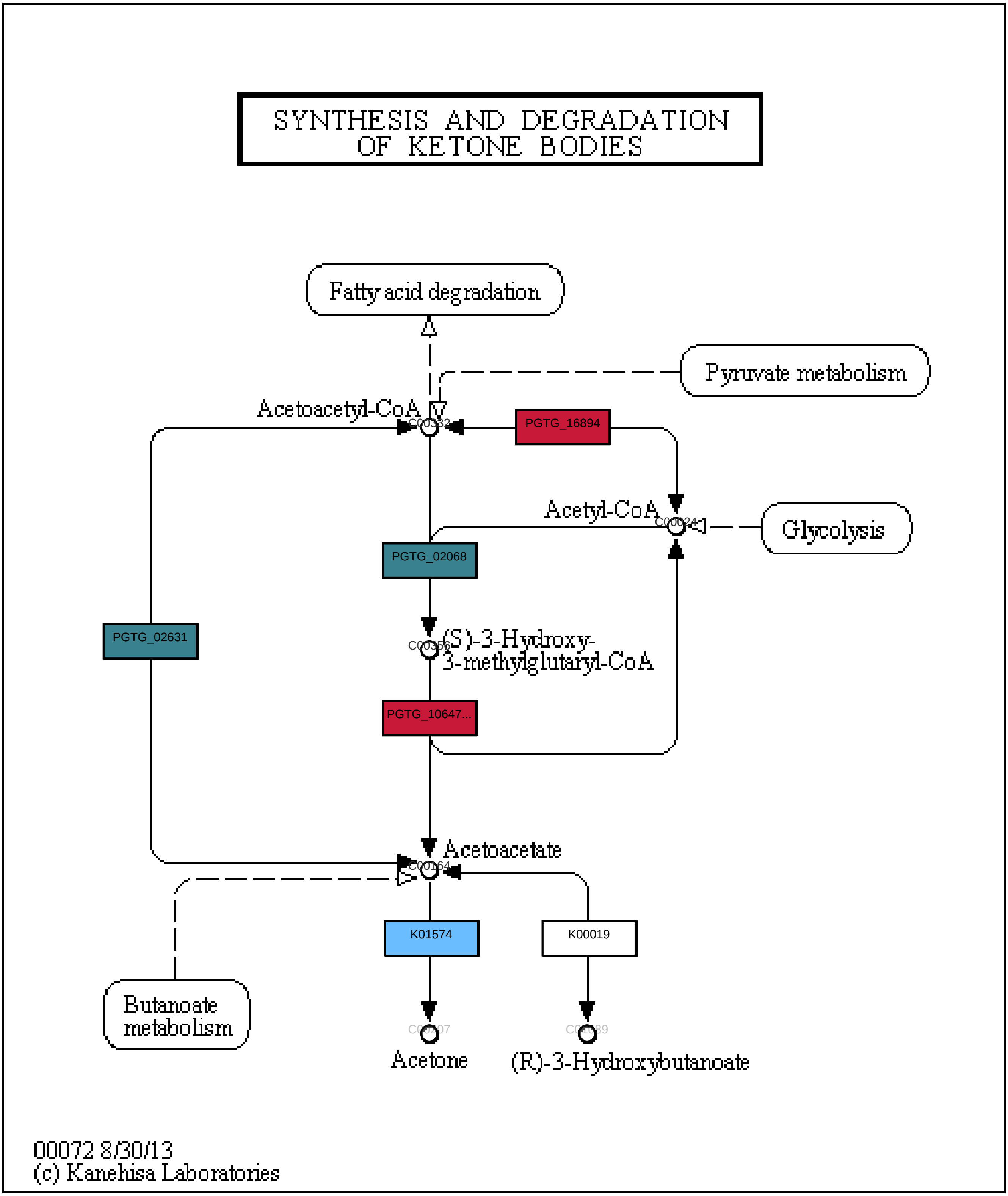

SYNTHESIS AND DEGRADATION OF KETONE BODIES
Fatty acid degradation
Pyruvate metabolism
Acetoacetyl-CoA
C00332
PGTG_16894
Acetyl-CoA
C00024
Glycolysis
PGTG_02068
PGTG_02631
(S)-3-Hydroxy-3-methylglutaryl-CoA
C00356
PGTG_10647...
Acetoacetate
C00164
K01574
K00019
Butanoate metabolism
Acetone
(R)-3-Hydroxybutanoate
00072 8/30/13
(c) Kanehisa Laboratories

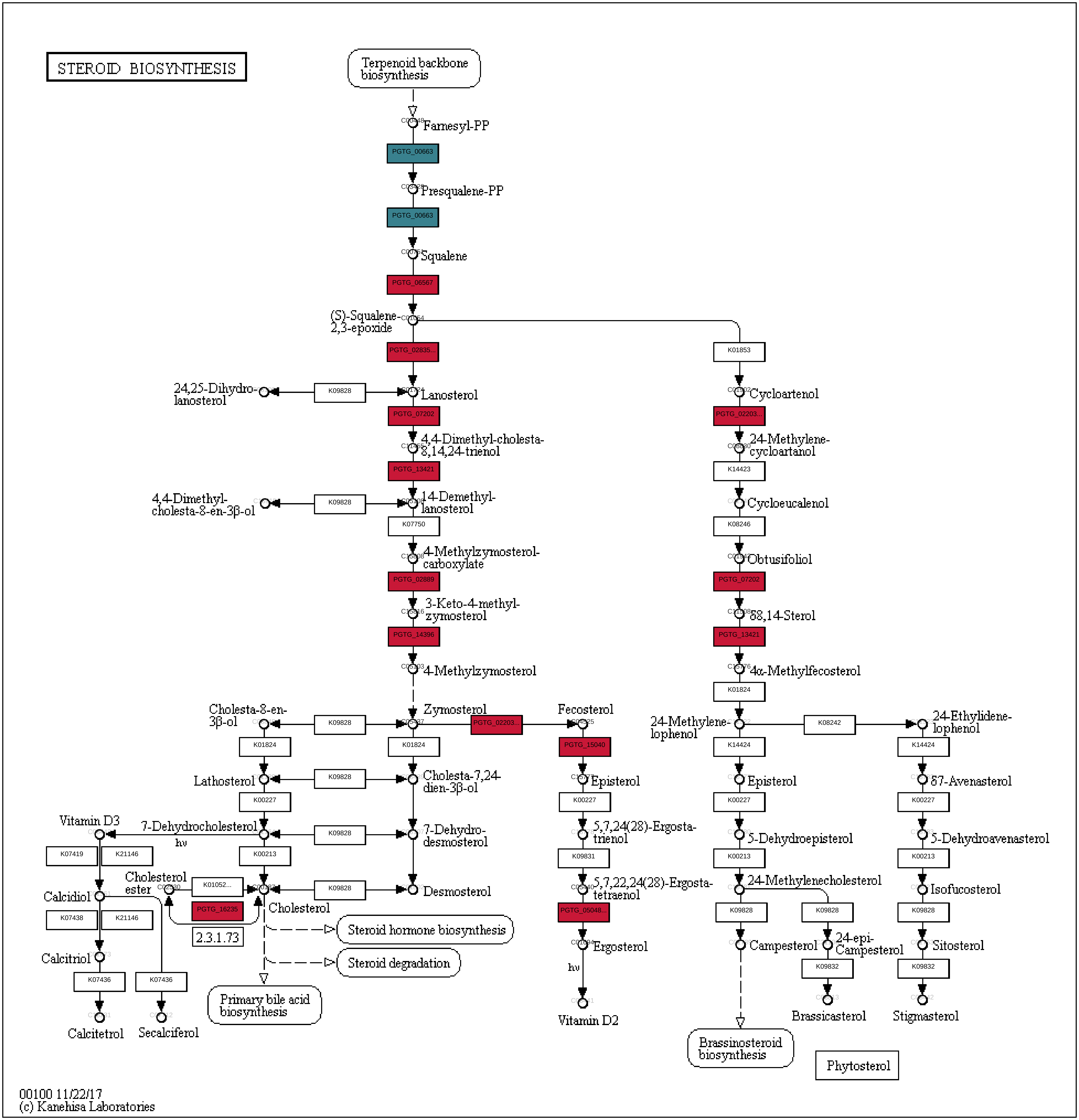
STEROID BIOSYNTHESIS
Terpenoid backbone biosynthesis
Farnesyl-PP
PGTG_00663
Presqualene-PP
PGTG_00663
Squalene
PGTG_06567
(S)-Squalene-2,3-epoxide
C01054
PGTG_02835...
K01853
24,25-Dihydro-lanosterol
K09828
Lanosterol
Cycloartenol
PGTG_07202
PGTG_02203...
4,4-Dimethyl-cholesta-8,14,24-trienol
24-Methylene-cycloartanol
PGTG_13421
K14423
4,4-Dimethyl-cholesta-8-en-3β-ol
K09828
14-Demethyl-lanosterol
Cycloeucalenol
K07750
K08246
4-Methylzymosterol-carboxylate
Obtusifoliol
PGTG_02889
PGTG_07202
3-Keto-4-methyl-zymosterol
δ8,14-Sterol
PGTG_14396
PGTG_13421
4-Methylzymosterol
4α-Methylfecosterol
K01824
Cholesta-8-en-3β-ol
K09828
Zymosterol
PGTG_02203...
Fecosterol
24-Methylene-lophenol
K08242
24-Ethylidene-lophenol
K01824
K01824
PGTG_15040
K14424
K14424
Lathosterol
K09828
Cholesta-7,24-dien-3β-ol
Episterol
Episterol
δ7-Avenasterol
K00227
K00227
K00227
K00227
Vitamin D3
7-Dehydrocholesterol
K09828
7-Dehydro-desmosterol
5,7,24(28)-Ergosta-trienol
5-Dehydroepisterol
5-Dehydroavenasterol
hν
K07419
K21146
K00213
K09831
K00213
K00213
Cholesterol ester
K01052...
K09828
Desmosterol
5,7,22,24(28)-Ergosta-tetraenol
24-Methylenecholesterol
Isofucosterol
Calcidiol
PGTG_16235
Cholesterol
PGTG_05048...
K09828
K09828
K09828
K07438
K21146
2.3.1.73
Steroid hormone biosynthesis
Calcitriol
Steroid degradation
Ergosterol
Campesterol
24-epi-Campesterol
Sitosterol
K07436
K07436
hν
K09832
K09832
Primary bile acid biosynthesis
Calcitetrol
Secalciferol
Vitamin D2
Brassicasterol
Stigmasterol
Brassinosteroid biosynthesis
Phytosterol
00100 11/22/17
(c) Kanehisa Laboratories

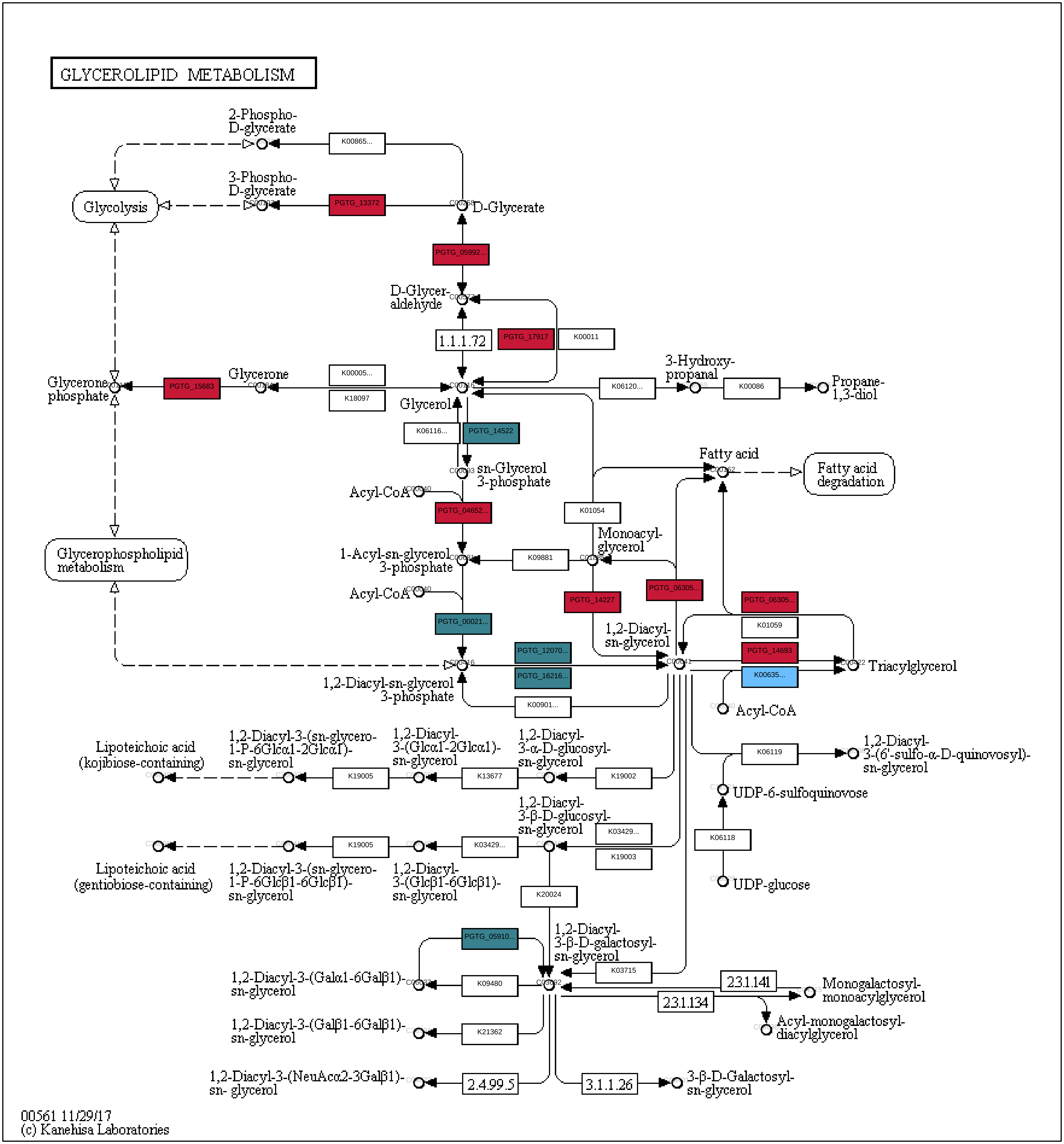
GLYCEROLIPID METABOLISM
2-Phospho-D-glycerate
K00865...
3-Phospho-D-glycerate
PGTG_13372
D-Glycerate
Glycolysis
PGTG_05992...
D-Glycer-aldehyde
1.1.1.72
PGTG_17917
K00011
Glycerone phosphate
PGTG_15683
Glycerone
K00005...
K18097
Glycerol
K06120...
3-Hydroxy-propanal
K00086
Propane-1,3-diol
K06116...
PGTG_14522
sn-Glycerol 3-phosphate
Fatty acid
Fatty acid degradation
Acyl-CoA
PGTG_04652...
K01054
Monoacyl-glycerol
Glycerophospholipid metabolism
1-Acyl-sn-glycerol 3-phosphate
K09881
PGTG_06305...
PGTG_14227
Acyl-CoA
PGTG_00021...
1,2-Diacyl-sn-glycerol
PGTG_06305...
K01059
PGTG_12070...
PGTG_14693
PGTG_16216...
K00635...
Triacylglycerol
1,2-Diacyl-sn-glycerol 3-phosphate
K00901...
Acyl-CoA
Lipoteichoic acid (kojibiose-containing)
1,2-Diacyl-3-(sn-glycero-1-P-6Glcα1-2Glcα1)-sn-glycerol
1,2-Diacyl-3-(Glcα1-2Glcα1)-sn-glycerol
1,2-Diacyl-3-α-D-glucosyl-sn-glycerol
K19005
K13677
K19002
K06119
1,2-Diacyl-3-(6'-sulfo-α-D-quinovosyl)-sn-glycerol
UDP-6-sulfoquinovose
1,2-Diacyl-3-β-D-glucosyl-sn-glycerol
K03429...
K19005
K03429...
K19003
K06118
Lipoteichoic acid (gentiobiose-containing)
1,2-Diacyl-3-(sn-glycero-1-P-6Glcβ1-6Glcβ1)-sn-glycerol
1,2-Diacyl-3-(Glcβ1-6Glcβ1)-sn-glycerol
UDP-glucose
K20024
PGTG_05910...
1,2-Diacyl-3-β-D-galactosyl-sn-glycerol
1,2-Diacyl-3-(Galα1-6Galβ1)-sn-glycerol
K09480
K03715
2.3.1.141
Monogalactosyl-monoacylglycerol
2.3.1.134
Acyl-monogalactosyl-diacylglycerol
1,2-Diacyl-3-(Galβ1-6Galβ1)-sn-glycerol
K21362
1,2-Diacyl-3-(NeuAcα2-3Galβ1)-sn- glycerol
2.4.99.5
3.1.1.26
3-β-D-Galactosyl-sn-glycerol
00561 11/29/17
(c) Kanehisa Laboratories

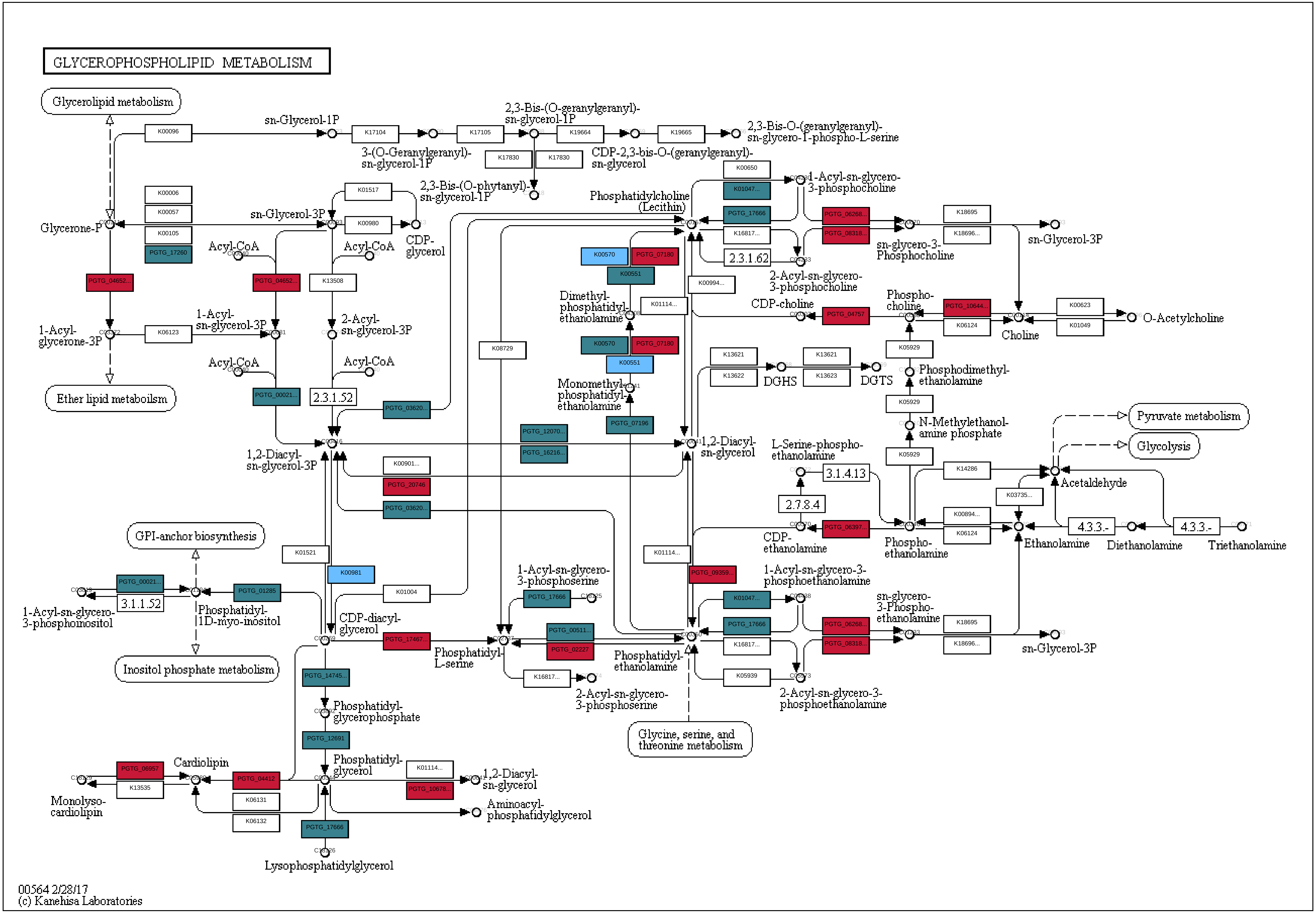
GLYCEROPHOSPHOLIPID METABOLISM
Glycerolipid metabolism
K00096
sn-Glycerol-1P
K17104
K17105
2,3-Bis-(O-geranylgeranyl)-sn-glycerol-1P
K19664
K19665
2,3-Bis-O-(geranylgeranyl)-sn-glycero-1-phospho-L-serine
3-(O-Geranylgeranyl)-sn-glycerol-1P
K17830
K17830
CDP-2,3-bis-O-(geranylgeranyl)-sn-glycerol
K00650
K01047...
PGTG_17666
K16817...
2.3.1.62
1-Acyl-sn-glycero-3-phosphocholine
PGTG_06268...
PGTG_08318...
K18695
K18696...
sn-Glycerol-3P
sn-glycero-3-Phosphocholine
2-Acyl-sn-glycero-3-phosphocholine
Phosphatidylcholine (Lecithin)
2,3-Bis-(O-phytanyl)-sn-glycerol-1P
K00006
K00057
K00105
PGTG_17260
Glycerone-P
sn-Glycerol-3P
K01517
K00980
CDP-glycerol
Acyl-CoA
Acyl-CoA
K13508
PGTG_04652...
PGTG_04652...
1-Acyl-glycerone-3P
K06123
1-Acyl-sn-glycerol-3P
2-Acyl-sn-glycerol-3P
Acyl-CoA
Acyl-CoA
PGTG_00021...
2.3.1.52
PGTG_03620...
Ether lipid metaboilsm
1,2-Diacyl-sn-glycerol-3P
K00901...
PGTG_20746
PGTG_03620...
K00570
PGTG_07180
K00551
Dimethyl-phosphatidyl-ethanolamine
K01114...
K00994...
K08729
K00570
PGTG_07180
K00551
Monomethyl-phosphatidyl-ethanolamine
PGTG_07196
PGTG_12070...
PGTG_16216...
1,2-Diacyl-sn-glycerol
CDP-choline
PGTG_04757
Phospho-choline
PGTG_10644...
K06124
Choline
K00623
K01049
O-Acetylcholine
K13621
K13622
DGHS
K13621
K13623
DGTS
K05929
Phosphodimethyl-ethanolamine
K05929
N-Methylethanol-amine phosphate
K05929
L-Serine-phospho-ethanolamine
3.1.4.13
2.7.8.4
Pyruvate metabolism
Glycolysis
K14286
Acetaldehyde
K03735...
K00894...
K06124
CDP-ethanolamine
PGTG_06397...
Phospho-ethanolamine
Ethanolamine
4.3.3.-
Diethanolamine
4.3.3.-
Triethanolamine
GPI-anchor biosynthesis
K01521
K00981
K01114...
PGTG_09359...
PGTG_00021...
3.1.1.52
PGTG_01285
K01004
1-Acyl-sn-glycero-3-phosphoinositol
Phosphatidyl-1D-myo-inositol
CDP-diacyl-glycerol
1-Acyl-sn-glycero-3-phosphoserine
PGTG_17666
1-Acyl-sn-glycero-3-phosphoethanolamine
K01047...
PGTG_17666
K16817...
PGTG_06268...
PGTG_08318...
sn-glycero-3-Phospho-ethanolamine
K18695
K18696...
sn-Glycerol-3P
Inositol phosphate metabolism
PGTG_17467...
Phosphatidyl-L-serine
PGTG_00511...
PGTG_02227
Phosphatidyl-ethanolamine
K16817...
2-Acyl-sn-glycero-3-phosphoserine
K05939
2-Acyl-sn-glycero-3-phosphoethanolamine
PGTG_14745...
Phosphatidyl-glycerophosphate
Glycine, serine, and threonine metabolism
PGTG_12691
PGTG_06957
K13535
Cardiolipin
PGTG_04412
K06131
K06132
Phosphatidyl-glycerol
K01114...
PGTG_10678...
1,2-Diacyl-sn-glycerol
Aminoacyl-phosphatidylglycerol
Monolyso-cardiolipin
PGTG_17666
Lysophosphatidylglycerol
00564 2/28/17
(c) Kanehisa Laboratories

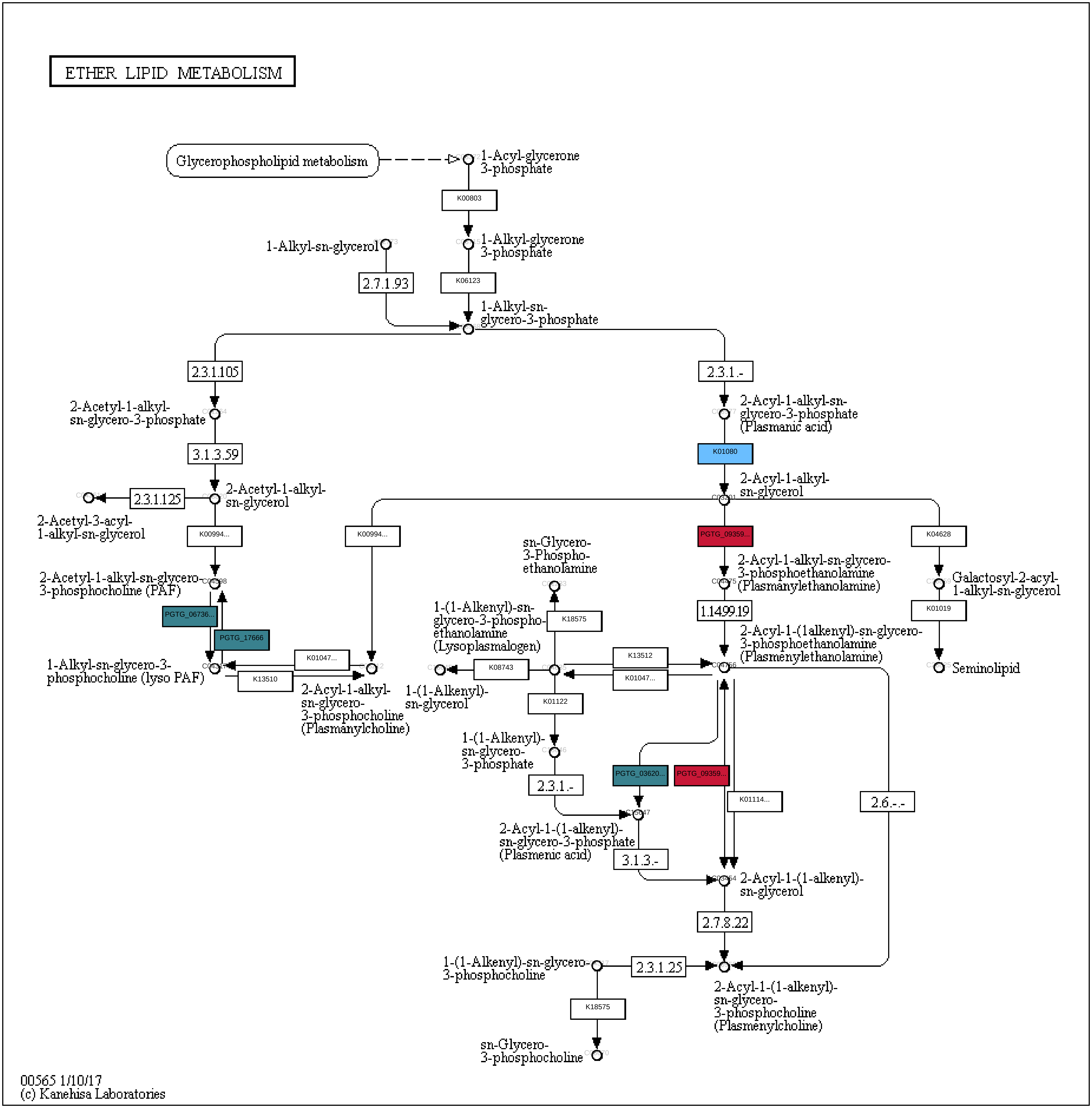
ETHER LIPID METABOLISM
Glycerophospholipid metabolism
1-Acyl-glycerone 3-phosphate
K00803
1-Alkyl-sn-glycerol
1-Alkyl-glycerone 3-phosphate
2.7.1.93
K06123
1-Alkyl-sn-glycero-3-phosphate
2.3.1.105
2.3.1.-
2-Acetyl-1-alkyl-sn-glycero-3-phosphate
2-Acyl-1-alkyl-sn-glycero-3-phosphate (Plasmanic acid)
3.1.3.59
K01080
2.3.1.125
2-Acetyl-1-alkyl-sn-glycerol
2-Acyl-1-alkyl-sn-glycerol
2-Acetyl-3-acyl-1-alkyl-sn-glycerol
K00994...
K00994...
PGTG_09359...
K04628
sn-Glycero-3-Phospho-ethanolamine
2-Acetyl-1-alkyl-sn-glycero-3-phosphocholine (PAF)
2-Acyl-1-alkyl-sn-glycero-3-phosphoethanolamine (Plasmanylethanolamine)
Galactosyl-2-acyl-1-alkyl-sn-glycerol
PGTG_06736...
PGTG_17666
1-(1-Alkenyl)-sn-glycero-3-phospho-ethanolamine (Lysoplasmalogen)
K18575
1.14.99.19
K01019
2-Acyl-1-(1alkenyl)-sn-glycero-3-phosphoethanolamine (Plasmenylethanolamine)
K01047...
K13512
1-Alkyl-sn-glycero-3-phosphocholine (lyso PAF)
K13510
K08743
K01047...
Seminolipid
2-Acyl-1-alkyl-sn-glycero-3-phosphocholine (Plasmanylcholine)
1-(1-Alkenyl)-sn-glycerol
K01122
1-(1-Alkenyl)-sn-glycero-3-phosphate
PGTG_03620...
PGTG_09359...
2.3.1.-
K01114...
2.6.-.-
2-Acyl-1-(1-alkenyl)-sn-glycero-3-phosphate (Plasmenic acid)
3.1.3.-
2-Acyl-1-(1-alkenyl)-sn-glycerol
2.7.8.22
1-(1-Alkenyl)-sn-glycero-3-phosphocholine
2.3.1.25
K18575
2-Acyl-1-(1-alkenyl)-sn-glycero-3-phosphocholine (Plasmenylcholine)
sn-Glycero-3-phosphocholine
00565 1/10/17
(c) Kanehisa Laboratories

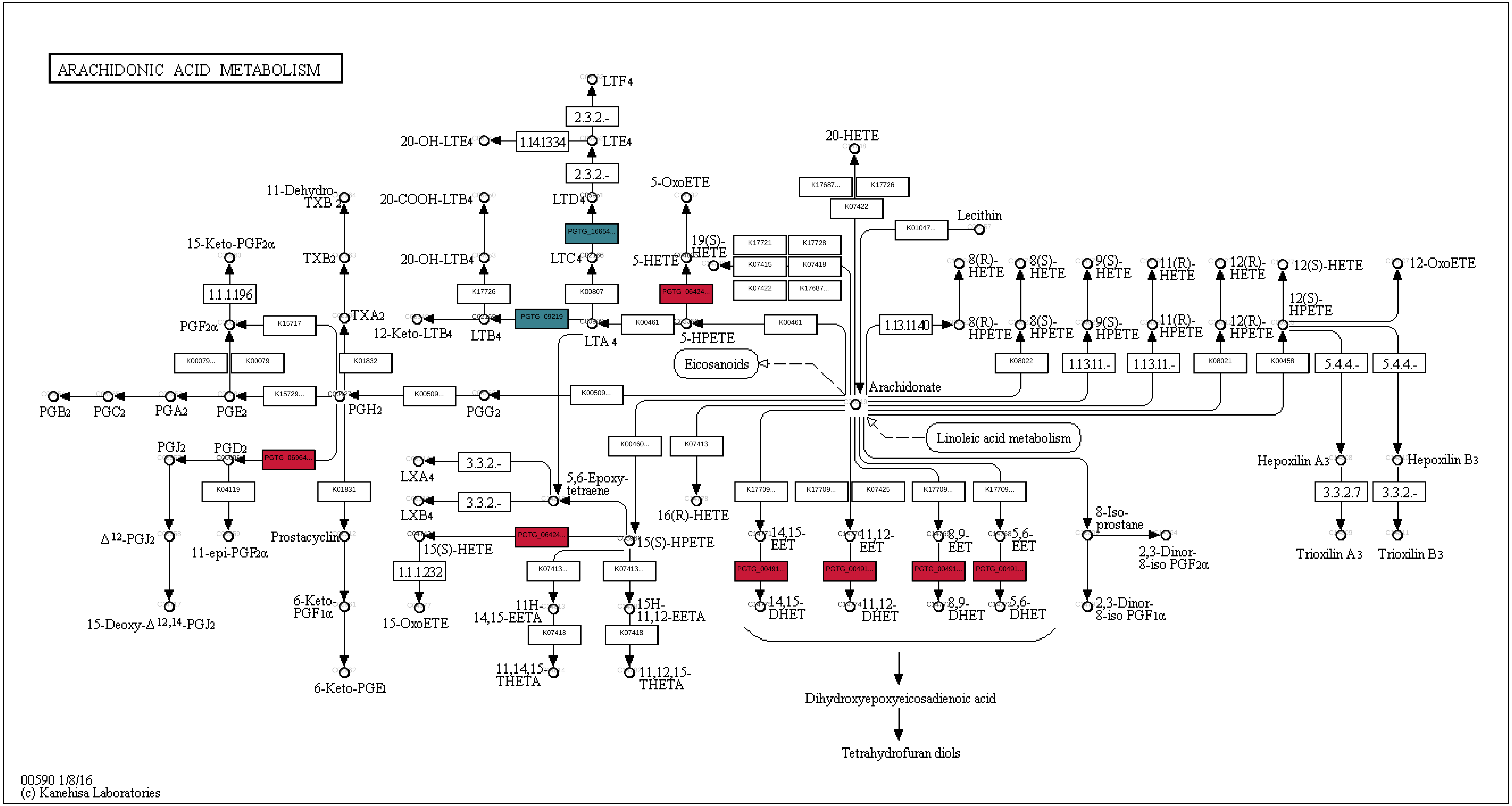
ARACHIDONIC ACID METABOLISM
LTF4
2.3.2.-
20-OH-LTE4
1.14.13.34
LTE4
2.3.2.-
11-Dehydro-TXB 2
20-COOH-LTB4
LTD4
5-OxoETE
PGTG_16654...
15-Keto-PGF2α
TXB2
20-OH-LTB4
LTC4
19(S)-HETE
5-HETE
1.1.1.196
K17726
K00807
PGTG_06424...
TXA2
PGTG_09219
12-Keto-LTB4
LTB4
LTA 4
K00461
K00461
5-HPETE
PGF2α
K15717
K01832
K00079...
K00079
Eicosanoids
PGB2
PGC2
PGA2
PGE2
K15729...
PGH2
K00509...
PGG2
K00509...
PGJ2
PGD2
PGTG_06964...
K04119
K01831
Δ12-PGJ2
11-epi-PGF2α
Prostacyclin
15-Deoxy-Δ12,14-PGJ2
6-Keto-PGF1α
6-Keto-PGE1
LXA4
3.3.2.-
5,6-Epoxy-tetraene
LXB4
3.3.2.-
PGTG_06424...
15(S)-HETE
15(S)-HPETE
1.1.1.232
15-OxoETE
K07413...
K07413...
11H-14,15-EETA
15H-11,12-EETA
K07418
K07418
11,14,15-THETA
11,12,15-THETA
K00460...
K07413
16(R)-HETE
20-HETE
K17687...
K17726
K07422
Lecithin
K01047...
K17721
K17728
K07415
K07418
K07422
K17687...
Arachidonate
Linoleic acid metabolism
K17709...
K17709...
K07425
K17709...
K17709...
14,15-EET
11,12-EET
8,9-EET
5,6-EET
PGTG_00491...
PGTG_00491...
PGTG_00491...
PGTG_00491...
14,15-DHET
11,12-DHET
8,9-DHET
5,6-DHET
Dihydroxyepoxyeicosadienoic acid
Tetrahydrofuran diols
8(R)-HETE
8(S)-HETE
9(S)-HETE
11(R)-HETE
12(R)-HETE
12(S)-HETE
12-OxoETE
12(S)-HPETE
1.13.11.40
8(R)-HPETE
8(S)-HPETE
9(S)-HPETE
11(R)-HPETE
12(R)-HPETE
K08022
1.13.11.-
1.13.11.-
K08021
K00458
5.4.4.-
5.4.4.-
Hepoxilin A3
Hepoxilin B3
3.3.2.7
3.3.2.-
Trioxilin A3
Trioxilin B3
8-Iso-prostane
2,3-Dinor-8-iso PGF2α
2,3-Dinor-8-iso PGF1α
00590 1/8/16
(c) Kanehisa Laboratories

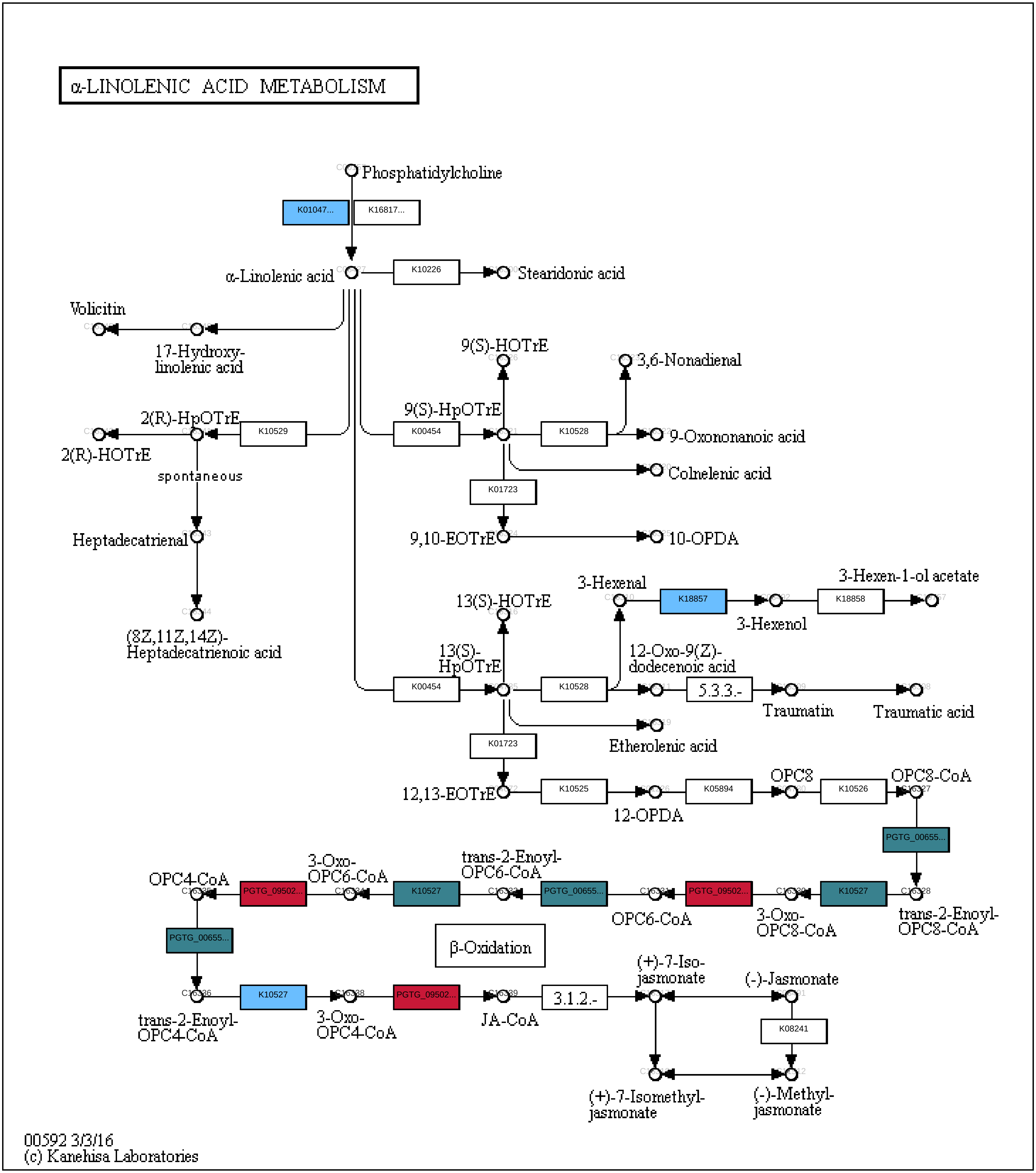

α-LINOLENIC ACID METABOLISM
Phosphatidylcholine
K01047...
K16817...
α-Linolenic acid
K10226
Stearidonic acid
Volicitin
17-Hydroxy-linolenic acid
9(S)-HOTrE
3,6-Nonadienal
2(R)-HpOTrE
K10529
9(S)-HpOTrE
K00454
K10528
9-Oxononanoic acid
2(R)-HOTrE
spontaneous
Colnelenic acid
K01723
Heptadecatrienal
9,10-EOTrE
10-OPDA
3-Hexenal
K18857
3-Hexen-1-ol acetate
K18858
13(S)-HOTrE
3-Hexenol
(8Z,11Z,14Z)-Heptadecatrienoic acid
13(S)-HpOTrE
12-Oxo-9(Z)-dodecenoic acid
K00454
K10528
5.3.3.-
Traumatin
Traumatic acid
Etherolenic acid
K01723
12,13-EOTrE
K10525
12-OPDA
K05894
OPC8
K10526
OPC8-CoA
PGTG_00655...
OPC4-CoA
3-Oxo-OPC6-CoA
trans-2-Enoyl-OPC6-CoA
PGTG_09502...
K10527
PGTG_00655...
PGTG_09502...
K10527
OPC6-CoA
3-Oxo-OPC8-CoA
trans-2-Enoyl-OPC8-CoA
PGTG_00655...
β-Oxidation
K10527
PGTG_09502...
3.1.2.-
(+)-7-Iso-jasmonate
(-)-Jasmonate
K08241
trans-2-Enoyl-OPC4-CoA
3-Oxo-OPC4-CoA
JA-CoA
(+)-7-Isomethyl-jasmonate
(-)-Methyl-jasmonate
00592 3/3/16
(c) Kanehisa Laboratories

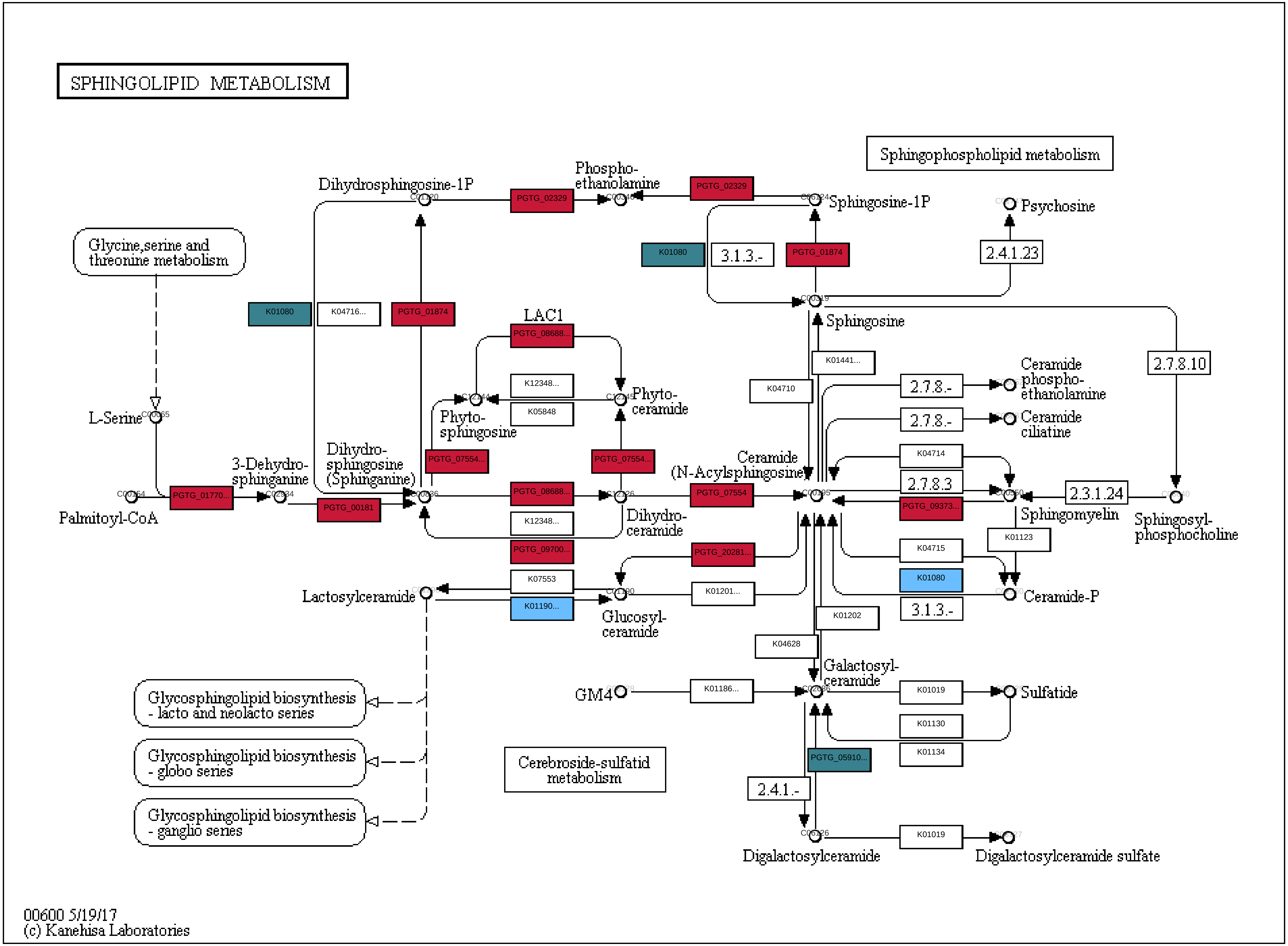
SPHINGOLIPID METABOLISM
Sphingophospholipid metabolism
Glycine,serine and threonine metabolism
Dihydrosphingosine-1P
Phospho-ethanolamine
PGTG_02329
PGTG_02329
Sphingosine-1P
Psychosine
K01080
3.1.3.-
PGTG_01874
2.4.1.23
K01080
K04716...
PGTG_01874
LAC1
PGTG_08688...
Sphingosine
K01441...
K04710
2.7.8.10
Ceramide phospho-ethanolamine
2.7.8.-
K12348...
K05848
Phyto-ceramide
Phyto-sphingosine
2.7.8.-
Ceramide ciliatine
L-Serine
Dihydro-sphingosine (Sphinganine)
3-Dehydro-sphinganine
PGTG_07554...
PGTG_07554...
Ceramide (N-Acylsphingosine)
K04714
2.7.8.3
Palmitoyl-CoA
PGTG_01770...
PGTG_00181
PGTG_08688...
PGTG_07554
PGTG_09373...
2.3.1.24
Sphingomyelin
Sphingosyl-phosphocholine
K12348...
Dihydro-ceramide
PGTG_09700...
PGTG_20281...
K01123
K04715
K01080
K07553
Lactosylceramide
K01190...
K01201...
Glucosyl-ceramide
Ceramide-P
3.1.3.-
K01202
K04628
Galactosyl-ceramide
GM4
K01186...
K01019
Sulfatide
K01130
K01134
PGTG_05910...
2.4.1.-
Glycosphingolipid biosynthesis - lacto and neolacto series
Glycosphingolipid biosynthesis - globo series
Glycosphingolipid biosynthesis - ganglio series
Cerebroside-sulfatid metabolism
Digalactosylceramide
K01019
Digalactosylceramide sulfate
00600 5/19/17
(c) Kanehisa Laboratories

# 4. Nucleotide metabolism

| MAP | PATHWAY |
|---|---|
| **230** | Purine metabolism |
| **240** | Pyrimidine metabolism |

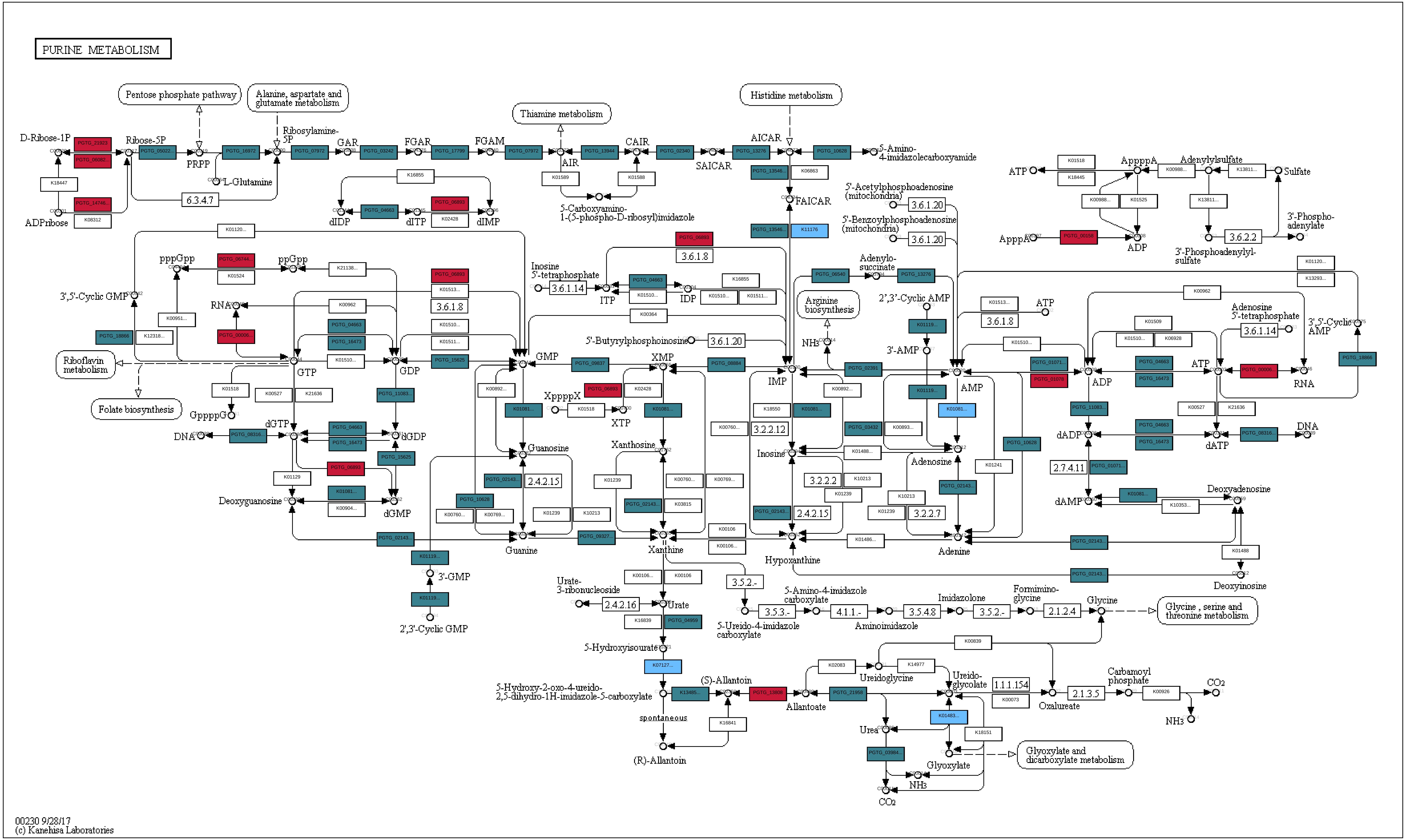
PURINE METABOLISM
Pentose phosphate pathway
Alanine, aspartate and glutamate metabolism
Thiamine metabolism
Histidine metabolism
D-Ribose-1P
Ribose-5P
PRPP
Ribosylamine-5P
GAR
FGAR
FGAM
AIR
CAIR
SAICAR
AICAR
5-Amino-4-imidazolecarboxyamide
L-Glutamine
6.3.4.7
ADPribose
5-Carboxyamino-1-(5-phospho-D-ribosyl)imidazole
dIDP
dITP
dIMP
FAICAR
ATP
ApppA
Adenylylsulfate
Sulfate
5'-Acetylphosphoadenosine (mitochondria)
5'-Benzoylphosphoadenosine (mitochondria)
3.6.1.20
AppppA
ADP
3'-Phosphoadenylyl-sulfate
3'-Phospho-adenylate
3.6.2.2
ppGpp
pppGpp
3',5'-Cyclic GMP
RNA
3.6.1.8
3.6.1.14
Inosine 5'-tetraphosphate
ITP
IDP
Adenylo-succinate
2',3'-Cyclic AMP
Arginine biosynthesis
Adenosine 5'-tetraphosphate
3',5'-Cyclic AMP
Riboflavin metabolism
Folate biosynthesis
GMP
GDP
GppppG
5'-Butyrylphosphoinosine
XMP
XppppX
XTP
IMP
NH3
3'-AMP
AMP
dGTP
dGDP
DNA
Guanosine
Xanthosine
Inosine
Adenosine
dADP
dATP
2.7.4.11
dAMP
Deoxyadenosine
Deoxyguanosine
dGMP
2.4.2.15
3.2.2.12
3.2.2.2
3.2.2.7
Guanine
Xanthine
Hypoxanthine
Adenine
Deoxyinosine
3'-GMP
2',3'-Cyclic GMP
Urate-3-ribonucleoside
2.4.2.16
Urate
3.5.2.-
5-Amino-4-imidazolone carboxylate
3.5.3.-
4.1.1.-
3.5.4.8
Imidazolone
3.5.2.-
Formimino-glycine
2.1.2.4
Glycine
Glycine, serine and threonine metabolism
5-Ureido-4-imidazole carboxylate
Aminoimidazolone
5-Hydroxyisourate
5-Hydroxy-2-oxo-4-ureido-2,5-dihydro-1H-imidazole-5-carboxylate
(S)-Allantoin
spontaneous
(R)-Allantoin
Allantoate
Ureidoglycine
Ureido-glycolate
1.1.1.154
Oxalureate
2.1.3.5
Carbamoyl phosphate
CO2
NH3
Urea
Glyoxylate
Glyoxylate and dicarboxylate metabolism
CO2
00230 9/28/17
(c) Kanehisa Laboratories

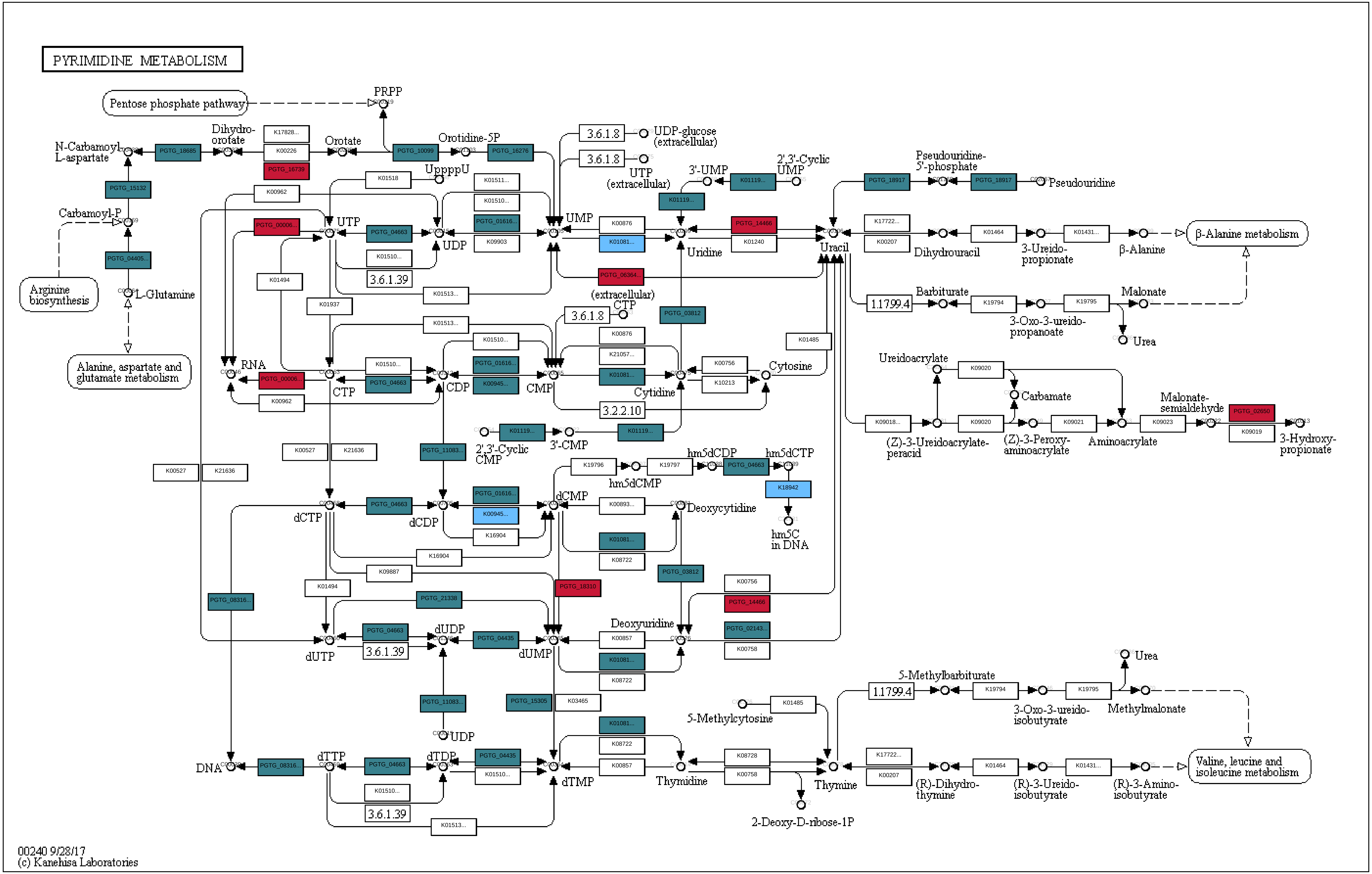
PYRIMIDINE METABOLISM
Pentose phosphate pathway
PRPP
N-Carbamoyl-
L-aspartate
PGTG_18685
Dihydro-
orotate
K17828...
K00226
PGTG_16739
Orotate
PGTG_10099
Orotidine-5P
PGTG_16276
3.6.1.8
UDP-glucose
(extracellular)
3.6.1.8
UTP
(extracellular)
PGTG_15132
Carbamoyl-P
PGTG_04405...
L-Glutamine
Arginine
biosynthesis
Alanine, aspartate and
glutamate metabolism
K01518
UppppU
K01511...
K01510...
PGTG_01616...
K09903
K00962
PGTG_00006...
UTP
PGTG_04663
UDP
K01510...
3.6.1.39
K01494
K01513...
K01937
UMP
K00876
K01081...
PGTG_06364...
(extracellular)
CTP
3'-UMP
K01119...
K01119...
2',3'-Cyclic
UMP
PGTG_14466
K01240
Uridine
Uracil
PGTG_03812
Pseudouridine-
5'-phosphate
PGTG_18917
PGTG_18917
Pseudouridine
K17722...
K00207
Dihydrouracil
K01464
3-Ureido-
propionate
K01431...
β-Alanine
β-Alanine metabolism
1.17.99.4
Barbiturate
K19794
3-Oxo-3-ureido-
propanoate
K19795
Malonate
Urea
K01485
Cytosine
K00756
K10213
3.6.1.8
K00876
K21057...
K01081...
Cytidine
3.2.2.10
RNA
PGTG_00006...
K00962
CTP
K01510...
PGTG_04663
K01513...
K01510...
PGTG_01616...
K00945...
CDP
CMP
K01119...
K01119...
2',3'-Cyclic
CMP
3'-CMP
K00527
K21636
K00527
K21636
PGTG_11083...
Ureidoacrylate
K09020
Carbamate
K09018...
K09020
K09021
(Z)-3-Ureidoacrylate-
peracid
(Z)-3-Peroxy-
aminoacrylate
Aminoacrylate
K09023
Malonate-
semialdehyde
PGTG_02650
K09019
3-Hydroxy-
propionate
K19796
hm5dCMP
K19797
hm5dCDP
PGTG_04663
hm5dCTP
K18942
hm5C
in DNA
dCTP
PGTG_04663
dCDP
PGTG_01616...
K00945...
K16904
dCMP
K00893...
Deoxycytidine
K01081...
K08722
K16904
K09887
PGTG_03812
K01494
PGTG_18310
K00756
PGTG_14466
PGTG_02143...
K00758
PGTG_08316...
PGTG_21338
PGTG_04663
dUDP
PGTG_04435
dUTP
3.6.1.39
dUMP
Deoxyuridine
K00857
K01081...
K08722
PGTG_11083...
PGTG_15305
K03465
UDP
Urea
5-Methylbarbiturate
1.17.99.4
K19794
K19795
3-Oxo-3-ureido-
isobutyrate
Methylmalonate
K01485
5-Methylcytosine
Valine, leucine and
isoleucine metabolism
K01081...
K08722
DNA
PGTG_08316...
dTTP
PGTG_04663
dTDP
PGTG_04435
K01510...
dTMP
K00857
Thymidine
K08728
K00758
Thymine
K17722...
K00207
(R)-Dihydro-
thymine
K01464
(R)-3-Ureido-
isobutyrate
K01431...
(R)-3-Amino-
isobutyrate
K01510...
3.6.1.39
K01513...
2-Deoxy-D-ribose-1P
00240 9/28/17
(c) Kanehisa Laboratories

# 5. Amino Acid metabolism

| MAP | PATHWAY |
|---|---|
| **220** | Arginine biosynthesis |
| **250** | Alanine, aspartate and glutamate metabolism |
| **260** | Glycine, serine and threonine metabolism |
| **270** | Cysteine and methionine metabolism |
| **280** | Valine, leucine and isoleucine degradation |
| **290** | Valine, leucine and isoleucine biosynthesis |
| **300** | Lysine biosynthesis |
| **310** | Lysine degradation |
| **330** | Arginine and proline metabolism |
| **340** | Histidine metabolism |
| **350** | Tyrosine metabolism |
| **360** | Phenylalanine metabolism |
| **380** | Tryptophan metabolism |
| **400** | Phenylalanine, tyrosine and tryptophan biosynthesis |

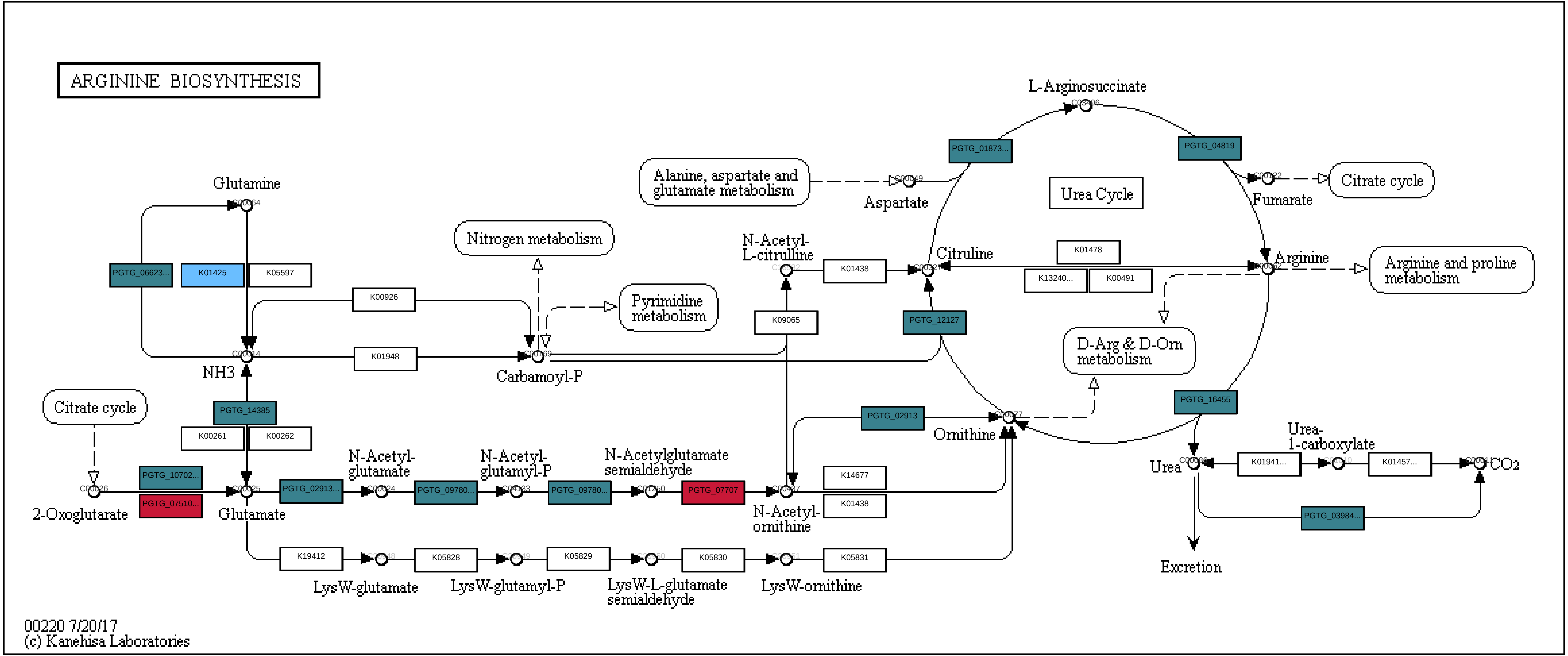
ARGININE BIOSYNTHESIS
Glutamine
PGTG_06623...
K01425
K05597
K00926
Nitrogen metabolism
Pyrimidine metabolism
K01948
NH3
Carbamoyl-P
Citrate cycle
PGTG_14385
K00261
K00262
PGTG_10702...
PGTG_07510...
2-Oxoglutarate
Glutamate
PGTG_02913...
N-Acetyl-glutamate
PGTG_09780...
N-Acetyl-glutamyl-P
PGTG_09780...
N-Acetylglutamate semialdehyde
PGTG_07707
N-Acetyl-ornithine
K14677
K01438
K19412
LysW-glutamate
K05828
LysW-glutamyl-P
K05829
LysW-L-glutamate semialdehyde
K05830
LysW-ornithine
K05831
Alanine, aspartate and glutamate metabolism
Aspartate
N-Acetyl-L-citrulline
K01438
K09065
Citrulline
PGTG_12127
PGTG_01873...
L-Arginosuccinate
PGTG_04819
Urea Cycle
Fumarate
Citrate cycle
K01478
K13240...
K00491
Arginine
Arginine and proline metabolism
D-Arg & D-Orn metabolism
PGTG_02913
Ornithine
PGTG_16455
Urea-1-carboxylate
Urea
K01941...
K01457...
CO2
PGTG_03984...
Excretion
00220 7/20/17
(c) Kanehisa Laboratories

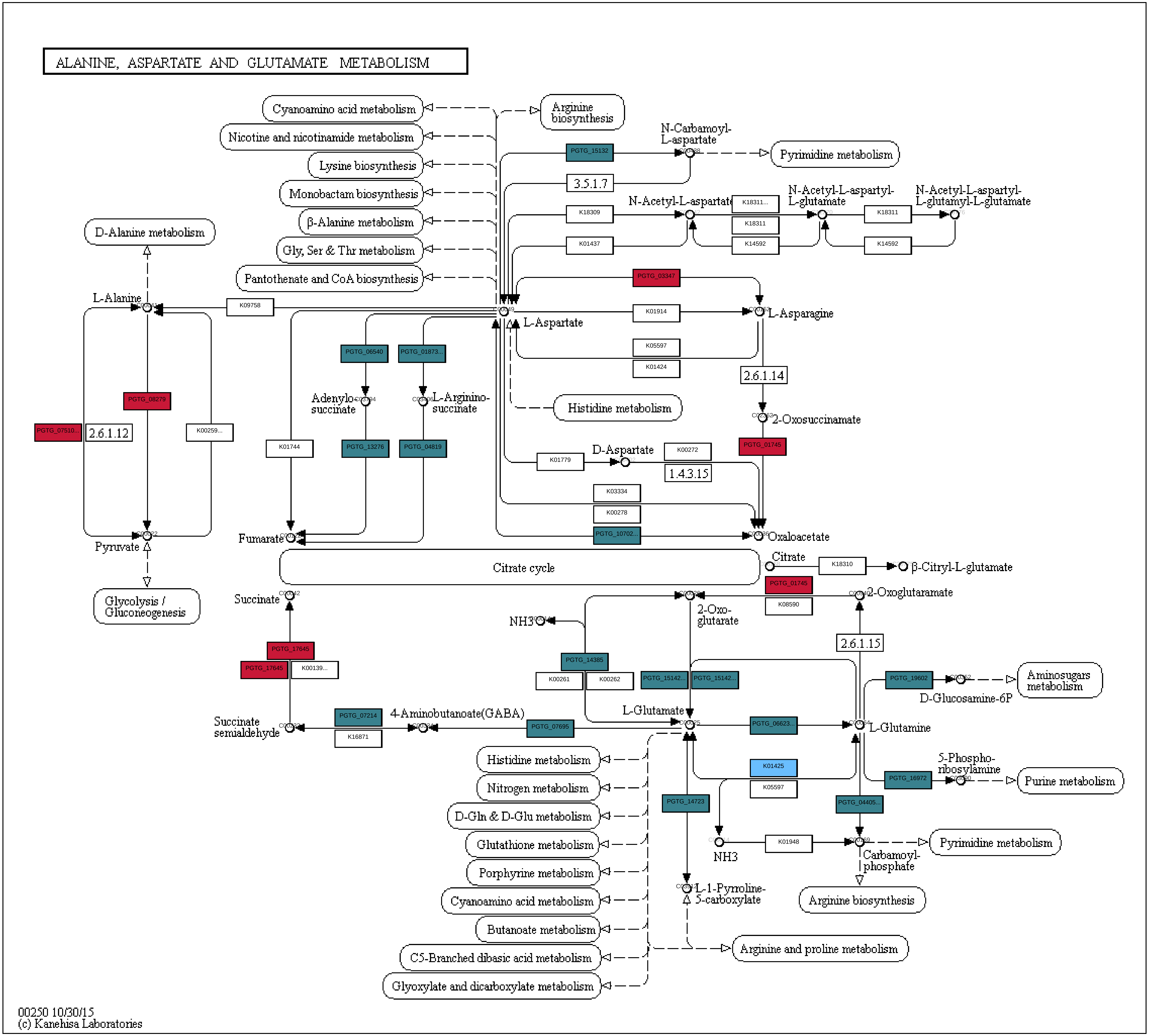
ALANINE, ASPARTATE AND GLUTAMATE METABOLISM
Cyanoamino acid metabolism
Nicotine and nicotinamide metabolism
Lysine biosynthesis
Monobactam biosynthesis
β-Alanine metabolism
Gly, Ser & Thr metabolism
Pantothenate and CoA biosynthesis
Arginine biosynthesis
N-Carbamoyl-L-aspartate
Pyrimidine metabolism
PGTG_15132
3.5.1.7
N-Acetyl-L-aspartate
N-Acetyl-L-aspartyl-L-glutamate
N-Acetyl-L-aspartyl-L-glutamyl-L-glutamate
K18309
K18311...
K18311
K14592
K01437
D-Alanine metabolism
L-Alanine
K09758
PGTG_03347
K01914
L-Aspartate
L-Asparagine
K05597
K01424
PGTG_06540
PGTG_01873...
Adenylo-succinate
L-Arginino-succinate
Histidine metabolism
2.6.1.14
2-Oxosuccinamate
PGTG_08279
PGTG_07510...
2.6.1.12
K00259...
K01744
PGTG_13276
PGTG_04819
D-Aspartate
K01779
K00272
1.4.3.15
PGTG_01745
K03334
K00278
PGTG_10702...
Fumarate
Oxaloacetate
Pyruvate
Citrate cycle
Citrate
K18310
β-Citryl-L-glutamate
Glycolysis / Gluconeogenesis
Succinate
K08590
2-Oxoglutaramate
NH3
2-Oxo-glutarate
2.6.1.15
PGTG_17645
K00139...
PGTG_14385
K00261
K00262
PGTG_15142...
PGTG_19602
Aminosugars metabolism
D-Glucosamine-6P
Succinate semialdehyde
PGTG_07214
K16871
4-Aminobutanoate(GABA)
PGTG_07695
L-Glutamate
PGTG_06623...
L-Glutamine
5-Phospho-ribosylamine
PGTG_16972
Purine metabolism
Nitrogen metabolism
D-Gln & D-Glu metabolism
Glutathione metabolism
Porphyrine metabolism
Butanoate metabolism
C5-Branched dibasic acid metabolism
Glyoxylate and dicarboxylate metabolism
K01425
PGTG_14723
PGTG_04405...
K01948
Carbamoyl-phosphate
L-1-Pyrroline-5-carboxylate
Arginine and proline metabolism
00250 10/30/15
(c) Kanehisa Laboratories

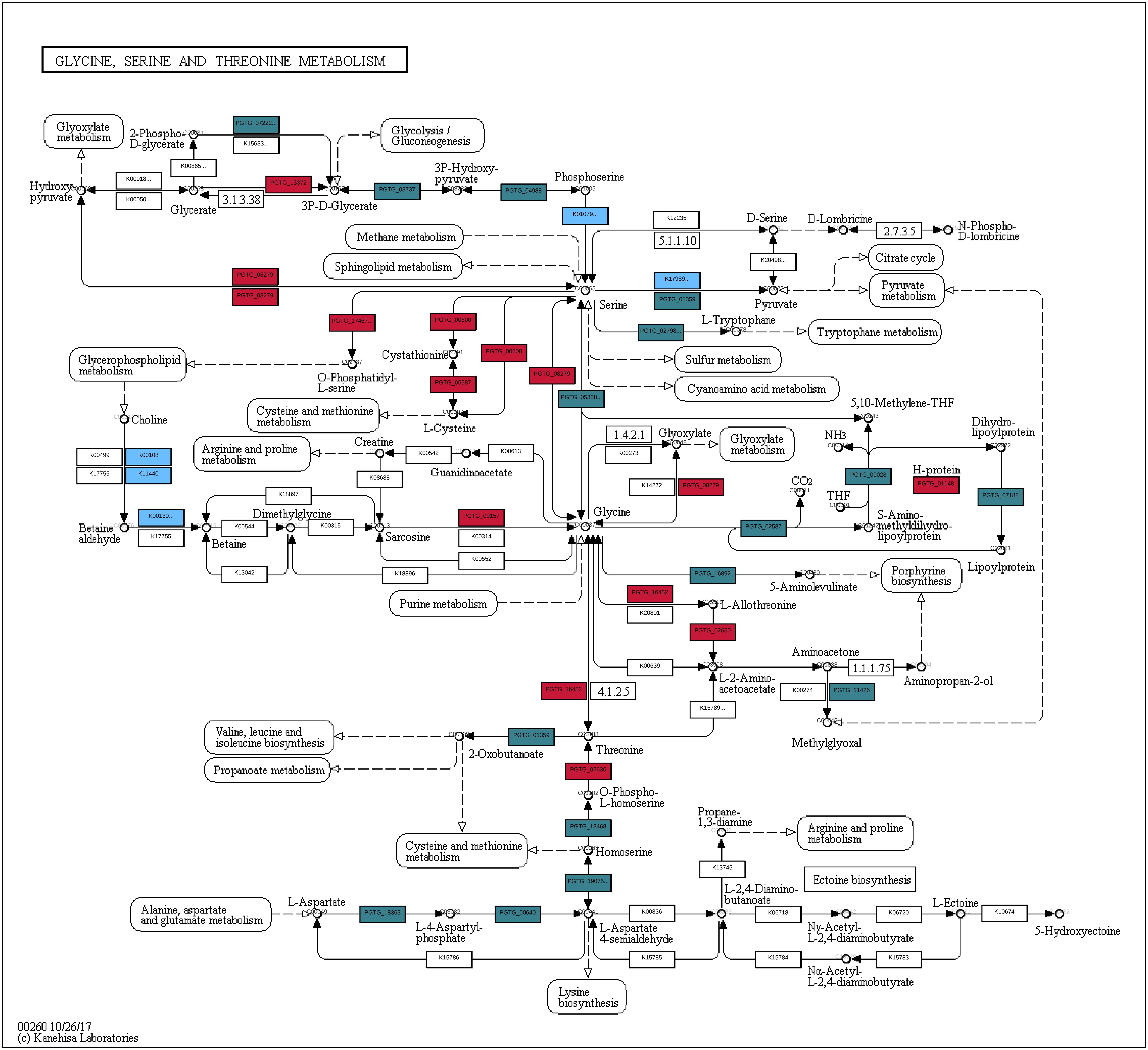
GLYCINE, SERINE AND THREONINE METABOLISM
Glyoxylate metabolism
2-Phospho-D-glycerate
PGTG_07222...
K15633...
Glycolysis / Gluconeogenesis
K00865...
K00018...
K00050...
Hydroxy-pyruvate
Glycerate
3.1.3.38
PGTG_13372
3P-D-Glycerate
PGTG_03737
3P-Hydroxy-pyruvate
PGTG_04988
Phosphoserine
K01079...
K12235
5.1.1.10
D-Serine
D-Lombricine
2.7.3.5
N-Phospho-D-lombricine
Methane metabolism
Sphingolipid metabolism
K20498...
Citrate cycle
PGTG_08279
PGTG_08279
K17989...
PGTG_01359
Pyruvate metabolism
Serine
Pyruvate
PGTG_17467...
PGTG_00600
L-Tryptophane
PGTG_02798...
Tryptophane metabolism
Glycerophospholipid metabolism
Cystathionine
PGTG_00600
PGTG_08279
Sulfur metabolism
O-Phosphatidyl-L-serine
PGTG_06587
PGTG_05338...
Cyanoamino acid metabolism
Cysteine and methionine metabolism
L-Cysteine
5,10-Methylene-THF
Choline
Creatine
K00542
K00613
1.4.2.1
Glyoxylate
Glyoxylate metabolism
NH3
Dihydro-lipoprotein
K00499
K00108
K17755
K11440
Arginine and proline metabolism
Guanidinoacetate
K08688
K00273
PGTG_00028
H-protein
PGTG_01148
K14272
PGTG_08279
CO2
THF
PGTG_07188
K18897
Dimethylglycine
PGTG_09157
Glycine
S-Amino-methyldihydro-lipoprotein
Betaine aldehyde
K00130...
K17755
K00544
K00315
Sarcosine
K00314
PGTG_02587
Betaine
K00552
Lipoylprotein
K13042
K18896
PGTG_16892
Porphyrine biosynthesis
5-Aminolevulinate
Purine metabolism
PGTG_16452
L-Allothreonine
K20801
PGTG_02650
Aminoacetone
K00639
1.1.1.75
Aminopropan-2-ol
L-2-Amino-acetoacetate
PGTG_16452
4.1.2.5
K00274
PGTG_11426
K15789...
Methylglyoxal
Valine, leucine and isoleucine biosynthesis
PGTG_01359
2-Oxobutanoate
Threonine
Propanoate metabolism
PGTG_02636
O-Phospho-L-homoserine
Propane-1,3-diamine
Arginine and proline metabolism
PGTG_18468
Cysteine and methionine metabolism
Homoserine
K13745
Ectoine biosynthesis
PGTG_19075...
L-2,4-Diamino-butanoate
L-Aspartate
Alanine, aspartate and glutamate metabolism
PGTG_18363
PGTG_00640
K00836
K06718
K06720
L-Ectoine
K10674
L-4-Aspartyl-phosphate
L-Aspartate 4-semialdehyde
Nγ-Acetyl-L-2,4-diaminobutyrate
5-Hydroxyectoine
K15786
K15785
K15784
K15783
Nα-Acetyl-L-2,4-diaminobutyrate
Lysine biosynthesis
00260 10/26/17
(c) Kanehisa Laboratories

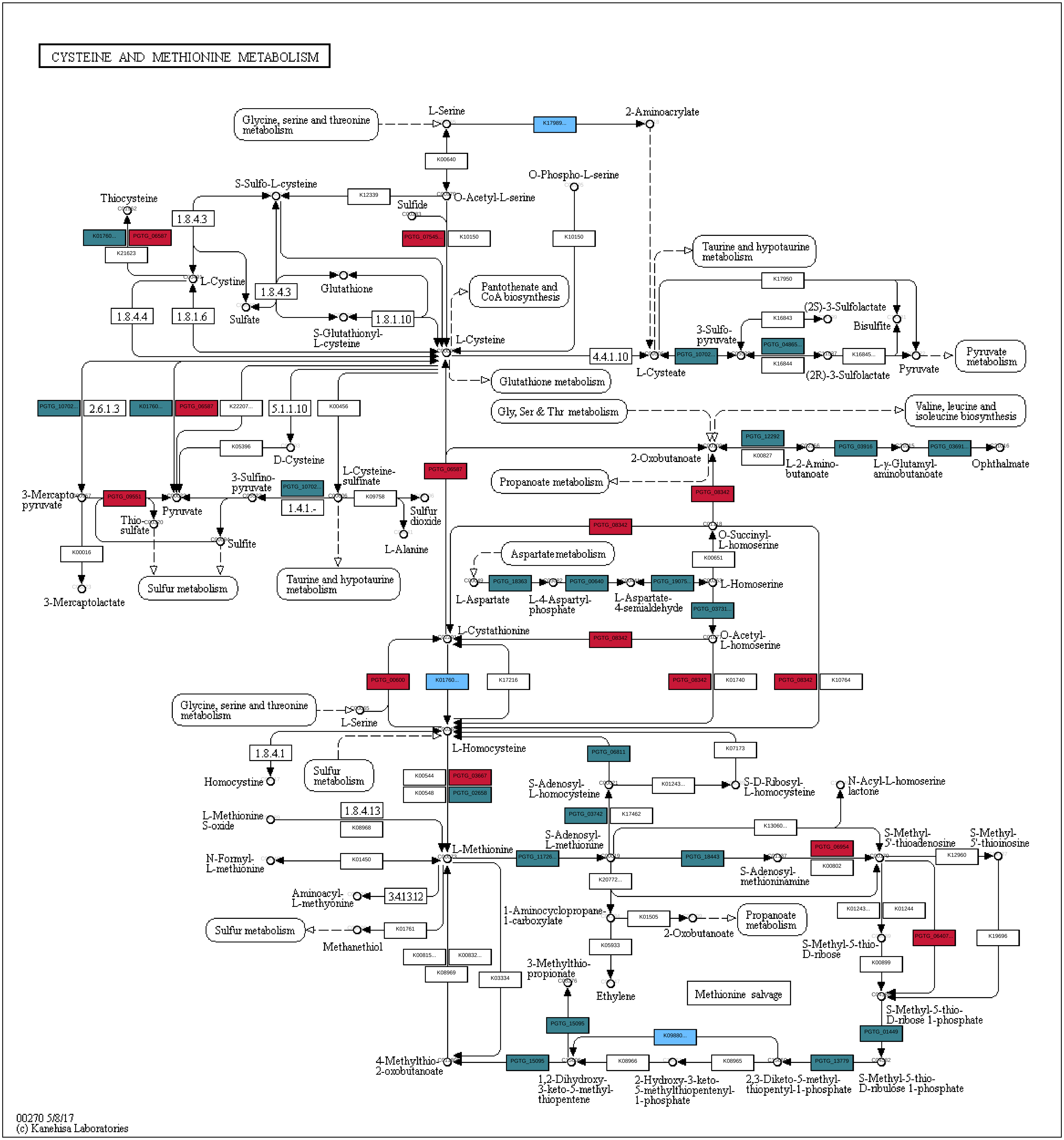
CYSTEINE AND METHIONINE METABOLISM
Glycine, serine and threonine metabolism
L-Serine
K17989...
2-Aminoacrylate
K00640
O-Phospho-L-serine
S-Sulfo-L-cysteine
K12339
O-Acetyl-L-serine
Sulfide
Thiocysteine
1.8.4.3
K01760...
PGTG_06587
PGTG_07545...
K10150
K10150
K21623
Taurine and hypotaurine metabolism
L-Cystine
1.8.4.3
Glutathione
Pantothenate and CoA biosynthesis
K17950
1.8.4.4
1.8.1.6
Sulfate
S-Glutathionyl-L-cysteine
1.8.1.10
L-Cysteine
(2S)-3-Sulfolactate
K16843
Bisulfite
3-Sulfo-pyruvate
PGTG_04865...
4.4.1.10
PGTG_10702...
K16845...
K16844
Pyruvate
Pyruvate metabolism
L-Cysteate
(2R)-3-Sulfolactate
Glutathione metabolism
PGTG_10702...
2.6.1.3
K01760...
PGTG_06587
K22207...
5.1.1.10
K00456
Gly, Ser & Thr metabolism
Valine, leucine and isoleucine biosynthesis
PGTG_12292
K05396
D-Cysteine
K00827
PGTG_03916
PGTG_03691...
2-Oxobutanoate
L-2-Amino-butanoate
L-γ-Glutamyl-aminobutanoate
Ophthalmate
PGTG_06587
L-Cysteine-sulfinate
Propanoate metabolism
3-Sulfino-pyruvate
PGTG_10702...
3-Mercapto-pyruvate
PGTG_09551
Pyruvate
K09758
1.4.1.-
PGTG_08342
Sulfur dioxide
Thio-sulfate
Sulfite
PGTG_08342
O-Succinyl-L-homoserine
K00016
L-Alanine
Aspartate metabolism
K00651
Sulfur metabolism
Taurine and hypotaurine metabolism
PGTG_18363
PGTG_00640
PGTG_19075...
L-Homoserine
3-Mercaptolactate
L-Aspartate
L-4-Aspartyl-phosphate
L-Aspartate-4-semialdehyde
PGTG_03731...
L-Cystathionine
PGTG_08342
O-Acetyl-L-homoserine
PGTG_00600
K01760...
K17216
PGTG_08342
K01740
PGTG_08342
K10764
Glycine, serine and threonine metabolism
L-Serine
L-Homocysteine
1.8.4.1
Sulfur metabolism
PGTG_06811
K07173
Homocystine
K00544
PGTG_03667
K00548
PGTG_02658
S-Adenosyl-L-homocysteine
K01243...
S-D-Ribosyl-L-homocysteine
N-Acyl-L-homoserine lactone
1.8.4.13
L-Methionine S-oxide
K08968
PGTG_03742
K17462
K13060...
S-Adenosyl-L-methionine
S-Methyl-5'-thioadenosine
S-Methyl-5'-thioinosine
N-Formyl-L-methionine
K01450
L-Methionine
PGTG_11726...
PGTG_18443
PGTG_06954
K00802
K12960
S-Adenosyl-methioninamine
K20772...
Aminoacyl-L-methyonine
3.4.13.12
1-Aminocyclopropane-1-carboxylate
K01505
2-Oxobutanoate
Propanoate metabolism
K01243...
K01244
Sulfur metabolism
K01761
Methanethiol
K05933
S-Methyl-5-thio-D-ribose
PGTG_06407...
K19696
K00815...
K00832...
K08969
K03334
3-Methylthio-propionate
Ethylene
Methionine salvage
K00899
S-Methyl-5-thio-D-ribose 1-phosphate
PGTG_15095
PGTG_01449
K09880...
4-Methylthio-2-oxobutanoate
PGTG_15095
K08966
K08965
PGTG_13779
1,2-Dihydroxy-3-keto-5-methyl-thiopentene
2-Hydroxy-3-keto-5-methylthiopentenyl-1-phosphate
2,3-Diketo-5-methyl-thiopentyl-1-phosphate
S-Methyl-5-thio-D-ribulose 1-phosphate
00270 5/8/17
(c) Kanehisa Laboratories

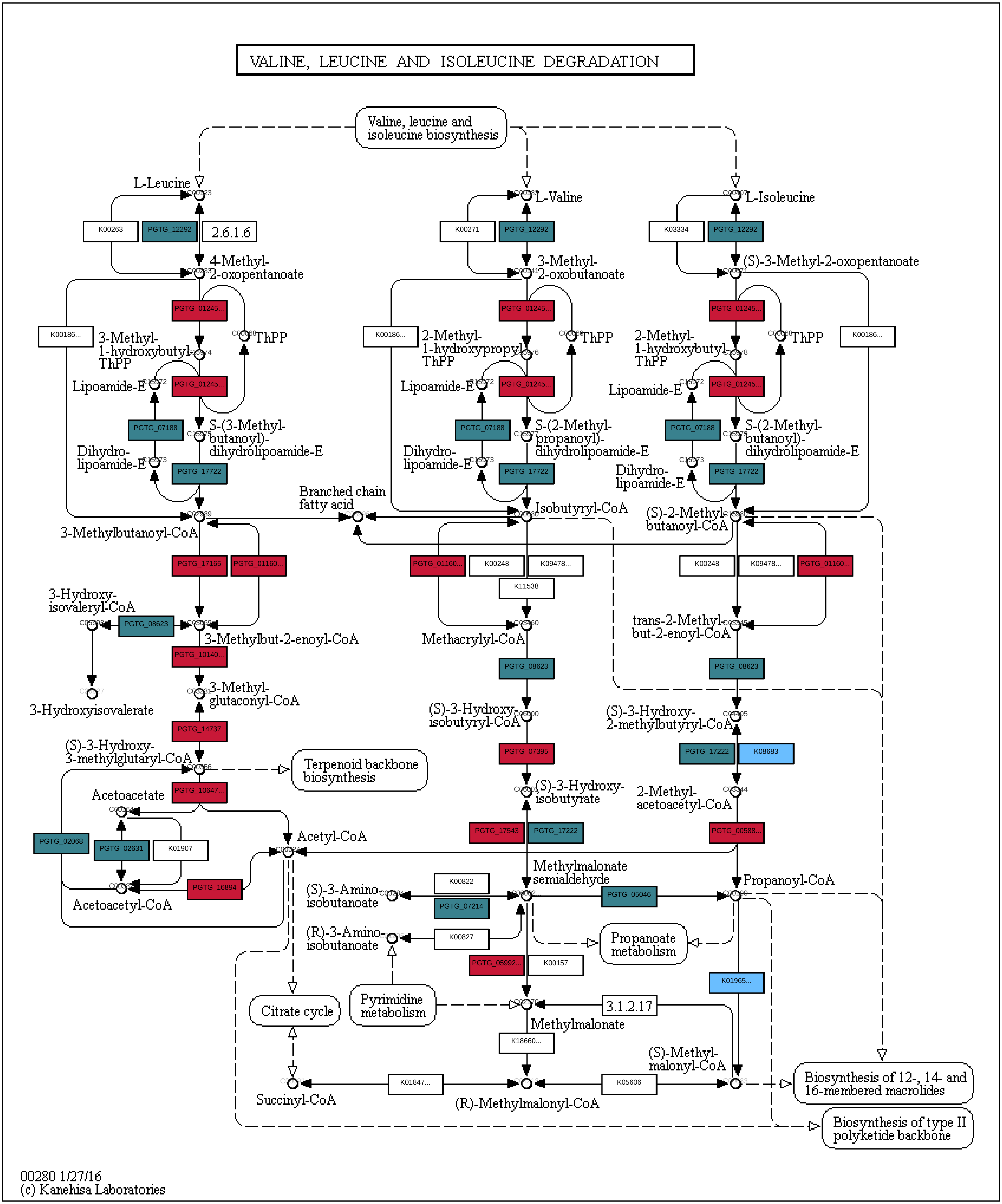

VALINE, LEUCINE AND ISOLEUCINE DEGRADATION
Valine, leucine and isoleucine biosynthesis
L-Leucine
L-Valine
L-Isoleucine
K00263
PGTG_12292
2.6.1.6
K00271
K03334
4-Methyl-2-oxopentanoate
3-Methyl-2-oxobutanoate
(S)-3-Methyl-2-oxopentanoate
PGTG_01245...
K00186...
3-Methyl-1-hydroxybutyl-ThPP
2-Methyl-1-hydroxypropyl-ThPP
2-Methyl-1-hydroxybutyl-ThPP
ThPP
Lipoamide-E
PGTG_07188
S-(3-Methylbutanoyl)-dihydrolipoamide-E
S-(2-Methylpropanoyl)-dihydrolipoamide-E
S-(2-Methylbutanoyl)-dihydrolipoamide-E
Dihydrolipoamide-E
PGTG_17722
Branched chain fatty acid
Isobutyryl-CoA
(S)-2-Methylbutanoyl-CoA
3-Methylbutanoyl-CoA
PGTG_17165
PGTG_01160...
K00248
K09478...
K11538
3-Hydroxy-isovaleryl-CoA
PGTG_08623
3-Methylbut-2-enoyl-CoA
Methacrylyl-CoA
trans-2-Methylbut-2-enoyl-CoA
PGTG_10140...
3-Hydroxyisovalerate
3-Methylglutaconyl-CoA
PGTG_14737
(S)-3-Hydroxyisobutyryl-CoA
(S)-3-Hydroxy-2-methylbutyryl-CoA
(S)-3-Hydroxy-3-methylglutaryl-CoA
Terpenoid backbone biosynthesis
PGTG_07395
PGTG_17222
K08683
PGTG_10647...
(S)-3-Hydroxyisobutyrate
2-Methylacetoacetyl-CoA
Acetoacetate
PGTG_17543
PGTG_00588...
PGTG_02068
PGTG_02631
K01907
Acetyl-CoA
Methylmalonate semialdehyde
Propanoyl-CoA
PGTG_16894
Acetoacetyl-CoA
K00822
(S)-3-Amino-isobutanoate
PGTG_07214
PGTG_05046
(R)-3-Amino-isobutanoate
K00827
Propanoate metabolism
PGTG_05992...
K00157
K01965...
Citrate cycle
Pyrimidine metabolism
3.1.2.17
Methylmalonate
K18660...
(S)-Methylmalonyl-CoA
K01847...
K05606
Succinyl-CoA
(R)-Methylmalonyl-CoA
Biosynthesis of 12-, 14- and 16-membered macrolides
Biosynthesis of type II polyketide backbone
00280 1/27/16
(c) Kanehisa Laboratories

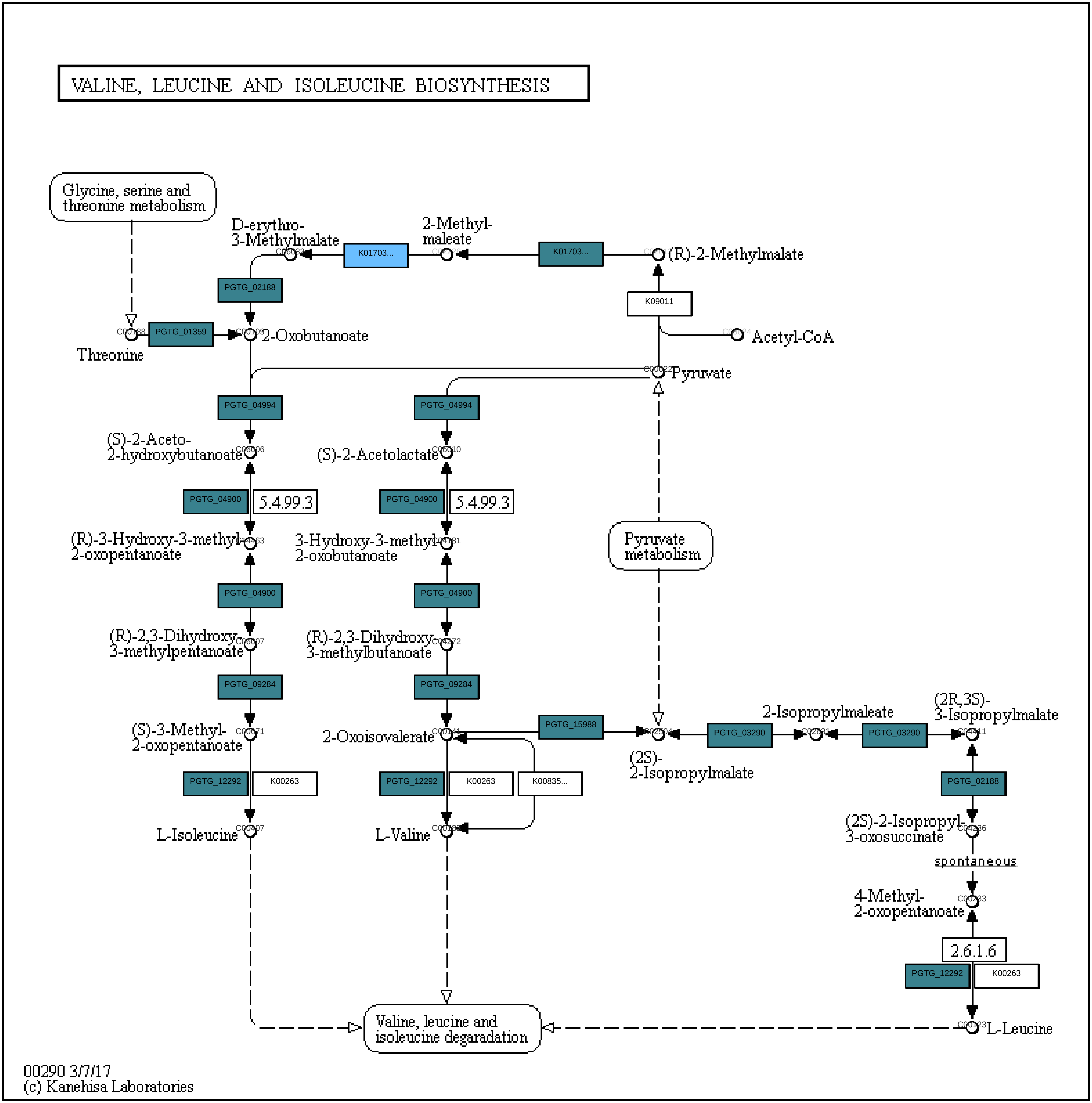
VALINE, LEUCINE AND ISOLEUCINE BIOSYNTHESIS
Glycine, serine and threonine metabolism
D-erythro-3-Methylmalate
K01703...
2-Methyl-maleate
K01703...
(R)-2-Methylmalate
PGTG_02188
K09011
Threonine
C00188
PGTG_01359
2-Oxobutanoate
Acetyl-CoA
Pyruvate
C00022
PGTG_04994
PGTG_04994
(S)-2-Aceto-2-hydroxybutanoate
C06006
(S)-2-Acetolactate
C06010
PGTG_04900
5.4.99.3
PGTG_04900
5.4.99.3
(R)-3-Hydroxy-3-methyl-2-oxopentanoate
3-Hydroxy-3-methyl-2-oxobutanoate
Pyruvate metabolism
PGTG_04900
PGTG_04900
(R)-2,3-Dihydroxy-3-methylpentanoate
C06007
(R)-2,3-Dihydroxy-3-methylbutanoate
C04272
PGTG_09284
PGTG_09284
(S)-3-Methyl-2-oxopentanoate
2-Oxoisovalerate
PGTG_15988
2-Isopropylmaleate
PGTG_03290
PGTG_03290
(2R,3S)-3-Isopropylmalate
(2S)-2-Isopropylmalate
PGTG_12292
K00263
PGTG_12292
K00263
K00835...
PGTG_02188
L-Isoleucine
L-Valine
(2S)-2-Isopropyl-3-oxosuccinate
spontaneous
4-Methyl-2-oxopentanoate
2.6.1.6
PGTG_12292
K00263
Valine, leucine and isoleucine degaradation
L-Leucine
00290 3/7/17
(c) Kanehisa Laboratories

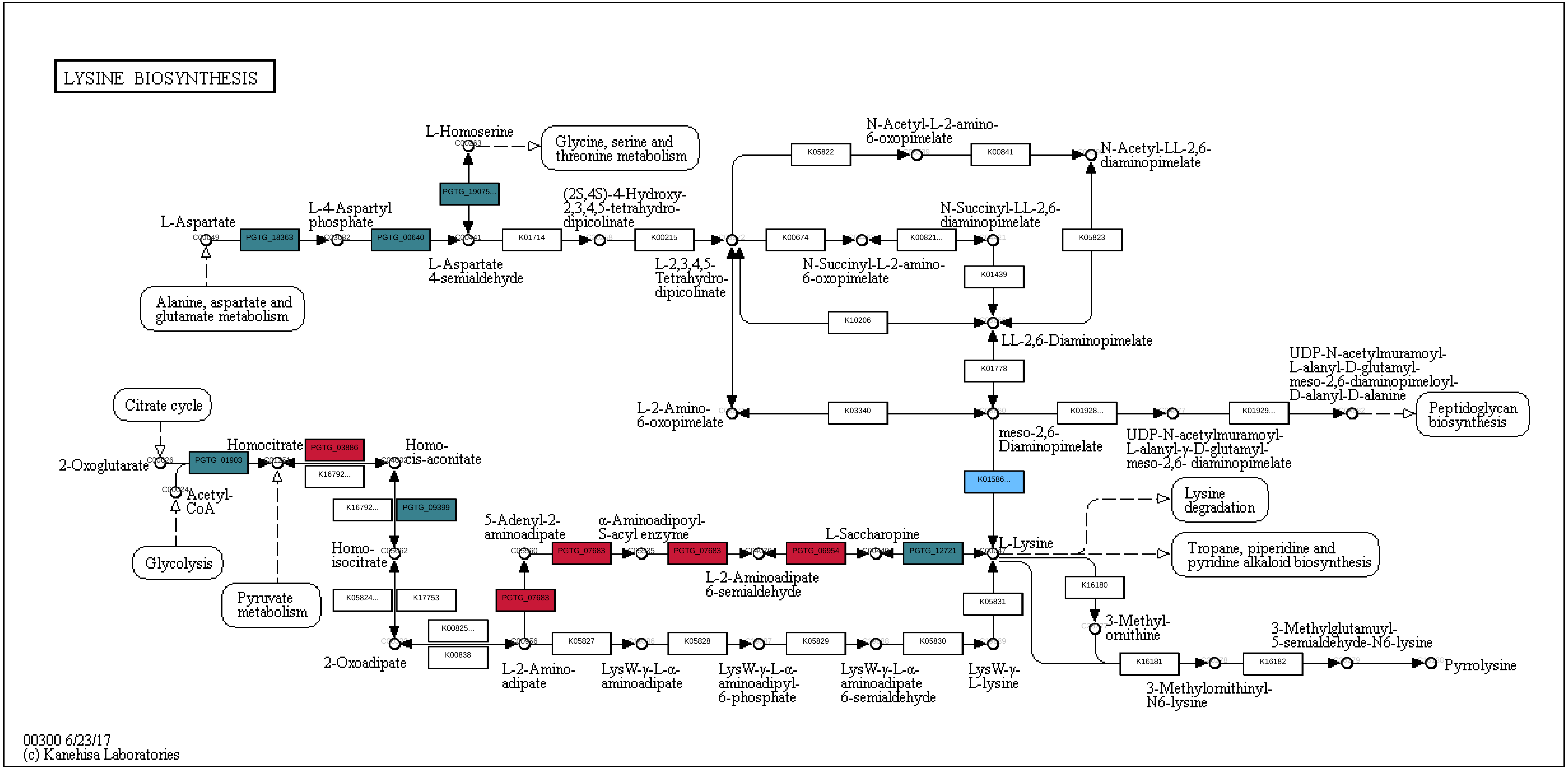

LYSINE BIOSYNTHESIS
L-Homoserine
Glycine, serine and threonine metabolism
PGTG_19075...
L-Aspartate
PGTG_18363
L-4-Aspartyl phosphate
PGTG_00640
K01714
(2S,4S)-4-Hydroxy-2,3,4,5-tetrahydro-dipicolinate
K00215
L-Aspartate 4-semialdehyde
L-2,3,4,5-Tetrahydro-dipicolinate
Alanine, aspartate and glutamate metabolism
N-Acetyl-L-2-amino-6-oxopimelate
K05822
K00841
N-Acetyl-LL-2,6-diaminopimelate
K00674
K00821...
N-Succinyl-LL-2,6-diaminopimelate
K05823
N-Succinyl-L-2-amino-6-oxopimelate
K01439
K10206
LL-2,6-Diaminopimelate
K01778
UDP-N-acetylmuramoyl-L-alanyl-D-glutamyl-meso-2,6-diaminopimeloyl-D-alanyl-D-alanine
L-2-Amino-6-oxopimelate
K03340
K01928...
K01929...
Peptidoglycan biosynthesis
meso-2,6-Diaminopimelate
UDP-N-acetylmuramoyl-L-alanyl-γ-D-glutamyl-meso-2,6- diaminopimelate
Citrate cycle
Homocitrate
PGTG_03886
Homo-cis-aconitate
2-Oxoglutarate
PGTG_01903
K16792...
Acetyl-CoA
K16792...
PGTG_09399
Glycolysis
Pyruvate metabolism
K01586...
Lysine degradation
5-Adenyl-2-aminoadipate
α-Aminoadipoyl-S-acyl enzyme
L-Saccharopine
Homo-isocitrate
PGTG_07683
PGTG_07683
PGTG_06954
PGTG_12721
L-Lysine
Tropane, piperidine and pyridine alkaloid biosynthesis
L-2-Aminoadipate 6-semialdehyde
K05824...
K17753
PGTG_07683
K16180
K05831
3-Methyl-ornithine
K00825...
K00838
K05827
K05828
K05829
K05830
3-Methylglutamuyl-5-semialdehyde-N6-lysine
K16181
K16182
Pyrrolysine
2-Oxoadipate
L-2-Amino-adipate
LysW-γ-L-α-aminoadipate
LysW-γ-L-α-aminoadipyl-6-phosphate
LysW-γ-L-α-aminoadipate 6-semialdehyde
LysW-γ-L-lysine
3-Methylornithinyl-N6-lysine
00300 6/23/17
(c) Kanehisa Laboratories

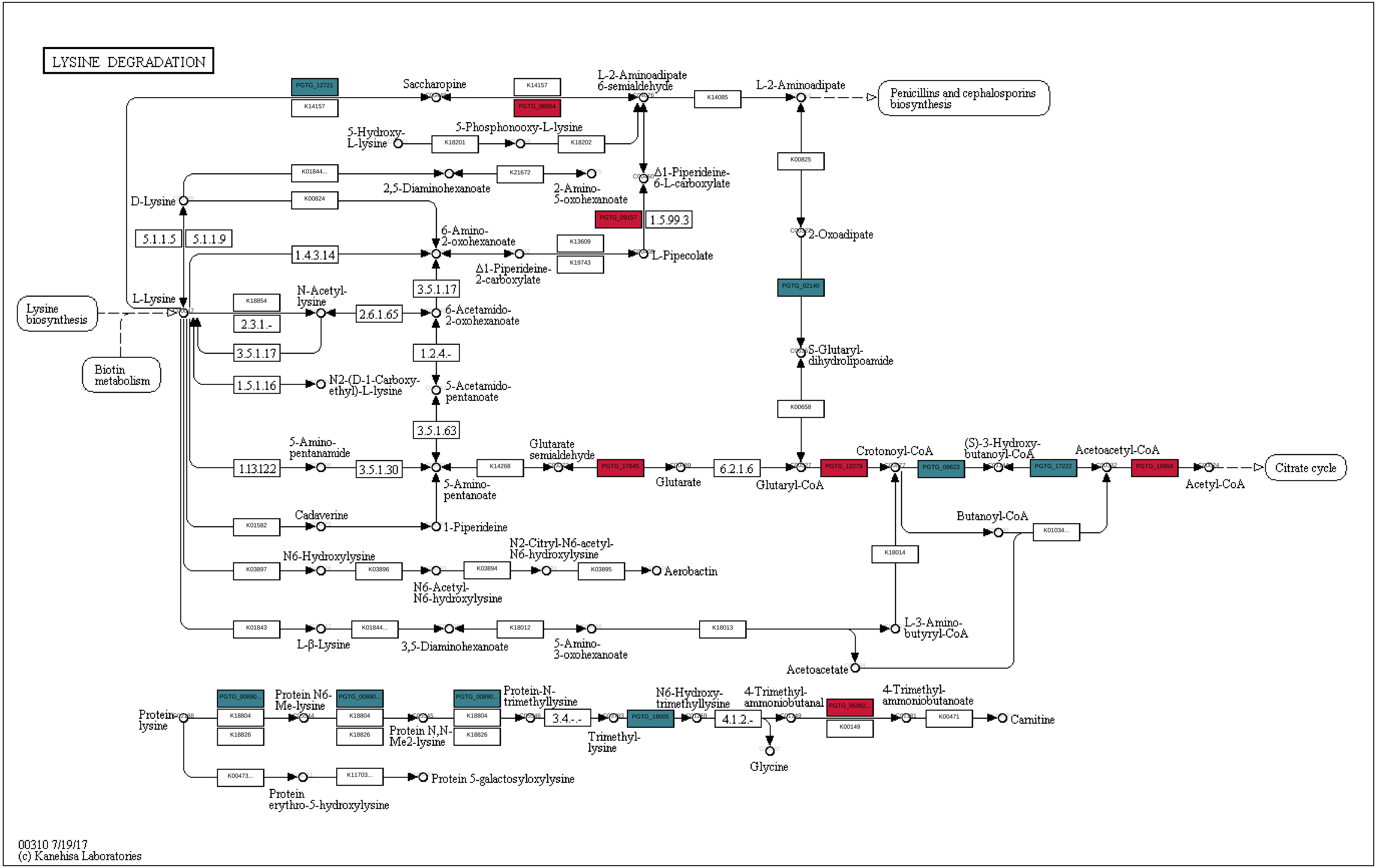
LYSINE DEGRADATION
PGTG_12721
K14157
Saccharopine
K14157
PGTG_06954
L-2-Aminoadipate 6-semialdehyde
K14085
L-2-Aminoadipate
Penicillins and cephalosporins biosynthesis
5-Hydroxy-L-lysine
K18201
5-Phosphonooxy-L-lysine
K18202
K01844...
2,5-Diaminohexanoate
K21672
2-Amino-5-oxohexanoate
Δ1-Piperideine-6-L-carboxylate
K00825
D-Lysine
K00824
PGTG_09157
1.5.99.3
5.1.1.5
5.1.1.9
1.4.3.14
6-Amino-2-oxohexanoate
K13609
K19743
L-Pipecolate
2-Oxoadipate
Δ1-Piperideine-2-carboxylate
PGTG_02140
Lysine biosynthesis
L-Lysine
K18854
2.3.1.-
N-Acetyl-lysine
2.6.1.65
3.5.1.17
6-Acetamido-2-oxohexanoate
Biotin metabolism
3.5.1.17
1.2.4.-
S-Glutaryl-dihydrolipoamide
1.5.1.16
N2-(D-1-Carboxy-ethyl)-L-lysine
5-Acetamido-pentanoate
K00658
3.5.1.63
5-Amino-pentanamide
Glutarate semialdehyde
Crotonoyl-CoA
(S)-3-Hydroxy-butanoyl-CoA
Acetoacetyl-CoA
1.13.12.2
3.5.1.30
K14268
PGTG_17645
6.2.1.6
PGTG_12279
PGTG_08623
PGTG_17222
PGTG_16894
Citrate cycle
Glutarate
Glutaryl-CoA
Acetyl-CoA
5-Amino-pentanoate
Cadaverine
K01582
1-Piperideine
Butanoyl-CoA
K01034...
K18014
N6-Hydroxylysine
N2-Citryl-N6-acetyl-N6-hydroxylysine
K03897
K03896
K03894
K03895
Aerobactin
N6-Acetyl-N6-hydroxylysine
K01843
K01844...
K18012
K18013
L-3-Amino-butyryl-CoA
L-β-Lysine
3,5-Diaminohexanoate
5-Amino-3-oxohexanoate
Acetoacetate
PGTG_00890...
Protein N6-Me-lysine
PGTG_00890...
PGTG_00890...
Protein-N-trimethyllysine
N6-Hydroxy-trimethyllysine
4-Trimethyl-ammoniobutanal
4-Trimethyl-ammoniobutanoate
Protein lysine
K18804
K18826
K18804
K18826
K18804
K18826
3.4.-.-
PGTG_19005
4.1.2.-
PGTG_05992...
K00149
K00471
Carnitine
Protein N,N-Me2-lysine
Trimethyl-lysine
Glycine
K00473...
K11703...
Protein 5-galactosyloxylysine
Protein erythro-5-hydroxylysine
00310 7/19/17
(c) Kanehisa Laboratories

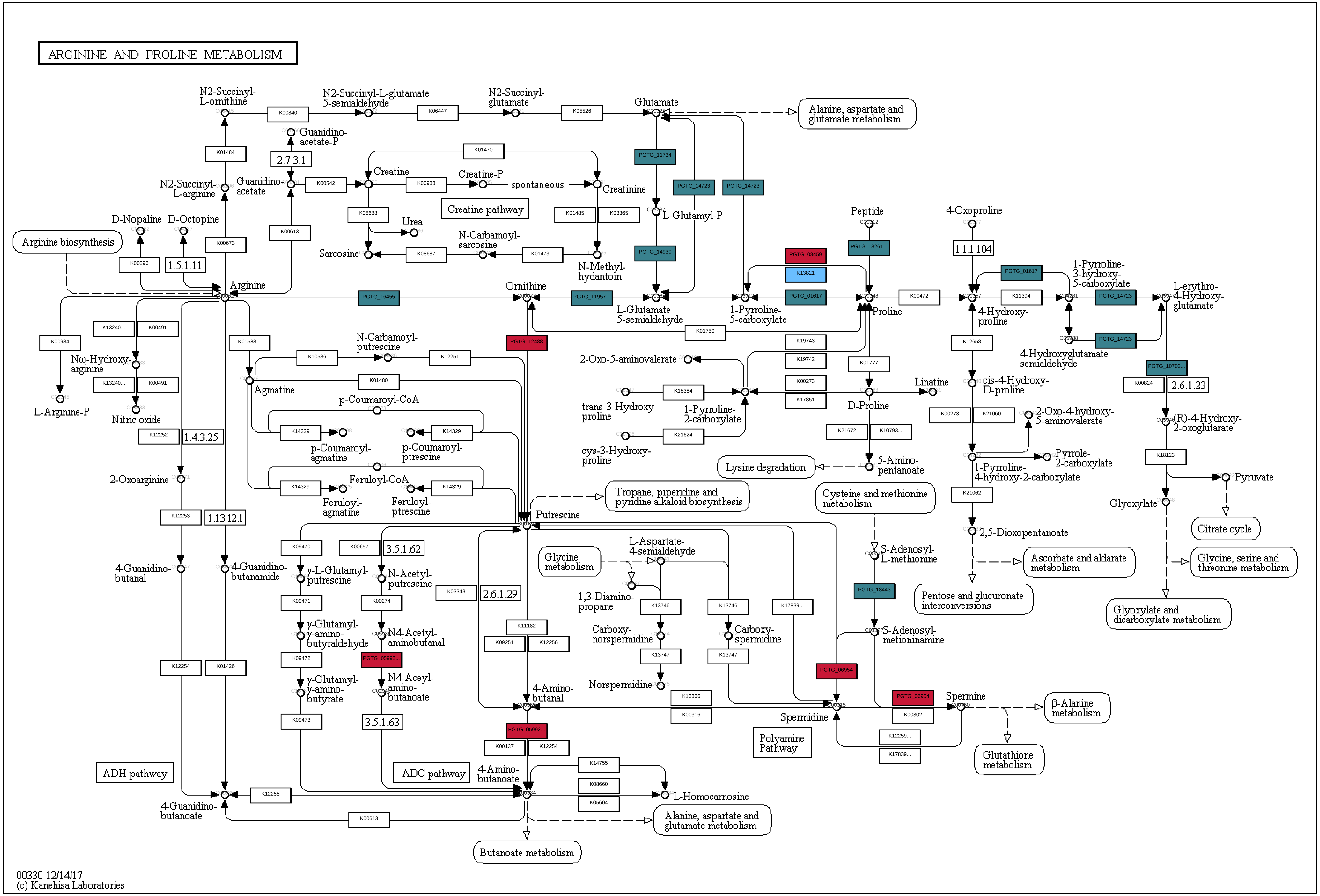
ARGININE AND PROLINE METABOLISM
N2-Succinyl-L-ornithine
N2-Succinyl-L-glutamate 5-semialdehyde
N2-Succinyl-glutamate
Glutamate
Alanine, aspartate and glutamate metabolism
K00840
K06447
K05526
Guanidino-acetate-P
2.7.3.1
K01484
K01470
Creatine
Creatine-P
spontaneous
Creatinine
N2-Succinyl-L-arginine
Guanidino-acetate
K00542
K00933
PGTG_11734
PGTG_14723
PGTG_14723
Creatine pathway
K08688
Urea
K01485
K03365
L-Glutamyl-P
D-Nopaline
D-Octopine
Arginine biosynthesis
K00673
K00613
Sarcosine
K08687
N-Carbamoyl-sarcosine
K01473...
N-Methyl-hydantoin
PGTG_14930
Peptide
4-Oxoproline
PGTG_13261...
1.1.1.104
PGTG_08459
K13821
PGTG_01617
K00296
1.5.1.11
Arginine
PGTG_16455
Ornithine
PGTG_11957...
L-Glutamate 5-semialdehyde
1-Pyrroline-5-carboxylate
PGTG_01617
K00472
Proline
K11394
4-Hydroxy-proline
1-Pyrroline-3-hydroxy-5-carboxylate
PGTG_14723
L-erythro-4-Hydroxy-glutamate
K01750
PGTG_14723
4-Hydroxyglutamate semialdehyde
K00934
K13240...
K00491
K01583...
PGTG_12488
K10536
N-Carbamoyl-putrescine
K12251
2-Oxo-5-aminovalerate
K19743
K19742
K00273
K17851
K01777
K12658
PGTG_10702...
Nω-Hydroxy-arginine
K13240...
K00491
K01480
Agmatine
trans-3-Hydroxy-proline
K18384
1-Pyrroline-2-carboxylate
D-Proline
Linatine
cis-4-Hydroxy-D-proline
K00824
2.6.1.23
L-Arginine-P
Nitric oxide
p-Coumaroyl-CoA
K14329
K14329
K00273
K21060...
2-Oxo-4-hydroxy-5-aminovalerate
(R)-4-Hydroxy-2-oxoglutarate
K12252
1.4.3.25
p-Coumaroyl-agmatine
p-Coumaroyl-ptrescine
cys-3-Hydroxy-proline
K21624
K21672
K10793...
Pyrrole-2-carboxylate
1-Pyrroline-4-hydroxy-2-carboxylate
K18123
2-Oxoarginine
Feruloyl-CoA
K14329
K14329
Lysine degradation
5-Amino-pentanoate
Pyruvate
Feruloyl-agmatine
Feruloyl-ptrescine
Tropane, piperidine and pyridine alkaloid biosynthesis
Cysteine and methionine metabolism
K21062
Glyoxylate
Citrate cycle
K12253
1.13.12.1
Putrescine
K09470
K00657
3.5.1.62
Glycine metabolism
L-Aspartate-4-semialdehyde
2,5-Dioxopentanoate
S-Adenosyl-L-methionine
4-Guanidino-butanal
4-Guanidino-butanamide
γ-L-Glutamyl-putrescine
N-Acetyl-putrescine
K03343
2.6.1.29
1,3-Diamino-propane
Ascorbate and aldarate metabolism
Glycine, serine and threonine metabolism
K09471
K00274
K13746
K13746
K17839...
PGTG_18443
Pentose and glucuronate interconversions
Glyoxylate and dicarboxylate metabolism
γ-Glutamyl-γ-amino-butyraldehyde
N4-Acetyl-aminobutanal
K11182
Carboxy-norspermidine
Carboxy-spermidine
S-Adenosyl-methioninamine
K12254
K01426
K09472
PGTG_05992...
K09251
K12256
K13747
K13747
PGTG_06954
γ-Glutamyl-γ-amino-butyrate
N4-Acetyl-amino-butanoate
4-Amino-butanal
Norspermidine
K13366
PGTG_06954
Spermine
β-Alanine metabolism
K09473
3.5.1.63
K00316
Spermidine
K00802
PGTG_05992...
K00137
K12254
Polyamine Pathway
K12259...
K17839...
Glutathione metabolism
ADH pathway
ADC pathway
4-Amino-butanoate
K14755
K08660
L-Homocarnosine
4-Guanidino-butanoate
K12255
K05604
K00613
Alanine, aspartate and glutamate metabolism
Butanoate metabolism
00330 12/14/17
(c) Kanehisa Laboratories

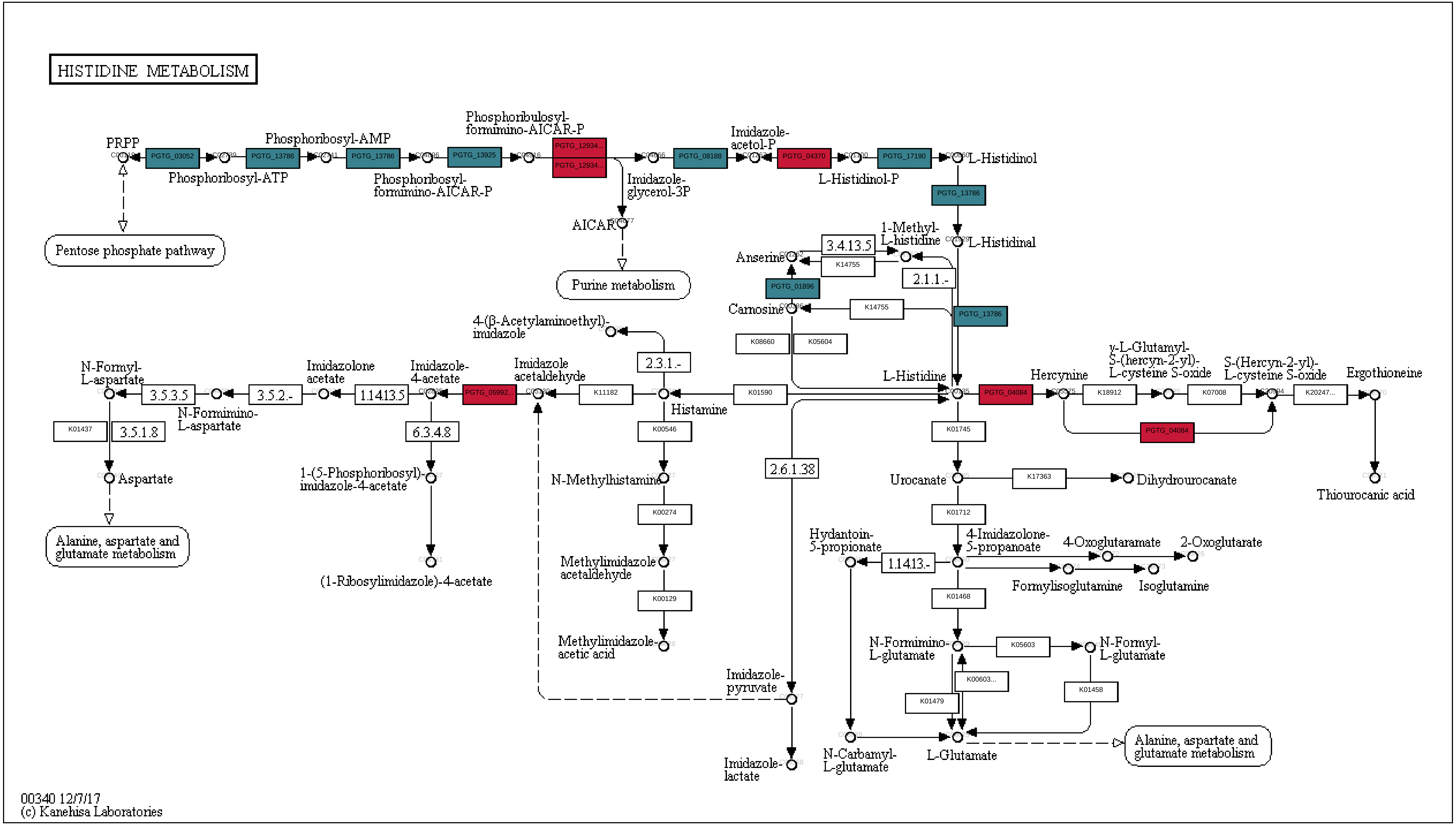

HISTIDINE METABOLISM
PRPP
Phosphoribosyl-ATP
Phosphoribosyl-AMP
Phosphoribosyl-formimino-AICAR-P
Phosphoribulosyl-formimino-AICAR-P
Imidazole-glycerol-3P
Imidazole-acetol-P
L-Histidinol-P
L-Histidinol
L-Histidinal
AICAR
Pentose phosphate pathway
Purine metabolism
PGTG_03052
PGTG_13786
PGTG_13925
PGTG_12934...
PGTG_08188
PGTG_04370
PGTG_17190
PGTG_01896
PGTG_05992...
PGTG_04084
1-Methyl-L-histidine
Anserine
Carnosine
3.4.13.5
2.1.1.-
K14755
K08660
K05604
4-(β-Acetylaminoethyl)-imidazole
2.3.1.-
L-Histidine
Hercynine
γ-L-Glutamyl-S-(hercyn-2-yl)-L-cysteine S-oxide
S-(Hercyn-2-yl)-L-cysteine S-oxide
Ergothioneine
K18912
K07008
K20247...
Thiourocanic acid
N-Formyl-L-aspartate
3.5.3.5
N-Formimino-L-aspartate
3.5.2.-
Imidazolone acetate
1.14.13.5
Imidazole-4-acetate
Imidazole acetaldehyde
K11182
Histamine
K01590
K01437
3.5.1.8
6.3.4.8
Aspartate
Alanine, aspartate and glutamate metabolism
1-(5-Phosphoribosyl)-imidazole-4-acetate
(1-Ribosylimidazole)-4-acetate
K00546
N-Methylhistamine
K00274
Methylimidazole acetaldehyde
K00129
Methylimidazole-acetic acid
2.6.1.38
Imidazole-pyruvate
Imidazole-lactate
K01745
Urocanate
K17363
Dihydrourocanate
K01712
4-Imidazolone-5-propanoate
Hydantoin-5-propionate
1.14.13.-
4-Oxoglutaramate
2-Oxoglutarate
Formylisoglutamine
Isoglutamine
K01468
N-Formimino-L-glutamate
K05603
N-Formyl-L-glutamate
K00603...
K01479
K01458
N-Carbamyl-L-glutamate
L-Glutamate
Alanine, aspartate and glutamate metabolism
00340 12/7/17
(c) Kanehisa Laboratories

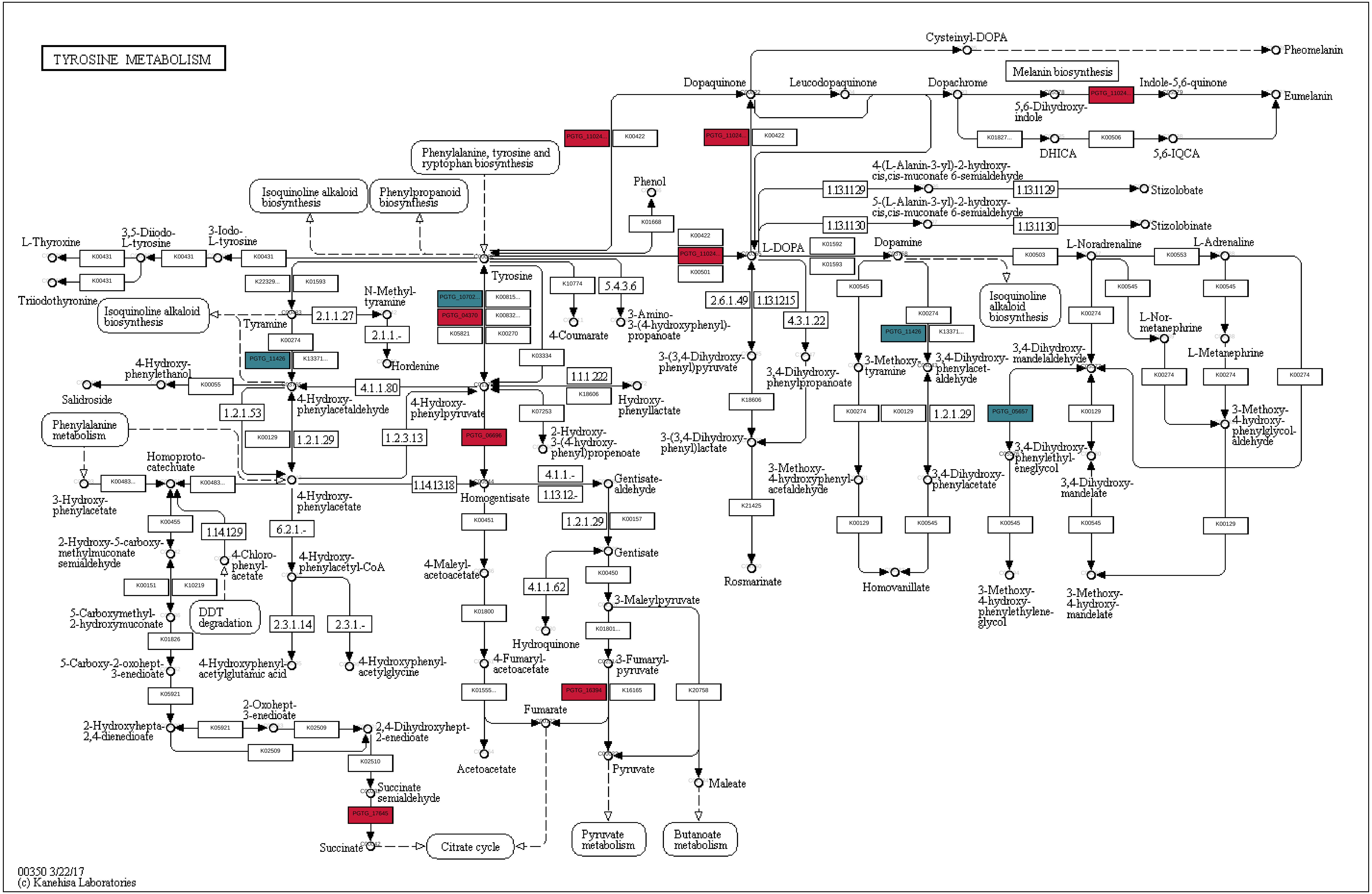
TYROSINE METABOLISM
Cysteinyl-DOPA
Pheomelanin
Melanin biosynthesis
Dopaquinone
Leucodopaquinone
Dopachrome
Indole-5,6-quinone
Eumelanin
5,6-Dihydroxy-indole
PGTG_11024...
K00422
K01827...
K00506
DHICA
5,6-IQCA
Phenylalanine, tyrosine and ryptophan biosynthesis
Isoquinoline alkaloid biosynthesis
Phenylpropanoid biosynthesis
Phenol
K01668
4-(L-Alanin-3-yl)-2-hydroxy-cis,cis-muconate 6-semialdehyde
1.13.11.29
Stizolobate
5-(L-Alanin-3-yl)-2-hydroxy-cis,cis-muconate 6-semialdehyde
1.13.11.30
Stizolobinate
L-Thyroxine
3,5-Diiodo-L-tyrosine
3-Iodo-L-tyrosine
K00431
Triiodothyronine
L-DOPA
K01592
K01593
K00501
Dopamine
K00503
L-Noradrenaline
K00553
L-Adrenaline
Tyrosine
K22329...
K10774
5.4.3.6
N-Methyl-tyramine
PGTG_10702...
K00815...
PGTG_04370
K00832...
K05821
K00270
2.1.1.27
2.1.1.-
Tyramine
K00274
PGTG_11426
K13371...
Hordenine
4-Coumarate
3-Amino-3-(4-hydroxyphenyl)-propanoate
2.6.1.49
1.13.12.15
4.3.1.22
K00545
Isoquinoline alkaloid biosynthesis
L-Nor-metanephrine
L-Metanephrine
4-Hydroxy-phenylethanol
K00055
Salidroside
K03334
4.1.1.80
1.1.1.222
K18606
Hydroxy-phenyllactate
3-(3,4-Dihydroxy-phenyl)pyruvate
3,4-Dihydroxy-phenylpropanoate
3-Methoxy-tyramine
3,4-Dihydroxy-phenylacet-aldehyde
3,4-Dihydroxy-mandelaldehyde
4-Hydroxy-phenylacetaldehyde
4-Hydroxy-phenylpyruvate
1.2.1.53
K07253
K00129
1.2.1.29
PGTG_05657
3-Methoxy-4-hydroxy-phenylglycol-aldehyde
Phenylalanine metabolism
1.2.3.13
PGTG_06696
2-Hydroxy-3-(4-hydroxy-phenyl)propenoate
3-(3,4-Dihydroxy-phenyl)lactate
3,4-Dihydroxy-phenylethyl-eneglycol
Homoproto-catechuate
K00483...
1.14.13.18
4.1.1.-
1.13.12.-
Gentisate-aldehyde
3-Methoxy-4-hydroxyphenyl-acetaldehyde
3,4-Dihydroxy-phenylacetate
3,4-Dihydroxy-mandelate
3-Hydroxy-phenylacetate
4-Hydroxy-phenylacetate
Homogentisate
K21425
K00455
1.14.12.9
6.2.1.-
K00451
1.2.1.29
K00157
2-Hydroxy-5-carboxy-methylmuconate semialdehyde
4-Chloro-phenyl-acetate
4-Hydroxy-phenylacetyl-CoA
4-Maleyl-acetoacetate
Gentisate
Rosmarinate
Homovanillate
K00151
K10219
4.1.1.62
K00450
3-Methoxy-4-hydroxy-phenylethylene-glycol
3-Methoxy-4-hydroxy-mandelate
5-Carboxymethyl-2-hydroxymuconate
DDT degradation
2.3.1.14
2.3.1.-
K01800
3-Maleylpyruvate
K01826
Hydroquinone
K01801...
5-Carboxy-2-oxohept-3-enedioate
4-Hydroxyphenyl-acetylglutamic acid
4-Hydroxyphenyl-acetylglycine
4-Fumaryl-acetoacetate
3-Fumaryl-pyruvate
K05921
K01555...
PGTG_16394
K16165
K20758
Fumarate
2-Oxohept-3-enedioate
2-Hydroxyhepta-2,4-dienedioate
K02509
2,4-Dihydroxyhept-2-enedioate
K02510
Acetoacetate
Pyruvate
Maleate
Succinate semialdehyde
PGTG_17645
Pyruvate metabolism
Butanoate metabolism
Succinate
Citrate cycle
00350 3/22/17
(c) Kanehisa Laboratories

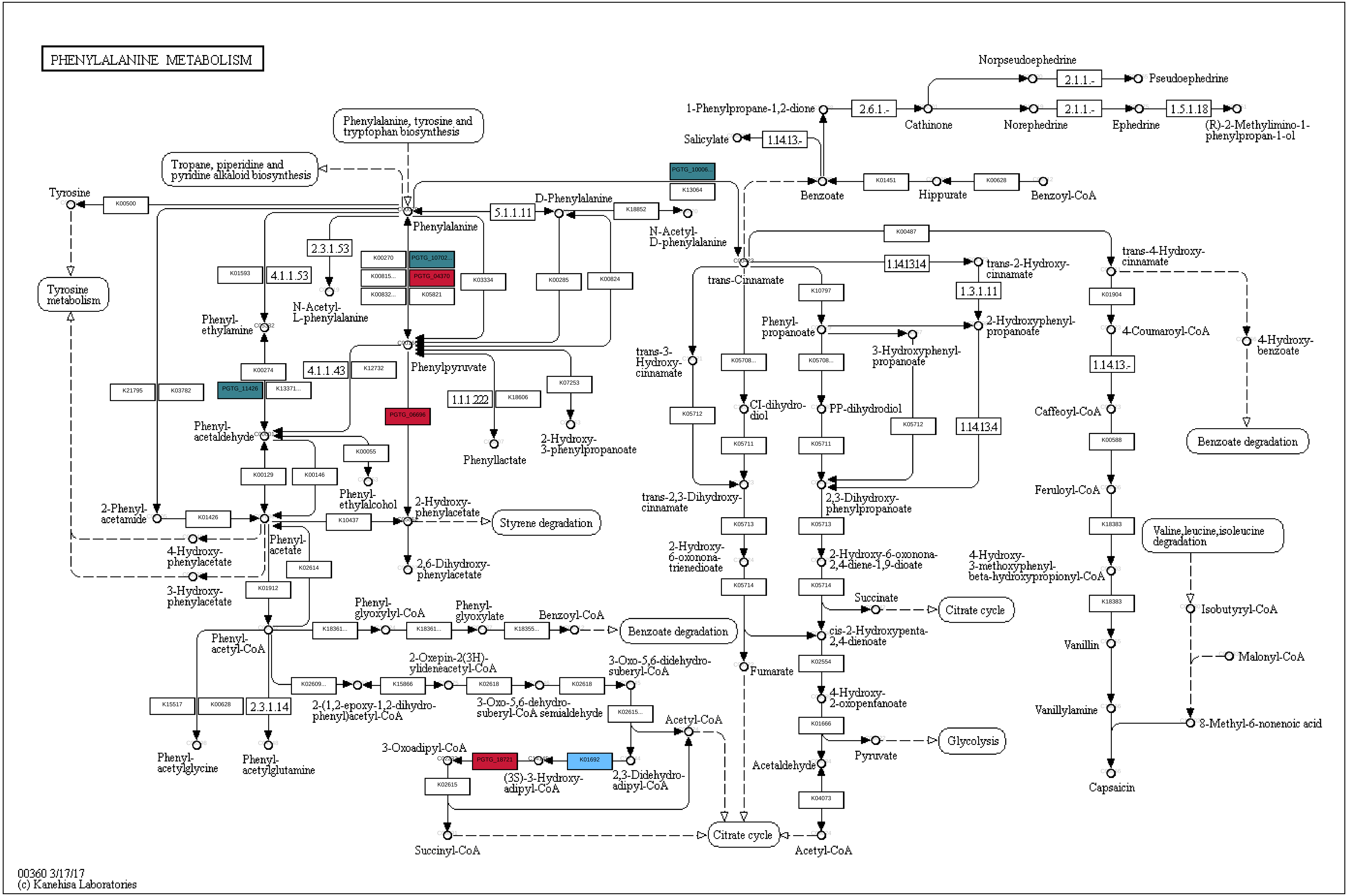
PHENYLALANINE METABOLISM
Phenylalanine, tyrosine and tryptophan biosynthesis
Tropane, piperidine and pyridine alkaloid biosynthesis
Tyrosine
K00500
Tyrosine metabolism
Phenylalanine
2.3.1.53
N-Acetyl-L-phenylalanine
K01593
4.1.1.53
K00270
PGTG_10702...
K00815...
PGTG_04370
K00832...
K05821
K03334
5.1.1.11
D-Phenylalanine
K18852
N-Acetyl-D-phenylalanine
K00285
K00824
PGTG_10006...
K13064
Phenyl-ethylamine
Phenylpyruvate
K00274
4.1.1.43
K12732
PGTG_11426
K13371...
K21795
K03782
PGTG_06696
1.1.1.222
K18606
K07253
Phenyllactate
2-Hydroxy-3-phenylpropanoate
Phenyl-acetaldehyde
K00055
Phenyl-ethylalcohol
K00129
K00146
2-Phenyl-acetamide
K01426
K10437
2-Hydroxy-phenylacetate
Styrene degradation
4-Hydroxy-phenylacetate
Phenyl-acetate
K02614
2,6-Dihydroxy-phenylacetate
3-Hydroxy-phenylacetate
K01912
Phenyl-acetyl-CoA
K18361...
Phenyl-glyoxylyl-CoA
K18361...
Phenyl-glyoxylate
K18355...
Benzoyl-CoA
Benzoate degradation
2-Oxepin-2(3H)-ylideneacetyl-CoA
K02609...
K15866
K02618
K02618
3-Oxo-5,6-didehydro-suberyl-CoA
2-(1,2-epoxy-1,2-dihydro-phenyl)acetyl-CoA
3-Oxo-5,6-dehydro-suberyl-CoA semialdehyde
K02615...
K15517
K00628
2.3.1.14
Phenyl-acetylglycine
Phenyl-acetylglutamine
3-Oxoadipyl-CoA
PGTG_18721
K01692
(3S)-3-Hydroxy-adipyl-CoA
2,3-Didehydro-adipyl-CoA
Acetyl-CoA
K02615
Succinyl-CoA
Citrate cycle
1-Phenylpropane-1,2-dione
2.6.1.-
Cathinone
Norpseudoephedrine
2.1.1.-
Pseudoephedrine
Norephedrine
2.1.1.-
Ephedrine
1.5.1.18
(R)-2-Methylimino-1-phenylpropan-1-ol
Salicylate
1.14.13.-
Benzoate
K01451
Hippurate
K00628
Benzoyl-CoA
K00487
trans-Cinnamate
1.14.13.14
trans-2-Hydroxy-cinnamate
trans-4-Hydroxy-cinnamate
K10797
1.3.1.11
Phenyl-propanoate
2-Hydroxyphenyl-propanoate
3-Hydroxyphenyl-propanoate
K01904
4-Coumaroyl-CoA
4-Hydroxy-benzoate
trans-3-Hydroxy-cinnamate
K05708...
K05708...
1.14.13.-
CI-dihydro-diol
PP-dihydrodiol
K05712
K05712
1.14.13.4
Caffeoyl-CoA
Benzoate degradation
K05711
K05711
K00588
trans-2,3-Dihydroxy-cinnamate
2,3-Dihydroxy-phenylpropanoate
Feruloyl-CoA
K05713
K05713
K18383
Valine, leucine, isoleucine degradation
2-Hydroxy-6-oxonona-trienedioate
2-Hydroxy-6-oxonona-2,4-diene-1,9-dioate
4-Hydroxy-3-methoxyphenyl-beta-hydroxypropionyl-CoA
K05714
K05714
Succinate
Citrate cycle
K18383
Isobutyryl-CoA
cis-2-Hydroxypenta-2,4-dienoate
Vanillin
Malonyl-CoA
Fumarate
K02554
4-Hydroxy-2-oxopentanoate
Vanillylamine
K01666
8-Methyl-6-nonenoic acid
Pyruvate
Glycolysis
Acetaldehyde
Capsaicin
K04073
Acetyl-CoA
00360 3/17/17
(c) Kanehisa Laboratories

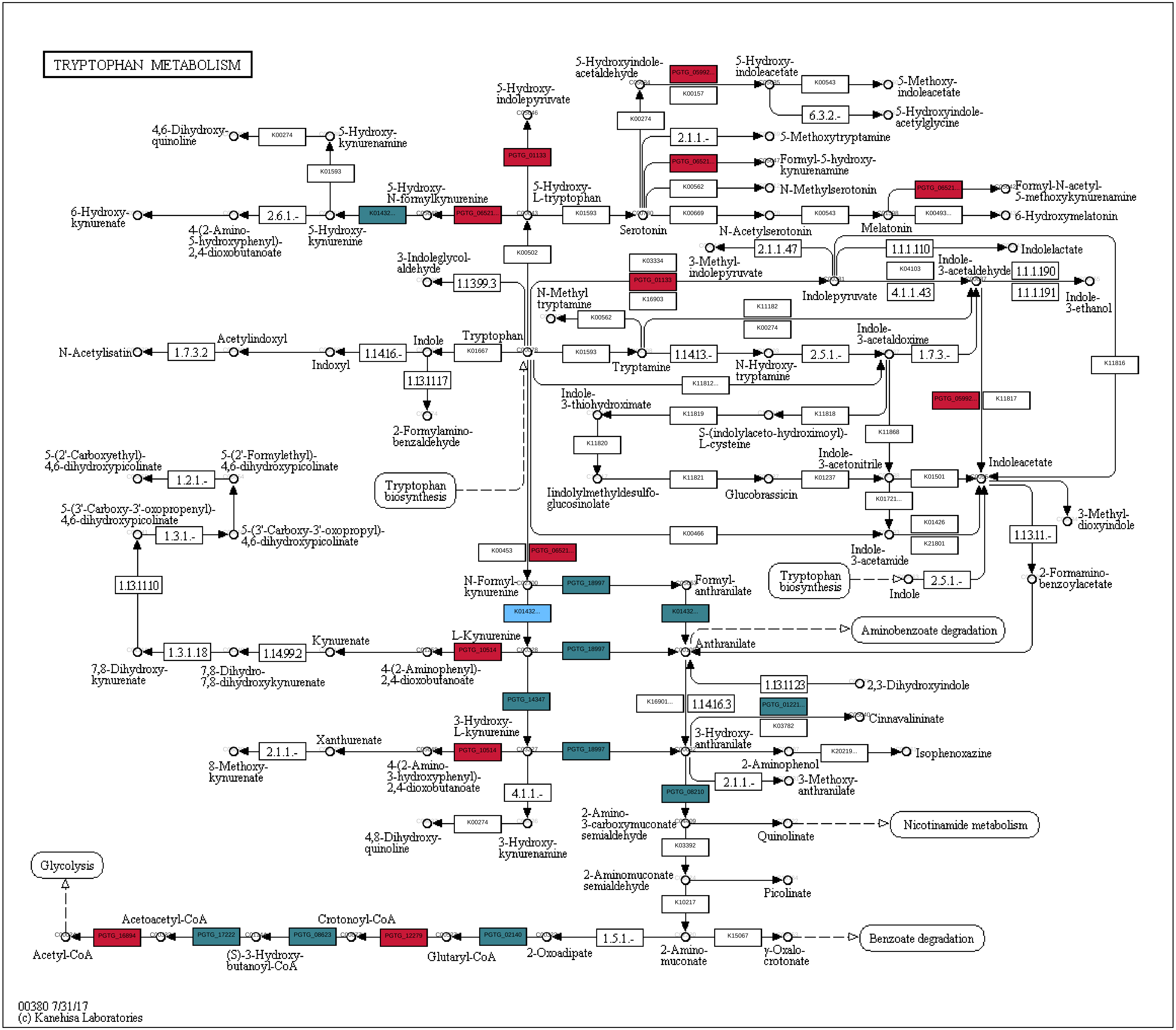

TRYPTOPHAN METABOLISM
5-Hydroxyindole-acetaldehyde
PGTG_05992...
K00157
5-Hydroxy-indoleacetate
K00543
5-Methoxy-indoleacetate
6.3.2.-
5-Hydroxyindole-acetylglycine
K00274
2.1.1.-
5-Methoxytryptamine
PGTG_06521...
Formyl-5-hydroxy-kynurenamine
K00562
N-Methylserotonin
PGTG_06521...
Formyl-N-acetyl-5-methoxykynurenamine
K00669
K00543
K00493...
6-Hydroxymelatonin
Serotonin
N-Acetylserotonin
Melatonin
5-Hydroxy-indolepyruvate
PGTG_01133
5-Hydroxy-L-tryptophan
K01593
4,6-Dihydroxy-quinoline
K00274
5-Hydroxy-kynurenamine
K01593
5-Hydroxy-N-formylkynurenine
6-Hydroxy-kynurenate
2.6.1.-
K01432...
PGTG_06521...
4-(2-Amino-5-hydroxyphenyl)-2,4-dioxobutanoate
5-Hydroxy-kynurenine
K00502
3-Indoleglycol-aldehyde
1.13.99.3
K03334
PGTG_01133
K16903
3-Methyl-indolepyruvate
2.1.1.47
1.1.1.110
Indolelactate
K04103
Indole-3-acetaldehyde
1.1.1.190
4.1.1.43
1.1.1.191
Indole-3-ethanol
Indolepyruvate
N-Methyl tryptamine
K00562
K11182
K00274
Acetylindoxyl
N-Acetylisatin
1.7.3.2
Indoxyl
1.14.16.-
Indole
K01667
Tryptophan
K01593
Tryptamine
1.14.13.-
N-Hydroxy-tryptamine
2.5.1.-
Indole-3-acetaldoxime
1.7.3.-
K11816
1.13.11.17
2-Formylamino-benzaldehyde
K11812...
Indole-3-thiohydroximate
K11819
K11818
S-(indolylaceto-hydroximoyl)-L-cysteine
PGTG_05992...
K11817
K11868
K11820
Indole-3-acetonitrile
Iindolylmethyldesulfo-glucosinolate
K11821
Glucobrassicin
K01237
K01501
Indoleacetate
K01721...
3-Methyl-dioxyindole
K00466
K01426
K21801
Indole-3-acetamide
1.13.11.-
5-(2'-Carboxyethyl)-4,6-dihydroxypicolinate
1.2.1.-
5-(2'-Formylethyl)-4,6-dihydroxypicolinate
Tryptophan biosynthesis
5-(3'-Carboxy-3'-oxopropenyl)-4,6-dihydroxypicolinate
1.3.1.-
5-(3'-Carboxy-3'-oxopropyl)-4,6-dihydroxypicolinate
K00453
PGTG_06521...
N-Formyl-kynurenine
PGTG_18997
Formyl-anthranilate
Tryptophan biosynthesis
2.5.1.-
Indole
2-Formamino-benzoylacetate
1.13.11.10
K01432...
K01432...
Aminobenzoate degradation
7,8-Dihydroxy-kynurenate
1.3.1.18
7,8-Dihydro-7,8-dihydroxykynurenate
1.14.99.2
Kynurenate
PGTG_10514
L-Kynurenine
4-(2-Aminophenyl)-2,4-dioxobutanoate
PGTG_18997
Anthranilate
1.13.11.23
2,3-Dihydroxyindole
PGTG_14347
K16901...
1.14.16.3
PGTG_01221...
K03782
Cinnavalininate
3-Hydroxy-L-kynurenine
3-Hydroxy-anthranilate
8-Methoxy-kynurenate
2.1.1.-
Xanthurenate
PGTG_10514
4-(2-Amino-3-hydroxyphenyl)-2,4-dioxobutanoate
PGTG_18997
2-Aminophenol
K20219...
Isophenoxazine
2.1.1.-
3-Methoxy-anthranilate
4.1.1.-
PGTG_08210
K00274
4,8-Dihydroxy-quinoline
3-Hydroxy-kynurenamine
2-Amino-3-carboxymuconate semialdehyde
Quinolinate
Nicotinamide metabolism
K03392
Glycolysis
2-Aminomuconate semialdehyde
Picolinate
K10217
Acetoacetyl-CoA
Crotonoyl-CoA
PGTG_16894
PGTG_17222
PGTG_08623
PGTG_12279
PGTG_02140
1.5.1.-
K15067
Benzoate degradation
Acetyl-CoA
(S)-3-Hydroxy-butanoyl-CoA
Glutaryl-CoA
2-Oxoadipate
2-Amino-muconate
γ-Oxalo-crotonate
00380 7/31/17
(c) Kanehisa Laboratories

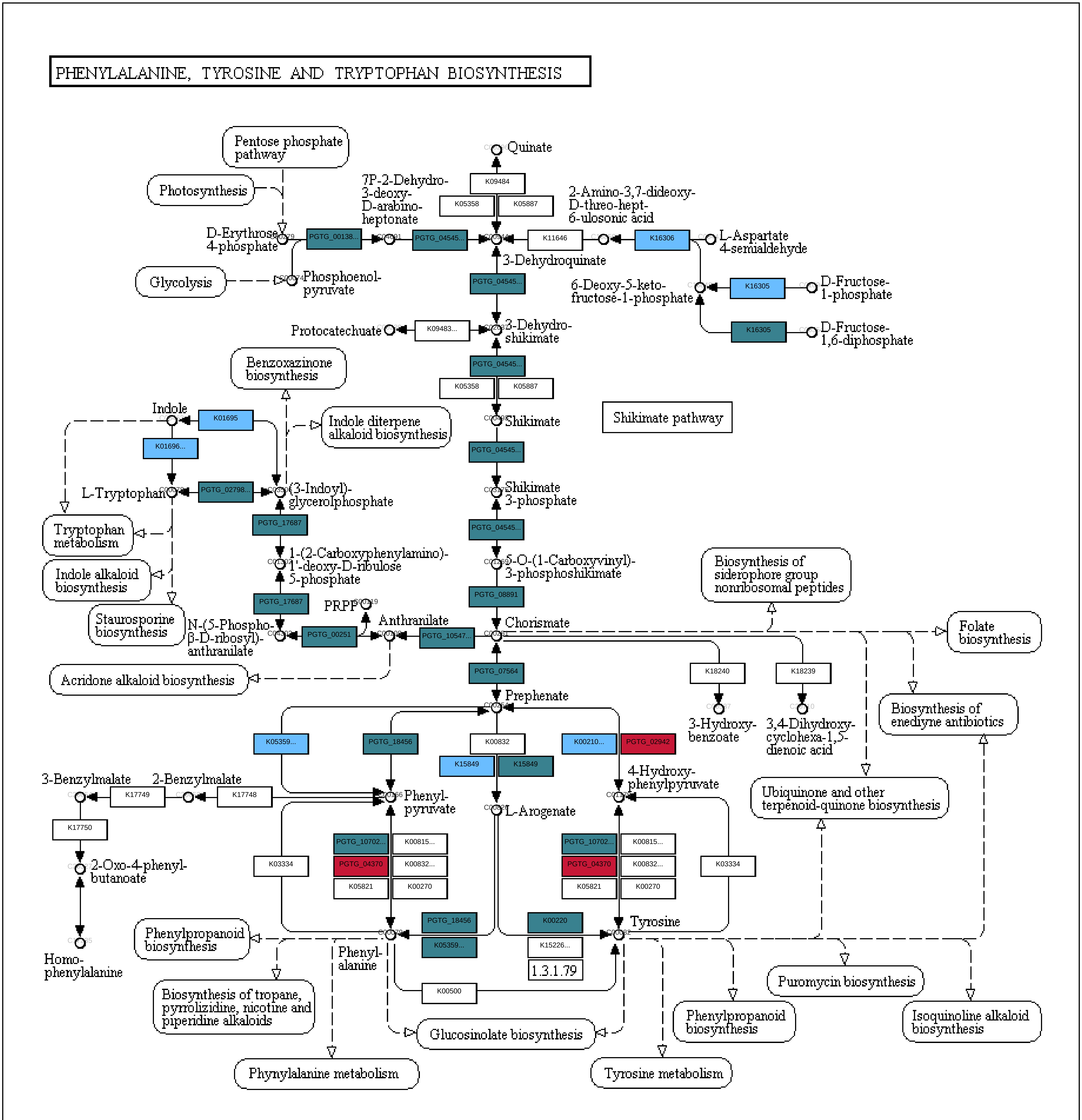

00400 2/14/17
(c) Kanehisa Laboratories

# 6. Metabolism of other amino acids

| MAP | PATHWAY |
|---|---|
| **410** | beta-Alanine metabolism |
| **430** | Taurine and hypotaurine metabolism |
| **440** | Phosphonate and phosphinate metabolism |
| **450** | Selenocompound metabolism |
| **460** | Cyanoamino acid metabolism |
| **480** | Glutathione metabolism |

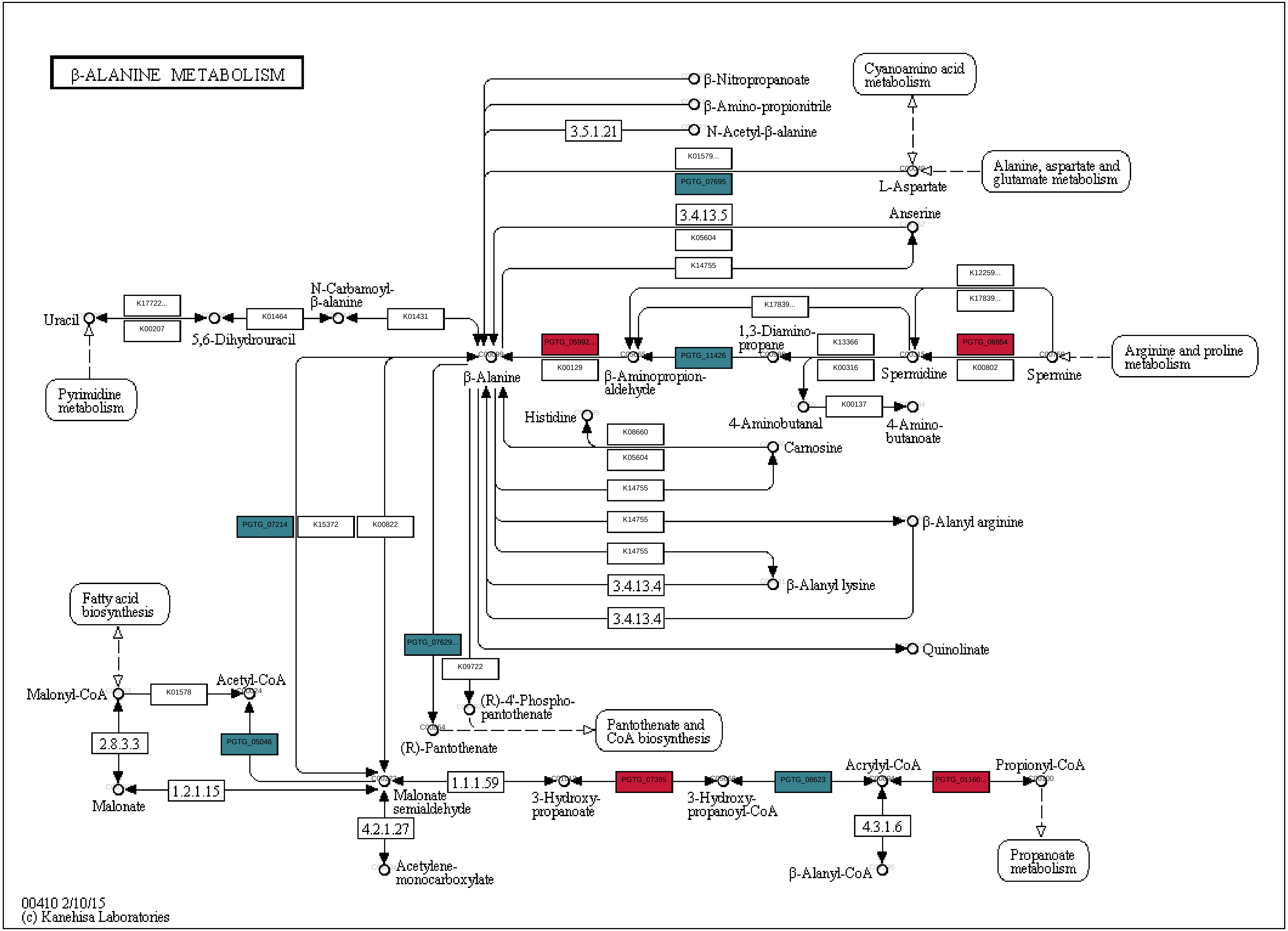

β-ALANINE METABOLISM
β-Nitropropanoate
β-Amino-propionitrile
3.5.1.21
N-Acetyl-β-alanine
Cyanoamino acid metabolism
K01579...
PGTG_07695
L-Aspartate
Alanine, aspartate and glutamate metabolism
3.4.13.5
Anserine
K05604
K14755
K12259...
K17839...
K17839...
Uracil
K17722...
K00207
5,6-Dihydrouracil
K01464
N-Carbamoyl-β-alanine
K01431
PGTG_05992...
K00129
β-Alanine
β-Aminopropion-aldehyde
PGTG_11426
1,3-Diamino-propane
K13366
K00316
Spermidine
PGTG_06954
K00802
Spermine
Arginine and proline metabolism
Pyrimidine metabolism
Histidine
K08660
K05604
4-Aminobutanal
K00137
4-Amino-butanoate
Carnosine
K14755
K14755
β-Alanyl arginine
K14755
β-Alanyl lysine
3.4.13.4
3.4.13.4
Quinolinate
PGTG_07214
K15372
K00822
Fatty acid biosynthesis
PGTG_07629...
K09722
(R)-4'-Phospho-pantothenate
Pantothenate and CoA biosynthesis
(R)-Pantothenate
Malonyl-CoA
K01578
Acetyl-CoA
2.8.3.3
PGTG_05046
Malonate
1.2.1.15
Malonate semialdehyde
1.1.1.59
3-Hydroxy-propanoate
PGTG_07395
3-Hydroxy-propanoyl-CoA
PGTG_08623
Acrylyl-CoA
PGTG_01160...
Propionyl-CoA
4.2.1.27
Acetylene-monocarboxylate
4.3.1.6
β-Alanyl-CoA
Propanoate metabolism
00410 2/10/15
(c) Kanehisa Laboratories

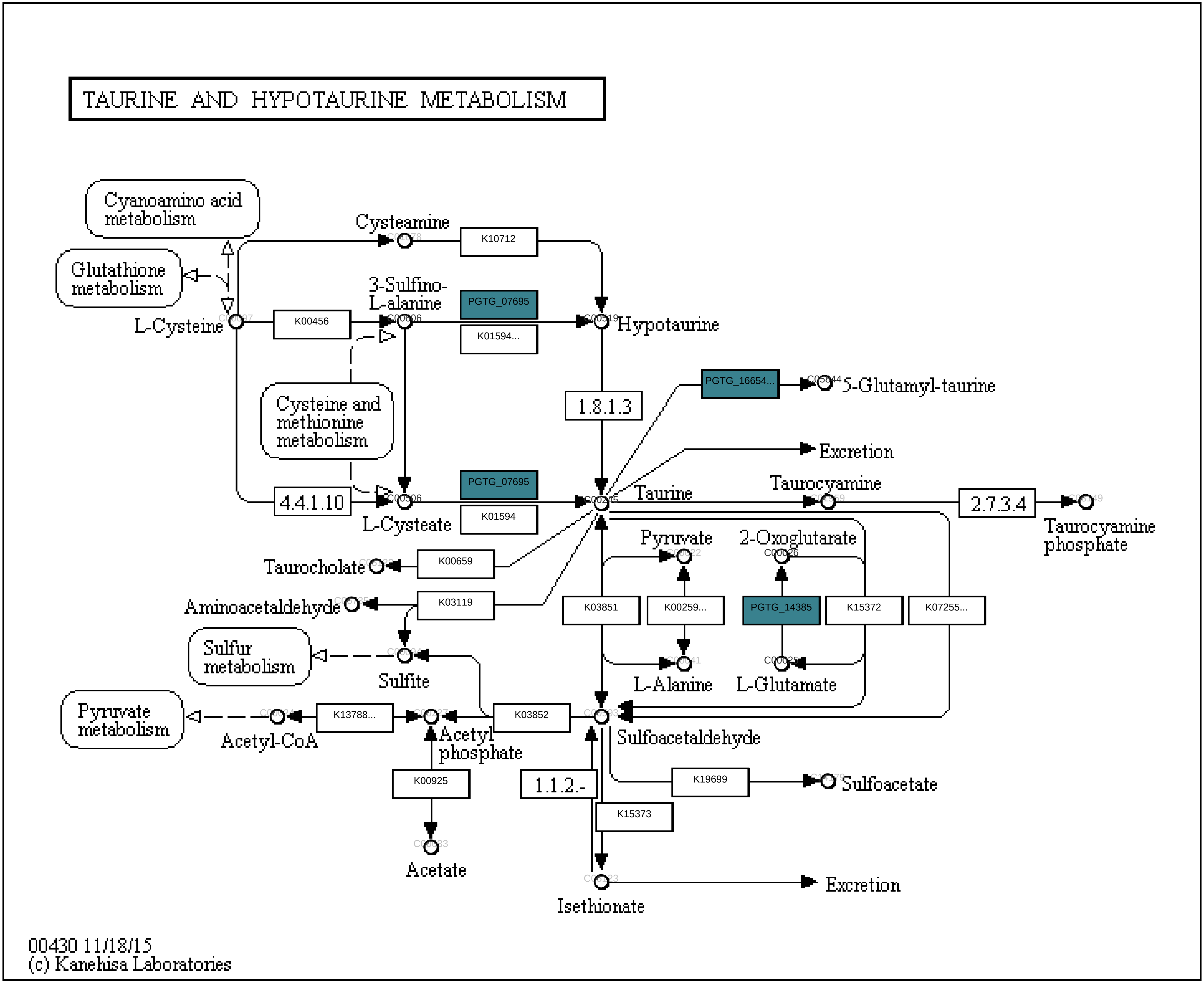
TAURINE AND HYPOTAURINE METABOLISM
Cyanoamino acid metabolism
Glutathione metabolism
Cysteamine
K10712
3-Sulfino-L-alanine
PGTG_07695
K01594...
L-Cysteine
K00456
Hypotaurine
PGTG_16654...
5-Glutamyl-taurine
Cysteine and methionine metabolism
1.8.1.3
Excretion
4.4.1.10
PGTG_07695
K01594
L-Cysteate
Taurine
Taurocyamine
2.7.3.4
Taurocyamine phosphate
Taurocholate
K00659
Pyruvate
2-Oxoglutarate
Aminoacetaldehyde
K03119
K03851
K00259...
PGTG_14385
K15372
K07255...
Sulfur metabolism
Sulfite
L-Alanine
L-Glutamate
Pyruvate metabolism
K13788...
K03852
Acetyl-CoA
Acetyl phosphate
Sulfoacetaldehyde
K00925
1.1.2.-
K19699
Sulfoacetate
K15373
Acetate
Isethionate
Excretion
00430 11/18/15
(c) Kanehisa Laboratories

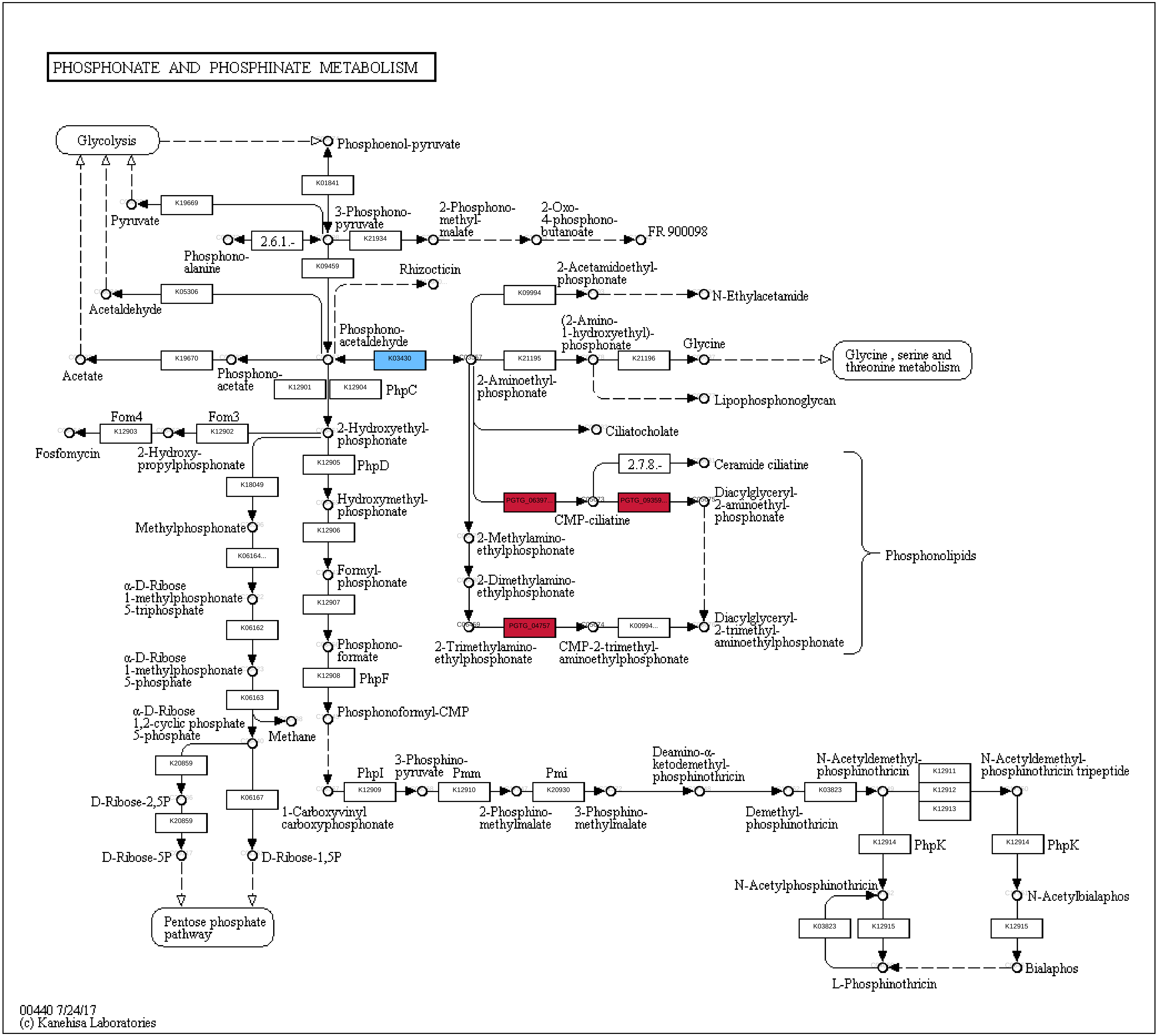
PHOSPHONATE AND PHOSPHINATE METABOLISM
Glycolysis
Phosphoenol-pyruvate
K01841
K19669
Pyruvate
3-Phosphono-pyruvate
2-Phosphono-methyl-malate
2-Oxo-4-phosphono-butanoate
FR 900098
2.6.1.-
K21934
Phosphono-alanine
K09459
Rhizocticin
K05306
Acetaldehyde
2-Acetamidoethyl-phosphonate
K09994
N-Ethylacetamide
(2-Amino-1-hydroxyethyl)-phosphonate
Glycine
Phosphono-acetaldehyde
K19670
K03430
K21195
K21196
Glycine , serine and threonine metabolism
Acetate
Phosphono-acetate
K12901
K12904
PhpC
2-Aminoethyl-phosphonate
Lipophosphonoglycan
Fom4
Fom3
K12903
K12902
2-Hydroxyethyl-phosphonate
Ciliatocholate
Fosfomycin
2-Hydroxy-propylphosphonate
K12905
PhpD
2.7.8.-
Ceramide ciliatine
K18049
Hydroxymethyl-phosphonate
PGTG_06397...
PGTG_09359...
Diacylglyceryl-2-aminoethyl-phosphonate
CMP-ciliatine
Methylphosphonate
K12906
K06164...
2-Methylamino-ethylphosphonate
Phosphonolipids
Formyl-phosphonate
α-D-Ribose 1-methylphosphonate 5-triphosphate
2-Dimethylamino-ethylphosphonate
K12907
K06162
PGTG_04757
K00994...
Diacylglyceryl-2-trimethyl-aminoethylphosphonate
Phosphono-formate
2-Trimethylamino-ethylphosphonate
CMP-2-trimethyl-aminoethylphosphonate
α-D-Ribose 1-methylphosphonate 5-phosphate
K12908
PhpF
K06163
α-D-Ribose 1,2-cyclic phosphate 5-phosphate
Phosphonoformyl-CMP
Methane
Deamino-α-ketodemethyl-phosphinothricin
N-Acetyldemethyl-phosphinothricin
N-Acetyldemethyl-phosphinothricin tripeptide
K20859
3-Phosphino-pyruvate
PhpI
Pmm
Pmi
K12911
K12912
K12913
K12909
K12910
K20930
K03823
D-Ribose-2,5P
K06167
1-Carboxyvinyl carboxyphosphonate
2-Phosphino-methylmalate
3-Phosphino-methylmalate
Demethyl-phosphinothricin
K20859
K12914
PhpK
K12914
PhpK
D-Ribose-5P
D-Ribose-1,5P
N-Acetylphosphinothricin
N-Acetylbialaphos
K03823
K12915
K12915
Pentose phosphate pathway
L-Phosphinothricin
Bialaphos
00440 7/24/17
(c) Kanehisa Laboratories

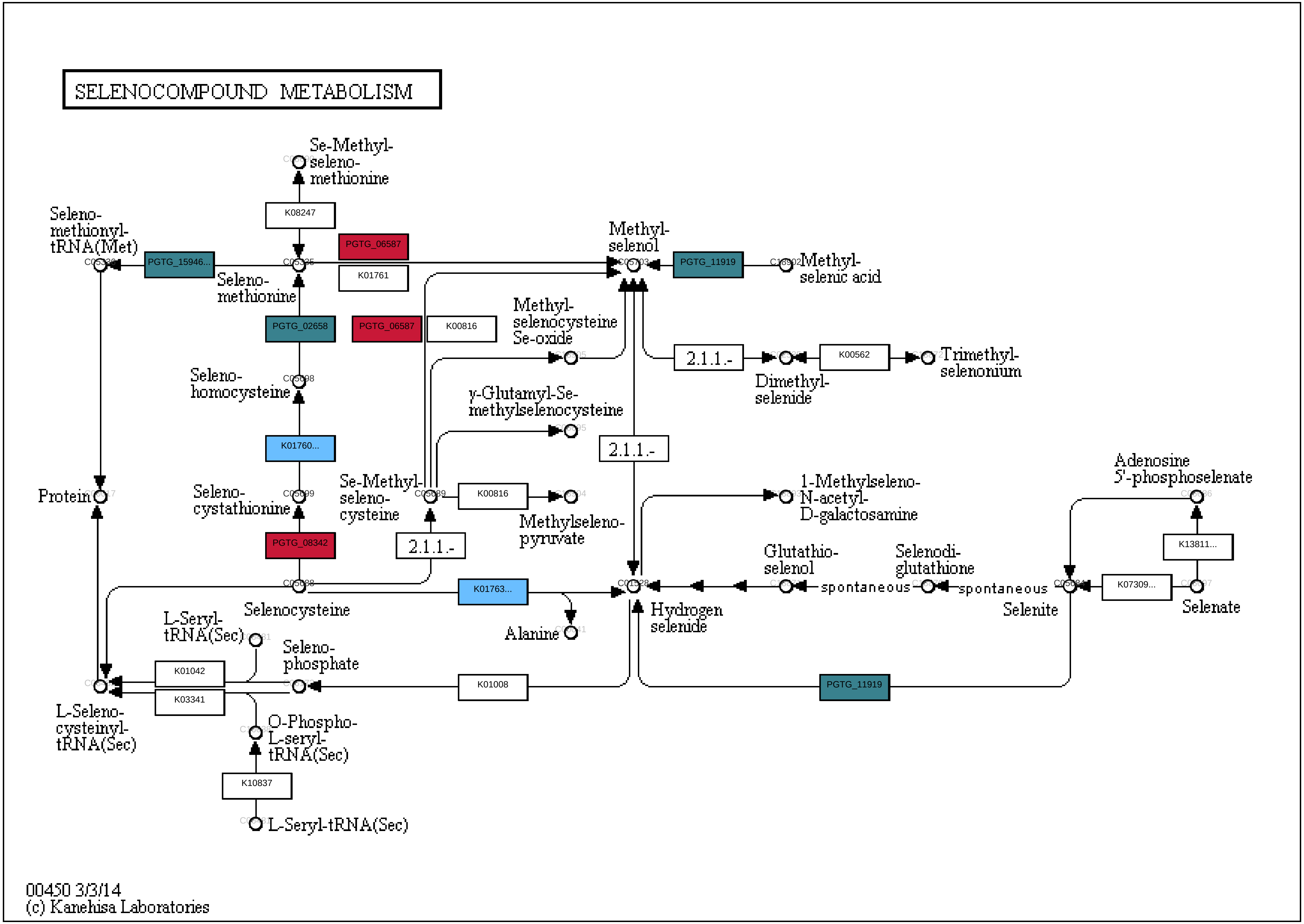
SELENOCOMPOUND METABOLISM
Se-Methyl-selenomethionine
K08247
Seleno-methionyl-tRNA(Met)
C05336
PGTG_15946...
C05335
PGTG_06587
K01761
Seleno-methionine
Methyl-selenol
C05703
PGTG_11919
C18902
Methyl-selenic acid
PGTG_02658
PGTG_06587
K00816
Methyl-selenocysteine Se-oxide
2.1.1.-
K00562
Trimethyl-selenonium
Dimethyl-selenide
Seleno-homocysteine
C05698
γ-Glutamyl-Se-methylselenocysteine
K01760...
2.1.1.-
Protein
Seleno-cystathionine
C05699
Se-Methyl-seleno-cysteine
C05689
K00816
Methylseleno-pyruvate
1-Methylseleno-N-acetyl-D-galactosamine
Adenosine 5'-phosphoselenate
PGTG_08342
2.1.1.-
K13811...
Glutathio-selenol
Selenodi-glutathione
C05688
K01763...
C01528
spontaneous
spontaneous
C05684
K07309...
Selenocysteine
Hydrogen selenide
Selenite
Selenate
L-Seryl-tRNA(Sec)
Alanine
Seleno-phosphate
K01042
K03341
PGTG_11919
K01008
L-Seleno-cysteinyl-tRNA(Sec)
O-Phospho-L-seryl-tRNA(Sec)
K10837
L-Seryl-tRNA(Sec)
00450 3/3/14
(c) Kanehisa Laboratories

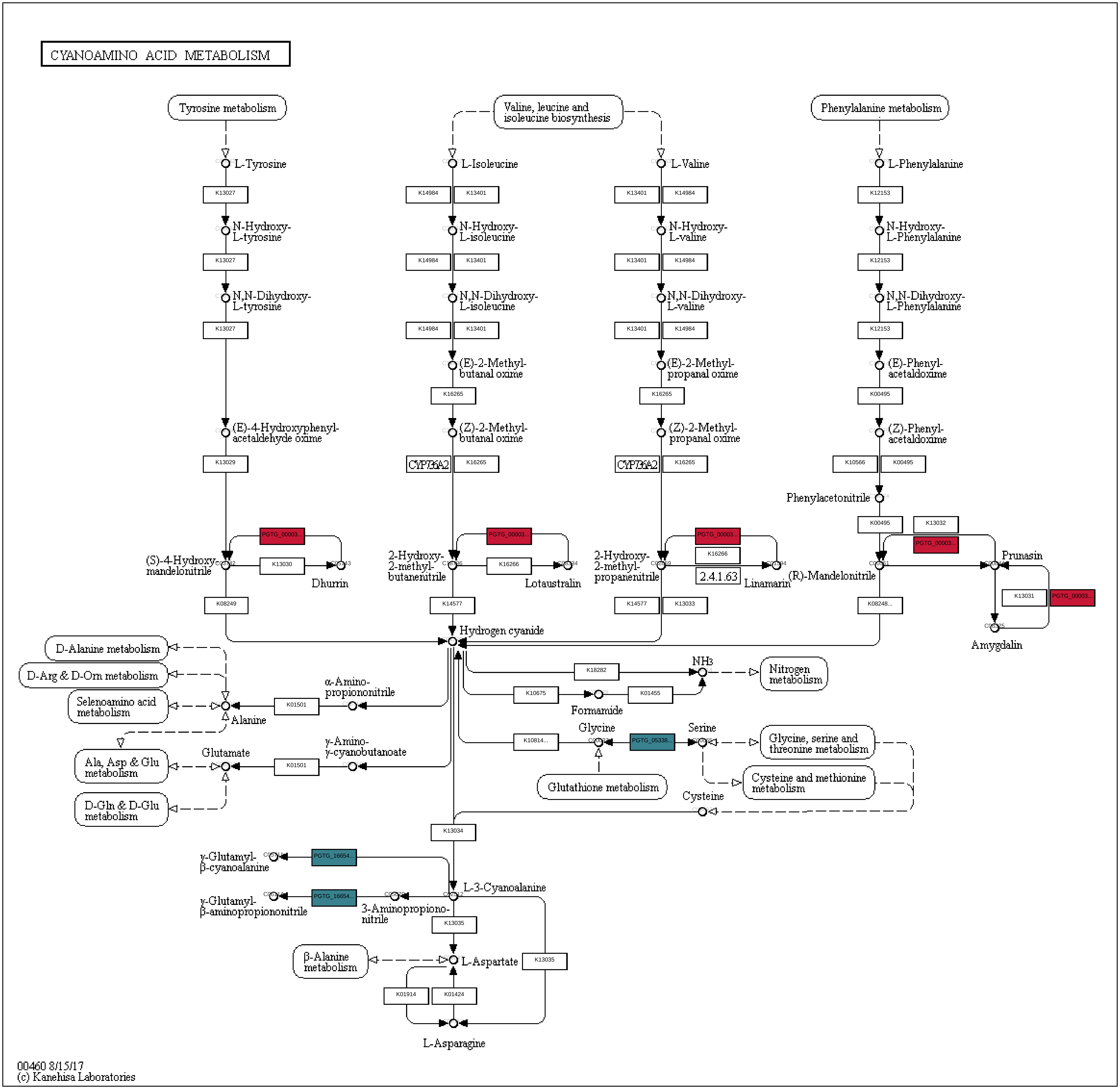
CYANOAMINO ACID METABOLISM
Tyrosine metabolism
Valine, leucine and isoleucine biosynthesis
Phenylalanine metabolism
L-Tyrosine
L-Isoleucine
L-Valine
L-Phenylalanine
K13027
K14984
K13401
K12153
N-Hydroxy-L-tyrosine
N-Hydroxy-L-isoleucine
N-Hydroxy-L-valine
N-Hydroxy-L-Phenylalanine
N,N-Dihydroxy-L-tyrosine
N,N-Dihydroxy-L-isoleucine
N,N-Dihydroxy-L-valine
N,N-Dihydroxy-L-Phenylalanine
(E)-2-Methyl-butanal oxime
(E)-2-Methyl-propanal oxime
(E)-Phenyl-acetaldoxime
K16265
K00495
(E)-4-Hydroxyphenyl-acetaldehyde oxime
(Z)-2-Methyl-butanal oxime
(Z)-2-Methyl-propanal oxime
(Z)-Phenyl-acetaldoxime
K13029
CYP736A2
K10566
Phenylacetonitrile
K13032
PGTG_00003...
K13030
K16266
2.4.1.63
(S)-4-Hydroxy-mandelonitrile
2-Hydroxy-2-methyl-butanenitrile
2-Hydroxy-2-methyl-propanenitrile
(R)-Mandelonitrile
Dhurrin
Lotaustralin
Linamarin
Prunasin
K13031
Amygdalin
K08249
K14577
K13033
K08248...
Hydrogen cyanide
D-Alanine metabolism
D-Arg & D-Orn metabolism
Selenoamino acid metabolism
K18282
NH3
Nitrogen metabolism
α-Amino-propiononitrile
K01501
K10675
K01455
Formamide
Alanine
Glycine
Serine
K10814...
PGTG_05338...
Glycine, serine and threonine metabolism
Glutamate
γ-Amino-γ-cyanobutanoate
Ala, Asp & Glu metabolism
D-Gln & D-Glu metabolism
Glutathione metabolism
Cysteine and methionine metabolism
Cysteine
K13034
γ-Glutamyl-β-cyanoalanine
PGTG_16654...
L-3-Cyanoalanine
γ-Glutamyl-β-aminopropiononitrile
3-Aminopropiono-nitrile
K13035
β-Alanine metabolism
L-Aspartate
K01914
K01424
L-Asparagine
00460 8/15/17
(c) Kanehisa Laboratories

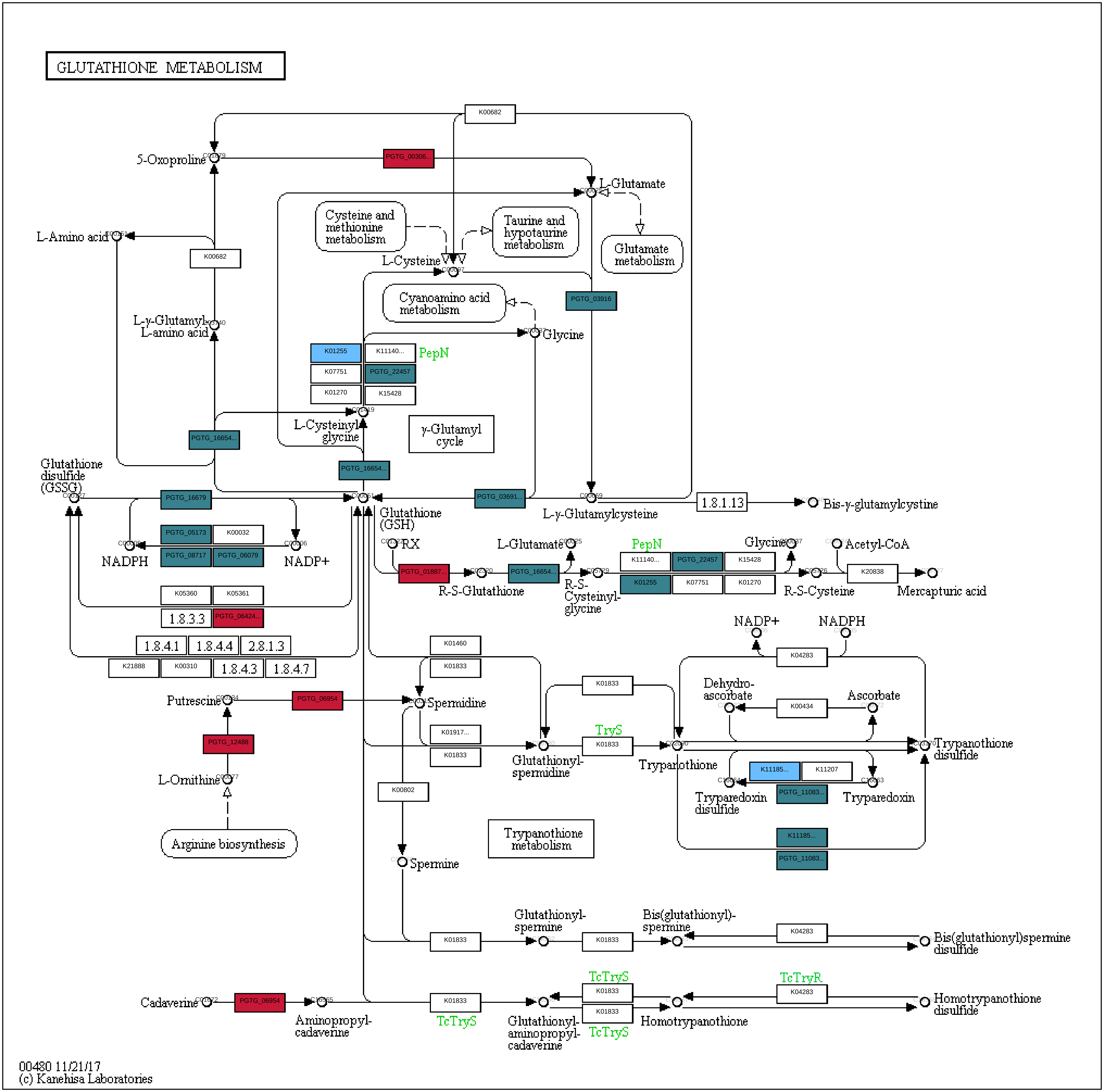
GLUTATHIONE METABOLISM
5-Oxoproline
PGTG_00306...
K00682
L-Glutamate
Cysteine and methionine metabolism
Taurine and hypotaurine metabolism
Glutamate metabolism
L-Amino acid
L-Cysteine
Cyanoamino acid metabolism
PGTG_03916
L-γ-Glutamyl-L-amino acid
Glycine
K01255
K11140...
PepN
K07751
PGTG_22457
K01270
K15428
L-Cysteinyl-glycine
γ-Glutamyl cycle
PGTG_16654...
Glutathione disulfide (GSSG)
PGTG_16679
PGTG_03691...
L-γ-Glutamylcysteine
1.8.1.13
Bis-γ-glutamylcystine
Glutathione (GSH)
PGTG_05173
K00032
PGTG_08717
PGTG_06079
NADPH
NADP+
RX
L-Glutamate
PepN
Glycine
Acetyl-CoA
PGTG_01887...
PGTG_16654...
K11140...
PGTG_22457
K15428
K20838
K01255
K07751
K01270
R-S-Glutathione
R-S-Cysteinyl-glycine
R-S-Cysteine
Mercapturic acid
K05360
K05361
1.8.3.3
PGTG_06424...
1.8.4.1
1.8.4.4
2.8.1.3
K21888
K00310
1.8.4.3
1.8.4.7
K01460
K01833
NADP+
NADPH
K04283
Putrescine
PGTG_06954
Spermidine
K01833
Dehydro-ascorbate
K00434
Ascorbate
PGTG_12488
TryS
K01917...
K01833
Trypanothione
Trypanothione disulfide
Glutathionyl-spermidine
K11185...
K11207
L-Ornithine
K00802
PGTG_11083...
Tryparedoxin disulfide
Tryparedoxin
Arginine biosynthesis
Trypanothione metabolism
Spermine
Glutathionyl-spermine
Bis(glutathionyl)-spermine
Bis(glutathionyl)spermine disulfide
TcTryS
TcTryR
Cadaverine
Aminopropyl-cadaverine
Glutathionyl-aminopropyl-cadaverine
Homotrypanothione
Homotrypanothione disulfide
00480 11/21/17
(c) Kanehisa Laboratories

# 7. Glycan biosynthesis and metabolism

| MAP | PATHWAY |
|---|---|
| **510** | N-Glycan biosynthesis |
| **513** | Various types of N-glycan biosynthesis |
| **531** | Glycosaminoglycan degradation |
| **563** | Glycosylphosphatidylinositol (GPI)-anchor biosynthesis |

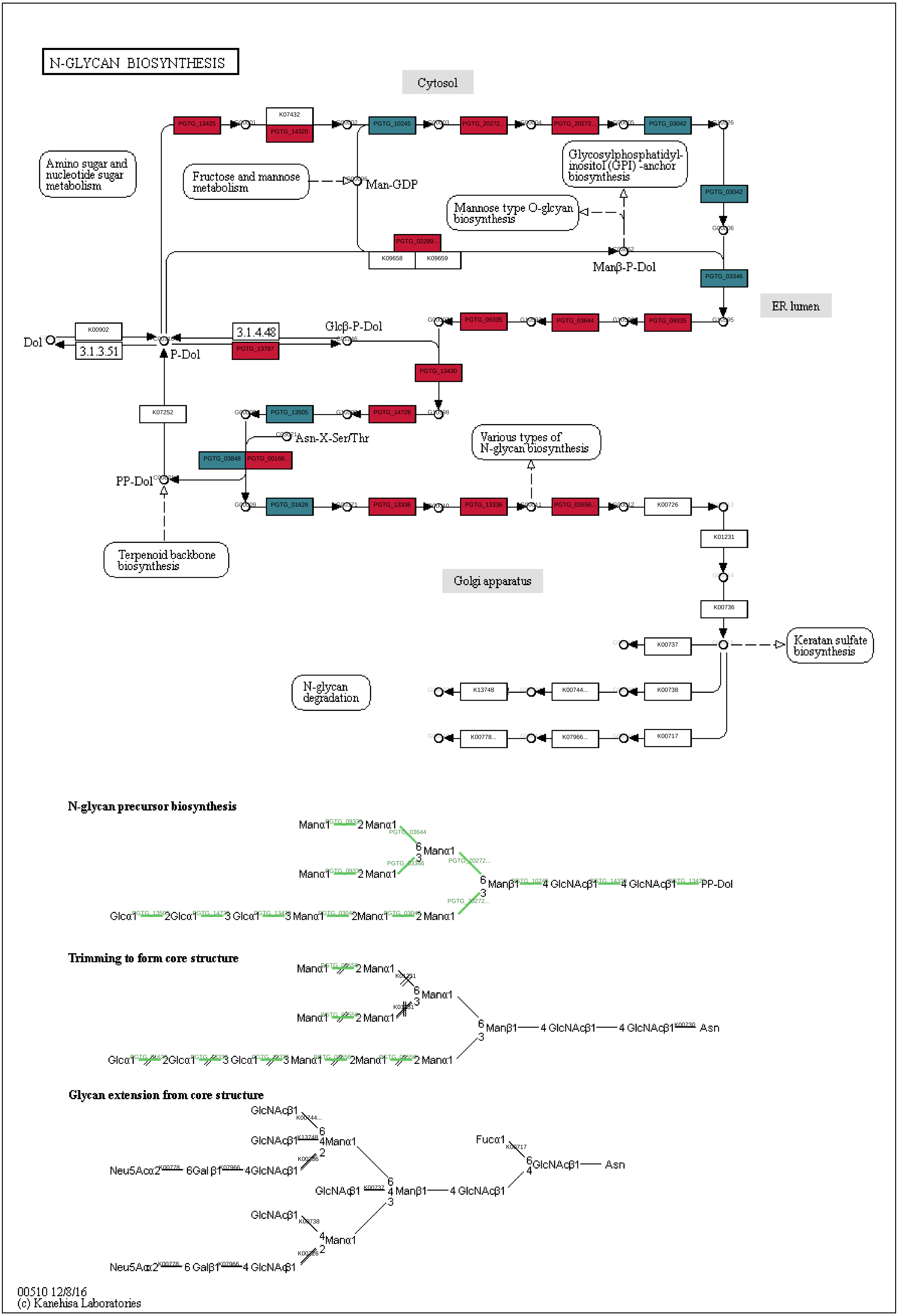

N-GLYCAN BIOSYNTHESIS
Cytosol
ER lumen
Golgi apparatus
Amino sugar and nucleotide sugar metabolism
Fructose and mannose metabolism
Glycosylphosphatidylinositol (GPI) -anchor biosynthesis
Mannose type O-glcyan biosynthesis
Various types of N-glycan biosynthesis
Terpenoid backbone biosynthesis
Keratan sulfate biosynthesis
N-glycan degradation
Man-GDP
Manβ-P-Dol
Glcβ-P-Dol
Dol
P-Dol
PP-Dol
Asn-X-Ser/Thr
N-glycan precursor biosynthesis
Trimming to form core structure
Glycan extension from core structure
00510 12/8/16
(c) Kanehisa Laboratories

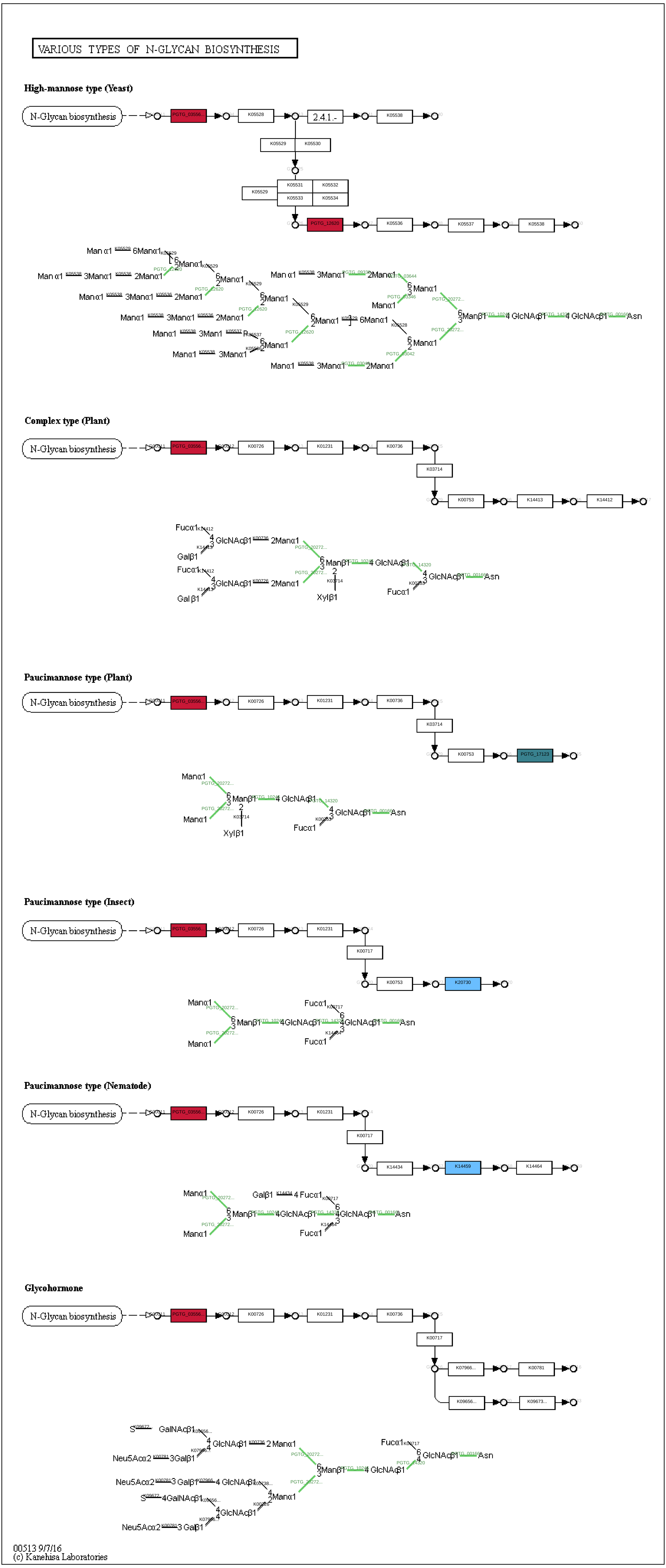
VARIOUS TYPES OF N-GLYCAN BIOSYNTHESIS
High-mannose type (Yeast)
N-Glycan biosynthesis
PGTG_03556
K05528
2.4.1.-
K05538
K05529
K05530
K05531
K05532
K05533
K05534
PGTG_12620
K05536
K05537
Complex type (Plant)
K00726
K01231
K00736
K03714
K00753
K14413
K14412
Paucimannose type (Plant)
PGTG_17123
Paucimannose type (Insect)
K00717
K20730
Paucimannose type (Nematode)
K14434
K14459
K14464
Glycohormone
K07966...
K00781
K09656...
K09673...
00513 9/7/16
(c) Kanehisa Laboratories

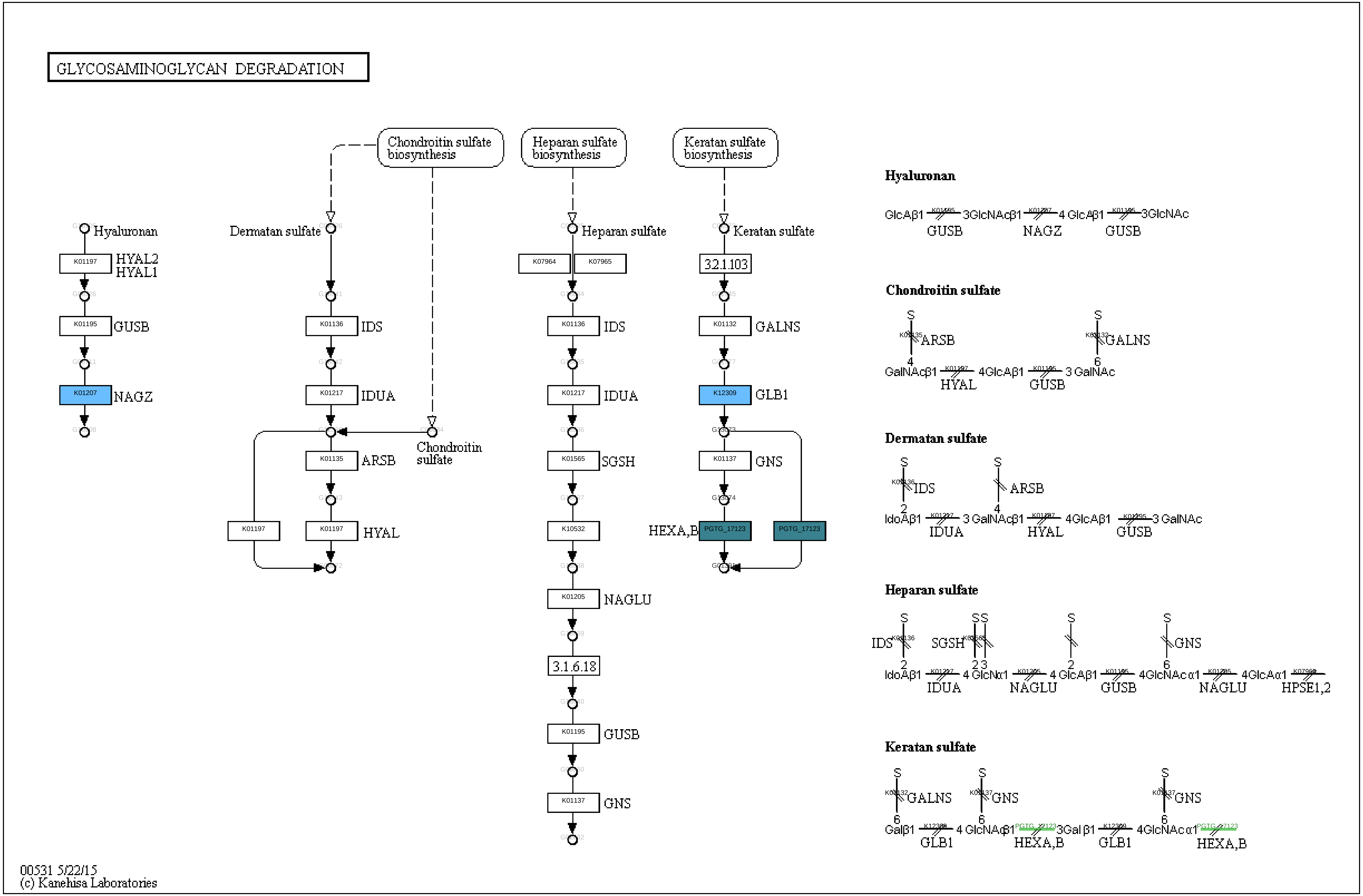
GLYCOSAMINOGLYCAN DEGRADATION
Chondroitin sulfate biosynthesis
Heparan sulfate biosynthesis
Keratan sulfate biosynthesis
Hyaluronan
K01197
HYAL2
HYAL1
K01195
GUSB
K01207
NAGZ
Dermatan sulfate
K01136
IDS
K01217
IDUA
Chondroitin sulfate
K01135
ARSB
K01197
K01197
HYAL
Heparan sulfate
K07964
K07965
K01136
IDS
K01217
IDUA
K01565
SGSH
K10532
K01205
NAGLU
3.1.6.18
K01195
GUSB
K01137
GNS
Keratan sulfate
3.2.1.103
K01132
GALNS
K12309
GLB1
K01137
GNS
HEXA,B
PGTG_17123
PGTG_17123
Hyaluronan
GlcAβ1 3GlcNAcβ1 4 GlcAβ1 3GlcNAc
GUSB NAGZ GUSB
Chondroitin sulfate
ARSB
GALNS
GalNAcβ1 4GlcAβ1 3 GalNAc
HYAL GUSB
Dermatan sulfate
IDS
ARSB
IdoAβ1 3 GalNAcβ1 4GlcAβ1 3 GalNAc
IDUA HYAL GUSB
Heparan sulfate
IDS
SGSH
GNS
IdoAβ1 4 GlcNα1 4 GlcAβ1 4GlcNAcα1 4GlcAα1
IDUA NAGLU GUSB NAGLU HPSE1,2
Keratan sulfate
GALNS
GNS
GNS
Galβ1 4 GlcNAcβ1 3Gal β1 4GlcNAcα1
GLB1 HEXA,B GLB1 HEXA,B
00531 5/22/15
(c) Kanehisa Laboratories

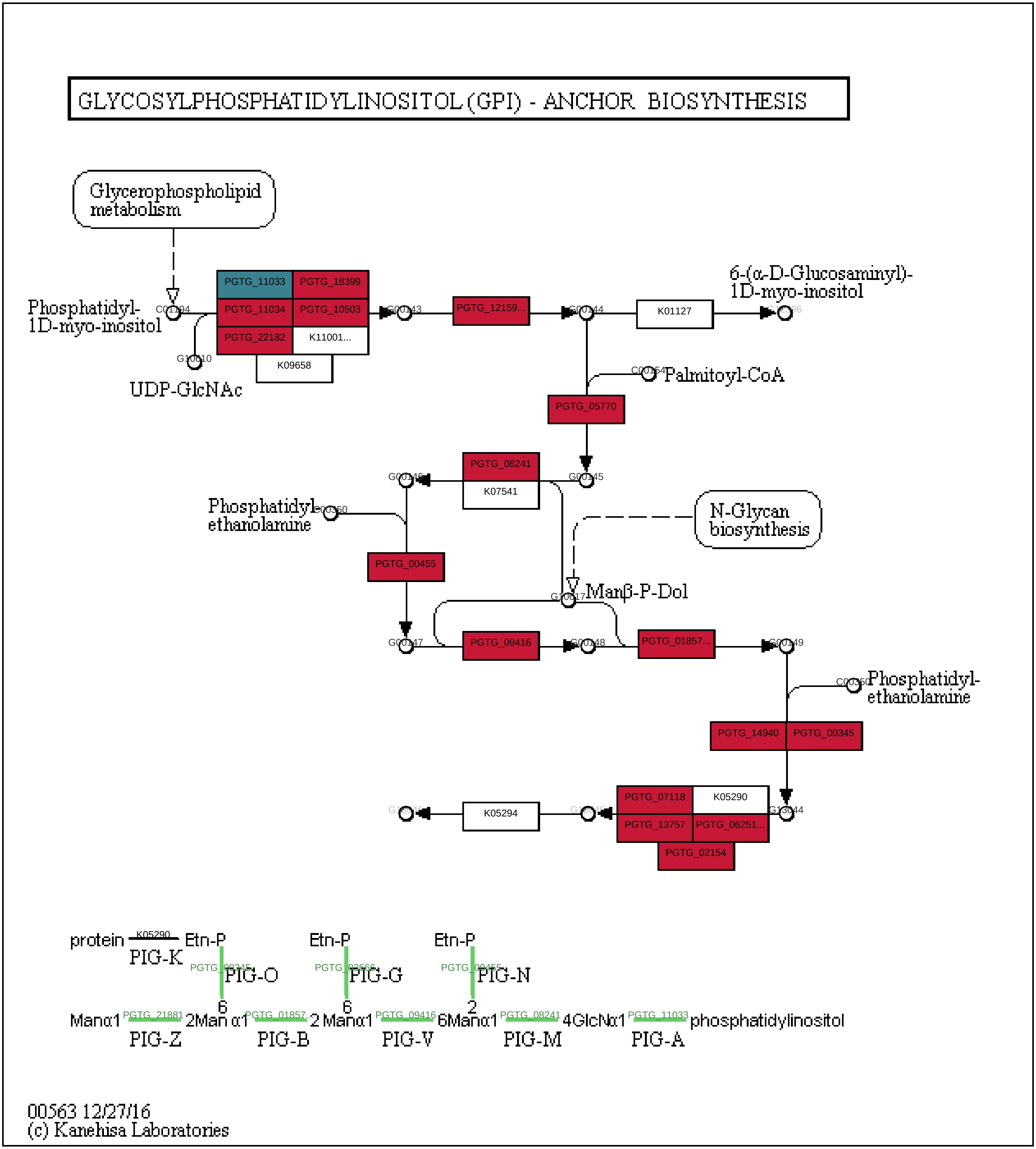
GLYCOSYLPHOSPHATIDYLINOSITOL (GPI) - ANCHOR BIOSYNTHESIS
Glycerophospholipid metabolism
Phosphatidyl-1D-myo-inositol
C01194
G10610
UDP-GlcNAc
PGTG_11033
PGTG_18399
PGTG_11034
PGTG_10503
PGTG_22182
K11001...
K09658
G00143
PGTG_12159...
G00144
K01127
6-(α-D-Glucosaminyl)-1D-myo-inositol
C00154
Palmitoyl-CoA
PGTG_05770
PGTG_08241
K07541
G00146
G00145
N-Glycan biosynthesis
Phosphatidyl-ethanolamine
C00350
PGTG_00455
G10617
Manβ-P-Dol
G00147
PGTG_09416
G00148
PGTG_01857...
G00149
C00350
Phosphatidyl-ethanolamine
PGTG_14940
PGTG_00345
PGTG_07118
K05290
PGTG_13757
PGTG_06251...
PGTG_02154
G13044
K05294
protein
K05290
PIG-K
Etn-P
PGTG_00345
PIG-O
6
Etn-P
PGTG_03666
PIG-G
6
Etn-P
PGTG_00455
PIG-N
2
Manα1
PGTG_21881
2Man α1
PGTG_01857
2 Manα1
PGTG_09416
6Manα1
PGTG_08241
4GlcNα1
PGTG_11033
phosphatidylinositol
PIG-Z
PIG-B
PIG-V
PIG-M
PIG-A
00563 12/27/16
(c) Kanehisa Laboratories

# 8. Metabolism of cofactors and vitamins

| MAP | PATHWAY |
|---|---|
| **130** | Ubiquinone and other terpenoid-quinone biosynthesis |
| **670** | One carbon pool by folate |
| **730** | Thiamine metabolism |
| **740** | Riboflavin metabolism |
| **750** | Vitamin B6 metabolism |
| **760** | Nicotinate and nicotinamide metabolism |
| **770** | Pantothenate and CoA biosynthesis |
| **780** | Biotin metabolism |
| **785** | Lipoic acid metabolism |
| **790** | Folate biosynthesis |
| **860** | Porphyrin and chlorophyll metabolism |

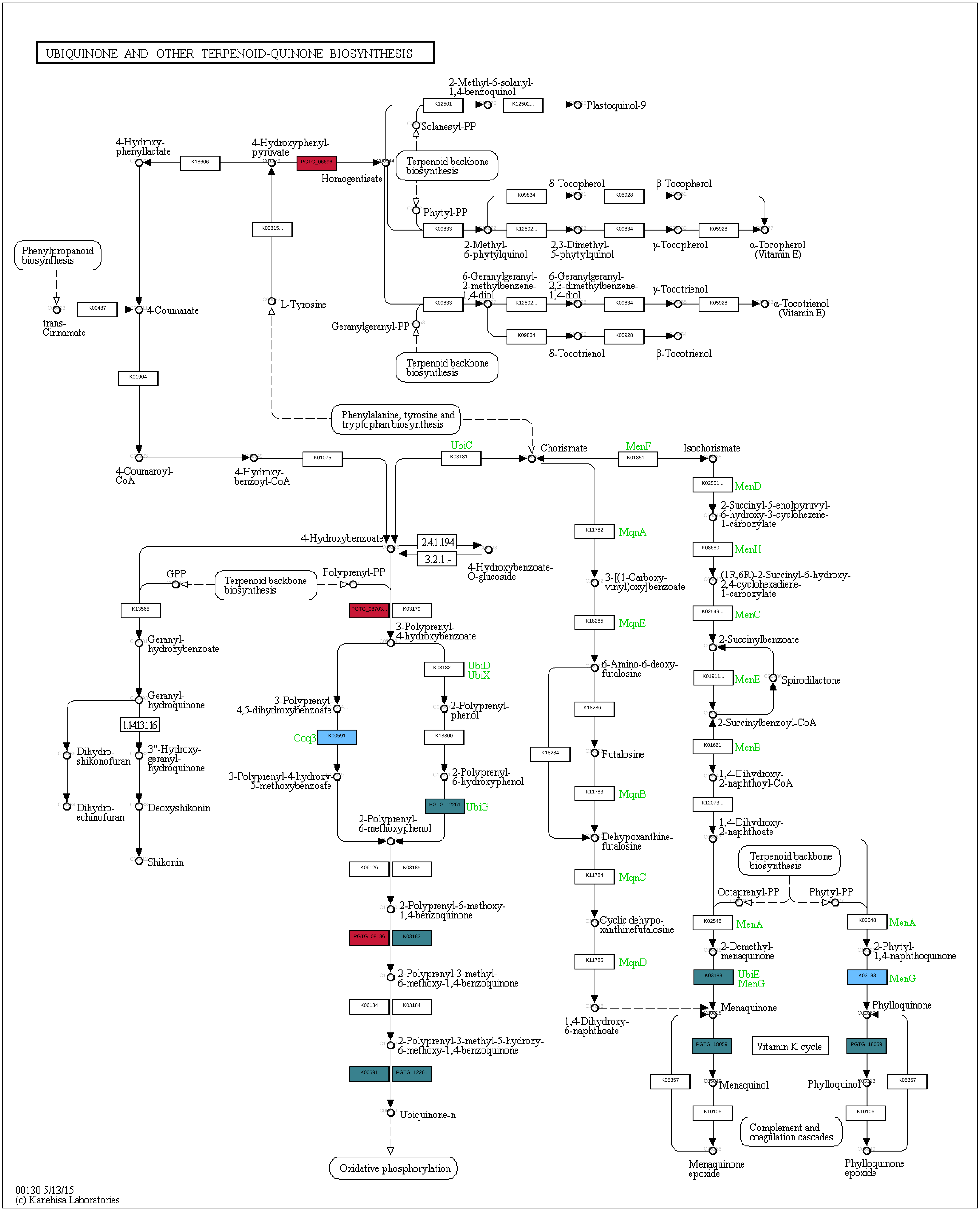
UBIQUINONE AND OTHER TERPENOID-QUINONE BIOSYNTHESIS
2-Methyl-6-solanyl-1,4-benzoquinol
Plastoquinol-9
Solanesyl-PP
4-Hydroxy-phenyllactate
4-Hydroxyphenyl-pyruvate
Homogentisate
Terpenoid backbone biosynthesis
δ-Tocopherol
β-Tocopherol
Phytyl-PP
2-Methyl-6-phytylquinol
2,3-Dimethyl-5-phytylquinol
γ-Tocopherol
α-Tocopherol (Vitamin E)
Phenylpropanoid biosynthesis
6-Geranylgeranyl-2-methylbenzene-1,4-diol
6-Geranylgeranyl-2,3-dimethylbenzene-1,4-diol
γ-Tocotrienol
α-Tocotrienol (Vitamin E)
trans-Cinnamate
4-Coumarate
L-Tyrosine
Geranylgeranyl-PP
δ-Tocotrienol
β-Tocotrienol
Phenylalanine, tyrosine and tryptophan biosynthesis
4-Coumaroyl-CoA
4-Hydroxy-benzoyl-CoA
UbiC
Chorismate
MenF
Isochorismate
MenD
2-Succinyl-5-enolpyruvyl-6-hydroxy-3-cyclohexene-1-carboxylate
MqnA
MenH
4-Hydroxybenzoate
2.4.1.194
3.2.1.-
4-Hydroxybenzoate-O-glucoside
GPP
Polyprenyl-PP
3-[(1-Carboxy-vinyl)oxy]benzoate
(1R,6R)-2-Succinyl-6-hydroxy-2,4-cyclohexadiene-1-carboxylate
MqnE
MenC
Geranyl-hydroxybenzoate
3-Polyprenyl-4-hydroxybenzoate
2-Succinylbenzoate
6-Amino-6-deoxy-futalosine
UbiD
UbiX
MenE
Spirodilactone
Geranyl-hydroquinone
3-Polyprenyl-4,5-dihydroxybenzoate
2-Polyprenyl-phenol
2-Succinylbenzoyl-CoA
1.14.13.116
Coq3
Dihydro-shikonofuran
3''-Hydroxy-geranyl-hydroquinone
Futalosine
MenB
3-Polyprenyl-4-hydroxy-5-methoxybenzoate
2-Polyprenyl-6-hydroxyphenol
1,4-Dihydroxy-2-naphthoyl-CoA
MqnB
Dihydro-echinofuran
Deoxyshikonin
UbiG
2-Polyprenyl-6-methoxyphenol
1,4-Dihydroxy-2-naphthoate
Dehypoxanthine-futalosine
Shikonin
MqnC
Octaprenyl-PP
Phytyl-PP
2-Polyprenyl-6-methoxy-1,4-benzoquinone
Cyclic dehypo-xanthinefutalosine
MenA
2-Demethyl-menaquinone
2-Phytyl-1,4-naphthoquinone
2-Polyprenyl-3-methyl-6-methoxy-1,4-benzoquinone
MqnD
UbiE
MenG
Menaquinone
Phylloquinone
1,4-Dihydroxy-6-naphthoate
2-Polyprenyl-3-methyl-5-hydroxy-6-methoxy-1,4-benzoquinone
Vitamin K cycle
Menaquinol
Phylloquinol
Ubiquinone-n
Complement and coagulation cascades
Oxidative phosphorylation
Menaquinone epoxide
Phylloquinone epoxide
00130 5/13/15
(c) Kanehisa Laboratories

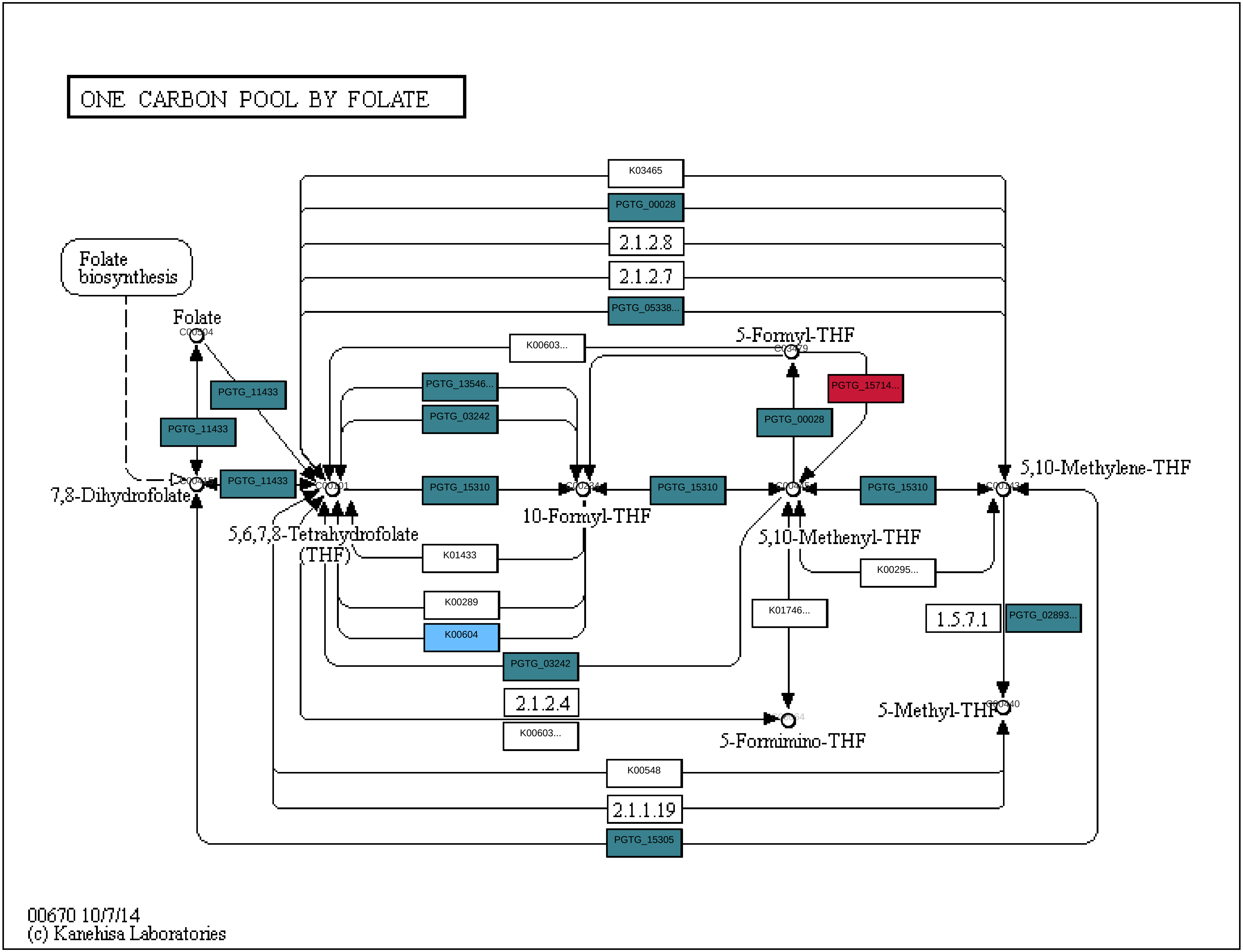

ONE CARBON POOL BY FOLATE
Folate biosynthesis
Folate
7,8-Dihydrofolate
5,6,7,8-Tetrahydrofolate (THF)
10-Formyl-THF
5-Formyl-THF
5,10-Methenyl-THF
5,10-Methylene-THF
5-Methyl-THF
5-Formimino-THF
K03465
PGTG_00028
2.1.2.8
2.1.2.7
PGTG_05338...
K00603...
PGTG_13546...
PGTG_03242
PGTG_15310
PGTG_15714...
PGTG_11433
K01433
K00289
K00604
K01746...
K00295...
1.5.7.1
PGTG_02893...
2.1.2.4
K00548
2.1.1.19
PGTG_15305
00670 10/7/14
(c) Kanehisa Laboratories

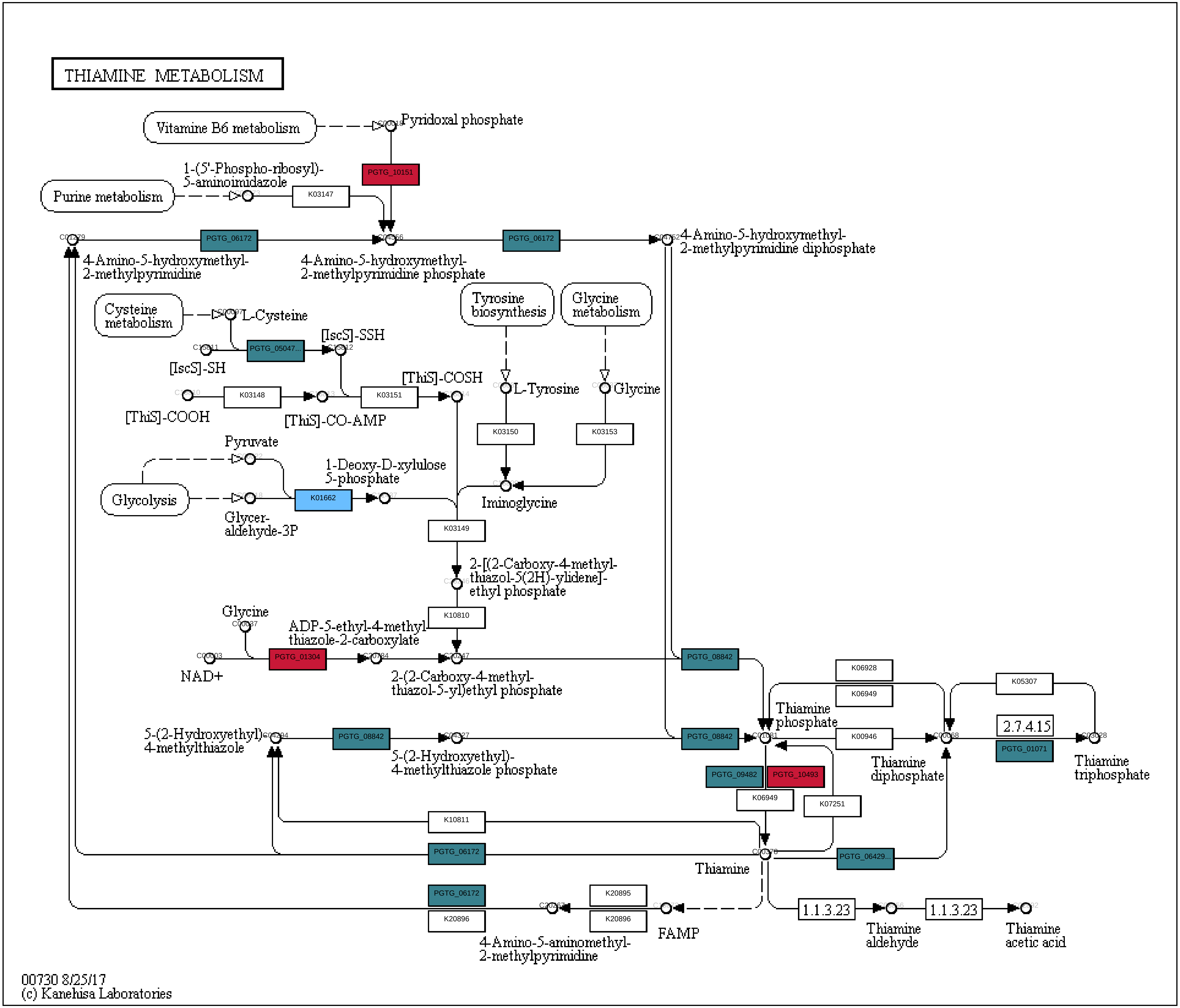
THIAMINE METABOLISM
Vitamine B6 metabolism
Pyridoxal phosphate
PGTG_10151
1-(5'-Phospho-ribosyl)-5-aminoimidazole
Purine metabolism
K03147
PGTG_06172
PGTG_06172
4-Amino-5-hydroxymethyl-2-methylpyrimidine diphosphate
4-Amino-5-hydroxymethyl-2-methylpyrimidine
4-Amino-5-hydroxymethyl-2-methylpyrimidine phosphate
Cysteine metabolism
L-Cysteine
Tyrosine biosynthesis
Glycine metabolism
[IscS]-SSH
PGTG_05047...
[IscS]-SH
[ThiS]-COSH
K03148
K03151
L-Tyrosine
Glycine
[ThiS]-COOH
[ThiS]-CO-AMP
K03150
K03153
Pyruvate
1-Deoxy-D-xylulose 5-phosphate
Glycolysis
K01662
Iminoglycine
Glycer-aldehyde-3P
K03149
2-[(2-Carboxy-4-methyl-thiazol-5(2H)-ylidene]-ethyl phosphate
Glycine
K10810
ADP-5-ethyl-4-methyl-thiazole-2-carboxylate
NAD+
PGTG_01304
PGTG_08842
2-(2-Carboxy-4-methyl-thiazol-5-yl)ethyl phosphate
K06928
K06949
K05307
Thiamine phosphate
2.7.4.15
5-(2-Hydroxyethyl)-4-methylthiazole
PGTG_08842
PGTG_08842
K00946
5-(2-Hydroxyethyl)-4-methylthiazole phosphate
PGTG_01071
Thiamine diphosphate
Thiamine triphosphate
PGTG_09482
PGTG_10493
K06949
K07251
K10811
PGTG_06172
PGTG_06429...
Thiamine
PGTG_06172
K20895
K20896
K20896
1.1.3.23
1.1.3.23
FAMP
Thiamine aldehyde
Thiamine acetic acid
4-Amino-5-aminomethyl-2-methylpyrimidine
00730 8/25/17
(c) Kanehisa Laboratories

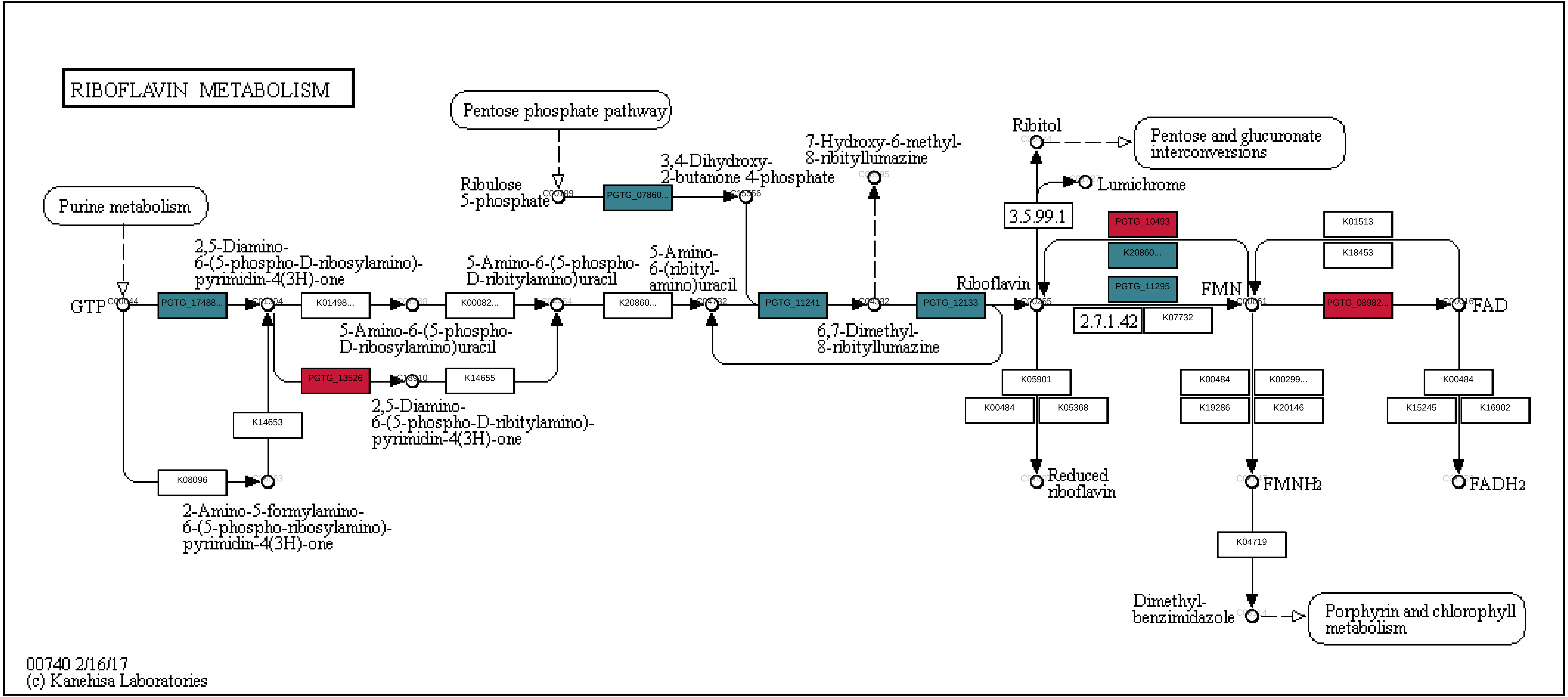
RIBOFLAVIN METABOLISM
Pentose phosphate pathway
Purine metabolism
Pentose and glucuronate interconversions
Porphyrin and chlorophyll metabolism
GTP
Ribulose 5-phosphate
3,4-Dihydroxy-2-butanone 4-phosphate
7-Hydroxy-6-methyl-8-ribityllumazine
Ribitol
Lumichrome
2,5-Diamino-6-(5-phospho-D-ribosylamino)-pyrimidin-4(3H)-one
5-Amino-6-(5-phospho-D-ribitylamino)uracil
5-Amino-6-(ribitylamino)uracil
5-Amino-6-(5-phospho-D-ribosylamino)uracil
2,5-Diamino-6-(5-phospho-D-ribitylamino)-pyrimidin-4(3H)-one
2-Amino-5-formylamino-6-(5-phospho-ribosylamino)-pyrimidin-4(3H)-one
6,7-Dimethyl-8-ribityllumazine
Riboflavin
FMN
FAD
Reduced riboflavin
FMNH2
FADH2
Dimethyl-benzimidazole
PGTG_17488...
K01498...
K00082...
K20860...
PGTG_07860...
PGTG_11241
PGTG_12133
PGTG_13526
K14655
K14653
K08096
3.5.99.1
2.7.1.42
K07732
PGTG_10493
K20860...
PGTG_11295
PGTG_08982...
K01513
K18453
K05901
K00484
K05368
K00484
K00299...
K19286
K20146
K00484
K15245
K16902
K04719
C00044
C01304
C00199
C15556
C04732
C04332
C00255
C00061
C00016
00740 2/16/17
(c) Kanehisa Laboratories

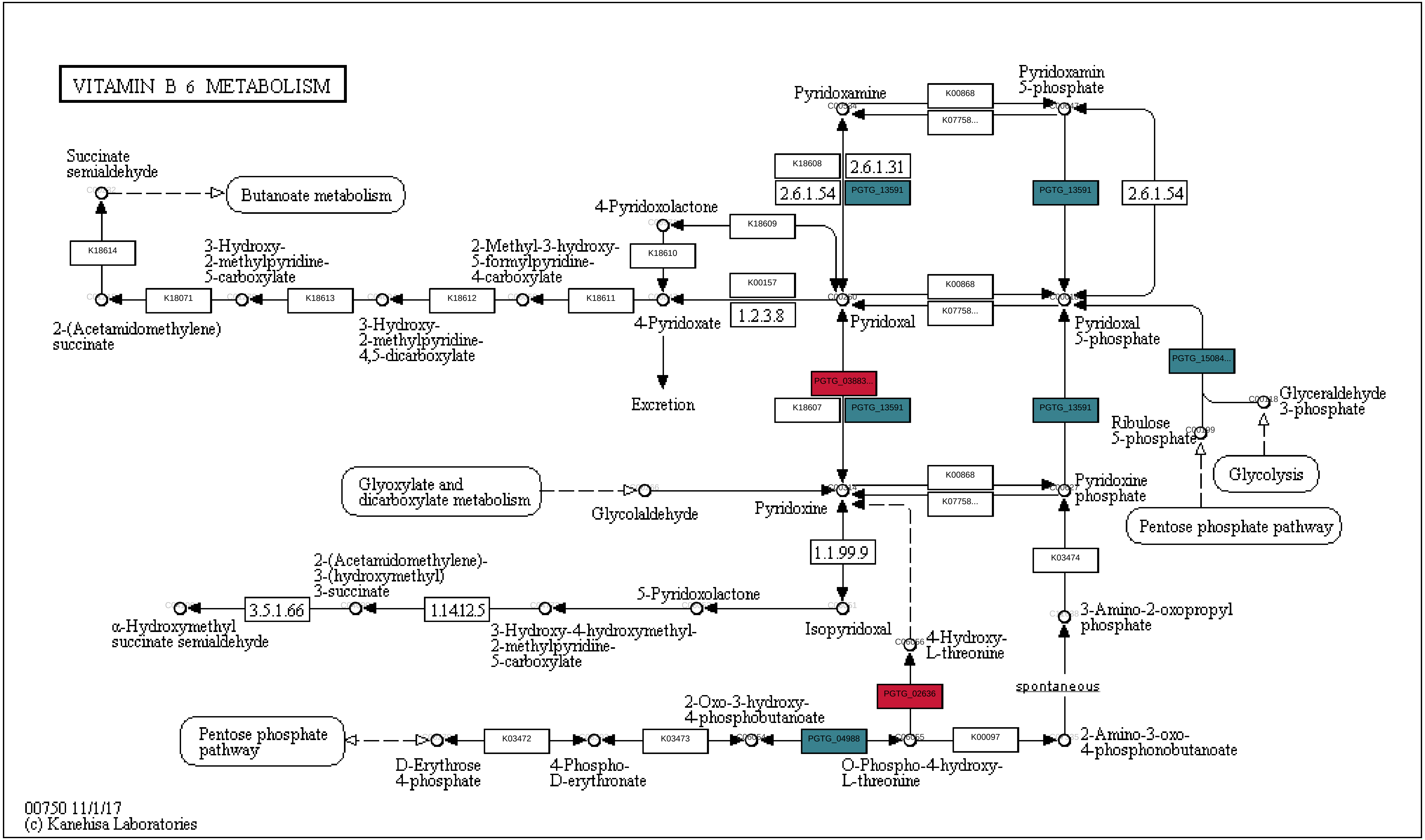

VITAMIN B 6 METABOLISM
Succinate semialdehyde
Butanoate metabolism
K18614
3-Hydroxy-2-methylpyridine-5-carboxylate
2-Methyl-3-hydroxy-5-formylpyridine-4-carboxylate
4-Pyridoxolactone
K18609
K18610
K18071
K18613
K18612
K18611
K00157
1.2.3.8
2-(Acetamidomethylene) succinate
3-Hydroxy-2-methylpyridine-4,5-dicarboxylate
4-Pyridoxate
Excretion
Pyridoxamine
K00868
K07758...
Pyridoxamin 5-phosphate
K18608
2.6.1.31
2.6.1.54
PGTG_13591
2.6.1.54
Pyridoxal
Pyridoxal 5-phosphate
PGTG_03883...
K18607
PGTG_13591
PGTG_15084...
Glyceraldehyde 3-phosphate
Ribulose 5-phosphate
Glycolysis
Glyoxylate and dicarboxylate metabolism
Glycolaldehyde
Pyridoxine
Pyridoxine phosphate
Pentose phosphate pathway
1.1.99.9
K03474
2-(Acetamidomethylene)-3-(hydroxymethyl)-3-succinate
5-Pyridoxolactone
3.5.1.66
1.14.12.5
α-Hydroxymethyl succinate semialdehyde
3-Hydroxy-4-hydroxymethyl-2-methylpyridine-5-carboxylate
Isopyridoxal
4-Hydroxy-L-threonine
3-Amino-2-oxopropyl phosphate
PGTG_02636
spontaneous
2-Oxo-3-hydroxy-4-phosphobutanoate
Pentose phosphate pathway
K03472
K03473
PGTG_04988
K00097
D-Erythrose 4-phosphate
4-Phospho-D-erythronate
O-Phospho-4-hydroxy-L-threonine
2-Amino-3-oxo-4-phosphonobutanoate
00750 11/1/17
(c) Kanehisa Laboratories

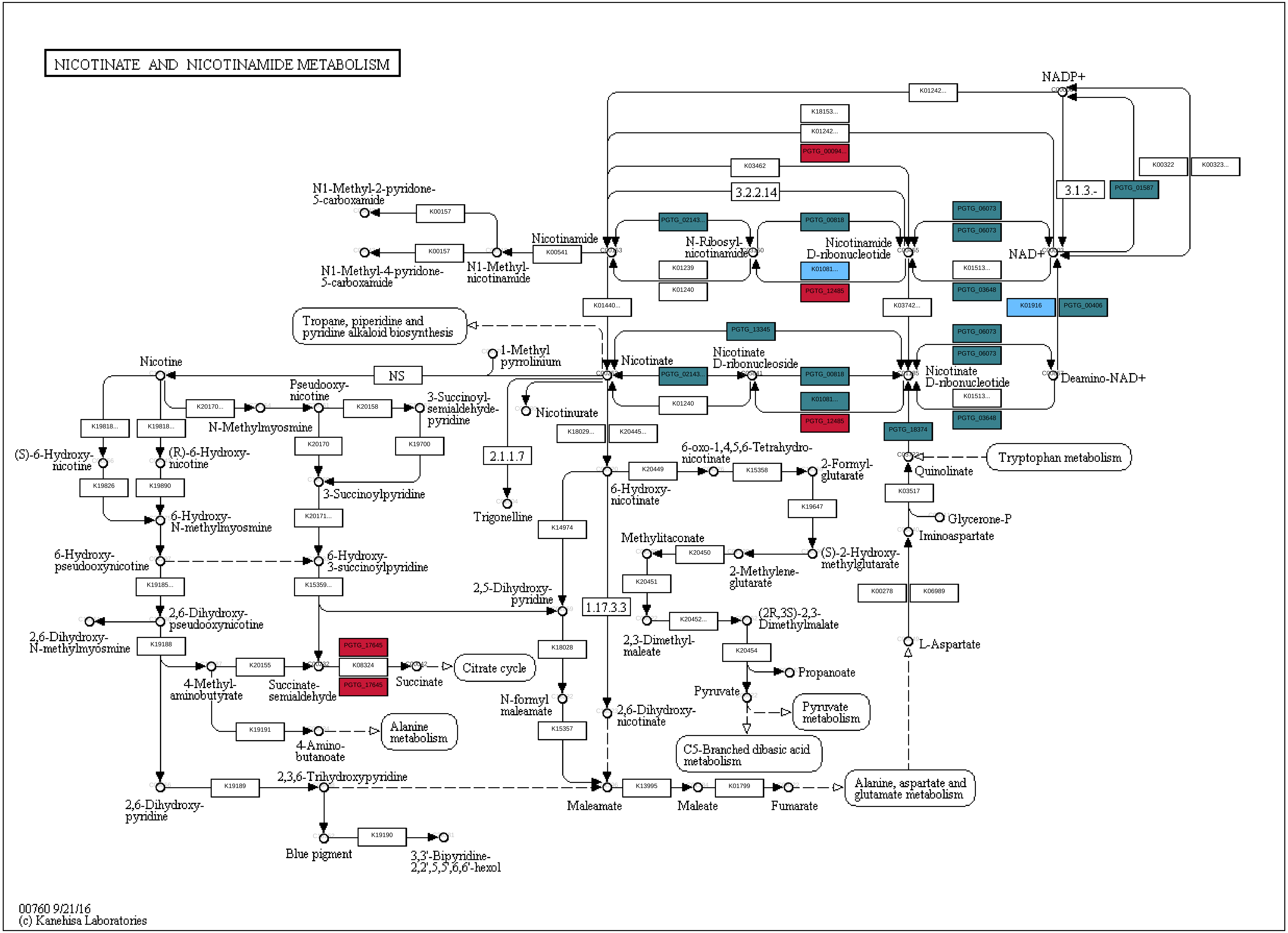
NICOTINATE AND NICOTINAMIDE METABOLISM
NADP+
K01242...
K18153...
K01242...
PGTG_00094...
K03462
3.2.2.14
K00322
K00323...
3.1.3.-
PGTG_01587
N1-Methyl-2-pyridone-5-carboxamide
K00157
K00157
N1-Methyl-4-pyridone-5-carboxamide
N1-Methyl-nicotinamide
K00541
Nicotinamide
PGTG_02143...
N-Ribosyl-nicotinamide
PGTG_00818
Nicotinamide D-ribonucleotide
PGTG_06073
PGTG_06073
NAD+
K01239
K01240
K01081...
PGTG_12485
K01513...
PGTG_03648
K01916
PGTG_00406
K01440...
K03742...
Tropane, piperidine and pyridine alkaloid biosynthesis
PGTG_13345
1-Methyl pyrrolinium
Nicotine
NS
Nicotinate
Nicotinate D-ribonucleoside
PGTG_02143...
PGTG_00818
PGTG_06073
PGTG_06073
Nicotinate D-ribonucleotide
Deamino-NAD+
K01240
K01081...
PGTG_12485
K01513...
PGTG_03648
PGTG_18374
Nicotinurate
Pseudooxy-nicotine
K20170...
K20158
3-Succinoyl-semialdehyde-pyridine
N-Methylmyosmine
K19818...
K19818...
K20170
K19700
K18029...
K20445...
6-oxo-1,4,5,6-Tetrahydro-nicotinate
2-Formyl-glutarate
Tryptophan metabolism
Quinolinate
(S)-6-Hydroxy-nicotine
(R)-6-Hydroxy-nicotine
2.1.1.7
K20449
K15358
K19826
K19890
3-Succinoylpyridine
6-Hydroxy-nicotinate
K03517
Trigonelline
K19647
Glycerone-P
6-Hydroxy-N-methylmyosmine
K20171...
K14974
Iminoaspartate
Methylitaconate
6-Hydroxy-pseudooxynicotine
6-Hydroxy-3-succinoylpyridine
K20450
(S)-2-Hydroxy-methylglutarate
2-Methylene-glutarate
K20451
K00278
K06989
K19185...
K15359...
2,5-Dihydroxy-pyridine
1.17.3.3
2,6-Dihydroxy-pseudooxynicotine
K20452...
(2R,3S)-2,3-Dimethylmalate
2,6-Dihydroxy-N-methylmyosmine
K19188
PGTG_17645
L-Aspartate
2,3-Dimethyl-maleate
K18028
K20454
K20155
K08324
Citrate cycle
PGTG_17645
Propanoate
4-Methyl-aminobutyrate
Succinate-semialdehyde
Succinate
Pyruvate
N-formyl maleamate
2,6-Dihydroxy-nicotinate
Pyruvate metabolism
K19191
Alanine metabolism
K15357
4-Amino-butanoate
C5-Branched dibasic acid metabolism
2,3,6-Trihydroxypyridine
K19189
K13995
K01799
Alanine, aspartate and glutamate metabolism
2,6-Dihydroxy-pyridine
Maleamate
Maleate
Fumarate
K19190
Blue pigment
3,3'-Bipyridine-2,2',5,5',6,6'-hexol
00760 9/21/16
(c) Kanehisa Laboratories

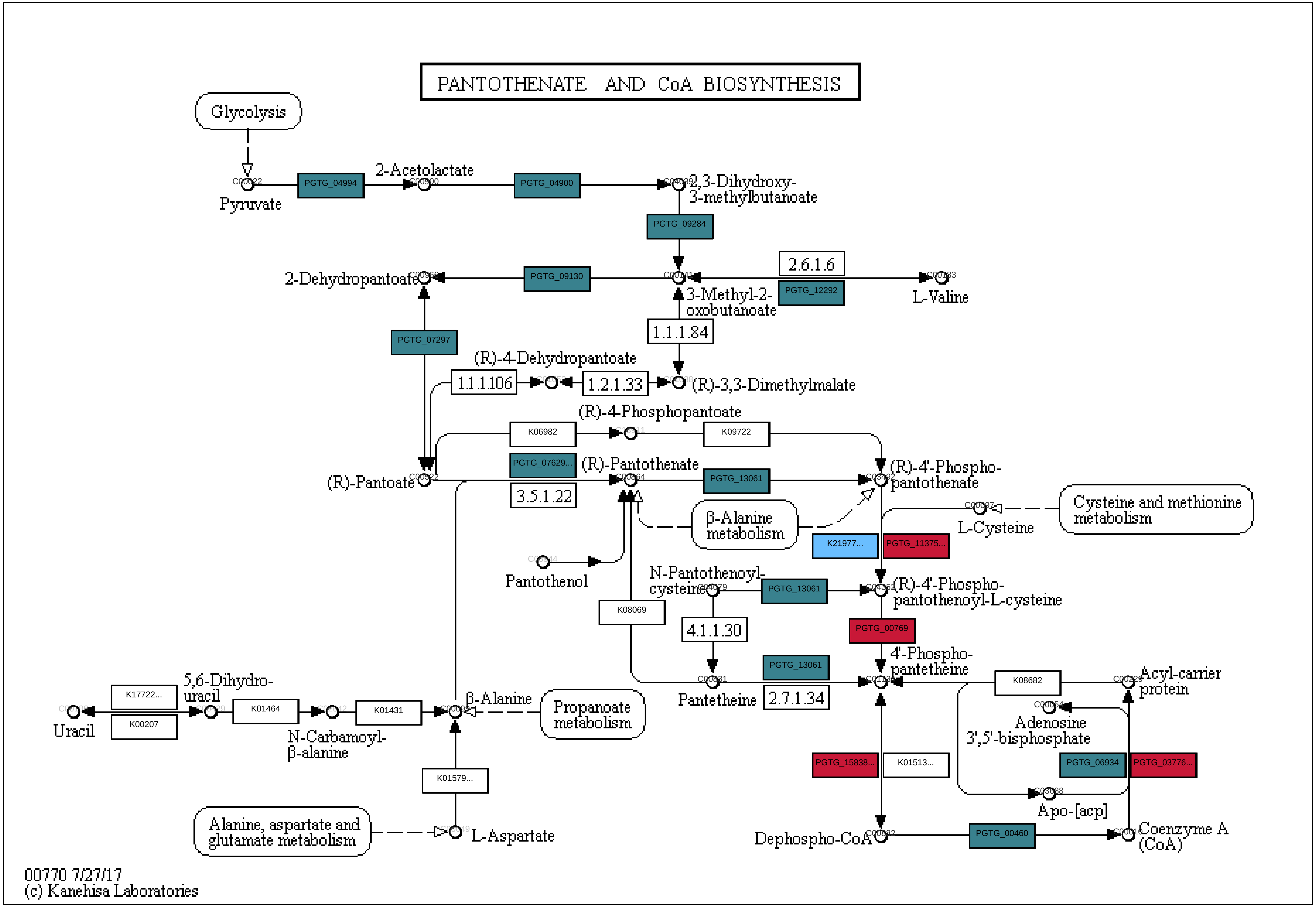
PANTOTHENATE AND CoA BIOSYNTHESIS
Glycolysis
Pyruvate
PGTG_04994
2-Acetolactate
PGTG_04900
2,3-Dihydroxy-
3-methylbutanoate
PGTG_09284
2.6.1.6
PGTG_12292
L-Valine
2-Dehydropantoate
PGTG_09130
3-Methyl-2-
oxobutanoate
1.1.1.84
PGTG_07297
(R)-4-Dehydropantoate
1.1.1.106
1.2.1.33
(R)-3,3-Dimethylmalate
(R)-4-Phosphopantoate
K06982
K09722
PGTG_07629...
(R)-Pantothenate
(R)-Pantoate
PGTG_13061
(R)-4'-Phospho-
pantothenate
3.5.1.22
β-Alanine
metabolism
Cysteine and methionine
metabolism
L-Cysteine
K21977...
PGTG_11375...
Pantothenol
N-Pantothenoyl-
cysteine
PGTG_13061
(R)-4'-Phospho-
pantothenoyl-L-cysteine
K08069
4.1.1.30
PGTG_00769
4'-Phospho-
pantetheine
PGTG_13061
Pantetheine
2.7.1.34
K08682
Acyl-carrier
protein
Adenosine
3',5'-bisphosphate
K17722...
K00207
Uracil
5,6-Dihydro-
uracil
K01464
N-Carbamoyl-
β-alanine
K01431
β-Alanine
Propanoate
metabolism
K01579...
PGTG_15838...
K01513...
PGTG_06934
PGTG_03776...
Apo-[acp]
Alanine, aspartate and
glutamate metabolism
L-Aspartate
Dephospho-CoA
PGTG_00460
Coenzyme A
(CoA)
00770 7/27/17
(c) Kanehisa Laboratories

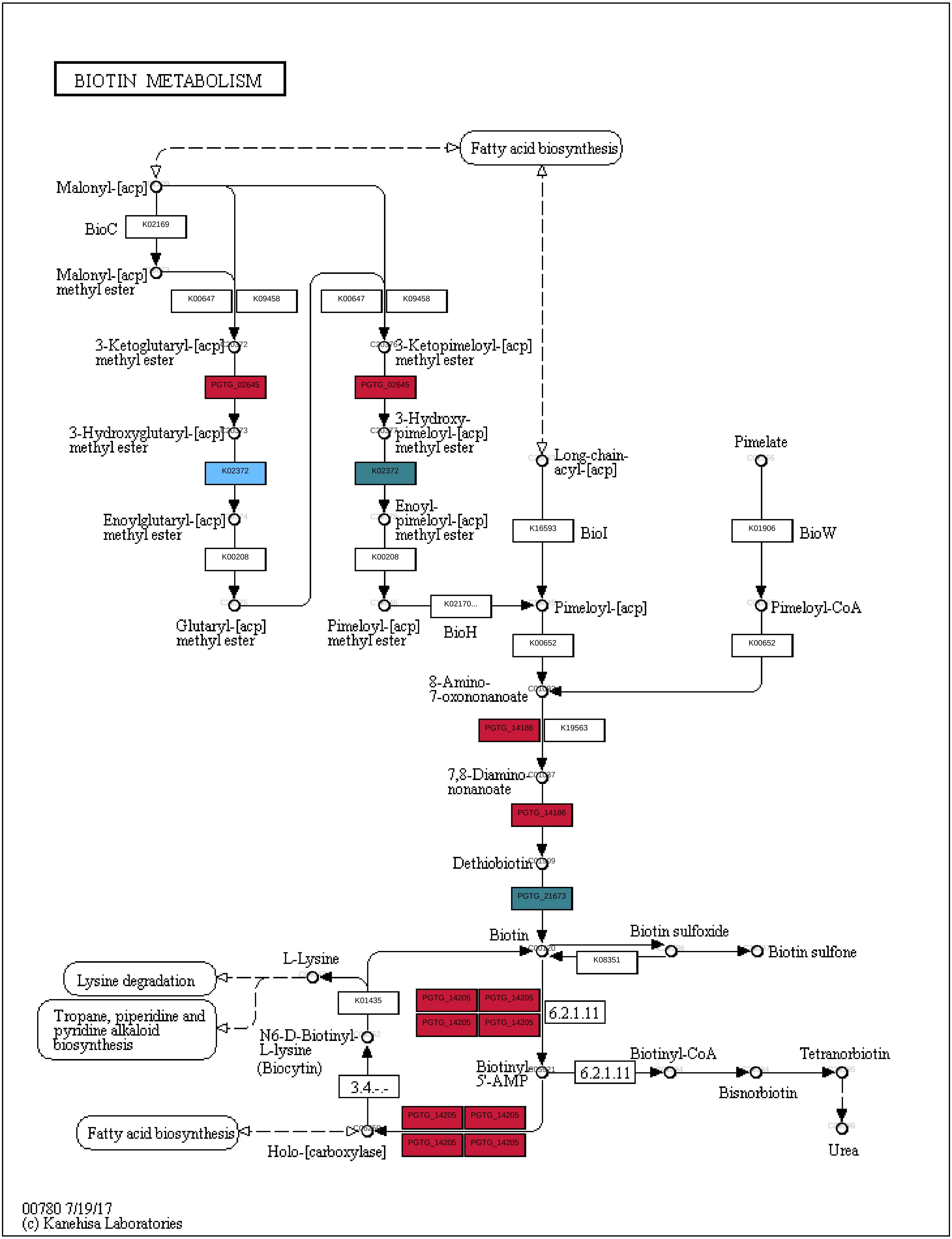

BIOTIN METABOLISM
Fatty acid biosynthesis
Malonyl-[acp]
BioC
K02169
Malonyl-[acp] methyl ester
K00647
K09458
K00647
K09458
3-Ketoglutaryl-[acp] methyl ester
C20372
C20376
3-Ketopimeloyl-[acp] methyl ester
PGTG_02645
PGTG_02645
3-Hydroxyglutaryl-[acp] methyl ester
C20373
3-Hydroxy-pimeloyl-[acp] methyl ester
K02372
K02372
Enoylglutaryl-[acp] methyl ester
Enoyl-pimeloyl-[acp] methyl ester
K00208
K00208
Glutaryl-[acp] methyl ester
Pimeloyl-[acp] methyl ester
K02170...
BioH
Long-chain-acyl-[acp]
K16593
BioI
Pimelate
K01906
BioW
Pimeloyl-[acp]
Pimeloyl-CoA
K00652
K00652
8-Amino-7-oxononanoate
PGTG_14186
K19563
7,8-Diamino-nonanoate
PGTG_14186
Dethiobiotin
PGTG_21673
Biotin
Biotin sulfoxide
Biotin sulfone
K08351
L-Lysine
Lysine degradation
Tropane, piperidine and pyridine alkaloid biosynthesis
K01435
PGTG_14205
PGTG_14205
PGTG_14205
PGTG_14205
6.2.1.11
N6-D-Biotinyl-L-lysine (Biocytin)
3.4.-.-
Biotinyl-5'-AMP
6.2.1.11
Biotinyl-CoA
Tetranorbiotin
Bisnorbiotin
Urea
PGTG_14205
PGTG_14205
PGTG_14205
PGTG_14205
Fatty acid biosynthesis
Holo-[carboxylase]
00780 7/19/17
(c) Kanehisa Laboratories

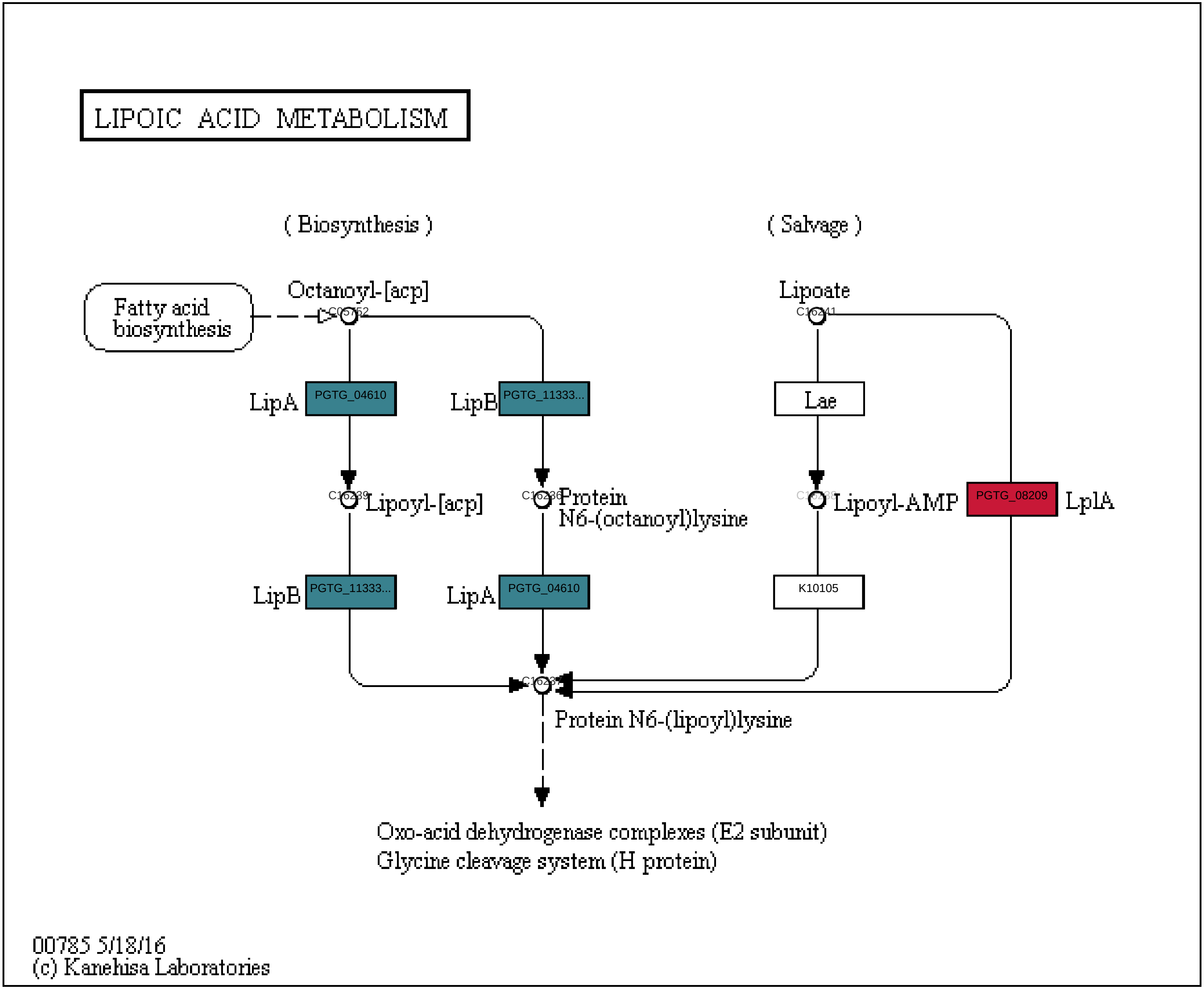

LIPOIC ACID METABOLISM
( Biosynthesis )
( Salvage )
Fatty acid biosynthesis
Octanoyl-[acp]
Lipoate
LipA
PGTG_04610
LipB
PGTG_11333...
Lae
Lipoyl-[acp]
Protein N6-(octanoyl)lysine
Lipoyl-AMP
PGTG_08209
LplA
LipB
PGTG_11333...
LipA
PGTG_04610
K10105
Protein N6-(lipoyl)lysine
Oxo-acid dehydrogenase complexes (E2 subunit)
Glycine cleavage system (H protein)
00785 5/18/16
(c) Kanehisa Laboratories

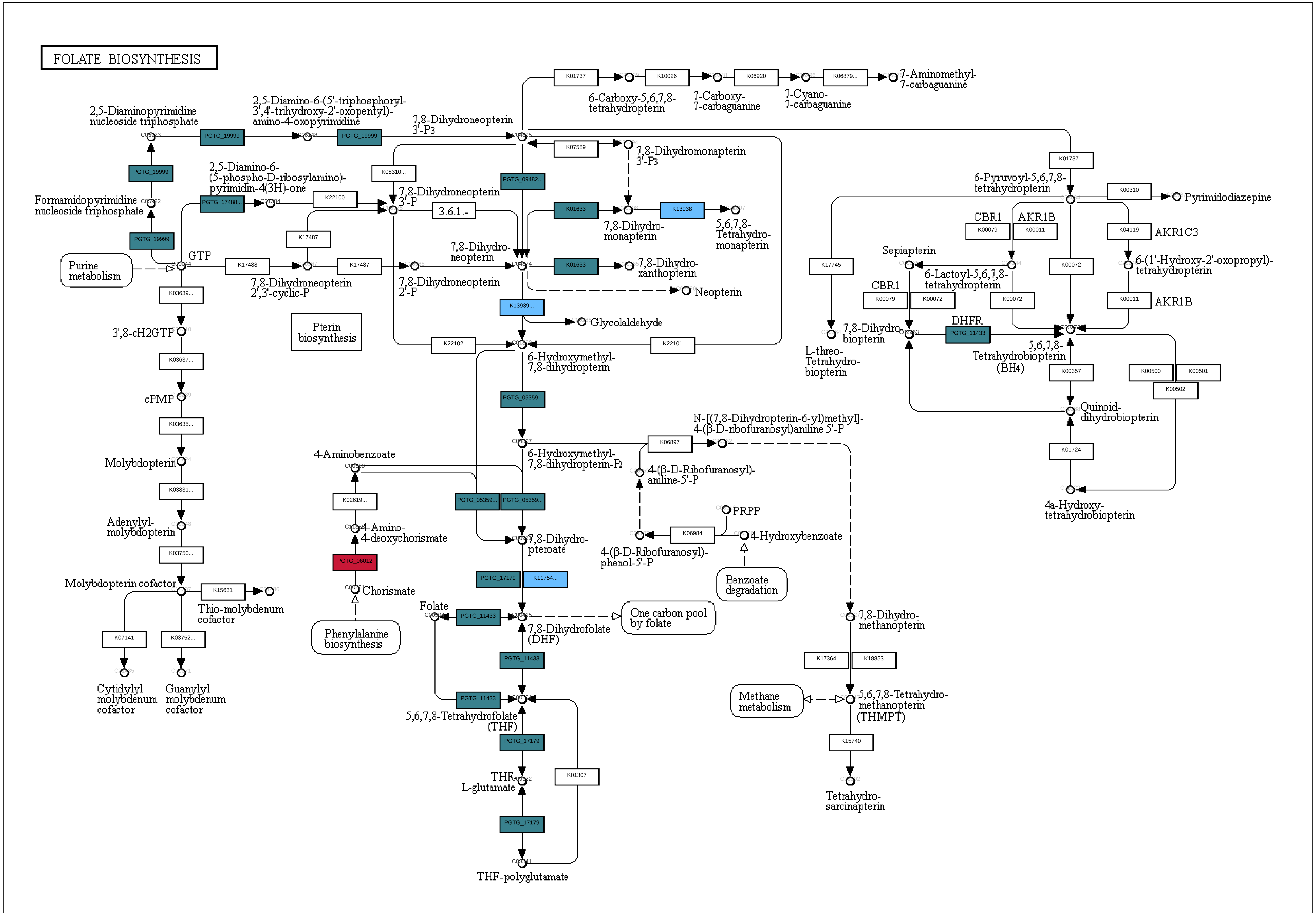

00790 9/5/17
(c) Kanehisa Laboratories

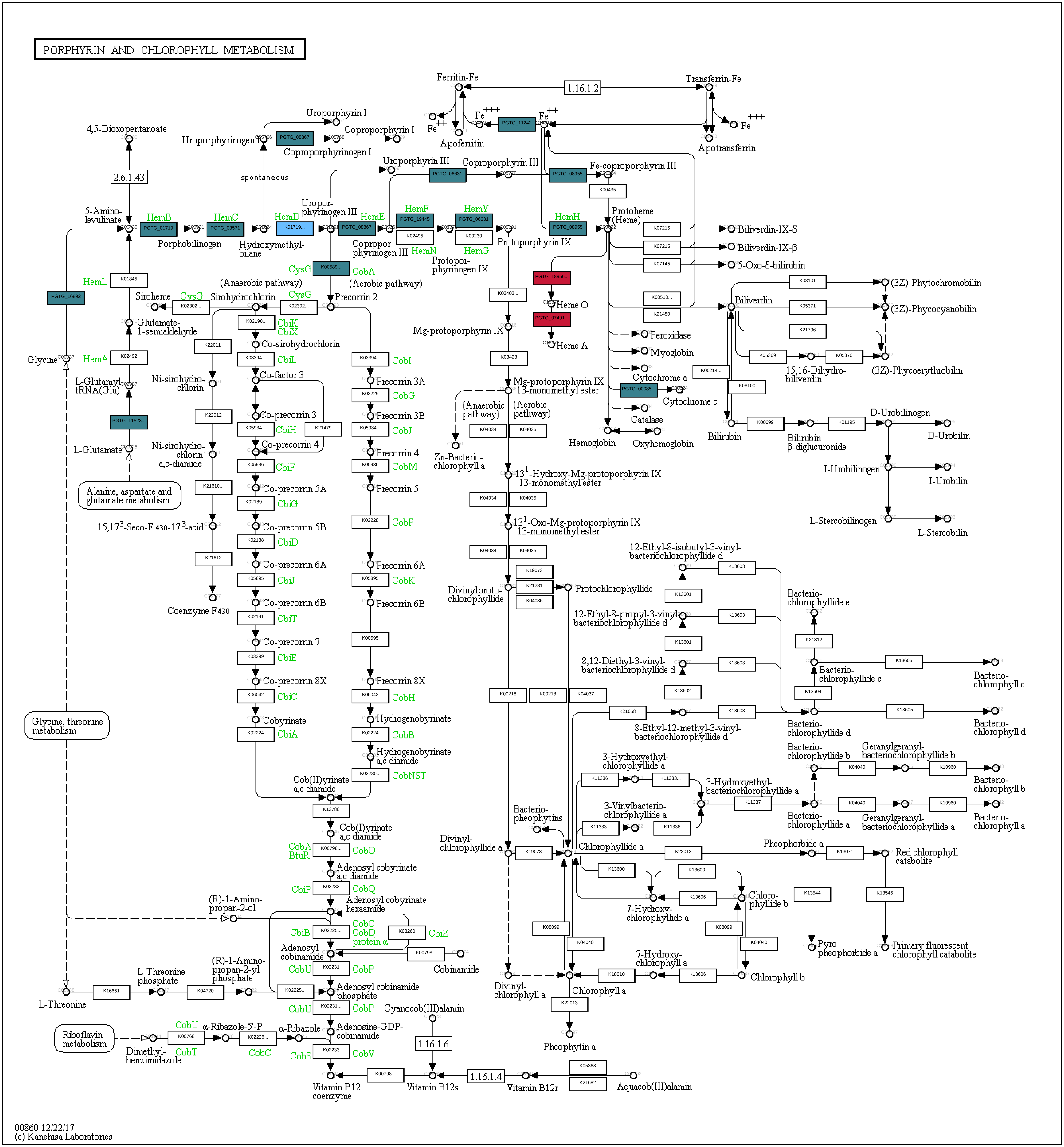
PORPHYRIN AND CHLOROPHYLL METABOLISM
Ferritin-Fe
1.16.1.2
Transferrin-Fe
Fe++
Fe+++
Apoferritin
Apotransferrin
PGTG_11242
Uroporphyrin I
Coproporphyrin I
Uroporphyrinogen I
PGTG_08867
Coproporphyrinogen I
4,5-Dioxopentanoate
2.6.1.43
spontaneous
Uroporphyrin III
Coproporphyrin III
PGTG_06631
PGTG_08955
Fe-coproporphyrin III
K00435
Protoheme (Heme)
5-Amino-levulinate
HemB
HemC
HemD
HemE
HemF
HemY
HemH
PGTG_01719
PGTG_08571
K01719...
PGTG_19445
K02495
K00230
Porphobilinogen
Hydroxymethyl-bilane
Uropor-phyrinogen III
Copropor-phyrinogen III
HemN
HemG
Protopor-phyrinogen IX
Protoporphyrin IX
K07215
Biliverdin-IX-δ
Biliverdin-IX-β
K07145
5-Oxo-δ-bilirubin
CysG
PGTG_00589...
CobA
(Anaerobic pathway)
(Aerobic pathway)
HemL
K01845
PGTG_16892
Siroheme
Sirohydrochlorin
Precorrin 2
K02302...
PGTG_18956...
Heme O
PGTG_07481...
Heme A
K03403...
Mg-protoporphyrin IX
K00510...
K21480
Biliverdin
K08101
(3Z)-Phytochromobilin
K05371
(3Z)-Phycocyanobilin
K21796
Glutamate-1-semialdehyde
K02190...
CbiK
CbiX
Co-sirohydrochlorin
K22011
Glycine
HemA
K02492
K03394...
CbiL
CobI
Peroxidase
Myoglobin
K05369
K05370
15,16-Dihydro-biliverdin
(3Z)-Phycoerythrobilin
K03428
Ni-sirohydro-chlorin
Co-factor 3
Precorrin 3A
K02229
CobG
K00214...
Cytochrome a
PGTG_00085
Cytochrome c
K08100
L-Glutamyl-tRNA(Glu)
PGTG_11523...
Mg-protoporphyrin IX 13-monomethyl ester
(Anaerobic pathway)
(Aerobic pathway)
K04034
K04035
Catalase
K22012
Co-precorrin 3
K05934...
CbiH
K21479
Precorrin 3B
CobJ
Hemoglobin
Oxyhemoglobin
Bilirubin
K00699
K01195
D-Urobilinogen
Bilirubin β-diglucuronide
D-Urobilin
L-Glutamate
Ni-sirohydro-chlorin a,c-diamide
Co-precorrin 4
Precorrin 4
Zn-Bacterio-chlorophyll a
K05936
CbiF
CobM
$13^1$-Hydroxy-Mg-protoporphyrin IX 13-monomethyl ester
I-Urobilinogen
I-Urobilin
Alanine, aspartate and glutamate metabolism
K21610...
Co-precorrin 5A
Precorrin 5
K02189...
CbiG
$13^1$-Oxo-Mg-protoporphyrin IX 13-monomethyl ester
L-Stercobilinogen
L-Stercobilin
15,17³-Seco-F430-17³-acid
Co-precorrin 5B
K02188
CbiD
K02228
CobF
K21612
Co-precorrin 6A
Precorrin 6A
K05895
CbiJ
CobK
12-Ethyl-8-isobutyl-3-vinyl-bacteriochlorophyllide d
K19073
K21231
K04036
K13603
Divinylproto-chlorophyllide
Protochlorophyllide
Coenzyme F430
Co-precorrin 6B
Precorrin 6B
K13601
Bacterio-chlorophyllide e
K02191
CbiT
12-Ethyl-8-propyl-3-vinyl-bacteriochlorophyllide d
K00595
Co-precorrin 7
K03399
CbiE
K21312
8,12-Diethyl-3-vinyl-bacteriochlorophyllide d
K13605
Bacterio-chlorophyllide c
Bacterio-chlorophyll c
Co-precorrin 8X
Precorrin 8X
K06042
CbiC
CobH
K13602
K13604
K00218
K04037...
K21058
Glycine, threonine metabolism
Cobyrinate
Hydrogenobyrinate
Bacterio-chlorophyllide d
Bacterio-chlorophyll d
K02224
CbiA
CobB
8-Ethyl-12-methyl-3-vinyl-bacteriochlorophyllide d
Hydrogenobyrinate a,c diamide
3-Hydroxyethyl-chlorophyllide a
Bacterio-chlorophyllide b
Geranylgeranyl-bacteriochlorophyllide b
K02230...
CobNST
K11336
K11333...
K04040
K10960
Bacterio-chlorophyll b
Cob(II)yrinate a,c diamide
3-Hydroxyethyl-bacteriochlorophyllide a
K13786
K11337
Bacterio-pheophytins
3-Vinylbacterio-chlorophyllide a
Bacterio-chlorophyllide a
Geranylgeranyl-bacteriochlorophyllide a
Bacterio-chlorophyll a
Cob(I)yrinate a,c diamide
Divinyl-chlorophyllide a
Chlorophyllide a
Pheophorbide a
K22013
K13071
Red chlorophyll catabolite
CobA
BtuR
K00798...
CobO
Adenosyl cobyrinate a,c diamide
K13600
CbiP
K02232
CobQ
K13606
Chloro-phyllide b
K13544
K13545
(R)-1-Amino-propan-2-ol
Adenosyl cobyrinate hexaamide
7-Hydroxy-chlorophyllide a
CbiB
K02225...
CobC
CobD
protein α
K08260
CbiZ
K08099
Adenosyl cobinamide
Cobinamide
Pyro-pheophorbide a
Primary fluorescent chlorophyll catabolite
7-Hydroxy-chlorophyll a
K02231
CobU
CobP
K18010
L-Threonine phosphate
(R)-1-Amino-propan-2-yl phosphate
K16651
K04720
Adenosyl cobinamide phosphate
Divinyl-chlorophyll a
Chlorophyll a
Chlorophyll b
L-Threonine
K02231...
Cyanocob(III)alamin
Riboflavin metabolism
CobU
α-Ribazole-5'-P
α-Ribazole
Adenosine-GDP-cobinamide
K00768
K02226...
Dimethyl-benzimidazole
CobT
CobC
CobS
K02233
CobV
1.16.1.6
Pheophytin a
K05368
K21682
1.16.1.4
Vitamin B12 coenzyme
Vitamin B12s
Vitamin B12r
Aquacob(III)alamin
00860 12/22/17
(c) Kanehisa Laboratories

# 9. Metabolism of terpenoids and polyketides

| MAP | PATHWAY |
|---|---|
| **900** | Terpenoid backbone biosynthesis |
| **909** | Sesquiterpenoid and triterpenoid biosynthesis |

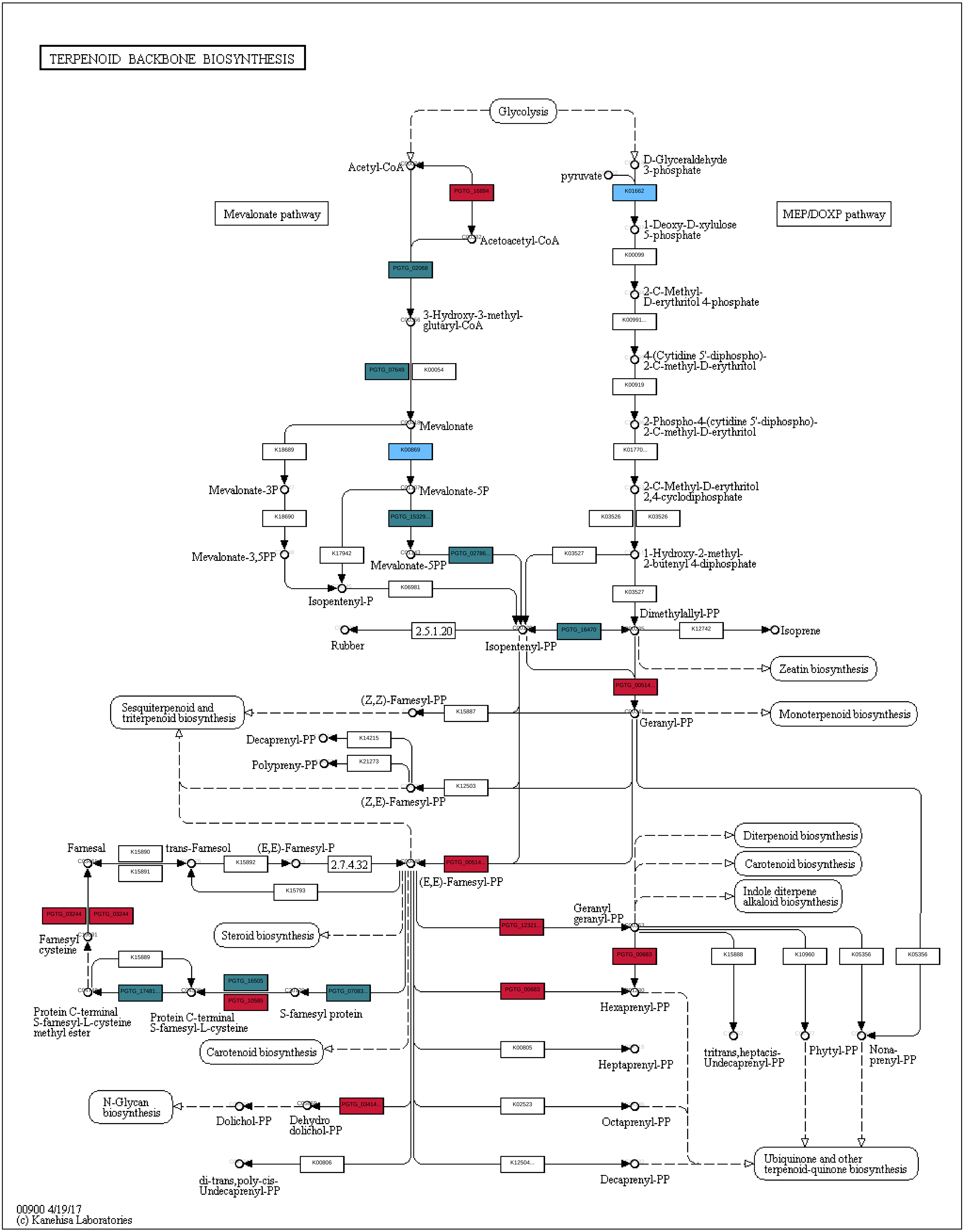

TERPENOID BACKBONE BIOSYNTHESIS
Glycolysis
Acetyl-CoA
PGTG_16894
D-Glyceraldehyde 3-phosphate
pyruvate
K01662
Mevalonate pathway
MEP/DOXP pathway
Acetoacetyl-CoA
1-Deoxy-D-xylulose 5-phosphate
PGTG_02068
K00099
2-C-Methyl-D-erythritol 4-phosphate
3-Hydroxy-3-methyl-glutaryl-CoA
K00991...
PGTG_07649
K00054
4-(Cytidine 5'-diphospho)-2-C-methyl-D-erythritol
K00919
Mevalonate
2-Phospho-4-(cytidine 5'-diphospho)-2-C-methyl-D-erythritol
K18689
K00869
K01770...
Mevalonate-3P
Mevalonate-5P
2-C-Methyl-D-erythritol 2,4-cyclodiphosphate
K18690
PGTG_15329...
K03526
K03526
Mevalonate-3,5PP
K17942
PGTG_02786...
K03527
1-Hydroxy-2-methyl-2-butenyl 4-diphosphate
Mevalonate-5PP
K06981
K03527
Isopentenyl-P
Dimethylallyl-PP
2.5.1.20
PGTG_16470
K12742
Isoprene
Rubber
Isopentenyl-PP
Zeatin biosynthesis
PGTG_00514...
(Z,Z)-Farnesyl-PP
Sesquiterpenoid and triterpenoid biosynthesis
K15887
Monoterpenoid biosynthesis
Geranyl-PP
Decaprenyl-PP
K14215
Polypreny-PP
K21273
K12503
(Z,E)-Farnesyl-PP
Diterpenoid biosynthesis
Farnesal
K15890
trans-Farnesol
(E,E)-Farnesyl-P
Carotenoid biosynthesis
K15891
K15892
2.7.4.32
PGTG_00514...
(E,E)-Farnesyl-PP
K15793
Indole diterpene alkaloid biosynthesis
PGTG_03244
PGTG_03244
Geranyl geranyl-PP
Farnesyl cysteine
Steroid biosynthesis
PGTG_12321...
K15889
PGTG_00683
K15888
K10960
K05356
K05356
PGTG_16505
PGTG_17481...
PGTG_07083...
PGTG_00683
PGTG_10585
Hexaprenyl-PP
Protein C-terminal S-farnesyl-L-cysteine methyl ester
Protein C-terminal S-farnesyl-L-cysteine
S-farnesyl protein
K00805
tritrans,heptacis-Undecaprenyl-PP
Phytyl-PP
Nona-prenyl-PP
Carotenoid biosynthesis
Heptaprenyl-PP
N-Glycan biosynthesis
PGTG_03414...
K02523
Dolichol-PP
Dehydro dolichol-PP
Octaprenyl-PP
Ubiquinone and other terpenoid-quinone biosynthesis
K00806
K12504...
di-trans,poly-cis-Undecaprenyl-PP
Decaprenyl-PP
00900 4/19/17
(c) Kanehisa Laboratories

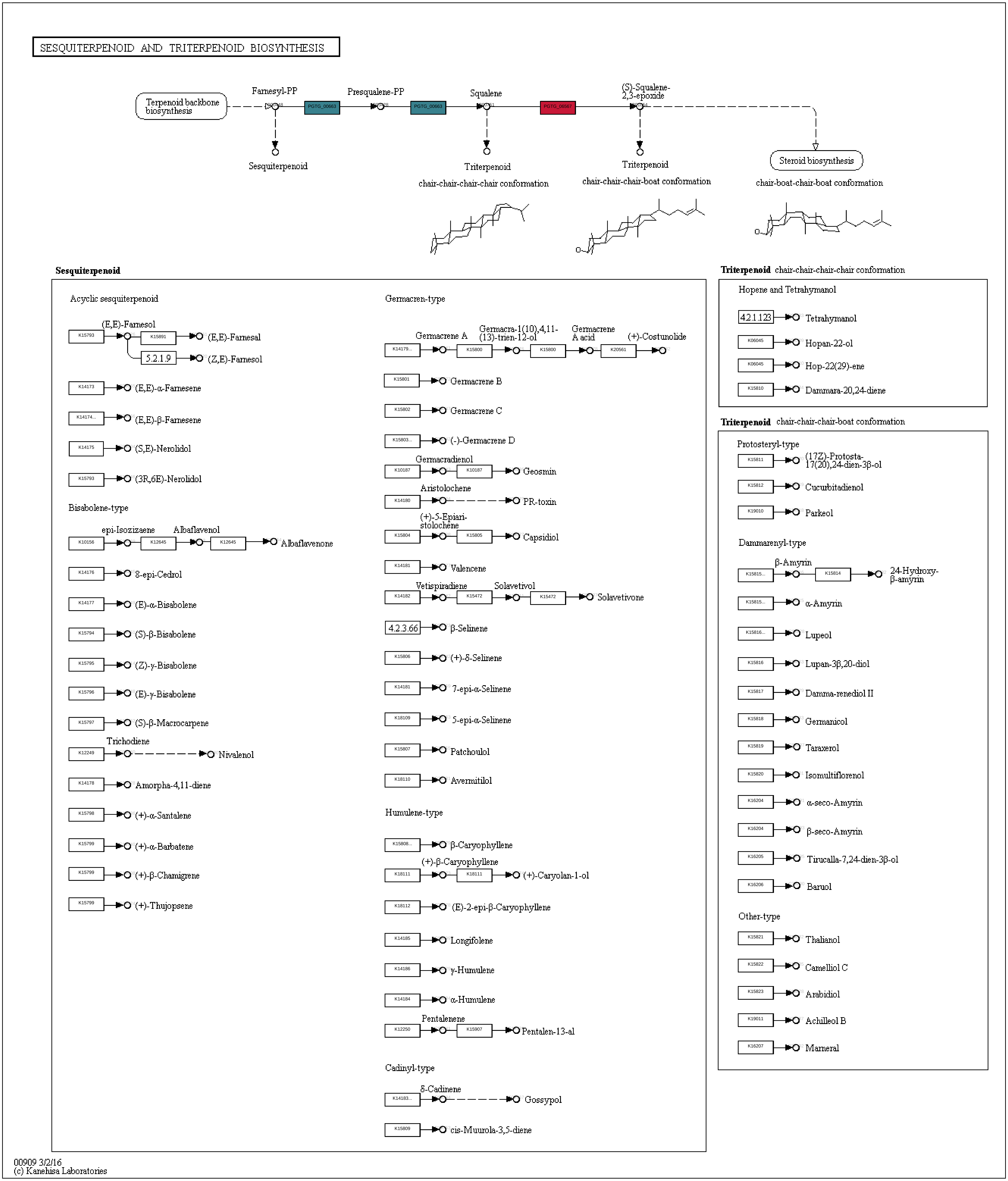
SESQUITERPENOID AND TRITERPENOID BIOSYNTHESIS
Terpenoid backbone biosynthesis
Farnesyl-PP
PGTG_00663
Presqualene-PP
PGTG_00663
Squalene
PGTG_06567
(S)-Squalene-2,3-epoxide
Sesquiterpenoid
Triterpenoid
chair-chair-chair-chair conformation
Triterpenoid
chair-chair-chair-boat conformation
Steroid biosynthesis
chair-boat-chair-boat conformation
Sesquiterpenoid
Acyclic sesquiterpenoid
(E,E)-Farnesol
(E,E)-Farnesal
5.2.1.9
(Z,E)-Farnesol
(E,E)-α-Farnesene
(E,E)-β-Farnesene
(S,E)-Nerolidol
(3R,6E)-Nerolidol
Bisabolene-type
epi-Isozizaene
Albaflavenol
Albaflavenone
8-epi-Cedrol
(E)-α-Bisabolene
(S)-β-Bisabolene
(Z)-γ-Bisabolene
(E)-γ-Bisabolene
(S)-β-Macrocarpene
Trichodiene
Nivalenol
Amorpha-4,11-diene
(+)-α-Santalene
(+)-α-Barbatene
(+)-β-Chamigrene
(+)-Thujopsene
Germacren-type
Germacrene A
Germacra-1(10),4,11-(13)-trien-12-ol
Germacrene A acid
(+)-Costunolide
Germacrene B
Germacrene C
(-)-Germacrene D
Germacradienol
Geosmin
Aristolochene
PR-toxin
(+)-5-Epiari-stolochene
Capsidiol
Valencene
Vetispiradiene
Solavetivol
Solavetivone
4.2.3.66
β-Selinene
(+)-δ-Selinene
7-epi-α-Selinene
5-epi-α-Selinene
Patchoulol
Avermitilol
Humulene-type
β-Caryophyllene
(+)-β-Caryophyllene
(+)-Caryolan-1-ol
(E)-2-epi-β-Caryophyllene
Longifolene
γ-Humulene
α-Humulene
Pentalenene
Pentalen-13-al
Cadinyl-type
δ-Cadinene
Gossypol
cis-Muurola-3,5-diene
Triterpenoid chair-chair-chair-chair conformation
Hopene and Tetrahymanol
4.2.1.123
Tetrahymanol
Hopan-22-ol
Hop-22(29)-ene
Dammara-20,24-diene
Triterpenoid chair-chair-chair-boat conformation
Protosteryl-type
(17Z)-Protosta-17(20),24-dien-3β-ol
Cucurbitadienol
Parkeol
Dammarenyl-type
β-Amyrin
24-Hydroxy-β-amyrin
α-Amyrin
Lupeol
Lupan-3β,20-diol
Damma-renediol II
Germanicol
Taraxerol
Isomultiflorenol
α-seco-Amyrin
β-seco-Amyrin
Tirucalla-7,24-dien-3β-ol
Baruol
Other-type
Thalianol
Camelliol C
Arabidiol
Achilleol B
Marneral
00909 3/2/16
(c) Kanehisa Laboratories

# 10. Biosynthesis of other secondary metabolites

| MAP | PATHWAY |
|---|---|
| **261** | Monobactam biosynthesis |
| **332** | Carbapenem biosynthesis |

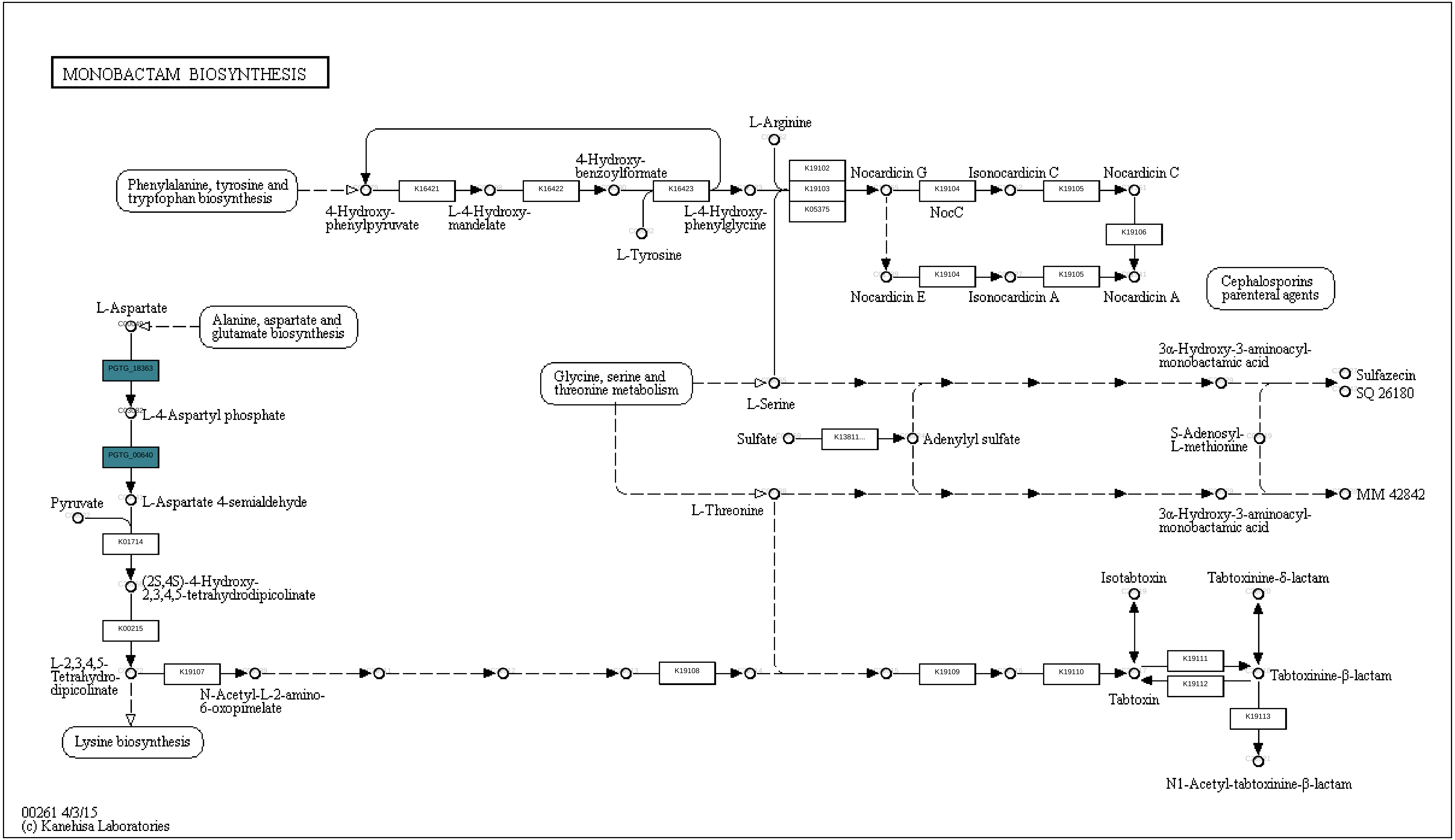
MONOBACTAM BIOSYNTHESIS
Phenylalanine, tyrosine and tryptophan biosynthesis
4-Hydroxy-phenylpyruvate
K16421
L-4-Hydroxy-mandelate
K16422
4-Hydroxy-benzoylformate
K16423
L-Tyrosine
L-4-Hydroxy-phenylglycine
L-Arginine
K19102
K19103
K05375
Nocardicin G
K19104
NocC
Isonocardicin C
K19105
Nocardicin C
K19106
Nocardicin E
K19104
Isonocardicin A
K19105
Nocardicin A
Cephalosporins parenteral agents
L-Aspartate
Alanine, aspartate and glutamate biosynthesis
PGTG_18363
L-4-Aspartyl phosphate
PGTG_00640
L-Aspartate 4-semialdehyde
Pyruvate
K01714
(2S,4S)-4-Hydroxy-2,3,4,5-tetrahydrodipicolinate
K00215
L-2,3,4,5-Tetrahydro-dipicolinate
K19107
N-Acetyl-L-2-amino-6-oxopimelate
Lysine biosynthesis
Glycine, serine and threonine metabolism
L-Serine
Sulfate
K13811...
Adenylyl sulfate
L-Threonine
3α-Hydroxy-3-aminoacyl-monobactamic acid
Sulfazecin
SQ 26180
S-Adenosyl-L-methionine
MM 42842
3α-Hydroxy-3-aminoacyl-monobactamic acid
K19108
K19109
K19110
Isotabtoxin
Tabtoxin
Tabtoxinine-δ-lactam
K19111
K19112
Tabtoxinine-β-lactam
K19113
N1-Acetyl-tabtoxinine-β-lactam
00261 4/3/15
(c) Kanehisa Laboratories

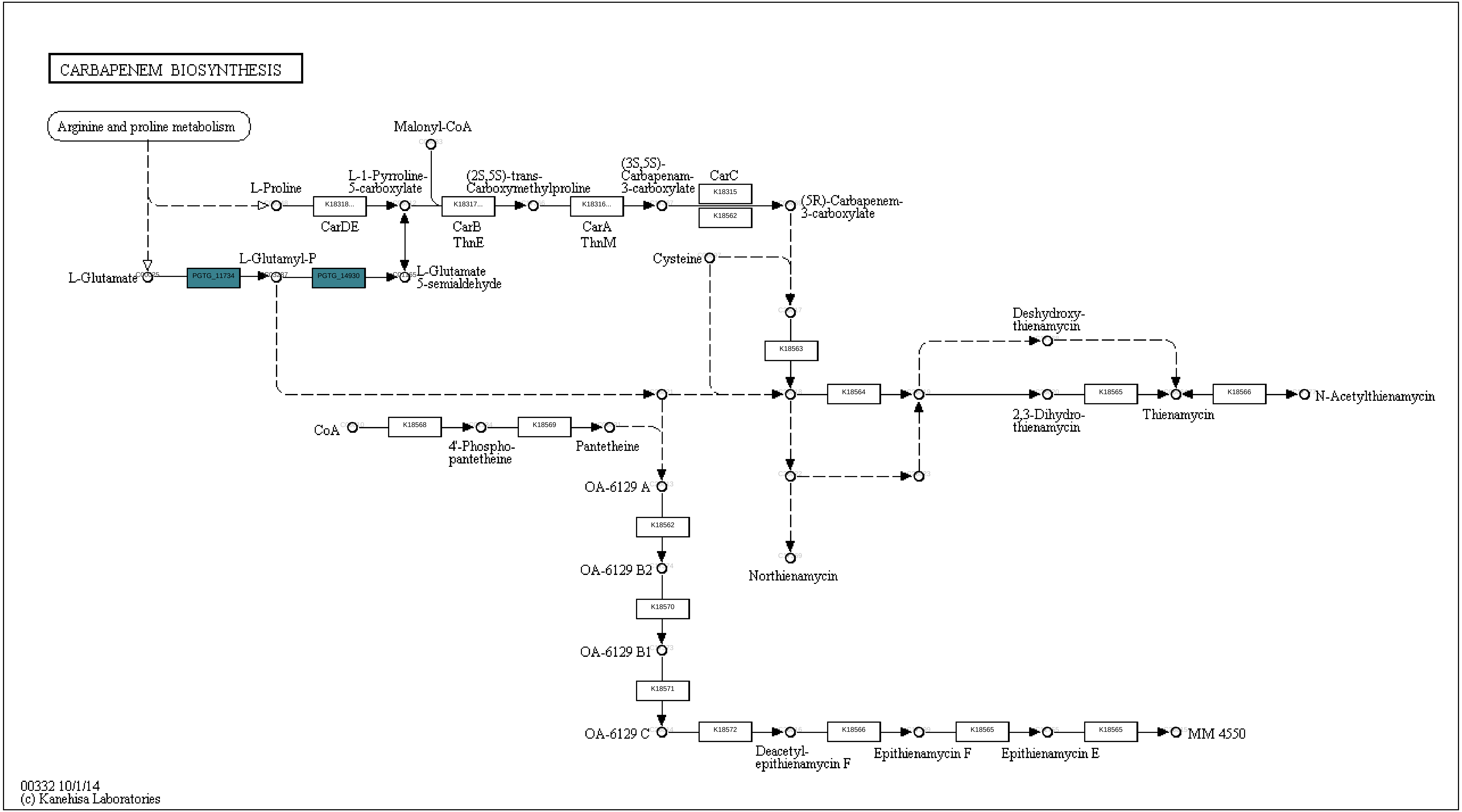
CARBAPENEM BIOSYNTHESIS
Arginine and proline metabolism
Malonyl-CoA
L-Proline
L-1-Pyrroline-
5-carboxylate
(2S,5S)-trans-
Carboxymethylproline
(3S,5S)-
Carbapenam-
3-carboxylate
CarC
K18315
K18562
(5R)-Carbapenem-
3-carboxylate
K18318...
CarDE
K18317...
CarB
ThnE
K18316...
CarA
ThnM
L-Glutamate
PGTG_11734
L-Glutamyl-P
PGTG_14930
L-Glutamate
5-semialdehyde
Cysteine
K18563
Deshydroxy-
thienamycin
K18564
K18565
K18566
N-Acetylthienamycin
2,3-Dihydro-
thienamycin
Thienamycin
CoA
K18568
4'-Phospho-
pantetheine
K18569
Pantetheine
OA-6129 A
K18562
OA-6129 B2
K18570
OA-6129 B1
K18571
OA-6129 C
K18572
Deacetyl-
epithienamycin F
K18566
Epithienamycin F
K18565
Epithienamycin E
K18565
MM 4550
Northienamycin
00332 10/1/14
(c) Kanehisa Laboratories

# 11. Xenobiotics biodegradation and metabolism

| MAP | PATHWAY |
|---|---|
| **791** | Atrazine degradation |

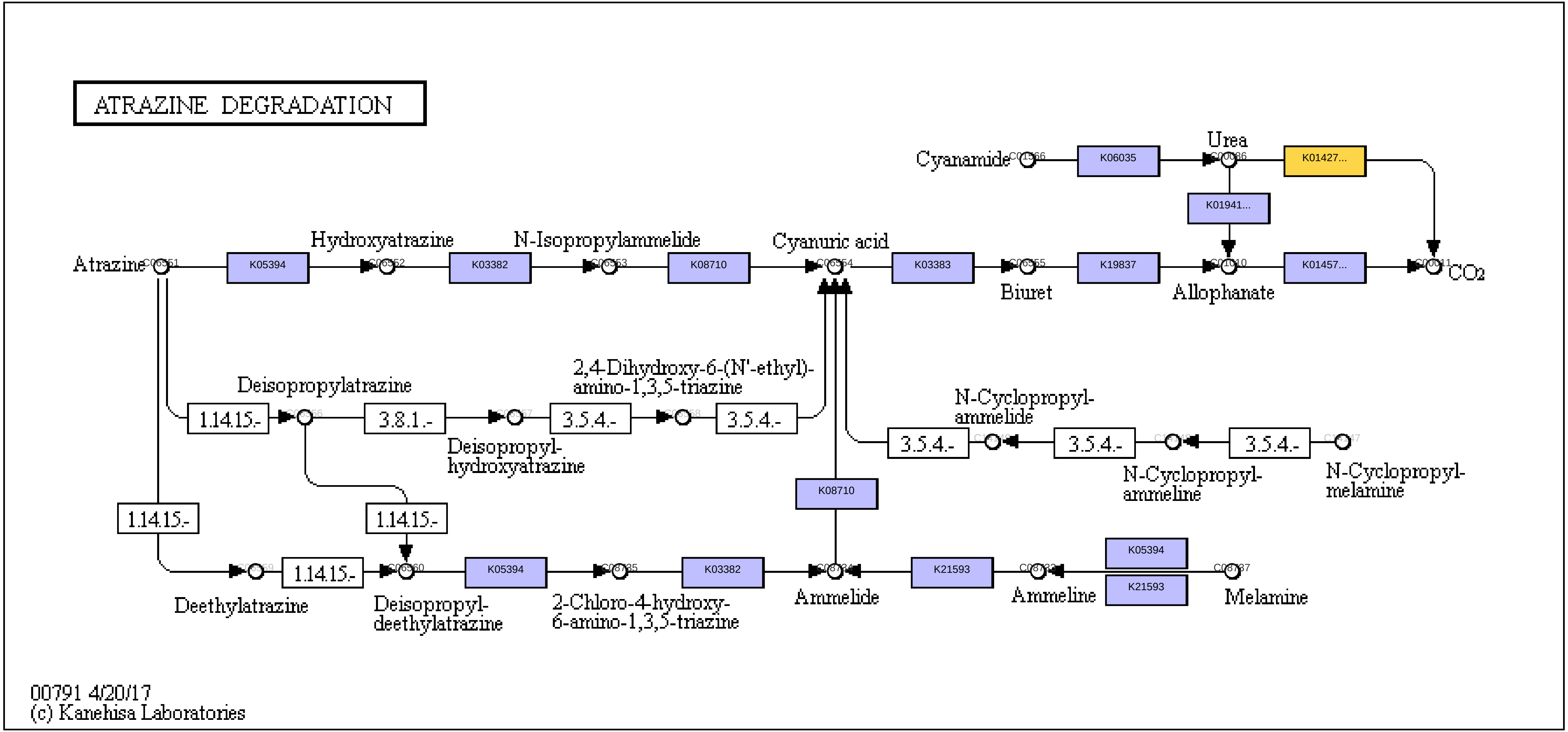
ATRAZINE DEGRADATION
Cyanamide
C01566
K06035
Urea
C00086
K01427...
K01941...
Atrazine
C06551
K05394
Hydroxyatrazine
C06552
K03382
N-Isopropylammelide
C06553
K08710
Cyanuric acid
C06554
K03383
C06555
Biuret
K19837
C01010
Allophanate
K01457...
C00011
$CO_2$
Deisopropylatrazine
1.14.15.-
3.8.1.-
2,4-Dihydroxy-6-(N'-ethyl)-amino-1,3,5-triazine
3.5.4.-
3.5.4.-
Deisopropyl-hydroxyatrazine
N-Cyclopropyl-ammelide
3.5.4.-
3.5.4.-
3.5.4.-
N-Cyclopropyl-ammeline
N-Cyclopropyl-melamine
K08710
1.14.15.-
1.14.15.-
1.14.15.-
K05394
C08735
K03382
C08734
K21593
C08733
K05394
K21593
C08737
Deethylatrazine
Deisopropyl-deethylatrazine
2-Chloro-4-hydroxy-6-amino-1,3,5-triazine
Ammelide
Ammeline
Melamine
00791 4/20/17
(c) Kanehisa Laboratories

**KEGG pathway analysis**

# *Positive controls*

**Supplementary file 4**

**The following color codes are used:**

- [teal] True positive (TP): element in the reference pathway correctly predicted by Interproscan
- [white] True negative (TN): element in the *not* reference pathway and *not* predicted by Interproscan
- [red] False negative (FN): element in the reference pathway but *not* predicted by Interproscan.
- [light blue] False positive (FP): element in the *not* reference pathway but predicted by Interproscan. Note: could also be a valid new prediction

**Species:** ***Melamspora larici-populina***

**Acronym:** **MLR**

**Lifestyle:** **Obligate biotrophic (rust)**

# 1. Carbohydrate metabolism

| MAP | PATHWAY |
|---|---|
| **10** | Glycolysis / Gluconeogenesis |
| **20** | Citrate cycle (TCA cycle) |
| **30** | Pentose phosphate pathway |
| **40** | Pentose and glucuronate interconversions |
| **51** | Fructose and mannose metabolism |
| **52** | Galactose metabolism |
| **53** | Ascorbate and aldarate metabolism |
| **500** | Starch and sucrose metabolism |
| **520** | Amino sugar and nucleotide sugar metabolism |
| **562** | Inositol phosphate metabolism |
| **620** | Pyruvate metabolism |
| **630** | Glyoxylate and dicarboxylate metabolism |
| **640** | Propanoate metabolism |
| **650** | Butanoate metabolism |
| **660** | C5-Branched dibasic acid metabolism |

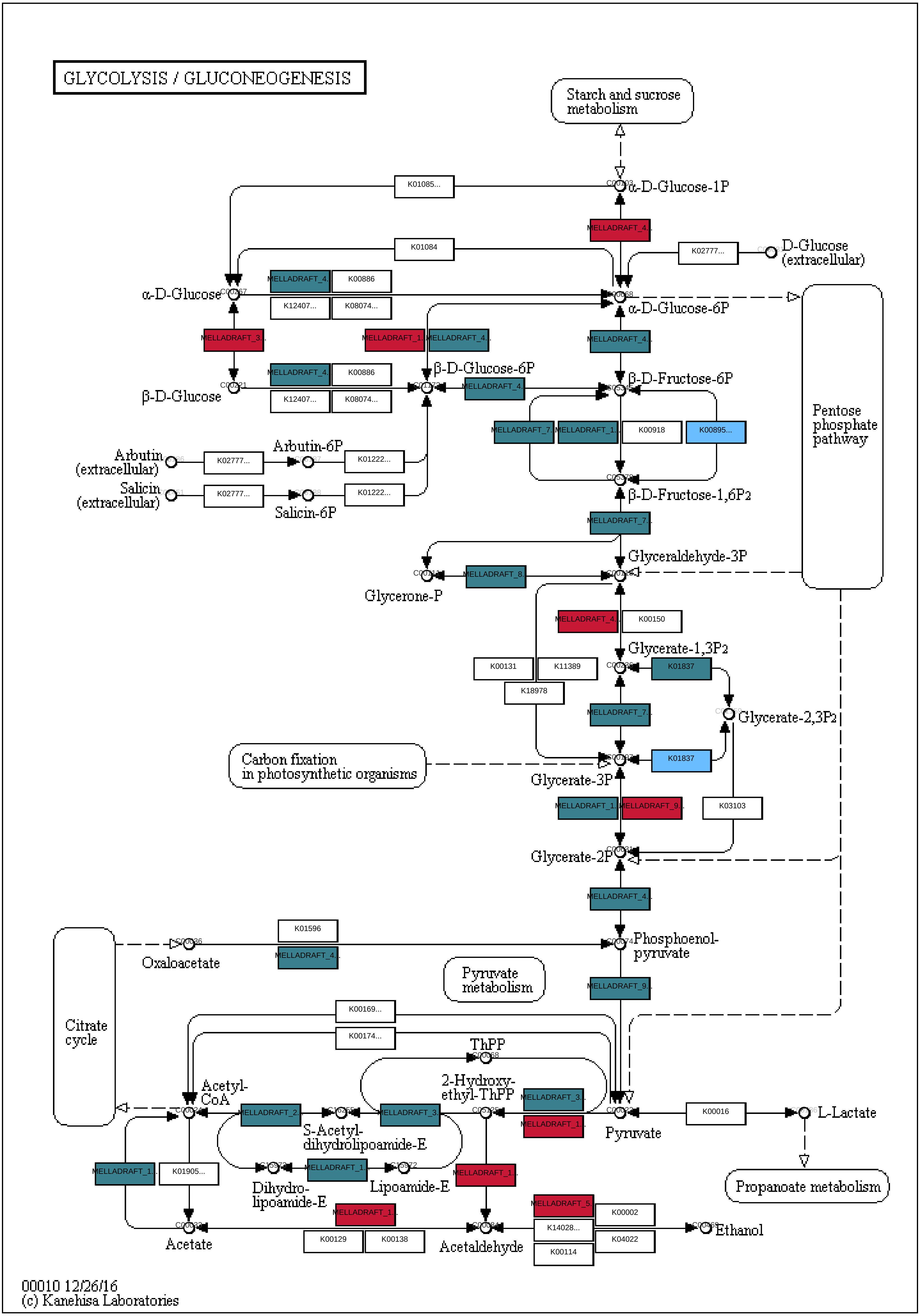
GLYCOLYSIS / GLUCONEOGENESIS
Starch and sucrose metabolism
α-D-Glucose-1P
K01085...
MELLADRAFT_4...
K01084
K02777...
D-Glucose (extracellular)
α-D-Glucose
MELLADRAFT_4...
K00886
K12407...
K08074...
α-D-Glucose-6P
MELLADRAFT_3...
MELLADRAFT_1...
MELLADRAFT_4...
β-D-Glucose-6P
β-D-Glucose
β-D-Fructose-6P
MELLADRAFT_7...
MELLADRAFT_1...
K00918
K00895...
Pentose phosphate pathway
Arbutin (extracellular)
Arbutin-6P
K01222...
Salicin (extracellular)
Salicin-6P
β-D-Fructose-1,6P2
Glyceraldehyde-3P
MELLADRAFT_8...
Glycerone-P
K00150
Glycerate-1,3P2
K00131
K11389
K18978
K01837
Glycerate-2,3P2
Carbon fixation in photosynthetic organisms
Glycerate-3P
MELLADRAFT_9...
K03103
Glycerate-2P
Phosphoenol-pyruvate
K01596
Oxaloacetate
Pyruvate metabolism
Citrate cycle
K00169...
K00174...
ThPP
2-Hydroxy-ethyl-ThPP
Acetyl-CoA
MELLADRAFT_2...
S-Acetyl-dihydrolipoamide-E
Pyruvate
K00016
L-Lactate
Propanoate metabolism
K01905...
Dihydro-lipoamide-E
Lipoamide-E
MELLADRAFT_5...
K00002
K14028...
K04022
K00114
Acetate
K00129
K00138
Acetaldehyde
Ethanol
00010 12/26/16
(c) Kanehisa Laboratories

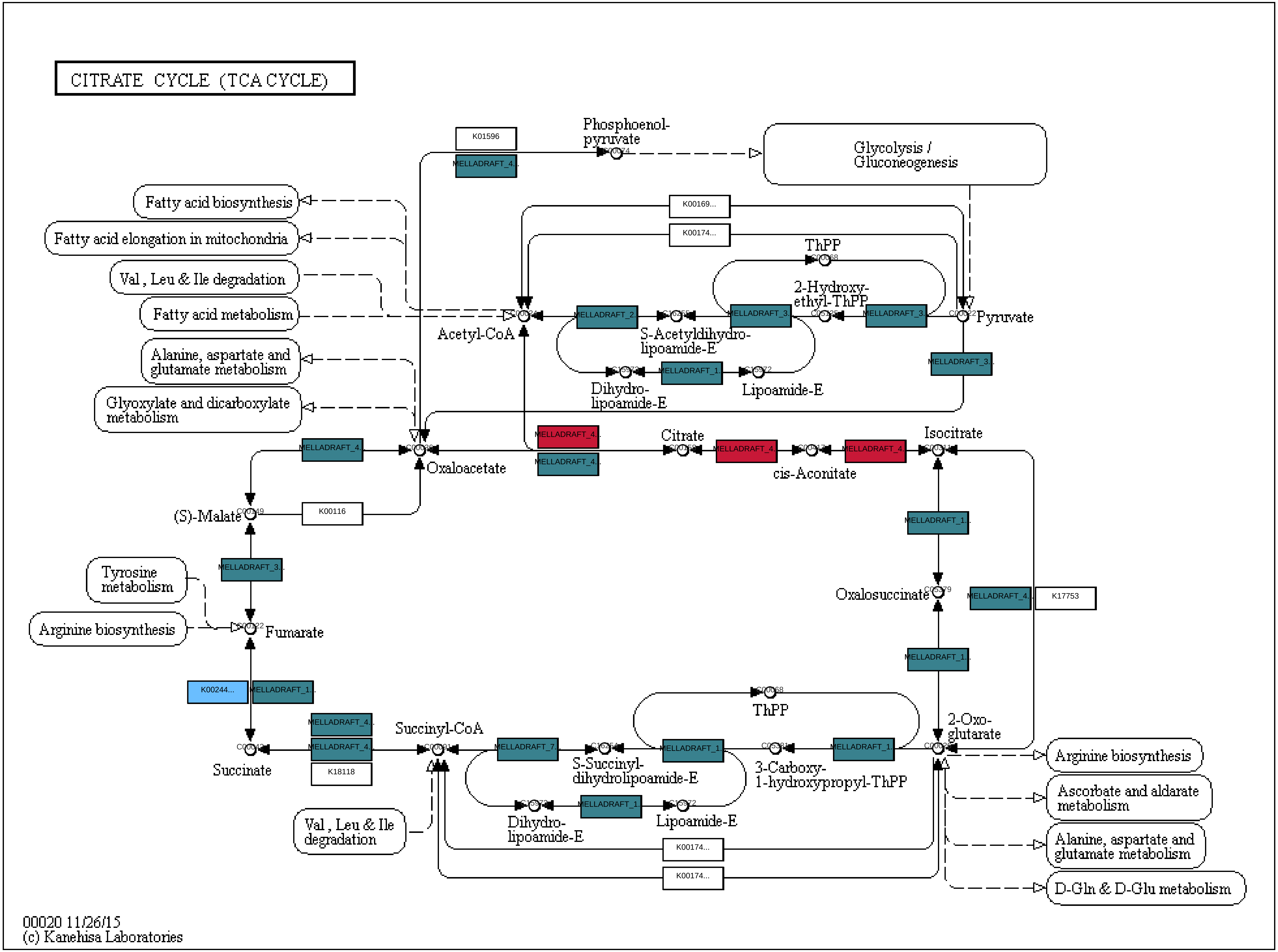
CITRATE CYCLE (TCA CYCLE)
Phosphoenol-pyruvate
K01596
MELLADRAFT_4...
Glycolysis / Gluconeogenesis
Fatty acid biosynthesis
Fatty acid elongation in mitochondria
Val, Leu & Ile degradation
Fatty acid metabolism
Alanine, aspartate and glutamate metabolism
Glyoxylate and dicarboxylate metabolism
K00169...
K00174...
ThPP
2-Hydroxy-ethyl-ThPP
Acetyl-CoA
S-Acetyldihydro-lipoamide-E
Pyruvate
Dihydro-lipoamide-E
Lipoamide-E
Citrate
Isocitrate
cis-Aconitate
Oxaloacetate
(S)-Malate
K00116
Tyrosine metabolism
Arginine biosynthesis
Fumarate
Oxalosuccinate
K17753
K00244...
Succinyl-CoA
ThPP
2-Oxo-glutarate
Succinate
K18118
S-Succinyl-dihydrolipoamide-E
3-Carboxy-1-hydroxypropyl-ThPP
Dihydro-lipoamide-E
Lipoamide-E
Val, Leu & Ile degradation
K00174...
K00174...
Arginine biosynthesis
Ascorbate and aldarate metabolism
Alanine, aspartate and glutamate metabolism
D-Gln & D-Glu metabolism
00020 11/26/15
(c) Kanehisa Laboratories

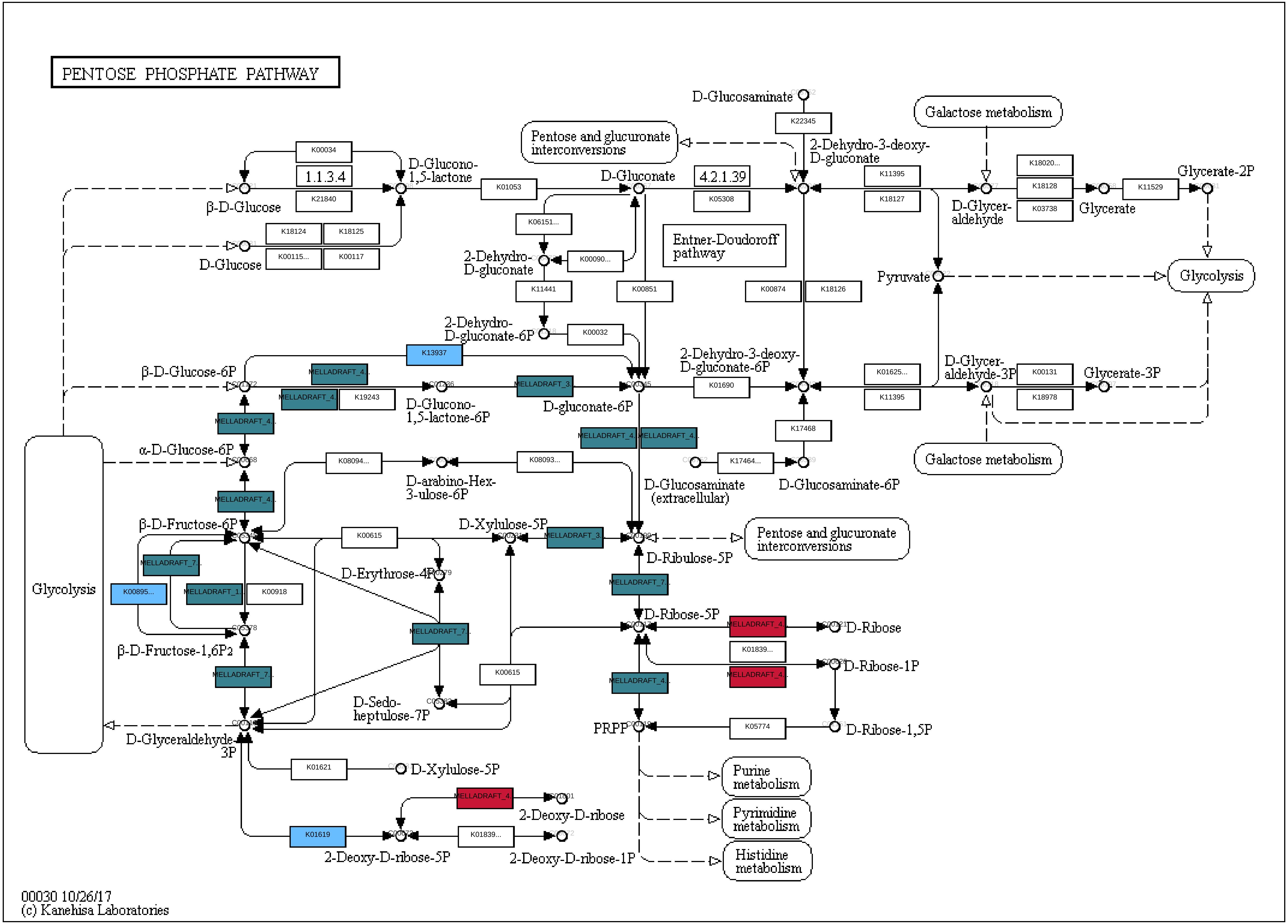
PENTOSE PHOSPHATE PATHWAY
D-Glucosaminate
K22345
Galactose metabolism
Pentose and glucuronate interconversions
2-Dehydro-3-deoxy-D-gluconate
K00034
D-Glucono-1,5-lactone
1.1.3.4
K01053
D-Gluconate
4.2.1.39
K11395
K18020...
Glycerate-2P
K21840
K05308
K18127
K18128
K11529
β-D-Glucose
K03738
D-Glyceraldehyde
Glycerate
K18124
K18125
K06151...
Entner-Doudoroff pathway
K00115...
K00117
2-Dehydro-D-gluconate
K00090...
D-Glucose
Pyruvate
Glycolysis
K11441
K00851
K00874
K18126
2-Dehydro-D-gluconate-6P
K00032
K13937
2-Dehydro-3-deoxy-D-gluconate-6P
β-D-Glucose-6P
MELLADRAFT_4..
K01625...
D-Glyceraldehyde-3P
K00131
Glycerate-3P
MELLADRAFT_3..
K01690
MELLADRAFT_4..
K19243
K11395
K18978
MELLADRAFT_4..
D-Glucono-1,5-lactone-6P
D-gluconate-6P
K17468
MELLADRAFT_4..
MELLADRAFT_4..
α-D-Glucose-6P
K08094...
K08093...
K17464...
D-arabino-Hex-3-ulose-6P
D-Glucosaminate (extracellular)
D-Glucosaminate-6P
MELLADRAFT_4..
β-D-Fructose-6P
D-Xylulose-5P
K00615
MELLADRAFT_3..
Pentose and glucuronate interconversions
MELLADRAFT_7..
D-Ribulose-5P
D-Erythrose-4P
Glycolysis
K00895...
MELLADRAFT_1..
K00918
MELLADRAFT_7..
D-Ribose-5P
MELLADRAFT_7..
MELLADRAFT_4..
D-Ribose
β-D-Fructose-1,6P2
K01839...
D-Ribose-1P
MELLADRAFT_7..
K00615
MELLADRAFT_4..
MELLADRAFT_4..
D-Sedo-heptulose-7P
D-Glyceraldehyde-3P
PRPP
K05774
D-Ribose-1,5P
K01621
D-Xylulose-5P
Purine metabolism
MELLADRAFT_4..
2-Deoxy-D-ribose
Pyrimidine metabolism
K01619
K01839...
2-Deoxy-D-ribose-5P
2-Deoxy-D-ribose-1P
Histidine metabolism
00030 10/26/17
(c) Kanehisa Laboratories

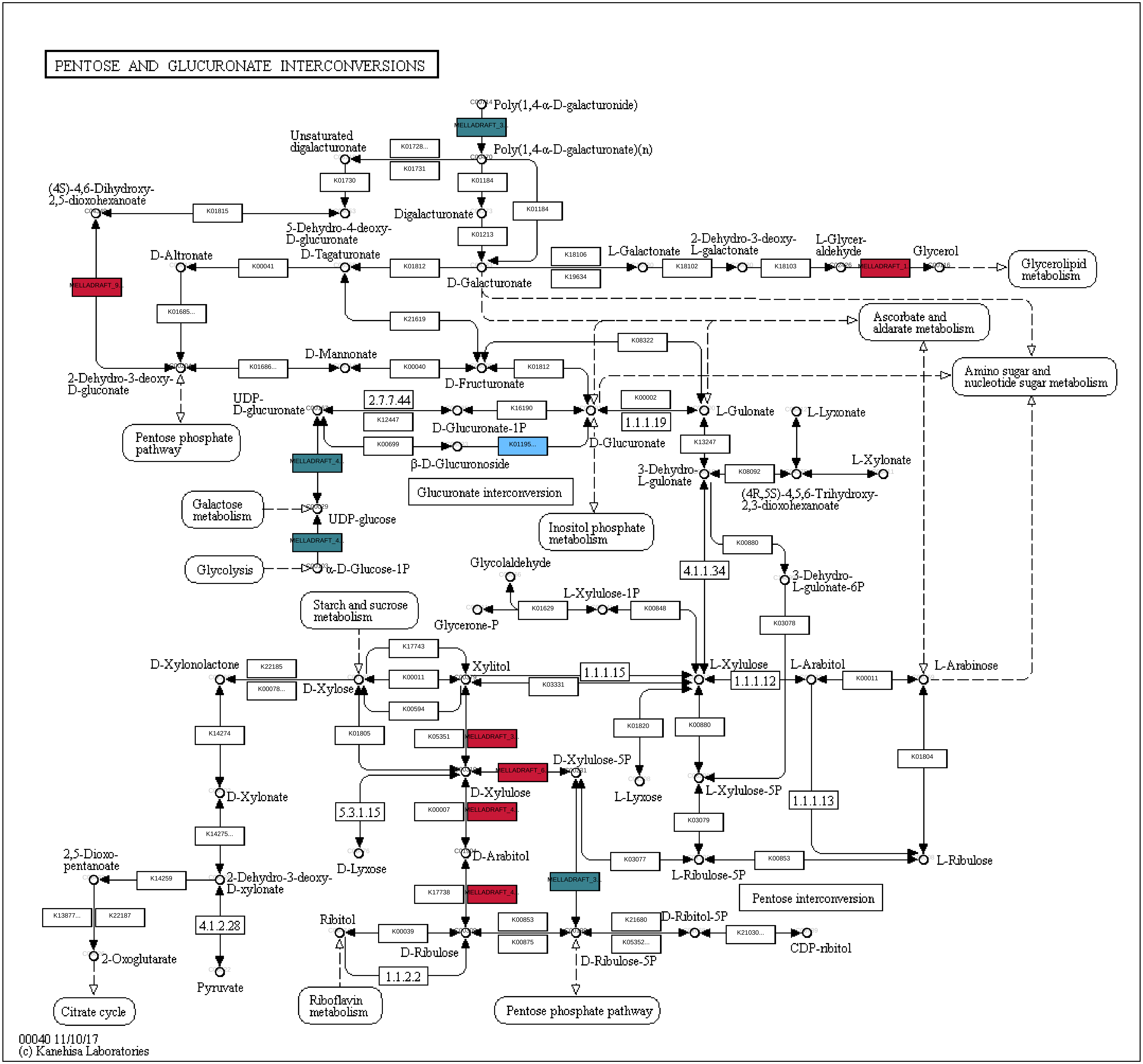
PENTOSE AND GLUCURONATE INTERCONVERSIONS
Poly(1,4-α-D-galacturonide)
MELLADRAFT_3...
Poly(1,4-α-D-galacturonate)(n)
Unsaturated digalacturonate
K01728...
K01731
K01730
K01184
K01184
(4S)-4,6-Dihydroxy-2,5-dioxohexanoate
K01815
5-Dehydro-4-deoxy-D-glucuronate
Digalacturonate
K01213
D-Altronate
K00041
D-Tagaturonate
K01812
D-Galacturonate
K18106
K19634
L-Galactonate
K18102
2-Dehydro-3-deoxy-L-galactonate
K18103
L-Glyceraldehyde
MELLADRAFT_1...
Glycerol
Glycerolipid metabolism
MELLADRAFT_9...
K01685...
K21619
Ascorbate and aldarate metabolism
K08322
Amino sugar and nucleotide sugar metabolism
D-Mannonate
K01686...
K00040
K01812
2-Dehydro-3-deoxy-D-gluconate
D-Fructuronate
UDP-D-glucuronate
2.7.7.44
K12447
K16190
D-Glucuronate-1P
K00002
1.1.1.19
L-Gulonate
L-Lyxonate
Pentose phosphate pathway
K00699
K01195...
D-Glucuronate
β-D-Glucuronoside
MELLADRAFT_4...
K13247
3-Dehydro-L-gulonate
K08092
L-Xylonate
(4R,5S)-4,5,6-Trihydroxy-2,3-dioxohexanoate
Glucuronate interconversion
Galactose metabolism
UDP-glucose
Inositol phosphate metabolism
K00880
MELLADRAFT_4...
Glycolysis
α-D-Glucose-1P
Glycolaldehyde
4.1.1.34
3-Dehydro-L-gulonate-6P
L-Xylulose-1P
K01629
K00848
Glycerone-P
Starch and sucrose metabolism
K03078
K17743
D-Xylonolactone
K22185
K00078...
D-Xylose
K00011
Xylitol
1.1.1.15
K03331
L-Xylulose
1.1.1.12
L-Arabitol
K00011
L-Arabinose
K00594
K14274
K01805
K05351
MELLADRAFT_3...
K01820
K00880
K01804
MELLADRAFT_6...
D-Xylulose-5P
D-Xylulose
L-Lyxose
L-Xylulose-5P
D-Xylonate
5.3.1.15
K00007
MELLADRAFT_4...
1.1.1.13
K14275...
K03079
D-Lyxose
D-Arabitol
2,5-Dioxopentanoate
K14259
2-Dehydro-3-deoxy-D-xylonate
K03077
K00853
L-Ribulose
MELLADRAFT_3...
L-Ribulose-5P
K17738
MELLADRAFT_4...
Pentose interconversion
K13877...
K22187
4.1.2.28
Ribitol
K00039
K00853
K21680
D-Ribitol-5P
K00875
K05352...
K21030...
CDP-ribitol
2-Oxoglutarate
D-Ribulose
D-Ribulose-5P
1.1.2.2
Pyruvate
Citrate cycle
Riboflavin metabolism
Pentose phosphate pathway
00040 11/10/17
(c) Kanehisa Laboratories

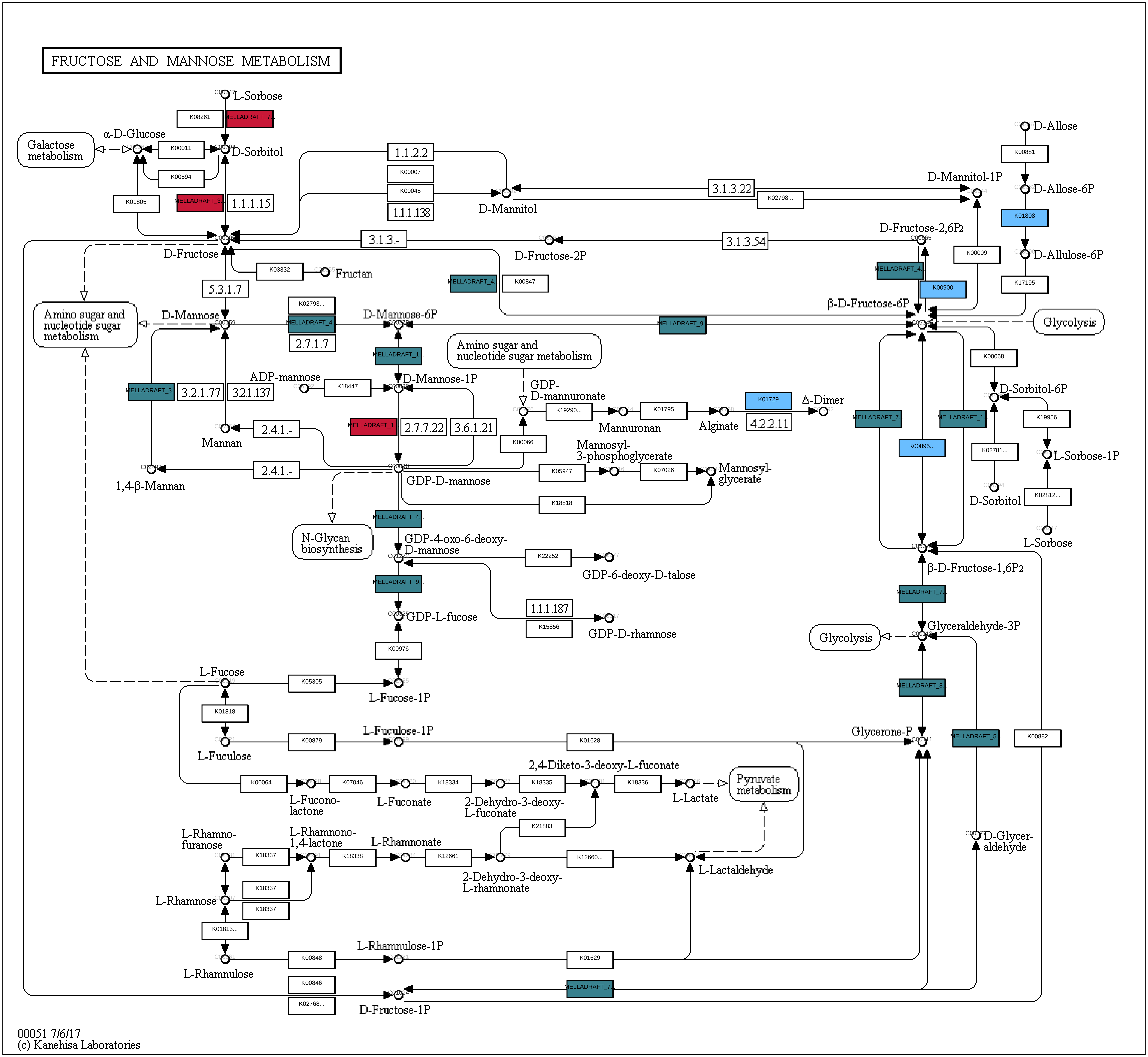
FRUCTOSE AND MANNOSE METABOLISM
L-Sorbose
K08261
α-D-Glucose
K00011
D-Sorbitol
Galactose metabolism
K00594
K01805
1.1.1.15
1.1.2.2
K00007
K00045
1.1.1.138
D-Mannitol
3.1.3.22
K02798...
D-Mannitol-1P
D-Allose
K00881
D-Allose-6P
K01808
D-Allulose-6P
K17195
D-Fructose
3.1.3.-
D-Fructose-2P
3.1.3.54
D-Fructose-2,6P2
K00009
K03332
Fructan
K00847
K00900
5.3.1.7
K02793...
2.7.1.7
D-Mannose
D-Mannose-6P
β-D-Fructose-6P
Glycolysis
Amino sugar and nucleotide sugar metabolism
ADP-mannose
K18447
D-Mannose-1P
3.2.1.77
3.2.1.137
K00068
D-Sorbitol-6P
GDP-D-mannuronate
K19290...
K01795
K01729
Δ-Dimer
Mannuronan
Alginate
4.2.2.11
K19956
2.7.7.22
3.6.1.21
2.4.1.-
Mannan
K00066
K00895...
K02781...
L-Sorbose-1P
Mannosyl-3-phosphoglycerate
K05947
K07026
Mannosyl-glycerate
1,4-β-Mannan
GDP-D-mannose
K18818
D-Sorbitol
K02812...
N-Glycan biosynthesis
GDP-4-oxo-6-deoxy-D-mannose
K22252
GDP-6-deoxy-D-talose
β-D-Fructose-1,6P2
1.1.1.187
K15856
GDP-D-rhamnose
GDP-L-fucose
Glyceraldehyde-3P
K00976
L-Fucose
K05305
L-Fucose-1P
K01818
L-Fuculose-1P
K00879
K01628
Glycerone-P
K00882
L-Fuculose
2,4-Diketo-3-deoxy-L-fuconate
K00064...
K07046
K18334
K18335
K18336
Pyruvate metabolism
L-Fucono-lactone
L-Fuconate
2-Dehydro-3-deoxy-L-fuconate
L-Lactate
K21883
L-Rhamno-furanose
K18337
L-Rhamnono-1,4-lactone
K18338
L-Rhamnonate
K12661
K12660...
D-Glycer-aldehyde
2-Dehydro-3-deoxy-L-rhamnonate
L-Lactaldehyde
L-Rhamnose
K01813...
K00848
L-Rhamnulose-1P
K01629
L-Rhamnulose
K00846
K02768...
D-Fructose-1P
00051 7/6/17
(c) Kanehisa Laboratories

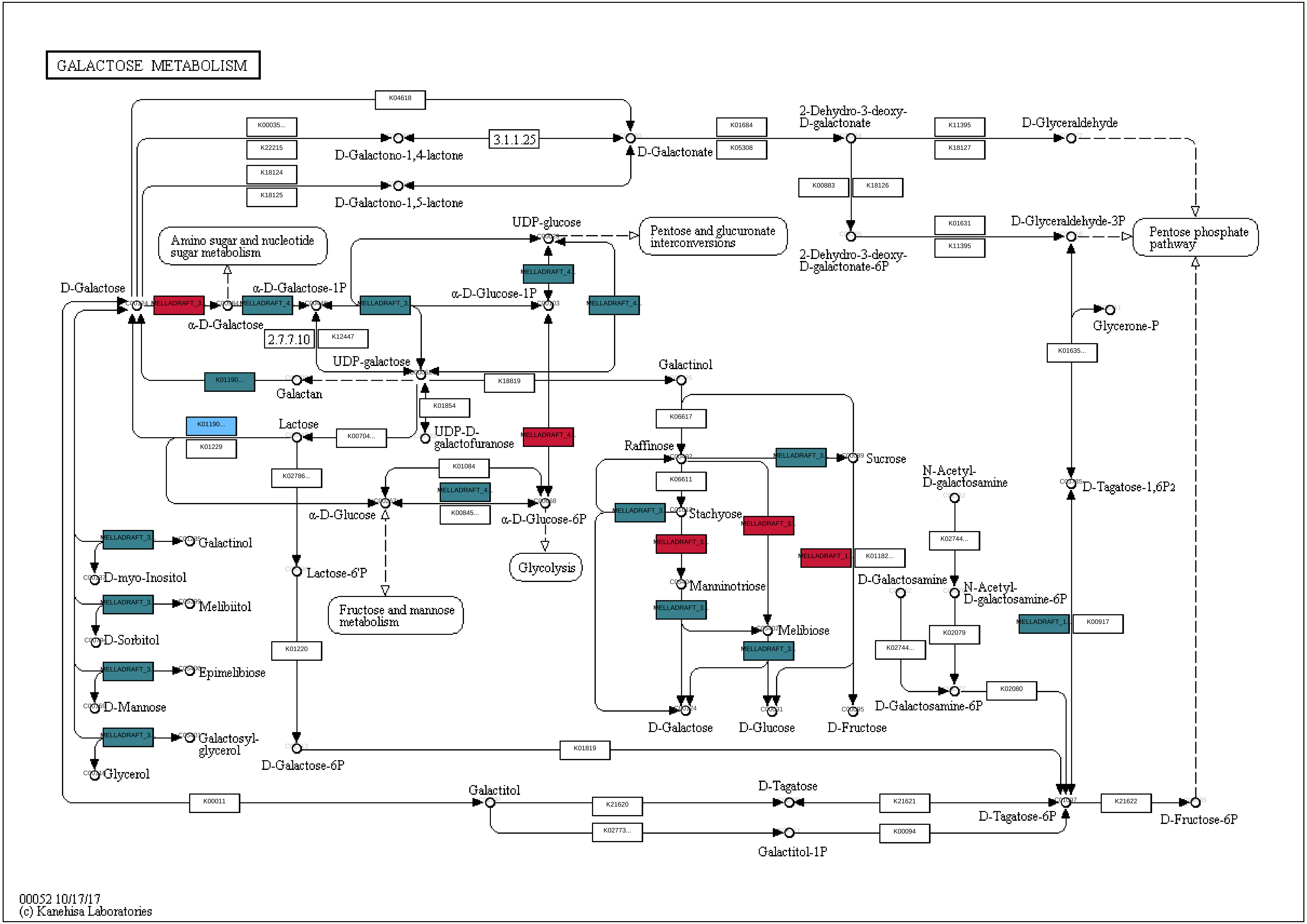
GALACTOSE METABOLISM
K04618
K00035...
K22215
K18124
K18125
3.1.1.25
D-Galactono-1,4-lactone
D-Galactono-1,5-lactone
D-Galactonate
K01684
K05308
2-Dehydro-3-deoxy-D-galactonate
K11395
K18127
D-Glyceraldehyde
K00883
K18126
K01631
K11395
D-Glyceraldehyde-3P
2-Dehydro-3-deoxy-D-galactonate-6P
Pentose phosphate pathway
Amino sugar and nucleotide sugar metabolism
UDP-glucose
Pentose and glucuronate interconversions
D-Galactose
α-D-Galactose
α-D-Galactose-1P
α-D-Glucose-1P
2.7.7.10
K12447
UDP-galactose
Galactan
K01190...
K18819
Galactinol
K01854
UDP-D-galactofuranose
Lactose
K01190...
K01229
K00704...
K02786...
K01084
K00845...
α-D-Glucose
α-D-Glucose-6P
Glycolysis
Galactinol
D-myo-Inositol
Melibiitol
D-Sorbitol
Epimelibiose
D-Mannose
Galactosyl-glycerol
Glycerol
Lactose-6P
Fructose and mannose metabolism
K01220
D-Galactose-6P
K01819
K06617
Raffinose
Sucrose
K06611
Stachyose
Manninotriose
Melibiose
K01182...
D-Galactose
D-Glucose
D-Fructose
Glycerone-P
K01635...
D-Tagatose-1,6P2
N-Acetyl-D-galactosamine
K02744...
D-Galactosamine
N-Acetyl-D-galactosamine-6P
K02079
K02744...
K00917
K02080
D-Galactosamine-6P
K00011
Galactitol
K21620
K02773...
D-Tagatose
K21621
K00094
Galactitol-1P
D-Tagatose-6P
K21622
D-Fructose-6P
00052 10/17/17
(c) Kanehisa Laboratories

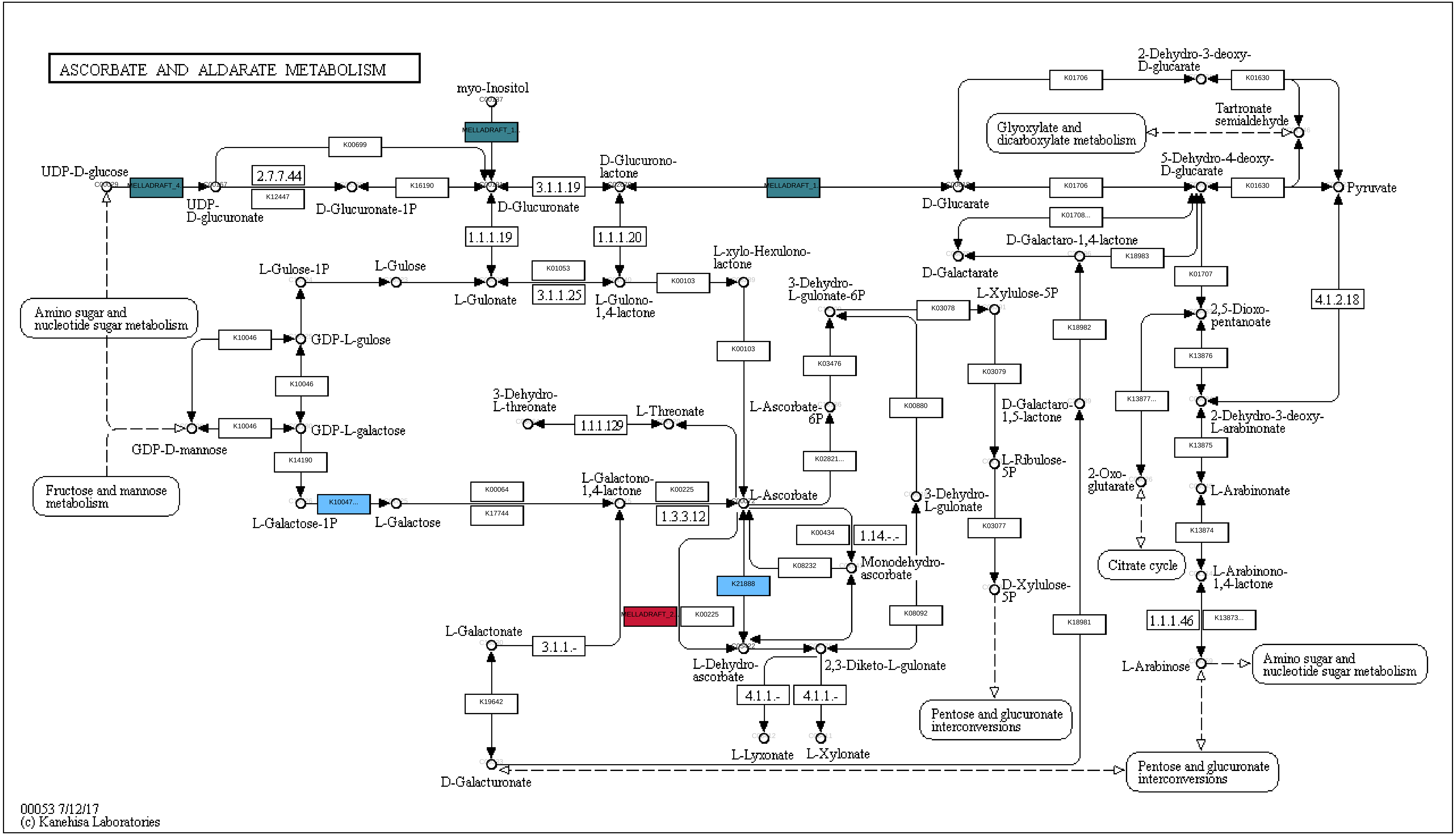
ASCORBATE AND ALDARATE METABOLISM
myo-Inositol
UDP-D-glucose
UDP-D-glucuronate
2.7.7.44
K12447
K00699
MELLADRAFT_4
MELLADRAFT_1
D-Glucuronate-1P
K16190
D-Glucuronate
3.1.1.19
D-Glucurono-lactone
1.1.1.19
1.1.1.20
D-Glucarate
D-Galactarate
D-Galactaro-1,4-lactone
K01706
K01630
K01708...
K18983
K01707
2-Dehydro-3-deoxy-D-glucarate
Tartronate semialdehyde
Glyoxylate and dicarboxylate metabolism
5-Dehydro-4-deoxy-D-glucarate
Pyruvate
4.1.2.18
2,5-Dioxo-pentanoate
K13876
K13877...
2-Dehydro-3-deoxy-L-arabinonate
K13875
L-Arabinonate
K13874
L-Arabinono-1,4-lactone
1.1.1.46
K13873...
L-Arabinose
Amino sugar and nucleotide sugar metabolism
2-Oxo-glutarate
Citrate cycle
Pentose and glucuronate interconversions
L-Gulose-1P
L-Gulose
L-Gulonate
K01053
3.1.1.25
L-Gulono-1,4-lactone
K00103
L-xylo-Hexulono-lactone
Amino sugar and nucleotide sugar metabolism
GDP-L-gulose
K10046
GDP-L-galactose
GDP-D-mannose
K14190
Fructose and mannose metabolism
L-Galactose-1P
K10047...
L-Galactose
K00064
K17744
L-Galactono-1,4-lactone
K00225
1.3.3.12
3-Dehydro-L-threonate
1.1.1.129
L-Threonate
L-Ascorbate
K00434
1.14.-.-
K08232
Monodehydro-ascorbate
K21888
MELLADRAFT_2
L-Galactonate
3.1.1.-
K19642
D-Galacturonate
L-Dehydro-ascorbate
2,3-Diketo-L-gulonate
4.1.1.-
L-Lyxonate
L-Xylonate
3-Dehydro-L-gulonate-6P
K03476
L-Ascorbate-6P
K02821...
K03078
L-Xylulose-5P
K00880
3-Dehydro-L-gulonate
K08092
K03079
L-Ribulose-5P
K03077
D-Xylulose-5P
D-Galactaro-1,5-lactone
K18982
K18981
00053 7/12/17
(c) Kanehisa Laboratories

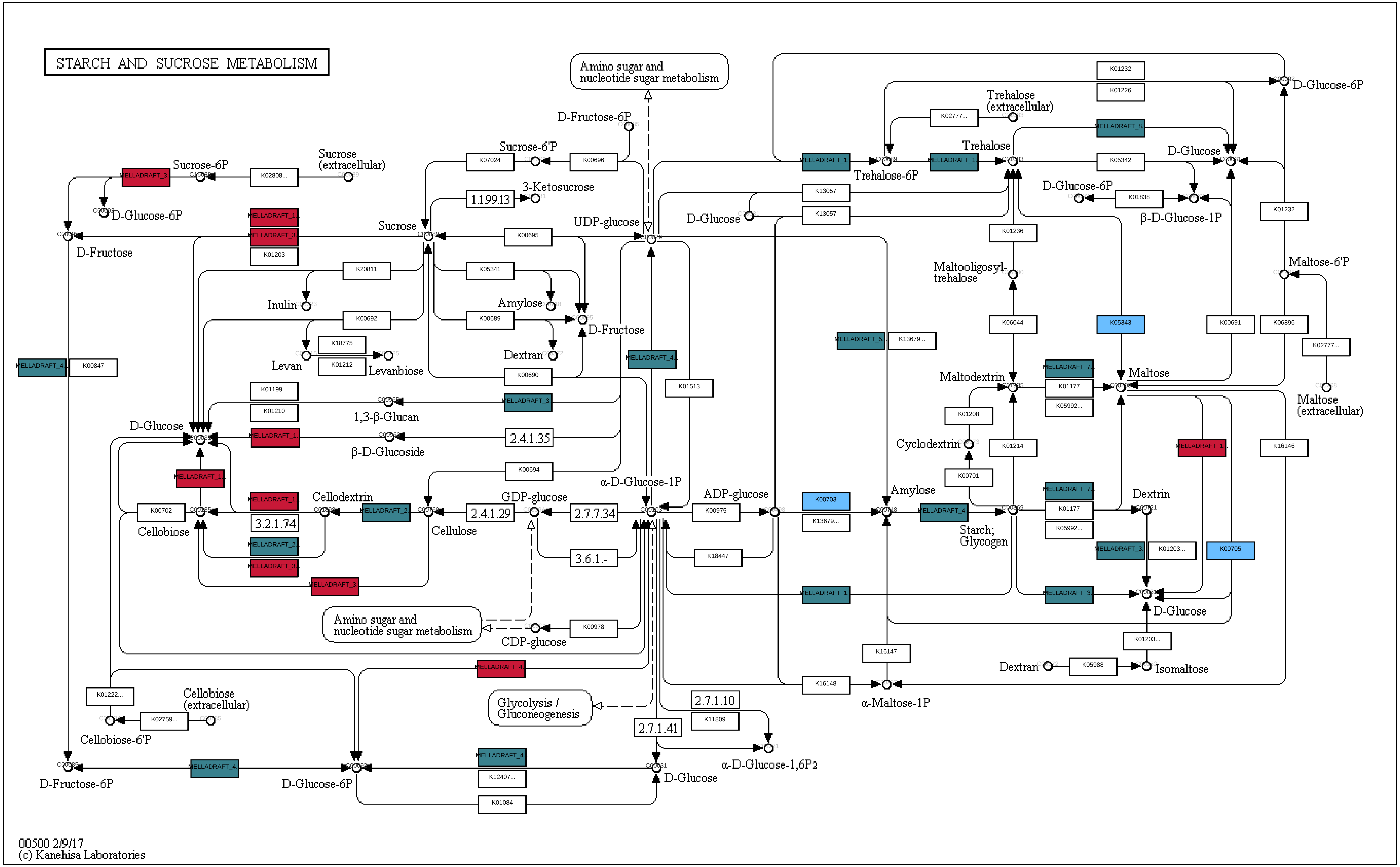

STARCH AND SUCROSE METABOLISM
Amino sugar and nucleotide sugar metabolism
D-Fructose-6P
Sucrose-6'P
Sucrose-6P
Sucrose (extracellular)
D-Glucose-6P
D-Fructose
Sucrose
3-Ketosucrose
1.1.99.13
UDP-glucose
D-Glucose
Trehalose (extracellular)
Trehalose
Trehalose-6P
D-Glucose-6P
β-D-Glucose-1P
Maltose-6'P
Maltooligosyl-trehalose
Maltose
Maltose (extracellular)
Maltodextrin
Cyclodextrin
Amylose
Starch; Glycogen
Dextrin
Isomaltose
Dextran
α-Maltose-1P
ADP-glucose
α-D-Glucose-1P
Inulin
Amylose
D-Fructose
Levan
Levanbiose
Dextran
1,3-β-Glucan
β-D-Glucoside
2.4.1.35
D-Glucose
Cellodextrin
GDP-glucose
2.4.1.29
2.7.7.34
3.2.1.74
Cellobiose
Cellulose
3.6.1.-
Amino sugar and nucleotide sugar metabolism
CDP-glucose
Glycolysis / Gluconeogenesis
2.7.1.10
2.7.1.41
α-D-Glucose-1,6P2
Cellobiose (extracellular)
Cellobiose-6'P
D-Fructose-6P
D-Glucose-6P
D-Glucose
K07024
K00696
K02808...
K00695
K01203
K20811
K05341
K00692
K00689
K18775
K01212
K00690
K01199...
K01210
K00694
K00702
K00847
K00975
K13679...
K18447
K01513
K11809
K12407...
K01084
K02759...
K01222...
K00978
K13057
K01236
K06044
K01232
K01226
K02777...
K05342
K01838
K05343
K00691
K06896
K01177
K05992...
K01208
K01214
K00701
K00703
K00705
K01203...
K16146
K16147
K16148
K05988
MELLADRAFT_1
MELLADRAFT_2
MELLADRAFT_3
MELLADRAFT_4
MELLADRAFT_5
MELLADRAFT_7
MELLADRAFT_8
00500 2/9/17
(c) Kanehisa Laboratories

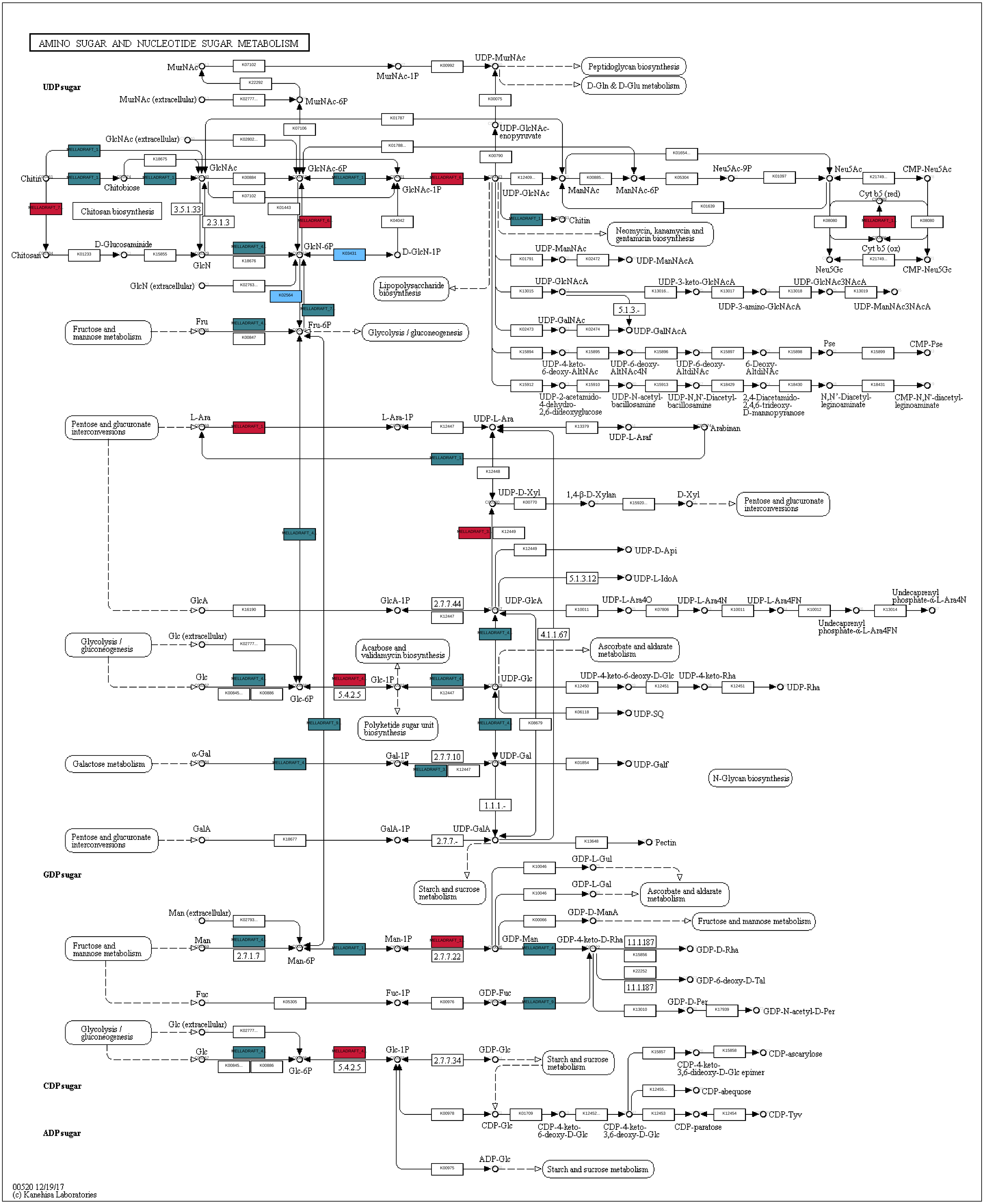

AMINO SUGAR AND NUCLEOTIDE SUGAR METABOLISM
UDP sugar
GDP sugar
CDP sugar
ADP sugar
MurNAc
MurNAc (extracellular)
MurNAc-6P
MurNAc-1P
UDP-MurNAc
Peptidoglycan biosynthesis
D-Gln & D-Glu metabolism
UDP-GlcNAc-enolpyruvate
GlcNAc (extracellular)
GlcNAc
GlcNAc-6P
GlcNAc-1P
UDP-GlcNAc
Chitin
Chitobiose
Chitosan
Chitosan biosynthesis
3.5.1.33
2.3.1.3
D-Glucosaminide
GlcN
GlcN-6P
D-GlcN-1P
GlcN (extracellular)
ManNAc
ManNAc-6P
Neu5Ac-9P
Neu5Ac
CMP-Neu5Ac
Cyt b5 (red)
Cyt b5 (ox)
Neu5Gc
CMP-Neu5Gc
Chitin
Neomycin, kanamycin and gentamicin biosynthesis
UDP-ManNAc
UDP-ManNAcA
UDP-GlcNAcA
UDP-3-keto-GlcNAcA
UDP-GlcNAc3NAcA
UDP-3-amino-GlcNAcA
UDP-ManNAc3NAcA
5.1.3.-
Lipopolysaccharide biosynthesis
UDP-GalNAc
UDP-GalNAcA
Fructose and mannose metabolism
Fru
Fru-6P
Glycolysis / gluconeogenesis
Pse
CMP-Pse
UDP-4-keto-6-deoxy-AltNAc
UDP-6-deoxy-AltNAc4N
UDP-6-deoxy-AltdiNAc
6-Deoxy-AltdiNAc
UDP-2-acetamido-4-dehydro-2,6-dideoxyglucose
UDP-N-acetyl-bacillosamine
UDP-N,N'-Diacetyl-bacillosamine
2,4-Diacetamido-2,4,6-trideoxy-D-mannopyranose
N,N'-Diacetyl-legionaminate
CMP-N,N'-diacetyl-legionaminate
Pentose and glucuronate interconversions
L-Ara
L-Ara-1P
UDP-L-Ara
UDP-L-Araf
Arabinan
UDP-D-Xyl
1,4-β-D-Xylan
D-Xyl
Pentose and glucuronate interconversions
UDP-D-Api
5.1.3.12
UDP-L-IdoA
GlcA
GlcA-1P
2.7.7.44
UDP-GlcA
UDP-L-Ara4O
UDP-L-Ara4N
UDP-L-Ara4FN
Undecaprenyl phosphate-α-L-Ara4N
Undecaprenyl phosphate-α-L-Ara4FN
4.1.1.67
Glycolysis / gluconeogenesis
Glc (extracellular)
Acarbose and validamycin biosynthesis
Ascorbate and aldarate metabolism
Glc
Glc-6P
Glc-1P
5.4.2.5
UDP-Glc
UDP-4-keto-6-deoxy-D-Glc
UDP-4-keto-Rha
UDP-Rha
UDP-SQ
Polyketide sugar unit biosynthesis
Galactose metabolism
α-Gal
Gal-1P
2.7.7.10
UDP-Gal
UDP-Galf
N-Glycan biosynthesis
1.1.1.-
Pentose and glucuronate interconversions
GalA
GalA-1P
2.7.7.-
UDP-GalA
Pectin
Starch and sucrose metabolism
GDP-L-Gul
GDP-L-Gal
Ascorbate and aldarate metabolism
GDP-D-ManA
Fructose and mannose metabolism
Man (extracellular)
Fructose and mannose metabolism
Man
2.7.1.7
Man-6P
Man-1P
2.7.7.22
GDP-Man
GDP-4-keto-D-Rha
1.1.1.187
GDP-D-Rha
GDP-6-deoxy-D-Tal
GDP-D-Per
GDP-N-acetyl-D-Per
Fuc
Fuc-1P
GDP-Fuc
Glycolysis / gluconeogenesis
Glc (extracellular)
Glc
Glc-6P
5.4.2.5
Glc-1P
2.7.7.34
GDP-Glc
Starch and sucrose metabolism
CDP-ascarylose
CDP-4-keto-3,6-dideoxy-D-Glc epimer
CDP-abequose
CDP-Glc
CDP-4-keto-6-deoxy-D-Glc
CDP-4-keto-3,6-deoxy-D-Glc
CDP-paratose
CDP-Tyv
ADP-Glc
Starch and sucrose metabolism
00520 12/19/17
(c) Kanehisa Laboratories

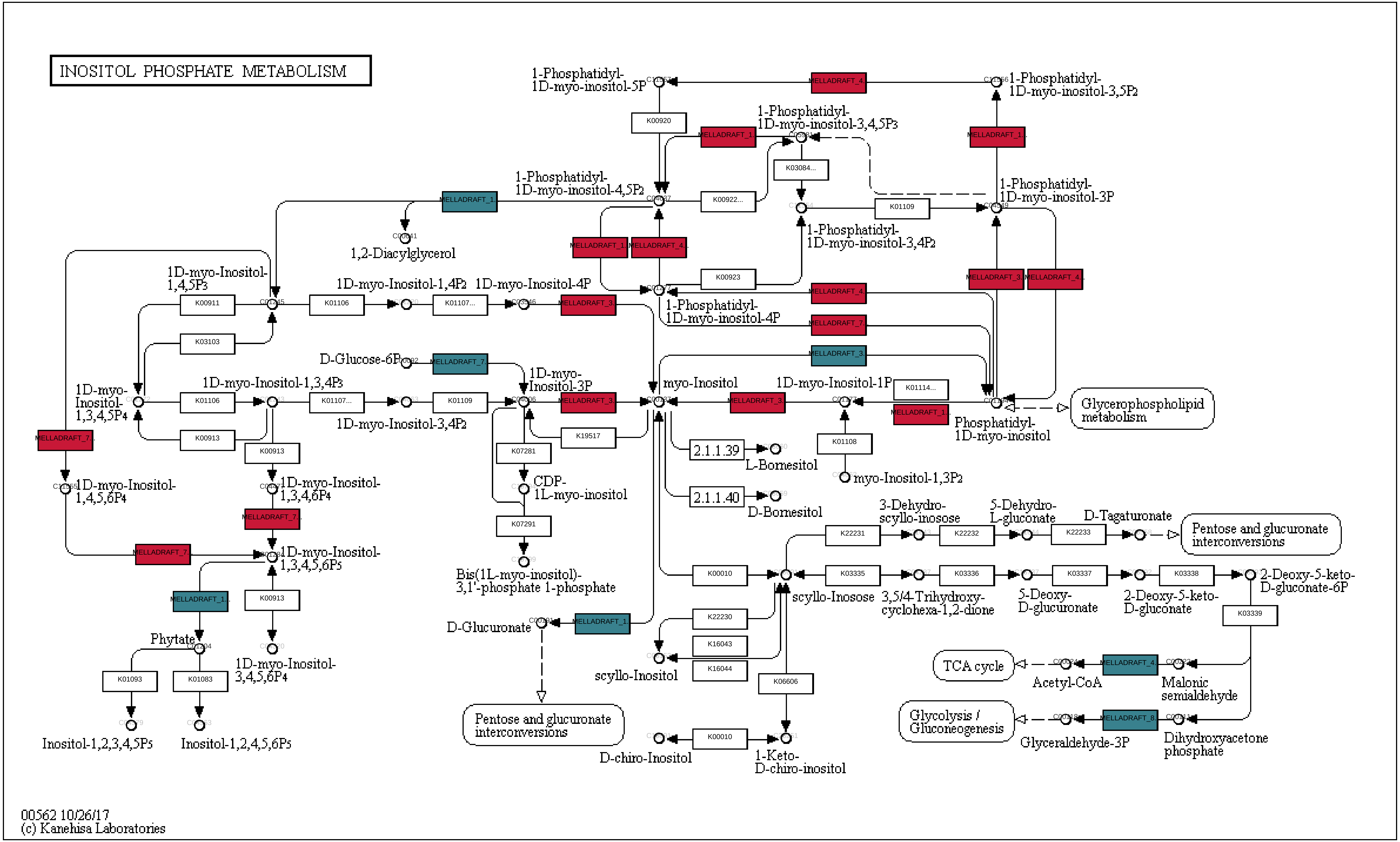
INOSITOL PHOSPHATE METABOLISM
1-Phosphatidyl-1D-myo-inositol-5P
1-Phosphatidyl-1D-myo-inositol-3,5P2
1-Phosphatidyl-1D-myo-inositol-3,4,5P3
1-Phosphatidyl-1D-myo-inositol-4,5P2
1-Phosphatidyl-1D-myo-inositol-3P
1-Phosphatidyl-1D-myo-inositol-3,4P2
1-Phosphatidyl-1D-myo-inositol-4P
1,2-Diacylglycerol
1D-myo-Inositol-1,4,5P3
1D-myo-Inositol-1,4P2
1D-myo-Inositol-4P
D-Glucose-6P
1D-myo-Inositol-1,3,4P3
1D-myo-Inositol-3P
myo-Inositol
1D-myo-Inositol-1P
1D-myo-Inositol-1,3,4,5P4
1D-myo-Inositol-3,4P2
Phosphatidyl-1D-myo-inositol
Glycerophospholipid metabolism
L-Bornesitol
myo-Inositol-1,3P2
D-Bornesitol
CDP-1L-myo-inositol
1D-myo-Inositol-1,4,5,6P4
1D-myo-Inositol-1,3,4,6P4
1D-myo-Inositol-1,3,4,5,6P5
Bis(1L-myo-inositol)-3,1'-phosphate 1-phosphate
3-Dehydro-scyllo-inosose
5-Dehydro-L-gluconate
D-Tagaturonate
Pentose and glucuronate interconversions
scyllo-Inosose
3,5/4-Trihydroxy-cyclohexa-1,2-dione
5-Deoxy-D-glucuronate
2-Deoxy-5-keto-D-gluconate
2-Deoxy-5-keto-D-gluconate-6P
D-Glucuronate
Phytate
1D-myo-Inositol-3,4,5,6P4
scyllo-Inositol
TCA cycle
Acetyl-CoA
Malonic semialdehyde
Glycolysis / Gluconeogenesis
Glyceraldehyde-3P
Dihydroxyacetone phosphate
Inositol-1,2,3,4,5P5
Inositol-1,2,4,5,6P5
D-chiro-Inositol
1-Keto-D-chiro-inositol
K00920
K03084...
K00922...
K01109
K00923
K00911
K01106
K01107...
K03103
K00913
K07281
K19517
K07291
K01114...
K01108
2.1.1.39
2.1.1.40
K22231
K22232
K22233
K00010
K03335
K03336
K03337
K03338
K03339
K22230
K16043
K16044
K06606
K01093
K01083
MELLADRAFT_1
MELLADRAFT_3
MELLADRAFT_4
MELLADRAFT_7
MELLADRAFT_8
00562 10/26/17
(c) Kanehisa Laboratories

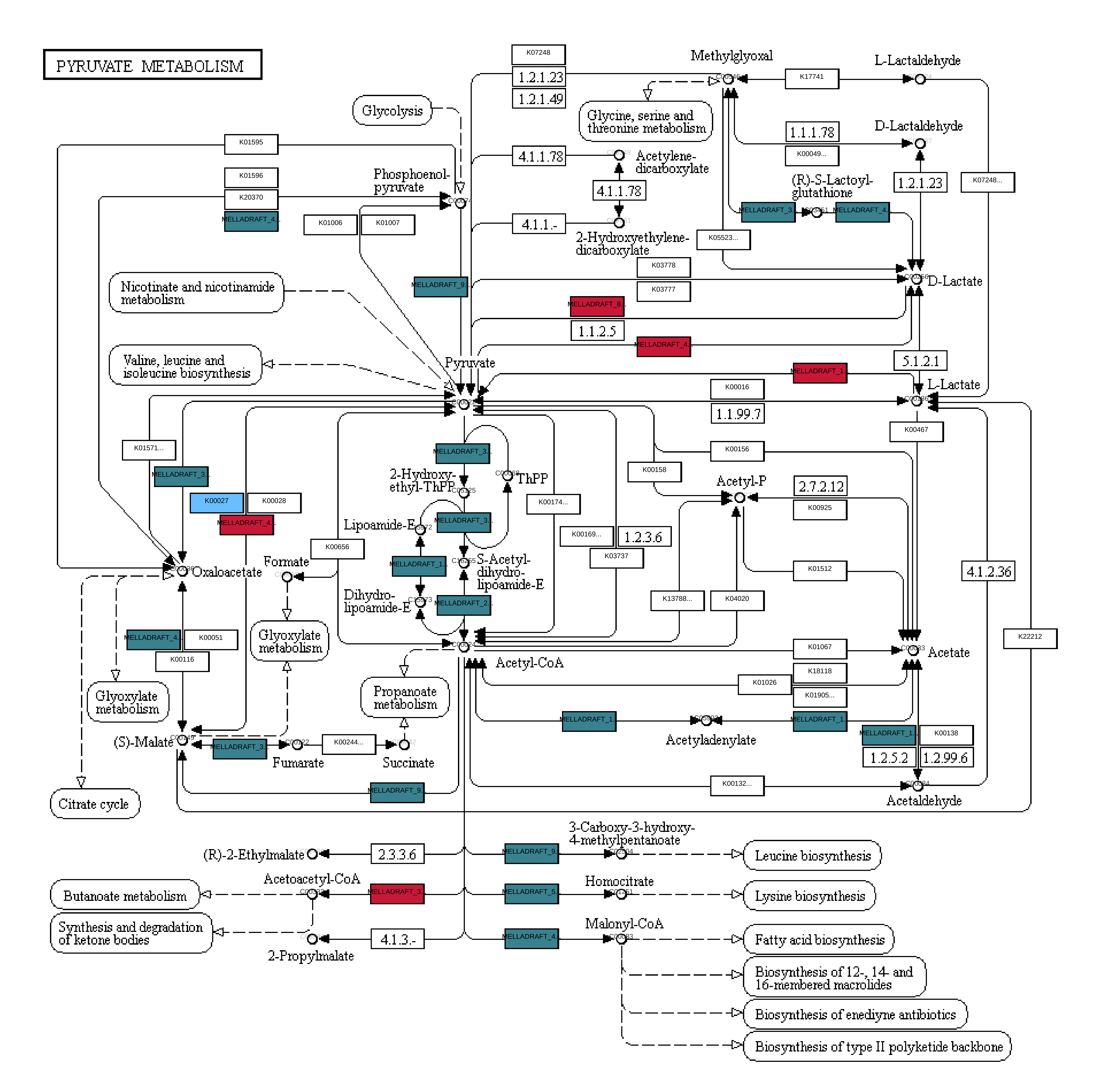


00620 10/26/17
(c) Kanehisa Laboratories

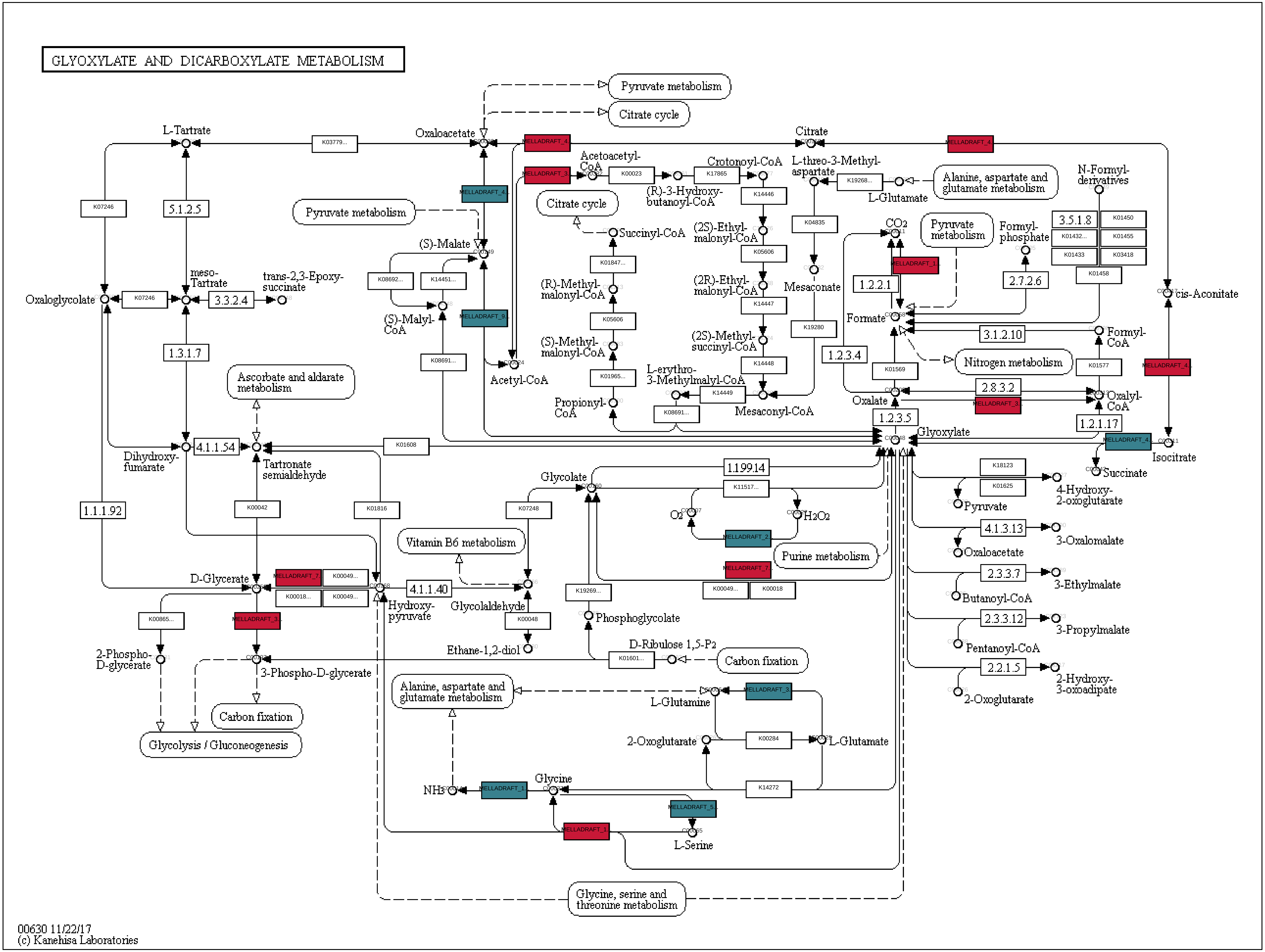
GLYOXYLATE AND DICARBOXYLATE METABOLISM
Pyruvate metabolism
Citrate cycle
L-Tartrate
Oxaloacetate
Citrate
K03779...
MELLADRAFT_4...
MELLADRAFT_3...
Acetoacetyl-CoA
K00023
Crotonoyl-CoA
K17865
(R)-3-Hydroxy-butanoyl-CoA
L-threo-3-Methyl-aspartate
K19268...
L-Glutamate
Alanine, aspartate and glutamate metabolism
N-Formyl-derivatives
K07246
5.1.2.5
Pyruvate metabolism
Citrate cycle
K14446
Succinyl-CoA
(2S)-Ethyl-malonyl-CoA
K04835
CO2
Pyruvate metabolism
Formyl-phosphate
3.5.1.8
K01450
K01432...
K01455
K01433
K03418
(S)-Malate
K08692...
K14451...
K01847...
K05606
MELLADRAFT_1...
1.2.2.1
2.7.2.6
K01458
meso-Tartrate
trans-2,3-Epoxy-succinate
(R)-Methyl-malonyl-CoA
(2R)-Ethyl-malonyl-CoA
Mesaconate
Oxaloglycolate
3.3.2.4
(S)-Malyl-CoA
MELLADRAFT_9...
K14447
K19280
Formate
cis-Aconitate
K05606
3.1.2.10
Formyl-CoA
1.3.1.7
(S)-Methyl-malonyl-CoA
(2S)-Methyl-succinyl-CoA
1.2.3.4
Ascorbate and aldarate metabolism
K08691...
Acetyl-CoA
K01965...
K14448
K01569
Nitrogen metabolism
K01577
L-erythro-3-Methylmalyl-CoA
K14449
Propionyl-CoA
K08691...
Mesaconyl-CoA
Oxalate
2.8.3.2
Oxalyl-CoA
1.2.3.5
1.2.1.17
Glyoxylate
4.1.1.54
K01608
Isocitrate
Dihydroxy-fumarate
Tartronate semialdehyde
K18123
Succinate
1.1.99.14
4-Hydroxy-2-oxoglutarate
Glycolate
K01625
K11517...
Pyruvate
1.1.1.92
K00042
K01816
K07248
O2
H2O2
4.1.3.13
Vitamin B6 metabolism
MELLADRAFT_2...
3-Oxalomalate
Purine metabolism
Oxaloacetate
D-Glycerate
MELLADRAFT_7...
K00049...
MELLADRAFT_7...
2.3.3.7
K00018...
K00049...
4.1.1.40
K19269...
K00049...
K00018
3-Ethylmalate
Hydroxy-pyruvate
Glycolaldehyde
Butanoyl-CoA
K00865...
K00048
Phosphoglycolate
2.3.3.12
3-Propylmalate
2-Phospho-D-glycerate
Ethane-1,2-diol
D-Ribulose 1,5-P2
K01601...
Carbon fixation
Pentanoyl-CoA
3-Phospho-D-glycerate
2.2.1.5
Alanine, aspartate and glutamate metabolism
MELLADRAFT_3...
2-Hydroxy-3-oxoadipate
L-Glutamine
2-Oxoglutarate
Carbon fixation
K00284
Glycolysis / Gluconeogenesis
2-Oxoglutarate
L-Glutamate
Glycine
NH3
MELLADRAFT_1...
K14272
MELLADRAFT_5...
MELLADRAFT_1...
L-Serine
Glycine, serine and threonine metabolism
00630 11/22/17
(c) Kanehisa Laboratories

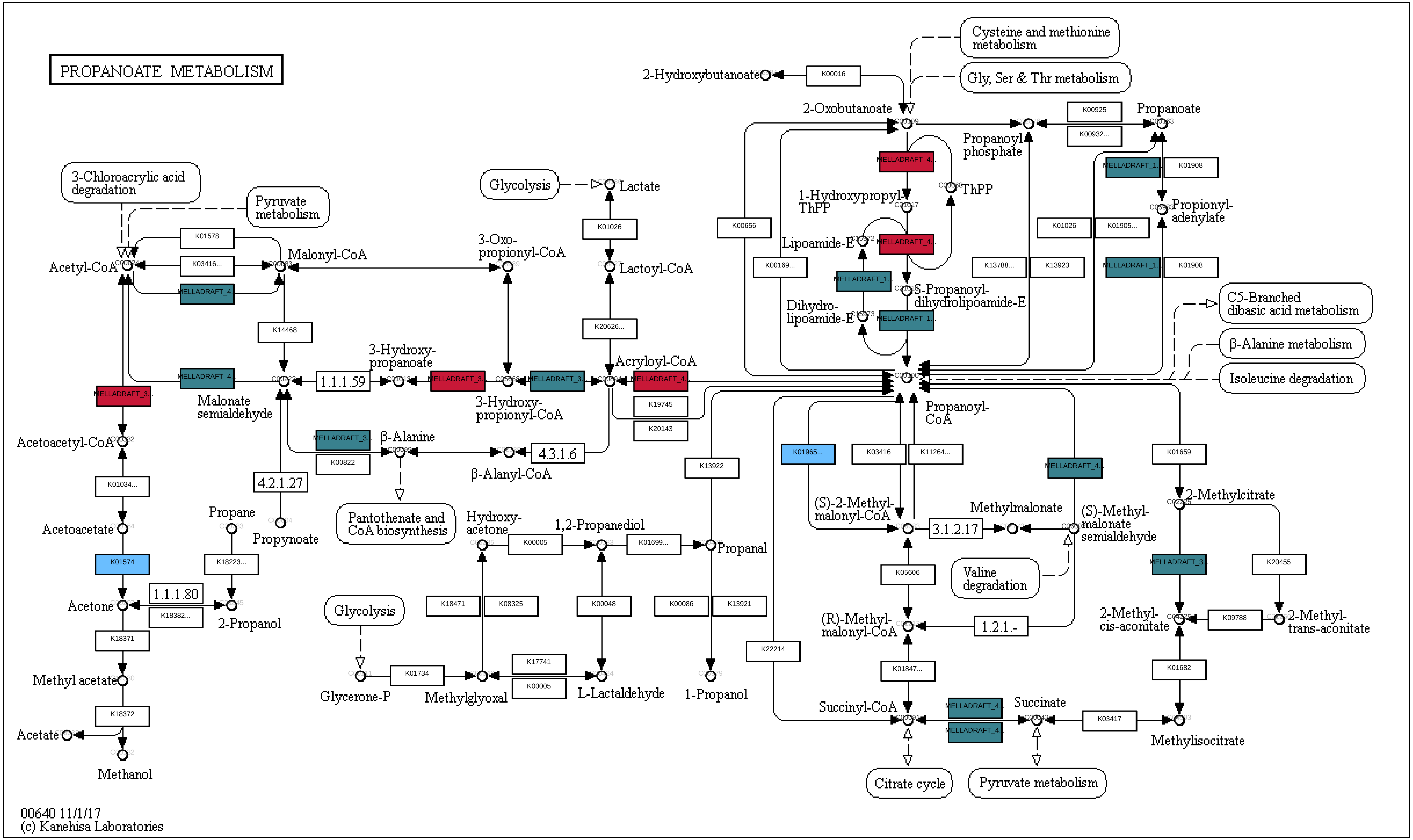

PROPANOATE METABOLISM
Cysteine and methionine metabolism
Gly, Ser & Thr metabolism
2-Hydroxybutanoate
K00016
2-Oxobutanoate
K00925
K00932...
Propanoate
Propanoyl phosphate
MELLADRAFT_4...
1-Hydroxypropyl-ThPP
ThPP
Lipoamide-E
MELLADRAFT_4...
MELLADRAFT_1...
S-Propanoyl-dihydrolipoamide-E
Dihydro-lipoamide-E
MELLADRAFT_1...
K00656
K00169...
K13788...
K13923
K01026
K01905...
MELLADRAFT_1...
K01908
MELLADRAFT_1...
K01908
Propionyl-adenylate
C5-Branched dibasic acid metabolism
β-Alanine metabolism
Isoleucine degradation
3-Chloroacrylic acid degradation
Pyruvate metabolism
Glycolysis
Lactate
K01026
Lactoyl-CoA
K20626...
Acryloyl-CoA
K01578
K03416...
MELLADRAFT_4...
Acetyl-CoA
Malonyl-CoA
3-Oxo-propionyl-CoA
K14468
3-Hydroxy-propanoate
1.1.1.59
MELLADRAFT_4...
MELLADRAFT_3...
MELLADRAFT_3...
MELLADRAFT_3...
MELLADRAFT_4...
Malonate semialdehyde
3-Hydroxy-propionyl-CoA
K19745
K20143
MELLADRAFT_3...
K00822
β-Alanine
β-Alanyl-CoA
4.3.1.6
4.2.1.27
Propynoate
Pantothenate and CoA biosynthesis
Acetoacetyl-CoA
K01034...
Acetoacetate
K01574
Acetone
1.1.1.80
K18382...
K18371
Methyl acetate
K18372
Acetate
Methanol
Propane
K18223...
2-Propanol
Glycolysis
Glycerone-P
K01734
Methylglyoxal
K18471
K08325
Hydroxy-acetone
K00005
1,2-Propanediol
K17741
K00005
K00048
L-Lactaldehyde
K01699...
Propanal
K00086
K13921
1-Propanol
K13922
Propanoyl-CoA
K01965...
K03416
K11264...
MELLADRAFT_4...
(S)-2-Methyl-malonyl-CoA
3.1.2.17
Methylmalonate
(S)-Methyl-malonate semialdehyde
Valine degradation
K05606
(R)-Methyl-malonyl-CoA
1.2.1.-
K22214
K01847...
Succinyl-CoA
MELLADRAFT_4...
MELLADRAFT_4...
Succinate
K03417
Citrate cycle
Pyruvate metabolism
K01659
2-Methylcitrate
MELLADRAFT_3...
K20455
2-Methyl-cis-aconitate
K09788
2-Methyl-trans-aconitate
K01682
Methylisocitrate
00640 11/1/17
(c) Kanehisa Laboratories

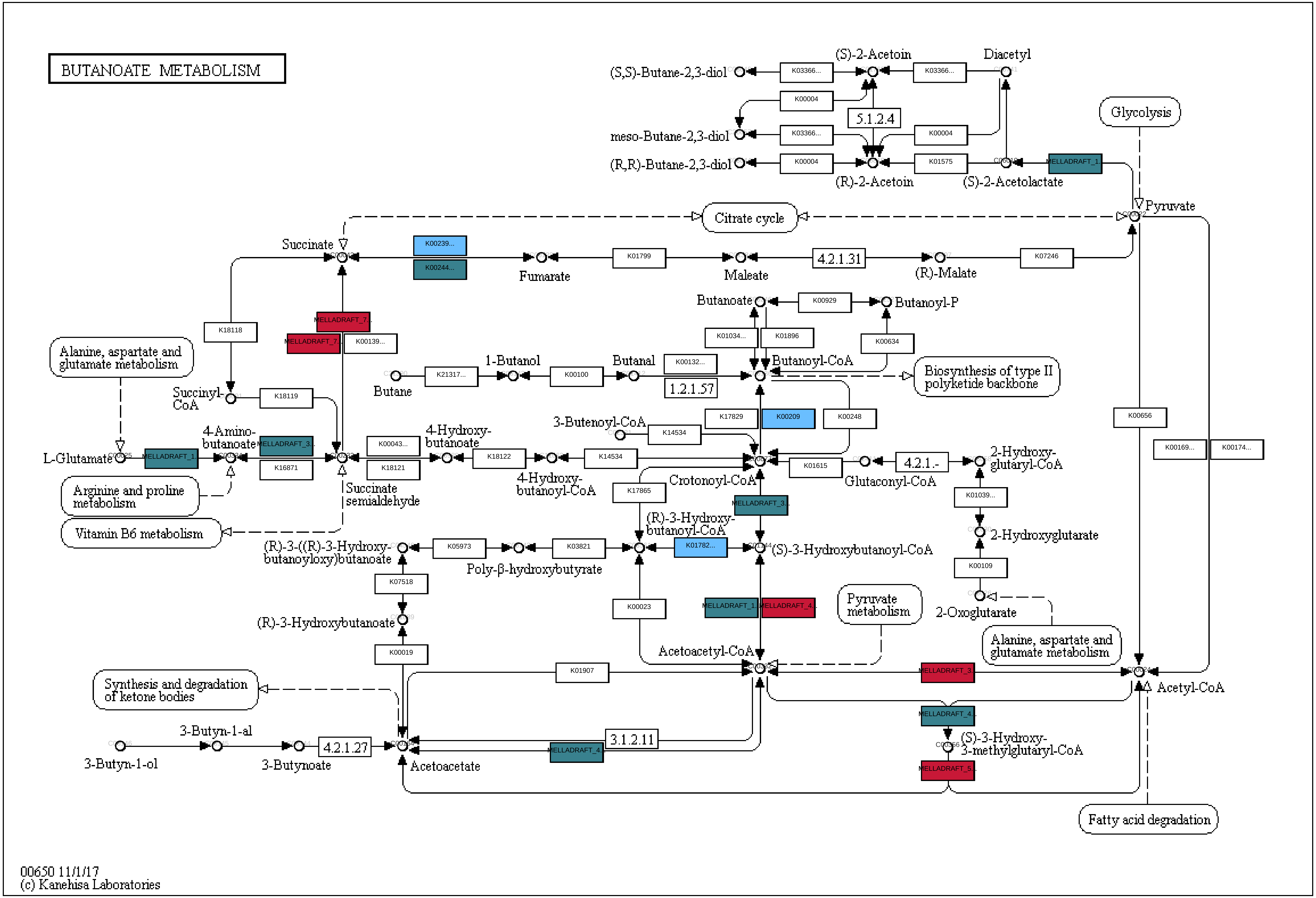
BUTANOATE METABOLISM
(S,S)-Butane-2,3-diol
(S)-2-Acetoin
Diacetyl
K03366...
K00004
5.1.2.4
meso-Butane-2,3-diol
(R,R)-Butane-2,3-diol
K01575
(R)-2-Acetoin
(S)-2-Acetolactate
MELLADRAFT_1...
Glycolysis
Pyruvate
Citrate cycle
Succinate
K00239...
K00244...
Fumarate
K01799
Maleate
4.2.1.31
(R)-Malate
K07246
Butanoate
K00929
Butanoyl-P
MELLADRAFT_7...
K18118
K00139...
K01034...
K01896
K00634
Alanine, aspartate and glutamate metabolism
1-Butanol
Butanal
K00132...
Butanoyl-CoA
Biosynthesis of type II polyketide backbone
K21317...
K00100
Butane
1.2.1.57
Succinyl-CoA
K18119
3-Butenoyl-CoA
K17829
K00209
K00248
K00656
4-Amino-butanoate
K00043...
4-Hydroxy-butanoate
K14534
K00169...
K00174...
L-Glutamate
K18122
K16871
K18121
K01615
Glutaconyl-CoA
4.2.1.-
2-Hydroxy-glutaryl-CoA
Arginine and proline metabolism
Succinate semialdehyde
4-Hydroxy-butanoyl-CoA
Crotonoyl-CoA
K17865
K01039...
Vitamin B6 metabolism
(R)-3-Hydroxy-butanoyl-CoA
2-Hydroxyglutarate
(R)-3-((R)-3-Hydroxy-butanoyloxy)butanoate
K05973
K03821
K01782...
(S)-3-Hydroxybutanoyl-CoA
Poly-β-hydroxybutyrate
K00109
K07518
K00023
MELLADRAFT_4...
Pyruvate metabolism
2-Oxoglutarate
(R)-3-Hydroxybutanoate
Alanine, aspartate and glutamate metabolism
K00019
Acetoacetyl-CoA
K01907
MELLADRAFT_3
Acetyl-CoA
Synthesis and degradation of ketone bodies
MELLADRAFT_4...
3-Butyn-1-al
3.1.2.11
(S)-3-Hydroxy-3-methylglutaryl-CoA
4.2.1.27
3-Butyn-1-ol
3-Butynoate
Acetoacetate
MELLADRAFT_5...
Fatty acid degradation
00650 11/1/17
(c) Kanehisa Laboratories

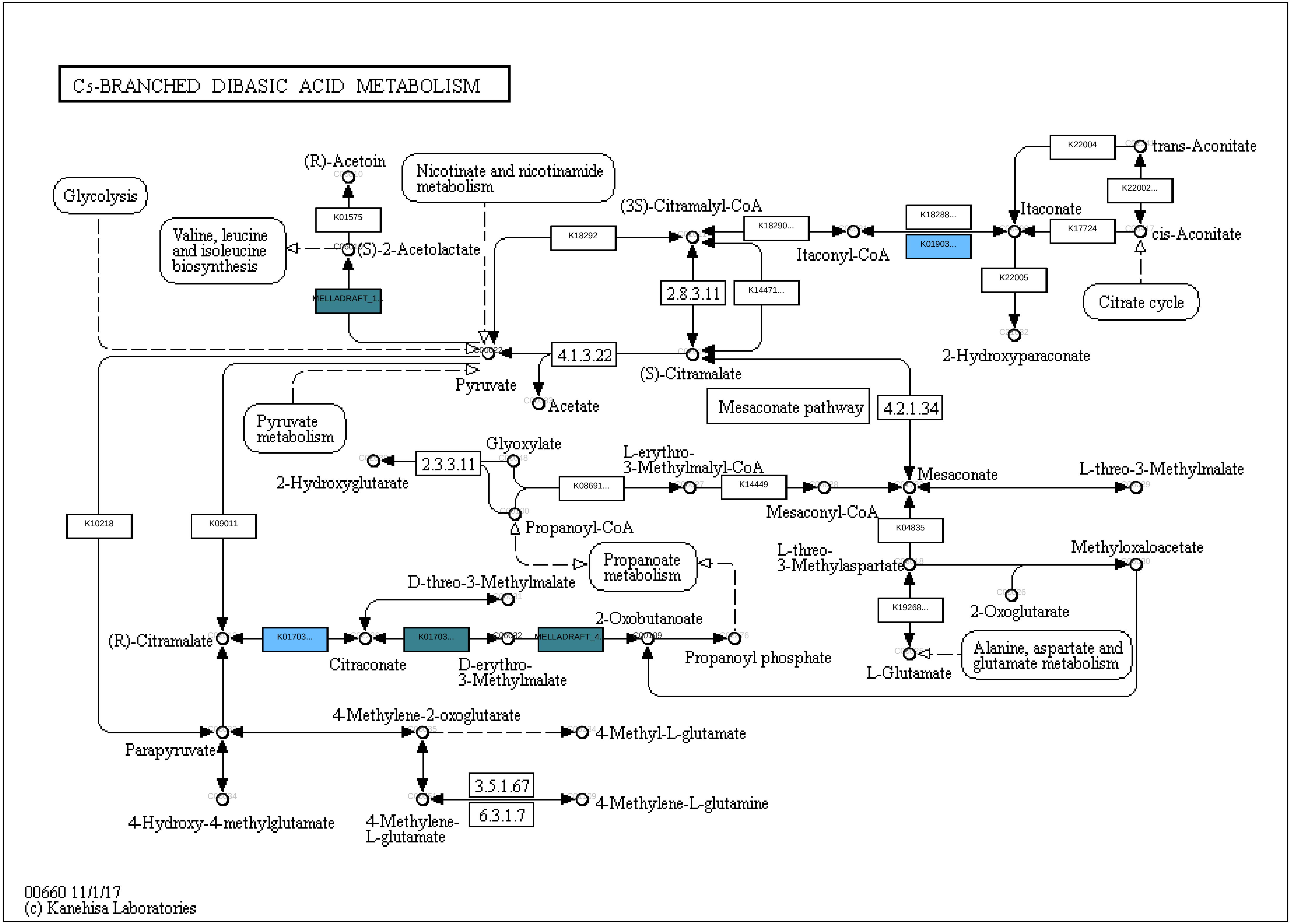

C5-BRANCHED DIBASIC ACID METABOLISM
(R)-Acetoin
Glycolysis
Nicotinate and nicotinamide metabolism
K01575
Valine, leucine and isoleucine biosynthesis
(S)-2-Acetolactate
MELLADRAFT_1...
K18292
(3S)-Citramalyl-CoA
K18290...
Itaconyl-CoA
K18288...
K01903...
K22004
trans-Aconitate
K22002...
Itaconate
K17724
cis-Aconitate
K22005
Citrate cycle
2-Hydroxyparaconate
2.8.3.11
K14471...
4.1.3.22
Pyruvate
Acetate
(S)-Citramalate
Mesaconate pathway
4.2.1.34
Pyruvate metabolism
Glyoxylate
2.3.3.11
2-Hydroxyglutarate
L-erythro-3-Methylmalyl-CoA
K08691...
K14449
Mesaconate
L-threo-3-Methylmalate
Mesaconyl-CoA
Propanoyl-CoA
K10218
K09011
K04835
L-threo-3-Methylaspartate
Methyloxaloacetate
Propanoate metabolism
D-threo-3-Methylmalate
2-Oxoglutarate
K19268...
2-Oxobutanoate
(R)-Citramalate
K01703...
Citraconate
K01703...
D-erythro-3-Methylmalate
MELLADRAFT_4...
Propanoyl phosphate
L-Glutamate
Alanine, aspartate and glutamate metabolism
4-Methylene-2-oxoglutarate
4-Methyl-L-glutamate
Parapyruvate
3.5.1.67
6.3.1.7
4-Methylene-L-glutamine
4-Hydroxy-4-methylglutamate
4-Methylene-L-glutamate
00660 11/1/17
(c) Kanehisa Laboratories

# 2. Energy metabolism

| MAP | PATHWAY |
|---|---|
| **190** | Oxidative phosphorylation |
| **680** | Methane metabolism |
| **910** | Nitrogen metabolism |
| **920** | Sulfur metabolism |

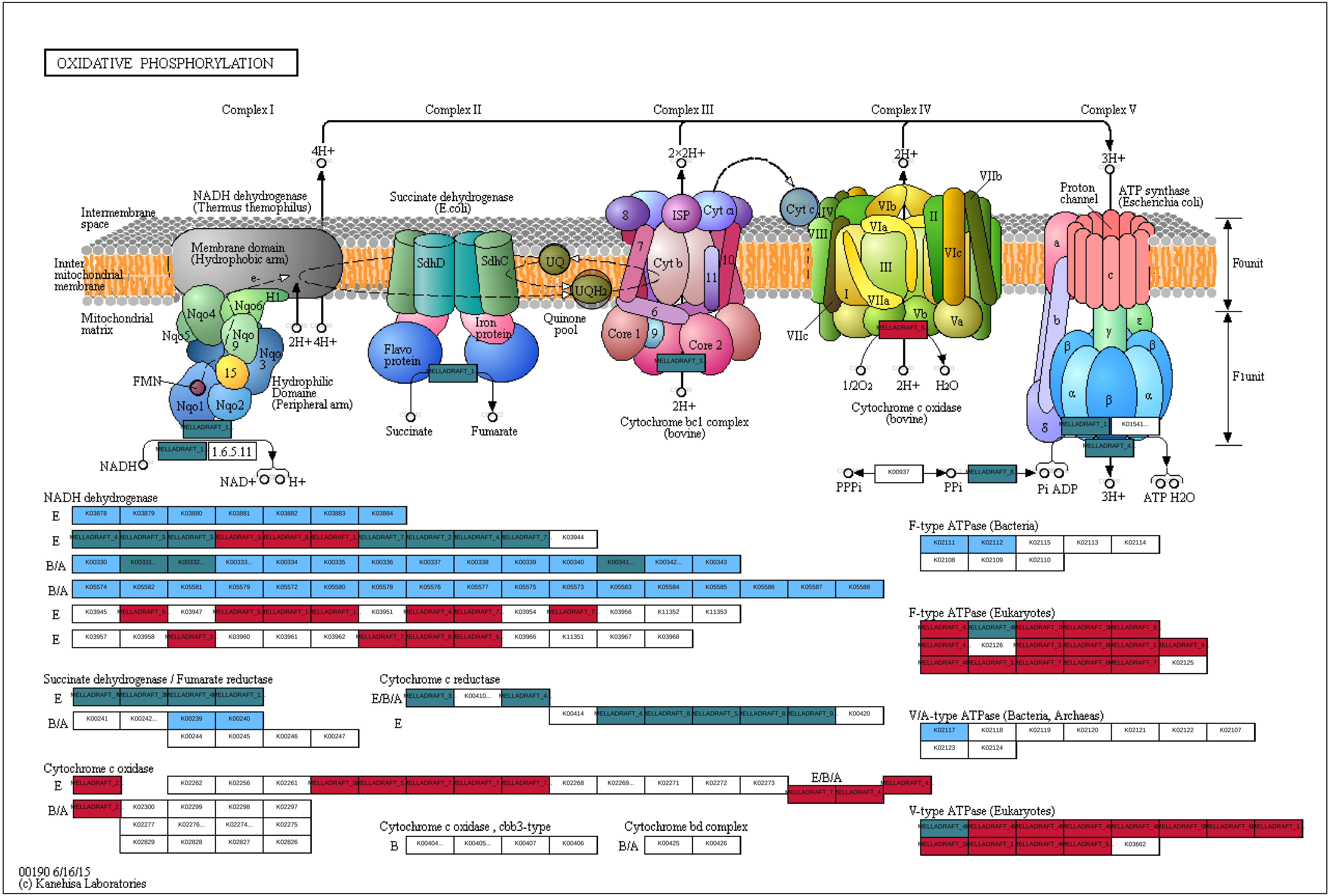

OXIDATIVE PHOSPHORYLATION
Complex I
Complex II
Complex III
Complex IV
Complex V
4H+
2×2H+
2H+
3H+
NADH dehydrogenase (Thermus themophilus)
Succinate dehydrogenase (E.coli)
Intermembrane space
Inner mitochondrial membrane
Mitochondrial matrix
Membrane domain (Hydrophobic arm)
Hydrophilic Domaine (Peripheral arm)
FMN
Nqo1
Nqo2
Nqo3
Nqo4
Nqo5
Nqo6
Nqo9
15
H1
e-
2H+ 4H+
1.6.5.11
NADH
NAD+
H+
SdhD
SdhC
Flavo protein
Iron protein
Succinate
Fumarate
UQ
UQH2
Quinone pool
Cyt b
Cyt c1
ISP
Core 1
Core 2
Cytochrome bc1 complex (bovine)
Cyt c
VIIb
VIb
VIa
III
VIc
VIIa
Vb
Va
VIIc
VIII
IV
II
I
1/2O2
H2O
Cytochrome c oxidase (bovine)
Proton channel
ATP synthase (Escherichia coli)
Fo unit
F1 unit
a
b
c
γ
ε
α
β
δ
PPPi
PPi
Pi ADP
ATP H2O
K00937
NADH dehydrogenase
Succinate dehydrogenase / Fumarate reductase
Cytochrome c reductase
Cytochrome c oxidase
Cytochrome c oxidase , cbb3-type
Cytochrome bd complex
F-type ATPase (Bacteria)
F-type ATPase (Eukaryotes)
V/A-type ATPase (Bacteria, Archaeas)
V-type ATPase (Eukaryotes)
00190 6/16/15
(c) Kanehisa Laboratories

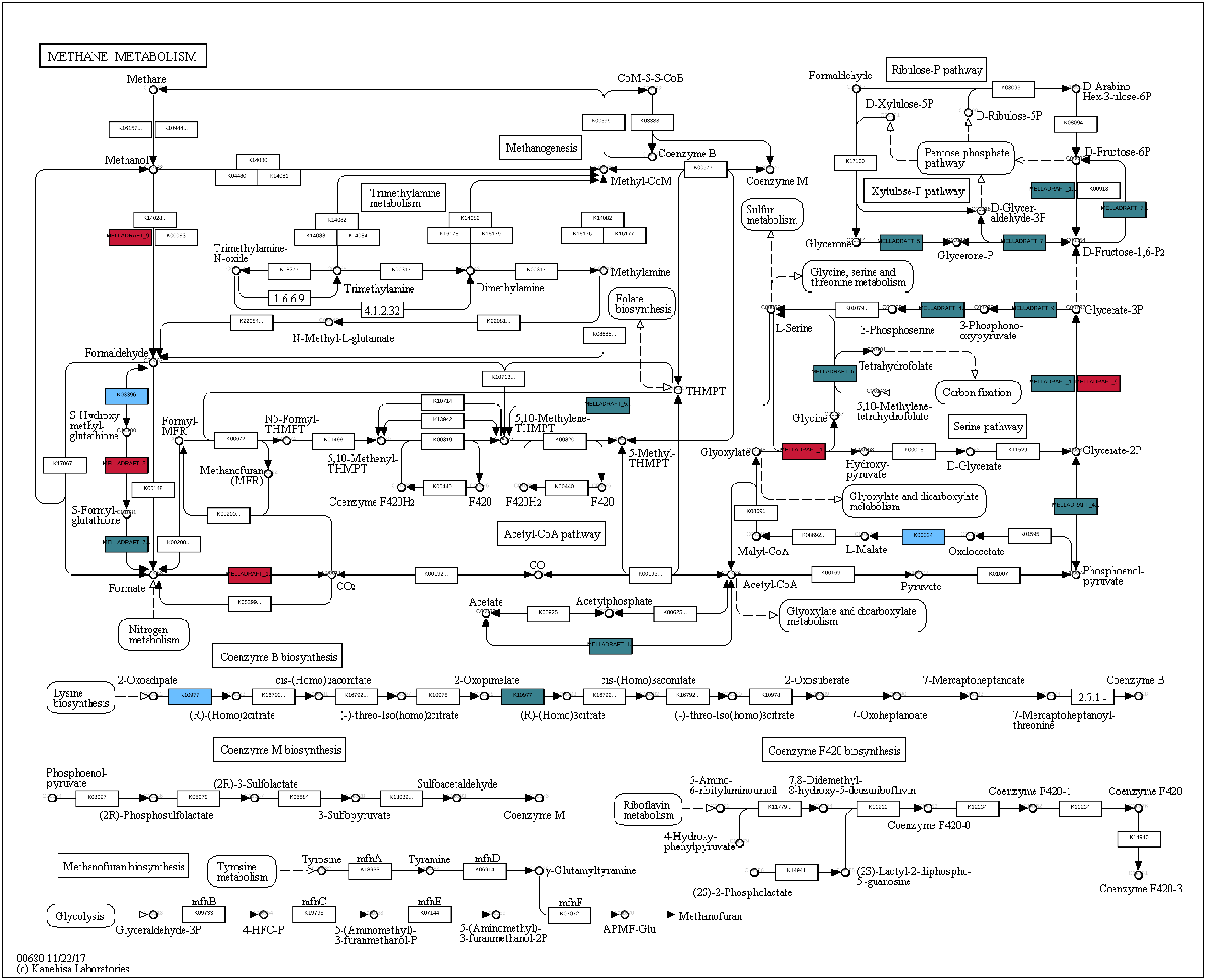
METHANE METABOLISM
Methane
CoM-S-S-CoB
Formaldehyde
Ribulose-P pathway
D-Arabino-Hex-3-ulose-6P
D-Xylulose-5P
D-Ribulose-5P
Methanogenesis
Coenzyme B
Methanol
Methyl-CoM
Coenzyme M
Pentose phosphate pathway
D-Fructose-6P
Trimethylamine metabolism
Sulfur metabolism
Xylulose-P pathway
D-Glyceraldehyde-3P
Trimethylamine-N-oxide
Glycerone
Glycerone-P
D-Fructose-1,6-P2
Trimethylamine
Dimethylamine
Methylamine
Glycine, serine and threonine metabolism
1.6.6.9
4.1.2.32
Folate biosynthesis
Glycerate-3P
N-Methyl-L-glutamate
L-Serine
3-Phosphoserine
3-Phosphonooxypyruvate
Formaldehyde
Tetrahydrofolate
THMPT
Carbon fixation
S-Hydroxymethylglutathione
Formyl-MFR
N5-Formyl-THMPT
5,10-Methylene-THMPT
5,10-Methylenetetrahydrofolate
Glycine
Serine pathway
Methanofuran (MFR)
5,10-Methenyl-THMPT
5-Methyl-THMPT
Glyoxylate
Hydroxypyruvate
D-Glycerate
Glycerate-2P
Coenzyme F420H2
F420
F420H2
F420
Glyoxylate and dicarboxylate metabolism
S-Formylglutathione
Acetyl-CoA pathway
Malyl-CoA
L-Malate
Oxaloacetate
CO
Formate
CO2
Acetyl-CoA
Pyruvate
Phosphoenolpyruvate
Acetate
Acetylphosphate
Glyoxylate and dicarboxylate metabolism
Nitrogen metabolism
Coenzyme B biosynthesis
Lysine biosynthesis
2-Oxoadipate
cis-(Homo)2aconitate
2-Oxopimelate
cis-(Homo)3aconitate
2-Oxosuberate
7-Mercaptoheptanoate
Coenzyme B
2.7.1.-
(R)-(Homo)2citrate
(-)-threo-Iso(homo)2citrate
(R)-(Homo)3citrate
(-)-threo-Iso(homo)3citrate
7-Oxoheptanoate
7-Mercaptoheptanoylthreonine
Coenzyme M biosynthesis
Coenzyme F420 biosynthesis
Phosphoenolpyruvate
(2R)-3-Sulfolactate
Sulfoacetaldehyde
(2R)-Phosphosulfolactate
3-Sulfopyruvate
Coenzyme M
5-Amino-6-ribitylaminouracil
7,8-Didemethyl-8-hydroxy-5-deazariboflavin
Coenzyme F420-1
Coenzyme F420
Riboflavin metabolism
Coenzyme F420-0
4-Hydroxyphenylpyruvate
Methanofuran biosynthesis
Tyrosine metabolism
Tyrosine
mfnA
Tyramine
mfnD
γ-Glutamyltyramine
(2S)-Lactyl-2-diphospho-5'-guanosine
(2S)-2-Phospholactate
Coenzyme F420-3
Glycolysis
mfnB
mfnC
mfnE
mfnF
Methanofuran
Glyceraldehyde-3P
4-HFC-P
5-(Aminomethyl)-3-furanmethanol-P
5-(Aminomethyl)-3-furanmethanol-2P
APMF-Glu
00680 11/22/17
(c) Kanehisa Laboratories

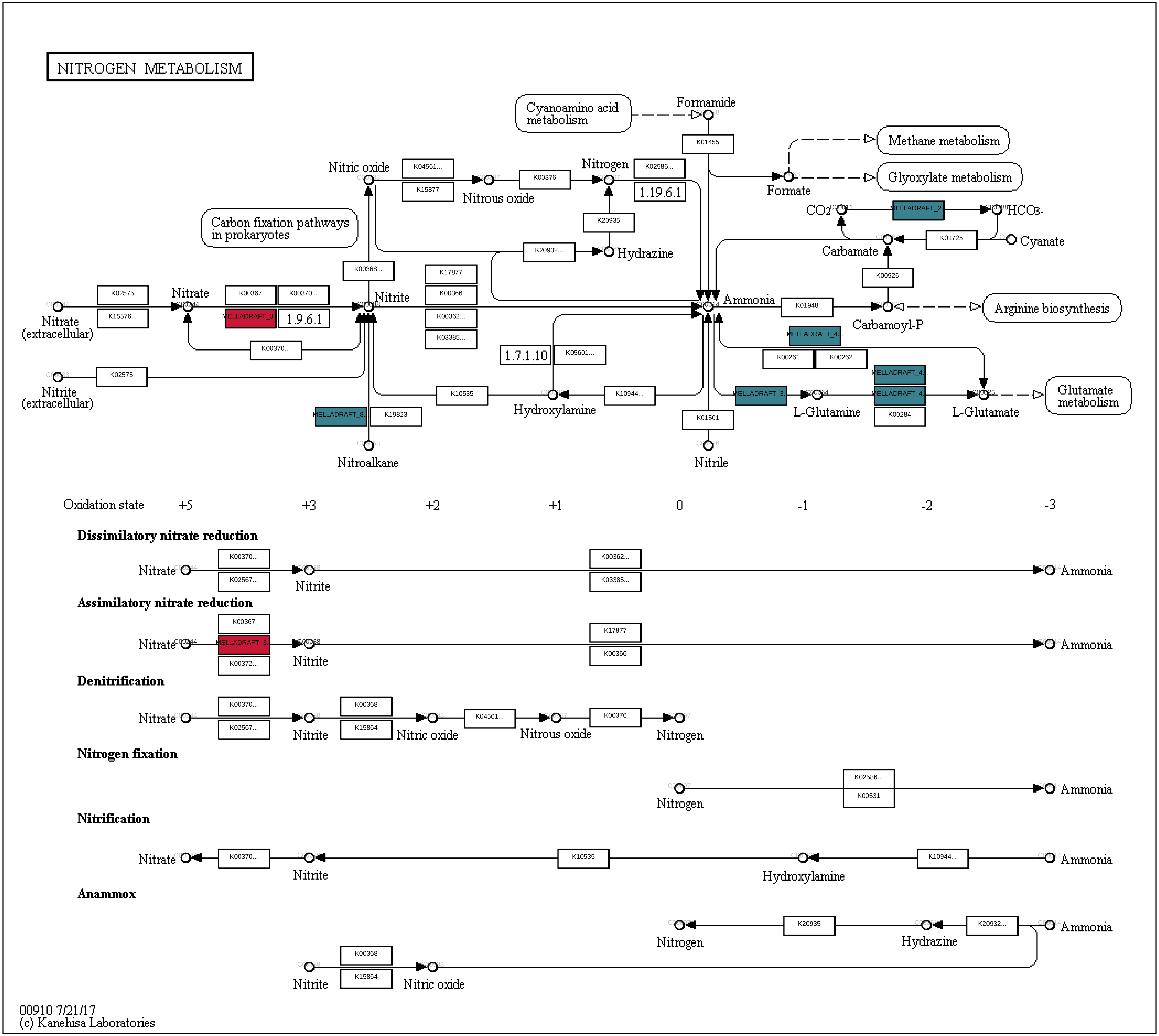

NITROGEN METABOLISM
Cyanoamino acid metabolism
Formamide
K01455
Methane metabolism
Glyoxylate metabolism
Formate
Nitric oxide
K04561...
K15877
Nitrous oxide
K00376
Nitrogen
K02586...
1.19.6.1
K20935
CO2
MELLADRAFT_2...
HCO3-
Carbamate
K01725
Cyanate
Carbon fixation pathways in prokaryotes
K20932...
Hydrazine
K00368...
K17877
K00366
K00362...
K03385...
K00926
K02575
K15576...
Nitrate
K00367
K00370...
MELLADRAFT_3...
1.9.6.1
Nitrite
Ammonia
K01948
Carbamoyl-P
Arginine biosynthesis
Nitrate (extracellular)
K00370...
1.7.1.10
K05601...
MELLADRAFT_4...
K00261
K00262
Nitrite (extracellular)
K02575
K10535
K10944...
Hydroxylamine
MELLADRAFT_3...
L-Glutamine
MELLADRAFT_4...
MELLADRAFT_4...
K00284
L-Glutamate
Glutamate metabolism
MELLADRAFT_8...
K19823
K01501
Nitroalkane
Nitrile
Oxidation state
+5
+3
+2
+1
0
-1
-2
-3
Dissimilatory nitrate reduction
Nitrate
K00370...
K02567...
Nitrite
K00362...
K03385...
Ammonia
Assimilatory nitrate reduction
Nitrate
K00367
MELLADRAFT_3...
K00372...
Nitrite
K17877
K00366
Ammonia
Denitrification
Nitrate
K00370...
K02567...
Nitrite
K00368
K15864
Nitric oxide
K04561...
Nitrous oxide
K00376
Nitrogen
Nitrogen fixation
Nitrogen
K02586...
K00531
Ammonia
Nitrification
Nitrate
K00370...
Nitrite
K10535
Hydroxylamine
K10944...
Ammonia
Anammox
Nitrogen
K20935
Hydrazine
K20932...
Ammonia
Nitrite
K00368
K15864
Nitric oxide
00910 7/21/17
(c) Kanehisa Laboratories

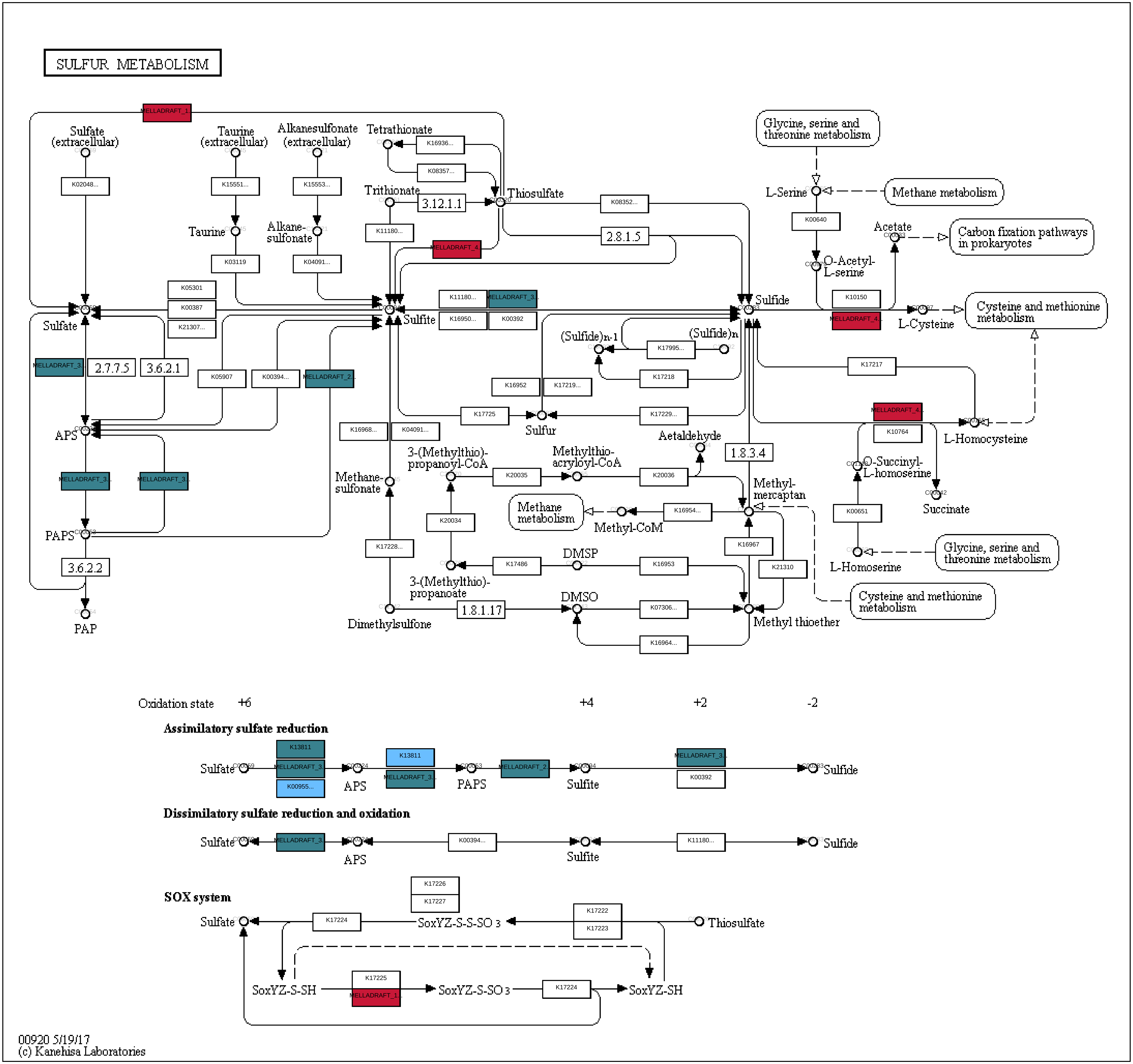
SULFUR METABOLISM
Sulfate (extracellular)
Taurine (extracellular)
Alkanesulfonate (extracellular)
Tetrathionate
Trithionate
Thiosulfate
Taurine
Alkane-sulfonate
Sulfate
Sulfite
Sulfide
APS
PAPS
PAP
3.12.1.1
2.8.1.5
2.7.7.5
3.6.2.1
3.6.2.2
1.8.3.4
1.8.1.17
(Sulfide)n-1
(Sulfide)n
Sulfur
Methane-sulfonate
3-(Methylthio)-propanoyl-CoA
Methylthio-acryloyl-CoA
Aetaldehyde
Methyl-mercaptan
Methane metabolism
Methyl-CoM
DMSP
3-(Methylthio)-propanoate
DMSO
Dimethylsulfone
Methyl thioether
Glycine, serine and threonine metabolism
L-Serine
Methane metabolism
Acetate
Carbon fixation pathways in prokaryotes
O-Acetyl-L-serine
L-Cysteine
Cysteine and methionine metabolism
L-Homocysteine
O-Succinyl-L-homoserine
Succinate
L-Homoserine
Glycine, serine and threonine metabolism
Cysteine and methionine metabolism
Oxidation state +6 +4 +2 -2
Assimilatory sulfate reduction
Sulfate APS PAPS Sulfite Sulfide
Dissimilatory sulfate reduction and oxidation
Sulfate APS Sulfite Sulfide
SOX system
Sulfate SoxYZ-S-S-SO3 Thiosulfate
SoxYZ-S-SH SoxYZ-S-SO3 SoxYZ-SH
00920 5/19/17
(c) Kanehisa Laboratories

# 3. Lipid metabolism

| MAP | PATHWAY |
|---|---|
| **61** | Fatty acid biosynthesis |
| **62** | Fatty acid elongation |
| **71** | Fatty acid degradation |
| **72** | Synthesis and degradation of ketone bodies |
| **100** | Steroid biosynthesis |
| **561** | Glycerolipid metabolism |
| **564** | Glycerophospholipid metabolism |
| **565** | Ether lipid metabolism |
| **590** | Arachidonic acid metabolism |
| **592** | alpha-Linolenic acid metabolism |
| **600** | Sphingolipid metabolism |

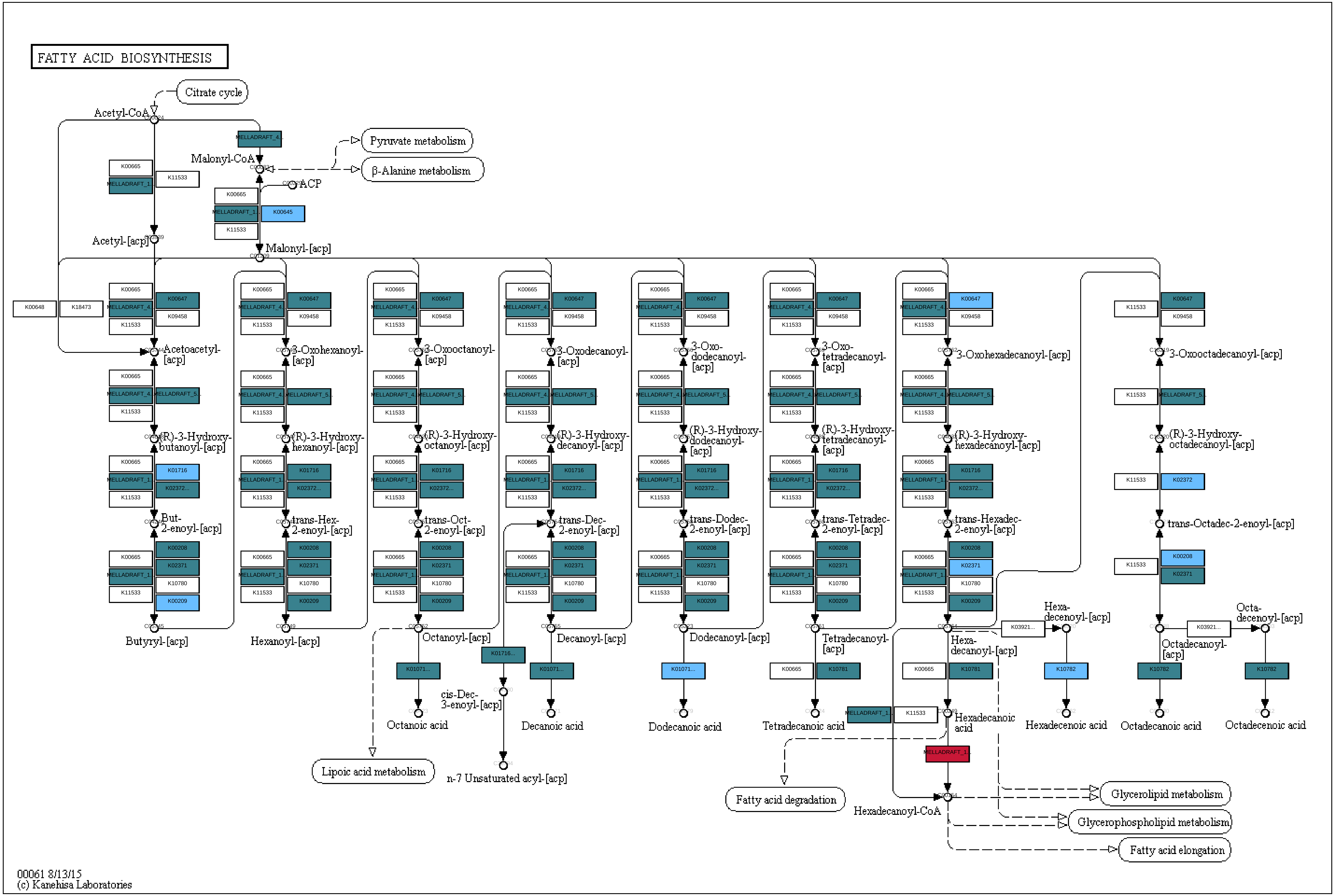
FATTY ACID BIOSYNTHESIS
Citrate cycle
Acetyl-CoA
Pyruvate metabolism
β-Alanine metabolism
Malonyl-CoA
ACP
Acetyl-[acp]
Malonyl-[acp]
K00665
K11533
K00645
K00647
K09458
K00648
K18473
K01716
K02372...
K00208
K02371
K10780
K00209
K01071...
K01716...
K03921...
K10781
K10782
Acetoacetyl-[acp]
3-Oxohexanoyl-[acp]
3-Oxooctanoyl-[acp]
3-Oxodecanoyl-[acp]
3-Oxo-dodecanoyl-[acp]
3-Oxo-tetradecanoyl-[acp]
3-Oxohexadecanoyl-[acp]
3-Oxooctadecanoyl-[acp]
(R)-3-Hydroxy-butanoyl-[acp]
(R)-3-Hydroxy-hexanoyl-[acp]
(R)-3-Hydroxy-octanoyl-[acp]
(R)-3-Hydroxy-decanoyl-[acp]
(R)-3-Hydroxy-dodecanoyl-[acp]
(R)-3-Hydroxy-tetradecanoyl-[acp]
(R)-3-Hydroxy-hexadecanoyl-[acp]
(R)-3-Hydroxy-octadecanoyl-[acp]
But-2-enoyl-[acp]
trans-Hex-2-enoyl-[acp]
trans-Oct-2-enoyl-[acp]
trans-Dec-2-enoyl-[acp]
trans-Dodec-2-enoyl-[acp]
trans-Tetradec-2-enoyl-[acp]
trans-Hexadec-2-enoyl-[acp]
trans-Octadec-2-enoyl-[acp]
Butyryl-[acp]
Hexanoyl-[acp]
Octanoyl-[acp]
Decanoyl-[acp]
Dodecanoyl-[acp]
Tetradecanoyl-[acp]
Hexa-decanoyl-[acp]
Hexa-decenoyl-[acp]
Octadecanoyl-[acp]
Octa-decenoyl-[acp]
cis-Dec-3-enoyl-[acp]
Octanoic acid
Decanoic acid
Dodecanoic acid
Tetradecanoic acid
Hexadecanoic acid
Hexadecenoic acid
Octadecanoic acid
Octadecenoic acid
Lipoic acid metabolism
n-7 Unsaturated acyl-[acp]
Fatty acid degradation
Hexadecanoyl-CoA
Glycerolipid metabolism
Glycerophospholipid metabolism
Fatty acid elongation
00061 8/13/15
(c) Kanehisa Laboratories

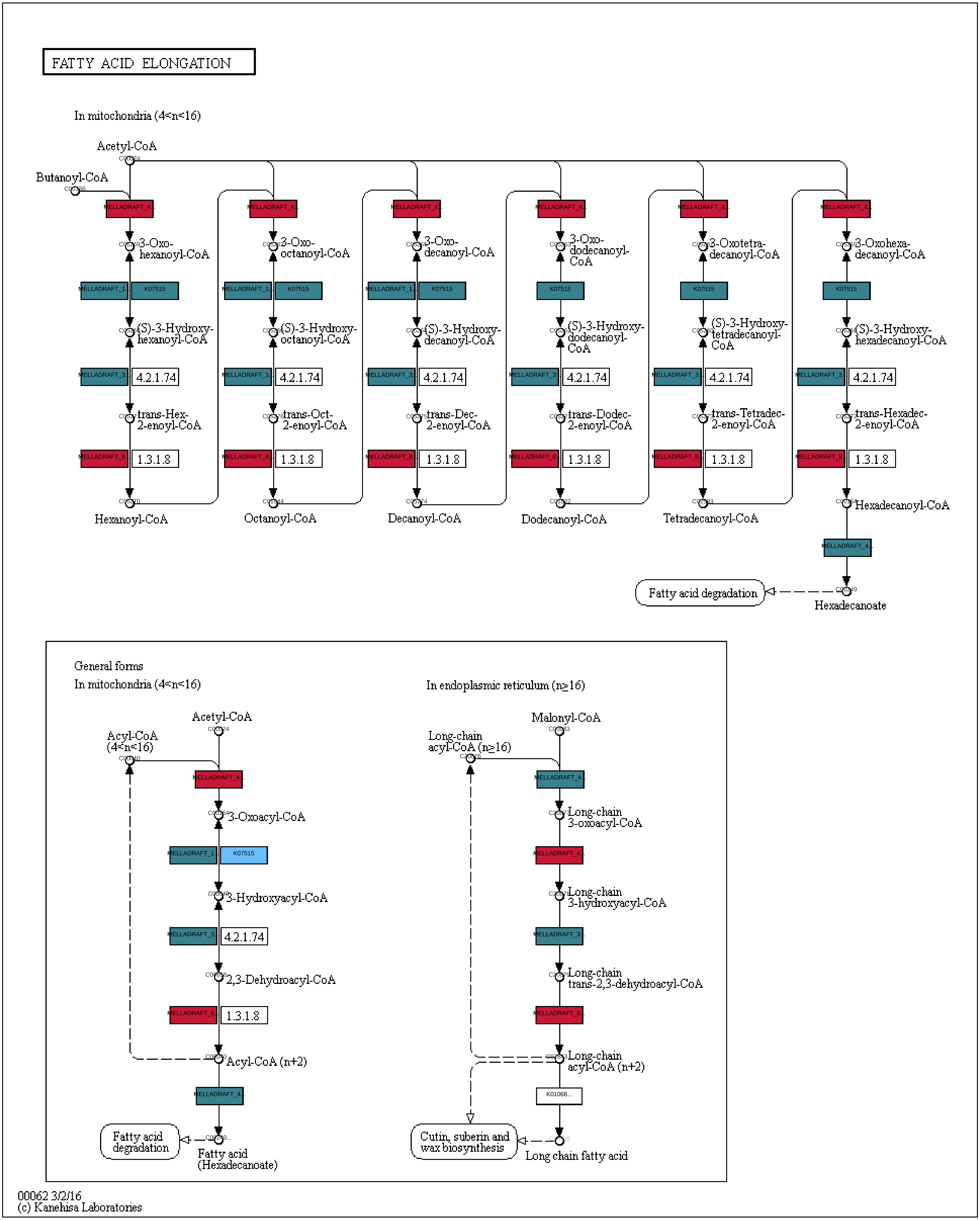

FATTY ACID ELONGATION
In mitochondria (4<n<16)
Acetyl-CoA
Butanoyl-CoA
3-Oxo-hexanoyl-CoA
3-Oxo-octanoyl-CoA
3-Oxo-decanoyl-CoA
3-Oxo-dodecanoyl-CoA
3-Oxotetra-decanoyl-CoA
3-Oxohexa-decanoyl-CoA
K07515
(S)-3-Hydroxy-hexanoyl-CoA
(S)-3-Hydroxy-octanoyl-CoA
(S)-3-Hydroxy-decanoyl-CoA
(S)-3-Hydroxy-dodecanoyl-CoA
(S)-3-Hydroxy-tetradecanoyl-CoA
(S)-3-Hydroxy-hexadecanoyl-CoA
4.2.1.74
trans-Hex-2-enoyl-CoA
trans-Oct-2-enoyl-CoA
trans-Dec-2-enoyl-CoA
trans-Dodec-2-enoyl-CoA
trans-Tetradec-2-enoyl-CoA
trans-Hexadec-2-enoyl-CoA
1.3.1.8
Hexanoyl-CoA
Octanoyl-CoA
Decanoyl-CoA
Dodecanoyl-CoA
Tetradecanoyl-CoA
Hexadecanoyl-CoA
Fatty acid degradation
Hexadecanoate
General forms
In mitochondria (4<n<16)
In endoplasmic reticulum (n≥16)
Acyl-CoA (4<n<16)
3-Oxoacyl-CoA
3-Hydroxyacyl-CoA
2,3-Dehydroacyl-CoA
Acyl-CoA (n+2)
Fatty acid degradation
Fatty acid (Hexadecanoate)
Malonyl-CoA
Long-chain acyl-CoA (n≥16)
Long-chain 3-oxoacyl-CoA
Long-chain 3-hydroxyacyl-CoA
Long-chain trans-2,3-dehydroacyl-CoA
Long-chain acyl-CoA (n+2)
K01068...
Cutin, suberin and wax biosynthesis
Long chain fatty acid
00062 3/2/16
(c) Kanehisa Laboratories

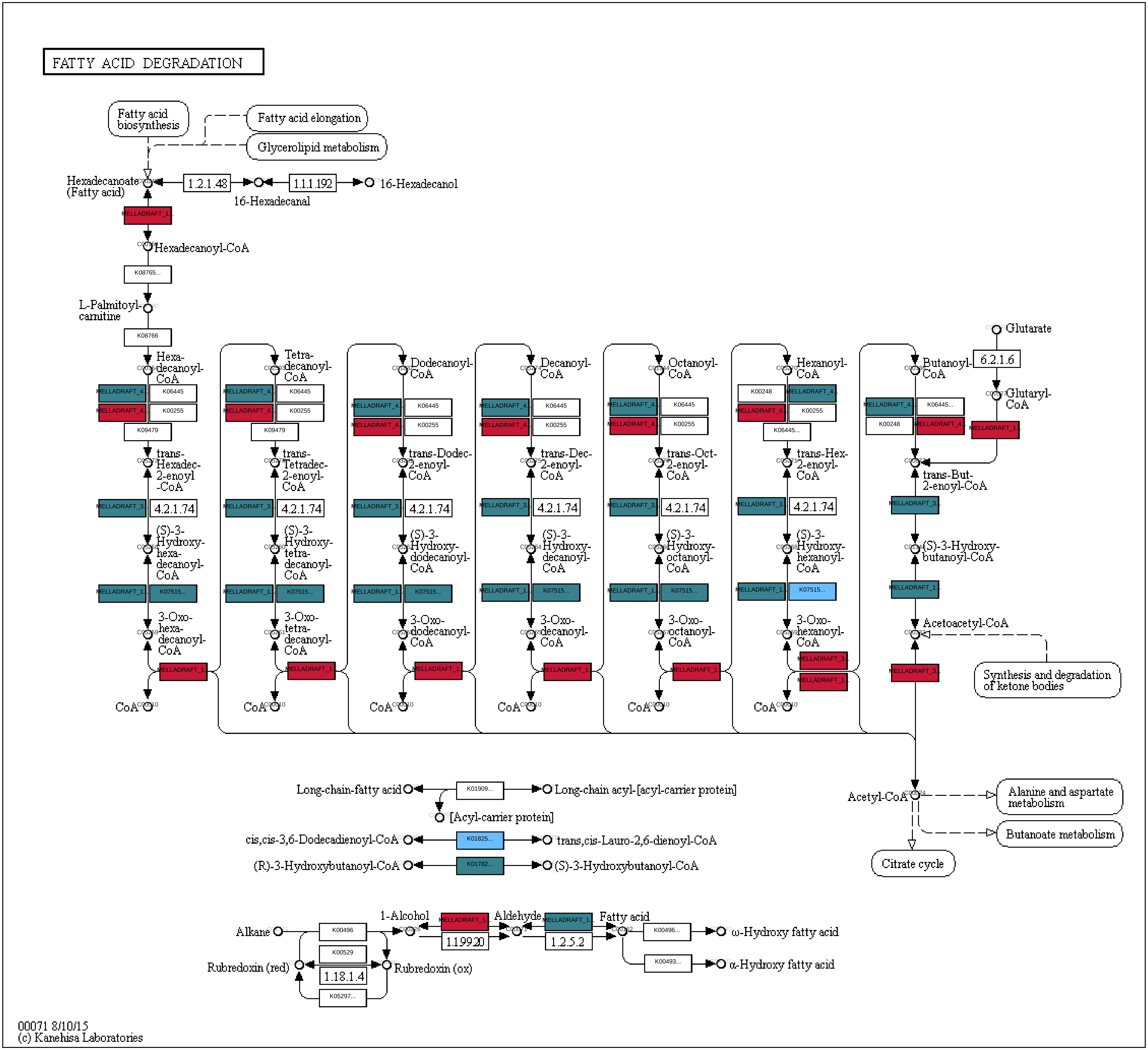
FATTY ACID DEGRADATION
Fatty acid biosynthesis
Fatty acid elongation
Glycerolipid metabolism
Hexadecanoate (Fatty acid)
1.2.1.48
16-Hexadecanal
1.1.1.192
16-Hexadecanol
Hexadecanoyl-CoA
K08765...
L-Palmitoyl-carnitine
K08766
Glutarate
6.2.1.6
Glutaryl-CoA
Hexa-decanoyl-CoA
Tetra-decanoyl-CoA
Dodecanoyl-CoA
Decanoyl-CoA
Octanoyl-CoA
Hexanoyl-CoA
Butanoyl-CoA
K06445
K00255
K09479
K00248
K06445...
trans-Hexadec-2-enoyl-CoA
trans-Tetradec-2-enoyl-CoA
trans-Dodec-2-enoyl-CoA
trans-Dec-2-enoyl-CoA
trans-Oct-2-enoyl-CoA
trans-Hex-2-enoyl-CoA
trans-But-2-enoyl-CoA
4.2.1.74
(S)-3-Hydroxy-hexa-decanoyl-CoA
(S)-3-Hydroxy-tetra-decanoyl-CoA
(S)-3-Hydroxy-dodecanoyl-CoA
(S)-3-Hydroxy-decanoyl-CoA
(S)-3-Hydroxy-octanoyl-CoA
(S)-3-Hydroxy-hexanoyl-CoA
(S)-3-Hydroxy-butanoyl-CoA
K07515...
3-Oxo-hexa-decanoyl-CoA
3-Oxo-tetra-decanoyl-CoA
3-Oxo-dodecanoyl-CoA
3-Oxo-decanoyl-CoA
3-Oxo-octanoyl-CoA
3-Oxo-hexanoyl-CoA
Acetoacetyl-CoA
Synthesis and degradation of ketone bodies
CoA
Acetyl-CoA
Alanine and aspartate metabolism
Butanoate metabolism
Citrate cycle
Long-chain-fatty acid
K01909...
Long-chain acyl-[acyl-carrier protein]
[Acyl-carrier protein]
cis,cis-3,6-Dodecadienoyl-CoA
K01825...
trans,cis-Lauro-2,6-dienoyl-CoA
(R)-3-Hydroxybutanoyl-CoA
K01782...
(S)-3-Hydroxybutanoyl-CoA
Alkane
K00496
K00529
1.18.1.4
K05297...
1-Alcohol
1.1.99.20
Aldehyde
1.2.5.2
Fatty acid
K00496...
ω-Hydroxy fatty acid
K00493...
α-Hydroxy fatty acid
Rubredoxin (red)
Rubredoxin (ox)
00071 8/10/15
(c) Kanehisa Laboratories

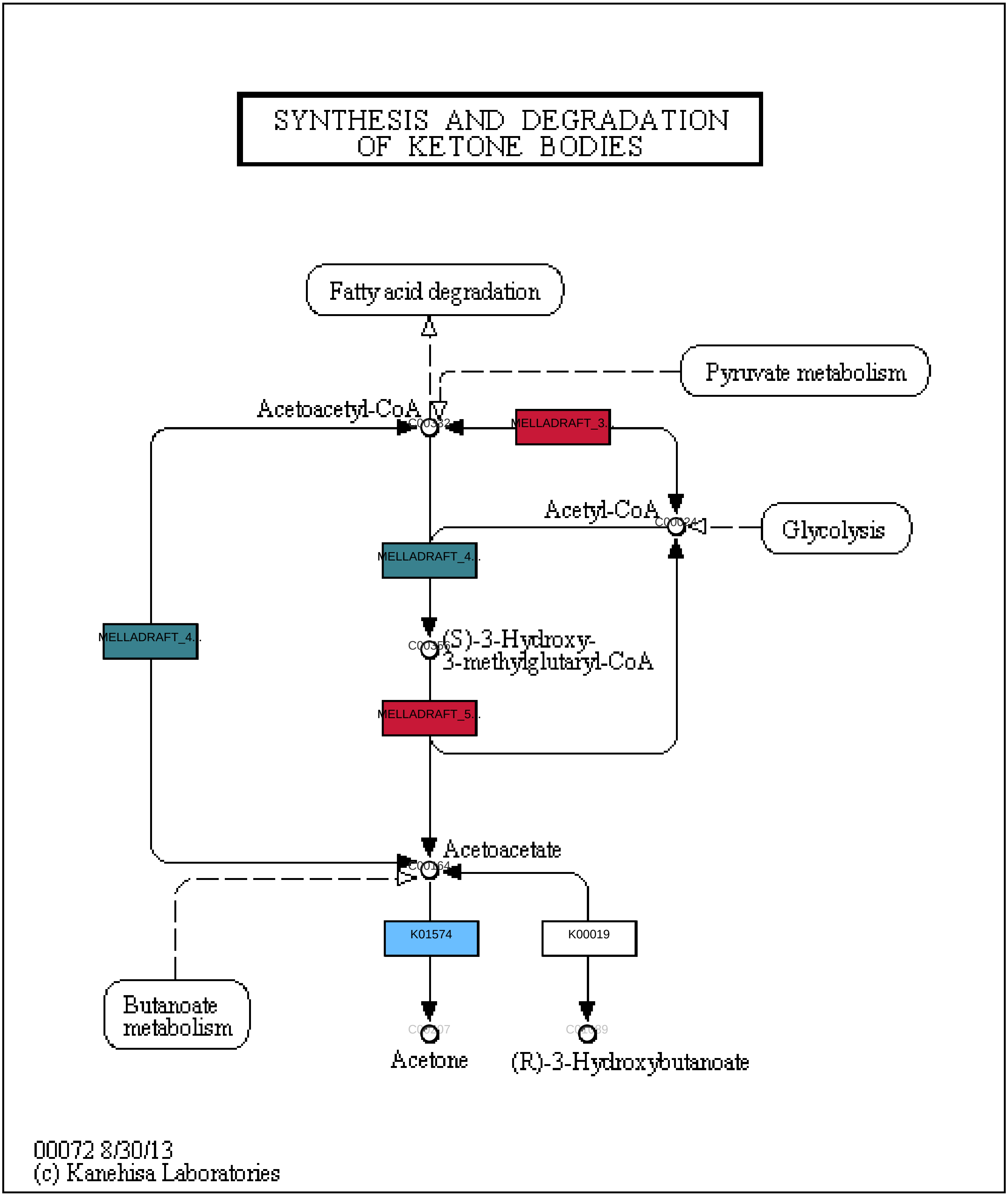

SYNTHESIS AND DEGRADATION OF KETONE BODIES
Fatty acid degradation
Pyruvate metabolism
Acetoacetyl-CoA
MELLADRAFT_3...
Acetyl-CoA
Glycolysis
MELLADRAFT_4...
MELLADRAFT_4...
(S)-3-Hydroxy-3-methylglutaryl-CoA
MELLADRAFT_5...
Acetoacetate
K01574
K00019
Butanoate metabolism
Acetone
(R)-3-Hydroxybutanoate
00072 8/30/13
(c) Kanehisa Laboratories

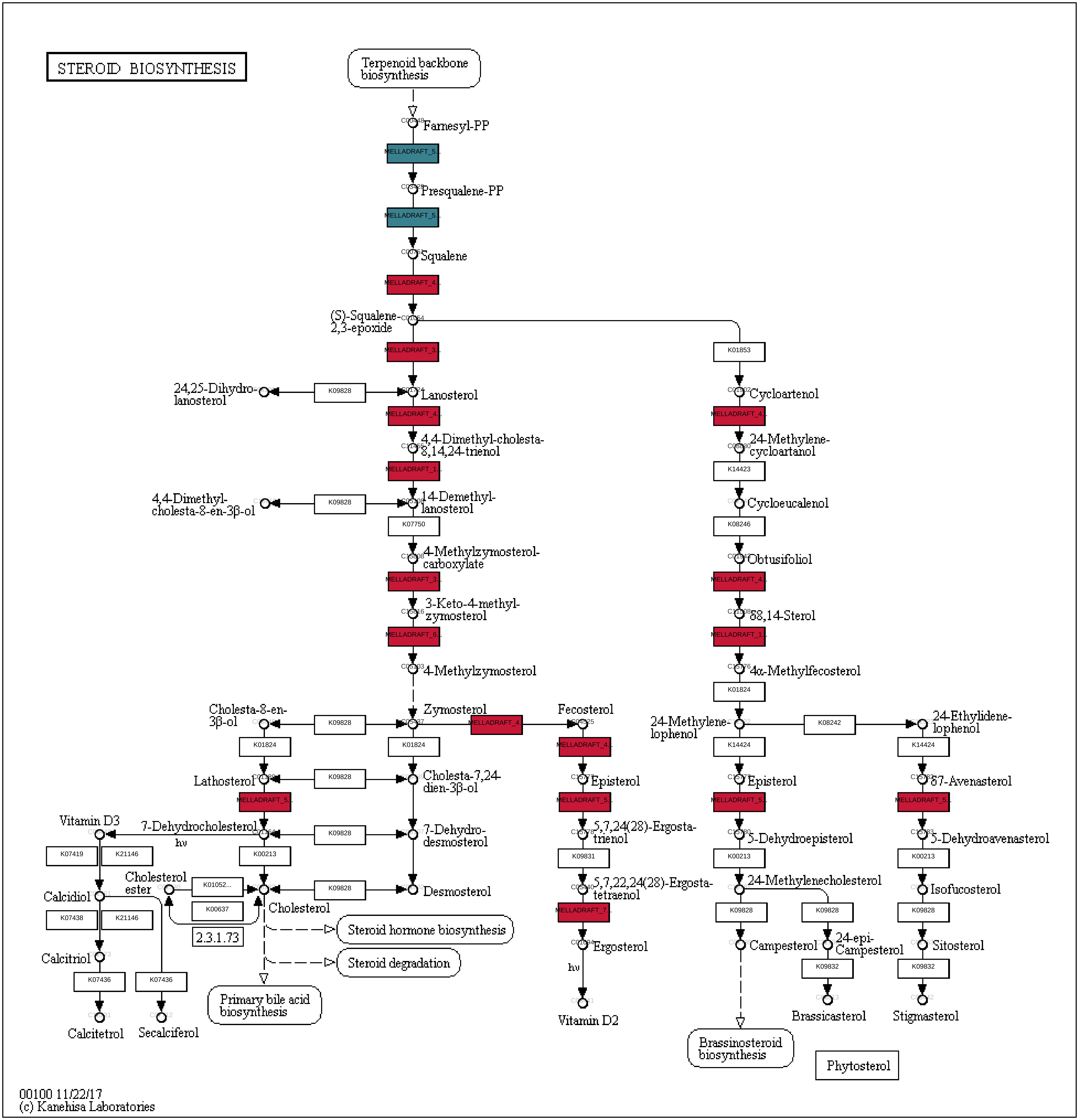
STEROID BIOSYNTHESIS
Terpenoid backbone biosynthesis
Farnesyl-PP
MELLADRAFT_5..
Presqualene-PP
MELLADRAFT_5..
Squalene
MELLADRAFT_4..
(S)-Squalene-2,3-epoxide
MELLADRAFT_3..
K01853
24,25-Dihydro-lanosterol
K09828
Lanosterol
Cycloartenol
MELLADRAFT_4..
4,4-Dimethyl-cholesta-8,14,24-trienol
24-Methylene-cycloartanol
MELLADRAFT_1..
K14423
4,4-Dimethyl-cholesta-8-en-3β-ol
K09828
14-Demethyl-lanosterol
Cycloeucalenol
K07750
K08246
4-Methylzymosterol-carboxylate
Obtusifoliol
MELLADRAFT_3..
MELLADRAFT_4..
3-Keto-4-methyl-zymosterol
δ8,14-Sterol
MELLADRAFT_6..
MELLADRAFT_1..
4-Methylzymosterol
4α-Methylfecosterol
K01824
Cholesta-8-en-3β-ol
K09828
Zymosterol
MELLADRAFT_4..
Fecosterol
24-Methylene-lophenol
K08242
24-Ethylidene-lophenol
K01824
K01824
MELLADRAFT_4..
K14424
K14424
Lathosterol
K09828
Cholesta-7,24-dien-3β-ol
Episterol
Episterol
δ7-Avenasterol
MELLADRAFT_5..
MELLADRAFT_5..
MELLADRAFT_5..
MELLADRAFT_5..
Vitamin D3
7-Dehydrocholesterol
hν
K09828
7-Dehydro-desmosterol
5,7,24(28)-Ergosta-trienol
5-Dehydroepisterol
5-Dehydroavenasterol
K07419
K21146
K00213
K09831
K00213
K00213
Cholesterol ester
K01052...
K09828
Desmosterol
5,7,22,24(28)-Ergosta-tetraenol
24-Methylenecholesterol
Isofucosterol
Calcidiol
K00637
Cholesterol
MELLADRAFT_7..
K09828
K09828
K09828
K07438
K21146
2.3.1.73
Steroid hormone biosynthesis
Campesterol
24-epi-Campesterol
Sitosterol
Calcitriol
Steroid degradation
Ergosterol
K07436
K07436
hν
K09832
K09832
Primary bile acid biosynthesis
Calcitetrol
Secalciferol
Vitamin D2
Brassicasterol
Stigmasterol
Brassinosteroid biosynthesis
Phytosterol
00100 11/22/17
(c) Kanehisa Laboratories

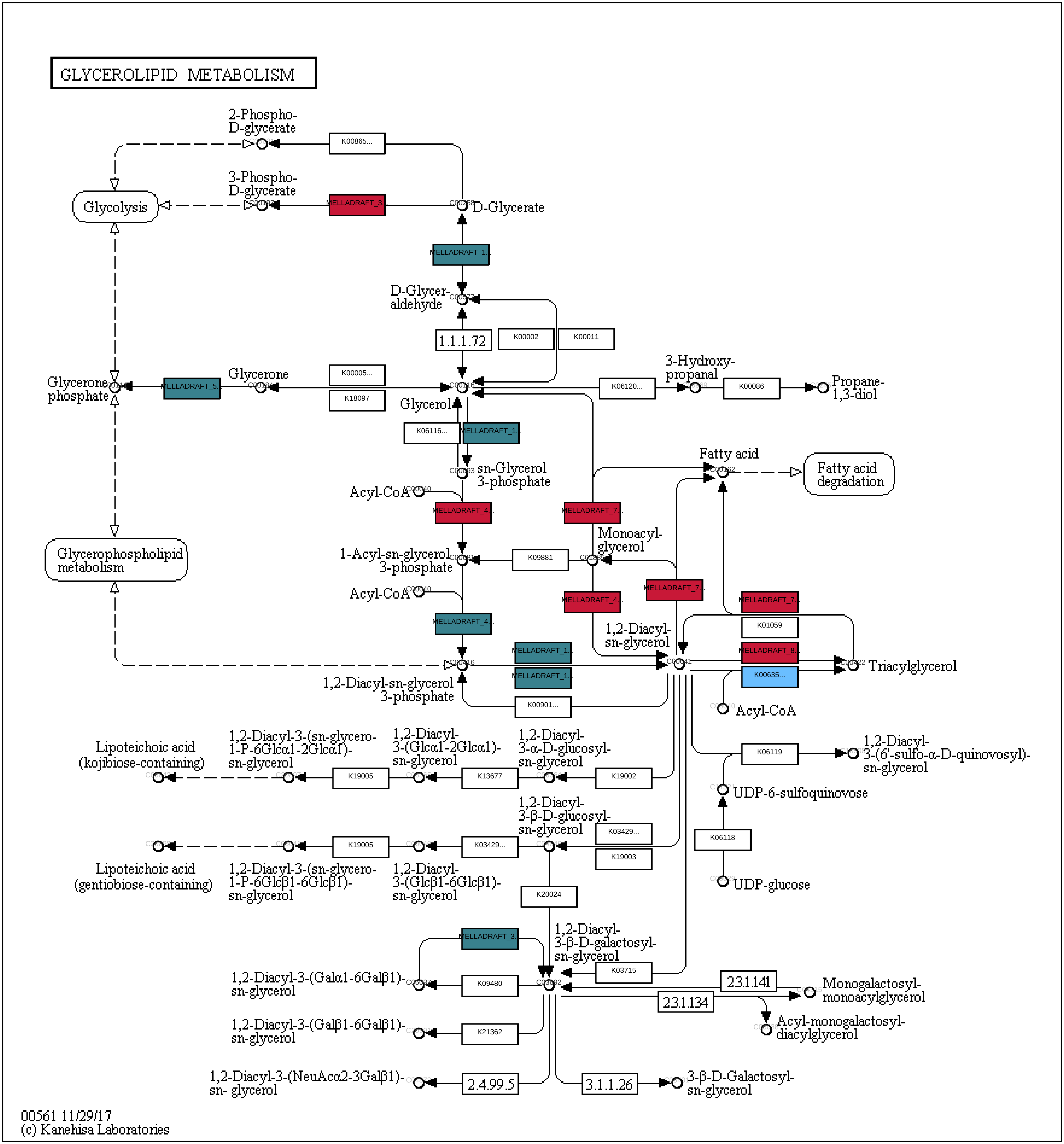
GLYCEROLIPID METABOLISM
2-Phospho-D-glycerate
K00865...
3-Phospho-D-glycerate
MELLADRAFT_3...
D-Glycerate
Glycolysis
MELLADRAFT_1...
D-Glycer-aldehyde
1.1.1.72
K00002
K00011
Glycerone phosphate
MELLADRAFT_5...
Glycerone
K00005...
K18097
Glycerol
K06120...
3-Hydroxy-propanal
K00086
Propane-1,3-diol
K06116...
sn-Glycerol 3-phosphate
Fatty acid
Fatty acid degradation
Acyl-CoA
MELLADRAFT_4...
MELLADRAFT_7...
Monoacyl-glycerol
Glycerophospholipid metabolism
1-Acyl-sn-glycerol 3-phosphate
K09881
1,2-Diacyl-sn-glycerol
K01059
MELLADRAFT_8...
K00635...
Triacylglycerol
1,2-Diacyl-sn-glycerol 3-phosphate
K00901...
Lipoteichoic acid (kojibiose-containing)
1,2-Diacyl-3-(sn-glycero-1-P-6Glcα1-2Glcα1)-sn-glycerol
1,2-Diacyl-3-(Glcα1-2Glcα1)-sn-glycerol
1,2-Diacyl-3-α-D-glucosyl-sn-glycerol
K19005
K13677
K19002
K06119
1,2-Diacyl-3-(6'-sulfo-α-D-quinovosyl)-sn-glycerol
UDP-6-sulfoquinovose
K06118
UDP-glucose
1,2-Diacyl-3-β-D-glucosyl-sn-glycerol
K03429...
K19003
Lipoteichoic acid (gentiobiose-containing)
1,2-Diacyl-3-(sn-glycero-1-P-6Glcβ1-6Glcβ1)-sn-glycerol
1,2-Diacyl-3-(Glcβ1-6Glcβ1)-sn-glycerol
K20024
1,2-Diacyl-3-β-D-galactosyl-sn-glycerol
K03715
2.3.1.141
Monogalactosyl-monoacylglycerol
2.3.1.134
Acyl-monogalactosyl-diacylglycerol
1,2-Diacyl-3-(Galα1-6Galβ1)-sn-glycerol
K09480
1,2-Diacyl-3-(Galβ1-6Galβ1)-sn-glycerol
K21362
1,2-Diacyl-3-(NeuAcα2-3Galβ1)-sn- glycerol
2.4.99.5
3.1.1.26
3-β-D-Galactosyl-sn-glycerol
00561 11/29/17
(c) Kanehisa Laboratories

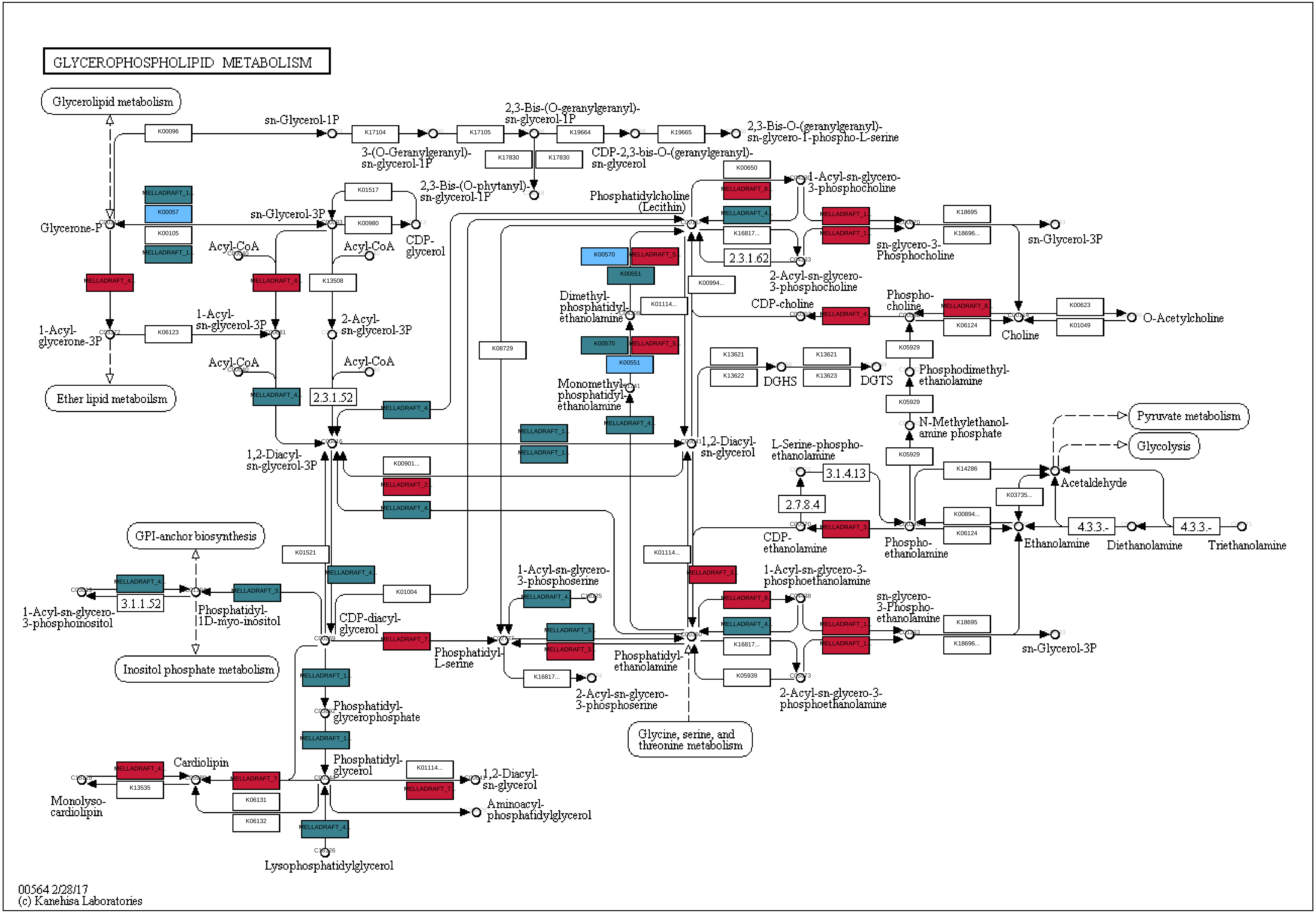
GLYCEROPHOSPHOLIPID METABOLISM
Glycerolipid metabolism
Ether lipid metaboilsm
GPI-anchor biosynthesis
Inositol phosphate metabolism
Glycine, serine, and threonine metabolism
Pyruvate metabolism
Glycolysis
sn-Glycerol-1P
3-(O-Geranylgeranyl)-sn-glycerol-1P
2,3-Bis-(O-geranylgeranyl)-sn-glycerol-1P
2,3-Bis-O-(geranylgeranyl)-sn-glycero-1-phospho-L-serine
CDP-2,3-bis-O-(geranylgeranyl)-sn-glycerol
2,3-Bis-(O-phytanyl)-sn-glycerol-1P
Glycerone-P
sn-Glycerol-3P
CDP-glycerol
Acyl-CoA
1-Acyl-glycerone-3P
1-Acyl-sn-glycerol-3P
2-Acyl-sn-glycerol-3P
1,2-Diacyl-sn-glycerol-3P
Phosphatidylcholine (Lecithin)
1-Acyl-sn-glycero-3-phosphocholine
sn-glycero-3-Phosphocholine
2-Acyl-sn-glycero-3-phosphocholine
CDP-choline
Phosphocholine
Choline
O-Acetylcholine
Dimethyl-phosphatidyl-ethanolamine
Monomethyl-phosphatidyl-ethanolamine
DGHS
DGTS
Phosphodimethyl-ethanolamine
N-Methylethanol-amine phosphate
1,2-Diacyl-sn-glycerol
L-Serine-phospho-ethanolamine
Acetaldehyde
CDP-ethanolamine
Phospho-ethanolamine
Ethanolamine
Diethanolamine
Triethanolamine
1-Acyl-sn-glycero-3-phosphoserine
1-Acyl-sn-glycero-3-phosphoethanolamine
sn-glycero-3-Phospho-ethanolamine
1-Acyl-sn-glycero-3-phosphoinositol
Phosphatidyl-1D-myo-inositol
CDP-diacyl-glycerol
Phosphatidyl-L-serine
Phosphatidyl-ethanolamine
2-Acyl-sn-glycero-3-phosphoserine
2-Acyl-sn-glycero-3-phosphoethanolamine
Phosphatidyl-glycerophosphate
Phosphatidyl-glycerol
Cardiolipin
Monolyso-cardiolipin
1,2-Diacyl-sn-glycerol
Aminoacyl-phosphatidylglycerol
Lysophosphatidylglycerol
2.3.1.62
2.3.1.52
3.1.4.13
2.7.8.4
4.3.3.-
3.1.1.52
00564 2/28/17
(c) Kanehisa Laboratories

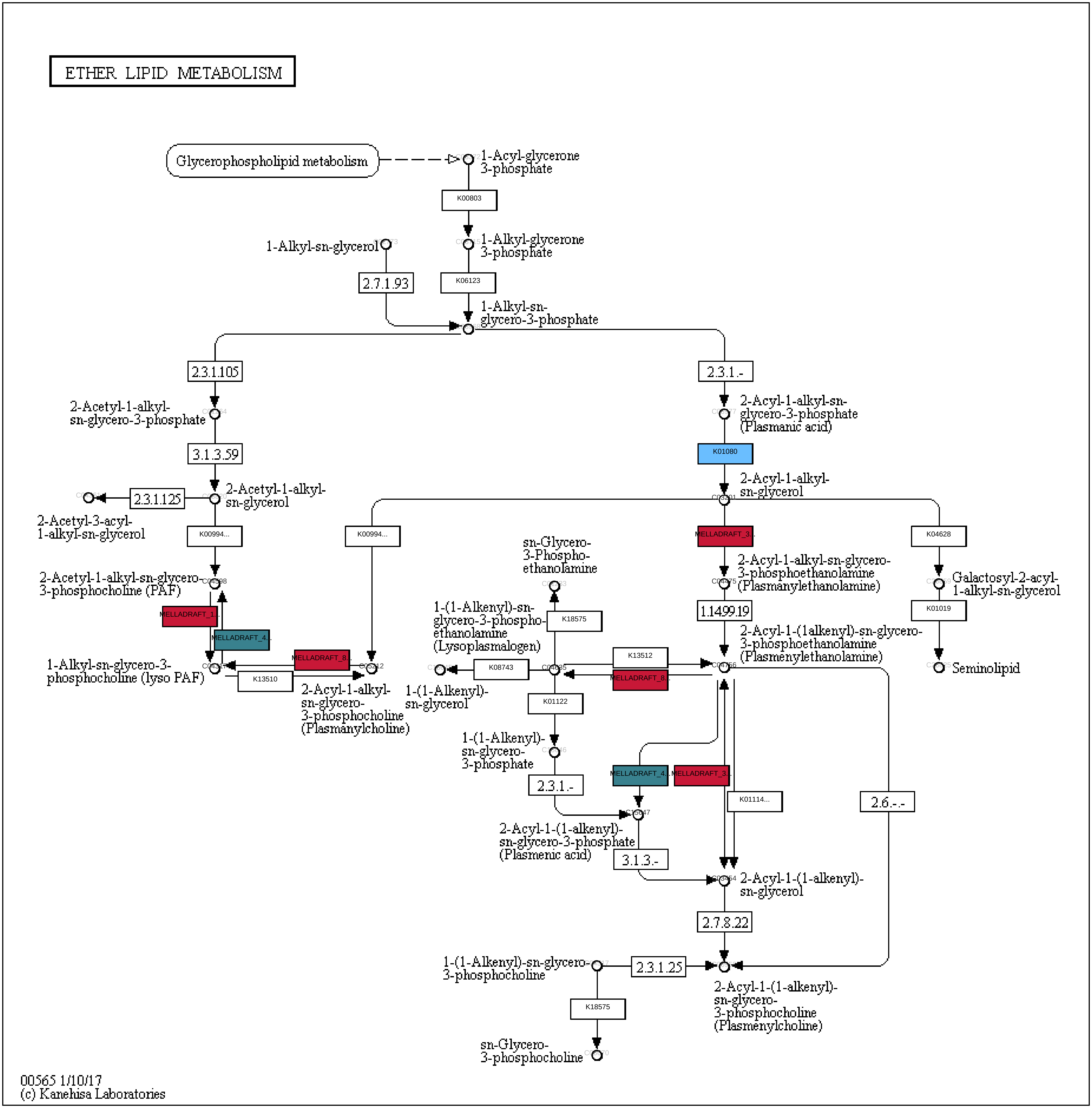
ETHER LIPID METABOLISM
Glycerophospholipid metabolism
1-Acyl-glycerone 3-phosphate
K00803
1-Alkyl-glycerone 3-phosphate
1-Alkyl-sn-glycerol
2.7.1.93
K06123
1-Alkyl-sn-glycero-3-phosphate
2.3.1.105
2.3.1.-
2-Acetyl-1-alkyl-sn-glycero-3-phosphate
2-Acyl-1-alkyl-sn-glycero-3-phosphate (Plasmanic acid)
3.1.3.59
K01080
2.3.1.125
2-Acetyl-1-alkyl-sn-glycerol
2-Acyl-1-alkyl-sn-glycerol
2-Acetyl-3-acyl-1-alkyl-sn-glycerol
K00994...
K00994...
MELLADRAFT_3...
K04628
sn-Glycero-3-Phospho-ethanolamine
2-Acyl-1-alkyl-sn-glycero-3-phosphoethanolamine (Plasmanylethanolamine)
Galactosyl-2-acyl-1-alkyl-sn-glycerol
2-Acetyl-1-alkyl-sn-glycero-3-phosphocholine (PAF)
1-(1-Alkenyl)-sn-glycero-3-phospho-ethanolamine (Lysoplasmalogen)
K18575
1.14.99.19
K01019
MELLADRAFT_1...
MELLADRAFT_4...
2-Acyl-1-(1alkenyl)-sn-glycero-3-phosphoethanolamine (Plasmenylethanolamine)
MELLADRAFT_8...
K13512
1-Alkyl-sn-glycero-3-phosphocholine (lyso PAF)
K13510
K08743
MELLADRAFT_8...
Seminolipid
2-Acyl-1-alkyl-sn-glycero-3-phosphocholine (Plasmanylcholine)
1-(1-Alkenyl)-sn-glycerol
K01122
1-(1-Alkenyl)-sn-glycero-3-phosphate
MELLADRAFT_4...
MELLADRAFT_3...
2.3.1.-
K01114...
2.6.-.-
2-Acyl-1-(1-alkenyl)-sn-glycero-3-phosphate (Plasmenic acid)
3.1.3.-
2-Acyl-1-(1-alkenyl)-sn-glycerol
2.7.8.22
1-(1-Alkenyl)-sn-glycero-3-phosphocholine
2.3.1.25
K18575
2-Acyl-1-(1-alkenyl)-sn-glycero-3-phosphocholine (Plasmenylcholine)
sn-Glycero-3-phosphocholine
00565 1/10/17
(c) Kanehisa Laboratories

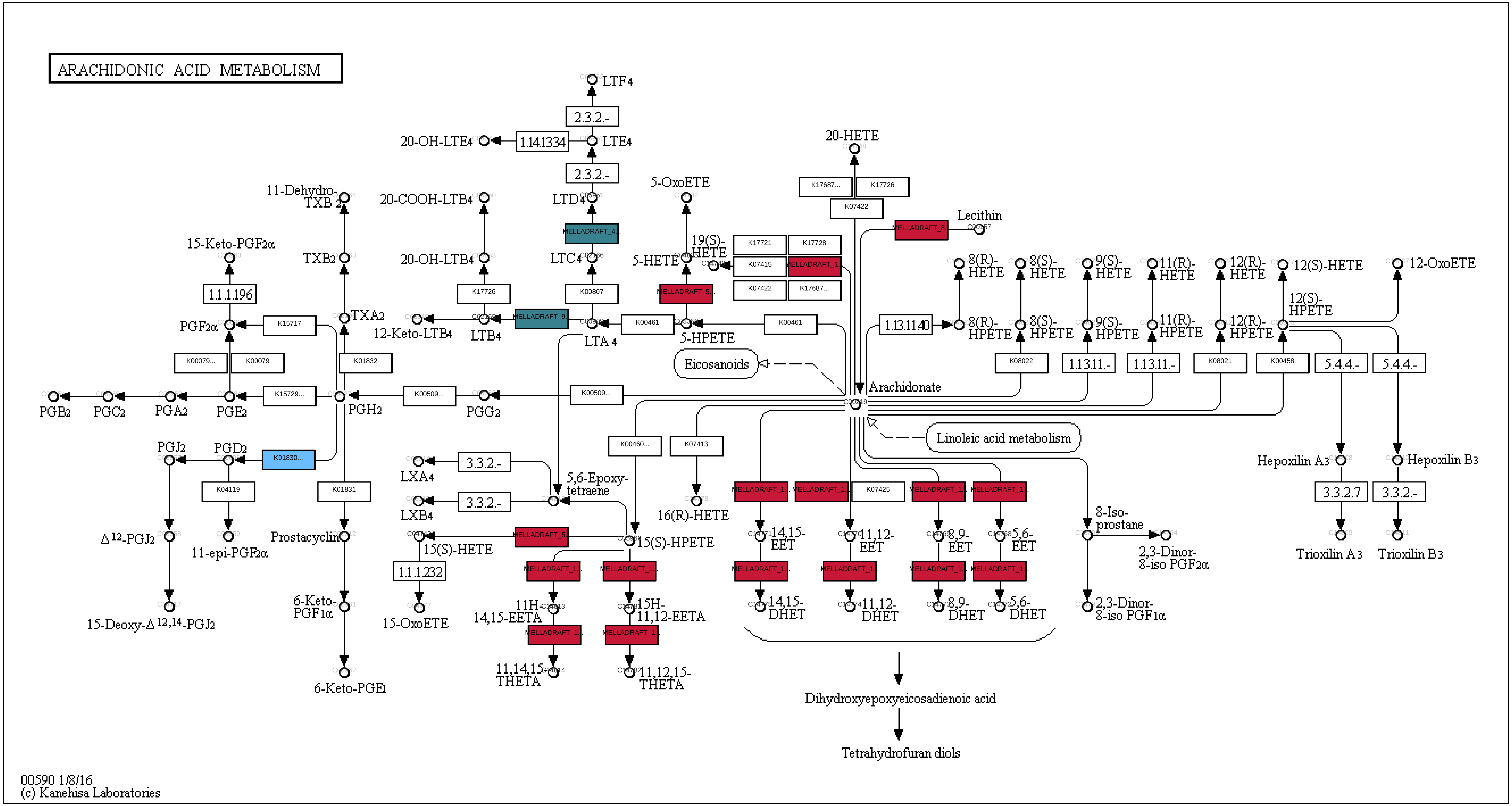
ARACHIDONIC ACID METABOLISM
LTF4
2.3.2.-
20-OH-LTE4
1.14.13.34
LTE4
2.3.2.-
20-COOH-LTB4
LTD4
5-OxoETE
20-HETE
K17687...
K17726
K07422
Lecithin
MELLADRAFT_8...
MELLADRAFT_4...
11-Dehydro-TXB2
TXB2
20-OH-LTB4
LTC4
19(S)-HETE
5-HETE
K17721
K17728
K07415
MELLADRAFT_1...
K07422
K17687...
MELLADRAFT_5...
15-Keto-PGF2α
1.1.1.196
K17726
K00807
MELLADRAFT_9...
TXA2
12-Keto-LTB4
LTB4
LTA4
K00461
5-HPETE
K00461
1.13.11.40
8(R)-HETE
8(S)-HETE
9(S)-HETE
11(R)-HETE
12(R)-HETE
12(S)-HETE
12-OxoETE
8(R)-HPETE
8(S)-HPETE
9(S)-HPETE
11(R)-HPETE
12(R)-HPETE
12(S)-HPETE
PGF2α
K15717
K00079...
K00079
K01832
Eicosanoids
K08022
1.13.11.-
1.13.11.-
K08021
K00458
5.4.4.-
5.4.4.-
Arachidonate
PGB2
PGC2
PGA2
PGE2
K15729...
PGH2
K00509...
PGG2
K00509...
PGJ2
PGD2
K01830...
K00460...
K07413
Linoleic acid metabolism
Hepoxilin A3
Hepoxilin B3
K04119
K01831
LXA4
3.3.2.-
5,6-Epoxy-tetraene
LXB4
3.3.2.-
MELLADRAFT_1...
MELLADRAFT_1...
K07425
MELLADRAFT_1...
MELLADRAFT_1...
3.3.2.7
3.3.2.-
Δ12-PGJ2
11-epi-PGF2α
Prostacyclin
MELLADRAFT_5...
15(S)-HETE
15(S)-HPETE
16(R)-HETE
14,15-EET
11,12-EET
8,9-EET
5,6-EET
8-Iso-prostane
2,3-Dinor-8-iso PGF2α
Trioxilin A3
Trioxilin B3
1.1.1.232
MELLADRAFT_1...
MELLADRAFT_1...
MELLADRAFT_1...
MELLADRAFT_1...
MELLADRAFT_1...
MELLADRAFT_1...
15-Deoxy-Δ12,14-PGJ2
6-Keto-PGF1α
15-OxoETE
11H-14,15-EETA
15H-11,12-EETA
14,15-DHET
11,12-DHET
8,9-DHET
5,6-DHET
2,3-Dinor-8-iso PGF1α
MELLADRAFT_1...
MELLADRAFT_1...
11,14,15-THETA
11,12,15-THETA
6-Keto-PGE1
Dihydroxyepoxyeicosadienoic acid
Tetrahydrofuran diols
00590 1/8/16
(c) Kanehisa Laboratories

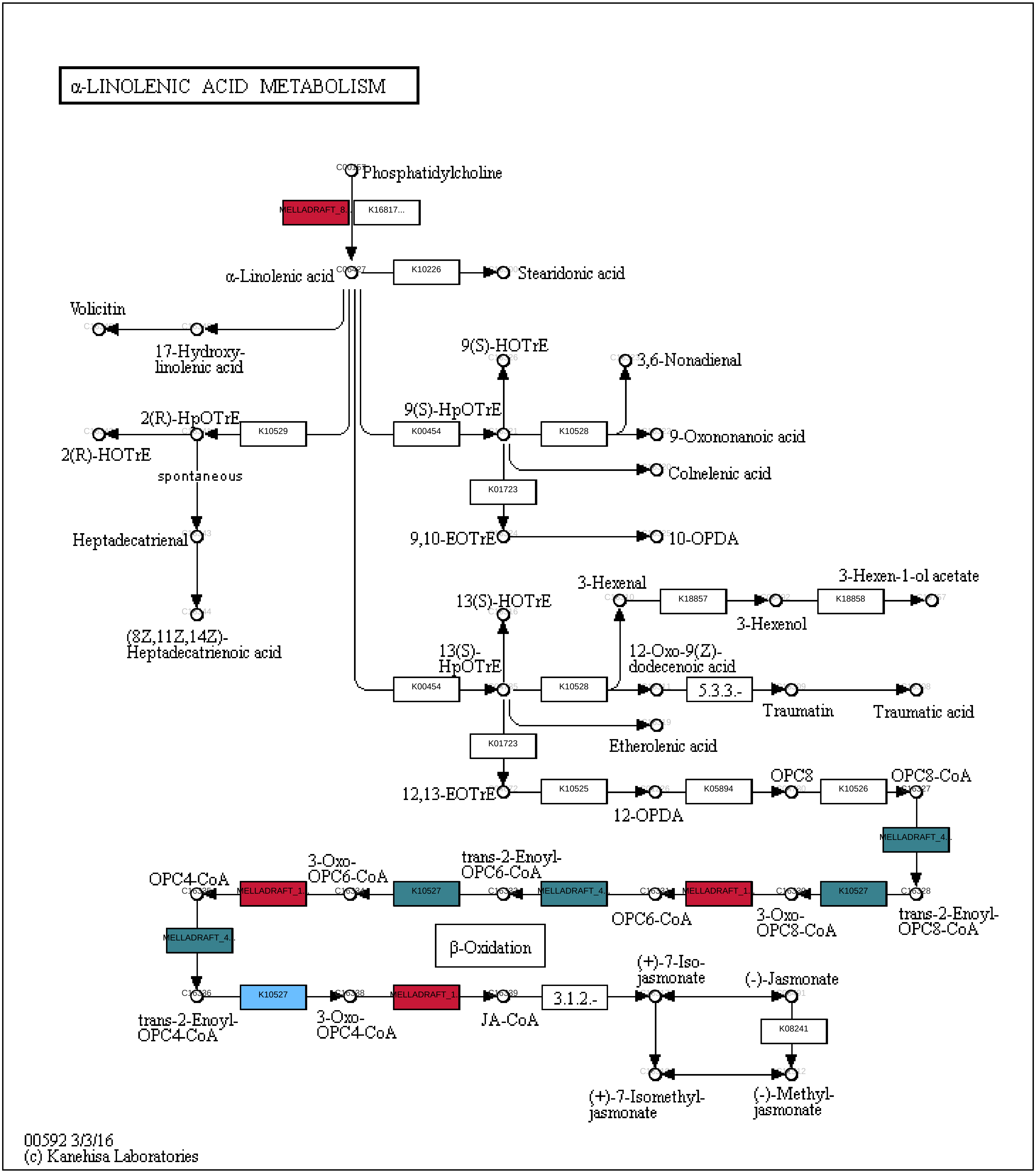
α-LINOLENIC ACID METABOLISM
Phosphatidylcholine
MELLADRAFT_8...
K16817...
α-Linolenic acid
K10226
Stearidonic acid
Volicitin
17-Hydroxy-linolenic acid
9(S)-HOTrE
3,6-Nonadienal
2(R)-HpOTrE
K10529
9(S)-HpOTrE
K00454
K10528
9-Oxononanoic acid
2(R)-HOTrE
spontaneous
Colnelenic acid
K01723
Heptadecatrienal
9,10-EOTrE
10-OPDA
(8Z,11Z,14Z)-Heptadecatrienoic acid
3-Hexenal
K18857
K18858
3-Hexen-1-ol acetate
3-Hexenol
13(S)-HOTrE
13(S)-HpOTrE
12-Oxo-9(Z)-dodecenoic acid
K00454
K10528
5.3.3.-
Traumatin
Traumatic acid
Etherolenic acid
K01723
12,13-EOTrE
K10525
K05894
K10526
OPC8
OPC8-CoA
12-OPDA
MELLADRAFT_4...
OPC4-CoA
3-Oxo-OPC6-CoA
trans-2-Enoyl-OPC6-CoA
MELLADRAFT_1...
K10527
MELLADRAFT_4...
MELLADRAFT_1...
K10527
OPC6-CoA
3-Oxo-OPC8-CoA
trans-2-Enoyl-OPC8-CoA
MELLADRAFT_4...
β-Oxidation
K10527
MELLADRAFT_1...
3.1.2.-
(+)-7-Iso-jasmonate
(-)-Jasmonate
K08241
trans-2-Enoyl-OPC4-CoA
3-Oxo-OPC4-CoA
JA-CoA
(+)-7-Isomethyl-jasmonate
(-)-Methyl-jasmonate
00592 3/3/16
(c) Kanehisa Laboratories

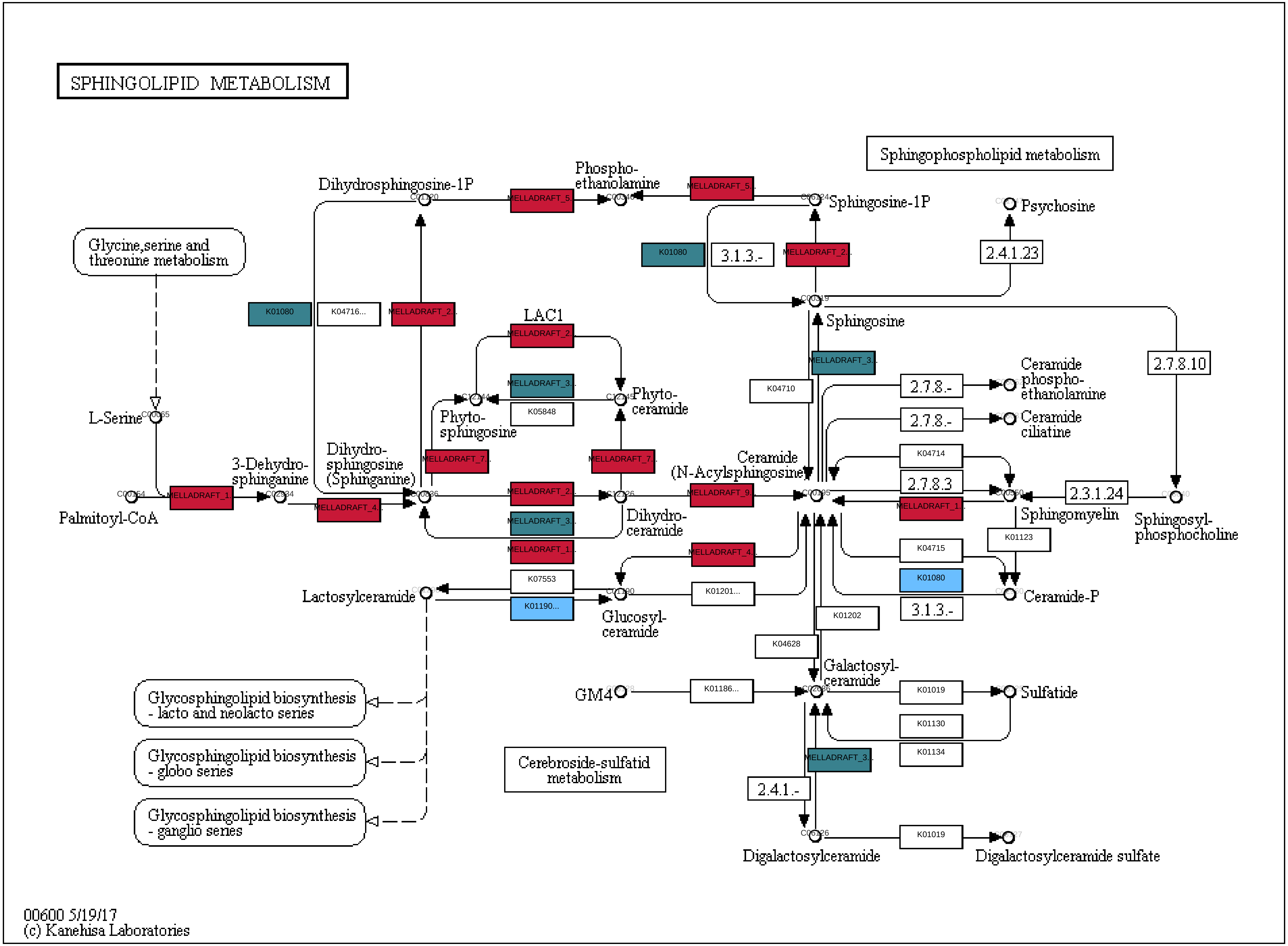
SPHINGOLIPID METABOLISM
Sphingophospholipid metabolism
Glycine,serine and threonine metabolism
L-Serine
C00065
Palmitoyl-CoA
C00154
MELLADRAFT_1
3-Dehydro-sphinganine
C02934
MELLADRAFT_4
Dihydro-sphingosine (Sphinganine)
C00836
K01080
K04716...
MELLADRAFT_2
Dihydrosphingosine-1P
C01120
MELLADRAFT_5
Phospho-ethanolamine
C00346
MELLADRAFT_5
C06124
Sphingosine-1P
K01080
3.1.3.-
MELLADRAFT_2
C00319
Sphingosine
Psychosine
2.4.1.23
2.7.8.10
LAC1
MELLADRAFT_2
MELLADRAFT_3
K05848
Phyto-sphingosine
C12144
Phyto-ceramide
C12145
MELLADRAFT_7
MELLADRAFT_7
MELLADRAFT_2
MELLADRAFT_3
MELLADRAFT_1
C12126
Dihydro-ceramide
MELLADRAFT_9
Ceramide (N-Acylsphingosine)
C00195
MELLADRAFT_3
K04710
2.7.8.-
Ceramide phospho-ethanolamine
2.7.8.-
Ceramide ciliatine
K04714
2.7.8.3
MELLADRAFT_1
Sphingomyelin
2.3.1.24
Sphingosyl-phosphocholine
K01123
K04715
K01080
Ceramide-P
3.1.3.-
MELLADRAFT_4
K07553
K01190...
Lactosylceramide
Glucosyl-ceramide
C01190
K01201...
K01202
K04628
Galactosyl-ceramide
GM4
K01186...
K01019
Sulfatide
K01130
K01134
MELLADRAFT_3
2.4.1.-
C06126
Digalactosylceramide
K01019
Digalactosylceramide sulfate
Glycosphingolipid biosynthesis - lacto and neolacto series
Glycosphingolipid biosynthesis - globo series
Glycosphingolipid biosynthesis - ganglio series
Cerebroside-sulfatid metabolism
00600 5/19/17
(c) Kanehisa Laboratories

# 4. Nucleotide metabolism

| MAP | PATHWAY |
|---|---|
| **230** | Purine metabolism |
| **240** | Pyrimidine metabolism |

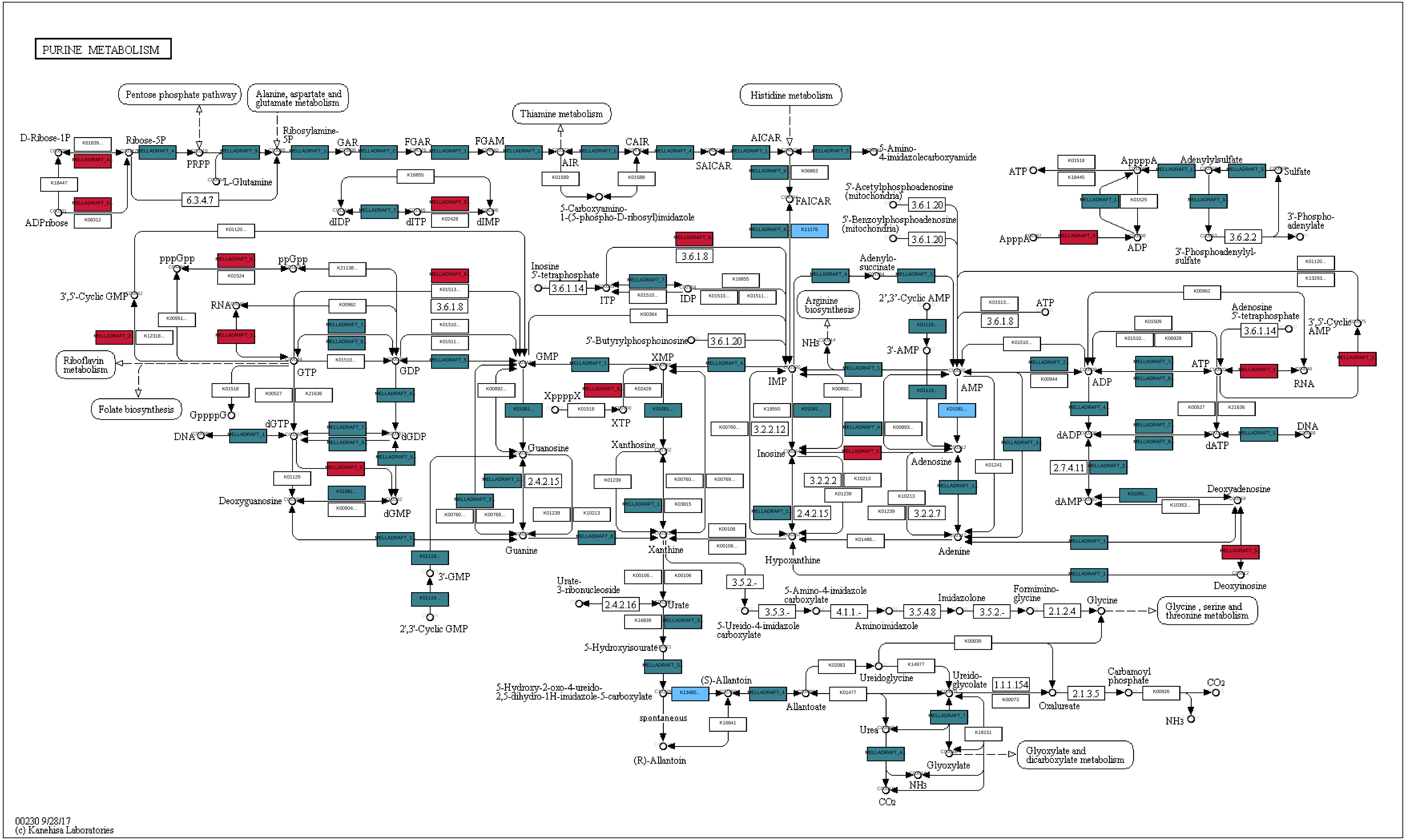
PURINE METABOLISM
Pentose phosphate pathway
Alanine, aspartate and glutamate metabolism
Thiamine metabolism
Histidine metabolism
D-Ribose-1P
Ribose-5P
PRPP
Ribosylamine-5P
L-Glutamine
6.3.4.7
ADPribose
GAR
FGAR
FGAM
AIR
CAIR
SAICAR
AICAR
5-Amino-4-imidazolecarboxyamide
FAICAR
5-Carboxyamino-1-(5-phospho-D-ribosyl)imidazole
dIDP
dITP
dIMP
ATP
ApppA
Adenylylsulfate
Sulfate
ADP
AppppA
3'-Phospho-adenylate
3'-Phosphoadenylyl-sulfate
3.6.2.2
5'-Acetylphosphoadenosine (mitochondria)
5'-Benzoylphosphoadenosine (mitochondria)
3.6.1.20
pppGpp
ppGpp
3',5'-Cyclic GMP
RNA
Riboflavin metabolism
Folate biosynthesis
GTP
GDP
GMP
3.6.1.8
GppppG
dGTP
dGDP
DNA
dGMP
Deoxyguanosine
Guanosine
Guanine
2.4.2.15
Inosine 5'-tetraphosphate
3.6.1.14
ITP
IDP
3.6.1.8
XppppX
XTP
XMP
5'-Butyrylphosphoinosine
3.6.1.20
IMP
Xanthosine
Xanthine
Inosine
3.2.2.12
3.2.2.2
2.4.2.15
Hypoxanthine
Adenylo-succinate
2',3'-Cyclic AMP
Arginine biosynthesis
NH3
3'-AMP
AMP
Adenosine
3.2.2.7
Adenine
3.6.1.8
Adenosine 5'-tetraphosphate
3.6.1.14
3',5'-Cyclic AMP
RNA
ADP
ATP
dADP
dATP
DNA
2.7.4.11
dAMP
Deoxyadenosine
Deoxyinosine
3'-GMP
2',3'-Cyclic GMP
Urate-3-ribonucleoside
2.4.2.16
Urate
5-Hydroxyisourate
5-Hydroxy-2-oxo-4-ureido-2,5-dihydro-1H-imidazole-5-carboxylate
spontaneous
(S)-Allantoin
(R)-Allantoin
3.5.2.-
5-Amino-4-imidazole carboxylate
3.5.3.-
4.1.1.-
3.5.4.8
3.5.2.-
2.1.2.4
5-Ureido-4-imidazole carboxylate
Aminoimidazole
Imidazolone
Formimino-glycine
Glycine
Glycine, serine and threonine metabolism
Allantoate
Ureidoglycine
Ureido-glycolate
1.1.1.154
Oxalureate
2.1.3.5
Carbamoyl phosphate
CO2
NH3
Urea
Glyoxylate
Glyoxylate and dicarboxylate metabolism
NH3
CO2
00230 9/28/17
(c) Kanehisa Laboratories

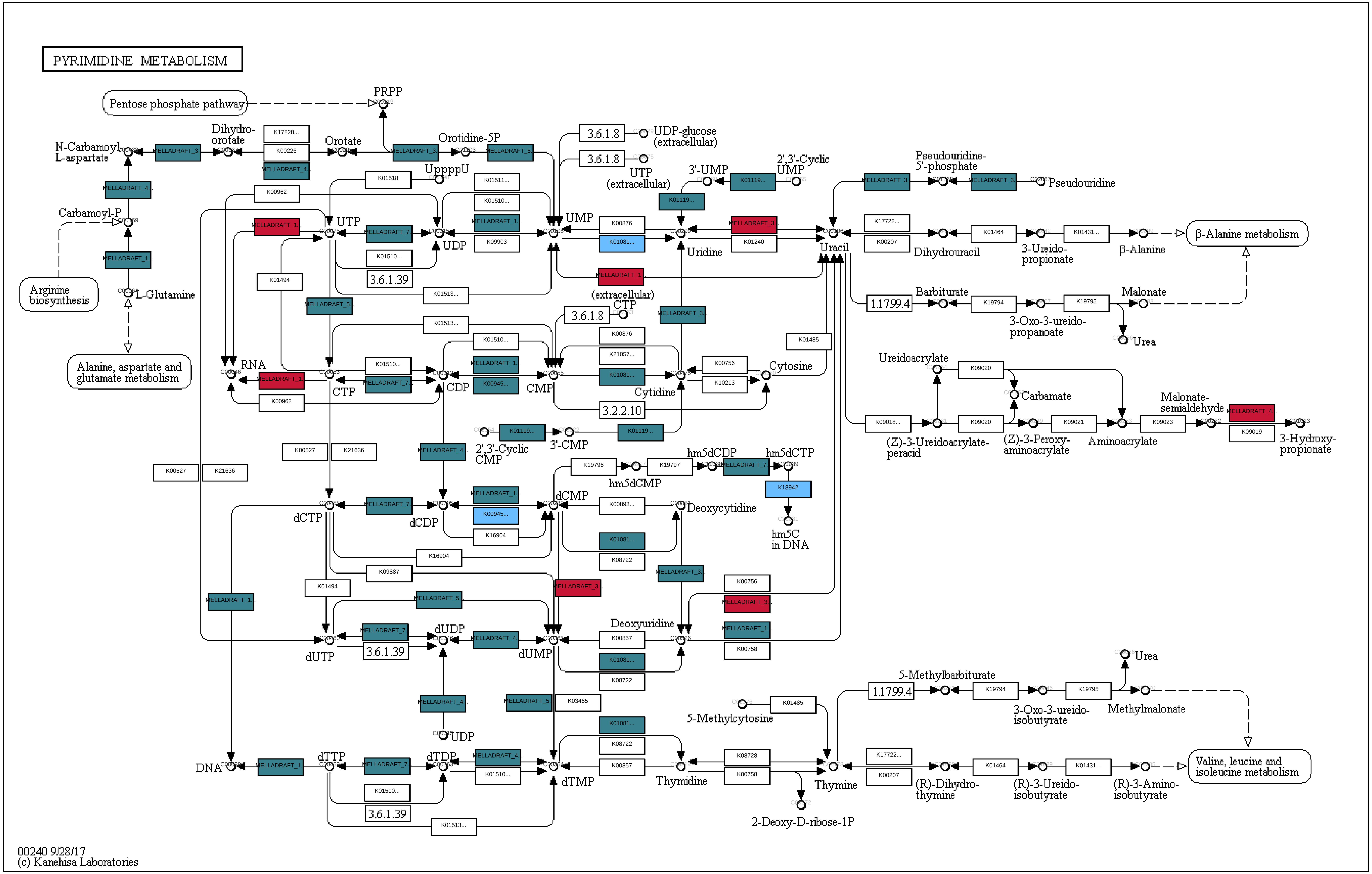
PYRIMIDINE METABOLISM
Pentose phosphate pathway
PRPP
N-Carbamoyl-
L-aspartate
Dihydro-
orotate
Orotate
Orotidine-5P
UppppU
UDP-glucose
(extracellular)
UTP
(extracellular)
3'-UMP
2',3'-Cyclic
UMP
Pseudouridine-
5'-phosphate
Pseudouridine
Carbamoyl-P
Arginine
biosynthesis
L-Glutamine
Alanine, aspartate and
glutamate metabolism
UTP
UDP
UMP
Uridine
Uracil
(extracellular)
Dihydrouracil
3-Ureido-
propionate
β-Alanine
β-Alanine metabolism
Barbiturate
3-Oxo-3-ureido-
propanoate
Malonate
Urea
CTP
RNA
CTP
CDP
CMP
Cytidine
Cytosine
Ureidoacrylate
Carbamate
Malonate-
semialdehyde
(Z)-3-Ureidoacrylate-
peracid
(Z)-3-Peroxy-
aminoacrylate
Aminoacrylate
3-Hydroxy-
propionate
2',3'-Cyclic
CMP
3'-CMP
hm5dCDP
hm5dCTP
hm5dCMP
hm5C
in DNA
dCTP
dCDP
dCMP
Deoxycytidine
dUTP
dUDP
dUMP
Deoxyuridine
UDP
DNA
dTTP
dTDP
dTMP
Thymidine
Thymine
5-Methylcytosine
2-Deoxy-D-ribose-1P
5-Methylbarbiturate
3-Oxo-3-ureido-
isobutyrate
Methylmalonate
Urea
(R)-Dihydro-
thymine
(R)-3-Ureido-
isobutyrate
(R)-3-Amino-
isobutyrate
Valine, leucine and
isoleucine metabolism
3.6.1.8
3.6.1.8
3.6.1.8
3.6.1.39
3.6.1.39
3.6.1.39
3.2.2.10
1.17.99.4
1.17.99.4
K17828...
K00226
K01518
K00962
K01511...
K01510...
K09903
K00876
K01081...
K01240
K17722...
K00207
K01464
K01431...
K19794
K19795
K01494
K01513...
K01510...
K00876
K21057...
K01081...
K00756
K10213
K01485
K09020
K09018...
K09021
K09023
K09019
K00945...
K00962
K00527
K21636
K00527
K21636
K01119...
K19796
K19797
K18942
K00893...
K16904
K16904
K09887
K08722
K01494
K00756
K00758
K00857
K08722
K03465
K08728
K00857
K08728
K00758
K01485
K01510...
00240 9/28/17
(c) Kanehisa Laboratories

# 5. Amino Acid metabolism

| MAP | PATHWAY |
|---|---|
| **220** | Arginine biosynthesis |
| **250** | Alanine, aspartate and glutamate metabolism |
| **260** | Glycine, serine and threonine metabolism |
| **270** | Cysteine and methionine metabolism |
| **280** | Valine, leucine and isoleucine degradation |
| **290** | Valine, leucine and isoleucine biosynthesis |
| **300** | Lysine biosynthesis |
| **310** | Lysine degradation |
| **330** | Arginine and proline metabolism |
| **340** | Histidine metabolism |
| **350** | Tyrosine metabolism |
| **360** | Phenylalanine metabolism |
| **380** | Tryptophan metabolism |
| **400** | Phenylalanine, tyrosine and tryptophan biosynthesis |

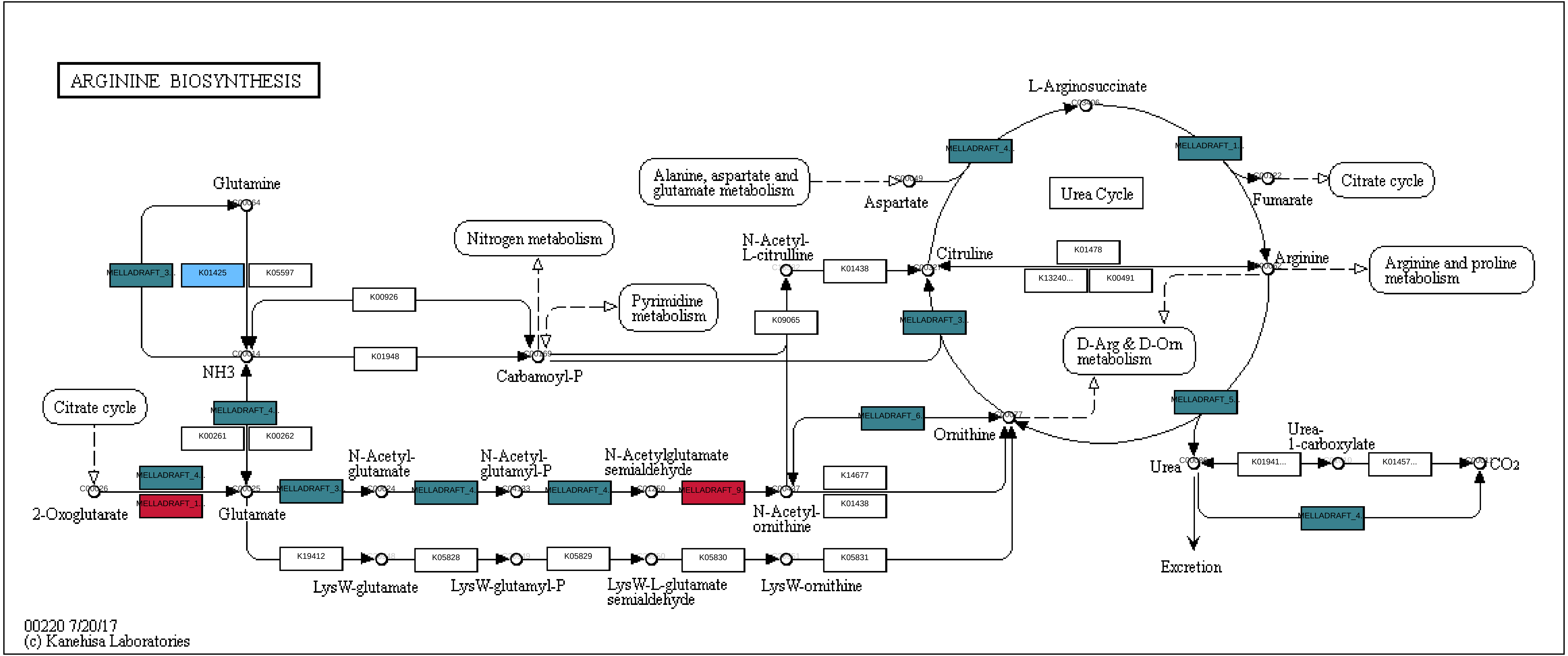
ARGININE BIOSYNTHESIS
L-Arginosuccinate
MELLADRAFT_4...
MELLADRAFT_1...
Alanine, aspartate and glutamate metabolism
Aspartate
Citrate cycle
Fumarate
Urea Cycle
Glutamine
Nitrogen metabolism
N-Acetyl-L-citrulline
K01438
Citrulline
K01478
K13240...
K00491
Arginine
Arginine and proline metabolism
MELLADRAFT_3...
K01425
K05597
K00926
Pyrimidine metabolism
K09065
D-Arg & D-Orn metabolism
K01948
NH3
Carbamoyl-P
Citrate cycle
K00261
K00262
MELLADRAFT_5...
MELLADRAFT_6...
Ornithine
Urea-1-carboxylate
N-Acetyl-glutamate
N-Acetyl-glutamyl-P
N-Acetylglutamate semialdehyde
K14677
K01941...
K01457...
Urea
CO2
2-Oxoglutarate
Glutamate
MELLADRAFT_9...
N-Acetyl-ornithine
Excretion
K19412
K05828
K05829
K05830
K05831
LysW-glutamate
LysW-glutamyl-P
LysW-L-glutamate semialdehyde
LysW-ornithine
00220 7/20/17
(c) Kanehisa Laboratories

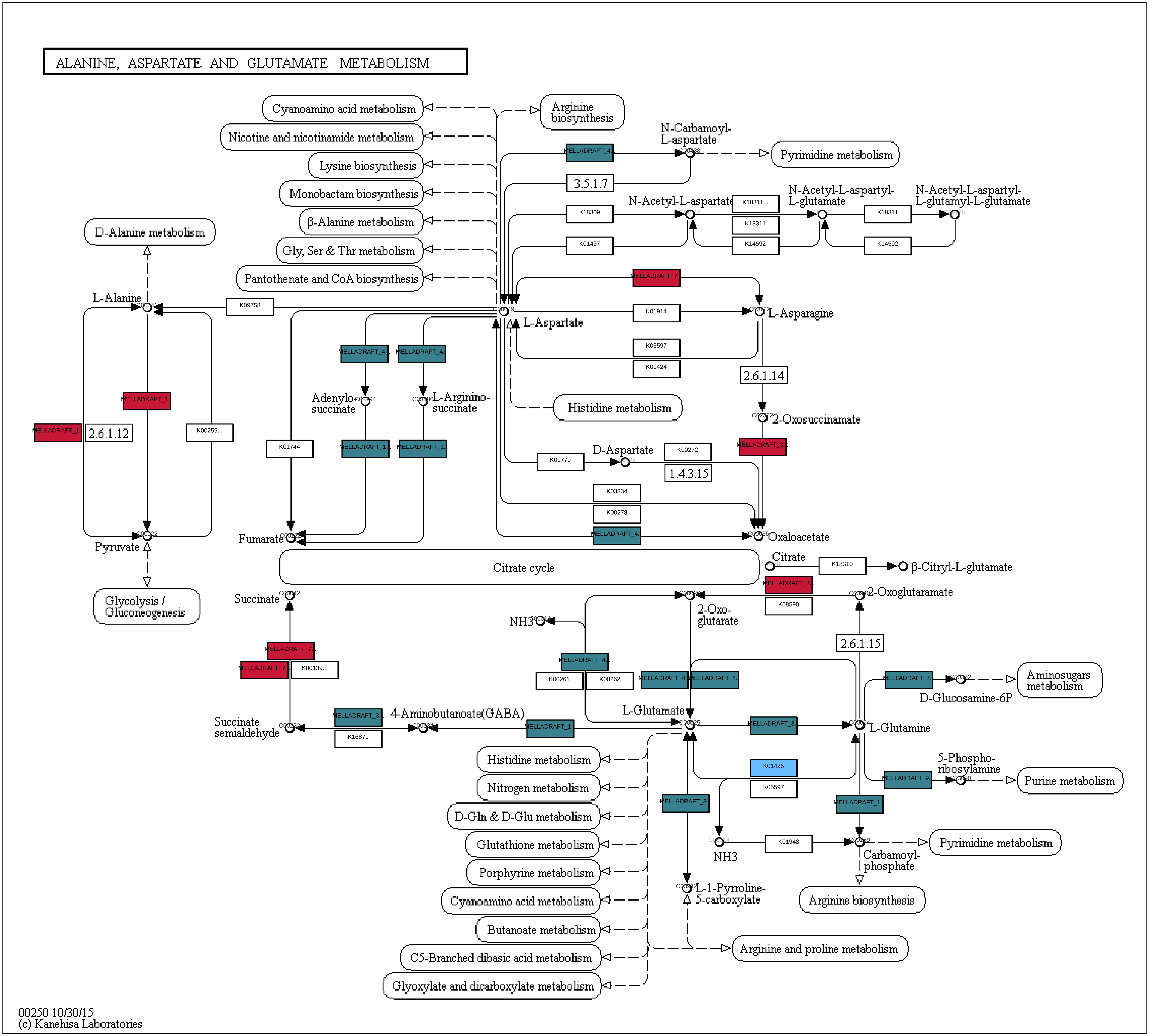
ALANINE, ASPARTATE AND GLUTAMATE METABOLISM
Cyanoamino acid metabolism
Nicotine and nicotinamide metabolism
Lysine biosynthesis
Monobactam biosynthesis
β-Alanine metabolism
Gly, Ser & Thr metabolism
Pantothenate and CoA biosynthesis
Arginine biosynthesis
N-Carbamoyl-L-aspartate
Pyrimidine metabolism
3.5.1.7
K18309
K01437
N-Acetyl-L-aspartate
K18311...
K18311
K14592
N-Acetyl-L-aspartyl-L-glutamate
N-Acetyl-L-aspartyl-L-glutamyl-L-glutamate
D-Alanine metabolism
L-Alanine
K09758
L-Aspartate
K01914
L-Asparagine
K05597
K01424
2.6.1.14
Adenylo-succinate
L-Arginino-succinate
Histidine metabolism
2-Oxosuccinamate
2.6.1.12
K00259...
K01744
K01779
D-Aspartate
K00272
1.4.3.15
K03334
K00278
Pyruvate
Fumarate
Oxaloacetate
Citrate cycle
Citrate
K18310
β-Citryl-L-glutamate
Glycolysis / Gluconeogenesis
Succinate
NH3
2-Oxo-glutarate
K08590
2-Oxoglutaramate
2.6.1.15
K00139...
K00261
K00262
Aminosugars metabolism
D-Glucosamine-6P
Succinate semialdehyde
4-Aminobutanoate(GABA)
K16871
L-Glutamate
L-Glutamine
K01425
K05597
5-Phospho-ribosylamine
Purine metabolism
Histidine metabolism
Nitrogen metabolism
D-Gln & D-Glu metabolism
Glutathione metabolism
Porphyrine metabolism
Cyanoamino acid metabolism
Butanoate metabolism
C5-Branched dibasic acid metabolism
Glyoxylate and dicarboxylate metabolism
NH3
K01948
Carbamoyl-phosphate
Pyrimidine metabolism
L-1-Pyrroline-5-carboxylate
Arginine biosynthesis
Arginine and proline metabolism
00250 10/30/15
(c) Kanehisa Laboratories

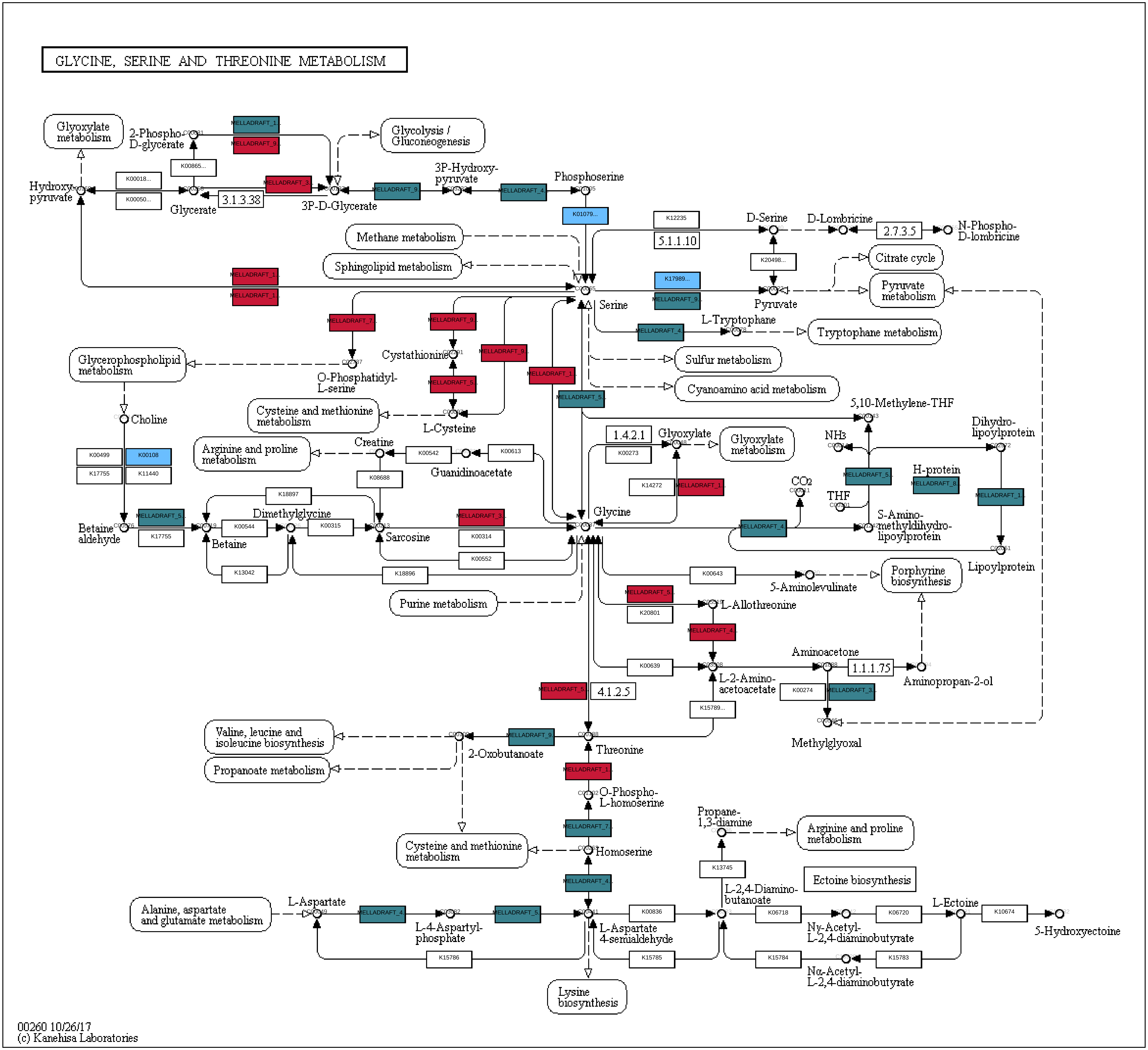
GLYCINE, SERINE AND THREONINE METABOLISM
Glyoxylate metabolism
2-Phospho-D-glycerate
Glycolysis / Gluconeogenesis
3P-Hydroxy-pyruvate
Phosphoserine
Hydroxy-pyruvate
Glycerate
3.1.3.38
3P-D-Glycerate
K00018...
K00050...
K00865...
K01079...
K12235
5.1.1.10
D-Serine
D-Lombricine
2.7.3.5
N-Phospho-D-lombricine
K20498...
Methane metabolism
Sphingolipid metabolism
Citrate cycle
K17989...
Pyruvate metabolism
Serine
Pyruvate
L-Tryptophane
Tryptophane metabolism
Glycerophospholipid metabolism
Cystathionine
O-Phosphatidyl-L-serine
Sulfur metabolism
Cyanoamino acid metabolism
Choline
Cysteine and methionine metabolism
L-Cysteine
5,10-Methylene-THF
Dihydro-lipoylprotein
NH3
Arginine and proline metabolism
Creatine
K00542
K00613
1.4.2.1
Glyoxylate
Glyoxylate metabolism
K00273
Guanidinoacetate
K00499
K00108
K17755
K11440
K08688
H-protein
K14272
CO2
THF
K18897
Dimethylglycine
Glycine
S-Amino-methyldihydro-lipoylprotein
Betaine aldehyde
K00544
K00315
Sarcosine
K00314
K17755
Betaine
K00552
K13042
K18896
K00643
5-Aminolevulinate
Porphyrine biosynthesis
Lipoylprotein
Purine metabolism
K20801
L-Allothreonine
K00639
Aminoacetone
1.1.1.75
Aminopropan-2-ol
4.1.2.5
L-2-Amino-acetoacetate
K00274
K15789...
Valine, leucine and isoleucine biosynthesis
2-Oxobutanoate
Threonine
Methylglyoxal
Propanoate metabolism
O-Phospho-L-homoserine
Propane-1,3-diamine
Arginine and proline metabolism
Cysteine and methionine metabolism
Homoserine
K13745
Ectoine biosynthesis
L-2,4-Diamino-butanoate
Alanine, aspartate and glutamate metabolism
L-Aspartate
L-4-Aspartyl-phosphate
K00836
L-Aspartate 4-semialdehyde
K06718
Nγ-Acetyl-L-2,4-diaminobutyrate
K06720
L-Ectoine
K10674
5-Hydroxyectoine
K15786
K15785
K15784
K15783
Nα-Acetyl-L-2,4-diaminobutyrate
Lysine biosynthesis
00260 10/26/17
(c) Kanehisa Laboratories

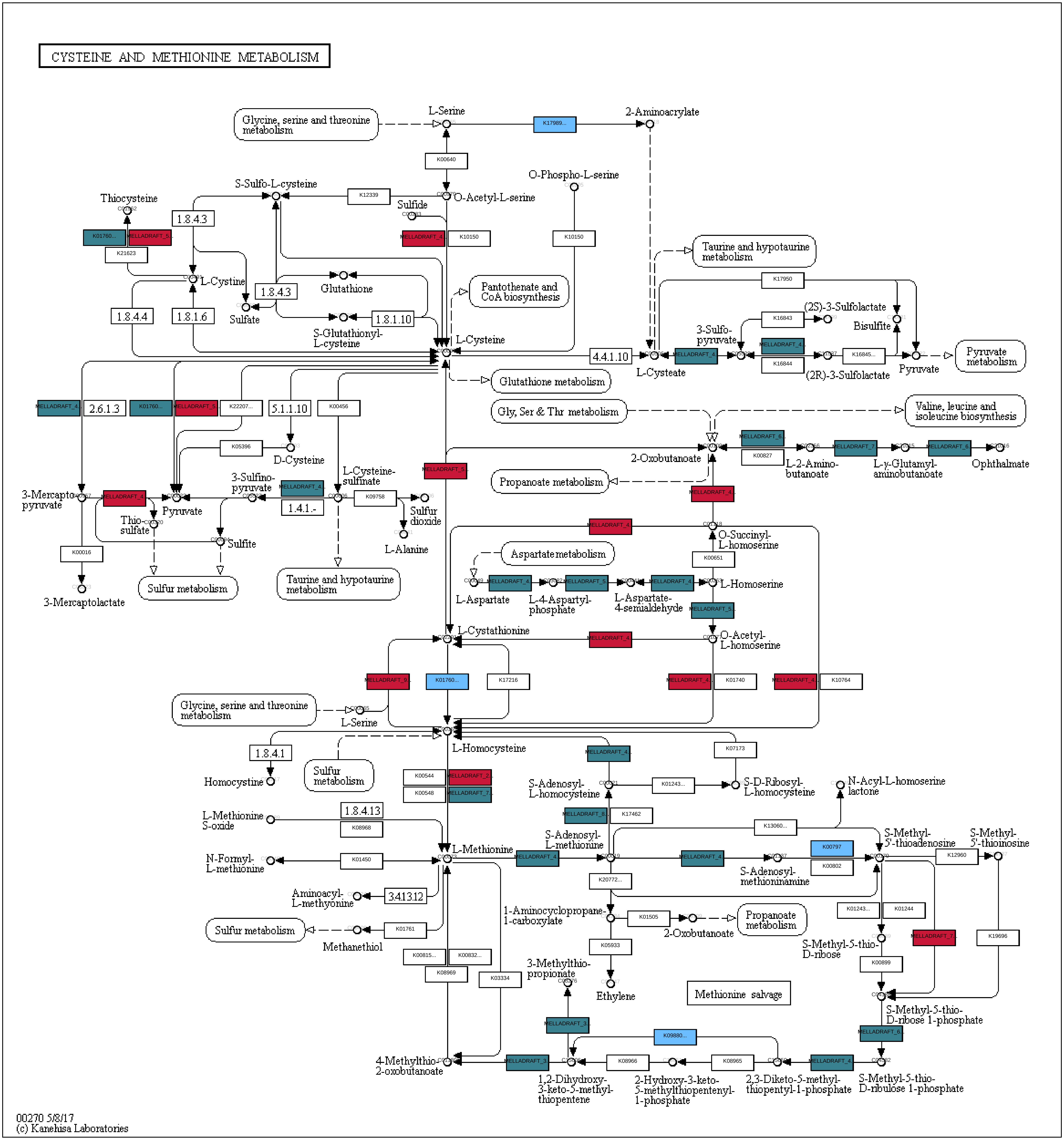
CYSTEINE AND METHIONINE METABOLISM
Glycine, serine and threonine metabolism
L-Serine
K17989...
2-Aminoacrylate
K00640
O-Phospho-L-serine
S-Sulfo-L-cysteine
K12339
O-Acetyl-L-serine
Sulfide
Thiocysteine
1.8.4.3
K01760...
MELLADRAFT_5...
MELLADRAFT_4...
K10150
K21623
L-Cystine
Taurine and hypotaurine metabolism
K17950
Glutathione
Pantothenate and CoA biosynthesis
1.8.4.4
1.8.1.6
Sulfate
S-Glutathionyl-L-cysteine
1.8.1.10
L-Cysteine
(2S)-3-Sulfolactate
K16843
Bisulfite
3-Sulfo-pyruvate
4.4.1.10
L-Cysteate
K16845...
K16844
Pyruvate
Pyruvate metabolism
(2R)-3-Sulfolactate
Glutathione metabolism
2.6.1.3
K22207...
5.1.1.10
K00456
Gly, Ser & Thr metabolism
Valine, leucine and isoleucine biosynthesis
K05396
D-Cysteine
K00827
2-Oxobutanoate
L-2-Amino-butanoate
L-γ-Glutamyl-aminobutanoate
Ophthalmate
3-Sulfino-pyruvate
L-Cysteine-sulfinate
Propanoate metabolism
3-Mercapto-pyruvate
K09758
Sulfur dioxide
1.4.1.-
Thio-sulfate
Sulfite
L-Alanine
O-Succinyl-L-homoserine
K00016
K00651
Aspartate metabolism
Sulfur metabolism
3-Mercaptolactate
L-Aspartate
L-4-Aspartyl-phosphate
L-Aspartate-4-semialdehyde
L-Homoserine
L-Cystathionine
O-Acetyl-L-homoserine
K17216
K01740
K10764
L-Serine
1.8.4.1
L-Homocysteine
K07173
Homocystine
K00544
K00548
S-Adenosyl-L-homocysteine
K01243...
S-D-Ribosyl-L-homocysteine
N-Acyl-L-homoserine lactone
1.8.4.13
K08968
K17462
K13060...
L-Methionine S-oxide
S-Adenosyl-L-methionine
S-Methyl-5'-thioadenosine
S-Methyl-5'-thioinosine
N-Formyl-L-methionine
K01450
L-Methionine
K00797
K12960
K00802
S-Adenosyl-methioninamine
K20772...
Aminoacyl-L-methyonine
3.4.13.12
1-Aminocyclopropane-1-carboxylate
K01505
2-Oxobutanoate
K01244
Sulfur metabolism
K01761
Methanethiol
K05933
S-Methyl-5-thio-D-ribose
K19696
K00815...
K00832...
K08969
K03334
3-Methylthio-propionate
Ethylene
K00899
Methionine salvage
S-Methyl-5-thio-D-ribose 1-phosphate
K09880...
4-Methylthio-2-oxobutanoate
K08966
K08965
1,2-Dihydroxy-3-keto-5-methyl-thiopentene
2-Hydroxy-3-keto-5-methylthiopentenyl-1-phosphate
2,3-Diketo-5-methyl-thiopentyl-1-phosphate
S-Methyl-5-thio-D-ribulose 1-phosphate
00270 5/8/17
(c) Kanehisa Laboratories

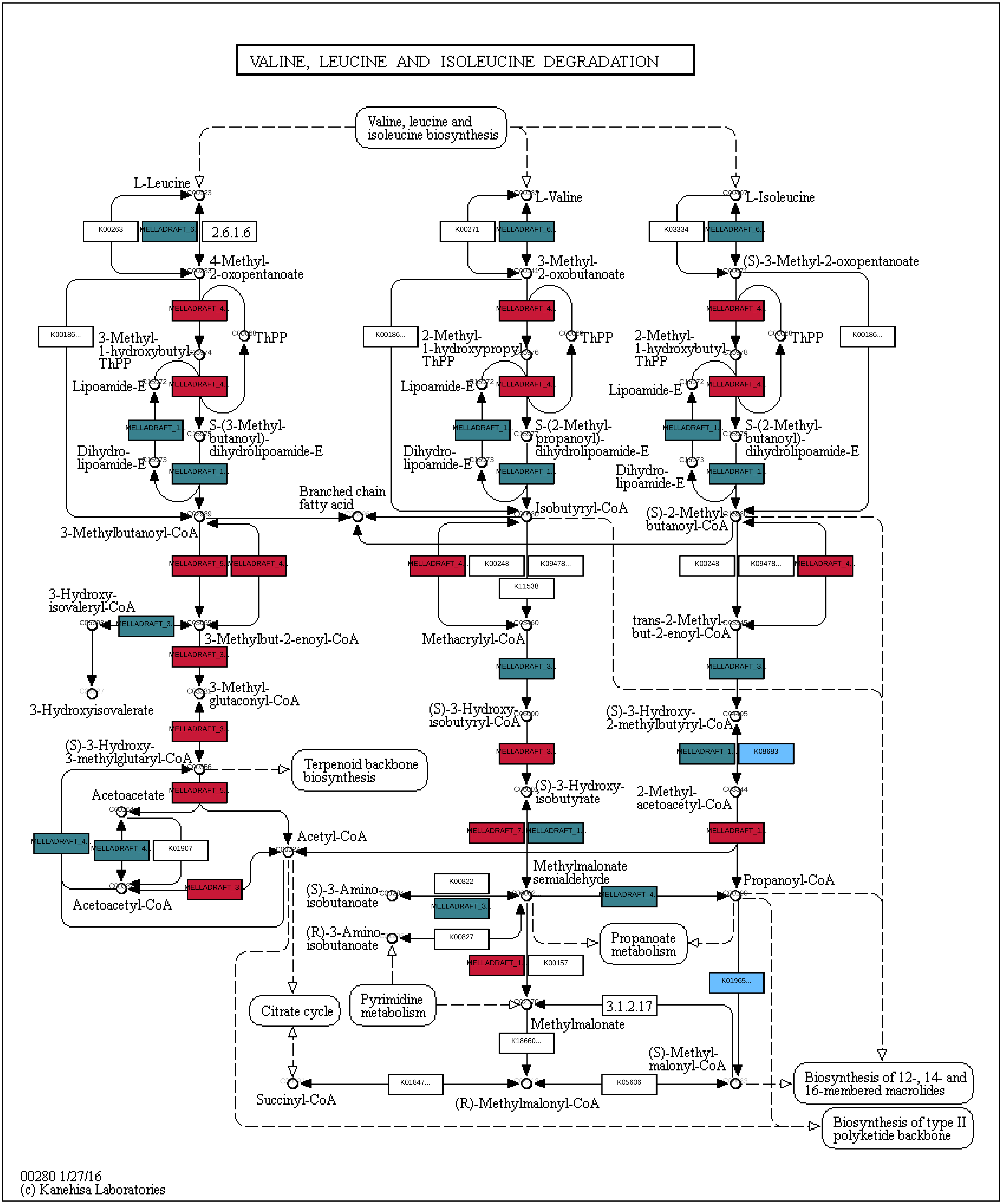

VALINE, LEUCINE AND ISOLEUCINE DEGRADATION
Valine, leucine and isoleucine biosynthesis
L-Leucine
L-Valine
L-Isoleucine
K00263
2.6.1.6
K00271
K03334
4-Methyl-2-oxopentanoate
3-Methyl-2-oxobutanoate
(S)-3-Methyl-2-oxopentanoate
K00186...
ThPP
3-Methyl-1-hydroxybutyl-ThPP
2-Methyl-1-hydroxypropyl-ThPP
2-Methyl-1-hydroxybutyl-ThPP
Lipoamide-E
S-(3-Methylbutanoyl)-dihydrolipoamide-E
S-(2-Methylpropanoyl)-dihydrolipoamide-E
S-(2-Methylbutanoyl)-dihydrolipoamide-E
Dihydro-lipoamide-E
Branched chain fatty acid
Isobutyryl-CoA
(S)-2-Methylbutanoyl-CoA
3-Methylbutanoyl-CoA
K00248
K09478...
K11538
3-Hydroxy-isovaleryl-CoA
3-Methylbut-2-enoyl-CoA
Methacrylyl-CoA
trans-2-Methylbut-2-enoyl-CoA
3-Hydroxyisovalerate
3-Methyl-glutaconyl-CoA
(S)-3-Hydroxy-isobutyryl-CoA
(S)-3-Hydroxy-2-methylbutyryl-CoA
(S)-3-Hydroxy-3-methylglutaryl-CoA
Terpenoid backbone biosynthesis
K08683
(S)-3-Hydroxy-isobutyrate
2-Methyl-acetoacetyl-CoA
Acetoacetate
K01907
Acetyl-CoA
Acetoacetyl-CoA
Methylmalonate semialdehyde
Propanoyl-CoA
K00822
(S)-3-Amino-isobutanoate
(R)-3-Amino-isobutanoate
K00827
Propanoate metabolism
K00157
K01965...
Citrate cycle
Pyrimidine metabolism
3.1.2.17
Methylmalonate
K18660...
(S)-Methyl-malonyl-CoA
K01847...
K05606
Succinyl-CoA
(R)-Methylmalonyl-CoA
Biosynthesis of 12-, 14- and 16-membered macrolides
Biosynthesis of type II polyketide backbone
00280 1/27/16
(c) Kanehisa Laboratories

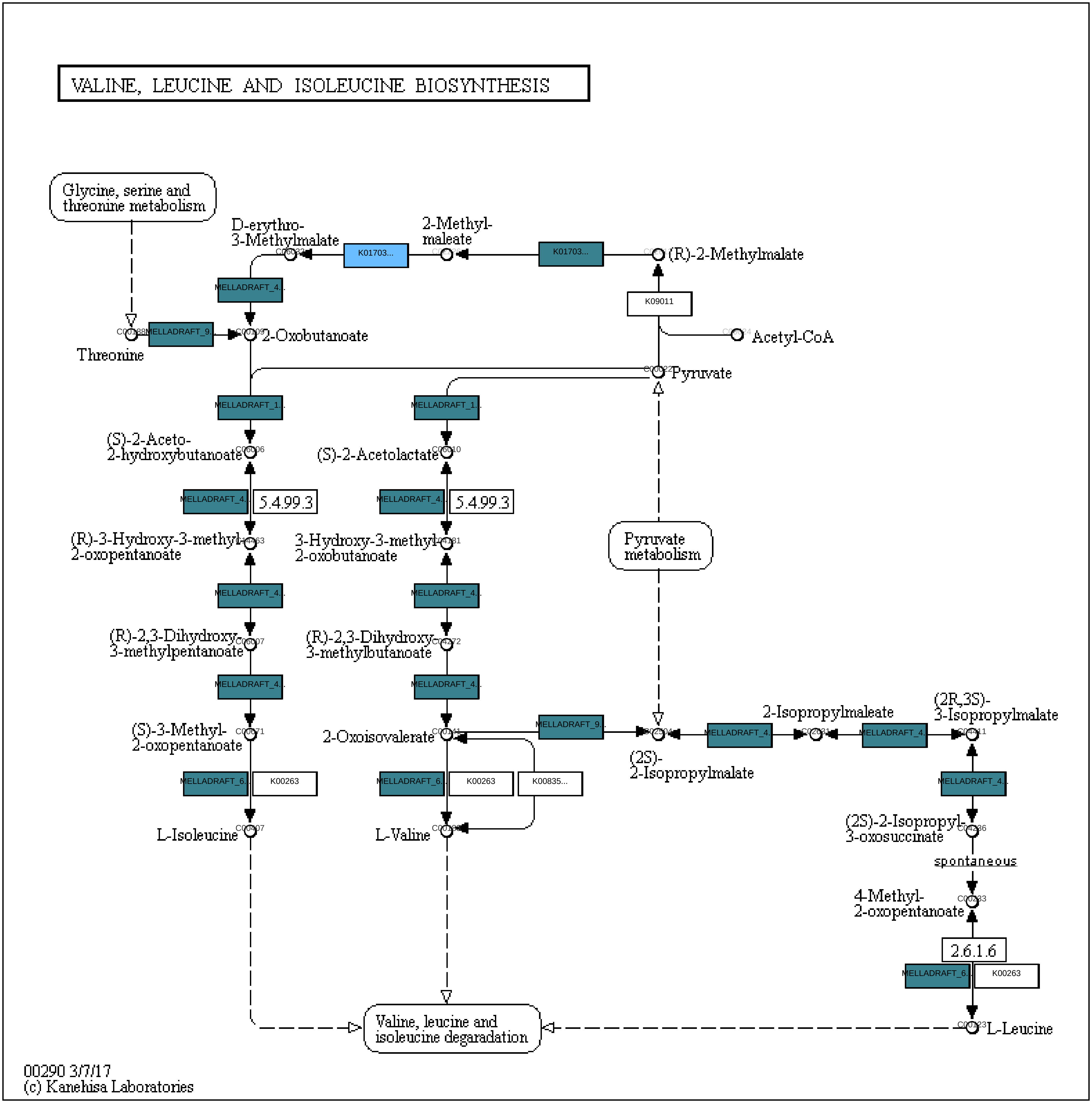
VALINE, LEUCINE AND ISOLEUCINE BIOSYNTHESIS
Glycine, serine and threonine metabolism
D-erythro-3-Methylmalate
2-Methyl-maleate
(R)-2-Methylmalate
K01703...
K01703...
K09011
Acetyl-CoA
Threonine
2-Oxobutanoate
Pyruvate
MELLADRAFT_9...
MELLADRAFT_4...
MELLADRAFT_1...
(S)-2-Aceto-2-hydroxybutanoate
(S)-2-Acetolactate
5.4.99.3
5.4.99.3
(R)-3-Hydroxy-3-methyl-2-oxopentanoate
3-Hydroxy-3-methyl-2-oxobutanoate
Pyruvate metabolism
(R)-2,3-Dihydroxy-3-methylpentanoate
(R)-2,3-Dihydroxy-3-methylbutanoate
(S)-3-Methyl-2-oxopentanoate
2-Oxoisovalerate
2-Isopropylmaleate
(2R,3S)-3-Isopropylmalate
(2S)-2-Isopropylmalate
K00263
K00263
K00835...
MELLADRAFT_6...
L-Isoleucine
L-Valine
(2S)-2-Isopropyl-3-oxosuccinate
spontaneous
4-Methyl-2-oxopentanoate
2.6.1.6
K00263
L-Leucine
Valine, leucine and isoleucine degaradation
00290 3/7/17
(c) Kanehisa Laboratories

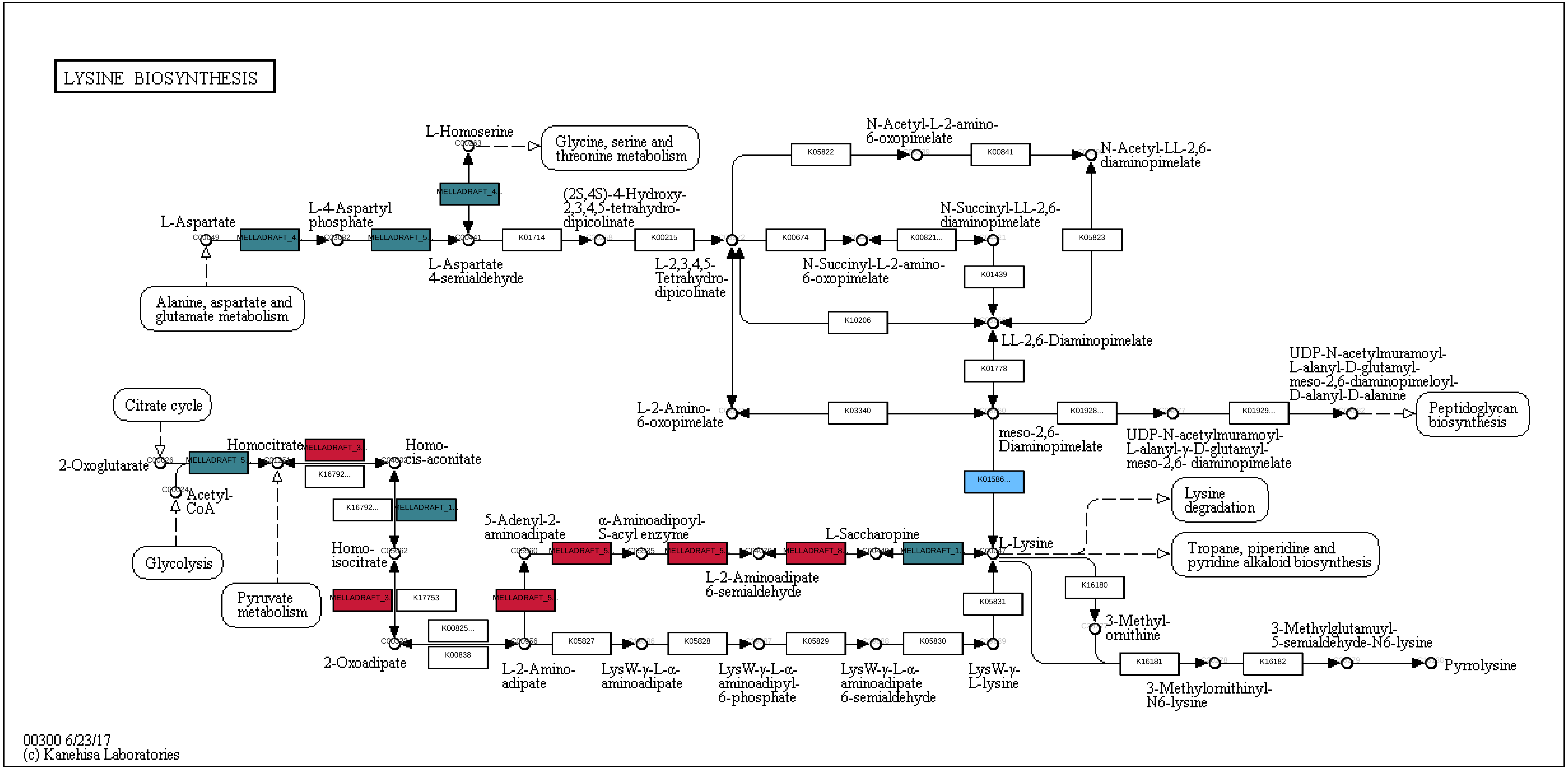
LYSINE BIOSYNTHESIS
L-Aspartate
L-4-Aspartyl phosphate
L-Homoserine
Glycine, serine and threonine metabolism
(2S,4S)-4-Hydroxy-2,3,4,5-tetrahydro-dipicolinate
L-Aspartate 4-semialdehyde
L-2,3,4,5-Tetrahydro-dipicolinate
Alanine, aspartate and glutamate metabolism
N-Acetyl-L-2-amino-6-oxopimelate
N-Acetyl-LL-2,6-diaminopimelate
N-Succinyl-LL-2,6-diaminopimelate
N-Succinyl-L-2-amino-6-oxopimelate
LL-2,6-Diaminopimelate
K05822
K00841
K01714
K00215
K00674
K00821...
K05823
K01439
K10206
K01778
K03340
K01928...
K01929...
K01586...
L-2-Amino-6-oxopimelate
meso-2,6-Diaminopimelate
UDP-N-acetylmuramoyl-L-alanyl-D-glutamyl-meso-2,6-diaminopimeloyl-D-alanyl-D-alanine
UDP-N-acetylmuramoyl-L-alanyl-γ-D-glutamyl-meso-2,6- diaminopimelate
Peptidoglycan biosynthesis
Citrate cycle
2-Oxoglutarate
Homocitrate
Homo-cis-aconitate
Acetyl-CoA
Glycolysis
Pyruvate metabolism
Homo-isocitrate
K16792...
K17753
K00825...
K00838
2-Oxoadipate
5-Adenyl-2-aminoadipate
α-Aminoadipoyl-S-acyl enzyme
L-Saccharopine
L-2-Aminoadipate 6-semialdehyde
L-Lysine
Lysine degradation
Tropane, piperidine and pyridine alkaloid biosynthesis
K16180
K05831
3-Methyl-ornithine
K16181
K16182
3-Methylglutamuyl-5-semialdehyde-N6-lysine
Pyrrolysine
3-Methylornithinyl-N6-lysine
K05827
K05828
K05829
K05830
L-2-Amino-adipate
LysW-γ-L-α-aminoadipate
LysW-γ-L-α-aminoadipyl-6-phosphate
LysW-γ-L-α-aminoadipate 6-semialdehyde
LysW-γ-L-lysine
00300 6/23/17
(c) Kanehisa Laboratories

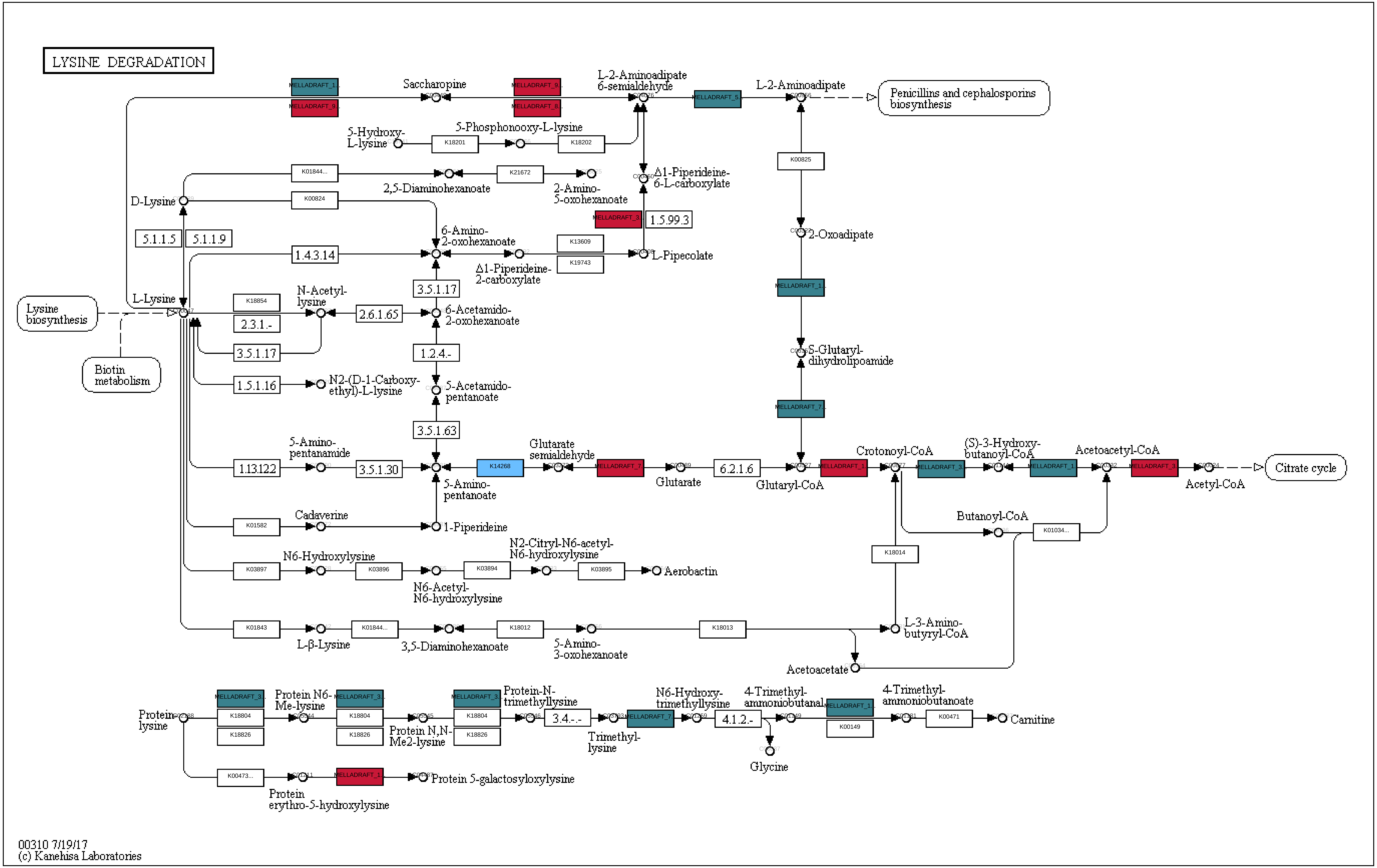

LYSINE DEGRADATION
Saccharopine
L-2-Aminoadipate 6-semialdehyde
L-2-Aminoadipate
Penicillins and cephalosporins biosynthesis
5-Hydroxy-L-lysine
5-Phosphonooxy-L-lysine
K18201
K18202
K01844...
K21672
K00824
2,5-Diaminohexanoate
2-Amino-5-oxohexanoate
Δ1-Piperideine-6-L-carboxylate
K00825
D-Lysine
1.5.99.3
5.1.1.5
5.1.1.9
1.4.3.14
6-Amino-2-oxohexanoate
K13609
K19743
L-Pipecolate
2-Oxoadipate
Δ1-Piperideine-2-carboxylate
3.5.1.17
Lysine biosynthesis
L-Lysine
K18854
N-Acetyl-lysine
2.6.1.65
6-Acetamido-2-oxohexanoate
2.3.1.-
Biotin metabolism
3.5.1.17
1.2.4.-
S-Glutaryl-dihydrolipoamide
1.5.1.16
N2-(D-1-Carboxy-ethyl)-L-lysine
5-Acetamido-pentanoate
3.5.1.63
5-Amino-pentanamide
1.13.12.2
3.5.1.30
K14268
Glutarate semialdehyde
Glutarate
6.2.1.6
Glutaryl-CoA
Crotonoyl-CoA
(S)-3-Hydroxy-butanoyl-CoA
Acetoacetyl-CoA
Acetyl-CoA
Citrate cycle
5-Amino-pentanoate
K01582
Cadaverine
1-Piperideine
Butanoyl-CoA
K01034...
K18014
K03897
N6-Hydroxylysine
K03896
N6-Acetyl-N6-hydroxylysine
K03894
N2-Citryl-N6-acetyl-N6-hydroxylysine
K03895
Aerobactin
K01843
L-β-Lysine
K01844...
3,5-Diaminohexanoate
K18012
5-Amino-3-oxohexanoate
K18013
L-3-Amino-butyryl-CoA
Acetoacetate
Protein lysine
K18804
K18826
Protein N6-Me-lysine
Protein N,N-Me2-lysine
Protein-N-trimethyllysine
3.4.-.-
Trimethyl-lysine
N6-Hydroxy-trimethyllysine
4.1.2.-
Glycine
4-Trimethyl-ammoniobutanal
K00149
4-Trimethyl-ammoniobutanoate
K00471
Carnitine
K00473...
Protein erythro-5-hydroxylysine
Protein 5-galactosyloxylysine
00310 7/19/17
(c) Kanehisa Laboratories

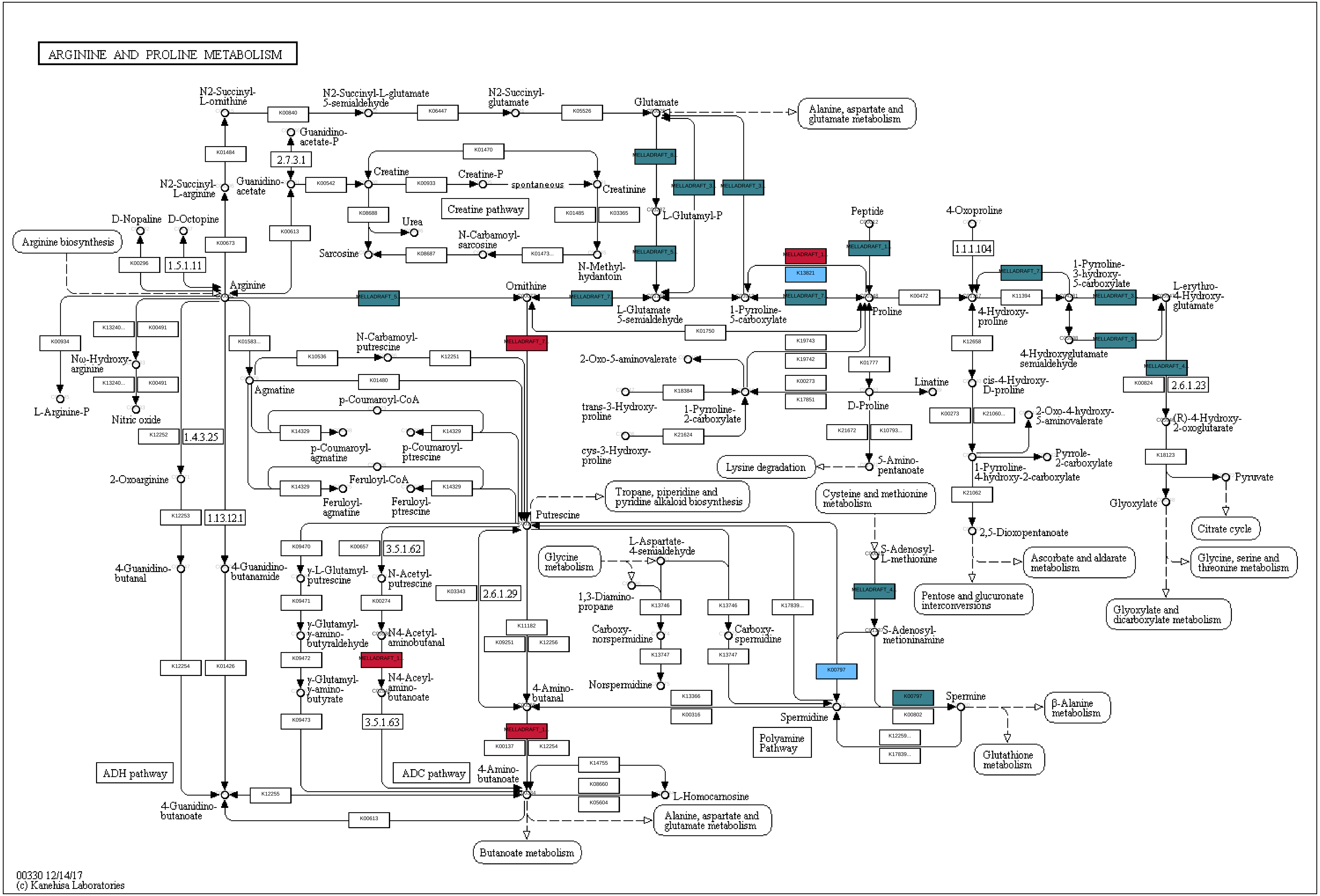
ARGININE AND PROLINE METABOLISM
N2-Succinyl-L-ornithine
N2-Succinyl-L-glutamate 5-semialdehyde
N2-Succinyl-glutamate
Glutamate
Alanine, aspartate and glutamate metabolism
K00840
K06447
K05526
Guanidino-acetate-P
2.7.3.1
K01484
K01470
Creatine
Creatine-P
spontaneous
Creatinine
N2-Succinyl-L-arginine
Guanidino-acetate
K00542
K00933
Creatine pathway
K01485
K03365
L-Glutamyl-P
Peptide
4-Oxoproline
D-Nopaline
D-Octopine
K08688
Urea
K00613
Arginine biosynthesis
K00673
Sarcosine
K08687
N-Carbamoyl-sarcosine
K01473...
N-Methyl-hydantoin
1.1.1.104
1-Pyrroline-3-hydroxy-5-carboxylate
K00296
1.5.1.11
Arginine
Ornithine
K13821
L-erythro-4-Hydroxy-glutamate
K00472
K11394
L-Glutamate 5-semialdehyde
1-Pyrroline-5-carboxylate
Proline
4-Hydroxy-proline
K00934
K13240...
K00491
K01583...
K01750
K19743
K12658
4-Hydroxyglutamate semialdehyde
K10536
N-Carbamoyl-putrescine
K12251
2-Oxo-5-aminovalerate
K19742
K01777
Nω-Hydroxy-arginine
K01480
Agmatine
K00273
Linatine
cis-4-Hydroxy-D-proline
K00824
2.6.1.23
K18384
K17851
D-Proline
L-Arginine-P
K13240...
K00491
p-Coumaroyl-CoA
trans-3-Hydroxy-proline
1-Pyrroline-2-carboxylate
K00273
K21060...
2-Oxo-4-hydroxy-5-aminovalerate
(R)-4-Hydroxy-2-oxoglutarate
Nitric oxide
K14329
K14329
K21624
K21672
K10793...
K12252
1.4.3.25
p-Coumaroyl-agmatine
p-Coumaroyl-ptrescine
cys-3-Hydroxy-proline
Pyrrole-2-carboxylate
K18123
Lysine degradation
5-Amino-pentanoate
1-Pyrroline-4-hydroxy-2-carboxylate
Feruloyl-CoA
2-Oxoarginine
K14329
K14329
Pyruvate
Cysteine and methionine metabolism
K21062
Tropane, piperidine and pyridine alkaloid biosynthesis
Feruloyl-agmatine
Feruloyl-ptrescine
Glyoxylate
K12253
1.13.12.1
Putrescine
Citrate cycle
2,5-Dioxopentanoate
K09470
K00657
3.5.1.62
L-Aspartate-4-semialdehyde
Glycine metabolism
S-Adenosyl-L-methionine
4-Guanidino-butanal
4-Guanidino-butanamide
γ-L-Glutamyl-putrescine
N-Acetyl-putrescine
Ascorbate and aldarate metabolism
Glycine, serine and threonine metabolism
K03343
2.6.1.29
1,3-Diamino-propane
K09471
K00274
K13746
K13746
K17839...
Pentose and glucuronate interconversions
Glyoxylate and dicarboxylate metabolism
K11182
γ-Glutamyl-γ-amino-butyraldehyde
N4-Acetyl-aminobutanal
Carboxy-norspermidine
Carboxy-spermidine
S-Adenosyl-metioninamine
K09472
K09251
K12256
K13747
K13747
K00797
K12254
K01426
γ-Glutamyl-γ-amino-butyrate
N4-Aceyl-amino-butanoate
4-Amino-butanal
Norspermidine
K13366
K00797
Spermine
β-Alanine metabolism
K00316
Spermidine
K00802
K09473
3.5.1.63
K00137
K12254
Polyamine Pathway
K12259...
K17839...
Glutathione metabolism
ADH pathway
ADC pathway
4-Amino-butanoate
K14755
K08660
L-Homocarnosine
K12255
K05604
4-Guanidino-butanoate
K00613
Alanine, aspartate and glutamate metabolism
Butanoate metabolism
00330 12/14/17
(c) Kanehisa Laboratories

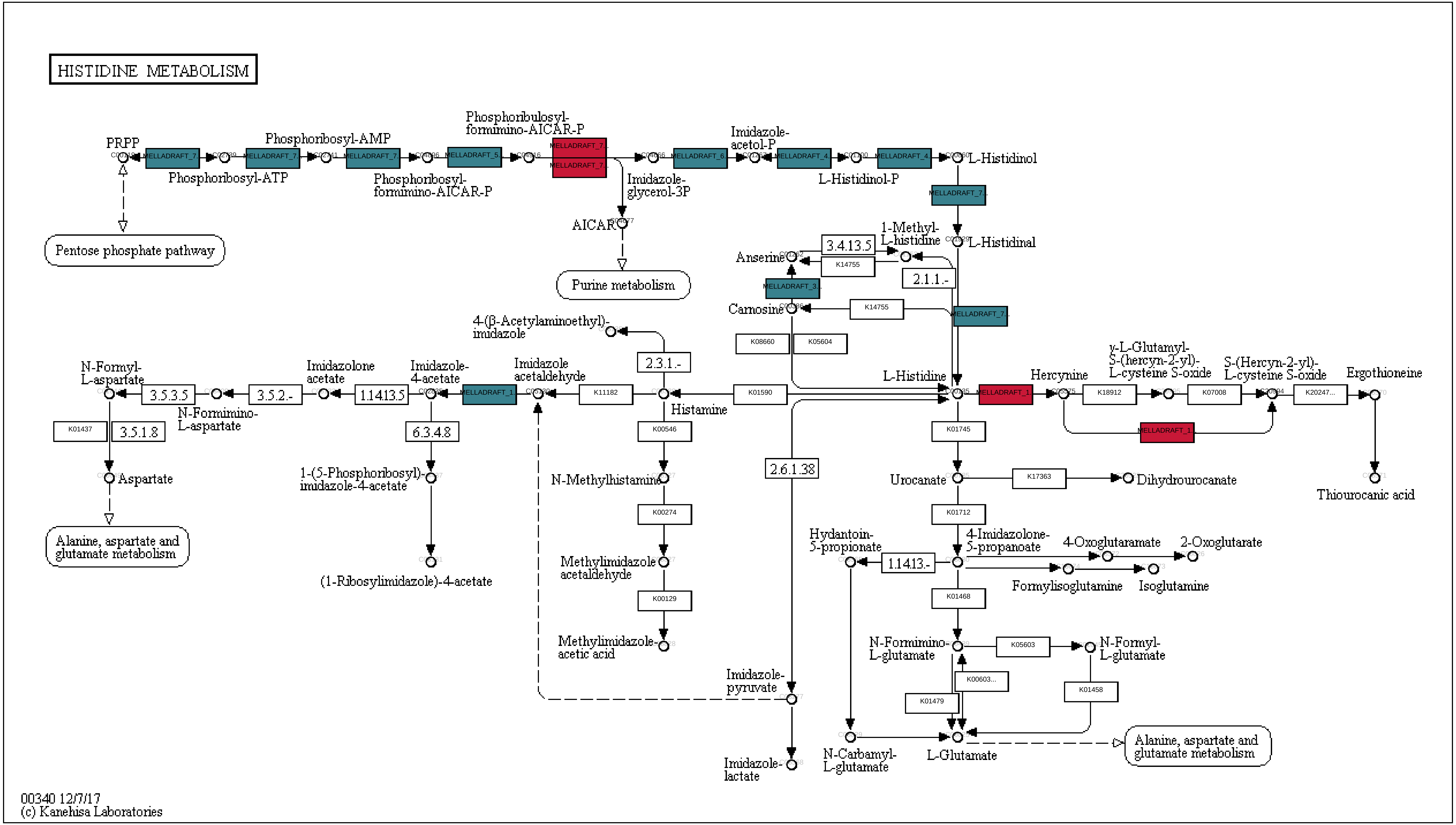
HISTIDINE METABOLISM
PRPP
Phosphoribosyl-ATP
Phosphoribosyl-AMP
Phosphoribosyl-formimino-AICAR-P
Phosphoribulosyl-formimino-AICAR-P
Imidazole-glycerol-3P
Imidazole-acetol-P
L-Histidinol-P
L-Histidinol
L-Histidinal
AICAR
Pentose phosphate pathway
Purine metabolism
1-Methyl-L-histidine
Anserine
Carnosine
3.4.13.5
2.1.1.-
K14755
K08660
K05604
L-Histidine
Hercynine
γ-L-Glutamyl-S-(hercyn-2-yl)-L-cysteine S-oxide
S-(Hercyn-2-yl)-L-cysteine S-oxide
Ergothioneine
Thiourocanic acid
K18912
K07008
K20247...
4-(β-Acetylaminoethyl)-imidazole
2.3.1.-
Imidazole acetaldehyde
K11182
K01590
Histamine
Imidazole-4-acetate
Imidazolone acetate
1.14.13.5
3.5.2.-
3.5.3.5
N-Formyl-L-aspartate
N-Formimino-L-aspartate
K01437
3.5.1.8
Aspartate
Alanine, aspartate and glutamate metabolism
6.3.4.8
1-(5-Phosphoribosyl)-imidazole-4-acetate
(1-Ribosylimidazole)-4-acetate
K00546
N-Methylhistamine
K00274
Methylimidazole acetaldehyde
K00129
Methylimidazole-acetic acid
2.6.1.38
Imidazole-pyruvate
Imidazole-lactate
K01745
Urocanate
K17363
Dihydrourocanate
K01712
4-Imidazolone-5-propanoate
Hydantoin-5-propionate
1.14.13.-
4-Oxoglutaramate
2-Oxoglutarate
Formylisoglutamine
Isoglutamine
K01468
N-Formimino-L-glutamate
K05603
N-Formyl-L-glutamate
K00603...
K01479
K01458
N-Carbamyl-L-glutamate
L-Glutamate
Alanine, aspartate and glutamate metabolism
00340 12/7/17
(c) Kanehisa Laboratories

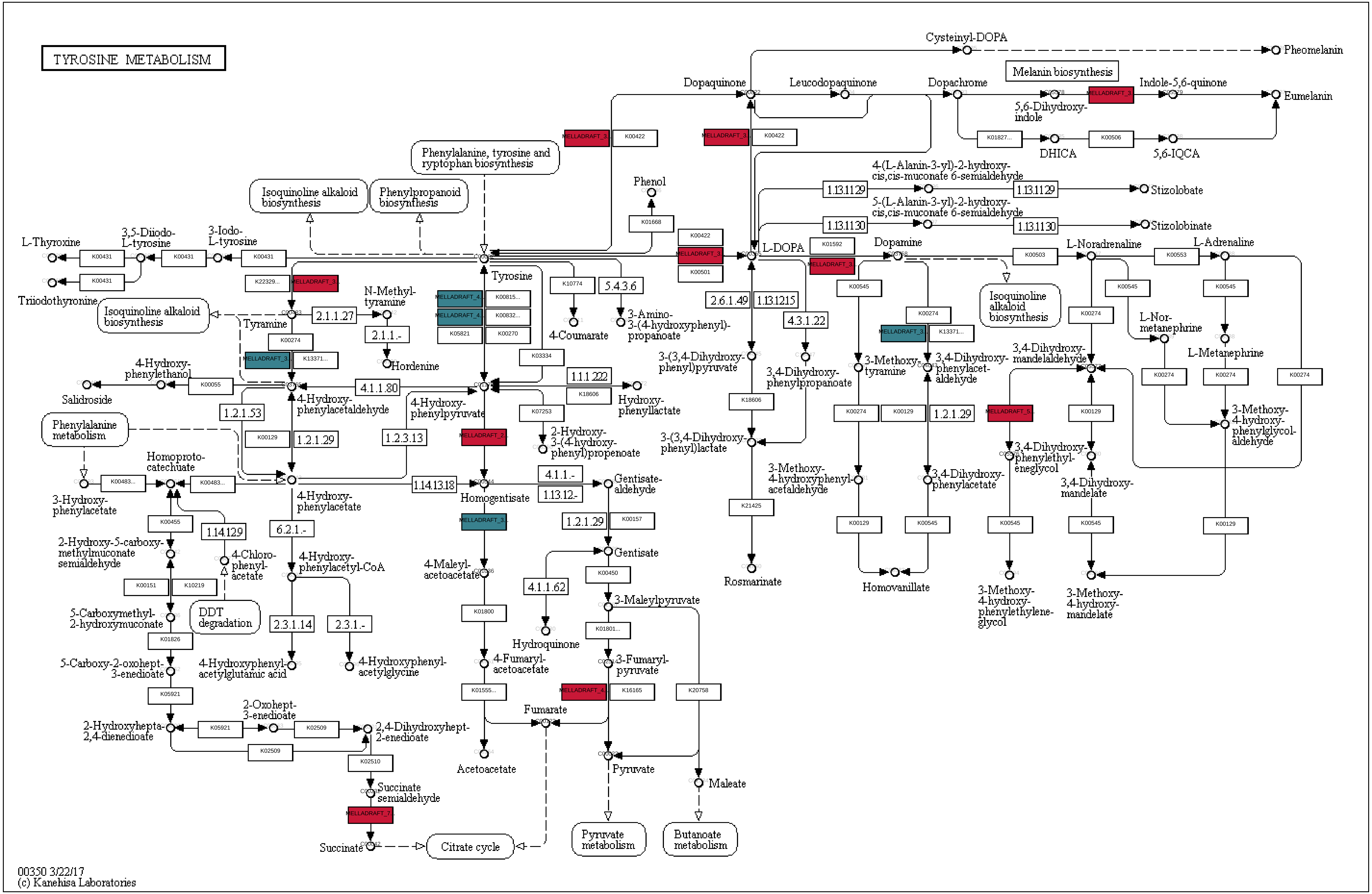

TYROSINE METABOLISM
Cysteinyl-DOPA
Pheomelanin
Melanin biosynthesis
Dopaquinone
Leucodopaquinone
Dopachrome
Indole-5,6-quinone
Eumelanin
5,6-Dihydroxy-indole
DHICA
5,6-IQCA
K01827...
K00506
MELLADRAFT_3
K00422
Phenylalanine, tyrosine and tryptophan biosynthesis
Isoquinoline alkaloid biosynthesis
Phenylpropanoid biosynthesis
Phenol
K01668
4-(L-Alanin-3-yl)-2-hydroxy-cis,cis-muconate 6-semialdehyde
1.13.11.29
Stizolobate
5-(L-Alanin-3-yl)-2-hydroxy-cis,cis-muconate 6-semialdehyde
1.13.11.30
Stizolobinate
L-Thyroxine
3,5-Diiodo-L-tyrosine
3-Iodo-L-tyrosine
K00431
Triiodothyronine
L-DOPA
K01592
Dopamine
K00503
L-Noradrenaline
K00553
L-Adrenaline
K00501
Tyrosine
K10774
5.4.3.6
2.6.1.49
1.13.12.15
4.3.1.22
K00545
K00274
K13371...
K22329...
N-Methyl-tyramine
2.1.1.27
2.1.1.-
Hordenine
Tyramine
K00815...
K00832...
K05821
K00270
K03334
4-Coumarate
3-Amino-3-(4-hydroxyphenyl)-propanoate
3-(3,4-Dihydroxy-phenyl)pyruvate
3,4-Dihydroxy-phenylpropanoate
3-Methoxy-tyramine
3,4-Dihydroxy-phenylacet-aldehyde
3,4-Dihydroxy-mandelaldehyde
L-Nor-metanephrine
L-Metanephrine
4-Hydroxy-phenylethanol
K00055
Salidroside
4.1.1.80
4-Hydroxy-phenylacetaldehyde
4-Hydroxy-phenylpyruvate
1.1.1.222
K18606
Hydroxy-phenyllactate
K07253
2-Hydroxy-3-(4-hydroxy-phenyl)propenoate
3-(3,4-Dihydroxy-phenyl)lactate
1.2.1.53
K00129
1.2.1.29
1.2.3.13
Phenylalanine metabolism
3-Methoxy-4-hydroxy-phenylglycol-aldehyde
3,4-Dihydroxy-phenylethyleneglycol
3,4-Dihydroxy-mandelate
3-Methoxy-4-hydroxyphenyl-acetaldehyde
3,4-Dihydroxy-phenylacetate
Homoproto-catechuate
K00483...
1.14.13.18
4.1.1.-
Gentisate-aldehyde
1.13.12.-
Homogentisate
3-Hydroxy-phenylacetate
4-Hydroxy-phenylacetate
K00455
1.14.12.9
6.2.1.-
K00157
Gentisate
K21425
Rosmarinate
Homovanillate
2-Hydroxy-5-carboxy-methylmuconate semialdehyde
4-Chloro-phenyl-acetate
4-Hydroxy-phenylacetyl-CoA
4-Maleyl-acetoacetate
4.1.1.62
K00450
3-Methoxy-4-hydroxy-phenylethylene-glycol
3-Methoxy-4-hydroxy-mandelate
K00151
K10219
5-Carboxymethyl-2-hydroxymuconate
DDT degradation
2.3.1.14
2.3.1.-
K01800
Hydroquinone
3-Maleylpyruvate
K01801...
K01826
5-Carboxy-2-oxohept-3-enedioate
4-Hydroxyphenyl-acetylglutamic acid
4-Hydroxyphenyl-acetylglycine
4-Fumaryl-acetoacetate
3-Fumaryl-pyruvate
K05921
K01555...
K16165
K20758
Fumarate
2-Oxohept-3-enedioate
K02509
2,4-Dihydroxyhept-2-enedioate
2-Hydroxyhepta-2,4-dienedioate
K02510
Acetoacetate
Pyruvate
Maleate
Succinate semialdehyde
MELLADRAFT_7
Succinate
Citrate cycle
Pyruvate metabolism
Butanoate metabolism
00350 3/22/17
(c) Kanehisa Laboratories

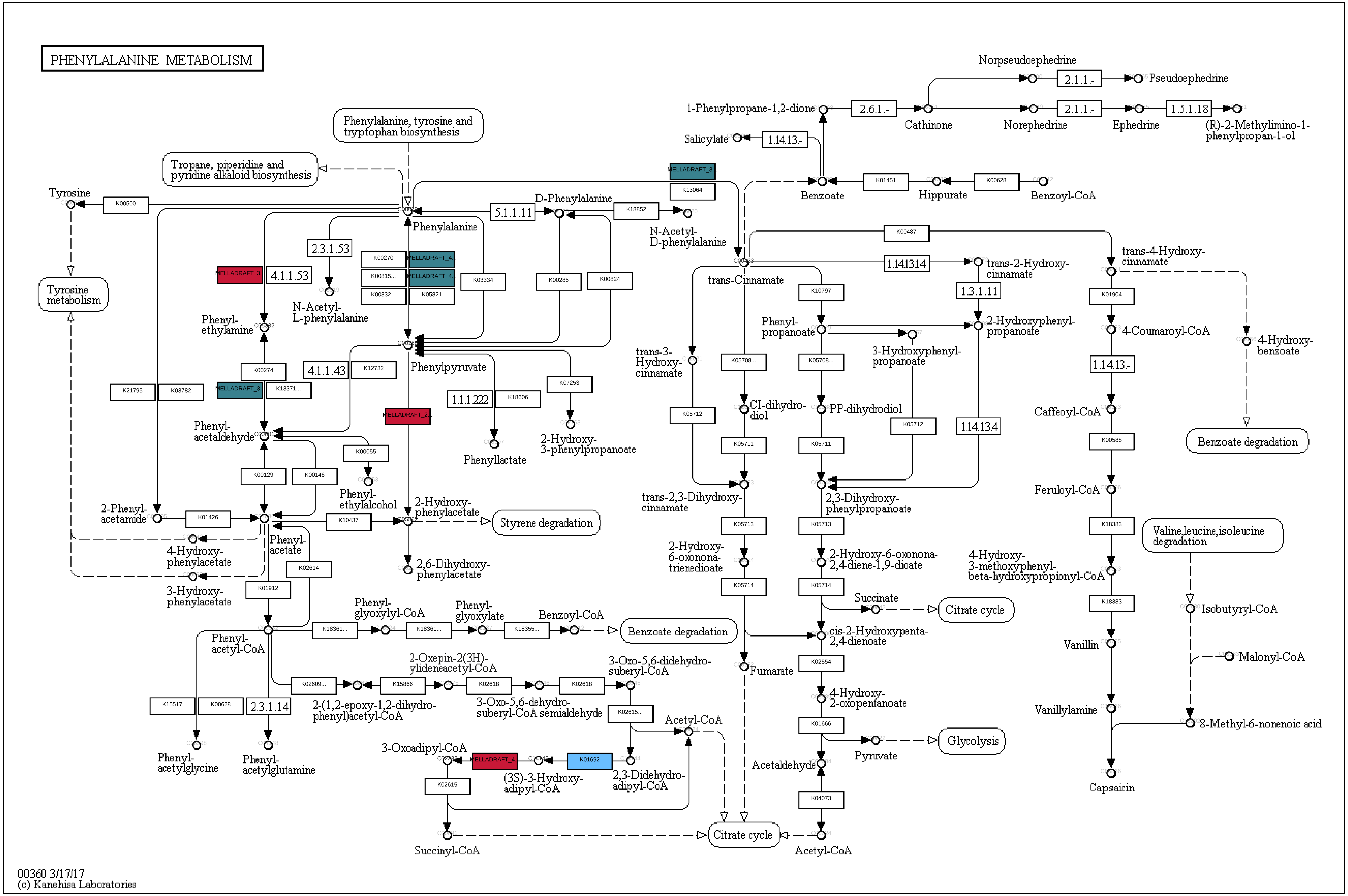
PHENYLALANINE METABOLISM
Phenylalanine, tyrosine and tryptophan biosynthesis
Tropane, piperidine and pyridine alkaloid biosynthesis
Tyrosine
K00500
Tyrosine metabolism
Phenylalanine
5.1.1.11
D-Phenylalanine
K18852
N-Acetyl-D-phenylalanine
2.3.1.53
N-Acetyl-L-phenylalanine
4.1.1.53
K00270
K00815...
K00832...
K05821
K03334
K00285
K00824
K13064
Phenyl-ethylamine
K00274
K13371...
4.1.1.43
K12732
Phenylpyruvate
K21795
K03782
1.1.1.222
K18606
K07253
Phenyllactate
2-Hydroxy-3-phenylpropanoate
Phenyl-acetaldehyde
K00055
K00129
K00146
Phenyl-ethylalcohol
2-Phenyl-acetamide
K01426
K10437
2-Hydroxy-phenylacetate
Styrene degradation
4-Hydroxy-phenylacetate
Phenyl-acetate
2,6-Dihydroxy-phenylacetate
3-Hydroxy-phenylacetate
K02614
K01912
Phenyl-glyoxylyl-CoA
Phenyl-glyoxylate
Benzoyl-CoA
K18361...
K18355...
Benzoate degradation
Phenyl-acetyl-CoA
2-Oxepin-2(3H)-ylideneacetyl-CoA
3-Oxo-5,6-didehydro-suberyl-CoA
K02609...
K15866
K02618
K15517
K00628
2.3.1.14
2-(1,2-epoxy-1,2-dihydro-phenyl)acetyl-CoA
3-Oxo-5,6-dehydro-suberyl-CoA semialdehyde
K02615...
Acetyl-CoA
Phenyl-acetylglycine
Phenyl-acetylglutamine
3-Oxoadipyl-CoA
K01692
(3S)-3-Hydroxy-adipyl-CoA
2,3-Didehydro-adipyl-CoA
K02615
Succinyl-CoA
Citrate cycle
Acetyl-CoA
Norpseudoephedrine
2.1.1.-
Pseudoephedrine
1-Phenylpropane-1,2-dione
2.6.1.-
Cathinone
Norephedrine
Ephedrine
1.5.1.18
(R)-2-Methylimino-1-phenylpropan-1-ol
Salicylate
1.14.13.-
Benzoate
K01451
Hippurate
Benzoyl-CoA
K00487
trans-Cinnamate
1.14.13.14
trans-2-Hydroxy-cinnamate
1.3.1.11
trans-4-Hydroxy-cinnamate
K10797
K01904
Phenyl-propanoate
2-Hydroxyphenyl-propanoate
4-Coumaroyl-CoA
3-Hydroxyphenyl-propanoate
4-Hydroxy-benzoate
trans-3-Hydroxy-cinnamate
K05708...
1.14.13.-
K05712
CI-dihydro-diol
PP-dihydrodiol
1.14.13.4
Caffeoyl-CoA
K05711
K00588
Benzoate degradation
trans-2,3-Dihydroxy-cinnamate
2,3-Dihydroxy-phenylpropanoate
Feruloyl-CoA
K05713
K18383
Valine, leucine, isoleucine degradation
2-Hydroxy-6-oxonona-trienedioate
2-Hydroxy-6-oxonona-2,4-diene-1,9-dioate
4-Hydroxy-3-methoxyphenyl-beta-hydroxypropionyl-CoA
K05714
Succinate
Citrate cycle
Isobutyryl-CoA
cis-2-Hydroxypenta-2,4-dienoate
Vanillin
Malonyl-CoA
Fumarate
K02554
4-Hydroxy-2-oxopentanoate
Vanillylamine
8-Methyl-6-nonenoic acid
K01666
Glycolysis
Pyruvate
Acetaldehyde
Capsaicin
K04073
Citrate cycle
Acetyl-CoA
00360 3/17/17
(c) Kanehisa Laboratories

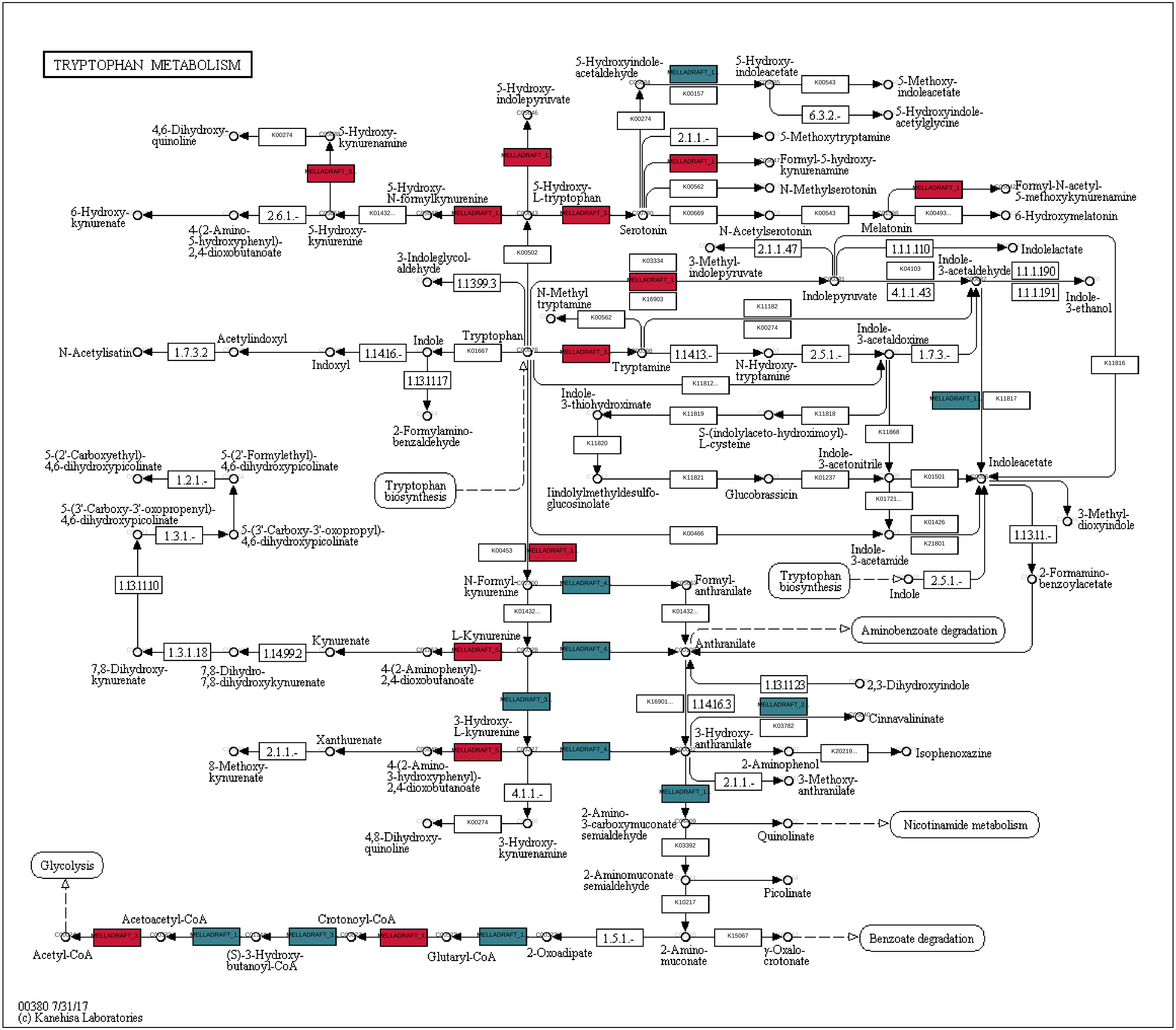
TRYPTOPHAN METABOLISM
5-Hydroxyindole-acetaldehyde
5-Hydroxy-indoleacetate
5-Methoxy-indoleacetate
5-Hydroxyindole-acetylglycine
5-Hydroxy-indolepyruvate
4,6-Dihydroxy-quinoline
5-Hydroxy-kynurenamine
5-Methoxytryptamine
Formyl-5-hydroxy-kynurenamine
N-Methylserotonin
Formyl-N-acetyl-5-methoxykynurenamine
6-Hydroxy-kynurenate
4-(2-Amino-5-hydroxyphenyl)-2,4-dioxobutanoate
5-Hydroxy-kynurenine
5-Hydroxy-N-formylkynurenine
5-Hydroxy-L-tryptophan
Serotonin
N-Acetylserotonin
Melatonin
6-Hydroxymelatonin
3-Indoleglycol-aldehyde
3-Methyl-indolepyruvate
Indolepyruvate
Indolelactate
Indole-3-acetaldehyde
Indole-3-ethanol
N-Methyl tryptamine
N-Acetylisatin
Acetylindoxyl
Indoxyl
Indole
Tryptophan
Tryptamine
N-Hydroxy-tryptamine
Indole-3-acetaldoxime
2-Formylamino-benzaldehyde
Indole-3-thiohydroximate
S-(indolylaceto-hydroximoyl)-L-cysteine
5-(2'-Carboxyethyl)-4,6-dihydroxypicolinate
5-(2'-Formylethyl)-4,6-dihydroxypicolinate
Tryptophan biosynthesis
Indolylmethyldesulfo-glucosinolate
Glucobrassicin
Indole-3-acetonitrile
Indoleacetate
5-(3'-Carboxy-3'-oxopropenyl)-4,6-dihydroxypicolinate
5-(3'-Carboxy-3'-oxopropyl)-4,6-dihydroxypicolinate
3-Methyl-dioxyindole
Indole-3-acetamide
N-Formyl-kynurenine
Formyl-anthranilate
Tryptophan biosynthesis
Indole
2-Formamino-benzoylacetate
Aminobenzoate degradation
L-Kynurenine
Kynurenate
Anthranilate
7,8-Dihydroxy-kynurenate
7,8-Dihydro-7,8-dihydroxykynurenate
4-(2-Aminophenyl)-2,4-dioxobutanoate
2,3-Dihydroxyindole
Cinnavalininate
3-Hydroxy-L-kynurenine
Xanthurenate
3-Hydroxy-anthranilate
Isophenoxazine
2-Aminophenol
8-Methoxy-kynurenate
4-(2-Amino-3-hydroxyphenyl)-2,4-dioxobutanoate
3-Methoxy-anthranilate
2-Amino-3-carboxymuconate semialdehyde
Quinolinate
Nicotinamide metabolism
4,8-Dihydroxy-quinoline
3-Hydroxy-kynurenamine
Glycolysis
2-Aminomuconate semialdehyde
Picolinate
Acetoacetyl-CoA
Crotonoyl-CoA
Acetyl-CoA
(S)-3-Hydroxy-butanoyl-CoA
Glutaryl-CoA
2-Oxoadipate
2-Amino-muconate
γ-Oxalo-crotonate
Benzoate degradation
K00157
K00274
K00543
6.3.2.-
2.1.1.-
K00562
K00669
K00493...
K00274
K01432...
2.6.1.-
K00502
2.1.1.47
1.1.1.110
K03334
K04103
1.1.1.190
1.13.99.3
K16903
4.1.1.43
1.1.1.191
K11182
K00274
K00562
1.7.3.2
1.14.16.-
K01667
1.14.13.-
2.5.1.-
1.7.3.-
K11816
1.13.11.17
K11812...
K11817
K11819
K11818
K11868
K11820
1.2.1.-
K11821
K01237
K01501
K01721...
1.3.1.-
K00466
K01426
K21801
1.13.11.-
K00453
1.13.11.10
2.5.1.-
K01432...
K01432...
1.3.1.18
1.14.99.2
1.13.11.23
K16901...
1.14.16.3
K03782
2.1.1.-
K20219...
2.1.1.-
4.1.1.-
K00274
K03392
K10217
1.5.1.-
K15067
MELLADRAFT_1...
MELLADRAFT_3...
MELLADRAFT_4...
MELLADRAFT_5...
MELLADRAFT_2...
00380 7/31/17
(c) Kanehisa Laboratories

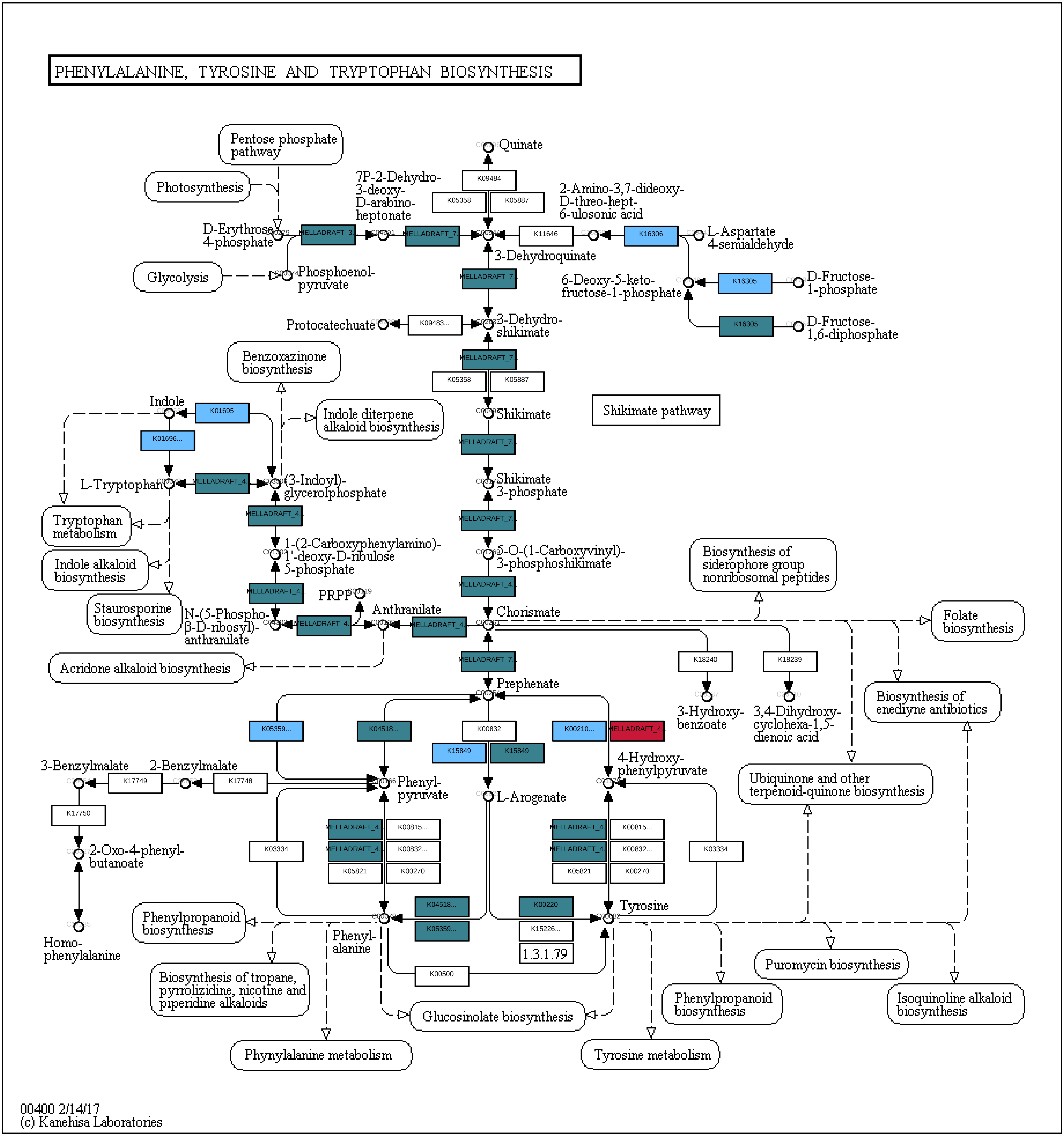
PHENYLALANINE, TYROSINE AND TRYPTOPHAN BIOSYNTHESIS
Pentose phosphate pathway
Photosynthesis
Glycolysis
D-Erythrose-4-phosphate
Phosphoenol-pyruvate
7P-2-Dehydro-3-deoxy-D-arabino-heptonate
Quinate
K09484
K05358
K05887
2-Amino-3,7-dideoxy-D-threo-hept-6-ulosonic acid
K11646
K16306
L-Aspartate 4-semialdehyde
3-Dehydroquinate
6-Deoxy-5-keto-fructose-1-phosphate
K16305
D-Fructose-1-phosphate
D-Fructose-1,6-diphosphate
Protocatechuate
K09483...
3-Dehydro-shikimate
Benzoxazinone biosynthesis
Indole
K01695
K01696...
Indole diterpene alkaloid biosynthesis
Shikimate
Shikimate pathway
L-Tryptophan
(3-Indoyl)-glycerolphosphate
Shikimate 3-phosphate
Tryptophan metabolism
Indole alkaloid biosynthesis
1-(2-Carboxyphenylamino)-1'-deoxy-D-ribulose 5-phosphate
5-O-(1-Carboxyvinyl)-3-phosphoshikimate
Biosynthesis of siderophore group nonribosomal peptides
Staurosporine biosynthesis
PRPP
N-(5-Phospho-β-D-ribosyl)-anthranilate
Anthranilate
Chorismate
Folate biosynthesis
Acridone alkaloid biosynthesis
K18240
K18239
Prephenate
3-Hydroxy-benzoate
3,4-Dihydroxy-cyclohexa-1,5-dienoic acid
Biosynthesis of enediyne antibiotics
K05359...
K04518...
K00832
K00210...
K15849
4-Hydroxy-phenylpyruvate
3-Benzylmalate
2-Benzylmalate
K17749
K17748
Phenyl-pyruvate
L-Arogenate
Ubiquinone and other terpenoid-quinone biosynthesis
K17750
K03334
K00815...
K00832...
K05821
K00270
2-Oxo-4-phenyl-butanoate
K00220
K15226...
1.3.1.79
Tyrosine
Homo-phenylalanine
Phenylpropanoid biosynthesis
Phenyl-alanine
K00500
Biosynthesis of tropane, pyrrolizidine, nicotine and piperidine alkaloids
Glucosinolate biosynthesis
Puromycin biosynthesis
Isoquinoline alkaloid biosynthesis
Phynylalanine metabolism
Tyrosine metabolism
00400 2/14/17
(c) Kanehisa Laboratories

# 6. Metabolism of other amino acids

| MAP | PATHWAY |
|---|---|
| **410** | beta-Alanine metabolism |
| **430** | Taurine and hypotaurine metabolism |
| **440** | Phosphonate and phosphinate metabolism |
| **450** | Selenocompound metabolism |
| **460** | Cyanoamino acid metabolism |
| **480** | Glutathione metabolism |

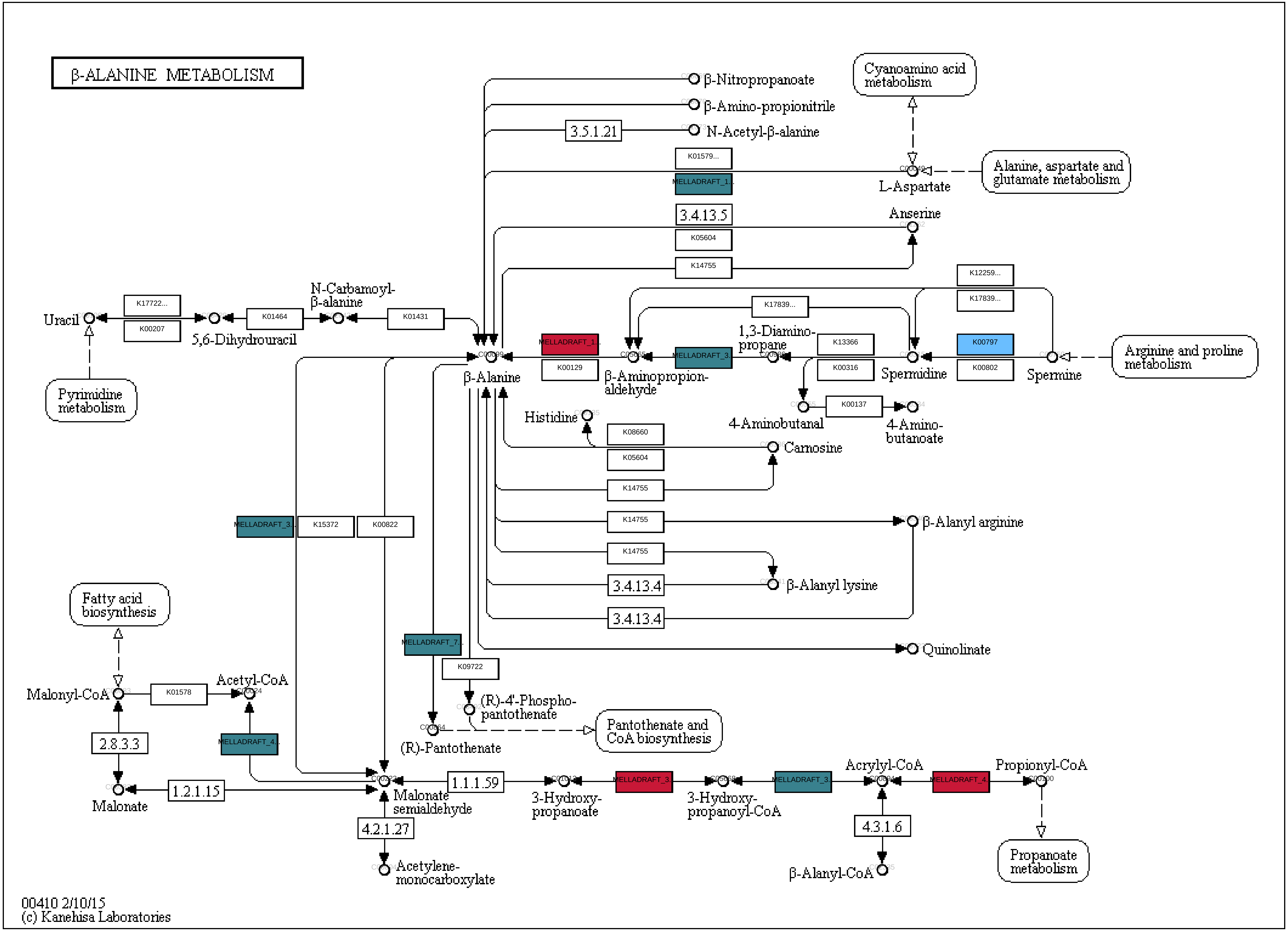
β-ALANINE METABOLISM
β-Nitropropanoate
β-Amino-propionitrile
3.5.1.21
N-Acetyl-β-alanine
Cyanoamino acid metabolism
K01579...
MELLADRAFT_1...
L-Aspartate
Alanine, aspartate and glutamate metabolism
3.4.13.5
K05604
K14755
Anserine
K12259...
K17839...
K17839...
Uracil
K17722...
K00207
5,6-Dihydrouracil
K01464
N-Carbamoyl-β-alanine
K01431
MELLADRAFT_1...
K00129
β-Alanine
β-Aminopropion-aldehyde
MELLADRAFT_3...
1,3-Diamino-propane
K13366
K00316
Spermidine
K00797
K00802
Spermine
Arginine and proline metabolism
Pyrimidine metabolism
Histidine
K08660
K05604
Carnosine
4-Aminobutanal
K00137
4-Amino-butanoate
K14755
K14755
β-Alanyl arginine
MELLADRAFT_3...
K15372
K00822
K14755
β-Alanyl lysine
3.4.13.4
3.4.13.4
Quinolinate
Fatty acid biosynthesis
MELLADRAFT_7...
K09722
Malonyl-CoA
K01578
Acetyl-CoA
(R)-4'-Phospho-pantothenate
Pantothenate and CoA biosynthesis
2.8.3.3
MELLADRAFT_4...
(R)-Pantothenate
1.1.1.59
MELLADRAFT_3...
MELLADRAFT_3...
Acrylyl-CoA
MELLADRAFT_4...
Propionyl-CoA
Malonate
1.2.1.15
Malonate semialdehyde
3-Hydroxy-propanoate
3-Hydroxy-propanoyl-CoA
4.2.1.27
4.3.1.6
Acetylene-monocarboxylate
β-Alanyl-CoA
Propanoate metabolism
00410 2/10/15
(c) Kanehisa Laboratories

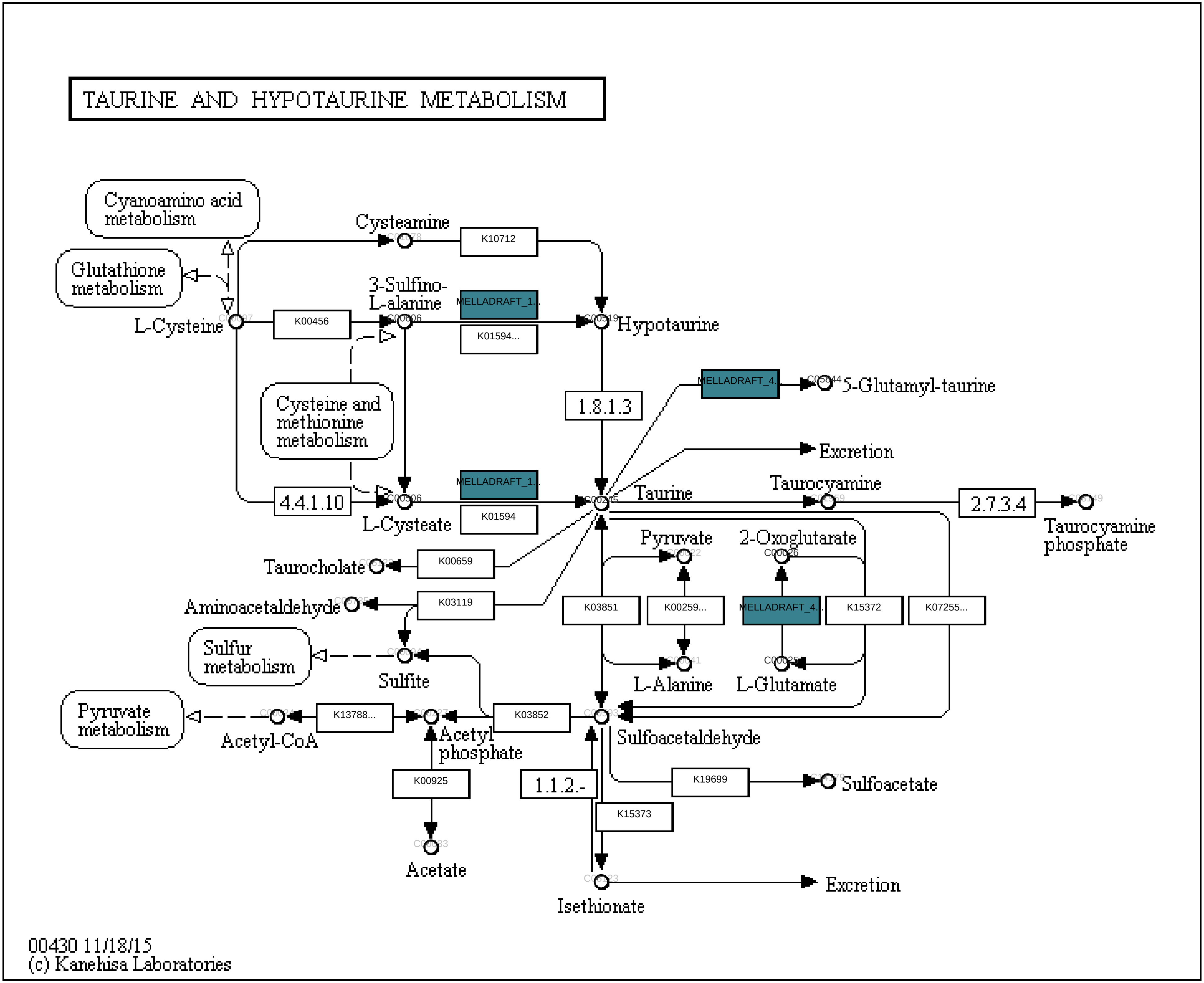
TAURINE AND HYPOTAURINE METABOLISM
Cyanoamino acid metabolism
Glutathione metabolism
L-Cysteine
Cysteamine
K10712
3-Sulfino-L-alanine
K00456
MELLADRAFT_1...
K01594...
Hypotaurine
Cysteine and methionine metabolism
1.8.1.3
MELLADRAFT_4...
5-Glutamyl-taurine
Excretion
4.4.1.10
MELLADRAFT_1...
K01594
L-Cysteate
Taurine
Taurocyamine
2.7.3.4
Taurocyamine phosphate
Taurocholate
K00659
Pyruvate
2-Oxoglutarate
C00026
Aminoacetaldehyde
K03119
K03851
K00259...
MELLADRAFT_4...
K15372
K07255...
Sulfur metabolism
Sulfite
L-Alanine
C00025
L-Glutamate
Pyruvate metabolism
K13788...
K03852
Acetyl-CoA
Acetyl phosphate
Sulfoacetaldehyde
K00925
1.1.2.-
K19699
Sulfoacetate
K15373
Acetate
Excretion
Isethionate
00430 11/18/15
(c) Kanehisa Laboratories

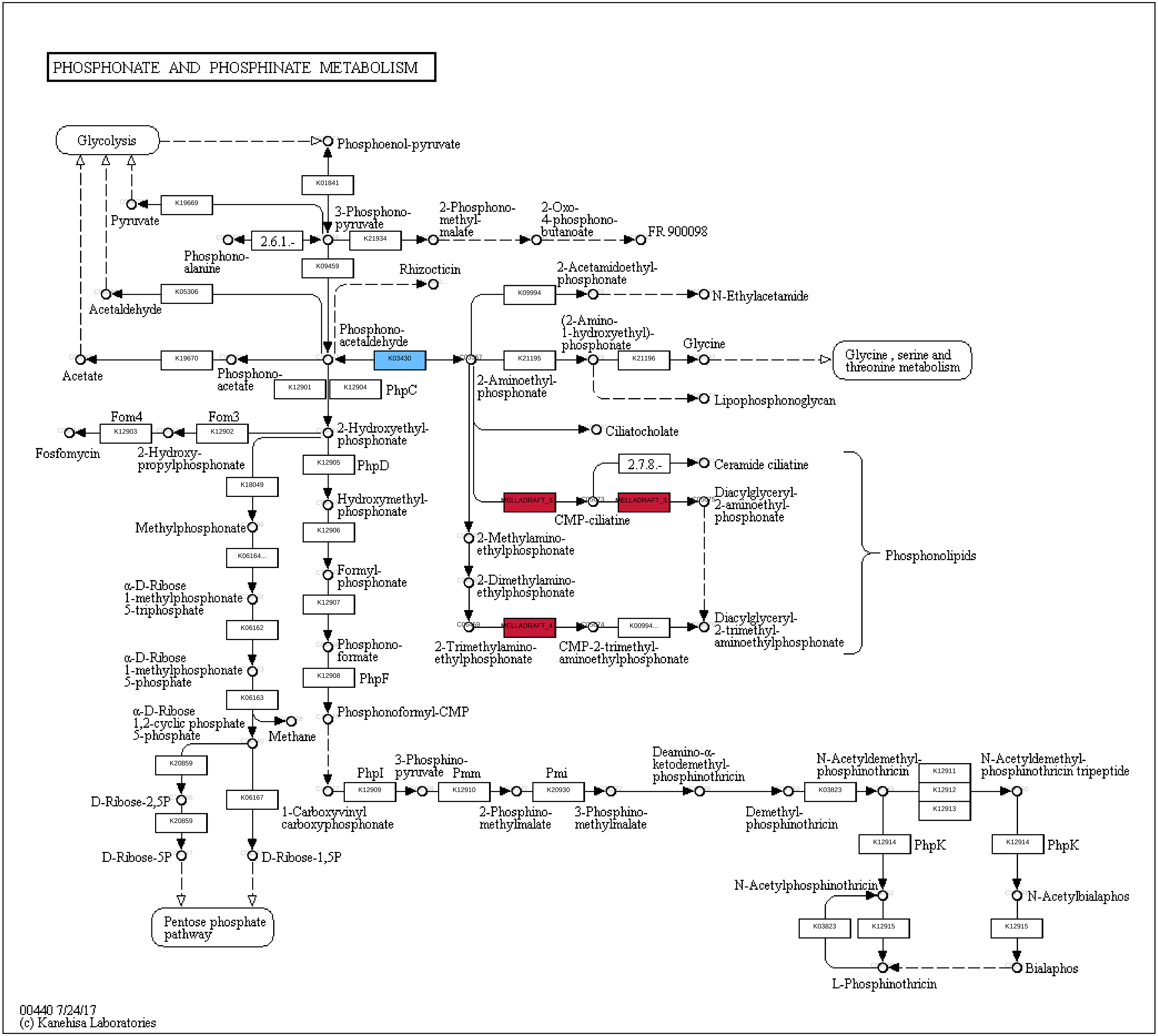
PHOSPHONATE AND PHOSPHINATE METABOLISM
Glycolysis
Phosphoenol-pyruvate
K01841
K19669
Pyruvate
3-Phosphono-pyruvate
2.6.1.-
K21934
2-Phosphono-methyl-malate
2-Oxo-4-phosphono-butanoate
FR 900098
Phosphono-alanine
K09459
Rhizocticin
K05306
Acetaldehyde
2-Acetamidoethyl-phosphonate
K09994
N-Ethylacetamide
Phosphono-acetaldehyde
(2-Amino-1-hydroxyethyl)-phosphonate
Glycine
K19670
K03430
K21195
K21196
Glycine, serine and threonine metabolism
Acetate
Phosphono-acetate
K12901
K12904
PhpC
2-Aminoethyl-phosphonate
Lipophosphonoglycan
Fom4
Fom3
K12903
K12902
2-Hydroxyethyl-phosphonate
Ciliatocholate
Fosfomycin
2-Hydroxy-propylphosphonate
K12905
PhpD
2.7.8.-
Ceramide ciliatine
K18049
Hydroxymethyl-phosphonate
MELLADRAFT_3
MELLADRAFT_3
Diacylglyceryl-2-aminoethyl-phosphonate
CMP-ciliatine
Methylphosphonate
K12906
2-Methylamino-ethylphosphonate
Phosphonolipids
K06164...
Formyl-phosphonate
α-D-Ribose 1-methylphosphonate 5-triphosphate
2-Dimethylamino-ethylphosphonate
K12907
K06162
MELLADRAFT_4
K00994...
Diacylglyceryl-2-trimethyl-aminoethylphosphonate
Phosphono-formate
2-Trimethylamino-ethylphosphonate
CMP-2-trimethyl-aminoethylphosphonate
α-D-Ribose 1-methylphosphonate 5-phosphate
K12908
PhpF
K06163
Phosphonoformyl-CMP
α-D-Ribose 1,2-cyclic phosphate 5-phosphate
Methane
3-Phosphino-pyruvate
Deamino-α-ketodemethyl-phosphinothricin
N-Acetyldemethyl-phosphinothricin
N-Acetyldemethyl-phosphinothricin tripeptide
K20859
PhpI
Prnn
Pmi
K12911
K12909
K12910
K20930
K03823
K12912
K12913
D-Ribose-2,5P
K06167
K20859
1-Carboxyvinyl carboxyphosphonate
2-Phosphino-methylmalate
3-Phosphino-methylmalate
Demethyl-phosphinothricin
K12914
PhpK
K12914
PhpK
D-Ribose-5P
D-Ribose-1,5P
N-Acetylphosphinothricin
N-Acetylbialaphos
Pentose phosphate pathway
K03823
K12915
K12915
Bialaphos
L-Phosphinothricin
00440 7/24/17
(c) Kanehisa Laboratories

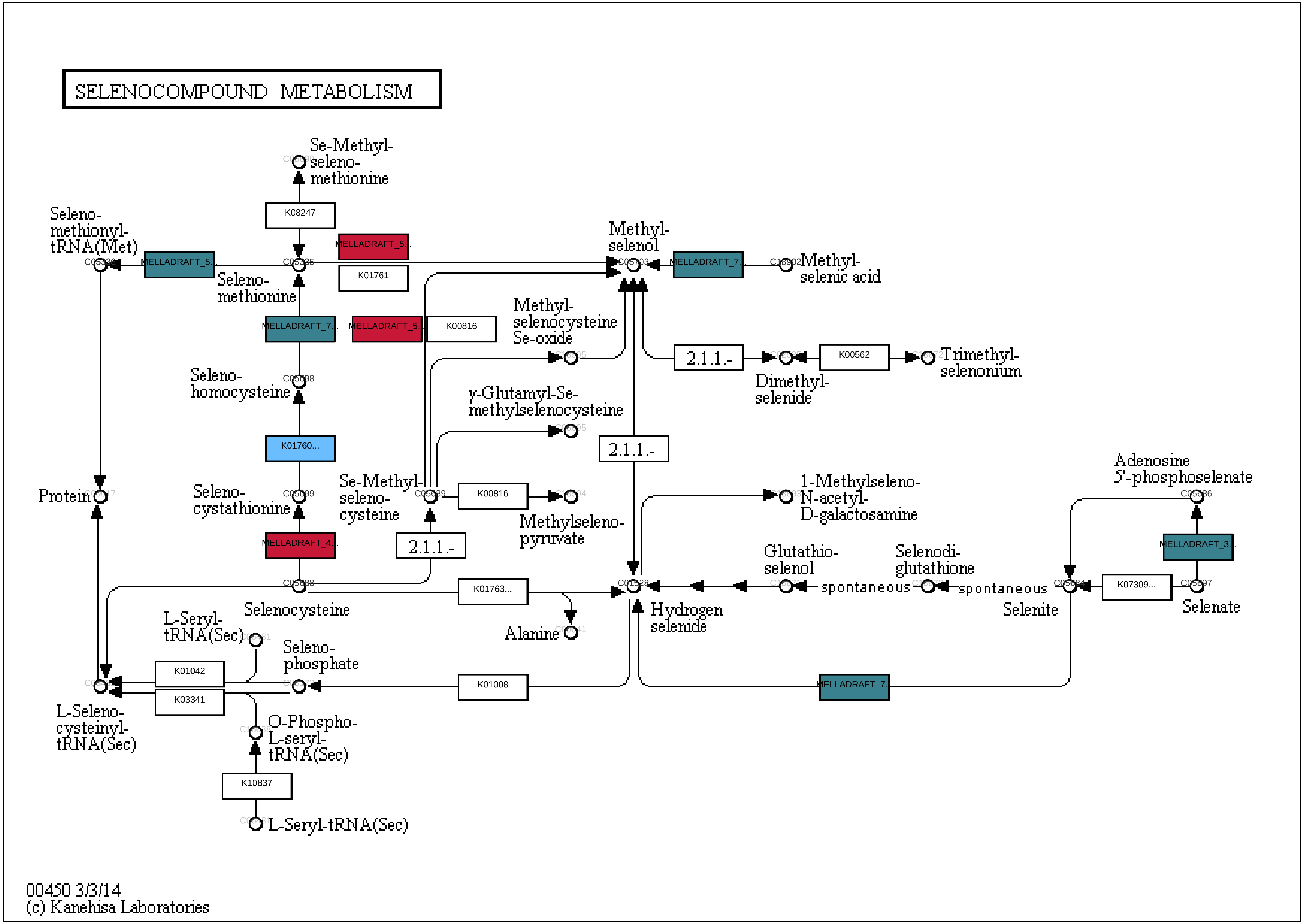
SELENOCOMPOUND METABOLISM
Se-Methyl-seleno-methionine
K08247
MELLADRAFT_5...
K01761
Seleno-methionyl-tRNA(Met)
C05336
MELLADRAFT_5...
C05335
Seleno-methionine
Methyl-selenol
C05703
MELLADRAFT_7...
C18902
Methyl-selenic acid
MELLADRAFT_7...
MELLADRAFT_5...
K00816
Methyl-selenocysteine Se-oxide
2.1.1.-
K00562
Trimethyl-selenonium
Dimethyl-selenide
Seleno-homocysteine
C05698
γ-Glutamyl-Se-methylselenocysteine
K01760...
2.1.1.-
Adenosine 5'-phosphoselenate
C05696
Protein
Seleno-cystathionine
C05699
Se-Methyl-seleno-cysteine
C05689
K00816
Methylseleno-pyruvate
1-Methylseleno-N-acetyl-D-galactosamine
MELLADRAFT_3...
MELLADRAFT_4...
2.1.1.-
Glutathio-selenol
Selenodi-glutathione
C05688
K01763...
C01528
spontaneous
spontaneous
C05684
K07309...
C05697
Selenocysteine
Alanine
Hydrogen selenide
Selenite
Selenate
L-Seryl-tRNA(Sec)
K01042
Seleno-phosphate
K03341
K01008
MELLADRAFT_7
L-Seleno-cysteinyl-tRNA(Sec)
O-Phospho-L-seryl-tRNA(Sec)
K10837
L-Seryl-tRNA(Sec)
00450 3/3/14
(c) Kanehisa Laboratories

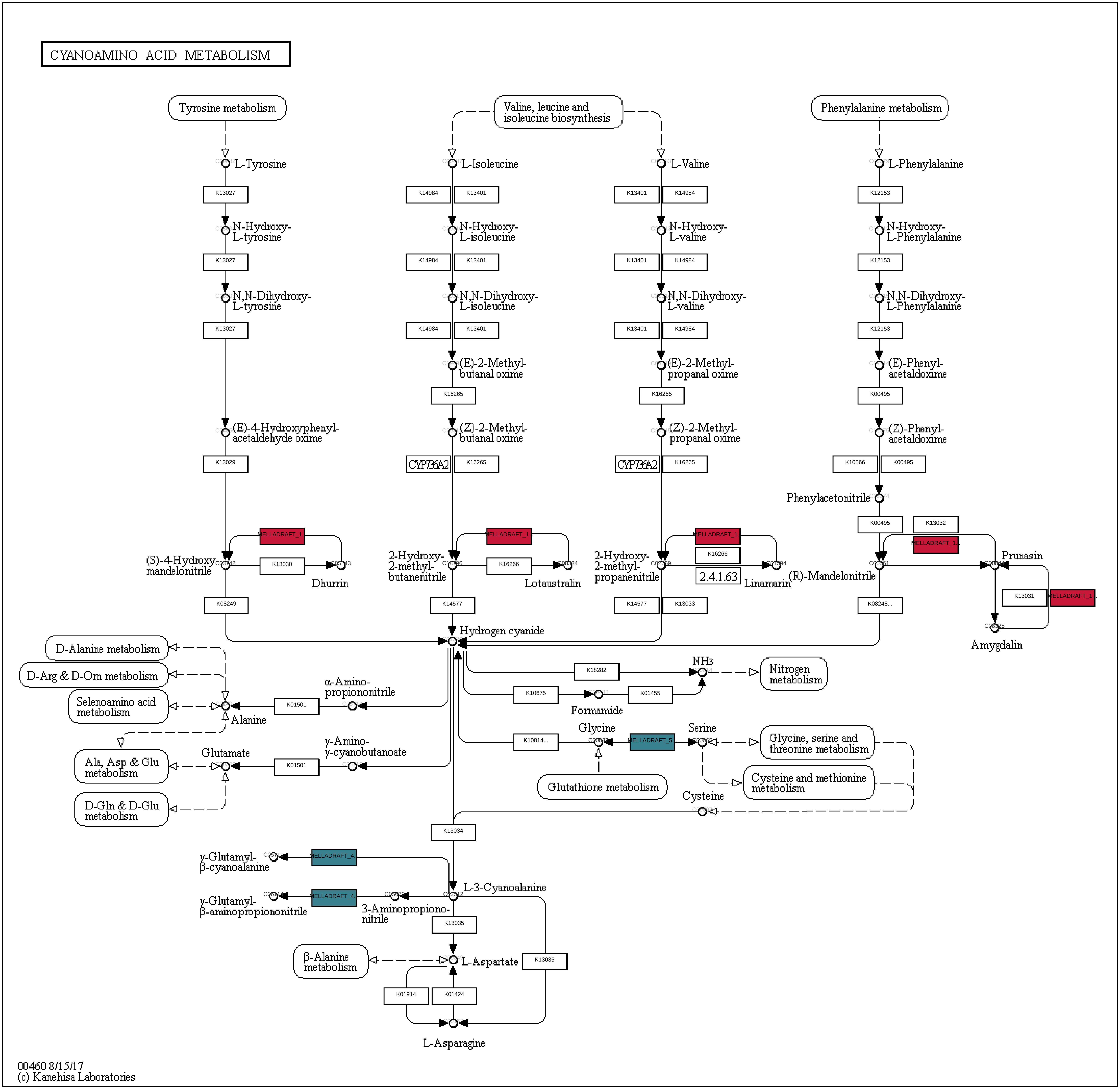
CYANOAMINO ACID METABOLISM
Tyrosine metabolism
Valine, leucine and isoleucine biosynthesis
Phenylalanine metabolism
L-Tyrosine
L-Isoleucine
L-Valine
L-Phenylalanine
K13027
K14984
K13401
K12153
N-Hydroxy-L-tyrosine
N-Hydroxy-L-isoleucine
N-Hydroxy-L-valine
N-Hydroxy-L-Phenylalanine
N,N-Dihydroxy-L-tyrosine
N,N-Dihydroxy-L-isoleucine
N,N-Dihydroxy-L-valine
N,N-Dihydroxy-L-Phenylalanine
(E)-2-Methyl-butanal oxime
(E)-2-Methyl-propanal oxime
(E)-Phenyl-acetaldoxime
K16265
K00495
(E)-4-Hydroxyphenyl-acetaldehyde oxime
(Z)-2-Methyl-butanal oxime
(Z)-2-Methyl-propanal oxime
(Z)-Phenyl-acetaldoxime
K13029
CYP736A2
K10566
Phenylacetonitrile
K13032
MELLADRAFT_1
(S)-4-Hydroxy-mandelonitrile
K13030
Dhurrin
2-Hydroxy-2-methyl-butanenitrile
K16266
Lotaustralin
2-Hydroxy-2-methyl-propanenitrile
2.4.1.63
Linamarin
(R)-Mandelonitrile
Prunasin
K13031
Amygdalin
K08249
K14577
K13033
K08248...
Hydrogen cyanide
D-Alanine metabolism
D-Arg & D-Orn metabolism
Selenoamino acid metabolism
Alanine
K01501
α-Amino-propiononitrile
K18282
NH3
Nitrogen metabolism
K10675
Formamide
K01455
Ala, Asp & Glu metabolism
Glutamate
γ-Amino-γ-cyanobutanoate
D-Gln & D-Glu metabolism
K10814...
Glycine
MELLADRAFT_5
Serine
Glycine, serine and threonine metabolism
Glutathione metabolism
Cysteine
Cysteine and methionine metabolism
K13034
γ-Glutamyl-β-cyanoalanine
MELLADRAFT_4
L-3-Cyanoalanine
γ-Glutamyl-β-aminopropiononitrile
3-Aminopropiono-nitrile
K13035
β-Alanine metabolism
L-Aspartate
K01914
K01424
L-Asparagine
00460 8/15/17
(c) Kanehisa Laboratories

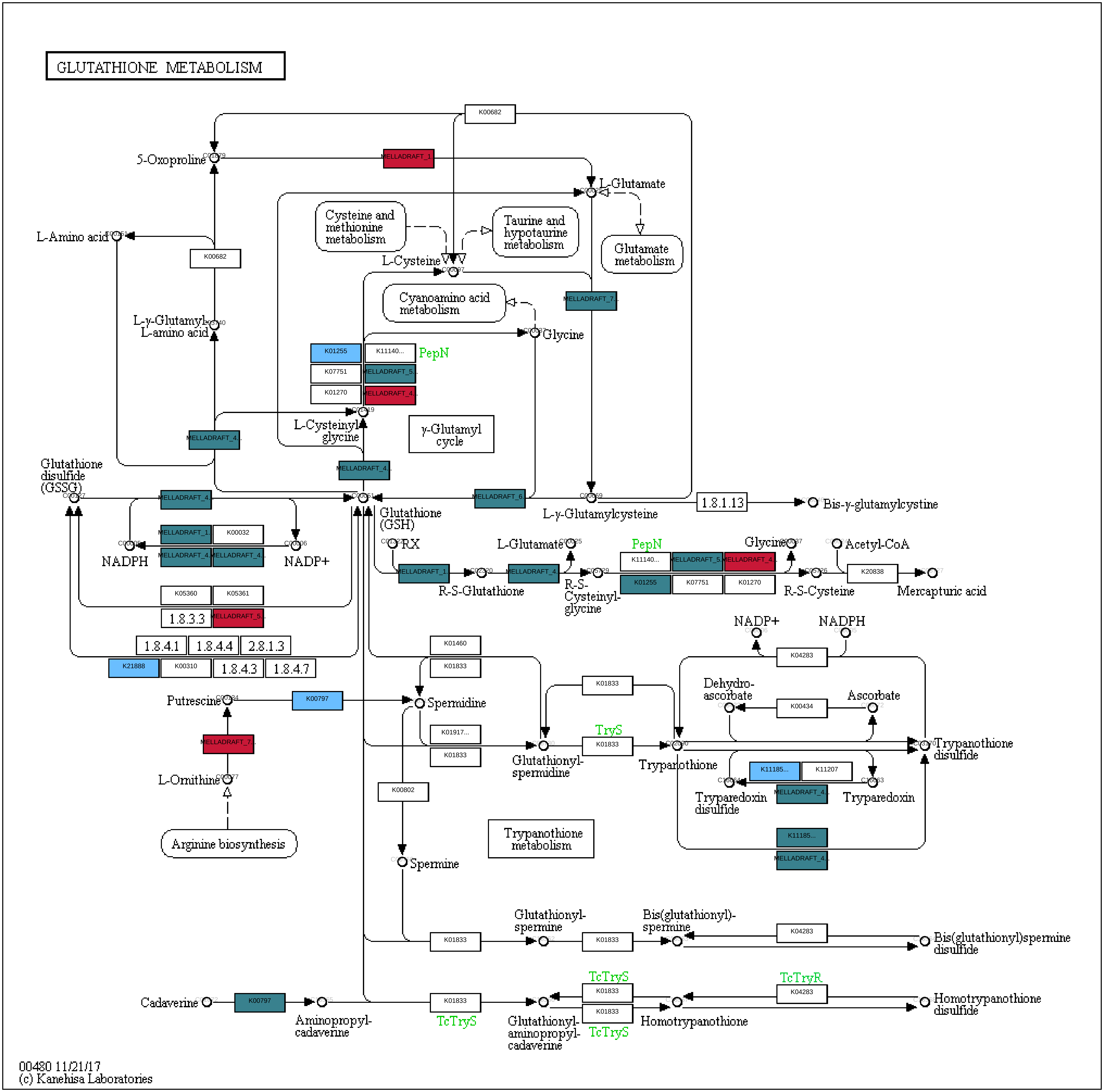
GLUTATHIONE METABOLISM
K00682
5-Oxoproline
MELLADRAFT_1
L-Glutamate
Cysteine and methionine metabolism
Taurine and hypotaurine metabolism
Glutamate metabolism
L-Amino acid
K00682
L-Cysteine
Cyanoamino acid metabolism
MELLADRAFT_7...
L-γ-Glutamyl-L-amino acid
Glycine
K01255
K11140...
PepN
K07751
MELLADRAFT_5...
K01270
MELLADRAFT_4...
MELLADRAFT_4...
L-Cysteinyl-glycine
γ-Glutamyl cycle
MELLADRAFT_4...
Glutathione disulfide (GSSG)
MELLADRAFT_4...
MELLADRAFT_6...
Glutathione (GSH)
L-γ-Glutamylcysteine
1.8.1.13
Bis-γ-glutamylcystine
MELLADRAFT_1...
K00032
MELLADRAFT_4...
MELLADRAFT_4...
NADPH
NADP+
RX
L-Glutamate
PepN
Glycine
Acetyl-CoA
K11140...
MELLADRAFT_5...
MELLADRAFT_4...
MELLADRAFT_1...
MELLADRAFT_4...
K20838
K01255
K07751
K01270
R-S-Glutathione
R-S-Cysteinyl-glycine
R-S-Cysteine
Mercapturic acid
K05360
K05361
1.8.3.3
MELLADRAFT_5...
1.8.4.1
1.8.4.4
2.8.1.3
K21888
K00310
1.8.4.3
1.8.4.7
K01460
K01833
NADP+
NADPH
K04283
Putrescine
K00797
Spermidine
K01833
Dehydro-ascorbate
Ascorbate
K00434
MELLADRAFT_7...
TryS
K01917...
K01833
K01833
Trypanothione disulfide
Glutathionyl-spermidine
Trypanothione
L-Ornithine
K11185...
K11207
K00802
Trypanedoxin disulfide
MELLADRAFT_4...
Tryparedoxin
Arginine biosynthesis
Trypanothione metabolism
K11185...
MELLADRAFT_4...
Spermine
Glutathionyl-spermine
Bis(glutathionyl)-spermine
K04283
K01833
K01833
Bis(glutathionyl)spermine disulfide
TcTryS
TcTryR
K01833
K04283
Cadaverine
K00797
K01833
K01833
Homotrypanothione disulfide
Aminopropyl-cadaverine
TcTryS
Glutathionyl-aminopropyl-cadaverine
TcTryS
Homotrypanothione
00480 11/21/17
(c) Kanehisa Laboratories

# 7. Glycan biosynthesis and metabolism

| MAP | PATHWAY |
|---|---|
| **510** | N-Glycan biosynthesis |
| **513** | Various types of N-glycan biosynthesis |
| **531** | Glycosaminoglycan degradation |
| **563** | Glycosylphosphatidylinositol (GPI)-anchor biosynthesis |

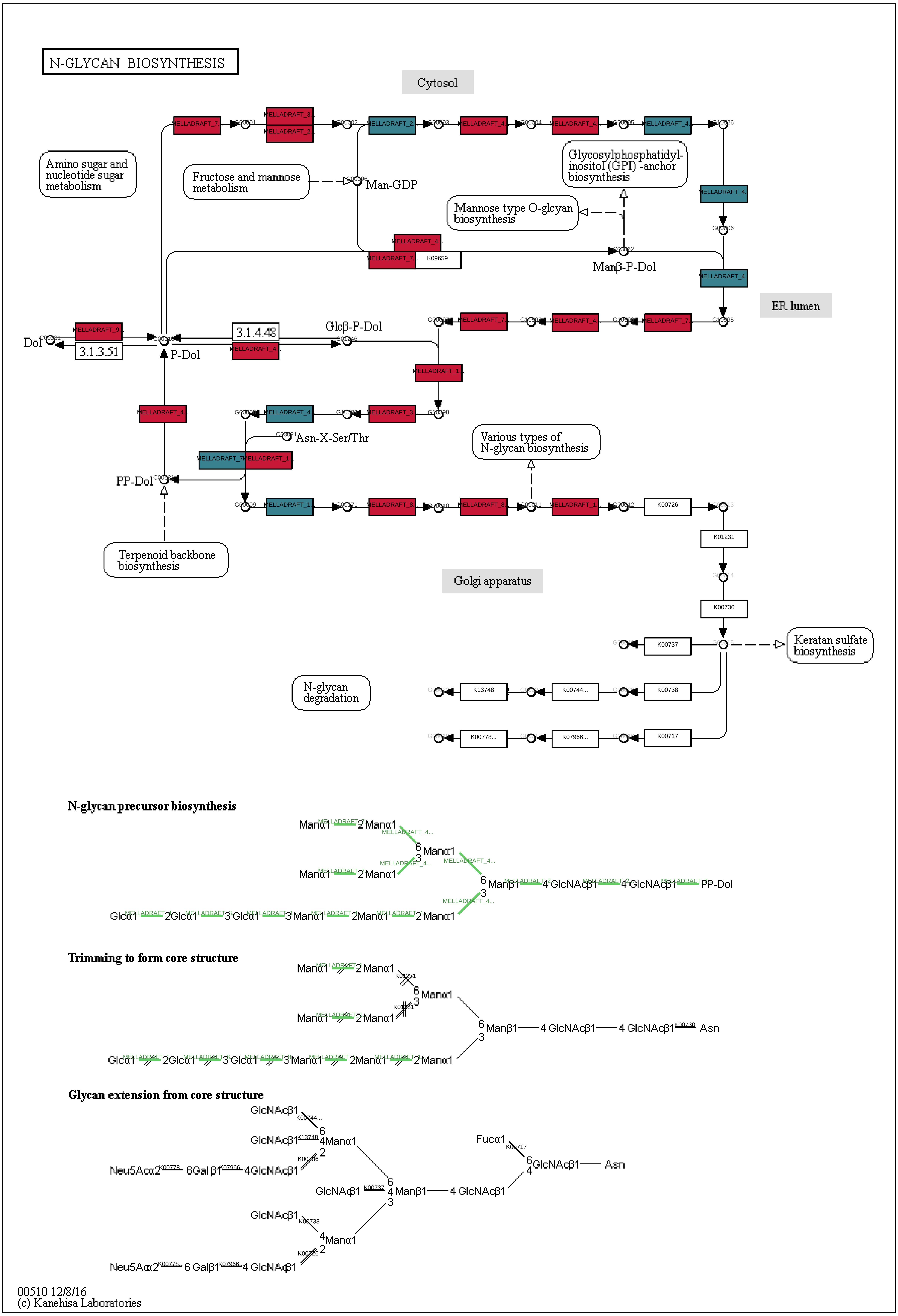
N-GLYCAN BIOSYNTHESIS
Cytosol
ER lumen
Golgi apparatus
Amino sugar and nucleotide sugar metabolism
Fructose and mannose metabolism
Glycosylphosphatidyl-inositol (GPI) -anchor biosynthesis
Mannose type O-glcyan biosynthesis
Various types of N-glycan biosynthesis
Terpenoid backbone biosynthesis
Keratan sulfate biosynthesis
N-glycan degradation
Man-GDP
Manβ-P-Dol
Glcβ-P-Dol
Dol
P-Dol
PP-Dol
Asn-X-Ser/Thr
3.1.4.48
3.1.3.51
K09659
K00726
K01231
K00736
K00737
K13748
K00744...
K00738
K00778...
K07966...
K00717
N-glycan precursor biosynthesis
Trimming to form core structure
Glycan extension from core structure
00510 12/8/16
(c) Kanehisa Laboratories

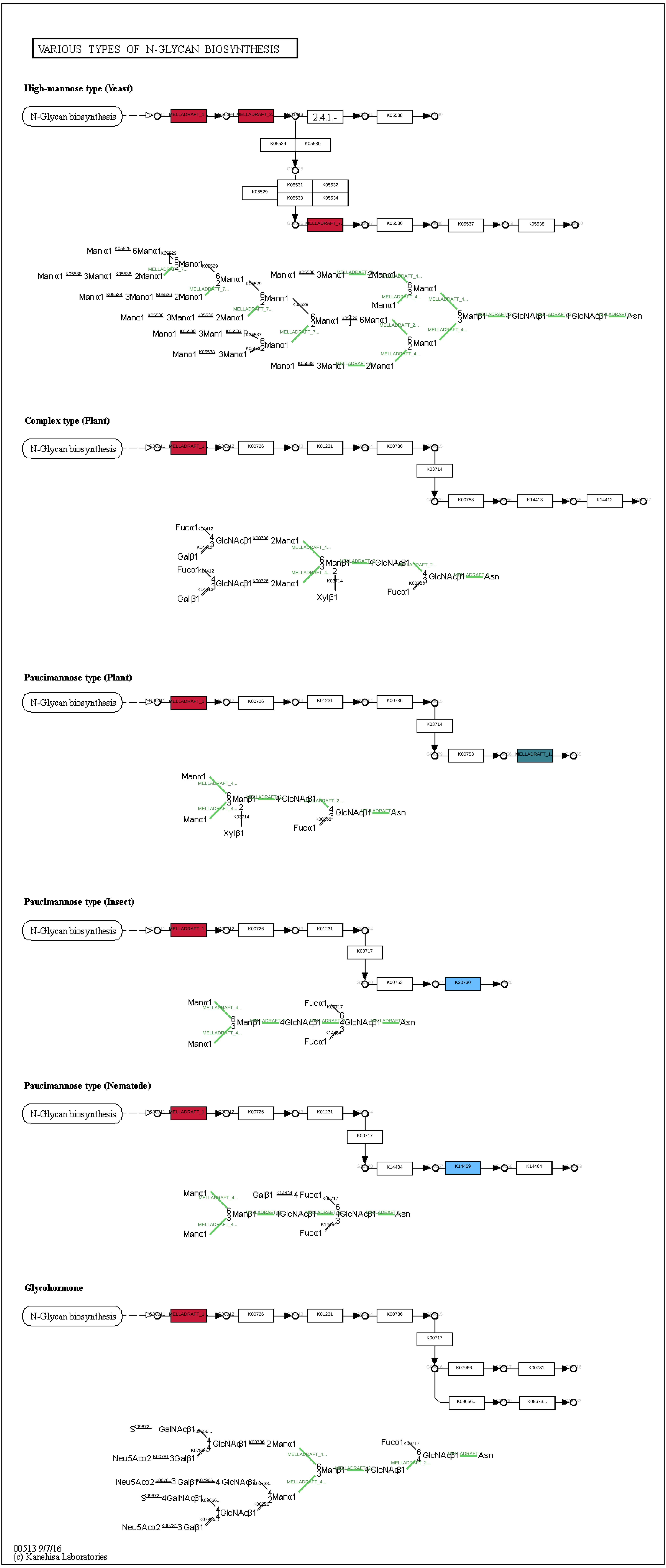

VARIOUS TYPES OF N-GLYCAN BIOSYNTHESIS
High-mannose type (Yeast)
N-Glycan biosynthesis
MELLADRAFT_1
MELLADRAFT_2
2.4.1.-
K05538
K05529
K05530
K05531
K05532
K05533
K05534
MELLADRAFT_7
K05536
K05537
Complex type (Plant)
K00726
K01231
K00736
K03714
K00753
K14413
K14412
Paucimannose type (Plant)
Paucimannose type (Insect)
K00717
K20730
Paucimannose type (Nematode)
K14434
K14459
K14464
Glycohormone
K00781
00513 9/7/16
(c) Kanehisa Laboratories

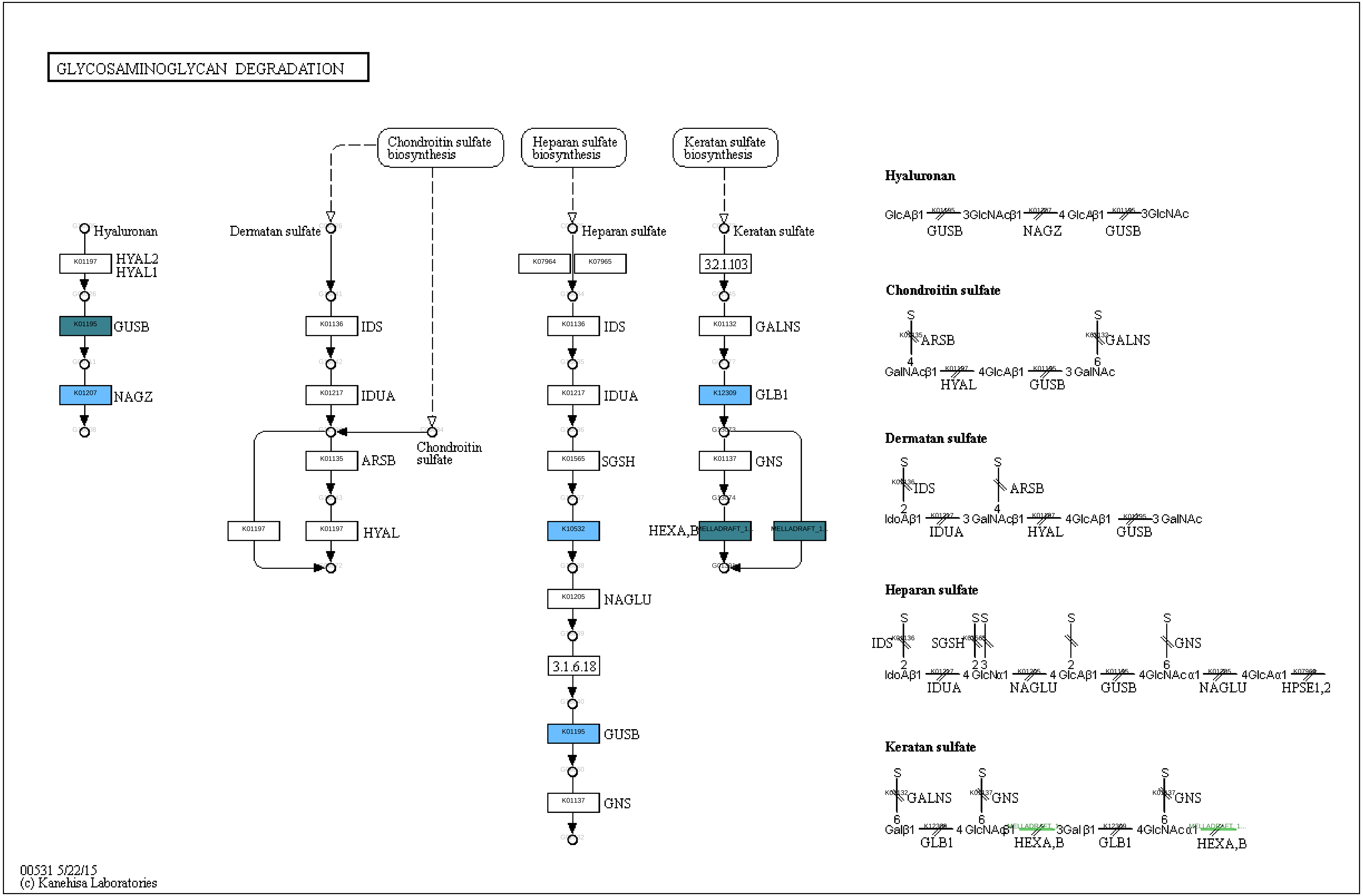
GLYCOSAMINOGLYCAN DEGRADATION
Chondroitin sulfate biosynthesis
Heparan sulfate biosynthesis
Keratan sulfate biosynthesis
Hyaluronan
K01197
HYAL2
HYAL1
K01195
GUSB
K01207
NAGZ
Dermatan sulfate
K01136
IDS
K01217
IDUA
K01135
ARSB
K01197
HYAL
Chondroitin sulfate
Heparan sulfate
K07964
K07965
K01565
SGSH
K10532
K01205
NAGLU
3.1.6.18
K01137
GNS
Keratan sulfate
3.2.1.103
K01132
GALNS
K12309
GLB1
HEXA,B
Hyaluronan
GlcAβ1 3GlcNAcβ1 4 GlcAβ1 3GlcNAc
GUSB NAGZ GUSB
Chondroitin sulfate
GalNAcβ1 4GlcAβ1 3 GalNAc
HYAL GUSB
Dermatan sulfate
IdoAβ1 3 GalNAcβ1 4GlcAβ1 3 GalNAc
IDUA HYAL GUSB
Heparan sulfate
IdoAβ1 4 GlcNα1 4 GlcAβ1 4GlcNAcα1 4GlcAα1
IDUA NAGLU GUSB NAGLU HPSE1,2
Keratan sulfate
Galβ1 4 GlcNAcβ1 3Galβ1 4GlcNAcα1
GLB1 HEXA,B GLB1 HEXA,B
00531 5/22/15
(c) Kanehisa Laboratories

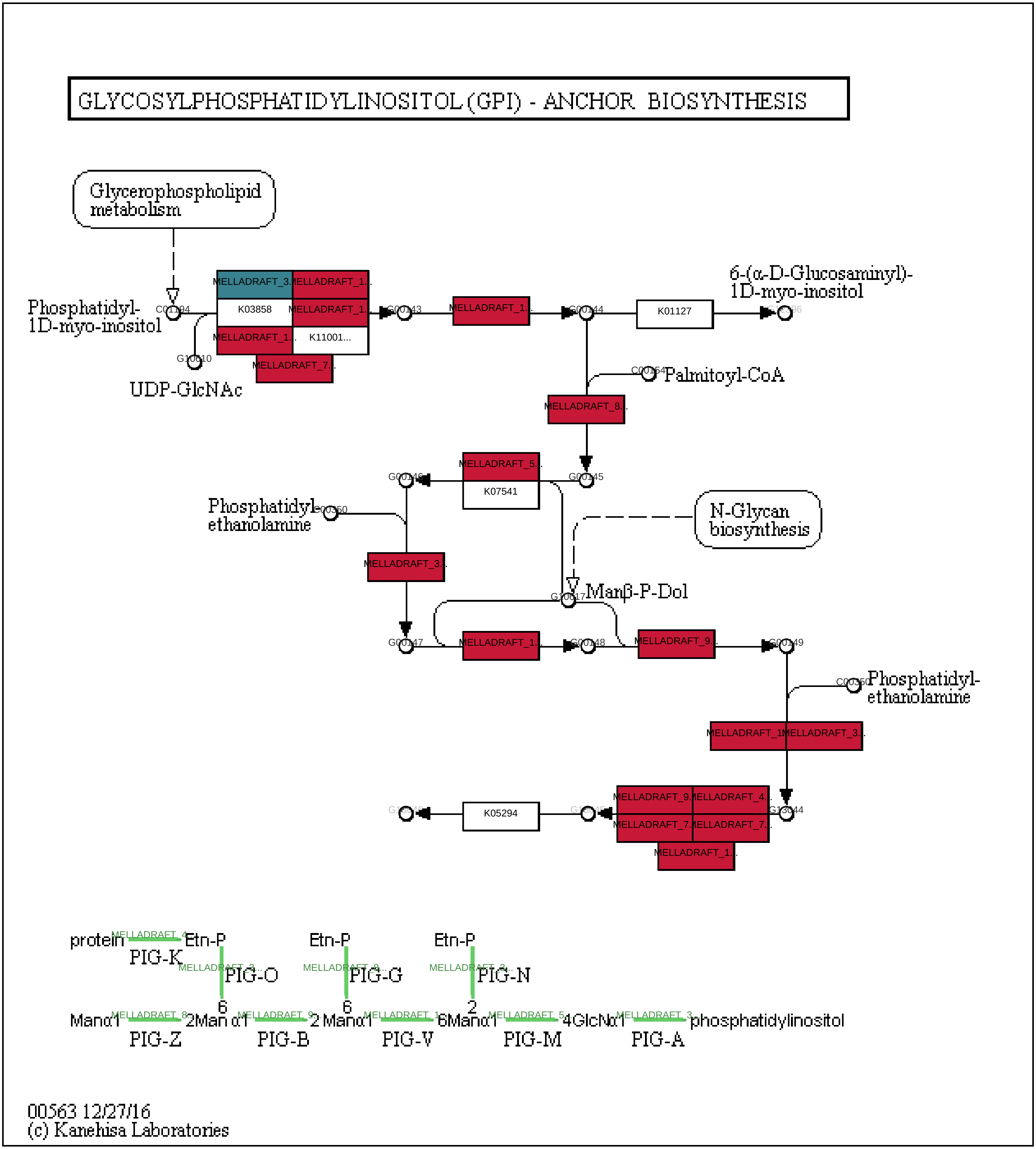
GLYCOSYLPHOSPHATIDYLINOSITOL (GPI) - ANCHOR BIOSYNTHESIS
Glycerophospholipid metabolism
Phosphatidyl-1D-myo-inositol
C01194
G10610
UDP-GlcNAc
K03858
K11001...
G00143
G00144
K01127
6-(α-D-Glucosaminyl)-1D-myo-inositol
C00154
Palmitoyl-CoA
G00145
G00146
K07541
Phosphatidyl-ethanolamine
C00350
N-Glycan biosynthesis
G10617
Manβ-P-Dol
G00147
G00148
G00149
Phosphatidyl-ethanolamine
G13044
K05294
protein
Etn-P
PIG-K
PIG-O
PIG-G
PIG-N
Manα1
2Man α1
2 Manα1
6Manα1
4GlcNα1
phosphatidylinositol
PIG-Z
PIG-B
PIG-V
PIG-M
PIG-A
00563 12/27/16
(c) Kanehisa Laboratories

# 8. Metabolism of cofactors and vitamins

| MAP | PATHWAY |
|---|---|
| **130** | Ubiquinone and other terpenoid-quinone biosynthesis |
| **670** | One carbon pool by folate |
| **730** | Thiamine metabolism |
| **740** | Riboflavin metabolism |
| **750** | Vitamin B6 metabolism |
| **760** | Nicotinate and nicotinamide metabolism |
| **770** | Pantothenate and CoA biosynthesis |
| **780** | Biotin metabolism |
| **785** | Lipoic acid metabolism |
| **790** | Folate biosynthesis |
| **860** | Porphyrin and chlorophyll metabolism |

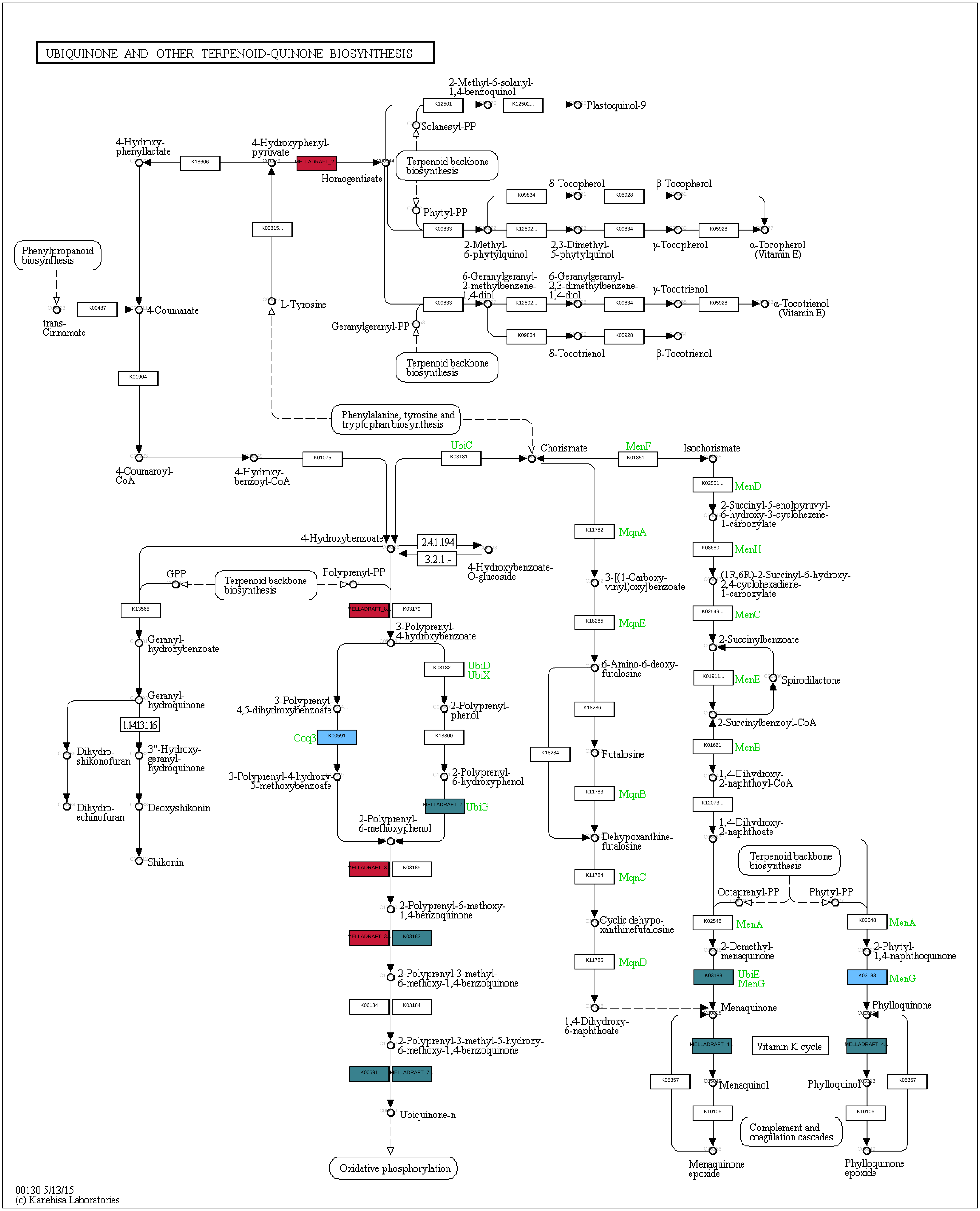
UBIQUINONE AND OTHER TERPENOID-QUINONE BIOSYNTHESIS
2-Methyl-6-solanyl-1,4-benzoquinol
K12501
K12502...
Plastoquinol-9
Solanesyl-PP
4-Hydroxy-phenyllactate
K18606
4-Hydroxyphenyl-pyruvate
MELLADRAFT_2...
Homogentisate
Terpenoid backbone biosynthesis
δ-Tocopherol
β-Tocopherol
K09834
K05928
Phytyl-PP
K09833
K12502...
K09834
K05928
2-Methyl-6-phytylquinol
2,3-Dimethyl-5-phytylquinol
γ-Tocopherol
α-Tocopherol (Vitamin E)
Phenylpropanoid biosynthesis
K00815...
6-Geranylgeranyl-2-methylbenzene-1,4-diol
6-Geranylgeranyl-2,3-dimethylbenzene-1,4-diol
γ-Tocotrienol
K00487
4-Coumarate
L-Tyrosine
K09833
K12502...
K09834
K05928
α-Tocotrienol (Vitamin E)
trans-Cinnamate
Geranylgeranyl-PP
K09834
K05928
δ-Tocotrienol
β-Tocotrienol
Terpenoid backbone biosynthesis
K01904
Phenylalanine, tyrosine and tryptophan biosynthesis
UbiC
MenF
Chorismate
Isochorismate
K01075
K03181...
K01851...
4-Coumaroyl-CoA
4-Hydroxy-benzoyl-CoA
K02551...
MenD
2-Succinyl-5-enolpyruvyl-6-hydroxy-3-cyclohexene-1-carboxylate
K11782
MqnA
4-Hydroxybenzoate
2.4.1.194
3.2.1.-
K08680...
MenH
4-Hydroxybenzoate-O-glucoside
GPP
Terpenoid backbone biosynthesis
Polyprenyl-PP
3-[(1-Carboxy-vinyl)oxy]benzoate
(1R,6R)-2-Succinyl-6-hydroxy-2,4-cyclohexadiene-1-carboxylate
K13565
MELLADRAFT_8...
K03179
K18285
MqnE
K02549...
MenC
Geranyl-hydroxybenzoate
3-Polyprenyl-4-hydroxybenzoate
2-Succinylbenzoate
K03182...
UbiD
UbiX
6-Amino-6-deoxy-futalosine
K01911...
MenE
Spirodilactone
Geranyl-hydroquinone
3-Polyprenyl-4,5-dihydroxybenzoate
2-Polyprenyl-phenol
K18286...
1.14.13.116
2-Succinylbenzoyl-CoA
Coq3
K00591
K18800
K18284
K01661
MenB
Dihydro-shikonofuran
3''-Hydroxy-geranyl-hydroquinone
3-Polyprenyl-4-hydroxy-5-methoxybenzoate
2-Polyprenyl-6-hydroxyphenol
Futalosine
1,4-Dihydroxy-2-naphthoyl-CoA
K11783
MqnB
Dihydro-echinofuran
Deoxyshikonin
MELLADRAFT_7...
UbiG
K12073...
1,4-Dihydroxy-2-naphthoate
2-Polyprenyl-6-methoxyphenol
Dehypoxanthine-futalosine
Shikonin
Terpenoid backbone biosynthesis
MELLADRAFT_3...
K03185
K11784
MqnC
Octaprenyl-PP
Phytyl-PP
2-Polyprenyl-6-methoxy-1,4-benzoquinone
Cyclic dehypo-xanthinefutalosine
K02548
MenA
K02548
MenA
MELLADRAFT_3...
K03183
2-Demethyl-menaquinone
2-Phytyl-1,4-naphthoquinone
2-Polyprenyl-3-methyl-6-methoxy-1,4-benzoquinone
K11785
MqnD
K03183
UbiE
MenG
K03183
MenG
K06134
K03184
Menaquinone
Phylloquinone
1,4-Dihydroxy-6-naphthoate
2-Polyprenyl-3-methyl-5-hydroxy-6-methoxy-1,4-benzoquinone
MELLADRAFT_4...
Vitamin K cycle
MELLADRAFT_4...
K00591
MELLADRAFT_7...
K05357
Menaquinol
Phylloquinol
K05357
Ubiquinone-n
K10106
K10106
Complement and coagulation cascades
Oxidative phosphorylation
Menaquinone epoxide
Phylloquinone epoxide
00130 5/13/15
(c) Kanehisa Laboratories

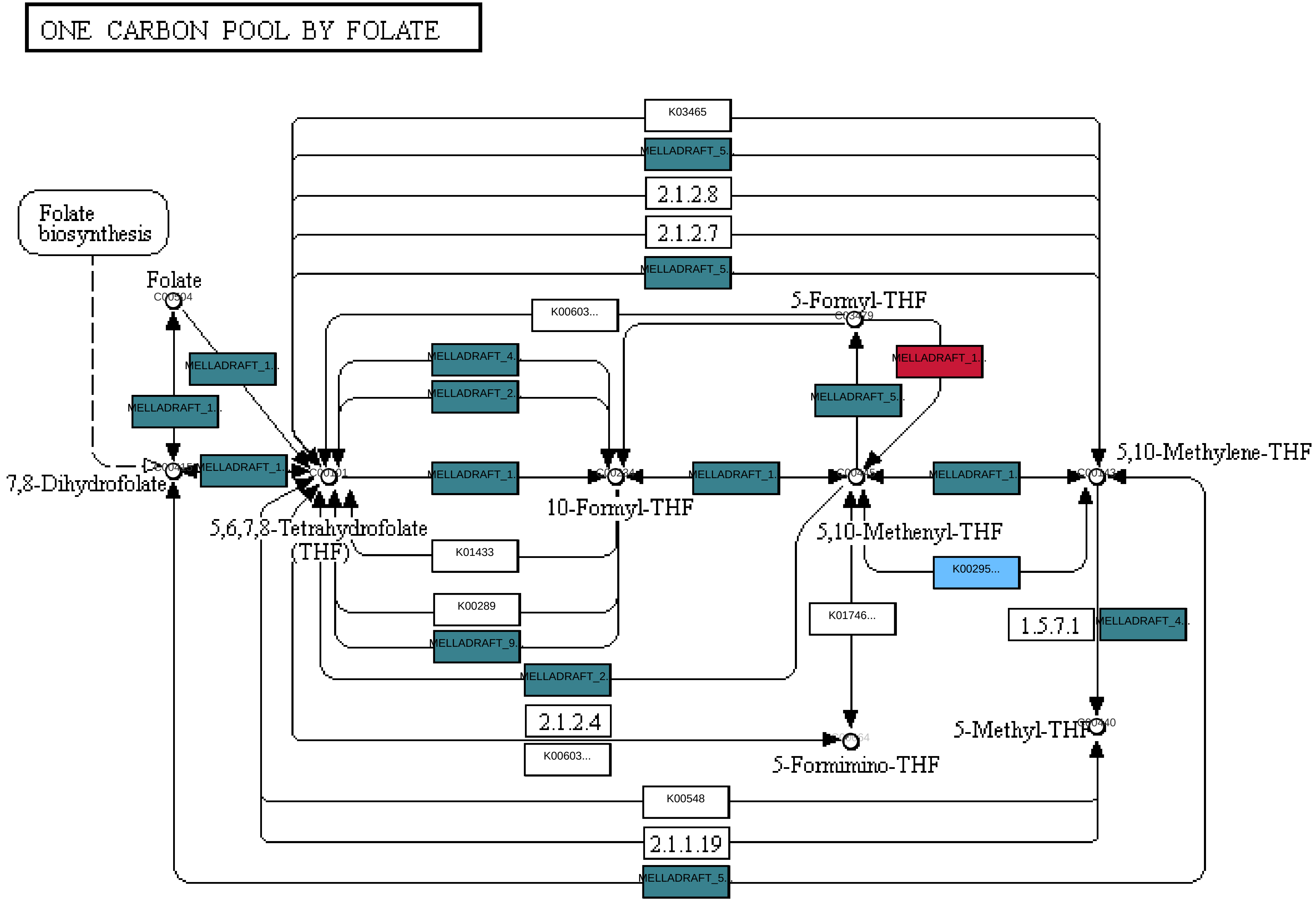


00670 10/7/14
(c) Kanehisa Laboratories

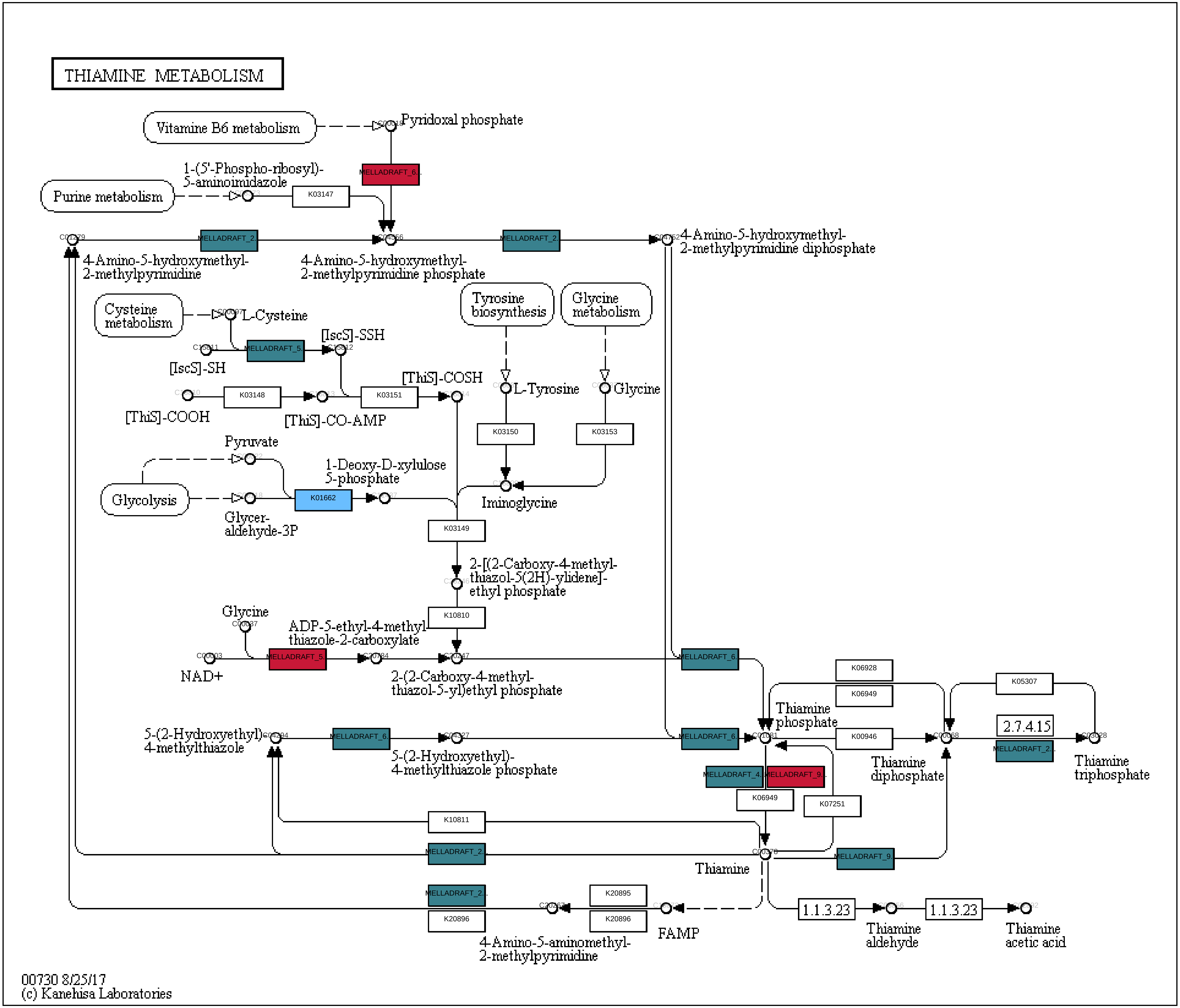

THIAMINE METABOLISM
Vitamine B6 metabolism
Pyridoxal phosphate
MELLADRAFT_6..
Purine metabolism
1-(5'-Phospho-ribosyl)-
5-aminoimidazole
K03147
C01279
MELLADRAFT_2
C04556
MELLADRAFT_2..
C04752
4-Amino-5-hydroxymethyl-
2-methylpyrimidine diphosphate
4-Amino-5-hydroxymethyl-
2-methylpyrimidine
4-Amino-5-hydroxymethyl-
2-methylpyrimidine phosphate
Cysteine
metabolism
C00097
L-Cysteine
Tyrosine
biosynthesis
Glycine
metabolism
[IscS]-SSH
MELLADRAFT_5..
[IscS]-SH
[ThiS]-COSH
K03148
K03151
L-Tyrosine
Glycine
[ThiS]-COOH
[ThiS]-CO-AMP
K03150
K03153
Pyruvate
1-Deoxy-D-xylulose
5-phosphate
Glycolysis
K01662
Iminoglycine
Glycer-
aldehyde-3P
K03149
2-[(2-Carboxy-4-methyl-
thiazol-5(2H)-ylidene]-
ethyl phosphate
Glycine
K10810
ADP-5-ethyl-4-methyl
thiazole-2-carboxylate
C00003
MELLADRAFT_5..
C20784
C20247
MELLADRAFT_6..
NAD+
2-(2-Carboxy-4-methyl-
thiazol-5-yl)ethyl phosphate
K06928
K05307
K06949
Thiamine
phosphate
5-(2-Hydroxyethyl)-
4-methylthiazole
C04294
MELLADRAFT_6..
C04327
MELLADRAFT_6..
C01081
K00946
C00068
2.7.4.15
C03028
MELLADRAFT_2..
5-(2-Hydroxyethyl)-
4-methylthiazole phosphate
Thiamine
diphosphate
Thiamine
triphosphate
MELLADRAFT_4..
MELLADRAFT_9..
K06949
K07251
K10811
MELLADRAFT_2..
C00378
MELLADRAFT_9..
Thiamine
MELLADRAFT_2..
K20895
C20267
K20896
K20896
FAMP
1.1.3.23
1.1.3.23
Thiamine
aldehyde
Thiamine
acetic acid
4-Amino-5-aminomethyl-
2-methylpyrimidine
00730 8/25/17
(c) Kanehisa Laboratories

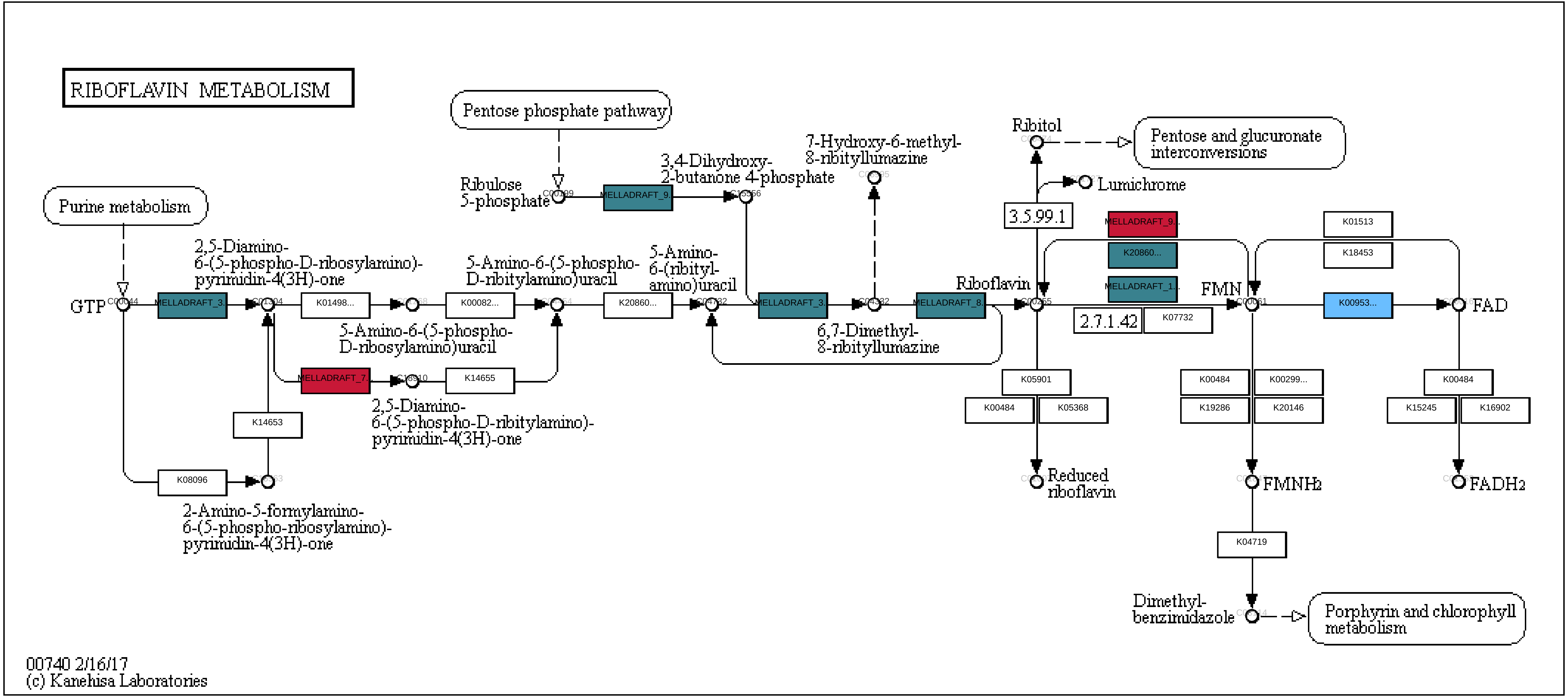
RIBOFLAVIN METABOLISM
Pentose phosphate pathway
Purine metabolism
Ribulose 5-phosphate
C00199
MELLADRAFT_9...
3,4-Dihydroxy-2-butanone 4-phosphate
7-Hydroxy-6-methyl-8-ribityllumazine
Ribitol
Pentose and glucuronate interconversions
Lumichrome
3.5.99.1
2,5-Diamino-6-(5-phospho-D-ribosylamino)-pyrimidin-4(3H)-one
5-Amino-6-(5-phospho-D-ribitylamino)uracil
5-Amino-6-(ribitylamino)uracil
GTP
C00044
MELLADRAFT_3...
C01304
K01498...
K00082...
K20860...
C04732
MELLADRAFT_3...
C04332
MELLADRAFT_8...
Riboflavin
C00255
MELLADRAFT_9...
K20860...
MELLADRAFT_1...
2.7.1.42
K07732
FMN
C00061
K01513
K18453
K00953...
FAD
5-Amino-6-(5-phospho-D-ribosylamino)uracil
6,7-Dimethyl-8-ribityllumazine
MELLADRAFT_7...
K14655
2,5-Diamino-6-(5-phospho-D-ribitylamino)-pyrimidin-4(3H)-one
K14653
K08096
2-Amino-5-formylamino-6-(5-phospho-ribosylamino)-pyrimidin-4(3H)-one
K05901
K00484
K05368
Reduced riboflavin
K00484
K00299...
K19286
K20146
FMNH2
K04719
Dimethyl-benzimidazole
Porphyrin and chlorophyll metabolism
K00484
K15245
K16902
FADH2
00740 2/16/17
(c) Kanehisa Laboratories

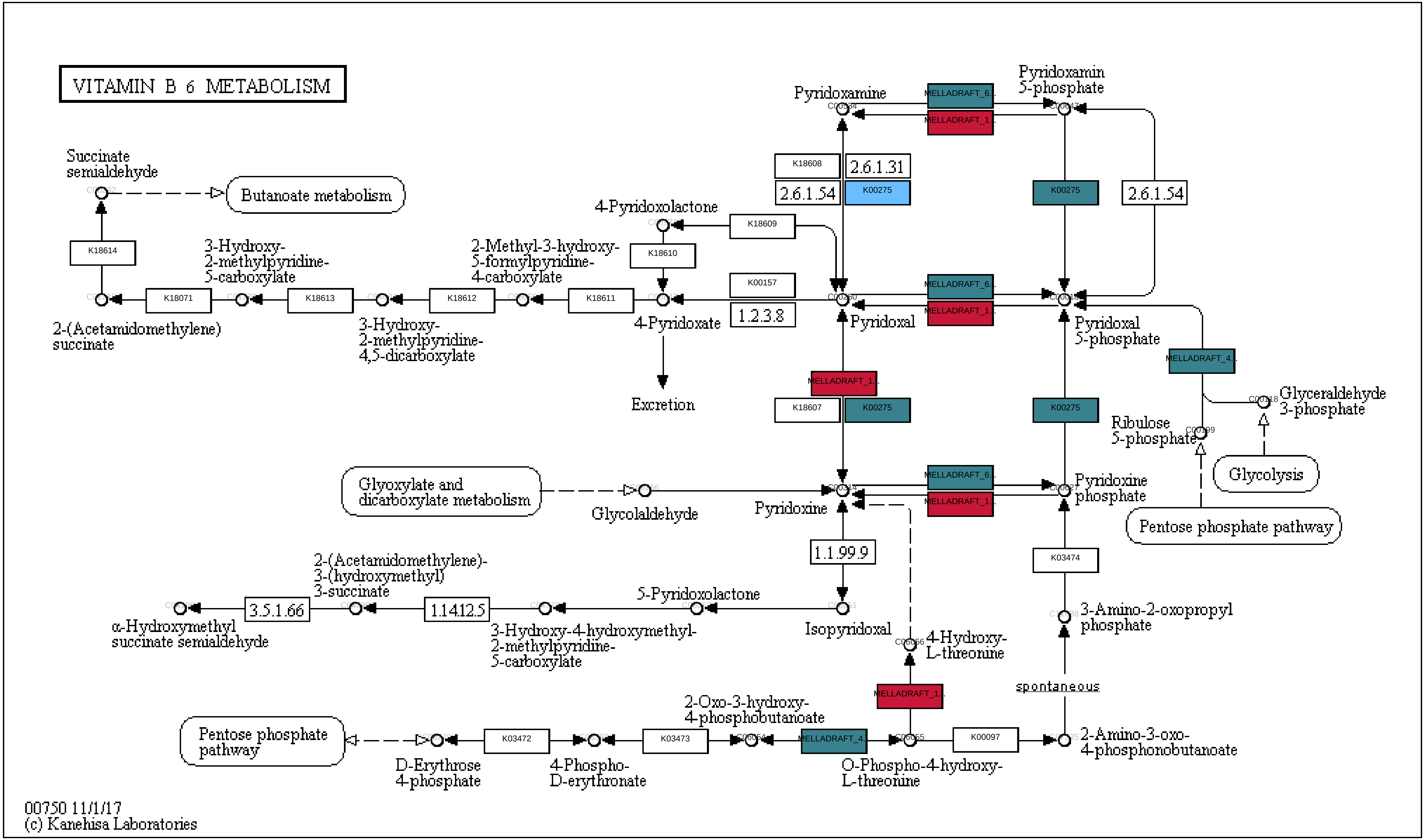
VITAMIN B 6 METABOLISM
Succinate semialdehyde
Butanoate metabolism
K18614
3-Hydroxy-2-methylpyridine-5-carboxylate
2-Methyl-3-hydroxy-5-formylpyridine-4-carboxylate
4-Pyridoxolactone
K18609
K18610
K18071
K18613
K18612
K18611
K00157
1.2.3.8
2-(Acetamidomethylene) succinate
3-Hydroxy-2-methylpyridine-4,5-dicarboxylate
4-Pyridoxate
Excretion
Pyridoxamine
Pyridoxamin 5-phosphate
MELLADRAFT_6..
MELLADRAFT_1..
K18608
2.6.1.31
2.6.1.54
K00275
2.6.1.54
Pyridoxal
Pyridoxal 5-phosphate
MELLADRAFT_4..
Glyceraldehyde 3-phosphate
Ribulose 5-phosphate
Glycolysis
K18607
Glyoxylate and dicarboxylate metabolism
Glycolaldehyde
Pyridoxine
Pyridoxine phosphate
Pentose phosphate pathway
1.1.99.9
K03474
2-(Acetamidomethylene)-3-(hydroxymethyl)3-succinate
5-Pyridoxolactone
3.5.1.66
1.14.12.5
α-Hydroxymethyl succinate semialdehyde
3-Hydroxy-4-hydroxymethyl-2-methylpyridine-5-carboxylate
Isopyridoxal
4-Hydroxy-L-threonine
3-Amino-2-oxopropyl phosphate
spontaneous
2-Oxo-3-hydroxy-4-phosphobutanoate
Pentose phosphate pathway
K03472
K03473
K00097
D-Erythrose 4-phosphate
4-Phospho-D-erythronate
O-Phospho-4-hydroxy-L-threonine
2-Amino-3-oxo-4-phosphonobutanoate
00750 11/1/17
(c) Kanehisa Laboratories

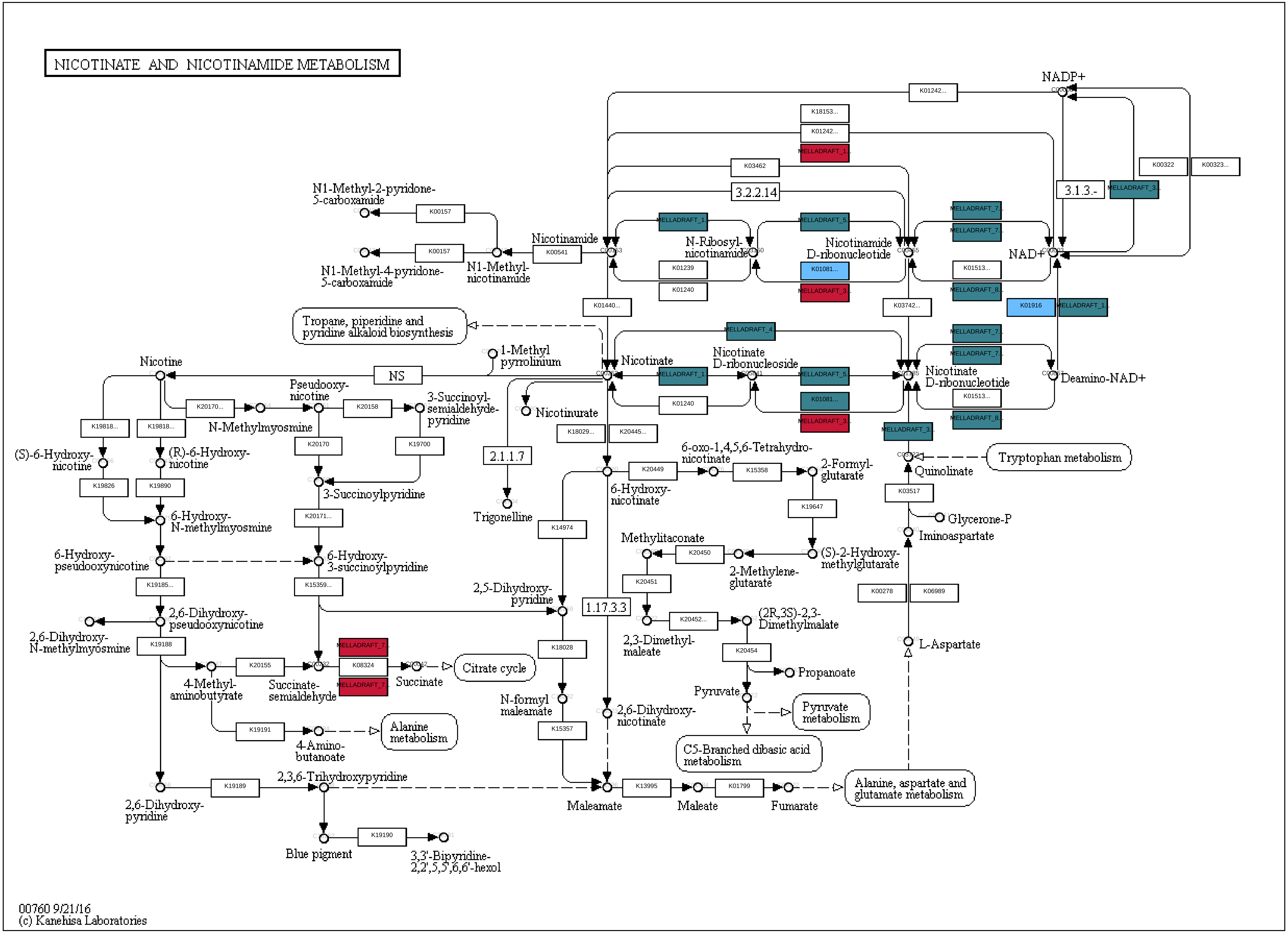
NICOTINATE AND NICOTINAMIDE METABOLISM
NADP+
K01242...
K18153...
K01242...
MELLADRAFT_1...
K03462
3.2.2.14
3.1.3.-
MELLADRAFT_3...
K00322
K00323...
N1-Methyl-2-pyridone-5-carboxamide
K00157
K00157
N1-Methyl-4-pyridone-5-carboxamide
N1-Methyl-nicotinamide
K00541
Nicotinamide
MELLADRAFT_1...
N-Ribosyl-nicotinamide
K01239
K01240
MELLADRAFT_5...
Nicotinamide D-ribonucleotide
K01081...
MELLADRAFT_3...
MELLADRAFT_7...
MELLADRAFT_7...
NAD+
K01513...
MELLADRAFT_8...
K01916
MELLADRAFT_1...
K01440...
K03742...
Tropane, piperidine and pyridine alkaloid biosynthesis
MELLADRAFT_4...
Nicotine
NS
1-Methyl pyrrolinium
Nicotinate
MELLADRAFT_1...
Nicotinate D-ribonucleoside
MELLADRAFT_5...
MELLADRAFT_7...
MELLADRAFT_7...
Nicotinate D-ribonucleotide
Deamino-NAD+
K01240
K01081...
K01513...
MELLADRAFT_3...
MELLADRAFT_8...
MELLADRAFT_3...
Pseudooxy-nicotine
K20170...
K20158
3-Succinoyl-semialdehyde-pyridine
N-Methylmyosmine
K19818...
K19818...
K20170
K19700
Nicotinurate
K18029...
K20445...
2.1.1.7
(S)-6-Hydroxy-nicotine
(R)-6-Hydroxy-nicotine
K19826
K19890
3-Succinoylpyridine
Trigonelline
6-oxo-1,4,5,6-Tetrahydro-nicotinate
K20449
K15358
2-Formyl-glutarate
6-Hydroxy-nicotinate
Tryptophan metabolism
Quinolinate
K03517
Glycerone-P
6-Hydroxy-N-methylmyosmine
K20171...
K14974
K19647
Iminoaspartate
6-Hydroxy-pseudooxynicotine
6-Hydroxy-3-succinoylpyridine
Methylitaconate
K20450
(S)-2-Hydroxy-methylglutarate
2-Methylene-glutarate
K19185...
K15359...
K20451
K00278
K06989
2,5-Dihydroxy-pyridine
1.17.3.3
2,6-Dihydroxy-pseudooxynicotine
K20452...
(2R,3S)-2,3-Dimethylmalate
2,6-Dihydroxy-N-methylmyosmine
K19188
MELLADRAFT_7...
K18028
2,3-Dimethyl-maleate
L-Aspartate
K20454
K20155
K08324
MELLADRAFT_7...
Citrate cycle
Propanoate
4-Methyl-aminobutyrate
Succinate-semialdehyde
Succinate
Pyruvate
N-formyl maleamate
2,6-Dihydroxy-nicotinate
Pyruvate metabolism
K19191
K15357
Alanine metabolism
4-Amino-butanoate
C5-Branched dibasic acid metabolism
2,3,6-Trihydroxypyridine
K19189
K13995
K01799
Alanine, aspartate and glutamate metabolism
2,6-Dihydroxy-pyridine
Maleamate
Maleate
Fumarate
K19190
Blue pigment
3,3'-Bipyridine-2,2',5,5',6,6'-hexol
00760 9/21/16
(c) Kanehisa Laboratories

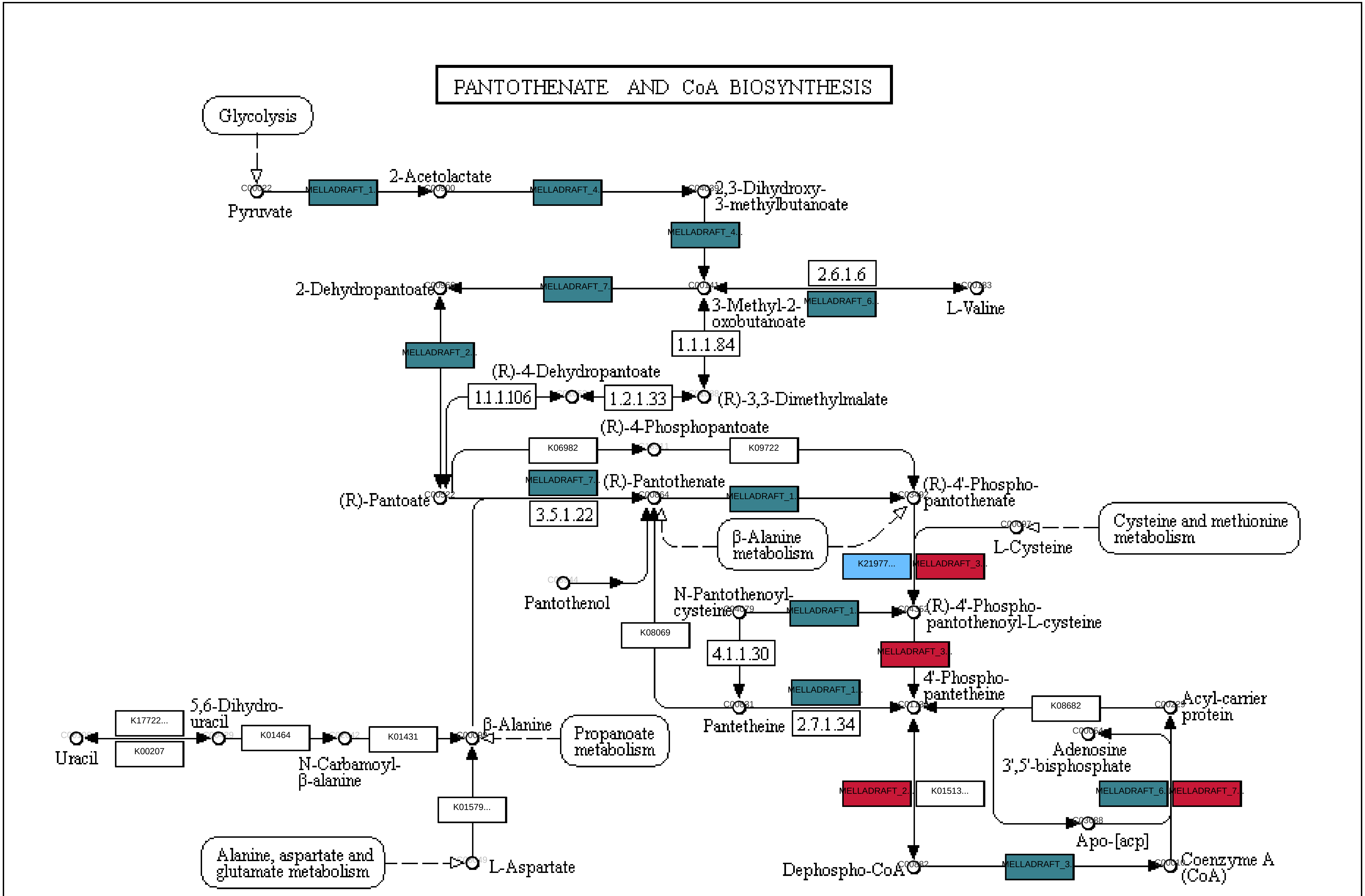


00770 7/27/17
(c) Kanehisa Laboratories

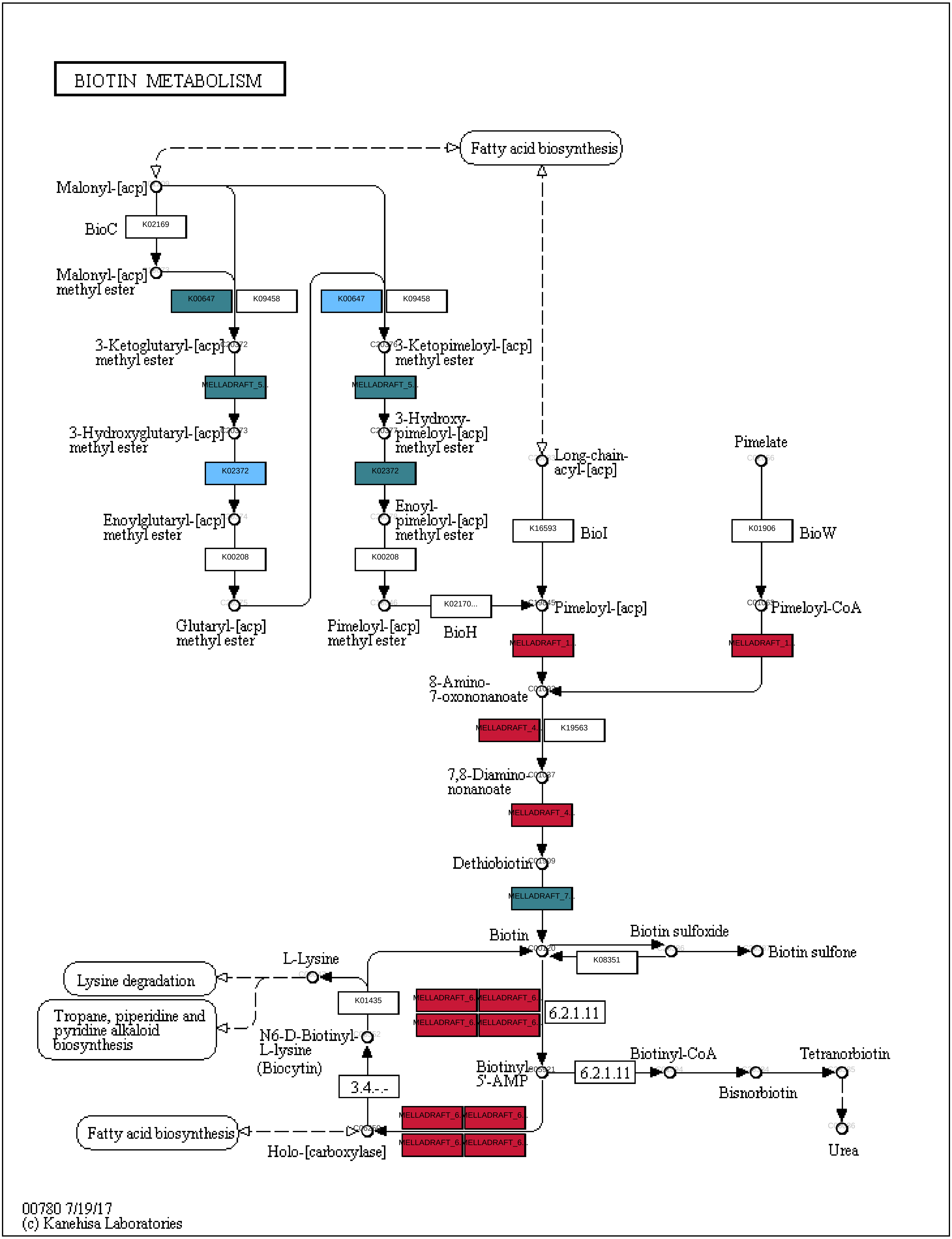
BIOTIN METABOLISM
Fatty acid biosynthesis
Malonyl-[acp]
BioC
K02169
Malonyl-[acp] methyl ester
K00647
K09458
K00647
K09458
3-Ketoglutaryl-[acp] methyl ester
3-Ketopimeloyl-[acp] methyl ester
MELLADRAFT_5...
MELLADRAFT_5...
3-Hydroxyglutaryl-[acp] methyl ester
3-Hydroxy-pimeloyl-[acp] methyl ester
K02372
K02372
Enoylglutaryl-[acp] methyl ester
Enoyl-pimeloyl-[acp] methyl ester
K00208
K00208
Long-chain-acyl-[acp]
Pimelate
K16593
BioI
K01906
BioW
Glutaryl-[acp] methyl ester
Pimeloyl-[acp] methyl ester
K02170...
BioH
Pimeloyl-[acp]
Pimeloyl-CoA
MELLADRAFT_1...
MELLADRAFT_1...
8-Amino-7-oxononanoate
MELLADRAFT_4...
K19563
7,8-Diamino-nonanoate
MELLADRAFT_4...
Dethiobiotin
MELLADRAFT_7...
Biotin
Biotin sulfoxide
Biotin sulfone
K08351
L-Lysine
Lysine degradation
Tropane, piperidine and pyridine alkaloid biosynthesis
K01435
MELLADRAFT_6...
6.2.1.11
N6-D-Biotinyl-L-lysine (Biocytin)
3.4.-.-
Biotinyl-5'-AMP
6.2.1.11
Biotinyl-CoA
Tetranorbiotin
Bisnorbiotin
Urea
Fatty acid biosynthesis
Holo-[carboxylase]
00780 7/19/17
(c) Kanehisa Laboratories

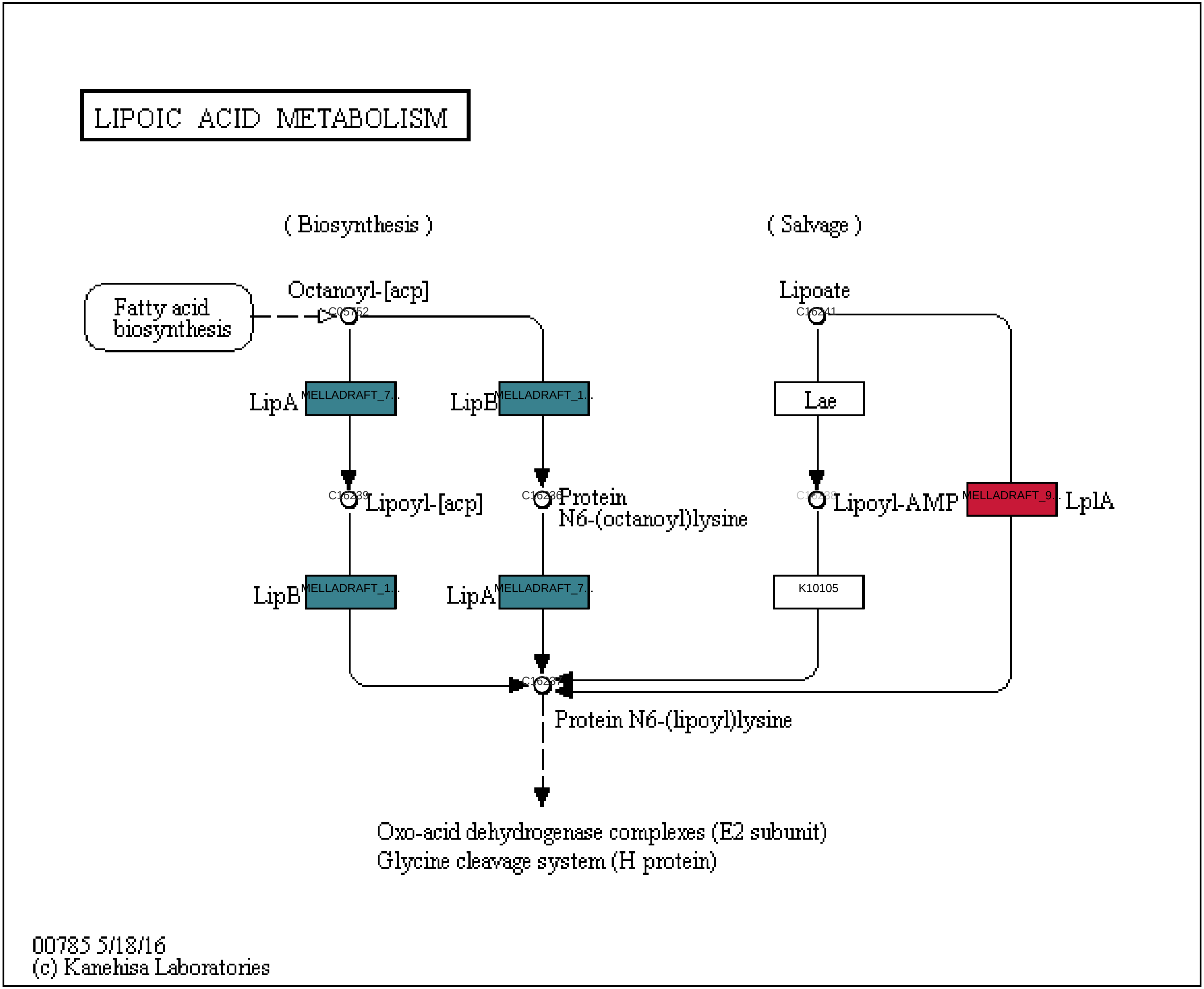
LIPOIC ACID METABOLISM
( Biosynthesis )
( Salvage )
Fatty acid biosynthesis
Octanoyl-[acp]
C05752
Lipoate
C16241
LipA
MELLADRAFT_7...
LipB
MELLADRAFT_1...
Lae
C16239
Lipoyl-[acp]
C16236
Protein N6-(octanoyl)lysine
Lipoyl-AMP
MELLADRAFT_9...
LplA
LipB
MELLADRAFT_1...
LipA
MELLADRAFT_7...
K10105
C16237
Protein N6-(lipoyl)lysine
Oxo-acid dehydrogenase complexes (E2 subunit)
Glycine cleavage system (H protein)
00785 5/18/16
(c) Kanehisa Laboratories

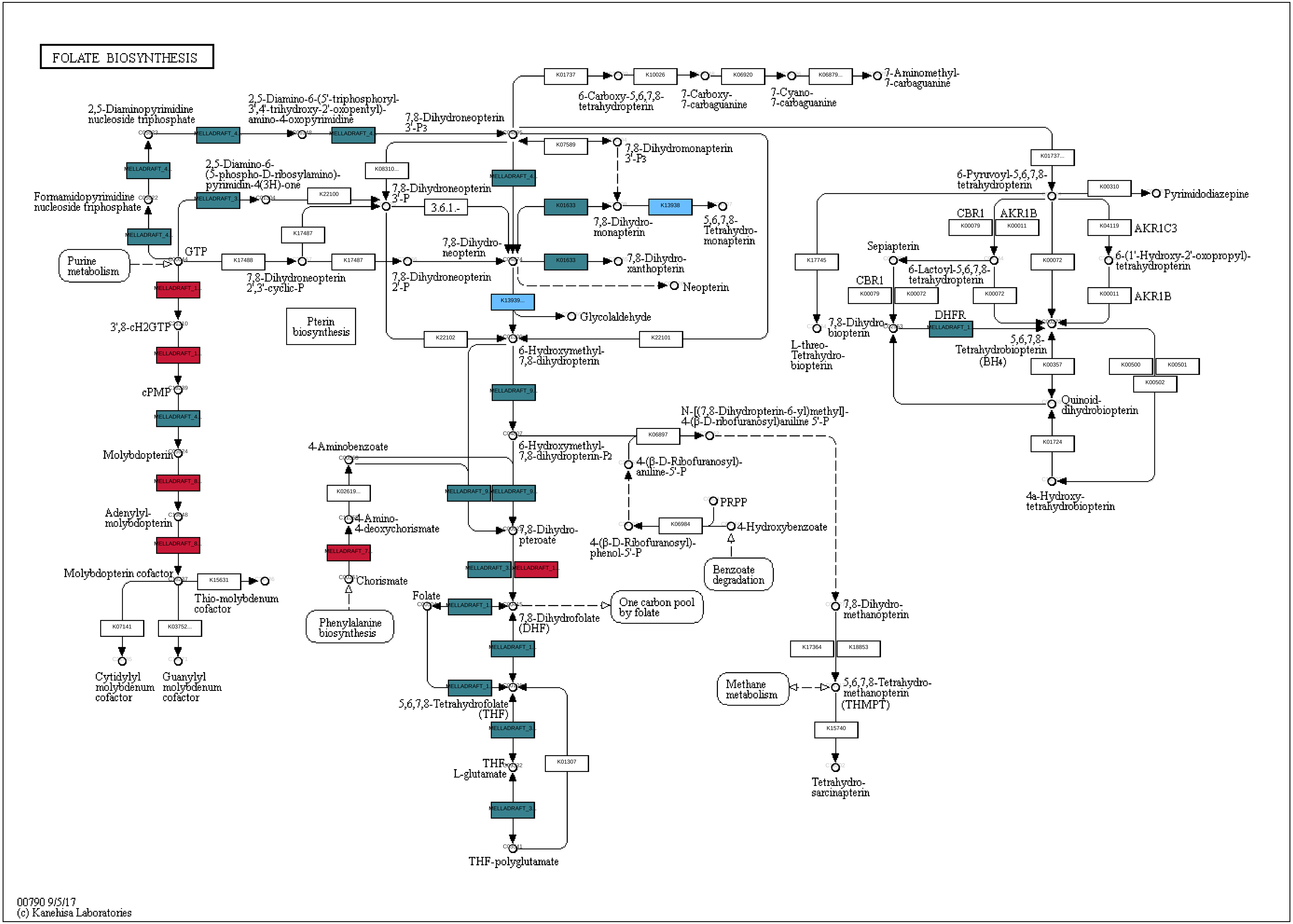
FOLATE BIOSYNTHESIS
2,5-Diaminopyrimidine nucleoside triphosphate
2,5-Diamino-6-(5'-triphosphoryl-3',4'-trihydroxy-2'-oxopentyl)-amino-4-oxopyrimidine
7,8-Dihydroneopterin 3'-P3
Formamidopyrimidine nucleoside triphosphate
2,5-Diamino-6-(5-phospho-D-ribosylamino)-pyrimidin-4(3H)-one
7,8-Dihydroneopterin 3'-P
3.6.1.-
Purine metabolism
GTP
7,8-Dihydroneopterin 2',3'-cyclic-P
7,8-Dihydroneopterin 2'-P
7,8-Dihydro-neopterin
Pterin biosynthesis
3',8-cH2GTP
cPMP
Molybdopterin
Adenylyl-molybdopterin
Molybdopterin cofactor
Thio-molybdenum cofactor
Cytidylyl molybdenum cofactor
Guanylyl molybdenum cofactor
7,8-Dihydromonapterin 3'-P3
7,8-Dihydro-monapterin
5,6,7,8-Tetrahydro-monapterin
7,8-Dihydro-xanthopterin
Neopterin
Glycolaldehyde
6-Hydroxymethyl-7,8-dihydropterin
6-Hydroxymethyl-7,8-dihydropterin-P2
4-Aminobenzoate
4-Amino-4-deoxychorismate
Chorismate
Phenylalanine biosynthesis
7,8-Dihydro-pteroate
Folate
7,8-Dihydrofolate (DHF)
One carbon pool by folate
5,6,7,8-Tetrahydrofolate (THF)
THF-L-glutamate
THF-polyglutamate
6-Carboxy-5,6,7,8-tetrahydropterin
7-Carboxy-7-carbaguanine
7-Cyano-7-carbaguanine
7-Aminomethyl-7-carbaguanine
6-Pyruvoyl-5,6,7,8-tetrahydropterin
Pyrimidodiazepine
CBR1
AKR1B
AKR1C3
Sepiapterin
6-Lactoyl-5,6,7,8-tetrahydropterin
6-(1'-Hydroxy-2'-oxopropyl)-tetrahydropterin
AKR1B
CBR1
DHFR
7,8-Dihydro-biopterin
L-threo-Tetrahydro-biopterin
5,6,7,8-Tetrahydrobiopterin (BH4)
Quinoid-dihydrobiopterin
4a-Hydroxy-tetrahydrobiopterin
N-[(7,8-Dihydropterin-6-yl)methyl]-4-(β-D-ribofuranosyl)aniline 5'-P
4-(β-D-Ribofuranosyl)-aniline-5'-P
PRPP
4-Hydroxybenzoate
4-(β-D-Ribofuranosyl)-phenol-5'-P
Benzoate degradation
7,8-Dihydro-methanopterin
Methane metabolism
5,6,7,8-Tetrahydro-methanopterin (THMPT)
Tetrahydro-sarcinapterin
K01737
K10026
K06920
K06879...
K07589
K13938
K08310...
K22100
K17487
K17488
K01633
K13939...
K22102
K22101
K15631
K07141
K03752...
K02619...
K06897
K06984
K01307
K01737...
K00310
K00079
K00011
K04119
K17745
K00072
K00357
K00500
K00501
K00502
K01724
K17364
K18853
K15740
00790 9/5/17
(c) Kanehisa Laboratories

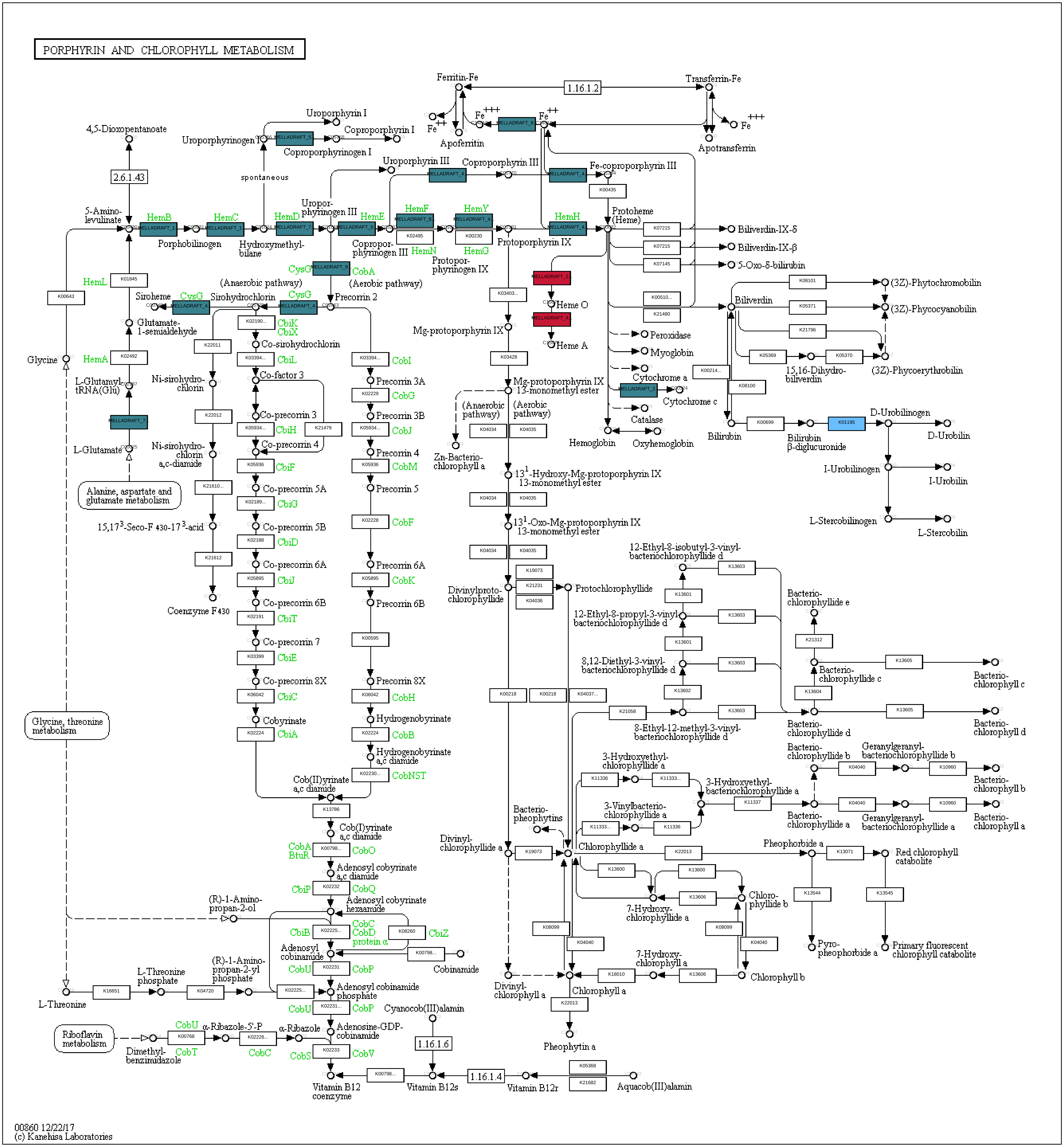
PORPHYRIN AND CHLOROPHYLL METABOLISM
Ferritin-Fe
1.16.1.2
Transferrin-Fe
Fe++
Fe+++
Fe++
Fe+++
Apoferritin
Apotransferrin
4,5-Dioxopentanoate
2.6.1.43
Uroporphyrin I
Uroporphyrinogen I
Coproporphyrin I
Coproporphyrinogen I
spontaneous
Uroporphyrin III
Coproporphyrin III
Fe-coproporphyrin III
K00435
5-Amino-levulinate
HemB
HemC
HemD
Uropor-phyrinogen III
HemE
HemF
HemY
HemH
Protoheme (Heme)
K07215
Biliverdin-IX-δ
K07215
Biliverdin-IX-β
K07145
5-Oxo-δ-bilirubin
Porphobilinogen
Hydroxymethyl-bilane
Copropor-phyrinogen III
HemN
K02495
K00230
HemG
Protopor-phyrinogen IX
Protoporphyrin IX
CysG
CobA
HemL
K01845
K00643
(Anaerobic pathway)
(Aerobic pathway)
Siroheme
CysG
Sirohydrochlorin
CysG
Precorrin 2
K03403...
Heme O
Heme A
K08101
(3Z)-Phytochromobilin
K00510...
K21480
Biliverdin
K05371
(3Z)-Phycocyanobilin
K21796
Glutamate-1-semialdehyde
K02190...
CbiK
CbiX
Co-sirohydrochlorin
K22011
Mg-protoporphyrin IX
Peroxidase
Myoglobin
K05369
K05370
15,16-Dihydro-biliverdin
(3Z)-Phycoerythrobilin
Glycine
HemA
K02492
K03394...
CbiL
K03394...
CobI
K03428
Co-factor 3
Ni-sirohydro-chlorin
Precorrin 3A
Mg-protoporphyrin IX 13-monomethyl ester
K00214...
Cytochrome a
K08100
Cytochrome c
L-Glutamyl-tRNA(Glu)
K22012
K02229
CobG
(Anaerobic pathway)
(Aerobic pathway)
Catalase
D-Urobilinogen
Co-precorrin 3
Precorrin 3B
K04034
K04035
K00699
K01195
K05934...
CbiH
K21479
K05934...
CobJ
Hemoglobin
Oxyhemoglobin
Bilirubin
Bilirubin β-diglucuronide
D-Urobilin
Zn-Bacterio-chlorophyll a
L-Glutamate
Ni-sirohydro-chlorin a,c-diamide
Co-precorrin 4
Precorrin 4
K05936
CbiF
K05936
CobM
I-Urobilinogen
I-Urobilin
13[1]-Hydroxy-Mg-protoporphyrin IX 13-monomethyl ester
Alanine, aspartate and glutamate metabolism
K21610...
Co-precorrin 5A
Precorrin 5
K04034
K04035
K02189...
CbiG
K02228
CobF
L-Stercobilinogen
L-Stercobilin
15,17[3]-Seco-F430-17[3]-acid
Co-precorrin 5B
13[1]-Oxo-Mg-protoporphyrin IX 13-monomethyl ester
K02188
CbiD
K21612
Co-precorrin 6A
Precorrin 6A
K04034
K04035
12-Ethyl-8-isobutyl-3-vinyl-bacteriochlorophyllide d
K13603
K05895
CbiJ
K05895
CobK
K19073
Coenzyme F430
Co-precorrin 6B
Precorrin 6B
Divinylproto-chlorophyllide
K21231
Protochlorophyllide
K04036
K13601
Bacterio-chlorophyllide e
12-Ethyl-8-propyl-3-vinyl-bacteriochlorophyllide d
K13603
K02191
CbiT
K00595
K13601
K21312
Co-precorrin 7
K03399
CbiE
8,12-Diethyl-3-vinyl-bacteriochlorophyllide d
K13603
K13605
Bacterio-chlorophyllide c
Bacterio-chlorophyll c
Co-precorrin 8X
Precorrin 8X
K06042
CbiC
K06042
CobH
K00218
K00218
K04037...
K13602
K13604
Glycine, threonine metabolism
Hydrogenobyrinate
K21058
K13603
K13605
Cobyrinate
K02224
CbiA
K02224
CobB
8-Ethyl-12-methyl-3-vinyl-bacteriochlorophyllide d
Bacterio-chlorophyllide d
Bacterio-chlorophyll d
Hydrogenobyrinate a,c diamide
3-Hydroxyethyl-chlorophyllide a
Bacterio-chlorophyllide b
Geranylgeranyl-bacteriochlorophyllide b
K02230...
CobNST
K11336
K11333...
K04040
K10960
Cob(II)yrinate a,c diamide
3-Hydroxyethyl-bacteriochlorophyllide a
Bacterio-chlorophyll b
K13786
Bacterio-pheophytins
3-Vinylbacterio-chlorophyllide a
K11337
K04040
K10960
Cob(I)yrinate a,c diamide
K11333...
K11336
Bacterio-chlorophyllide a
Geranylgeranyl-bacteriochlorophyllide a
Bacterio-chlorophyll a
Divinyl-chlorophyllide a
CobA
BtuR
K00798...
CobO
K19073
Chlorophyllide a
K22013
Pheophorbide a
K13071
Red chlorophyll catabolite
Adenosyl cobyrinate a,c diamide
K13600
K13600
CbiP
K02232
CobQ
Adenosyl cobyrinate hexaamide
K13606
Chloro-phyllide b
K13544
K13545
(R)-1-Amino-propan-2-ol
7-Hydroxy-chlorophyllide a
CbiB
K02225...
CobC
CobD
protein α
K08260
CbiZ
K08099
K08099
Adenosyl cobinamide
K00798...
K04040
K04040
Pyro-pheophorbide a
Primary fluorescent chlorophyll catabolite
CobU
K02231
CobP
Cobinamide
7-Hydroxy-chlorophyll a
L-Threonine phosphate
(R)-1-Amino-propan-2-yl phosphate
K16651
K04720
K02225...
Adenosyl cobinamide phosphate
K18010
K13606
Chlorophyll b
Divinyl-chlorophyll a
Chlorophyll a
L-Threonine
CobU
K02231...
CobP
Cyanocob(III)alamin
K22013
Riboflavin metabolism
CobU
α-Ribazole-5'-P
α-Ribazole
Adenosine-GDP-cobinamide
K00768
K02226...
1.16.1.6
Dimethyl-benzimidazole
CobT
CobC
CobS
K02233
CobV
Pheophytin a
K05368
K21682
K00798...
1.16.1.4
Vitamin B12 coenzyme
Vitamin B12s
Vitamin B12r
Aquacob(III)alamin
00860 12/22/17
(c) Kanehisa Laboratories

# 9. Metabolism of terpenoids and polyketides

| MAP | PATHWAY |
|---|---|
| **900** | Terpenoid backbone biosynthesis |
| **909** | Sesquiterpenoid and triterpenoid biosynthesis |

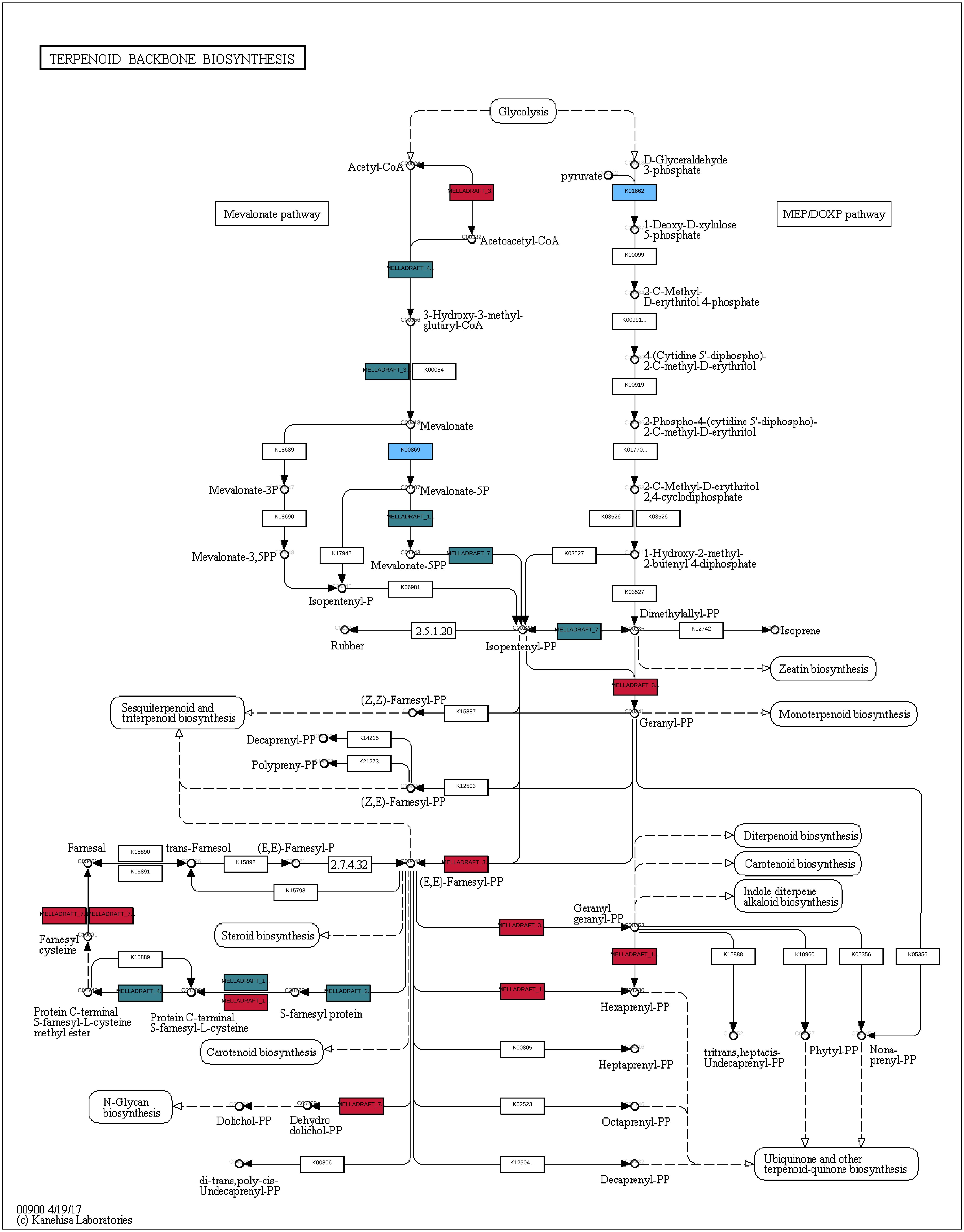
TERPENOID BACKBONE BIOSYNTHESIS
Glycolysis
Acetyl-CoA
MELLADRAFT_3...
Acetoacetyl-CoA
Mevalonate pathway
MELLADRAFT_4...
3-Hydroxy-3-methyl-
glutaryl-CoA
MELLADRAFT_3...
K00054
Mevalonate
K18689
K00869
Mevalonate-3P
Mevalonate-5P
K18690
MELLADRAFT_1...
Mevalonate-3,5PP
K17942
Mevalonate-5PP
MELLADRAFT_7...
Isopentenyl-P
K06981
Rubber
2.5.1.20
Isopentenyl-PP
MELLADRAFT_7...
D-Glyceraldehyde
3-phosphate
pyruvate
K01662
MEP/DOXP pathway
1-Deoxy-D-xylulose
5-phosphate
K00099
2-C-Methyl-
D-erythritol 4-phosphate
K00991...
4-(Cytidine 5'-diphospho)-
2-C-methyl-D-erythritol
K00919
2-Phospho-4-(cytidine 5'-diphospho)-
2-C-methyl-D-erythritol
K01770...
2-C-Methyl-D-erythritol
2,4-cyclodiphosphate
K03526
K03526
K03527
1-Hydroxy-2-methyl-
2-butenyl 4-diphosphate
K03527
Dimethylallyl-PP
K12742
Isoprene
Zeatin biosynthesis
MELLADRAFT_3...
Geranyl-PP
Monoterpenoid biosynthesis
(Z,Z)-Farnesyl-PP
K15887
Sesquiterpenoid and
triterpenoid biosynthesis
Decaprenyl-PP
K14215
Polypreny-PP
K21273
K12503
(Z,E)-Farnesyl-PP
Diterpenoid biosynthesis
Carotenoid biosynthesis
Indole diterpene
alkaloid biosynthesis
Farnesal
K15890
K15891
trans-Farnesol
K15892
(E,E)-Farnesyl-P
2.7.4.32
MELLADRAFT_3...
(E,E)-Farnesyl-PP
K15793
MELLADRAFT_7...
MELLADRAFT_7...
Farnesyl
cysteine
Steroid biosynthesis
K15889
MELLADRAFT_3...
Geranyl
geranyl-PP
MELLADRAFT_1...
K15888
K10960
K05356
K05356
MELLADRAFT_4...
MELLADRAFT_1...
MELLADRAFT_1...
MELLADRAFT_2...
MELLADRAFT_1...
Hexaprenyl-PP
Protein C-terminal
S-farnesyl-L-cysteine
methyl ester
Protein C-terminal
S-farnesyl-L-cysteine
S-farnesyl protein
Carotenoid biosynthesis
K00805
Heptaprenyl-PP
tritrans,heptacis-
Undecaprenyl-PP
Phytyl-PP
Nona-
prenyl-PP
N-Glycan
biosynthesis
Dolichol-PP
Dehydro
dolichol-PP
MELLADRAFT_7...
K02523
Octaprenyl-PP
Ubiquinone and other
terpenoid-quinone biosynthesis
K00806
di-trans,poly-cis-
Undecaprenyl-PP
K12504...
Decaprenyl-PP
00900 4/19/17
(c) Kanehisa Laboratories

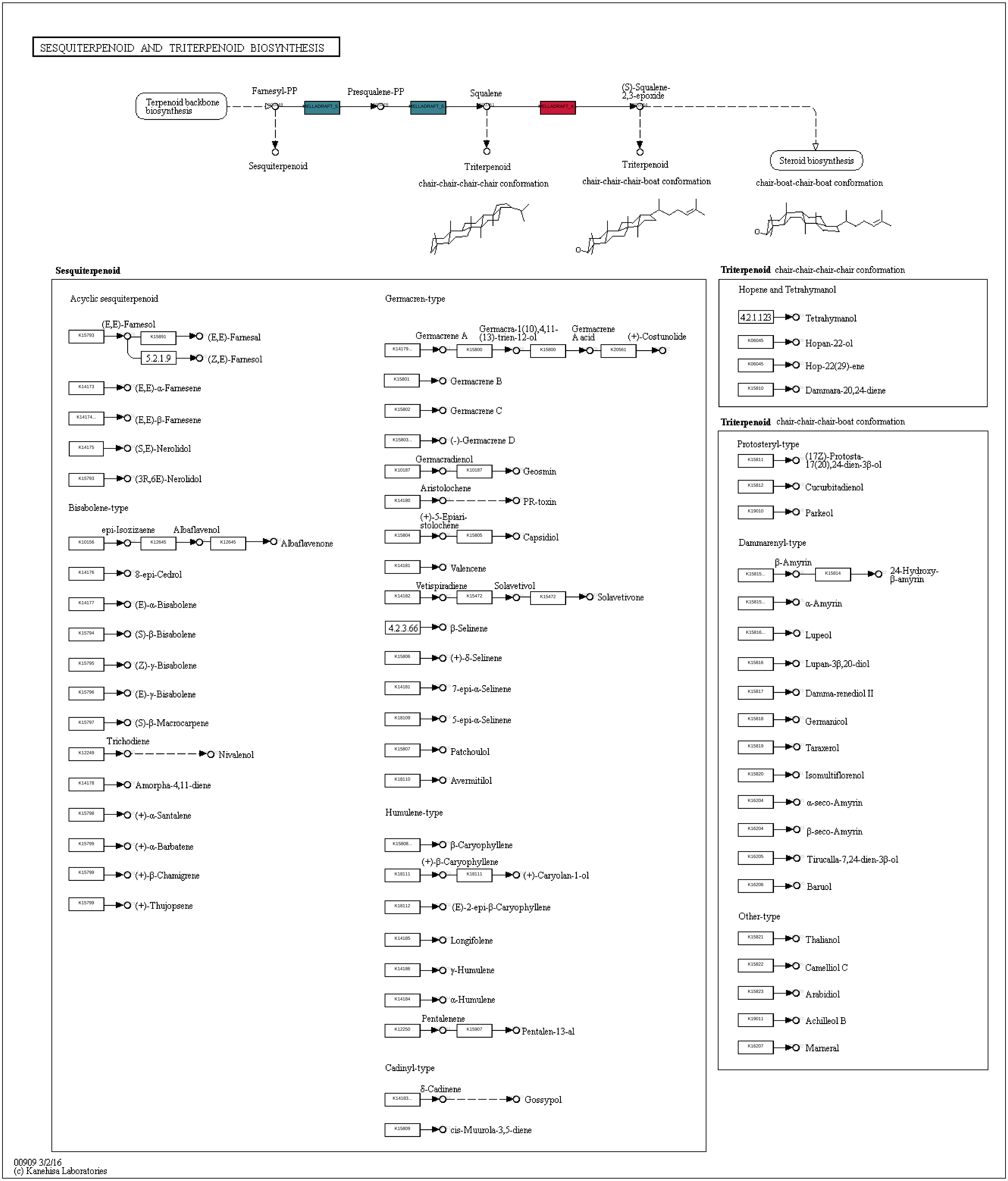
SESQUITERPENOID AND TRITERPENOID BIOSYNTHESIS
Terpenoid backbone biosynthesis
Farnesyl-PP
Presqualene-PP
Squalene
(S)-Squalene-2,3-epoxide
Sesquiterpenoid
Triterpenoid
chair-chair-chair-chair conformation
Triterpenoid
chair-chair-chair-boat conformation
Steroid biosynthesis
chair-boat-chair-boat conformation
Sesquiterpenoid
Acyclic sesquiterpenoid
(E,E)-Farnesol
(E,E)-Farnesal
5.2.1.9
(Z,E)-Farnesol
(E,E)-α-Farnesene
(E,E)-β-Farnesene
(S,E)-Nerolidol
(3R,6E)-Nerolidol
Bisabolene-type
epi-Isozizaene
Albaflavenol
Albaflavenone
8-epi-Cedrol
(E)-α-Bisabolene
(S)-β-Bisabolene
(Z)-γ-Bisabolene
(E)-γ-Bisabolene
(S)-β-Macrocarpene
Trichodiene
Nivalenol
Amorpha-4,11-diene
(+)-α-Santalene
(+)-α-Barbatene
(+)-β-Chamigrene
(+)-Thujopsene
Germacren-type
Germacrene A
Germacra-1(10),4,11-(13)-trien-12-ol
Germacrene A acid
(+)-Costunolide
Germacrene B
Germacrene C
(-)-Germacrene D
Germacradienol
Geosmin
Aristolochene
PR-toxin
(+)-5-Epiaristolochene
Capsidiol
Valencene
Vetispiradiene
Solavetivol
Solavetivone
4.2.3.66
β-Selinene
(+)-δ-Selinene
7-epi-α-Selinene
5-epi-α-Selinene
Patchoulol
Avermitilol
Humulene-type
β-Caryophyllene
(+)-β-Caryophyllene
(+)-Caryolan-1-ol
(E)-2-epi-β-Caryophyllene
Longifolene
γ-Humulene
α-Humulene
Pentalenene
Pentalen-13-al
Cadinyl-type
δ-Cadinene
Gossypol
cis-Muurola-3,5-diene
Triterpenoid chair-chair-chair-chair conformation
Hopene and Tetrahymanol
4.2.1.123
Tetrahymanol
Hopan-22-ol
Hop-22(29)-ene
Dammara-20,24-diene
Triterpenoid chair-chair-chair-boat conformation
Protosteryl-type
(17Z)-Protosta-17(20),24-dien-3β-ol
Cucurbitadienol
Parkeol
Dammarenyl-type
β-Amyrin
24-Hydroxy-β-amyrin
α-Amyrin
Lupeol
Lupan-3β,20-diol
Damma-renediol II
Germanicol
Taraxerol
Isomultiflorenol
α-seco-Amyrin
β-seco-Amyrin
Tirucalla-7,24-dien-3β-ol
Baruol
Other-type
Thalianol
Camelliol C
Arabidiol
Achilleol B
Marneral
00909 3/2/16
(c) Kanehisa Laboratories

# 10. Biosynthesis of other secondary metabolites

| MAP | PATHWAY |
|---|---|
| **261** | Monobactam biosynthesis |
| **332** | Carbapenem biosynthesis |

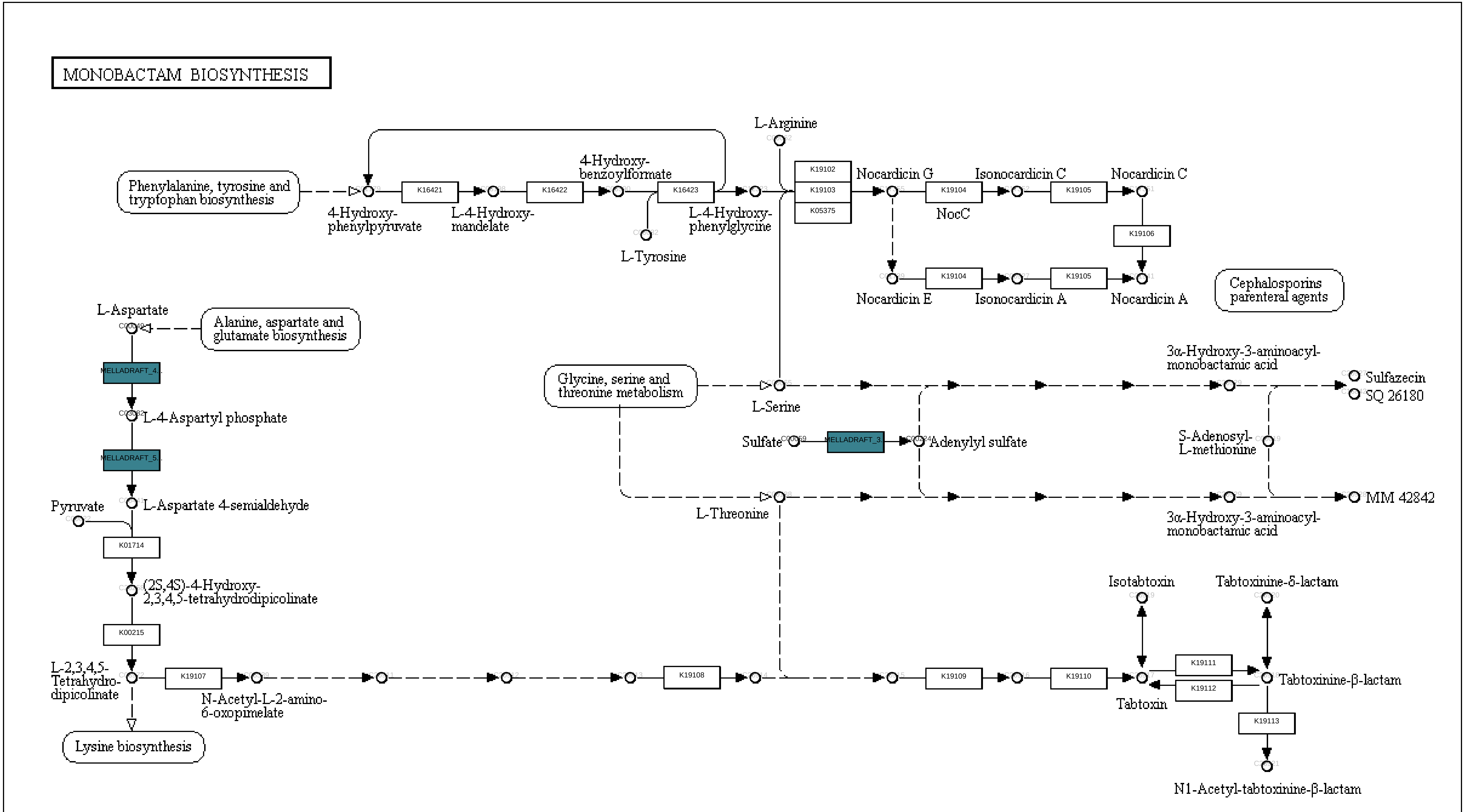


00261 4/3/15

(c) Kanehisa Laboratories

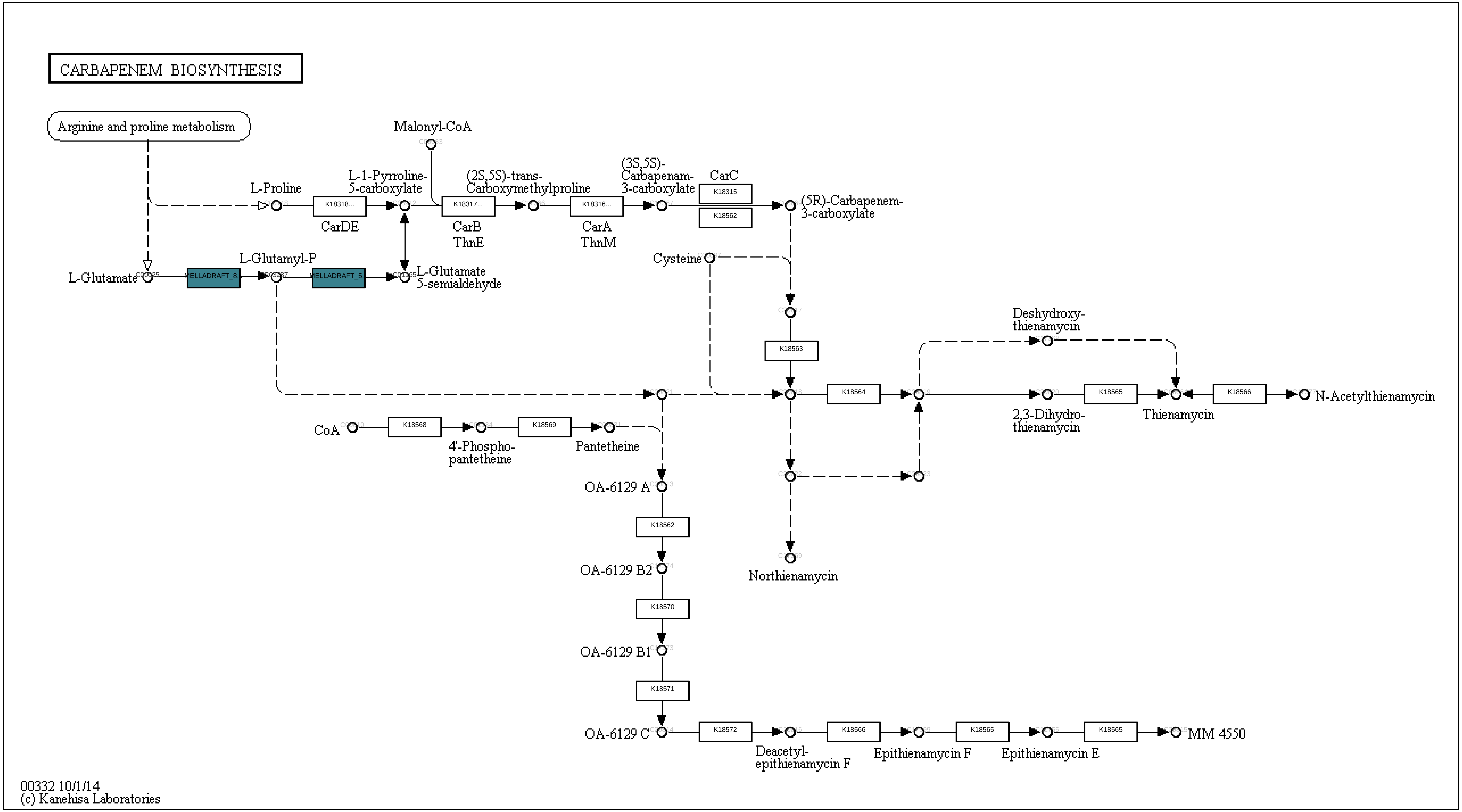
CARBAPENEM BIOSYNTHESIS
Arginine and proline metabolism
Malonyl-CoA
L-Proline
L-1-Pyrroline-
5-carboxylate
(2S,5S)-trans-
Carboxymethylproline
(3S,5S)-
Carbapenam-
3-carboxylate
CarC
K18315
K18562
(5R)-Carbapenem-
3-carboxylate
K18318...
CarDE
K18317...
CarB
ThnE
K18316...
CarA
ThnM
L-Glutamyl-P
L-Glutamate
MELLADRAFT_8...
MELLADRAFT_5...
L-Glutamate
5-semialdehyde
Cysteine
K18563
Deshydroxy-
thienamycin
K18564
K18565
K18566
N-Acetylthienamycin
2,3-Dihydro-
thienamycin
Thienamycin
CoA
K18568
4'-Phospho-
pantetheine
K18569
Pantetheine
OA-6129 A
K18562
OA-6129 B2
K18570
OA-6129 B1
K18571
OA-6129 C
K18572
Deacetyl-
epithienamycin F
K18566
Epithienamycin F
K18565
Epithienamycin E
K18565
MM 4550
Northienamycin
00332 10/1/14
(c) Kanehisa Laboratories

# 11. Xenobiotics biodegradation and metabolism

| MAP | PATHWAY |
|---|---|
| **791** | Atrazine degradation |

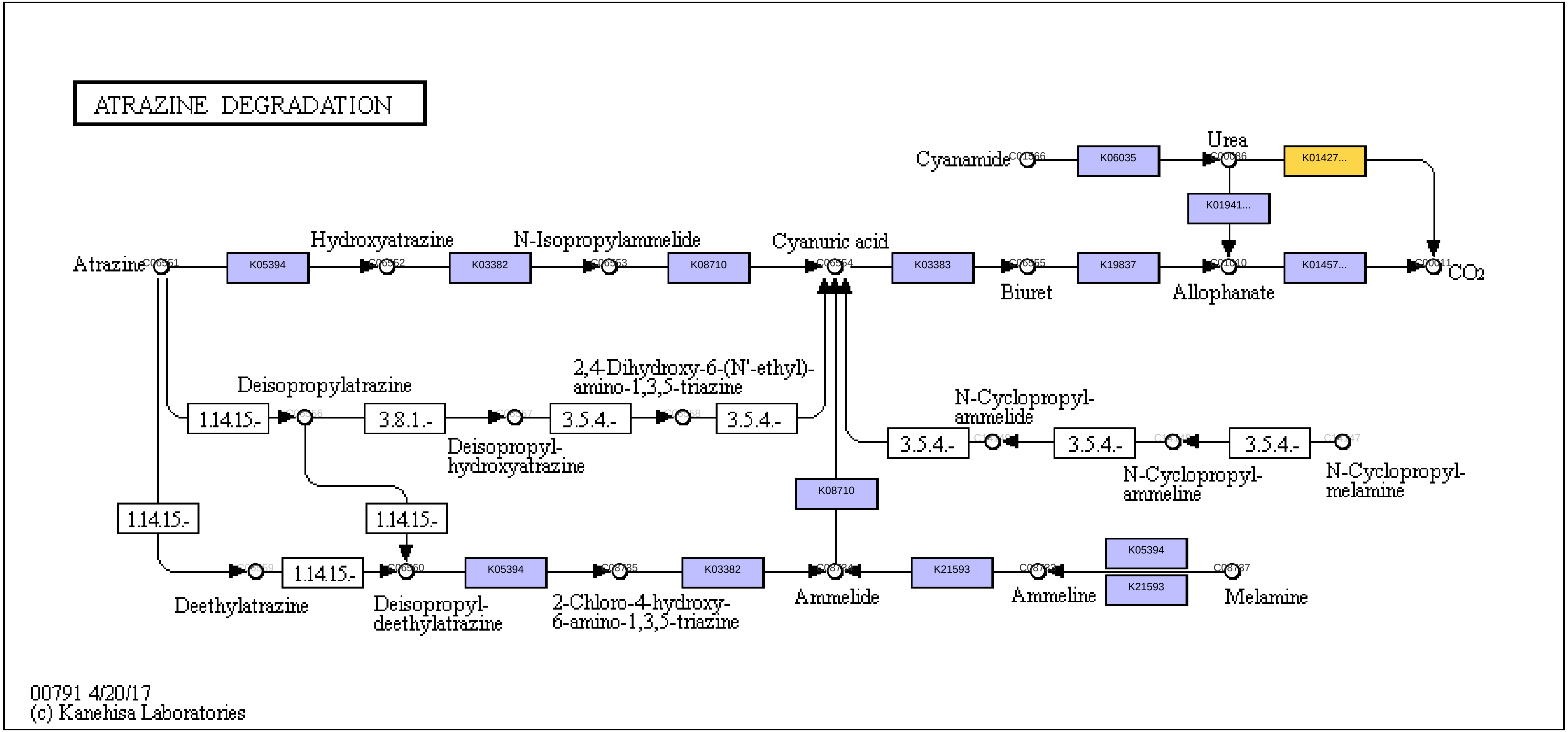
ATRAZINE DEGRADATION
Cyanamide
K06035
Urea
K01427...
K01941...
Atrazine
K05394
Hydroxyatrazine
K03382
N-Isopropylammelide
K08710
Cyanuric acid
K03383
Biuret
K19837
Allophanate
K01457...
CO2
Deisopropylatrazine
1.14.15.-
3.8.1.-
Deisopropyl-
hydroxyatrazine
2,4-Dihydroxy-6-(N'-ethyl)-
amino-1,3,5-triazine
3.5.4.-
3.5.4.-
N-Cyclopropyl-
ammelide
3.5.4.-
3.5.4.-
3.5.4.-
N-Cyclopropyl-
ammeline
N-Cyclopropyl-
melamine
K08710
1.14.15.-
1.14.15.-
1.14.15.-
Deethylatrazine
Deisopropyl-
deethylatrazine
K05394
2-Chloro-4-hydroxy-
6-amino-1,3,5-triazine
K03382
Ammelide
K21593
Ammeline
K05394
K21593
Melamine
00791 4/20/17
(c) Kanehisa Laboratories
